# Altered ribosomal profile in acquired resistance and reversal associates with pathological response to chemotherapy in inflammatory breast cancer

**DOI:** 10.1038/s41523-024-00664-0

**Published:** 2024-07-29

**Authors:** Gayathri R. Devi, Pritha Pai, Seayoung Lee, Matthew W. Foster, Dorababu S. Sannareddy, Francois Bertucci, Naoto Ueno, Steven Van Laere

**Affiliations:** 1 Department of Surgery, Division of Surgical Sciences, Duke University School of Medicine, Durham, NC USA; 2 Department of Pathology, Duke University School of Medicine, Durham, NC USA; 3 Duke Consortium for Inflammatory Breast Cancer, Duke Cancer Institute, Durham, NC USA; 4 Proteomics and Metabolomics Core Facility, Duke University School of Medicine, Durham, NC USA; 5 Predictive Oncology team, Centre de Recherche en Cancérologie de Marseille (CRCM), Inserm, CNRS, Aix-Marseille Université, Institut Paoli-Calmettes, Marseille, France; 6 University of Hawaii Cancer Center, Honolulu, HI USA; 7 Center for Oncological Research (CORE), Integrated Personalized and Precision Oncology Network (IPPON), University of Antwerp, Antwerp, Belgium

**Keywords:** Breast cancer, Prognostic markers

## Abstract

Therapeutic resistance presents a significant hurdle in combating inflammatory breast cancer (IBC), adding to the complexity of its management. To investigate these mechanisms, we conducted a comprehensive analysis using transcriptomic and proteomic profiling in a preclinical model alone with correlates of treatment response in IBC patients. This included SUM149 cell lines derived from treatment-naïve patients, along with acquired drug resistance (rSUM149) and others in a state of resistance reversal (rrSUM149), aiming to uncover drug resistance networks. We identified specific ribosomal proteins associated with acquiring resistance. These correlated with elevated levels of molecular markers such as pERK, CDK1, XIAP, and SOD2. While resistance reversal in rrSUM149 cells largely normalized the expression profile, VIPER analysis revealed persistent alterations in ribosomal process-related proteins (AGO2, Exportin 1, RPL5), suggesting their continued involvement in drug resistance. Moreover, genes linked to ribosomal processes were significantly enriched (*P* < 0.001) among overexpressed genes in IBC patients (*n* = 87) who exhibited a pathological complete response (pCR) to neoadjuvant chemotherapy. Given the common hyperactivation of MAPK in IBC tumors, including rSUM149, we evaluated Merestinib, a multikinase inhibitor in clinical trials. It effectively targeted pERK and peIF4E pathways, suppressed downstream targets, induced cell death in drug-resistant rSUM149 cells, and showed synergistic effects with another tyrosine kinase inhibitor (Lapatinib) in parental cells. This underscores its significant impact on protein synthesis signaling, crucial for combating translational dependence in cancer cells. In summary, our study elucidates adaptive changes in IBC cells in response to therapy and treatment pauses, guiding precision medicine approaches for this challenging cancer type.

## Introduction

Drug resistance in cancer, which can be categorized as either intrinsic (or de novo) or acquired, occurring after an initial treatment response^1^, is associated with adaptive changes leading to clonal selection within a population of tumor cells. The greater degree of tumor heterogeneity as the disease progresses is recognized as a major factor in the evolution of cell states that can either compete or cooperate, resulting in therapeutic failure^2^. Inflammatory breast cancer (IBC) is a type of locally advanced breast cancer wherein the clinicopathological hallmark is the presence of diffuse tumor cell clusters that show a high level of heterogeneity and plasticity in terms of epithelial, mesenchymal, and stem-like features^3,4^. Currently, there are no specific therapeutic strategies tailored for IBC, and despite being rare (less than 4% cases), IBC is responsible for 8–10% of breast cancer-related deaths, primarily due to aggressive progression and development of drug resistance^5,6^. One promising target is the epidermal growth factor receptor (EGFR), which is frequently overexpressed in IBC and has been linked to high recurrence rates and shorter survival durations^7^. Upon binding to its ligand, EGFR forms an active homodimer or heterodimerizes with other ErbB family members such as HER2, leading to activation of mitogen-activated protein kinase (MAPK), AKT, c-Jun N-terminal kinase (JNK), and phosphoinositide phospholipase C/protein kinase C (PLC/PKC) signaling pathways that are involved in cell growth, proliferation, migration, and differentiation. Various strategies targeting EGFR, such as TKI inhibitors and monoclonal antibodies, are currently being tested in IBC clinical trials, in combination with standard adjuvant or neoadjuvant therapies, with some showing promising clinical benefit^8,9^. In patient-derived IBC cell lines (triple-negative/basal-like SUM149; SUM190 with HER2 amplification), we previously found that a subpopulation of tumor cells were drug-tolerant and resistant to apoptotic stimuli, including EGFR targeting agents; these cells were consistently detected at a frequency higher than what would be expected due to mutational changes^10–12^. When this subpopulation was clonally expanded under selective pressure using an EGFR tyrosine kinase inhibitor (Lapatinib), it had a higher degree of cross-resistance to multiple chemotherapy and targeted drugs within the NCI Developmental Therapeutics Program (NCI-DTP) Approved Oncology Drug Set II^13^. This drug resistance phenotype was attributed to pathway dysregulations in the expression of anti-apoptotic and oxidative stress response proteins that allowed the therapy-resistant cells to be able to adapt to the redox imbalance by upregulating antioxidants, survive even in high ROS conditions (even when exposed to chemotherapy), and evade apoptosis^13,14^.

Interestingly, the removal of the primary drug to which the model was developed led to re-sensitization to not only the primary drug to which resistance developed but also to multiple drugs to a degree comparable to the parental cell line, leading to the generation of a resistance reversal model (rrSUM149). The concept of a planned cessation of cancer treatment for a period of time or a drug holiday is a strategy that has been explored in the treatment of various cancers, including breast, to mitigate treatment-related toxicities, improve patients’ quality of life, and potentially lessen drug resistance that would render the therapy ineffective^15–17^. Therefore, the current study was undertaken to conduct a comprehensive transcriptomic and proteomic analysis and compare the expression profiles of parental pretreatment SUM149, treatment-resistant rSUM149, and resistant reversal model rrSUM149, with the goal of identifying the molecular basis of drug resistance frequently observed in IBC patients for development of targeted therapeutics.

## Results

### Gene expression analysis shows significant similarities between parental and resistance-reversal cells compared to drug-resistant cells

**Fig. 1:**
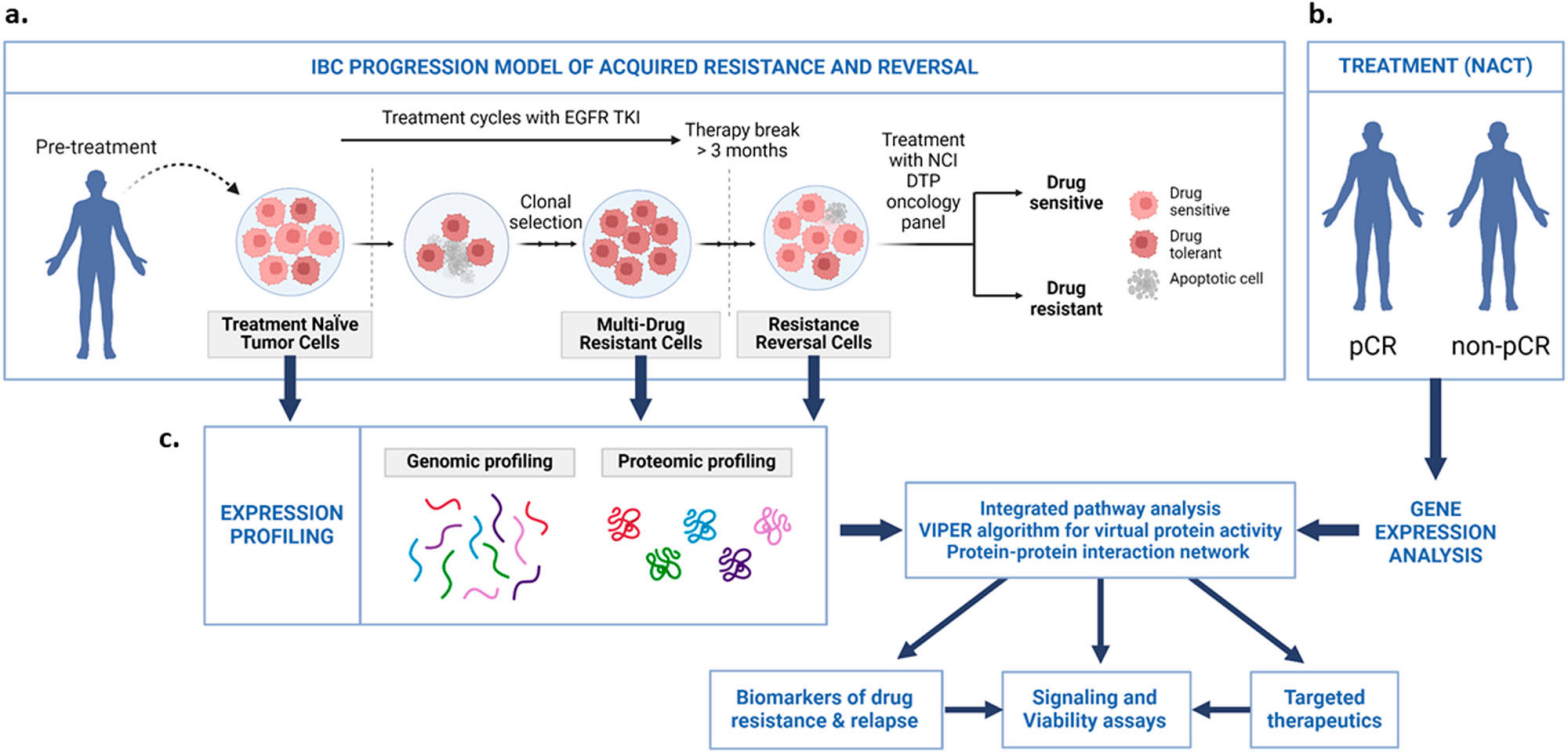
Model description and workflow. **a** Patient-derived inflammatory breast cancer (IBC) SUM149 cells, a treatment-naïve model, were chronically exposed to therapeutic stress with the EGFR-TKI Lapatinib and clonally selected to create a multidrug-resistant model rSUM149. To generate a resistance reversal model, Lapatinib was removed from the culture medium of rSUM149 cells, and the cells were allowed to grow in a “drug holiday” for over 3 months to become rrSUM149. **b** In order to further elucidate the mechanisms of resistance acquisition and reversal, data from IBC patients that did or did not achieve pathological complete response (pCR) to neoadjuvant chemotherapy (NACT) were investigated with gene expression analysis. **c** Integrated pathway analysis of all the RNA sequencing and proteomics datasets using the VIPER algorithm to identify key signals, which were subsequently validated using immunoblot analysis, viability assays, and targeted agents. (*generated in BioRender.com*).

**Fig. 2:**
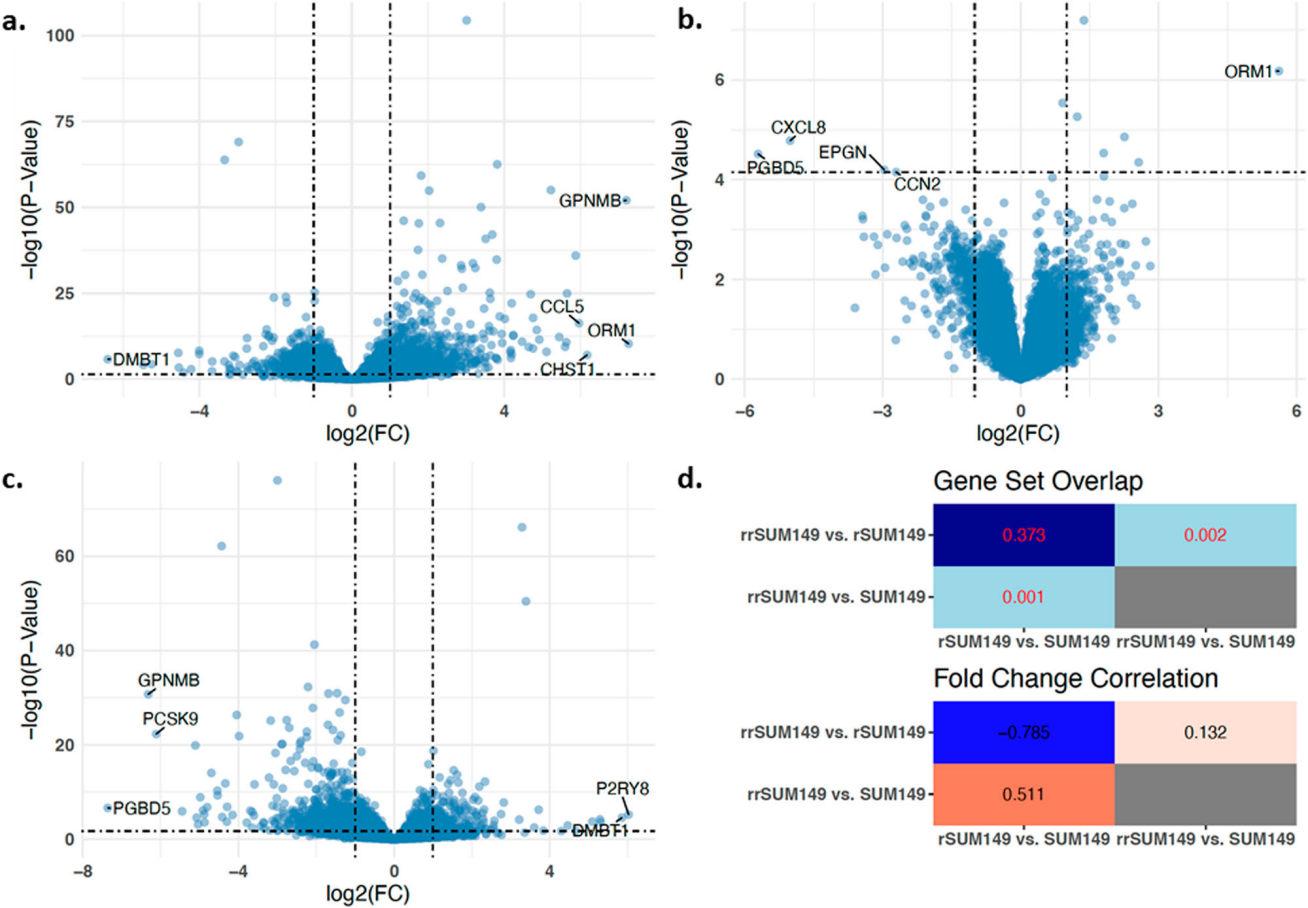
Volcano plots of gene expression data showing relatively up- and down-regulated genes. **a** rSUM149 compared to SUM149, **b** rrSUM149 compared to SUM149, and **c** rrSUM149 compared to rSUM149. The X-axis indicates log2 fold change with a threshold of 1, and the Y-axis indicates –log10 *p* value with a threshold of 0.10. The top five genes are shown in each plot. **d** Heatmap of the pairwise overlaps between the differentially expressed genes quantified using JI (top panel) and correlation coefficient between the vectors of log2 fold changes (bottom panel). Numbers indicate JI values and correlation coefficients respectively.

The IBC patient-derived preclinical model of acquired resistance and reversal consists of the treatment-naïve SUM149 cells that were used to clonally select for EGFR-TKI (Lapatinib)-tolerant cells (designated rSUM149) followed by removal of the inhibitor for >3 months to isolate a population of cells (designated rrSUM149) exhibiting therapeutic sensitivity similar to the parental cells for various EGFR/Her2 TKI (Fig. 1). Previously, we had reported that rSUM149 variant exhibited multidrug resistance to various chemotherapeutic classes of drugs in the NCI-DTP oncology panel compared to the rrSUM149, which was similar to SUM149 in sensitivity but exhibited resistance to platinum, alkylating, topoisomerase, based drugs similar to rSUM149^13^. Therefore, to elucidate the underlying mechanisms associated with acquired multidrug resistance and therapy break/therapeutic relapse, we first conducted RNA sequencing to characterize the differences in gene expression for individual genes between the three states. First, we explored global themes in mRNA expression in our dataset using PCA. Results are shown in Supplementary Figure 1a. The resulting scattering pattern demonstrates that the strongest variation in gene expression, captured by PC1, separates rSUM149 cells from both SUM149 and rrSUM149 cells. In addition, PC2 primarily captures heterogeneity amongst replicate samples from rSUM149 and rrSUM149 cells. This analysis suggests that the gene expression profiles of SUM149 and rrSUM149 cells are very similar. Then, differences in gene expression were assessed, as shown by volcano plots (Fig. 2a–c). The numbers of differentially expressed genes (DEGs) grouped by the target variables reveal 6,007 DEGs between SUM149 and rSUM149 cells (Fig. 2a), 2,842 between rSUM149 and rrSUM149 cells (Fig. 2b) and interestingly, only 11 DEGs (*ORM1, MEFV, C1S, CXCL8, DOC2B, ATP6ViG2, PGBD5, FCGBP, EPGN,* and *CCN2*) between SUM149 and rrSUM149 cells (Fig. 2c). Differential gene expression statistics for all comparisons are provided in Supplementary Table 1. Pairwise overlaps between the lists of differentially expressed genes, quantified using JI, are shown in heatmap format in Fig. 2d and identifies similarities amongst differentially expressed genes between SUM149 and rSUM149 cells on the one hand and between rSUM149 and rrSUM149 cells on the other hand (JI = 0.373; *P* < 0.001). In addition, a comparison of the vectors of log2-transformed fold-changes resulting from both comparisons revealed a significant negative correlation coefficient (*R* = −0.785; *P* < 0.001). Together, these data suggest that the gene expression profile in resistance reversal, as observed in the rrSUM149 variant is similar to the baseline condition (i.e., parental SUM149 cells). Furthermore, limited similarities amongst differentially expressed genes between SUM149 and rSUM149 cells on one hand and between SUM149 and rrSUM149 cells on the other hand were noted (JI = 0.001; *P* = 0.036), but a significant positive correlation coefficient between the respective log2-transformed fold-changes was observed (*R* = 0.511; *P* < 0.001). This suggests that resistance reversal (rrSUM149) to the baseline profile is incomplete and acquired resistance (rSUM149) induces more permanent gene expression changes.

### Patterns of protein and gene expression changes during resistance reversal are similar to treatment-naïve parental cells

**Fig. 3:**
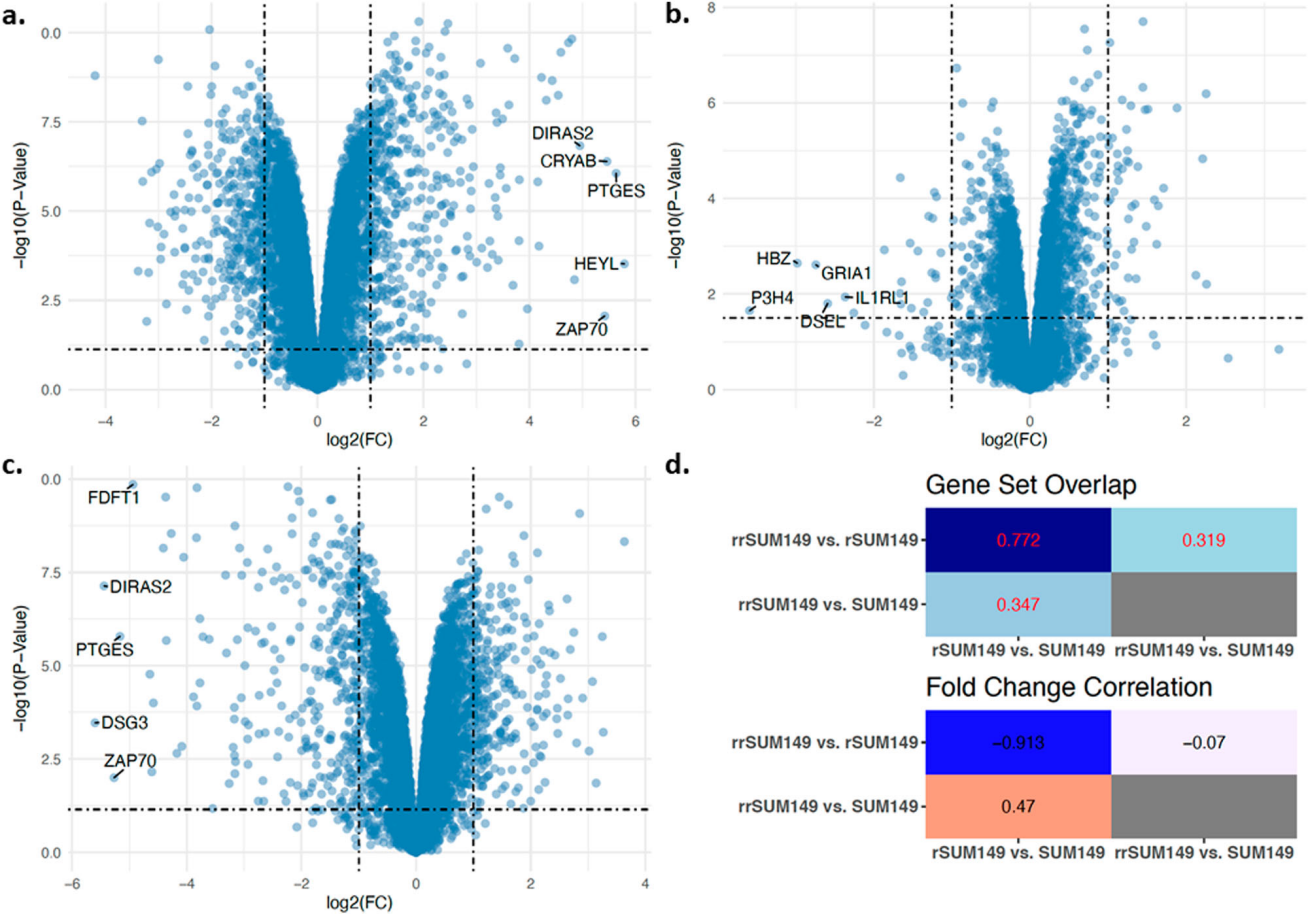
Volcano plots of proteomics data showing relatively up- and down-regulated proteins. **a** rSUM149 compared to SUM149, **b** rrSUM149 compared to SUM149, and **c** rrSUM149 compared to rSUM149. The X-axis indicates log2 fold change with a threshold of 1, and the Y-axis indicates –log10 *p* value with a threshold of 0.10. The top five proteins are shown in each plot. **d** Heatmap of the pairwise overlaps between the differentially expressed proteins quantified using JI (top panel) and correlation coefficient between the vectors of log2 fold changes (bottom panel). Numbers indicate JI values and correlation coefficients respectively.

Next, we assessed individual protein expression differences between the three cell lines. To explore global themes in protein expression in our dataset, PCA was performed. Results are shown in Supplementary Fig. 1b. The resulting scattering pattern again demonstrates that the strongest variation in protein expression resides in the difference between rSUM149 cells on the one hand and both SUM149 and rrSUM149 cells on the other hand. In contrast to the mRNA analysis, PC2 is associated with the difference between SUM149 and rrSUM149 cells. This analysis corroborates the hypothesis that the molecular profiles of SUM149 and rrSUM149 cells are similar. Then, we assessed the difference in protein expression, as shown in the volcano plot format in Fig. 3a–c. The numbers of differentially expressed proteins between samples grouped by the target variables are as follows: 4658 proteins between SUM149 and rSUM149 (Fig. 3a), 1930 proteins between SUM149 and rrSUM149 (Fig. 3b), and 4349 proteins between rSUM149 and rrSUM149 (Fig. 3c). Differential protein expression statistics for all comparisons are provided in Supplementary Table 2. Pairwise overlaps between the lists of differentially expressed proteins, quantified using JI, are shown in heatmap format in Fig. 3d, which identifies similarities amongst differentially expressed proteins between SUM149 and rSUM149 on the one hand and between rSUM149 and rrSUM149 on the other (JI = 0.772; *P* < 0.001). In addition, a comparison of the vectors of log2-transformed fold-changes resulting from both comparisons revealed a significant negative correlation coefficient (*R* = −0.913; *P* < 0.001). Again, similarities amongst differentially expressed proteins between SUM149 vs. rSUM149 cells on the one hand, and those between SUM149 vs. rrSUM149 cells on the other are limited (JI = 0.347; *P* < 0.001), but a significant positive correlation coefficient between the respective log2-transformed fold-changes was observed (*R* = 0.470; *P* < 0.001). Notably, the fact that differences at the protein level between SUM149 and rrSUM149 cells are larger than those at the mRNA level suggests an important role in post-transcriptional processes.

### Integrated pathway analysis identifies biological themes of resistance acquisition and reversal including deregulated metabolic and DNA repair pathways

Based on the aforementioned datasets, we performed an integrated pathway analysis wherein, gene expression differences were first translated into virtual protein activity differences using the VIPER algorithm. The number of proteins predicted to be differentially activated (i.e., FDR <10% and absolute NES >5) between samples grouped by the target variables are as follows: 521 proteins between SUM149 and rSUM149 cells, 325 proteins between rrSUM149 and SUM149, and 330 proteins between rSUM149 and rrSUM149 cells. Plots showing the top 20 differentially activated proteins per contrast are shown in Supplementary Fig. 1, along with complete results in Supplementary Table 3. Respectively, 142 (28%), 93 (29%), and 85 (26%) proteins with pleiotropic interactions were identified. Network plots showing pleiotropic interactions are shown in Supplementary Fig. 2, and the degree of in-degree for each node in these networks is provided in Supplementary Table 3.

Based on the combined information from the protein expression and activity (i.e., VIPER) datasets, 136 and 82 seed proteins with a significant difference in expression (FDR <10%) and virtual protein activity (FDR <10% and absolute NES >5) in the same direction were identified when comparing SUM149 to rSUM149 cells and rSUM149 to rrSUM149 cells respectively. Fifteen seed proteins were identified for the comparison of SUM149 and rrSUM149 cells, which is in line with the limited differences reported between these isoforms. All sets of proteins were mapped onto the STRING PPI network and all pairwise shortest paths were calculated. Graphs of all interactions between proteins involved in resistance acquisition (i.e., SUM149 vs. rSUM149; 1626 proteins and 32,262 edges), resistance reversal (i.e., rSUM149 vs. rrSUM149 cells; 1051 proteins and 16,528 edges), and between SUM149 vs. rrSUM149 cells (i.e., 105 proteins and 475 edges) are shown as an example in Supplementary Fig. 3. The node degree distribution for proteins of interest is shown in bar plot format in Supplementary Fig. 4. Importantly, both graphs are significantly enriched for additional proteins that were not part of the lists of seed proteins but were significantly differentially expressed and/or activated with respect to the contrasts of interest (SUM149 vs. rSUM149: OR = 2.857 – *P* < 0.001; rSUM149 vs. rrSUM149: 2.406 – *P* < 0.001; SUM149 vs. rrSUM149: OR = 2.675 – *P* < 0.001), suggesting that the identified subgraphs recapitulate molecular interactions that are relevant to the biology of resistance and resistance reversal.

**Fig. 4:**
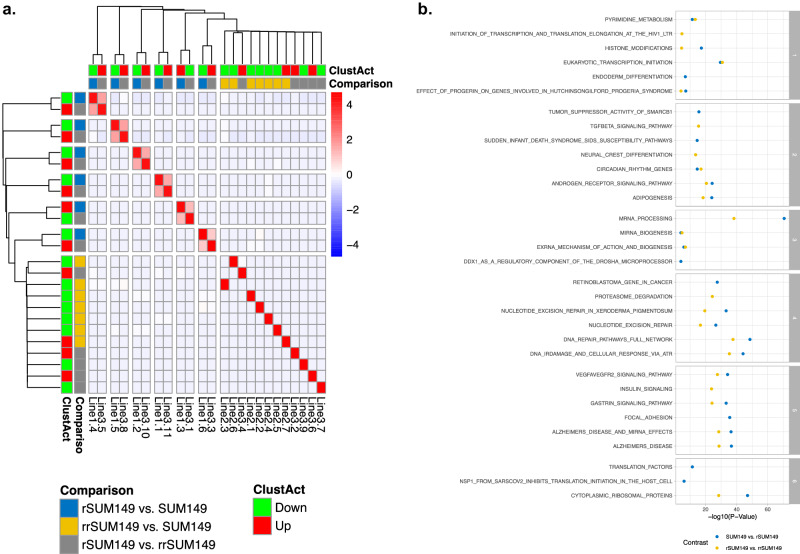
Integrated pathway analysis to identify biological themes of resistance acquisition and reversal. **a** Heatmap showing the comparative analysis of the different gene clusters found in the graphs resulting from the comparison of rSUM149 vs. SUM149 cells, rrSUM149 vs. SUM149 cells, and rSUM149 vs. rrSUM149 cells. Pairwise overlaps between each of these gene clusters are calculated using Jaccard indices, which are color-coded in the heatmap according to the color bar shown to the right (i.e., blue to red indicating limited to strong overlap). The matrix of Jaccard indices was subjected to unsupervised hierarchical clustering and the resulting dendrograms on top and to the left of the heatmap determine the order of the gene clusters along the X- and Y-axis. For each gene cluster, its activity score, calculated as explained in the text, is shown underneath the dendrogram with green and red indicating, respectively, a larger number of proteins with downregulated or upregulated activity or expression. The second label row underneath the dendrogram indicates in which graph the respective gene clusters were identified using blue (i.e., rSUM149 vs. SUM149), yellow (i.e., rrSUM149 vs. SUM149), and gray (i.e., rSUM149 vs. rrSUM149) labels. One should note that all gene clusters identified in the graph representing molecular differences between the rSUM149 and SUM149 cells (blue labels) cluster on terminal branches with one gene cluster identified in the graph representing molecular differences between the rrSUM149 and rSUM149 cells (gray) labels but that the activity of these cluster changes from up (green label) to down (red label). This indicates that these gene clusters are found in both graphs but with opposing activities. The remaining gene clusters (i.e., those from the rrSUM149 vs. SUM149 graph, and 5 gene clusters from the rrSUM149 vs. rSUM149 graph) are unique. **b** Facetted dot plot showing the enrichment profiles of the six overlapping gene clusters of the rSUM149 vs. SUM149 (blue dots) and rrSUM149 vs. rSUM149 (yellow dots) networks. Only significant gene sets are shown.

In order to identify modules of interacting proteins that are responsible for resistance acquisition and reversal in SUM149 cells, we subjected all graphs (Supplementary Fig. 3) to network clustering. In the SUM149 vs. rSUM149 and rSUM149 vs. rrSUM149 graphs, respectively 6 and 11 clusters were identified (average modularity of respectively 0.53 and 0.46). In the graph capturing molecular differences between SUM149 and rrSUM149 cells, seven clusters were identified (average modularity of 0.39). For each cluster in each network, the number of upregulated (i.e., either activated or overexpressed) and downregulated (i.e., either repressed or under-expressed) proteins were investigated. Cluster activity was defined as down or up based on respectively a negative or positive difference between the number of repressed and activated proteins within that cluster. Also, overlaps in terms of gene membership were quantified using the Jaccard Index between all pairs of clusters identified in the three graphs. Results are provided in heatmap format in Fig. 4a. Interestingly, all clusters identified in the rSUM149 vs. SUM149 comparison (*N* = 6) strongly overlapped with a cluster from the rrSUM149 vs. rSUM149 comparison. Moreover, clusters activated during resistance acquisition were inactivated during reversal and vice versa. Pathway enrichment profiles for these six clusters are provided in Fig. 4b and in Supplementary Table 4. Logically, overlapping clusters also exhibit comparable pathway enrichment profiles. The VEGF signaling and focal adhesion enriched network (i.e., cluster 5) was the only one observed to be activated upon resistance acquisition. The other clusters identified are involved in metabolism and transcription (i.e., cluster 1), SMARC1 and TGF-beta signaling (i.e., cluster 2), microRNA processing (i.e., cluster 3), DNA metabolism and cell cycle (i.e. cluster 4), and translation (i.e., cluster 6).

### Changes in cytosolic ribosomal protein network in drug resistance are only partly reversed

**Fig. 5:**
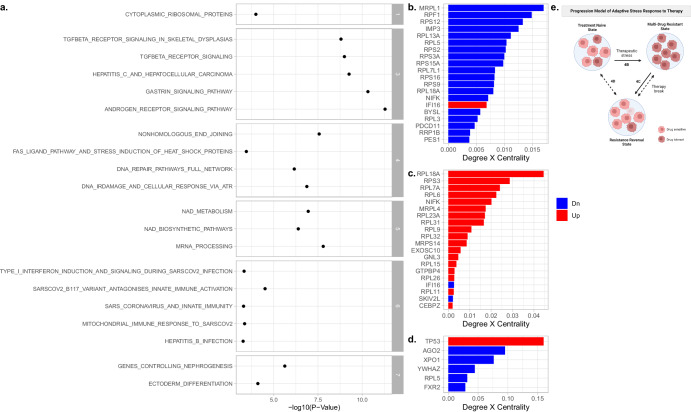
Pathway enrichment analysis. **a** Facetted dot plot showing the enrichment profiles of the seven gene clusters of the rrSUM149 vs. SUM149 networks. Only significant gene sets are shown. For the second cluster, no significant results were retained, and hence, this cluster is not shown. **b**–**d** Bar plots showing the topological characteristics of ribosomal proteins, expressed as the product of node degree and betweenness centrality (X-axis), in the rSUM149 vs. SUM149 (**b**), rrSUM149 vs. rSUM149 (**c**), and rrSUM149 vs. SUM149 (**d**) networks. Proteins with higher values are more important in the network. Each bar is color-coded red or blue depending on the change in activity level (i.e., Up vs. Down). **e** A simplified schematic rendition to summarize the three states.

Interestingly, all clusters identified in the rrSUM149 vs. SUM149 comparison (*N* = 7) did not reveal similarities in protein membership with clusters identified in the two other comparisons, suggesting that differences between rrSUM149 and SUM149 cells reflect more permanent changes acquired by SUM149 cells during development of drug resistance (rSUM149). Pathway enrichment profiles for these clusters are provided in Fig. 5a and in Supplementary Table 5. Interestingly, several gene sets and biological pathways identified in part of the reversal process from rSUM149 to rrSUM149 (i.e., cytoplasmic ribosomal proteins, TGF-beta signaling, AR signaling, and DNA repair pathways) were reidentified, suggesting these pathways may have pleiotropic effects in governing drug resistance. This was further demonstrated by examining activity changes of ribosomal proteins in the graphs representing the different contrasts (Fig. 5b–d). As shown in Fig. 5a, the activity of ribosomal proteins is collectively repressed in rSUM149 relative to SUM149 cells. This pattern is reversed when comparing rrSUM149 cells relative to rSUM149 cells (Fig. 5c), but not completely as some proteins associated with ribosomal protein biology are differentially activated between SUM149 and rrSUM149 cells (Fig. 5d) and a schematic representation of the three states of cell populations shown in Fig. 5e. Collectively, these datasets show the potential of altered expression of an individual component or as a group can impact therapeutic sensitivity.

### Correlation of global ribosomal gene expression to pathological complete response in IBC patients

**Fig. 6:**
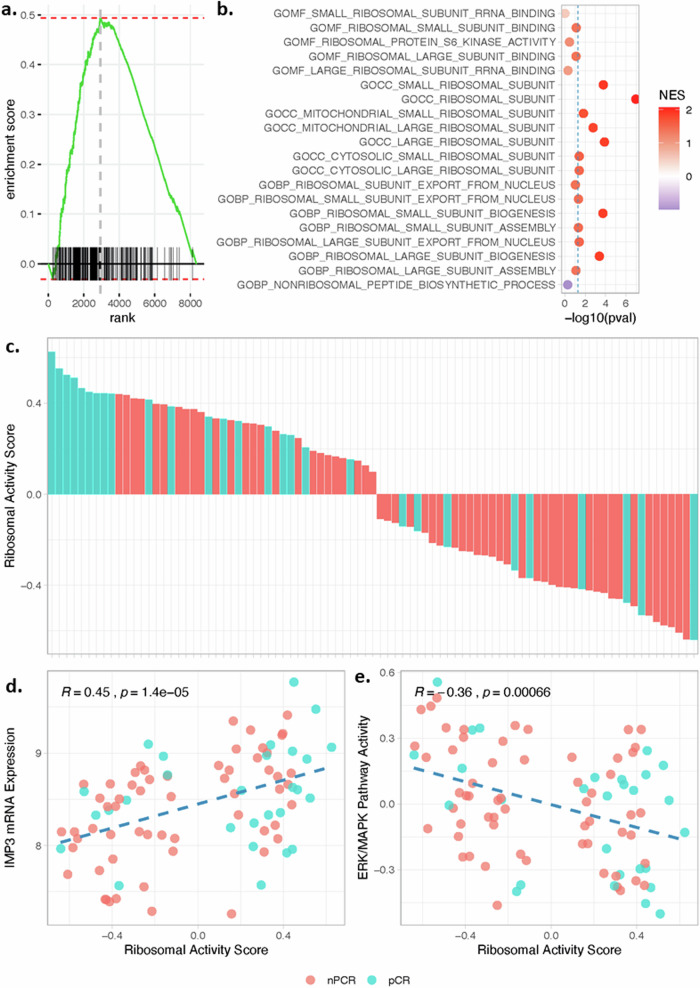
Gene expression analysis associated with pCR to NACT in IBC cohort. **a** Gene set enrichment profile of all genes associated with each of the 20 ribosomal gene ontology terms when comparing patients with IBC with vs. without pathological complete response to neoadjuvant chemotherapy. All genes are ordered along the X-axis based on a decreasing fold change, with high and low values indicating genes overexpressed in patients with and without pCR respectively. The black vertical ticks along the X-axis denote the positions of the ribosomal genes. The cumulative distribution of these genes along the ordered fold-change vector is given by the green curve, with a positive value revealing a global overexpression of these genes in the patients with pCR. The vertical blue dashed line indicates the maximum enrichment value. All ribosomal genes (i.e., black ticks along the X-axis) to the left of this line constitute the leading-edge genes. **b** Dot plot showing the gene set enrichment analysis results (X-axis denotes the significance level and the color of the dot the normalized enrichment score with positive values reflective of gene sets overexpressed in patients with pCR) for each of the individual ribosomal gene ontology terms. **c** Bar plot showing the ribosomal activity scores (Y-axis) for each patient in the IBC series (X-axis). Ribosomal activity scores are calculated using the leading-edge genes, that are indicated by the black ticks along the X-axis and to the left of the vertical gray line in Fig. 6a. Bars are color-coded according to the absence (red) or presence (blue) of pathological complete response as indicated in the legend at the bottom of the figure. The *p* value denoting the significance level of the difference is indicated in the top right corner. **d** Scatter plot showing the relation between IMP3 mRNA expression (Y-axis) and the ribosomal activity score (X-axis) in the patient series. Each dot represents one patient, and the dots are color-coded according to the absence (red) or presence (blue) of pathological complete response as indicated in the legend at the bottom of the figure. In addition, a regression line (dashed blue line) and results of a Spearman correlation analysis (top left) are provided. **e** Scatter plot showing the relation between ERK/MAPK pathway activity (Y-axis) and the ribosomal activity score (X-axis) in the patient series. ERK/MAPK pathway activities are calculated using a set of ERK/MAPK target genes identified in the Reactome knowledge base. Each dot represents one patient, and the dots are color-coded according to the absence (red) or presence (blue) of pathological complete response as indicated in the legend at the bottom of the figure. In addition, a regression line (dashed blue line) and results of a Spearman correlation analysis (top left) are provided.

To validate our findings in a clinical cohort of IBC pretreatment tumor samples with recorded pathological responses to neoadjuvant chemotherapy (*n* = 87), we performed gene set enrichment analysis (GSEA) on the vector of fold-changes representing genome-wide expression changes between patients with and without pCR. The full list of 341 genes associated with any of the 20 ribosome-related gene ontology terms was significantly enriched amongst genes overexpressed in patients with pCR (NES = 2.060; *P* < 0.001) as shown in Fig. 6a. In addition, enrichment analysis was performed for the 20 individual sets of ribosomal genes, revealing significant overexpression of genes belonging to 11 of the gene ontology terms (Fig. 6b). To note, none of the sets of ribosomal genes exhibited the opposite expression pattern. When calculating a ribosomal activation score based on the leading-edge genes identified through gene set enrichment analysis, significant differences between patients with IBC with vs. without pCR were revealed (*P* = 0.013), corroborating our results. Data were shown in Fig. 6c. Finally, we also tested the sets of genes identified in previous analysis and reported in Fig. 5b–d. Most importantly, this analysis revealed that the only genes reported to be repressed in rSUM149 vs. SUM149 also showed significant enrichment in IBC patients with pCR (NES = 1.491; *P* = 0.041). Intersection of the leading-edge genes of the ribosomal genes shown in Fig. 5b and the full list of ribosomal genes retrieved from the Molecular Signatures Database identified *RPS7, RPL35, RPL31, FAU, RPS16, RPS9, RPS15A, RPS12, RPS14, RPL3, RPS18, RPL11, RPS5, RPS4X, RPL10A, RPLP0, RPS3,* and *RPS27* as candidate ribosomal genes involved in acquired resistance.

### Merestinib-mediated targeting of pERK/peIFE signaling inhibits CDK1, SOD2, IMP3, inhibits cell viability alone and in combination with lapatinib

**Fig. 7:**
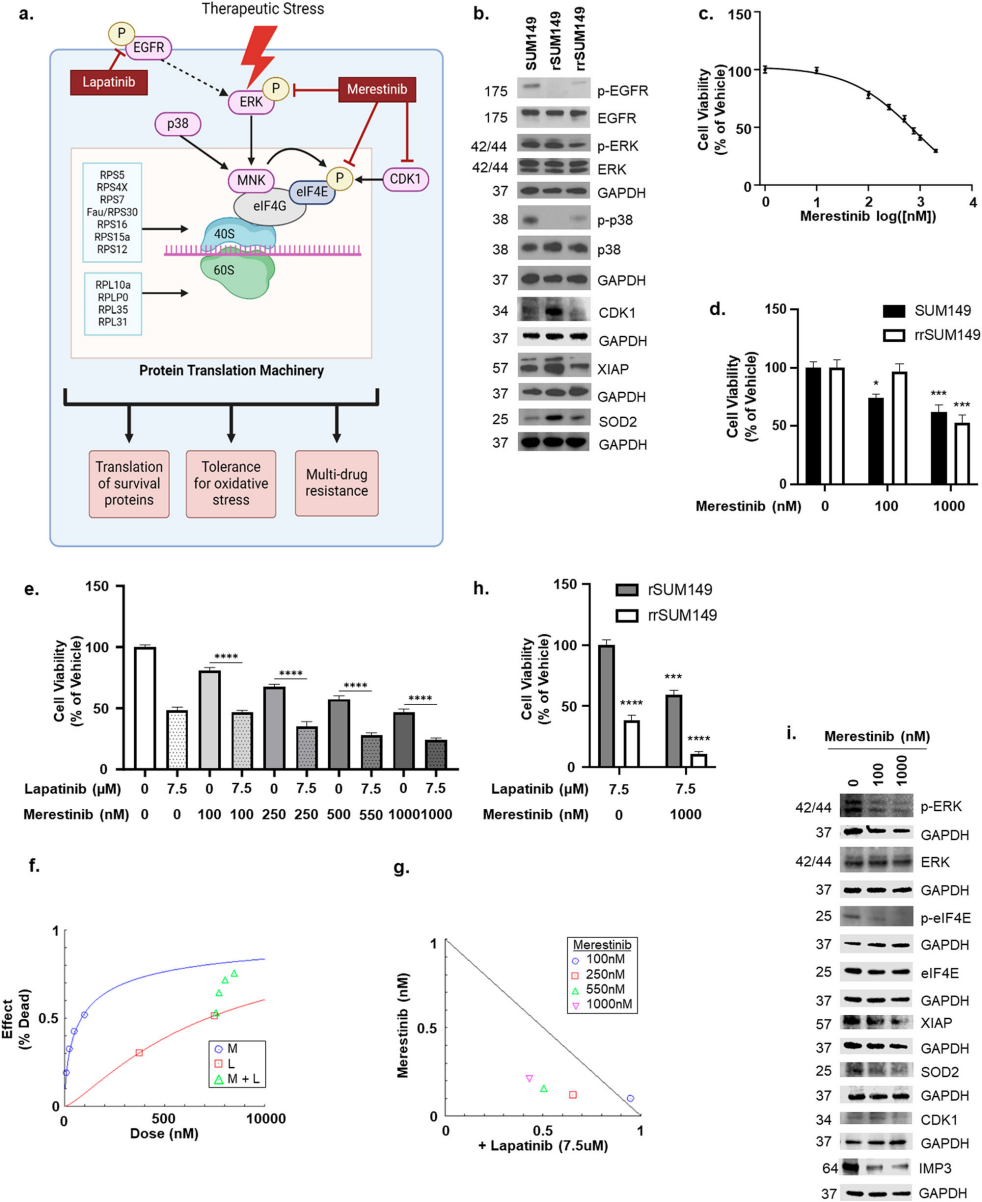
Merestinib suppresses pERK/peIF4E survival signaling in translational control. **a** Chronic exposure of IBC cells to the primary drug lapatinib, a dual TKI, activates MAPK signaling that leads to phosphorylation of ERK and expression of the p38 MAPK^11,13,33^. This leads to the activation of MNK, an eIF4G-associated factor, and the phosphorylation of eIF4E and initiation of protein translation. Our previous work has the role of MNK-XIAP-NFkB crosstalk in aggressive breast cancer progression and therapeutic resistance^33^. Herein, based on our datasets the targets of a MNK inhibitor Merestinib are shown. (*generated in BioRender.com*) **b** Immunoblot of indicated proteins in SUM149, rSUM149, and rrSUM149 cells. GAPDH was used as a loading control. **c** Twelve replicate seven-point dose-response (viability) curve of SUM149 treated for 24 h with Merestinib as indicated via luciferase assay. Bars represent mean ± s.e.m. as a percentage of DMSO vehicle. **d** Graph showing luciferase activity of SUM149, and rrSUM149 GFP-Luc cells after 24 h of treatment with Merestinib as indicated. Bars represent mean ± s.e.m. as a percentage of DMSO vehicle (*n* = 3(3–6); *P < 0.05; ***P ≤ 0.001). **e** Graph showing luciferase activity of SUM149-Luc cells after 24 h of treatment with Merestinib and Lapatinib as single agents and in combination as indicated. Bars represent mean ± s.e.m. as a percentage of DMSO vehicle (*n* = 3(6); ****P < 0.0001). **f** Graph showing the dose-effect (death) curve of Merestinib and Lapatinib and their combination treatment using CompuSyn software. **g** Normalized Isobologram of combination treatment of Merestinib and Lapatinib as indicated using CompuSyn. **h** Graph showing luciferase activity of rSUM149, and rrSUM149 GFP-Luc cells after 24 h of treatment with Merestinib and Lapatinib as indicated. Bars represent mean ± s.e.m. as a percentage of DMSO vehicle (*n* = 3(3–6); ***P ≤ 0.001; ****P < 0.0001). **i** Immunoblot of indicated prote**i**ns in SUM149 cells treated with vehicle or Merestinib at dosages 0.1 and 1 uM for 24 h. GAPDH was used as a loading control.

The aforementioned datasets identify altered expression of ribosomal proteins, which are known to be integral in the MAPK/eI4FE mediated protein translation during stress (Figs. 1; 7a), upon acquisition of drug resistance. Furthermore, MAPK hyperactivation is a hallmark of IBC patient tumors, which is recapitulated in our preclinical model of drug resistance with sustained pERK levels despite suppression of pEGFR by the EGFR-TKI (Lapatinib) treatment (Fig. 7b). Therefore, we tested the effect of Merestinib, a multikinase inhibitor currently in cancer clinical trials^18^ (relevant to this study shown to be active against receptor tyrosine kinases, serine/threonine kinases, inhibit MNK induced phosphorylation of eIF4E) to target this translational signaling as a single agent and in combination with Lapatinib. Based on the dose-response data with Merestinib alone (calculated IC50 = 830 nM; Fig. 7c), we treated the cells with 100 and 1000 nM doses and observed a significant decrease in cell viability in both SUM149 (*p* = 0.0001) and in rrSUM149 (*p* < 0.0001) cells (Fig. 7d). Next, we treated SUM149 with both Merestinib, in a non-constant ratio, and Lapatinib (Fig. 7e) to assess if these two agents have any combinatorial effect using the Chou–Talalay method, a derivation of the mass–action law principle. The dose-effect graph (Fig. 7f) and isobologram (Fig. 7g) reveal the combination index (CI) to be <1 at 250–1000 nM doses, which is indicative of drug synergism and an additive effect (CI = 1) at 100 nM. Furthermore, in the case of multidrug-resistant rSUM149 cells maintained in culture in the presence of pEGFR TKI (Lapatinib), the addition of Merestinib caused a significant inhibition of cell viability (*p* < 0.0001) (Fig. 7h).

Immunoblot analysis Fig. 7i of Merestinib-treated lysates showed significant inhibition of pERK and peIF4E along with decreases in CDK1, XIAP, and SOD2 protein levels. This correlated with the upregulation of these prosurvival, anti-apoptotic, and antioxidant proteins in rSUM149 multidrug resistant cells as observed in immunoblot analysis of the three cell lines at basal conditions (Fig. 7b). Next, we evaluated the expression of IMP3 as a proof of principle ribosomal protein relevant to this study, as it has been shown to act as an RNA destabilizing factor resulting in increased eIF4E mediated translation and cell proliferation^19^ and it is upregulated during EGFR-mediated MAPK activation in triple-negative breast cancer cells^20^. Immunoblot analysis (Fig. 7i) shows significant inhibition of IMP3 in Merestinib-treated lysates. Notably, both IMP3 mRNA expression and ERK/MAPK activation were respectively positively and negatively correlated with the ribosomal activity score in our series of patients with IBC with and without pCR (Fig. 6d, e).

Taken together, these data reveal Merestinib’s ability to sensitize the multidrug-resistant rSUM149 cells and potentiate cell death in the parental and resistant reversal cells by targeting multiple signaling inputs to the protein translational machinery.

## Discussion

IBC is a complex and heterogeneous disease, characterized by a dismal prognosis and significantly lower overall survival compared to non-IBC at both stage III and stage IV breast cancer^5,21^. Considering IBC’s designation as a cancer health disparity due to disproportionately higher incidence and treatment resistance observed in minoritized populations and younger women, there is a critical need to identify biomarkers and targeted therapeutic options^5,22^. We report herein a comprehensive characterization of an IBC progression model composed of treatment naïve, treatment-resistant, and resistance reversal cell lines using an integrated approach that analyzes RNA sequencing and proteomics datasets. Our results show proliferative signatures that are similar in SUM149 and rSUM149. In contrast, limited differences between SUM149 and rrSUM149 when compared with rSUM149 indicate a nearly complete normalization of rrSUM149 to the profile of SUM149. This supports our previous observation of a return to a state in rrSUM149 that responds or is “resensitized” to many agents in the NCI-DTP oncology drug panel^13^. Retrospective patient studies in diverse tumor types have demonstrated that individuals who took a treatment break after experiencing disease progression showed more favorable treatment responses^15–17^. This suggests that a period of discontinuation and subsequent reintroduction of therapy can lead to improved outcomes in these patients. Indeed, the concept of drug holiday has recently gained increasing interest during the COVID-19 pandemic, when many patients due to fear of coming in to clinics for treatment took a break from therapy, akin to a drug holiday albeit unplanned^23^.

Our current study demonstrates transcriptional and translational changes across the IBC progression model, identifying differential expression of proteins involved in ribosomal processes across the different contrasts. Most importantly, genes associated with ribosomal processes were significantly enriched amongst the group of genes overexpressed in IBC patients with pCR. This ribosomal heterogeneity in expression and activity has the potential for selective pressure and outgrowth of clonal populations of tumor cells that exhibit treatment-resistant phenotypes. This is apparent in our datasets, wherein a suppressed ribosomal protein profile (Fig. 5) is observed in the acquired drug-resistant model (rSUM149) and supported by prior studies reporting temporal impact on ribosomal protein activation in tumor cells exposed to chronic stressors including resistance to chemotherapeutic agents that are associated with redox status^24–27^.

Protein synthesis, a highly regulated and ordered process, involves ribosomes and the recruitment of a set of translation factors and ribosomal proteins that respond to stress in the surrounding environment by coregulating gene networks to conserve energy while initiating DNA repair processes^28^. Thus, differential expression of ribosomal proteins can impair cell survival, growth, proliferation, and other extra-ribosomal functions, including DNA repair during cellular stress, accumulation of reactive oxygen species, and apoptotic sensitivity to therapeutic agents. However, tumor cells often exhibit a heightened threshold to withstand stress stimuli and continue protein synthesis of key proteins involved in cell survival by initiating the alternate cap-independent, internal ribosome entry site (IRES) mediated protein translation^29^. Here, and in our previous studies, one such protein upregulated in rSUM149 and reversed to pretreatment levels in rrSUM149 is XIAP. XIAP acts as a stress sensor during death stimuli, including when tumor cells are exposed to therapeutic agents, as it is translationally upregulated *via* a unique IRES element in its 5′ untranslated RNA^30^. Furthermore, IBC patient tumors have also been observed to express high levels of eIF4G, a translational initiation factor, that is involved in the modulation of protein synthetic machinery for increased translation of mRNAs with internal ribosome entry sites (IRESs) like XIAP during cellular stress^31^. XIAP has the ability to bridge MAPK activation and downstream NFkB prosurvival signaling and increase antioxidants, leading to aggressive disease progression, tolerance to oxidative stress, and therapeutic resistance^32,33^. Many of the anti-cancer agents (e.g., oxaliplatin, cisplatin, actinomycin D, 5-FU, poly-ADP ribose polymerase/PARP inhibitors) kill cells by predominantly inducing DNA damage and engage various mechanisms related to rRNA synthesis, processing and/or ribosome biogenesis^25,34^. Indeed, rrSUM149 cells regain sensitivity to various chemotherapeutics and kinase inhibitors but remain resistant when treated with platinum-based drugs, certain alkylating and topoisomerase inhibitors^13^. Interestingly, despite a small number of differentially expressed genes in resistance reversal cells compared to the parental cells, our data show a large number of differences in protein expression, which suggests the involvement of the epigenome that needs to be explored in future studies.

This study identifies ribosomal proteins that can be developed as prognostic markers for an understudied, rare cancer. Recent studies in various cancers, including breast, report altered gene expression of several of the identified ribosomal proteins in poor survival outcomes^35^, and preclinical models like the ones described in the current study can be used for understanding the underlying mechanisms of translational control in tumor cells. The set of RPs (TP53, AGO2, XPO11, YWHAZ, RPL5, and FXR2) that our analysis shows to be similar in expression in both rrSUM149 and rSUM149 are known to play key roles in regulating cellular stress responses and in the transport of tumor suppressive proteins^34^.

Our results showing the efficacy of Merestinib^18,36^ in suppressing not only MAPK-eIF4E signals but also CDK1 and the oncofetal RNA binding protein, IMP3, which is induced by MAPK signals and overexpressed linked to many types of aggressive tumors^37^ are highly significant toward developing IBC-specific therapeutic strategies. Furthermore, based on recent studies reporting cell cycle-independent function of CDK1 in coupling proliferation with activation of eIF4E protein synthesis^38^ via its ability to phosphorylate translational regulators/repressors including eIF4BP1^39^, LARP1 involved in regulating 5’ Terminal OligoPyrimidine (5′ TOP) motif-containing mRNAs^40,41^, RPS3^42^, we envision employing this preclinical IBC model to evaluate the efficacy of CDK1 inhibitors alone and in combination with TKIs and other chemotherapeutics.

Due to the rarity of IBC and the challenges associated with obtaining tumor tissue samples from patients, this longitudinal preclinical model that recapitulates adaptive changes in response to therapeutic stress can serve as IBC patient’s avatars for investigation of the intricate aspects of acquired resistance, relapse, and in pharmacological interrogation for precision medicine applications.

## Methods

### Cell culture

SUM149PT cells were obtained from Asterand Inc. (Detroit, MI) and were routinely cultured in Ham’s F12 medium (Mediatech Inc., Manassas, VA) supplemented with 5% FBS (Atlanta Biologicals, Lawrenceville, GA), 1% penicillin/streptomycin, 1% antibiotic/antimycotic, hydrocortisone (Invitrogen, Carlsbad, CA) and an insulin/transferrin/selenium cocktail (Gibco, Carlsbad, CA). To generate the resistance model rSUM149, SUM149 cells were routinely maintained in a normal culture medium supplemented with 7.5 μM research grade Lapatinib (GW572016, Selleckchem, Houston, TX) in increasing concentrations (0.25– 7.5 μM) for a period of 3 months. Cells were grown for two weeks in each dosage concentration. Following an initial decrease in cell growth and cell death, small colonies of viable cells were seen by the end of the two weeks and were cultured to confluence before the next drug concentration increased. The resistance reversal model rrSUM149 was generated from rSUM149 after removal of Lapatinib >3 months and were cultured in regular SUM149 media. All cell lines were cultured at 37 °C with 5% CO_2_ for no more than 2 months prior to use in this study.

### RNA extraction

Monolayer cells of SUM149, rSUM149, and rrSUM149 (*n* = 3 for each cell line) seeded with their respective medium in 100 mm dishes (250,000 cells/dish) were harvested 72 h after they were seeded. Monolayer cells were lifted using a cell scraper (Corning, Corning, NY) instead of trypsin-EDTA. After collection, monolayer cells were pelleted, washed with ice-cold PBS, and re-pelleted to be stored at −80 °C until RNA extraction. Total RNA was extracted was conducted at Azenta US, Inc (South Plainfield, NJ, USA) using Qiagen RNeasy Plus Universal mini kit (Qiagen, Hilden, Germany) following the manufacturer’s instructions. Extracted RNA samples were quantified using Qubit 2.0 Fluorometer (Life Technologies, Carlsbad, CA, USA) and RNA integrity was checked using Agilent TapeStation 4200 (Agilent Technologies, Palo Alto, CA, USA).

### RNA library preparation and sequencing

RNA sequencing libraries (*n* = 15) were prepared using the NEBNext Ultra II RNA Library Prep Kit for Illumina following the manufacturer’s instructions (NEB, Ipswich, MA, USA). Briefly, mRNAs were enriched with Oligo(dT) beads and fragmented for 15 min at 94 °C. First-strand and second-strand cDNAs were subsequently synthesized. cDNA fragments were end-repaired and adenylated at 3′ends, and universal adapters were ligated to cDNA fragments, followed by index addition and library enrichment by limited-cycle PCR. The sequencing libraries were validated on the Agilent TapeStation (Agilent Technologies, Palo Alto, CA, USA), and quantified by using Qubit 2.0 Fluorometer (Invitrogen, Carlsbad, CA) as well as by quantitative PCR (KAPA Biosystems, Wil-mington, MA, USA). Library preparation was conducted at Azenta US, Inc (South Plainfield, NJ, USA).

The sequencing libraries were clustered on a flowcell lane. After clustering, the flow cell was loaded on the Illumina HiSeq instrument (4000 or equivalent) according to the manufacturer’s instructions. The samples were sequenced using a 2 × 150 bp paired-end (PE) configuration. Image analysis and base calling were conducted by the HiSeq Control Software (HCS). Raw sequence data (.bcl files) generated from Illumina HiSeq was converted into fastq files and de-multiplexed using Illumina’s bcl2fastq 2.17 software. One mismatch was allowed for index sequence identification. Sequencing was conducted at Azenta US, Inc (South Plainfield, NJ, USA).

### Protein extraction

Monolayer cells of SUM149, rSUM149, and rrSUM149 (*n* = 3 for each cell line) seeded with their respective medium in 100 mm dishes (250,000 cells/dish) were harvested 72 h after they were seeded. Monolayer cells were lifted with 2 mL of 0.25% trypsin-EDTA and collected. Cells were then pelleted, washed twice with PBS, and lysed with 150 mM sodium chloride, 1% Triton X-100, 50 mM Tris pH 8.0 with 1X Halt Protease Inhibitor Cocktail (Thermo Fisher, Waltham, MA) and 2 mM DTT (Thermo Fisher, Waltham, MA) for western immunoblotting or RIPA buffer (Sigma, St. Louis, MO) for mass spectrometry.

### Protein sample preparation and quantitative mass spectrometry

Monolayer cells seeded at 250,000 cells/well at 72 h were lysed with RIPA buffer (Sigma, St. Louis, MO; catalog R0278), and a BCA assay (Pierce) was performed on supernatants. Up to 25 μg of protein was normalized with 5% SDS and (w/v) triethylammonium bicarbonate buffer, pH 8.5 (TEAB) followed by addition of 10 mM DTT and reduction at 80 °C for 15 min. After cooling, samples were alkylated with 25 mM iodoacetamide in the dark for 30 min at room temperature. SDS was removed, and samples were digested with 1:15 of Sequencing Grade Modified Trypsin (Promega) using an S-trap micro device (Protifi) according to the manufacturer’s instruction (digestions at 47 °C for 1 h). Recovered peptides were lyophilized to dryness and reconstituted at 0.5 μg/μL in 1% trifluoroacetic acid (TFA) and 2% acetonitrile (MeCN). Equal volumes of each sample were mixed to generate a study pool quality control (SPQC) sample.

One-dimensional liquid chromatography and tandem mass spectrometry (1D-LC-MS/MS) was performed on 1.5 μL of each sample in a block-randomized order. Samples were analyzed using an M-Class UPLC system (Waters) coupled to a coupled to a Fusion Lumos mass spectrometer (Thermo) via a Nanospray Flex ionization source. Samples were first trapped on a Symmetry C18 180 μm × 20 mm trapping column (5 μl/min at 99.9/0.1 v/v H2O/MeCN) followed by an analytical separation using a 1.7 μm ACQUITY HSS T3 C18 75 μm × 250 mm column (Waters) with a 90 min gradient of 5 to 30% MeCN with 0.1% formic acid at a flow rate of 400 nl/min and column temperature of 55 °C. MS data were collected using BoxCar data-independent acquisition (BoxCarDIA) with variable window BoxCar and DIA scans defined based on scouting runs. Each cycle utilized a full scan with 120,000 resolution from m/z 400 to 1200 with a normalized AGC target of 300% and 50 ms injection time (IT), two BoxCar scans at 120 K resolution with 10 windows each, 100% AGC and auto IT, and a 23 window variable window DIA scan with 30 K resolution, AGC target of 1000%, a 60 ms IT and NCE of 33. All data were collected in centroid mode.

### Gene expression analysis

Raw reads were mapped onto the reference genome (hg38) using the splice-aware aligner HISAT2. Resulting SAM-files were converted into BAM-files and position sorted using samtools. Reads overlapping with the genomic positions of genes were counted using summariseOverlaps-function in the BioC-package GenomicAlignments. Next, genes with more than 10 counts in at least 20% of the samples were retained, vouching for a total of 14,540 unique genes. To explore global differences in mRNA expression, principal component analysis (PCA) was performed.

Differences in gene expression were analyzed using negative binomial models (BioC-package DESeq2). The design matrix was set up using a model containing only cell type (i.e., SUM149, rSUM149, and rrSUM149) as an independent variable and without intercept terms. Genes differentially expressed between all pairs of cell types were identified. Only genes with a false discovery rate (FDR)-corrected *p* value inferior to 10% were considered significant. Results are represented in volcano plot format. List of differentially expressed genes were compared using the Jaccard Index (JI; BioC-package GeneOverlap) and vectors of log2-transformed fold-changes using the Pearson correlation coefficient.

To identify regulators of differential gene expression in each of the evaluated conditions, the BioC-package VIPER was used. The VIPER algorithm virtually infers protein activity levels of both transcription factors and signal transduction proteins based on target gene mRNA expression. It considers the mode of action (i.e. activation or suppression), the regulator-target gene interaction confidence, and the pleiotropic nature of each target gene regulation. The VIPER algorithm was run without a null model and with lists of target genes for 6053 regulators in a background specific for breast cancer cells (BioC-package aracne.networks). Prior to the VIPER analysis, nominal *P* values resulting from differential gene expression analysis (vide supra) were Z-transformed. Secondary to the core VIPER analysis, leading-edge genes were identified, and a shadow analysis was performed to identify pleiotropic interactions amongst significant regulators. Pleiotropic interactions were visualized as interaction maps using the R-packages igraph, ggraph, and tidygraph. Proteins with an in-degree of at least 1 indicative of a shadow effect and identified through topological analysis of the interaction maps, were labeled throughout the analysis. Furthermore, proteins with an FDR-corrected *P* value inferior to 10% and an absolute normalized enrichment score (NES) greater than 5 were subjected to overrepresentation analysis (ORA) for the WikiPathways gene sets using the BioC-package fgsea. Gene sets with a minimal size of 40 and a nominal *P* value inferior to 5% were considered significant.

### Protein expression analysis

Proteomics data were processed using Spectronaut v. 15.5.211111.50606 (Biognosys). Raw files were converted to .htrms using HTRMS converter. A spectral library was generated using direct-DIA searching with Spectronaut Pulsar and a SwissProt database with *Homo sapiens* taxonomy and with the addition of porcine trypsin and BSA (20,385 entries). Database searching used default parameters, including trypsin/P specificity, up to two missed cleavages, N-terminal protein acetylation, and Met oxidation. For protein quantification, .htrms files were analyzed in Spectronaut using default settings except that workflow settings used iRT profiling of non-identified precursors, protein quantification used the MaxLFQ algorithm and *q* value filtering with 0.2 percentile. Local normalization was applied using precursors identified in all runs (*q* value complete).

Prior to analysis, between array quantile normalization was performed using the limma package. To explore global differences in protein expression, PCA was performed. Differences in protein expression were analyzed using generalized linear models containing only cell type (i.e., SUM149, rSUM149, and rrSUM149) as an independent variable and without intercept terms. Proteins differentially expressed between all pairs of cell types were identified. Only proteins with an FDR-corrected *p* value <0.1 were considered significant. Results are represented in volcano plot format. Significant proteins were subjected to ORA for the WikiPathways gene sets (using the BioC-package fgsea). Gene sets with a minimal size of 40 and a nominal *P* value inferior to 5% were considered significant.

### Pathway and network analysis

Datasets of differentially activated proteins identified using VIPER and through proteomic analysis were integrated through protein-protein interaction (PPI) networks to unravel signal transduction mechanisms of resistance and resistance reversal in SUM149 cells. For this analysis, proteins with a significant difference in expression (FDR <10%) and virtual protein activity (FDR <10% and absolute NES >5) in the same direction were used as seeds and mapped onto the PPI network STRING that was filtered to contain only physical interactions with a minimal score of 0.4 and that was downloaded from the website (https://www.string-db.org) and converted into a igraph object for manipulation in R (R-package igraph). Using the STRING PPI network, all shortest paths between each pair of seed proteins was calculated. To evaluate the significance, the shortest path analysis was repeated for the same set of input proteins on ten randomized networks with identical node degree distribution. The subgraph connecting all pairs of proteins was used to calculate node degree and centrality statistics and was subjected to Louvain clustering to detect communities. For each community as well as for the full subgraph, overrepresentation analysis (ORA – BioC-package fgsea) was performed for WikiPathways gene sets. These gene sets were chosen to obtain additional granularity as compared to the Hallmark gene set and results were collapsed to identify parental gene sets. Enrichment in the full subgraph was tested against all proteins in the STRING network, whereas enrichment in the communities was tested against all proteins in the subgraph. For each of the communities, global changes in protein expression and/or activity between conditions of interest were also analyzed. Networks are visualized R-packages tidygraph, ggraph, and ggpubr.

### Western immunoblotting

IBC cells were seeded in 100 mm dishes (250,000 cells/dish) and allowed to adhere overnight. Cells were treated with 0.01% DMSO and Merestinib (Selleckchem, Houston, TX) at dosages 100 nM and 1000 nM for 24 h. Following treatment, cells were collected and lysed using 150 mM sodium chloride, 1% Triton X-100, 50 mM Tris pH 8.0 with 100X Halt Protease Inhibitor Cocktail (Thermo Fisher, Waltham, MA), and 1 M DTT (Thermo Fisher, Waltham, MA). Protein concentration was determined using the Pierce 660 nm Protein Assay Reagent (Thermo Fisher, Waltham, MA) and read at 660 nm with the Nanodrop 2000 (Thermo Fisher, Waltham, MA). Cell lysates were boiled for 6 min and immediately cooled on ice. The lysates were then subject to gel electrophoresis on NuPage 4–12% Bis-Tris gels (Invitrogen, Waltham, MA) with MES SDS Page running buffer (Invitrogen, Waltham, MA). The protein was transferred onto a nitrocellulose blotting membrane (Amersham, St. Louis, MO) previously soaked in transfer buffer by the XCell II Blot Module transfer cell (Life Technologies, Carlsbad, CA). After the transfer, the membranes were incubated with a blocking buffer (5% BSA in 1X TBS-0.1% Tween 20) for 1 h in room temperature. Membranes were incubated with primary antibodies for peIF4E, eIF4E, pERK, ERK, CDK1, SOD2 (all 1:1000, Cell Signaling Technology), or GAPDH (1:5000, Santa Cruz Biotechnology) overnight at 4 °C. Membranes were washed and incubated with anti-mouse or anti-rabbit HRP-conjugated antibodies (Santa Cruz Biotechnology, Dallas, TX) for 1 h at room temperature. Chemiluminescent reagent (Thermo Scientific, Waltham, MA) and LI-COR Odyssey FC imager with Image Studio software (LI-COR) was used for detection as previously described^43^. Molecular weight standards included precision plus protein kaleidoscope prestained (BIORAD, Cat #: 1610375), Invitrogen MagicMark XP (Invitrogen, Cat #: LC5603). Densitometric analysis was performed using the Image Studio Lite software. The full membranes are shown in Supplementary Fig. 5.

### Luciferase activity

SUM149, rSUM149, and rrSUM149 cells expressing green fluorescent protein and Luciferase dual reporters were generated by stably transducing with lentivirus expressing GFP using the pBMN(CMV-copGFP-Luc2-Puro plasmid (Addgene plasmid # 80389) and co-transfected with pMD2.G VSVG envelope expressing plasmid (Addgene plasmid #12259) and psPAX2 (Addgene plasmid #12260), both second-generation lentiviral packaging plasmids. Briefly, 293T cells were transfected with constructs using jetPRIME® Packaging Mix (Polyplus, NY, USA) for lentiviruses production, according to the manufacturer’s instructions. The supernatants containing the lentiviral particles were collected 72 h after transfection. Target cells were infected with lentiviral particles following the manufacturer’s instructions and selected using 1 μg/ml puromycin (Gibco, Thermo Fisher). Approximately 1 week later, antibiotic-resistant cells were expanded and maintained in puromycin-supplemented growth media. Cell infection was considered effective when the percentage of GFP-positive cells and luciferase activity was >95%. For luciferase assays, cells were seeded at a density of 4000 cells/well in 96-well plates and allowed to adhere overnight. Treatments included 0.01% DMSO vehicle control, EGFR-TKI, Lapatinib, (3.75 and 7.5 μM) as previously described^11,13^, Merestinib dose range (0.01, 0.1, 0.25, 0.5, 0.75, 1, and 2 μM) or a combination of non-constant ratio of Merestinib (0.1, 0.25, 0.55, and 1 μM) with 7.5 μM Lapatinib. After 24 h of treatment, 100 uL of Bright-Glo luciferase detection mix (Promega) was added to each well. Plates were incubated in room temperature for 5 min, and luminescence intensity was measured by a FLUOstar OPTIMA reader (BMG Labtech).

### Drug synergism analysis

Drug synergism was performed using the CompuSyn software developed using the Chou–Talalay method as previously described^44–46^ where a combination index or CI <1 indicates synergism, =1 indicates additive effect, and >1 indicates antagonism.

### Breast cancer samples and expression profiling

We queried the publicly available gene expression data and pathological responses to neoadjuvant chemotherapy from patients with IBC (*N* = 87), which were collected by the Inflammatory Breast Cancer International Consortium (IBC-IC) as described previously^47,48^. Gene expression differences between patients with and without pathological complete responses (pCR) were calculated using generalized linear models using the BioC-package limma. Then, the vector of gene-wise fold-changes was used to evaluate the enrichment of ribosomal gene sets using the BioC-package fgsea. Ribosomal gene sets were defined using the gene ontology category of the Molecular Signatures Database (Broad Institute). In addition, sets of ribosomal genes associated with the acquisition of resistance and resistance reversal in SUM149 cells treated with Lapatinib were also tested. Nominal *p* values inferior to 5% were considered significant with positive normalized enrichment scores indicating genes set that are enriched amongst genes overexpressed in patients with pCR. Results were visualized using enrichment plots and dotplots in ggplot2. In addition, the ribosomal activity score was calculated using gene set variation analysis (GSVA; BioC-package GSVA) with Gaussian kernels. For this analysis, the leading-edge genes identified through gene set enrichment analysis (vide supra) for all ribosomal genes from gene ontology were utilized. Those leading-edge genes constitute the set of genes that contribute most to the enrichment signal. In addition, based on a set of ERK/MAPK target genes from Reactome, activity scores for this pathway were also calculated using GSVA with identical settings.

### Supplementary information

Supplemental Figures and tables

## Data Availability

The datasets used in this manuscript are available from the corresponding repositories without restrictions. Gene expression data (i.e., FASTQ-files, BAM-files, and associated metadata) of the SUM149, rSUM149, and rrSUM149 cell lines have been deposited to ArrayExpress (E-MTAB-13929). Proteomic data, including raw files, associated metadata, and spectral libraries are available at the ProteomeXchange Consortium (PXD050448) via the MassIVE repository (ftp://massive.ucsd.edu/v07/MSV000094251/). Gene expression data (i.e., CEL-files and associated metadata) of the IBC-IC patient series have been deposited to ArrayExpress (MTAB-1006 and E-MTAB-1547) and the Gene Expression Omnibus (E-GEOD-22597).

## Code availability

The code used for data analysis is accessible without restrictions through the corresponding author’s github page: https://github.com/StevenVanLaere/IBCModels.
