# Supplementary material for: Altered ribosomal profile in acquired resistance and reversal associates with pathological response to chemotherapy in inflammatory breast cancer
Source: NPJ Breast Cancer. 2024 Jul 29;10:65. doi: 10.1038/s41523-024-00664-0 (PMC11286775; doi:10.1038/s41523-024-00664-0)
Supplement: Supplementary file 1 — Supplemental Figures and tables [file 41523_2024_664_MOESM1_ESM.pdf]

Supplementary Data

Supplementary Figure 1: a. Results of the principal component analysis using the mRNA expression data set are shown in scatter plot format with the X- and Y-axis representing PC1 and PC2 respectively. Dots are color-coded as shown by the legend underneath, recapitulating the different SUM149 isogenic cell lines. **b.** Results of the principal component analysis using the protein expression data set are shown in scatter plot format with the X- and Y-axis representing PC1 and PC2 respectively. Dots are color-coded similarly to panel A.

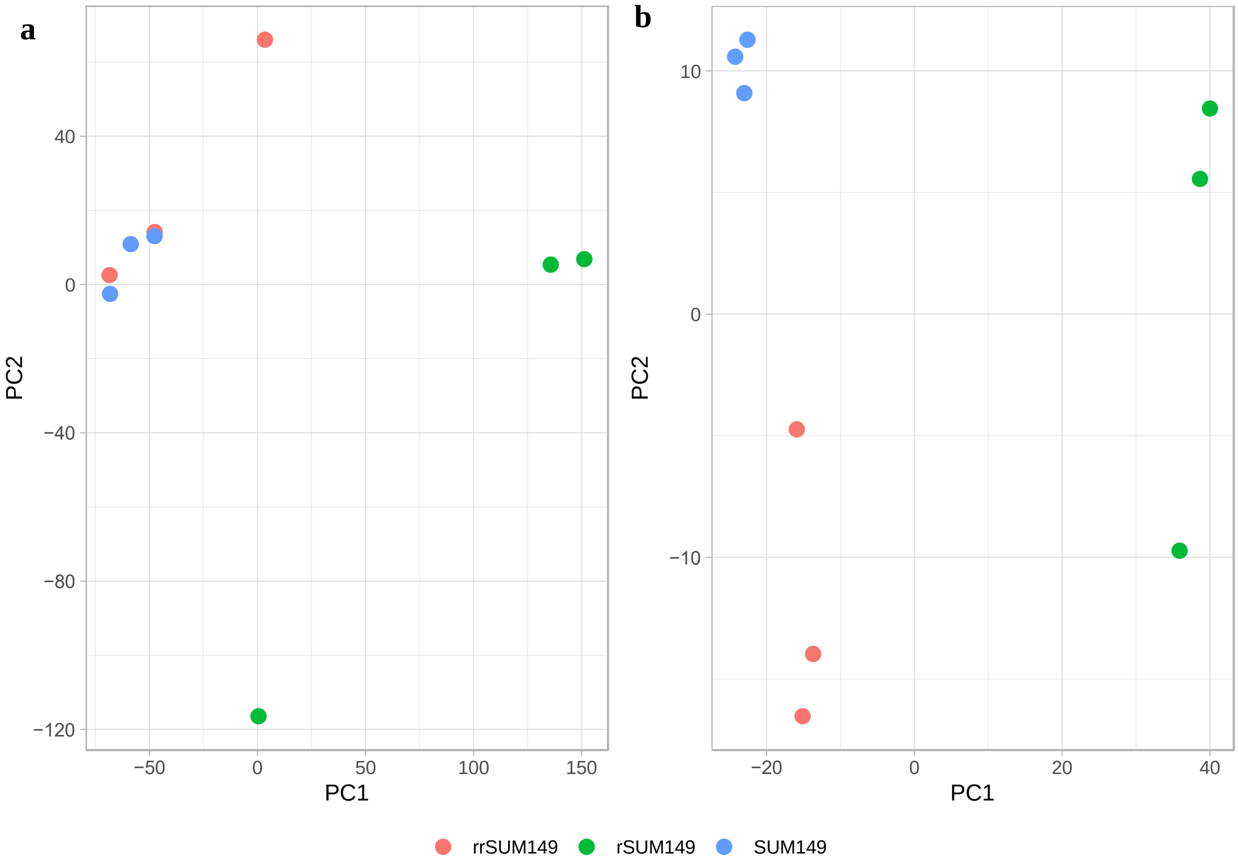

Supplementary Figure 2: Plots of the top 20 differentially activated proteins identified from the integrated pathway analysis per contrast. **a.** SUM149 and rSUM149; **b.** rSUM149 and rrSUM149; c**.** SUM149 and rrSUM149. For each heatmap, the p-value of the enrichment, the enrichment profile or the protein's target genes with every gene being indicated by a red or blue tick, the name of the protein regulating the target gene set (i.e. Set), the inferred activity score of the protein (i.e. Act) and the associated mRNA expression (i.e. Exp) are provided. Red and blue colors denote respectively high and low expression (i.e for Exp), activity (for Act), or a positively/negatively regulated target gene (i.e. enrichment profile).

a

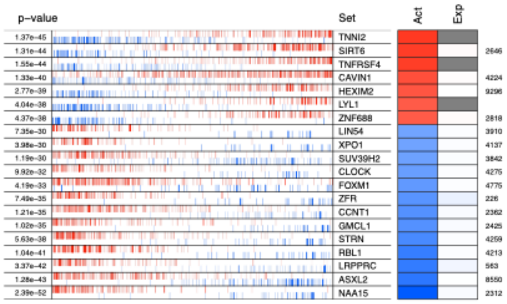

b

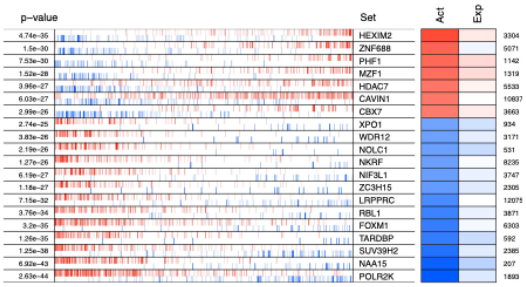

c

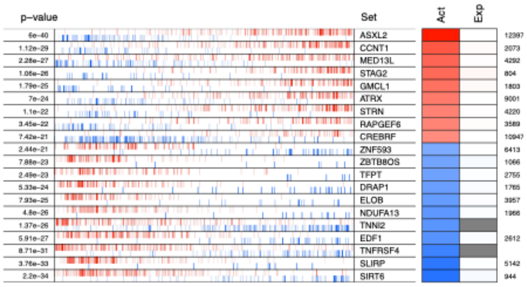

Supplementary Figure 3: Network plots showing pleiotropic interactions from the integrated pathway analysis are shown. Edges indicate pleiotropic interactions between master regulators exist. Colors of the dots indicate the activity change between conditions of interest: **a.** rSUM149 vs. SUM149; **b.** rrSUM149 vs. rSUM149; and **c.** rrSUM149 vs. SUM149. Activity changes are color-coded red to blue denoting higher to lower activities in the first relative to the last condition.

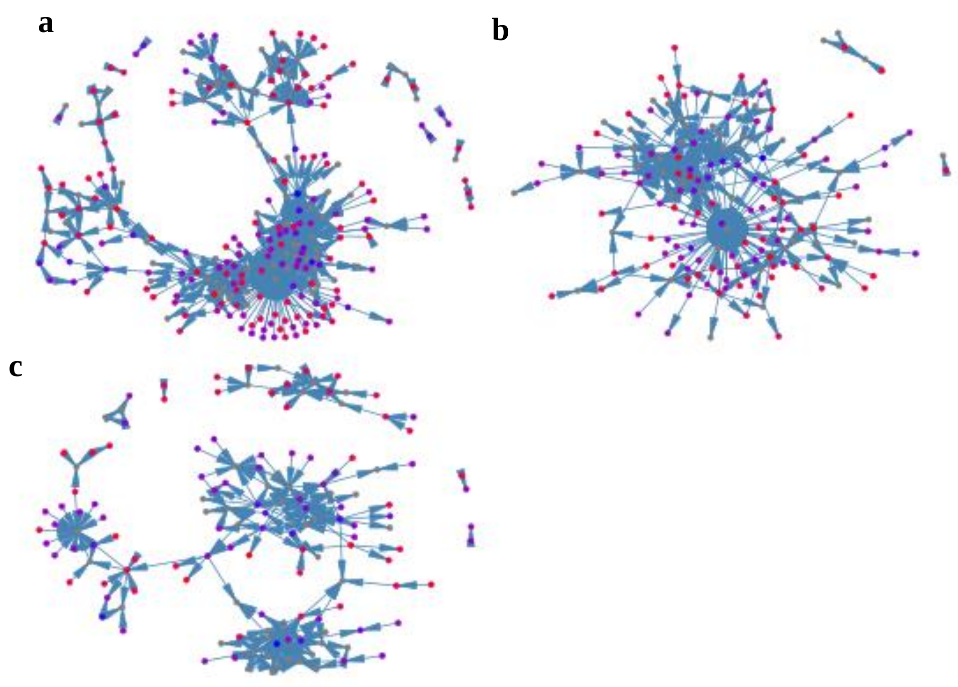

Supplementary Figure 4: Graphs of all interactions between proteins involved in

a. resistance acquisition (SUM149 vs. rSUM149; 1,626 proteins and 32,262 edges),

b. resistance reversal (SUM149 vs. rrSUM149 cells; 1,051 proteins and 16,528 edges),

c. between SUM149 vs. rrSUM149 cells (i.e. 105 proteins and 475 edges) are shown.

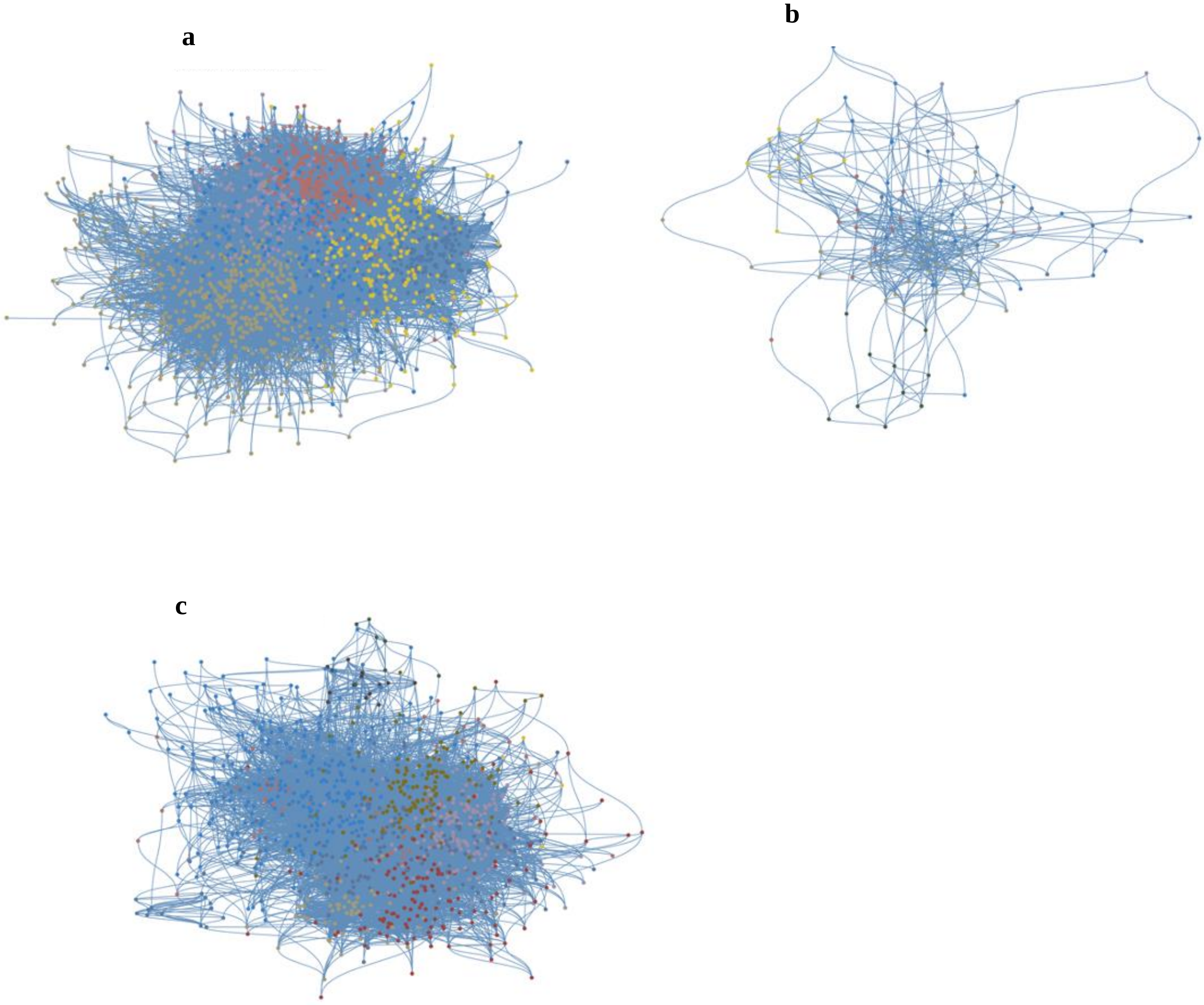

Supplementary Figure 5: Uncropped WIB images for figure 6b and 6i.

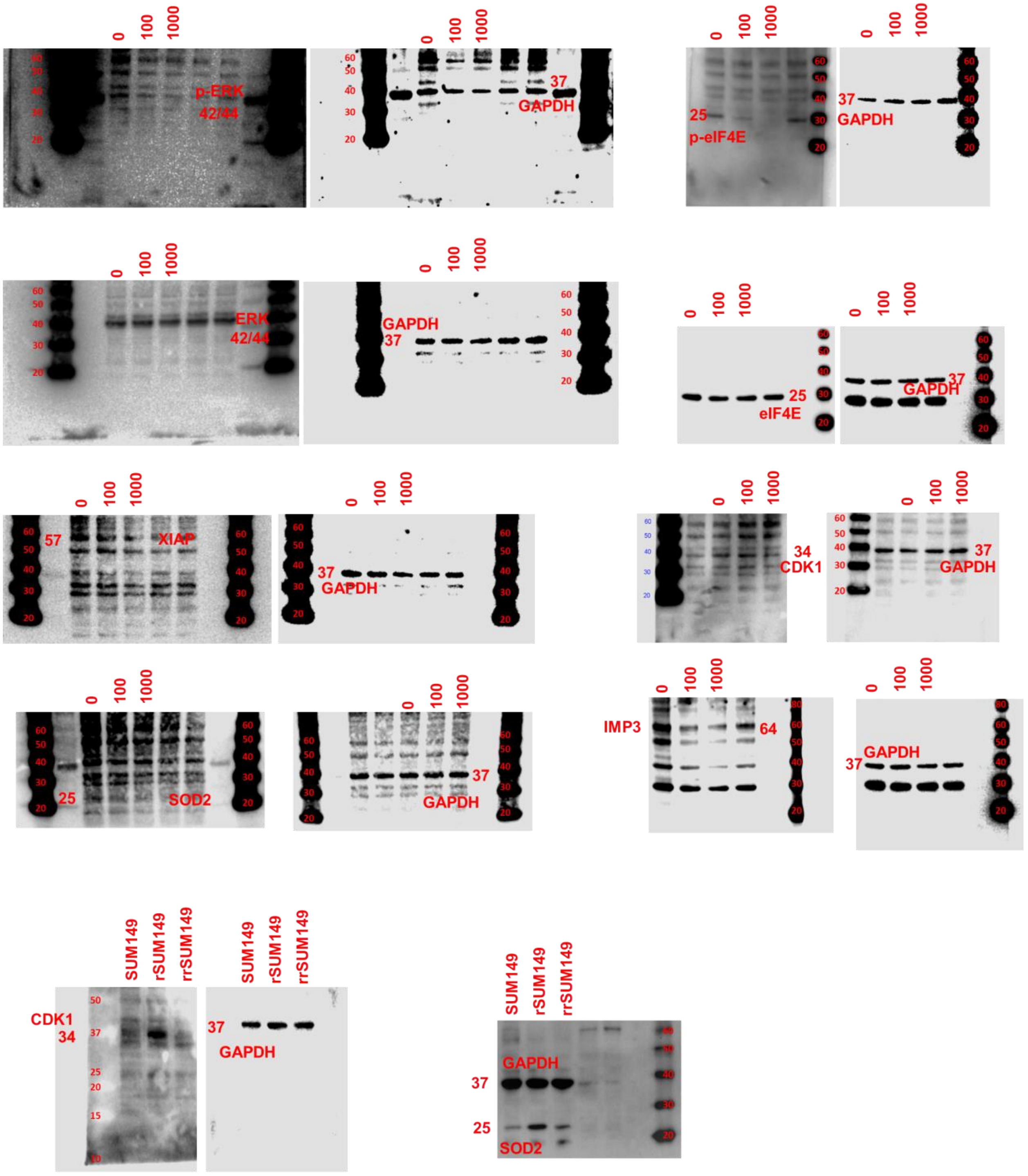

Supplementary Table 1: Differential gene expression statistics for all comparisons amongst SUM149, rSUM149 and rrSUM149 are provided.

	rSUM149 vs. SUM149					rrSUM149 vs. SUM149					rrSUM149 vs. rSUM149				
SYMBOL	*<Expression>*	*logFC*	*SE*	*P-Value*	*FDR*	*<Expression>*	*logFC*	*SE*	*P-Value*	*FDR*	*<Expression>*	*logFC*	*SE*	*P-Value*	*FDR*
A1BG	104.508	1.511	0.331	0.000	0.000	104.508	0.850	0.335	0.011	0.302	104.508	-0.661	0.380	0.082	0.247
ADA	448.744	0.633	0.259	0.015	0.045	448.744	0.283	0.259	0.275	0.545	448.744	-0.350	0.299	0.242	0.473
CDH2	1046.065	-0.267	0.162	0.100	0.195	1046.065	-0.005	0.162	0.976	0.987	1046.065	0.262	0.187	0.162	0.375
AKT3	802.391	-0.192	0.177	0.279	0.416	802.391	-0.218	0.177	0.219	0.498	802.391	-0.026	0.205	0.899	0.953
ZBTB11-AS1	173.525	-0.763	0.237	0.001	0.007	173.525	-0.325	0.232	0.161	0.452	173.525	0.438	0.274	0.109	0.295
MED6	387.972	-0.339	0.231	0.143	0.255	387.972	-0.486	0.231	0.035	0.324	387.972	-0.147	0.268	0.583	0.765
DDTL	2038.829	0.763	0.243	0.002	0.009	2038.829	0.253	0.244	0.300	0.566	2038.829	-0.510	0.281	0.069	0.225
GUSBP9	67.240	-0.221	0.341	0.517	0.644	67.240	0.285	0.334	0.395	0.639	67.240	0.506	0.391	0.196	0.419
ACOT8	218.844	0.618	0.237	0.009	0.032	218.844	0.134	0.238	0.574	0.759	218.844	-0.484	0.274	0.078	0.241
ABI1	1010.819	-0.580	0.170	0.001	0.004	1010.819	-0.110	0.169	0.514	0.720	1010.819	0.470	0.196	0.017	0.091
GNPDA1	1224.099	0.386	0.260	0.139	0.249	1224.099	-0.544	0.261	0.037	0.325	1224.099	-0.930	0.301	0.002	0.022
KCNE3	35.269	0.661	0.315	0.036	0.090	35.269	0.328	0.319	0.304	0.570	35.269	-0.333	0.363	0.359	0.591
ZBTB33	820.535	-0.832	0.234	0.000	0.003	820.535	-0.028	0.233	0.905	0.952	820.535	0.804	0.270	0.003	0.028
SNHG8	74.831	0.689	0.362	0.057	0.128	74.831	0.387	0.363	0.287	0.556	74.831	-0.302	0.418	0.469	0.684
CDH3	59.578	1.520	0.426	0.000	0.003	59.578	0.762	0.431	0.077	0.368	59.578	-0.758	0.490	0.122	0.313
TANK	542.485	-0.085	0.166	0.608	0.720	542.485	0.114	0.165	0.489	0.705	542.485	0.199	0.191	0.298	0.533
POM121C	1489.575	-0.277	0.129	0.032	0.082	1489.575	-0.135	0.129	0.293	0.561	1489.575	0.142	0.149	0.341	0.575
PMS2P7	96.973	-0.260	0.215	0.226	0.358	96.973	-0.232	0.212	0.275	0.545	96.973	0.028	0.250	0.910	0.959
ZSCAN30	129.841	0.288	0.340	0.397	0.535	129.841	0.460	0.338	0.174	0.463	129.841	0.172	0.391	0.660	0.818
SRA1	798.528	-0.140	0.383	0.715	0.805	798.528	-0.738	0.384	0.054	0.342	798.528	-0.598	0.443	0.177	0.394
UCKL1-AS1	45.966	0.114	0.241	0.636	0.744	45.966	0.081	0.238	0.733	0.857	45.966	-0.033	0.278	0.905	0.957
TMEM170B	11.589	4.239	0.573	0.000	0.000	11.589	1.758	0.639	0.006	0.297	11.589	-2.481	0.577	0.000	0.001
HOTAIR	41.649	1.039	0.363	0.004	0.017	41.649	0.666	0.366	0.069	0.357	41.649	-0.373	0.416	0.369	0.601
FAM86JP	139.770	-1.239	0.324	0.000	0.001	139.770	-0.511	0.317	0.107	0.399	139.770	0.729	0.374	0.051	0.185
LOC100126784	83.887	2.491	0.331	0.000	0.000	83.887	0.761	0.344	0.027	0.319	83.887	-1.729	0.380	0.000	0.000
GHRLOS	10.062	2.183	0.712	0.002	0.010	10.062	0.901	0.744	0.226	0.504	10.062	-1.281	0.809	0.113	0.301
SLCO4A1-AS1	1392.996	1.085	0.151	0.000	0.000	1392.996	0.362	0.152	0.017	0.306	1392.996	-0.722	0.174	0.000	0.001
C8orf88	317.330	-0.647	0.126	0.000	0.000	317.330	0.019	0.120	0.874	0.936	317.330	0.666	0.144	0.000	0.000
SMARCA5-AS1	231.706	-0.965	0.405	0.017	0.051	231.706	-0.358	0.402	0.373	0.622	231.706	0.607	0.467	0.194	0.417
FAM229A	75.595	2.462	0.567	0.000	0.000	75.595	1.253	0.572	0.028	0.321	75.595	-1.209	0.650	0.063	0.211
LOC100128079	15.359	-1.088	0.481	0.024	0.066	15.359	-0.176	0.437	0.688	0.831	15.359	0.912	0.548	0.096	0.274
UST-AS1	11.224	-0.031	0.633	0.961	0.977	11.224	-0.766	0.660	0.246	0.521	11.224	-0.735	0.762	0.335	0.570
TMPO-AS1	635.929	-1.538	0.390	0.000	0.001	635.929	-0.608	0.388	0.117	0.409	635.929	0.930	0.450	0.039	0.156
TNK2-AS1	103.724	-0.267	0.354	0.451	0.586	103.724	0.500	0.348	0.151	0.444	103.724	0.767	0.406	0.059	0.203
DLG5-AS1	13.099	1.396	0.679	0.040	0.097	13.099	1.024	0.683	0.134	0.426	13.099	-0.372	0.771	0.630	0.798
LOC100128317	162.379	-0.860	0.244	0.000	0.003	162.379	0.199	0.233	0.395	0.639	162.379	1.058	0.279	0.000	0.003
FAM83H-AS1	63.873	0.788	0.409	0.054	0.122	63.873	0.975	0.406	0.016	0.306	63.873	0.187	0.467	0.689	0.837
FAM225B	33.312	-0.018	0.386	0.964	0.978	33.312	0.140	0.380	0.713	0.846	33.312	0.158	0.444	0.723	0.856
LOC100128494	2049.256	-0.958	0.320	0.003	0.012	2049.256	0.205	0.319	0.520	0.725	2049.256	1.164	0.369	0.002	0.019
NEBL-AS1	24.842	-1.117	0.707	0.114	0.216	24.842	-0.051	0.687	0.941	0.970	24.842	1.067	0.812	0.189	0.410
MIR29B2CHG	17.112	0.752	0.790	0.341	0.482	17.112	0.772	0.787	0.327	0.587	17.112	0.020	0.905	0.982	0.990
CTAGE4	70.614	0.602	0.342	0.078	0.163	70.614	0.276	0.343	0.421	0.658	70.614	-0.325	0.394	0.409	0.636
C19orf71	78.494	0.401	0.275	0.144	0.257	78.494	-0.261	0.281	0.353	0.607	78.494	-0.662	0.323	0.040	0.159
ACVR2B-AS1	38.167	-0.586	0.477	0.220	0.350	38.167	0.516	0.456	0.258	0.530	38.167	1.102	0.543	0.042	0.165
OST4	710.591	0.759	0.229	0.001	0.005	710.591	0.224	0.230	0.330	0.590	710.591	-0.535	0.265	0.043	0.167
RBPMS-AS1	57.652	0.209	0.223	0.349	0.489	57.652	0.046	0.223	0.838	0.916	57.652	-0.164	0.259	0.528	0.728
LINC00476	118.558	0.327	0.249	0.188	0.313	118.558	0.251	0.248	0.311	0.575	118.558	-0.076	0.287	0.791	0.896
SRRM2-AS1	8.408	-0.140	0.625	0.822	0.884	8.408	0.078	0.607	0.898	0.949	8.408	0.218	0.719	0.762	0.879
LINC01003	89.174	0.299	0.197	0.130	0.238	89.174	0.053	0.198	0.788	0.889	89.174	-0.245	0.229	0.284	0.520
VPS9D1-AS1	452.836	-0.556	0.299	0.062	0.137	452.836	-0.513	0.298	0.085	0.378	452.836	0.044	0.345	0.900	0.953
GATA6-AS1	15.131	0.183	0.505	0.718	0.808	15.131	0.223	0.499	0.655	0.812	15.131	0.040	0.581	0.945	0.975
ZBTB42	53.535	0.958	0.361	0.008	0.029	53.535	0.278	0.368	0.450	0.679	53.535	-0.680	0.418	0.104	0.287
MAPT-AS1	21.207	0.030	0.752	0.968	0.981	21.207	0.696	0.740	0.347	0.603	21.207	0.666	0.861	0.440	0.660
PKP4-AS1	99.590	-1.292	0.224	0.000	0.000	99.590	-0.093	0.204	0.649	0.808	99.590	1.199	0.255	0.000	0.000
BCAR3-AS1	61.956	-1.597	0.541	0.003	0.014	61.956	-0.649	0.527	0.219	0.498	61.956	0.948	0.624	0.129	0.324
SEMA3F-AS1	215.303	0.266	0.175	0.129	0.236	215.303	0.206	0.175	0.237	0.514	215.303	-0.060	0.202	0.767	0.881
KTN1-AS1	94.128	-0.403	0.211	0.057	0.127	94.128	0.136	0.202	0.500	0.713	94.128	0.539	0.242	0.026	0.119
LOC100129138	117.013	-0.461	0.444	0.299	0.437	117.013	0.312	0.439	0.478	0.698	117.013	0.773	0.511	0.131	0.328
ZSCAN16-AS1	119.406	0.691	0.350	0.048	0.112	119.406	0.519	0.350	0.138	0.429	119.406	-0.172	0.403	0.669	0.824
MATN1-AS1	25.481	1.291	0.425	0.002	0.011	25.481	0.940	0.429	0.028	0.321	25.481	-0.351	0.478	0.464	0.680
SMIM27	57.411	0.177	0.222	0.426	0.562	57.411	0.238	0.217	0.273	0.544	57.411	0.061	0.254	0.809	0.904
SMIM10L1	841.028	0.223	0.161	0.165	0.284	841.028	-0.092	0.161	0.567	0.754	841.028	-0.315	0.186	0.090	0.262
GABPB1-AS1	109.240	-1.192	0.244	0.000	0.000	109.240	-0.296	0.230	0.199	0.482	109.240	0.896	0.280	0.001	0.017
MSTO2P	212.888	-0.572	0.348	0.100	0.196	212.888	-0.914	0.348	0.009	0.297	212.888	-0.342	0.404	0.396	0.624
DPY19L1P1	50.188	-0.054	0.301	0.858	0.907	50.188	0.026	0.296	0.930	0.965	50.188	0.080	0.347	0.818	0.910

LYRM4-AS1	66.199	-0.225	0.289	0.436	0.572	66.199	0.184	0.281	0.511	0.719	66.199	0.409	0.331	0.217	0.443
LINC01546	10.605	-0.812	1.251	0.516	0.644	10.605	0.928	1.216	0.445	0.676	10.605	1.740	1.430	0.223	0.451
ZNF37BP	138.976	0.095	0.271	0.727	0.815	138.976	0.225	0.269	0.404	0.646	138.976	0.130	0.312	0.677	0.829
LOC100129484	47.348	-0.685	0.302	0.023	0.064	47.348	-0.147	0.287	0.608	0.783	47.348	0.538	0.347	0.121	0.312
LINC02035	58.106	1.270	0.414	0.002	0.010	58.106	0.740	0.417	0.076	0.365	58.106	-0.530	0.476	0.265	0.500
LOC100129603	21.217	-1.013	0.574	0.078	0.162	21.217	0.923	0.511	0.071	0.360	21.217	1.936	0.638	0.002	0.025
MIR193BHG	63.264	1.788	0.390	0.000	0.000	63.264	0.302	0.414	0.465	0.689	63.264	-1.485	0.456	0.001	0.014
CCDC152	210.840	-0.191	0.593	0.747	0.831	210.840	0.683	0.591	0.248	0.522	210.840	0.874	0.684	0.201	0.425
SLC25A21-AS1	23.056	-0.660	0.444	0.137	0.247	23.056	-0.384	0.432	0.374	0.622	23.056	0.276	0.515	0.592	0.772
COL4A2-AS2	48.648	-0.400	0.351	0.254	0.389	48.648	-0.413	0.349	0.237	0.514	48.648	-0.012	0.409	0.976	0.987
PCOLCE-AS1	23.779	1.373	0.483	0.004	0.018	23.779	0.417	0.495	0.399	0.643	23.779	-0.956	0.558	0.087	0.257
LOC100129917	266.002	-0.145	0.178	0.415	0.553	266.002	0.109	0.176	0.533	0.733	266.002	0.255	0.205	0.214	0.440
TMEM269	47.614	0.032	0.353	0.927	0.954	47.614	0.441	0.344	0.200	0.483	47.614	0.408	0.403	0.311	0.546
LOC100129931	752.086	-0.435	0.132	0.001	0.006	752.086	0.075	0.130	0.563	0.751	752.086	0.511	0.152	0.001	0.011
CCNT2-AS1	27.840	0.393	0.333	0.237	0.370	27.840	0.576	0.324	0.075	0.364	27.840	0.183	0.376	0.627	0.797
LINC01460	12.901	-0.044	0.445	0.921	0.950	12.901	0.023	0.434	0.957	0.978	12.901	0.067	0.514	0.896	0.951
HDAC6	589.482	0.179	0.327	0.584	0.701	589.482	0.528	0.326	0.106	0.398	589.482	0.349	0.377	0.355	0.588
URAHP	111.934	0.981	0.276	0.000	0.003	111.934	0.361	0.279	0.196	0.480	111.934	-0.620	0.319	0.052	0.188
HSFX2	10.219	1.655	0.612	0.007	0.025	10.219	0.554	0.643	0.389	0.635	10.219	-1.101	0.704	0.118	0.308
LOC100130172	12.662	-1.840	0.614	0.003	0.012	12.662	0.178	0.458	0.698	0.838	12.662	2.017	0.669	0.003	0.026
EFCAB14-AS1	49.406	-0.418	0.490	0.393	0.532	49.406	-0.183	0.485	0.707	0.842	49.406	0.236	0.566	0.677	0.829
TFAP2A-AS1	46.406	0.002	0.242	0.992	0.996	46.406	0.064	0.237	0.788	0.889	46.406	0.062	0.280	0.826	0.914
LOC100130283	320.128	1.114	0.198	0.000	0.000	320.128	0.406	0.200	0.042	0.330	320.128	-0.708	0.228	0.002	0.021
LOC100130357	19.911	0.496	0.552	0.369	0.509	19.911	-0.638	0.586	0.277	0.547	19.911	-1.134	0.665	0.088	0.260
LINC02593	19.918	1.684	0.497	0.001	0.004	19.918	0.146	0.527	0.782	0.885	19.918	-1.538	0.584	0.008	0.058
CECR7	45.183	-1.162	0.293	0.000	0.001	45.183	-0.357	0.267	0.181	0.469	45.183	0.805	0.335	0.016	0.090
WAKMAR2	28.579	0.076	0.435	0.861	0.909	28.579	0.102	0.425	0.810	0.900	28.579	0.026	0.501	0.958	0.981
PARD6G-AS1	7.797	2.077	0.649	0.001	0.007	7.797	0.893	0.689	0.195	0.479	7.797	-1.185	0.732	0.105	0.289
NFYC-AS1	67.657	-0.524	0.202	0.009	0.032	67.657	-0.132	0.190	0.486	0.703	67.657	0.392	0.232	0.091	0.264
LINC00910	22.635	-0.748	0.454	0.099	0.195	22.635	0.404	0.418	0.334	0.593	22.635	1.152	0.512	0.024	0.115
LOC100130691	52.191	-0.410	0.412	0.319	0.459	52.191	0.053	0.404	0.895	0.948	52.191	0.463	0.474	0.328	0.562
HDHD5-AS1	7.483	1.610	1.015	0.113	0.213	7.483	1.471	1.014	0.147	0.438	7.483	-0.139	1.144	0.903	0.955
EFCAB10	83.847	-0.001	0.313	0.998	0.999	83.847	-0.564	0.318	0.076	0.365	83.847	-0.563	0.367	0.126	0.320
AGAP2-AS1	412.505	0.835	0.185	0.000	0.000	412.505	0.194	0.187	0.298	0.565	412.505	-0.641	0.214	0.003	0.027
USP3-AS1	224.527	-0.733	0.212	0.001	0.004	224.527	-0.070	0.205	0.733	0.857	224.527	0.663	0.243	0.006	0.049
LOC100130872	102.495	3.241	0.270	0.000	0.000	102.495	0.583	0.295	0.048	0.337	102.495	-2.658	0.314	0.000	0.000
PSORS1C3	120.693	1.058	0.651	0.104	0.202	120.693	0.534	0.653	0.413	0.652	120.693	-0.524	0.752	0.486	0.697
TSTD3	442.674	-0.773	0.233	0.001	0.005	442.674	-0.118	0.230	0.607	0.783	442.674	0.655	0.268	0.015	0.083
VAC14-AS1	75.441	0.079	0.306	0.796	0.866	75.441	-0.307	0.308	0.319	0.581	75.441	-0.386	0.357	0.279	0.514
CSAG4	9.671	2.025	0.785	0.010	0.034	9.671	1.773	0.787	0.024	0.316	9.671	-0.252	0.846	0.766	0.881
LOC100130987	50.950	0.007	0.395	0.986	0.992	50.950	0.357	0.388	0.357	0.611	50.950	0.350	0.453	0.439	0.660
ZNF316	1116.402	-0.157	0.210	0.455	0.590	1116.402	-0.236	0.210	0.262	0.535	1116.402	-0.079	0.243	0.747	0.870
LINC01135	7.510	0.301	0.658	0.648	0.753	7.510	-0.514	0.705	0.466	0.690	7.510	-0.815	0.803	0.310	0.545
CKMT2-AS1	94.614	-0.167	0.222	0.452	0.587	94.614	0.346	0.214	0.106	0.398	94.614	0.513	0.253	0.043	0.165
SRP14-AS1	76.582	0.038	0.315	0.903	0.938	76.582	0.397	0.310	0.200	0.483	76.582	0.359	0.361	0.321	0.556
DPP9-AS1	264.825	-0.175	0.265	0.508	0.638	264.825	-0.621	0.266	0.020	0.306	264.825	-0.446	0.308	0.148	0.356
TSTD1	23.837	0.705	0.349	0.043	0.104	23.837	0.466	0.351	0.184	0.471	23.837	-0.239	0.399	0.549	0.744
CCDC183-AS1	9.048	0.045	0.567	0.936	0.960	9.048	-0.196	0.571	0.731	0.856	9.048	-0.242	0.666	0.717	0.853
RPL21P28	83.864	1.625	0.350	0.000	0.000	83.864	0.692	0.356	0.052	0.339	83.864	-0.933	0.402	0.020	0.103
SNAP25-AS1	20.109	-0.440	0.395	0.266	0.402	20.109	-0.010	0.375	0.980	0.989	20.109	0.430	0.453	0.342	0.576
NEMP2	419.289	-1.316	0.290	0.000	0.000	419.289	-0.452	0.287	0.116	0.408	419.289	0.864	0.335	0.010	0.065
ZNF503-AS2	68.216	0.143	0.288	0.618	0.729	68.216	-0.722	0.300	0.016	0.306	68.216	-0.865	0.344	0.012	0.073
LINC00887	45.012	1.607	0.808	0.047	0.110	45.012	1.453	0.808	0.072	0.361	45.012	-0.155	0.925	0.867	0.936
CCDC18-AS1	155.230	-0.422	0.242	0.081	0.168	155.230	0.220	0.237	0.352	0.606	155.230	0.642	0.278	0.021	0.104
LOC100131635	307.924	-0.381	0.394	0.333	0.473	307.924	0.310	0.392	0.429	0.664	307.924	0.691	0.454	0.128	0.324
ZNF236-DT	12.344	0.699	0.518	0.178	0.300	12.344	-0.742	0.570	0.193	0.478	12.344	-1.441	0.638	0.024	0.114
MZF1-AS1	139.644	0.112	0.319	0.725	0.813	139.644	0.444	0.316	0.159	0.451	139.644	0.332	0.366	0.365	0.597
USP30-AS1	16.257	-0.935	0.440	0.033	0.085	16.257	-0.044	0.396	0.912	0.956	16.257	0.891	0.499	0.074	0.233
PET100	242.527	1.228	0.308	0.000	0.001	242.527	0.091	0.312	0.770	0.878	242.527	-1.137	0.357	0.001	0.017
LINC00271	14.978	-0.678	0.459	0.140	0.250	14.978	-0.049	0.427	0.909	0.955	14.978	0.629	0.525	0.230	0.459
CADM3-AS1	96.524	2.020	0.340	0.000	0.000	96.524	0.140	0.350	0.689	0.832	96.524	-1.880	0.396	0.000	0.000
ZNF717	88.741	0.444	0.250	0.076	0.160	88.741	0.348	0.250	0.163	0.454	88.741	-0.096	0.288	0.740	0.867
INSYN2B	175.904	-1.491	0.550	0.007	0.025	175.904	-1.600	0.549	0.004	0.297	175.904	-0.109	0.637	0.864	0.935
FAM27E3	40.019	-1.913	0.479	0.000	0.001	40.019	-0.689	0.449	0.125	0.416	40.019	1.224	0.549	0.026	0.119
RRN3P3	51.620	-0.318	0.235	0.176	0.298	51.620	-0.307	0.230	0.183	0.470	51.620	0.011	0.275	0.967	0.985
LOC100132057	11.633	0.724	0.542	0.182	0.305	11.633	0.100	0.560	0.859	0.928	11.633	-0.624	0.633	0.324	0.559
FOXO6	25.996	0.581	0.853	0.495	0.627	25.996	0.195	0.855	0.820	0.906	25.996	-0.387	0.985	0.695	0.840

ACTA2-AS1	160.261	0.548	0.433	0.206	0.334	160.261	0.845	0.431	0.050	0.337	160.261	0.297	0.498	0.551	0.745
PHKA2-AS1	80.047	0.727	0.312	0.020	0.057	80.047	0.242	0.315	0.441	0.672	80.047	-0.485	0.361	0.179	0.398
LOC100132215	71.859	0.463	0.255	0.069	0.148	71.859	-0.177	0.261	0.497	0.711	71.859	-0.641	0.299	0.032	0.138
NPIPB5	1299.041	1.902	0.375	0.000	0.000	1299.041	0.265	0.376	0.481	0.700	1299.041	-1.637	0.434	0.000	0.003
LOC100132249	9.052	-1.175	0.798	0.141	0.252	9.052	0.451	0.727	0.535	0.734	9.052	1.626	0.899	0.070	0.226
NDUFA6-DT	145.062	0.095	0.381	0.803	0.870	145.062	0.234	0.380	0.537	0.735	145.062	0.139	0.440	0.751	0.873
LOC100132287	51.643	0.310	0.341	0.364	0.505	51.643	0.198	0.341	0.562	0.750	51.643	-0.112	0.394	0.776	0.886
TEKT4P2	81.884	0.891	0.286	0.002	0.009	81.884	0.218	0.292	0.455	0.682	81.884	-0.673	0.332	0.042	0.165
CLUHP3	72.173	-0.491	0.246	0.046	0.109	72.173	0.163	0.234	0.488	0.705	72.173	0.653	0.281	0.020	0.102
FRG1HP	167.222	-0.297	0.154	0.054	0.123	167.222	0.121	0.148	0.413	0.652	167.222	0.418	0.177	0.018	0.095
FAM157B	8.456	0.202	0.794	0.799	0.868	8.456	0.951	0.761	0.212	0.492	8.456	0.749	0.893	0.402	0.629
NBPF10	372.480	0.405	0.250	0.105	0.202	372.480	0.240	0.250	0.336	0.595	372.480	-0.165	0.288	0.567	0.757
ZRANB2-AS1	204.609	-1.198	0.292	0.000	0.000	204.609	-0.400	0.286	0.162	0.453	204.609	0.798	0.336	0.018	0.094
PAXIP1-AS2	154.448	0.065	0.172	0.706	0.798	154.448	-0.047	0.171	0.784	0.887	154.448	-0.111	0.199	0.576	0.761
KDM4A-AS1	235.337	-0.169	0.191	0.377	0.517	235.337	-0.194	0.190	0.307	0.573	235.337	-0.025	0.221	0.909	0.959
MSC-AS1	320.923	0.067	0.204	0.743	0.827	320.923	0.362	0.203	0.074	0.364	320.923	0.295	0.235	0.210	0.436
DPH3P1	16.182	0.063	0.403	0.875	0.919	16.182	-0.211	0.407	0.604	0.781	16.182	-0.274	0.474	0.563	0.754
FAM27C	50.947	-0.696	0.409	0.089	0.179	50.947	0.369	0.392	0.346	0.602	50.947	1.065	0.466	0.022	0.109
RASA4DP	71.260	0.844	0.708	0.233	0.366	71.260	0.928	0.707	0.189	0.475	71.260	0.085	0.815	0.917	0.961
FAM95B1	19.993	1.644	0.653	0.012	0.039	19.993	0.373	0.668	0.577	0.762	19.993	-1.271	0.756	0.093	0.268
LOC100133091	285.224	-0.137	0.194	0.482	0.615	285.224	-0.179	0.194	0.355	0.609	285.224	-0.042	0.225	0.851	0.928
LINC01001	19.734	0.367	0.439	0.403	0.542	19.734	0.859	0.423	0.042	0.330	19.734	0.492	0.493	0.318	0.554
C9orf147	16.652	-0.847	0.531	0.110	0.211	16.652	0.558	0.476	0.241	0.517	16.652	1.405	0.593	0.018	0.095
LINC00240	54.376	0.665	0.421	0.114	0.216	54.376	0.672	0.419	0.109	0.401	54.376	0.007	0.482	0.988	0.993
LOC100133286	694.492	0.045	0.167	0.786	0.858	694.492	-0.250	0.167	0.136	0.427	694.492	-0.295	0.194	0.128	0.323
HOXA-AS3	30.000	0.396	0.404	0.326	0.467	30.000	0.701	0.394	0.075	0.364	30.000	0.305	0.457	0.505	0.712
LOC100133315	14.320	-0.019	0.587	0.974	0.984	14.320	-0.173	0.586	0.768	0.877	14.320	-0.154	0.683	0.822	0.912
LOC100133331	167.833	0.759	0.285	0.008	0.028	167.833	0.403	0.286	0.159	0.451	167.833	-0.356	0.329	0.279	0.514
LINC01134	8.438	-0.634	0.780	0.416	0.554	8.438	0.865	0.688	0.209	0.490	8.438	1.499	0.861	0.082	0.248
LY6E-DT	9.150	-0.683	0.805	0.396	0.535	9.150	0.744	0.753	0.323	0.584	9.150	1.427	0.908	0.116	0.305
CD24	160.183	0.602	0.354	0.089	0.179	160.183	0.111	0.355	0.754	0.868	160.183	-0.491	0.409	0.230	0.459
UXT-AS1	260.375	0.053	0.189	0.778	0.852	260.375	0.364	0.186	0.050	0.337	260.375	0.311	0.217	0.151	0.360
MAP3K14-AS1	104.111	0.108	0.276	0.697	0.791	104.111	-0.125	0.277	0.651	0.810	104.111	-0.233	0.321	0.468	0.683
UBOX5-AS1	15.812	-0.362	0.469	0.440	0.576	15.812	0.342	0.438	0.435	0.668	15.812	0.704	0.530	0.184	0.404
KDM7A-DT	92.181	-0.379	0.247	0.126	0.232	92.181	-0.009	0.238	0.969	0.984	92.181	0.369	0.284	0.194	0.417
LOC100134368	36.330	1.137	0.359	0.002	0.008	36.330	0.633	0.365	0.083	0.374	36.330	-0.504	0.411	0.220	0.446
NDUFB2-AS1	379.438	0.224	0.186	0.229	0.361	379.438	-0.246	0.187	0.188	0.474	379.438	-0.470	0.216	0.030	0.131
LOC100134868	14.261	-0.421	0.457	0.358	0.498	14.261	0.093	0.432	0.829	0.911	14.261	0.514	0.522	0.325	0.559
UBE2Q2P2	54.268	-0.250	0.384	0.514	0.642	54.268	0.378	0.372	0.309	0.574	54.268	0.629	0.438	0.151	0.361
HDAC5	1433.996	0.677	0.285	0.018	0.052	1433.996	0.606	0.285	0.034	0.322	1433.996	-0.071	0.329	0.830	0.917
CHKB-DT	15.429	0.436	0.430	0.311	0.450	15.429	-0.351	0.452	0.438	0.670	15.429	-0.787	0.514	0.126	0.321
PDCD6IP	3165.826	-0.142	0.142	0.317	0.457	3165.826	0.058	0.142	0.681	0.827	3165.826	0.200	0.164	0.222	0.449
PDCD6	101.220	0.382	0.256	0.136	0.246	101.220	0.277	0.256	0.279	0.549	101.220	-0.105	0.296	0.722	0.856
EPOP	513.420	0.658	0.250	0.008	0.030	513.420	-0.022	0.251	0.931	0.965	513.420	-0.680	0.289	0.019	0.098
TRIM59-IFT80	10.543	-1.052	0.621	0.090	0.181	10.543	-0.436	0.581	0.453	0.680	10.543	0.616	0.717	0.390	0.619
BCL2L11	297.019	-0.545	0.348	0.117	0.220	297.019	0.309	0.345	0.370	0.620	297.019	0.854	0.401	0.033	0.140
DNMBP-AS1	81.561	-1.750	0.561	0.002	0.009	81.561	-0.161	0.546	0.769	0.877	81.561	1.589	0.644	0.014	0.080
DCTN1-AS1	31.877	-1.135	0.476	0.017	0.051	31.877	-0.205	0.453	0.651	0.810	31.877	0.931	0.546	0.088	0.259
SH2B3	976.949	-1.030	0.311	0.001	0.006	976.949	-0.693	0.311	0.026	0.316	976.949	0.338	0.360	0.348	0.581
TPT1-AS1	349.973	0.430	0.270	0.111	0.212	349.973	0.439	0.270	0.103	0.396	349.973	0.009	0.312	0.977	0.988
GNE	315.954	0.295	0.227	0.193	0.318	315.954	0.246	0.226	0.277	0.547	315.954	-0.049	0.262	0.851	0.928
HCN4	11.642	0.551	0.554	0.320	0.460	11.642	-0.569	0.589	0.334	0.593	11.642	-1.120	0.668	0.093	0.268
FRAT1	39.132	0.413	0.323	0.201	0.328	39.132	0.224	0.323	0.489	0.705	39.132	-0.189	0.373	0.611	0.785
TROAP	517.925	0.253	0.248	0.307	0.446	517.925	0.341	0.247	0.168	0.457	517.925	0.088	0.286	0.759	0.877
MED16	1209.022	-0.046	0.119	0.700	0.793	1209.022	0.076	0.118	0.520	0.724	1209.022	0.122	0.137	0.374	0.606
PIGK	538.424	-0.219	0.250	0.381	0.521	538.424	-0.375	0.250	0.134	0.426	538.424	-0.155	0.290	0.592	0.772
LOC100268168	8.225	0.848	0.630	0.179	0.301	8.225	-0.375	0.683	0.583	0.766	8.225	-1.223	0.762	0.109	0.294
CASC11	2571.022	-1.048	0.229	0.000	0.000	2571.022	-0.188	0.229	0.411	0.650	2571.022	0.859	0.265	0.001	0.015
LOC100270746	36.232	0.345	0.492	0.483	0.616	36.232	0.409	0.489	0.403	0.645	36.232	0.064	0.565	0.910	0.959
LINC00899	10.309	0.451	0.576	0.434	0.570	10.309	1.130	0.548	0.039	0.327	10.309	0.679	0.638	0.287	0.523
LIMS3-LOC440895	117.967	2.251	0.411	0.000	0.000	117.967	0.882	0.419	0.035	0.324	117.967	-1.368	0.472	0.004	0.034
SMG1P3	381.016	-0.800	0.303	0.008	0.029	381.016	-0.062	0.300	0.836	0.916	381.016	0.738	0.349	0.035	0.144
RASA4B	180.338	0.845	0.769	0.272	0.409	180.338	0.712	0.769	0.354	0.608	180.338	-0.132	0.887	0.882	0.943
CMC4	64.756	0.900	0.380	0.018	0.053	64.756	0.004	0.387	0.991	0.995	64.756	-0.896	0.442	0.043	0.166
LINC00894	10.138	0.660	0.613	0.282	0.420	10.138	1.160	0.590	0.049	0.337	10.138	0.501	0.680	0.461	0.678
BCDIN3D-AS1	80.377	-0.495	0.212	0.020	0.057	80.377	-0.021	0.201	0.916	0.958	80.377	0.474	0.243	0.052	0.186
LOC100286925	393.594	0.019	0.206	0.928	0.954	393.594	-0.195	0.206	0.343	0.600	393.594	-0.214	0.238	0.369	0.601

VPS13A-AS1	11.755	-1.180	0.925	0.202	0.329	11.755	-0.607	0.901	0.501	0.713	11.755	0.573	1.069	0.592	0.772
LOC100287015	98.296	0.000	0.198	1.000	1.000	98.296	-0.016	0.196	0.936	0.968	98.296	-0.016	0.229	0.946	0.975
LOC100287036	10.949	1.266	0.541	0.019	0.056	10.949	0.364	0.580	0.531	0.731	10.949	-0.902	0.628	0.151	0.360
LOC100287042	124.778	-0.651	0.425	0.125	0.231	124.778	-0.861	0.425	0.043	0.331	124.778	-0.210	0.493	0.671	0.825
LOC100287049	97.893	-0.913	0.366	0.013	0.041	97.893	0.216	0.355	0.543	0.739	97.893	1.129	0.420	0.007	0.052
TONSL-AS1	20.608	-0.604	0.528	0.253	0.387	20.608	-0.893	0.531	0.093	0.387	20.608	-0.289	0.622	0.643	0.806
WASHC1	183.978	0.765	0.318	0.016	0.049	183.978	0.426	0.319	0.182	0.469	183.978	-0.340	0.367	0.355	0.588
LOC100287175	8.409	-0.095	1.115	0.932	0.957	8.409	0.636	1.084	0.557	0.747	8.409	0.731	1.271	0.565	0.755
EML2-AS1	183.524	-0.125	0.246	0.611	0.723	183.524	0.469	0.239	0.050	0.337	183.524	0.594	0.281	0.034	0.144
SH3RF3-AS1	46.658	-1.611	0.498	0.001	0.007	46.658	-0.008	0.471	0.986	0.992	46.658	1.603	0.569	0.005	0.040
LINC00941	107.514	-0.686	0.383	0.073	0.154	107.514	-0.297	0.378	0.433	0.667	107.514	0.390	0.442	0.378	0.609
SMKR1	8.290	1.202	0.566	0.034	0.086	8.290	-0.719	0.675	0.287	0.555	8.290	-1.921	0.729	0.008	0.058
LOC100287497	22.242	0.782	0.419	0.062	0.137	22.242	0.226	0.428	0.597	0.776	22.242	-0.556	0.486	0.253	0.485
ADPGK-AS1	85.288	-0.594	0.413	0.150	0.265	85.288	-0.622	0.412	0.131	0.423	85.288	-0.028	0.480	0.953	0.978
LOXL1-AS1	150.154	1.120	0.199	0.000	0.000	150.154	0.287	0.204	0.159	0.451	150.154	-0.833	0.231	0.000	0.006
MTHFD2P1	17.397	1.564	0.470	0.001	0.005	17.397	1.287	0.472	0.006	0.297	17.397	-0.276	0.524	0.598	0.776
AP4B1-AS1	80.059	-0.822	0.400	0.040	0.097	80.059	-0.977	0.399	0.014	0.306	80.059	-0.155	0.467	0.740	0.867
LINC00630	79.960	-0.999	0.372	0.007	0.026	79.960	-0.237	0.362	0.512	0.719	79.960	0.761	0.428	0.075	0.236
LOC100287808	55.470	-0.066	0.328	0.840	0.894	55.470	0.529	0.317	0.096	0.391	55.470	0.595	0.373	0.111	0.298
LOC100287837	56.560	-0.579	0.486	0.233	0.365	56.560	0.241	0.476	0.613	0.786	56.560	0.820	0.558	0.141	0.346
LOC100287896	58.889	0.784	0.479	0.102	0.198	58.889	0.639	0.479	0.182	0.469	58.889	-0.145	0.551	0.792	0.896
LGALS8-AS1	37.933	-0.035	0.363	0.923	0.951	37.933	-0.500	0.370	0.177	0.465	37.933	-0.465	0.429	0.279	0.513
TIMM23	1107.252	-0.351	0.402	0.383	0.523	1107.252	-0.939	0.402	0.020	0.306	1107.252	-0.589	0.465	0.205	0.429
LOC100287944	33.780	-1.752	0.400	0.000	0.000	33.780	-0.557	0.357	0.119	0.411	33.780	1.195	0.456	0.009	0.060
LOC100288123	38.121	0.079	0.376	0.834	0.890	38.121	-0.071	0.376	0.850	0.923	38.121	-0.150	0.437	0.731	0.861
NBPF20	790.948	0.166	0.225	0.462	0.597	790.948	-0.358	0.226	0.113	0.405	790.948	-0.524	0.261	0.045	0.170
SLC9A3-AS1	635.856	1.320	0.429	0.002	0.010	635.856	0.938	0.430	0.029	0.322	635.856	-0.382	0.495	0.440	0.661
LOC100288175	476.958	1.484	0.479	0.002	0.010	476.958	1.056	0.479	0.028	0.321	476.958	-0.428	0.551	0.437	0.658
LNCOC1	524.437	0.080	0.237	0.735	0.821	524.437	-0.143	0.237	0.545	0.740	524.437	-0.223	0.274	0.415	0.640
HMGN3-AS1	201.982	0.391	0.204	0.055	0.124	201.982	-0.148	0.206	0.472	0.693	201.982	-0.539	0.237	0.023	0.110
LOC100288203	50.795	-0.117	0.294	0.691	0.787	50.795	0.114	0.286	0.691	0.833	50.795	0.231	0.338	0.495	0.703
NPIPA5	246.694	0.604	0.574	0.293	0.432	246.694	1.018	0.573	0.076	0.365	246.694	0.414	0.662	0.532	0.731
NCAM1-AS1	22.520	0.844	0.439	0.055	0.124	22.520	0.984	0.431	0.022	0.315	22.520	0.140	0.490	0.775	0.886
ERVMER34-1	422.230	-0.684	0.169	0.000	0.001	422.230	-0.219	0.166	0.189	0.475	422.230	0.466	0.195	0.017	0.092
IL10RB-DT	197.839	1.287	0.230	0.000	0.000	197.839	-0.286	0.238	0.230	0.507	197.839	-1.573	0.270	0.000	0.000
WHAMMP1	57.744	0.456	0.432	0.291	0.429	57.744	-0.110	0.436	0.801	0.896	57.744	-0.566	0.502	0.259	0.494
LOC100288637	164.947	-0.226	0.270	0.402	0.540	164.947	0.122	0.267	0.647	0.808	164.947	0.349	0.311	0.262	0.496
LIMS4	22.204	1.847	0.613	0.003	0.012	22.204	-0.111	0.654	0.866	0.931	22.204	-1.958	0.726	0.007	0.052
WASH8P	238.531	0.397	0.292	0.174	0.295	238.531	0.298	0.292	0.307	0.572	238.531	-0.099	0.337	0.769	0.882
LOC100288798	50.871	0.128	0.351	0.715	0.805	50.871	0.411	0.345	0.233	0.510	50.871	0.283	0.402	0.481	0.694
B3GNT10	76.246	-1.536	0.329	0.000	0.000	76.246	-0.476	0.310	0.125	0.416	76.246	1.059	0.377	0.005	0.041
LOC100288846	11.761	-0.703	0.574	0.220	0.351	11.761	-0.332	0.546	0.543	0.739	11.761	0.372	0.665	0.576	0.762
LOC100288866	23.198	0.320	0.961	0.739	0.824	23.198	0.769	0.956	0.421	0.658	23.198	0.449	1.105	0.684	0.833
SLC25A25-AS1	82.578	0.602	0.370	0.103	0.200	82.578	-0.222	0.375	0.554	0.745	82.578	-0.825	0.431	0.056	0.196
ATP2A1-AS1	33.004	0.646	0.482	0.180	0.303	33.004	-0.187	0.490	0.702	0.840	33.004	-0.833	0.562	0.138	0.341
FRG1CP	99.282	0.824	0.242	0.001	0.004	99.282	0.487	0.243	0.045	0.334	99.282	-0.337	0.278	0.226	0.455
GS1-124K5.4	10.368	0.767	0.692	0.267	0.404	10.368	1.034	0.679	0.128	0.420	10.368	0.266	0.778	0.732	0.861
GNG12-AS1	91.456	1.114	0.350	0.001	0.008	91.456	0.286	0.355	0.421	0.658	91.456	-0.828	0.405	0.041	0.161
LINC00624	57.699	-0.821	0.382	0.032	0.082	57.699	0.140	0.367	0.704	0.841	57.699	0.960	0.437	0.028	0.126
DNAJC3-DT	28.984	0.269	0.373	0.472	0.606	28.984	0.452	0.366	0.217	0.497	28.984	0.183	0.426	0.667	0.823
MAN1B1-DT	61.122	-0.113	0.272	0.679	0.776	61.122	0.532	0.258	0.039	0.327	61.122	0.644	0.307	0.036	0.148
LOC100289361	11.309	0.552	0.470	0.241	0.374	11.309	-0.198	0.496	0.689	0.832	11.309	-0.750	0.561	0.181	0.400
UBAC2-AS1	17.856	-0.486	0.500	0.331	0.471	17.856	0.260	0.474	0.583	0.767	17.856	0.746	0.569	0.190	0.411
KCTD21-AS1	115.127	-0.191	0.299	0.524	0.651	115.127	0.426	0.294	0.146	0.438	115.127	0.617	0.343	0.072	0.229
LOC100289495	8.776	0.653	0.748	0.383	0.522	8.776	0.657	0.743	0.377	0.624	8.776	0.003	0.854	0.997	0.998
LOC100289580	364.265	-0.592	0.467	0.205	0.333	364.265	0.045	0.466	0.923	0.961	364.265	0.638	0.539	0.237	0.467
ZNF605	116.572	-0.263	0.254	0.300	0.438	116.572	0.115	0.250	0.644	0.806	116.572	0.378	0.292	0.195	0.418
ZNF783	150.190	-0.148	0.185	0.423	0.559	150.190	0.142	0.181	0.432	0.666	150.190	0.290	0.213	0.172	0.389
LOC100291105	41.679	0.933	0.296	0.002	0.008	41.679	0.085	0.308	0.783	0.886	41.679	-0.848	0.346	0.014	0.083
ZNF587B	34.443	-0.381	0.405	0.347	0.487	34.443	-0.839	0.411	0.041	0.329	34.443	-0.458	0.480	0.339	0.574
C4B_2	112.434	2.973	0.880	0.001	0.005	112.434	1.137	0.883	0.198	0.482	112.434	-1.837	1.015	0.070	0.226
RNF213-AS1	148.870	-0.441	0.384	0.251	0.385	148.870	0.139	0.381	0.716	0.847	148.870	0.580	0.443	0.190	0.411
MIR1184-1	381.342	1.049	0.273	0.000	0.001	381.342	0.103	0.275	0.707	0.842	381.342	-0.945	0.316	0.003	0.027
MIR1282	2872.672	1.146	0.265	0.000	0.000	2872.672	0.293	0.265	0.269	0.541	2872.672	-0.853	0.306	0.005	0.043
MIR1244-1	955.209	0.926	0.269	0.001	0.004	955.209	0.206	0.270	0.446	0.677	955.209	-0.720	0.311	0.021	0.103
RASAL2-AS1	17.003	-0.007	0.508	0.988	0.993	17.003	0.255	0.494	0.606	0.782	17.003	0.263	0.581	0.651	0.813
LINC00882	41.700	-0.204	0.464	0.660	0.762	41.700	0.536	0.453	0.236	0.513	41.700	0.740	0.530	0.163	0.377

LINC00184	6.036	-0.024	0.891	0.979	0.987	6.036	0.793	0.854	0.353	0.607	6.036	0.817	1.007	0.417	0.642
FTX	524.234	0.134	0.209	0.521	0.649	524.234	0.137	0.209	0.513	0.720	524.234	0.002	0.241	0.992	0.996
TMED7-TICAM2	17.839	-1.100	0.569	0.053	0.121	17.839	-0.612	0.543	0.260	0.532	17.839	0.488	0.660	0.460	0.677
FAS-AS1	48.254	-0.583	0.363	0.108	0.207	48.254	-0.320	0.355	0.367	0.618	48.254	0.263	0.421	0.532	0.732
TSNAX-DISC1	12.901	0.308	0.572	0.591	0.706	12.901	0.108	0.573	0.850	0.923	12.901	-0.200	0.663	0.763	0.880
ZEB2-AS1	11.558	0.032	0.570	0.955	0.973	11.558	-0.024	0.565	0.966	0.982	11.558	-0.056	0.661	0.932	0.969
SLC25A5-AS1	1299.490	-0.651	0.234	0.005	0.021	1299.490	0.167	0.233	0.476	0.697	1299.490	0.818	0.270	0.002	0.025
PET117	171.886	-0.441	0.251	0.079	0.163	171.886	-0.293	0.249	0.239	0.516	171.886	0.148	0.291	0.610	0.785
PITPNA-AS1	56.884	0.201	0.310	0.516	0.644	56.884	0.132	0.308	0.669	0.821	56.884	-0.069	0.358	0.846	0.926
SPDYE2B	28.160	1.336	0.423	0.002	0.008	28.160	-0.698	0.467	0.135	0.426	28.160	-2.035	0.518	0.000	0.002
MIR762	97.831	0.240	0.250	0.337	0.477	97.831	0.073	0.250	0.769	0.878	97.831	-0.167	0.289	0.565	0.755
HOTTIP	22.983	0.623	0.395	0.115	0.217	22.983	0.565	0.392	0.150	0.442	22.983	-0.059	0.450	0.896	0.951
CHAF1A	902.876	-0.656	0.395	0.097	0.191	902.876	-0.952	0.395	0.016	0.306	902.876	-0.296	0.456	0.516	0.720
LOC100379224	109.910	0.826	0.301	0.006	0.023	109.910	0.647	0.301	0.032	0.322	109.910	-0.179	0.345	0.605	0.782
BOK-AS1	18.610	-0.323	0.416	0.438	0.574	18.610	-0.084	0.398	0.832	0.914	18.610	0.239	0.480	0.619	0.791
PARP2	450.574	-0.885	0.345	0.010	0.035	450.574	-0.840	0.345	0.015	0.306	450.574	0.045	0.399	0.910	0.959
PARP3	372.728	0.754	0.293	0.010	0.035	372.728	0.587	0.293	0.045	0.334	372.728	-0.167	0.338	0.622	0.793
CDH6	42.299	-0.993	0.589	0.092	0.184	42.299	0.191	0.574	0.739	0.861	42.299	1.184	0.676	0.080	0.244
TOM1L1	1296.681	-0.406	0.181	0.025	0.068	1296.681	0.175	0.180	0.333	0.592	1296.681	0.580	0.209	0.005	0.043
LOC100419583	571.677	0.283	0.190	0.136	0.246	571.677	0.253	0.189	0.181	0.469	571.677	-0.029	0.219	0.893	0.949
HMGXB4	746.817	-0.277	0.117	0.018	0.052	746.817	-0.076	0.116	0.509	0.718	746.817	0.201	0.135	0.137	0.339
FAM21EP	13.244	0.651	0.592	0.272	0.408	13.244	0.321	0.598	0.591	0.772	13.244	-0.329	0.682	0.629	0.798
LOC100421746	131.048	-0.383	0.568	0.500	0.631	131.048	0.611	0.563	0.278	0.548	131.048	0.994	0.654	0.128	0.324
SMG1P6	16.430	-0.423	0.636	0.506	0.635	16.430	0.226	0.615	0.713	0.846	16.430	0.649	0.728	0.372	0.604
LOC100422781	53.978	1.032	0.281	0.000	0.002	53.978	0.340	0.290	0.241	0.517	53.978	-0.692	0.325	0.033	0.141
MIR4253	14.506	-1.322	0.577	0.022	0.062	14.506	-1.021	0.556	0.066	0.354	14.506	0.300	0.678	0.658	0.817
MIR1260B	8.301	-0.585	0.680	0.389	0.529	8.301	-0.419	0.664	0.528	0.729	8.301	0.167	0.792	0.833	0.919
TOM1	1070.429	0.540	0.221	0.015	0.045	1070.429	0.384	0.221	0.083	0.374	1070.429	-0.156	0.255	0.541	0.737
SH2D3C	22.049	1.758	0.907	0.053	0.120	22.049	1.149	0.911	0.207	0.489	22.049	-0.609	1.042	0.558	0.750
SH2D3A	131.862	0.355	0.347	0.307	0.447	131.862	0.710	0.345	0.040	0.327	131.862	0.356	0.399	0.373	0.605
MAMLD1	154.277	-0.928	0.196	0.000	0.000	154.277	-0.169	0.188	0.366	0.617	154.277	0.758	0.226	0.001	0.011
RANBP9	1250.441	-0.506	0.088	0.000	0.000	1250.441	0.067	0.087	0.442	0.673	1250.441	0.572	0.102	0.000	0.000
DNAJB6	1426.906	0.440	0.109	0.000	0.001	1426.906	-0.175	0.109	0.109	0.401	1426.906	-0.615	0.126	0.000	0.000
THAP9-AS1	489.284	-0.393	0.284	0.167	0.287	489.284	-0.415	0.284	0.144	0.435	489.284	-0.022	0.329	0.946	0.975
USP2-AS1	71.183	-0.287	0.324	0.376	0.516	71.183	-0.296	0.322	0.358	0.611	71.183	-0.009	0.376	0.981	0.990
LINC00987	21.695	0.445	0.365	0.223	0.354	21.695	0.361	0.363	0.319	0.581	21.695	-0.084	0.418	0.841	0.923
LINC00674	42.829	-0.468	0.367	0.202	0.329	42.829	0.068	0.354	0.847	0.921	42.829	0.536	0.421	0.203	0.426
LINC00673	587.621	-0.327	0.231	0.157	0.274	587.621	-0.335	0.230	0.146	0.438	587.621	-0.008	0.267	0.975	0.987
SUGT1P4-STRA6LP	7.841	0.240	0.697	0.731	0.818	7.841	-0.071	0.702	0.920	0.960	7.841	-0.311	0.812	0.702	0.845
MIR3654	5076.499	0.081	0.241	0.735	0.821	5076.499	0.453	0.241	0.060	0.350	5076.499	0.371	0.278	0.181	0.400
MIR3918	33.722	1.106	0.373	0.003	0.013	33.722	0.458	0.380	0.228	0.506	33.722	-0.647	0.430	0.132	0.330
MIR3648-1	173.262	0.707	0.532	0.183	0.307	173.262	0.801	0.530	0.131	0.423	173.262	0.094	0.611	0.878	0.942
MIR3911	35.817	1.283	0.406	0.002	0.008	35.817	0.646	0.412	0.117	0.409	35.817	-0.638	0.466	0.171	0.388
MIR3661	409.191	-0.968	0.316	0.002	0.010	409.191	-0.678	0.314	0.031	0.322	409.191	0.290	0.365	0.427	0.650
IQCJ-SCHIP1	58.808	-0.317	0.259	0.221	0.352	58.808	-0.186	0.254	0.463	0.689	58.808	0.131	0.300	0.663	0.821
PRKAG2-AS1	48.923	0.175	0.462	0.704	0.797	48.923	0.713	0.451	0.114	0.405	48.923	0.538	0.526	0.306	0.542
LINC01301	13.809	-0.592	0.694	0.393	0.532	13.809	0.717	0.647	0.268	0.540	13.809	1.309	0.781	0.094	0.269
RBM26-AS1	27.277	0.150	0.515	0.770	0.847	27.277	0.502	0.506	0.322	0.583	27.277	0.351	0.589	0.551	0.745
LOC100505549	76.909	0.093	0.253	0.713	0.804	76.909	0.131	0.250	0.602	0.779	76.909	0.038	0.291	0.897	0.951
INAFM2	709.260	0.111	0.124	0.370	0.510	709.260	-0.246	0.125	0.049	0.337	709.260	-0.357	0.144	0.013	0.079
SNHG21	51.454	0.132	0.326	0.685	0.782	51.454	-0.001	0.325	0.997	0.999	51.454	-0.134	0.378	0.724	0.857
GTF3C2-AS1	249.219	-1.126	0.333	0.001	0.004	249.219	-0.364	0.329	0.269	0.541	249.219	0.762	0.384	0.047	0.176
LINC01133	34.541	1.913	0.357	0.000	0.000	34.541	1.091	0.366	0.003	0.297	34.541	-0.822	0.403	0.041	0.162
MALINC1	32.097	-0.482	0.355	0.174	0.296	32.097	-0.137	0.341	0.687	0.831	32.097	0.344	0.409	0.400	0.627
FGD5-AS1	1096.881	0.146	0.111	0.188	0.312	1096.881	-0.238	0.111	0.032	0.322	1096.881	-0.384	0.128	0.003	0.028
RAD51-AS1	30.581	0.784	0.328	0.017	0.050	30.581	0.343	0.333	0.303	0.569	30.581	-0.442	0.378	0.243	0.474
DCST1-AS1	8.721	0.472	0.591	0.425	0.561	8.721	0.348	0.589	0.555	0.746	8.721	-0.124	0.678	0.854	0.930
STARD4-AS1	254.955	0.773	0.254	0.002	0.011	254.955	0.127	0.255	0.618	0.790	254.955	-0.646	0.294	0.028	0.125
NAPA-AS1	198.387	0.153	0.164	0.349	0.489	198.387	-0.373	0.167	0.025	0.316	198.387	-0.526	0.193	0.006	0.048
LINC00888	92.900	0.243	0.282	0.389	0.528	92.900	0.225	0.280	0.421	0.658	92.900	-0.018	0.325	0.957	0.980
SH3BP5-AS1	332.262	0.706	0.147	0.000	0.000	332.262	0.276	0.149	0.064	0.350	332.262	-0.430	0.170	0.011	0.071
LINC01094	9.453	-0.532	0.654	0.417	0.554	9.453	-0.391	0.640	0.541	0.738	9.453	0.141	0.763	0.853	0.930
LOC100505715	14.728	1.009	0.544	0.063	0.138	14.728	0.215	0.561	0.702	0.839	14.728	-0.795	0.632	0.209	0.434
MIR4458HG	56.826	0.968	0.281	0.001	0.004	56.826	0.552	0.285	0.053	0.341	56.826	-0.416	0.321	0.195	0.418
SPATA1	141.167	0.220	0.230	0.338	0.478	141.167	0.388	0.227	0.087	0.380	141.167	0.168	0.264	0.524	0.726
PRMT5-AS1	1415.245	-1.053	0.227	0.000	0.000	1415.245	-0.295	0.226	0.192	0.477	1415.245	0.758	0.262	0.004	0.034
RPARP-AS1	140.707	0.097	0.165	0.555	0.676	140.707	0.162	0.162	0.317	0.579	140.707	0.065	0.190	0.732	0.861

MHENCR	122.170	0.898	0.430	0.037	0.091	122.170	1.155	0.428	0.007	0.297	122.170	0.257	0.493	0.603	0.780
LOC100505774	10.975	0.059	0.504	0.907	0.941	10.975	0.820	0.470	0.081	0.372	10.975	0.761	0.560	0.174	0.392
OSER1-DT	48.397	0.524	0.507	0.301	0.440	48.397	1.205	0.499	0.016	0.306	48.397	0.681	0.578	0.238	0.468
SNHG18	131.869	0.902	0.323	0.005	0.021	131.869	0.453	0.325	0.163	0.454	131.869	-0.449	0.372	0.228	0.457
CARD8-AS1	21.175	-0.859	0.456	0.060	0.132	21.175	-0.029	0.417	0.945	0.973	21.175	0.831	0.519	0.109	0.295
APTR	108.342	-0.248	0.200	0.216	0.345	108.342	0.149	0.193	0.440	0.672	108.342	0.396	0.229	0.084	0.251
LINC00920	31.984	-0.534	0.452	0.238	0.371	31.984	0.288	0.434	0.507	0.717	31.984	0.822	0.516	0.112	0.298
CEBPZOS	957.705	0.246	0.153	0.108	0.207	957.705	0.109	0.153	0.478	0.698	957.705	-0.137	0.177	0.437	0.658
TMEM161B-AS1	133.256	0.240	0.218	0.271	0.407	133.256	0.219	0.217	0.312	0.576	133.256	-0.021	0.251	0.933	0.970
LOC100505915	55.511	0.401	0.549	0.465	0.600	55.511	-0.402	0.556	0.470	0.692	55.511	-0.803	0.640	0.209	0.434
ADD3-AS1	22.214	0.359	0.666	0.590	0.706	22.214	0.752	0.658	0.253	0.527	22.214	0.393	0.762	0.606	0.782
LOC100505938	58.009	-1.019	0.293	0.001	0.003	58.009	-0.052	0.273	0.848	0.922	58.009	0.967	0.335	0.004	0.034
ENO1-AS1	12.513	-1.287	0.592	0.030	0.078	12.513	-0.167	0.524	0.750	0.867	12.513	1.121	0.670	0.094	0.270
LUCAT1	205.361	1.098	0.271	0.000	0.001	205.361	0.653	0.273	0.017	0.306	205.361	-0.446	0.310	0.151	0.360
RTCA-AS1	21.279	0.855	0.567	0.131	0.239	21.279	0.742	0.566	0.190	0.475	21.279	-0.114	0.648	0.861	0.933
PPP5D1	10.920	0.200	0.573	0.727	0.814	10.920	0.888	0.525	0.091	0.385	10.920	0.687	0.627	0.273	0.508
ARAP1-AS2	126.836	-0.182	0.332	0.583	0.700	126.836	0.344	0.328	0.295	0.562	126.836	0.526	0.382	0.168	0.384
LINC02606	8.316	-0.109	0.663	0.870	0.916	8.316	0.858	0.619	0.165	0.455	8.316	0.967	0.740	0.191	0.413
PTOV1-AS1	144.628	0.059	0.254	0.817	0.880	144.628	0.277	0.252	0.272	0.543	144.628	0.218	0.293	0.457	0.675
RNASEH1-AS1	84.930	-1.051	0.256	0.000	0.000	84.930	-0.178	0.240	0.459	0.685	84.930	0.874	0.293	0.003	0.028
SMG1P7	33.769	1.498	0.460	0.001	0.006	33.769	0.640	0.469	0.172	0.461	33.769	-0.858	0.529	0.105	0.288
LOC100506076	28.743	1.921	0.410	0.000	0.000	28.743	-0.022	0.441	0.961	0.979	28.743	-1.943	0.486	0.000	0.002
LOC100506083	64.074	-0.178	0.222	0.422	0.559	64.074	-0.050	0.216	0.818	0.905	64.074	0.128	0.257	0.617	0.790
ARL17B	111.433	0.345	0.250	0.167	0.287	111.433	-0.335	0.254	0.188	0.475	111.433	-0.680	0.293	0.020	0.102
LOC100506100	41.240	0.859	0.506	0.089	0.180	41.240	0.543	0.507	0.284	0.554	41.240	-0.316	0.582	0.587	0.768
LINC01503	189.760	0.669	0.442	0.130	0.237	189.760	0.707	0.441	0.109	0.401	189.760	0.037	0.509	0.941	0.973
LOC100506123	18.604	2.627	0.629	0.000	0.000	18.604	0.619	0.659	0.348	0.603	18.604	-2.008	0.727	0.006	0.045
LINC00867	81.012	-0.648	0.200	0.001	0.007	81.012	-0.139	0.187	0.457	0.683	81.012	0.509	0.230	0.027	0.123
GVQW3	23.553	-0.157	0.434	0.718	0.808	23.553	-0.432	0.437	0.322	0.583	23.553	-0.276	0.510	0.589	0.769
TTC21B-AS1	29.940	-1.430	0.658	0.030	0.078	29.940	-0.152	0.634	0.810	0.900	29.940	1.278	0.755	0.090	0.263
TMEM35B	16.446	0.259	0.430	0.547	0.670	16.446	-0.011	0.433	0.980	0.989	16.446	-0.270	0.500	0.590	0.770
SRP54-AS1	11.579	0.494	0.606	0.415	0.553	11.579	0.362	0.605	0.550	0.743	11.579	-0.132	0.695	0.849	0.927
TAF1A-AS1	19.985	-0.762	0.391	0.051	0.118	19.985	-0.284	0.366	0.438	0.671	19.985	0.478	0.451	0.289	0.525
HSFX1	9.581	1.698	0.630	0.007	0.026	9.581	0.565	0.662	0.394	0.639	9.581	-1.134	0.725	0.118	0.308
LINC00963	684.890	0.558	0.259	0.031	0.081	684.890	0.503	0.259	0.052	0.339	684.890	-0.055	0.298	0.855	0.930
LOC100506207	8.767	-0.895	0.783	0.253	0.388	8.767	-0.054	0.734	0.941	0.970	8.767	0.841	0.895	0.347	0.581
MIR210HG	565.618	1.286	0.929	0.166	0.286	565.618	0.748	0.929	0.421	0.658	565.618	-0.538	1.072	0.616	0.789
PAXBP1-AS1	49.960	0.501	0.243	0.039	0.096	49.960	-0.042	0.250	0.867	0.932	49.960	-0.543	0.285	0.057	0.199
RAB30-DT	65.314	0.579	0.328	0.078	0.162	65.314	-0.064	0.333	0.847	0.921	65.314	-0.643	0.382	0.092	0.267
LOC100506282	69.670	0.514	0.211	0.015	0.046	69.670	0.120	0.215	0.577	0.762	69.670	-0.394	0.246	0.109	0.294
TBC1D8-AS1	8.385	-0.449	0.693	0.517	0.645	8.385	0.209	0.657	0.751	0.867	8.385	0.657	0.789	0.405	0.632
ID2-AS1	19.305	0.805	0.862	0.350	0.490	19.305	0.692	0.861	0.422	0.658	19.305	-0.113	0.991	0.909	0.959
LOC100506302	21.225	-0.740	0.380	0.051	0.118	21.225	-0.339	0.358	0.343	0.600	21.225	0.401	0.440	0.362	0.594
LINC00958	1433.442	0.087	0.107	0.415	0.553	1433.442	-0.183	0.107	0.088	0.381	1433.442	-0.270	0.124	0.029	0.130
HOTAIRM1	106.294	0.960	0.244	0.000	0.001	106.294	0.241	0.249	0.334	0.593	106.294	-0.720	0.283	0.011	0.069
GPRC5D-AS1	48.261	0.215	0.315	0.493	0.625	48.261	0.236	0.311	0.449	0.678	48.261	0.020	0.362	0.955	0.979
LOC100506321	262.061	-0.659	0.263	0.012	0.040	262.061	-0.591	0.262	0.024	0.316	262.061	0.068	0.305	0.823	0.913
SP2-AS1	29.062	-1.006	0.396	0.011	0.037	29.062	-0.067	0.367	0.854	0.925	29.062	0.939	0.452	0.038	0.153
LINC00649	472.239	0.037	0.317	0.908	0.941	472.239	0.356	0.316	0.261	0.533	472.239	0.319	0.366	0.383	0.612
OTUD6B-AS1	311.762	-0.820	0.180	0.000	0.000	311.762	-0.166	0.175	0.342	0.599	311.762	0.653	0.207	0.002	0.019
LINC01006	19.939	0.642	0.430	0.136	0.245	19.939	0.560	0.427	0.190	0.475	19.939	-0.082	0.488	0.867	0.936
SLC16A1-AS1	203.985	-0.013	0.254	0.959	0.976	203.985	-0.160	0.253	0.528	0.729	203.985	-0.147	0.294	0.617	0.789
GAS6-DT	12.803	1.082	0.655	0.099	0.194	12.803	0.267	0.681	0.695	0.836	12.803	-0.814	0.761	0.285	0.520
PANTR1	145.181	0.689	0.307	0.025	0.068	145.181	0.604	0.306	0.049	0.337	145.181	-0.085	0.353	0.809	0.904
CBR3-AS1	135.598	1.111	0.257	0.000	0.000	135.598	0.324	0.261	0.214	0.493	135.598	-0.787	0.297	0.008	0.056
LINC00648	57.467	0.129	0.313	0.681	0.778	57.467	0.365	0.307	0.235	0.513	57.467	0.236	0.358	0.509	0.715
SPAG5-AS1	389.401	-0.260	0.249	0.296	0.434	389.401	-0.478	0.249	0.055	0.343	389.401	-0.217	0.288	0.451	0.670
RASSF8-AS1	71.993	0.900	0.242	0.000	0.002	71.993	0.060	0.253	0.811	0.901	71.993	-0.840	0.284	0.003	0.030
SRD5A3-AS1	51.032	0.007	0.393	0.986	0.992	51.032	0.419	0.385	0.276	0.547	51.032	0.412	0.450	0.360	0.593
TMEM147-AS1	337.143	0.097	0.239	0.685	0.783	337.143	-0.213	0.240	0.373	0.622	337.143	-0.310	0.277	0.263	0.497
LOC100506476	8.285	-0.105	0.703	0.881	0.923	8.285	0.104	0.689	0.880	0.939	8.285	0.209	0.809	0.796	0.898
LIFR-AS1	26.084	-0.116	0.551	0.833	0.890	26.084	0.180	0.544	0.740	0.861	26.084	0.296	0.634	0.640	0.805
PRKCZ-AS1	13.311	1.515	0.796	0.057	0.127	13.311	1.246	0.797	0.118	0.409	13.311	-0.269	0.904	0.766	0.881
SPTY2D1OS	81.807	-0.902	0.340	0.008	0.029	81.807	-0.135	0.326	0.680	0.827	81.807	0.767	0.391	0.050	0.182
L3MBTL2-AS1	80.305	-1.139	0.459	0.013	0.041	80.305	-1.064	0.456	0.020	0.306	80.305	0.075	0.534	0.889	0.947
LOC100506551	28.680	-0.466	0.319	0.145	0.257	28.680	0.125	0.298	0.673	0.823	28.680	0.592	0.363	0.104	0.286
NECTIN3-AS1	62.964	0.050	0.281	0.857	0.907	62.964	0.313	0.275	0.256	0.529	62.964	0.263	0.322	0.415	0.640

C16orf95	54.840	-0.421	0.265	0.112	0.213	54.840	0.093	0.251	0.711	0.845	54.840	0.514	0.302	0.089	0.262
PPP1R26-AS1	24.091	0.715	0.385	0.063	0.138	24.091	0.550	0.385	0.153	0.445	24.091	-0.165	0.439	0.707	0.848
VASH1-AS1	53.813	1.410	0.355	0.000	0.001	53.813	0.131	0.369	0.723	0.852	53.813	-1.279	0.414	0.002	0.022
LOC100506606	7.361	-0.099	0.644	0.879	0.921	7.361	-1.138	0.699	0.104	0.396	7.361	-1.040	0.802	0.195	0.417
PRKAR2A-AS1	119.257	-1.065	0.492	0.030	0.079	119.257	-0.355	0.487	0.466	0.690	119.257	0.710	0.568	0.211	0.436
LOC100506639	29.397	0.303	0.298	0.310	0.449	29.397	0.369	0.291	0.205	0.488	29.397	0.066	0.339	0.845	0.925
PXN-AS1	4593.493	0.533	0.226	0.018	0.054	4593.493	0.446	0.226	0.048	0.337	4593.493	-0.086	0.261	0.741	0.867
OCLN	13.672	0.725	0.462	0.117	0.220	13.672	0.252	0.472	0.594	0.774	13.672	-0.473	0.535	0.377	0.608
DDX11-AS1	11.112	-0.103	0.514	0.841	0.896	11.112	0.096	0.496	0.847	0.921	11.112	0.198	0.590	0.737	0.865
NRAV	182.954	0.046	0.142	0.745	0.829	182.954	-0.089	0.142	0.528	0.729	182.954	-0.136	0.166	0.413	0.638
MRPS30-DT	33.802	0.530	0.514	0.302	0.441	33.802	0.544	0.512	0.288	0.556	33.802	0.014	0.590	0.981	0.990
LOC100506679	15.320	0.737	0.476	0.121	0.226	15.320	0.996	0.463	0.031	0.322	15.320	0.259	0.529	0.625	0.795
IQCH-AS1	147.624	-0.347	0.196	0.077	0.161	147.624	0.552	0.185	0.003	0.297	147.624	0.899	0.222	0.000	0.001
PCAT6	63.884	1.384	0.677	0.041	0.099	63.884	0.943	0.679	0.165	0.455	63.884	-0.441	0.776	0.570	0.759
EBLN3P	1043.411	-0.504	0.224	0.024	0.066	1043.411	0.028	0.223	0.901	0.950	1043.411	0.532	0.258	0.039	0.156
ALOX12-AS1	29.884	0.738	0.326	0.023	0.065	29.884	-0.050	0.342	0.884	0.942	29.884	-0.787	0.385	0.041	0.161
NUP50-DT	113.772	0.112	0.237	0.637	0.745	113.772	0.092	0.236	0.697	0.837	113.772	-0.020	0.274	0.941	0.973
AFF1-AS1	47.325	1.220	0.475	0.010	0.035	47.325	0.555	0.479	0.246	0.521	47.325	-0.665	0.547	0.224	0.452
AZIN1-AS1	13.630	0.107	0.501	0.831	0.889	13.630	0.399	0.486	0.411	0.651	13.630	0.293	0.571	0.608	0.783
MIR497HG	34.513	0.301	0.307	0.328	0.468	34.513	0.498	0.300	0.097	0.392	34.513	0.197	0.349	0.572	0.760
TSPOAP1-AS1	452.247	0.156	0.112	0.166	0.286	452.247	0.008	0.112	0.942	0.971	452.247	-0.148	0.130	0.258	0.492
HOXD-AS2	17.140	1.622	0.502	0.001	0.007	17.140	0.863	0.514	0.093	0.388	17.140	-0.759	0.571	0.184	0.403
LOC100506804	25.853	0.522	0.496	0.293	0.431	25.853	0.211	0.498	0.672	0.823	25.853	-0.311	0.573	0.588	0.769
MYLK-AS1	30.191	0.093	0.329	0.779	0.853	30.191	0.485	0.317	0.125	0.417	30.191	0.393	0.373	0.292	0.528
LINC02475	31.226	0.414	0.659	0.530	0.656	31.226	0.364	0.658	0.580	0.764	31.226	-0.050	0.760	0.948	0.976
GAS1RR	64.382	-0.628	0.541	0.246	0.379	64.382	0.470	0.532	0.377	0.624	64.382	1.098	0.621	0.077	0.240
GIHCG	227.233	0.477	0.275	0.083	0.170	227.233	0.332	0.275	0.227	0.506	227.233	-0.145	0.317	0.647	0.810
TTN-AS1	21.848	-0.012	0.351	0.973	0.983	21.848	-0.239	0.353	0.497	0.711	21.848	-0.228	0.413	0.582	0.765
MKLN1-AS	93.503	-0.146	0.240	0.544	0.668	93.503	0.099	0.234	0.672	0.823	93.503	0.245	0.276	0.375	0.607
LOC100506885	6.219	-1.095	0.870	0.208	0.337	6.219	0.537	0.773	0.487	0.704	6.219	1.632	0.972	0.093	0.268
ELOA-AS1	198.000	-0.335	0.208	0.107	0.206	198.000	-0.098	0.205	0.632	0.799	198.000	0.237	0.240	0.324	0.559
NCMAP-DT	44.367	0.513	0.335	0.126	0.232	44.367	0.354	0.335	0.291	0.558	44.367	-0.159	0.386	0.680	0.831
PTPRG-AS1	123.002	-0.512	0.309	0.098	0.193	123.002	-0.219	0.306	0.475	0.696	123.002	0.294	0.357	0.411	0.637
TMCC1-AS1	149.631	-0.530	0.258	0.040	0.097	149.631	0.413	0.250	0.099	0.393	149.631	0.943	0.295	0.001	0.017
PITRM1-AS1	585.802	-0.588	0.304	0.053	0.121	585.802	-0.619	0.304	0.041	0.329	585.802	-0.031	0.351	0.929	0.967
LOC100507053	14.536	0.408	0.562	0.468	0.602	14.536	0.159	0.564	0.778	0.882	14.536	-0.249	0.650	0.702	0.845
CCAT1	5295.180	-1.432	0.190	0.000	0.000	5295.180	-0.245	0.189	0.195	0.478	5295.180	1.187	0.219	0.000	0.000
MELTF-AS1	28.048	1.270	0.544	0.019	0.056	28.048	0.577	0.550	0.294	0.561	28.048	-0.693	0.626	0.268	0.503
PSMD6-AS2	9.246	1.255	0.709	0.077	0.160	9.246	0.815	0.718	0.256	0.529	9.246	-0.440	0.804	0.584	0.766
LOC100507065	11.329	2.175	0.504	0.000	0.000	11.329	0.723	0.542	0.182	0.469	11.329	-1.452	0.575	0.012	0.072
CLIP1-AS1	12.341	-0.942	0.621	0.129	0.237	12.341	-0.240	0.583	0.680	0.827	12.341	0.702	0.713	0.324	0.559
LOC100507071	16.337	0.185	0.492	0.708	0.799	16.337	0.487	0.477	0.307	0.572	16.337	0.303	0.559	0.588	0.769
DLG1-AS1	22.553	-1.821	0.407	0.000	0.000	22.553	-0.642	0.346	0.063	0.350	22.553	1.179	0.463	0.011	0.069
PRC1-AS1	1493.704	-0.898	0.233	0.000	0.001	1493.704	-0.352	0.233	0.130	0.422	1493.704	0.545	0.269	0.043	0.166
LINC00707	148.440	-1.938	0.347	0.000	0.000	148.440	-0.450	0.334	0.179	0.467	148.440	1.489	0.398	0.000	0.004
SPART-AS1	18.489	-0.696	0.434	0.109	0.208	18.489	-0.475	0.417	0.255	0.528	18.489	0.221	0.507	0.663	0.820
LOC100507156	466.986	0.096	0.477	0.840	0.894	466.986	0.674	0.476	0.157	0.449	466.986	0.578	0.550	0.293	0.529
BOLA3-AS1	18.280	-0.031	0.560	0.956	0.973	18.280	0.465	0.541	0.390	0.635	18.280	0.496	0.637	0.437	0.658
LINC01012	27.015	0.150	0.398	0.706	0.798	27.015	-0.116	0.401	0.772	0.879	27.015	-0.266	0.466	0.568	0.758
SLFNL1-AS1	107.723	0.052	0.209	0.805	0.872	107.723	0.409	0.204	0.045	0.333	107.723	0.358	0.239	0.134	0.334
LINC00943	87.422	0.598	0.299	0.045	0.107	87.422	0.216	0.302	0.476	0.697	87.422	-0.382	0.346	0.269	0.504
LINC01578	493.667	-0.469	0.197	0.017	0.052	493.667	0.338	0.194	0.082	0.373	493.667	0.806	0.226	0.000	0.006
RNF157-AS1	59.826	0.814	0.212	0.000	0.001	59.826	-0.077	0.223	0.731	0.856	59.826	-0.891	0.251	0.000	0.007
SNHG16	1950.763	0.309	0.166	0.063	0.138	1950.763	0.341	0.166	0.040	0.327	1950.763	0.032	0.192	0.868	0.937
LOC100507250	28.859	-1.231	0.477	0.010	0.034	28.859	-1.176	0.471	0.013	0.305	28.859	0.055	0.561	0.922	0.964
STX18-AS1	50.432	-0.118	0.298	0.693	0.788	50.432	0.121	0.292	0.679	0.827	50.432	0.239	0.343	0.487	0.697
LOC100507283	16.551	0.591	0.656	0.368	0.508	16.551	0.280	0.659	0.671	0.822	16.551	-0.311	0.757	0.681	0.831
LOC100507291	54.075	0.323	0.443	0.466	0.600	54.075	0.672	0.438	0.125	0.417	54.075	0.349	0.507	0.491	0.701
TMEM44-AS1	70.648	-0.314	0.287	0.273	0.410	70.648	-0.205	0.283	0.468	0.691	70.648	0.109	0.332	0.744	0.868
ALKBH3-AS1	7.214	-0.092	0.686	0.893	0.931	7.214	-1.496	0.765	0.050	0.338	7.214	-1.403	0.870	0.107	0.291
SNHG19	951.866	0.891	0.345	0.010	0.033	951.866	0.637	0.345	0.065	0.354	951.866	-0.255	0.398	0.522	0.724
TARID	19.043	-0.850	0.481	0.077	0.161	19.043	0.182	0.445	0.683	0.829	19.043	1.032	0.545	0.058	0.202
MINCR	76.627	0.334	0.274	0.222	0.353	76.627	0.647	0.268	0.016	0.306	76.627	0.313	0.312	0.316	0.552
ERVK13-1	34.081	-0.277	0.403	0.493	0.625	34.081	-0.817	0.413	0.048	0.336	34.081	-0.540	0.480	0.260	0.495
ZSWIM8-AS1	40.965	0.466	0.356	0.191	0.316	40.965	0.154	0.359	0.667	0.819	40.965	-0.312	0.412	0.450	0.669
LOC100507334	11.631	1.882	0.478	0.000	0.001	11.631	1.013	0.505	0.045	0.334	11.631	-0.869	0.527	0.099	0.279
VIM-AS1	24584.764	-0.210	0.224	0.347	0.488	24584.764	0.320	0.224	0.152	0.444	24584.764	0.531	0.258	0.040	0.159

LOC100507373	161.399	-0.764	0.229	0.001	0.005	161.399	-0.159	0.223	0.476	0.697	161.399	0.605	0.263	0.022	0.106
INTS6-AS1	46.787	-1.150	0.356	0.001	0.007	46.787	0.170	0.328	0.603	0.781	46.787	1.320	0.403	0.001	0.014
LRP4-AS1	39.852	-0.716	0.333	0.032	0.082	39.852	0.205	0.312	0.512	0.719	39.852	0.921	0.379	0.015	0.085
TMLHE-AS1	17.935	0.644	0.550	0.241	0.374	17.935	0.030	0.559	0.957	0.978	17.935	-0.615	0.639	0.336	0.571
C1QTNF1-AS1	25.496	1.113	0.431	0.010	0.033	25.496	0.149	0.454	0.742	0.862	25.496	-0.964	0.505	0.056	0.197
LOC100507412	443.240	1.011	0.307	0.001	0.006	443.240	0.626	0.308	0.042	0.330	443.240	-0.385	0.354	0.278	0.512
MMP25-AS1	59.325	0.878	0.257	0.001	0.004	59.325	0.324	0.263	0.218	0.497	59.325	-0.554	0.297	0.062	0.210
LINC01605	81.419	0.091	0.374	0.809	0.875	81.419	-0.695	0.382	0.069	0.357	81.419	-0.786	0.440	0.074	0.234
TMEM178B	83.859	0.723	0.323	0.025	0.069	83.859	0.074	0.326	0.821	0.906	83.859	-0.649	0.374	0.083	0.250
MKNK1-AS1	49.585	-0.064	0.268	0.810	0.876	49.585	0.337	0.257	0.189	0.475	49.585	0.402	0.305	0.187	0.407
MICA	1684.566	0.230	0.148	0.122	0.226	1684.566	0.106	0.148	0.475	0.697	1684.566	-0.124	0.171	0.470	0.684
LOC100507437	40.391	0.419	0.464	0.366	0.507	40.391	0.186	0.465	0.689	0.832	40.391	-0.233	0.536	0.664	0.821
ZNF213-AS1	18.684	1.178	0.458	0.010	0.035	18.684	0.556	0.469	0.236	0.513	18.684	-0.622	0.526	0.237	0.466
NRSN2-AS1	99.043	-0.081	0.320	0.801	0.869	99.043	0.485	0.315	0.123	0.414	99.043	0.566	0.367	0.123	0.315
PSMB8-AS1	3245.961	1.063	0.275	0.000	0.001	3245.961	0.530	0.275	0.054	0.342	3245.961	-0.533	0.317	0.093	0.267
LINC02615	8.199	2.874	0.750	0.000	0.001	8.199	1.089	0.819	0.184	0.470	8.199	-1.785	0.833	0.032	0.138
SDCBP2-AS1	45.064	-0.518	0.467	0.267	0.403	45.064	0.348	0.453	0.442	0.673	45.064	0.866	0.534	0.105	0.289
EGFR-AS1	158.840	-1.578	0.521	0.002	0.011	158.840	0.114	0.514	0.825	0.909	158.840	1.691	0.600	0.005	0.040
LINC01569	35.760	0.776	0.563	0.168	0.288	35.760	1.000	0.559	0.074	0.363	35.760	0.225	0.644	0.727	0.859
LOC100507507	109.269	0.821	0.241	0.001	0.004	109.269	0.492	0.243	0.043	0.330	109.269	-0.329	0.277	0.234	0.463
LOC100507516	109.950	0.054	0.529	0.918	0.948	109.950	0.325	0.528	0.538	0.735	109.950	0.271	0.610	0.657	0.817
MCPH1-AS1	54.117	0.214	0.346	0.537	0.662	54.117	0.325	0.342	0.342	0.599	54.117	0.111	0.397	0.780	0.889
LINC01504	164.446	-0.377	0.208	0.070	0.150	164.446	-0.209	0.206	0.308	0.573	164.446	0.168	0.241	0.487	0.697
LOC100507547	206.057	0.439	0.295	0.137	0.247	206.057	0.557	0.294	0.058	0.348	206.057	0.118	0.340	0.728	0.859
LOC100507551	251.005	0.091	0.238	0.704	0.796	251.005	0.483	0.236	0.041	0.329	251.005	0.392	0.274	0.152	0.362
LOC100507557	184.041	-0.311	0.170	0.067	0.145	184.041	-0.115	0.167	0.489	0.705	184.041	0.196	0.197	0.320	0.555
LOC100507564	102.334	0.988	0.334	0.003	0.014	102.334	-0.634	0.346	0.067	0.354	102.334	-1.622	0.394	0.000	0.001
RAB11B-AS1	193.445	0.100	0.208	0.632	0.741	193.445	0.263	0.206	0.203	0.486	193.445	0.163	0.240	0.496	0.704
BHLHE40-AS1	828.264	1.061	0.351	0.002	0.012	828.264	0.434	0.351	0.216	0.495	828.264	-0.626	0.405	0.122	0.314
TGFBR3L	205.479	0.883	0.340	0.009	0.032	205.479	0.553	0.340	0.104	0.397	205.479	-0.330	0.391	0.400	0.627
LOC100507599	40.933	-0.169	0.281	0.547	0.671	40.933	0.160	0.270	0.555	0.746	40.933	0.329	0.321	0.307	0.542
TRIM52-AS1	147.145	0.142	0.240	0.555	0.677	147.145	0.287	0.238	0.228	0.506	147.145	0.145	0.276	0.600	0.778
NPIPB9	13.793	2.618	0.688	0.000	0.001	13.793	1.179	0.714	0.099	0.393	13.793	-1.439	0.777	0.064	0.213
RNF212B	9.041	1.707	0.725	0.019	0.055	9.041	0.911	0.742	0.220	0.499	9.041	-0.796	0.824	0.334	0.569
ZNF687-AS1	16.020	-1.015	0.563	0.071	0.152	16.020	0.205	0.507	0.686	0.831	16.020	1.220	0.634	0.054	0.193
GMDS-DT	34.210	1.037	0.420	0.013	0.042	34.210	0.765	0.421	0.069	0.358	34.210	-0.272	0.478	0.569	0.758
LINC02616	88.184	0.531	0.269	0.048	0.113	88.184	-0.312	0.276	0.257	0.530	88.184	-0.843	0.316	0.008	0.054
CPB2-AS1	188.027	-0.512	0.425	0.229	0.361	188.027	-0.718	0.425	0.092	0.386	188.027	-0.206	0.493	0.675	0.828
SMC4	4705.122	-0.608	0.202	0.003	0.012	4705.122	-0.281	0.202	0.164	0.454	4705.122	0.327	0.233	0.161	0.375
TBC1D3J	11.534	1.614	0.829	0.052	0.118	11.534	1.254	0.834	0.133	0.424	11.534	-0.360	0.933	0.700	0.843
GJC1	514.565	0.417	0.155	0.007	0.026	514.565	-0.294	0.157	0.061	0.350	514.565	-0.711	0.180	0.000	0.002
ATP5MF-PTCD1	103.870	-1.115	0.383	0.004	0.015	103.870	-0.518	0.376	0.168	0.457	103.870	0.597	0.442	0.177	0.394
RNF103-CHMP3	11.251	-1.702	0.657	0.010	0.033	11.251	-0.949	0.555	0.087	0.380	11.251	0.753	0.764	0.324	0.559
STIMATE-MUSTN1	16.571	-0.614	0.558	0.272	0.408	16.571	-1.377	0.579	0.017	0.306	16.571	-0.763	0.674	0.258	0.492
SLX1A-SULT1A3	45.929	1.546	0.412	0.000	0.001	45.929	0.669	0.421	0.112	0.405	45.929	-0.876	0.473	0.064	0.213
SEPT5-GP1BB	54.490	1.389	0.527	0.008	0.030	54.490	0.787	0.530	0.137	0.429	54.490	-0.602	0.605	0.320	0.555
EEF1E1-BLOC1S5	9.507	-1.768	0.808	0.029	0.076	9.507	-1.597	0.784	0.042	0.330	9.507	0.171	0.961	0.859	0.932
LHX4-AS1	186.437	-0.030	0.160	0.850	0.902	186.437	0.248	0.156	0.111	0.405	186.437	0.279	0.183	0.128	0.324
ST20-MTHFS	41.842	-0.266	0.297	0.369	0.510	41.842	-0.059	0.287	0.838	0.916	41.842	0.208	0.342	0.544	0.740
BLOC1S1-RDH5	12.567	2.567	0.545	0.000	0.000	12.567	0.350	0.609	0.565	0.753	12.567	-2.217	0.643	0.001	0.009
ARMCX5-GPRASP2	27.335	-0.131	0.325	0.688	0.785	27.335	0.099	0.313	0.752	0.867	27.335	0.230	0.373	0.539	0.736
NEDD8-MDP1	18.507	0.251	0.366	0.492	0.625	18.507	-0.055	0.370	0.881	0.940	18.507	-0.307	0.428	0.474	0.688
BCL2L2-PABPN1	63.868	0.773	0.306	0.011	0.038	63.868	0.261	0.311	0.400	0.644	63.868	-0.512	0.354	0.148	0.355
TEN1-CDK3	14.451	1.339	0.500	0.007	0.027	14.451	0.537	0.518	0.300	0.567	14.451	-0.803	0.574	0.162	0.375
RNASEK-C17orf49	34.698	0.024	0.327	0.941	0.963	34.698	0.427	0.315	0.176	0.465	34.698	0.402	0.372	0.279	0.514
CHURC1-FNTB	25.850	0.571	0.341	0.094	0.187	25.850	-0.387	0.363	0.287	0.555	25.850	-0.958	0.410	0.020	0.100
AP1M2	115.079	0.722	0.286	0.012	0.038	115.079	-0.268	0.292	0.359	0.611	115.079	-0.990	0.334	0.003	0.029
NPHP3-ACAD11	9.793	-0.102	0.732	0.889	0.928	9.793	0.151	0.711	0.832	0.913	9.793	0.253	0.840	0.763	0.880
NDUFC2-KCTD14	9.399	-0.022	0.570	0.969	0.981	9.399	-0.170	0.569	0.765	0.875	9.399	-0.149	0.666	0.824	0.913
COMMD3-BMI1	45.808	-1.062	0.514	0.039	0.096	45.808	0.027	0.498	0.956	0.978	45.808	1.089	0.590	0.065	0.215
MSH5-SAPCD1	26.555	1.744	0.870	NA	NA	26.555	0.420	0.879	NA	NA	26.555	-1.324	1.005	NA	NA
ATP6V1G2-DDX39B	1211.621	0.708	0.263	0.007	0.026	1211.621	0.096	0.263	0.714	0.847	1211.621	-0.612	0.304	0.044	0.169
PPT2-EGFL8	4786.687	0.031	0.172	0.859	0.908	4786.687	0.176	0.172	0.306	0.572	4786.687	0.145	0.199	0.465	0.681
PRH1-PRR4	88.746	0.183	0.224	0.414	0.552	88.746	-0.234	0.228	0.304	0.570	88.746	-0.417	0.263	0.113	0.301
SENP3-EIF4A1	9.004	-1.348	0.753	0.073	0.155	9.004	-0.984	0.716	0.169	0.459	9.004	0.364	0.884	0.681	0.831
P2RX5-TAX1BP3	338.457	0.041	0.302	0.892	0.930	338.457	-0.324	0.303	0.285	0.554	338.457	-0.365	0.350	0.297	0.532
SLMO2-ATP5E	13.964	-1.544	0.550	0.005	0.020	13.964	-1.066	0.510	0.037	0.324	13.964	0.479	0.646	0.459	0.676

TM4SF19-TCTEX1D2	47.473	2.051	0.429	0.000	0.000	47.473	0.514	0.451	0.254	0.528	47.473	-1.537	0.496	0.002	0.021
UBA2	3123.270	-0.353	0.151	0.019	0.056	3123.270	-0.328	0.151	0.030	0.322	3123.270	0.025	0.175	0.886	0.945
SAE1	2723.923	-0.598	0.306	0.050	0.116	2723.923	-0.695	0.306	0.023	0.316	2723.923	-0.097	0.353	0.784	0.892
FARSB	1610.539	-1.063	0.282	0.000	0.001	1610.539	-0.619	0.281	0.028	0.321	1610.539	0.444	0.326	0.173	0.390
ABCC5	150.684	-0.016	0.173	0.928	0.954	150.684	-0.274	0.174	0.116	0.408	150.684	-0.259	0.203	0.203	0.426
ABCB6	886.405	0.311	0.254	0.221	0.352	886.405	0.424	0.254	0.095	0.389	886.405	0.113	0.293	0.700	0.843
DNM1L	1925.610	-0.462	0.161	0.004	0.017	1925.610	-0.350	0.160	0.029	0.322	1925.610	0.112	0.186	0.545	0.741
CDH8	201.660	1.307	0.208	0.000	0.000	201.660	0.029	0.213	0.893	0.946	201.660	-1.278	0.242	0.000	0.000
ABCC9	105.721	-0.372	0.988	0.707	0.799	105.721	-3.106	1.007	0.002	0.297	105.721	-2.734	1.158	0.018	0.096
ABCF2	425.173	-0.251	0.426	0.556	0.678	425.173	-0.862	0.427	0.043	0.331	425.173	-0.611	0.493	0.215	0.441
MIR4738	39.078	-0.815	0.412	0.048	0.112	39.078	-0.035	0.396	0.930	0.965	39.078	0.780	0.472	0.099	0.278
MIR4426	424.900	0.048	0.238	0.840	0.895	424.900	0.390	0.237	0.100	0.394	424.900	0.342	0.274	0.213	0.438
MIR4444-1	34.775	-1.715	0.564	0.002	0.011	34.775	-1.469	0.554	0.008	0.297	34.775	0.246	0.659	0.709	0.849
MIR4442	96.792	-1.367	0.506	0.007	0.026	96.792	-0.385	0.499	0.441	0.672	96.792	0.983	0.584	0.092	0.266
TPTE2P5	14.984	1.455	0.587	0.013	0.042	14.984	-1.060	0.667	0.112	0.405	14.984	-2.515	0.735	0.001	0.009
NR1H3	345.402	-0.322	0.338	0.341	0.481	345.402	0.542	0.336	0.106	0.398	345.402	0.864	0.389	0.026	0.121
DNM3OS	75.200	-0.505	0.248	0.042	0.101	75.200	-0.401	0.244	0.100	0.394	75.200	0.104	0.289	0.719	0.854
COX17	521.322	1.078	0.347	0.002	0.009	521.322	-0.424	0.349	0.224	0.503	521.322	-1.502	0.401	0.000	0.004
PARTICL	70.492	-0.382	0.331	0.249	0.383	70.492	-0.081	0.326	0.804	0.897	70.492	0.301	0.382	0.431	0.653
LOC100630923	155.418	0.011	0.284	0.969	0.981	155.418	0.045	0.282	0.874	0.936	155.418	0.034	0.328	0.918	0.962
LINC01311	14.033	0.587	0.623	0.345	0.486	14.033	0.409	0.623	0.512	0.719	14.033	-0.179	0.716	0.803	0.901
RAET1E-AS1	306.876	-0.200	0.337	0.553	0.675	306.876	0.521	0.335	0.120	0.412	306.876	0.722	0.389	0.063	0.211
TIMM23B	54.033	-1.039	0.541	0.055	0.123	54.033	-1.526	0.545	0.005	0.297	54.033	-0.487	0.634	0.443	0.663
LOC100652758	14.265	0.174	0.485	0.720	0.809	14.265	0.177	0.478	0.711	0.845	14.265	0.003	0.558	0.995	0.997
PRICKLE2-AS1	79.522	-0.032	0.575	0.955	0.973	79.522	0.369	0.573	0.519	0.724	79.522	0.402	0.663	0.545	0.740
DSG2-AS1	312.987	-0.723	0.388	0.063	0.138	312.987	0.133	0.386	0.730	0.856	312.987	0.856	0.448	0.056	0.197
NNT-AS1	441.250	-0.583	0.231	0.012	0.038	441.250	0.112	0.229	0.625	0.794	441.250	0.695	0.266	0.009	0.062
LINC01023	24.875	-0.236	0.460	0.608	0.720	24.875	0.343	0.441	0.437	0.670	24.875	0.579	0.524	0.269	0.504
LINC00539	7.837	0.713	0.568	0.209	0.338	7.837	-1.046	0.678	0.123	0.414	7.837	-1.759	0.744	0.018	0.096
LOC100652999	205.206	-0.851	0.409	0.037	0.092	205.206	0.184	0.405	0.649	0.808	205.206	1.036	0.471	0.028	0.125
SCAMP2	794.856	0.299	0.114	0.009	0.030	794.856	-0.061	0.114	0.596	0.775	794.856	-0.359	0.132	0.006	0.049
SCAMP3	1899.665	-0.282	0.264	0.284	0.422	1899.665	-0.184	0.263	0.484	0.701	1899.665	0.098	0.304	0.748	0.871
IL18BP	683.452	1.177	0.342	0.001	0.004	683.452	0.572	0.342	0.094	0.388	683.452	-0.604	0.394	0.125	0.320
RWDD2B	778.023	-0.350	0.181	0.053	0.120	778.023	0.120	0.179	0.504	0.715	778.023	0.469	0.208	0.024	0.115
DPP3	1215.697	-0.196	0.218	0.370	0.510	1215.697	-0.133	0.218	0.541	0.737	1215.697	0.062	0.252	0.804	0.902
SNUPN	497.857	-0.465	0.112	0.000	0.000	497.857	0.051	0.109	0.636	0.801	497.857	0.517	0.129	0.000	0.002
HUWE1	5178.960	-0.623	0.166	0.000	0.001	5178.960	-0.053	0.165	0.750	0.867	5178.960	0.571	0.191	0.003	0.028
HIF1A-AS1	991.932	-1.370	0.535	0.010	0.035	991.932	-0.765	0.534	0.152	0.444	991.932	0.605	0.617	0.327	0.562
RCAN3AS	23.515	0.114	0.405	0.778	0.852	23.515	-0.069	0.405	0.864	0.931	23.515	-0.183	0.471	0.697	0.841
PTPRU	3779.573	-0.196	0.241	0.416	0.554	3779.573	0.374	0.241	0.120	0.412	3779.573	0.569	0.278	0.040	0.160
KLHL7-DT	17.233	-0.045	0.568	0.936	0.960	17.233	0.510	0.552	0.355	0.609	17.233	0.555	0.648	0.391	0.619
RBM5-AS1	77.041	-0.579	0.329	0.078	0.162	77.041	0.378	0.316	0.232	0.509	77.041	0.958	0.375	0.011	0.069
TSSC4	537.957	-0.156	0.399	0.696	0.790	537.957	-0.729	0.399	0.068	0.357	537.957	-0.573	0.461	0.214	0.440
ATP9A	1192.952	-0.142	0.225	0.527	0.653	1192.952	-0.006	0.225	0.977	0.987	1192.952	0.136	0.260	0.601	0.779
PDCD7	538.443	0.214	0.168	0.203	0.330	538.443	0.086	0.168	0.607	0.783	538.443	-0.127	0.194	0.511	0.717
GPC6	46.046	-0.769	0.428	0.072	0.153	46.046	0.693	0.407	0.088	0.382	46.046	1.461	0.485	0.003	0.026
MYZAP	11.236	-1.022	0.824	0.215	0.345	11.236	-0.183	0.760	0.809	0.900	11.236	0.838	0.942	0.373	0.605
PQBP1	1094.203	0.266	0.211	0.209	0.338	1094.203	0.027	0.212	0.899	0.949	1094.203	-0.239	0.244	0.328	0.562
EDIL3	228.868	0.472	0.451	0.296	0.434	228.868	0.339	0.451	0.453	0.680	228.868	-0.133	0.521	0.798	0.899
CARS-AS1	1057.552	-1.747	0.412	0.000	0.000	1057.552	-0.435	0.411	0.290	0.558	1057.552	1.312	0.476	0.006	0.045
HEIH	384.030	-0.044	0.164	0.789	0.861	384.030	0.196	0.162	0.227	0.506	384.030	0.239	0.189	0.204	0.428
CERS6-AS1	1076.586	-0.221	0.161	0.169	0.289	1076.586	0.105	0.160	0.510	0.718	1076.586	0.326	0.185	0.078	0.242
FSBP	52.170	-0.893	0.435	0.040	0.097	52.170	-0.721	0.430	0.094	0.388	52.170	0.173	0.505	0.732	0.861
ZMYM4-AS1	77.485	-1.402	0.579	0.015	0.047	77.485	-0.251	0.570	0.659	0.814	77.485	1.151	0.667	0.084	0.252
OCIAD1-AS1	8.578	-0.250	0.598	0.676	0.775	8.578	-0.864	0.626	0.168	0.457	8.578	-0.614	0.731	0.401	0.628
P4HA2-AS1	303.937	1.237	0.389	0.001	0.008	303.937	0.540	0.390	0.167	0.456	303.937	-0.697	0.449	0.120	0.312
PINK1-AS	1521.681	1.002	0.219	0.000	0.000	1521.681	-0.098	0.220	0.656	0.812	1521.681	-1.100	0.253	0.000	0.000
ALDH1L1-AS2	9.485	-0.812	0.842	0.335	0.475	9.485	0.768	0.785	0.328	0.588	9.485	1.579	0.950	0.096	0.274
UPK1A-AS1	16.992	1.499	0.889	0.092	0.184	16.992	1.265	0.890	0.155	0.448	16.992	-0.234	1.004	0.815	0.908
CERT1	875.687	0.308	0.305	0.313	0.452	875.687	0.111	0.305	0.717	0.848	875.687	-0.197	0.352	0.576	0.761
DPYD-AS1	76.178	-0.533	0.446	0.232	0.365	76.178	0.306	0.438	0.485	0.702	76.178	0.838	0.512	0.102	0.283
MTOR-AS1	27.048	-0.267	0.600	0.657	0.760	27.048	-0.775	0.605	0.200	0.483	27.048	-0.508	0.701	0.469	0.684
OPA1-AS1	130.804	-1.429	0.488	0.003	0.015	130.804	-0.608	0.482	0.208	0.490	130.804	0.822	0.563	0.144	0.350
HLTF-AS1	9.822	-1.005	0.746	0.178	0.300	9.822	-0.319	0.712	0.655	0.811	9.822	0.686	0.859	0.424	0.648
LMLN-AS1	22.097	-0.444	0.599	0.459	0.593	22.097	0.136	0.584	0.817	0.904	22.097	0.579	0.688	0.400	0.627
IPO9-AS1	298.778	0.377	0.149	0.011	0.037	298.778	-0.263	0.151	0.083	0.374	298.778	-0.640	0.174	0.000	0.005
SZT2-AS1	33.408	0.245	0.310	0.428	0.565	33.408	0.090	0.309	0.772	0.879	33.408	-0.156	0.359	0.664	0.821

SNRK-AS1	124.199	-1.570	0.416	0.000	0.001	124.199	-0.726	0.408	0.076	0.364	124.199	0.844	0.481	0.079	0.243
MED4-AS1	165.798	0.393	0.202	0.051	0.118	165.798	-0.104	0.204	0.610	0.785	165.798	-0.498	0.235	0.034	0.144
RBMS3-AS3	13.691	0.282	0.703	0.689	0.785	13.691	0.512	0.695	0.461	0.687	13.691	0.230	0.806	0.775	0.886
MED14OS	32.540	-1.109	0.696	0.111	0.211	32.540	-0.145	0.680	0.831	0.913	32.540	0.965	0.800	0.228	0.457
NCBP2-AS1	56.044	-0.901	0.371	0.015	0.047	56.044	-0.751	0.365	0.040	0.327	56.044	0.150	0.433	0.729	0.859
OXCT1-AS1	74.137	-1.465	0.421	0.000	0.003	74.137	-0.607	0.409	0.138	0.429	74.137	0.857	0.485	0.077	0.240
TTC3-AS1	120.540	-0.071	0.311	0.818	0.881	120.540	0.277	0.308	0.367	0.618	120.540	0.349	0.358	0.330	0.564
VIPR1-AS1	12.761	-0.410	0.723	0.571	0.690	12.761	-0.342	0.717	0.633	0.800	12.761	0.068	0.840	0.936	0.971
NAV2-AS2	113.777	-1.205	0.377	0.001	0.008	113.777	-0.064	0.368	0.863	0.930	113.777	1.141	0.434	0.009	0.059
SGO1-AS1	115.983	-0.506	0.239	0.034	0.087	115.983	-0.410	0.237	0.083	0.374	115.983	0.096	0.278	0.729	0.859
PRRT3-AS1	73.506	-0.337	0.223	0.131	0.239	73.506	0.105	0.209	0.616	0.788	73.506	0.442	0.254	0.082	0.249
UBXN7-AS1	41.710	-1.092	0.475	0.021	0.061	41.710	-0.298	0.459	0.517	0.722	41.710	0.794	0.547	0.146	0.353
TPRG1-AS1	23.484	0.164	0.369	0.656	0.759	23.484	0.004	0.368	0.991	0.995	23.484	-0.160	0.428	0.709	0.849
DGUOK-AS1	231.680	0.790	0.244	0.001	0.007	231.680	-0.041	0.248	0.869	0.933	231.680	-0.831	0.284	0.003	0.032
LINC00534	7.571	-1.034	0.607	0.088	0.178	7.571	-0.224	0.544	0.680	0.827	7.571	0.810	0.692	0.241	0.472
COX10-AS1	60.035	-0.468	0.243	0.054	0.122	60.035	0.038	0.230	0.869	0.933	60.035	0.506	0.278	0.068	0.222
NUCB1-AS1	822.476	0.463	0.245	0.058	0.130	822.476	0.335	0.245	0.170	0.460	822.476	-0.128	0.282	0.651	0.812
UBE2Q1-AS1	231.411	-0.048	0.199	0.808	0.874	231.411	0.104	0.197	0.597	0.776	231.411	0.152	0.229	0.506	0.712
STEAP3-AS1	430.736	0.466	0.381	0.221	0.352	430.736	0.342	0.380	0.369	0.619	430.736	-0.124	0.439	0.778	0.888
YEATS2-AS1	451.817	-0.593	0.363	0.102	0.199	451.817	0.343	0.361	0.341	0.599	451.817	0.936	0.418	0.025	0.118
LINC00364	9.687	-0.182	0.594	0.759	0.840	9.687	1.212	0.536	0.024	0.316	9.687	1.394	0.653	0.033	0.139
LINC00571	57.473	-0.453	0.324	0.162	0.281	57.473	-0.321	0.318	0.313	0.576	57.473	0.133	0.377	0.725	0.858
SAMSN1-AS1	8.835	4.193	0.773	0.000	0.000	8.835	2.197	0.830	0.008	0.297	8.835	-1.996	0.754	0.008	0.057
DIAPH3-AS1	43.075	-1.841	0.501	0.000	0.002	43.075	-0.796	0.479	0.097	0.392	43.075	1.045	0.577	0.070	0.226
B4GALT4-AS1	55.553	-0.426	0.309	0.169	0.289	55.553	0.336	0.295	0.255	0.528	55.553	0.762	0.352	0.031	0.133
COL4A2-AS1	178.709	1.340	0.242	0.000	0.000	178.709	0.215	0.247	0.383	0.629	178.709	-1.125	0.281	0.000	0.002
ALDH1L1-AS1	20.242	0.517	0.687	0.452	0.587	20.242	0.453	0.685	0.508	0.717	20.242	-0.064	0.790	0.936	0.971
ST3GAL6-AS1	68.420	-0.369	0.320	0.248	0.382	68.420	0.242	0.310	0.435	0.668	68.420	0.611	0.366	0.095	0.271
MYCBP2-AS1	179.704	-1.017	0.386	0.008	0.030	179.704	-0.232	0.382	0.543	0.739	179.704	0.785	0.445	0.078	0.241
TM4SF19-AS1	282.535	-0.017	0.208	0.934	0.959	282.535	-0.314	0.209	0.133	0.425	282.535	-0.297	0.242	0.221	0.448
OBI1-AS1	90.616	-1.531	0.389	0.000	0.001	90.616	-1.048	0.381	0.006	0.297	90.616	0.483	0.451	0.285	0.520
DENND5B-AS1	27.161	0.459	0.386	0.234	0.366	27.161	0.437	0.382	0.252	0.526	27.161	-0.022	0.440	0.959	0.981
SLC25A30-AS1	7.969	-0.884	0.708	0.212	0.342	7.969	1.009	0.610	0.098	0.392	7.969	1.893	0.778	0.015	0.085
UBE2CP5	16.001	1.134	0.491	0.021	0.060	16.001	-0.432	0.538	0.422	0.659	16.001	-1.565	0.596	0.009	0.059
ALMS1-IT1	9.370	-0.040	0.620	0.949	0.969	9.370	-1.655	0.705	0.019	0.306	9.370	-1.615	0.797	0.043	0.166
HOXA10-AS	201.982	-0.096	0.139	0.491	0.623	201.982	-0.115	0.138	0.403	0.645	201.982	-0.019	0.162	0.906	0.957
HOXC-AS1	27.566	0.343	0.496	0.489	0.622	27.566	0.795	0.486	0.102	0.394	27.566	0.452	0.564	0.423	0.647
HOXC-AS2	10.935	-0.748	0.520	0.151	0.265	10.935	-0.126	0.477	0.791	0.891	10.935	0.622	0.595	0.296	0.532
HOXC-AS3	144.243	-0.443	0.394	0.261	0.396	144.243	0.644	0.388	0.098	0.392	144.243	1.087	0.452	0.016	0.090
HOXC13-AS	132.674	0.419	0.262	0.111	0.211	132.674	-0.504	0.268	0.060	0.350	132.674	-0.923	0.308	0.003	0.027
APOBEC3B-AS1	133.909	0.917	0.345	0.008	0.028	133.909	0.174	0.347	0.615	0.788	133.909	-0.743	0.399	0.062	0.210
UGDH-AS1	22.268	-0.571	0.554	0.303	0.442	22.268	0.749	0.517	0.148	0.440	22.268	1.320	0.624	0.034	0.144
NALCN-AS1	23.499	-0.335	0.472	0.478	0.612	23.499	0.701	0.447	0.116	0.409	23.499	1.036	0.533	0.052	0.187
MRPS31P5	39.199	-0.579	0.342	0.090	0.181	39.199	-0.527	0.336	0.117	0.409	39.199	0.051	0.400	0.898	0.952
UST	406.114	0.003	0.144	0.981	0.988	406.114	-0.233	0.145	0.106	0.399	406.114	-0.237	0.168	0.158	0.370
APOBEC3A_B	13.818	0.921	0.491	0.060	0.133	13.818	0.618	0.494	0.211	0.491	13.818	-0.303	0.559	0.588	0.769
ARPC5	3467.828	0.212	0.147	0.148	0.262	3467.828	-0.177	0.147	0.227	0.505	3467.828	-0.389	0.169	0.022	0.106
ARPC4	1062.658	0.376	0.198	0.058	0.128	1062.658	-0.171	0.199	0.389	0.635	1062.658	-0.547	0.229	0.017	0.092
ARPC3	2435.226	0.725	0.186	0.000	0.001	2435.226	0.007	0.186	0.969	0.984	2435.226	-0.718	0.215	0.001	0.012
ARPC1B	1810.645	0.689	0.256	0.007	0.026	1810.645	-0.319	0.257	0.213	0.493	1810.645	-1.008	0.296	0.001	0.010
ACTR3	5068.870	-0.566	0.234	0.015	0.047	5068.870	-0.627	0.234	0.007	0.297	5068.870	-0.062	0.270	0.820	0.911
ACTR2	9042.277	-0.506	0.173	0.003	0.015	9042.277	-0.389	0.173	0.024	0.316	9042.277	0.117	0.200	0.557	0.749
TSPAN5	23.703	0.626	0.410	0.127	0.233	23.703	0.311	0.413	0.452	0.680	23.703	-0.315	0.472	0.505	0.711
TSPAN3	3158.055	0.211	0.122	0.083	0.170	3158.055	-0.005	0.122	0.965	0.982	3158.055	-0.216	0.141	0.124	0.317
FOXD3-AS1	31.982	1.885	0.498	0.000	0.001	31.982	0.460	0.513	0.370	0.619	31.982	-1.426	0.576	0.013	0.079
LIPE-AS1	21.172	-0.006	0.387	0.987	0.993	21.172	0.364	0.369	0.323	0.584	21.172	0.371	0.438	0.398	0.626
LOC100996351	281.016	-0.538	0.209	0.010	0.034	281.016	-0.163	0.206	0.430	0.664	281.016	0.375	0.241	0.120	0.311
LOC100996419	28.462	-0.425	0.502	0.397	0.535	28.462	0.290	0.487	0.552	0.744	28.462	0.715	0.574	0.213	0.439
ARLNC1	692.776	0.338	0.220	0.126	0.232	692.776	0.207	0.220	0.347	0.603	692.776	-0.131	0.254	0.608	0.783
LOC100996437	17.427	-0.763	0.394	0.053	0.120	17.427	0.063	0.343	0.855	0.926	17.427	0.826	0.443	0.062	0.210
LOC100996447	117.707	0.753	0.333	0.024	0.066	117.707	-0.152	0.338	0.653	0.811	117.707	-0.905	0.388	0.020	0.100
C5orf66	665.950	0.200	0.180	0.268	0.404	665.950	-0.244	0.181	0.177	0.466	665.950	-0.445	0.209	0.033	0.141
SEC22B3P	13.515	0.212	0.509	0.676	0.775	13.515	0.016	0.510	0.976	0.986	13.515	-0.197	0.592	0.739	0.866
ITPR1-DT	13.664	0.495	0.590	0.402	0.540	13.664	1.045	0.573	0.068	0.357	13.664	0.551	0.664	0.407	0.634
LINC01806	47.882	-0.626	0.272	0.021	0.060	47.882	-0.161	0.259	0.534	0.733	47.882	0.465	0.313	0.137	0.340
SRGAP2D	180.967	-0.756	0.373	0.043	0.103	180.967	0.103	0.369	0.781	0.885	180.967	0.859	0.430	0.046	0.173
SEC22B2P	18.730	0.564	0.455	0.215	0.345	18.730	-0.154	0.473	0.744	0.863	18.730	-0.718	0.537	0.181	0.400

NOTCH2NLC	31.920	-0.623	0.461	0.176	0.298	31.920	-0.259	0.451	0.566	0.753	31.920	0.364	0.532	0.494	0.703
LOC100996720	214.436	0.202	0.163	0.217	0.347	214.436	0.211	0.162	0.192	0.477	214.436	0.009	0.188	0.960	0.981
LOC100996724	120.284	0.259	0.567	0.648	0.754	120.284	0.473	0.566	0.403	0.645	120.284	0.215	0.654	0.743	0.868
HERC2P11	31.087	1.458	0.534	0.006	0.024	31.087	0.728	0.540	0.177	0.466	31.087	-0.730	0.613	0.234	0.463
LOC100996740	216.906	0.393	0.264	0.137	0.248	216.906	0.433	0.263	0.101	0.394	216.906	0.040	0.304	0.896	0.951
SPDYE11	23.574	-0.092	0.538	0.864	0.912	23.574	0.109	0.530	0.837	0.916	23.574	0.201	0.620	0.746	0.869
NOTCH2NLB	30.137	0.827	0.500	0.098	0.193	30.137	0.309	0.505	0.540	0.737	30.137	-0.518	0.577	0.369	0.601
LOC100996842	202.456	0.661	0.261	0.011	0.037	202.456	0.417	0.261	0.111	0.404	202.456	-0.244	0.300	0.416	0.642
LINC00621	7.910	0.458	0.808	0.571	0.690	7.910	0.662	0.796	0.405	0.646	7.910	0.204	0.921	0.825	0.914
CDH12	307.615	-0.198	0.294	0.500	0.631	307.615	0.259	0.293	0.376	0.624	307.615	0.457	0.339	0.178	0.395
NUBP2	1149.882	0.425	0.228	0.062	0.137	1149.882	-0.189	0.228	0.408	0.649	1149.882	-0.614	0.263	0.020	0.101
TSFM	621.893	-0.094	0.233	0.688	0.784	621.893	-0.370	0.233	0.113	0.405	621.893	-0.276	0.270	0.305	0.541
TSPAN1	104.962	0.312	0.310	0.314	0.454	104.962	0.441	0.308	0.152	0.444	104.962	0.129	0.356	0.718	0.853
PPIF	1803.129	-0.751	0.370	0.042	0.102	1803.129	-0.828	0.370	0.025	0.316	1803.129	-0.077	0.427	0.857	0.931
NPIPA7	120.686	0.564	0.548	0.304	0.443	120.686	-0.103	0.550	0.852	0.924	120.686	-0.667	0.634	0.293	0.528
NPIPA8	33.936	0.709	0.602	0.239	0.372	33.936	0.303	0.605	0.616	0.788	33.936	-0.406	0.695	0.559	0.751
CTDSP2	4325.086	-0.258	0.285	0.365	0.505	4325.086	0.334	0.285	0.240	0.516	4325.086	0.592	0.329	0.072	0.229
CHMP1B2P	31.085	-1.524	0.339	0.000	0.000	31.085	0.075	0.280	0.789	0.890	31.085	1.599	0.377	0.000	0.001
ZNF891	91.861	0.568	0.255	0.026	0.070	91.861	0.303	0.256	0.237	0.514	91.861	-0.265	0.294	0.366	0.598
NBPF19	1886.953	-0.201	0.282	0.476	0.610	1886.953	0.311	0.282	0.270	0.542	1886.953	0.511	0.326	0.116	0.305
FOXP4-AS1	74.595	1.016	0.333	0.002	0.011	74.595	0.522	0.336	0.120	0.412	74.595	-0.494	0.383	0.197	0.420
TBC1D3G	120.562	1.669	0.561	0.003	0.013	120.562	1.106	0.562	0.049	0.337	120.562	-0.563	0.645	0.382	0.611
TBC1D3K	49.504	1.928	0.609	0.002	0.008	49.504	1.881	0.608	0.002	0.297	49.504	-0.047	0.689	0.946	0.975
TBC1D3D	309.364	0.809	0.779	0.299	0.438	309.364	0.364	0.779	0.640	0.804	309.364	-0.444	0.899	0.621	0.792
NBPF26	688.645	-0.202	0.236	0.394	0.532	688.645	0.020	0.236	0.933	0.966	688.645	0.222	0.273	0.417	0.642
NUTM2B-AS1	215.318	-0.149	0.185	0.423	0.559	215.318	-0.097	0.184	0.596	0.775	215.318	0.051	0.214	0.811	0.906
TRIM10	21.895	-0.201	0.530	0.705	0.797	21.895	-0.358	0.530	0.499	0.712	21.895	-0.157	0.619	0.799	0.900
ARPC2	4191.930	-0.060	0.134	0.652	0.756	4191.930	-0.182	0.134	0.174	0.463	4191.930	-0.121	0.154	0.432	0.654
LINC00601	53.601	-0.251	0.267	0.347	0.488	53.601	0.080	0.257	0.757	0.871	53.601	0.330	0.306	0.281	0.515
RAD50	2754.593	-0.459	0.126	0.000	0.002	2754.593	0.032	0.126	0.800	0.896	2754.593	0.491	0.146	0.001	0.011
KIF20A	2029.630	0.550	0.255	0.031	0.080	2029.630	0.151	0.255	0.552	0.744	2029.630	-0.399	0.294	0.175	0.393
PREB	677.245	-0.284	0.337	0.400	0.538	677.245	-0.572	0.337	0.090	0.384	677.245	-0.288	0.390	0.461	0.678
HIPK3	644.241	-0.227	0.253	0.370	0.510	644.241	0.032	0.253	0.899	0.949	644.241	0.260	0.293	0.375	0.607
FEM1B	1308.648	-0.693	0.188	0.000	0.002	1308.648	-0.087	0.187	0.641	0.805	1308.648	0.606	0.217	0.005	0.042
CDH13	2020.280	0.004	0.397	0.991	0.995	2020.280	0.465	0.397	0.241	0.517	2020.280	0.461	0.458	0.314	0.550
ACTR1B	1181.993	0.171	0.152	0.260	0.396	1181.993	0.310	0.152	0.041	0.329	1181.993	0.139	0.175	0.429	0.652
ACTR1A	1364.067	0.199	0.275	0.470	0.604	1364.067	-0.610	0.275	0.027	0.318	1364.067	-0.809	0.318	0.011	0.069
ARL4C	211.821	-0.254	0.905	0.779	0.853	211.821	0.461	0.904	0.610	0.785	211.821	0.716	1.044	0.493	0.702
ARL4A	273.242	-0.400	0.176	0.023	0.064	273.242	-0.271	0.173	0.119	0.411	273.242	0.130	0.205	0.526	0.726
LINC02067	543.384	0.186	0.244	0.446	0.581	543.384	0.456	0.243	0.061	0.350	543.384	0.270	0.281	0.336	0.570
DNAL4	267.425	0.341	0.252	0.175	0.297	267.425	0.537	0.251	0.032	0.322	267.425	0.195	0.290	0.500	0.707
ZNF263	608.292	-0.435	0.208	0.036	0.090	608.292	-0.234	0.207	0.257	0.530	608.292	0.201	0.240	0.402	0.629
LRPPRC	4975.559	-0.937	0.187	0.000	0.000	4975.559	-0.019	0.187	0.919	0.960	4975.559	0.918	0.216	0.000	0.001
FRY	46.247	-0.861	0.301	0.004	0.017	46.247	0.265	0.263	0.314	0.577	46.247	1.125	0.336	0.001	0.011
PDIA6	5341.788	-0.178	0.187	0.342	0.482	5341.788	-0.135	0.187	0.472	0.693	5341.788	0.043	0.216	0.841	0.923
TRAP1	5291.856	-0.496	0.169	0.003	0.015	5291.856	-0.106	0.169	0.532	0.732	5291.856	0.390	0.195	0.046	0.173
OPTN	2648.726	0.395	0.240	0.100	0.196	2648.726	0.417	0.240	0.082	0.374	2648.726	0.022	0.277	0.936	0.971
BCAP31	4445.824	0.417	0.142	0.003	0.015	4445.824	-0.007	0.142	0.959	0.979	4445.824	-0.424	0.164	0.010	0.065
NAMPT	8024.025	0.210	0.274	0.443	0.579	8024.025	-0.467	0.274	0.088	0.380	8024.025	-0.677	0.316	0.032	0.138
RBM12	1142.547	-0.324	0.222	0.146	0.259	1142.547	-0.459	0.222	0.039	0.327	1142.547	-0.136	0.257	0.598	0.776
YAF2	372.720	-0.188	0.184	0.309	0.449	372.720	-0.328	0.184	0.075	0.364	372.720	-0.141	0.214	0.511	0.716
ARFRP1	515.561	-0.581	0.306	0.058	0.129	515.561	-0.709	0.306	0.021	0.312	515.561	-0.129	0.355	0.717	0.853
TOB1	870.149	0.452	0.209	0.031	0.080	870.149	0.316	0.209	0.131	0.423	870.149	-0.137	0.241	0.571	0.759
EZR-AS1	487.133	-0.876	0.307	0.004	0.018	487.133	-0.456	0.306	0.136	0.427	487.133	0.420	0.355	0.237	0.466
OSGEPL1-AS1	11.459	-0.694	0.511	0.174	0.295	11.459	-0.155	0.477	0.745	0.864	11.459	0.539	0.586	0.358	0.591
OGFR-AS1	14.140	0.601	0.546	0.271	0.408	14.140	0.647	0.539	0.230	0.507	14.140	0.047	0.620	0.940	0.973
YTHDF3-AS1	9.322	-0.181	0.687	0.792	0.863	9.322	-0.314	0.685	0.647	0.807	9.322	-0.133	0.803	0.868	0.937
LAMTOR5-AS1	294.165	0.586	0.159	0.000	0.002	294.165	0.243	0.160	0.129	0.420	294.165	-0.343	0.183	0.061	0.208
MMP24OS	967.333	0.709	0.318	0.026	0.070	967.333	0.641	0.318	0.044	0.331	967.333	-0.068	0.367	0.854	0.930
AKAP9	684.157	-0.063	0.176	0.723	0.812	684.157	-0.176	0.176	0.317	0.579	684.157	-0.114	0.204	0.578	0.763
FAM13A	783.109	-0.437	0.361	0.227	0.359	783.109	0.342	0.360	0.342	0.599	783.109	0.779	0.417	0.062	0.208
G3BP1	3534.583	-0.527	0.226	0.020	0.057	3534.583	-0.434	0.225	0.054	0.342	3534.583	0.092	0.260	0.723	0.856
SUGP2	986.774	-0.047	0.155	0.761	0.842	986.774	-0.084	0.155	0.585	0.768	986.774	-0.037	0.179	0.835	0.920
EBI3	45.398	3.612	0.466	0.000	0.000	45.398	1.185	0.515	0.021	0.314	45.398	-2.427	0.514	0.000	0.000
ADGRG2	630.275	-1.901	0.408	0.000	0.000	630.275	0.209	0.405	0.605	0.782	630.275	2.110	0.470	0.000	0.000
CDH17	48.257	0.386	0.284	0.174	0.296	48.257	0.468	0.279	0.093	0.388	48.257	0.082	0.324	0.799	0.899
MBNL2	1430.557	-0.917	0.242	0.000	0.001	1430.557	0.077	0.241	0.750	0.867	1430.557	0.993	0.279	0.000	0.006

ABI2	1361.896	-0.637	0.125	0.000	0.000	1361.896	-0.119	0.124	0.336	0.594	1361.896	0.517	0.144	0.000	0.006
CEBPZ	1501.577	-0.499	0.166	0.003	0.012	1501.577	-0.266	0.166	0.108	0.400	1501.577	0.233	0.192	0.225	0.453
PLXNC1	32.404	0.384	0.272	0.157	0.274	32.404	0.275	0.270	0.309	0.573	32.404	-0.109	0.312	0.726	0.858
TRIM28	8382.858	-0.271	0.127	0.032	0.084	8382.858	0.003	0.127	0.982	0.989	8382.858	0.274	0.146	0.061	0.208
RASA4	46.858	1.092	0.880	0.215	0.344	46.858	0.851	0.880	0.333	0.593	46.858	-0.241	1.013	0.812	0.906
AASS	111.103	-0.026	0.399	0.949	0.969	111.103	0.397	0.397	0.317	0.579	111.103	0.422	0.460	0.359	0.591
PDZK1IP1	776.003	2.882	0.763	0.000	0.001	776.003	1.207	0.763	0.114	0.406	776.003	-1.675	0.880	0.057	0.199
ATP6AP2	1832.501	0.431	0.097	0.000	0.000	1832.501	-0.004	0.097	0.968	0.983	1832.501	-0.435	0.112	0.000	0.003
CDH18	27.782	1.385	0.373	0.000	0.002	27.782	0.115	0.393	0.770	0.878	27.782	-1.270	0.437	0.004	0.033
FARP1	1162.253	-0.244	0.125	0.051	0.116	1162.253	0.096	0.124	0.437	0.670	1162.253	0.340	0.144	0.018	0.095
LPAR6	83.853	0.468	0.491	0.341	0.481	83.853	0.379	0.491	0.441	0.672	83.853	-0.089	0.567	0.875	0.940
LPCAT3	884.172	0.338	0.143	0.018	0.053	884.172	-0.109	0.143	0.445	0.675	884.172	-0.447	0.165	0.007	0.051
WASF2	5831.089	-0.378	0.309	0.221	0.352	5831.089	0.385	0.309	0.212	0.493	5831.089	0.763	0.357	0.032	0.139
SLC25A13	360.309	-0.889	0.330	0.007	0.026	360.309	-0.915	0.329	0.005	0.297	360.309	-0.026	0.382	0.946	0.975
SLC25A15	399.826	-0.701	0.316	0.027	0.072	399.826	-0.783	0.316	0.013	0.305	399.826	-0.082	0.366	0.823	0.913
ZNF197	329.351	0.021	0.127	0.868	0.914	329.351	-0.074	0.127	0.560	0.749	329.351	-0.095	0.148	0.519	0.722
SERF2	4973.863	1.143	0.261	0.000	0.000	4973.863	0.323	0.262	0.216	0.496	4973.863	-0.819	0.302	0.007	0.050
CDK2	1157.854	-0.265	0.204	0.194	0.319	1157.854	-0.360	0.204	0.077	0.368	1157.854	-0.095	0.235	0.685	0.834
RCL1	171.034	-0.826	0.246	0.001	0.005	171.034	-0.480	0.242	0.047	0.336	171.034	0.346	0.285	0.225	0.453
SORBS3	1751.967	0.649	0.230	0.005	0.019	1751.967	0.359	0.230	0.118	0.410	1751.967	-0.289	0.266	0.276	0.511
CNIH1	2423.403	-0.687	0.184	0.000	0.002	2423.403	0.115	0.184	0.531	0.731	2423.403	0.802	0.213	0.000	0.003
STAG3L5P-PVRIG2P-PILRB	175.242	1.315	0.356	0.000	0.002	175.242	0.554	0.358	0.122	0.413	175.242	-0.761	0.411	0.064	0.213
CAPN10-DT	12.550	0.459	0.495	0.354	0.494	12.550	0.341	0.493	0.489	0.705	12.550	-0.118	0.568	0.836	0.920
TENM1	136.719	0.211	0.600	0.725	0.813	136.719	0.379	0.599	0.527	0.729	136.719	0.167	0.692	0.809	0.904
RBM7	486.662	-0.831	0.194	0.000	0.000	486.662	-0.127	0.191	0.506	0.716	486.662	0.704	0.224	0.002	0.019
RBM6	769.701	0.089	0.191	0.642	0.748	769.701	-0.226	0.191	0.238	0.515	769.701	-0.314	0.221	0.155	0.365
CASC9	174.244	0.690	0.385	0.073	0.154	174.244	0.608	0.385	0.114	0.406	174.244	-0.083	0.443	0.852	0.929
RBM5	744.203	0.062	0.173	0.719	0.808	744.203	0.202	0.172	0.241	0.517	744.203	0.140	0.200	0.483	0.696
LHFPL2	762.342	-0.260	0.372	0.484	0.617	762.342	-0.911	0.372	0.014	0.306	762.342	-0.651	0.430	0.130	0.326
LHFPL6	601.008	0.488	0.153	0.001	0.007	601.008	0.142	0.153	0.353	0.607	601.008	-0.346	0.177	0.050	0.183
PRNCR1	25.621	0.851	0.340	0.012	0.040	25.621	-0.086	0.360	0.811	0.901	25.621	-0.937	0.403	0.020	0.102
TNK2	1107.967	0.404	0.254	0.111	0.212	1107.967	0.403	0.254	0.112	0.405	1107.967	-0.001	0.293	0.997	0.998
ALYREF	1196.976	-0.596	0.419	0.155	0.271	1196.976	-1.117	0.419	0.008	0.297	1196.976	-0.521	0.484	0.282	0.516
CDK4	3469.661	0.122	0.187	0.515	0.643	3469.661	-0.168	0.187	0.371	0.620	3469.661	-0.289	0.216	0.181	0.400
TXNDC9	380.164	-0.478	0.281	0.088	0.178	380.164	-0.675	0.281	0.016	0.306	380.164	-0.196	0.325	0.546	0.741
RALY-AS1	8.642	-0.267	0.559	0.633	0.742	8.642	0.255	0.523	0.626	0.794	8.642	0.522	0.634	0.410	0.636
CRTC3-AS1	18.744	-1.020	0.402	0.011	0.037	18.744	-0.161	0.358	0.653	0.810	18.744	0.858	0.457	0.060	0.205
VPS33B-DT	9.331	0.219	0.633	0.729	0.816	9.331	0.556	0.611	0.363	0.615	9.331	0.337	0.716	0.638	0.803
LOC101926913	24.355	-0.878	0.481	0.068	0.146	24.355	0.652	0.438	0.137	0.428	24.355	1.530	0.539	0.005	0.038
ANKRD18CP	35.972	0.953	0.308	0.002	0.010	35.972	0.305	0.317	0.336	0.595	35.972	-0.648	0.356	0.069	0.223
LINC01358	10.346	1.089	0.817	0.183	0.307	10.346	0.266	0.833	0.750	0.867	10.346	-0.823	0.947	0.385	0.614
LOC101926933	106.182	-0.605	0.298	0.042	0.102	106.182	0.536	0.288	0.062	0.350	106.182	1.141	0.340	0.001	0.011
SPRY4-AS1	9.238	0.230	0.740	0.756	0.838	9.238	0.151	0.737	0.837	0.916	9.238	-0.078	0.855	0.927	0.967
LOC101926943	447.438	-0.791	0.303	0.009	0.032	447.438	-0.073	0.301	0.808	0.899	447.438	0.718	0.350	0.040	0.159
DLGAP4-AS1	88.687	0.589	0.306	0.054	0.123	88.687	-0.032	0.310	0.919	0.960	88.687	-0.620	0.356	0.081	0.247
LOC101927021	156.473	0.020	0.207	0.923	0.951	156.473	0.415	0.203	0.040	0.328	156.473	0.395	0.237	0.095	0.272
CHROMR	315.951	0.162	0.150	0.282	0.420	315.951	0.140	0.149	0.349	0.604	315.951	-0.022	0.173	0.900	0.953
LOC101927045	16.780	-0.966	0.418	0.021	0.059	16.780	-0.223	0.375	0.551	0.744	16.780	0.742	0.477	0.120	0.311
LOC101927051	165.341	0.330	0.213	0.122	0.227	165.341	-0.018	0.214	0.932	0.966	165.341	-0.348	0.247	0.159	0.372
LINC02614	25.502	0.539	0.515	0.295	0.433	25.502	0.351	0.515	0.495	0.710	25.502	-0.188	0.593	0.751	0.872
CLMAT3	70.098	-0.340	0.368	0.356	0.496	70.098	-0.006	0.364	0.987	0.992	70.098	0.334	0.425	0.432	0.654
LOC101927100	229.728	-0.825	0.409	0.044	0.104	229.728	-0.220	0.406	0.589	0.770	229.728	0.606	0.472	0.199	0.423
ITFG1-AS1	69.147	0.034	0.246	0.889	0.928	69.147	0.057	0.243	0.816	0.904	69.147	0.022	0.285	0.938	0.972
SUCLG2-AS1	35.910	0.404	0.426	0.343	0.484	35.910	0.040	0.429	0.925	0.962	35.910	-0.363	0.494	0.462	0.679
PLCG1-AS1	479.003	-0.999	0.270	0.000	0.002	479.003	-0.627	0.269	0.020	0.306	479.003	0.372	0.312	0.233	0.462
LOC101927124	152.924	-0.094	0.262	0.719	0.809	152.924	-0.330	0.263	0.208	0.490	152.924	-0.236	0.305	0.438	0.659
ELFN1-AS1	81.421	-0.212	0.363	0.559	0.680	81.421	-0.538	0.365	0.140	0.431	81.421	-0.325	0.423	0.442	0.662
LOC101927151	232.726	0.072	0.168	0.670	0.770	232.726	0.087	0.167	0.601	0.779	232.726	0.016	0.194	0.936	0.971
LMO7-AS1	114.769	0.072	0.330	0.827	0.886	114.769	-0.262	0.331	0.429	0.664	114.769	-0.334	0.383	0.383	0.612
LOC101927157	14.576	-0.866	0.542	0.110	0.210	14.576	-1.318	0.554	0.017	0.306	14.576	-0.452	0.655	0.490	0.700
LOC101927164	39.805	-0.041	0.429	0.924	0.952	39.805	-0.413	0.432	0.339	0.597	39.805	-0.372	0.500	0.457	0.675
GATA2-AS1	305.709	0.222	0.148	0.135	0.244	305.709	-0.207	0.150	0.167	0.456	305.709	-0.429	0.173	0.013	0.078
LOC101927178	58.280	-1.143	0.369	0.002	0.010	58.280	-0.253	0.351	0.471	0.693	58.280	0.889	0.424	0.036	0.148
LOC101927179	54.323	-1.283	0.322	0.000	0.001	54.323	-0.682	0.305	0.025	0.316	54.323	0.601	0.373	0.107	0.291
ADGRL3-AS1	12.802	0.915	0.567	0.106	0.204	12.802	0.642	0.569	0.259	0.532	12.802	-0.274	0.647	0.672	0.826
LOC101927188	66.778	1.078	0.262	0.000	0.000	66.778	0.213	0.269	0.428	0.663	66.778	-0.864	0.304	0.005	0.038
FAM111A-DT	82.347	0.265	0.398	0.505	0.635	82.347	0.360	0.397	0.365	0.616	82.347	0.094	0.459	0.838	0.921

ZNF451-AS1	194.982	-1.412	0.336	0.000	0.000	194.982	-0.158	0.329	0.630	0.797	194.982	1.254	0.387	0.001	0.015
UBR5-AS1	126.590	0.553	0.280	0.048	0.112	126.590	-0.088	0.283	0.757	0.871	126.590	-0.641	0.325	0.049	0.180
LINC01969	54.640	-1.022	0.550	0.063	0.138	54.640	0.232	0.538	0.666	0.819	54.640	1.254	0.632	0.047	0.176
LOC101927245	25.310	0.164	0.395	0.678	0.776	25.310	0.996	0.370	0.007	0.297	25.310	0.832	0.438	0.057	0.200
LOC101927267	36.735	-0.122	0.324	0.706	0.798	36.735	-0.151	0.322	0.639	0.803	36.735	-0.028	0.377	0.940	0.973
LINC02466	10.478	-0.854	0.561	0.128	0.235	10.478	0.407	0.488	0.404	0.646	10.478	1.261	0.624	0.043	0.167
MANEA-DT	9.780	-0.428	0.771	0.579	0.697	9.780	0.568	0.736	0.440	0.672	9.780	0.996	0.876	0.255	0.489
LOC101927322	53.654	-1.430	0.369	0.000	0.001	53.654	-0.226	0.344	0.512	0.719	53.654	1.204	0.421	0.004	0.037
PLS3-AS1	15.542	0.898	0.451	0.046	0.109	15.542	1.122	0.441	0.011	0.301	15.542	0.225	0.501	0.653	0.814
CACNA2D1-AS1	10.086	-0.154	1.143	0.893	0.931	10.086	1.123	1.109	0.311	0.575	10.086	1.276	1.299	0.326	0.560
LOC101927365	112.255	-0.199	0.242	0.411	0.550	112.255	0.084	0.238	0.725	0.853	112.255	0.282	0.279	0.311	0.546
LOC101927377	8.600	0.928	0.669	0.165	0.285	8.600	0.451	0.679	0.507	0.716	8.600	-0.478	0.770	0.535	0.733
LOC101927391	35.914	-0.367	0.393	0.350	0.490	35.914	0.335	0.373	0.370	0.619	35.914	0.702	0.446	0.116	0.305
TMED2-DT	1106.897	-0.786	0.294	0.007	0.027	1106.897	-0.233	0.293	0.427	0.663	1106.897	0.553	0.339	0.103	0.286
FHAD1-AS1	21.973	-1.027	0.446	0.021	0.061	21.973	-0.124	0.408	0.760	0.872	21.973	0.902	0.508	0.076	0.237
LOC101927420	8.532	-1.221	0.599	0.042	0.101	8.532	0.026	0.501	0.959	0.979	8.532	1.247	0.669	0.062	0.210
PANO1	29.866	-0.430	0.362	0.235	0.368	29.866	-0.262	0.353	0.458	0.685	29.866	0.168	0.421	0.689	0.837
LOC101927437	33.436	0.701	0.531	0.187	0.311	33.436	0.529	0.531	0.320	0.581	33.436	-0.173	0.611	0.778	0.888
MRPS9-AS1	402.513	-0.022	0.181	0.904	0.939	402.513	-0.099	0.181	0.586	0.768	402.513	-0.077	0.210	0.714	0.851
LINC01273	19.610	0.317	0.578	0.584	0.700	19.610	0.381	0.573	0.506	0.716	19.610	0.064	0.663	0.923	0.964
LOC101927550	72.110	-0.793	0.296	0.007	0.027	72.110	-0.235	0.285	0.408	0.649	72.110	0.558	0.341	0.102	0.284
CEBPB-AS1	1287.541	0.622	0.303	0.040	0.098	1287.541	0.299	0.303	0.325	0.586	1287.541	-0.323	0.350	0.356	0.590
LOC101927572	19.784	0.511	0.429	0.234	0.366	19.784	0.166	0.433	0.701	0.839	19.784	-0.345	0.497	0.488	0.698
LINC01186	17.208	-0.038	0.392	0.923	0.951	17.208	0.138	0.381	0.717	0.849	17.208	0.176	0.450	0.696	0.841
LINC01271	13.240	-0.119	0.468	0.799	0.868	13.240	0.338	0.440	0.442	0.673	13.240	0.457	0.529	0.388	0.616
NCK1-DT	175.175	-0.139	0.202	0.491	0.623	175.175	0.473	0.196	0.016	0.306	175.175	0.613	0.231	0.008	0.056
ZNF529-AS1	34.486	0.397	0.357	0.267	0.403	34.486	0.458	0.353	0.194	0.478	34.486	0.062	0.408	0.880	0.943
LOC101927604	125.704	0.843	0.348	0.015	0.047	125.704	-0.049	0.352	0.889	0.945	125.704	-0.892	0.404	0.027	0.123
RNF139-AS1	9.936	0.296	0.695	0.670	0.770	9.936	-0.235	0.713	0.742	0.862	9.936	-0.531	0.820	0.517	0.721
LINC01534	18.025	-0.712	0.571	0.213	0.342	18.025	0.355	0.534	0.506	0.716	18.025	1.068	0.647	0.099	0.279
ADNP-AS1	26.826	-1.145	0.456	0.012	0.039	26.826	-0.334	0.429	0.436	0.669	26.826	0.811	0.524	0.121	0.313
LOC101927668	2132.827	-0.259	0.290	0.371	0.512	2132.827	0.423	0.289	0.144	0.435	2132.827	0.682	0.335	0.041	0.163
CD81-AS1	159.926	0.092	0.198	0.643	0.749	159.926	0.232	0.196	0.236	0.513	159.926	0.140	0.228	0.539	0.736
HSFX4	21.534	-0.750	0.499	0.133	0.241	21.534	-0.693	0.491	0.158	0.451	21.534	0.058	0.585	0.922	0.963
LINC02541	13.183	0.503	0.569	0.377	0.517	13.183	0.289	0.570	0.612	0.786	13.183	-0.214	0.656	0.745	0.869
LOC101927699	15.792	0.565	0.822	0.492	0.624	15.792	0.374	0.822	0.649	0.808	15.792	-0.190	0.947	0.841	0.923
FOXCUT	8.248	-0.082	0.579	0.888	0.927	8.248	-0.589	0.602	0.327	0.588	8.248	-0.508	0.702	0.470	0.684
LINC01029	52.548	-1.112	0.251	0.000	0.000	52.548	0.434	0.210	0.039	0.327	52.548	1.546	0.277	0.000	0.000
LOC101927727	10.602	1.221	0.583	0.036	0.091	10.602	-0.083	0.628	0.895	0.948	10.602	-1.304	0.694	0.061	0.206
LINC02202	9.694	3.003	0.983	0.002	0.011	9.694	0.013	1.063	0.991	0.995	9.694	-2.991	1.165	0.010	0.067
EMSLR	175.162	-0.409	0.203	0.044	0.105	175.162	-0.112	0.199	0.573	0.758	175.162	0.296	0.235	0.206	0.431
LOC101927751	29.914	-0.386	0.309	0.212	0.341	29.914	0.159	0.290	0.585	0.768	29.914	0.544	0.351	0.122	0.313
LOC101927752	333.652	0.606	0.327	0.064	0.140	333.652	0.689	0.327	0.035	0.323	333.652	0.083	0.377	0.826	0.914
RNFT1-DT	29.145	-1.371	0.672	0.041	0.100	29.145	0.338	0.637	0.596	0.775	29.145	1.709	0.765	0.026	0.119
TH2LCRR	403.485	-0.158	0.122	0.195	0.321	403.485	0.139	0.120	0.244	0.519	403.485	0.298	0.140	0.034	0.143
COA6-AS1	208.856	0.118	0.245	0.631	0.740	208.856	-0.422	0.247	0.088	0.380	208.856	-0.539	0.285	0.059	0.202
HDAC2-AS2	7.248	-1.084	1.091	0.320	0.460	7.248	0.258	1.033	0.803	0.897	7.248	1.342	1.242	0.280	0.514
LOC101927770	29.016	-0.634	0.385	0.099	0.194	29.016	-0.129	0.366	0.725	0.853	29.016	0.506	0.442	0.253	0.485
SATB1-AS1	25.963	-0.493	0.485	0.309	0.449	25.963	0.541	0.463	0.243	0.518	25.963	1.034	0.551	0.061	0.206
LINC01303	20.083	0.533	0.376	0.156	0.273	20.083	-0.211	0.398	0.596	0.775	20.083	-0.744	0.450	0.098	0.277
LOC101927789	236.383	1.957	0.511	0.000	0.001	236.383	0.700	0.515	0.174	0.463	236.383	-1.256	0.590	0.033	0.141
BZW1-AS1	137.686	-1.100	0.394	0.005	0.021	137.686	-0.405	0.389	0.298	0.565	137.686	0.695	0.455	0.127	0.322
MIR548XHG	92.853	-0.340	0.228	0.137	0.247	92.853	-0.023	0.223	0.917	0.959	92.853	0.317	0.263	0.229	0.457
LOC101927809	50.541	1.166	0.410	0.005	0.018	50.541	0.678	0.414	0.101	0.394	50.541	-0.488	0.471	0.300	0.535
HMMR-AS1	163.015	-0.727	0.167	0.000	0.000	163.015	0.013	0.159	0.935	0.967	163.015	0.740	0.191	0.000	0.003
PAQR9-AS1	15.480	0.788	0.437	0.071	0.152	15.480	-0.351	0.472	0.456	0.683	15.480	-1.139	0.528	0.031	0.134
LINC01687	23.548	0.561	0.450	0.212	0.342	23.548	0.437	0.449	0.331	0.591	23.548	-0.125	0.517	0.809	0.904
MYOSLID-AS1	83.338	-0.764	0.292	0.009	0.031	83.338	-0.288	0.283	0.308	0.573	83.338	0.476	0.337	0.158	0.370
GBAT2	14.635	0.346	0.463	0.455	0.590	14.635	-0.701	0.493	0.155	0.448	14.635	-1.047	0.561	0.062	0.210
PKN2-AS1	7.403	-0.263	0.886	0.767	0.846	7.403	1.125	0.830	0.175	0.464	7.403	1.388	0.992	0.162	0.375
EMC1-AS1	191.047	-0.945	0.301	0.002	0.009	191.047	-0.090	0.296	0.760	0.872	191.047	0.854	0.347	0.014	0.080
LINC01843	47.599	-0.917	0.573	0.109	0.209	47.599	0.344	0.558	0.537	0.735	47.599	1.261	0.656	0.055	0.194
LINC01572	26.787	-0.487	0.411	0.236	0.369	26.787	0.105	0.394	0.791	0.891	26.787	0.592	0.470	0.208	0.433
LOC101927974	26.241	-0.268	0.421	0.525	0.652	26.241	0.355	0.402	0.377	0.625	26.241	0.623	0.479	0.193	0.416
EDRF1-AS1	44.211	-1.110	0.362	0.002	0.010	44.211	0.157	0.334	0.638	0.803	44.211	1.267	0.410	0.002	0.022
LOC101927989	25.345	-0.287	0.349	0.410	0.548	25.345	0.013	0.334	0.970	0.984	25.345	0.300	0.401	0.454	0.673
LOC101928000	12.586	-1.503	0.560	0.007	0.026	12.586	0.015	0.468	0.974	0.986	12.586	1.518	0.624	0.015	0.085

LOC101928012	20.818	-0.979	0.673	0.146	0.259	20.818	-0.002	0.649	0.998	0.999	20.818	0.977	0.772	0.206	0.430
LOC101928021	26.215	1.113	0.616	0.071	0.151	26.215	0.099	0.636	0.876	0.937	26.215	-1.014	0.719	0.158	0.371
LOC101928042	13.942	0.600	0.691	0.385	0.524	13.942	0.067	0.698	0.924	0.961	13.942	-0.533	0.801	0.506	0.712
CNOT2-DT	63.900	0.424	0.333	0.202	0.329	63.900	0.477	0.329	0.147	0.438	63.900	0.053	0.380	0.889	0.947
LOC101928063	27.863	0.498	0.395	0.207	0.335	27.863	0.218	0.397	0.582	0.766	27.863	-0.280	0.456	0.539	0.736
LOC101928069	78.376	0.061	0.282	0.829	0.887	78.376	0.532	0.274	0.052	0.341	78.376	0.471	0.321	0.142	0.348
PCAT7	36.163	0.224	0.355	0.528	0.654	36.163	0.225	0.352	0.524	0.727	36.163	0.001	0.409	0.999	0.999
LOC101928100	39.687	1.325	0.393	0.001	0.005	39.687	0.645	0.399	0.106	0.398	39.687	-0.679	0.452	0.133	0.331
SNHG31	7.169	0.403	0.922	0.662	0.765	7.169	0.085	0.927	0.927	0.963	7.169	-0.318	1.068	0.766	0.881
HDAC4-AS1	10.276	-0.343	0.781	0.660	0.763	10.276	1.422	0.717	0.047	0.336	10.276	1.765	0.866	0.042	0.163
LOC101928120	953.831	1.531	0.306	0.000	0.000	953.831	0.648	0.307	0.035	0.322	953.831	-0.883	0.354	0.013	0.076
LOC101928140	60.660	-0.867	0.460	0.059	0.131	60.660	-0.245	0.451	0.587	0.768	60.660	0.622	0.530	0.241	0.471
LOC101928143	28.623	-1.103	0.451	0.015	0.045	28.623	0.405	0.408	0.321	0.582	28.623	1.508	0.507	0.003	0.028
FAM243A	22.564	-0.316	0.575	0.583	0.700	22.564	0.115	0.563	0.838	0.916	22.564	0.431	0.661	0.515	0.719
LINC01179	19.756	-1.157	0.407	0.004	0.018	19.756	0.214	0.345	0.536	0.734	19.756	1.371	0.453	0.002	0.025
ITGA9-AS1	36.100	0.451	0.513	0.379	0.519	36.100	0.552	0.510	0.279	0.549	36.100	0.101	0.589	0.864	0.935
LAMA5-AS1	27.413	-0.388	0.722	0.591	0.706	27.413	0.490	0.710	0.490	0.706	27.413	0.879	0.829	0.289	0.525
ZNF426-DT	10.990	0.601	0.680	0.377	0.517	10.990	0.712	0.669	0.287	0.556	10.990	0.111	0.769	0.885	0.945
DARS-AS1	210.729	-0.677	0.283	0.017	0.050	210.729	0.351	0.278	0.207	0.489	210.729	1.027	0.325	0.002	0.018
LOC101928323	38.112	-0.572	0.395	0.147	0.261	38.112	0.227	0.377	0.546	0.741	38.112	0.799	0.450	0.076	0.237
LOC101928371	175.785	-0.272	0.349	0.435	0.571	175.785	0.175	0.346	0.613	0.786	175.785	0.447	0.402	0.266	0.500
PTOV1-AS2	706.870	1.052	0.238	0.000	0.000	706.870	0.349	0.238	0.143	0.435	706.870	-0.703	0.275	0.010	0.068
EIF2AK3-DT	18.285	-0.412	0.462	0.373	0.513	18.285	0.248	0.437	0.571	0.757	18.285	0.659	0.525	0.209	0.435
LOC101928424	22.823	-1.098	0.456	0.016	0.049	22.823	0.177	0.411	0.666	0.819	22.823	1.275	0.514	0.013	0.078
ATXN1-AS1	9.465	-0.232	0.784	0.767	0.846	9.465	0.911	0.740	0.218	0.498	9.465	1.143	0.882	0.195	0.417
LOC101928445	70.863	0.679	0.195	0.001	0.003	70.863	-0.082	0.206	0.690	0.832	70.863	-0.761	0.232	0.001	0.013
NALT1	12.574	0.718	0.778	0.356	0.496	12.574	1.245	0.764	0.103	0.396	12.574	0.527	0.881	0.549	0.744
LOC101928489	55.973	-0.566	0.475	0.233	0.366	55.973	0.272	0.466	0.559	0.748	55.973	0.838	0.546	0.124	0.318
OR2A1-AS1	372.540	0.450	0.257	0.080	0.165	372.540	0.468	0.257	0.068	0.357	372.540	0.018	0.296	0.951	0.978
LOC101928504	8.198	-1.424	0.663	0.032	0.082	8.198	-0.107	0.560	0.849	0.922	8.198	1.318	0.743	0.076	0.237
LOC101928525	555.544	-0.353	0.144	0.014	0.044	555.544	0.087	0.141	0.540	0.737	555.544	0.439	0.165	0.008	0.056
PIGBOS1	176.433	0.177	0.227	0.437	0.573	176.433	-0.087	0.228	0.703	0.840	176.433	-0.264	0.263	0.317	0.553
SEPTIN7-AS1	11.258	-0.466	0.569	0.413	0.551	11.258	-0.075	0.546	0.891	0.945	11.258	0.392	0.655	0.550	0.744
ZNF649-AS1	17.326	-0.925	0.502	0.065	0.141	17.326	-0.142	0.468	0.761	0.872	17.326	0.783	0.574	0.173	0.390
RAP2C-AS1	47.504	-0.785	0.472	0.096	0.191	47.504	-0.283	0.463	0.542	0.738	47.504	0.502	0.545	0.357	0.590
P4HA3-AS1	6.256	0.431	0.765	0.573	0.692	6.256	-1.116	0.869	0.199	0.482	6.256	-1.547	0.973	0.112	0.298
LINC01775	7.436	-0.572	0.963	0.552	0.674	7.436	0.820	0.911	0.368	0.618	7.436	1.393	1.089	0.201	0.424
ARHGEF35-AS1	111.678	-0.206	0.319	0.518	0.646	111.678	0.150	0.315	0.633	0.800	111.678	0.356	0.367	0.332	0.567
LOC101928617	874.601	0.281	0.349	0.421	0.558	874.601	0.476	0.349	0.172	0.461	874.601	0.195	0.403	0.628	0.797
LOC101928626	719.106	1.181	0.352	0.001	0.005	719.106	0.290	0.352	0.411	0.650	719.106	-0.891	0.406	0.028	0.126
SMIM15-AS1	437.905	-1.057	0.266	0.000	0.001	437.905	-0.312	0.264	0.237	0.514	437.905	0.745	0.307	0.015	0.086
ARNTL2-AS1	12.503	-1.085	0.753	0.149	0.263	12.503	-0.778	0.734	0.290	0.558	12.503	0.308	0.877	0.726	0.858
CTC-338M12.4	136.012	0.712	0.434	0.101	0.197	136.012	0.776	0.433	0.073	0.362	136.012	0.064	0.500	0.899	0.952
LOC101928659	12.635	0.696	0.583	0.233	0.365	12.635	1.132	0.569	0.047	0.335	12.635	0.436	0.654	0.505	0.712
LOC101928673	28.875	-0.335	0.345	0.332	0.472	28.875	0.126	0.329	0.701	0.839	28.875	0.461	0.395	0.243	0.474
SCAT1	76.087	0.286	0.374	0.445	0.581	76.087	0.763	0.367	0.038	0.327	76.087	0.478	0.427	0.263	0.497
MIR762HG	186.471	1.017	0.305	0.001	0.005	186.471	0.461	0.306	0.133	0.424	186.471	-0.557	0.352	0.113	0.301
LOC101928737	10.990	-0.930	0.577	0.107	0.206	10.990	-0.343	0.540	0.525	0.727	10.990	0.586	0.665	0.378	0.609
LINC01807	35.938	0.066	0.398	0.869	0.915	35.938	0.453	0.390	0.246	0.521	35.938	0.387	0.456	0.396	0.624
LINC02328	14.531	0.427	0.798	0.593	0.708	14.531	0.858	0.788	0.277	0.547	14.531	0.431	0.912	0.637	0.803
MAST4-AS1	7.476	0.782	0.560	0.163	0.282	7.476	-0.646	0.627	0.303	0.569	7.476	-1.428	0.696	0.040	0.159
LOC101928837	13.453	1.029	0.629	0.102	0.198	13.453	0.914	0.628	0.145	0.438	13.453	-0.115	0.714	0.872	0.939
LOC101928841	161.210	0.267	0.656	0.684	0.782	161.210	0.861	0.652	0.186	0.473	161.210	0.594	0.754	0.431	0.653
LOC101928858	17.967	0.111	0.849	0.896	0.933	17.967	0.732	0.831	0.378	0.626	17.967	0.621	0.968	0.521	0.724
LOC101928909	25.563	-0.095	0.348	0.785	0.858	25.563	0.037	0.340	0.913	0.957	25.563	0.132	0.402	0.742	0.867
LINC01515	69.932	-0.100	0.397	0.801	0.869	69.932	0.491	0.392	0.210	0.491	69.932	0.592	0.456	0.194	0.417
HSFX3	11.880	-0.308	0.521	0.554	0.675	11.880	-0.695	0.532	0.191	0.477	11.880	-0.387	0.627	0.537	0.735
LOC101928978	10.230	-1.172	0.568	0.039	0.096	10.230	-0.571	0.524	0.276	0.547	10.230	0.601	0.657	0.360	0.593
LINC01719	23.944	0.932	0.585	0.111	0.211	23.944	0.857	0.583	0.142	0.433	23.944	-0.074	0.668	0.912	0.959
LOC101929054	27.517	-0.591	0.482	0.220	0.351	27.517	0.960	0.450	0.033	0.322	27.517	1.551	0.542	0.004	0.037
LOC101929057	15.334	-2.212	0.594	0.000	0.002	15.334	-1.014	0.519	0.051	0.338	15.334	1.198	0.680	0.078	0.241
LOC101929066	13.582	0.401	0.462	0.385	0.525	13.582	0.158	0.465	0.733	0.857	13.582	-0.243	0.535	0.650	0.811
PIK3CD-AS2	9.349	0.238	0.900	0.791	0.862	9.349	0.359	0.891	0.687	0.831	9.349	0.121	1.033	0.907	0.957
LOC101929089	63.903	-0.894	0.286	0.002	0.009	63.903	-0.003	0.268	0.991	0.995	63.903	0.891	0.326	0.006	0.048
C1QTNF7-AS1	16.472	-1.654	0.535	0.002	0.010	16.472	-0.211	0.434	0.627	0.795	16.472	1.443	0.597	0.016	0.088
LOC101929140	23.174	-0.498	0.529	0.347	0.488	23.174	0.600	0.504	0.234	0.511	23.174	1.097	0.600	0.068	0.220
PPP3CB-AS1	215.668	-0.280	0.257	0.276	0.414	215.668	0.313	0.254	0.218	0.497	215.668	0.593	0.296	0.045	0.171

WARS2-AS1	277.368	-0.829	0.182	0.000	0.000	277.368	0.245	0.175	0.163	0.454	277.368	1.074	0.208	0.000	0.000
P3H2-AS1	8.149	0.770	0.589	0.191	0.316	8.149	0.531	0.591	0.369	0.619	8.149	-0.239	0.671	0.722	0.856
LOC101929162	61.000	-0.767	0.262	0.003	0.015	61.000	-0.324	0.249	0.194	0.478	61.000	0.444	0.303	0.143	0.349
DDX59-AS1	7.441	-0.875	0.727	0.229	0.360	7.441	0.181	0.655	0.782	0.885	7.441	1.056	0.819	0.197	0.420
MMADHC-DT	75.070	-0.805	0.266	0.003	0.012	75.070	-0.189	0.254	0.456	0.683	75.070	0.616	0.306	0.044	0.169
LOC101929243	222.150	-0.954	0.364	0.009	0.031	222.150	0.073	0.360	0.840	0.917	222.150	1.026	0.419	0.014	0.083
KCNMA1-AS1	115.896	-2.036	0.550	0.000	0.002	115.896	0.272	0.533	0.610	0.785	115.896	2.307	0.630	0.000	0.005
ADAMTS9-AS1	69.601	-0.320	0.533	0.549	0.672	69.601	-0.974	0.540	0.072	0.361	69.601	-0.654	0.626	0.296	0.531
LINC01876	13.028	-0.458	0.612	0.454	0.589	13.028	0.360	0.581	0.535	0.734	13.028	0.818	0.695	0.239	0.470
ERLNC1	12.066	-1.857	0.786	0.018	0.053	12.066	0.528	0.683	0.440	0.672	12.066	2.385	0.876	0.006	0.049
LINC02428	24.859	-0.875	0.342	0.011	0.036	24.859	-0.217	0.313	0.489	0.705	24.859	0.658	0.392	0.093	0.268
LURAP1L-AS1	7.332	-1.120	0.843	0.184	0.308	7.332	0.960	0.718	0.181	0.469	7.332	2.081	0.926	0.025	0.116
CXXC4-AS1	6.482	-1.552	1.013	0.126	0.232	6.482	0.271	0.913	0.767	0.877	6.482	1.823	1.141	0.110	0.296
LOC101929479	254.700	-1.065	0.340	0.002	0.009	254.700	0.174	0.335	0.603	0.780	254.700	1.239	0.391	0.002	0.018
LINC01615	103.961	-0.305	0.362	0.400	0.539	103.961	0.340	0.357	0.341	0.599	103.961	0.644	0.416	0.122	0.313
LINC01970	8.767	0.532	0.636	0.404	0.542	8.767	0.189	0.642	0.769	0.877	8.767	-0.343	0.736	0.641	0.805
LOC101929512	15.301	-1.037	0.465	0.026	0.070	15.301	0.229	0.405	0.572	0.757	15.301	1.266	0.520	0.015	0.085
PABPC4-AS1	1269.821	-0.489	0.197	0.013	0.041	1269.821	0.216	0.196	0.271	0.542	1269.821	0.705	0.227	0.002	0.021
LOC101929523	36.329	0.461	0.324	0.155	0.271	36.329	0.088	0.328	0.789	0.890	36.329	-0.373	0.377	0.321	0.556
LOC101929524	141.472	-1.054	0.320	0.001	0.006	141.472	-0.238	0.313	0.447	0.677	141.472	0.816	0.368	0.027	0.122
LOC101929579	41.688	-1.768	0.350	0.000	0.000	41.688	-0.013	0.302	0.967	0.982	41.688	1.756	0.392	0.000	0.000
KIZ-AS1	160.838	-0.307	0.497	0.537	0.662	160.838	0.650	0.493	0.188	0.474	160.838	0.956	0.572	0.095	0.271
LOC101929595	114.485	-0.815	0.429	0.058	0.129	114.485	-0.217	0.425	0.609	0.785	114.485	0.598	0.496	0.228	0.456
B4GALT1-AS1	290.206	-0.940	0.463	0.042	0.102	290.206	0.253	0.461	0.583	0.766	290.206	1.193	0.534	0.026	0.119
TMC3-AS1	8.398	0.026	0.634	0.967	0.980	8.398	0.233	0.618	0.706	0.842	8.398	0.207	0.726	0.776	0.886
LINC02109	9.322	0.079	0.686	0.908	0.941	9.322	0.642	0.662	0.332	0.592	9.322	0.563	0.778	0.469	0.684
HORMAD2-AS1	22.910	-1.246	0.453	0.006	0.023	22.910	-0.066	0.405	0.871	0.935	22.910	1.180	0.512	0.021	0.105
SCN1A-AS1	9.256	-0.245	0.901	0.786	0.858	9.256	0.045	0.889	0.959	0.979	9.256	0.290	1.039	0.780	0.889
LOC101929709	46.397	-0.331	0.302	0.273	0.410	46.397	0.101	0.290	0.727	0.854	46.397	0.432	0.346	0.212	0.437
ODC1-DT	14.516	-1.348	0.442	0.002	0.011	14.516	-0.188	0.363	0.605	0.781	14.516	1.160	0.495	0.019	0.099
LOC101929718	127.022	0.295	0.255	0.248	0.382	127.022	-0.077	0.257	0.765	0.875	127.022	-0.372	0.296	0.210	0.435
LOC101929767	123.216	0.041	0.301	0.891	0.930	123.216	0.496	0.297	0.095	0.389	123.216	0.455	0.345	0.187	0.407
OSMR-AS1	18.059	0.828	0.501	0.098	0.193	18.059	0.922	0.494	0.062	0.350	18.059	0.094	0.563	0.867	0.936
NBPF25P	156.548	0.596	0.195	0.002	0.011	156.548	0.176	0.197	0.371	0.620	156.548	-0.420	0.226	0.063	0.211
NOTCH2NLR	7.633	-0.550	0.711	0.439	0.575	7.633	-0.795	0.715	0.266	0.538	7.633	-0.245	0.845	0.772	0.884
LOC101929829	19.452	1.449	0.565	0.010	0.035	19.452	0.195	0.585	0.739	0.860	19.452	-1.254	0.658	0.057	0.198
ITGA6-AS1	220.561	-1.322	0.511	NA	NA	220.561	-0.332	0.508	NA	NA	220.561	0.989	0.590	NA	NA
RNF41	1398.890	-0.853	0.202	0.000	0.000	1398.890	-0.418	0.201	0.037	0.326	1398.890	0.435	0.233	0.062	0.209
LOC101930071	10.006	0.490	0.617	0.427	0.564	10.006	0.009	0.628	0.988	0.993	10.006	-0.480	0.719	0.504	0.711
LOC101930085	192.122	-0.338	0.175	0.053	0.121	192.122	-0.220	0.172	0.201	0.484	192.122	0.117	0.202	0.562	0.753
FAM239A	190.064	-0.591	0.180	0.001	0.006	190.064	-0.116	0.174	0.506	0.716	190.064	0.475	0.207	0.022	0.107
LOC101930370	40.793	-0.596	0.295	0.043	0.103	40.793	-0.077	0.279	0.783	0.886	40.793	0.519	0.338	0.125	0.318
TSHZ1	424.698	-0.408	0.227	0.072	0.152	424.698	0.091	0.225	0.687	0.831	424.698	0.499	0.261	0.056	0.198
ALG3	1274.541	0.117	0.253	0.643	0.749	1274.541	-0.338	0.253	0.182	0.469	1274.541	-0.455	0.292	0.120	0.311
PRMT3	985.793	-0.990	0.247	0.000	0.001	985.793	-0.529	0.247	0.032	0.322	985.793	0.461	0.286	0.106	0.291
PSME3	2868.561	-0.665	0.369	0.071	0.152	2868.561	-0.933	0.369	0.011	0.305	2868.561	-0.268	0.426	0.530	0.729
MPHOSPH9	484.217	-0.797	0.205	0.000	0.001	484.217	0.102	0.203	0.615	0.788	484.217	0.899	0.237	0.000	0.003
MPHOSPH10	656.300	0.026	0.243	0.914	0.946	656.300	-0.332	0.243	0.172	0.461	656.300	-0.358	0.281	0.202	0.425
ADAM10	1461.945	-0.455	0.191	0.017	0.052	1461.945	-0.371	0.191	0.052	0.341	1461.945	0.084	0.221	0.703	0.845
CDK5	188.551	0.603	0.283	0.033	0.084	188.551	0.000	0.285	1.000	1.000	188.551	-0.603	0.328	0.066	0.216
MPHOSPH6	228.580	-0.252	0.340	0.459	0.594	228.580	-0.832	0.342	0.015	0.306	228.580	-0.580	0.395	0.142	0.348
NME6	311.867	-0.409	0.191	0.032	0.084	311.867	-0.348	0.190	0.067	0.356	311.867	0.061	0.222	0.783	0.891
DHRS2	13.131	2.133	0.474	0.000	0.000	13.131	0.319	0.519	0.539	0.736	13.131	-1.814	0.557	0.001	0.014
CALCRL	9.853	-1.789	0.612	0.003	0.015	9.853	-0.800	0.531	0.132	0.424	9.853	0.988	0.703	0.160	0.372
NUTF2	2351.947	0.429	0.225	0.056	0.126	2351.947	-0.243	0.225	0.280	0.549	2351.947	-0.673	0.260	0.010	0.064
MPZL2	56.892	0.815	0.334	0.015	0.045	56.892	0.668	0.334	0.045	0.334	56.892	-0.147	0.382	0.700	0.843
TRIM13	304.775	-0.098	0.196	0.616	0.727	304.775	0.118	0.195	0.546	0.740	304.775	0.216	0.227	0.340	0.575
PATJ	84.571	0.773	0.262	0.003	0.014	84.571	0.263	0.265	0.320	0.581	84.571	-0.509	0.303	0.092	0.267
USPL1	204.689	-0.504	0.218	0.021	0.059	204.689	-0.010	0.214	0.964	0.981	204.689	0.494	0.251	0.049	0.180
EIF1	14502.259	-0.250	0.172	0.145	0.258	14502.259	0.307	0.172	0.074	0.364	14502.259	0.558	0.199	0.005	0.041
CDK6	866.714	-1.160	0.322	0.000	0.002	866.714	-0.511	0.321	0.111	0.405	866.714	0.648	0.371	0.081	0.246
TOPORS	471.209	-0.140	0.159	0.379	0.519	471.209	-0.222	0.159	0.162	0.453	471.209	-0.082	0.185	0.656	0.816
FLOT1	14051.994	0.878	0.222	0.000	0.001	14051.994	0.365	0.222	0.100	0.394	14051.994	-0.513	0.256	0.045	0.171
DDX39A	1315.991	-0.067	0.421	0.874	0.919	1315.991	-1.087	0.421	0.010	0.298	1315.991	-1.020	0.487	0.036	0.148
PSMD14	1908.017	-0.461	0.295	0.118	0.222	1908.017	-0.716	0.295	0.015	0.306	1908.017	-0.255	0.341	0.454	0.673
AK6	1246.494	0.271	0.304	0.374	0.514	1246.494	-0.437	0.305	0.152	0.444	1246.494	-0.707	0.352	0.044	0.170
PRG4	112.417	1.137	0.369	0.002	0.010	112.417	-0.081	0.376	0.830	0.913	112.417	-1.218	0.429	0.005	0.038

CTDSPL	668.000	0.051	0.304	0.867	0.914	668.000	0.251	0.304	0.409	0.649	668.000	0.200	0.351	0.569	0.758
KLRG1	12.109	-1.205	0.520	0.020	0.059	12.109	-0.535	0.470	0.255	0.529	12.109	0.670	0.600	0.264	0.498
FBXW7-AS1	12.998	-1.067	0.508	0.036	0.090	12.998	0.067	0.447	0.880	0.940	12.998	1.134	0.571	0.047	0.176
CDK7	2616.508	0.379	0.159	0.017	0.051	2616.508	0.010	0.159	0.950	0.975	2616.508	-0.369	0.183	0.044	0.170
GDF11	362.469	0.226	0.125	0.070	0.149	362.469	-0.247	0.127	0.051	0.338	362.469	-0.474	0.146	0.001	0.015
TRIB1	560.550	0.719	0.173	0.000	0.000	560.550	0.096	0.174	0.580	0.764	560.550	-0.623	0.200	0.002	0.020
ZNF443	21.692	0.930	0.310	0.003	0.012	21.692	0.298	0.323	0.358	0.611	21.692	-0.632	0.360	0.079	0.242
PLIN3	3495.386	0.121	0.113	0.286	0.424	3495.386	0.027	0.113	0.810	0.900	3495.386	-0.094	0.131	0.474	0.688
MFSD10	1866.350	0.686	0.177	0.000	0.001	1866.350	0.236	0.178	0.183	0.470	1866.350	-0.450	0.205	0.028	0.126
STX6	1210.777	-0.302	0.116	0.009	0.032	1210.777	-0.035	0.115	0.760	0.872	1210.777	0.267	0.134	0.046	0.172
C11orf98	80.845	-0.164	0.330	0.620	0.730	80.845	-0.543	0.332	0.102	0.394	80.845	-0.379	0.385	0.325	0.559
COQ7	243.463	-0.348	0.161	0.031	0.080	243.463	-0.409	0.160	0.011	0.299	243.463	-0.062	0.188	0.743	0.868
NBR2	102.292	-0.063	0.287	0.825	0.885	102.292	0.398	0.282	0.158	0.451	102.292	0.462	0.329	0.161	0.375
LRRC23	188.890	0.672	0.279	0.016	0.049	188.890	0.514	0.279	0.065	0.354	188.890	-0.158	0.321	0.623	0.794
LRRC17	25.405	-1.922	0.531	0.000	0.002	25.405	-0.637	0.480	0.185	0.472	25.405	1.285	0.606	0.034	0.143
HNRNPR	3612.610	-1.017	0.238	0.000	0.000	3612.610	-0.628	0.238	0.008	0.297	3612.610	0.389	0.275	0.157	0.369
SLC35B1	438.018	-0.199	0.376	0.597	0.711	438.018	-0.937	0.377	0.013	0.305	438.018	-0.738	0.436	0.090	0.263
DCAF7	2763.035	-0.005	0.154	0.975	0.985	2763.035	0.056	0.154	0.715	0.847	2763.035	0.061	0.178	0.732	0.861
AP3S2	308.167	-0.013	0.161	0.937	0.960	308.167	-0.116	0.160	0.468	0.691	308.167	-0.104	0.186	0.578	0.763
CDK8	422.480	-0.820	0.206	0.000	0.001	422.480	-0.351	0.204	0.085	0.378	422.480	0.469	0.238	0.049	0.181
MRPS31	268.237	0.405	0.255	0.112	0.213	268.237	-0.256	0.257	0.319	0.581	268.237	-0.661	0.296	0.025	0.118
CALCOCO2	2061.570	-0.278	0.216	0.198	0.324	2061.570	0.342	0.215	0.112	0.405	2061.570	0.620	0.249	0.013	0.076
GPHN	437.711	-0.267	0.297	0.368	0.509	437.711	0.489	0.295	0.097	0.392	437.711	0.756	0.342	0.027	0.123
RABEPK	542.737	-0.504	0.243	0.038	0.094	542.737	-0.268	0.242	0.268	0.540	542.737	0.236	0.281	0.400	0.627
TIMM17B	793.025	0.381	0.245	0.119	0.223	793.025	-0.109	0.245	0.656	0.813	793.025	-0.491	0.283	0.083	0.250
MIR6126	69.601	-0.163	0.322	0.612	0.724	69.601	-0.116	0.319	0.716	0.848	69.601	0.047	0.373	0.900	0.953
MIR6747	3.569	-1.742	1.646	0.290	0.428	3.569	-0.232	1.543	0.881	0.940	3.569	1.510	1.878	0.421	0.646
MIR1199	9.485	1.646	0.833	0.048	0.112	9.485	-0.745	0.987	0.451	0.679	9.485	-2.391	1.064	0.025	0.116
MIR6720	91.384	-0.618	0.275	0.024	0.067	91.384	0.125	0.265	0.636	0.801	91.384	0.744	0.315	0.018	0.095
MIR6859-1	28.055	2.070	0.640	0.001	0.007	28.055	0.378	0.657	0.565	0.753	28.055	-1.692	0.742	0.022	0.109
RIDA	417.812	0.815	0.191	0.000	0.000	417.812	0.221	0.192	0.249	0.523	417.812	-0.594	0.220	0.007	0.052
LINC02056	10.158	0.546	0.583	0.349	0.489	10.158	0.395	0.582	0.497	0.711	10.158	-0.151	0.667	0.820	0.911
POP7	396.859	0.516	0.293	0.079	0.163	396.859	-0.238	0.295	0.420	0.657	396.859	-0.754	0.340	0.027	0.122
CDK9	1156.607	0.279	0.146	0.057	0.127	1156.607	0.001	0.147	0.994	0.997	1156.607	-0.278	0.169	0.101	0.282
SRRM1	1247.185	0.035	0.195	0.858	0.907	1247.185	-0.258	0.195	0.185	0.472	1247.185	-0.293	0.225	0.193	0.415
SPRY3	240.921	-0.176	0.279	0.528	0.654	240.921	0.630	0.276	0.022	0.315	240.921	0.807	0.320	0.012	0.073
SPRY1	99.177	1.050	0.460	0.022	0.063	99.177	0.746	0.461	0.105	0.398	99.177	-0.304	0.528	0.565	0.755
SPRY2	334.016	-1.252	0.197	0.000	0.000	334.016	-0.133	0.190	0.484	0.702	334.016	1.119	0.226	0.000	0.000
STAM2	360.789	-0.630	0.159	0.000	0.001	360.789	-0.264	0.157	0.092	0.387	360.789	0.366	0.184	0.047	0.175
LOC102546294	36.321	0.520	0.361	0.149	0.263	36.321	0.357	0.361	0.322	0.583	36.321	-0.163	0.415	0.694	0.840
NDST1-AS1	14.172	-1.097	0.487	0.024	0.067	14.172	0.347	0.416	0.404	0.646	14.172	1.444	0.541	0.008	0.054
CNKSR1	23.251	0.980	0.429	0.022	0.063	23.251	0.205	0.443	0.643	0.805	23.251	-0.774	0.499	0.121	0.312
ABCC4	492.673	-0.720	0.217	0.001	0.005	492.673	-0.209	0.216	0.333	0.592	492.673	0.511	0.251	0.041	0.163
CDKN1A	3348.694	-0.081	0.146	0.579	0.697	3348.694	-0.040	0.146	0.787	0.889	3348.694	0.042	0.169	0.805	0.903
DENND4A	956.556	-1.165	0.241	0.000	0.000	956.556	-0.116	0.240	0.629	0.796	956.556	1.049	0.278	0.000	0.003
LOC102606465	65.920	-0.279	0.260	0.283	0.420	65.920	0.268	0.248	0.280	0.550	65.920	0.547	0.296	0.064	0.214
SF3B4	920.602	0.111	0.296	0.707	0.799	920.602	-0.518	0.296	0.080	0.371	920.602	-0.629	0.342	0.066	0.216
CDK2AP2	627.352	1.520	0.263	0.000	0.000	627.352	0.147	0.265	0.578	0.762	627.352	-1.372	0.304	0.000	0.000
IRX5	62.087	0.090	0.330	0.786	0.858	62.087	0.185	0.326	0.570	0.756	62.087	0.096	0.380	0.801	0.901
THRIL	203.008	-0.695	0.377	0.065	0.141	203.008	-0.275	0.375	0.463	0.689	203.008	0.420	0.435	0.335	0.569
RAMP1	308.603	0.470	0.294	0.110	0.210	308.603	0.571	0.293	0.051	0.339	308.603	0.101	0.338	0.765	0.881
ZMPSTE24	861.332	-0.700	0.374	0.061	0.134	861.332	-1.171	0.374	0.002	0.297	861.332	-0.471	0.432	0.276	0.511
CDKN1B	1604.827	-0.598	0.315	0.058	0.129	1604.827	0.401	0.315	0.203	0.486	1604.827	0.999	0.364	0.006	0.047
AKAP8	643.491	-0.358	0.203	0.078	0.162	643.491	-0.202	0.203	0.320	0.581	643.491	0.157	0.235	0.505	0.712
FSTL3	4445.904	0.080	0.167	0.632	0.741	4445.904	0.224	0.167	0.181	0.469	4445.904	0.144	0.193	0.457	0.675
FRG1DP	18.932	-1.042	0.415	0.012	0.039	18.932	0.163	0.361	0.652	0.810	18.932	1.205	0.464	0.009	0.063
LINC02298	37.633	0.762	0.585	0.193	0.318	37.633	0.734	0.584	0.209	0.490	37.633	-0.027	0.672	0.968	0.985
ZFPM2-AS1	120.851	0.629	0.215	0.004	0.015	120.851	0.057	0.220	0.795	0.893	120.851	-0.572	0.251	0.023	0.109
LOC102723360	370.602	0.535	0.277	0.054	0.122	370.602	0.398	0.277	0.152	0.444	370.602	-0.137	0.320	0.668	0.824
LOC102723385	26.706	-0.157	0.359	0.662	0.764	26.706	-0.520	0.364	0.153	0.445	26.706	-0.363	0.425	0.393	0.621
FRG1EP	49.658	-0.539	0.403	0.181	0.304	49.658	0.473	0.388	0.222	0.501	49.658	1.012	0.459	0.027	0.124
ELF3-AS1	626.726	-0.001	0.107	0.995	0.997	626.726	-0.007	0.106	0.951	0.976	626.726	-0.006	0.124	0.962	0.982
KCNE1B	8.619	4.177	0.840	0.000	0.000	8.619	2.723	0.869	0.002	0.297	8.619	-1.454	0.830	0.080	0.244
KANTR	88.016	1.054	0.368	0.004	0.017	88.016	0.363	0.372	0.328	0.588	88.016	-0.691	0.425	0.104	0.288
SMIM11B	687.776	0.259	0.219	0.237	0.371	687.776	-0.092	0.219	0.674	0.824	687.776	-0.351	0.253	0.166	0.381
LOC102723566	236.923	0.213	0.352	0.546	0.670	236.923	-0.417	0.353	0.238	0.515	236.923	-0.630	0.408	0.123	0.315
LOC102723582	530.688	-0.310	0.239	0.195	0.321	530.688	-0.485	0.239	0.042	0.330	530.688	-0.176	0.277	0.526	0.727

LOC102723672	20.414	-0.515	0.389	0.186	0.310	20.414	-0.126	0.370	0.733	0.857	20.414	0.388	0.448	0.386	0.615
LOC102723701	15.751	-1.136	0.501	0.023	0.065	15.751	-0.303	0.455	0.506	0.716	15.751	0.833	0.573	0.146	0.353
LOC102723729	707.445	0.261	0.103	0.012	0.039	707.445	-0.102	0.104	0.326	0.586	707.445	-0.363	0.120	0.003	0.026
LINC01315	175.910	1.088	0.796	0.172	0.292	175.910	0.852	0.796	0.285	0.554	175.910	-0.236	0.918	0.797	0.899
LOC102723809	14.614	1.656	0.665	0.013	0.041	14.614	1.589	0.661	0.016	0.306	14.614	-0.067	0.719	0.926	0.966
EML4-AS1	47.444	-1.394	0.538	0.010	0.033	47.444	-0.853	0.528	0.106	0.398	47.444	0.540	0.623	0.386	0.615
TBC1D3E	211.196	1.949	0.589	0.001	0.005	211.196	1.232	0.590	0.037	0.324	211.196	-0.717	0.678	0.290	0.526
LOC102723885	301.012	-0.338	0.204	0.097	0.192	301.012	0.030	0.202	0.883	0.941	301.012	0.368	0.235	0.118	0.307
LOC102723996	58.450	-0.370	0.269	0.169	0.289	58.450	-0.037	0.258	0.886	0.943	58.450	0.333	0.310	0.282	0.517
KIF1C-AS1	179.670	-0.495	0.280	0.077	0.161	179.670	-0.290	0.278	0.296	0.563	179.670	0.204	0.324	0.528	0.728
LOC102724050	514.543	-0.446	0.375	0.235	0.367	514.543	0.328	0.373	0.379	0.626	514.543	0.774	0.432	0.073	0.232
LOC102724064	96.989	0.932	0.189	0.000	0.000	96.989	0.188	0.195	0.336	0.594	96.989	-0.744	0.220	0.001	0.011
LOC102724153	149.348	-1.094	0.448	0.015	0.045	149.348	0.370	0.439	0.399	0.643	149.348	1.464	0.514	0.004	0.038
LOC102724159	876.602	-0.653	0.287	0.023	0.064	876.602	-0.617	0.287	0.031	0.322	876.602	0.036	0.332	0.915	0.960
LOC102724163	791.587	0.798	0.386	0.039	0.095	791.587	0.632	0.386	0.101	0.394	791.587	-0.166	0.445	0.709	0.849
LINC02289	72.227	-1.292	0.430	0.003	0.012	72.227	0.846	0.405	0.037	0.324	72.227	2.138	0.488	0.000	0.000
LOC102724238	34.125	-0.635	0.416	0.127	0.233	34.125	0.255	0.394	0.518	0.723	34.125	0.890	0.473	0.060	0.205
SVIL-AS1	462.617	-0.528	0.179	0.003	0.014	462.617	-0.334	0.178	0.060	0.350	462.617	0.194	0.207	0.349	0.582
SPINT1-AS1	11.671	0.902	0.417	0.031	0.080	11.671	0.006	0.449	0.989	0.994	11.671	-0.896	0.497	0.071	0.228
SIK1B	1573.786	-0.042	0.279	0.879	0.921	1573.786	0.484	0.279	0.082	0.374	1573.786	0.527	0.322	0.102	0.284
LOC102724488	19.327	0.787	0.635	0.215	0.345	19.327	0.975	0.629	0.121	0.413	19.327	0.188	0.723	0.795	0.897
LINC01291	57.277	-0.115	0.231	0.618	0.728	57.277	0.016	0.225	0.945	0.972	57.277	0.131	0.266	0.623	0.794
LOC102724532	15.418	0.056	0.524	0.915	0.946	15.418	0.531	0.505	0.294	0.561	15.418	0.475	0.595	0.424	0.648
LINC02015	659.423	1.127	0.424	0.008	0.028	659.423	-0.916	0.428	0.033	0.322	659.423	-2.043	0.493	0.000	0.001
CBSL	87.283	-1.777	0.304	0.000	0.000	87.283	-0.089	0.278	0.748	0.866	87.283	1.688	0.345	0.000	0.000
LNCTAM34A	27.366	0.611	0.444	0.168	0.288	27.366	0.809	0.437	0.064	0.353	27.366	0.197	0.503	0.695	0.841
U2AF1L5	134.647	0.610	0.317	0.054	0.122	134.647	-0.209	0.320	0.513	0.720	134.647	-0.820	0.368	0.026	0.120
DPY19L1P2	7.461	-1.611	0.730	0.027	0.073	7.461	-0.172	0.595	0.772	0.879	7.461	1.438	0.815	0.078	0.240
LINC02043	11.268	1.306	0.562	0.020	0.058	11.268	1.121	0.563	0.046	0.335	11.268	-0.184	0.630	0.770	0.883
LOC102724770	265.431	1.272	0.418	0.002	0.011	265.431	0.747	0.418	0.074	0.364	265.431	-0.525	0.482	0.275	0.510
LOC102724776	18.094	0.161	0.445	0.718	0.808	18.094	-0.552	0.462	0.232	0.509	18.094	-0.713	0.531	0.180	0.398
LOC102724788	123.538	0.889	0.879	0.312	0.452	123.538	0.624	0.879	0.478	0.698	123.538	-0.265	1.014	0.794	0.896
LOC102724814	163.262	0.542	0.301	0.072	0.152	163.262	0.484	0.300	0.107	0.399	163.262	-0.057	0.347	0.869	0.937
ZNF337-AS1	377.876	0.353	0.216	0.103	0.200	377.876	0.344	0.216	0.111	0.405	377.876	-0.008	0.250	0.973	0.987
MIATNB	20.708	-0.208	0.449	0.644	0.750	20.708	0.524	0.421	0.213	0.493	20.708	0.731	0.506	0.148	0.356
LOC102724843	138.509	-1.032	0.434	0.017	0.052	138.509	-0.408	0.430	0.343	0.600	138.509	0.625	0.501	0.213	0.438
LINC02861	17.632	0.961	0.582	0.099	0.194	17.632	0.386	0.591	0.513	0.720	17.632	-0.575	0.671	0.392	0.620
LOC102724951	8.891	-0.611	0.561	0.276	0.414	8.891	-0.336	0.537	0.531	0.732	8.891	0.275	0.652	0.674	0.827
LOC102725021	17.532	0.659	0.512	0.198	0.324	17.532	-0.080	0.525	0.879	0.939	17.532	-0.739	0.599	0.217	0.443
LINC02352	23.718	0.461	0.697	0.508	0.638	23.718	0.784	0.690	0.256	0.529	23.718	0.323	0.798	0.686	0.834
LINC02588	28.473	0.218	0.383	0.569	0.688	28.473	-0.156	0.388	0.688	0.831	28.473	-0.374	0.448	0.404	0.631
LOC102725072	14.663	1.579	0.404	0.000	0.001	14.663	0.141	0.457	0.758	0.871	14.663	-1.439	0.484	0.003	0.028
LINC01232	50.238	0.271	0.499	0.588	0.703	50.238	0.668	0.494	0.176	0.465	50.238	0.398	0.573	0.487	0.698
STUB1	2532.205	0.128	0.201	0.525	0.652	2532.205	-0.167	0.201	0.405	0.646	2532.205	-0.295	0.232	0.203	0.427
STAG1	802.197	-0.896	0.182	0.000	0.000	802.197	-0.445	0.181	0.014	0.306	802.197	0.452	0.211	0.032	0.138
NET1	1648.018	-0.220	0.152	0.148	0.261	1648.018	0.009	0.152	0.954	0.978	1648.018	0.229	0.176	0.192	0.414
UBE4B	1323.518	-0.514	0.251	0.041	0.098	1323.518	0.311	0.250	0.214	0.493	1323.518	0.824	0.289	0.004	0.037
PRSS16	100.882	0.482	0.575	0.402	0.540	100.882	0.985	0.573	0.085	0.378	100.882	0.503	0.662	0.447	0.666
CDKN1C	331.931	-0.012	1.023	0.991	0.995	331.931	0.690	1.022	0.500	0.713	331.931	0.702	1.181	0.552	0.746
SIGMAR1	1975.143	0.089	0.261	0.735	0.821	1975.143	-0.480	0.261	0.066	0.354	1975.143	-0.568	0.302	0.060	0.204
TPTEP2-CSNK1E	11.803	1.676	0.709	0.018	0.053	11.803	1.492	0.710	0.036	0.324	11.803	-0.185	0.785	0.814	0.907
BET1	415.066	-0.501	0.198	0.011	0.038	415.066	-0.136	0.196	0.488	0.705	415.066	0.365	0.229	0.110	0.296
CWC27	1172.523	-0.334	0.126	0.008	0.028	1172.523	-0.070	0.125	0.574	0.759	1172.523	0.264	0.145	0.070	0.225
SAP18	2125.502	0.426	0.183	0.020	0.057	2125.502	0.084	0.183	0.648	0.808	2125.502	-0.342	0.211	0.105	0.289
SMNDC1	619.590	-0.172	0.211	0.416	0.553	619.590	-0.334	0.211	0.113	0.405	619.590	-0.163	0.244	0.505	0.712
BCAS2	774.423	-0.512	0.274	0.062	0.136	774.423	-0.615	0.274	0.025	0.316	774.423	-0.103	0.317	0.744	0.869
RGS19	102.710	0.731	0.287	0.011	0.036	102.710	0.091	0.292	0.754	0.868	102.710	-0.639	0.333	0.055	0.195
EIF1B	716.478	-0.678	0.210	0.001	0.007	716.478	-0.155	0.208	0.457	0.683	716.478	0.523	0.242	0.031	0.134
SPEG	186.098	2.065	0.409	0.000	0.000	186.098	0.683	0.412	0.097	0.392	186.098	-1.382	0.472	0.003	0.032
SF3A1	2534.096	-0.372	0.158	0.019	0.054	2534.096	-0.090	0.158	0.569	0.756	2534.096	0.283	0.183	0.122	0.313
TRAIP	126.445	-0.470	0.402	0.242	0.375	126.445	-0.267	0.400	0.505	0.716	126.445	0.204	0.465	0.661	0.819
DNAJA2	2037.329	-0.188	0.114	0.099	0.194	2037.329	-0.187	0.114	0.099	0.393	2037.329	0.000	0.131	0.998	0.999
BCKDK	991.788	0.086	0.141	0.544	0.668	991.788	0.261	0.141	0.063	0.350	991.788	0.175	0.163	0.281	0.515
MAEA	818.314	-0.398	0.193	0.039	0.096	818.314	-0.389	0.193	0.044	0.331	818.314	0.009	0.224	0.968	0.985
APC2	101.031	0.667	0.349	0.056	0.126	101.031	0.551	0.349	0.114	0.406	101.031	-0.116	0.402	0.773	0.885
PAK4	1015.171	0.153	0.121	0.207	0.336	1015.171	0.146	0.121	0.228	0.506	1015.171	-0.007	0.140	0.959	0.981
MARCHF6	3741.019	-0.448	0.226	0.048	0.112	3741.019	0.225	0.226	0.320	0.581	3741.019	0.673	0.261	0.010	0.066

ADAR	3268.850	-0.663	0.213	0.002	0.009	3268.850	-0.212	0.212	0.317	0.579	3268.850	0.451	0.245	0.066	0.217
KATNB1	914.499	-0.347	0.222	0.119	0.222	914.499	-0.178	0.222	0.422	0.659	914.499	0.169	0.257	0.511	0.716
DLEU1	191.297	-0.444	0.212	0.037	0.091	191.297	0.286	0.207	0.166	0.456	191.297	0.730	0.243	0.003	0.027
SNAPC5	760.839	-0.017	0.223	0.941	0.963	760.839	-0.170	0.223	0.446	0.677	760.839	-0.153	0.258	0.552	0.746
CASC21	52.225	0.806	0.371	0.030	0.078	52.225	0.274	0.375	0.465	0.689	52.225	-0.532	0.429	0.215	0.441
CASC19	283.642	0.395	0.264	0.134	0.243	283.642	0.507	0.263	0.054	0.342	283.642	0.112	0.304	0.712	0.850
ABALON	2316.640	-0.509	0.197	0.010	0.034	2316.640	0.026	0.197	0.896	0.948	2316.640	0.535	0.228	0.019	0.098
APBB3	213.533	0.933	0.348	0.007	0.027	213.533	0.549	0.349	0.116	0.408	213.533	-0.384	0.402	0.339	0.574
ZNF267	136.088	-0.320	0.237	0.178	0.300	136.088	-0.328	0.236	0.165	0.455	136.088	-0.007	0.276	0.979	0.989
CCNO	118.800	0.250	0.236	0.288	0.426	118.800	-0.001	0.236	0.998	0.999	118.800	-0.251	0.273	0.358	0.591
SNHG22	9.112	0.150	0.524	0.775	0.850	9.112	0.253	0.511	0.620	0.791	9.112	0.104	0.600	0.863	0.935
LINC01145	543.925	0.434	0.285	0.128	0.235	543.925	0.496	0.285	0.081	0.372	543.925	0.063	0.329	0.849	0.927
CDKN2C	958.985	0.134	0.378	0.723	0.812	958.985	0.504	0.377	0.182	0.469	958.985	0.370	0.436	0.396	0.624
VPS26C	1072.356	0.102	0.254	0.688	0.785	1072.356	0.175	0.253	0.489	0.705	1072.356	0.074	0.293	0.801	0.901
TCIRG1	631.802	0.862	0.208	0.000	0.000	631.802	0.410	0.209	0.050	0.337	631.802	-0.452	0.241	0.060	0.205
RTN3	3211.400	0.226	0.186	0.225	0.356	3211.400	0.239	0.186	0.198	0.482	3211.400	0.013	0.215	0.950	0.977
LANCL1	1128.033	-0.687	0.300	0.022	0.062	1128.033	0.080	0.300	0.790	0.890	1128.033	0.767	0.347	0.027	0.123
PCAT2	17.471	0.413	0.706	0.559	0.680	17.471	0.655	0.698	0.348	0.603	17.471	0.242	0.808	0.764	0.880
TNIP1	7646.163	-0.126	0.194	0.515	0.643	7646.163	-0.319	0.194	0.099	0.394	7646.163	-0.193	0.224	0.388	0.616
CDKN2D	203.849	1.105	0.339	0.001	0.006	203.849	0.351	0.341	0.304	0.570	203.849	-0.754	0.392	0.054	0.193
CRISP3	9.123	-0.438	0.609	0.471	0.606	9.123	-0.016	0.583	0.978	0.987	9.123	0.422	0.699	0.546	0.741
SMYD5	298.327	-0.435	0.205	0.034	0.086	298.327	-0.368	0.204	0.070	0.359	298.327	0.067	0.237	0.778	0.888
RRAGB	187.939	0.245	0.280	0.382	0.522	187.939	0.180	0.279	0.519	0.724	187.939	-0.065	0.323	0.841	0.923
SIRPB1	67.176	0.509	0.456	0.264	0.400	67.176	-0.221	0.464	0.635	0.801	67.176	-0.730	0.533	0.171	0.387
AKR1A1	1491.832	0.341	0.167	0.041	0.100	1491.832	0.087	0.167	0.601	0.779	1491.832	-0.254	0.193	0.188	0.409
EMC8	382.763	-0.258	0.351	0.463	0.597	382.763	-0.698	0.352	0.047	0.336	382.763	-0.440	0.407	0.280	0.514
RXYLT1	356.641	-0.595	0.240	0.013	0.042	356.641	-0.193	0.238	0.418	0.656	356.641	0.403	0.277	0.146	0.353
CDKN3	754.441	-0.079	0.240	0.740	0.825	754.441	-0.122	0.240	0.611	0.785	754.441	-0.043	0.277	0.878	0.942
CNPY2	1313.466	0.338	0.220	0.124	0.230	1313.466	-0.242	0.221	0.272	0.544	1313.466	-0.580	0.255	0.023	0.109
B3GNT3	12.506	0.654	0.466	0.160	0.278	12.506	-0.055	0.488	0.910	0.955	12.506	-0.710	0.551	0.197	0.420
TLR6	7.081	0.355	0.554	0.522	0.649	7.081	-1.731	0.723	0.017	0.306	7.081	-2.086	0.790	0.008	0.058
LINCR-0002	643.446	0.089	0.384	0.817	0.880	643.446	0.444	0.383	0.247	0.521	643.446	0.355	0.443	0.423	0.647
KIAA1614-AS1	12.563	-0.108	0.441	0.807	0.873	12.563	-0.673	0.459	0.142	0.434	12.563	-0.565	0.534	0.290	0.525
LOC103344931	58.453	-0.165	0.349	0.635	0.743	58.453	0.059	0.344	0.864	0.931	58.453	0.225	0.402	0.577	0.762
MRVI1	7.058	0.571	1.079	0.597	0.711	7.058	1.397	1.056	0.186	0.473	7.058	0.826	1.222	0.499	0.706
LINC01410	52.738	-0.288	0.241	0.232	0.365	52.738	-0.522	0.242	0.031	0.322	52.738	-0.234	0.285	0.412	0.638
PCGF3	697.430	0.050	0.169	0.769	0.847	697.430	0.069	0.169	0.685	0.830	697.430	0.019	0.196	0.924	0.965
TFG	2004.987	0.024	0.133	0.859	0.908	2004.987	-0.115	0.133	0.387	0.633	2004.987	-0.139	0.154	0.367	0.599
PKDREJ	7.286	-1.148	0.856	0.180	0.303	7.286	0.438	0.774	0.571	0.757	7.286	1.587	0.962	0.099	0.279
TRIM22	220.372	1.007	0.328	0.002	0.010	220.372	0.347	0.329	0.291	0.559	220.372	-0.660	0.378	0.081	0.247
ABCA7	448.354	1.122	0.353	0.001	0.008	448.354	0.667	0.354	0.059	0.349	448.354	-0.455	0.408	0.264	0.499
ABCA10	80.949	-1.596	0.329	0.000	0.000	80.949	0.295	0.285	0.301	0.567	80.949	1.891	0.367	0.000	0.000
ABCA9	24.287	0.604	0.652	0.354	0.495	24.287	1.345	0.632	0.033	0.322	24.287	0.741	0.731	0.311	0.546
ABCA8	317.226	-1.011	0.742	0.173	0.294	317.226	0.700	0.740	0.344	0.600	317.226	1.711	0.856	0.046	0.172
WARS2	439.025	-0.622	0.168	0.000	0.002	439.025	0.227	0.165	0.168	0.457	439.025	0.849	0.193	0.000	0.000
NPM3	798.564	-0.342	0.320	0.286	0.424	798.564	-0.500	0.320	0.118	0.410	798.564	-0.158	0.370	0.669	0.824
LOC103611081	32.789	0.759	0.278	0.006	0.024	32.789	-0.355	0.300	0.236	0.514	32.789	-1.114	0.336	0.001	0.012
HMG20B	2930.026	0.437	0.208	0.036	0.090	2930.026	0.361	0.208	0.083	0.374	2930.026	-0.075	0.241	0.754	0.874
LLPH-DT	16.369	-1.126	0.558	0.043	0.104	16.369	0.239	0.507	0.637	0.802	16.369	1.365	0.629	0.030	0.132
HMG20A	829.857	-0.629	0.178	0.000	0.003	829.857	0.110	0.176	0.533	0.733	829.857	0.739	0.205	0.000	0.006
KLF2	62.176	0.624	0.468	0.183	0.307	62.176	-0.693	0.492	0.159	0.451	62.176	-1.317	0.559	0.019	0.097
MICU1	759.199	0.473	0.153	0.002	0.010	759.199	0.141	0.154	0.358	0.611	759.199	-0.331	0.177	0.062	0.209
TBX2-AS1	56.585	1.232	0.399	0.002	0.010	56.585	0.230	0.407	0.572	0.758	56.585	-1.002	0.462	0.030	0.132
FOXG1-AS1	32.916	-0.837	0.453	0.064	0.140	32.916	-0.994	0.452	0.028	0.321	32.916	-0.157	0.533	0.769	0.882
LINC01322	49.226	-0.343	0.306	0.262	0.397	49.226	-0.330	0.303	0.277	0.547	49.226	0.014	0.356	0.969	0.985
CITED2	5181.608	0.032	0.374	0.932	0.957	5181.608	0.480	0.374	0.199	0.482	5181.608	0.448	0.432	0.299	0.534
SEMA3A	13.350	0.524	0.523	0.317	0.457	13.350	0.524	0.517	0.311	0.575	13.350	0.000	0.596	1.000	1.000
LINC01287	247.544	-0.190	0.213	0.372	0.512	247.544	0.114	0.211	0.589	0.770	247.544	0.305	0.246	0.215	0.441
SPATA17-AS1	9.530	0.525	0.916	0.567	0.687	9.530	0.560	0.912	0.539	0.736	9.530	0.035	1.052	0.973	0.987
FOXC2-AS1	112.548	-1.249	0.370	0.001	0.005	112.548	-0.650	0.362	0.072	0.361	112.548	0.599	0.428	0.161	0.374
TMEM92-AS1	7.561	0.677	0.670	0.312	0.452	7.561	1.083	0.647	0.094	0.388	7.561	0.406	0.744	0.585	0.767
TUBA1B	17005.020	-0.010	0.238	0.965	0.979	17005.020	-0.394	0.238	0.098	0.392	17005.020	-0.384	0.275	0.163	0.377
IRF9	12.120	2.076	0.598	0.001	0.003	12.120	0.938	0.624	0.133	0.424	12.120	-1.138	0.676	0.092	0.267
CDR1	15.925	-0.749	0.632	0.236	0.369	15.925	-0.111	0.610	0.855	0.926	15.925	0.638	0.727	0.380	0.610
BPNT1	521.809	-0.087	0.124	0.484	0.617	521.809	-0.095	0.124	0.444	0.675	521.809	-0.008	0.144	0.958	0.980
TUBB3	1507.377	1.016	0.145	0.000	0.000	1507.377	0.226	0.146	0.122	0.413	1507.377	-0.790	0.168	0.000	0.000
TUBB4B	5790.729	0.599	0.221	0.007	0.025	5790.729	-0.287	0.221	0.193	0.478	5790.729	-0.886	0.255	0.001	0.008

BTN3A3	177.095	0.346	0.594	0.560	0.681	177.095	0.535	0.593	0.367	0.618	177.095	0.189	0.685	0.783	0.891
BTN2A2	191.345	0.348	0.248	0.160	0.279	191.345	0.454	0.247	0.066	0.354	191.345	0.106	0.285	0.711	0.850
SCML2	155.371	-0.698	0.260	0.007	0.027	155.371	-0.067	0.255	0.791	0.891	155.371	0.630	0.299	0.035	0.146
CDR2	492.800	-0.504	0.281	0.073	0.154	492.800	-0.499	0.280	0.075	0.364	492.800	0.005	0.325	0.987	0.993
CEPT1	989.149	-0.063	0.103	0.541	0.666	989.149	0.137	0.102	0.181	0.469	989.149	0.200	0.119	0.093	0.268
LOC103908605	15.579	-0.390	0.581	0.502	0.632	15.579	0.203	0.555	0.715	0.847	15.579	0.593	0.663	0.371	0.603
MYL12B	2891.965	0.425	0.195	0.029	0.077	2891.965	-0.303	0.196	0.121	0.413	2891.965	-0.728	0.226	0.001	0.016
NOD1	146.094	0.437	0.186	0.019	0.056	146.094	0.241	0.186	0.196	0.479	146.094	-0.195	0.215	0.363	0.596
ANAPC10	223.966	-0.651	0.211	0.002	0.010	223.966	-0.300	0.208	0.149	0.442	223.966	0.351	0.244	0.151	0.360
DLC1	379.260	-1.040	0.460	0.024	0.065	379.260	-1.673	0.461	0.000	0.211	379.260	-0.632	0.533	0.235	0.465
ATP8A1	65.434	-0.357	0.436	0.412	0.550	65.434	0.385	0.428	0.369	0.619	65.434	0.742	0.500	0.138	0.341
NDRG1	43933.575	0.521	0.931	0.576	0.694	43933.575	0.934	0.931	0.316	0.578	43933.575	0.413	1.075	0.701	0.844
MYL9	38.107	2.284	0.446	0.000	0.000	38.107	0.020	0.470	0.966	0.982	38.107	-2.264	0.524	0.000	0.001
RACK1	48708.332	0.455	0.255	0.075	0.157	48708.332	0.385	0.255	0.131	0.423	48708.332	-0.070	0.295	0.813	0.907
ADARB1	2736.238	-0.503	0.397	0.205	0.333	2736.238	0.463	0.397	0.243	0.519	2736.238	0.966	0.458	0.035	0.146
CDS1	99.366	0.162	0.180	0.369	0.509	99.366	0.040	0.180	0.822	0.907	99.366	-0.122	0.209	0.560	0.752
PEMT	201.140	0.013	0.224	0.953	0.972	201.140	0.087	0.223	0.697	0.837	201.140	0.074	0.258	0.776	0.886
PIAS3	529.327	0.436	0.137	0.002	0.008	529.327	0.082	0.138	0.551	0.744	529.327	-0.354	0.159	0.026	0.120
ST3GAL6	657.804	0.301	0.231	0.192	0.317	657.804	0.441	0.230	0.055	0.343	657.804	0.140	0.266	0.599	0.777
NDC80	429.626	-0.158	0.257	0.540	0.665	429.626	-0.152	0.257	0.555	0.746	429.626	0.006	0.297	0.984	0.991
CPQ	213.579	1.095	0.359	0.002	0.011	213.579	0.502	0.360	0.163	0.454	213.579	-0.594	0.414	0.152	0.361
BASP1	3890.105	0.561	0.179	0.002	0.009	3890.105	-0.149	0.180	0.408	0.649	3890.105	-0.710	0.207	0.001	0.009
CDSN	47.663	0.514	0.446	0.249	0.383	47.663	0.594	0.444	0.180	0.469	47.663	0.080	0.512	0.875	0.940
IFITM3	9359.725	1.788	0.301	0.000	0.000	9359.725	0.596	0.301	0.048	0.337	9359.725	-1.192	0.348	0.001	0.009
RAPGEF3	44.662	0.659	0.303	0.029	0.077	44.662	-0.255	0.315	0.418	0.656	44.662	-0.914	0.358	0.011	0.068
NSA2	3772.097	0.458	0.230	0.046	0.109	3772.097	0.149	0.230	0.517	0.722	3772.097	-0.309	0.265	0.244	0.475
YAP1	3260.922	-0.471	0.165	0.004	0.018	3260.922	-0.034	0.165	0.837	0.916	3260.922	0.437	0.191	0.022	0.108
RXYLT1-AS1	64.118	-0.147	0.260	0.571	0.690	64.118	-0.294	0.259	0.256	0.529	64.118	-0.147	0.303	0.628	0.797
SPON2	71.872	1.194	0.402	0.003	0.013	71.872	0.576	0.405	0.155	0.448	71.872	-0.618	0.463	0.182	0.401
PRMT5	2536.870	-1.186	0.259	0.000	0.000	2536.870	-0.294	0.259	0.255	0.528	2536.870	0.892	0.299	0.003	0.028
TESK2	44.868	0.925	0.477	0.053	0.120	44.868	0.677	0.478	0.156	0.448	44.868	-0.247	0.547	0.651	0.812
CD2BP2	996.142	0.018	0.164	0.914	0.946	996.142	-0.053	0.164	0.748	0.866	996.142	-0.070	0.189	0.710	0.849
UBAC1	1269.452	0.025	0.116	0.829	0.887	1269.452	-0.031	0.115	0.788	0.889	1269.452	-0.056	0.134	0.675	0.828
CDIPT	1667.818	0.450	0.138	0.001	0.006	1667.818	0.104	0.138	0.452	0.680	1667.818	-0.346	0.160	0.030	0.133
PGRMC2	1550.805	0.168	0.191	0.380	0.520	1550.805	0.191	0.191	0.316	0.578	1550.805	0.024	0.220	0.914	0.960
ARIH2	1478.307	-1.039	0.270	0.000	0.001	1478.307	-0.576	0.269	0.032	0.322	1478.307	0.463	0.311	0.137	0.339
TUBGCP3	712.916	-0.578	0.218	0.008	0.028	712.916	-0.469	0.217	0.031	0.322	712.916	0.109	0.252	0.665	0.822
SEC24B	809.619	-0.437	0.205	0.033	0.085	809.619	0.217	0.204	0.286	0.555	809.619	0.655	0.237	0.006	0.045
CFDP1	1179.223	0.595	0.213	0.005	0.021	1179.223	0.374	0.213	0.080	0.371	1179.223	-0.221	0.246	0.370	0.602
TMEM147	1121.305	0.502	0.290	0.083	0.171	1121.305	-0.325	0.291	0.264	0.536	1121.305	-0.827	0.336	0.014	0.080
LINC01399	22.706	-1.068	0.390	0.006	0.023	22.706	0.238	0.338	0.481	0.700	22.706	1.306	0.435	0.003	0.027
RBM14	642.508	-0.235	0.274	0.391	0.530	642.508	-0.421	0.274	0.124	0.416	642.508	-0.186	0.316	0.557	0.749
SMAD1-AS1	88.078	-1.326	0.387	0.001	0.004	88.078	-0.252	0.376	0.502	0.714	88.078	1.074	0.445	0.016	0.088
LINC01202	8.091	0.666	0.667	0.318	0.458	8.091	0.300	0.674	0.656	0.812	8.091	-0.366	0.770	0.634	0.801
LYPLA1	1346.714	-0.818	0.250	0.001	0.006	1346.714	-0.186	0.249	0.456	0.683	1346.714	0.633	0.289	0.028	0.127
CDC42EP2	61.711	-0.021	0.301	0.944	0.965	61.711	-0.230	0.301	0.444	0.675	61.711	-0.209	0.351	0.551	0.745
SGMS1-AS1	20.793	-0.045	0.420	0.915	0.946	20.793	0.241	0.409	0.556	0.747	20.793	0.286	0.482	0.553	0.746
LENG8-AS1	28.670	0.255	0.344	0.458	0.593	28.670	0.557	0.333	0.094	0.389	28.670	0.302	0.389	0.438	0.659
EMG1	1373.624	-0.077	0.280	0.785	0.858	1373.624	-0.484	0.280	0.084	0.376	1373.624	-0.407	0.324	0.208	0.433
C1D	260.092	-0.687	0.204	0.001	0.005	260.092	-0.535	0.202	0.008	0.297	260.092	0.152	0.237	0.522	0.724
TIMM17A	1273.326	-0.703	0.285	0.014	0.043	1273.326	-0.620	0.285	0.029	0.322	1273.326	0.082	0.329	0.803	0.901
SAPCD1-AS1	45.649	2.387	0.566	0.000	0.000	45.649	1.347	0.571	0.018	0.306	45.649	-1.041	0.647	0.108	0.293
N4BP2L2	634.207	-0.297	0.119	0.013	0.040	634.207	-0.155	0.118	0.189	0.475	634.207	0.142	0.137	0.301	0.537
ZER1	695.591	0.637	0.323	0.049	0.113	695.591	0.594	0.323	0.066	0.354	695.591	-0.042	0.373	0.910	0.959
MCRS1	774.729	0.056	0.277	0.839	0.894	774.729	-0.405	0.277	0.144	0.435	774.729	-0.461	0.320	0.150	0.358
FAM3C	1425.418	-0.525	0.296	0.076	0.159	1425.418	-0.617	0.296	0.037	0.325	1425.418	-0.092	0.342	0.788	0.894
SNHG14	302.971	-0.578	0.223	0.009	0.033	302.971	-0.317	0.221	0.151	0.444	302.971	0.262	0.257	0.310	0.545
ACAA2	974.464	0.018	0.142	0.900	0.935	974.464	0.200	0.142	0.157	0.449	974.464	0.182	0.164	0.266	0.500
CDX2	40.117	0.395	0.306	0.197	0.323	40.117	0.519	0.301	0.084	0.376	40.117	0.124	0.348	0.721	0.856
PPIE	627.865	-0.123	0.113	0.277	0.414	627.865	-0.033	0.112	0.767	0.877	627.865	0.090	0.130	0.492	0.701
VAV3	64.030	0.523	0.298	0.079	0.164	64.030	-0.470	0.309	0.128	0.420	64.030	-0.993	0.353	0.005	0.040
TOMM40	2604.366	-0.453	0.417	0.277	0.414	2604.366	-1.045	0.417	0.012	0.305	2604.366	-0.591	0.482	0.220	0.446
TAB1	663.206	-0.117	0.131	0.372	0.512	663.206	0.085	0.130	0.513	0.720	663.206	0.202	0.151	0.181	0.400
ECI2	1840.374	0.044	0.184	0.812	0.876	1840.374	0.265	0.183	0.148	0.440	1840.374	0.222	0.212	0.296	0.531
HAX1	1451.674	-0.103	0.172	0.551	0.674	1451.674	0.015	0.172	0.932	0.966	1451.674	0.117	0.199	0.556	0.748
GPNMB	425.734	7.206	0.471	0.000	0.000	425.734	0.887	0.504	0.078	0.370	425.734	-6.319	0.542	0.000	0.000
BAIAP2	975.238	0.296	0.361	0.412	0.550	975.238	0.443	0.361	0.220	0.499	975.238	0.147	0.417	0.725	0.858

MAD2L2	981.553	0.616	0.197	0.002	0.009	981.553	0.096	0.197	0.625	0.794	981.553	-0.519	0.227	0.022	0.108
TACC3	1346.797	0.114	0.262	0.665	0.766	1346.797	-0.256	0.262	0.329	0.590	1346.797	-0.370	0.303	0.223	0.450
MERTK	21.241	0.771	0.418	0.065	0.141	21.241	0.685	0.416	0.099	0.394	21.241	-0.086	0.474	0.856	0.931
SLC30A9	1397.755	-0.708	0.192	0.000	0.002	1397.755	0.092	0.191	0.631	0.798	1397.755	0.800	0.222	0.000	0.006
PIBF1	351.260	-0.483	0.167	0.004	0.016	351.260	-0.352	0.166	0.033	0.322	351.260	0.131	0.194	0.498	0.706
PPIH	597.642	0.282	0.233	0.226	0.358	597.642	-0.268	0.234	0.251	0.525	597.642	-0.550	0.270	0.041	0.162
COG5	964.049	-0.447	0.122	0.000	0.002	964.049	-0.118	0.121	0.327	0.587	964.049	0.328	0.141	0.020	0.100
ZNHIT1	1054.372	0.900	0.215	0.000	0.000	1054.372	0.158	0.216	0.464	0.689	1054.372	-0.742	0.248	0.003	0.028
TIMM44	1813.812	-0.633	0.288	0.028	0.074	1813.812	-0.378	0.288	0.189	0.475	1813.812	0.255	0.332	0.442	0.662
CLGN	56.765	0.121	0.480	0.802	0.869	56.765	0.969	0.470	0.039	0.327	56.765	0.848	0.547	0.121	0.313
PFDN6	4108.412	0.278	0.243	0.253	0.387	4108.412	-0.178	0.243	0.464	0.689	4108.412	-0.456	0.281	0.104	0.288
ZBTB18	114.897	-0.148	0.182	0.416	0.553	114.897	-0.020	0.178	0.910	0.955	114.897	0.128	0.210	0.542	0.739
HMGN4	1329.725	-0.185	0.129	0.151	0.265	1329.725	0.014	0.129	0.914	0.957	1329.725	0.199	0.149	0.181	0.399
TADA3	1442.445	0.326	0.170	0.056	0.125	1442.445	0.298	0.170	0.079	0.371	1442.445	-0.027	0.197	0.890	0.947
TRIM38	366.337	0.351	0.147	0.017	0.050	366.337	0.063	0.147	0.667	0.819	366.337	-0.288	0.170	0.090	0.263
ATP5PD	3107.574	1.196	0.252	0.000	0.000	3107.574	0.337	0.252	0.181	0.469	3107.574	-0.859	0.291	0.003	0.030
UBE2E3	331.566	-0.382	0.153	0.012	0.040	331.566	-0.102	0.151	0.499	0.713	331.566	0.281	0.177	0.112	0.299
SLC25A17	284.196	-0.592	0.211	0.005	0.020	284.196	-0.480	0.210	0.022	0.314	284.196	0.111	0.245	0.649	0.811
SLC9A6	376.756	-0.129	0.187	0.491	0.624	376.756	-0.157	0.187	0.400	0.643	376.756	-0.028	0.217	0.896	0.951
KCNMB2-AS1	70.932	-0.102	0.387	0.791	0.862	70.932	0.663	0.378	0.080	0.371	70.932	0.766	0.442	0.083	0.250
EIF3M	4449.907	-0.267	0.096	0.005	0.020	4449.907	0.062	0.095	0.513	0.720	4449.907	0.330	0.110	0.003	0.028
NXF1	623.911	-0.129	0.161	0.425	0.562	623.911	-0.335	0.161	0.038	0.327	623.911	-0.206	0.187	0.271	0.506
SEC23B	1112.700	-0.589	0.299	0.048	0.113	1112.700	-0.594	0.298	0.046	0.335	1112.700	-0.005	0.345	0.988	0.993
SEC23A	2243.354	-0.650	0.235	0.006	0.022	2243.354	-0.262	0.235	0.264	0.536	2243.354	0.388	0.272	0.153	0.363
CAP2	140.146	-0.060	0.203	0.769	0.847	140.146	-0.365	0.204	0.073	0.362	140.146	-0.305	0.237	0.197	0.420
CAP1	4384.527	-0.219	0.211	0.300	0.438	4384.527	-0.558	0.211	0.008	0.297	4384.527	-0.339	0.244	0.164	0.379
CREB3	794.818	0.224	0.142	0.115	0.218	794.818	-0.015	0.143	0.917	0.959	794.818	-0.239	0.165	0.147	0.354
LRRC41	1426.597	-0.087	0.160	0.586	0.702	1426.597	0.319	0.159	0.045	0.334	1426.597	0.406	0.184	0.028	0.125
VTI1B	1353.651	-0.232	0.102	0.023	0.064	1353.651	0.130	0.101	0.196	0.479	1353.651	0.362	0.117	0.002	0.022
CRTAP	7775.442	0.164	0.268	0.539	0.664	7775.442	0.408	0.267	0.127	0.419	7775.442	0.244	0.309	0.429	0.652
SYNCRIP	5664.842	-0.829	0.213	0.000	0.001	5664.842	-0.553	0.213	0.009	0.297	5664.842	0.276	0.246	0.262	0.496
VAT1	5044.147	0.815	0.293	0.005	0.021	5044.147	0.537	0.293	0.067	0.354	5044.147	-0.278	0.338	0.411	0.637
STK25	1845.436	0.030	0.116	0.793	0.863	1845.436	-0.075	0.115	0.517	0.722	1845.436	-0.105	0.134	0.431	0.653
ENOX2	368.858	-0.845	0.247	0.001	0.004	368.858	-0.402	0.245	0.101	0.394	368.858	0.443	0.286	0.121	0.313
LOC104968399	845.808	0.446	0.151	0.003	0.013	845.808	-0.103	0.151	0.497	0.711	845.808	-0.549	0.174	0.002	0.019
UNC13B	275.723	-0.406	0.200	0.042	0.102	275.723	-0.525	0.200	0.009	0.297	275.723	-0.119	0.232	0.608	0.784
CARM1	2400.038	0.132	0.153	0.386	0.526	2400.038	-0.145	0.153	0.344	0.600	2400.038	-0.277	0.176	0.117	0.306
NCOA2	514.252	-0.697	0.257	0.007	0.025	514.252	-0.082	0.256	0.747	0.865	514.252	0.614	0.297	0.039	0.155
CEBPA	70.561	1.385	0.609	0.023	0.064	70.561	0.665	0.611	0.277	0.547	70.561	-0.721	0.702	0.305	0.540
SEMA6C	36.864	0.995	0.523	0.057	0.127	36.864	0.768	0.523	0.142	0.433	36.864	-0.227	0.600	0.705	0.847
SEMA6B	2896.755	1.149	0.218	0.000	0.000	2896.755	0.162	0.218	0.458	0.685	2896.755	-0.987	0.251	0.000	0.002
SEMA4F	82.614	0.692	0.286	0.016	0.048	82.614	0.435	0.287	0.129	0.421	82.614	-0.257	0.329	0.435	0.657
SEMA4D	159.222	0.596	0.241	0.014	0.043	159.222	0.248	0.243	0.307	0.572	159.222	-0.348	0.279	0.212	0.437
SEMA4B	1886.120	0.962	0.340	0.005	0.018	1886.120	0.623	0.340	0.067	0.354	1886.120	-0.340	0.392	0.386	0.615
CEBPB	7151.399	0.941	0.234	0.000	0.001	7151.399	0.222	0.235	0.345	0.601	7151.399	-0.719	0.271	0.008	0.056
SEMA3C	345.233	0.250	0.269	0.353	0.493	345.233	-0.188	0.270	0.486	0.703	345.233	-0.438	0.311	0.160	0.373
APPBP2	562.769	-0.321	0.236	0.174	0.296	562.769	0.146	0.235	0.536	0.734	562.769	0.467	0.273	0.087	0.257
MYBBP1A	1043.909	-0.512	0.344	0.136	0.246	1043.909	-0.658	0.344	0.055	0.343	1043.909	-0.146	0.397	0.712	0.850
FBLN5	1439.493	1.023	0.183	0.000	0.000	1439.493	0.168	0.184	0.361	0.613	1439.493	-0.855	0.211	0.000	0.001
CIB1	1296.522	0.576	0.218	0.008	0.029	1296.522	-0.178	0.218	0.415	0.653	1296.522	-0.754	0.252	0.003	0.027
CEBPD	5522.776	0.883	0.207	0.000	0.000	5522.776	0.258	0.207	0.212	0.492	5522.776	-0.625	0.239	0.009	0.060
ZNF211	86.173	0.112	0.249	0.653	0.757	86.173	0.050	0.248	0.839	0.916	86.173	-0.062	0.288	0.830	0.917
DDX17	2695.035	0.522	0.251	0.037	0.092	2695.035	0.413	0.251	0.099	0.394	2695.035	-0.109	0.289	0.707	0.848
DEAF1	990.699	0.483	0.168	0.004	0.016	990.699	0.262	0.168	0.118	0.409	990.699	-0.221	0.194	0.254	0.487
BISPR	162.439	0.527	0.458	0.250	0.384	162.439	0.772	0.457	0.091	0.385	162.439	0.245	0.527	0.643	0.806
CHERP	1370.367	-0.276	0.160	0.085	0.173	1370.367	-0.186	0.160	0.245	0.520	1370.367	0.090	0.185	0.626	0.796
KAT5	695.135	0.020	0.105	0.849	0.901	695.135	0.008	0.105	0.939	0.969	695.135	-0.012	0.122	0.921	0.963
HYOU1	5433.361	-0.585	0.320	0.068	0.145	5433.361	-0.438	0.320	0.171	0.460	5433.361	0.146	0.370	0.692	0.839
IPO8	1086.357	-0.784	0.210	0.000	0.002	1086.357	-0.187	0.209	0.371	0.620	1086.357	0.597	0.242	0.014	0.081
IPO7	7338.686	-0.637	0.164	0.000	0.001	7338.686	-0.166	0.164	0.310	0.575	7338.686	0.471	0.189	0.013	0.077
LOC105274304	8.175	0.485	0.543	0.371	0.512	8.175	-0.430	0.583	0.461	0.687	8.175	-0.915	0.659	0.165	0.379
NOP56	1114.323	-1.185	0.290	0.000	0.000	1114.323	-0.544	0.289	0.060	0.350	1114.323	0.641	0.335	0.056	0.196
NEBL	1109.620	-0.902	0.425	0.034	0.086	1109.620	0.275	0.424	0.516	0.722	1109.620	1.177	0.490	0.016	0.090
PITRM1	2164.422	-0.695	0.289	0.016	0.049	2164.422	-0.614	0.289	0.034	0.322	2164.422	0.082	0.334	0.807	0.903
ATG7	955.349	0.073	0.174	0.674	0.773	955.349	-0.259	0.174	0.136	0.427	955.349	-0.332	0.201	0.098	0.277
ZNRD2	430.703	-0.382	0.290	0.188	0.312	430.703	-0.511	0.290	0.078	0.369	430.703	-0.129	0.336	0.700	0.843
RNASEH2A	662.168	-0.127	0.304	0.676	0.775	662.168	-0.395	0.305	0.195	0.478	662.168	-0.268	0.352	0.446	0.666

P3H3	545.855	1.331	0.271	0.000	0.000	545.855	-0.211	0.273	0.440	0.672	545.855	-1.542	0.314	0.000	0.000
LOC105369147	15.981	0.192	0.457	0.675	0.774	15.981	0.048	0.456	0.915	0.958	15.981	-0.143	0.529	0.787	0.894
PKD1P6-NPIPP1	1420.310	-0.325	0.258	0.208	0.336	1420.310	0.303	0.257	0.239	0.515	1420.310	0.628	0.298	0.035	0.145
LOC105369332	362.299	0.070	0.346	0.839	0.894	362.299	-0.324	0.346	0.350	0.605	362.299	-0.394	0.400	0.325	0.559
PPP1R14B-AS1	81.246	0.889	0.266	0.001	0.005	81.246	0.282	0.272	0.301	0.567	81.246	-0.607	0.308	0.048	0.179
LOC105369438	13.580	0.084	0.451	0.852	0.903	13.580	-0.084	0.450	0.852	0.924	13.580	-0.168	0.528	0.750	0.872
DDN-AS1	48.516	0.583	0.351	0.096	0.190	48.516	0.027	0.356	0.939	0.969	48.516	-0.556	0.408	0.173	0.390
LNCOG	12.823	0.537	0.724	0.458	0.593	12.823	-2.410	0.852	0.005	0.297	12.823	-2.948	0.947	0.002	0.021
PPP1R12A-AS1	84.605	-0.791	0.262	0.003	0.012	84.605	-0.153	0.252	0.544	0.739	84.605	0.638	0.302	0.034	0.144
PCCA-DT	28.267	-0.756	0.509	0.137	0.248	28.267	-0.984	0.510	0.054	0.342	28.267	-0.228	0.600	0.704	0.846
G2E3-AS1	22.648	-1.396	0.429	0.001	0.006	22.648	-0.278	0.384	0.468	0.691	22.648	1.118	0.488	0.022	0.107
SEC23A-AS1	34.195	-1.484	0.423	0.000	0.003	34.195	-0.425	0.391	0.277	0.547	34.195	1.059	0.484	0.029	0.128
LOC105370489	11.886	0.074	0.607	0.903	0.938	11.886	-0.364	0.615	0.555	0.746	11.886	-0.438	0.712	0.539	0.736
LOC105370792	157.690	0.309	0.277	0.264	0.400	157.690	-0.614	0.282	0.029	0.322	157.690	-0.923	0.324	0.004	0.037
LOC105371049	191.565	-0.385	0.218	0.078	0.161	191.565	0.012	0.214	0.954	0.978	191.565	0.398	0.251	0.114	0.302
LIX1L-AS1	808.285	-0.462	0.153	0.003	0.012	808.285	-0.173	0.152	0.255	0.529	808.285	0.289	0.177	0.102	0.285
LOC105371267	10.139	0.550	0.648	0.396	0.535	10.139	0.264	0.652	0.686	0.831	10.139	-0.286	0.748	0.702	0.845
ATP2C2-AS1	24.043	1.816	0.489	0.000	0.002	24.043	0.448	0.510	0.380	0.626	24.043	-1.368	0.566	0.016	0.088
LOC105371414	12.443	1.471	0.761	0.053	0.121	12.443	-0.774	0.821	0.346	0.602	12.443	-2.245	0.919	0.015	0.083
LOC105371730	136.776	-0.528	0.292	0.070	0.150	136.776	0.081	0.287	0.778	0.883	136.776	0.609	0.336	0.070	0.225
SNHG30	173.577	0.406	0.358	0.257	0.391	173.577	-0.600	0.363	0.098	0.393	173.577	-1.006	0.418	0.016	0.089
LOC105371795	144.872	0.448	0.415	0.280	0.418	144.872	0.781	0.413	0.058	0.348	144.872	0.334	0.477	0.484	0.696
LOC105371814	151.849	0.029	0.195	0.882	0.924	151.849	0.222	0.191	0.246	0.521	151.849	0.193	0.224	0.389	0.617
LOC105371849	328.619	0.118	0.139	0.393	0.532	328.619	0.220	0.137	0.107	0.399	328.619	0.102	0.159	0.522	0.724
SEPTIN9-DT	8.271	1.863	0.609	0.002	0.011	8.271	1.671	0.610	0.006	0.297	8.271	-0.193	0.632	0.760	0.878
LOC105371925	145.945	0.446	0.250	0.075	0.157	145.945	-0.267	0.254	0.293	0.561	145.945	-0.712	0.292	0.015	0.083
LOC105371998	124.084	-0.773	0.214	0.000	0.002	124.084	-0.310	0.206	0.132	0.424	124.084	0.462	0.247	0.061	0.208
GEMIN7-AS1	259.221	-0.182	0.265	0.491	0.624	259.221	-0.812	0.268	0.002	0.297	259.221	-0.630	0.310	0.042	0.164
LOC105372480	520.785	-0.184	0.328	0.575	0.693	520.785	-0.529	0.328	0.107	0.399	520.785	-0.345	0.379	0.363	0.595
ERVK3-1	203.664	-0.458	0.391	0.241	0.374	203.664	-0.832	0.392	0.034	0.322	203.664	-0.374	0.454	0.410	0.636
LINC01684	31.308	0.845	0.452	0.061	0.135	31.308	0.394	0.456	0.387	0.633	31.308	-0.451	0.521	0.387	0.615
LINC01694	8.221	-0.074	1.176	0.950	0.969	8.221	1.102	1.149	0.338	0.596	8.221	1.176	1.341	0.381	0.610
LOC105373021	18.094	0.383	0.410	0.350	0.490	18.094	-0.123	0.422	0.771	0.878	18.094	-0.506	0.483	0.295	0.530
LINC01703	12.402	0.406	0.491	0.408	0.546	12.402	1.202	0.459	0.009	0.297	12.402	0.796	0.539	0.139	0.343
LOC105373159	151.596	-0.197	0.293	0.501	0.631	151.596	0.485	0.288	0.092	0.387	151.596	0.682	0.336	0.042	0.164
MYOSLID	168.429	-0.753	0.224	0.001	0.005	168.429	-0.218	0.218	0.318	0.580	168.429	0.535	0.259	0.039	0.155
LINC02482	26.810	1.007	0.526	0.055	0.125	26.810	0.564	0.530	0.287	0.556	26.810	-0.443	0.604	0.463	0.680
LINC02241	75.591	-0.059	0.477	0.901	0.936	75.591	0.643	0.472	0.173	0.462	75.591	0.702	0.548	0.200	0.424
LINC02211	89.533	-0.388	0.446	0.384	0.524	89.533	0.161	0.442	0.716	0.848	89.533	0.549	0.514	0.286	0.521
LINC02228	8.915	-0.005	0.727	0.995	0.997	8.915	0.768	0.698	0.271	0.542	8.915	0.773	0.822	0.347	0.581
LOC105374727	9.790	0.331	0.635	0.602	0.715	9.790	0.201	0.634	0.751	0.867	9.790	-0.130	0.733	0.859	0.932
LOC105374952	290.084	-0.990	0.320	0.002	0.010	290.084	-0.408	0.317	0.198	0.482	290.084	0.582	0.369	0.115	0.304
HCG20	550.563	-0.203	0.462	0.660	0.763	550.563	-0.997	0.464	0.031	0.322	550.563	-0.794	0.535	0.138	0.341
LOC105375304	153.227	0.713	0.322	0.027	0.072	153.227	0.123	0.324	0.705	0.842	153.227	-0.590	0.373	0.113	0.301
TMBIM7P	68.201	-0.741	0.451	0.100	0.196	68.201	0.145	0.441	0.741	0.862	68.201	0.887	0.518	0.087	0.257
LINC02476	87.364	0.148	0.413	0.720	0.810	87.364	0.716	0.408	0.079	0.370	87.364	0.569	0.473	0.230	0.459
LOC105375545	8.420	1.270	0.605	0.036	0.090	8.420	-0.201	0.663	0.762	0.874	8.420	-1.471	0.729	0.044	0.168
MAL2-AS1	13.905	0.430	0.481	0.372	0.512	13.905	0.220	0.482	0.648	0.808	13.905	-0.210	0.555	0.705	0.847
LOC105376114	407.340	-1.321	0.420	0.002	0.009	407.340	-0.394	0.418	0.346	0.602	407.340	0.927	0.485	0.056	0.196
EXOSC10-AS1	37.671	-1.111	0.319	0.000	0.003	37.671	-0.337	0.294	0.251	0.525	37.671	0.774	0.366	0.034	0.144
LOC105376805	34.827	0.424	0.425	0.318	0.458	34.827	0.692	0.418	0.098	0.393	34.827	0.268	0.484	0.580	0.764
SNHG25	13.558	0.775	0.585	0.185	0.309	13.558	0.429	0.591	0.468	0.691	13.558	-0.347	0.671	0.606	0.782
LINC01980	118.866	0.157	0.360	0.662	0.765	118.866	0.482	0.358	0.178	0.467	118.866	0.324	0.414	0.434	0.656
FLNB-AS1	584.924	0.092	0.124	0.456	0.591	584.924	0.045	0.123	0.713	0.846	584.924	-0.047	0.143	0.742	0.868
LINC02562	28.969	0.339	0.574	0.554	0.676	28.969	0.521	0.569	0.361	0.613	28.969	0.182	0.659	0.783	0.891
UBE2D3-AS1	11.750	0.801	0.481	0.096	0.190	11.750	0.708	0.478	0.138	0.429	11.750	-0.093	0.541	0.864	0.935
LOC105377590	29.090	0.667	0.369	0.071	0.151	29.090	0.382	0.372	0.304	0.570	29.090	-0.285	0.424	0.502	0.709
LOC105377621	12.216	1.625	0.561	0.004	0.016	12.216	0.246	0.596	0.680	0.827	12.216	-1.379	0.657	0.036	0.148
LOC105377623	7.291	-3.670	1.301	0.005	0.019	7.291	0.793	0.830	0.340	0.598	7.291	4.463	1.381	0.001	0.015
LOC105377763	17.754	-0.027	0.458	0.953	0.972	17.754	0.593	0.430	0.168	0.457	17.754	0.620	0.514	0.228	0.456
LINC02518	8.657	-3.238	1.448	0.025	0.069	8.657	0.117	1.264	0.926	0.963	8.657	3.354	1.622	0.039	0.155
LOC105378663	30.685	1.423	0.549	0.010	0.033	30.685	0.291	0.565	0.606	0.782	30.685	-1.132	0.637	0.076	0.237
LINC01748	106.480	-0.582	0.206	0.005	0.019	106.480	-0.087	0.196	0.659	0.814	106.480	0.495	0.236	0.036	0.148
LINC02609	133.427	0.138	0.237	0.560	0.681	133.427	0.573	0.232	0.014	0.306	133.427	0.435	0.271	0.108	0.294
LINC02057	15.856	-0.656	0.656	0.318	0.458	15.856	0.054	0.635	0.932	0.966	15.856	0.710	0.753	0.346	0.580
LOC105379511	7.286	-0.457	0.877	0.602	0.715	7.286	0.800	0.823	0.331	0.591	7.286	1.256	0.987	0.203	0.427
IER3-AS1	1852.557	0.240	0.451	0.594	0.708	1852.557	-0.829	0.451	0.066	0.354	1852.557	-1.070	0.521	0.040	0.159

LOC105379807	27.913	-3.062	0.602	0.000	0.000	27.913	-0.486	0.497	0.328	0.588	27.913	2.576	0.670	0.000	0.003
GLRX3	2182.927	-0.475	0.198	0.017	0.050	2182.927	-0.276	0.198	0.163	0.454	2182.927	0.199	0.229	0.386	0.615
CEBPG	1726.360	-1.204	0.296	0.000	0.001	1726.360	-0.610	0.296	0.039	0.327	1726.360	0.595	0.342	0.082	0.248
DCTN2	2173.720	-0.024	0.119	0.841	0.896	2173.720	0.005	0.119	0.964	0.981	2173.720	0.029	0.137	0.832	0.918
ANP32B	7631.740	0.063	0.156	0.687	0.784	7631.740	0.178	0.156	0.254	0.527	7631.740	0.116	0.181	0.522	0.724
NKILA	22.609	0.388	0.342	0.257	0.391	22.609	-0.300	0.358	0.403	0.645	22.609	-0.688	0.409	0.092	0.267
LAMTOR5	1319.471	0.768	0.160	0.000	0.000	1319.471	0.151	0.161	0.347	0.603	1319.471	-0.616	0.185	0.001	0.012
PROCR	1224.664	-0.080	0.169	0.635	0.743	1224.664	0.042	0.168	0.805	0.898	1224.664	0.121	0.195	0.533	0.732
TM9SF1	931.381	-0.104	0.167	0.533	0.659	931.381	0.074	0.167	0.659	0.814	931.381	0.178	0.193	0.357	0.590
PRDX4	1621.785	-0.222	0.182	0.222	0.353	1621.785	-0.123	0.182	0.498	0.711	1621.785	0.099	0.210	0.637	0.803
ARL6IP5	2928.236	0.020	0.265	0.940	0.963	2928.236	0.255	0.265	0.337	0.595	2928.236	0.235	0.306	0.443	0.663
AGR2	211.588	0.489	0.241	0.043	0.102	211.588	0.266	0.241	0.270	0.541	211.588	-0.222	0.278	0.424	0.648
ARPC1A	281.937	1.077	0.313	0.001	0.004	281.937	-0.016	0.316	0.961	0.979	281.937	-1.093	0.363	0.003	0.026
HTATIP2	890.059	0.026	0.257	0.921	0.950	890.059	0.388	0.257	0.131	0.423	890.059	0.362	0.297	0.222	0.450
AGPAT1	6658.211	-0.018	0.174	0.919	0.949	6658.211	0.167	0.174	0.335	0.594	6658.211	0.185	0.201	0.356	0.590
AGPAT2	1064.787	0.897	0.167	0.000	0.000	1064.787	0.239	0.168	0.155	0.447	1064.787	-0.658	0.193	0.001	0.010
RPP30	470.401	-0.148	0.248	0.550	0.672	470.401	-0.447	0.248	0.072	0.361	470.401	-0.298	0.287	0.299	0.534
RPP38	259.197	-0.139	0.258	0.589	0.705	259.197	-0.451	0.259	0.081	0.372	259.197	-0.311	0.299	0.298	0.534
SPTLC1	1271.408	-0.170	0.142	0.233	0.365	1271.408	-0.106	0.142	0.456	0.683	1271.408	0.064	0.164	0.697	0.841
SLC35A1	458.189	0.445	0.126	0.000	0.003	458.189	0.139	0.126	0.269	0.541	458.189	-0.305	0.145	0.036	0.148
CEL	41.188	0.041	1.195	0.972	0.983	41.188	0.724	1.192	0.544	0.739	41.188	0.683	1.378	0.620	0.792
SLC19A2	709.038	-0.581	0.182	0.001	0.007	709.038	-0.234	0.180	0.195	0.479	709.038	0.348	0.210	0.098	0.276
IFI44	694.635	1.646	0.367	0.000	0.000	694.635	0.769	0.367	0.036	0.324	694.635	-0.878	0.423	0.038	0.154
ARFGEF2	894.678	-0.839	0.238	0.000	0.003	894.678	-0.466	0.238	0.050	0.337	894.678	0.373	0.276	0.175	0.393
ARFGEF1	944.765	-0.835	0.260	0.001	0.007	944.765	-0.026	0.259	0.920	0.960	944.765	0.809	0.300	0.007	0.052
AKAP3	14.915	-0.900	0.579	0.120	0.224	14.915	0.337	0.530	0.524	0.727	14.915	1.238	0.653	0.058	0.201
RABAC1	1277.107	1.288	0.287	0.000	0.000	1277.107	0.437	0.287	0.128	0.420	1277.107	-0.851	0.331	0.010	0.066
SLU7	1060.356	0.207	0.114	0.071	0.151	1060.356	-0.035	0.115	0.758	0.871	1060.356	-0.242	0.132	0.068	0.221
DPYSL4	101.929	0.153	1.115	0.891	0.930	101.929	1.150	1.111	0.301	0.567	101.929	0.997	1.285	0.438	0.659
SIVA1	793.960	0.525	0.269	0.051	0.118	793.960	-0.370	0.270	0.171	0.460	793.960	-0.895	0.312	0.004	0.036
MRPL28	1144.838	0.312	0.290	0.282	0.420	1144.838	-0.366	0.290	0.207	0.489	1144.838	-0.677	0.335	0.043	0.166
CCT7	8004.921	-0.222	0.218	0.309	0.449	8004.921	-0.392	0.218	0.072	0.361	8004.921	-0.170	0.252	0.499	0.706
CCT4	4757.425	-0.710	0.157	0.000	0.000	4757.425	-0.103	0.157	0.512	0.719	4757.425	0.607	0.181	0.001	0.011
LRP1-AS	52.522	-0.935	1.266	0.460	0.595	52.522	0.560	1.259	0.656	0.813	52.522	1.495	1.459	0.305	0.541
CCT2	5159.790	-0.618	0.214	0.004	0.016	5159.790	-0.445	0.214	0.037	0.325	5159.790	0.173	0.247	0.483	0.695
NPC2	2559.246	1.485	0.215	0.000	0.000	2559.246	0.178	0.216	0.409	0.649	2559.246	-1.306	0.249	0.000	0.000
TACC2	498.812	-0.648	0.215	0.003	0.012	498.812	-0.258	0.213	0.227	0.505	498.812	0.390	0.248	0.116	0.306
CENPA	351.060	-0.429	0.260	0.098	0.193	351.060	0.044	0.258	0.866	0.931	351.060	0.473	0.299	0.114	0.302
SORBS1	74.086	1.387	0.345	0.000	0.001	74.086	0.031	0.354	0.931	0.965	74.086	-1.356	0.402	0.001	0.011
IFITM2	1711.922	1.489	0.258	0.000	0.000	1711.922	0.568	0.258	0.028	0.321	1711.922	-0.921	0.298	0.002	0.022
POMT1	349.283	0.247	0.236	0.294	0.432	349.283	0.330	0.235	0.160	0.451	349.283	0.083	0.272	0.761	0.878
TXNRD2	826.967	-0.076	0.251	0.762	0.842	826.967	-0.300	0.251	0.231	0.508	826.967	-0.224	0.290	0.439	0.659
MTHFS	346.045	0.266	0.219	0.225	0.356	346.045	0.117	0.219	0.594	0.775	346.045	-0.149	0.253	0.555	0.748
DRAP1	2709.009	0.589	0.306	0.054	0.123	2709.009	-0.364	0.307	0.235	0.513	2709.009	-0.953	0.354	0.007	0.052
CENPB	2372.310	0.127	0.177	0.473	0.607	2372.310	-0.190	0.177	0.284	0.553	2372.310	-0.318	0.205	0.121	0.313
DNPH1	1403.631	0.636	0.239	0.008	0.028	1403.631	0.121	0.240	0.613	0.786	1403.631	-0.514	0.276	0.063	0.211
SMC2	1327.932	-0.838	0.161	0.000	0.000	1327.932	-0.248	0.160	0.120	0.412	1327.932	0.591	0.185	0.001	0.017
PRPF8	5937.176	-0.923	0.178	0.000	0.000	5937.176	-0.207	0.178	0.245	0.520	5937.176	0.717	0.206	0.000	0.008
ERN2	54.344	0.410	0.273	0.134	0.243	54.344	0.456	0.270	0.091	0.386	54.344	0.046	0.312	0.882	0.943
TRAPPC2B	72.816	0.942	0.310	0.002	0.011	72.816	-0.029	0.319	0.928	0.964	72.816	-0.971	0.362	0.007	0.054
AHSA1	1818.428	0.008	0.355	0.982	0.989	1818.428	-0.981	0.355	0.006	0.297	1818.428	-0.989	0.410	0.016	0.088
CENPC	137.912	-0.173	0.196	0.377	0.517	137.912	0.093	0.192	0.630	0.797	137.912	0.266	0.226	0.239	0.469
USP16	908.498	-0.797	0.164	0.000	0.000	908.498	-0.315	0.163	0.053	0.342	908.498	0.482	0.190	0.011	0.070
CDC42EP3	278.950	0.313	0.207	0.132	0.240	278.950	-0.585	0.211	0.006	0.297	278.950	-0.897	0.243	0.000	0.004
SH2B2	155.349	1.137	0.408	0.005	0.021	155.349	0.640	0.409	0.117	0.409	155.349	-0.497	0.470	0.290	0.525
PAIP1	2875.016	-0.339	0.086	0.000	0.001	2875.016	-0.042	0.086	0.622	0.791	2875.016	0.297	0.100	0.003	0.028
PAICS	5613.017	-0.251	0.120	0.037	0.092	5613.017	-0.178	0.120	0.138	0.429	5613.017	0.073	0.139	0.602	0.780
TBL3	1076.262	-0.126	0.330	0.704	0.796	1076.262	-0.474	0.330	0.151	0.444	1076.262	-0.348	0.381	0.361	0.594
MXD4	3896.314	0.370	0.412	0.368	0.509	3896.314	0.569	0.411	0.166	0.456	3896.314	0.199	0.475	0.675	0.828
P3H4	1484.768	0.620	0.121	0.000	0.000	1484.768	0.017	0.121	0.888	0.944	1484.768	-0.603	0.140	0.000	0.001
ST6GALNAC2	1001.226	0.912	0.227	0.000	0.001	1001.226	0.518	0.227	0.023	0.315	1001.226	-0.395	0.262	0.132	0.330
PDLIM5	1028.874	-0.424	0.187	0.023	0.065	1028.874	-0.331	0.187	0.076	0.365	1028.874	0.093	0.216	0.668	0.823
TRIM3	132.293	0.316	0.176	0.072	0.153	132.293	-0.087	0.178	0.625	0.794	132.293	-0.403	0.205	0.050	0.182
ERLIN1	1556.600	-0.731	0.176	0.000	0.000	1556.600	-0.144	0.176	0.413	0.652	1556.600	0.587	0.204	0.004	0.035
HEXIM1	1437.207	0.329	0.154	0.032	0.084	1437.207	0.182	0.154	0.238	0.515	1437.207	-0.148	0.178	0.406	0.633
PLA2G4C-AS1	16.324	3.088	0.613	0.000	0.000	16.324	0.888	0.730	0.224	0.503	16.324	-2.200	0.682	0.001	0.016
GRPEL2-AS1	43.096	-1.614	0.504	0.001	0.007	43.096	-0.935	0.488	0.055	0.343	43.096	0.679	0.584	0.244	0.475

ARF4-AS1	12.057	0.072	0.488	0.883	0.924	12.057	0.546	0.462	0.237	0.514	12.057	0.474	0.549	0.387	0.616
NOP53-AS1	108.040	0.939	0.442	0.034	0.086	108.040	0.626	0.443	0.157	0.450	108.040	-0.313	0.509	0.539	0.736
FARSA-AS1	456.325	-1.045	0.281	0.000	0.002	456.325	-0.442	0.279	0.113	0.405	456.325	0.602	0.325	0.064	0.212
SPAG5	975.759	-0.376	0.181	0.038	0.094	975.759	0.062	0.181	0.732	0.857	975.759	0.438	0.209	0.036	0.149
RBCK1	1915.829	0.063	0.135	0.640	0.747	1915.829	0.209	0.135	0.121	0.413	1915.829	0.146	0.156	0.350	0.583
STAMBP	744.285	-0.154	0.140	0.270	0.406	744.285	0.057	0.139	0.680	0.827	744.285	0.212	0.162	0.190	0.411
TGOLN2	6583.529	-0.177	0.207	0.394	0.532	6583.529	0.150	0.207	0.469	0.691	6583.529	0.327	0.239	0.172	0.389
CENPE	763.392	-0.452	0.218	0.038	0.094	763.392	-0.276	0.218	0.206	0.488	763.392	0.177	0.252	0.483	0.696
ARID3B	59.678	0.153	0.268	0.567	0.687	59.678	0.424	0.261	0.104	0.397	59.678	0.271	0.305	0.375	0.607
POLR3F	124.185	-0.367	0.179	0.040	0.097	124.185	-0.404	0.177	0.022	0.315	124.185	-0.037	0.209	0.858	0.932
POLR3G	408.688	-1.352	0.437	0.002	0.010	408.688	-1.434	0.437	0.001	0.297	408.688	-0.082	0.506	0.872	0.939
POLR3C	618.875	-0.776	0.224	0.001	0.003	618.875	-0.446	0.223	0.045	0.334	618.875	0.331	0.259	0.201	0.424
IVNS1ABP	1844.049	-1.062	0.214	0.000	0.000	1844.049	-0.310	0.214	0.146	0.438	1844.049	0.752	0.248	0.002	0.025
TRIM16	729.747	0.188	0.154	0.222	0.352	729.747	0.224	0.153	0.145	0.436	729.747	0.036	0.177	0.841	0.923
MYL12A	2852.282	0.520	0.137	0.000	0.001	2852.282	-0.052	0.137	0.703	0.841	2852.282	-0.572	0.158	0.000	0.005
TXNIP	24403.232	-0.602	0.474	0.204	0.332	24403.232	0.689	0.474	0.146	0.438	24403.232	1.291	0.547	0.018	0.096
TAF6L	256.468	0.372	0.228	0.102	0.199	256.468	-0.282	0.230	0.221	0.500	256.468	-0.654	0.265	0.014	0.080
CENPF	3772.636	-0.163	0.202	0.419	0.556	3772.636	-0.070	0.202	0.730	0.856	3772.636	0.094	0.233	0.688	0.836
PDPN	6122.830	0.260	0.129	0.044	0.104	6122.830	0.012	0.129	0.927	0.963	6122.830	-0.248	0.149	0.096	0.272
ATP5MG	2913.562	0.524	0.184	0.004	0.018	2913.562	0.185	0.184	0.314	0.577	2913.562	-0.339	0.212	0.110	0.296
GAS2L1	672.507	0.100	0.157	0.526	0.653	672.507	-0.027	0.157	0.863	0.930	672.507	-0.127	0.182	0.486	0.697
RAD51AP1	349.708	-1.028	0.352	0.003	0.015	349.708	-0.457	0.350	0.192	0.477	349.708	0.571	0.407	0.160	0.373
RGS14	64.197	1.183	0.328	0.000	0.002	64.197	0.356	0.336	0.289	0.557	64.197	-0.827	0.379	0.029	0.130
EXOC5	1301.641	-0.645	0.219	0.003	0.014	1301.641	-0.435	0.218	0.047	0.335	1301.641	0.210	0.253	0.406	0.632
NPRL2	363.768	0.343	0.208	0.099	0.194	363.768	0.351	0.207	0.090	0.384	363.768	0.009	0.239	0.971	0.986
IGF2BP3	20.821	-0.931	0.566	0.100	0.196	20.821	-0.480	0.548	0.381	0.627	20.821	0.451	0.656	0.492	0.702
IGF2BP2	1495.601	-0.540	0.165	0.001	0.006	1495.601	-0.165	0.164	0.315	0.578	1495.601	0.375	0.191	0.049	0.181
CAMKK2	1371.016	-0.475	0.166	0.004	0.017	1371.016	0.041	0.165	0.802	0.896	1371.016	0.516	0.191	0.007	0.052
MDC1-AS1	718.064	-0.328	0.247	0.184	0.308	718.064	-0.142	0.246	0.565	0.753	718.064	0.186	0.285	0.514	0.718
DDX39B-AS1	5.268	-0.179	2.807	0.949	0.969	5.268	-1.448	2.829	0.609	0.784	5.268	-1.269	3.264	0.697	0.841
BMS1P22	86.281	0.181	0.280	0.517	0.645	86.281	0.363	0.277	0.189	0.475	86.281	0.182	0.321	0.571	0.759
PRELID3A	109.281	-0.228	0.293	0.436	0.572	109.281	-0.196	0.291	0.501	0.713	109.281	0.032	0.339	0.925	0.965
MTX2	486.943	-0.004	0.162	0.981	0.989	486.943	-0.082	0.162	0.610	0.785	486.943	-0.078	0.187	0.675	0.828
YKT6	1431.370	-0.532	0.349	0.128	0.235	1431.370	-0.848	0.349	0.015	0.306	1431.370	-0.316	0.404	0.434	0.656
SPINT2	270.027	0.396	0.178	0.026	0.070	270.027	0.304	0.178	0.087	0.380	270.027	-0.092	0.205	0.653	0.813
PMVK	456.454	0.140	0.188	0.457	0.592	456.454	0.414	0.186	0.026	0.318	456.454	0.274	0.216	0.204	0.428
KHDRBS3	282.438	0.694	0.274	0.011	0.038	282.438	-0.101	0.277	0.715	0.847	282.438	-0.795	0.318	0.013	0.076
KHDRBS1	6659.482	-0.200	0.140	0.152	0.267	6659.482	0.170	0.139	0.223	0.502	6659.482	0.370	0.161	0.022	0.107
CELF1	1356.143	0.168	0.152	0.268	0.404	1356.143	-0.112	0.152	0.461	0.687	1356.143	-0.280	0.176	0.110	0.296
CES1	1476.660	2.600	0.282	0.000	0.000	1476.660	0.035	0.283	0.903	0.951	1476.660	-2.565	0.326	0.000	0.000
MIR34AHG	7.796	1.119	0.723	0.122	0.226	7.796	0.112	0.755	0.882	0.940	7.796	-1.007	0.846	0.234	0.463
SNORD141A	84850.208	0.211	0.308	0.493	0.625	84850.208	0.403	0.308	0.190	0.475	84850.208	0.192	0.355	0.588	0.769
CTCF	1014.127	-1.298	0.256	0.000	0.000	1014.127	-0.527	0.255	0.038	0.327	1014.127	0.771	0.296	0.009	0.062
LOC106660606	10.646	0.113	0.579	0.845	0.898	10.646	0.060	0.573	0.917	0.959	10.646	-0.053	0.670	0.937	0.972
LINC00680	46.177	-0.688	0.371	0.063	0.138	46.177	-0.354	0.361	0.327	0.587	46.177	0.334	0.429	0.436	0.657
FARS2	227.474	-0.388	0.128	0.003	0.012	227.474	-0.045	0.124	0.718	0.849	227.474	0.343	0.148	0.020	0.102
CGRRF1	127.117	-0.535	0.270	0.048	0.112	127.117	-0.431	0.267	0.107	0.399	127.117	0.104	0.314	0.741	0.867
CGREF1	625.370	0.614	0.143	0.000	0.000	625.370	0.236	0.143	0.099	0.393	625.370	-0.377	0.165	0.022	0.108
RRAGA	374.227	0.496	0.144	0.001	0.004	374.227	0.047	0.146	0.749	0.867	374.227	-0.450	0.168	0.007	0.053
DCTN6	564.804	0.110	0.150	0.465	0.600	564.804	-0.019	0.150	0.898	0.949	564.804	-0.129	0.174	0.459	0.676
GNA13	1178.758	-0.512	0.184	0.005	0.021	1178.758	0.037	0.183	0.840	0.917	1178.758	0.549	0.213	0.010	0.065
PCF11-AS1	20.180	1.865	0.500	0.000	0.002	20.180	0.555	0.523	0.289	0.558	20.180	-1.311	0.577	0.023	0.111
CSPG5	28.338	1.443	0.462	0.002	0.009	28.338	0.341	0.486	0.483	0.701	28.338	-1.102	0.537	0.040	0.159
AVIL	25.857	0.699	0.334	0.037	0.091	25.857	-0.440	0.361	0.222	0.501	25.857	-1.139	0.405	0.005	0.040
B3GNT2	178.588	-0.241	0.285	0.398	0.536	178.588	-0.817	0.288	0.005	0.297	178.588	-0.576	0.334	0.084	0.252
GNB5	490.700	0.198	0.114	0.081	0.167	490.700	0.011	0.114	0.924	0.961	490.700	-0.187	0.132	0.155	0.365
EBP	1117.590	1.588	0.260	0.000	0.000	1117.590	-0.252	0.262	0.335	0.594	1117.590	-1.841	0.301	0.000	0.000
CETN2	387.003	0.985	0.181	0.000	0.000	387.003	0.029	0.183	0.876	0.937	387.003	-0.956	0.210	0.000	0.000
GMEB1	359.011	-0.495	0.233	0.034	0.086	359.011	-0.500	0.233	0.031	0.322	359.011	-0.006	0.270	0.983	0.991
CCT6B	10.573	-0.460	0.795	0.563	0.684	10.573	0.775	0.750	0.301	0.568	10.573	1.234	0.897	0.169	0.384
CCT8	8828.517	-0.213	0.108	0.048	0.112	8828.517	-0.123	0.108	0.254	0.527	8828.517	0.090	0.125	0.470	0.684
CNPY3	870.203	0.718	0.172	0.000	0.000	870.203	-0.005	0.173	0.975	0.986	870.203	-0.723	0.199	0.000	0.005
CORIN	8.230	-1.043	0.791	0.188	0.312	8.230	-0.146	0.727	0.841	0.917	8.230	0.897	0.902	0.320	0.555
CETN3	273.744	0.398	0.219	0.069	0.148	273.744	-0.059	0.220	0.790	0.890	273.744	-0.456	0.253	0.072	0.229
MIR3142HG	23.117	0.988	0.604	0.102	0.198	23.117	-0.156	0.630	0.805	0.898	23.117	-1.144	0.711	0.108	0.293
FAM189B	2731.202	0.314	0.138	0.023	0.064	2731.202	0.369	0.138	0.007	0.297	2731.202	0.056	0.160	0.728	0.859
USP39	2038.875	-0.588	0.301	0.051	0.117	2038.875	-0.786	0.301	0.009	0.297	2038.875	-0.198	0.348	0.569	0.758

SMURF2P1-LRRC37BP1	503.839	0.112	0.204	0.584	0.701	503.839	0.243	0.203	0.233	0.510	503.839	0.131	0.235	0.578	0.763
POLD3	326.916	-0.457	0.278	0.100	0.196	326.916	-0.502	0.277	0.070	0.359	326.916	-0.045	0.322	0.888	0.947
CERS1	15.083	2.366	0.662	0.000	0.003	15.083	1.998	0.669	0.003	0.297	15.083	-0.368	0.682	0.590	0.771
AP4B1	206.429	-0.510	0.314	0.104	0.201	206.429	-0.767	0.314	0.015	0.306	206.429	-0.257	0.365	0.482	0.694
CFL1	9775.085	0.541	0.251	0.031	0.080	9775.085	-0.368	0.251	0.142	0.433	9775.085	-0.910	0.290	0.002	0.019
POLQ	297.353	-0.663	0.354	0.061	0.135	297.353	-0.669	0.354	0.059	0.349	297.353	-0.006	0.410	0.988	0.994
SLC12A7	4251.283	0.013	0.238	0.955	0.973	4251.283	0.320	0.238	0.179	0.467	4251.283	0.306	0.275	0.264	0.499
OGA	2238.985	-0.350	0.166	0.035	0.088	2238.985	-0.005	0.165	0.974	0.986	2238.985	0.345	0.191	0.072	0.229
NFAT5	538.672	0.275	0.160	0.086	0.174	538.672	0.158	0.160	0.322	0.583	538.672	-0.117	0.185	0.527	0.728
NUDC	2573.051	-0.207	0.363	0.569	0.688	2573.051	-0.751	0.363	0.038	0.327	2573.051	-0.545	0.419	0.193	0.416
PTGES3	6211.208	-0.634	0.233	0.007	0.024	6211.208	-0.472	0.233	0.042	0.330	6211.208	0.161	0.269	0.549	0.744
BOLA2-SMG1P6	38.625	-0.837	0.361	0.020	0.058	38.625	-0.380	0.347	0.273	0.544	38.625	0.458	0.417	0.273	0.508
CFL2	1150.590	0.220	0.091	0.016	0.048	1150.590	-0.067	0.092	0.465	0.689	1150.590	-0.287	0.106	0.007	0.050
YME1L1	4017.142	-0.904	0.204	0.000	0.000	4017.142	-0.316	0.203	0.120	0.412	4017.142	0.587	0.235	0.012	0.076
TCFL5	521.530	-0.005	0.188	0.978	0.986	521.530	0.322	0.186	0.084	0.376	521.530	0.327	0.216	0.130	0.327
PLK4	306.621	-0.743	0.251	0.003	0.014	306.621	-0.414	0.249	0.097	0.391	306.621	0.329	0.290	0.257	0.491
STAG2	2871.782	-0.828	0.213	0.000	0.001	2871.782	0.039	0.212	0.855	0.926	2871.782	0.866	0.246	0.000	0.007
RBBP9	202.494	-0.523	0.198	0.008	0.029	202.494	0.052	0.193	0.789	0.890	202.494	0.575	0.227	0.011	0.071
RAI1	792.610	-0.293	0.120	0.015	0.045	792.610	-0.098	0.119	0.409	0.649	792.610	0.195	0.139	0.160	0.373
PHTF1	290.702	-0.063	0.207	0.762	0.842	290.702	-0.231	0.207	0.263	0.536	290.702	-0.169	0.240	0.482	0.694
MAP3K2	969.389	0.046	0.155	0.767	0.846	969.389	-0.055	0.155	0.723	0.852	969.389	-0.101	0.180	0.574	0.761
KIF1C	2435.509	-0.050	0.191	0.792	0.863	2435.509	-0.340	0.191	0.075	0.364	2435.509	-0.289	0.221	0.190	0.411
CTSC	3685.107	-0.252	0.268	0.346	0.487	3685.107	-0.481	0.268	0.073	0.362	3685.107	-0.229	0.309	0.460	0.677
GIPC1	2313.646	0.253	0.204	0.215	0.345	2313.646	-0.219	0.205	0.285	0.554	2313.646	-0.472	0.236	0.046	0.172
TRAF3IP2	1682.317	0.405	0.223	0.069	0.147	1682.317	0.452	0.222	0.042	0.330	1682.317	0.047	0.257	0.854	0.930
NUP50	1606.196	-1.001	0.248	0.000	0.001	1606.196	-0.574	0.248	0.020	0.312	1606.196	0.427	0.287	0.136	0.338
KDM5B	1460.647	-0.056	0.297	0.852	0.903	1460.647	0.416	0.297	0.160	0.452	1460.647	0.472	0.343	0.169	0.384
TOB2	1675.593	-0.770	0.187	0.000	0.000	1675.593	0.118	0.186	0.527	0.729	1675.593	0.888	0.216	0.000	0.001
HBS1L	1196.250	-0.748	0.133	0.000	0.000	1196.250	-0.155	0.132	0.242	0.518	1196.250	0.593	0.154	0.000	0.003
AHCYL1	4555.100	-0.570	0.169	0.001	0.005	4555.100	-0.084	0.169	0.621	0.791	4555.100	0.487	0.196	0.013	0.077
PLK2	404.845	0.032	0.375	0.932	0.957	404.845	-1.045	0.377	0.006	0.297	404.845	-1.077	0.435	0.013	0.079
ZMYND11	1085.450	-0.352	0.165	0.033	0.084	1085.450	-0.035	0.164	0.832	0.913	1085.450	0.317	0.191	0.096	0.273
SRSF10	1679.522	-0.656	0.264	0.013	0.041	1679.522	-0.722	0.264	0.006	0.297	1679.522	-0.066	0.305	0.829	0.916
ZBTB6	220.546	-0.313	0.158	0.048	0.112	220.546	-0.010	0.154	0.947	0.974	220.546	0.303	0.182	0.096	0.274
POP4	336.061	0.463	0.222	0.037	0.093	336.061	-0.447	0.225	0.047	0.336	336.061	-0.910	0.259	0.000	0.007
ARPP19	1530.367	0.299	0.155	0.054	0.123	1530.367	0.138	0.155	0.373	0.622	1530.367	-0.160	0.179	0.371	0.602
ZNF271P	322.654	-0.021	0.151	0.890	0.929	322.654	-0.260	0.151	0.086	0.379	322.654	-0.239	0.176	0.174	0.391
ZNF234	115.742	-0.072	0.194	0.711	0.802	115.742	0.113	0.190	0.552	0.744	115.742	0.185	0.223	0.407	0.633
ZNF266	243.370	-0.090	0.174	0.605	0.718	243.370	0.153	0.172	0.373	0.622	243.370	0.244	0.201	0.225	0.453
ZNF274	359.459	-0.297	0.196	0.128	0.235	359.459	-0.170	0.194	0.381	0.627	359.459	0.127	0.226	0.574	0.761
NEK6	1180.184	-0.340	0.161	0.034	0.087	1180.184	0.055	0.160	0.731	0.856	1180.184	0.395	0.185	0.033	0.140
WDR4	468.211	-1.271	0.522	0.015	0.046	468.211	-1.651	0.523	0.002	0.297	468.211	-0.380	0.604	0.530	0.729
NCKAP1	2182.154	-0.454	0.164	0.006	0.022	2182.154	-0.214	0.164	0.193	0.478	2182.154	0.240	0.190	0.207	0.431
IQGAP2	691.905	-0.244	0.251	0.331	0.471	691.905	-0.739	0.251	0.003	0.297	691.905	-0.495	0.290	0.088	0.259
ZNF273	33.609	0.348	0.301	0.247	0.381	33.609	0.115	0.302	0.704	0.841	33.609	-0.233	0.349	0.504	0.711
ZNF460	24.769	-1.504	0.465	0.001	0.007	24.769	-0.698	0.428	0.103	0.396	24.769	0.807	0.537	0.133	0.332
ZNF268	181.438	-0.047	0.158	0.768	0.846	181.438	-0.298	0.159	0.061	0.350	181.438	-0.252	0.185	0.175	0.392
MTHFD2	6386.396	-0.988	0.189	0.000	0.000	6386.396	-0.146	0.189	0.441	0.672	6386.396	0.842	0.218	0.000	0.003
LOC107984036	29.570	0.612	0.381	0.108	0.207	29.570	0.077	0.388	0.843	0.918	29.570	-0.535	0.443	0.227	0.455
LOC107984208	18.753	-1.363	0.544	0.012	0.040	18.753	-0.349	0.504	0.488	0.705	18.753	1.014	0.622	0.103	0.286
LINC01772	7.712	3.270	0.717	0.000	0.000	7.712	2.152	0.751	0.004	0.297	7.712	-1.119	0.707	0.113	0.301
LOC107985246	56.102	0.927	0.293	0.002	0.008	56.102	0.320	0.298	0.283	0.553	56.102	-0.607	0.339	0.073	0.231
ACBD3-AS1	135.635	-0.795	0.382	0.038	0.093	135.635	-0.124	0.377	0.742	0.862	135.635	0.671	0.441	0.128	0.324
LOC107985911	596.184	0.532	0.217	0.014	0.045	596.184	0.408	0.217	0.060	0.350	596.184	-0.124	0.250	0.621	0.792
LOC107985939	19.031	1.301	0.552	0.018	0.054	19.031	-0.374	0.589	0.526	0.728	19.031	-1.675	0.659	0.011	0.070
LINC02246	21.527	-0.614	0.580	0.290	0.428	21.527	0.621	0.534	0.245	0.520	21.527	1.235	0.651	0.058	0.201
RPP40	134.984	-0.614	0.274	0.025	0.068	134.984	-0.316	0.270	0.242	0.518	134.984	0.298	0.317	0.348	0.581
CFTR	27.458	0.415	0.400	0.300	0.438	27.458	0.695	0.390	0.075	0.364	27.458	0.280	0.453	0.537	0.735
SEPTIN9	8759.207	-0.011	0.136	0.937	0.961	8759.207	0.194	0.135	0.151	0.444	8759.207	0.205	0.156	0.190	0.412
SEC24A	1025.346	-0.194	0.324	0.549	0.672	1025.346	-0.210	0.323	0.515	0.721	1025.346	-0.017	0.374	0.965	0.984
SDCCAG8	682.834	-0.315	0.177	0.074	0.156	682.834	0.438	0.175	0.012	0.305	682.834	0.753	0.203	0.000	0.004
ENTR1	1211.014	-0.773	0.279	0.006	0.022	1211.014	-0.381	0.278	0.171	0.460	1211.014	0.392	0.322	0.224	0.451
HSPH1	1700.358	-1.237	0.489	0.011	0.038	1700.358	-1.599	0.489	0.001	0.297	1700.358	-0.361	0.565	0.522	0.724
STARD10	493.136	1.044	0.236	0.000	0.000	493.136	0.497	0.237	0.036	0.324	493.136	-0.547	0.272	0.045	0.170
WASF3	14.285	-1.544	0.520	0.003	0.013	14.285	0.034	0.428	0.937	0.968	14.285	1.578	0.578	0.006	0.048
NOXA1	90.558	1.733	0.589	0.003	0.014	90.558	1.407	0.590	0.017	0.306	90.558	-0.326	0.677	0.630	0.799
UTP14A	797.346	-0.386	0.220	0.080	0.166	797.346	-0.359	0.220	0.103	0.395	797.346	0.026	0.255	0.918	0.962

CPLX1	40.527	1.217	0.594	0.041	0.099	40.527	1.080	0.594	0.069	0.357	40.527	-0.137	0.681	0.841	0.923
FRS3	90.457	0.334	0.339	0.324	0.464	90.457	0.365	0.338	0.279	0.549	90.457	0.031	0.390	0.937	0.972
FRS2	444.360	-0.425	0.222	0.055	0.125	444.360	-0.219	0.221	0.320	0.581	444.360	0.205	0.256	0.423	0.647
DIAPH2-AS1	14.739	-0.918	0.652	0.159	0.277	14.739	0.452	0.603	0.453	0.680	14.739	1.370	0.736	0.063	0.211
NEU3	353.615	-0.771	0.228	0.001	0.005	353.615	-0.013	0.225	0.953	0.977	353.615	0.758	0.263	0.004	0.035
FAXDC2	137.955	-0.470	0.424	0.268	0.404	137.955	0.391	0.419	0.350	0.605	137.955	0.861	0.488	0.077	0.240
FAM114A2	362.292	-0.488	0.141	0.001	0.004	362.292	-0.334	0.140	0.017	0.306	362.292	0.154	0.164	0.348	0.582
ZNF275	322.316	-0.412	0.170	0.015	0.047	322.316	0.145	0.167	0.384	0.629	322.316	0.557	0.196	0.004	0.037
ALDH1L1	270.240	-0.450	0.556	0.418	0.555	270.240	0.775	0.554	0.162	0.453	270.240	1.225	0.641	0.056	0.197
TUBGCP2	1495.118	-0.379	0.130	0.004	0.015	1495.118	-0.077	0.130	0.553	0.745	1495.118	0.302	0.150	0.045	0.170
CLPX	963.916	-0.727	0.143	0.000	0.000	963.916	-0.156	0.141	0.268	0.539	963.916	0.570	0.165	0.001	0.008
SRCAP	1572.816	-0.524	0.157	0.001	0.005	1572.816	-0.291	0.157	0.063	0.350	1572.816	0.233	0.182	0.200	0.423
PPP1R13L	3472.203	0.661	0.348	0.057	0.128	3472.203	0.705	0.348	0.043	0.330	3472.203	0.044	0.402	0.912	0.960
CD3EAP	1054.227	-0.251	0.282	0.373	0.513	1054.227	-0.373	0.282	0.186	0.473	1054.227	-0.122	0.326	0.709	0.849
HPSE	256.091	-0.515	0.370	0.164	0.284	256.091	0.373	0.368	0.310	0.575	256.091	0.888	0.427	0.037	0.152
RUVBL2	4035.299	0.110	0.213	0.605	0.718	4035.299	-0.137	0.213	0.519	0.724	4035.299	-0.247	0.246	0.314	0.550
PGRMC1	3477.554	0.031	0.101	0.759	0.840	3477.554	0.005	0.101	0.959	0.979	3477.554	-0.026	0.117	0.825	0.914
LINP1	66.164	0.447	0.308	0.148	0.261	66.164	0.154	0.310	0.620	0.790	66.164	-0.293	0.357	0.412	0.637
ADAM28	161.585	0.388	0.185	0.036	0.090	161.585	0.252	0.184	0.171	0.460	161.585	-0.136	0.213	0.524	0.725
ARID5A	237.041	1.288	0.252	0.000	0.000	237.041	0.013	0.259	0.961	0.979	237.041	-1.275	0.294	0.000	0.001
HCP5	71.349	0.984	0.684	0.150	0.264	71.349	0.579	0.685	0.397	0.641	71.349	-0.405	0.788	0.607	0.783
TSPAN9	584.322	0.001	0.137	0.993	0.996	584.322	0.051	0.136	0.708	0.843	584.322	0.050	0.158	0.753	0.874
USP20	545.999	-0.066	0.131	0.614	0.726	545.999	0.005	0.131	0.971	0.984	545.999	0.071	0.152	0.640	0.805
USP19	1371.130	-0.685	0.140	0.000	0.000	1371.130	-0.031	0.138	0.821	0.906	1371.130	0.653	0.161	0.000	0.001
ME3	11.987	0.631	0.485	0.193	0.318	11.987	0.328	0.490	0.503	0.714	11.987	-0.303	0.557	0.587	0.768
NMU	60.214	1.127	0.630	0.074	0.155	60.214	0.933	0.630	0.139	0.430	60.214	-0.194	0.724	0.789	0.895
CFHR3	69.277	-0.414	0.490	0.398	0.537	69.277	0.790	0.481	0.101	0.394	69.277	1.204	0.562	0.032	0.138
LOC108783654	523.642	-0.328	0.149	0.028	0.075	523.642	-0.171	0.148	0.249	0.523	523.642	0.157	0.173	0.365	0.597
C1QL1	691.312	1.413	0.744	0.058	0.128	691.312	0.825	0.745	0.268	0.540	691.312	-0.589	0.859	0.493	0.702
MRPS30	1378.799	-0.491	0.149	0.001	0.006	1378.799	-0.194	0.149	0.192	0.477	1378.799	0.297	0.172	0.085	0.254
WDR3	1736.394	-0.928	0.151	0.000	0.000	1736.394	-0.227	0.150	0.131	0.423	1736.394	0.701	0.174	0.000	0.002
RAB10	3819.982	-0.070	0.121	0.563	0.683	3819.982	-0.112	0.121	0.354	0.608	3819.982	-0.042	0.140	0.763	0.880
ZNF8-ERVK3-1	11.246	-1.552	0.649	0.017	0.050	11.246	-1.242	0.614	0.043	0.331	11.246	0.309	0.769	0.688	0.836
PPARGC1A	22.255	1.337	1.009	0.185	0.309	22.255	0.268	1.016	0.792	0.892	22.255	-1.069	1.166	0.359	0.592
MALT1	532.420	-0.733	0.181	0.000	0.001	532.420	-0.235	0.179	0.189	0.475	532.420	0.498	0.209	0.017	0.093
MMP24	253.731	0.786	0.349	0.024	0.067	253.731	0.805	0.349	0.021	0.312	253.731	0.020	0.402	0.961	0.982
YIF1A	1386.111	0.168	0.256	0.512	0.641	1386.111	-0.271	0.256	0.290	0.558	1386.111	-0.439	0.296	0.138	0.341
CPSF4	531.100	-0.905	0.280	0.001	0.007	531.100	-0.494	0.279	0.077	0.367	531.100	0.412	0.324	0.203	0.427
JTB	180.732	0.851	0.330	0.010	0.034	180.732	0.277	0.331	0.403	0.645	180.732	-0.574	0.381	0.132	0.330
ADCY3	1384.782	-0.344	0.291	0.237	0.370	1384.782	-0.593	0.291	0.041	0.329	1384.782	-0.250	0.336	0.457	0.675
DHRS4	809.641	0.234	0.147	0.112	0.212	809.641	0.139	0.147	0.345	0.602	809.641	-0.095	0.170	0.574	0.761
BRD8	1120.040	-0.240	0.204	0.238	0.371	1120.040	0.260	0.203	0.201	0.484	1120.040	0.500	0.235	0.033	0.141
MTMR11	162.322	0.419	0.398	0.292	0.431	162.322	0.795	0.396	0.045	0.334	162.322	0.376	0.458	0.412	0.637
BLCAP	1560.230	0.232	0.086	0.007	0.026	1560.230	-0.049	0.086	0.568	0.755	1560.230	-0.281	0.100	0.005	0.040
MAN1A2	1118.196	-0.195	0.221	0.378	0.518	1118.196	-0.022	0.220	0.920	0.960	1118.196	0.172	0.255	0.499	0.706
TRAFD1	560.237	0.257	0.227	0.257	0.392	560.237	0.283	0.226	0.211	0.491	560.237	0.026	0.262	0.920	0.963
TXNL4A	1262.435	0.031	0.241	0.898	0.934	1262.435	-0.339	0.241	0.160	0.451	1262.435	-0.370	0.278	0.184	0.404
PNPLA6	2205.404	0.339	0.159	0.033	0.084	2205.404	0.318	0.159	0.046	0.334	2205.404	-0.021	0.184	0.907	0.957
SUGT1	1038.304	-0.274	0.172	0.111	0.211	1038.304	-0.191	0.171	0.264	0.536	1038.304	0.083	0.198	0.678	0.829
PAPOLA	4019.096	-0.786	0.210	0.000	0.001	4019.096	-0.447	0.209	0.033	0.322	4019.096	0.340	0.242	0.160	0.373
TCERG1	1317.675	-0.331	0.224	0.140	0.250	1317.675	-0.407	0.224	0.069	0.358	1317.675	-0.076	0.259	0.770	0.883
MAGED2	806.527	0.345	0.221	0.119	0.222	806.527	0.357	0.221	0.106	0.398	806.527	0.012	0.255	0.962	0.982
EHMT2	6583.809	0.326	0.148	0.028	0.075	6583.809	0.214	0.148	0.150	0.442	6583.809	-0.112	0.171	0.513	0.718
COPS8	824.620	-0.280	0.207	0.177	0.299	824.620	-0.538	0.207	0.009	0.297	824.620	-0.258	0.240	0.281	0.515
RNPS1	2977.226	-0.585	0.201	0.004	0.015	2977.226	-0.300	0.200	0.134	0.426	2977.226	0.285	0.232	0.219	0.446
FASTK	569.214	0.475	0.174	0.006	0.024	569.214	-0.003	0.175	0.988	0.993	569.214	-0.478	0.201	0.018	0.094
SUB1	3640.078	0.109	0.170	0.522	0.649	3640.078	-0.260	0.170	0.126	0.419	3640.078	-0.369	0.196	0.060	0.205
SMPDL3A	90.430	0.002	0.218	0.993	0.996	90.430	-0.069	0.216	0.748	0.866	90.430	-0.071	0.253	0.778	0.888
DBF4	295.906	-0.445	0.241	0.065	0.141	295.906	-0.428	0.240	0.075	0.364	295.906	0.017	0.279	0.951	0.978
SPIN1	1053.319	-0.521	0.155	0.001	0.005	1053.319	-0.103	0.154	0.502	0.714	1053.319	0.418	0.179	0.019	0.099
RALBP1	1719.693	-0.052	0.079	0.505	0.635	1719.693	-0.039	0.078	0.621	0.791	1719.693	0.014	0.091	0.880	0.943
ARHGAP27P1-BPTFP1-KPNA2P3	45.489	1.896	0.409	0.000	0.000	45.489	0.454	0.424	0.284	0.554	45.489	-1.442	0.474	0.002	0.024
SRSF8	400.891	-0.288	0.242	0.235	0.368	400.891	-0.278	0.242	0.250	0.523	400.891	0.010	0.280	0.973	0.987
MORF4L1	5005.964	-0.285	0.128	0.026	0.070	5005.964	-0.003	0.127	0.981	0.989	5005.964	0.282	0.147	0.056	0.196
PRDX3	2817.851	-0.602	0.136	0.000	0.000	2817.851	-0.210	0.136	0.122	0.413	2817.851	0.392	0.157	0.013	0.076
EHD1	1325.792	-0.271	0.116	0.019	0.056	1325.792	-0.037	0.115	0.748	0.866	1325.792	0.234	0.133	0.080	0.244
AFG3L2	2131.281	-0.626	0.187	0.001	0.005	2131.281	-0.349	0.187	0.062	0.350	2131.281	0.277	0.216	0.200	0.423

POP1	364.656	-0.647	0.331	0.051	0.117	364.656	-1.030	0.332	0.002	0.297	364.656	-0.383	0.384	0.318	0.553
MSL3	461.415	-0.253	0.107	0.018	0.054	461.415	0.039	0.105	0.708	0.843	461.415	0.292	0.123	0.018	0.095
C11orf58	3511.161	-0.663	0.148	0.000	0.000	3511.161	-0.276	0.148	0.062	0.350	3511.161	0.387	0.171	0.024	0.114
KDELR1	3439.051	0.784	0.231	0.001	0.004	3439.051	0.433	0.231	0.061	0.350	3439.051	-0.351	0.267	0.188	0.408
SF3A3	1966.539	-0.752	0.231	0.001	0.006	1966.539	-0.476	0.231	0.039	0.327	1966.539	0.276	0.267	0.300	0.535
AP3M2	342.964	-0.408	0.193	0.035	0.088	342.964	0.007	0.191	0.972	0.985	342.964	0.415	0.223	0.063	0.211
STARD3	746.074	0.223	0.152	0.141	0.252	746.074	0.155	0.151	0.306	0.572	746.074	-0.068	0.175	0.696	0.841
HNRNPA0	5477.711	-0.189	0.108	0.080	0.165	5477.711	0.100	0.108	0.354	0.608	5477.711	0.289	0.125	0.020	0.102
BTG3	773.723	0.254	0.188	0.176	0.298	773.723	-0.282	0.188	0.134	0.426	773.723	-0.536	0.217	0.014	0.080
CBX1	2134.308	-0.184	0.177	0.298	0.436	2134.308	-0.063	0.176	0.721	0.850	2134.308	0.121	0.204	0.554	0.747
SEC61B	1234.873	0.863	0.228	0.000	0.001	1234.873	-0.051	0.229	0.825	0.909	1234.873	-0.913	0.264	0.001	0.008
TOMM34	1099.381	-0.180	0.193	0.350	0.490	1099.381	-0.402	0.193	0.038	0.327	1099.381	-0.221	0.223	0.322	0.556
PDIA5	780.430	0.605	0.139	0.000	0.000	780.430	-0.122	0.141	0.385	0.631	780.430	-0.728	0.162	0.000	0.000
SERINC3	2771.148	-0.050	0.214	0.816	0.879	2771.148	0.255	0.214	0.233	0.510	2771.148	0.305	0.247	0.217	0.443
OS9	4910.249	0.339	0.289	0.241	0.374	4910.249	0.528	0.289	0.068	0.357	4910.249	0.189	0.334	0.572	0.760
PNRC1	4116.766	0.417	0.142	0.003	0.014	4116.766	0.283	0.142	0.046	0.335	4116.766	-0.134	0.164	0.413	0.639
TMED2	5411.233	-0.361	0.183	0.048	0.112	5411.233	-0.238	0.183	0.193	0.478	5411.233	0.124	0.211	0.558	0.750
LMAN2	2775.399	0.212	0.122	0.081	0.167	2775.399	0.074	0.122	0.542	0.738	2775.399	-0.138	0.141	0.326	0.561
ERP29	2033.766	0.506	0.162	0.002	0.009	2033.766	-0.003	0.162	0.987	0.992	2033.766	-0.509	0.187	0.007	0.049
SNORA21B	341.483	0.085	0.320	0.792	0.862	341.483	-0.497	0.321	0.122	0.413	341.483	-0.581	0.371	0.117	0.307
SNORD63B	34.462	0.698	0.418	0.095	0.189	34.462	0.064	0.427	0.880	0.939	34.462	-0.634	0.487	0.193	0.416
MLLT11	757.362	-0.135	0.227	0.550	0.673	757.362	-0.351	0.227	0.121	0.413	757.362	-0.216	0.262	0.410	0.636
STIP1	4485.403	-0.269	0.338	0.427	0.563	4485.403	-0.954	0.338	0.005	0.297	4485.403	-0.686	0.390	0.079	0.242
IFI44L	228.972	0.177	0.511	0.729	0.816	228.972	0.676	0.510	0.185	0.472	228.972	0.498	0.590	0.398	0.625
ACOT2	340.686	0.166	0.192	0.387	0.526	340.686	0.104	0.192	0.586	0.768	340.686	-0.062	0.222	0.781	0.890
RAB40B	175.671	0.165	0.399	0.678	0.776	175.671	0.248	0.398	0.532	0.732	175.671	0.083	0.460	0.857	0.931
EBNA1BP2	2839.332	-0.935	0.392	0.017	0.051	2839.332	-0.896	0.392	0.022	0.315	2839.332	0.040	0.453	0.930	0.968
CKAP4	1940.729	-0.089	0.301	0.767	0.846	1940.729	-0.580	0.301	0.054	0.342	1940.729	-0.491	0.348	0.158	0.370
YWHAQ	5728.427	-0.467	0.127	0.000	0.002	5728.427	-0.088	0.127	0.486	0.703	5728.427	0.379	0.147	0.010	0.065
TMED10	2677.646	-0.110	0.153	0.472	0.606	2677.646	0.070	0.152	0.648	0.808	2677.646	0.179	0.176	0.309	0.544
KRT7-AS	14199.553	0.770	0.320	0.016	0.049	14199.553	-0.193	0.320	0.547	0.741	14199.553	-0.963	0.370	0.009	0.062
LINC02236	11.638	-0.494	0.624	0.428	0.565	11.638	0.037	0.601	0.951	0.976	11.638	0.531	0.716	0.458	0.676
POC1B-AS1	11.442	0.058	0.654	0.929	0.955	11.442	-0.020	0.652	0.976	0.986	11.442	-0.078	0.758	0.918	0.962
LINC02154	381.725	3.687	0.269	0.000	0.000	381.725	0.795	0.290	0.006	0.297	381.725	-2.892	0.309	0.000	0.000
TFAP2A-AS2	142.831	0.031	0.235	0.897	0.933	142.831	0.216	0.233	0.354	0.608	142.831	0.185	0.271	0.494	0.703
MIR4713HG	26.260	-0.686	0.486	0.158	0.275	26.260	0.715	0.454	0.115	0.407	26.260	1.400	0.548	0.011	0.068
ZNF710-AS1	107.608	0.931	0.282	0.001	0.006	107.608	0.235	0.286	0.412	0.651	107.608	-0.696	0.326	0.033	0.139
DM1-AS	190.682	0.576	0.305	0.059	0.130	190.682	0.361	0.305	0.236	0.513	190.682	-0.215	0.351	0.541	0.737
ASCC3	1426.728	-1.245	0.273	0.000	0.000	1426.728	-0.539	0.272	0.048	0.336	1426.728	0.706	0.315	0.025	0.117
UQCR11	590.041	0.282	0.227	0.214	0.344	590.041	-0.044	0.227	0.848	0.922	590.041	-0.325	0.262	0.215	0.441
CLP1	229.724	-0.357	0.239	0.134	0.244	229.724	-0.169	0.236	0.475	0.697	229.724	0.188	0.276	0.495	0.703
FERMT2	2284.173	-0.845	0.263	0.001	0.007	2284.173	-0.312	0.263	0.235	0.513	2284.173	0.533	0.304	0.079	0.243
COPS6	1768.349	0.198	0.175	0.258	0.393	1768.349	-0.066	0.175	0.706	0.842	1768.349	-0.264	0.202	0.192	0.414
RAB32	1529.285	-0.261	0.209	0.212	0.341	1529.285	0.027	0.209	0.898	0.949	1529.285	0.288	0.241	0.233	0.462
MAPRE2	960.657	-0.361	0.150	0.016	0.048	960.657	-0.069	0.149	0.645	0.806	960.657	0.293	0.173	0.091	0.264
CCNI	4949.498	0.003	0.348	0.994	0.997	4949.498	0.579	0.348	0.096	0.391	4949.498	0.576	0.402	0.152	0.361
KCNQ1OT1	31.025	1.969	0.418	0.000	0.000	31.025	0.502	0.440	0.253	0.527	31.025	-1.466	0.484	0.002	0.025
GCN1	3394.136	-0.516	0.207	0.013	0.041	3394.136	-0.197	0.207	0.342	0.600	3394.136	0.319	0.240	0.183	0.401
MIR302CHG	161.284	0.513	0.250	0.041	0.099	161.284	-0.238	0.254	0.349	0.604	161.284	-0.750	0.292	0.010	0.066
RNA5-8SN4	2483.116	0.891	0.336	0.008	0.028	2483.116	0.416	0.336	0.215	0.494	2483.116	-0.475	0.387	0.220	0.447
COPS5	1231.240	-0.165	0.149	0.269	0.405	1231.240	-0.221	0.149	0.137	0.428	1231.240	-0.057	0.172	0.742	0.867
METAP2	3191.812	-0.067	0.137	0.624	0.734	3191.812	-0.062	0.137	0.652	0.810	3191.812	0.005	0.159	0.973	0.987
IMMT	4389.849	-0.605	0.105	0.000	0.000	4389.849	-0.117	0.105	0.263	0.536	4389.849	0.488	0.122	0.000	0.002
SF3B2	4820.964	-0.020	0.138	0.886	0.926	4820.964	-0.104	0.138	0.450	0.679	4820.964	-0.084	0.159	0.597	0.776
ILVBL	1416.953	0.230	0.200	0.250	0.384	1416.953	0.447	0.200	0.026	0.316	1416.953	0.216	0.231	0.349	0.583
PKD1P3-NPIPA1	2682.949	-0.571	0.270	0.035	0.088	2682.949	0.356	0.270	0.186	0.473	2682.949	0.927	0.312	0.003	0.028
SLC27A5	265.463	0.503	0.183	0.006	0.023	265.463	0.152	0.184	0.410	0.649	265.463	-0.351	0.212	0.098	0.277
SLC27A4	598.860	-0.562	0.493	0.254	0.388	598.860	-1.348	0.494	0.006	0.297	598.860	-0.786	0.571	0.169	0.384
SLC27A3	46.736	1.996	0.448	0.000	0.000	46.736	0.817	0.456	0.073	0.362	46.736	-1.178	0.514	0.022	0.108
PKD1P5-LOC105376752	1709.879	0.119	0.275	0.665	0.767	1709.879	0.454	0.275	0.099	0.394	1709.879	0.335	0.318	0.292	0.528
PKD1P4-NPIPA8	1421.110	-0.950	0.327	0.004	0.016	1421.110	0.262	0.326	0.421	0.658	1421.110	1.212	0.377	0.001	0.016
PPIP5K1P1-CATSPER2	297.463	-0.709	0.318	0.026	0.070	297.463	0.199	0.315	0.528	0.729	297.463	0.908	0.366	0.013	0.078
SLC27A2	232.206	-0.538	0.176	0.002	0.010	232.206	-0.185	0.172	0.284	0.554	232.206	0.354	0.203	0.081	0.247
KIF2C	1307.541	-0.315	0.231	0.173	0.294	1307.541	0.236	0.231	0.306	0.571	1307.541	0.551	0.267	0.039	0.155
SPINK5	20.497	0.642	0.524	0.221	0.351	20.497	0.309	0.527	0.557	0.747	20.497	-0.333	0.604	0.582	0.765
CCDC85B	1698.076	0.275	0.232	0.236	0.369	1698.076	0.008	0.233	0.972	0.985	1698.076	-0.267	0.268	0.320	0.555
GLIPR1	1227.109	-0.618	0.200	0.002	0.010	1227.109	-0.470	0.200	0.019	0.306	1227.109	0.147	0.231	0.525	0.726

TLK2	768.794	-0.814	0.203	0.000	0.001	768.794	-0.388	0.202	0.055	0.342	768.794	0.426	0.235	0.069	0.225
KDELR2	3933.079	0.123	0.144	0.394	0.532	3933.079	-0.163	0.144	0.256	0.529	3933.079	-0.286	0.166	0.085	0.254
KDELR3	308.938	1.346	0.228	0.000	0.000	308.938	0.086	0.234	0.713	0.846	308.938	-1.260	0.265	0.000	0.000
ATF7	557.582	0.064	0.228	0.780	0.854	557.582	0.242	0.228	0.287	0.555	557.582	0.179	0.263	0.497	0.705
SNRNP27	361.648	0.054	0.161	0.735	0.821	361.648	-0.145	0.161	0.370	0.620	361.648	-0.199	0.187	0.287	0.522
TMED1	631.257	0.473	0.157	0.003	0.012	631.257	0.147	0.158	0.353	0.607	631.257	-0.326	0.182	0.073	0.231
LIAS	260.265	-0.854	0.145	0.000	0.000	260.265	-0.207	0.139	0.136	0.427	260.265	0.647	0.167	0.000	0.003
RCBTB2	110.230	-0.328	0.406	0.420	0.557	110.230	0.341	0.402	0.395	0.640	110.230	0.669	0.468	0.152	0.361
IFT27	57.638	0.945	0.240	0.000	0.001	57.638	0.147	0.251	0.558	0.748	57.638	-0.798	0.281	0.004	0.038
RAB35	1192.263	-0.669	0.262	0.011	0.036	1192.263	-0.484	0.262	0.064	0.353	1192.263	0.185	0.303	0.541	0.738
TDRKH	41.272	1.483	0.337	0.000	0.000	41.272	-0.028	0.356	0.937	0.968	41.272	-1.511	0.397	0.000	0.003
LILRB3	7.700	1.278	0.998	0.200	0.327	7.700	-1.911	1.161	0.100	0.394	7.700	-3.189	1.285	0.013	0.078
RBPMS	251.970	0.746	0.177	0.000	0.000	251.970	0.152	0.179	0.397	0.641	251.970	-0.594	0.205	0.004	0.034
RAB31	1557.508	0.038	0.211	0.856	0.906	1557.508	-0.198	0.211	0.347	0.603	1557.508	-0.236	0.243	0.332	0.567
ADAP1	154.373	1.806	0.276	0.000	0.000	154.373	0.339	0.287	0.238	0.515	154.373	-1.467	0.320	0.000	0.000
DSTN	2838.508	0.098	0.108	0.365	0.505	2838.508	-0.208	0.108	0.055	0.342	2838.508	-0.305	0.125	0.014	0.083
STON1	49.000	0.468	0.607	0.441	0.576	49.000	1.028	0.603	0.088	0.381	49.000	0.560	0.697	0.422	0.646
GUSBP14	263.254	-0.135	0.363	0.711	0.802	263.254	0.276	0.362	0.446	0.677	263.254	0.411	0.419	0.327	0.562
RCC1	3889.884	-0.352	0.133	0.008	0.029	3889.884	-0.078	0.133	0.557	0.747	3889.884	0.274	0.154	0.075	0.235
PIM2	422.739	0.423	0.276	0.125	0.231	422.739	0.313	0.276	0.257	0.530	422.739	-0.111	0.319	0.729	0.859
B4GAT1	652.158	0.497	0.352	0.158	0.276	652.158	0.633	0.352	0.072	0.361	652.158	0.136	0.406	0.738	0.865
TENT4A	1295.233	-0.340	0.199	0.087	0.176	1295.233	0.123	0.198	0.535	0.734	1295.233	0.463	0.229	0.043	0.167
ADRM1	1871.650	0.093	0.393	0.812	0.877	1871.650	-0.835	0.394	0.034	0.322	1871.650	-0.928	0.455	0.041	0.162
CHD1	1004.212	-0.965	0.275	0.000	0.003	1004.212	-0.649	0.274	0.018	0.306	1004.212	0.316	0.317	0.320	0.555
NUDT21	2962.464	-0.600	0.138	0.000	0.000	2962.464	-0.140	0.138	0.312	0.575	2962.464	0.460	0.160	0.004	0.035
CPSF6	1213.475	0.047	0.117	0.689	0.785	1213.475	-0.382	0.118	0.001	0.297	1213.475	-0.429	0.136	0.002	0.019
OGFR	1841.684	0.577	0.157	0.000	0.002	1841.684	0.227	0.157	0.150	0.442	1841.684	-0.350	0.182	0.054	0.193
DDX52	728.601	-0.424	0.273	0.120	0.224	728.601	-0.653	0.273	0.017	0.306	728.601	-0.229	0.315	0.468	0.684
ABHD2	1952.054	-0.554	0.291	0.057	0.128	1952.054	0.024	0.291	0.934	0.967	1952.054	0.578	0.336	0.086	0.255
WWP1	768.515	-0.337	0.159	0.034	0.087	768.515	-0.216	0.158	0.172	0.460	768.515	0.120	0.184	0.513	0.718
EEF1AKMT4	160.687	-0.047	0.387	0.903	0.938	160.687	-0.816	0.390	0.037	0.324	160.687	-0.769	0.450	0.088	0.259
CHD2	1871.921	-0.213	0.167	0.201	0.328	1871.921	-0.077	0.167	0.643	0.805	1871.921	0.136	0.193	0.481	0.694
WWP2	621.551	-0.399	0.162	0.014	0.043	621.551	-0.129	0.161	0.423	0.660	621.551	0.270	0.187	0.148	0.356
DUS4L	135.049	-0.427	0.204	0.037	0.091	135.049	0.116	0.197	0.558	0.748	135.049	0.542	0.234	0.020	0.103
CNTRL	421.801	-0.738	0.174	0.000	0.000	421.801	-0.040	0.171	0.817	0.904	421.801	0.698	0.200	0.000	0.008
UBE2C	898.171	0.319	0.260	0.220	0.350	898.171	0.177	0.260	0.495	0.709	898.171	-0.141	0.300	0.637	0.803
SNRNP35	414.019	0.778	0.226	0.001	0.004	414.019	-0.062	0.228	0.786	0.888	414.019	-0.840	0.262	0.001	0.017
DEPP1	1367.050	1.875	1.120	0.094	0.188	1367.050	1.207	1.121	0.281	0.551	1367.050	-0.668	1.294	0.606	0.782
CYB561D2	454.989	0.302	0.166	0.069	0.147	454.989	-0.121	0.167	0.469	0.692	454.989	-0.423	0.193	0.028	0.126
RAPGEF4	9.711	1.044	0.857	0.223	0.354	9.711	0.429	0.869	0.621	0.791	9.711	-0.615	0.988	0.534	0.732
CHD3	3412.979	-0.365	0.224	0.104	0.201	3412.979	0.271	0.224	0.225	0.504	3412.979	0.636	0.259	0.014	0.081
TMEM115	881.864	0.366	0.142	0.010	0.034	881.864	0.069	0.143	0.626	0.795	881.864	-0.297	0.165	0.072	0.229
DUSP14	1629.684	0.075	0.179	0.676	0.775	1629.684	-0.137	0.179	0.445	0.676	1629.684	-0.212	0.207	0.307	0.542
TOPBP1	966.833	-0.952	0.250	0.000	0.001	966.833	-0.540	0.249	0.030	0.322	966.833	0.412	0.288	0.153	0.363
TRIM31	21.663	0.456	0.662	0.491	0.624	21.663	-0.434	0.675	0.520	0.724	21.663	-0.890	0.775	0.251	0.483
TPPP	100.994	-0.194	0.564	0.730	0.817	100.994	0.617	0.560	0.271	0.542	100.994	0.811	0.649	0.212	0.437
HSF2BP	10.960	0.788	0.465	0.090	0.181	10.960	0.793	0.457	0.083	0.374	10.960	0.005	0.517	0.993	0.996
TRIOBP	2113.967	0.085	0.184	0.645	0.751	2113.967	0.239	0.184	0.192	0.477	2113.967	0.155	0.212	0.466	0.681
RER1	2011.926	-0.055	0.143	0.702	0.795	2011.926	-0.145	0.143	0.310	0.574	2011.926	-0.090	0.165	0.584	0.766
CHD4	5821.146	-0.162	0.106	0.126	0.232	5821.146	-0.162	0.106	0.127	0.419	5821.146	0.000	0.123	0.998	0.998
DNAJB4	377.414	-0.238	0.270	0.379	0.519	377.414	0.204	0.269	0.448	0.677	377.414	0.442	0.312	0.156	0.368
BTG3-AS1	178.069	0.087	0.193	0.651	0.756	178.069	0.057	0.192	0.768	0.877	178.069	-0.031	0.223	0.890	0.947
SLC5A4-AS1	17.479	1.015	0.637	0.111	0.211	17.479	0.397	0.644	0.537	0.735	17.479	-0.618	0.735	0.401	0.628
LINC02561	10.703	0.246	0.612	0.687	0.784	10.703	-1.094	0.685	0.110	0.403	10.703	-1.340	0.773	0.083	0.250
C17orf113	32.787	0.341	0.705	0.629	0.738	32.787	0.982	0.698	0.160	0.451	32.787	0.641	0.808	0.428	0.651
DIDO1	546.413	-0.286	0.161	0.075	0.157	546.413	-0.118	0.160	0.459	0.685	546.413	0.168	0.186	0.365	0.597
WDR5	1740.113	-0.317	0.169	0.060	0.133	1740.113	-0.266	0.168	0.114	0.406	1740.113	0.051	0.195	0.792	0.896
SPACA9	108.917	0.456	0.530	0.390	0.529	108.917	0.661	0.529	0.212	0.492	108.917	0.204	0.611	0.738	0.866
ADAMTS13	28.878	0.861	0.445	0.053	0.120	28.878	0.972	0.441	0.027	0.321	28.878	0.111	0.505	0.826	0.914
CACFD1	40.589	1.801	0.379	0.000	0.000	40.589	1.094	0.387	0.005	0.297	40.589	-0.707	0.427	0.098	0.277
NUP42	299.325	-0.287	0.191	0.133	0.242	299.325	-0.161	0.190	0.397	0.641	299.325	0.126	0.221	0.568	0.758
PRSS23	557.507	1.223	0.168	0.000	0.000	557.507	-0.040	0.171	0.814	0.903	557.507	-1.263	0.196	0.000	0.000
PTPN21	315.828	-0.674	0.223	0.002	0.011	315.828	-0.211	0.220	0.338	0.596	315.828	0.463	0.257	0.071	0.228
HNRNPUL1	6664.864	-0.344	0.087	0.000	0.001	6664.864	-0.058	0.087	0.504	0.715	6664.864	0.286	0.101	0.004	0.038
ATE1	441.412	-0.852	0.285	0.003	0.013	441.412	-0.624	0.284	0.028	0.321	441.412	0.228	0.330	0.490	0.700
RPP14	238.419	-0.081	0.172	0.638	0.745	238.419	-0.157	0.171	0.358	0.611	238.419	-0.076	0.199	0.702	0.845
KRR1	1258.711	-0.609	0.150	0.000	0.001	1258.711	-0.392	0.149	0.009	0.297	1258.711	0.216	0.173	0.212	0.437

KATNA1	552.292	-0.820	0.229	0.000	0.002	552.292	-0.356	0.227	0.117	0.409	552.292	0.464	0.264	0.079	0.242
TPM1-AS	29.345	-0.162	0.291	0.577	0.695	29.345	0.082	0.278	0.767	0.877	29.345	0.244	0.333	0.464	0.680
PRDM5	19.633	0.080	0.440	0.856	0.906	19.633	0.153	0.434	0.725	0.853	19.633	0.072	0.507	0.886	0.945
PRDM4	759.145	-0.463	0.207	0.025	0.069	759.145	-0.363	0.207	0.079	0.370	759.145	0.100	0.239	0.676	0.828
LINC02569	12.297	-1.035	1.128	0.359	0.499	12.297	-0.582	1.114	0.601	0.779	12.297	0.453	1.305	0.728	0.859
MMP24-AS1-EDEM2	27.735	0.587	0.498	0.238	0.371	27.735	0.723	0.492	0.142	0.434	27.735	0.135	0.567	0.811	0.906
CHEK1	560.905	-1.015	0.178	0.000	0.000	560.905	-0.216	0.175	0.218	0.497	560.905	0.799	0.205	0.000	0.002
HIBADH	1301.133	-0.588	0.110	0.000	0.000	1301.133	-0.046	0.109	0.670	0.821	1301.133	0.542	0.127	0.000	0.001
CIT	1133.801	-0.398	0.288	0.168	0.288	1133.801	0.276	0.288	0.336	0.595	1133.801	0.674	0.333	0.043	0.165
FGFR1OP	210.967	-0.094	0.153	0.539	0.664	210.967	0.110	0.150	0.463	0.689	210.967	0.205	0.177	0.247	0.478
EMILIN1	7.796	0.528	0.601	0.379	0.519	7.796	-0.298	0.652	0.647	0.808	7.796	-0.827	0.732	0.259	0.494
BTN3A2	1003.315	0.550	0.286	0.055	0.123	1003.315	0.443	0.286	0.121	0.413	1003.315	-0.107	0.330	0.747	0.870
BTN3A1	354.040	0.011	0.388	0.978	0.986	354.040	0.476	0.387	0.219	0.498	354.040	0.465	0.447	0.299	0.534
FOXN3	1094.077	-0.796	0.258	0.002	0.010	1094.077	0.053	0.257	0.837	0.916	1094.077	0.849	0.298	0.004	0.037
BTN2A1	472.838	-0.006	0.236	0.981	0.988	472.838	0.121	0.235	0.606	0.782	472.838	0.127	0.272	0.641	0.805
RCAN3	313.209	0.365	0.215	0.089	0.179	313.209	0.000	0.215	0.999	1.000	313.209	-0.365	0.248	0.142	0.347
FAF1	1145.770	-0.616	0.152	0.000	0.001	1145.770	-0.189	0.151	0.211	0.491	1145.770	0.427	0.176	0.015	0.086
KIF3A	157.183	-0.035	0.207	0.864	0.911	157.183	-0.379	0.208	0.068	0.357	157.183	-0.344	0.241	0.154	0.364
POLR3A	629.882	-0.635	0.217	0.004	0.015	629.882	-0.380	0.216	0.079	0.370	629.882	0.255	0.251	0.311	0.546
CLASRP	935.269	0.202	0.198	0.306	0.445	935.269	0.047	0.198	0.812	0.901	935.269	-0.155	0.228	0.496	0.704
ZWINT	888.235	-0.683	0.315	0.030	0.080	888.235	-0.553	0.315	0.079	0.370	888.235	0.129	0.365	0.723	0.856
CAPN10	223.687	0.680	0.227	0.003	0.012	223.687	-0.102	0.230	0.656	0.813	223.687	-0.783	0.264	0.003	0.029
KPTN	134.963	-0.050	0.199	0.802	0.870	134.963	-0.270	0.199	0.175	0.464	134.963	-0.220	0.232	0.342	0.576
CDC42EP1	2380.800	-0.781	0.319	0.015	0.045	2380.800	-0.504	0.319	0.114	0.406	2380.800	0.277	0.369	0.453	0.672
PWP1	1242.735	-0.640	0.150	0.000	0.000	1242.735	-0.303	0.149	0.042	0.330	1242.735	0.337	0.173	0.052	0.186
TBC1D8	944.179	-0.522	0.192	0.007	0.025	944.179	0.194	0.191	0.310	0.575	944.179	0.716	0.222	0.001	0.016
CDC37	4193.404	0.218	0.214	0.307	0.446	4193.404	-0.266	0.214	0.213	0.493	4193.404	-0.484	0.247	0.050	0.182
PKIG	483.320	0.763	0.198	0.000	0.001	483.320	0.387	0.198	0.051	0.339	483.320	-0.377	0.228	0.099	0.279
KAT7	762.150	-0.527	0.135	0.000	0.001	762.150	-0.010	0.133	0.938	0.968	762.150	0.517	0.156	0.001	0.012
PLAAT3	373.473	0.850	0.191	0.000	0.000	373.473	0.338	0.192	0.079	0.370	373.473	-0.511	0.221	0.021	0.103
GLMN	139.354	-0.237	0.273	0.384	0.523	139.354	-0.522	0.273	0.056	0.344	139.354	-0.285	0.317	0.369	0.601
HHLA3	116.996	1.279	0.456	0.005	0.020	116.996	0.688	0.457	0.132	0.424	116.996	-0.590	0.525	0.261	0.495
BVES	23.757	1.080	0.390	0.006	0.022	23.757	-0.288	0.421	0.494	0.709	23.757	-1.368	0.469	0.004	0.032
CORO1A	29.503	0.182	0.326	0.577	0.695	29.503	0.088	0.324	0.787	0.889	29.503	-0.094	0.377	0.803	0.901
WDR45	294.494	0.255	0.171	0.136	0.246	294.494	0.008	0.171	0.964	0.981	294.494	-0.247	0.198	0.212	0.437
FICD	117.371	-0.353	0.315	0.263	0.398	117.371	-0.318	0.312	0.308	0.573	117.371	0.034	0.366	0.925	0.965
AP4S1	193.793	-0.649	0.190	0.001	0.004	193.793	-0.205	0.185	0.268	0.539	193.793	0.444	0.219	0.042	0.165
PTP4A3	58.499	0.303	0.472	0.521	0.649	58.499	0.898	0.467	0.054	0.342	58.499	0.595	0.541	0.271	0.506
LSM6	300.787	-0.225	0.186	0.227	0.359	300.787	-0.200	0.185	0.281	0.551	300.787	0.025	0.216	0.906	0.957
RABL2B	146.469	-0.005	0.193	0.977	0.986	146.469	-0.065	0.192	0.734	0.857	146.469	-0.060	0.224	0.789	0.895
RABL2A	75.248	0.530	0.257	0.039	0.096	75.248	0.627	0.254	0.014	0.306	75.248	0.097	0.293	0.741	0.867
CHI3L1	91.059	3.473	0.492	0.000	0.000	91.059	0.352	0.546	0.519	0.724	91.059	-3.120	0.580	0.000	0.000
ERLIN2	807.514	-0.427	0.183	0.019	0.056	807.514	-0.009	0.182	0.963	0.980	807.514	0.419	0.211	0.047	0.176
ERG28	594.022	2.080	0.216	0.000	0.000	594.022	-0.316	0.220	0.152	0.444	594.022	-2.396	0.251	0.000	0.000
NUDT6	222.734	-0.170	0.237	0.473	0.607	222.734	-0.549	0.238	0.021	0.313	222.734	-0.379	0.275	0.169	0.384
NUDT4	2166.629	-0.978	0.093	0.000	0.000	2166.629	-0.095	0.091	0.298	0.565	2166.629	0.883	0.107	0.000	0.000
NUDT5	1043.708	0.114	0.245	0.641	0.748	1043.708	-0.486	0.246	0.048	0.337	1043.708	-0.600	0.284	0.034	0.144
NUDT3	1390.702	-0.085	0.139	0.539	0.664	1390.702	0.076	0.138	0.583	0.767	1390.702	0.161	0.160	0.315	0.550
FSTL1	3941.457	0.542	0.191	0.005	0.018	3941.457	-0.048	0.191	0.800	0.896	3941.457	-0.590	0.220	0.007	0.054
PSIP1	1508.414	-0.308	0.170	0.070	0.149	1508.414	-0.012	0.169	0.944	0.972	1508.414	0.296	0.196	0.131	0.328
WDHD1	489.716	-1.472	0.377	0.000	0.001	489.716	-0.857	0.375	0.022	0.315	489.716	0.615	0.436	0.158	0.370
CHI3L2	25.199	1.443	0.584	0.013	0.043	25.199	0.738	0.594	0.214	0.493	25.199	-0.705	0.669	0.292	0.527
STRAP	3345.836	-0.350	0.212	0.099	0.194	3345.836	-0.409	0.212	0.054	0.342	3345.836	-0.059	0.245	0.810	0.905
ADAMTS7	192.885	1.238	0.168	0.000	0.000	192.885	0.300	0.172	0.081	0.372	192.885	-0.937	0.194	0.000	0.000
ADAMTS6	67.714	-1.512	0.569	0.008	0.028	67.714	-1.255	0.564	0.026	0.318	67.714	0.258	0.660	0.697	0.841
BAZ2A	2007.135	-0.019	0.145	0.897	0.934	2007.135	0.057	0.145	0.696	0.837	2007.135	0.075	0.168	0.653	0.813
BAZ1A	1143.238	-0.116	0.255	0.650	0.755	1143.238	-0.528	0.255	0.038	0.327	1143.238	-0.412	0.294	0.162	0.375
ZNF277	423.180	0.247	0.228	0.278	0.415	423.180	0.263	0.228	0.247	0.522	423.180	0.016	0.263	0.952	0.978
WDR6	1913.001	0.076	0.176	0.665	0.766	1913.001	0.273	0.176	0.122	0.413	1913.001	0.196	0.203	0.335	0.569
TREH	196.389	1.547	0.433	0.000	0.003	196.389	0.506	0.435	0.244	0.519	196.389	-1.041	0.500	0.037	0.152
SLC2A6	283.111	1.534	0.298	0.000	0.000	283.111	-0.515	0.305	0.092	0.386	283.111	-2.049	0.349	0.000	0.000
MAP4K5	402.128	-0.801	0.245	0.001	0.006	402.128	-0.435	0.243	0.073	0.362	402.128	0.365	0.283	0.197	0.419
RASSF1	646.337	0.140	0.237	0.553	0.675	646.337	0.100	0.236	0.673	0.823	646.337	-0.040	0.273	0.882	0.943
PKP3	1576.793	0.396	0.199	0.047	0.110	1576.793	0.524	0.199	0.008	0.297	1576.793	0.128	0.230	0.577	0.762
NISCH	801.385	0.366	0.249	0.141	0.253	801.385	0.290	0.249	0.244	0.519	801.385	-0.076	0.287	0.790	0.896
CHKA	201.767	0.810	0.191	0.000	0.000	201.767	-0.069	0.196	0.726	0.853	201.767	-0.879	0.223	0.000	0.002
CEP250	640.332	0.404	0.258	0.117	0.220	640.332	0.259	0.258	0.314	0.577	640.332	-0.145	0.298	0.627	0.796

PTENP1	18.660	0.123	0.393	0.755	0.837	18.660	0.751	0.359	0.037	0.324	18.660	0.628	0.432	0.146	0.353
WBP4	314.234	0.376	0.215	0.081	0.167	314.234	-0.266	0.217	0.221	0.500	314.234	-0.642	0.250	0.010	0.067
ABCB8	265.097	-0.079	0.326	0.810	0.875	265.097	-0.629	0.327	0.054	0.342	265.097	-0.551	0.378	0.145	0.351
SEC23IP	864.026	-0.753	0.286	0.008	0.030	864.026	-0.613	0.285	0.032	0.322	864.026	0.140	0.330	0.670	0.825
SUPT16H	3488.037	-0.961	0.260	0.000	0.002	3488.037	-0.619	0.260	0.017	0.306	3488.037	0.343	0.300	0.254	0.486
ADCY6	675.752	0.213	0.261	0.414	0.552	675.752	0.341	0.260	0.190	0.475	675.752	0.129	0.301	0.669	0.824
CHKB	11.278	1.941	0.616	0.002	0.008	11.278	0.591	0.659	0.370	0.619	11.278	-1.350	0.709	0.057	0.199
CHEK2	762.782	0.234	0.137	0.088	0.177	762.782	-0.061	0.137	0.659	0.814	762.782	-0.295	0.159	0.063	0.212
POLI	228.372	-0.124	0.235	0.598	0.711	228.372	0.308	0.232	0.184	0.471	228.372	0.432	0.270	0.109	0.295
MST1P2	24.491	1.482	0.676	0.028	0.075	24.491	1.271	0.677	0.060	0.350	24.491	-0.212	0.772	0.784	0.892
CHM	341.744	-0.769	0.335	0.022	0.062	341.744	-0.152	0.333	0.648	0.808	341.744	0.617	0.387	0.111	0.298
PLPBP	753.299	0.091	0.134	0.495	0.627	753.299	-0.058	0.134	0.665	0.818	753.299	-0.150	0.155	0.335	0.570
AKAP13	1431.954	-0.293	0.176	0.096	0.190	1431.954	0.030	0.176	0.865	0.931	1431.954	0.323	0.203	0.112	0.300
AKAP11	896.234	-0.654	0.269	0.015	0.047	896.234	-0.078	0.268	0.772	0.879	896.234	0.576	0.311	0.064	0.213
AKAP10	299.427	-0.413	0.162	0.011	0.036	299.427	0.133	0.158	0.399	0.642	299.427	0.546	0.186	0.003	0.031
DDX20	401.947	-0.789	0.285	0.006	0.022	401.947	-0.653	0.284	0.022	0.314	401.947	0.136	0.330	0.680	0.831
DUSP10	206.922	0.446	0.233	0.055	0.125	206.922	0.362	0.232	0.119	0.411	206.922	-0.084	0.268	0.753	0.874
MRPL3	4035.336	-0.748	0.241	0.002	0.009	4035.336	-0.549	0.241	0.022	0.315	4035.336	0.199	0.278	0.473	0.687
MST1L	9.149	1.689	0.877	0.054	0.123	9.149	1.203	0.883	0.173	0.463	9.149	-0.486	0.995	0.625	0.795
RPL35	23943.045	0.685	0.278	0.014	0.043	23943.045	0.569	0.278	0.041	0.328	23943.045	-0.116	0.321	0.718	0.854
GALNT6	32.107	0.567	0.291	0.051	0.117	32.107	0.351	0.291	0.228	0.506	32.107	-0.215	0.333	0.518	0.721
SBNO1-AS1	13.182	0.264	0.595	0.657	0.760	13.182	0.550	0.579	0.342	0.599	13.182	0.286	0.676	0.672	0.826
LOC112268124	171.882	-0.293	0.189	0.121	0.225	171.882	-0.101	0.186	0.586	0.768	171.882	0.192	0.218	0.379	0.610
LOC112268354	10.082	0.677	0.680	0.320	0.460	10.082	0.931	0.664	0.161	0.452	10.082	0.254	0.762	0.739	0.866
GALNT5	711.127	-0.174	0.320	0.586	0.702	711.127	0.173	0.319	0.587	0.769	711.127	0.348	0.369	0.346	0.580
RASSF8	678.678	-0.110	0.327	0.736	0.822	678.678	-0.714	0.328	0.029	0.322	678.678	-0.604	0.379	0.110	0.296
CHN1	1105.639	-0.310	0.389	0.426	0.563	1105.639	0.440	0.389	0.258	0.530	1105.639	0.749	0.449	0.095	0.272
PRAF2	520.240	1.206	0.264	0.000	0.000	520.240	0.434	0.265	0.102	0.394	520.240	-0.772	0.305	0.011	0.071
SEC63	3426.775	-0.453	0.150	0.002	0.011	3426.775	0.030	0.149	0.843	0.918	3426.775	0.483	0.173	0.005	0.042
POLG2	231.508	-0.494	0.131	0.000	0.001	231.508	0.051	0.124	0.679	0.827	231.508	0.546	0.149	0.000	0.005
HPS5	561.411	-0.337	0.198	0.089	0.180	561.411	-0.380	0.198	0.055	0.343	561.411	-0.043	0.230	0.853	0.930
PDCD10	1490.249	-0.227	0.212	0.285	0.423	1490.249	-0.351	0.212	0.097	0.392	1490.249	-0.125	0.245	0.611	0.785
RNF139	676.105	-0.524	0.104	0.000	0.000	676.105	-0.066	0.101	0.513	0.720	676.105	0.458	0.119	0.000	0.003
RNF24	592.316	0.566	0.212	0.007	0.027	592.316	0.396	0.212	0.061	0.350	592.316	-0.170	0.244	0.485	0.697
CA5B	116.843	-0.933	0.338	0.006	0.022	116.843	0.587	0.323	0.069	0.357	116.843	1.520	0.385	0.000	0.002
EGLN2	161.696	-0.027	0.231	0.908	0.941	161.696	0.027	0.230	0.908	0.954	161.696	0.053	0.267	0.842	0.923
EGLN3	4597.949	0.875	0.768	0.255	0.389	4597.949	0.689	0.768	0.369	0.619	4597.949	-0.185	0.887	0.834	0.919
PMF1	137.369	0.670	0.266	0.012	0.039	137.369	0.218	0.268	0.416	0.654	137.369	-0.452	0.307	0.142	0.347
ZHX1	1120.481	-0.513	0.125	0.000	0.000	1120.481	-0.125	0.124	0.315	0.577	1120.481	0.388	0.145	0.007	0.053
SHLD3	75.854	-0.392	0.252	0.120	0.223	75.854	-0.262	0.247	0.289	0.557	75.854	0.130	0.292	0.657	0.817
GPR176	639.285	0.864	0.137	0.000	0.000	639.285	0.050	0.139	0.718	0.849	639.285	-0.814	0.159	0.000	0.000
CAVIN3	639.675	1.248	0.388	0.001	0.007	639.675	0.560	0.388	0.149	0.442	639.675	-0.688	0.448	0.124	0.318
NXPH4	472.400	0.715	0.224	0.001	0.008	472.400	0.573	0.224	0.010	0.299	472.400	-0.142	0.258	0.582	0.765
PRRT2	16.409	1.181	0.751	0.116	0.218	16.409	0.482	0.759	0.526	0.728	16.409	-0.700	0.865	0.419	0.643
ERI2	330.584	-0.869	0.172	0.000	0.000	330.584	-0.168	0.167	0.314	0.577	330.584	0.700	0.198	0.000	0.007
SAT2	329.340	1.432	0.343	0.000	0.000	329.340	0.726	0.344	0.035	0.322	329.340	-0.706	0.395	0.074	0.233
DTD2	289.658	-0.512	0.172	0.003	0.013	289.658	-0.327	0.170	0.054	0.342	289.658	0.186	0.199	0.351	0.584
GTF3C6	573.410	0.390	0.317	0.219	0.349	573.410	-0.438	0.318	0.168	0.457	573.410	-0.827	0.367	0.024	0.114
PTGDR2	61.655	0.208	0.489	0.671	0.771	61.655	-0.647	0.497	0.193	0.478	61.655	-0.855	0.572	0.135	0.336
PACSIN2	2020.211	-0.761	0.142	0.000	0.000	2020.211	-0.159	0.141	0.260	0.533	2020.211	0.602	0.164	0.000	0.005
MAN1B1	1361.603	0.236	0.217	0.277	0.415	1361.603	0.411	0.217	0.058	0.348	1361.603	0.175	0.251	0.485	0.697
TP53TG1	118.189	0.614	0.468	0.190	0.314	118.189	0.632	0.468	0.177	0.466	118.189	0.018	0.540	0.974	0.987
SNX18	551.322	-0.811	0.240	0.001	0.005	551.322	-0.622	0.239	0.009	0.297	551.322	0.189	0.278	0.497	0.705
DCTN3	1050.360	0.237	0.219	0.280	0.418	1050.360	0.386	0.219	0.078	0.369	1050.360	0.149	0.253	0.556	0.749
CYTOR	1444.967	0.470	0.203	0.021	0.059	1444.967	0.378	0.203	0.063	0.350	1444.967	-0.092	0.235	0.694	0.840
XPOT	6561.655	-1.036	0.192	0.000	0.000	6561.655	-0.023	0.192	0.905	0.952	6561.655	1.014	0.222	0.000	0.000
CHP1	2198.980	0.421	0.194	0.030	0.079	2198.980	0.222	0.194	0.254	0.527	2198.980	-0.199	0.224	0.375	0.607
RWDD2A	112.777	-0.089	0.279	0.749	0.832	112.777	0.198	0.275	0.470	0.692	112.777	0.287	0.321	0.370	0.602
CMTM7	375.794	0.289	0.177	0.103	0.199	375.794	0.022	0.178	0.902	0.951	375.794	-0.267	0.205	0.192	0.415
SP140	16.064	-0.132	0.843	0.875	0.919	16.064	0.650	0.825	0.431	0.665	16.064	0.783	0.964	0.417	0.642
SREBF2-AS1	264.426	-0.118	0.396	0.766	0.845	264.426	-0.129	0.395	0.744	0.863	264.426	-0.011	0.457	0.980	0.990
PXMP4	166.671	0.261	0.319	0.412	0.550	166.671	0.320	0.318	0.314	0.577	166.671	0.059	0.368	0.873	0.939
DUSP12	292.305	-0.902	0.169	0.000	0.000	292.305	-0.220	0.163	0.176	0.465	292.305	0.683	0.194	0.000	0.007
SNF8	880.418	0.179	0.191	0.347	0.487	880.418	-0.133	0.191	0.487	0.704	880.418	-0.312	0.221	0.157	0.369
DDX19B	524.601	-0.022	0.205	0.915	0.946	524.601	-0.389	0.206	0.059	0.348	524.601	-0.368	0.238	0.122	0.314
LOC112694756	96.208	-0.254	0.211	0.228	0.359	96.208	0.190	0.202	0.348	0.604	96.208	0.444	0.241	0.065	0.216
NRM	2343.196	0.592	0.272	0.029	0.077	2343.196	0.310	0.272	0.254	0.528	2343.196	-0.282	0.314	0.368	0.601

FAM71E1	112.937	0.086	0.185	0.642	0.749	112.937	-0.037	0.184	0.843	0.918	112.937	-0.122	0.215	0.569	0.758
RDH13	1481.342	1.012	0.181	0.000	0.000	1481.342	0.096	0.181	0.597	0.776	1481.342	-0.916	0.209	0.000	0.000
ATXN2L	4537.278	-0.069	0.117	0.555	0.677	4537.278	0.222	0.116	0.057	0.345	4537.278	0.290	0.134	0.031	0.134
USP18	96.339	1.264	0.235	0.000	0.000	96.339	0.113	0.244	0.643	0.805	96.339	-1.151	0.275	0.000	0.001
KLHL2	544.712	-0.997	0.141	0.000	0.000	544.712	-0.047	0.137	0.729	0.855	544.712	0.950	0.162	0.000	0.000
IFT43	303.148	0.664	0.200	0.001	0.005	303.148	0.270	0.201	0.179	0.467	303.148	-0.394	0.231	0.089	0.261
STX1B	10.818	1.435	0.489	0.003	0.015	10.818	0.794	0.504	0.115	0.406	10.818	-0.640	0.553	0.246	0.478
SYNRG	926.394	-0.285	0.207	0.169	0.289	926.394	0.094	0.206	0.648	0.808	926.394	0.379	0.239	0.113	0.301
GLMP	252.407	1.012	0.191	0.000	0.000	252.407	0.314	0.193	0.104	0.397	252.407	-0.698	0.220	0.002	0.018
KLF12	184.717	-0.061	0.326	0.851	0.903	184.717	-0.020	0.325	0.951	0.976	184.717	0.041	0.377	0.913	0.960
KLF8	33.920	0.317	0.509	0.534	0.660	33.920	0.491	0.504	0.330	0.590	33.920	0.175	0.584	0.765	0.881
POU6F2	52.187	-0.585	0.343	0.088	0.178	52.187	0.029	0.332	0.931	0.965	52.187	0.614	0.394	0.119	0.310
FDX2	166.746	0.309	0.401	0.441	0.577	166.746	-0.504	0.405	0.213	0.493	166.746	-0.813	0.467	0.081	0.247
HOGA1	14.062	-0.194	0.818	0.812	0.877	14.062	0.550	0.801	0.492	0.708	14.062	0.744	0.937	0.427	0.650
MGAT4B	4675.220	0.951	0.189	0.000	0.000	4675.220	-0.076	0.189	0.688	0.832	4675.220	-1.027	0.218	0.000	0.000
PNKP	641.118	0.868	0.240	0.000	0.002	641.118	0.070	0.241	0.770	0.878	641.118	-0.798	0.278	0.004	0.036
WDR89	284.303	-0.183	0.181	0.312	0.451	284.303	-0.295	0.180	0.102	0.394	284.303	-0.112	0.210	0.593	0.773
L3HYPDH	174.535	-0.544	0.250	0.029	0.077	174.535	-0.324	0.247	0.190	0.475	174.535	0.220	0.289	0.446	0.666
B4GALT7	290.091	0.379	0.281	0.177	0.299	290.091	-0.435	0.283	0.125	0.416	290.091	-0.814	0.326	0.013	0.076
TP53RK	251.981	-0.307	0.309	0.320	0.460	251.981	-0.871	0.311	0.005	0.297	251.981	-0.564	0.360	0.117	0.306
SGF29	386.312	0.702	0.244	0.004	0.017	386.312	0.346	0.245	0.157	0.449	386.312	-0.356	0.282	0.206	0.430
LINC02605	9.323	1.562	0.685	0.023	0.063	9.323	-0.337	0.778	0.665	0.818	9.323	-1.898	0.843	0.024	0.115
SCAT8	57.416	-1.622	0.302	0.000	0.000	57.416	0.048	0.237	0.840	0.917	57.416	1.670	0.332	0.000	0.000
VPS26B	1003.539	-0.349	0.135	0.010	0.034	1003.539	0.007	0.134	0.961	0.980	1003.539	0.356	0.156	0.023	0.110
NACC1	1705.224	0.137	0.141	0.330	0.470	1705.224	-0.216	0.141	0.126	0.419	1705.224	-0.354	0.163	0.030	0.133
CFAP36	666.509	0.795	0.303	0.009	0.031	666.509	0.409	0.303	0.178	0.466	666.509	-0.386	0.350	0.270	0.505
MED8	500.403	-0.595	0.356	0.094	0.187	500.403	-0.798	0.356	0.025	0.316	500.403	-0.202	0.411	0.623	0.794
KTI12	42.394	0.629	0.390	0.107	0.206	42.394	-0.305	0.404	0.450	0.679	42.394	-0.934	0.460	0.042	0.165
ADCY7	566.746	-0.833	0.243	0.001	0.004	566.746	-0.241	0.242	0.320	0.581	566.746	0.592	0.281	0.035	0.146
LYST	148.885	-0.370	0.276	0.180	0.303	148.885	-0.105	0.273	0.699	0.838	148.885	0.264	0.318	0.407	0.633
RPUSD1	620.766	-0.040	0.204	0.843	0.897	620.766	-0.154	0.203	0.449	0.678	620.766	-0.114	0.235	0.629	0.798
PLCD3	1202.832	0.612	0.319	0.055	0.124	1202.832	0.405	0.319	0.204	0.487	1202.832	-0.206	0.368	0.576	0.761
CHRM3	173.471	-0.767	0.309	0.013	0.041	173.471	0.162	0.303	0.594	0.774	173.471	0.929	0.355	0.009	0.060
VPS45	377.264	0.073	0.130	0.574	0.693	377.264	-0.055	0.130	0.674	0.824	377.264	-0.128	0.151	0.397	0.625
MTFR2	142.793	-1.169	0.383	0.002	0.011	142.793	-0.818	0.380	0.031	0.322	142.793	0.351	0.443	0.428	0.651
LYPLA2	1098.192	0.486	0.177	0.006	0.023	1098.192	-0.234	0.178	0.188	0.474	1098.192	-0.720	0.205	0.000	0.007
CDCA5	874.135	-0.540	0.283	0.056	0.126	874.135	-0.014	0.282	0.959	0.979	874.135	0.525	0.327	0.108	0.293
AHNAK2	7414.264	0.388	0.531	0.465	0.600	7414.264	0.731	0.531	0.169	0.458	7414.264	0.343	0.613	0.576	0.761
PARK7	4957.328	0.394	0.208	0.057	0.128	4957.328	-0.095	0.208	0.647	0.808	4957.328	-0.489	0.240	0.041	0.162
RPLP0P2	83.468	1.672	0.549	0.002	0.011	83.468	0.715	0.566	0.206	0.488	83.468	-0.956	0.629	0.128	0.324
COPE	1933.945	0.694	0.203	0.001	0.004	1933.945	0.063	0.204	0.755	0.869	1933.945	-0.631	0.235	0.007	0.053
SAAL1	414.704	-0.585	0.280	0.037	0.091	414.704	-0.513	0.279	0.066	0.354	414.704	0.072	0.324	0.824	0.914
IZUMO4	49.582	0.454	1.342	0.735	0.821	49.582	1.132	1.339	0.398	0.642	49.582	0.678	1.547	0.661	0.819
SCAMP4	663.782	0.796	0.192	0.000	0.000	663.782	-0.139	0.194	0.474	0.696	663.782	-0.935	0.223	0.000	0.001
ADAT3	95.530	0.243	0.326	0.456	0.591	95.530	-0.293	0.330	0.375	0.622	95.530	-0.536	0.380	0.159	0.371
CHST14	225.136	0.823	0.235	0.000	0.003	225.136	0.340	0.236	0.150	0.443	225.136	-0.483	0.272	0.076	0.236
ECD	486.971	-0.830	0.275	0.003	0.012	486.971	-0.723	0.274	0.008	0.297	486.971	0.107	0.318	0.737	0.865
MGAT4A	14.648	0.141	0.461	0.760	0.841	14.648	-0.439	0.477	0.357	0.611	14.648	-0.580	0.550	0.292	0.528
CASC4	1390.184	0.229	0.148	0.123	0.228	1390.184	0.166	0.148	0.262	0.535	1390.184	-0.063	0.171	0.713	0.851
GPN1	607.995	-0.450	0.233	0.054	0.122	607.995	-0.434	0.233	0.062	0.350	607.995	0.016	0.270	0.954	0.979
MIR10394	61.969	0.939	0.429	0.029	0.076	61.969	1.068	0.426	0.012	0.305	61.969	0.128	0.488	0.793	0.896
MIR10393	38.529	1.263	0.527	0.017	0.050	38.529	0.949	0.528	0.072	0.361	38.529	-0.314	0.603	0.603	0.780
MIR10527	22.695	0.185	0.504	0.713	0.804	22.695	-0.053	0.505	0.917	0.959	22.695	-0.238	0.585	0.684	0.833
MIR12136	1662.115	1.922	0.442	0.000	0.000	1662.115	0.560	0.442	0.205	0.488	1662.115	-1.362	0.510	0.008	0.054
TMC6	59.756	1.227	0.258	0.000	0.000	59.756	0.719	0.263	0.006	0.297	59.756	-0.508	0.293	0.083	0.250
SLC46A1	998.747	0.064	0.114	0.577	0.695	998.747	0.099	0.114	0.385	0.631	998.747	0.035	0.132	0.791	0.896
C12orf57	969.958	0.831	0.256	0.001	0.007	969.958	0.395	0.256	0.124	0.416	969.958	-0.437	0.296	0.140	0.344
DDX42	2668.530	-0.705	0.152	0.000	0.000	2668.530	-0.299	0.151	0.048	0.337	2668.530	0.405	0.175	0.021	0.104
LARP4	1711.970	-0.809	0.340	0.017	0.051	1711.970	-0.894	0.340	0.008	0.297	1711.970	-0.085	0.392	0.828	0.915
GLCCI1	139.959	0.147	0.339	0.665	0.766	139.959	0.348	0.337	0.301	0.567	139.959	0.202	0.390	0.605	0.782
FKBP9	1767.850	0.133	0.238	0.576	0.695	1767.850	0.265	0.238	0.266	0.538	1767.850	0.132	0.275	0.633	0.800
STK38	390.615	0.208	0.144	0.149	0.263	390.615	-0.075	0.145	0.605	0.781	390.615	-0.283	0.167	0.090	0.263
PHB2	6422.436	-0.048	0.127	0.706	0.798	6422.436	0.107	0.127	0.401	0.644	6422.436	0.155	0.147	0.292	0.527
ACOT7	1492.596	0.223	0.282	0.427	0.564	1492.596	-0.415	0.282	0.141	0.431	1492.596	-0.639	0.325	0.050	0.182
PDAP1	3320.124	-0.055	0.250	0.825	0.885	3320.124	-0.397	0.250	0.112	0.405	3320.124	-0.342	0.288	0.236	0.465
TUSC2	322.517	0.409	0.229	0.075	0.157	322.517	-0.207	0.231	0.370	0.619	322.517	-0.616	0.266	0.021	0.104
CBX3	4607.768	-0.100	0.091	0.270	0.406	4607.768	-0.102	0.091	0.261	0.534	4607.768	-0.002	0.105	0.987	0.993

EXOC3	2217.507	-0.082	0.107	0.442	0.578	2217.507	0.061	0.107	0.566	0.753	2217.507	0.144	0.124	0.245	0.476
GABARAP	1432.102	0.883	0.245	0.000	0.002	1432.102	0.348	0.245	0.156	0.448	1432.102	-0.535	0.283	0.058	0.202
U2AF2	3850.043	-0.360	0.209	0.085	0.173	3850.043	-0.387	0.209	0.065	0.353	3850.043	-0.026	0.242	0.914	0.960
OIP5	173.679	-0.476	0.296	0.108	0.207	173.679	-0.303	0.294	0.303	0.569	173.679	0.173	0.342	0.613	0.786
EXOSC8	626.430	-0.627	0.315	0.046	0.109	626.430	-0.470	0.314	0.135	0.426	626.430	0.157	0.364	0.666	0.822
SFT2D1	857.274	0.155	0.136	0.255	0.390	857.274	-0.128	0.136	0.348	0.604	857.274	-0.283	0.158	0.073	0.231
TEX261	751.046	-0.557	0.228	0.014	0.045	751.046	-0.497	0.227	0.029	0.322	751.046	0.060	0.263	0.821	0.911
RNF13	838.860	-0.289	0.111	0.009	0.032	838.860	-0.058	0.110	0.597	0.776	838.860	0.231	0.128	0.071	0.228
MGLL	120.124	1.567	0.479	0.001	0.006	120.124	-1.439	0.501	0.004	0.297	120.124	-3.006	0.569	0.000	0.000
TWF2	1160.859	0.439	0.121	0.000	0.002	1160.859	0.148	0.122	0.224	0.503	1160.859	-0.291	0.140	0.038	0.154
SMIM12	527.004	-0.351	0.299	0.241	0.374	527.004	-0.772	0.299	0.010	0.298	527.004	-0.422	0.346	0.223	0.450
GABARAPL2	863.205	0.267	0.100	0.007	0.027	863.205	0.030	0.100	0.763	0.874	863.205	-0.237	0.115	0.040	0.159
AZIN2	23.987	1.574	0.541	0.004	0.015	23.987	1.135	0.544	0.037	0.325	23.987	-0.439	0.613	0.474	0.688
TMEM54	123.166	2.023	0.207	0.000	0.000	123.166	-0.053	0.223	0.814	0.902	123.166	-2.076	0.246	0.000	0.000
DERPC	24.290	-0.590	0.407	0.148	0.261	24.290	0.096	0.384	0.803	0.897	24.290	0.686	0.465	0.140	0.344
SYNPO	3911.666	0.153	0.422	0.718	0.808	3911.666	0.546	0.422	0.196	0.479	3911.666	0.394	0.488	0.420	0.644
HELQ	122.025	-0.616	0.284	0.030	0.078	122.025	0.042	0.277	0.880	0.940	122.025	0.658	0.326	0.044	0.168
CMTM1	51.372	-1.034	0.423	0.015	0.045	51.372	0.104	0.405	0.798	0.895	51.372	1.138	0.484	0.019	0.098
CHRNA3	27.013	0.337	0.443	0.446	0.581	27.013	-0.428	0.454	0.345	0.602	27.013	-0.766	0.521	0.142	0.347
CYP2U1	151.940	0.655	0.373	0.079	0.164	151.940	0.422	0.373	0.257	0.530	151.940	-0.232	0.430	0.589	0.769
ADPRHL1	10.426	1.108	0.642	0.084	0.172	10.426	0.837	0.645	0.194	0.478	10.426	-0.271	0.727	0.710	0.849
MFSD3	539.634	0.603	0.207	0.004	0.015	539.634	0.201	0.207	0.332	0.592	539.634	-0.402	0.239	0.092	0.266
SDSL	126.618	0.418	0.276	0.129	0.236	126.618	-0.102	0.278	0.714	0.847	126.618	-0.520	0.320	0.104	0.287
KLHDC7B	10.006	-0.509	0.806	0.528	0.654	10.006	-0.020	0.779	0.979	0.988	10.006	0.488	0.927	0.598	0.776
PIK3IP1	336.442	1.448	0.741	0.051	0.117	336.442	0.542	0.742	0.465	0.689	336.442	-0.905	0.856	0.290	0.525
CHRNA5	178.455	-0.445	0.320	0.165	0.284	178.455	-0.531	0.320	0.097	0.392	178.455	-0.086	0.371	0.817	0.909
HENMT1	14.112	0.504	0.406	0.215	0.345	14.112	0.582	0.397	0.143	0.435	14.112	0.077	0.458	0.866	0.936
FAM83F	10.646	-0.556	0.618	0.368	0.509	10.646	0.102	0.578	0.861	0.929	10.646	0.658	0.704	0.350	0.583
SLC35A4	1754.693	-0.309	0.131	0.018	0.054	1754.693	-0.121	0.130	0.352	0.606	1754.693	0.187	0.151	0.215	0.440
DTX2	456.211	1.208	0.175	0.000	0.000	456.211	0.183	0.178	0.304	0.570	456.211	-1.025	0.203	0.000	0.000
CHRNA7	37.333	0.251	0.416	0.546	0.670	37.333	0.672	0.407	0.099	0.393	37.333	0.421	0.474	0.374	0.606
CHRNB1	164.996	0.556	0.426	0.192	0.317	164.996	0.594	0.425	0.163	0.453	164.996	0.038	0.491	0.938	0.972
PARGP1-AGAP4	8.860	-0.145	0.585	0.804	0.870	8.860	-0.539	0.596	0.366	0.617	8.860	-0.394	0.697	0.572	0.760
TOE1	191.497	-0.928	0.418	0.027	0.072	191.497	-0.779	0.417	0.062	0.350	191.497	0.149	0.484	0.758	0.877
LINC00334	80.926	1.604	0.326	0.000	0.000	80.926	0.406	0.337	0.228	0.506	80.926	-1.199	0.377	0.001	0.018
MCM3AP-AS1	610.141	-0.212	0.168	0.206	0.334	610.141	-0.397	0.168	0.018	0.306	610.141	-0.185	0.194	0.342	0.576
BUD23	1406.075	0.031	0.270	0.908	0.941	1406.075	-0.469	0.270	0.082	0.374	1406.075	-0.501	0.312	0.109	0.294
TXNRD3	313.327	-0.378	0.254	0.136	0.246	313.327	0.087	0.252	0.730	0.855	313.327	0.465	0.293	0.112	0.298
SLC2A13	376.388	0.692	0.342	0.043	0.103	376.388	0.232	0.342	0.498	0.711	376.388	-0.460	0.395	0.244	0.475
LACTB	641.004	1.185	0.123	0.000	0.000	641.004	0.127	0.126	0.314	0.577	641.004	-1.058	0.143	0.000	0.000
EFHC1	180.666	-0.462	0.254	0.069	0.148	180.666	0.395	0.249	0.113	0.405	180.666	0.857	0.292	0.003	0.031
LOC114483834	29.553	-1.066	0.533	0.045	0.107	29.553	-1.325	0.535	0.013	0.305	29.553	-0.258	0.629	0.682	0.832
CHRNE	53.691	1.751	0.455	0.000	0.001	53.691	0.455	0.464	0.327	0.588	53.691	-1.296	0.526	0.014	0.081
MAL2	77.528	0.290	0.228	0.203	0.330	77.528	0.336	0.225	0.134	0.426	77.528	0.046	0.261	0.859	0.932
TIRAP	81.805	-0.425	0.261	0.104	0.201	81.805	0.343	0.249	0.168	0.457	81.805	0.767	0.297	0.010	0.065
MIR155HG	33.032	1.062	0.417	0.011	0.037	33.032	0.490	0.427	0.251	0.525	33.032	-0.571	0.479	0.233	0.462
ERMAP	272.127	-0.327	0.414	0.430	0.566	272.127	0.629	0.412	0.126	0.419	272.127	0.956	0.477	0.045	0.171
LRRC37B	95.847	0.499	0.365	0.171	0.292	95.847	0.429	0.364	0.239	0.515	95.847	-0.070	0.420	0.867	0.936
CHUK	446.805	-1.303	0.423	0.002	0.010	446.805	-1.187	0.422	0.005	0.297	446.805	0.116	0.489	0.812	0.906
CARD16	111.188	1.666	0.297	0.000	0.000	111.188	-0.010	0.307	0.974	0.986	111.188	-1.676	0.347	0.000	0.000
BTBD9	173.276	-0.094	0.296	0.751	0.834	173.276	0.321	0.293	0.272	0.543	173.276	0.415	0.340	0.223	0.450
LMTK3	12.654	1.418	0.658	0.031	0.081	12.654	0.913	0.666	0.170	0.460	12.654	-0.504	0.746	0.499	0.706
MBD6	751.347	-0.213	0.117	0.069	0.148	751.347	0.166	0.115	0.149	0.442	751.347	0.379	0.135	0.005	0.040
XKR4	59.264	-0.162	0.313	0.606	0.718	59.264	0.411	0.304	0.176	0.465	59.264	0.572	0.357	0.109	0.295
GPRIN1	137.723	0.386	0.418	0.356	0.496	137.723	-1.203	0.426	0.005	0.297	137.723	-1.588	0.490	0.001	0.015
CSMD3	15.765	0.995	0.540	0.066	0.142	15.765	0.632	0.547	0.248	0.522	15.765	-0.363	0.615	0.555	0.747
SLC25A25	323.325	-1.056	0.361	0.003	0.015	323.325	-0.591	0.359	0.100	0.394	323.325	0.465	0.417	0.265	0.499
STK11IP	305.313	-0.270	0.156	0.083	0.170	305.313	0.334	0.152	0.028	0.321	305.313	0.603	0.178	0.001	0.010
TUBGCP5	569.305	-0.497	0.291	0.088	0.177	569.305	-0.533	0.291	0.067	0.354	569.305	-0.036	0.336	0.914	0.960
FMNL2	1423.714	-0.582	0.223	0.009	0.031	1423.714	-0.099	0.222	0.657	0.813	1423.714	0.483	0.257	0.060	0.205
ELFN2	1154.324	-0.325	0.186	0.080	0.165	1154.324	0.179	0.185	0.334	0.593	1154.324	0.504	0.214	0.019	0.097
PSMG3-AS1	36.424	0.584	0.589	0.322	0.461	36.424	0.394	0.589	0.504	0.715	36.424	-0.190	0.679	0.780	0.889
ESCO1	374.334	-0.446	0.236	0.058	0.130	374.334	0.029	0.233	0.901	0.950	374.334	0.475	0.272	0.081	0.245
TMEM200A	41.518	0.344	0.312	0.270	0.406	41.518	0.331	0.309	0.283	0.552	41.518	-0.013	0.358	0.972	0.986
MYSM1	358.640	-0.584	0.249	0.019	0.055	358.640	-0.032	0.247	0.895	0.948	358.640	0.551	0.287	0.055	0.194
RNF157	104.858	0.625	0.177	0.000	0.003	104.858	-0.132	0.183	0.471	0.693	104.858	-0.757	0.208	0.000	0.005
GNRHR2	223.665	-0.573	0.173	0.001	0.006	223.665	-0.267	0.170	0.117	0.409	223.665	0.306	0.201	0.127	0.323

SORCS1	58.035	0.323	0.461	0.483	0.616	58.035	0.710	0.453	0.117	0.409	58.035	0.387	0.525	0.461	0.678
KLHL29	802.898	-0.993	0.204	0.000	0.000	802.898	-0.162	0.202	0.422	0.658	802.898	0.831	0.235	0.000	0.007
CROCCP3	14.421	2.330	0.671	0.001	0.003	14.421	0.766	0.699	0.273	0.544	14.421	-1.563	0.770	0.042	0.165
ZBED9	112.918	-0.588	0.281	0.036	0.090	112.918	0.007	0.274	0.981	0.989	112.918	0.594	0.323	0.065	0.216
RHPN1	16.385	1.439	0.573	0.012	0.039	16.385	0.768	0.585	0.189	0.475	16.385	-0.670	0.653	0.304	0.540
LENG8	3383.658	0.982	0.281	0.000	0.003	3383.658	0.588	0.281	0.037	0.324	3383.658	-0.395	0.324	0.224	0.451
PWWP2A	266.270	-0.440	0.179	0.014	0.043	266.270	-0.103	0.176	0.557	0.747	266.270	0.337	0.206	0.103	0.285
SMYD4	114.933	-0.222	0.217	0.306	0.445	114.933	-0.086	0.213	0.686	0.831	114.933	0.136	0.251	0.588	0.769
FHAD1	138.712	0.153	0.187	0.413	0.551	138.712	0.262	0.184	0.155	0.447	138.712	0.109	0.215	0.610	0.785
LOC114841035	142.210	0.749	0.302	0.013	0.042	142.210	0.455	0.303	0.133	0.425	142.210	-0.294	0.348	0.399	0.626
OSBPL1A	1078.873	0.148	0.225	0.509	0.639	1078.873	0.286	0.224	0.202	0.485	1078.873	0.138	0.259	0.595	0.774
OSBPL5	4886.047	0.200	0.343	0.559	0.681	4886.047	0.631	0.342	0.066	0.354	4886.047	0.431	0.395	0.276	0.511
OSBPL6	352.258	-0.376	0.284	0.185	0.309	352.258	-0.424	0.283	0.135	0.426	352.258	-0.048	0.328	0.884	0.945
OSBPL7	141.451	0.452	0.357	0.205	0.333	141.451	0.527	0.356	0.139	0.430	141.451	0.075	0.411	0.855	0.930
OSBPL8	1408.596	-0.699	0.272	0.010	0.035	1408.596	-0.326	0.272	0.230	0.507	1408.596	0.373	0.314	0.236	0.465
OSBPL9	1779.875	0.094	0.154	0.544	0.668	1779.875	0.102	0.154	0.509	0.718	1779.875	0.008	0.178	0.964	0.983
OSBPL10	617.495	-1.005	0.262	0.000	0.001	617.495	0.238	0.259	0.357	0.611	617.495	1.244	0.301	0.000	0.001
OSBPL11	594.418	-0.730	0.173	0.000	0.000	594.418	-0.486	0.171	0.005	0.297	594.418	0.244	0.200	0.223	0.450
C1QTNF1	429.085	0.952	0.107	0.000	0.000	429.085	0.237	0.110	0.031	0.322	429.085	-0.715	0.124	0.000	0.000
C1QTNF3	26.663	0.128	0.492	0.796	0.865	26.663	0.597	0.482	0.215	0.494	26.663	0.469	0.562	0.403	0.630
C1QTNF6	52.924	0.794	0.693	0.252	0.386	52.924	1.055	0.691	0.127	0.419	52.924	0.261	0.796	0.743	0.868
FBXO32	689.352	1.428	0.759	0.060	0.132	689.352	0.815	0.759	0.283	0.552	689.352	-0.613	0.876	0.484	0.696
TMEM123	10051.718	-0.635	0.233	0.006	0.024	10051.718	0.205	0.233	0.379	0.626	10051.718	0.840	0.269	0.002	0.020
EPB41L4A-AS1	1409.160	0.455	0.371	0.221	0.352	1409.160	0.713	0.371	0.055	0.342	1409.160	0.258	0.429	0.547	0.742
SMIM19	268.181	0.008	0.277	0.976	0.985	268.181	0.365	0.275	0.184	0.471	268.181	0.357	0.319	0.263	0.498
GPRASP2	20.128	-0.294	0.457	0.520	0.648	20.128	-0.129	0.448	0.773	0.879	20.128	0.165	0.529	0.755	0.875
MRFAP1L1	774.301	0.417	0.147	0.004	0.018	774.301	-0.074	0.147	0.616	0.788	774.301	-0.491	0.170	0.004	0.034
PTPMT1	556.585	0.518	0.192	0.007	0.026	556.585	-0.004	0.193	0.982	0.990	556.585	-0.522	0.222	0.019	0.097
FLYWCH2	376.894	0.700	0.250	0.005	0.020	376.894	0.054	0.251	0.831	0.913	376.894	-0.646	0.289	0.026	0.119
WDR31	115.816	0.014	0.447	0.975	0.985	115.816	0.456	0.444	0.305	0.571	115.816	0.442	0.515	0.390	0.619
VASN	1267.898	1.354	0.739	0.067	0.144	1267.898	1.022	0.740	0.167	0.456	1267.898	-0.332	0.854	0.698	0.841
ZNF618	516.095	-0.240	0.264	0.364	0.505	516.095	-0.061	0.264	0.818	0.905	516.095	0.179	0.305	0.558	0.749
ADCY9	1189.966	-0.106	0.197	0.592	0.707	1189.966	0.197	0.196	0.317	0.579	1189.966	0.302	0.227	0.184	0.403
CGAS	515.257	-0.534	0.288	0.063	0.138	515.257	-0.643	0.288	0.025	0.316	515.257	-0.109	0.333	0.744	0.868
NT5C3B	1023.299	-0.014	0.109	0.896	0.933	1023.299	0.131	0.108	0.226	0.505	1023.299	0.145	0.126	0.248	0.479
CCDC124	3116.769	0.674	0.275	0.014	0.044	3116.769	-0.180	0.275	0.512	0.719	3116.769	-0.854	0.317	0.007	0.052
HAUS1	353.918	0.237	0.248	0.339	0.479	353.918	-0.234	0.249	0.347	0.602	353.918	-0.471	0.287	0.101	0.282
TNFRSF14-AS1	31.372	1.154	0.548	0.035	0.089	31.372	0.672	0.552	0.223	0.502	31.372	-0.482	0.630	0.444	0.664
MARCHF3	114.804	-0.116	0.243	0.632	0.741	114.804	-0.241	0.243	0.320	0.581	114.804	-0.125	0.284	0.660	0.818
ZNF554	83.633	0.078	0.256	0.762	0.842	83.633	0.471	0.250	0.060	0.350	83.633	0.393	0.292	0.179	0.397
CKB	359.214	0.367	0.742	0.621	0.731	359.214	0.864	0.741	0.244	0.519	359.214	0.497	0.856	0.561	0.752
ATG4A	112.201	0.147	0.255	0.565	0.686	112.201	-0.326	0.258	0.205	0.488	112.201	-0.473	0.298	0.112	0.299
KCTD12	776.386	-0.581	0.355	0.102	0.198	776.386	0.166	0.354	0.640	0.804	776.386	0.746	0.410	0.069	0.223
OMA1	241.227	-0.413	0.181	0.022	0.063	241.227	-0.282	0.179	0.115	0.406	241.227	0.130	0.209	0.534	0.733
DDIT4L	44.419	0.387	0.452	0.392	0.531	44.419	0.664	0.436	0.128	0.420	44.419	0.277	0.508	0.586	0.767
SLC25A26	611.515	-0.296	0.171	0.083	0.170	611.515	0.114	0.169	0.500	0.713	611.515	0.410	0.197	0.037	0.152
FBXO17	286.772	0.815	0.224	0.000	0.002	286.772	0.307	0.225	0.173	0.462	286.772	-0.507	0.259	0.050	0.183
PCMTD1	789.606	0.247	0.352	0.482	0.615	789.606	0.519	0.351	0.140	0.431	789.606	0.271	0.406	0.504	0.711
CIRBP	2660.981	-0.179	0.207	0.385	0.525	2660.981	0.385	0.206	0.062	0.350	2660.981	0.565	0.238	0.018	0.095
GPR146	338.016	1.271	0.907	0.161	0.280	338.016	0.996	0.907	0.272	0.543	338.016	-0.275	1.046	0.793	0.896
LRRC42	767.053	-0.241	0.142	0.089	0.179	767.053	0.124	0.140	0.378	0.626	767.053	0.365	0.163	0.025	0.118
GBP4	86.541	3.234	0.455	0.000	0.000	86.541	0.609	0.468	0.193	0.478	86.541	-2.624	0.526	0.000	0.000
GBP5	13.481	4.856	0.620	0.000	0.000	13.481	2.429	0.673	0.000	0.211	13.481	-2.427	0.567	0.000	0.001
LRRC56	14.986	1.198	0.653	0.066	0.143	14.986	0.487	0.665	0.464	0.689	14.986	-0.711	0.752	0.344	0.578
CISH	27.130	1.693	0.477	0.000	0.003	27.130	0.849	0.485	0.080	0.371	27.130	-0.845	0.545	0.121	0.313
MALSU1	390.522	-0.197	0.249	0.427	0.564	390.522	-0.418	0.249	0.093	0.388	390.522	-0.220	0.288	0.444	0.664
UHRF2	472.703	-0.827	0.201	0.000	0.000	472.703	-0.116	0.198	0.559	0.748	472.703	0.712	0.231	0.002	0.022
TBCB	1043.639	0.711	0.200	0.000	0.003	1043.639	0.141	0.200	0.481	0.700	1043.639	-0.570	0.231	0.014	0.080
ZNF689	193.186	-0.482	0.242	0.046	0.109	193.186	-0.274	0.240	0.252	0.525	193.186	0.208	0.280	0.458	0.675
FCHO2	532.666	-0.583	0.228	0.011	0.036	532.666	-0.099	0.226	0.662	0.816	532.666	0.484	0.263	0.066	0.217
ARHGEF25	127.654	0.414	0.339	0.221	0.352	127.654	0.682	0.336	0.043	0.330	127.654	0.268	0.389	0.491	0.701
ZNF501	33.958	-0.731	0.308	0.018	0.052	33.958	0.179	0.278	0.521	0.725	33.958	0.909	0.347	0.009	0.060
TENT5B	104.853	0.097	0.531	0.855	0.906	104.853	0.200	0.530	0.706	0.842	104.853	0.103	0.613	0.866	0.936
KIR3DL3	19.933	-1.418	1.512	0.349	0.489	19.933	-0.278	1.497	0.853	0.925	19.933	1.140	1.744	0.513	0.718
NOSTRIN	21.924	1.010	0.373	0.007	0.025	21.924	-0.058	0.396	0.884	0.942	21.924	-1.068	0.442	0.016	0.087
ALPK2	71.448	-1.461	0.375	0.000	0.001	71.448	-0.767	0.364	0.035	0.324	71.448	0.694	0.434	0.110	0.295
ARHGAP33	93.323	0.335	0.509	0.510	0.639	93.323	0.923	0.506	0.068	0.357	93.323	0.588	0.585	0.315	0.551

EVI5L	844.659	0.318	0.166	0.055	0.124	844.659	0.218	0.166	0.189	0.475	844.659	-0.100	0.192	0.600	0.778
TRMT61A	366.054	-0.868	0.412	0.035	0.088	366.054	-1.035	0.412	0.012	0.305	366.054	-0.167	0.477	0.727	0.858
C12orf56	26.743	0.298	0.460	0.518	0.645	26.743	1.174	0.444	0.008	0.297	26.743	0.876	0.518	0.091	0.264
DIS3L	172.850	-0.168	0.261	0.519	0.646	172.850	0.296	0.257	0.249	0.523	172.850	0.464	0.300	0.121	0.313
FANCD2OS	86.945	-0.825	0.376	0.028	0.075	86.945	-0.602	0.373	0.106	0.399	86.945	0.222	0.436	0.610	0.785
IQCD	33.178	0.434	0.427	0.309	0.449	33.178	0.113	0.429	0.792	0.892	33.178	-0.321	0.494	0.516	0.720
DHRS1	175.250	0.723	0.248	0.004	0.015	175.250	0.327	0.249	0.189	0.475	175.250	-0.396	0.286	0.166	0.381
WDFY2	604.261	-1.056	0.205	0.000	0.000	604.261	-0.311	0.203	0.126	0.418	604.261	0.745	0.237	0.002	0.019
CKMT1B	249.344	-0.120	0.232	0.606	0.718	249.344	0.059	0.231	0.799	0.895	249.344	0.179	0.268	0.505	0.712
TSR3	960.015	0.183	0.174	0.293	0.431	960.015	-0.060	0.174	0.730	0.855	960.015	-0.243	0.201	0.227	0.455
CCDC151	319.694	-0.161	0.268	0.548	0.671	319.694	0.434	0.266	0.102	0.394	319.694	0.595	0.308	0.054	0.191
ZNF653	152.132	0.371	0.247	0.133	0.242	152.132	0.044	0.248	0.861	0.929	152.132	-0.327	0.286	0.253	0.485
RNF166	328.896	-0.313	0.258	0.226	0.357	328.896	-0.217	0.257	0.398	0.642	328.896	0.095	0.299	0.749	0.871
RMI2	215.588	0.033	0.247	0.892	0.931	215.588	0.039	0.246	0.873	0.936	215.588	0.006	0.285	0.983	0.991
OSR2	18.448	0.303	0.452	0.503	0.632	18.448	0.173	0.451	0.702	0.839	18.448	-0.130	0.522	0.803	0.901
LRRC58	2328.645	-0.215	0.139	0.123	0.229	2328.645	-0.155	0.139	0.264	0.536	2328.645	0.059	0.161	0.712	0.850
LYSMD3	708.710	-0.626	0.263	0.017	0.052	708.710	-0.030	0.262	0.909	0.955	708.710	0.596	0.304	0.050	0.182
BATF2	16.046	0.410	0.504	0.416	0.554	16.046	0.540	0.494	0.274	0.545	16.046	0.131	0.572	0.819	0.911
DNTTIP1	581.264	0.545	0.269	0.042	0.102	581.264	-0.223	0.270	0.408	0.649	581.264	-0.769	0.311	0.013	0.080
ERCC8	325.809	-0.631	0.201	0.002	0.009	325.809	-0.466	0.199	0.019	0.306	325.809	0.165	0.233	0.478	0.691
FOXP4	974.964	0.370	0.125	0.003	0.014	974.964	-0.293	0.126	0.020	0.307	974.964	-0.663	0.145	0.000	0.000
ZNF526	314.901	-0.204	0.125	0.102	0.198	314.901	-0.042	0.122	0.732	0.857	314.901	0.162	0.144	0.261	0.495
KLHDC3	1734.394	-0.135	0.149	0.366	0.506	1734.394	-0.122	0.149	0.414	0.652	1734.394	0.013	0.173	0.939	0.973
WDR92	277.353	-0.341	0.272	0.210	0.339	277.353	-0.577	0.272	0.034	0.322	277.353	-0.237	0.315	0.452	0.671
NUS1	1955.921	-0.828	0.216	0.000	0.001	1955.921	-0.490	0.216	0.023	0.316	1955.921	0.338	0.250	0.176	0.394
FAM210B	985.018	0.616	0.192	0.001	0.007	985.018	0.191	0.192	0.320	0.581	985.018	-0.425	0.222	0.055	0.195
TM4SF19	190.654	-0.371	0.268	0.167	0.287	190.654	-0.404	0.267	0.130	0.422	190.654	-0.034	0.311	0.914	0.960
FAM122A	226.160	-0.270	0.164	0.099	0.194	226.160	-0.243	0.162	0.135	0.426	226.160	0.028	0.190	0.884	0.945
ZMYND19	624.508	-0.060	0.403	0.881	0.923	624.508	-0.631	0.403	0.118	0.409	624.508	-0.571	0.465	0.220	0.447
COX20	640.734	0.080	0.185	0.666	0.767	640.734	-0.142	0.185	0.441	0.672	640.734	-0.222	0.214	0.298	0.534
ABHD15	121.917	1.070	0.190	0.000	0.000	121.917	0.272	0.196	0.164	0.454	121.917	-0.797	0.220	0.000	0.005
TLCD1	184.409	0.097	0.321	0.764	0.843	184.409	-0.211	0.322	0.512	0.719	184.409	-0.308	0.372	0.408	0.635
GINM1	927.885	0.367	0.107	0.001	0.004	927.885	0.074	0.107	0.488	0.705	927.885	-0.293	0.124	0.018	0.094
CKS1B	1538.036	-0.507	0.191	0.008	0.028	1538.036	0.010	0.190	0.958	0.978	1538.036	0.517	0.220	0.019	0.098
EXOC3-AS1	93.435	-0.106	0.329	0.748	0.832	93.435	-0.594	0.332	0.074	0.363	93.435	-0.488	0.385	0.204	0.428
LYPD1	37.811	1.631	0.315	0.000	0.000	37.811	-0.023	0.343	0.947	0.973	37.811	-1.654	0.375	0.000	0.000
CKS2	1675.098	0.477	0.189	0.012	0.038	1675.098	-0.084	0.189	0.658	0.813	1675.098	-0.561	0.218	0.010	0.067
ZNF837	28.417	0.699	0.449	0.120	0.224	28.417	0.885	0.443	0.046	0.334	28.417	0.186	0.508	0.715	0.852
GRIN3B	68.685	1.242	0.420	0.003	0.014	68.685	-0.085	0.430	0.844	0.919	68.685	-1.326	0.490	0.007	0.050
TOP1MT	888.424	0.067	0.152	0.660	0.762	888.424	0.091	0.152	0.550	0.743	888.424	0.024	0.176	0.892	0.949
TSEN15	868.497	-0.443	0.309	0.152	0.267	868.497	-0.592	0.309	0.056	0.343	868.497	-0.149	0.357	0.677	0.829
NIBAN1	292.906	-0.643	0.265	0.015	0.047	292.906	-0.234	0.263	0.374	0.622	292.906	0.410	0.306	0.181	0.399
MRPL53	14.743	0.353	0.481	0.462	0.597	14.743	-0.135	0.495	0.786	0.888	14.743	-0.488	0.567	0.390	0.618
MRPL54	515.844	0.325	0.201	0.105	0.202	515.844	0.236	0.201	0.240	0.516	515.844	-0.090	0.232	0.699	0.843
HSPA12B	6.694	0.574	0.733	0.434	0.570	6.694	-1.698	0.885	0.055	0.342	6.694	-2.272	0.976	0.020	0.102
CNTROB	457.965	0.234	0.157	0.137	0.248	457.965	0.115	0.157	0.464	0.689	457.965	-0.119	0.182	0.514	0.718
SNAP47	725.263	-0.374	0.159	0.018	0.054	725.263	-0.214	0.158	0.175	0.464	725.263	0.160	0.183	0.383	0.612
SLC18B1	294.287	-0.634	0.134	0.000	0.000	294.287	-0.276	0.130	0.034	0.322	294.287	0.358	0.155	0.021	0.104
LRG1	40.573	3.601	0.442	0.000	0.000	40.573	0.783	0.492	0.112	0.405	40.573	-2.818	0.507	0.000	0.000
MED12L	37.038	-0.772	0.334	0.021	0.060	37.038	-0.410	0.323	0.204	0.487	37.038	0.362	0.388	0.350	0.583
WDR17	91.776	-1.153	0.340	0.001	0.004	91.776	0.142	0.327	0.665	0.818	91.776	1.295	0.389	0.001	0.012
ACAP3	1204.438	0.515	0.167	0.002	0.010	1204.438	0.285	0.167	0.088	0.380	1204.438	-0.230	0.193	0.233	0.461
ARAP2	56.064	-0.784	0.279	0.005	0.019	56.064	-0.019	0.263	0.943	0.971	56.064	0.765	0.319	0.016	0.090
ARAP1	2076.548	0.261	0.255	0.306	0.445	2076.548	0.316	0.254	0.214	0.493	2076.548	0.055	0.294	0.850	0.928
AGAP2	404.276	0.821	0.177	0.000	0.000	404.276	0.135	0.179	0.452	0.680	404.276	-0.686	0.205	0.001	0.012
AGAP1	1062.248	-0.491	0.255	0.054	0.122	1062.248	0.247	0.254	0.330	0.590	1062.248	0.739	0.294	0.012	0.073
AGAP3	1374.560	0.272	0.129	0.035	0.088	1374.560	-0.005	0.129	0.968	0.983	1374.560	-0.277	0.149	0.063	0.211
ADCYAP1R1	20.527	-0.891	1.157	0.441	0.577	20.527	1.126	1.134	0.321	0.582	20.527	2.017	1.326	0.128	0.324
TADA1	310.198	-0.461	0.157	0.003	0.015	310.198	0.000	0.154	1.000	1.000	310.198	0.461	0.181	0.011	0.069
CATSPER1	19.108	1.680	0.571	0.003	0.014	19.108	0.547	0.597	0.359	0.611	19.108	-1.132	0.658	0.085	0.254
THEM4	250.303	-0.081	0.163	0.621	0.732	250.303	-0.323	0.164	0.048	0.337	250.303	-0.242	0.190	0.202	0.426
CATSPER2	10.986	2.220	0.657	0.001	0.005	10.986	0.946	0.682	0.165	0.455	10.986	-1.273	0.747	0.088	0.259
WFIKKN1	24.012	1.220	0.767	0.111	0.212	24.012	0.849	0.771	0.271	0.542	24.012	-0.372	0.876	0.671	0.825
RAB3IP	535.734	-0.225	0.114	0.048	0.112	535.734	-0.252	0.113	0.026	0.316	535.734	-0.027	0.132	0.839	0.922
SSX2IP	634.327	-0.585	0.165	0.000	0.003	634.327	-0.404	0.164	0.014	0.306	634.327	0.180	0.191	0.345	0.580
MRGPRX3	11.015	3.791	0.768	0.000	0.000	11.015	0.435	0.943	0.645	0.806	11.015	-3.356	0.918	0.000	0.005
FTSJ3	1005.377	-0.725	0.378	0.055	0.125	1005.377	-0.993	0.378	0.009	0.297	1005.377	-0.268	0.437	0.540	0.737

GALNT15	618.408	-0.094	0.732	0.897	0.934	618.408	1.152	0.731	0.115	0.407	618.408	1.247	0.845	0.140	0.344
IP6K3	251.038	0.966	0.550	0.079	0.163	251.038	0.835	0.550	0.129	0.421	251.038	-0.131	0.634	0.836	0.920
AP2M1	13091.487	-0.205	0.116	0.076	0.159	13091.487	-0.162	0.116	0.162	0.453	13091.487	0.043	0.134	0.745	0.869
AP1S1	1354.345	0.202	0.145	0.165	0.284	1354.345	-0.160	0.145	0.272	0.543	1354.345	-0.362	0.168	0.031	0.136
AP2S1	2671.758	0.416	0.200	0.038	0.093	2671.758	-0.131	0.200	0.514	0.720	2671.758	-0.547	0.231	0.018	0.095
TWIST2	852.468	1.086	0.189	0.000	0.000	852.468	0.008	0.190	0.964	0.981	852.468	-1.077	0.218	0.000	0.000
PARD3B	150.638	-0.072	0.419	0.864	0.911	150.638	0.277	0.417	0.507	0.716	150.638	0.349	0.483	0.470	0.684
RFFL	498.588	-0.596	0.163	0.000	0.002	498.588	-0.150	0.161	0.351	0.605	498.588	0.446	0.188	0.018	0.094
AP3S1	1885.944	0.418	0.222	0.060	0.133	1885.944	0.422	0.222	0.057	0.347	1885.944	0.005	0.257	0.985	0.992
ZNF354B	57.241	0.052	0.240	0.830	0.888	57.241	-0.009	0.238	0.970	0.984	57.241	-0.061	0.278	0.828	0.915
TRIM6	38.338	0.426	0.295	0.149	0.263	38.338	0.521	0.291	0.073	0.362	38.338	0.095	0.336	0.778	0.888
ADD1	2099.986	-0.200	0.120	0.095	0.189	2099.986	-0.007	0.119	0.955	0.978	2099.986	0.193	0.138	0.163	0.376
CLCN2	268.694	0.372	0.205	0.070	0.149	268.694	-0.099	0.206	0.632	0.799	268.694	-0.471	0.238	0.048	0.177
CLCN3	1029.760	-0.492	0.201	0.014	0.044	1029.760	-0.017	0.200	0.934	0.967	1029.760	0.475	0.231	0.040	0.159
CLCN4	30.576	-1.025	0.362	0.005	0.019	30.576	0.132	0.327	0.687	0.831	30.576	1.157	0.409	0.005	0.039
CLCN5	275.112	-0.108	0.238	0.651	0.756	275.112	-0.609	0.240	0.011	0.302	275.112	-0.501	0.277	0.071	0.227
UBE2J2	1555.633	-0.305	0.276	0.270	0.406	1555.633	-0.474	0.276	0.086	0.378	1555.633	-0.170	0.319	0.595	0.774
BORCS5	130.927	-0.303	0.164	0.065	0.140	130.927	-0.222	0.160	0.165	0.455	130.927	0.081	0.191	0.671	0.825
ANTXR2	394.167	-0.512	0.253	0.043	0.103	394.167	-0.264	0.251	0.293	0.560	394.167	0.248	0.292	0.396	0.624
RPL23AP7	109.183	0.168	0.218	0.442	0.578	109.183	0.370	0.214	0.083	0.375	109.183	0.202	0.249	0.417	0.642
GPR62	12.793	1.807	0.610	0.003	0.014	12.793	-0.202	0.674	0.764	0.875	12.793	-2.008	0.735	0.006	0.048
EXOSC6	484.303	0.228	0.210	0.276	0.414	484.303	-0.171	0.210	0.417	0.655	484.303	-0.399	0.243	0.101	0.281
ZNF511	303.771	0.128	0.213	0.548	0.671	303.771	0.057	0.213	0.789	0.890	303.771	-0.071	0.247	0.773	0.885
CHCHD1	369.231	0.352	0.251	0.162	0.281	369.231	-0.143	0.252	0.571	0.757	369.231	-0.494	0.291	0.089	0.262
CFAP70	18.710	-0.322	0.357	0.366	0.507	18.710	0.041	0.336	0.902	0.950	18.710	0.364	0.408	0.373	0.605
CLCN6	412.041	0.788	0.257	0.002	0.010	412.041	0.384	0.257	0.135	0.426	412.041	-0.403	0.296	0.173	0.390
CLCN7	1627.398	0.881	0.246	0.000	0.003	1627.398	-0.446	0.247	0.071	0.360	1627.398	-1.328	0.285	0.000	0.000
C10orf90	208.837	-0.161	0.275	0.559	0.680	208.837	0.437	0.268	0.103	0.395	208.837	0.598	0.315	0.057	0.199
PSTK	88.346	-0.760	0.223	0.001	0.004	88.346	-0.186	0.212	0.379	0.626	88.346	0.574	0.257	0.025	0.118
ZNF488	45.964	-0.696	0.294	0.018	0.053	45.964	-0.229	0.282	0.417	0.655	45.964	0.467	0.339	0.169	0.384
MORN4	207.972	-0.216	0.211	0.305	0.444	207.972	0.240	0.207	0.247	0.522	207.972	0.456	0.242	0.060	0.204
ZFYVE27	362.028	-0.228	0.211	0.278	0.416	362.028	-0.174	0.210	0.405	0.646	362.028	0.054	0.244	0.825	0.914
MMP21	22.100	-0.212	0.402	0.598	0.711	22.100	-0.348	0.401	0.385	0.631	22.100	-0.136	0.472	0.774	0.885
COMTD1	390.976	0.385	0.192	0.045	0.107	390.976	0.124	0.192	0.520	0.724	390.976	-0.261	0.222	0.240	0.471
FRA10AC1	725.895	0.117	0.243	0.630	0.739	725.895	-0.184	0.243	0.448	0.677	725.895	-0.302	0.281	0.283	0.518
ANKRD22	16.733	-0.606	0.499	0.224	0.355	16.733	-0.770	0.498	0.122	0.413	16.733	-0.164	0.591	0.781	0.890
SFXN2	48.640	-1.144	0.462	0.013	0.042	48.640	-0.906	0.455	0.047	0.335	48.640	0.238	0.537	0.657	0.817
PDZD8	1353.029	-0.865	0.184	0.000	0.000	1353.029	-0.072	0.183	0.695	0.836	1353.029	0.793	0.213	0.000	0.004
ADD2	917.360	-0.458	0.165	0.006	0.021	917.360	-0.101	0.164	0.540	0.737	917.360	0.358	0.191	0.061	0.206
AGAP4	54.981	1.129	0.395	0.004	0.017	54.981	0.323	0.401	0.420	0.657	54.981	-0.806	0.457	0.078	0.241
BORCS7	149.922	0.172	0.299	0.565	0.686	149.922	0.134	0.298	0.652	0.810	149.922	-0.038	0.345	0.913	0.960
CLU	1884.331	0.469	0.358	0.191	0.316	1884.331	0.420	0.358	0.242	0.518	1884.331	-0.049	0.414	0.906	0.957
CLIC1	35748.507	0.206	0.249	0.409	0.547	35748.507	-0.256	0.249	0.304	0.570	35748.507	-0.463	0.288	0.108	0.294
CLIC2	31.496	2.131	0.383	0.000	0.000	31.496	0.135	0.463	0.771	0.878	31.496	-1.997	0.469	0.000	0.001
GSTO2	49.581	0.434	0.297	0.144	0.257	49.581	0.569	0.293	0.052	0.341	49.581	0.135	0.339	0.691	0.837
SFR1	331.927	-0.625	0.282	0.027	0.072	331.927	-0.367	0.281	0.191	0.477	331.927	0.258	0.327	0.429	0.652
CLK1	585.414	0.063	0.374	0.866	0.913	585.414	0.925	0.373	0.013	0.305	585.414	0.862	0.431	0.046	0.172
ANAPC16	1612.581	0.527	0.260	0.043	0.103	1612.581	0.452	0.260	0.083	0.374	1612.581	-0.075	0.300	0.803	0.902
SFXN4	398.445	-0.542	0.219	0.013	0.042	398.445	-0.163	0.217	0.451	0.679	398.445	0.378	0.252	0.134	0.333
CLK2	1214.894	0.374	0.208	0.072	0.153	1214.894	0.163	0.208	0.433	0.667	1214.894	-0.211	0.240	0.380	0.610
C11orf74	181.733	0.848	0.264	0.001	0.007	181.733	0.332	0.266	0.212	0.492	181.733	-0.516	0.305	0.090	0.263
CLK3	1230.333	0.408	0.171	0.017	0.051	1230.333	0.372	0.171	0.030	0.322	1230.333	-0.036	0.198	0.854	0.930
SERPINA3	1392.882	1.716	0.778	0.027	0.073	1392.882	1.060	0.778	0.173	0.463	1392.882	-0.657	0.899	0.465	0.681
ADD3	512.276	0.248	0.479	0.605	0.718	512.276	0.655	0.478	0.171	0.460	512.276	0.408	0.553	0.461	0.678
TPP1	1889.995	0.873	0.327	0.008	0.028	1889.995	0.459	0.327	0.161	0.452	1889.995	-0.414	0.378	0.274	0.509
LARGE2	13.582	0.192	0.408	0.638	0.745	13.582	0.434	0.391	0.267	0.538	13.582	0.242	0.461	0.600	0.778
CLN3	635.148	0.881	0.191	0.000	0.000	635.148	0.276	0.192	0.150	0.443	635.148	-0.605	0.221	0.006	0.047
SLC36A4	554.400	-0.297	0.199	0.136	0.246	554.400	-0.030	0.198	0.881	0.940	554.400	0.267	0.230	0.245	0.476
TMEM45B	14.594	0.675	0.365	0.065	0.141	14.594	0.054	0.384	0.888	0.944	14.594	-0.620	0.431	0.150	0.359
CYP2R1	202.289	-0.544	0.193	0.005	0.019	202.289	0.092	0.187	0.621	0.791	202.289	0.637	0.221	0.004	0.035
CLN5	563.227	-0.288	0.207	0.165	0.284	563.227	0.039	0.206	0.851	0.923	563.227	0.326	0.239	0.172	0.389
PIH1D2	16.223	0.510	0.464	0.271	0.408	16.223	-0.802	0.504	0.112	0.405	16.223	-1.312	0.569	0.021	0.104
DNAJC24	342.989	-0.027	0.118	0.821	0.883	342.989	-0.104	0.117	0.376	0.624	342.989	-0.077	0.137	0.573	0.760
ARL14EP	645.688	-0.459	0.121	0.000	0.001	645.688	-0.133	0.119	0.265	0.537	645.688	0.326	0.140	0.020	0.100
CLNS1A	2777.625	-0.082	0.118	0.489	0.622	2777.625	-0.005	0.118	0.968	0.983	2777.625	0.077	0.137	0.572	0.760
DEPDC4	20.801	0.013	0.396	0.973	0.983	20.801	-0.111	0.395	0.779	0.883	20.801	-0.124	0.461	0.787	0.894
LRRK2	122.583	-0.947	0.605	0.118	0.221	122.583	0.635	0.599	0.289	0.558	122.583	1.581	0.697	0.023	0.111

CLPTM1	1962.815	0.251	0.112	0.025	0.068	1962.815	-0.103	0.112	0.357	0.611	1962.815	-0.354	0.129	0.006	0.048
TMEM52B	28.384	0.373	0.335	0.266	0.402	28.384	0.399	0.330	0.227	0.505	28.384	0.026	0.383	0.945	0.975
C12orf45	211.476	0.383	0.263	0.145	0.258	211.476	-0.240	0.265	0.365	0.617	211.476	-0.623	0.306	0.041	0.163
CLTA	3106.131	0.296	0.172	0.085	0.173	3106.131	-0.108	0.172	0.529	0.730	3106.131	-0.404	0.199	0.042	0.163
CLTB	1576.202	0.385	0.306	0.207	0.336	1576.202	-0.509	0.306	0.096	0.391	1576.202	-0.894	0.353	0.011	0.071
LRIG3	107.410	-0.652	0.313	0.037	0.092	107.410	0.011	0.306	0.973	0.985	107.410	0.663	0.359	0.065	0.215
TMEM132D	35.377	-1.450	0.338	0.000	0.000	35.377	0.064	0.287	0.824	0.908	35.377	1.513	0.377	0.000	0.002
SLC15A4	752.624	0.313	0.150	0.036	0.091	752.624	0.066	0.150	0.657	0.813	752.624	-0.247	0.173	0.154	0.364
RHEBL1	46.213	1.504	0.388	0.000	0.001	46.213	-0.491	0.421	0.244	0.519	46.213	-1.995	0.468	0.000	0.001
ZNF641	106.511	-0.896	0.272	0.001	0.006	106.511	-0.441	0.265	0.096	0.391	106.511	0.455	0.315	0.148	0.356
CLTC	6106.674	-0.484	0.220	0.028	0.075	6106.674	-0.407	0.220	0.065	0.353	6106.674	0.077	0.254	0.763	0.880
NEDD1	801.620	-0.110	0.191	0.566	0.686	801.620	-0.462	0.191	0.016	0.306	801.620	-0.353	0.221	0.111	0.298
IKBIP	1564.789	0.338	0.152	0.027	0.072	1564.789	0.048	0.153	0.752	0.867	1564.789	-0.289	0.176	0.100	0.281
FGD4	679.484	-0.813	0.235	0.001	0.004	679.484	0.174	0.233	0.454	0.681	679.484	0.987	0.270	0.000	0.005
AEBP2	641.111	-0.298	0.172	0.083	0.170	641.111	-0.144	0.171	0.400	0.643	641.111	0.154	0.199	0.438	0.659
ALKBH2	751.507	0.227	0.240	0.344	0.485	751.507	0.090	0.240	0.709	0.843	751.507	-0.137	0.277	0.620	0.792
SPPL3	540.068	0.231	0.110	0.035	0.088	540.068	0.243	0.109	0.026	0.316	540.068	0.011	0.126	0.929	0.968
TDRD9	18.008	-1.641	0.851	0.054	0.122	18.008	0.855	0.780	0.273	0.544	18.008	2.495	0.958	0.009	0.062
ANKRD9	376.544	1.094	0.246	0.000	0.000	376.544	-0.359	0.250	0.151	0.443	376.544	-1.453	0.286	0.000	0.000
AK7	12.164	0.404	0.863	0.640	0.747	12.164	0.393	0.861	0.648	0.808	12.164	-0.010	0.994	0.992	0.995
IFI27L1	290.325	1.082	0.334	0.001	0.007	290.325	0.343	0.335	0.306	0.572	290.325	-0.740	0.386	0.055	0.195
C14orf28	47.803	0.301	0.367	0.412	0.550	47.803	0.333	0.363	0.358	0.611	47.803	0.032	0.421	0.938	0.972
TRAPPC6B	353.769	-0.786	0.187	0.000	0.000	353.769	-0.396	0.185	0.032	0.322	353.769	0.389	0.217	0.072	0.230
CLBA1	263.413	0.005	0.184	0.978	0.986	263.413	0.020	0.183	0.913	0.957	263.413	0.015	0.213	0.944	0.974
PLD4	661.620	2.767	0.553	0.000	0.000	661.620	1.153	0.554	0.037	0.326	661.620	-1.614	0.638	0.011	0.071
ADSS1	1591.616	-0.039	1.093	0.971	0.982	1591.616	0.872	1.093	0.425	0.661	1591.616	0.911	1.262	0.470	0.685
MRPL52	426.334	0.080	0.255	0.753	0.835	426.334	-0.247	0.256	0.334	0.593	426.334	-0.327	0.295	0.268	0.503
LRR1	274.958	-0.901	0.425	0.034	0.087	274.958	-1.214	0.425	0.004	0.297	274.958	-0.313	0.492	0.525	0.726
KLHDC1	8.199	-0.413	1.004	0.681	0.779	8.199	0.289	0.975	0.767	0.877	8.199	0.702	1.149	0.541	0.738
FRMD6	1039.768	-0.947	0.268	0.000	0.003	1039.768	-0.562	0.268	0.036	0.324	1039.768	0.385	0.310	0.214	0.440
SOCS4	352.465	-0.795	0.342	0.020	0.058	352.465	-0.869	0.342	0.011	0.302	352.465	-0.074	0.396	0.853	0.930
NAA30	632.808	-0.663	0.221	0.003	0.013	632.808	-0.204	0.220	0.353	0.607	632.808	0.458	0.256	0.073	0.231
JDP2	2118.587	0.046	0.194	0.814	0.878	2118.587	-0.122	0.194	0.528	0.729	2118.587	-0.168	0.224	0.453	0.672
ISCA2	218.763	0.501	0.195	0.010	0.035	218.763	0.093	0.197	0.635	0.801	218.763	-0.408	0.226	0.071	0.228
PLIN2	24.311	1.757	0.581	0.003	0.012	24.311	1.198	0.593	0.043	0.331	24.311	-0.560	0.644	0.385	0.614
TTC8	201.309	0.267	0.193	0.165	0.284	201.309	0.259	0.192	0.177	0.466	201.309	-0.009	0.222	0.968	0.985
TC2N	30.981	0.577	0.337	0.087	0.176	30.981	0.847	0.328	0.010	0.298	30.981	0.270	0.378	0.475	0.688
SLC25A29	203.325	1.678	0.160	0.000	0.000	203.325	0.212	0.172	0.218	0.497	203.325	-1.466	0.188	0.000	0.000
DEGS2	85.677	2.267	0.376	0.000	0.000	85.677	0.196	0.389	0.614	0.787	85.677	-2.071	0.438	0.000	0.000
LEO1	805.137	-0.492	0.146	0.001	0.005	805.137	-0.284	0.145	0.050	0.337	805.137	0.207	0.169	0.220	0.446
C15orf40	392.199	-0.079	0.140	0.573	0.692	392.199	-0.017	0.139	0.903	0.951	392.199	0.062	0.162	0.702	0.845
SENP8	75.104	-0.078	0.270	0.773	0.849	75.104	-0.070	0.268	0.793	0.892	75.104	0.008	0.312	0.981	0.990
MTFMT	304.098	-0.335	0.171	0.050	0.116	304.098	-0.084	0.169	0.620	0.790	304.098	0.251	0.198	0.204	0.427
TARS3	215.880	-0.236	0.227	0.298	0.437	215.880	0.281	0.223	0.207	0.489	215.880	0.518	0.261	0.047	0.176
LRRC28	117.229	-0.040	0.227	0.860	0.908	117.229	0.267	0.222	0.229	0.507	117.229	0.307	0.260	0.237	0.466
TMEM266	11.072	1.211	0.744	0.104	0.201	11.072	0.733	0.751	0.329	0.590	11.072	-0.478	0.852	0.575	0.761
NIPA1	796.092	-0.442	0.212	0.037	0.091	796.092	-0.304	0.211	0.150	0.442	796.092	0.138	0.245	0.573	0.760
HYKK	21.528	0.817	0.449	0.069	0.147	21.528	0.155	0.459	0.736	0.859	21.528	-0.662	0.522	0.205	0.428
WHAMM	207.775	0.067	0.222	0.762	0.842	207.775	0.037	0.221	0.867	0.932	207.775	-0.030	0.257	0.906	0.957
FSD2	28.833	0.414	0.347	0.233	0.365	28.833	0.423	0.343	0.218	0.497	28.833	0.008	0.396	0.983	0.991
C16orf46	24.357	-0.651	0.418	0.119	0.222	24.357	0.222	0.385	0.564	0.752	24.357	0.873	0.473	0.065	0.214
NTAN1	684.777	0.479	0.191	0.012	0.039	684.777	0.349	0.191	0.068	0.356	684.777	-0.130	0.220	0.555	0.747
FOPNL	1522.362	-0.314	0.250	0.209	0.337	1522.362	0.301	0.249	0.227	0.506	1522.362	0.616	0.288	0.033	0.139
DNAAF1	64.021	1.096	0.380	0.004	0.016	64.021	0.207	0.387	0.593	0.774	64.021	-0.889	0.441	0.044	0.168
DCUN1D3	718.249	-0.414	0.178	0.020	0.058	718.249	-0.309	0.178	0.082	0.373	718.249	0.105	0.206	0.610	0.785
CMTM3	221.491	0.236	0.161	0.142	0.254	221.491	-0.028	0.162	0.860	0.929	221.491	-0.265	0.187	0.156	0.368
SPATA2L	269.707	0.040	0.227	0.860	0.908	269.707	-0.136	0.227	0.550	0.743	269.707	-0.176	0.263	0.503	0.711
SPATA33	70.572	0.173	0.315	0.584	0.700	70.572	-0.641	0.324	0.048	0.337	70.572	-0.814	0.373	0.029	0.128
NOXO1	45.696	-0.409	0.542	0.450	0.586	45.696	-1.206	0.553	0.029	0.322	45.696	-0.796	0.640	0.213	0.439
CCDC78	23.459	1.181	0.407	0.004	0.016	23.459	0.070	0.432	0.872	0.935	23.459	-1.111	0.480	0.020	0.103
LTB4R	39.507	2.213	0.542	0.000	0.000	39.507	0.373	0.559	0.504	0.715	39.507	-1.840	0.629	0.003	0.032
IQCK	125.948	0.425	0.205	0.038	0.094	125.948	0.112	0.206	0.586	0.768	125.948	-0.313	0.237	0.187	0.407
ZG16B	54.099	0.209	0.370	0.573	0.692	54.099	0.552	0.362	0.127	0.420	54.099	0.343	0.422	0.416	0.641
PAQR4	641.971	0.161	0.395	0.683	0.781	641.971	-0.841	0.396	0.034	0.322	641.971	-1.002	0.457	0.028	0.126
ZC3H18	1369.538	-0.339	0.264	0.199	0.325	1369.538	-0.597	0.264	0.024	0.316	1369.538	-0.258	0.305	0.397	0.625
CDYL2	207.009	-0.697	0.297	0.019	0.055	207.009	-0.236	0.294	0.422	0.658	207.009	0.461	0.343	0.179	0.398
ABCC2	258.581	0.828	0.366	0.024	0.065	258.581	0.574	0.366	0.117	0.409	258.581	-0.255	0.422	0.546	0.742

ANKS3	182.409	0.435	0.315	0.167	0.286	182.409	-0.010	0.316	0.975	0.986	182.409	-0.445	0.364	0.222	0.449
UBALD1	654.303	0.019	0.286	0.947	0.967	654.303	-0.459	0.286	0.109	0.401	654.303	-0.478	0.331	0.148	0.356
ZNF720	151.401	-0.039	0.184	0.831	0.888	151.401	0.064	0.181	0.723	0.852	151.401	0.103	0.212	0.625	0.795
TMEM219	634.486	1.406	0.340	0.000	0.000	634.486	0.670	0.341	0.049	0.337	634.486	-0.736	0.392	0.061	0.207
EARS2	579.031	-0.829	0.196	0.000	0.000	579.031	-0.314	0.194	0.106	0.398	579.031	0.515	0.226	0.023	0.111
TMEM170A	280.186	0.009	0.152	0.955	0.973	280.186	0.223	0.150	0.137	0.429	280.186	0.214	0.175	0.221	0.449
METTL23	447.786	-0.074	0.130	0.567	0.687	447.786	0.098	0.128	0.443	0.674	447.786	0.173	0.149	0.248	0.480
MSI2	1561.190	-0.350	0.149	0.019	0.055	1561.190	-0.045	0.148	0.761	0.873	1561.190	0.305	0.172	0.076	0.237
SLC38A10	1500.679	0.599	0.135	0.000	0.000	1500.679	0.045	0.136	0.740	0.861	1500.679	-0.554	0.156	0.000	0.007
CANT1	1172.001	-0.316	0.193	0.101	0.197	1172.001	0.035	0.192	0.856	0.927	1172.001	0.351	0.223	0.115	0.303
CYB5D1	186.502	-0.499	0.192	0.009	0.032	186.502	-0.173	0.188	0.355	0.609	186.502	0.326	0.221	0.141	0.345
USP43	8.765	-0.013	0.662	0.984	0.991	8.765	-0.058	0.657	0.929	0.964	8.765	-0.045	0.768	0.953	0.978
KRBA2	11.567	1.120	0.560	0.045	0.107	11.567	0.978	0.559	0.080	0.371	11.567	-0.142	0.630	0.822	0.912
HEXIM2	156.333	0.339	0.387	0.382	0.522	156.333	0.634	0.385	0.100	0.394	156.333	0.296	0.446	0.507	0.713
LSM12	1419.134	-0.010	0.222	0.963	0.978	1419.134	-0.334	0.222	0.133	0.424	1419.134	-0.324	0.257	0.207	0.431
CCDC43	540.896	-0.333	0.123	0.007	0.025	540.896	-0.136	0.122	0.264	0.536	540.896	0.197	0.143	0.167	0.381
CNTD1	341.392	0.171	0.142	0.228	0.359	341.392	-0.100	0.142	0.482	0.700	341.392	-0.271	0.165	0.100	0.280
FLJ40194	83.930	0.741	0.195	0.000	0.001	83.930	0.248	0.199	0.212	0.492	83.930	-0.493	0.225	0.029	0.128
B4GALNT2	15.693	0.074	0.757	0.922	0.951	15.693	0.716	0.744	0.336	0.594	15.693	0.642	0.866	0.459	0.676
RSKR	48.159	1.320	0.370	0.000	0.003	48.159	-0.204	0.387	0.598	0.777	48.159	-1.525	0.436	0.000	0.008
ANKRD13B	574.335	-0.113	0.168	0.500	0.631	574.335	0.168	0.167	0.314	0.577	574.335	0.281	0.193	0.147	0.354
SLC43A2	492.345	1.015	0.167	0.000	0.000	492.345	-0.338	0.170	0.047	0.336	492.345	-1.352	0.194	0.000	0.000
CYB5D2	224.668	-0.294	0.435	0.499	0.630	224.668	0.594	0.432	0.170	0.459	224.668	0.888	0.501	0.077	0.238
SPNS2	41.787	0.677	0.401	0.091	0.183	41.787	-0.259	0.413	0.530	0.731	41.787	-0.936	0.471	0.047	0.175
MRPL10	1206.518	-0.111	0.208	0.594	0.708	1206.518	0.319	0.207	0.124	0.416	1206.518	0.430	0.240	0.073	0.231
WDR81	750.111	0.125	0.186	0.504	0.634	750.111	0.174	0.186	0.349	0.604	750.111	0.050	0.215	0.818	0.910
RN7SK	59.605	0.750	0.321	0.020	0.057	59.605	0.178	0.333	0.593	0.774	59.605	-0.572	0.375	0.127	0.322
TBC1D16	3467.609	-0.105	0.314	0.739	0.823	3467.609	0.241	0.314	0.444	0.675	3467.609	0.345	0.363	0.341	0.575
AFMID	361.891	-0.080	0.186	0.666	0.767	361.891	0.327	0.184	0.075	0.364	361.891	0.408	0.214	0.057	0.199
GJD3	208.992	0.040	0.191	0.833	0.890	208.992	-0.032	0.191	0.866	0.932	208.992	-0.073	0.222	0.743	0.868
KRT222	6.204	-0.243	0.696	0.728	0.815	6.204	0.067	0.672	0.921	0.960	6.204	0.310	0.800	0.699	0.843
SNHG29	13812.706	0.000	0.364	1.000	1.000	13812.706	0.569	0.364	0.118	0.410	13812.706	0.569	0.421	0.176	0.394
ZSWIM7	91.171	0.172	0.233	0.462	0.596	91.171	0.162	0.231	0.484	0.701	91.171	-0.010	0.269	0.971	0.986
MIEF2	72.660	0.419	0.428	0.327	0.467	72.660	0.624	0.425	0.142	0.433	72.660	0.205	0.491	0.676	0.828
SLC5A10	112.371	0.457	0.265	0.084	0.172	112.371	0.144	0.266	0.588	0.769	112.371	-0.313	0.306	0.307	0.542
FAM210A	802.352	-0.244	0.221	0.269	0.406	802.352	-0.249	0.220	0.259	0.531	802.352	-0.005	0.255	0.984	0.991
INO80C	331.926	-0.010	0.178	0.956	0.973	331.926	-0.126	0.178	0.480	0.700	331.926	-0.116	0.207	0.575	0.761
TTC39C	396.170	-0.130	0.204	0.525	0.652	396.170	0.207	0.202	0.307	0.572	396.170	0.336	0.235	0.152	0.361
CLDND2	8.748	0.231	0.720	0.748	0.831	8.748	1.066	0.680	0.117	0.409	8.748	0.835	0.801	0.297	0.533
ZNF816	66.771	-0.272	0.265	0.305	0.444	66.771	0.166	0.256	0.516	0.721	66.771	0.438	0.303	0.149	0.357
ZNF543	67.143	0.305	0.244	0.210	0.339	67.143	0.314	0.241	0.193	0.478	67.143	0.008	0.280	0.976	0.988
RAVER1	1392.914	0.118	0.123	0.335	0.475	1392.914	-0.100	0.123	0.417	0.655	1392.914	-0.218	0.142	0.124	0.318
MICOS13	844.208	0.648	0.249	0.009	0.032	844.208	-0.021	0.250	0.934	0.967	844.208	-0.668	0.288	0.020	0.102
TRAPPC5	1049.922	0.925	0.269	0.001	0.004	1049.922	0.403	0.269	0.134	0.426	1049.922	-0.522	0.311	0.093	0.267
PCP2	13.961	2.191	0.736	0.003	0.013	13.961	1.214	0.751	0.106	0.398	13.961	-0.977	0.831	0.240	0.470
OSCAR	25.877	1.293	0.740	0.080	0.166	25.877	1.125	0.740	0.128	0.420	25.877	-0.168	0.839	0.841	0.923
ZNF813	26.959	0.527	0.374	0.159	0.277	26.959	0.304	0.375	0.419	0.656	26.959	-0.223	0.431	0.604	0.782
ZNF441	44.964	-0.522	0.366	0.154	0.270	44.964	-0.481	0.363	0.186	0.472	44.964	0.041	0.427	0.923	0.964
ZNF440	70.749	0.149	0.288	0.605	0.718	70.749	0.449	0.282	0.112	0.405	70.749	0.301	0.329	0.361	0.594
SWSAP1	30.363	0.324	0.364	0.373	0.514	30.363	0.325	0.360	0.366	0.617	30.363	0.002	0.417	0.997	0.998
CCDC159	899.949	0.316	0.202	0.118	0.221	899.949	0.488	0.201	0.015	0.306	899.949	0.172	0.233	0.460	0.677
JOSD2	599.580	0.620	0.248	0.012	0.040	599.580	0.412	0.248	0.097	0.392	599.580	-0.208	0.286	0.467	0.682
CPT1C	15.058	2.321	0.577	0.000	0.001	15.058	0.816	0.611	0.182	0.469	15.058	-1.505	0.658	0.022	0.108
ALDH16A1	608.001	0.365	0.202	0.071	0.151	608.001	-0.088	0.203	0.665	0.818	608.001	-0.453	0.234	0.053	0.190
ZNF787	1293.177	-0.163	0.248	0.510	0.640	1293.177	-0.314	0.248	0.206	0.488	1293.177	-0.150	0.287	0.601	0.779
ZNF573	9.509	-0.496	0.491	0.312	0.452	9.509	-0.227	0.465	0.625	0.794	9.509	0.269	0.569	0.637	0.803
EID2B	78.409	0.216	0.235	0.357	0.497	78.409	0.150	0.234	0.521	0.725	78.409	-0.067	0.271	0.806	0.903
TNFAIP8L1	451.474	0.478	0.355	0.178	0.300	451.474	0.459	0.354	0.195	0.478	451.474	-0.018	0.409	0.965	0.984
ZNF57	130.845	-0.038	0.187	0.839	0.894	130.845	0.096	0.184	0.602	0.780	130.845	0.134	0.215	0.535	0.733
IRGQ	168.678	0.389	0.226	0.085	0.174	168.678	-0.356	0.230	0.122	0.413	168.678	-0.745	0.264	0.005	0.040
ZNF428	587.341	1.273	0.281	0.000	0.000	587.341	0.225	0.282	0.425	0.661	587.341	-1.047	0.325	0.001	0.016
PLK3	414.821	-0.385	0.419	0.358	0.498	414.821	-0.934	0.419	0.026	0.316	414.821	-0.549	0.485	0.257	0.491
JSRP1	5003.573	-0.150	0.156	0.337	0.477	5003.573	-0.056	0.156	0.722	0.851	5003.573	0.094	0.180	0.601	0.779
MOB3A	1629.172	0.476	0.423	0.260	0.395	1629.172	0.570	0.423	0.178	0.466	1629.172	0.093	0.488	0.848	0.926
MFSD12	2303.703	0.136	0.237	0.566	0.686	2303.703	-0.398	0.238	0.094	0.388	2303.703	-0.535	0.274	0.051	0.186
NDUFA11	321.920	0.842	0.278	0.002	0.011	321.920	0.186	0.280	0.505	0.715	321.920	-0.656	0.322	0.042	0.163
MISP	59.174	0.464	0.262	0.076	0.159	59.174	0.570	0.257	0.027	0.319	59.174	0.105	0.297	0.723	0.857

WTIP	15.984	1.340	0.458	0.003	0.015	15.984	0.413	0.484	0.393	0.637	15.984	-0.927	0.530	0.080	0.245
ZNF792	25.158	0.080	0.676	0.906	0.940	25.158	0.620	0.667	0.353	0.607	25.158	0.540	0.775	0.486	0.697
NR2C2AP	476.326	-0.122	0.337	0.717	0.807	476.326	-0.382	0.337	0.258	0.530	476.326	-0.259	0.390	0.506	0.712
RINL	129.257	0.483	0.219	0.028	0.073	129.257	0.389	0.218	0.075	0.364	129.257	-0.094	0.252	0.709	0.849
CNN2	3707.134	0.596	0.179	0.001	0.005	3707.134	0.186	0.179	0.301	0.567	3707.134	-0.411	0.207	0.047	0.176
C19orf47	195.873	-0.261	0.333	0.433	0.569	195.873	-0.808	0.335	0.016	0.306	195.873	-0.547	0.387	0.158	0.370
CNN3	591.501	0.347	0.280	0.215	0.345	591.501	-0.560	0.281	0.046	0.335	591.501	-0.907	0.324	0.005	0.042
GABPB2	171.495	0.144	0.233	0.537	0.662	171.495	0.499	0.230	0.030	0.322	171.495	0.355	0.267	0.183	0.402
CCDC163	51.172	-0.042	0.397	0.917	0.947	51.172	0.293	0.389	0.451	0.679	51.172	0.335	0.456	0.462	0.679
CNP	1345.616	0.359	0.128	0.005	0.020	1345.616	0.023	0.129	0.856	0.927	1345.616	-0.335	0.148	0.024	0.114
CCSAP	529.538	-0.541	0.292	0.064	0.139	529.538	0.132	0.290	0.649	0.808	529.538	0.673	0.336	0.045	0.172
PUSL1	255.593	0.085	0.317	0.789	0.860	255.593	-0.535	0.319	0.094	0.388	255.593	-0.620	0.369	0.092	0.267
B3GALT6	572.240	0.377	0.224	0.093	0.185	572.240	-0.141	0.225	0.530	0.730	572.240	-0.518	0.259	0.046	0.173
CNR1	1952.748	-0.750	0.474	0.114	0.216	1952.748	0.536	0.474	0.258	0.530	1952.748	1.286	0.548	0.019	0.098
WDR63	9.120	0.748	0.629	0.234	0.367	9.120	0.104	0.647	0.872	0.935	9.120	-0.644	0.733	0.380	0.610
MAB21L3	104.124	-0.321	0.426	0.452	0.587	104.124	-0.190	0.424	0.654	0.811	104.124	0.130	0.492	0.791	0.896
IFFO2	374.864	-0.089	0.164	0.586	0.703	374.864	-0.032	0.163	0.845	0.919	374.864	0.057	0.190	0.762	0.879
H3C14	59.006	-0.096	0.467	0.838	0.893	59.006	0.435	0.461	0.345	0.602	59.006	0.531	0.536	0.322	0.557
SLC44A3	10.909	0.671	0.454	0.139	0.250	10.909	0.055	0.474	0.908	0.954	10.909	-0.616	0.533	0.248	0.480
ATXN7L2	139.587	-0.129	0.222	0.561	0.682	139.587	-0.055	0.220	0.803	0.897	139.587	0.074	0.257	0.772	0.884
LYPLAL1	236.655	0.290	0.240	0.225	0.357	236.655	-0.219	0.241	0.363	0.615	236.655	-0.510	0.278	0.067	0.218
TYW3	715.271	-0.539	0.175	0.002	0.010	715.271	-0.181	0.174	0.298	0.565	715.271	0.359	0.202	0.075	0.236
ERICH3	7.746	0.110	0.832	0.894	0.932	7.746	0.823	0.806	0.307	0.572	7.746	0.713	0.944	0.450	0.669
LRRIQ3	25.135	-0.682	0.327	0.037	0.092	25.135	0.325	0.290	0.263	0.535	25.135	1.007	0.366	0.006	0.046
TPRG1L	882.703	0.349	0.217	0.109	0.208	882.703	0.112	0.217	0.605	0.782	882.703	-0.236	0.251	0.347	0.581
PRXL2B	898.052	0.409	0.249	0.100	0.196	898.052	-0.508	0.250	0.042	0.330	898.052	-0.917	0.288	0.001	0.017
MYOM3	52.372	0.258	0.450	0.566	0.687	52.372	0.451	0.446	0.312	0.575	52.372	0.193	0.517	0.709	0.849
DMBX1	33.110	0.514	0.404	0.203	0.330	33.110	-0.168	0.412	0.684	0.829	33.110	-0.682	0.472	0.149	0.357
ZNF684	32.925	-0.034	0.297	0.909	0.942	32.925	-0.631	0.308	0.040	0.328	32.925	-0.596	0.356	0.094	0.270
TCEANC2	203.602	-0.176	0.153	0.248	0.382	203.602	-0.076	0.151	0.611	0.786	203.602	0.100	0.177	0.572	0.760
LRRC39	54.074	-0.517	0.278	0.063	0.138	54.074	-0.074	0.267	0.781	0.885	54.074	0.443	0.319	0.166	0.381
RNF19B	764.514	-0.809	0.286	0.005	0.018	764.514	-0.271	0.284	0.340	0.598	764.514	0.538	0.330	0.103	0.285
DNAH14	130.083	-0.123	0.293	0.674	0.773	130.083	-0.474	0.294	0.108	0.400	130.083	-0.350	0.341	0.305	0.540
C1orf122	539.499	-0.393	0.366	0.283	0.421	539.499	-0.475	0.366	0.194	0.478	539.499	-0.082	0.423	0.846	0.926
COL1A1	983.765	0.020	0.341	0.952	0.971	983.765	0.529	0.341	0.120	0.412	983.765	0.509	0.394	0.196	0.419
OSCP1	95.206	0.582	0.394	0.140	0.251	95.206	0.554	0.393	0.159	0.451	95.206	-0.027	0.453	0.952	0.978
C1orf216	251.787	-0.028	0.179	0.876	0.920	251.787	-0.472	0.181	0.009	0.297	251.787	-0.444	0.210	0.035	0.144
ARL8A	642.071	0.604	0.153	0.000	0.001	642.071	0.184	0.154	0.231	0.508	642.071	-0.420	0.177	0.017	0.094
UHMK1	1774.863	-0.165	0.225	0.464	0.598	1774.863	-0.215	0.225	0.339	0.597	1774.863	-0.050	0.260	0.846	0.926
FCRLB	36.755	-0.037	0.316	0.906	0.940	36.755	-0.445	0.321	0.166	0.456	36.755	-0.408	0.374	0.275	0.510
ADH5	2969.289	0.366	0.138	0.008	0.028	2969.289	0.064	0.138	0.644	0.806	2969.289	-0.303	0.159	0.057	0.199
C1orf131	273.356	0.104	0.205	0.613	0.725	273.356	-0.295	0.207	0.153	0.445	273.356	-0.399	0.239	0.095	0.271
LIX1L	1126.115	0.056	0.224	0.803	0.870	1126.115	0.469	0.223	0.035	0.324	1126.115	0.414	0.258	0.109	0.295
COL3A1	11.076	0.180	0.870	0.836	0.892	11.076	-0.799	0.893	0.371	0.620	11.076	-0.980	1.027	0.340	0.574
SPATA17	48.642	0.364	0.580	0.530	0.656	48.642	0.833	0.575	0.148	0.440	48.642	0.469	0.666	0.481	0.694
EDARADD	58.834	0.424	0.255	0.096	0.190	58.834	0.171	0.256	0.503	0.715	58.834	-0.253	0.295	0.391	0.619
COL4A1	1487.493	0.442	0.177	0.012	0.040	1487.493	-0.264	0.177	0.136	0.427	1487.493	-0.706	0.204	0.001	0.009
KLF17	9.094	1.239	0.505	0.014	0.044	9.094	0.604	0.524	0.249	0.523	9.094	-0.635	0.575	0.269	0.504
TMEM125	11.599	1.115	0.455	0.014	0.045	11.599	0.410	0.476	0.389	0.635	11.599	-0.705	0.525	0.179	0.398
IQGAP3	909.828	0.055	0.209	0.794	0.864	909.828	0.298	0.208	0.153	0.445	909.828	0.243	0.241	0.312	0.547
NAXE	1331.610	0.156	0.224	0.486	0.619	1331.610	-0.190	0.224	0.397	0.641	1331.610	-0.346	0.259	0.181	0.400
ARHGEF19	129.357	0.099	0.257	0.702	0.795	129.357	0.338	0.254	0.184	0.471	129.357	0.239	0.295	0.418	0.643
MRPL55	243.782	0.624	0.256	0.015	0.046	243.782	-0.110	0.259	0.671	0.822	243.782	-0.734	0.297	0.014	0.080
H2BU1	14.086	0.030	0.612	0.961	0.977	14.086	-0.023	0.607	0.969	0.984	14.086	-0.054	0.709	0.940	0.973
DRAM2	902.504	0.205	0.273	0.453	0.587	902.504	0.352	0.272	0.197	0.480	902.504	0.147	0.315	0.641	0.805
TATDN3	241.741	-0.112	0.265	0.673	0.773	241.741	-0.500	0.266	0.060	0.350	241.741	-0.388	0.308	0.208	0.433
COL4A2	3510.727	0.715	0.124	0.000	0.000	3510.727	0.089	0.124	0.474	0.696	3510.727	-0.626	0.143	0.000	0.000
VSTM2L	155.767	1.526	0.493	0.002	0.010	155.767	0.830	0.495	0.093	0.388	155.767	-0.697	0.568	0.220	0.447
SNHG11	81.558	-0.038	0.289	0.896	0.932	81.558	0.132	0.284	0.643	0.805	81.558	0.170	0.332	0.610	0.785
FITM2	252.183	-0.442	0.253	0.081	0.166	252.183	0.074	0.250	0.768	0.877	252.183	0.516	0.292	0.077	0.239
TSHZ2	296.173	1.054	0.508	0.038	0.094	296.173	0.676	0.508	0.183	0.470	296.173	-0.377	0.586	0.520	0.723
COL4A4	15.643	2.127	0.697	0.002	0.011	15.643	0.595	0.718	0.407	0.648	15.643	-1.532	0.804	0.057	0.199
TBC1D20	593.600	0.203	0.177	0.252	0.386	593.600	-0.100	0.177	0.573	0.758	593.600	-0.303	0.205	0.140	0.343
COL4A5	1711.529	-0.200	0.331	0.546	0.670	1711.529	0.430	0.331	0.194	0.478	1711.529	0.630	0.383	0.100	0.279
SLX4IP	179.231	0.057	0.235	0.808	0.873	179.231	0.296	0.233	0.203	0.486	179.231	0.239	0.270	0.376	0.607
COL4A6	1982.557	-1.515	0.270	0.000	0.000	1982.557	0.262	0.269	0.331	0.590	1982.557	1.776	0.311	0.000	0.000
CHMP4B	1686.072	0.249	0.166	0.135	0.245	1686.072	-0.113	0.167	0.499	0.713	1686.072	-0.361	0.192	0.060	0.206

PIGU	273.100	0.377	0.151	0.013	0.041	273.100	0.091	0.152	0.551	0.744	273.100	-0.286	0.175	0.102	0.285
COL5A1	1142.211	1.382	0.447	0.002	0.010	1142.211	0.598	0.447	0.181	0.469	1142.211	-0.784	0.516	0.128	0.324
C22orf39	297.115	-0.346	0.164	0.035	0.088	297.115	-0.095	0.162	0.556	0.747	297.115	0.251	0.190	0.186	0.405
TANGO2	196.820	0.267	0.341	0.435	0.571	196.820	-0.687	0.345	0.046	0.335	196.820	-0.953	0.397	0.016	0.090
COL5A2	1347.133	0.114	0.390	0.770	0.847	1347.133	0.396	0.390	0.310	0.574	1347.133	0.282	0.450	0.531	0.731
COL6A1	278.091	0.759	0.732	0.300	0.438	278.091	0.698	0.732	0.341	0.599	278.091	-0.062	0.845	0.942	0.974
ANKRD54	480.255	-0.184	0.257	0.473	0.607	480.255	-0.364	0.257	0.156	0.448	480.255	-0.180	0.297	0.544	0.740
COL6A2	190.408	0.883	0.385	0.022	0.061	190.408	0.819	0.384	0.033	0.322	190.408	-0.063	0.443	0.886	0.945
PPP1R21	269.837	0.070	0.120	0.558	0.680	269.837	-0.087	0.120	0.470	0.692	269.837	-0.157	0.139	0.261	0.495
TRABD2A	2137.491	0.229	0.249	0.359	0.500	2137.491	-0.108	0.249	0.665	0.818	2137.491	-0.337	0.288	0.242	0.473
COL6A3	717.286	-0.225	0.258	0.383	0.522	717.286	0.170	0.257	0.509	0.718	717.286	0.395	0.298	0.185	0.404
TMEM150A	255.291	0.906	0.232	0.000	0.001	255.291	0.371	0.233	0.112	0.405	255.291	-0.534	0.267	0.046	0.172
COL7A1	1673.407	1.019	0.506	0.044	0.105	1673.407	1.069	0.506	0.035	0.322	1673.407	0.051	0.584	0.931	0.969
NUP35	181.133	-0.554	0.284	0.051	0.117	181.133	-0.625	0.283	0.027	0.319	181.133	-0.071	0.330	0.828	0.916
TYW5	168.782	-0.397	0.212	0.061	0.134	168.782	-0.314	0.210	0.134	0.426	168.782	0.083	0.246	0.735	0.864
COL8A1	9681.007	0.124	0.286	0.663	0.765	9681.007	-0.361	0.286	0.207	0.489	9681.007	-0.485	0.330	0.142	0.346
MITD1	365.153	0.779	0.199	0.000	0.001	365.153	0.013	0.201	0.949	0.975	365.153	-0.766	0.231	0.001	0.012
DIS3L2	271.826	0.132	0.233	0.572	0.691	271.826	-0.339	0.234	0.148	0.440	271.826	-0.470	0.270	0.082	0.248
MBOAT2	526.227	0.286	0.258	0.268	0.404	526.227	0.339	0.258	0.189	0.475	526.227	0.053	0.298	0.860	0.933
TAF8	493.256	0.407	0.158	0.010	0.035	493.256	0.381	0.158	0.016	0.306	493.256	-0.026	0.182	0.888	0.946
TMEM18	445.610	0.073	0.201	0.716	0.806	445.610	0.212	0.200	0.290	0.558	445.610	0.138	0.232	0.550	0.744
RBM45	98.423	-0.430	0.265	0.105	0.202	98.423	-0.484	0.264	0.067	0.354	98.423	-0.054	0.309	0.862	0.934
BBS5	53.149	-0.237	0.286	0.407	0.545	53.149	-0.400	0.286	0.162	0.453	53.149	-0.162	0.335	0.629	0.798
COL9A3	9.924	1.451	0.722	0.044	0.105	9.924	1.388	0.719	0.054	0.342	9.924	-0.063	0.808	0.937	0.972
AADAC	7.508	1.522	0.827	0.066	0.142	7.508	1.165	0.833	0.162	0.453	7.508	-0.358	0.927	0.700	0.843
ICA1L	67.985	-0.506	0.395	0.200	0.326	67.985	0.129	0.387	0.738	0.860	67.985	0.635	0.454	0.162	0.375
FAM168B	2188.259	-0.829	0.196	0.000	0.000	2188.259	-0.391	0.195	0.045	0.334	2188.259	0.438	0.226	0.053	0.189
CLHC1	57.793	0.040	0.270	0.883	0.924	57.793	0.330	0.263	0.210	0.491	57.793	0.290	0.309	0.348	0.582
COL11A2	33.687	1.091	0.770	0.157	0.274	33.687	0.209	0.778	0.788	0.889	33.687	-0.881	0.891	0.323	0.557
PLEKHH2	17.569	-0.244	0.726	0.737	0.822	17.569	0.500	0.711	0.482	0.700	17.569	0.745	0.832	0.371	0.602
COL12A1	1938.854	0.169	0.257	0.510	0.639	1938.854	-0.015	0.257	0.952	0.976	1938.854	-0.185	0.297	0.533	0.732
AP1S3	162.848	0.132	0.273	0.629	0.739	162.848	-0.584	0.276	0.035	0.322	162.848	-0.716	0.319	0.025	0.116
C2orf76	87.811	0.343	0.237	0.147	0.261	87.811	-0.013	0.239	0.956	0.978	87.811	-0.357	0.276	0.196	0.419
SGPP2	50.495	-1.014	0.454	0.026	0.069	50.495	0.470	0.429	0.273	0.544	50.495	1.484	0.516	0.004	0.035
ACVR1C	24.753	-0.963	0.589	0.102	0.198	24.753	0.254	0.558	0.649	0.808	24.753	1.217	0.670	0.070	0.225
COL13A1	10.006	1.882	0.920	0.041	0.099	10.006	2.389	0.905	0.008	0.297	10.006	0.508	1.006	0.614	0.787
TTC32	63.840	0.188	0.317	0.554	0.676	63.840	0.306	0.314	0.329	0.590	63.840	0.119	0.365	0.745	0.869
UBR3	736.762	-0.732	0.273	0.007	0.027	736.762	0.067	0.272	0.804	0.898	736.762	0.799	0.315	0.011	0.070
KCTD18	324.360	-0.350	0.313	0.262	0.398	324.360	0.328	0.310	0.291	0.558	324.360	0.678	0.360	0.060	0.204
ZNF513	99.472	0.360	0.222	0.105	0.202	99.472	0.369	0.220	0.093	0.388	99.472	0.009	0.254	0.972	0.986
LYPD6	10.326	1.157	0.473	0.014	0.045	10.326	-0.167	0.520	0.748	0.866	10.326	-1.324	0.571	0.020	0.103
LYPD6B	8.491	-0.069	0.563	0.902	0.937	8.491	0.093	0.544	0.864	0.931	8.491	0.162	0.647	0.802	0.901
TMEM198	55.387	0.509	0.302	0.092	0.184	55.387	0.528	0.299	0.077	0.368	55.387	0.020	0.344	0.954	0.979
ZFAND2B	216.343	0.145	0.246	0.555	0.677	216.343	-0.097	0.246	0.692	0.834	216.343	-0.242	0.285	0.394	0.622
COL16A1	2806.519	0.476	0.329	0.148	0.261	2806.519	0.616	0.329	0.061	0.350	2806.519	0.139	0.380	0.714	0.851
MDH1B	10.534	0.238	0.595	0.690	0.786	10.534	0.477	0.580	0.411	0.651	10.534	0.239	0.677	0.724	0.857
COL17A1	129.670	-0.107	0.238	0.654	0.758	129.670	0.323	0.234	0.168	0.457	129.670	0.429	0.273	0.116	0.305
SLC66A3	109.350	0.855	0.377	0.023	0.065	109.350	0.510	0.378	0.177	0.466	109.350	-0.345	0.435	0.428	0.651
TMEM182	131.340	-0.777	0.263	0.003	0.014	131.340	-0.012	0.255	0.963	0.980	131.340	0.765	0.302	0.011	0.070
AHSA2P	474.861	0.806	0.197	0.000	0.000	474.861	0.177	0.198	0.370	0.619	474.861	-0.629	0.227	0.006	0.045
FBXO36	37.446	0.201	0.339	0.554	0.676	37.446	0.033	0.339	0.922	0.960	37.446	-0.167	0.393	0.670	0.824
MTERF4	238.349	-0.240	0.130	0.064	0.139	238.349	-0.104	0.127	0.411	0.650	238.349	0.136	0.150	0.365	0.597
CCDC58	567.231	0.208	0.302	0.490	0.623	567.231	-0.524	0.303	0.083	0.375	567.231	-0.732	0.349	0.036	0.148
KCNH8	7.166	0.407	0.780	0.602	0.715	7.166	0.502	0.770	0.514	0.720	7.166	0.095	0.891	0.915	0.960
DNAJC19	717.370	0.480	0.225	0.033	0.084	717.370	-0.012	0.225	0.958	0.978	717.370	-0.492	0.260	0.058	0.201
COMT	3143.791	0.477	0.190	0.012	0.040	3143.791	0.229	0.190	0.229	0.507	3143.791	-0.248	0.220	0.259	0.493
COPA	5636.874	-0.153	0.127	0.230	0.361	5636.874	-0.024	0.127	0.850	0.923	5636.874	0.129	0.147	0.380	0.610
FAM131A	463.043	0.092	0.244	0.706	0.798	463.043	-0.246	0.245	0.316	0.578	463.043	-0.338	0.283	0.232	0.461
CHCHD4	444.352	-0.724	0.279	0.009	0.032	444.352	-0.439	0.278	0.114	0.406	444.352	0.285	0.322	0.376	0.607
COPB1	2341.946	-0.194	0.139	0.163	0.281	2341.946	-0.190	0.138	0.170	0.460	2341.946	0.004	0.160	0.982	0.990
CRYBG3	432.259	-0.110	0.214	0.606	0.718	432.259	-0.094	0.213	0.660	0.815	432.259	0.017	0.247	0.946	0.975
DCBLD2	704.086	-0.084	0.203	0.679	0.776	704.086	0.001	0.203	0.996	0.998	704.086	0.085	0.235	0.716	0.853
FAM43A	1180.819	1.305	0.350	0.000	0.002	1180.819	0.627	0.350	0.074	0.362	1180.819	-0.678	0.404	0.093	0.268
KLF6	3358.926	0.225	0.256	0.379	0.519	3358.926	-0.356	0.256	0.164	0.455	3358.926	-0.581	0.296	0.049	0.181
TPRA1	1897.296	0.030	0.138	0.825	0.885	1897.296	-0.064	0.138	0.644	0.806	1897.296	-0.094	0.159	0.554	0.747
TMEM42	103.504	0.589	0.323	0.068	0.146	103.504	0.358	0.323	0.267	0.539	103.504	-0.231	0.372	0.535	0.733
SLC31A1	2013.780	-0.960	0.258	0.000	0.002	2013.780	-0.434	0.257	0.092	0.387	2013.780	0.526	0.298	0.077	0.240

SLC31A2	129.212	0.761	0.220	0.001	0.004	129.212	-0.233	0.230	0.311	0.575	129.212	-0.994	0.260	0.000	0.003
NUDT16	910.459	0.105	0.146	0.471	0.605	910.459	0.033	0.146	0.823	0.908	910.459	-0.073	0.169	0.667	0.823
METTL6	362.383	-0.698	0.187	0.000	0.002	362.383	-0.102	0.184	0.580	0.764	362.383	0.597	0.216	0.006	0.045
ADK	1104.537	-0.185	0.102	0.070	0.149	1104.537	-0.248	0.102	0.015	0.306	1104.537	-0.063	0.118	0.596	0.775
TAMM41	271.234	-0.179	0.264	0.497	0.629	271.234	-0.363	0.264	0.169	0.458	271.234	-0.184	0.306	0.547	0.742
IL17RE	35.694	0.743	0.686	0.279	0.416	35.694	0.619	0.686	0.367	0.618	35.694	-0.124	0.790	0.875	0.940
GLYCTK	77.479	-0.787	0.290	0.007	0.025	77.479	-0.198	0.280	0.480	0.699	77.479	0.589	0.334	0.078	0.241
PPM1M	122.240	0.180	0.543	0.741	0.825	122.240	0.941	0.540	0.082	0.373	122.240	0.761	0.625	0.224	0.451
LSMEM2	338.934	0.257	0.367	0.483	0.616	338.934	-0.427	0.368	0.246	0.521	338.934	-0.685	0.425	0.107	0.292
RPL32P3	94.055	0.371	0.276	0.179	0.302	94.055	0.239	0.275	0.385	0.631	94.055	-0.132	0.318	0.679	0.830
OCIAD2	1663.167	0.785	0.195	0.000	0.001	1663.167	0.202	0.195	0.300	0.567	1663.167	-0.583	0.225	0.010	0.064
SCLT1	524.229	-0.323	0.125	0.010	0.034	524.229	-0.269	0.125	0.031	0.322	524.229	0.054	0.145	0.709	0.849
C4orf33	142.340	0.061	0.162	0.707	0.799	142.340	0.020	0.161	0.903	0.951	142.340	-0.041	0.188	0.826	0.914
MAP3K8	269.292	0.954	0.175	0.000	0.000	269.292	0.309	0.178	0.082	0.374	269.292	-0.646	0.203	0.001	0.017
LIN54	314.274	-0.660	0.260	0.011	0.037	314.274	-0.375	0.258	0.146	0.438	314.274	0.284	0.300	0.344	0.578
SPATA18	20.397	0.474	0.601	0.430	0.567	20.397	0.662	0.595	0.266	0.538	20.397	0.188	0.688	0.785	0.892
COX4I1	8780.272	0.479	0.176	0.007	0.025	8780.272	0.151	0.176	0.390	0.635	8780.272	-0.327	0.203	0.108	0.293
FAM241A	681.896	-1.479	0.308	0.000	0.000	681.896	-0.656	0.307	0.032	0.322	681.896	0.823	0.356	0.021	0.104
GNPDA2	586.698	-0.345	0.152	0.024	0.065	586.698	-0.069	0.151	0.646	0.807	586.698	0.276	0.176	0.117	0.306
CPEB2	250.629	-0.204	0.230	0.376	0.516	250.629	-0.183	0.229	0.424	0.660	250.629	0.021	0.266	0.938	0.972
COX5B	2049.131	0.947	0.238	0.000	0.001	2049.131	0.179	0.239	0.454	0.681	2049.131	-0.769	0.275	0.005	0.042
AASDH	212.277	-0.550	0.260	0.035	0.088	212.277	0.029	0.256	0.910	0.955	212.277	0.578	0.300	0.054	0.191
C4orf36	21.738	-0.399	0.467	0.393	0.531	21.738	0.166	0.449	0.712	0.846	21.738	0.565	0.534	0.290	0.526
ADM	2996.004	2.089	0.861	0.015	0.047	2996.004	1.271	0.861	0.140	0.431	2996.004	-0.818	0.993	0.410	0.636
PACRGL	221.339	-0.687	0.236	0.004	0.015	221.339	-0.282	0.232	0.224	0.503	221.339	0.404	0.272	0.138	0.341
TRAM1L1	48.723	-0.175	0.408	0.668	0.769	48.723	0.271	0.402	0.500	0.713	48.723	0.446	0.469	0.342	0.576
SLC9B2	455.526	-0.479	0.189	0.011	0.037	455.526	-0.337	0.188	0.072	0.361	455.526	0.142	0.218	0.515	0.719
SETD9	118.796	0.040	0.225	0.857	0.907	118.796	-0.152	0.226	0.500	0.713	118.796	-0.193	0.262	0.462	0.679
IL31RA	71.997	-1.606	0.453	0.000	0.003	71.997	0.235	0.433	0.586	0.768	71.997	1.841	0.517	0.000	0.006
EMB	1155.423	-0.558	0.272	0.041	0.099	1155.423	-0.232	0.272	0.393	0.637	1155.423	0.325	0.315	0.301	0.536
PPARGC1B	188.913	-0.819	0.275	0.003	0.013	188.913	-0.406	0.271	0.135	0.426	188.913	0.413	0.318	0.194	0.416
EGFLAM	243.903	-0.237	0.206	0.249	0.383	243.903	0.110	0.203	0.588	0.769	243.903	0.347	0.237	0.143	0.348
PRRC1	1020.060	-0.286	0.194	0.142	0.253	1020.060	-0.064	0.194	0.743	0.863	1020.060	0.222	0.224	0.322	0.557
NADK2	857.301	-0.602	0.134	0.000	0.000	857.301	-0.060	0.132	0.653	0.811	857.301	0.542	0.155	0.000	0.007
COX6A1	2669.197	0.989	0.249	0.000	0.001	2669.197	0.209	0.249	0.400	0.643	2669.197	-0.780	0.287	0.007	0.050
JMY	906.440	-1.062	0.258	0.000	0.000	906.440	-0.113	0.257	0.660	0.814	906.440	0.950	0.298	0.001	0.017
CCDC127	700.984	0.357	0.180	0.046	0.109	700.984	0.158	0.180	0.380	0.626	700.984	-0.200	0.207	0.335	0.570
COX6B1	2670.957	1.284	0.287	0.000	0.000	2670.957	0.241	0.287	0.402	0.645	2670.957	-1.044	0.332	0.002	0.019
ATPSCKMT	222.241	-0.660	0.258	0.010	0.035	222.241	-0.732	0.257	0.004	0.297	222.241	-0.072	0.299	0.809	0.904
CMBL	3142.155	-0.062	0.250	0.803	0.870	3142.155	0.333	0.250	0.183	0.470	3142.155	0.395	0.289	0.171	0.388
DNAJC21	1692.456	-0.215	0.157	0.170	0.290	1692.456	-0.302	0.156	0.054	0.342	1692.456	-0.087	0.181	0.631	0.799
AFAP1L1	1120.433	-0.003	0.186	0.988	0.993	1120.433	0.132	0.185	0.477	0.697	1120.433	0.134	0.214	0.530	0.730
GRPEL2	435.838	-1.313	0.362	0.000	0.002	435.838	-0.708	0.360	0.049	0.337	435.838	0.605	0.419	0.148	0.356
LSM11	292.336	-0.109	0.203	0.593	0.708	292.336	0.088	0.202	0.663	0.817	292.336	0.197	0.235	0.402	0.629
POC5	221.132	-0.768	0.284	0.007	0.025	221.132	-0.510	0.281	0.070	0.359	221.132	0.258	0.328	0.431	0.653
STARD4	328.554	1.397	0.240	0.000	0.000	328.554	-0.121	0.244	0.619	0.790	328.554	-1.518	0.279	0.000	0.000
WDR36	746.189	-1.207	0.336	0.000	0.002	746.189	-0.821	0.336	0.014	0.306	746.189	0.386	0.389	0.321	0.556
NUDCD2	1442.095	0.113	0.175	0.519	0.647	1442.095	0.237	0.174	0.174	0.463	1442.095	0.124	0.202	0.537	0.735
COX6C	1146.927	1.093	0.258	0.000	0.000	1146.927	0.082	0.259	0.750	0.867	1146.927	-1.011	0.299	0.001	0.010
UBLCP1	520.758	-0.415	0.293	0.156	0.273	520.758	-0.616	0.293	0.035	0.324	520.758	-0.201	0.339	0.553	0.747
SHROOM1	219.539	0.835	0.183	0.000	0.000	219.539	0.222	0.185	0.231	0.509	219.539	-0.614	0.211	0.004	0.033
C5orf24	1163.515	-0.607	0.210	0.004	0.016	1163.515	-0.097	0.209	0.641	0.805	1163.515	0.510	0.242	0.035	0.146
ADAT2	290.723	-0.716	0.259	0.006	0.022	290.723	-0.381	0.257	0.138	0.429	290.723	0.335	0.299	0.263	0.497
COX7A2	1881.336	0.493	0.176	0.005	0.020	1881.336	0.127	0.176	0.472	0.693	1881.336	-0.367	0.203	0.071	0.228
IRAK1BP1	246.379	-0.688	0.280	0.014	0.044	246.379	0.086	0.277	0.757	0.871	246.379	0.773	0.323	0.016	0.090
COX7B	992.054	0.908	0.229	0.000	0.001	992.054	0.024	0.230	0.917	0.959	992.054	-0.885	0.265	0.001	0.012
STXBP5	700.205	-0.467	0.221	0.034	0.087	700.205	-0.177	0.220	0.420	0.657	700.205	0.290	0.255	0.256	0.490
COX7C	4705.854	0.708	0.239	0.003	0.013	4705.854	0.317	0.239	0.184	0.471	4705.854	-0.391	0.276	0.156	0.368
COX8A	1253.804	0.421	0.226	0.063	0.138	1253.804	0.044	0.227	0.847	0.921	1253.804	-0.377	0.262	0.149	0.357
NCOA7	1094.533	-0.785	0.232	0.001	0.005	1094.533	-0.178	0.231	0.442	0.673	1094.533	0.607	0.268	0.024	0.113
HINT3	593.526	0.061	0.237	0.797	0.866	593.526	0.190	0.237	0.423	0.659	593.526	0.129	0.274	0.638	0.803
B3GAT2	665.297	0.362	0.265	0.171	0.291	665.297	0.392	0.264	0.138	0.429	665.297	0.029	0.305	0.923	0.964
SDHAF4	213.906	0.526	0.337	0.118	0.222	213.906	0.704	0.336	0.036	0.324	213.906	0.177	0.388	0.647	0.810
COX10	205.766	-0.650	0.193	0.001	0.005	205.766	-0.354	0.190	0.062	0.350	205.766	0.296	0.223	0.185	0.404
CD109	5370.746	-0.129	0.348	0.711	0.802	5370.746	0.268	0.348	0.441	0.673	5370.746	0.397	0.402	0.323	0.558
RAET1E	129.308	0.688	0.255	0.007	0.026	129.308	0.495	0.255	0.052	0.341	129.308	-0.193	0.293	0.510	0.715
PM20D2	1450.998	-0.710	0.251	0.005	0.019	1450.998	-0.173	0.251	0.489	0.705	1450.998	0.536	0.290	0.065	0.214

COX11	942.519	-0.216	0.107	0.043	0.102	942.519	-0.067	0.106	0.529	0.730	942.519	0.150	0.123	0.225	0.453
COX15	862.935	-0.539	0.185	0.004	0.015	862.935	0.143	0.184	0.436	0.669	862.935	0.682	0.214	0.001	0.017
CP	512.724	1.416	0.309	0.000	0.000	512.724	0.491	0.311	0.114	0.406	512.724	-0.925	0.357	0.010	0.064
TMEM139	10.063	0.848	0.518	0.101	0.197	10.063	-0.078	0.552	0.888	0.944	10.063	-0.926	0.616	0.133	0.331
ADORA2B	90.391	0.241	0.259	0.353	0.493	90.391	0.377	0.255	0.140	0.431	90.391	0.136	0.297	0.647	0.809
ZNF786	90.256	0.124	0.269	0.645	0.751	90.256	-0.600	0.276	0.030	0.322	90.256	-0.724	0.318	0.023	0.110
CPD	3556.452	-0.085	0.256	0.740	0.825	3556.452	0.138	0.256	0.591	0.773	3556.452	0.223	0.296	0.452	0.671
CPE	28.628	0.372	0.308	0.226	0.358	28.628	0.045	0.313	0.886	0.943	28.628	-0.327	0.359	0.363	0.595
MTPN	2958.087	-0.555	0.207	0.007	0.027	2958.087	-0.174	0.207	0.400	0.643	2958.087	0.381	0.239	0.111	0.298
CLDN4	71.094	0.845	0.244	0.001	0.003	71.094	0.496	0.246	0.044	0.331	71.094	-0.349	0.279	0.211	0.436
CLDN7	83.152	0.428	0.228	0.061	0.134	83.152	0.241	0.228	0.291	0.559	83.152	-0.187	0.263	0.477	0.690
MPLKIP	750.191	0.320	0.193	0.097	0.192	750.191	0.016	0.193	0.933	0.966	750.191	-0.304	0.223	0.173	0.390
CPM	48.788	0.060	0.312	0.848	0.900	48.788	0.120	0.307	0.695	0.836	48.788	0.060	0.360	0.867	0.936
SSC4D	27.032	0.445	0.572	0.437	0.573	27.032	0.686	0.567	0.226	0.504	27.032	0.242	0.655	0.712	0.850
C7orf31	49.129	0.127	0.359	0.724	0.813	49.129	0.117	0.356	0.743	0.863	49.129	-0.009	0.414	0.982	0.990
CPOX	1069.517	-0.393	0.245	0.109	0.209	1069.517	0.215	0.245	0.380	0.627	1069.517	0.607	0.283	0.032	0.137
ZNF572	8.732	-0.701	0.575	0.223	0.354	8.732	-0.137	0.536	0.799	0.895	8.732	0.564	0.659	0.392	0.620
CPS1	837.300	-0.544	0.355	0.126	0.232	837.300	0.364	0.355	0.305	0.571	837.300	0.907	0.410	0.027	0.123
FAM92A	371.820	0.164	0.174	0.347	0.487	371.820	0.111	0.174	0.524	0.727	371.820	-0.053	0.201	0.791	0.896
CPT1A	410.575	0.783	0.202	0.000	0.001	410.575	0.043	0.204	0.832	0.914	410.575	-0.739	0.234	0.002	0.018
VPS37A	714.615	-0.043	0.158	0.787	0.859	714.615	-0.264	0.158	0.095	0.389	714.615	-0.221	0.183	0.227	0.455
CPT1B	33.196	1.529	0.531	0.004	0.017	33.196	0.864	0.537	0.107	0.400	33.196	-0.665	0.608	0.274	0.509
CPT2	436.638	-0.326	0.140	0.020	0.057	436.638	0.150	0.138	0.276	0.546	436.638	0.476	0.161	0.003	0.030
NDUFAF6	138.601	-0.336	0.236	0.154	0.270	138.601	-0.333	0.234	0.154	0.447	138.601	0.003	0.274	0.991	0.995
TMEM68	376.133	-0.104	0.183	0.571	0.690	376.133	0.011	0.182	0.950	0.975	376.133	0.115	0.211	0.586	0.767
SGCZ	19.217	-0.392	1.004	0.696	0.790	19.217	0.830	0.989	0.401	0.644	19.217	1.222	1.152	0.289	0.524
UBXN2B	711.027	-0.738	0.242	0.002	0.011	711.027	0.232	0.241	0.334	0.593	711.027	0.970	0.279	0.001	0.008
PXDNL	11.015	0.847	0.986	0.390	0.529	11.015	1.316	0.977	0.178	0.467	11.015	0.469	1.125	0.677	0.829
GPAT4	1596.972	0.216	0.141	0.125	0.230	1596.972	-0.214	0.141	0.129	0.421	1596.972	-0.431	0.163	0.008	0.057
LETM2	145.897	-0.987	0.452	0.029	0.077	145.897	-1.222	0.453	0.007	0.297	145.897	-0.236	0.525	0.654	0.814
CR2	25.371	0.941	0.460	0.041	0.099	25.371	0.168	0.470	0.720	0.850	25.371	-0.773	0.534	0.148	0.356
HGSNAT	749.325	0.627	0.229	0.006	0.023	749.325	0.212	0.229	0.355	0.608	749.325	-0.415	0.264	0.117	0.306
NACC2	1100.060	-0.591	0.216	0.006	0.023	1100.060	-0.286	0.215	0.184	0.470	1100.060	0.306	0.249	0.220	0.446
C9orf116	86.007	0.612	0.225	0.006	0.024	86.007	0.231	0.228	0.310	0.575	86.007	-0.381	0.260	0.143	0.348
CARNMT1	367.770	-0.213	0.162	0.189	0.313	367.770	-0.268	0.162	0.097	0.392	367.770	-0.055	0.188	0.771	0.883
CRABP2	338.634	0.620	0.771	0.421	0.558	338.634	1.039	0.770	0.177	0.466	338.634	0.419	0.889	0.638	0.803
C9orf85	299.302	-0.176	0.198	0.375	0.516	299.302	0.178	0.196	0.363	0.615	299.302	0.354	0.228	0.121	0.313
DIPK1B	229.081	0.613	0.342	0.073	0.155	229.081	0.639	0.342	0.061	0.350	229.081	0.026	0.394	0.948	0.976
CRAT	194.363	0.501	0.357	0.160	0.278	194.363	0.605	0.356	0.089	0.383	194.363	0.104	0.411	0.801	0.900
PTRH1	173.173	0.131	0.452	0.773	0.849	173.173	-1.399	0.459	0.002	0.297	173.173	-1.530	0.528	0.004	0.034
PIP5KL1	12.838	1.454	0.542	0.007	0.027	12.838	1.157	0.546	0.034	0.322	12.838	-0.297	0.598	0.620	0.792
CREB1	731.997	-0.447	0.195	0.022	0.061	731.997	-0.212	0.194	0.273	0.544	731.997	0.234	0.225	0.297	0.533
ATF2	820.673	-0.342	0.215	0.111	0.212	820.673	0.179	0.214	0.402	0.644	820.673	0.522	0.248	0.035	0.147
PTPDC1	446.684	-0.625	0.169	0.000	0.002	446.684	0.002	0.166	0.992	0.996	446.684	0.626	0.194	0.001	0.016
CREBBP	1117.747	-0.827	0.155	0.000	0.000	1117.747	-0.044	0.153	0.773	0.879	1117.747	0.782	0.179	0.000	0.000
RPP25L	553.748	-0.094	0.159	0.554	0.675	553.748	0.135	0.157	0.391	0.636	553.748	0.229	0.183	0.211	0.436
ATF6B	4389.110	0.385	0.158	0.015	0.045	4389.110	0.420	0.158	0.008	0.297	4389.110	0.035	0.182	0.846	0.926
CREBL2	549.302	-0.078	0.252	0.756	0.838	549.302	0.104	0.252	0.680	0.827	549.302	0.182	0.291	0.532	0.731
CREM	329.712	-0.364	0.269	0.177	0.299	329.712	-0.564	0.270	0.036	0.324	329.712	-0.201	0.312	0.520	0.723
PWWP3B	129.407	-0.736	0.551	0.181	0.305	129.407	0.192	0.547	0.726	0.854	129.407	0.928	0.635	0.144	0.350
FAM199X	687.543	-0.633	0.184	0.001	0.004	687.543	-0.366	0.183	0.046	0.334	687.543	0.267	0.213	0.209	0.435
AMER1	310.952	-0.887	0.201	0.000	0.000	310.952	-0.228	0.197	0.247	0.522	310.952	0.659	0.232	0.005	0.038
APOOL	444.809	-0.920	0.280	0.001	0.006	444.809	0.046	0.278	0.868	0.932	444.809	0.966	0.323	0.003	0.027
HDX	74.413	-0.602	0.252	0.017	0.050	74.413	-0.187	0.242	0.441	0.673	74.413	0.415	0.290	0.153	0.362
FUNDC1	141.947	0.696	0.236	0.003	0.014	141.947	0.325	0.237	0.170	0.459	141.947	-0.371	0.272	0.172	0.389
UPRT	210.732	-0.068	0.167	0.686	0.783	210.732	-0.287	0.168	0.087	0.380	210.732	-0.219	0.196	0.262	0.496
CRIP2	722.078	0.609	0.283	0.031	0.081	722.078	0.360	0.283	0.203	0.486	722.078	-0.249	0.326	0.444	0.664
PNCK	210.797	-0.352	0.282	0.211	0.341	210.797	0.269	0.277	0.330	0.590	210.797	0.622	0.324	0.055	0.194
CRK	895.017	-0.176	0.134	0.189	0.313	895.017	-0.202	0.134	0.131	0.423	895.017	-0.026	0.155	0.868	0.936
DOCK11	97.387	0.028	0.310	0.927	0.954	97.387	-0.695	0.315	0.027	0.319	97.387	-0.724	0.363	0.046	0.174
SPIN4	131.684	-0.853	0.239	0.000	0.003	131.684	-0.180	0.231	0.436	0.669	131.684	0.673	0.275	0.014	0.083
CRKL	2139.986	-0.334	0.164	0.042	0.101	2139.986	-0.101	0.164	0.537	0.735	2139.986	0.233	0.190	0.219	0.446
AAMP	1306.572	-0.069	0.185	0.709	0.801	1306.572	-0.212	0.185	0.253	0.526	1306.572	-0.143	0.214	0.505	0.712
CRMP1	108.198	0.672	0.320	0.036	0.090	108.198	0.277	0.322	0.390	0.635	108.198	-0.395	0.370	0.285	0.520
ASB6	741.938	0.093	0.292	0.749	0.832	741.938	-0.563	0.293	0.054	0.342	741.938	-0.657	0.338	0.052	0.187
ASB7	324.293	-0.576	0.164	0.000	0.003	324.293	-0.135	0.161	0.400	0.644	324.293	0.440	0.189	0.020	0.102
ASB8	332.986	-0.066	0.146	0.649	0.755	332.986	0.091	0.144	0.528	0.729	332.986	0.157	0.168	0.350	0.583

ASB9	123.995	-0.529	0.602	0.380	0.520	123.995	0.468	0.599	0.434	0.667	123.995	0.997	0.694	0.151	0.360
MYL6B	646.334	0.628	0.299	0.036	0.090	646.334	0.477	0.299	0.111	0.404	646.334	-0.151	0.345	0.661	0.819
ZNF358	1292.694	0.917	0.417	0.028	0.074	1292.694	0.631	0.417	0.130	0.422	1292.694	-0.286	0.481	0.553	0.746
MYO3B	12.110	1.558	0.985	0.114	0.215	12.110	0.863	0.998	0.387	0.633	12.110	-0.696	1.127	0.537	0.735
RNF32	53.183	0.444	0.253	0.079	0.163	53.183	0.254	0.253	0.314	0.577	53.183	-0.190	0.291	0.514	0.718
APOBEC3D	17.046	-5.257	1.281	0.000	0.000	17.046	-0.163	0.800	0.839	0.916	17.046	5.094	1.363	0.000	0.004
S100A16	4120.176	-0.021	0.162	0.895	0.932	4120.176	-0.036	0.162	0.826	0.909	4120.176	-0.014	0.187	0.939	0.973
CHODL	9.660	0.224	0.602	0.711	0.802	9.660	0.364	0.591	0.538	0.736	9.660	0.140	0.689	0.839	0.922
SELENOM	1500.440	1.479	0.211	0.000	0.000	1500.440	0.208	0.212	0.327	0.588	1500.440	-1.272	0.244	0.000	0.000
NEK7	703.206	-0.424	0.186	0.023	0.064	703.206	-0.191	0.185	0.304	0.570	703.206	0.233	0.215	0.279	0.514
ZFP28	43.499	0.170	0.263	0.518	0.646	43.499	0.198	0.258	0.443	0.674	43.499	0.028	0.302	0.926	0.965
C20orf96	102.308	0.491	0.239	0.040	0.098	102.308	0.304	0.239	0.204	0.487	102.308	-0.187	0.276	0.498	0.705
ZBTB46	63.884	1.159	0.296	0.000	0.001	63.884	0.397	0.304	0.192	0.477	63.884	-0.762	0.342	0.026	0.119
WFDC3	45.860	2.622	0.602	0.000	0.000	45.860	1.484	0.631	0.019	0.306	45.860	-1.138	0.652	0.081	0.247
NOL4L	74.146	1.850	0.499	0.000	0.002	74.146	0.569	0.505	0.261	0.533	74.146	-1.281	0.576	0.026	0.121
MROH8	354.291	-0.825	0.337	0.014	0.045	354.291	-0.084	0.335	0.802	0.896	354.291	0.741	0.389	0.057	0.199
CRY1	110.165	-0.856	0.579	0.140	0.250	110.165	-1.799	0.586	0.002	0.297	110.165	-0.944	0.678	0.164	0.378
SAMD10	41.565	0.972	0.311	0.002	0.009	41.565	0.022	0.327	0.947	0.973	41.565	-0.950	0.366	0.009	0.063
ABHD16B	59.810	0.234	0.325	0.472	0.606	59.810	-0.293	0.330	0.375	0.623	59.810	-0.527	0.380	0.166	0.381
BRI3BP	698.664	-0.167	0.211	0.428	0.564	698.664	-0.355	0.211	0.093	0.387	698.664	-0.188	0.244	0.442	0.662
SOGA1	956.031	-0.231	0.139	0.098	0.193	956.031	0.006	0.139	0.963	0.980	956.031	0.237	0.161	0.140	0.344
TLDC2	37.124	0.349	0.461	0.449	0.584	37.124	-0.390	0.469	0.405	0.646	37.124	-0.739	0.539	0.170	0.386
MACROD2	13.756	0.427	0.635	0.502	0.631	13.756	0.734	0.623	0.239	0.516	13.756	0.307	0.722	0.671	0.825
DYNLL2	1608.578	0.203	0.105	0.053	0.121	1608.578	0.006	0.105	0.952	0.976	1608.578	-0.196	0.121	0.105	0.289
TMEM37	45.224	1.382	0.578	0.017	0.050	45.224	0.525	0.582	0.367	0.618	45.224	-0.857	0.667	0.199	0.422
UBE2F	236.023	-0.296	0.251	0.239	0.372	236.023	-0.256	0.250	0.305	0.571	236.023	0.040	0.291	0.891	0.948
SMCR8	289.152	-0.264	0.221	0.233	0.365	289.152	0.046	0.219	0.834	0.915	289.152	0.310	0.255	0.225	0.453
CRY2	138.105	0.575	0.257	0.025	0.069	138.105	0.531	0.256	0.038	0.327	138.105	-0.044	0.295	0.881	0.943
SRXN1	1465.849	-0.462	0.230	0.045	0.106	1465.849	-0.286	0.230	0.215	0.494	1465.849	0.176	0.266	0.507	0.713
ROMO1	585.186	0.934	0.235	0.000	0.001	585.186	-0.008	0.236	0.972	0.985	585.186	-0.942	0.272	0.001	0.008
NEURL2	13.518	0.374	0.562	0.506	0.635	13.518	0.356	0.554	0.521	0.725	13.518	-0.018	0.642	0.977	0.988
ZSWIM3	21.366	0.309	0.465	0.507	0.636	21.366	0.232	0.462	0.615	0.788	21.366	-0.076	0.535	0.887	0.946
NANP	169.631	-0.464	0.305	0.128	0.235	169.631	-0.669	0.305	0.028	0.321	169.631	-0.205	0.354	0.562	0.753
ISM1	78.186	0.275	0.277	0.321	0.460	78.186	-0.325	0.282	0.249	0.523	78.186	-0.600	0.324	0.065	0.214
SIRPA	1872.917	0.775	0.149	0.000	0.000	1872.917	0.179	0.150	0.232	0.509	1872.917	-0.596	0.173	0.001	0.009
SREK1	1186.848	-0.079	0.212	0.711	0.802	1186.848	-0.220	0.212	0.300	0.566	1186.848	-0.141	0.245	0.564	0.754
CNBD2	10.966	0.296	0.549	0.590	0.706	10.966	0.139	0.548	0.800	0.896	10.966	-0.157	0.635	0.805	0.902
STK35	503.949	0.132	0.140	0.346	0.487	503.949	0.297	0.139	0.033	0.322	503.949	0.165	0.162	0.307	0.543
ADPRH	13.342	1.428	0.450	0.001	0.008	13.342	-0.867	0.550	0.115	0.407	13.342	-2.295	0.590	0.000	0.002
CRYAB	66.287	4.196	0.427	0.000	0.000	66.287	-0.911	0.515	0.077	0.368	66.287	-5.107	0.549	0.000	0.000
CRYBB2P1	206.583	-0.635	0.332	0.056	0.125	206.583	-0.708	0.331	0.033	0.322	206.583	-0.073	0.386	0.850	0.928
PARP1	5681.580	-0.592	0.267	0.026	0.071	5681.580	-0.533	0.267	0.045	0.334	5681.580	0.058	0.308	0.849	0.927
MIB2	791.844	0.951	0.336	0.005	0.019	791.844	0.708	0.336	0.035	0.324	791.844	-0.243	0.388	0.531	0.731
DUSP19	31.213	0.364	0.462	0.431	0.567	31.213	0.410	0.459	0.371	0.620	31.213	0.047	0.530	0.930	0.968
RAB40A	11.897	-0.577	0.734	0.432	0.568	11.897	-0.131	0.715	0.855	0.926	11.897	0.446	0.846	0.598	0.776
ASB14	68.545	0.310	0.387	0.424	0.560	68.545	0.457	0.385	0.235	0.513	68.545	0.147	0.445	0.741	0.867
SAMD8	728.347	-0.248	0.241	0.303	0.442	728.347	0.197	0.240	0.411	0.651	728.347	0.445	0.278	0.109	0.295
CRYZ	822.115	-0.139	0.120	0.249	0.383	822.115	0.040	0.119	0.739	0.861	822.115	0.178	0.139	0.199	0.422
CFL1P1	21.050	-1.052	0.384	0.006	0.023	21.050	-0.398	0.351	0.257	0.529	21.050	0.655	0.442	0.138	0.341
TRUB1	490.225	-0.890	0.184	0.000	0.000	490.225	-0.506	0.182	0.005	0.297	490.225	0.383	0.213	0.072	0.229
PARP4	1373.628	-0.257	0.148	0.082	0.168	1373.628	0.064	0.147	0.662	0.816	1373.628	0.321	0.170	0.059	0.203
MPP7	30.303	-1.089	0.345	0.002	0.008	30.303	0.075	0.306	0.806	0.898	30.303	1.164	0.388	0.003	0.027
CS	3971.773	-0.522	0.140	0.000	0.002	3971.773	-0.109	0.139	0.433	0.667	3971.773	0.413	0.161	0.010	0.068
VTI1A	293.590	-0.362	0.191	0.057	0.128	293.590	-0.263	0.189	0.164	0.454	293.590	0.099	0.221	0.655	0.815
MAPK14	1316.550	-0.633	0.249	0.011	0.037	1316.550	0.087	0.249	0.727	0.854	1316.550	0.720	0.288	0.012	0.075
EIF5AL1	109.680	0.244	0.368	0.508	0.638	109.680	-0.563	0.373	0.131	0.423	109.680	-0.806	0.430	0.061	0.206
HECTD2	119.087	-0.612	0.291	0.035	0.089	119.087	-0.222	0.286	0.438	0.671	119.087	0.390	0.336	0.245	0.476
FGFBP3	9.488	0.266	0.616	0.666	0.767	9.488	0.703	0.593	0.236	0.513	9.488	0.437	0.694	0.529	0.729
CACUL1	1480.655	-0.299	0.165	0.070	0.149	1480.655	-0.052	0.164	0.751	0.867	1480.655	0.247	0.190	0.194	0.417
CSE1L	2884.153	-1.143	0.322	0.000	0.003	2884.153	-0.775	0.322	0.016	0.306	2884.153	0.368	0.372	0.323	0.557
LDLRAD3	2316.471	-0.588	0.102	0.000	0.000	2316.471	0.109	0.101	0.281	0.550	2316.471	0.697	0.117	0.000	0.000
CSF1	2814.078	1.361	0.095	0.000	0.000	2814.078	0.108	0.096	0.260	0.532	2814.078	-1.253	0.110	0.000	0.000
XRRA1	785.345	-0.267	0.137	0.050	0.116	785.345	0.133	0.135	0.327	0.587	785.345	0.400	0.158	0.011	0.070
FAM76B	204.433	-0.247	0.162	0.129	0.236	204.433	-0.197	0.160	0.218	0.498	204.433	0.049	0.188	0.793	0.896
SESN3	1225.495	-0.807	0.858	0.347	0.487	1225.495	0.420	0.858	0.625	0.794	1225.495	1.227	0.991	0.216	0.441
CSF2RA	132.301	-0.213	0.212	0.315	0.455	132.301	-0.071	0.208	0.733	0.857	132.301	0.142	0.245	0.563	0.754
KBTBD3	40.480	0.493	0.461	0.284	0.422	40.480	0.414	0.460	0.368	0.618	40.480	-0.079	0.530	0.881	0.943

CWF19L2	375.277	0.285	0.204	0.162	0.281	375.277	-0.231	0.205	0.260	0.533	375.277	-0.515	0.236	0.029	0.130
POGLUT3	87.574	0.279	0.253	0.270	0.406	87.574	0.210	0.252	0.405	0.646	87.574	-0.069	0.292	0.812	0.906
LAYN	444.398	-0.493	0.177	0.005	0.021	444.398	-0.325	0.176	0.064	0.353	444.398	0.168	0.204	0.411	0.637
CSF3	692.386	-0.543	0.488	0.267	0.403	692.386	-0.824	0.489	0.092	0.386	692.386	-0.282	0.565	0.618	0.790
SPINDOC	489.047	0.543	0.192	0.005	0.019	489.047	-0.138	0.193	0.477	0.697	489.047	-0.681	0.223	0.002	0.023
PLEKHA7	28.169	-0.062	0.394	0.875	0.919	28.169	-0.836	0.413	0.043	0.330	28.169	-0.774	0.476	0.104	0.287
SPTY2D1	527.294	-0.437	0.275	0.112	0.213	527.294	-0.291	0.275	0.289	0.557	527.294	0.146	0.318	0.646	0.809
DNHD1	115.485	1.813	0.320	0.000	0.000	115.485	0.508	0.326	0.119	0.412	115.485	-1.305	0.370	0.000	0.007
PRICKLE1	8.499	0.126	0.555	0.821	0.882	8.499	-0.679	0.597	0.256	0.529	8.499	-0.805	0.684	0.240	0.470
AMDHD1	20.334	0.107	0.384	0.780	0.854	20.334	0.479	0.367	0.191	0.477	20.334	0.372	0.434	0.391	0.619
SLC2A14	69.965	0.712	0.686	0.299	0.438	69.965	0.440	0.687	0.522	0.725	69.965	-0.272	0.792	0.731	0.861
BCDIN3D	131.496	-0.314	0.157	0.046	0.108	131.496	0.036	0.150	0.808	0.899	131.496	0.350	0.180	0.052	0.188
ALG10B	123.047	-0.381	0.221	0.084	0.171	123.047	-0.158	0.217	0.465	0.689	123.047	0.223	0.255	0.382	0.611
ZNF664	2403.952	-0.528	0.140	0.000	0.001	2403.952	-0.168	0.140	0.229	0.507	2403.952	0.360	0.162	0.026	0.121
ETFRF1	264.607	-0.296	0.182	0.104	0.201	264.607	-0.013	0.180	0.944	0.972	264.607	0.284	0.210	0.177	0.394
CPNE8	438.934	-0.078	0.177	0.657	0.760	438.934	-0.166	0.176	0.346	0.602	438.934	-0.088	0.204	0.668	0.824
TMEM120B	935.247	-0.127	0.218	0.561	0.682	935.247	-0.257	0.218	0.238	0.515	935.247	-0.131	0.252	0.604	0.781
WDR66	30.163	-0.105	0.559	0.851	0.902	30.163	0.364	0.548	0.507	0.716	30.163	0.469	0.641	0.464	0.680
E2F7	593.290	-1.972	0.473	0.000	0.000	593.290	-1.133	0.471	0.016	0.306	593.290	0.839	0.546	0.125	0.318
CSK	1146.880	0.082	0.209	0.693	0.788	1146.880	-0.087	0.209	0.677	0.825	1146.880	-0.170	0.241	0.482	0.695
KRT80	1689.174	-0.537	0.282	0.057	0.127	1689.174	-0.239	0.282	0.396	0.640	1689.174	0.298	0.326	0.361	0.593
C12orf66	162.066	-0.641	0.219	0.003	0.015	162.066	0.069	0.212	0.743	0.863	162.066	0.710	0.251	0.005	0.039
C12orf60	57.816	0.062	0.204	0.762	0.842	57.816	0.065	0.200	0.746	0.865	57.816	0.003	0.235	0.989	0.994
FBXL14	366.519	-0.369	0.222	0.096	0.190	366.519	0.007	0.220	0.973	0.985	366.519	0.376	0.256	0.141	0.346
RAD9B	11.164	-0.242	0.518	0.640	0.747	11.164	-0.095	0.500	0.849	0.922	11.164	0.147	0.600	0.806	0.903
PHETA1	130.870	0.506	0.216	0.019	0.055	130.870	0.079	0.218	0.717	0.849	130.870	-0.427	0.250	0.088	0.259
LINC00355	15.883	-0.379	0.473	0.423	0.560	15.883	0.213	0.447	0.634	0.800	15.883	0.592	0.539	0.272	0.506
LACC1	96.109	0.139	0.196	0.476	0.609	96.109	0.022	0.195	0.909	0.955	96.109	-0.117	0.227	0.606	0.782
HNRNPA1L2	122.516	-1.135	0.247	0.000	0.000	122.516	-0.106	0.235	0.650	0.809	122.516	1.028	0.282	0.000	0.005
ST13P4	41.933	-0.517	0.317	0.103	0.200	41.933	0.102	0.298	0.733	0.857	41.933	0.619	0.362	0.087	0.258
B3GLCT	163.484	0.070	0.257	0.785	0.858	163.484	-0.520	0.260	0.045	0.334	163.484	-0.590	0.300	0.049	0.181
CSNK1A1	5344.520	-0.605	0.142	0.000	0.000	5344.520	-0.177	0.142	0.211	0.492	5344.520	0.428	0.164	0.009	0.061
LINC00239	11.562	1.799	0.611	0.003	0.014	11.562	0.261	0.681	0.701	0.839	11.562	-1.538	0.722	0.033	0.141
MIPOL1	152.975	-0.436	0.179	0.015	0.045	152.975	-0.175	0.175	0.317	0.579	152.975	0.261	0.206	0.206	0.430
CSNK1D	4010.702	0.188	0.103	0.069	0.148	4010.702	0.037	0.103	0.720	0.850	4010.702	-0.151	0.119	0.206	0.430
SLC38A6	129.399	0.706	0.216	0.001	0.006	129.399	-0.308	0.224	0.168	0.458	129.399	-1.015	0.255	0.000	0.002
CSNK1E	1349.470	0.058	0.224	0.796	0.866	1349.470	-0.151	0.224	0.501	0.713	1349.470	-0.209	0.259	0.420	0.644
FRMD6-AS1	13.782	-0.633	0.552	0.252	0.386	13.782	-1.068	0.564	0.058	0.348	13.782	-0.435	0.664	0.512	0.718
PTGR2	197.911	0.034	0.172	0.844	0.898	197.911	0.173	0.170	0.308	0.573	197.911	0.139	0.198	0.481	0.694
FAM161B	334.735	-0.184	0.267	0.490	0.623	334.735	-0.437	0.267	0.102	0.395	334.735	-0.252	0.310	0.415	0.640
CSNK1G2	1858.712	0.350	0.145	0.016	0.048	1858.712	-0.138	0.145	0.341	0.599	1858.712	-0.488	0.168	0.004	0.033
ISM2	20.735	0.794	1.002	0.428	0.565	20.735	0.973	0.998	0.330	0.590	20.735	0.179	1.149	0.876	0.941
CEP128	253.393	-1.328	0.300	0.000	0.000	253.393	-0.762	0.297	0.010	0.299	253.393	0.566	0.347	0.103	0.285
MDP1	59.353	-0.018	0.257	0.946	0.966	59.353	0.137	0.251	0.586	0.768	59.353	0.154	0.295	0.601	0.779
TTC7B	317.894	0.383	0.186	0.040	0.097	317.894	-0.247	0.188	0.190	0.475	317.894	-0.630	0.217	0.004	0.033
CSNK1G3	1037.301	-0.979	0.187	0.000	0.000	1037.301	-0.314	0.186	0.091	0.385	1037.301	0.665	0.216	0.002	0.022
LOC145694	375.463	-1.261	0.241	0.000	0.000	375.463	-0.088	0.235	0.707	0.842	375.463	1.172	0.277	0.000	0.001
CSNK2A1	3036.306	-0.844	0.118	0.000	0.000	3036.306	-0.128	0.117	0.274	0.545	3036.306	0.715	0.136	0.000	0.000
LYSMD4	253.342	0.604	0.185	0.001	0.006	253.342	0.199	0.186	0.285	0.554	253.342	-0.405	0.214	0.058	0.202
FAM81A	97.039	-0.599	0.369	0.105	0.202	97.039	-0.379	0.366	0.300	0.566	97.039	0.219	0.427	0.608	0.783
GCOM1	35.585	-2.477	0.659	0.000	0.001	35.585	-1.066	0.607	0.079	0.370	35.585	1.411	0.755	0.062	0.208
C15orf65	30.605	1.252	0.489	0.011	0.035	30.605	0.290	0.503	0.564	0.752	30.605	-0.962	0.568	0.090	0.263
C15orf61	203.522	-0.171	0.218	0.431	0.567	203.522	0.485	0.212	0.022	0.315	203.522	0.656	0.249	0.008	0.058
HAPLN3	71.481	1.727	0.237	0.000	0.000	71.481	0.062	0.258	0.812	0.901	71.481	-1.666	0.281	0.000	0.000
CSNK2A2	2204.940	0.113	0.130	0.384	0.523	2204.940	0.116	0.130	0.371	0.620	2204.940	0.003	0.150	0.984	0.991
NRG4	181.819	0.579	0.601	0.335	0.476	181.819	0.575	0.600	0.338	0.596	181.819	-0.004	0.693	0.996	0.997
CSNK2B	1610.718	0.383	0.227	0.092	0.184	1610.718	0.111	0.227	0.625	0.794	1610.718	-0.272	0.262	0.300	0.535
ZSCAN29	301.167	-0.485	0.178	0.006	0.024	301.167	-0.351	0.176	0.047	0.335	301.167	0.134	0.206	0.515	0.719
TTBK2	572.483	0.339	0.200	0.089	0.180	572.483	0.154	0.200	0.441	0.672	572.483	-0.185	0.230	0.422	0.646
CDAN1	212.999	0.026	0.161	0.871	0.917	212.999	-0.130	0.161	0.418	0.656	212.999	-0.157	0.187	0.403	0.630
ZFP90	370.899	-0.139	0.331	0.674	0.773	370.899	0.348	0.330	0.291	0.559	370.899	0.488	0.382	0.201	0.425
VCAN	24.957	0.568	0.542	0.294	0.432	24.957	-0.620	0.559	0.268	0.539	24.957	-1.188	0.639	0.063	0.211
CARMIL2	211.791	0.400	0.422	0.343	0.483	211.791	-0.554	0.424	0.191	0.477	211.791	-0.955	0.489	0.051	0.185
CMTM4	524.202	-0.037	0.097	0.700	0.793	524.202	-0.012	0.096	0.902	0.951	524.202	0.026	0.113	0.820	0.911
BEAN1	10.821	1.666	0.748	0.026	0.070	10.821	0.864	0.763	0.257	0.530	10.821	-0.802	0.852	0.347	0.581
FBXL16	34.562	0.590	0.687	0.390	0.530	34.562	1.349	0.678	0.047	0.335	34.562	0.759	0.783	0.333	0.568
CSPG4	725.590	0.650	0.241	0.007	0.026	725.590	0.449	0.241	0.062	0.350	725.590	-0.200	0.278	0.471	0.685

ZNF597	46.640	-0.601	0.368	0.103	0.200	46.640	-0.832	0.369	0.024	0.316	46.640	-0.232	0.434	0.593	0.773
BICDL2	17.096	1.258	0.638	0.049	0.113	17.096	0.512	0.650	0.431	0.665	17.096	-0.746	0.735	0.310	0.546
CSRP1	3352.318	0.018	0.252	0.944	0.965	3352.318	-0.521	0.252	0.039	0.327	3352.318	-0.539	0.291	0.064	0.213
ZNF785	37.667	0.813	0.365	0.026	0.070	37.667	0.216	0.373	0.562	0.750	37.667	-0.597	0.423	0.159	0.371
ZNF688	23.391	1.309	0.434	0.003	0.012	23.391	-0.649	0.490	0.185	0.472	23.391	-1.958	0.538	0.000	0.005
PRSS36	18.431	0.755	0.500	0.131	0.239	18.431	0.858	0.493	0.082	0.373	18.431	0.103	0.564	0.855	0.930
CSRP2	454.834	0.925	0.180	0.000	0.000	454.834	0.195	0.181	0.281	0.551	454.834	-0.730	0.208	0.000	0.007
TOM1L2	480.539	0.095	0.202	0.639	0.746	480.539	0.133	0.201	0.509	0.718	480.539	0.038	0.233	0.869	0.937
TEPSIN	207.355	0.481	0.216	0.026	0.071	207.355	-0.086	0.219	0.693	0.834	207.355	-0.568	0.251	0.024	0.114
B3GNTL1	424.197	0.772	0.286	0.007	0.026	424.197	-0.157	0.287	0.586	0.768	424.197	-0.928	0.331	0.005	0.041
RTN4RL1	59.484	-0.626	0.333	0.060	0.132	59.484	-0.187	0.320	0.559	0.748	59.484	0.439	0.383	0.252	0.485
SLC25A10	743.443	-0.540	0.409	0.187	0.311	743.443	-0.881	0.410	0.031	0.322	743.443	-0.341	0.473	0.471	0.685
SLFN13	122.648	-0.090	0.337	0.790	0.861	122.648	-0.044	0.336	0.895	0.948	122.648	0.046	0.390	0.907	0.957
KIF18B	466.165	-0.400	0.231	0.083	0.170	466.165	-0.022	0.229	0.923	0.961	466.165	0.378	0.266	0.155	0.367
RUNDC1	485.605	0.264	0.142	0.063	0.138	485.605	-0.322	0.144	0.025	0.316	485.605	-0.587	0.166	0.000	0.007
EME1	383.605	-0.015	0.326	0.964	0.978	383.605	-0.642	0.327	0.050	0.337	383.605	-0.628	0.378	0.097	0.275
ADRA1B	51.140	0.183	0.278	0.510	0.640	51.140	0.213	0.275	0.438	0.670	51.140	0.030	0.320	0.925	0.965
TMEM199	303.369	-0.214	0.262	0.414	0.552	303.369	-0.604	0.263	0.022	0.314	303.369	-0.389	0.305	0.202	0.425
DHRS13	1006.832	0.786	0.208	0.000	0.001	1006.832	0.455	0.208	0.029	0.321	1006.832	-0.331	0.240	0.167	0.382
KCTD11	587.946	1.377	0.479	0.004	0.017	587.946	0.695	0.479	0.147	0.439	587.946	-0.682	0.553	0.217	0.443
LINC02210	215.840	0.495	0.328	0.132	0.240	215.840	0.641	0.327	0.050	0.337	215.840	0.147	0.378	0.697	0.841
CST3	1537.009	1.231	0.269	0.000	0.000	1537.009	0.176	0.269	0.514	0.720	1537.009	-1.055	0.311	0.001	0.010
NOTUM	34.489	1.012	0.383	0.008	0.029	34.489	0.369	0.394	0.349	0.604	34.489	-0.643	0.442	0.146	0.353
TRIM16L	797.677	0.227	0.147	0.123	0.228	797.677	0.052	0.147	0.722	0.852	797.677	-0.174	0.170	0.304	0.540
WIPF2	1065.921	0.227	0.156	0.145	0.258	1065.921	0.067	0.156	0.666	0.818	1065.921	-0.160	0.180	0.375	0.607
TMEM99	254.916	-0.429	0.199	0.031	0.080	254.916	0.154	0.194	0.427	0.663	254.916	0.583	0.228	0.011	0.069
C18orf25	438.230	-0.275	0.137	0.045	0.107	438.230	-0.038	0.135	0.781	0.885	438.230	0.237	0.158	0.134	0.334
CCBE1	99.264	0.074	0.624	0.906	0.940	99.264	-1.851	0.633	0.003	0.297	99.264	-1.925	0.729	0.008	0.058
ANKRD29	96.578	0.455	0.206	0.027	0.073	96.578	-0.161	0.211	0.447	0.677	96.578	-0.615	0.242	0.011	0.070
APCDD1	17.347	0.256	0.403	0.526	0.652	17.347	0.363	0.392	0.355	0.609	17.347	0.107	0.458	0.815	0.908
CSTA	506.799	-0.436	0.249	0.080	0.166	506.799	0.562	0.247	0.023	0.315	506.799	0.999	0.287	0.001	0.008
LINC00526	92.237	-0.318	0.232	0.171	0.292	92.237	0.125	0.225	0.580	0.764	92.237	0.443	0.267	0.097	0.275
CSTB	4154.086	1.951	0.310	0.000	0.000	4154.086	0.643	0.310	0.038	0.327	4154.086	-1.309	0.357	0.000	0.005
VSIG10L	38.770	1.444	0.384	0.000	0.001	38.770	0.469	0.396	0.236	0.514	38.770	-0.975	0.444	0.028	0.126
SPACA6	80.679	0.894	0.395	0.024	0.065	80.679	0.517	0.397	0.193	0.478	80.679	-0.377	0.454	0.407	0.633
ZNF480	269.260	-0.038	0.181	0.833	0.890	269.260	-0.513	0.183	0.005	0.297	269.260	-0.475	0.212	0.025	0.117
ZNF417	56.730	-0.434	0.299	0.147	0.260	56.730	0.180	0.287	0.530	0.731	56.730	0.614	0.342	0.072	0.230
ZNF548	106.801	0.206	0.198	0.299	0.437	106.801	0.389	0.195	0.045	0.334	106.801	0.183	0.227	0.419	0.643
PPM1N	55.402	1.014	0.445	0.023	0.063	55.402	0.597	0.447	0.182	0.469	55.402	-0.417	0.511	0.415	0.640
CSTF1	422.205	-0.137	0.146	0.350	0.490	422.205	-0.054	0.145	0.710	0.845	422.205	0.083	0.169	0.624	0.794
KLC3	173.411	0.259	0.221	0.241	0.374	173.411	-0.350	0.224	0.119	0.411	173.411	-0.609	0.258	0.018	0.096
ILF3-DT	431.988	0.017	0.229	0.942	0.964	431.988	-0.152	0.229	0.507	0.717	431.988	-0.169	0.265	0.525	0.726
TMC4	90.177	0.603	0.217	0.005	0.021	90.177	0.395	0.217	0.069	0.357	90.177	-0.208	0.249	0.404	0.631
CSTF2	377.643	-0.760	0.333	0.022	0.063	377.643	-0.768	0.332	0.021	0.312	377.643	-0.008	0.385	0.983	0.991
TPM3P9	105.186	-0.137	0.185	0.460	0.594	105.186	-0.599	0.189	0.002	0.297	105.186	-0.462	0.220	0.035	0.147
ZNF524	328.613	0.872	0.335	0.009	0.032	328.613	0.702	0.335	0.036	0.324	328.613	-0.170	0.386	0.660	0.818
ZNF784	86.356	0.015	0.228	0.948	0.968	86.356	0.355	0.222	0.109	0.401	86.356	0.341	0.260	0.190	0.412
SPC24	437.011	0.621	0.139	0.000	0.000	437.011	0.083	0.140	0.556	0.747	437.011	-0.538	0.161	0.001	0.012
CSTF3	796.732	0.324	0.145	0.026	0.070	796.732	-0.027	0.146	0.855	0.926	796.732	-0.350	0.168	0.037	0.152
SIX5	928.186	0.410	0.216	0.058	0.130	928.186	0.400	0.216	0.064	0.353	928.186	-0.010	0.250	0.969	0.985
ZNF420	122.400	-0.477	0.291	0.101	0.197	122.400	0.414	0.283	0.143	0.434	122.400	0.891	0.333	0.007	0.053
ZNF565	42.457	0.003	0.307	0.991	0.995	42.457	0.188	0.300	0.531	0.731	42.457	0.185	0.352	0.600	0.778
ZNF583	15.441	-0.080	0.519	0.878	0.921	15.441	-0.565	0.530	0.287	0.556	15.441	-0.485	0.617	0.431	0.654
FAM98C	273.378	0.215	0.267	0.421	0.558	273.378	0.572	0.265	0.031	0.322	273.378	0.357	0.307	0.245	0.476
CAPN12	658.743	2.234	0.376	0.000	0.000	658.743	0.143	0.378	0.706	0.842	658.743	-2.091	0.435	0.000	0.000
DPY19L3	317.831	-0.754	0.186	0.000	0.001	317.831	-0.307	0.183	0.093	0.388	317.831	0.447	0.215	0.037	0.152
TICAM1	1014.047	0.106	0.125	0.395	0.533	1014.047	0.116	0.124	0.350	0.605	1014.047	0.010	0.144	0.944	0.975
ZNF599	43.344	-0.013	0.330	0.968	0.980	43.344	0.448	0.320	0.162	0.453	43.344	0.461	0.376	0.221	0.448
PROSER3	123.036	-0.333	0.201	0.096	0.190	123.036	0.070	0.195	0.720	0.850	123.036	0.403	0.230	0.080	0.245
ZNF558	255.012	-0.119	0.210	0.569	0.688	255.012	0.258	0.207	0.212	0.493	255.012	0.378	0.241	0.117	0.307
CDC42EP5	98.379	2.866	0.947	0.002	0.012	98.379	0.523	0.953	0.583	0.767	98.379	-2.343	1.094	0.032	0.138
LINC00662	409.900	-0.209	0.179	0.243	0.376	409.900	-0.051	0.178	0.774	0.880	409.900	0.158	0.207	0.444	0.664
NKX2-5	517.233	-0.097	0.292	0.738	0.823	517.233	-0.296	0.292	0.311	0.575	517.233	-0.198	0.337	0.557	0.749
ZNF738	35.699	0.954	0.335	0.004	0.018	35.699	-0.030	0.349	0.931	0.965	35.699	-0.984	0.394	0.012	0.075
ZNF714	47.594	0.171	0.277	0.537	0.662	47.594	0.184	0.274	0.502	0.714	47.594	0.013	0.318	0.967	0.985
C19orf25	412.444	-0.192	0.260	0.462	0.596	412.444	-0.463	0.261	0.076	0.365	412.444	-0.271	0.302	0.369	0.601
ATP8B3	264.252	-0.076	0.540	0.888	0.927	264.252	0.976	0.538	0.070	0.358	264.252	1.053	0.623	0.091	0.264

DIRAS1	66.274	0.749	0.372	0.044	0.105	66.274	-0.072	0.380	0.851	0.923	66.274	-0.820	0.434	0.059	0.202
ZNF555	63.386	0.395	0.279	0.157	0.273	63.386	0.390	0.277	0.158	0.450	63.386	-0.005	0.320	0.989	0.994
ZNF569	90.667	-0.704	0.231	0.002	0.011	90.667	-0.069	0.220	0.755	0.869	90.667	0.635	0.265	0.017	0.091
ZNF570	40.512	0.245	0.342	0.475	0.608	40.512	0.427	0.335	0.203	0.486	40.512	0.182	0.391	0.640	0.805
C1orf74	191.517	-0.166	0.160	0.299	0.438	191.517	-0.200	0.158	0.208	0.490	191.517	-0.034	0.185	0.855	0.930
CREB3L4	162.114	0.244	0.250	0.329	0.469	162.114	0.439	0.249	0.077	0.368	162.114	0.195	0.288	0.499	0.706
BROX	707.812	-0.302	0.149	0.043	0.102	707.812	0.023	0.148	0.874	0.936	707.812	0.326	0.172	0.058	0.201
SAMD11	840.263	0.923	0.310	0.003	0.013	840.263	-0.209	0.311	0.502	0.714	840.263	-1.132	0.359	0.002	0.019
MRPL20-AS1	376.151	0.061	0.193	0.754	0.836	376.151	-0.228	0.194	0.240	0.516	376.151	-0.288	0.224	0.198	0.421
SAMD13	11.079	-0.440	0.780	0.573	0.692	11.079	0.458	0.752	0.542	0.738	11.079	0.898	0.889	0.313	0.548
C1orf52	303.574	-0.129	0.171	0.448	0.583	303.574	-0.228	0.170	0.181	0.469	303.574	-0.098	0.198	0.620	0.792
PHF13	754.681	0.158	0.190	0.404	0.542	754.681	0.324	0.189	0.087	0.380	754.681	0.165	0.219	0.450	0.669
TLCD4	264.628	0.452	0.260	0.083	0.169	264.628	0.188	0.261	0.471	0.693	264.628	-0.264	0.301	0.379	0.610
CTBS	179.393	0.112	0.259	0.666	0.767	179.393	0.414	0.257	0.107	0.399	179.393	0.302	0.298	0.310	0.546
LINC00337	99.213	-0.903	0.521	0.083	0.170	99.213	-1.099	0.521	0.035	0.323	99.213	-0.196	0.605	0.746	0.869
CTBP1	2186.734	0.433	0.170	0.011	0.036	2186.734	-0.073	0.170	0.670	0.822	2186.734	-0.506	0.197	0.010	0.066
B3GALNT2	975.764	-0.689	0.209	0.001	0.006	975.764	-0.284	0.208	0.173	0.462	975.764	0.406	0.241	0.093	0.268
CTBP2	2315.046	-0.477	0.158	0.003	0.012	2315.046	-0.270	0.158	0.088	0.380	2315.046	0.207	0.183	0.257	0.491
SLC30A7	438.220	0.100	0.284	0.726	0.814	438.220	-0.487	0.285	0.088	0.381	438.220	-0.586	0.329	0.075	0.236
ZNF436-AS1	52.760	0.377	0.599	0.529	0.655	52.760	1.180	0.593	0.046	0.335	52.760	0.804	0.686	0.241	0.472
MOB3C	204.516	0.245	0.172	0.153	0.268	204.516	0.201	0.171	0.240	0.516	204.516	-0.045	0.198	0.821	0.911
GLIS1	30.508	-0.783	0.574	0.173	0.293	30.508	0.747	0.549	0.174	0.463	30.508	1.530	0.653	0.019	0.099
CCN2	152.302	-0.620	0.671	0.356	0.496	152.302	-2.707	0.681	0.000	0.093	152.302	-2.087	0.785	0.008	0.056
NBPF12	293.178	0.129	0.232	0.578	0.696	293.178	0.219	0.231	0.344	0.600	293.178	0.090	0.268	0.737	0.865
RC3H1	285.139	0.145	0.228	0.525	0.652	285.139	-0.232	0.229	0.311	0.575	285.139	-0.377	0.265	0.154	0.364
ZNF362	110.705	0.958	0.404	0.018	0.053	110.705	0.339	0.406	0.404	0.646	110.705	-0.620	0.467	0.184	0.404
DCST1	8.850	-0.447	0.684	0.514	0.642	8.850	0.652	0.626	0.298	0.565	8.850	1.098	0.765	0.151	0.360
CTH	478.306	-2.751	0.387	0.000	0.000	478.306	-0.808	0.380	0.034	0.322	478.306	1.943	0.445	0.000	0.000
CNIH3	13.200	-0.512	0.435	0.240	0.373	13.200	0.174	0.392	0.657	0.813	13.200	0.686	0.491	0.162	0.376
MANEAL	231.146	0.194	0.210	0.355	0.495	231.146	-0.348	0.212	0.101	0.394	231.146	-0.542	0.245	0.027	0.122
FAM78B	9.825	-0.427	0.614	0.486	0.619	9.825	0.016	0.585	0.978	0.987	9.825	0.444	0.704	0.528	0.729
SHISA4	129.642	1.208	0.436	0.006	0.021	129.642	0.629	0.437	0.151	0.443	129.642	-0.579	0.502	0.249	0.481
EXOC8	247.542	-0.696	0.213	0.001	0.006	247.542	-0.245	0.209	0.242	0.518	247.542	0.452	0.246	0.066	0.217
PDIK1L	136.496	-0.447	0.211	0.035	0.087	136.496	0.192	0.204	0.347	0.603	136.496	0.639	0.242	0.008	0.058
CFAP57	309.964	-1.146	0.367	0.002	0.009	309.964	-0.765	0.365	0.036	0.324	309.964	0.381	0.424	0.369	0.601
CCDC24	96.343	1.373	0.259	0.000	0.000	96.343	0.146	0.269	0.587	0.769	96.343	-1.227	0.303	0.000	0.001
BTBD19	18.632	0.825	0.489	0.092	0.184	18.632	-0.116	0.511	0.820	0.906	18.632	-0.941	0.577	0.103	0.285
CTNNA1	6660.370	-0.255	0.099	0.010	0.033	6660.370	-0.001	0.098	0.994	0.997	6660.370	0.254	0.114	0.026	0.119
RNF187	2956.713	0.389	0.269	0.148	0.262	2956.713	0.519	0.269	0.054	0.342	2956.713	0.130	0.310	0.675	0.828
CTNS	126.531	0.363	0.220	0.099	0.194	126.531	-0.137	0.223	0.539	0.736	126.531	-0.500	0.257	0.052	0.186
APCDD1L-DT	182.562	-0.058	0.259	0.823	0.885	182.562	-0.290	0.260	0.264	0.536	182.562	-0.232	0.301	0.440	0.661
SHLD1	28.995	-0.022	0.307	0.943	0.964	28.995	-0.021	0.301	0.945	0.973	28.995	0.001	0.356	0.997	0.998
CTNNB1	6335.895	-0.102	0.177	0.564	0.685	6335.895	-0.155	0.177	0.381	0.627	6335.895	-0.053	0.205	0.796	0.897
COMMD7	342.402	0.558	0.203	0.006	0.023	342.402	-0.060	0.205	0.768	0.877	342.402	-0.619	0.236	0.009	0.059
LSM14B	1004.473	-0.155	0.117	0.184	0.308	1004.473	0.076	0.116	0.513	0.720	1004.473	0.231	0.135	0.087	0.257
LIPI	36.775	-0.275	0.621	0.657	0.760	36.775	0.595	0.608	0.328	0.588	36.775	0.870	0.711	0.221	0.448
CTNND1	664.660	-0.016	0.240	0.945	0.966	664.660	0.241	0.240	0.314	0.577	664.660	0.258	0.277	0.352	0.586
LCA5L	15.693	0.043	0.484	0.930	0.956	15.693	-0.179	0.486	0.713	0.846	15.693	-0.221	0.566	0.696	0.841
ZNF295-AS1	9.478	-0.650	0.727	0.372	0.512	9.478	0.869	0.572	0.128	0.420	9.478	1.519	0.775	0.050	0.183
SLC9B1	301.431	-0.322	0.185	0.082	0.168	301.431	-0.133	0.183	0.465	0.689	301.431	0.188	0.214	0.378	0.609
XKR3	119.817	1.261	0.229	0.000	0.000	119.817	0.467	0.233	0.045	0.334	119.817	-0.794	0.264	0.003	0.026
RIMBP3C	18.069	1.748	0.489	0.000	0.003	18.069	0.850	0.504	0.092	0.386	18.069	-0.898	0.556	0.106	0.290
YDJC	886.340	-0.049	0.303	0.870	0.916	886.340	-0.431	0.303	0.155	0.447	886.340	-0.381	0.350	0.276	0.511
C22orf15	283.999	1.246	0.377	0.001	0.006	283.999	-0.070	0.379	0.854	0.925	283.999	-1.316	0.436	0.003	0.026
HSCB	500.781	0.368	0.165	0.026	0.071	500.781	0.173	0.166	0.296	0.563	500.781	-0.195	0.191	0.308	0.543
CCDC117	503.938	-0.291	0.215	0.176	0.297	503.938	-0.383	0.215	0.074	0.364	503.938	-0.092	0.249	0.711	0.850
DUSP18	29.496	0.071	0.293	0.807	0.873	29.496	-0.313	0.299	0.294	0.562	29.496	-0.384	0.347	0.268	0.503
MORC2-AS1	65.832	1.013	0.337	0.003	0.012	65.832	0.175	0.344	0.612	0.786	65.832	-0.838	0.391	0.032	0.138
CTPS1	963.404	-0.993	0.448	0.027	0.072	963.404	-1.171	0.448	0.009	0.297	963.404	-0.178	0.518	0.731	0.861
DNAJB7	24.307	-0.612	0.338	0.070	0.150	24.307	-0.204	0.318	0.522	0.726	24.307	0.409	0.390	0.295	0.530
CHADL	21.299	-0.570	0.435	0.190	0.314	21.299	-1.122	0.449	0.012	0.305	21.299	-0.552	0.528	0.296	0.531
NFAM1	24.100	1.366	1.059	0.197	0.323	24.100	0.680	1.062	0.522	0.726	24.100	-0.686	1.221	0.574	0.761
PRR34-AS1	63.527	-0.226	0.406	0.577	0.695	63.527	-0.523	0.408	0.200	0.483	63.527	-0.297	0.475	0.532	0.731
CDPF1	126.240	-0.304	0.280	0.277	0.414	126.240	-0.090	0.277	0.745	0.864	126.240	0.214	0.323	0.508	0.713
GTSE1-DT	10.260	-0.658	0.616	0.285	0.423	10.260	-1.355	0.645	0.036	0.324	10.260	-0.697	0.755	0.356	0.590
TTL	1046.917	-0.624	0.325	0.055	0.124	1046.917	-0.766	0.325	0.019	0.306	1046.917	-0.141	0.376	0.707	0.848
CKAP2L	822.849	-0.883	0.188	0.000	0.000	822.849	-0.160	0.187	0.391	0.635	822.849	0.722	0.217	0.001	0.012

CBWD2	548.972	-0.195	0.167	0.244	0.377	548.972	-0.259	0.167	0.120	0.412	548.972	-0.064	0.194	0.740	0.867
SATB2-AS1	13.097	-0.691	0.567	0.223	0.354	13.097	-0.141	0.539	0.794	0.892	13.097	0.550	0.652	0.399	0.626
C2orf15	79.469	-0.690	0.309	0.026	0.070	79.469	-0.429	0.304	0.157	0.450	79.469	0.261	0.359	0.467	0.682
CTRL	39.843	0.622	0.398	0.119	0.222	39.843	-0.588	0.418	0.159	0.451	39.843	-1.210	0.475	0.011	0.069
COPS9	279.582	0.436	0.354	0.218	0.348	279.582	-0.468	0.356	0.189	0.475	279.582	-0.904	0.410	0.028	0.125
COMMD1	208.981	0.598	0.194	0.002	0.010	208.981	0.073	0.197	0.710	0.845	208.981	-0.525	0.225	0.020	0.102
ANKAR	29.536	-0.292	0.323	0.366	0.506	29.536	0.347	0.301	0.248	0.522	29.536	0.640	0.365	0.080	0.244
FBXO41	145.826	-0.205	0.331	0.537	0.662	145.826	0.458	0.327	0.161	0.452	145.826	0.663	0.380	0.081	0.247
TTC30B	42.593	-0.259	0.439	0.555	0.677	42.593	0.393	0.429	0.360	0.612	42.593	0.651	0.503	0.195	0.417
LINC00342	30.685	2.155	0.475	0.000	0.000	30.685	0.610	0.491	0.214	0.493	30.685	-1.546	0.548	0.005	0.040
GPAT2	18.664	1.624	0.520	0.002	0.009	18.664	0.594	0.536	0.268	0.540	18.664	-1.030	0.599	0.085	0.254
LOC150776	191.169	0.657	0.284	0.021	0.059	191.169	-0.146	0.287	0.611	0.785	191.169	-0.803	0.330	0.015	0.084
RAB6D	12.381	0.635	0.475	0.181	0.304	12.381	0.002	0.499	0.997	0.999	12.381	-0.633	0.561	0.259	0.494
CTSB	7211.330	0.974	0.149	0.000	0.000	7211.330	0.268	0.149	0.072	0.361	7211.330	-0.706	0.172	0.000	0.001
FAM117B	671.343	-0.356	0.335	0.287	0.425	671.343	0.395	0.333	0.236	0.513	671.343	0.751	0.386	0.052	0.186
CTSD	30298.277	1.577	0.427	0.000	0.002	30298.277	0.831	0.427	0.051	0.339	30298.277	-0.745	0.493	0.130	0.328
GAREM2	32.268	-0.195	0.364	0.591	0.706	32.268	-0.429	0.365	0.240	0.516	32.268	-0.233	0.427	0.585	0.767
PUS10	101.337	-0.077	0.187	0.680	0.778	101.337	-0.073	0.185	0.694	0.835	101.337	0.004	0.217	0.984	0.991
LINC01963	41.873	0.236	0.427	0.580	0.698	41.873	-0.429	0.433	0.322	0.583	41.873	-0.665	0.499	0.183	0.402
LINC01106	36.017	-0.146	0.319	0.648	0.754	36.017	0.145	0.309	0.638	0.803	36.017	0.291	0.366	0.426	0.650
SEPTIN10	2375.178	-0.147	0.176	0.402	0.540	2375.178	0.107	0.176	0.543	0.739	2375.178	0.254	0.203	0.211	0.436
KANSL1L	224.322	0.250	0.209	0.233	0.365	224.322	0.257	0.208	0.217	0.497	224.322	0.007	0.241	0.976	0.987
ZNF385B	422.947	-0.980	0.472	0.038	0.094	422.947	0.377	0.470	0.423	0.659	422.947	1.357	0.545	0.013	0.076
ERFE	281.325	-0.726	0.257	0.005	0.019	281.325	-0.045	0.254	0.860	0.929	281.325	0.681	0.296	0.021	0.106
ARL6IP6	491.221	-0.635	0.214	0.003	0.014	491.221	0.176	0.212	0.405	0.646	491.221	0.812	0.247	0.001	0.013
METTL21A	139.466	0.149	0.234	0.523	0.650	139.466	-0.509	0.238	0.032	0.322	139.466	-0.658	0.274	0.016	0.090
CCNYL1	126.588	-0.193	0.320	0.548	0.671	126.588	-0.787	0.324	0.015	0.306	126.588	-0.594	0.375	0.113	0.301
CTSH	274.886	1.638	0.512	0.001	0.007	274.886	0.462	0.513	0.368	0.618	274.886	-1.176	0.591	0.047	0.175
KLHL23	155.662	-0.179	0.187	0.337	0.477	155.662	-0.101	0.185	0.583	0.766	155.662	0.078	0.216	0.718	0.854
PPP1R1C	11.694	1.067	0.508	0.036	0.090	11.694	0.945	0.506	0.062	0.350	11.694	-0.122	0.570	0.831	0.917
SGO2	387.702	-0.243	0.182	0.182	0.306	387.702	-0.010	0.181	0.957	0.978	387.702	0.233	0.210	0.267	0.502
CTSK	14.111	2.726	0.663	0.000	0.000	14.111	0.502	0.736	0.496	0.710	14.111	-2.225	0.773	0.004	0.035
FAHD2B	248.162	0.268	0.207	0.194	0.319	248.162	0.256	0.206	0.214	0.494	248.162	-0.013	0.238	0.957	0.980
LRATD1	176.055	0.993	0.233	0.000	0.000	176.055	0.193	0.236	0.414	0.652	176.055	-0.800	0.270	0.003	0.029
RMDN2	26.391	0.039	0.333	0.907	0.941	26.391	-0.370	0.341	0.278	0.548	26.391	-0.409	0.397	0.303	0.538
CTSL	1431.386	0.633	0.299	0.034	0.087	1431.386	-0.348	0.299	0.245	0.520	1431.386	-0.981	0.345	0.004	0.038
LINC00471	9.564	-1.074	0.514	0.037	0.091	9.564	-0.468	0.460	0.309	0.574	9.564	0.607	0.593	0.307	0.542
CTSV	71.679	-0.031	0.462	0.947	0.967	71.679	0.105	0.460	0.819	0.905	71.679	0.136	0.534	0.798	0.899
WDSUB1	148.236	0.166	0.199	0.403	0.541	148.236	0.341	0.196	0.083	0.374	148.236	0.174	0.228	0.446	0.665
LBX2-AS1	9.229	1.874	0.616	0.002	0.011	9.229	0.917	0.641	0.153	0.445	9.229	-0.958	0.694	0.168	0.382
GPR155	219.857	0.507	0.474	0.285	0.423	219.857	0.569	0.474	0.230	0.508	219.857	0.062	0.547	0.910	0.959
TTC14	149.160	0.226	0.227	0.321	0.460	149.160	-0.111	0.228	0.628	0.796	149.160	-0.336	0.264	0.202	0.426
DTX3L	1252.899	0.058	0.127	0.650	0.755	1252.899	0.088	0.127	0.491	0.707	1252.899	0.030	0.147	0.840	0.923
SGO1	315.818	-0.578	0.273	0.034	0.087	315.818	-0.318	0.272	0.243	0.519	315.818	0.261	0.316	0.409	0.636
PP2D1	175.349	-0.132	0.232	0.570	0.689	175.349	-0.279	0.232	0.229	0.507	175.349	-0.147	0.270	0.586	0.767
PPM1L	105.676	-0.065	0.275	0.812	0.877	105.676	0.049	0.273	0.858	0.928	105.676	0.114	0.318	0.720	0.854
CCDC80	1121.736	-0.334	0.662	0.614	0.725	1121.736	0.399	0.662	0.546	0.741	1121.736	0.733	0.765	0.338	0.572
CTSO	166.766	1.042	0.581	0.073	0.154	166.766	0.615	0.582	0.291	0.558	166.766	-0.427	0.671	0.524	0.726
CCDC12	650.619	0.529	0.237	0.026	0.070	650.619	-0.154	0.238	0.518	0.723	650.619	-0.683	0.275	0.013	0.077
MB21D2	220.440	1.521	0.599	0.011	0.037	220.440	-0.690	0.603	0.253	0.526	220.440	-2.211	0.695	0.001	0.017
PPP4R2	1405.021	-0.661	0.163	0.000	0.001	1405.021	-0.241	0.162	0.137	0.428	1405.021	0.420	0.188	0.025	0.118
XXYLT1	450.849	0.384	0.125	0.002	0.010	450.849	-0.184	0.126	0.146	0.438	450.849	-0.568	0.145	0.000	0.002
RNF38	565.229	-0.135	0.267	0.613	0.725	565.229	0.324	0.266	0.224	0.503	565.229	0.459	0.308	0.137	0.339
GLIPR2	51.989	0.169	0.275	0.540	0.665	51.989	-0.115	0.277	0.678	0.826	51.989	-0.284	0.321	0.377	0.609
LOC152048	109.005	-0.584	0.378	0.123	0.228	109.005	-0.038	0.373	0.920	0.960	109.005	0.546	0.436	0.210	0.435
ZCWPW2	136.428	-0.651	0.274	0.017	0.052	136.428	0.225	0.266	0.398	0.642	136.428	0.875	0.313	0.005	0.042
CMC1	1338.070	0.122	0.151	0.418	0.555	1338.070	0.258	0.151	0.086	0.379	1338.070	0.136	0.174	0.435	0.656
CCDC50	3083.723	-0.384	0.080	0.000	0.000	3083.723	-0.100	0.080	0.211	0.491	3083.723	0.284	0.093	0.002	0.023
SPICE1	31.651	0.303	0.404	0.453	0.588	31.651	-0.079	0.409	0.848	0.921	31.651	-0.382	0.471	0.418	0.643
CMTM8	15.569	0.691	0.498	0.165	0.284	15.569	-0.354	0.535	0.508	0.717	15.569	-1.045	0.600	0.082	0.248
NUDT16P1	118.993	-0.378	0.330	0.252	0.387	118.993	0.514	0.324	0.113	0.405	118.993	0.891	0.378	0.018	0.097
CTSZ	2570.202	0.752	0.155	0.000	0.000	2570.202	-0.114	0.155	0.464	0.689	2570.202	-0.866	0.179	0.000	0.000
NCBP2AS2	601.234	-0.069	0.258	0.788	0.860	601.234	-0.364	0.259	0.159	0.451	601.234	-0.295	0.299	0.324	0.558
CUX1	1486.380	0.194	0.200	0.331	0.471	1486.380	0.246	0.200	0.218	0.498	1486.380	0.051	0.231	0.824	0.913
CIDECP1	115.591	0.040	0.228	0.860	0.908	115.591	-0.274	0.229	0.231	0.508	115.591	-0.314	0.266	0.237	0.466
IGSF11	36.571	-0.854	0.508	0.093	0.185	36.571	0.312	0.489	0.524	0.727	36.571	1.166	0.581	0.045	0.170
ZNF827	574.923	-0.355	0.140	0.011	0.037	574.923	-0.302	0.139	0.030	0.322	574.923	0.053	0.162	0.745	0.869

CXADR	231.274	1.349	0.276	0.000	0.000	231.274	0.766	0.277	0.006	0.297	231.274	-0.584	0.318	0.066	0.217
SH3D19	462.029	-0.650	0.201	0.001	0.007	462.029	0.014	0.199	0.942	0.971	462.029	0.664	0.232	0.004	0.036
NFXL1	312.426	-0.919	0.215	0.000	0.000	312.426	-0.383	0.211	0.069	0.358	312.426	0.536	0.248	0.031	0.134
NIPAL1	34.248	-0.381	0.346	0.270	0.407	34.248	-0.674	0.349	0.053	0.342	34.248	-0.293	0.410	0.475	0.689
PAQR3	592.649	-0.608	0.346	0.079	0.163	592.649	-0.845	0.346	0.015	0.306	592.649	-0.237	0.400	0.554	0.747
SCFD2	294.358	-0.447	0.232	0.054	0.122	294.358	-0.512	0.231	0.027	0.319	294.358	-0.065	0.269	0.809	0.904
WWC2-AS2	11.681	-0.064	0.606	0.916	0.947	11.681	0.740	0.573	0.196	0.480	11.681	0.804	0.681	0.238	0.467
FAM218A	12.372	-0.060	0.500	0.904	0.938	12.372	-0.605	0.514	0.239	0.516	12.372	-0.544	0.596	0.361	0.594
CYB5A	1145.688	0.789	0.184	0.000	0.000	1145.688	0.207	0.184	0.260	0.532	1145.688	-0.582	0.212	0.006	0.047
THAP6	235.316	-0.730	0.194	0.000	0.001	235.316	-0.264	0.190	0.165	0.455	235.316	0.466	0.224	0.038	0.153
FAM53A	46.358	-0.776	0.284	0.006	0.024	46.358	-0.350	0.269	0.194	0.478	46.358	0.426	0.329	0.195	0.417
PPM1K	256.809	0.669	0.176	0.000	0.001	256.809	0.221	0.177	0.211	0.491	256.809	-0.448	0.203	0.027	0.124
TRMT44	211.257	0.029	0.159	0.855	0.906	211.257	0.004	0.158	0.982	0.989	211.257	-0.025	0.184	0.890	0.948
ADRB1	10.536	0.654	0.506	0.196	0.322	10.536	0.279	0.513	0.586	0.768	10.536	-0.375	0.584	0.521	0.723
DAB2IP	762.390	-0.005	0.367	0.990	0.994	762.390	0.136	0.367	0.710	0.845	762.390	0.141	0.424	0.740	0.866
SLC38A9	201.255	-0.067	0.211	0.751	0.834	201.255	-0.621	0.214	0.004	0.297	201.255	-0.554	0.248	0.025	0.118
CREBRF	332.749	0.227	0.328	0.488	0.621	332.749	0.339	0.327	0.299	0.566	332.749	0.112	0.378	0.767	0.881
CEP120	491.670	-1.442	0.242	0.000	0.000	491.670	-0.410	0.238	0.085	0.378	491.670	1.032	0.279	0.000	0.004
TMEM167A	1062.710	-0.130	0.191	0.497	0.628	1062.710	-0.521	0.192	0.007	0.297	1062.710	-0.391	0.222	0.077	0.240
MBLAC2	295.264	-0.712	0.223	0.001	0.008	295.264	-0.106	0.220	0.630	0.798	295.264	0.606	0.257	0.018	0.096
TMEM161B	291.972	-0.678	0.200	0.001	0.004	291.972	-0.148	0.196	0.452	0.680	291.972	0.531	0.230	0.021	0.105
CYB561	876.173	-0.163	0.146	0.265	0.401	876.173	-0.157	0.146	0.280	0.550	876.173	0.005	0.169	0.974	0.987
SRFBP1	936.587	-0.584	0.316	0.064	0.139	936.587	0.123	0.314	0.696	0.837	936.587	0.707	0.364	0.052	0.187
CYBA	633.919	1.363	0.255	0.000	0.000	633.919	0.335	0.256	0.191	0.477	633.919	-1.028	0.295	0.000	0.008
ZMAT2	885.722	-0.164	0.123	0.185	0.309	885.722	-0.049	0.123	0.687	0.831	885.722	0.114	0.143	0.424	0.648
MARVELD2	25.536	-0.315	0.378	0.405	0.543	25.536	-0.069	0.365	0.850	0.923	25.536	0.246	0.436	0.573	0.760
C5orf38	19951.680	0.700	0.270	0.009	0.033	19951.680	0.484	0.270	0.073	0.362	19951.680	-0.216	0.311	0.488	0.698
IRX2	72963.488	-0.365	0.150	0.015	0.047	72963.488	-0.065	0.150	0.666	0.819	72963.488	0.300	0.174	0.084	0.251
ARSK	614.316	-0.256	0.352	0.468	0.602	614.316	0.398	0.351	0.257	0.529	614.316	0.654	0.406	0.107	0.292
LOC153684	42.858	-0.612	0.267	0.022	0.061	42.858	-0.165	0.253	0.513	0.720	42.858	0.447	0.307	0.146	0.353
CYC1	4721.949	-0.222	0.180	0.217	0.347	4721.949	0.016	0.180	0.931	0.965	4721.949	0.238	0.208	0.252	0.485
CCDC112	273.630	0.071	0.205	0.730	0.817	273.630	-0.028	0.204	0.892	0.946	273.630	-0.098	0.237	0.678	0.829
PRELID2	137.648	0.481	0.345	0.164	0.284	137.648	0.573	0.344	0.096	0.391	137.648	0.092	0.397	0.817	0.909
SH3RF2	430.299	0.118	0.194	0.544	0.668	430.299	0.270	0.193	0.162	0.453	430.299	0.152	0.224	0.496	0.704
RNF145	1012.291	0.406	0.163	0.013	0.041	1012.291	0.015	0.163	0.925	0.962	1012.291	-0.391	0.189	0.038	0.154
ADRB2	814.931	-0.133	0.262	0.611	0.723	814.931	0.237	0.262	0.365	0.617	814.931	0.370	0.303	0.221	0.449
CYLD	935.809	0.100	0.189	0.599	0.712	935.809	-0.301	0.190	0.114	0.405	935.809	-0.400	0.219	0.068	0.222
SNRNP48	483.492	-0.786	0.161	0.000	0.000	483.492	-0.022	0.157	0.890	0.945	483.492	0.764	0.185	0.000	0.001
CNKSR3	506.444	0.526	0.198	0.008	0.028	506.444	-0.371	0.200	0.064	0.351	506.444	-0.897	0.230	0.000	0.002
SAMD3	12.065	-0.255	0.484	0.599	0.712	12.065	0.048	0.463	0.918	0.959	12.065	0.302	0.555	0.586	0.767
SLC2A12	95.299	0.926	0.592	0.118	0.221	95.299	0.200	0.594	0.736	0.859	95.299	-0.726	0.684	0.289	0.524
MBOAT1	160.570	-0.211	0.176	0.230	0.363	160.570	-0.105	0.173	0.544	0.739	160.570	0.106	0.204	0.603	0.780
RNF217	341.095	-0.032	0.242	0.895	0.932	341.095	0.165	0.241	0.495	0.709	341.095	0.197	0.280	0.482	0.694
CFAP206	10.115	0.022	0.750	0.977	0.986	10.115	0.261	0.737	0.723	0.852	10.115	0.240	0.861	0.781	0.890
BVES-AS1	7.876	1.338	0.589	0.023	0.064	7.876	0.065	0.662	0.921	0.960	7.876	-1.272	0.708	0.072	0.230
CCDC167	166.269	1.069	0.231	0.000	0.000	166.269	-0.001	0.237	0.997	0.999	166.269	-1.070	0.270	0.000	0.002
CYP1B1	10.274	0.498	0.616	0.418	0.555	10.274	0.140	0.623	0.822	0.907	10.274	-0.358	0.714	0.616	0.789
RUNDC3B	128.701	-0.205	0.244	0.400	0.538	128.701	0.000	0.241	0.999	0.999	128.701	0.206	0.281	0.465	0.681
ABCA13	37.341	1.854	0.497	0.000	0.002	37.341	-0.577	0.589	0.327	0.587	37.341	-2.431	0.630	0.000	0.003
BMT2	225.236	-0.103	0.309	0.739	0.824	225.236	0.091	0.307	0.768	0.877	225.236	0.193	0.356	0.587	0.768
LOC154761	475.530	0.777	0.291	0.008	0.027	475.530	0.552	0.291	0.058	0.348	475.530	-0.225	0.335	0.502	0.709
FMC1	101.761	0.431	0.295	0.144	0.257	101.761	0.378	0.294	0.199	0.482	101.761	-0.053	0.340	0.875	0.940
AMOT	90.691	0.087	0.624	0.889	0.928	90.691	0.565	0.622	0.363	0.615	90.691	0.478	0.719	0.506	0.712
VKORC1L1	1093.953	-0.770	0.162	0.000	0.000	1093.953	-0.083	0.161	0.605	0.781	1093.953	0.687	0.187	0.000	0.005
AMOTL1	937.910	-0.782	0.265	0.003	0.014	937.910	-0.187	0.265	0.480	0.699	937.910	0.595	0.306	0.052	0.187
FEZF1-AS1	8.371	1.170	0.615	0.057	0.128	8.371	1.982	0.585	0.001	0.258	8.371	0.812	0.658	0.217	0.443
IQUB	10.124	0.608	0.747	0.416	0.554	10.124	0.770	0.739	0.298	0.565	10.124	0.162	0.852	0.849	0.927
KCTD7	56.818	-0.238	0.367	0.517	0.645	56.818	0.172	0.360	0.633	0.800	56.818	0.410	0.422	0.331	0.566
TMEM213	11.123	0.083	0.517	0.873	0.918	11.123	-0.559	0.540	0.300	0.567	11.123	-0.642	0.623	0.302	0.537
ZNF425	24.893	-0.224	0.570	0.694	0.789	24.893	0.287	0.554	0.604	0.781	24.893	0.512	0.653	0.433	0.655
LOC155060	25.879	0.454	0.472	0.336	0.476	25.879	0.532	0.468	0.255	0.529	25.879	0.078	0.541	0.885	0.945
ZNF746	378.976	-0.239	0.222	0.283	0.420	378.976	-0.267	0.222	0.229	0.506	378.976	-0.028	0.257	0.913	0.960
ATP6V0E2	39.357	0.827	0.292	0.005	0.018	39.357	-0.215	0.308	0.485	0.702	39.357	-1.042	0.346	0.003	0.026
AMZ1	58.386	1.987	0.320	0.000	0.000	58.386	0.939	0.332	0.005	0.297	58.386	-1.048	0.363	0.004	0.034
METTL27	91.000	0.698	0.243	0.004	0.017	91.000	0.509	0.243	0.036	0.324	91.000	-0.189	0.278	0.497	0.704
SBDSP1	294.756	-1.127	0.338	0.001	0.005	294.756	-0.564	0.335	0.092	0.387	294.756	0.563	0.391	0.149	0.358
VPS37D	30.654	0.602	0.821	0.464	0.598	30.654	0.662	0.819	0.419	0.657	30.654	0.060	0.945	0.949	0.976

NSUN5P1	147.264	0.758	0.252	0.003	0.012	147.264	0.246	0.254	0.334	0.593	147.264	-0.512	0.292	0.080	0.244
RBM33	801.559	-0.379	0.148	0.011	0.036	801.559	-0.005	0.147	0.971	0.985	801.559	0.374	0.171	0.029	0.129
GRK2	1590.449	0.068	0.084	0.416	0.554	1590.449	-0.051	0.084	0.538	0.735	1590.449	-0.119	0.097	0.217	0.443
GRK3	175.650	-0.179	0.227	0.430	0.566	175.650	-0.270	0.226	0.233	0.510	175.650	-0.091	0.263	0.730	0.860
PRAG1	172.455	0.338	0.283	0.232	0.365	172.455	-0.219	0.285	0.442	0.673	172.455	-0.557	0.329	0.090	0.263
CYP2J2	118.899	1.481	0.242	0.000	0.000	118.899	0.396	0.248	0.110	0.403	118.899	-1.085	0.280	0.000	0.003
PEBP4	12.032	-0.906	0.670	0.177	0.299	12.032	-0.076	0.635	0.905	0.952	12.032	0.830	0.767	0.279	0.514
CDCA2	1025.690	-0.070	0.267	0.794	0.864	1025.690	0.278	0.267	0.298	0.565	1025.690	0.347	0.308	0.260	0.494
TMEM65	661.176	0.006	0.148	0.969	0.981	661.176	-0.018	0.147	0.903	0.951	661.176	-0.024	0.171	0.889	0.947
LINC00964	16.073	0.251	0.562	0.655	0.758	16.073	1.094	0.539	0.042	0.330	16.073	0.842	0.631	0.182	0.400
SDAD1P1	66.334	0.379	0.556	0.496	0.627	66.334	0.768	0.552	0.164	0.454	66.334	0.390	0.639	0.542	0.738
RDH10	87.723	-0.286	0.211	0.176	0.298	87.723	-0.367	0.210	0.080	0.371	87.723	-0.081	0.248	0.743	0.868
ANKRD46	268.249	-0.349	0.227	0.124	0.229	268.249	0.165	0.224	0.460	0.686	268.249	0.515	0.261	0.049	0.180
ESCO2	191.460	-0.950	0.447	0.033	0.085	191.460	-1.360	0.448	0.002	0.297	191.460	-0.410	0.519	0.429	0.652
FBXO16	8.845	0.776	0.751	0.301	0.440	8.845	0.525	0.753	0.486	0.703	8.845	-0.251	0.860	0.770	0.883
LRATD2	82.456	-1.145	0.280	0.000	0.000	82.456	-0.565	0.267	0.034	0.322	82.456	0.580	0.324	0.073	0.231
C8orf37	71.826	0.063	0.272	0.818	0.880	71.826	0.290	0.267	0.277	0.547	71.826	0.228	0.312	0.465	0.681
VPS13B	304.850	-0.704	0.320	0.028	0.074	304.850	0.114	0.317	0.719	0.849	304.850	0.818	0.369	0.026	0.121
ERICH1	390.606	0.489	0.126	0.000	0.001	390.606	-0.041	0.128	0.751	0.867	390.606	-0.530	0.147	0.000	0.006
CYP3A5	27.794	-0.309	0.370	0.404	0.542	27.794	0.047	0.357	0.895	0.948	27.794	0.356	0.425	0.402	0.629
TMEM74	27.923	-0.321	0.356	0.366	0.507	27.923	-0.351	0.352	0.318	0.580	27.923	-0.030	0.416	0.943	0.974
FAM91A1	1057.593	-0.607	0.263	0.021	0.059	1057.593	-0.514	0.262	0.050	0.337	1057.593	0.093	0.303	0.758	0.877
MCMDC2	879.386	0.686	0.331	0.038	0.094	879.386	0.550	0.331	0.096	0.391	879.386	-0.137	0.382	0.720	0.855
CLVS1	555.838	0.798	0.261	0.002	0.011	555.838	0.283	0.261	0.279	0.549	555.838	-0.515	0.301	0.087	0.258
SBSPON	16.840	-0.492	0.484	0.310	0.449	16.840	-0.047	0.462	0.920	0.960	16.840	0.445	0.556	0.423	0.647
CAMSAP1	1106.103	-1.050	0.195	0.000	0.000	1106.103	-0.129	0.193	0.503	0.715	1106.103	0.920	0.225	0.000	0.001
ADSL	354.927	-0.881	0.211	0.000	0.000	354.927	-0.326	0.208	0.117	0.409	354.927	0.555	0.243	0.023	0.109
CYP4B1	566.244	-0.302	0.737	0.682	0.780	566.244	0.427	0.736	0.562	0.751	566.244	0.729	0.851	0.392	0.620
LINGO2	31.009	-0.723	0.580	0.213	0.342	31.009	0.339	0.555	0.542	0.738	31.009	1.061	0.661	0.109	0.294
MAMDC4	61.354	0.484	0.548	0.377	0.517	61.354	0.894	0.545	0.101	0.394	61.354	0.410	0.630	0.515	0.719
AK8	13.449	0.672	0.584	0.250	0.384	13.449	1.420	0.558	0.011	0.302	13.449	0.749	0.644	0.245	0.476
TTLL11	257.246	-0.936	0.337	0.006	0.021	257.246	-0.680	0.336	0.043	0.331	257.246	0.256	0.390	0.512	0.717
RASEF	21.257	0.446	0.344	0.195	0.320	21.257	-0.019	0.353	0.958	0.978	21.257	-0.465	0.404	0.250	0.482
HSD17B7P2	10.997	2.416	0.571	0.000	0.000	10.997	0.206	0.647	0.751	0.867	10.997	-2.210	0.682	0.001	0.015
TTC39B	30.254	0.890	0.305	0.004	0.015	30.254	0.751	0.304	0.014	0.306	30.254	-0.139	0.343	0.685	0.834
TRMT10B	136.542	-0.386	0.221	0.080	0.166	136.542	-0.033	0.216	0.877	0.937	136.542	0.353	0.255	0.166	0.381
LINC02603	11.575	1.258	0.569	0.027	0.073	11.575	0.213	0.603	0.724	0.852	11.575	-1.044	0.666	0.117	0.306
FAM120AOS	1098.109	-0.581	0.165	0.000	0.003	1098.109	-0.026	0.164	0.872	0.935	1098.109	0.555	0.190	0.004	0.032
CYP11A1	13.583	0.869	0.701	0.215	0.345	13.583	1.139	0.692	0.100	0.394	13.583	0.270	0.793	0.733	0.862
KIAA2026	215.184	0.271	0.169	0.109	0.209	215.184	0.174	0.168	0.302	0.568	215.184	-0.097	0.195	0.620	0.792
KIAA1958	263.281	-0.495	0.296	0.094	0.188	263.281	0.049	0.293	0.868	0.933	263.281	0.544	0.341	0.111	0.298
TSTD2	458.925	-1.017	0.330	0.002	0.010	458.925	-0.682	0.329	0.038	0.327	458.925	0.335	0.381	0.379	0.610
ZNF782	48.501	0.000	0.276	1.000	1.000	48.501	-0.096	0.274	0.728	0.855	48.501	-0.096	0.321	0.766	0.881
CSAG1	457.933	1.100	0.274	0.000	0.001	457.933	0.383	0.275	0.164	0.454	457.933	-0.718	0.316	0.023	0.111
FAAH2	89.060	0.397	0.315	0.208	0.336	89.060	1.022	0.310	0.001	0.294	89.060	0.625	0.359	0.082	0.247
ZXDB	274.037	-1.177	0.236	0.000	0.000	274.037	-0.205	0.230	0.374	0.622	274.037	0.973	0.272	0.000	0.006
MOSPD2	264.939	-0.198	0.195	0.310	0.449	264.939	-0.089	0.194	0.645	0.806	264.939	0.109	0.225	0.629	0.798
CYP19A1	11.875	0.282	0.557	0.612	0.724	11.875	0.768	0.535	0.152	0.444	11.875	0.486	0.626	0.438	0.659
NKAPP1	20.691	0.566	0.427	0.185	0.309	20.691	0.707	0.420	0.092	0.387	20.691	0.141	0.484	0.771	0.883
CYP21A2	56.566	1.146	0.593	0.053	0.121	56.566	0.510	0.597	0.393	0.637	56.566	-0.636	0.684	0.352	0.585
CH17-340M24.3	10.761	0.818	0.574	0.154	0.270	10.761	0.686	0.572	0.231	0.508	10.761	-0.132	0.648	0.838	0.922
ADSS2	1162.352	-0.702	0.169	0.000	0.000	1162.352	-0.208	0.168	0.216	0.494	1162.352	0.494	0.195	0.011	0.070
CXorf38	449.666	-0.334	0.178	0.060	0.133	449.666	0.053	0.176	0.765	0.876	449.666	0.386	0.205	0.059	0.203
FAM122B	375.144	0.012	0.168	0.942	0.963	375.144	-0.132	0.168	0.432	0.666	375.144	-0.145	0.195	0.459	0.676
FAM122C	92.873	-0.187	0.248	0.452	0.587	92.873	-0.129	0.246	0.601	0.779	92.873	0.058	0.287	0.840	0.923
USP54	621.771	-0.210	0.351	0.549	0.672	621.771	0.460	0.350	0.188	0.474	621.771	0.670	0.405	0.098	0.276
SLC35G1	243.355	-0.736	0.306	0.016	0.049	243.355	-0.573	0.305	0.060	0.350	243.355	0.163	0.354	0.645	0.808
CYP27B1	168.723	0.478	0.652	0.463	0.598	168.723	0.771	0.651	0.236	0.513	168.723	0.293	0.752	0.697	0.841
CYP51A1	2166.106	1.115	0.224	0.000	0.000	2166.106	-0.218	0.225	0.331	0.591	2166.106	-1.334	0.259	0.000	0.000
CFAP58	19.738	1.725	0.428	0.000	0.001	19.738	0.187	0.459	0.683	0.829	19.738	-1.537	0.503	0.002	0.023
AARS1	8939.589	-1.139	0.238	0.000	0.000	8939.589	-0.190	0.238	0.424	0.660	8939.589	0.949	0.275	0.001	0.009
AP2A1	3873.862	0.261	0.200	0.192	0.317	3873.862	0.338	0.200	0.091	0.386	3873.862	0.077	0.231	0.740	0.867
DAB2	263.022	1.182	0.283	0.000	0.000	263.022	-0.790	0.290	0.006	0.297	263.022	-1.971	0.332	0.000	0.000
C11orf65	14.571	-0.246	0.534	0.645	0.751	14.571	0.301	0.512	0.556	0.747	14.571	0.548	0.609	0.368	0.601
DACH1	60.387	-0.307	0.460	0.505	0.635	60.387	0.356	0.454	0.433	0.667	60.387	0.663	0.529	0.210	0.435
DAD1	1867.194	0.143	0.170	0.402	0.540	1867.194	-0.044	0.170	0.797	0.895	1867.194	-0.186	0.196	0.343	0.577
TMTC2	256.933	0.128	0.281	0.650	0.755	256.933	0.316	0.280	0.259	0.531	256.933	0.189	0.324	0.561	0.752

CD55	6449.150	-0.539	0.184	0.003	0.015	6449.150	-0.257	0.184	0.161	0.452	6449.150	0.282	0.212	0.185	0.404
TMTC3	403.878	-0.113	0.161	0.486	0.619	403.878	-0.299	0.162	0.064	0.351	403.878	-0.187	0.187	0.319	0.554
ALDH1L2	12.157	-0.171	0.586	0.770	0.847	12.157	-0.127	0.578	0.827	0.910	12.157	0.045	0.680	0.948	0.976
DAG1	1707.339	0.674	0.188	0.000	0.002	1707.339	0.124	0.188	0.509	0.718	1707.339	-0.550	0.217	0.011	0.070
DENND5B	162.815	0.051	0.206	0.804	0.871	162.815	-0.077	0.205	0.708	0.843	162.815	-0.128	0.238	0.592	0.772
DGKA	553.862	0.286	0.269	0.288	0.426	553.862	0.379	0.269	0.159	0.451	553.862	0.093	0.311	0.764	0.880
PPTC7	1051.777	-1.259	0.259	0.000	0.000	1051.777	-0.284	0.257	0.269	0.540	1051.777	0.974	0.298	0.001	0.014
DGKG	74.871	-0.233	0.285	0.413	0.551	74.871	0.362	0.273	0.185	0.472	74.871	0.595	0.324	0.066	0.218
DGKH	380.884	-0.568	0.279	0.042	0.101	380.884	-0.409	0.278	0.142	0.433	380.884	0.159	0.323	0.622	0.793
CCDC122	70.469	-0.158	0.278	0.568	0.688	70.469	0.152	0.272	0.577	0.762	70.469	0.310	0.319	0.331	0.566
GPR180	228.021	-0.332	0.260	0.202	0.329	228.021	-0.246	0.259	0.343	0.600	228.021	0.086	0.301	0.774	0.886
DGKQ	217.475	0.210	0.239	0.380	0.520	217.475	-0.258	0.241	0.284	0.553	217.475	-0.468	0.278	0.092	0.267
AP2A2	3436.295	-0.335	0.134	0.012	0.039	3436.295	-0.116	0.133	0.383	0.629	3436.295	0.219	0.154	0.156	0.368
DAP	2385.322	-0.112	0.141	0.427	0.563	2385.322	0.019	0.140	0.890	0.945	2385.322	0.131	0.162	0.419	0.643
TMEM229B	151.572	-0.226	0.846	0.789	0.861	151.572	0.568	0.844	0.501	0.714	151.572	0.794	0.976	0.416	0.641
SYNE3	136.205	-0.100	0.223	0.655	0.758	136.205	0.266	0.219	0.223	0.502	136.205	0.366	0.256	0.153	0.362
DAPK1	11.405	0.107	0.465	0.818	0.880	11.405	0.124	0.456	0.786	0.888	11.405	0.017	0.536	0.975	0.987
REM2	7.987	1.335	0.714	0.062	0.135	7.987	-0.060	0.767	0.937	0.968	7.987	-1.395	0.848	0.100	0.281
TMEM30B	12.942	-0.007	0.447	0.988	0.993	12.942	-0.135	0.444	0.761	0.873	12.942	-0.128	0.524	0.807	0.903
DAPK3	1843.744	0.594	0.192	0.002	0.010	1843.744	-0.140	0.193	0.467	0.690	1843.744	-0.735	0.222	0.001	0.013
MDGA2	324.764	-1.076	0.238	0.000	0.000	324.764	-0.473	0.235	0.044	0.331	324.764	0.603	0.274	0.028	0.126
SAMD15	7.391	-0.457	0.609	0.453	0.588	7.391	-1.288	0.655	0.049	0.337	7.391	-0.831	0.763	0.276	0.511
NOP9	540.424	-0.625	0.282	0.027	0.071	540.424	-0.596	0.281	0.034	0.322	540.424	0.029	0.326	0.928	0.967
EML5	235.659	-0.001	0.192	0.997	0.999	235.659	-0.197	0.192	0.305	0.570	235.659	-0.196	0.223	0.378	0.609
DARS1	1628.200	-0.101	0.191	0.598	0.711	1628.200	0.198	0.190	0.299	0.566	1628.200	0.298	0.220	0.175	0.393
DNAAF4	8.869	1.068	0.829	0.198	0.324	8.869	0.999	0.826	0.227	0.505	8.869	-0.069	0.939	0.941	0.973
DAXX	11227.039	-0.072	0.160	0.651	0.756	11227.039	-0.189	0.160	0.237	0.514	11227.039	-0.117	0.185	0.526	0.727
SPRED1	840.237	-0.550	0.167	0.001	0.006	840.237	-0.129	0.166	0.437	0.670	840.237	0.421	0.193	0.029	0.130
PGBD4	33.373	0.070	0.288	0.807	0.873	33.373	-0.312	0.293	0.286	0.555	33.373	-0.383	0.341	0.261	0.495
ADAL	177.134	-0.183	0.158	0.247	0.380	177.134	-0.045	0.155	0.771	0.878	177.134	0.138	0.183	0.450	0.669
FSIP1	111.330	-1.193	0.323	0.000	0.002	111.330	-0.255	0.312	0.415	0.653	111.330	0.938	0.371	0.011	0.071
ZFPM1	217.639	0.312	0.297	0.294	0.432	217.639	-0.275	0.299	0.358	0.611	217.639	-0.586	0.344	0.089	0.261
AP1B1	1708.626	-0.203	0.280	0.468	0.602	1708.626	-0.679	0.280	0.015	0.306	1708.626	-0.476	0.323	0.141	0.345
ITPRIPL2	2454.700	-0.200	0.138	0.147	0.260	2454.700	0.024	0.138	0.862	0.930	2454.700	0.224	0.159	0.160	0.372
DBI	1540.202	1.237	0.208	0.000	0.000	1540.202	0.155	0.208	0.457	0.683	1540.202	-1.082	0.240	0.000	0.000
ZFP1	249.464	-0.580	0.249	0.020	0.057	249.464	0.157	0.245	0.521	0.725	249.464	0.737	0.287	0.010	0.066
ANKFN1	13.563	-2.516	0.650	0.000	0.001	13.563	-0.854	0.538	0.113	0.405	13.563	1.662	0.733	0.023	0.112
SLFN5	2392.871	0.042	0.192	0.826	0.886	2392.871	-0.298	0.192	0.122	0.413	2392.871	-0.340	0.222	0.126	0.321
NAGS	76.157	-0.860	0.338	0.011	0.037	76.157	-0.101	0.326	0.756	0.870	76.157	0.758	0.389	0.051	0.185
RETREG3	811.214	0.149	0.113	0.187	0.312	811.214	-0.036	0.113	0.753	0.868	811.214	-0.185	0.131	0.158	0.371
TMEM92	198.549	0.937	0.274	0.001	0.004	198.549	0.617	0.275	0.025	0.316	198.549	-0.320	0.316	0.310	0.546
PHOSPHO1	7.303	0.345	0.577	0.549	0.672	7.303	-3.440	0.993	0.001	0.253	7.303	-3.785	1.048	0.000	0.005
ZNF519	70.771	-0.276	0.349	0.429	0.565	70.771	0.015	0.344	0.965	0.981	70.771	0.291	0.402	0.469	0.684
C18orf54	169.046	-1.166	0.345	0.001	0.005	169.046	-0.445	0.340	0.191	0.477	169.046	0.721	0.398	0.070	0.226
DBN1	2982.588	-0.313	0.243	0.198	0.324	2982.588	-0.388	0.243	0.111	0.404	2982.588	-0.075	0.281	0.791	0.896
DBP	368.339	0.364	0.741	0.624	0.734	368.339	0.804	0.740	0.277	0.548	368.339	0.441	0.855	0.606	0.783
DBT	755.623	-0.447	0.216	0.039	0.095	755.623	0.164	0.215	0.446	0.677	755.623	0.611	0.249	0.014	0.083
ZNF836	66.030	0.306	0.538	0.570	0.689	66.030	0.393	0.536	0.463	0.689	66.030	0.088	0.620	0.887	0.946
ZNF600	81.790	0.283	0.225	0.209	0.337	81.790	0.215	0.224	0.336	0.595	81.790	-0.068	0.259	0.793	0.896
ZNF497	76.338	0.327	0.193	0.089	0.180	76.338	0.264	0.190	0.166	0.456	76.338	-0.064	0.221	0.774	0.885
ZNF550	164.007	-0.360	0.276	0.192	0.317	164.007	0.227	0.271	0.402	0.645	164.007	0.587	0.317	0.064	0.213
ZNF296	125.378	0.701	0.316	0.027	0.072	125.378	0.779	0.314	0.013	0.305	125.378	0.079	0.362	0.828	0.915
DEDD2	694.114	0.194	0.215	0.366	0.507	694.114	0.221	0.215	0.304	0.570	694.114	0.026	0.248	0.916	0.961
ZNF846	13.618	0.465	0.513	0.364	0.505	13.618	0.395	0.509	0.438	0.671	13.618	-0.070	0.587	0.905	0.956
AP2B1	4984.081	-0.439	0.225	0.051	0.118	4984.081	0.079	0.225	0.724	0.853	4984.081	0.519	0.260	0.046	0.174
ZNF579	721.790	0.726	0.276	0.009	0.030	721.790	0.509	0.276	0.065	0.354	721.790	-0.217	0.319	0.497	0.704
ZNF791	300.428	0.326	0.192	0.089	0.179	300.428	-0.088	0.193	0.650	0.809	300.428	-0.414	0.222	0.063	0.211
ZNF564	65.791	-0.327	0.248	0.188	0.312	65.791	-0.031	0.240	0.897	0.948	65.791	0.296	0.286	0.301	0.536
ZNF114	7.728	0.186	0.581	0.749	0.832	7.728	0.188	0.573	0.742	0.862	7.728	0.002	0.669	0.998	0.998
ZNF567	84.635	-0.468	0.238	0.049	0.114	84.635	-0.185	0.231	0.423	0.659	84.635	0.282	0.275	0.304	0.539
ZNF383	143.763	0.024	0.339	0.944	0.965	143.763	0.454	0.336	0.176	0.465	143.763	0.430	0.389	0.269	0.504
EID2	339.944	0.460	0.333	0.167	0.287	339.944	-0.570	0.335	0.089	0.382	339.944	-1.030	0.386	0.008	0.054
ZNF780B	82.617	-0.228	0.222	0.306	0.445	82.617	-0.426	0.222	0.056	0.343	82.617	-0.198	0.261	0.448	0.668
PRR22	14.602	0.470	0.524	0.370	0.511	14.602	-0.624	0.556	0.262	0.534	14.602	-1.093	0.631	0.083	0.250
ECI1	1684.066	0.566	0.253	0.026	0.069	1684.066	0.172	0.254	0.499	0.712	1684.066	-0.395	0.293	0.178	0.395
ZNF100	58.487	-0.366	0.433	0.398	0.537	58.487	-0.364	0.431	0.399	0.642	58.487	0.002	0.502	0.997	0.998
DENND2C	51.092	0.523	0.346	0.130	0.238	51.092	0.383	0.345	0.267	0.538	51.092	-0.140	0.398	0.726	0.858

DCK	357.091	-0.528	0.207	0.011	0.036	357.091	-0.209	0.205	0.307	0.572	357.091	0.319	0.239	0.182	0.401
DCN	1340.867	-1.298	0.532	0.015	0.045	1340.867	0.799	0.522	0.126	0.419	1340.867	2.097	0.610	0.001	0.009
DENND1B	165.696	-0.673	0.237	0.004	0.018	165.696	-0.398	0.233	0.089	0.382	165.696	0.276	0.274	0.314	0.550
DCTD	1317.180	-0.122	0.124	0.325	0.465	1317.180	-0.215	0.124	0.083	0.375	1317.180	-0.093	0.144	0.519	0.722
TOR1AIP2	901.962	0.209	0.132	0.113	0.214	901.962	-0.101	0.132	0.445	0.675	901.962	-0.310	0.153	0.042	0.165
IFNLR1	77.306	0.476	0.322	0.139	0.249	77.306	0.463	0.320	0.148	0.441	77.306	-0.014	0.369	0.970	0.986
CITED4	22.945	0.860	0.493	0.081	0.167	22.945	0.118	0.506	0.816	0.904	22.945	-0.742	0.574	0.196	0.419
SASS6	209.384	-0.680	0.209	0.001	0.006	209.384	-0.124	0.205	0.546	0.740	209.384	0.556	0.241	0.021	0.104
SDE2	407.213	-0.639	0.188	0.001	0.004	407.213	-0.334	0.186	0.073	0.362	407.213	0.305	0.218	0.161	0.375
CNST	288.517	-1.329	0.249	0.000	0.000	288.517	-0.422	0.243	0.083	0.374	288.517	0.907	0.287	0.002	0.018
DCTN1	2322.945	-0.347	0.179	0.052	0.120	2322.945	0.061	0.179	0.731	0.856	2322.945	0.409	0.207	0.048	0.177
AP1G1	1013.835	-0.283	0.142	0.046	0.107	1013.835	-0.190	0.141	0.178	0.466	1013.835	0.093	0.164	0.570	0.759
PAQR7	398.629	0.262	0.497	0.598	0.711	398.629	0.589	0.496	0.235	0.513	398.629	0.327	0.573	0.568	0.758
DDB1	6820.233	-0.376	0.148	0.011	0.036	6820.233	-0.073	0.147	0.619	0.790	6820.233	0.303	0.170	0.075	0.236
APCDD1L	504.921	0.556	0.178	0.002	0.009	504.921	-0.094	0.180	0.602	0.779	504.921	-0.650	0.207	0.002	0.019
DDB2	158.939	-0.176	0.259	0.496	0.627	158.939	0.363	0.255	0.155	0.447	158.939	0.539	0.297	0.070	0.225
AKR1C1	3234.675	1.133	0.344	0.001	0.006	3234.675	-0.862	0.346	0.013	0.305	3234.675	-1.995	0.399	0.000	0.000
AKR1C2	13227.304	0.903	0.252	0.000	0.002	13227.304	-0.223	0.252	0.376	0.624	13227.304	-1.126	0.291	0.000	0.003
CABP7	117.302	0.940	0.310	0.002	0.011	117.302	0.715	0.310	0.021	0.313	117.302	-0.224	0.355	0.527	0.728
GADD45A	6325.566	-0.840	0.344	0.015	0.045	6325.566	-0.819	0.344	0.017	0.306	6325.566	0.022	0.397	0.957	0.980
DDIT3	766.121	-0.293	0.299	0.328	0.468	766.121	-0.261	0.299	0.382	0.628	766.121	0.031	0.346	0.928	0.967
DDOST	6002.733	0.091	0.095	0.337	0.477	6002.733	0.074	0.095	0.439	0.671	6002.733	-0.018	0.110	0.872	0.939
CCDC138	141.527	-0.676	0.324	0.037	0.092	141.527	-0.635	0.323	0.049	0.337	141.527	0.041	0.376	0.913	0.960
ADGRF3	37.487	-1.128	0.501	0.024	0.067	37.487	-0.844	0.493	0.087	0.380	37.487	0.284	0.583	0.626	0.796
DDT	2173.307	0.730	0.248	0.003	0.014	2173.307	0.271	0.248	0.276	0.546	2173.307	-0.460	0.287	0.109	0.295
FAM171B	128.378	-0.125	0.259	0.629	0.739	128.378	-0.064	0.257	0.802	0.896	128.378	0.060	0.299	0.840	0.923
DDX1	3089.787	-0.699	0.193	0.000	0.002	3089.787	-0.460	0.193	0.017	0.306	3089.787	0.238	0.223	0.285	0.521
UBXN2A	364.768	-0.355	0.149	0.017	0.051	364.768	-0.298	0.148	0.044	0.332	364.768	0.057	0.173	0.741	0.867
DDX3X	5378.527	-0.697	0.236	0.003	0.014	5378.527	-0.488	0.236	0.039	0.327	5378.527	0.208	0.273	0.445	0.665
DDX5	2908.295	0.571	0.179	0.001	0.007	2908.295	-0.375	0.179	0.036	0.324	2908.295	-0.946	0.206	0.000	0.000
DDX6	1460.536	0.185	0.092	0.044	0.105	1460.536	-0.035	0.092	0.707	0.842	1460.536	-0.220	0.107	0.039	0.156
SPTSSB	18.899	0.280	0.518	0.589	0.705	18.899	0.410	0.510	0.422	0.658	18.899	0.130	0.593	0.826	0.914
DMXL1	478.673	-0.510	0.332	0.124	0.230	478.673	0.295	0.330	0.371	0.620	478.673	0.805	0.382	0.035	0.146
DHX8	823.810	-0.651	0.208	0.002	0.009	823.810	-0.395	0.207	0.056	0.344	823.810	0.256	0.240	0.286	0.521
RNF168	647.760	-0.432	0.254	0.089	0.179	647.760	-0.608	0.254	0.017	0.306	647.760	-0.176	0.294	0.550	0.745
TLE5	6471.637	0.701	0.196	0.000	0.003	6471.637	0.243	0.196	0.215	0.494	6471.637	-0.458	0.226	0.043	0.167
DHX9	2395.215	-0.518	0.253	0.041	0.099	2395.215	-0.748	0.253	0.003	0.297	2395.215	-0.230	0.293	0.432	0.654
DDX10	419.197	-0.641	0.267	0.016	0.049	419.197	-0.675	0.267	0.011	0.305	419.197	-0.033	0.309	0.914	0.960
DDX11	402.016	-0.043	0.299	0.885	0.925	402.016	-0.435	0.299	0.146	0.438	402.016	-0.391	0.346	0.258	0.492
PRICKLE2	172.661	0.179	0.429	0.676	0.775	172.661	0.492	0.428	0.250	0.524	172.661	0.313	0.494	0.527	0.727
SPATA5	119.636	-0.406	0.359	0.258	0.393	119.636	-0.789	0.360	0.028	0.321	119.636	-0.383	0.417	0.359	0.592
BBS12	91.459	0.560	0.283	0.048	0.111	91.459	0.500	0.282	0.076	0.365	91.459	-0.060	0.325	0.854	0.930
DHX15	2023.548	-0.945	0.238	0.000	0.001	2023.548	-0.372	0.238	0.118	0.410	2023.548	0.573	0.275	0.037	0.151
DECR1	963.326	0.085	0.268	0.750	0.833	963.326	0.338	0.268	0.207	0.489	963.326	0.253	0.310	0.414	0.640
DCLK2	72.788	0.596	0.271	0.028	0.074	72.788	0.138	0.275	0.615	0.788	72.788	-0.458	0.314	0.145	0.351
ADGRA3	803.067	-0.242	0.123	0.049	0.114	803.067	0.096	0.122	0.429	0.664	803.067	0.338	0.142	0.017	0.092
MMAA	99.658	-0.384	0.287	0.181	0.305	99.658	-0.726	0.289	0.012	0.305	99.658	-0.342	0.337	0.310	0.545
ZBTB49	38.860	-0.366	0.364	0.315	0.454	38.860	-0.508	0.363	0.162	0.453	38.860	-0.142	0.428	0.740	0.867
TIGD2	162.366	-0.319	0.144	0.027	0.073	162.366	0.103	0.138	0.455	0.682	162.366	0.422	0.165	0.011	0.069
RASSF6	8.696	0.593	0.551	0.282	0.420	8.696	-0.364	0.585	0.534	0.733	8.696	-0.957	0.661	0.148	0.355
SGMS2	904.106	-0.514	0.258	0.046	0.109	904.106	-0.046	0.257	0.859	0.928	904.106	0.468	0.298	0.116	0.305
MIER3	388.243	-0.395	0.205	0.055	0.124	388.243	-0.308	0.205	0.132	0.424	388.243	0.087	0.238	0.715	0.852
TENT2	701.590	-0.629	0.180	0.000	0.003	701.590	0.041	0.178	0.818	0.905	701.590	0.670	0.207	0.001	0.016
DEFB1	39.928	0.296	0.481	0.539	0.663	39.928	0.983	0.471	0.037	0.325	39.928	0.687	0.547	0.210	0.435
DCP2	454.990	-0.313	0.145	0.031	0.081	454.990	0.084	0.143	0.556	0.747	454.990	0.398	0.167	0.018	0.094
NIM1K	93.339	0.605	0.553	0.274	0.410	93.339	0.962	0.548	0.079	0.370	93.339	0.356	0.632	0.573	0.760
CFD	428.905	0.930	0.557	0.095	0.188	428.905	1.091	0.557	0.050	0.337	428.905	0.160	0.643	0.803	0.901
FAM151B	13.756	-0.713	0.469	0.128	0.235	13.756	-0.332	0.440	0.451	0.680	13.756	0.382	0.544	0.483	0.695
DFFA	1451.170	-0.018	0.197	0.926	0.953	1451.170	-0.234	0.197	0.235	0.512	1451.170	-0.216	0.228	0.343	0.577
LCA5	179.156	-0.401	0.285	0.159	0.277	179.156	0.197	0.281	0.484	0.701	179.156	0.598	0.328	0.068	0.222
DFFB	70.825	-0.078	0.196	0.691	0.787	70.825	-0.059	0.192	0.760	0.872	70.825	0.019	0.227	0.933	0.970
TIMM8A	211.526	-0.727	0.335	0.030	0.079	211.526	-0.596	0.334	0.075	0.364	211.526	0.132	0.388	0.734	0.863
ZNF92	67.927	-0.018	0.232	0.940	0.962	67.927	-0.305	0.234	0.192	0.477	67.927	-0.287	0.273	0.292	0.527
THAP5	401.318	-0.569	0.165	0.001	0.004	401.318	-0.314	0.163	0.054	0.342	401.318	0.256	0.191	0.180	0.398
CCDC71L	458.582	0.421	0.286	0.141	0.252	458.582	-0.485	0.287	0.091	0.385	458.582	-0.906	0.331	0.006	0.048
ZNF467	34.202	1.096	0.737	0.137	0.247	34.202	1.002	0.736	0.173	0.463	34.202	-0.094	0.844	0.911	0.959
BHLHA15	26.623	-4.541	1.288	0.000	0.003	26.623	0.750	0.561	0.181	0.469	26.623	5.292	1.323	0.000	0.002

GSDME	1121.850	-0.321	0.131	0.015	0.045	1121.850	-0.061	0.131	0.639	0.803	1121.850	0.260	0.152	0.087	0.257
ZNF800	320.048	-0.527	0.212	0.013	0.041	320.048	-0.291	0.210	0.165	0.455	320.048	0.236	0.245	0.336	0.570
COCH	27.295	0.039	0.296	0.896	0.932	27.295	-0.202	0.298	0.497	0.711	27.295	-0.241	0.349	0.489	0.700
TMEM64	359.885	-0.302	0.221	0.171	0.291	359.885	0.198	0.218	0.365	0.617	359.885	0.500	0.254	0.049	0.181
ZNF596	23.433	-0.036	0.361	0.921	0.950	23.433	-0.203	0.361	0.574	0.759	23.433	-0.167	0.423	0.692	0.839
STKLD1	14.672	0.678	0.621	0.275	0.412	14.672	0.067	0.634	0.916	0.958	14.672	-0.612	0.723	0.398	0.625
OLFML2A	701.095	0.690	0.821	0.401	0.539	701.095	0.730	0.821	0.374	0.622	701.095	0.041	0.948	0.966	0.984
QSOX2	1246.333	-0.577	0.232	0.013	0.041	1246.333	-0.263	0.231	0.255	0.529	1246.333	0.313	0.268	0.242	0.473
ZNF169	47.015	-0.189	0.302	0.532	0.658	47.015	0.277	0.291	0.342	0.599	47.015	0.465	0.345	0.177	0.394
SPIN3	234.738	-0.526	0.221	0.017	0.052	234.738	0.274	0.216	0.205	0.488	234.738	0.800	0.254	0.002	0.019
TCEANC	35.356	-0.586	0.370	0.113	0.214	35.356	-0.423	0.362	0.242	0.518	35.356	0.162	0.431	0.706	0.847
FUT11	724.478	1.004	0.541	0.063	0.138	724.478	0.800	0.541	0.139	0.430	724.478	-0.204	0.624	0.744	0.868
PWWP2B	572.342	-0.051	0.200	0.801	0.869	572.342	-0.287	0.200	0.153	0.445	572.342	-0.236	0.232	0.309	0.544
SSBP4	1149.744	0.346	0.222	0.120	0.224	1149.744	0.027	0.223	0.903	0.951	1149.744	-0.319	0.257	0.215	0.441
ACTL10	22.902	-0.332	0.459	0.470	0.604	22.902	-0.318	0.454	0.483	0.701	22.902	0.014	0.536	0.979	0.989
DHX36	1371.841	-0.399	0.146	0.006	0.023	1371.841	-0.375	0.146	0.010	0.298	1371.841	0.024	0.169	0.885	0.945
COMMD6	494.184	0.484	0.294	0.100	0.195	494.184	0.435	0.293	0.139	0.430	494.184	-0.049	0.339	0.885	0.945
PSORS1C1	91.016	0.800	0.426	0.060	0.133	91.016	0.596	0.426	0.162	0.453	91.016	-0.204	0.491	0.677	0.829
NUDT10	24.408	-1.278	0.373	0.001	0.004	24.408	-0.200	0.325	0.537	0.735	24.408	1.078	0.422	0.011	0.069
ADAMTS15	7.912	1.847	0.975	0.058	0.129	7.912	0.246	1.008	0.807	0.899	7.912	-1.602	1.134	0.158	0.370
ADAMTS16	21.999	-0.910	0.382	0.017	0.051	21.999	-0.314	0.353	0.375	0.622	21.999	0.597	0.439	0.174	0.391
PPP1R18	15167.414	0.622	0.151	0.000	0.000	15167.414	0.235	0.151	0.120	0.412	15167.414	-0.387	0.174	0.026	0.121
ZNF525	78.929	-0.339	0.281	0.227	0.358	78.929	0.015	0.274	0.957	0.978	78.929	0.354	0.323	0.273	0.508
ZNF431	72.499	0.233	0.266	0.381	0.521	72.499	-0.025	0.267	0.926	0.962	72.499	-0.258	0.309	0.403	0.630
ZNF721	180.121	-0.275	0.163	0.092	0.184	180.121	0.016	0.159	0.918	0.959	180.121	0.292	0.188	0.121	0.313
ANKRD24	21.819	1.561	0.809	0.054	0.122	21.819	1.038	0.813	0.202	0.485	21.819	-0.523	0.927	0.573	0.760
ZNF384	726.688	-0.405	0.159	0.011	0.036	726.688	0.023	0.157	0.882	0.941	726.688	0.428	0.183	0.019	0.100
ASXL1	1276.021	-0.324	0.184	0.079	0.163	1276.021	0.120	0.184	0.515	0.721	1276.021	0.444	0.213	0.037	0.151
SYNPO2	14.076	0.561	0.593	0.344	0.484	14.076	0.032	0.602	0.957	0.978	14.076	-0.529	0.690	0.443	0.663
RHOV	13.827	1.053	0.536	0.050	0.115	13.827	1.031	0.531	0.052	0.341	13.827	-0.022	0.598	0.970	0.985
DSTNP2	26.452	0.619	0.364	0.089	0.179	26.452	0.381	0.365	0.296	0.564	26.452	-0.238	0.417	0.568	0.758
LOC171391	52.049	1.442	0.302	0.000	0.000	52.049	0.273	0.316	0.387	0.633	52.049	-1.168	0.352	0.001	0.012
ZNF675	30.888	0.324	0.291	0.265	0.401	30.888	0.065	0.293	0.826	0.909	30.888	-0.259	0.338	0.443	0.663
PDIA3P1	230.785	0.453	0.227	0.046	0.108	230.785	0.055	0.228	0.808	0.899	230.785	-0.398	0.263	0.130	0.327
CLYBL	90.874	0.452	0.491	0.358	0.498	90.874	0.518	0.490	0.290	0.558	90.874	0.067	0.566	0.906	0.957
SPTSSA	1560.992	0.176	0.181	0.332	0.472	1560.992	-0.074	0.181	0.681	0.827	1560.992	-0.250	0.209	0.232	0.461
POLR3H	1660.462	-0.130	0.241	0.588	0.704	1660.462	-0.400	0.241	0.097	0.391	1660.462	-0.270	0.278	0.332	0.567
ABHD3	87.612	-0.019	0.393	0.962	0.977	87.612	-1.144	0.403	0.005	0.297	87.612	-1.125	0.464	0.015	0.086
DGUOK	781.111	0.570	0.203	0.005	0.019	781.111	-0.086	0.203	0.671	0.822	781.111	-0.656	0.234	0.005	0.042
DHCR7	3484.872	1.851	0.160	0.000	0.000	3484.872	-0.360	0.161	0.025	0.316	3484.872	-2.211	0.185	0.000	0.000
DHCR24	7974.129	1.227	0.160	0.000	0.000	7974.129	-0.114	0.160	0.477	0.697	7974.129	-1.341	0.185	0.000	0.000
DHFR	1328.303	-0.303	0.263	0.249	0.383	1328.303	-0.741	0.263	0.005	0.297	1328.303	-0.438	0.304	0.150	0.359
AFG3L1P	94.459	0.519	0.350	0.138	0.249	94.459	-0.428	0.356	0.229	0.507	94.459	-0.946	0.408	0.020	0.103
DHODH	247.470	-0.241	0.290	0.406	0.544	247.470	-0.388	0.290	0.180	0.469	247.470	-0.147	0.336	0.661	0.819
DHPS	1382.647	0.193	0.167	0.247	0.380	1382.647	0.264	0.167	0.113	0.405	1382.647	0.071	0.193	0.714	0.851
CYB5R3	4264.723	0.589	0.121	0.000	0.000	4264.723	0.053	0.121	0.660	0.814	4264.723	-0.536	0.140	0.000	0.003
NQO1	4841.398	0.534	0.224	0.017	0.051	4841.398	-0.378	0.224	0.092	0.386	4841.398	-0.912	0.258	0.000	0.007
DIAPH1	3457.767	-0.611	0.223	0.006	0.023	3457.767	-0.380	0.222	0.088	0.380	3457.767	0.231	0.257	0.369	0.601
DIAPH2	332.320	-0.287	0.229	0.211	0.341	332.320	0.313	0.227	0.167	0.456	332.320	0.600	0.264	0.023	0.111
SEPTIN1	33.500	0.823	0.529	0.120	0.223	33.500	0.934	0.525	0.076	0.364	33.500	0.111	0.603	0.854	0.930
DIO2	158.132	-1.287	0.449	0.004	0.017	158.132	-1.338	0.448	0.003	0.297	158.132	-0.051	0.521	0.923	0.964
DKC1	2816.069	-1.029	0.291	0.000	0.003	2816.069	-0.632	0.291	0.030	0.322	2816.069	0.398	0.336	0.237	0.467
DLAT	671.956	-0.738	0.238	0.002	0.010	671.956	-0.579	0.237	0.015	0.306	671.956	0.159	0.275	0.564	0.754
DLD	1529.025	-0.390	0.133	0.003	0.015	1529.025	-0.073	0.132	0.580	0.764	1529.025	0.317	0.154	0.039	0.157
DLG1	2441.844	-0.870	0.204	0.000	0.000	2441.844	-0.053	0.203	0.795	0.893	2441.844	0.818	0.235	0.001	0.008
DLG2	8.732	-0.171	0.580	0.769	0.847	8.732	0.534	0.543	0.325	0.586	8.732	0.705	0.653	0.280	0.515
DLG3	78.212	0.375	0.186	0.044	0.105	78.212	0.166	0.186	0.373	0.622	78.212	-0.209	0.215	0.331	0.566
DLG4	423.325	-0.259	0.394	0.511	0.640	423.325	0.558	0.392	0.155	0.447	423.325	0.816	0.454	0.072	0.229
DLST	1368.372	-0.590	0.159	0.000	0.002	1368.372	-0.231	0.158	0.144	0.435	1368.372	0.359	0.183	0.051	0.184
DLX1	134.255	-0.381	0.323	0.238	0.371	134.255	-0.671	0.323	0.038	0.327	134.255	-0.291	0.375	0.439	0.659
DLX2	29.605	-0.835	0.399	0.037	0.091	29.605	-0.474	0.382	0.215	0.494	29.605	0.360	0.464	0.438	0.659
DLX4	19.926	-0.094	0.746	0.900	0.936	19.926	0.718	0.729	0.325	0.586	19.926	0.812	0.852	0.341	0.575
AGA	133.815	0.173	0.213	0.416	0.554	133.815	0.131	0.211	0.535	0.734	133.815	-0.042	0.245	0.865	0.935
DMBT1	11.396	-6.409	1.335	0.000	0.000	11.396	-0.556	0.672	0.408	0.649	11.396	5.853	1.396	0.000	0.001
DMD	117.563	-0.201	0.249	0.420	0.557	117.563	-0.148	0.247	0.549	0.743	117.563	0.053	0.288	0.855	0.930
DNM1	185.514	0.548	0.271	0.043	0.103	185.514	0.366	0.271	0.177	0.466	185.514	-0.183	0.312	0.558	0.750
DMPK	592.961	1.194	0.286	0.000	0.000	592.961	0.477	0.287	0.096	0.391	592.961	-0.717	0.330	0.030	0.131

DMWD	763.658	-0.409	0.166	0.013	0.043	763.658	-0.131	0.165	0.427	0.663	763.658	0.278	0.191	0.145	0.352
DNA2	108.839	-0.869	0.308	0.005	0.019	108.839	-0.387	0.303	0.201	0.484	108.839	0.482	0.356	0.176	0.394
DNAH5	57.100	0.390	0.261	0.135	0.244	57.100	0.605	0.255	0.018	0.306	57.100	0.214	0.296	0.469	0.684
DNAH6	18.532	0.614	0.548	0.263	0.398	18.532	0.865	0.539	0.108	0.401	18.532	0.251	0.621	0.686	0.834
AGER	174.819	1.459	0.406	0.000	0.002	174.819	0.966	0.407	0.018	0.306	174.819	-0.493	0.468	0.292	0.527
DNASE1	2591.378	-0.120	0.202	0.552	0.674	2591.378	-0.008	0.202	0.967	0.982	2591.378	0.112	0.233	0.632	0.799
DNASE1L1	734.439	0.616	0.172	0.000	0.003	734.439	0.327	0.173	0.058	0.348	734.439	-0.289	0.199	0.147	0.354
DNASE1L2	25.462	1.560	0.466	0.001	0.005	25.462	0.486	0.483	0.314	0.577	25.462	-1.074	0.538	0.046	0.173
DNASE2	920.005	0.310	0.160	0.052	0.119	920.005	0.347	0.159	0.029	0.322	920.005	0.037	0.184	0.843	0.924
DYNC1H1	7109.364	-0.262	0.142	0.064	0.139	7109.364	-0.142	0.141	0.315	0.577	7109.364	0.120	0.163	0.462	0.679
AGL	546.500	-0.396	0.288	0.169	0.289	546.500	0.054	0.287	0.850	0.923	546.500	0.451	0.333	0.176	0.393
DYNC1I2	1391.636	0.207	0.111	0.063	0.138	1391.636	-0.062	0.112	0.580	0.764	1391.636	-0.269	0.129	0.037	0.151
DYNC1LI2	2148.719	-0.479	0.190	0.012	0.038	2148.719	-0.083	0.190	0.660	0.815	2148.719	0.396	0.220	0.072	0.229
DNM2	3755.531	0.273	0.080	0.001	0.004	3755.531	-0.024	0.080	0.761	0.873	3755.531	-0.297	0.093	0.001	0.016
DNMT1	2314.475	-0.673	0.365	0.065	0.141	2314.475	-0.891	0.365	0.015	0.306	2314.475	-0.217	0.422	0.606	0.783
TRDMT1	157.526	-1.172	0.320	0.000	0.002	157.526	-0.472	0.314	0.133	0.424	157.526	0.700	0.369	0.058	0.200
DNMT3A	158.815	0.408	0.178	0.022	0.061	158.815	0.000	0.180	0.999	0.999	158.815	-0.408	0.207	0.048	0.179
DNMT3B	89.243	0.656	0.212	0.002	0.010	89.243	-0.479	0.223	0.032	0.322	89.243	-1.135	0.253	0.000	0.000
DOCK1	940.974	-0.747	0.208	0.000	0.002	940.974	0.008	0.207	0.970	0.984	940.974	0.755	0.240	0.002	0.019
DOCK3	74.820	0.392	0.406	0.334	0.474	74.820	0.332	0.405	0.412	0.651	74.820	-0.060	0.468	0.898	0.952
DOK1	127.671	0.484	0.290	0.095	0.189	127.671	0.519	0.289	0.073	0.362	127.671	0.035	0.334	0.917	0.961
DXO	1120.955	0.327	0.220	0.138	0.248	1120.955	-0.006	0.220	0.977	0.987	1120.955	-0.333	0.254	0.190	0.412
DPAGT1	1000.671	-0.028	0.221	0.899	0.935	1000.671	-0.363	0.221	0.100	0.394	1000.671	-0.336	0.256	0.189	0.410
ABAT	68.764	1.176	0.609	0.054	0.122	68.764	0.664	0.610	0.277	0.547	68.764	-0.512	0.702	0.466	0.681
DPH1	250.260	-0.523	0.172	0.002	0.011	250.260	0.059	0.167	0.723	0.852	250.260	0.582	0.197	0.003	0.030
DPH2	516.440	-1.102	0.417	0.008	0.029	516.440	-0.834	0.416	0.045	0.334	516.440	0.268	0.481	0.578	0.763
DPYD	1118.808	-0.255	0.303	0.400	0.538	1118.808	0.402	0.302	0.184	0.470	1118.808	0.657	0.350	0.060	0.205
DPYSL2	2173.384	0.320	0.184	0.082	0.169	2173.384	0.477	0.184	0.009	0.297	2173.384	0.157	0.212	0.459	0.676
DPYSL3	41.821	0.459	0.290	0.114	0.215	41.821	0.173	0.292	0.553	0.745	41.821	-0.286	0.336	0.395	0.623
DR1	1925.250	-0.670	0.144	0.000	0.000	1925.250	-0.237	0.143	0.097	0.391	1925.250	0.433	0.166	0.009	0.061
DRD4	11.347	1.798	0.742	0.015	0.047	11.347	0.885	0.757	0.242	0.518	11.347	-0.912	0.847	0.282	0.516
DRG2	305.765	-0.016	0.149	0.917	0.947	305.765	-0.118	0.148	0.428	0.663	305.765	-0.102	0.173	0.555	0.747
JAG1	967.204	-0.680	0.296	0.022	0.061	967.204	-0.596	0.296	0.044	0.332	967.204	0.084	0.342	0.806	0.903
ARID3A	596.911	0.561	0.256	0.029	0.076	596.911	0.300	0.257	0.242	0.518	596.911	-0.260	0.296	0.379	0.610
ATN1	3081.413	0.236	0.181	0.192	0.317	3081.413	0.315	0.181	0.081	0.372	3081.413	0.079	0.209	0.705	0.847
DSC2	38.258	-0.028	0.496	0.956	0.973	38.258	0.420	0.487	0.389	0.635	38.258	0.447	0.568	0.431	0.654
RCAN1	965.575	0.233	0.120	0.052	0.119	965.575	-0.160	0.120	0.182	0.469	965.575	-0.393	0.139	0.005	0.039
DSG2	4097.635	-0.211	0.202	0.298	0.436	4097.635	0.155	0.202	0.443	0.674	4097.635	0.366	0.234	0.117	0.307
AGT	41.426	4.159	0.732	0.000	0.000	41.426	1.095	0.759	0.149	0.442	41.426	-3.065	0.837	0.000	0.005
TSC22D3	11495.339	-1.155	0.495	0.020	0.057	11495.339	0.759	0.495	0.125	0.417	11495.339	1.913	0.571	0.001	0.011
DSP	2593.007	-0.349	0.336	0.299	0.438	2593.007	0.440	0.336	0.191	0.476	2593.007	0.789	0.388	0.042	0.164
SLC26A2	398.591	-0.730	0.350	0.037	0.092	398.591	-0.288	0.349	0.409	0.649	398.591	0.442	0.404	0.273	0.508
DTNA	377.196	-0.122	0.274	0.657	0.760	377.196	0.213	0.273	0.435	0.669	377.196	0.334	0.316	0.290	0.525
DTNB	230.671	0.023	0.202	0.908	0.941	230.671	0.202	0.200	0.314	0.577	230.671	0.178	0.233	0.443	0.663
HBEGF	123.059	-1.361	0.525	0.010	0.033	123.059	-1.530	0.525	0.004	0.297	123.059	-0.170	0.610	0.781	0.890
DTYMK	807.422	-0.415	0.324	0.200	0.326	807.422	-0.694	0.324	0.032	0.322	807.422	-0.279	0.374	0.457	0.675
ECM2	156.477	-1.813	0.358	0.000	0.000	156.477	0.203	0.344	0.556	0.747	156.477	2.015	0.410	0.000	0.000
DUSP1	6955.831	-0.767	0.233	0.001	0.006	6955.831	0.318	0.233	0.173	0.462	6955.831	1.085	0.269	0.000	0.002
DUSP2	151.425	-0.490	0.420	0.243	0.377	151.425	-0.811	0.421	0.054	0.342	151.425	-0.321	0.488	0.510	0.715
DUSP3	1325.494	0.654	0.131	0.000	0.000	1325.494	-0.202	0.133	0.128	0.420	1325.494	-0.856	0.152	0.000	0.000
DUSP4	592.755	0.247	0.183	0.176	0.298	592.755	0.509	0.181	0.005	0.297	592.755	0.262	0.210	0.213	0.438
DUSP5	1871.626	-1.079	0.667	0.106	0.204	1871.626	-1.657	0.668	0.013	0.305	1871.626	-0.577	0.771	0.454	0.672
DUSP6	347.051	-0.832	0.499	0.096	0.189	347.051	-1.338	0.500	0.007	0.297	347.051	-0.506	0.579	0.382	0.611
DUSP7	1188.825	-0.121	0.127	0.341	0.481	1188.825	0.081	0.126	0.519	0.724	1188.825	0.202	0.146	0.167	0.382
DUSP8	842.009	-0.067	0.331	0.838	0.894	842.009	-0.476	0.331	0.150	0.443	842.009	-0.409	0.383	0.285	0.520
DUSP9	35.315	-0.573	0.581	0.324	0.464	35.315	0.131	0.569	0.818	0.905	35.315	0.704	0.668	0.292	0.527
DUT	1684.691	-0.286	0.240	0.234	0.367	1684.691	-0.422	0.240	0.079	0.370	1684.691	-0.136	0.278	0.624	0.794
DVL1	3813.472	-0.116	0.180	0.521	0.649	3813.472	-0.111	0.180	0.537	0.735	3813.472	0.004	0.208	0.983	0.991
DVL2	638.756	0.017	0.149	0.909	0.942	638.756	0.183	0.148	0.217	0.497	638.756	0.166	0.172	0.335	0.569
DVL3	1959.497	-0.133	0.069	0.054	0.122	1959.497	0.013	0.068	0.846	0.920	1959.497	0.146	0.080	0.066	0.217
DYRK1A	632.425	-0.171	0.127	0.177	0.300	632.425	-0.044	0.126	0.725	0.853	632.425	0.127	0.146	0.387	0.615
TOR1A	628.323	-0.118	0.211	0.575	0.694	628.323	-0.360	0.211	0.087	0.380	628.323	-0.243	0.244	0.320	0.555
E2F1	281.716	-0.602	0.609	0.322	0.462	281.716	-1.725	0.611	0.005	0.297	281.716	-1.122	0.705	0.112	0.298
E2F2	224.159	-0.573	0.522	0.273	0.409	224.159	-1.178	0.524	0.024	0.316	224.159	-0.605	0.605	0.318	0.553
E2F3	281.789	-0.242	0.237	0.306	0.445	281.789	-0.594	0.238	0.012	0.305	281.789	-0.352	0.275	0.202	0.425
E2F4	1228.881	-0.314	0.324	0.333	0.473	1228.881	-0.729	0.324	0.025	0.316	1228.881	-0.416	0.375	0.267	0.502
E2F5	632.301	0.211	0.153	0.168	0.289	632.301	0.288	0.153	0.059	0.349	632.301	0.077	0.177	0.663	0.821

E2F6	184.581	-0.544	0.220	0.013	0.042	184.581	-0.371	0.217	0.087	0.380	184.581	0.173	0.255	0.499	0.706
E4F1	535.136	0.526	0.221	0.017	0.051	535.136	-0.052	0.222	0.816	0.904	535.136	-0.577	0.255	0.024	0.114
EBF1	125.552	1.278	0.382	0.001	0.005	125.552	-0.174	0.388	0.654	0.811	125.552	-1.452	0.444	0.001	0.014
ECE1	2933.197	-0.158	0.152	0.300	0.439	2933.197	-0.131	0.152	0.390	0.635	2933.197	0.027	0.176	0.879	0.942
TYMP	2344.694	1.171	0.199	0.000	0.000	2344.694	0.228	0.200	0.254	0.528	2344.694	-0.943	0.230	0.000	0.001
ECH1	2138.055	0.881	0.302	0.004	0.015	2138.055	0.537	0.302	0.075	0.364	2138.055	-0.344	0.348	0.324	0.558
ECHS1	2203.337	0.149	0.142	0.293	0.431	2203.337	0.090	0.142	0.525	0.728	2203.337	-0.059	0.164	0.718	0.854
ECM1	56.493	2.029	0.302	0.000	0.000	56.493	0.048	0.340	0.888	0.944	56.493	-1.981	0.363	0.000	0.000
ECT2	1467.528	-1.193	0.308	0.000	0.001	1467.528	-0.455	0.307	0.138	0.429	1467.528	0.738	0.355	0.038	0.153
EDA	184.326	0.001	0.255	0.998	0.999	184.326	0.615	0.251	0.014	0.306	184.326	0.614	0.292	0.035	0.147
ABCA1	259.150	-2.060	0.523	0.000	0.001	259.150	0.687	0.513	0.180	0.469	259.150	2.746	0.601	0.000	0.000
S1PR1	35.047	0.373	0.366	0.307	0.446	35.047	-0.387	0.383	0.311	0.575	35.047	-0.761	0.437	0.082	0.247
LPAR1	84.126	0.968	0.326	0.003	0.013	84.126	0.419	0.331	0.205	0.488	84.126	-0.549	0.376	0.144	0.350
EDN1	288.305	0.083	0.174	0.634	0.742	288.305	0.058	0.174	0.740	0.861	288.305	-0.025	0.201	0.900	0.953
EDN2	1460.518	1.044	0.884	0.238	0.371	1460.518	0.975	0.884	0.270	0.541	1460.518	-0.069	1.020	0.946	0.975
EDNRA	332.128	-1.194	0.255	0.000	0.000	332.128	0.364	0.248	0.142	0.433	332.128	1.558	0.292	0.000	0.000
AHCY	4605.898	-0.414	0.139	0.003	0.013	4605.898	0.152	0.139	0.272	0.543	4605.898	0.567	0.161	0.000	0.007
EDNRB	646.705	-0.201	0.170	0.237	0.370	646.705	-0.037	0.169	0.825	0.909	646.705	0.163	0.196	0.405	0.631
PHC1	1174.069	0.206	0.129	0.110	0.210	1174.069	-0.095	0.129	0.464	0.689	1174.069	-0.301	0.149	0.044	0.168
PHC2	4233.228	-0.214	0.168	0.203	0.330	4233.228	-0.011	0.168	0.947	0.974	4233.228	0.203	0.194	0.296	0.531
EEF1A1	471986.652	0.006	0.310	0.986	0.992	471986.652	0.424	0.310	0.172	0.461	471986.652	0.418	0.358	0.243	0.474
EEF1A2	6290.664	-0.043	0.197	0.827	0.886	6290.664	0.309	0.197	0.116	0.409	6290.664	0.352	0.227	0.121	0.313
PGAM5	1371.052	-0.632	0.367	0.085	0.173	1371.052	-0.842	0.367	0.022	0.314	1371.052	-0.209	0.424	0.621	0.792
HIGD2A	1454.163	0.738	0.292	0.012	0.038	1454.163	0.344	0.292	0.238	0.515	1454.163	-0.393	0.337	0.244	0.475
AGO3	422.945	-0.107	0.161	0.506	0.636	422.945	-0.004	0.160	0.981	0.989	422.945	0.104	0.186	0.579	0.763
AGO4	257.158	0.036	0.198	0.857	0.907	257.158	0.258	0.197	0.189	0.475	257.158	0.223	0.228	0.330	0.564
SCAMP5	54.027	0.273	0.351	0.436	0.572	54.027	-0.465	0.359	0.194	0.478	54.027	-0.739	0.412	0.073	0.232
EEF1B2	6459.065	-0.056	0.175	0.747	0.831	6459.065	0.262	0.175	0.133	0.425	6459.065	0.319	0.202	0.114	0.302
EEF1D	13060.607	0.325	0.197	0.100	0.196	13060.607	0.178	0.197	0.367	0.617	13060.607	-0.147	0.228	0.520	0.723
EEF2	148354.521	-0.127	0.262	0.627	0.737	148354.521	0.395	0.262	0.132	0.424	148354.521	0.522	0.302	0.084	0.252
EIF2D	1477.038	0.135	0.191	0.479	0.612	1477.038	0.329	0.190	0.084	0.376	1477.038	0.194	0.220	0.379	0.610
EFNA1	681.981	1.605	0.348	0.000	0.000	681.981	0.813	0.349	0.020	0.306	681.981	-0.791	0.402	0.049	0.180
EFNA3	363.931	1.301	0.596	0.029	0.077	363.931	0.968	0.596	0.105	0.397	363.931	-0.333	0.688	0.628	0.797
EFNA4	134.559	1.307	0.405	0.001	0.007	134.559	0.751	0.406	0.065	0.353	134.559	-0.556	0.466	0.233	0.462
EFNA5	507.635	0.448	0.162	0.006	0.022	507.635	0.103	0.163	0.527	0.729	507.635	-0.345	0.188	0.066	0.218
EFNB1	938.869	0.619	0.127	0.000	0.000	938.869	0.021	0.128	0.869	0.933	938.869	-0.598	0.148	0.000	0.001
EFNB2	215.464	-1.146	0.382	0.003	0.012	215.464	-1.049	0.381	0.006	0.297	215.464	0.097	0.443	0.827	0.915
CELSR3	719.381	0.200	0.328	0.542	0.666	719.381	0.456	0.327	0.164	0.454	719.381	0.256	0.378	0.499	0.706
CELSR2	537.432	-0.096	0.482	0.842	0.896	537.432	0.454	0.481	0.346	0.602	537.432	0.549	0.556	0.323	0.557
MEGF6	145.015	1.230	0.924	0.183	0.307	145.015	0.747	0.925	0.419	0.657	145.015	-0.483	1.067	0.651	0.812
MEGF8	1435.545	0.521	0.359	0.147	0.261	1435.545	0.544	0.359	0.130	0.422	1435.545	0.023	0.414	0.955	0.979
MEGF9	593.223	0.469	0.346	0.176	0.298	593.223	0.385	0.346	0.266	0.538	593.223	-0.084	0.400	0.834	0.919
EGFR	4409.618	-1.003	0.319	0.002	0.009	4409.618	0.149	0.319	0.641	0.805	4409.618	1.152	0.369	0.002	0.020
EGR1	558.043	-1.597	0.728	0.028	0.075	558.043	-2.136	0.728	0.003	0.297	558.043	-0.539	0.842	0.522	0.724
SDR16C5	28.375	-0.962	0.446	0.031	0.081	28.375	-0.419	0.427	0.326	0.586	28.375	0.542	0.515	0.292	0.528
PRXL2C	379.896	0.578	0.181	0.001	0.007	379.896	0.073	0.182	0.687	0.831	379.896	-0.505	0.209	0.016	0.088
ZNF367	499.356	-0.941	0.403	0.019	0.056	499.356	-0.925	0.403	0.022	0.314	499.356	0.017	0.466	0.971	0.986
AHR	813.155	-1.146	0.236	0.000	0.000	813.155	-0.676	0.235	0.004	0.297	813.155	0.470	0.272	0.084	0.252
METTL15	323.252	-0.149	0.184	0.418	0.555	323.252	0.095	0.182	0.603	0.780	323.252	0.244	0.212	0.250	0.482
EHHADH	201.880	-0.075	0.256	0.771	0.848	201.880	-0.052	0.255	0.840	0.917	201.880	0.023	0.296	0.938	0.972
MPZL3	27.366	0.112	0.409	0.785	0.858	27.366	-0.247	0.414	0.551	0.743	27.366	-0.358	0.479	0.455	0.673
IMMP1L	117.466	-0.162	0.281	0.564	0.685	117.466	-0.387	0.281	0.169	0.458	117.466	-0.225	0.327	0.491	0.701
RILPL2	674.489	0.803	0.180	0.000	0.000	674.489	0.187	0.181	0.301	0.567	674.489	-0.616	0.208	0.003	0.029
DNAH10	9.170	1.719	0.675	0.011	0.036	9.170	1.110	0.688	0.107	0.399	9.170	-0.609	0.757	0.421	0.645
EIF1AX	2362.886	-0.564	0.172	0.001	0.006	2362.886	-0.441	0.172	0.010	0.299	2362.886	0.123	0.199	0.537	0.735
DTX3	64.886	0.903	0.336	0.007	0.026	64.886	0.444	0.339	0.190	0.476	64.886	-0.460	0.387	0.235	0.465
METTL7B	10.615	1.485	0.516	0.004	0.017	10.615	0.449	0.564	0.426	0.662	10.615	-1.036	0.597	0.083	0.250
ZFC3H1	403.466	-0.051	0.295	0.862	0.910	403.466	0.217	0.294	0.462	0.687	403.466	0.268	0.341	0.431	0.653
MYRFL	8.808	2.476	0.833	0.003	0.013	8.808	0.719	0.883	0.415	0.654	8.808	-1.756	0.957	0.066	0.218
PLBD2	1493.823	1.296	0.163	0.000	0.000	1493.823	0.143	0.164	0.384	0.629	1493.823	-1.153	0.189	0.000	0.000
EEF2KMT	429.031	-0.553	0.439	0.208	0.337	429.031	-1.176	0.440	0.008	0.297	429.031	-0.623	0.509	0.221	0.448
EIF2S1	2172.074	-0.973	0.280	0.001	0.003	2172.074	-0.660	0.280	0.018	0.306	2172.074	0.312	0.323	0.334	0.569
DCP1B	77.960	-0.070	0.335	0.834	0.890	77.960	0.318	0.329	0.334	0.593	77.960	0.388	0.385	0.313	0.548
ANO6	2794.669	-0.470	0.366	0.198	0.324	2794.669	0.289	0.365	0.429	0.664	2794.669	0.759	0.422	0.072	0.229
ARID2	482.168	-0.666	0.147	0.000	0.000	482.168	-0.018	0.144	0.901	0.950	482.168	0.648	0.169	0.000	0.003
EIF2B1	881.425	-0.422	0.218	0.053	0.120	881.425	-0.459	0.217	0.035	0.322	881.425	-0.037	0.252	0.883	0.945
PAOX	13.702	1.110	0.490	0.024	0.065	13.702	0.273	0.513	0.594	0.774	13.702	-0.837	0.570	0.142	0.348

FAM24B	95.800	-0.620	0.297	0.037	0.092	95.800	0.166	0.286	0.563	0.751	95.800	0.785	0.340	0.021	0.104
EIF2S3	12590.398	-0.132	0.209	0.527	0.653	12590.398	0.268	0.209	0.199	0.482	12590.398	0.400	0.241	0.097	0.274
LINC00638	13.457	0.698	0.415	0.093	0.185	13.457	-0.090	0.440	0.837	0.916	13.457	-0.789	0.494	0.110	0.296
EPHA2	4275.057	-0.619	0.224	0.006	0.022	4275.057	-0.288	0.224	0.197	0.481	4275.057	0.330	0.258	0.201	0.425
LINC01599	11.445	1.445	0.584	0.013	0.042	11.445	0.795	0.604	0.188	0.474	11.445	-0.650	0.658	0.323	0.558
FAM227B	271.502	0.218	0.181	0.227	0.359	271.502	0.151	0.180	0.404	0.646	271.502	-0.068	0.209	0.745	0.869
LCTL	119.714	-0.764	0.436	0.079	0.165	119.714	-1.035	0.436	0.018	0.306	119.714	-0.270	0.506	0.594	0.773
UBR1	430.461	-0.858	0.229	0.000	0.002	430.461	-0.287	0.227	0.206	0.488	430.461	0.571	0.265	0.031	0.134
SNAI3-AS1	23.891	1.673	0.403	0.000	0.000	23.891	-0.238	0.456	0.602	0.779	23.891	-1.911	0.492	0.000	0.002
LDHD	48.937	-0.531	0.896	0.554	0.676	48.937	0.638	0.889	0.473	0.695	48.937	1.169	1.032	0.257	0.491
FCSK	259.281	0.345	0.168	0.040	0.098	259.281	0.443	0.167	0.008	0.297	259.281	0.097	0.193	0.615	0.788
MLKL	197.740	0.027	0.260	0.916	0.947	197.740	-0.350	0.261	0.181	0.469	197.740	-0.377	0.302	0.212	0.438
EIF4A1	587.113	0.218	0.147	0.138	0.249	587.113	0.092	0.147	0.529	0.730	587.113	-0.125	0.170	0.461	0.678
ZNF778	201.037	-0.738	0.292	0.012	0.038	201.037	-0.525	0.290	0.070	0.359	201.037	0.213	0.338	0.530	0.729
ACSF3	424.158	0.104	0.135	0.440	0.576	424.158	0.095	0.134	0.479	0.699	424.158	-0.009	0.156	0.953	0.978
WDR90	379.824	0.050	0.251	0.843	0.897	379.824	0.540	0.249	0.030	0.322	379.824	0.490	0.289	0.090	0.262
EME2	1143.505	0.313	0.156	0.045	0.106	1143.505	0.086	0.156	0.582	0.766	1143.505	-0.228	0.180	0.207	0.431
NLRC3	14.549	0.269	0.573	0.639	0.746	14.549	0.103	0.573	0.858	0.927	14.549	-0.166	0.663	0.802	0.901
NSMCE1	992.907	0.175	0.217	0.420	0.557	992.907	0.314	0.216	0.147	0.439	992.907	0.139	0.250	0.578	0.763
EIF4A2	13983.982	-0.020	0.279	0.944	0.965	13983.982	0.429	0.279	0.124	0.416	13983.982	0.449	0.322	0.164	0.379
ZNF48	251.573	-0.071	0.149	0.632	0.741	251.573	0.082	0.147	0.577	0.762	251.573	0.153	0.172	0.372	0.604
EIF4B	33400.178	-0.737	0.342	0.031	0.081	33400.178	0.416	0.342	0.224	0.503	33400.178	1.153	0.395	0.004	0.032
EIF4E	768.621	-0.783	0.271	0.004	0.016	768.621	-0.675	0.270	0.013	0.305	768.621	0.109	0.313	0.729	0.859
EIF4EBP1	3273.526	0.030	0.252	0.905	0.940	3273.526	-0.002	0.252	0.993	0.996	3273.526	-0.032	0.291	0.912	0.960
EIF4EBP2	1047.717	-0.146	0.193	0.449	0.584	1047.717	0.043	0.192	0.821	0.906	1047.717	0.189	0.222	0.394	0.622
EIF4G1	11416.799	-0.663	0.297	0.026	0.070	11416.799	-0.654	0.297	0.028	0.321	11416.799	0.010	0.343	0.978	0.988
EIF4G2	12195.611	-0.627	0.171	0.000	0.002	12195.611	-0.387	0.171	0.024	0.316	12195.611	0.241	0.198	0.223	0.451
EIF5	4872.301	-1.138	0.324	0.000	0.003	4872.301	-0.887	0.324	0.006	0.297	4872.301	0.250	0.374	0.503	0.710
EIF5A	11778.500	0.051	0.308	0.870	0.916	11778.500	-0.579	0.308	0.061	0.350	11778.500	-0.629	0.356	0.077	0.240
ELANE	52.162	0.886	1.422	0.533	0.659	52.162	1.397	1.421	0.326	0.586	52.162	0.510	1.640	0.756	0.875
SERPINB1	1739.824	0.452	0.288	0.117	0.220	1739.824	0.521	0.288	0.071	0.360	1739.824	0.068	0.333	0.838	0.921
DZIP1L	69.891	0.421	0.197	0.033	0.084	69.891	0.132	0.199	0.507	0.716	69.891	-0.289	0.228	0.206	0.430
TTC21A	33.829	1.019	0.395	0.010	0.034	33.829	0.261	0.403	0.517	0.722	33.829	-0.757	0.458	0.098	0.277
ELAVL2	52.191	-1.343	0.360	0.000	0.002	52.191	-0.166	0.337	0.623	0.792	52.191	1.178	0.411	0.004	0.036
ELAVL1	1492.213	-0.214	0.217	0.325	0.465	1492.213	-0.408	0.217	0.060	0.350	1492.213	-0.194	0.251	0.439	0.659
ZNF627	166.690	-0.342	0.178	0.055	0.124	166.690	0.203	0.172	0.238	0.515	166.690	0.545	0.204	0.008	0.054
ELF1	830.509	-0.909	0.251	0.000	0.002	830.509	-0.110	0.249	0.660	0.815	830.509	0.800	0.290	0.006	0.045
ZNF585A	101.540	0.249	0.232	0.283	0.421	101.540	0.155	0.231	0.501	0.714	101.540	-0.093	0.268	0.727	0.859
GGN	10.621	0.703	0.705	0.319	0.458	10.621	-0.133	0.727	0.855	0.926	10.621	-0.836	0.827	0.312	0.547
CADM4	57.151	1.183	0.473	0.012	0.040	57.151	0.221	0.481	0.646	0.807	57.151	-0.961	0.548	0.079	0.244
THAP8	96.072	1.159	0.477	0.015	0.046	96.072	0.763	0.478	0.110	0.403	96.072	-0.396	0.549	0.471	0.685
U2AF1L4	52.032	0.033	0.311	0.915	0.946	52.032	0.054	0.307	0.860	0.929	52.032	0.021	0.359	0.954	0.979
ELF2	572.378	-0.462	0.111	0.000	0.000	572.378	-0.131	0.108	0.227	0.505	572.378	0.331	0.128	0.009	0.063
ADM5	18.668	1.425	0.625	0.023	0.063	18.668	0.794	0.634	0.210	0.491	18.668	-0.631	0.714	0.377	0.608
ALG14	135.561	0.068	0.219	0.757	0.839	135.561	-0.313	0.221	0.157	0.449	135.561	-0.381	0.256	0.137	0.339
FAM76A	144.143	0.402	0.160	0.012	0.039	144.143	-0.364	0.166	0.028	0.321	144.143	-0.766	0.190	0.000	0.001
ELF3	225.815	3.392	0.226	0.000	0.000	225.815	0.632	0.243	0.009	0.297	225.815	-2.760	0.262	0.000	0.000
TMEM201	391.109	-0.436	0.347	0.209	0.338	391.109	-0.718	0.348	0.039	0.327	391.109	-0.282	0.402	0.484	0.696
FAAP20	653.705	0.898	0.229	0.000	0.001	653.705	0.352	0.229	0.125	0.417	653.705	-0.547	0.264	0.038	0.154
ABCA2	3019.294	0.292	0.359	0.417	0.554	3019.294	0.639	0.359	0.075	0.364	3019.294	0.347	0.415	0.402	0.629
ELF4	812.866	-0.071	0.106	0.500	0.631	812.866	-0.015	0.105	0.887	0.944	812.866	0.056	0.122	0.644	0.807
CC2D1B	707.264	0.430	0.091	0.000	0.000	707.264	0.051	0.091	0.575	0.760	707.264	-0.379	0.105	0.000	0.006
NBPF11	116.507	-0.239	0.257	0.353	0.493	116.507	0.097	0.253	0.701	0.839	116.507	0.336	0.296	0.256	0.490
NUDT17	105.555	1.641	0.359	0.000	0.000	105.555	0.499	0.364	0.170	0.460	105.555	-1.143	0.414	0.006	0.045
TXLNA	1201.915	-0.245	0.207	0.237	0.370	1201.915	-0.251	0.207	0.226	0.505	1201.915	-0.005	0.239	0.982	0.990
SPAG17	136.385	-0.917	0.237	0.000	0.001	136.385	-0.294	0.229	0.198	0.482	136.385	0.623	0.273	0.022	0.109
KRTCAP2	870.493	1.174	0.251	0.000	0.000	870.493	0.324	0.251	0.198	0.481	870.493	-0.850	0.289	0.003	0.031
CRTC2	784.506	0.217	0.139	0.119	0.223	784.506	0.104	0.139	0.454	0.681	784.506	-0.113	0.161	0.484	0.696
TMEM51-AS1	18.919	2.090	0.622	0.001	0.005	18.919	1.811	0.625	0.004	0.297	18.919	-0.279	0.673	0.679	0.830
ELK1	1027.291	0.259	0.129	0.045	0.106	1027.291	-0.018	0.129	0.889	0.944	1027.291	-0.277	0.149	0.063	0.212
IBA57	154.733	-0.273	0.206	0.184	0.308	154.733	0.217	0.200	0.278	0.548	154.733	0.490	0.236	0.038	0.153
LINC00528	19.132	0.623	0.489	0.203	0.330	19.132	1.044	0.475	0.028	0.321	19.132	0.421	0.548	0.442	0.663
RNF215	143.495	0.895	0.246	0.000	0.002	143.495	0.629	0.247	0.011	0.300	143.495	-0.266	0.282	0.346	0.580
APOBEC3F	93.770	0.342	0.362	0.345	0.486	93.770	0.552	0.360	0.124	0.416	93.770	0.211	0.416	0.612	0.786
ELK3	1511.604	-0.776	0.203	0.000	0.001	1511.604	-0.021	0.202	0.916	0.958	1511.604	0.755	0.235	0.001	0.016
CREG2	16.576	-0.643	0.791	0.416	0.554	16.576	0.284	0.764	0.710	0.845	16.576	0.927	0.905	0.306	0.541
TET3	393.835	-0.897	0.257	0.000	0.003	393.835	-0.458	0.255	0.073	0.361	393.835	0.438	0.297	0.140	0.344

ELK4	934.476	-0.232	0.171	0.176	0.297	934.476	-0.305	0.171	0.075	0.364	934.476	-0.072	0.198	0.715	0.852
ANKRD23	113.614	0.403	0.194	0.037	0.092	113.614	0.049	0.196	0.802	0.896	113.614	-0.354	0.225	0.116	0.305
APLF	38.638	0.119	0.424	0.779	0.853	38.638	0.351	0.418	0.401	0.644	38.638	0.232	0.487	0.633	0.801
PIKFYVE	513.239	-0.510	0.260	0.050	0.116	513.239	-0.177	0.259	0.495	0.710	513.239	0.333	0.301	0.269	0.504
ELN	18.172	-0.153	0.863	0.859	0.908	18.172	0.522	0.851	0.540	0.737	18.172	0.675	0.991	0.496	0.704
KRTCAP3	21.930	0.954	0.467	0.041	0.099	21.930	0.327	0.475	0.491	0.707	21.930	-0.627	0.540	0.245	0.476
TMEM17	9.801	0.662	0.493	0.179	0.302	9.801	-0.373	0.531	0.483	0.701	9.801	-1.034	0.596	0.083	0.250
SPRED2	943.685	-0.419	0.120	0.000	0.003	943.685	-0.165	0.119	0.165	0.455	943.685	0.254	0.139	0.068	0.220
TIGD1	65.176	0.118	0.307	0.700	0.793	65.176	-0.589	0.314	0.061	0.350	65.176	-0.707	0.362	0.051	0.185
ALG1L	53.968	-0.438	0.302	0.148	0.261	53.968	-0.231	0.296	0.436	0.669	53.968	0.207	0.350	0.555	0.747
C3orf67	107.657	0.120	0.392	0.759	0.840	107.657	0.486	0.388	0.211	0.492	107.657	0.365	0.450	0.417	0.642
KCTD6	123.617	0.405	0.160	0.011	0.038	123.617	0.054	0.162	0.738	0.860	123.617	-0.350	0.186	0.060	0.205
LIPH	45.369	0.074	0.301	0.807	0.873	45.369	-0.207	0.303	0.495	0.710	45.369	-0.280	0.352	0.426	0.649
ARL13B	396.429	-0.585	0.354	0.098	0.194	396.429	-0.793	0.354	0.025	0.316	396.429	-0.209	0.409	0.610	0.785
DHFR2	175.650	0.233	0.192	0.224	0.355	175.650	0.051	0.192	0.791	0.891	175.650	-0.182	0.222	0.411	0.637
EML1	119.398	-0.234	0.429	0.585	0.701	119.398	-0.905	0.432	0.036	0.324	119.398	-0.670	0.499	0.179	0.398
RPL22L1	1044.138	0.304	0.230	0.186	0.310	1044.138	0.121	0.230	0.598	0.777	1044.138	-0.183	0.266	0.490	0.700
SLC51A	93.030	-0.193	0.283	0.495	0.627	93.030	-0.351	0.283	0.215	0.494	93.030	-0.158	0.329	0.631	0.799
FBXO45	984.432	-1.255	0.326	0.000	0.001	984.432	-0.746	0.325	0.022	0.314	984.432	0.509	0.376	0.176	0.393
KLHDC8B	541.284	-0.033	0.728	0.963	0.978	541.284	0.532	0.727	0.464	0.689	541.284	0.566	0.840	0.501	0.708
MUC20	52.878	1.230	0.312	0.000	0.001	52.878	0.741	0.316	0.019	0.306	52.878	-0.489	0.355	0.168	0.383
EMD	1928.954	0.269	0.201	0.179	0.302	1928.954	0.323	0.200	0.107	0.399	1928.954	0.054	0.232	0.816	0.908
MARK2	1078.222	-0.394	0.143	0.006	0.022	1078.222	-0.193	0.142	0.175	0.464	1078.222	0.202	0.165	0.222	0.449
CEP112	146.241	0.420	0.346	0.224	0.355	146.241	0.455	0.345	0.187	0.473	146.241	0.035	0.398	0.929	0.968
TVP23C	55.415	0.489	0.393	0.214	0.344	55.415	0.275	0.394	0.485	0.702	55.415	-0.214	0.454	0.638	0.803
CENPV	225.443	-0.395	0.266	0.137	0.248	225.443	-0.040	0.264	0.878	0.938	225.443	0.355	0.307	0.248	0.479
FLCN	420.463	0.472	0.229	0.039	0.096	420.463	0.241	0.229	0.293	0.561	420.463	-0.231	0.265	0.382	0.611
PLD6	131.576	0.024	0.240	0.922	0.950	131.576	0.276	0.235	0.240	0.516	131.576	0.252	0.275	0.359	0.591
ARHGAP27	242.879	1.113	0.374	0.003	0.013	242.879	0.307	0.375	0.413	0.652	242.879	-0.806	0.432	0.062	0.210
ZNF385C	17.262	1.254	0.501	0.012	0.040	17.262	0.475	0.516	0.357	0.611	17.262	-0.779	0.577	0.177	0.394
SAMD14	105.669	0.524	0.445	0.239	0.372	105.669	0.559	0.444	0.208	0.490	105.669	0.035	0.512	0.946	0.975
EMP1	2394.634	-1.305	0.171	0.000	0.000	2394.634	0.079	0.169	0.641	0.805	2394.634	1.384	0.197	0.000	0.000
LYRM9	33.023	0.604	0.716	0.399	0.537	33.023	0.903	0.712	0.205	0.488	33.023	0.299	0.822	0.716	0.853
CENPX	888.126	-0.073	0.334	0.827	0.886	888.126	-0.521	0.335	0.119	0.412	888.126	-0.448	0.387	0.247	0.478
LRRC45	339.484	0.749	0.229	0.001	0.006	339.484	-0.043	0.230	0.853	0.925	339.484	-0.792	0.265	0.003	0.027
SLC39A11	139.566	0.211	0.158	0.180	0.303	139.566	-0.241	0.161	0.133	0.424	139.566	-0.453	0.185	0.015	0.083
AMZ2P1	98.328	-0.015	0.327	0.962	0.977	98.328	0.343	0.323	0.288	0.557	98.328	0.358	0.375	0.340	0.574
TRIM65	878.675	-0.268	0.171	0.117	0.219	878.675	-0.186	0.170	0.274	0.545	878.675	0.082	0.197	0.679	0.830
UNC13D	53.172	0.392	0.397	0.324	0.464	53.172	0.374	0.396	0.344	0.600	53.172	-0.018	0.457	0.969	0.985
RDM1	82.126	-0.301	0.620	0.628	0.737	82.126	0.388	0.616	0.529	0.730	82.126	0.689	0.714	0.335	0.569
EMP2	25.902	0.193	0.332	0.560	0.681	25.902	0.219	0.326	0.502	0.714	25.902	0.026	0.381	0.947	0.975
EMP3	1554.360	1.007	0.270	0.000	0.002	1554.360	0.248	0.270	0.360	0.612	1554.360	-0.760	0.312	0.015	0.085
RAB12	681.220	0.057	0.118	0.630	0.739	681.220	0.055	0.117	0.636	0.801	681.220	-0.001	0.136	0.993	0.996
ADGRE1	25.711	1.380	0.408	0.001	0.004	25.711	0.624	0.418	0.135	0.426	25.711	-0.755	0.467	0.106	0.290
ZNF584	359.705	-0.675	0.255	0.008	0.029	359.705	-0.551	0.254	0.030	0.322	359.705	0.124	0.296	0.675	0.828
HACD2	2215.842	-0.561	0.166	0.001	0.005	2215.842	-0.272	0.166	0.101	0.394	2215.842	0.289	0.192	0.131	0.329
STT3B	5494.472	-0.441	0.144	0.002	0.011	5494.472	-0.076	0.144	0.596	0.775	5494.472	0.365	0.166	0.028	0.127
PDE12	575.702	-0.436	0.285	0.126	0.232	575.702	-0.671	0.285	0.019	0.306	575.702	-0.234	0.330	0.478	0.691
DENND6A	420.169	-0.354	0.223	0.112	0.213	420.169	-0.200	0.222	0.366	0.617	420.169	0.153	0.257	0.552	0.745
AADACP1	24.592	-0.860	0.588	0.144	0.256	24.592	-0.905	0.585	0.122	0.413	24.592	-0.045	0.688	0.948	0.976
CTTN	3625.452	0.110	0.258	0.669	0.769	3625.452	-0.544	0.258	0.035	0.322	3625.452	-0.654	0.298	0.028	0.126
C4orf46	277.974	-0.479	0.242	0.048	0.113	277.974	-0.328	0.241	0.174	0.464	277.974	0.151	0.280	0.590	0.771
TMEM154	189.055	-1.030	0.452	0.023	0.064	189.055	-1.234	0.452	0.006	0.297	189.055	-0.204	0.524	0.697	0.841
EMX2	98.907	-0.869	0.342	0.011	0.036	98.907	0.226	0.319	0.480	0.699	98.907	1.095	0.388	0.005	0.040
SMIM14	354.959	0.568	0.481	0.237	0.370	354.959	0.563	0.480	0.241	0.517	354.959	-0.005	0.554	0.993	0.996
EN1	287.183	0.962	0.236	0.000	0.001	287.183	0.393	0.237	0.097	0.392	287.183	-0.569	0.273	0.037	0.151
TMEM192	752.925	-0.009	0.115	0.938	0.961	752.925	-0.124	0.115	0.279	0.549	752.925	-0.115	0.133	0.386	0.615
RWDD4	408.651	-0.388	0.208	0.061	0.135	408.651	-0.547	0.208	0.008	0.297	408.651	-0.159	0.241	0.509	0.715
PRIMPOL	213.422	-0.465	0.176	0.008	0.029	213.422	-0.177	0.173	0.307	0.572	213.422	0.288	0.203	0.157	0.368
CRYBG1	47.002	0.393	0.318	0.216	0.346	47.002	0.371	0.315	0.239	0.516	47.002	-0.022	0.364	0.952	0.978
EN2	177.178	-0.091	0.464	0.844	0.898	177.178	-0.913	0.467	0.051	0.338	177.178	-0.822	0.540	0.127	0.323
TAPT1	260.960	-0.448	0.150	0.003	0.013	260.960	-0.178	0.146	0.224	0.503	260.960	0.270	0.173	0.119	0.310
TAPT1-AS1	10.024	-0.550	0.645	0.394	0.533	10.024	0.168	0.610	0.782	0.886	10.024	0.718	0.735	0.329	0.563
SPATA24	59.349	0.458	0.285	0.108	0.208	59.349	0.738	0.279	0.008	0.297	59.349	0.279	0.324	0.388	0.616
DNAJC18	263.809	-0.292	0.452	0.518	0.646	263.809	0.562	0.450	0.211	0.492	263.809	0.855	0.521	0.101	0.282
ENDOG	656.350	0.399	0.277	0.150	0.264	656.350	-0.243	0.278	0.382	0.629	656.350	-0.642	0.321	0.045	0.171
FAM153B	9.168	2.608	1.022	0.011	0.036	9.168	2.820	1.013	0.005	0.297	9.168	0.212	1.086	0.846	0.925

LOC202181	13.732	0.864	0.494	0.081	0.167	13.732	0.656	0.495	0.185	0.472	13.732	-0.208	0.562	0.711	0.850
ENG	1040.955	0.008	0.308	0.979	0.987	1040.955	-0.468	0.309	0.130	0.422	1040.955	-0.476	0.357	0.182	0.400
CCDC125	372.938	-0.305	0.188	0.105	0.202	372.938	-0.195	0.187	0.298	0.565	372.938	0.111	0.218	0.611	0.786
ENO1	116689.774	0.158	0.248	0.523	0.650	116689.774	0.468	0.248	0.059	0.349	116689.774	0.309	0.286	0.280	0.514
STK32A	18.598	1.480	0.478	0.002	0.010	18.598	0.560	0.495	0.257	0.530	18.598	-0.919	0.550	0.094	0.270
ENO2	2896.746	1.661	0.833	0.046	0.109	2896.746	1.044	0.833	0.210	0.491	2896.746	-0.618	0.962	0.521	0.723
ENO3	3334.645	-0.052	0.223	0.817	0.880	3334.645	-0.208	0.223	0.351	0.605	3334.645	-0.156	0.257	0.544	0.740
PAXIP1-AS1	148.120	0.087	0.189	0.644	0.750	148.120	0.245	0.186	0.189	0.475	148.120	0.157	0.217	0.469	0.684
ENSA	2474.833	0.463	0.183	0.011	0.037	2474.833	-0.090	0.183	0.622	0.791	2474.833	-0.553	0.211	0.009	0.060
TMEM184A	789.071	0.773	0.267	0.004	0.016	789.071	0.014	0.268	0.959	0.979	789.071	-0.760	0.309	0.014	0.081
AK1	160.688	0.693	0.312	0.026	0.071	160.688	-0.581	0.319	0.069	0.357	160.688	-1.274	0.366	0.000	0.008
SLC29A1	1175.986	-0.515	0.271	0.058	0.128	1175.986	-0.400	0.271	0.140	0.431	1175.986	0.115	0.313	0.713	0.851
ADCK5	93.654	0.993	0.217	0.000	0.000	93.654	0.026	0.226	0.909	0.955	93.654	-0.967	0.255	0.000	0.003
TSNARE1	119.685	0.735	0.362	0.042	0.101	119.685	0.756	0.361	0.036	0.324	119.685	0.021	0.415	0.960	0.981
TUBB	140938.790	0.219	0.102	0.032	0.083	140938.790	0.063	0.102	0.538	0.735	140938.790	-0.156	0.118	0.186	0.406
R3HCC1	486.064	-0.155	0.144	0.283	0.421	486.064	0.136	0.143	0.341	0.599	486.064	0.291	0.166	0.080	0.245
TMEM268	373.356	-1.066	0.217	0.000	0.000	373.356	-0.324	0.214	0.129	0.422	373.356	0.742	0.250	0.003	0.029
C9orf72	124.649	-0.046	0.220	0.833	0.890	124.649	-0.194	0.220	0.378	0.625	124.649	-0.147	0.256	0.564	0.754
CCDC171	14.215	0.031	0.497	0.950	0.970	14.215	0.459	0.479	0.338	0.596	14.215	0.428	0.565	0.449	0.668
NAIF1	323.029	-0.439	0.141	0.002	0.009	323.029	-0.081	0.137	0.556	0.747	323.029	0.358	0.162	0.027	0.124
FAM219A	917.177	0.300	0.179	0.094	0.187	917.177	0.136	0.179	0.446	0.677	917.177	-0.163	0.207	0.429	0.652
CCDC107	603.037	1.700	0.258	0.000	0.000	603.037	0.226	0.260	0.384	0.630	603.037	-1.474	0.298	0.000	0.000
ANKS6	529.646	-0.221	0.161	0.171	0.292	529.646	-0.210	0.161	0.192	0.477	529.646	0.011	0.187	0.952	0.978
EP300	1242.325	-0.789	0.215	0.000	0.002	1242.325	-0.263	0.214	0.220	0.499	1242.325	0.526	0.249	0.034	0.144
EPAS1	3890.118	-0.225	0.257	0.382	0.521	3890.118	-0.539	0.257	0.036	0.324	3890.118	-0.314	0.297	0.290	0.525
LINC01560	32.449	-0.146	0.337	0.665	0.766	32.449	0.298	0.323	0.356	0.610	32.449	0.444	0.384	0.247	0.479
SLC25A43	685.897	0.152	0.158	0.335	0.475	685.897	0.215	0.157	0.171	0.460	685.897	0.063	0.182	0.728	0.859
EPB41	982.124	0.109	0.195	0.575	0.694	982.124	0.273	0.195	0.161	0.452	982.124	0.164	0.225	0.467	0.682
ZNF449	56.611	-0.443	0.318	0.163	0.282	56.611	0.223	0.306	0.465	0.689	56.611	0.667	0.363	0.067	0.218
VMA21	591.871	0.216	0.208	0.299	0.438	591.871	-0.204	0.209	0.328	0.588	591.871	-0.420	0.241	0.081	0.247
EPB41L1	377.868	1.369	0.200	0.000	0.000	377.868	0.211	0.203	0.299	0.566	377.868	-1.158	0.232	0.000	0.000
EPB41L2	2453.068	-0.657	0.274	0.017	0.050	2453.068	0.166	0.274	0.545	0.740	2453.068	0.823	0.317	0.009	0.062
DMTN	21.935	0.202	0.570	0.723	0.812	21.935	0.409	0.563	0.467	0.691	21.935	0.207	0.654	0.751	0.873
AK2	4990.097	-0.016	0.110	0.883	0.924	4990.097	-0.014	0.110	0.897	0.948	4990.097	0.002	0.127	0.987	0.993
STOM	10.920	1.156	0.483	0.017	0.050	10.920	0.294	0.517	0.570	0.756	10.920	-0.862	0.563	0.126	0.321
EPHA1	11.857	0.787	0.537	0.143	0.255	11.857	-0.218	0.568	0.702	0.839	11.857	-1.004	0.639	0.116	0.305
EPHA3	28.996	-0.558	0.587	0.342	0.483	28.996	0.697	0.557	0.211	0.492	28.996	1.255	0.665	0.059	0.203
EPHA4	91.547	-0.985	0.425	0.021	0.059	91.547	0.471	0.412	0.254	0.527	91.547	1.456	0.487	0.003	0.027
EPHA7	352.108	-0.786	0.313	0.012	0.039	352.108	0.174	0.310	0.576	0.761	352.108	0.960	0.360	0.008	0.055
EPHB2	311.561	-0.400	0.239	0.095	0.188	311.561	-0.411	0.239	0.086	0.378	311.561	-0.011	0.277	0.970	0.985
HIPK1	855.930	-0.706	0.223	0.002	0.008	855.930	-0.358	0.222	0.107	0.399	855.930	0.348	0.258	0.176	0.394
EPHB3	26.262	0.585	0.332	0.078	0.162	26.262	0.270	0.335	0.422	0.658	26.262	-0.316	0.382	0.409	0.636
SLC44A5	270.265	-0.204	0.443	0.645	0.751	270.265	0.669	0.441	0.129	0.421	270.265	0.873	0.511	0.087	0.258
AK4	912.148	0.677	0.265	0.011	0.036	912.148	0.273	0.266	0.304	0.570	912.148	-0.404	0.307	0.188	0.408
EPHB4	1421.533	-0.264	0.183	0.148	0.262	1421.533	0.175	0.182	0.338	0.596	1421.533	0.439	0.211	0.037	0.152
EPHB6	35.348	-0.050	0.340	0.883	0.924	35.348	0.164	0.332	0.621	0.791	35.348	0.215	0.391	0.583	0.766
EPHX1	2534.215	1.059	0.356	0.003	0.013	2534.215	0.439	0.356	0.217	0.496	2534.215	-0.620	0.411	0.131	0.329
MTLN	333.496	0.737	0.332	0.027	0.072	333.496	-0.302	0.335	0.367	0.618	333.496	-1.039	0.385	0.007	0.052
EPHX2	201.281	-0.016	0.345	0.963	0.978	201.281	0.559	0.343	0.103	0.396	201.281	0.574	0.397	0.148	0.356
C2orf69	203.388	-0.058	0.237	0.806	0.872	203.388	-0.423	0.238	0.075	0.364	203.388	-0.365	0.276	0.185	0.405
STX2	489.966	-0.233	0.174	0.181	0.304	489.966	-0.327	0.174	0.059	0.349	489.966	-0.095	0.201	0.638	0.803
DIPK2A	929.583	0.596	0.301	0.048	0.112	929.583	0.322	0.301	0.286	0.555	929.583	-0.274	0.348	0.430	0.653
CLN8	644.888	0.626	0.194	0.001	0.007	644.888	0.111	0.195	0.571	0.757	644.888	-0.515	0.225	0.022	0.108
SENP5	456.225	-0.464	0.170	0.006	0.024	456.225	-0.390	0.169	0.021	0.314	456.225	0.075	0.197	0.705	0.847
EPOR	153.773	0.737	0.305	0.016	0.048	153.773	0.442	0.306	0.149	0.441	153.773	-0.295	0.352	0.402	0.629
USF3	276.647	-0.598	0.224	0.008	0.028	276.647	-0.043	0.221	0.845	0.920	276.647	0.554	0.259	0.032	0.138
EPRS1	5528.881	-1.223	0.191	0.000	0.000	5528.881	-0.324	0.191	0.089	0.382	5528.881	0.899	0.220	0.000	0.001
TRIML2	23.312	1.366	0.477	0.004	0.017	23.312	-0.632	0.533	0.235	0.513	23.312	-1.998	0.587	0.001	0.010
EPS8	1881.170	-0.904	0.283	0.001	0.008	1881.170	-0.335	0.283	0.236	0.513	1881.170	0.569	0.327	0.082	0.247
EPS15	1145.154	-0.275	0.120	0.022	0.061	1145.154	-0.031	0.119	0.792	0.892	1145.154	0.243	0.138	0.078	0.241
NR2F6	2686.215	0.598	0.149	0.000	0.001	2686.215	0.146	0.149	0.328	0.588	2686.215	-0.452	0.172	0.009	0.060
LVRN	19.286	-0.640	0.853	0.453	0.588	19.286	0.914	0.799	0.253	0.527	19.286	1.554	0.961	0.106	0.290
SLC36A1	261.563	0.141	0.308	0.647	0.753	261.563	-0.885	0.311	0.004	0.297	261.563	-1.026	0.359	0.004	0.037
ERBB2	1238.173	0.207	0.195	0.289	0.427	1238.173	0.206	0.195	0.290	0.558	1238.173	-0.001	0.225	0.998	0.999
ERBB3	35.598	0.145	0.302	0.631	0.740	35.598	0.267	0.295	0.366	0.617	35.598	0.122	0.345	0.725	0.858
ERCC1	2259.102	-0.214	0.192	0.266	0.402	2259.102	-0.168	0.192	0.382	0.628	2259.102	0.046	0.222	0.837	0.921
ERCC2	777.282	0.035	0.196	0.859	0.908	777.282	-0.356	0.196	0.070	0.358	777.282	-0.391	0.227	0.085	0.254

EREG	1987.403	-3.225	0.808	0.000	0.001	1987.403	-2.335	0.807	0.004	0.297	1987.403	0.890	0.933	0.340	0.575
AKT1	3450.740	0.176	0.170	0.301	0.439	3450.740	-0.312	0.171	0.067	0.355	3450.740	-0.489	0.197	0.013	0.078
EYA4	118.918	-0.616	0.840	0.464	0.598	118.918	0.553	0.837	0.509	0.718	118.918	1.169	0.969	0.228	0.456
DHRSX	587.465	-0.133	0.149	0.372	0.512	587.465	-0.150	0.148	0.312	0.575	587.465	-0.017	0.172	0.920	0.963
ERCC3	521.367	-0.121	0.123	0.324	0.464	521.367	-0.188	0.122	0.124	0.416	521.367	-0.067	0.142	0.638	0.803
SFTA1P	31.202	-0.347	0.589	0.556	0.678	31.202	-0.673	0.591	0.255	0.528	31.202	-0.326	0.686	0.634	0.801
ERCC4	199.369	-0.555	0.309	0.073	0.154	199.369	-0.823	0.310	0.008	0.297	199.369	-0.268	0.360	0.456	0.674
ERCC5	119.936	-0.465	0.397	0.241	0.374	119.936	0.515	0.391	0.188	0.474	119.936	0.980	0.456	0.031	0.136
ERCC6	302.986	-0.596	0.236	0.011	0.038	302.986	-0.082	0.233	0.726	0.854	302.986	0.514	0.272	0.058	0.201
ERF	2096.790	0.248	0.182	0.174	0.295	2096.790	0.020	0.182	0.914	0.957	2096.790	-0.228	0.210	0.278	0.513
ERG	284.889	-0.078	0.189	0.678	0.776	284.889	-0.217	0.188	0.249	0.523	284.889	-0.139	0.219	0.526	0.726
ERH	2942.673	0.408	0.326	0.211	0.340	2942.673	-0.791	0.326	0.015	0.306	2942.673	-1.199	0.377	0.001	0.017
AKT2	1872.483	-0.114	0.122	0.350	0.490	1872.483	-0.234	0.122	0.055	0.343	1872.483	-0.120	0.141	0.397	0.625
ERN1	358.798	-1.453	0.298	0.000	0.000	358.798	-0.649	0.294	0.028	0.321	358.798	0.805	0.344	0.019	0.099
ERV3-1	25.711	0.911	0.379	0.016	0.049	25.711	0.704	0.380	0.064	0.351	25.711	-0.207	0.431	0.631	0.799
FBL	6928.548	-0.114	0.123	0.357	0.497	6928.548	0.226	0.123	0.067	0.354	6928.548	0.339	0.142	0.017	0.092
ESD	2093.969	0.105	0.145	0.468	0.602	2093.969	0.167	0.145	0.250	0.523	2093.969	0.062	0.168	0.713	0.851
ABCA3	118.132	0.915	0.476	0.054	0.123	118.132	0.982	0.475	0.039	0.327	118.132	0.067	0.547	0.902	0.954
ALAD	927.253	0.580	0.278	0.037	0.092	927.253	0.325	0.278	0.243	0.518	927.253	-0.255	0.321	0.426	0.649
ESR2	15.182	0.038	0.405	0.925	0.953	15.182	-0.142	0.405	0.726	0.854	15.182	-0.180	0.475	0.705	0.847
ESRRA	3019.577	0.164	0.173	0.341	0.482	3019.577	-0.086	0.173	0.619	0.790	3019.577	-0.250	0.199	0.210	0.435
ETF1	4213.031	-1.093	0.259	0.000	0.000	4213.031	-0.572	0.259	0.027	0.319	4213.031	0.521	0.299	0.081	0.247
ETFA	3281.408	-0.338	0.142	0.017	0.051	3281.408	-0.085	0.141	0.549	0.743	3281.408	0.253	0.164	0.122	0.314
ETFB	2149.079	0.836	0.247	0.001	0.004	2149.079	0.210	0.247	0.395	0.640	2149.079	-0.626	0.285	0.028	0.126
ALAS1	1098.922	-0.095	0.190	0.619	0.730	1098.922	-0.266	0.190	0.163	0.453	1098.922	-0.171	0.220	0.437	0.658
ETFDH	381.886	0.039	0.192	0.839	0.894	381.886	-0.357	0.193	0.064	0.353	381.886	-0.396	0.223	0.076	0.237
ETS1	3247.741	-0.635	0.330	0.054	0.123	3247.741	-0.749	0.330	0.023	0.316	3247.741	-0.113	0.382	0.766	0.881
ETS2	6568.746	0.783	0.218	0.000	0.002	6568.746	0.343	0.218	0.115	0.406	6568.746	-0.440	0.251	0.080	0.245
ETV1	606.005	-1.031	0.276	0.000	0.002	606.005	0.216	0.274	0.430	0.664	606.005	1.248	0.318	0.000	0.002
ETV2	12.417	0.697	0.664	0.294	0.432	12.417	1.291	0.639	0.043	0.331	12.417	0.594	0.736	0.420	0.644
ETV3	579.400	-0.122	0.211	0.563	0.684	579.400	0.061	0.210	0.770	0.878	579.400	0.183	0.243	0.451	0.670
ETV4	626.736	-0.679	0.389	0.081	0.167	626.736	-0.514	0.389	0.186	0.473	626.736	0.165	0.450	0.713	0.851
ETV5	986.742	-1.514	0.392	0.000	0.001	986.742	-0.631	0.391	0.106	0.398	986.742	0.882	0.452	0.051	0.186
ETV6	383.929	0.061	0.247	0.804	0.870	383.929	-0.183	0.247	0.460	0.686	383.929	-0.244	0.286	0.393	0.621
EVC	559.271	-0.493	0.353	0.163	0.282	559.271	0.258	0.352	0.464	0.689	559.271	0.751	0.408	0.066	0.216
MECOM	603.773	-0.875	0.215	0.000	0.001	603.773	-0.068	0.213	0.748	0.866	603.773	0.807	0.248	0.001	0.015
EVI2A	36.049	-0.217	0.405	0.592	0.707	36.049	-0.702	0.413	0.089	0.383	36.049	-0.485	0.482	0.314	0.549
EVI2B	36.332	-0.002	0.422	0.997	0.999	36.332	-0.146	0.421	0.729	0.855	36.332	-0.144	0.491	0.770	0.883
EVPL	15.924	0.802	0.399	0.044	0.105	15.924	0.250	0.413	0.545	0.739	15.924	-0.552	0.463	0.233	0.462
EWSR1	4084.433	-0.256	0.263	0.330	0.470	4084.433	-0.523	0.263	0.047	0.335	4084.433	-0.267	0.303	0.379	0.610
EXT1	698.754	0.136	0.097	0.162	0.281	698.754	-0.205	0.097	0.035	0.323	698.754	-0.341	0.113	0.003	0.026
EXT2	1905.911	0.017	0.179	0.925	0.953	1905.911	-0.221	0.179	0.217	0.496	1905.911	-0.238	0.207	0.250	0.482
EXTL2	306.673	-0.672	0.157	0.000	0.000	306.673	-0.214	0.153	0.164	0.454	306.673	0.458	0.181	0.011	0.071
EXTL3	588.601	-0.212	0.255	0.406	0.544	588.601	-0.617	0.255	0.016	0.306	588.601	-0.405	0.295	0.170	0.385
EYA1	62.588	-0.238	0.673	0.723	0.812	62.588	0.296	0.668	0.658	0.813	62.588	0.534	0.775	0.491	0.701
EYA2	27.488	-0.527	0.411	0.200	0.326	27.488	-0.187	0.396	0.637	0.802	27.488	0.340	0.475	0.474	0.688
ALCAM	229.708	-0.934	0.247	0.000	0.001	229.708	-0.633	0.245	0.010	0.298	229.708	0.301	0.286	0.293	0.528
EYA3	387.758	-0.048	0.138	0.727	0.814	387.758	0.063	0.137	0.645	0.806	387.758	0.111	0.160	0.485	0.696
EZH1	321.961	-0.068	0.193	0.726	0.814	321.961	0.333	0.191	0.082	0.373	321.961	0.401	0.223	0.072	0.229
EZH2	545.601	-0.913	0.227	0.000	0.001	545.601	-0.492	0.226	0.029	0.322	545.601	0.421	0.262	0.108	0.294
F2R	909.810	-0.273	0.315	0.386	0.525	909.810	0.364	0.314	0.246	0.521	909.810	0.637	0.363	0.079	0.243
ABCD1	561.638	0.511	0.480	0.287	0.425	561.638	0.760	0.479	0.113	0.405	561.638	0.249	0.553	0.652	0.813
F2RL1	513.002	-0.019	0.165	0.908	0.941	513.002	-0.277	0.166	0.095	0.389	513.002	-0.258	0.192	0.179	0.397
F2RL2	5.932	0.206	0.786	0.793	0.863	5.932	-1.771	0.903	0.050	0.337	5.932	-1.977	1.013	0.051	0.185
F3	953.162	0.652	0.389	0.094	0.187	953.162	-0.671	0.390	0.085	0.377	953.162	-1.323	0.450	0.003	0.030
F8	133.255	0.290	0.326	0.373	0.513	133.255	0.157	0.326	0.630	0.798	133.255	-0.134	0.376	0.723	0.856
ALDH1A1	14686.868	0.737	0.427	0.085	0.173	14686.868	-1.060	0.427	0.013	0.305	14686.868	-1.797	0.493	0.000	0.005
F12	78.649	1.062	0.287	0.000	0.002	78.649	0.338	0.294	0.251	0.525	78.649	-0.724	0.332	0.029	0.130
FAAH	23.567	1.673	0.476	0.000	0.003	23.567	0.979	0.486	0.044	0.331	23.567	-0.694	0.539	0.198	0.421
ALDH2	47.872	0.488	0.277	0.078	0.162	47.872	0.312	0.277	0.259	0.531	47.872	-0.175	0.318	0.581	0.764
FABP3	10.485	0.095	0.557	0.864	0.912	10.485	-0.196	0.561	0.727	0.854	10.485	-0.291	0.652	0.656	0.816
FABP5	1563.954	0.368	0.258	0.154	0.270	1563.954	-0.325	0.259	0.209	0.490	1563.954	-0.693	0.299	0.020	0.102
FABP6	10.571	1.157	0.634	0.068	0.146	10.571	0.427	0.653	0.513	0.720	10.571	-0.729	0.731	0.319	0.554
FANCA	480.048	-0.020	0.302	0.948	0.968	480.048	-0.510	0.303	0.092	0.387	480.048	-0.490	0.350	0.161	0.375
FANCC	307.247	-0.458	0.191	0.017	0.050	307.247	-0.101	0.189	0.594	0.774	307.247	0.357	0.221	0.106	0.289
FANCD2	622.192	-1.021	0.345	0.003	0.014	622.192	-0.765	0.344	0.026	0.318	622.192	0.256	0.398	0.520	0.723
FANCE	1020.706	-0.836	0.251	0.001	0.005	1020.706	0.374	0.250	0.134	0.426	1020.706	1.211	0.289	0.000	0.001

ALDH3A1	13.455	0.245	0.547	0.654	0.758	13.455	0.113	0.546	0.836	0.916	13.455	-0.132	0.633	0.834	0.919
ACSL1	6999.236	0.512	0.212	0.016	0.048	6999.236	0.370	0.212	0.081	0.372	6999.236	-0.142	0.245	0.563	0.754
ACSL3	1841.560	-0.332	0.251	0.187	0.311	1841.560	-0.592	0.251	0.018	0.306	1841.560	-0.261	0.290	0.369	0.601
ACSL4	2742.759	-0.056	0.197	0.777	0.852	2742.759	-0.067	0.197	0.734	0.858	2742.759	-0.011	0.227	0.961	0.982
FAH	407.028	0.216	0.133	0.103	0.200	407.028	0.287	0.132	0.029	0.322	407.028	0.071	0.153	0.642	0.805
PTK2B	1456.861	-0.118	0.307	0.701	0.794	1456.861	0.551	0.307	0.072	0.361	1456.861	0.669	0.354	0.059	0.203
BPTF	1021.224	-0.263	0.212	0.214	0.344	1021.224	0.030	0.211	0.887	0.944	1021.224	0.293	0.245	0.231	0.459
FANCB	50.866	-1.108	0.365	0.002	0.011	50.866	-0.612	0.354	0.084	0.376	50.866	0.495	0.423	0.242	0.473
FANCF	341.068	-0.134	0.150	0.372	0.512	341.068	0.115	0.148	0.439	0.672	341.068	0.249	0.173	0.151	0.360
FANCG	410.895	-0.440	0.155	0.005	0.018	410.895	0.106	0.152	0.488	0.705	410.895	0.545	0.178	0.002	0.023
ALDH1B1	829.336	-0.215	0.188	0.253	0.387	829.336	-0.264	0.188	0.160	0.451	829.336	-0.049	0.217	0.823	0.913
FBLN1	7753.272	0.791	0.310	0.011	0.036	7753.272	0.426	0.310	0.169	0.458	7753.272	-0.365	0.357	0.307	0.543
SAMD9L	93.698	0.887	0.336	0.008	0.029	93.698	0.308	0.338	0.362	0.614	93.698	-0.578	0.388	0.136	0.337
ATAD3C	15.253	-2.324	1.689	0.169	0.289	15.253	-3.604	1.730	0.037	0.326	15.253	-1.280	2.003	0.523	0.725
FARSA	3157.408	-0.681	0.251	0.007	0.025	3157.408	-0.365	0.251	0.146	0.438	3157.408	0.316	0.290	0.275	0.510
USP12	423.911	-0.677	0.246	0.006	0.023	423.911	-0.462	0.245	0.059	0.349	423.911	0.214	0.285	0.452	0.671
TMEM254-AS1	13.480	-0.087	0.519	0.867	0.914	13.480	-0.062	0.513	0.904	0.952	13.480	0.025	0.602	0.967	0.984
FASN	17241.651	1.332	0.151	0.000	0.000	17241.651	0.059	0.151	0.695	0.836	17241.651	-1.273	0.174	0.000	0.000
MTIF3	422.064	0.438	0.201	0.029	0.077	422.064	0.087	0.201	0.666	0.818	422.064	-0.351	0.232	0.131	0.329
FAT1	2397.688	-0.153	0.165	0.355	0.495	2397.688	-0.276	0.165	0.094	0.388	2397.688	-0.123	0.191	0.518	0.721
YPEL4	6.154	0.966	1.057	0.361	0.501	6.154	1.154	1.047	0.270	0.542	6.154	0.188	1.199	0.875	0.940
MED19	238.586	-0.017	0.222	0.939	0.962	238.586	-0.292	0.223	0.190	0.475	238.586	-0.275	0.258	0.286	0.521
FAT2	97.664	-0.099	0.701	0.887	0.927	97.664	0.596	0.698	0.393	0.637	97.664	0.695	0.808	0.389	0.618
ZCCHC24	203.234	1.237	0.222	0.000	0.000	203.234	0.281	0.226	0.214	0.493	203.234	-0.956	0.257	0.000	0.004
ENKUR	284.447	-0.285	0.223	0.201	0.328	284.447	-0.069	0.222	0.755	0.869	284.447	0.216	0.258	0.402	0.629
UNC5B	1663.491	-3.340	0.197	0.000	0.000	1663.491	0.048	0.189	0.798	0.895	1663.491	3.389	0.225	0.000	0.000
FAU	5802.037	0.593	0.214	0.005	0.021	5802.037	0.258	0.214	0.228	0.506	5802.037	-0.336	0.247	0.174	0.390
PROSER2-AS1	79.794	-0.708	0.276	0.010	0.035	79.794	-0.377	0.270	0.162	0.453	79.794	0.331	0.320	0.302	0.537
STOX1	10.901	0.919	0.735	0.211	0.341	10.901	-0.321	0.763	0.674	0.824	10.901	-1.239	0.866	0.152	0.361
TYSND1	645.130	0.368	0.197	0.062	0.136	645.130	0.402	0.197	0.041	0.329	645.130	0.034	0.227	0.882	0.943
ZNF25	137.453	-0.717	0.299	0.017	0.050	137.453	0.252	0.292	0.388	0.635	137.453	0.969	0.344	0.005	0.040
CCNY	2070.752	-0.449	0.260	0.084	0.171	2070.752	0.336	0.259	0.194	0.478	2070.752	0.785	0.300	0.009	0.060
RTKN2	33.650	0.537	0.363	0.139	0.250	33.650	0.413	0.362	0.254	0.527	33.650	-0.124	0.416	0.767	0.881
C11orf45	39.776	-0.313	0.369	0.396	0.535	39.776	0.398	0.355	0.261	0.534	39.776	0.712	0.421	0.091	0.264
HYLS1	41.323	0.289	0.277	0.297	0.435	41.323	-0.458	0.290	0.114	0.405	41.323	-0.748	0.332	0.024	0.115
TMEM218	227.149	-0.299	0.191	0.117	0.220	227.149	-0.132	0.189	0.484	0.702	227.149	0.167	0.221	0.450	0.669
SLC37A2	230.463	0.261	0.166	0.117	0.220	230.463	0.261	0.165	0.114	0.406	230.463	0.001	0.192	0.997	0.998
TBCEL	624.238	-0.742	0.188	0.000	0.001	624.238	-0.201	0.186	0.280	0.549	624.238	0.541	0.217	0.013	0.076
TLCD5	122.053	0.050	0.237	0.832	0.889	122.053	0.232	0.235	0.322	0.583	122.053	0.182	0.273	0.505	0.712
MRPL21	791.151	0.176	0.221	0.425	0.562	791.151	-0.196	0.221	0.377	0.624	791.151	-0.372	0.256	0.146	0.352
TPCN2	142.383	-0.349	0.193	0.071	0.151	142.383	-0.233	0.190	0.222	0.501	142.383	0.116	0.224	0.604	0.781
GLYATL2	8.069	0.583	0.596	0.328	0.468	8.069	0.820	0.580	0.157	0.450	8.069	0.237	0.668	0.723	0.856
PATL1	1485.307	0.177	0.191	0.355	0.496	1485.307	-0.005	0.191	0.977	0.987	1485.307	-0.182	0.221	0.410	0.636
ABCB7	935.991	-0.470	0.262	0.073	0.155	935.991	0.402	0.261	0.124	0.416	935.991	0.872	0.303	0.004	0.035
ALDH1A3	10.849	-0.442	0.530	0.405	0.543	10.849	-0.045	0.506	0.929	0.964	10.849	0.397	0.610	0.515	0.719
FBN1	31.368	1.102	0.381	0.004	0.016	31.368	0.125	0.395	0.752	0.867	31.368	-0.977	0.445	0.028	0.126
VWCE	9.886	2.266	0.719	0.002	0.008	9.886	1.225	0.755	0.104	0.397	9.886	-1.041	0.791	0.188	0.409
CYB561A3	1814.806	-0.584	0.245	0.017	0.051	1814.806	0.221	0.244	0.366	0.617	1814.806	0.805	0.283	0.004	0.038
PPP1R32	11.467	2.121	1.030	0.039	0.096	11.467	1.386	1.041	0.183	0.470	11.467	-0.736	1.168	0.529	0.729
DDIAS	157.726	-1.320	0.500	0.008	0.029	157.726	-1.106	0.498	0.026	0.318	157.726	0.214	0.579	0.711	0.850
LTO1	1351.250	0.545	0.189	0.004	0.017	1351.250	0.062	0.190	0.743	0.863	1351.250	-0.483	0.219	0.027	0.124
LRTOMT	421.477	0.234	0.220	0.287	0.425	421.477	-0.067	0.220	0.761	0.873	421.477	-0.301	0.254	0.237	0.466
ERICH6B	26.614	-0.259	0.411	0.528	0.655	26.614	0.232	0.393	0.554	0.746	26.614	0.491	0.469	0.295	0.530
FAM124A	7.411	1.111	0.852	0.193	0.318	7.411	-1.235	1.027	0.229	0.507	7.411	-2.346	1.119	0.036	0.148
SKA1	439.007	-0.887	0.403	0.028	0.074	439.007	-0.627	0.403	0.119	0.412	439.007	0.260	0.466	0.577	0.762
CFAP53	38.108	0.865	0.438	0.048	0.112	38.108	1.057	0.434	0.015	0.306	38.108	0.192	0.497	0.700	0.843
EFEMP1	2355.010	0.329	0.154	0.033	0.085	2355.010	0.230	0.154	0.136	0.427	2355.010	-0.099	0.178	0.579	0.764
OTUD1	212.348	0.020	0.176	0.910	0.943	212.348	0.321	0.172	0.062	0.350	212.348	0.301	0.201	0.135	0.335
OAF	2054.862	0.717	0.242	0.003	0.013	2054.862	-0.159	0.242	0.511	0.719	2054.862	-0.876	0.279	0.002	0.019
FCAR	2991.164	-0.205	0.117	0.080	0.166	2991.164	0.170	0.117	0.145	0.437	2991.164	0.375	0.135	0.006	0.044
RNF152	43.107	-1.053	0.354	0.003	0.013	43.107	-0.059	0.332	0.860	0.929	43.107	0.995	0.404	0.014	0.081
USP32P2	49.663	1.693	0.558	0.002	0.011	49.663	0.591	0.564	0.295	0.562	49.663	-1.102	0.644	0.087	0.258
LOC220729	158.993	0.058	0.177	0.743	0.827	158.993	0.004	0.176	0.980	0.989	158.993	-0.054	0.205	0.794	0.896
CBWD5	738.698	-0.211	0.340	0.535	0.660	738.698	-0.715	0.341	0.036	0.324	738.698	-0.504	0.394	0.201	0.424
WAC-AS1	441.395	0.031	0.118	0.795	0.865	441.395	-0.017	0.118	0.884	0.942	441.395	-0.048	0.137	0.726	0.858
ZNF438	197.346	0.020	0.177	0.911	0.943	197.346	0.090	0.175	0.608	0.783	197.346	0.070	0.204	0.732	0.861
ZEB1-AS1	155.120	-0.596	0.177	0.001	0.005	155.120	0.008	0.170	0.965	0.981	155.120	0.603	0.203	0.003	0.029

SLC16A9	44.835	-1.186	0.399	0.003	0.013	44.835	-1.060	0.393	0.007	0.297	44.835	0.126	0.468	0.787	0.894
MARCHF8	836.350	-0.159	0.260	0.541	0.665	836.350	0.052	0.259	0.840	0.917	836.350	0.211	0.300	0.481	0.694
C10orf25	12.003	0.304	0.625	0.627	0.737	12.003	0.699	0.604	0.247	0.522	12.003	0.395	0.705	0.575	0.761
HNRNPA3	9676.710	-0.361	0.192	0.061	0.134	9676.710	-0.457	0.192	0.017	0.306	9676.710	-0.096	0.222	0.665	0.821
ALDH3B1	1464.259	0.090	0.538	0.868	0.914	1464.259	0.766	0.537	0.154	0.447	1464.259	0.676	0.621	0.276	0.511
REEP3	1160.479	0.052	0.155	0.737	0.823	1160.479	-0.106	0.155	0.495	0.709	1160.479	-0.158	0.179	0.379	0.609
JMJD1C	967.488	-1.005	0.184	0.000	0.000	967.488	-0.194	0.182	0.288	0.557	967.488	0.811	0.212	0.000	0.003
RPP38-DT	7.801	-1.348	0.686	0.049	0.114	7.801	-0.459	0.610	0.452	0.680	7.801	0.889	0.784	0.257	0.491
FAM171A1	763.473	-0.679	0.229	0.003	0.014	763.473	-0.445	0.229	0.052	0.339	763.473	0.234	0.265	0.377	0.609
NSUN6	140.615	-0.855	0.314	0.006	0.024	140.615	-0.811	0.312	0.009	0.297	140.615	0.044	0.364	0.904	0.955
ARL5B	422.656	-0.493	0.273	0.070	0.150	422.656	0.016	0.271	0.954	0.978	422.656	0.509	0.314	0.106	0.289
HNRNPUL2	1536.633	0.024	0.129	0.856	0.906	1536.633	-0.095	0.129	0.463	0.689	1536.633	-0.118	0.150	0.429	0.651
ALKBH3	284.736	-0.749	0.216	0.001	0.003	284.736	-0.191	0.212	0.366	0.617	284.736	0.557	0.249	0.025	0.117
EEF1AKMT1	97.536	0.002	0.209	0.991	0.995	97.536	-0.221	0.210	0.293	0.561	97.536	-0.223	0.244	0.362	0.594
SKA3	325.520	-0.958	0.264	0.000	0.002	325.520	-0.377	0.261	0.149	0.441	325.520	0.580	0.305	0.057	0.198
MICU2	396.758	-0.598	0.159	0.000	0.001	396.758	-0.282	0.157	0.072	0.361	396.758	0.316	0.184	0.086	0.255
SPATA13	94.772	-1.122	0.355	0.002	0.008	94.772	-0.839	0.350	0.017	0.306	94.772	0.283	0.413	0.493	0.702
CPNE2	495.309	0.506	0.233	0.030	0.078	495.309	0.306	0.233	0.189	0.475	495.309	-0.200	0.269	0.456	0.674
PRSS54	17.997	-0.145	0.437	0.740	0.824	17.997	0.255	0.421	0.545	0.739	17.997	0.400	0.499	0.423	0.647
FCGR2A	51.961	2.014	0.553	0.000	0.002	51.961	0.937	0.560	0.094	0.388	51.961	-1.077	0.635	0.090	0.262
CCDC162P	49.337	0.088	0.322	0.784	0.857	49.337	-0.032	0.322	0.920	0.960	49.337	-0.120	0.374	0.747	0.870
AK9	117.272	-0.311	0.328	0.343	0.483	117.272	0.358	0.322	0.267	0.538	117.272	0.669	0.377	0.076	0.237
NT5DC1	859.639	0.047	0.255	0.854	0.905	859.639	0.293	0.255	0.250	0.523	859.639	0.246	0.294	0.403	0.630
ZUP1	195.737	-0.294	0.145	0.043	0.102	195.737	-0.147	0.142	0.301	0.567	195.737	0.147	0.168	0.381	0.611
TBC1D32	37.350	-0.292	0.266	0.272	0.409	37.350	0.175	0.250	0.483	0.701	37.350	0.467	0.302	0.122	0.315
BEND6	106.641	0.305	0.290	0.293	0.431	106.641	0.224	0.289	0.439	0.672	106.641	-0.081	0.334	0.808	0.904
ADGRF4	21.861	0.437	0.600	0.467	0.601	21.861	0.804	0.590	0.173	0.462	21.861	0.368	0.683	0.590	0.771
ADGRF5	689.557	-0.909	0.290	0.002	0.009	689.557	-0.029	0.288	0.921	0.960	689.557	0.880	0.334	0.008	0.059
RSPH9	289.122	-0.086	0.256	0.737	0.823	289.122	-0.054	0.255	0.832	0.913	289.122	0.032	0.295	0.914	0.960
LRRC73	10.707	0.153	0.591	0.796	0.866	10.707	0.244	0.577	0.673	0.823	10.707	0.091	0.677	0.893	0.949
ADCY10P1	15.657	1.406	0.509	0.006	0.022	15.657	0.942	0.516	0.068	0.357	15.657	-0.465	0.575	0.418	0.643
OARD1	492.466	-0.125	0.209	0.551	0.674	492.466	0.199	0.208	0.338	0.596	492.466	0.324	0.241	0.179	0.398
TMEM217	48.536	-0.418	0.339	0.217	0.347	48.536	-0.196	0.332	0.555	0.746	48.536	0.222	0.392	0.571	0.759
C6orf89	1322.975	-0.239	0.157	0.128	0.235	1322.975	-0.039	0.157	0.802	0.896	1322.975	0.200	0.182	0.271	0.506
SMIM29	437.414	1.000	0.352	0.005	0.018	437.414	-0.544	0.355	0.125	0.416	437.414	-1.544	0.408	0.000	0.003
LEMD2	1106.592	0.058	0.155	0.708	0.799	1106.592	0.038	0.155	0.804	0.897	1106.592	-0.020	0.179	0.912	0.960
ZBTB9	334.830	-0.365	0.319	0.251	0.385	334.830	-0.577	0.319	0.070	0.359	334.830	-0.211	0.369	0.567	0.757
ZBTB12	1192.797	0.304	0.411	0.460	0.594	1192.797	0.678	0.411	0.099	0.393	1192.797	0.374	0.474	0.430	0.653
C6orf136	869.118	0.458	0.240	0.056	0.126	869.118	-0.041	0.241	0.863	0.930	869.118	-0.499	0.278	0.072	0.229
ZSCAN12P1	19.177	0.942	0.451	0.037	0.091	19.177	0.482	0.457	0.292	0.559	19.177	-0.460	0.518	0.375	0.607
KDM1B	498.597	-0.523	0.262	0.046	0.109	498.597	-0.369	0.262	0.158	0.451	498.597	0.154	0.303	0.611	0.786
PHACTR1	110.955	0.596	0.304	0.050	0.115	110.955	-0.514	0.312	0.099	0.394	110.955	-1.110	0.357	0.002	0.021
FCGRT	8.202	2.041	0.727	0.005	0.020	8.202	1.902	0.726	0.009	0.297	8.202	-0.139	0.793	0.861	0.933
SMIM13	426.641	-0.891	0.317	0.005	0.020	426.641	-0.761	0.316	0.016	0.306	426.641	0.130	0.367	0.724	0.857
PXDC1	870.882	0.671	0.197	0.001	0.004	870.882	-0.160	0.198	0.418	0.656	870.882	-0.832	0.228	0.000	0.005
ZSCAN25	186.053	-0.479	0.194	0.014	0.043	186.053	-0.311	0.191	0.104	0.397	186.053	0.168	0.225	0.455	0.673
FAM200A	104.250	0.165	0.245	0.500	0.630	104.250	-0.154	0.247	0.532	0.732	104.250	-0.320	0.285	0.262	0.496
FKTN	492.391	-0.679	0.276	0.014	0.044	492.391	-0.159	0.275	0.565	0.752	492.391	0.520	0.319	0.103	0.286
VWDE	116.967	-0.065	0.445	0.883	0.924	116.967	0.743	0.441	0.092	0.387	116.967	0.808	0.511	0.114	0.302
TWISTNB	508.237	-0.529	0.240	0.027	0.073	508.237	-0.555	0.240	0.021	0.312	508.237	-0.025	0.278	0.928	0.967
SP8	122.965	1.109	0.387	0.004	0.017	122.965	-1.089	0.428	0.011	0.302	122.965	-2.198	0.478	0.000	0.000
HOXA11-AS	17.626	0.662	0.433	0.126	0.232	17.626	0.291	0.438	0.506	0.716	17.626	-0.371	0.499	0.457	0.675
JAZF1	77.327	0.222	0.229	0.331	0.472	77.327	-0.021	0.230	0.927	0.963	77.327	-0.244	0.266	0.360	0.593
PPP1R35	440.735	0.361	0.347	0.299	0.437	440.735	0.183	0.347	0.597	0.776	440.735	-0.177	0.401	0.658	0.817
GPC2	8.797	0.437	0.523	0.404	0.542	8.797	-0.157	0.549	0.775	0.881	8.797	-0.594	0.624	0.341	0.576
BRAT1	1335.239	0.470	0.154	0.002	0.011	1335.239	0.185	0.154	0.231	0.508	1335.239	-0.285	0.178	0.109	0.294
FOXK1	1934.171	0.467	0.167	0.005	0.020	1934.171	0.398	0.166	0.017	0.306	1934.171	-0.068	0.192	0.722	0.856
DAGLB	997.859	-0.299	0.135	0.027	0.073	997.859	0.226	0.134	0.091	0.385	997.859	0.525	0.156	0.001	0.011
CCZ1B	1812.612	-0.200	0.234	0.393	0.532	1812.612	-0.553	0.234	0.018	0.306	1812.612	-0.353	0.270	0.192	0.414
TMED4	1005.011	-0.028	0.203	0.889	0.928	1005.011	0.421	0.202	0.037	0.325	1005.011	0.449	0.234	0.055	0.194
DKFZP586I1420	15.885	1.118	0.491	0.023	0.064	15.885	-0.397	0.533	0.456	0.682	15.885	-1.515	0.593	0.011	0.068
MTURN	711.691	0.068	0.149	0.650	0.755	711.691	-0.044	0.149	0.768	0.877	711.691	-0.112	0.172	0.517	0.721
SRRM3	61.480	0.438	0.262	0.095	0.189	61.480	0.153	0.264	0.561	0.750	61.480	-0.284	0.303	0.348	0.582
RSBN1L	372.171	0.301	0.196	0.125	0.230	372.171	0.004	0.196	0.984	0.991	372.171	-0.297	0.227	0.190	0.412
FDFT1	2922.552	1.921	0.222	0.000	0.000	2922.552	-0.499	0.223	0.026	0.316	2922.552	-2.419	0.257	0.000	0.000
KIAA1324L	341.344	-0.942	0.221	0.000	0.000	341.344	0.191	0.216	0.375	0.623	341.344	1.134	0.253	0.000	0.000
LRWD1	266.250	-0.437	0.407	0.284	0.422	266.250	-0.801	0.408	0.050	0.337	266.250	-0.364	0.472	0.440	0.661

FAM185A	49.101	-0.435	0.234	0.063	0.138	49.101	-0.349	0.227	0.124	0.416	49.101	0.085	0.274	0.755	0.875
FBXL13	186.750	-1.735	0.305	0.000	0.000	186.750	-0.337	0.293	0.250	0.524	186.750	1.398	0.349	0.000	0.002
NAPEPLD	262.916	-0.130	0.210	0.535	0.660	262.916	0.062	0.208	0.767	0.877	262.916	0.192	0.242	0.428	0.651
ATXN7L1	41.987	0.137	0.365	0.708	0.799	41.987	0.459	0.358	0.200	0.483	41.987	0.322	0.417	0.440	0.661
BEND7	82.615	-0.088	0.186	0.637	0.744	82.615	-0.204	0.185	0.269	0.541	82.615	-0.116	0.217	0.592	0.772
FDPS	3064.802	1.379	0.186	0.000	0.000	3064.802	-0.310	0.187	0.097	0.391	3064.802	-1.689	0.215	0.000	0.000
LNX2	284.026	-1.026	0.201	0.000	0.000	284.026	-0.137	0.195	0.481	0.700	284.026	0.889	0.231	0.000	0.003
FAM83B	115.994	-0.483	0.313	0.123	0.228	115.994	-0.416	0.311	0.182	0.469	115.994	0.067	0.363	0.853	0.930
UNC5CL	8.972	1.417	0.736	0.054	0.123	8.972	1.484	0.730	0.042	0.330	8.972	0.067	0.819	0.934	0.970
KCTD20	2031.526	-0.368	0.141	0.009	0.032	2031.526	-0.272	0.141	0.053	0.342	2031.526	0.096	0.163	0.556	0.748
LHFPL5	904.074	-0.858	0.253	0.001	0.004	904.074	-0.530	0.252	0.036	0.324	904.074	0.329	0.292	0.261	0.495
ZSCAN23	20.134	-0.695	0.713	0.330	0.470	20.134	0.314	0.693	0.651	0.810	20.134	1.008	0.817	0.217	0.443
TOB2P1	15.716	0.115	0.521	0.826	0.886	15.716	0.605	0.502	0.228	0.506	15.716	0.490	0.590	0.406	0.633
RPL23P8	33.677	0.313	0.347	0.368	0.508	33.677	0.167	0.346	0.630	0.798	33.677	-0.146	0.401	0.715	0.852
NYAP1	114.113	1.043	0.616	0.091	0.182	114.113	0.845	0.616	0.170	0.460	114.113	-0.197	0.710	0.781	0.890
SLC29A4	34.261	1.532	0.452	0.001	0.004	34.261	0.885	0.460	0.054	0.342	34.261	-0.647	0.513	0.208	0.432
RSPH10B	388.836	0.496	0.299	0.098	0.193	388.836	-0.337	0.301	0.263	0.535	388.836	-0.833	0.347	0.016	0.090
ALDH9A1	1753.442	-0.170	0.173	0.327	0.467	1753.442	0.169	0.173	0.328	0.588	1753.442	0.339	0.200	0.090	0.263
FDX1	229.555	-0.009	0.244	0.970	0.981	229.555	-0.493	0.245	0.045	0.333	229.555	-0.483	0.284	0.088	0.259
ZNRF2	211.986	0.400	0.157	0.011	0.036	211.986	-0.020	0.159	0.898	0.948	211.986	-0.421	0.183	0.021	0.105
FDXR	535.849	-0.318	0.242	0.188	0.312	535.849	0.300	0.241	0.213	0.493	535.849	0.618	0.279	0.027	0.122
FECH	497.817	-0.858	0.272	0.002	0.008	497.817	-0.220	0.270	0.417	0.655	497.817	0.638	0.314	0.042	0.164
FEN1	604.084	-0.196	0.455	0.668	0.768	604.084	-1.435	0.457	0.002	0.297	604.084	-1.239	0.527	0.019	0.098
ALDH3A2	1356.891	0.054	0.186	0.770	0.848	1356.891	-0.049	0.186	0.794	0.892	1356.891	-0.103	0.215	0.632	0.800
FER	499.780	-0.803	0.244	0.001	0.006	499.780	-0.391	0.242	0.106	0.399	499.780	0.412	0.281	0.143	0.349
FGD1	498.462	0.411	0.163	0.012	0.038	498.462	0.346	0.163	0.033	0.322	498.462	-0.065	0.188	0.729	0.859
FGF2	927.933	-0.357	0.349	0.306	0.446	927.933	-0.887	0.349	0.011	0.302	927.933	-0.531	0.403	0.188	0.408
ABCD2	37.232	-0.369	0.429	0.389	0.528	37.232	0.601	0.413	0.146	0.438	37.232	0.970	0.488	0.047	0.175
FGF11	42.476	0.161	0.432	0.710	0.801	42.476	0.395	0.426	0.354	0.608	42.476	0.234	0.495	0.637	0.803
FGF12	117.950	-0.149	0.400	0.710	0.802	117.950	0.636	0.396	0.108	0.401	117.950	0.785	0.460	0.088	0.259
FGF13	25.410	0.709	0.334	0.034	0.086	25.410	0.228	0.342	0.506	0.716	25.410	-0.481	0.387	0.214	0.439
ALDOA	11040.778	-0.143	0.379	0.706	0.798	11040.778	0.528	0.379	0.163	0.454	11040.778	0.671	0.438	0.125	0.319
FGFR1	1298.409	0.029	0.172	0.868	0.915	1298.409	-0.083	0.172	0.630	0.797	1298.409	-0.111	0.199	0.575	0.761
FGFR3	16.337	0.314	0.519	0.545	0.669	16.337	0.009	0.523	0.987	0.992	16.337	-0.305	0.603	0.613	0.787
FH	1678.094	-0.192	0.284	0.500	0.631	1678.094	-0.433	0.285	0.128	0.420	1678.094	-0.241	0.329	0.463	0.679
FHIT	66.031	0.793	0.360	0.028	0.073	66.031	0.244	0.363	0.502	0.714	66.031	-0.549	0.416	0.187	0.407
FHL1	12.362	0.419	0.598	0.484	0.617	12.362	0.298	0.596	0.617	0.789	12.362	-0.121	0.688	0.861	0.933
FHL2	527.458	-0.479	0.258	0.063	0.138	527.458	-0.483	0.258	0.061	0.350	527.458	-0.004	0.299	0.989	0.994
FHL3	579.155	0.344	0.197	0.081	0.167	579.155	0.112	0.198	0.571	0.757	579.155	-0.233	0.228	0.308	0.543
CASC3	2003.974	-0.778	0.175	0.000	0.000	2003.974	-0.011	0.174	0.950	0.975	2003.974	0.767	0.201	0.000	0.003
NID2	9.310	-0.755	0.576	0.189	0.314	9.310	-0.177	0.530	0.738	0.860	9.310	0.579	0.660	0.381	0.610
COG2	385.475	-0.652	0.237	0.006	0.023	385.475	-0.428	0.236	0.069	0.358	385.475	0.224	0.274	0.414	0.640
FKBP1A	3341.264	0.329	0.130	0.011	0.038	3341.264	-0.148	0.130	0.257	0.529	3341.264	-0.477	0.150	0.002	0.018
RRAS2	1627.415	-0.542	0.184	0.003	0.014	1627.415	-0.419	0.184	0.023	0.315	1627.415	0.123	0.213	0.563	0.754
XRN2	1963.936	-0.809	0.213	0.000	0.001	1963.936	-0.223	0.213	0.294	0.561	1963.936	0.586	0.246	0.017	0.093
IKZF2	204.682	-0.588	0.375	0.117	0.220	204.682	-0.655	0.375	0.081	0.372	204.682	-0.067	0.435	0.877	0.941
MRAS	519.176	-0.040	0.179	0.824	0.885	519.176	-0.192	0.179	0.283	0.552	519.176	-0.153	0.208	0.462	0.679
ATF5	514.773	0.064	0.131	0.626	0.736	514.773	-0.013	0.130	0.918	0.959	514.773	-0.077	0.151	0.610	0.785
COPZ1	2528.798	-0.158	0.095	0.096	0.190	2528.798	-0.006	0.095	0.948	0.974	2528.798	0.152	0.110	0.166	0.381
COPG1	2782.775	0.342	0.135	0.012	0.038	2782.775	-0.094	0.136	0.489	0.705	2782.775	-0.435	0.157	0.005	0.043
RASA3	1268.043	0.050	0.154	0.747	0.830	1268.043	0.227	0.153	0.139	0.430	1268.043	0.177	0.177	0.318	0.554
PHLDA1	1496.800	-2.461	0.747	0.001	0.006	1496.800	-2.099	0.746	0.005	0.297	1496.800	0.362	0.862	0.675	0.828
MTF2	307.151	-0.109	0.211	0.605	0.718	307.151	-0.347	0.211	0.100	0.394	307.151	-0.238	0.244	0.331	0.565
HSPA4L	578.217	-0.814	0.195	0.000	0.000	578.217	-0.384	0.193	0.047	0.336	578.217	0.430	0.225	0.056	0.196
DNAJC8	2107.371	0.152	0.223	0.495	0.627	2107.371	-0.454	0.224	0.042	0.330	2107.371	-0.606	0.258	0.019	0.098
PUF60	1763.117	-0.617	0.332	0.063	0.138	1763.117	-0.675	0.332	0.042	0.330	1763.117	-0.057	0.384	0.881	0.943
SCAF8	655.651	-0.977	0.238	0.000	0.000	655.651	-0.469	0.236	0.047	0.336	655.651	0.509	0.275	0.064	0.213
CEP162	122.656	0.358	0.194	0.065	0.142	122.656	-0.425	0.200	0.034	0.322	122.656	-0.782	0.229	0.001	0.010
ZNF652	314.140	0.824	0.163	0.000	0.000	314.140	0.020	0.165	0.906	0.953	314.140	-0.804	0.189	0.000	0.001
ZFP30	24.383	0.242	0.474	0.610	0.722	24.383	0.531	0.465	0.254	0.527	24.383	0.289	0.541	0.593	0.773
RHOBTB3	1915.943	-0.434	0.279	0.120	0.223	1915.943	0.174	0.279	0.531	0.731	1915.943	0.609	0.322	0.059	0.202
COBLL1	135.156	-1.314	0.251	0.000	0.000	135.156	-0.219	0.239	0.359	0.611	135.156	1.095	0.288	0.000	0.003
RNF44	1165.604	-0.156	0.159	0.326	0.466	1165.604	0.254	0.158	0.109	0.401	1165.604	0.409	0.183	0.025	0.118
DLGAP4	919.077	0.365	0.136	0.007	0.026	919.077	0.075	0.136	0.579	0.763	919.077	-0.290	0.157	0.065	0.214
RAB11FIP2	712.388	-1.570	0.278	0.000	0.000	712.388	-0.180	0.275	0.514	0.720	712.388	1.390	0.321	0.000	0.001
PPM1E	405.610	-1.833	0.325	0.000	0.000	405.610	0.331	0.318	0.298	0.565	405.610	2.164	0.373	0.000	0.000
DOLK	209.693	0.835	0.281	0.003	0.013	209.693	-0.391	0.286	0.170	0.460	209.693	-1.226	0.327	0.000	0.004

VASH1	77.108	1.350	0.301	0.000	0.000	77.108	0.155	0.311	0.618	0.790	77.108	-1.195	0.351	0.001	0.010
ZNF507	470.065	-1.117	0.245	0.000	0.000	470.065	-0.335	0.243	0.167	0.457	470.065	0.782	0.283	0.006	0.045
AAK1	633.256	0.405	0.136	0.003	0.013	633.256	-0.182	0.137	0.186	0.472	633.256	-0.587	0.158	0.000	0.004
CPEB3	71.561	-0.569	0.268	0.034	0.086	71.561	0.019	0.256	0.941	0.970	71.561	0.588	0.307	0.056	0.196
ADNP2	683.534	-0.885	0.227	0.000	0.001	683.534	-0.237	0.225	0.293	0.561	683.534	0.648	0.262	0.013	0.079
ANKRD26	186.186	-0.049	0.239	0.837	0.892	186.186	-0.540	0.241	0.025	0.316	186.186	-0.491	0.279	0.078	0.241
LMTK2	324.393	-0.498	0.231	0.031	0.080	324.393	-0.227	0.229	0.322	0.583	324.393	0.271	0.266	0.308	0.544
CHSY1	860.193	-0.378	0.162	0.020	0.056	860.193	-0.209	0.161	0.194	0.478	860.193	0.169	0.187	0.366	0.599
ICK	308.704	-0.254	0.242	0.293	0.431	308.704	0.200	0.240	0.404	0.646	308.704	0.454	0.278	0.103	0.286
ADGRL1	2001.830	0.111	0.121	0.359	0.499	2001.830	0.007	0.120	0.953	0.977	2001.830	-0.103	0.139	0.458	0.676
FKBP2	305.545	0.738	0.262	0.005	0.020	305.545	0.137	0.264	0.602	0.780	305.545	-0.600	0.304	0.048	0.178
NLRP1	100.199	0.034	0.236	0.884	0.925	100.199	0.450	0.231	0.052	0.339	100.199	0.415	0.270	0.124	0.318
FNDC3A	931.849	-0.093	0.224	0.679	0.777	931.849	-0.009	0.223	0.966	0.982	931.849	0.083	0.258	0.748	0.870
ATG14	456.916	0.075	0.344	0.828	0.887	456.916	0.519	0.343	0.130	0.422	456.916	0.445	0.397	0.263	0.497
R3HDM2	1133.794	-0.301	0.249	0.227	0.359	1133.794	0.480	0.248	0.053	0.342	1133.794	0.781	0.287	0.007	0.049
FASTKD2	446.536	-0.666	0.255	0.009	0.031	446.536	-0.544	0.254	0.032	0.322	446.536	0.122	0.295	0.678	0.830
ZNF510	156.141	-0.348	0.233	0.135	0.245	156.141	-0.137	0.230	0.553	0.745	156.141	0.212	0.270	0.432	0.654
FKBP3	1267.878	0.270	0.209	0.196	0.323	1267.878	-0.249	0.209	0.233	0.511	1267.878	-0.519	0.242	0.032	0.137
PPP6R1	2271.827	-0.147	0.258	0.569	0.688	2271.827	-0.500	0.258	0.053	0.341	2271.827	-0.353	0.298	0.236	0.466
SEC31A	3356.297	-0.165	0.144	0.253	0.387	3356.297	-0.088	0.144	0.542	0.738	3356.297	0.077	0.167	0.644	0.807
DZIP1	813.081	-0.110	0.119	0.356	0.496	813.081	-0.159	0.119	0.180	0.468	813.081	-0.049	0.138	0.721	0.855
PLEKHA6	59.271	1.144	0.417	0.006	0.023	59.271	0.750	0.419	0.073	0.362	59.271	-0.394	0.479	0.411	0.637
ENPP4	123.825	0.072	0.329	0.827	0.886	123.825	0.259	0.326	0.427	0.663	123.825	0.188	0.379	0.620	0.792
INPP5F	334.631	-0.620	0.180	0.001	0.004	334.631	-0.097	0.177	0.583	0.766	334.631	0.523	0.207	0.012	0.072
MLXIP	1364.242	0.135	0.106	0.202	0.329	1364.242	-0.046	0.106	0.662	0.816	1364.242	-0.181	0.123	0.139	0.342
TRAPPC8	474.526	-0.618	0.219	0.005	0.019	474.526	-0.395	0.218	0.070	0.358	474.526	0.223	0.253	0.378	0.609
MON1B	822.112	-0.223	0.138	0.106	0.204	822.112	-0.168	0.138	0.222	0.501	822.112	0.055	0.160	0.729	0.859
FKBP4	3500.096	-0.723	0.262	0.006	0.022	3500.096	-0.497	0.262	0.058	0.347	3500.096	0.226	0.302	0.455	0.673
MORC2	632.364	-0.136	0.138	0.324	0.464	632.364	0.054	0.137	0.693	0.834	632.364	0.190	0.159	0.232	0.461
ANKRD6	24.806	0.451	0.350	0.197	0.323	24.806	0.286	0.349	0.414	0.652	24.806	-0.165	0.402	0.681	0.831
ZHX2	72.752	-0.285	0.223	0.200	0.327	72.752	-0.085	0.216	0.694	0.835	72.752	0.200	0.257	0.437	0.658
CLSTN1	6245.748	0.076	0.271	0.778	0.853	6245.748	0.424	0.271	0.117	0.409	6245.748	0.348	0.313	0.266	0.500
WDR37	234.901	0.097	0.192	0.614	0.726	234.901	0.257	0.190	0.175	0.464	234.901	0.161	0.221	0.466	0.682
ABLIM3	5252.849	-0.837	0.135	0.000	0.000	5252.849	0.029	0.135	0.830	0.913	5252.849	0.866	0.156	0.000	0.000
FOXJ3	881.241	-0.506	0.159	0.001	0.008	881.241	-0.058	0.158	0.712	0.845	881.241	0.448	0.184	0.015	0.084
UBOX5	111.893	-0.239	0.189	0.206	0.334	111.893	0.203	0.181	0.261	0.534	111.893	0.442	0.215	0.040	0.160
KHDC4	533.915	0.198	0.209	0.342	0.482	533.915	0.394	0.208	0.058	0.348	533.915	0.196	0.241	0.415	0.641
FKBP5	17606.047	-0.751	0.270	0.005	0.021	17606.047	0.134	0.270	0.618	0.790	17606.047	0.885	0.311	0.004	0.038
ZBTB1	738.681	-0.039	0.268	0.886	0.926	738.681	0.256	0.267	0.338	0.596	738.681	0.295	0.309	0.340	0.575
ZNF365	16.488	0.284	0.756	0.707	0.799	16.488	-2.174	0.828	0.009	0.297	16.488	-2.458	0.936	0.009	0.059
BAHD1	877.065	-0.254	0.138	0.066	0.142	877.065	0.147	0.137	0.285	0.554	877.065	0.401	0.159	0.012	0.073
DIS3	759.985	-0.717	0.216	0.001	0.005	759.985	-0.402	0.215	0.062	0.350	759.985	0.314	0.250	0.208	0.433
CEP164	488.941	-0.020	0.130	0.878	0.921	488.941	0.136	0.129	0.290	0.558	488.941	0.156	0.150	0.297	0.532
DENND3	538.981	-0.316	0.177	0.073	0.155	538.981	0.335	0.175	0.055	0.342	538.981	0.652	0.203	0.001	0.016
CARD8	196.412	0.251	0.277	0.364	0.505	196.412	0.305	0.276	0.269	0.541	196.412	0.054	0.319	0.866	0.936
ARSG	228.454	1.260	0.250	0.000	0.000	228.454	0.262	0.254	0.302	0.568	228.454	-0.998	0.290	0.001	0.009
RUFY3	206.034	0.169	0.139	0.226	0.357	206.034	0.100	0.138	0.471	0.693	206.034	-0.069	0.161	0.668	0.824
BTBD3	879.222	-0.635	0.223	0.005	0.018	879.222	-0.066	0.223	0.767	0.877	879.222	0.568	0.258	0.027	0.124
SBNO2	7300.819	0.102	0.213	0.630	0.739	7300.819	-0.089	0.213	0.675	0.824	7300.819	-0.192	0.246	0.435	0.657
EPN2	431.938	-0.452	0.189	0.017	0.050	431.938	0.131	0.186	0.482	0.701	431.938	0.583	0.217	0.007	0.053
TRAK1	689.445	-0.135	0.175	0.440	0.576	689.445	-0.001	0.174	0.994	0.997	689.445	0.134	0.202	0.508	0.714
DHX30	1993.246	-0.445	0.187	0.017	0.052	1993.246	-0.253	0.187	0.176	0.465	1993.246	0.192	0.216	0.374	0.606
SACM1L	901.051	-0.702	0.176	0.000	0.001	901.051	-0.321	0.175	0.066	0.354	901.051	0.382	0.203	0.060	0.205
FAN1	1114.969	-0.667	0.204	0.001	0.006	1114.969	-0.231	0.203	0.256	0.529	1114.969	0.436	0.236	0.064	0.214
WDR47	400.080	-0.588	0.239	0.014	0.043	400.080	0.075	0.236	0.750	0.867	400.080	0.663	0.275	0.016	0.088
RALY	2499.766	0.354	0.200	0.078	0.161	2499.766	-0.032	0.200	0.872	0.935	2499.766	-0.386	0.231	0.095	0.272
NCBP2	2083.725	-0.232	0.152	0.126	0.233	2083.725	-0.332	0.152	0.029	0.322	2083.725	-0.100	0.175	0.568	0.758
ZP1	89.321	1.082	1.271	0.395	0.533	89.321	1.522	1.270	0.231	0.508	89.321	0.440	1.465	0.764	0.880
MAPRE1	1897.940	-0.272	0.232	0.241	0.374	1897.940	-0.615	0.232	0.008	0.297	1897.940	-0.343	0.268	0.200	0.423
KIFAP3	438.729	-0.170	0.114	0.136	0.246	438.729	0.092	0.112	0.412	0.651	438.729	0.262	0.132	0.046	0.173
MSRB2	233.136	0.935	0.399	0.019	0.055	233.136	0.417	0.400	0.297	0.565	233.136	-0.519	0.460	0.260	0.494
MAPRE3	434.422	0.707	0.175	0.000	0.001	434.422	0.258	0.176	0.143	0.434	434.422	-0.448	0.203	0.027	0.123
PLA2R1	220.794	0.179	0.328	0.584	0.701	220.794	0.389	0.326	0.233	0.510	220.794	0.210	0.377	0.578	0.763
ATF6	1142.634	-0.525	0.185	0.004	0.018	1142.634	-0.292	0.184	0.113	0.405	1142.634	0.233	0.214	0.276	0.510
HABP4	300.423	0.989	0.188	0.000	0.000	300.423	-0.360	0.193	0.062	0.350	300.423	-1.349	0.220	0.000	0.000
SEPHS2	1324.432	-0.387	0.191	0.043	0.103	1324.432	-0.335	0.191	0.079	0.370	1324.432	0.051	0.221	0.816	0.908
SEPHS1	1209.184	-0.506	0.212	0.017	0.051	1209.184	-0.498	0.212	0.019	0.306	1209.184	0.008	0.245	0.975	0.987

RAB3GAP1	1340.532	-0.548	0.134	0.000	0.000	1340.532	-0.032	0.133	0.808	0.899	1340.532	0.516	0.154	0.001	0.011
RAB18	1326.354	-0.511	0.162	0.002	0.008	1326.354	-0.493	0.162	0.002	0.297	1326.354	0.018	0.187	0.922	0.964
POMZP3	195.098	0.154	0.202	0.445	0.580	195.098	0.049	0.201	0.809	0.900	195.098	-0.106	0.233	0.651	0.812
SIRT2	670.454	0.692	0.163	0.000	0.000	670.454	0.262	0.163	0.109	0.401	670.454	-0.430	0.188	0.022	0.108
RPIA	747.220	-0.747	0.298	0.012	0.040	747.220	-0.582	0.297	0.050	0.337	747.220	0.165	0.344	0.632	0.800
ELL2	4068.420	-0.312	0.195	0.111	0.211	4068.420	0.012	0.195	0.951	0.976	4068.420	0.324	0.226	0.151	0.360
SCAP	2094.380	-0.274	0.116	0.018	0.054	2094.380	0.089	0.116	0.439	0.672	2094.380	0.363	0.134	0.007	0.050
SNW1	1682.462	-0.288	0.176	0.102	0.199	1682.462	-0.239	0.176	0.175	0.464	1682.462	0.049	0.204	0.808	0.904
FOXF1	60.675	0.399	0.399	0.317	0.457	60.675	-1.019	0.418	0.015	0.306	60.675	-1.418	0.477	0.003	0.028
DKK1	8.867	1.350	0.846	0.110	0.211	8.867	-2.081	1.013	0.040	0.327	8.867	-3.431	1.113	0.002	0.022
KIN	243.133	0.166	0.180	0.355	0.495	243.133	-0.254	0.181	0.162	0.453	243.133	-0.420	0.210	0.045	0.171
CCT5	10247.528	-0.455	0.275	0.098	0.193	10247.528	-0.683	0.275	0.013	0.305	10247.528	-0.227	0.318	0.474	0.688
PTGR1	186.041	0.182	0.348	0.601	0.715	186.041	0.206	0.348	0.554	0.746	186.041	0.023	0.402	0.953	0.978
FOXF2	694.984	-0.117	0.151	0.439	0.575	694.984	0.085	0.150	0.570	0.756	694.984	0.202	0.174	0.246	0.477
SLC4A1AP	553.252	-0.244	0.218	0.263	0.399	553.252	-0.262	0.218	0.230	0.507	553.252	-0.017	0.253	0.945	0.975
TRIM32	590.683	-0.373	0.200	0.062	0.137	590.683	0.154	0.199	0.439	0.672	590.683	0.527	0.231	0.022	0.109
SCMH1	793.602	-0.208	0.104	0.044	0.106	793.602	0.047	0.102	0.647	0.808	793.602	0.255	0.119	0.033	0.139
FOXC1	552.206	-0.289	0.233	0.214	0.344	552.206	-0.459	0.233	0.049	0.337	552.206	-0.170	0.269	0.529	0.729
FOXD1	532.740	1.074	0.211	0.000	0.000	532.740	0.398	0.213	0.061	0.350	532.740	-0.675	0.243	0.006	0.044
TPX2	3811.089	-0.551	0.207	0.008	0.028	3811.089	-0.225	0.206	0.276	0.546	3811.089	0.326	0.239	0.172	0.389
PAXIP1	317.084	-0.663	0.183	0.000	0.002	317.084	-0.456	0.181	0.012	0.305	317.084	0.207	0.212	0.329	0.563
NT5C2	653.263	0.125	0.166	0.452	0.587	653.263	-0.203	0.167	0.224	0.503	653.263	-0.328	0.193	0.089	0.261
TCF25	1534.608	-0.007	0.124	0.954	0.972	1534.608	0.188	0.123	0.127	0.420	1534.608	0.195	0.143	0.172	0.389
DIP2C	649.216	-0.534	0.244	0.028	0.075	649.216	0.086	0.242	0.724	0.852	649.216	0.620	0.281	0.027	0.124
MAST1	109.787	0.996	0.841	0.236	0.369	109.787	1.245	0.840	0.139	0.430	109.787	0.249	0.969	0.798	0.899
PDCD11	1824.398	-1.205	0.339	0.000	0.003	1824.398	-0.702	0.339	0.038	0.327	1824.398	0.503	0.392	0.199	0.423
ACIN1	2226.829	0.070	0.188	0.710	0.802	2226.829	-0.275	0.188	0.144	0.435	2226.829	-0.345	0.217	0.113	0.300
PCNX1	1560.052	-0.848	0.244	0.001	0.003	1560.052	-0.112	0.243	0.644	0.806	1560.052	0.736	0.282	0.009	0.061
KDM2A	1929.573	-0.174	0.127	0.170	0.291	1929.573	-0.040	0.127	0.751	0.867	1929.573	0.134	0.147	0.361	0.593
HMGXB3	1026.332	0.016	0.179	0.930	0.956	1026.332	-0.344	0.179	0.055	0.342	1026.332	-0.360	0.207	0.082	0.248
CEP131	434.706	0.547	0.243	0.024	0.066	434.706	0.245	0.243	0.314	0.577	434.706	-0.302	0.280	0.280	0.515
CEP152	193.140	-0.239	0.227	0.293	0.431	193.140	-0.252	0.226	0.266	0.538	193.140	-0.013	0.263	0.962	0.982
TTC39A	13.956	0.952	0.503	0.058	0.129	13.956	0.549	0.510	0.282	0.552	13.956	-0.403	0.574	0.482	0.694
LIMCH1	2541.507	-0.320	0.205	0.118	0.221	2541.507	0.136	0.205	0.508	0.717	2541.507	0.456	0.237	0.054	0.192
ABCF1	7997.420	-0.126	0.270	0.640	0.747	7997.420	-0.516	0.270	0.056	0.343	7997.420	-0.390	0.311	0.210	0.435
ALDOC	2376.245	1.076	1.049	0.305	0.444	2376.245	1.051	1.049	0.316	0.578	2376.245	-0.025	1.211	0.984	0.991
FOXL1	100.679	0.194	0.560	0.729	0.816	100.679	0.729	0.557	0.191	0.476	100.679	0.535	0.645	0.407	0.633
WDFY3	626.086	-0.720	0.230	0.002	0.009	626.086	-0.197	0.228	0.389	0.635	626.086	0.523	0.265	0.048	0.179
DAAM1	52.586	0.367	0.268	0.172	0.292	52.586	-0.646	0.283	0.022	0.315	52.586	-1.012	0.322	0.002	0.019
MAPKBP1	391.801	0.100	0.154	0.515	0.643	391.801	-0.079	0.154	0.610	0.785	391.801	-0.179	0.178	0.316	0.552
PLCH1	7.390	0.503	0.704	0.475	0.609	7.390	0.539	0.697	0.440	0.672	7.390	0.036	0.804	0.964	0.983
KLHDC10	846.642	-0.227	0.264	0.390	0.529	846.642	0.131	0.263	0.619	0.790	846.642	0.358	0.305	0.240	0.470
RAB21	1400.459	-0.359	0.219	0.101	0.197	1400.459	-0.381	0.219	0.082	0.373	1400.459	-0.022	0.253	0.932	0.969
STK38L	494.182	0.181	0.211	0.391	0.530	494.182	-0.486	0.212	0.022	0.314	494.182	-0.667	0.245	0.006	0.049
SPEN	1911.475	-0.693	0.205	0.001	0.005	1911.475	-0.142	0.205	0.488	0.705	1911.475	0.551	0.237	0.020	0.102
FBXO21	943.928	-0.427	0.184	0.020	0.059	943.928	0.140	0.183	0.445	0.676	943.928	0.566	0.212	0.008	0.055
GOLGA8A	81.139	1.418	0.475	0.003	0.013	81.139	0.514	0.479	0.283	0.553	81.139	-0.904	0.548	0.099	0.279
EXOSC7	825.327	0.344	0.250	0.168	0.288	825.327	-0.365	0.251	0.145	0.438	825.327	-0.709	0.289	0.014	0.082
CNOT1	4609.969	-1.165	0.243	0.000	0.000	4609.969	-0.158	0.243	0.516	0.722	4609.969	1.007	0.281	0.000	0.006
SNRNP200	4884.141	-0.664	0.165	0.000	0.001	4884.141	-0.022	0.165	0.892	0.946	4884.141	0.642	0.190	0.001	0.011
PALLD	2449.877	-0.199	0.218	0.362	0.502	2449.877	0.119	0.218	0.585	0.768	2449.877	0.317	0.251	0.207	0.431
TMCC1	371.326	-0.179	0.181	0.321	0.461	371.326	0.008	0.179	0.967	0.982	371.326	0.187	0.209	0.371	0.602
UNC13A	17.720	1.668	0.791	0.035	0.088	17.720	1.171	0.802	0.144	0.436	17.720	-0.497	0.884	0.574	0.761
KDM1A	3298.297	-0.571	0.143	0.000	0.001	3298.297	-0.213	0.143	0.137	0.428	3298.297	0.358	0.165	0.030	0.133
RBM34	24.944	-0.384	0.419	0.360	0.500	24.944	-0.467	0.416	0.261	0.534	24.944	-0.083	0.494	0.866	0.936
FOXC2	808.314	-1.422	0.367	0.000	0.001	808.314	-0.639	0.365	0.080	0.371	808.314	0.783	0.423	0.064	0.213
KDM4B	2189.539	0.149	0.335	0.657	0.760	2189.539	0.515	0.335	0.124	0.416	2189.539	0.366	0.387	0.343	0.577
MAST3	267.344	0.166	0.342	0.629	0.738	267.344	0.487	0.341	0.154	0.446	267.344	0.321	0.395	0.416	0.641
USP33	991.371	-0.398	0.140	0.004	0.018	991.371	-0.129	0.139	0.353	0.607	991.371	0.269	0.161	0.095	0.272
DOP1A	326.315	-0.390	0.218	0.073	0.155	326.315	-0.114	0.216	0.596	0.775	326.315	0.275	0.251	0.273	0.508
SAMD4A	938.501	-0.728	0.252	0.004	0.016	938.501	-0.296	0.251	0.239	0.515	938.501	0.432	0.291	0.138	0.341
PHLPP2	190.788	-0.027	0.279	0.922	0.950	190.788	-0.443	0.280	0.113	0.405	190.788	-0.416	0.324	0.199	0.422
ZNF292	911.479	0.264	0.345	0.445	0.580	911.479	0.284	0.345	0.410	0.650	911.479	0.021	0.399	0.959	0.981
PDZD2	356.308	0.500	0.737	0.497	0.629	356.308	0.556	0.737	0.451	0.679	356.308	0.056	0.851	0.948	0.976
WDTC1	1013.714	0.419	0.316	0.185	0.309	1013.714	0.393	0.316	0.214	0.493	1013.714	-0.026	0.365	0.943	0.974
XPO7	1492.931	-0.580	0.122	0.000	0.000	1492.931	-0.216	0.122	0.075	0.364	1492.931	0.364	0.141	0.010	0.065
MON2	551.466	-0.755	0.266	0.005	0.018	551.466	-0.215	0.265	0.417	0.655	551.466	0.541	0.307	0.079	0.242

PDXDC1	3595.618	-0.131	0.172	0.447	0.582	3595.618	0.064	0.172	0.711	0.845	3595.618	0.194	0.198	0.327	0.562
TNIK	236.092	-0.648	0.267	0.015	0.047	236.092	0.121	0.263	0.646	0.807	236.092	0.769	0.307	0.012	0.075
KIF21B	23.100	-2.743	0.752	0.000	0.002	23.100	-0.411	0.668	0.539	0.736	23.100	2.332	0.849	0.006	0.047
PDS5B	518.655	-0.570	0.176	0.001	0.007	518.655	-0.286	0.174	0.100	0.394	518.655	0.283	0.203	0.163	0.377
FNBP1	481.342	0.104	0.298	0.727	0.814	481.342	0.435	0.297	0.143	0.435	481.342	0.331	0.344	0.336	0.570
SMG1	2252.836	-0.990	0.291	0.001	0.004	2252.836	-0.245	0.290	0.399	0.643	2252.836	0.746	0.336	0.026	0.121
FOXM1	1546.444	-0.568	0.256	0.026	0.071	1546.444	-0.287	0.255	0.262	0.534	1546.444	0.281	0.295	0.341	0.575
ZHX3	998.307	-0.564	0.195	0.004	0.016	998.307	0.081	0.194	0.675	0.825	998.307	0.645	0.225	0.004	0.036
ENDOD1	68.847	1.321	0.290	0.000	0.000	68.847	-0.575	0.310	0.063	0.350	68.847	-1.897	0.347	0.000	0.000
ZSWIM8	969.197	0.477	0.154	0.002	0.010	969.197	0.306	0.154	0.047	0.335	969.197	-0.171	0.178	0.337	0.571
NCOA6	845.173	-0.438	0.137	0.001	0.007	845.173	0.023	0.135	0.863	0.930	845.173	0.461	0.157	0.003	0.031
CLUAP1	579.942	-0.385	0.194	0.047	0.110	579.942	0.079	0.192	0.682	0.827	579.942	0.464	0.223	0.038	0.153
FOXD2	67.565	0.151	0.279	0.588	0.704	67.565	0.396	0.274	0.149	0.442	67.565	0.244	0.320	0.445	0.664
ZNF609	1284.057	-1.022	0.188	0.000	0.000	1284.057	-0.404	0.187	0.031	0.322	1284.057	0.618	0.217	0.004	0.037
TBC1D9B	2963.638	0.225	0.269	0.404	0.542	2963.638	0.362	0.269	0.179	0.467	2963.638	0.137	0.311	0.659	0.818
GGA2	410.009	0.347	0.206	0.092	0.184	410.009	-0.096	0.207	0.642	0.805	410.009	-0.443	0.239	0.063	0.211
WAPL	1338.110	-0.997	0.211	0.000	0.000	1338.110	-0.375	0.210	0.075	0.364	1338.110	0.621	0.244	0.011	0.069
SETX	762.133	-0.642	0.271	0.018	0.053	762.133	-0.507	0.270	0.061	0.350	762.133	0.135	0.313	0.666	0.822
EMC1	2191.725	-0.449	0.143	0.002	0.009	2191.725	-0.038	0.142	0.788	0.889	2191.725	0.411	0.165	0.013	0.076
CAND2	21.073	0.246	0.357	0.492	0.624	21.073	0.145	0.355	0.684	0.829	21.073	-0.101	0.413	0.806	0.903
SETD1B	425.226	-0.437	0.142	0.002	0.010	425.226	0.018	0.139	0.896	0.948	425.226	0.455	0.163	0.005	0.043
CMTR1	860.046	-0.306	0.139	0.028	0.075	860.046	-0.116	0.139	0.401	0.644	860.046	0.189	0.161	0.239	0.469
ERP44	1053.273	0.420	0.140	0.003	0.012	1053.273	-0.092	0.141	0.513	0.719	1053.273	-0.512	0.162	0.002	0.019
HECW1	96.535	0.045	0.266	0.866	0.913	96.535	0.024	0.265	0.929	0.964	96.535	-0.021	0.307	0.945	0.975
UHRF1BP1L	809.504	-0.894	0.224	0.000	0.001	809.504	-0.101	0.223	0.651	0.810	809.504	0.794	0.259	0.002	0.023
SWAP70	1244.010	-0.150	0.154	0.332	0.472	1244.010	-0.316	0.154	0.041	0.328	1244.010	-0.166	0.178	0.351	0.585
RRP1B	1165.021	-0.898	0.252	0.000	0.003	1165.021	-0.662	0.251	0.008	0.297	1165.021	0.237	0.291	0.416	0.641
MYCBP2	999.232	-0.767	0.246	0.002	0.009	999.232	-0.287	0.246	0.243	0.519	999.232	0.480	0.284	0.091	0.265
VWA8	535.530	-0.557	0.166	0.001	0.005	535.530	-0.117	0.165	0.477	0.698	535.530	0.440	0.192	0.022	0.107
FOXO1	1090.981	-0.901	0.306	0.003	0.014	1090.981	0.309	0.305	0.311	0.575	1090.981	1.210	0.353	0.001	0.009
AVL9	654.400	-0.425	0.151	0.005	0.019	654.400	-0.129	0.150	0.390	0.635	654.400	0.297	0.174	0.089	0.261
KDM4C	365.704	-0.196	0.217	0.367	0.507	365.704	0.184	0.215	0.392	0.636	365.704	0.380	0.250	0.128	0.324
PPRC1	1101.077	-1.047	0.338	0.002	0.010	1101.077	-0.725	0.338	0.032	0.322	1101.077	0.322	0.391	0.410	0.636
ERC1	1578.459	-0.693	0.268	0.010	0.033	1578.459	-0.097	0.267	0.716	0.848	1578.459	0.596	0.309	0.054	0.192
EXPH5	26.506	0.568	0.321	0.077	0.161	26.506	0.765	0.313	0.014	0.306	26.506	0.197	0.360	0.584	0.766
TRIM35	883.699	-0.466	0.144	0.001	0.007	883.699	0.074	0.143	0.604	0.781	883.699	0.540	0.166	0.001	0.015
PEG10	2701.445	-0.705	0.285	0.013	0.042	2701.445	0.072	0.285	0.801	0.896	2701.445	0.777	0.329	0.018	0.096
FOXO3	3930.281	-0.570	0.311	0.067	0.145	3930.281	0.274	0.311	0.379	0.626	3930.281	0.844	0.360	0.019	0.098
ZC3H13	1036.643	-0.128	0.176	0.467	0.601	1036.643	-0.359	0.176	0.041	0.329	1036.643	-0.231	0.203	0.256	0.490
ARHGAP26	173.555	-0.277	0.296	0.349	0.489	173.555	-0.281	0.295	0.341	0.599	173.555	-0.004	0.343	0.992	0.995
TTLL5	568.571	-0.282	0.127	0.026	0.071	568.571	-0.085	0.126	0.498	0.712	568.571	0.197	0.147	0.179	0.398
SIPA1L3	875.561	-0.159	0.139	0.252	0.386	875.561	-0.022	0.138	0.874	0.936	875.561	0.137	0.160	0.393	0.621
KIF1B	1635.612	-0.689	0.193	0.000	0.003	1635.612	-0.226	0.192	0.239	0.516	1635.612	0.463	0.223	0.038	0.153
IQSEC2	552.444	0.486	0.381	0.202	0.329	552.444	0.705	0.380	0.064	0.350	552.444	0.219	0.439	0.617	0.790
CDK19	488.004	-0.040	0.552	0.943	0.964	488.004	0.623	0.551	0.259	0.531	488.004	0.662	0.637	0.299	0.534
SARM1	1128.309	-0.057	0.134	0.670	0.770	1128.309	0.015	0.133	0.910	0.955	1128.309	0.072	0.154	0.641	0.805
ZBTB43	620.704	-0.488	0.183	0.008	0.028	620.704	-0.104	0.182	0.566	0.754	620.704	0.383	0.211	0.070	0.225
AKR1B1	18608.560	0.255	0.335	0.446	0.581	18608.560	-0.654	0.335	0.051	0.338	18608.560	-0.909	0.386	0.019	0.097
FOXO3B	256.720	-1.092	0.402	0.007	0.025	256.720	0.186	0.398	0.640	0.803	256.720	1.278	0.463	0.006	0.045
MCF2L2	452.380	0.085	0.338	0.802	0.869	452.380	-0.710	0.340	0.037	0.324	452.380	-0.795	0.392	0.043	0.165
TBC1D2B	1459.204	-0.754	0.281	0.007	0.027	1459.204	0.253	0.280	0.367	0.617	1459.204	1.007	0.324	0.002	0.021
FSTL4	13.174	0.868	0.742	0.242	0.375	13.174	0.559	0.744	0.453	0.680	13.174	-0.309	0.853	0.717	0.853
MRPS27	2981.690	-0.271	0.121	0.025	0.067	2981.690	-0.021	0.120	0.862	0.930	2981.690	0.250	0.139	0.072	0.230
RAP1GAP2	331.891	-1.534	0.214	0.000	0.000	331.891	-0.147	0.206	0.477	0.698	331.891	1.387	0.245	0.000	0.000
DDN	7.684	0.750	0.618	0.225	0.356	7.684	-1.201	0.752	0.111	0.404	7.684	-1.951	0.823	0.018	0.094
SPART	1964.672	-0.260	0.117	0.026	0.070	1964.672	-0.006	0.116	0.958	0.978	1964.672	0.254	0.135	0.059	0.204
TNRC6B	420.834	0.244	0.136	0.074	0.156	420.834	0.129	0.136	0.342	0.599	420.834	-0.115	0.158	0.467	0.682
CUL9	364.883	0.310	0.254	0.223	0.353	364.883	0.370	0.254	0.144	0.435	364.883	0.061	0.293	0.836	0.921
TOGARAM1	352.983	-0.737	0.301	0.014	0.044	352.983	0.248	0.297	0.404	0.646	352.983	0.985	0.346	0.004	0.038
NPIPB3	598.914	1.401	0.379	0.000	0.002	598.914	0.011	0.380	0.977	0.987	598.914	-1.390	0.438	0.001	0.018
TAB2	1476.052	-0.876	0.171	0.000	0.000	1476.052	-0.159	0.169	0.347	0.602	1476.052	0.717	0.197	0.000	0.005
HIC2	99.761	-0.309	0.307	0.314	0.454	99.761	-0.204	0.304	0.501	0.714	99.761	0.104	0.355	0.769	0.882
FLG	6.895	-0.372	0.961	0.698	0.792	6.895	-0.536	0.961	0.577	0.762	6.895	-0.163	1.122	0.884	0.945
ATP10B	12.320	0.514	0.681	0.450	0.586	12.320	0.589	0.676	0.384	0.629	12.320	0.075	0.779	0.924	0.964
CLASP2	830.767	-0.993	0.283	0.000	0.003	830.767	-0.565	0.282	0.045	0.334	830.767	0.428	0.327	0.190	0.412
CAMTA2	427.613	0.225	0.111	0.044	0.104	427.613	0.039	0.111	0.723	0.852	427.613	-0.185	0.129	0.151	0.360
POGZ	647.096	-0.025	0.155	0.874	0.919	647.096	-0.053	0.155	0.731	0.856	647.096	-0.029	0.179	0.873	0.939

PLXND1	135.077	1.302	0.382	0.001	0.004	135.077	0.280	0.385	0.467	0.690	135.077	-1.022	0.442	0.021	0.104
FLI1	8.900	0.493	0.704	0.484	0.617	8.900	-3.155	1.189	0.008	0.297	8.900	-3.648	1.255	0.004	0.033
ATG2A	600.021	0.186	0.152	0.222	0.352	600.021	-0.129	0.153	0.397	0.641	600.021	-0.315	0.176	0.074	0.233
GPATCH8	937.689	-0.597	0.126	0.000	0.000	937.689	-0.152	0.124	0.224	0.502	937.689	0.445	0.145	0.002	0.023
RAD54L2	291.371	0.109	0.204	0.594	0.708	291.371	-0.415	0.205	0.043	0.331	291.371	-0.523	0.237	0.027	0.123
PHF8	350.754	-0.576	0.159	0.000	0.002	350.754	-0.332	0.157	0.034	0.322	350.754	0.245	0.184	0.183	0.403
KDM6B	611.000	0.330	0.192	0.087	0.176	611.000	0.151	0.192	0.432	0.666	611.000	-0.178	0.222	0.422	0.646
EPB41L3	2136.262	-0.461	0.151	0.002	0.011	2136.262	-0.084	0.151	0.577	0.762	2136.262	0.377	0.175	0.031	0.134
SMC5	563.276	-0.679	0.256	0.008	0.029	563.276	-0.205	0.255	0.421	0.658	563.276	0.474	0.295	0.109	0.294
N4BP3	72.502	0.601	0.366	0.101	0.197	72.502	-0.021	0.374	0.954	0.978	72.502	-0.622	0.427	0.145	0.352
MAST2	1015.429	0.175	0.212	0.408	0.546	1015.429	-0.405	0.212	0.057	0.344	1015.429	-0.580	0.245	0.018	0.095
FLII	2150.313	0.024	0.124	0.845	0.898	2150.313	0.074	0.124	0.551	0.744	2150.313	0.050	0.144	0.730	0.860
ZZEF1	403.782	-0.710	0.216	0.001	0.006	403.782	-0.206	0.214	0.335	0.594	403.782	0.504	0.249	0.043	0.167
ANKLE2	1587.067	-0.120	0.160	0.455	0.590	1587.067	-0.169	0.160	0.292	0.560	1587.067	-0.049	0.185	0.792	0.896
DCUN1D4	679.592	-0.449	0.154	0.004	0.015	679.592	-0.221	0.153	0.150	0.442	679.592	0.228	0.178	0.201	0.424
LRCH1	362.283	-0.405	0.231	0.080	0.165	362.283	-0.233	0.230	0.311	0.575	362.283	0.172	0.267	0.520	0.723
ZC3H3	536.879	0.229	0.178	0.199	0.325	536.879	-0.220	0.179	0.219	0.498	536.879	-0.450	0.207	0.030	0.131
NACAD	21.258	1.002	0.946	0.289	0.427	21.258	1.124	0.944	0.233	0.511	21.258	0.122	1.086	0.910	0.959
FCHO1	16.989	0.850	0.618	0.169	0.289	16.989	0.558	0.620	0.368	0.618	16.989	-0.292	0.710	0.681	0.831
FRMD4B	674.996	-0.289	0.251	0.250	0.384	674.996	-0.392	0.251	0.119	0.411	674.996	-0.103	0.291	0.723	0.856
GRAMD4	397.474	0.559	0.303	0.065	0.140	397.474	0.354	0.303	0.242	0.518	397.474	-0.206	0.349	0.556	0.749
CIC	2252.061	0.429	0.348	0.217	0.347	2252.061	0.405	0.348	0.244	0.519	2252.061	-0.024	0.402	0.952	0.978
NCDN	552.760	-0.230	0.171	0.179	0.301	552.760	-0.209	0.170	0.219	0.499	552.760	0.021	0.198	0.917	0.961
CLCC1	1028.147	0.008	0.164	0.963	0.978	1028.147	0.069	0.163	0.674	0.824	1028.147	0.061	0.189	0.746	0.870
SEPTIN6	172.124	0.994	0.259	0.000	0.001	172.124	0.595	0.260	0.022	0.314	172.124	-0.399	0.298	0.180	0.398
TBC1D9	1370.957	-0.606	0.342	0.076	0.159	1370.957	0.272	0.341	0.426	0.662	1370.957	0.878	0.394	0.026	0.120
FLNA	45563.779	-0.137	0.207	0.510	0.639	45563.779	0.240	0.207	0.246	0.521	45563.779	0.377	0.239	0.115	0.304
WDR43	1997.890	-1.164	0.250	0.000	0.000	1997.890	-0.630	0.250	0.012	0.305	1997.890	0.534	0.289	0.065	0.214
SNX13	362.604	-0.518	0.211	0.014	0.044	362.604	0.011	0.208	0.957	0.978	362.604	0.529	0.243	0.029	0.129
MAPK8IP3	713.923	1.469	0.448	0.001	0.006	713.923	0.875	0.448	0.051	0.338	713.923	-0.594	0.517	0.250	0.482
GGA3	474.212	-0.236	0.163	0.149	0.262	474.212	-0.124	0.162	0.444	0.675	474.212	0.112	0.189	0.554	0.747
MPRIP	4108.621	-0.455	0.127	0.000	0.003	4108.621	-0.114	0.127	0.368	0.618	4108.621	0.340	0.147	0.021	0.104
NUP205	1709.928	-1.092	0.247	0.000	0.000	1709.928	-0.442	0.246	0.073	0.362	1709.928	0.650	0.285	0.023	0.109
STAB1	81.707	0.543	0.387	0.161	0.279	81.707	-0.312	0.394	0.428	0.663	81.707	-0.855	0.452	0.059	0.202
EFR3A	685.966	-0.486	0.204	0.017	0.051	685.966	-0.335	0.203	0.099	0.394	685.966	0.151	0.236	0.523	0.724
RTF1	1212.097	0.359	0.156	0.021	0.060	1212.097	-0.130	0.156	0.403	0.645	1212.097	-0.489	0.180	0.007	0.050
SLC35D1	254.601	-0.247	0.298	0.407	0.545	254.601	-0.049	0.296	0.869	0.933	254.601	0.198	0.344	0.564	0.754
FLNB	9119.174	-0.589	0.147	0.000	0.001	9119.174	-0.010	0.147	0.948	0.974	9119.174	0.579	0.170	0.001	0.010
TTLL12	3948.151	-0.729	0.219	0.001	0.005	3948.151	-0.223	0.219	0.308	0.573	3948.151	0.506	0.253	0.045	0.172
GPD1L	914.299	-0.762	0.211	0.000	0.002	914.299	-0.018	0.210	0.932	0.966	914.299	0.744	0.244	0.002	0.024
ABRAXAS2	434.625	-0.551	0.178	0.002	0.010	434.625	-0.403	0.177	0.023	0.315	434.625	0.148	0.207	0.475	0.688
METAP1	1617.779	-0.592	0.126	0.000	0.000	1617.779	-0.084	0.126	0.503	0.715	1617.779	0.508	0.146	0.001	0.008
ZCCHC14	889.822	0.168	0.267	0.529	0.655	889.822	0.369	0.267	0.166	0.456	889.822	0.201	0.308	0.514	0.718
LPIN1	936.037	0.150	0.230	0.514	0.643	936.037	-0.019	0.230	0.934	0.967	936.037	-0.169	0.266	0.525	0.726
SEPTIN8	1033.621	0.251	0.132	0.059	0.130	1033.621	-0.071	0.133	0.595	0.775	1033.621	-0.321	0.153	0.036	0.149
CEP68	504.442	-0.461	0.311	0.138	0.248	504.442	0.279	0.309	0.367	0.618	504.442	0.740	0.358	0.039	0.156
PASK	102.100	-0.136	0.334	0.684	0.782	102.100	-0.505	0.335	0.132	0.424	102.100	-0.369	0.389	0.342	0.576
RGL1	149.664	0.431	0.297	0.146	0.260	149.664	-0.090	0.299	0.762	0.874	149.664	-0.522	0.344	0.130	0.326
RFTN1	627.184	0.125	0.356	0.726	0.814	627.184	-0.929	0.357	0.009	0.297	627.184	-1.053	0.412	0.011	0.069
DIP2A	647.116	-0.052	0.232	0.821	0.883	647.116	0.209	0.231	0.366	0.617	647.116	0.261	0.267	0.328	0.562
MESD	705.389	-0.202	0.158	0.203	0.330	705.389	-0.257	0.158	0.103	0.396	705.389	-0.056	0.183	0.761	0.879
LARP4B	1366.965	-0.636	0.209	0.002	0.011	1366.965	-0.383	0.208	0.066	0.354	1366.965	0.253	0.241	0.295	0.531
RCOR1	971.510	-0.743	0.199	0.000	0.002	971.510	0.001	0.197	0.997	0.999	971.510	0.744	0.229	0.001	0.015
PHLDB1	1218.391	0.204	0.335	0.543	0.668	1218.391	0.353	0.335	0.292	0.559	1218.391	0.149	0.387	0.699	0.843
KANK1	224.957	-0.286	0.252	0.257	0.392	224.957	-0.295	0.251	0.241	0.517	224.957	-0.009	0.292	0.975	0.987
FLOT2	4997.848	0.044	0.207	0.831	0.889	4997.848	0.292	0.207	0.157	0.449	4997.848	0.248	0.239	0.298	0.534
UBXN4	1767.341	0.071	0.108	0.512	0.641	1767.341	-0.049	0.108	0.652	0.810	1767.341	-0.120	0.125	0.338	0.573
CYFIP1	4450.255	-0.514	0.193	0.008	0.028	4450.255	-0.080	0.193	0.679	0.827	4450.255	0.434	0.223	0.051	0.186
ATG4B	502.064	0.400	0.157	0.011	0.036	502.064	-0.183	0.158	0.248	0.522	502.064	-0.583	0.182	0.001	0.017
GANAB	11353.641	-0.209	0.161	0.195	0.321	11353.641	0.195	0.161	0.226	0.504	11353.641	0.405	0.186	0.030	0.132
FBXL7	105.771	0.481	0.293	0.101	0.197	105.771	0.458	0.292	0.117	0.409	105.771	-0.023	0.337	0.946	0.975
MDN1	1062.417	-0.948	0.285	0.001	0.005	1062.417	-0.578	0.285	0.042	0.330	1062.417	0.370	0.329	0.261	0.495
FAM120A	5839.937	-0.553	0.201	0.006	0.023	5839.937	0.066	0.201	0.743	0.863	5839.937	0.619	0.232	0.008	0.055
FAF2	1377.752	-0.526	0.151	0.000	0.003	1377.752	-0.427	0.150	0.005	0.297	1377.752	0.099	0.174	0.571	0.759
PSME4	1115.376	-0.957	0.232	0.000	0.000	1115.376	-0.618	0.232	0.008	0.297	1115.376	0.339	0.269	0.207	0.431
GSE1	1838.330	-0.639	0.162	0.000	0.001	1838.330	0.039	0.161	0.807	0.899	1838.330	0.679	0.187	0.000	0.005
ATP11B	1855.955	-0.530	0.221	0.017	0.050	1855.955	-0.056	0.221	0.799	0.896	1855.955	0.474	0.255	0.063	0.212

FAM168A	1328.091	-0.051	0.367	0.889	0.928	1328.091	0.353	0.366	0.336	0.594	1328.091	0.404	0.423	0.340	0.575
PMPCA	1709.168	-0.233	0.181	0.198	0.324	1709.168	-0.323	0.181	0.074	0.364	1709.168	-0.090	0.209	0.667	0.823
ARL6IP1	5750.568	-0.115	0.283	0.686	0.783	5750.568	0.286	0.283	0.312	0.575	5750.568	0.401	0.327	0.220	0.447
ACSBG1	512.020	-0.285	0.271	0.294	0.432	512.020	-0.693	0.272	0.011	0.300	512.020	-0.408	0.314	0.194	0.417
PLEKHM2	2197.930	0.158	0.107	0.142	0.253	2197.930	-0.018	0.107	0.865	0.931	2197.930	-0.176	0.124	0.156	0.368
SYT11	21.971	-0.488	0.605	0.420	0.557	21.971	0.533	0.574	0.352	0.607	21.971	1.021	0.686	0.137	0.339
JMJD6	891.823	0.637	0.196	0.001	0.006	891.823	0.227	0.196	0.248	0.522	891.823	-0.410	0.226	0.070	0.225
ZC3H4	584.867	-0.526	0.130	0.000	0.001	584.867	-0.021	0.128	0.869	0.933	584.867	0.505	0.150	0.001	0.011
RRS1	595.017	-0.051	0.373	0.892	0.930	595.017	-0.928	0.374	0.013	0.305	595.017	-0.878	0.432	0.042	0.165
SULF1	112.598	0.493	0.934	0.597	0.711	112.598	0.746	0.933	0.424	0.660	112.598	0.253	1.078	0.815	0.908
XPO6	3295.593	-0.726	0.236	0.002	0.010	3295.593	-0.319	0.236	0.175	0.464	3295.593	0.407	0.272	0.135	0.336
PRRC2C	3455.773	0.044	0.196	0.823	0.884	3455.773	-0.256	0.196	0.192	0.477	3455.773	-0.300	0.227	0.185	0.405
TBC1D1	682.935	0.040	0.146	0.782	0.856	682.935	0.074	0.146	0.611	0.786	682.935	0.034	0.169	0.842	0.923
NBEAL2	508.223	0.056	0.144	0.697	0.791	508.223	-0.073	0.144	0.612	0.786	508.223	-0.129	0.167	0.439	0.659
FBXO28	688.167	-0.717	0.132	0.000	0.000	688.167	-0.312	0.130	0.017	0.306	688.167	0.405	0.153	0.008	0.057
DTX4	135.881	0.343	0.820	0.676	0.775	135.881	0.998	0.819	0.223	0.502	135.881	0.655	0.946	0.489	0.699
RHOBTB2	538.988	0.214	0.181	0.238	0.371	538.988	0.192	0.181	0.288	0.557	538.988	-0.022	0.209	0.916	0.961
RRP12	1167.688	-0.945	0.444	0.033	0.085	1167.688	-1.000	0.444	0.024	0.316	1167.688	-0.056	0.513	0.914	0.960
SYNE2	7973.826	-1.218	0.281	0.000	0.000	7973.826	0.460	0.281	0.102	0.394	7973.826	1.677	0.324	0.000	0.000
NUP210	118.536	0.125	0.182	0.491	0.624	118.536	-0.364	0.186	0.050	0.337	118.536	-0.490	0.215	0.022	0.109
PLCL2	66.141	-0.005	0.374	0.989	0.994	66.141	0.296	0.370	0.425	0.661	66.141	0.301	0.431	0.485	0.696
ARHGEF9	261.696	-0.225	0.208	0.279	0.417	261.696	0.192	0.206	0.351	0.605	261.696	0.417	0.240	0.082	0.247
FLT3LG	130.005	0.869	0.276	0.002	0.008	130.005	0.466	0.277	0.093	0.388	130.005	-0.404	0.318	0.204	0.428
VPS13A	1009.508	-0.472	0.244	0.053	0.120	1009.508	-0.131	0.243	0.589	0.770	1009.508	0.341	0.281	0.226	0.454
SEL1L3	2770.448	0.050	0.203	0.806	0.872	2770.448	0.082	0.203	0.687	0.831	2770.448	0.032	0.234	0.892	0.949
TBC1D12	282.799	-0.272	0.211	0.197	0.323	282.799	0.053	0.208	0.800	0.896	282.799	0.324	0.243	0.182	0.400
EXOC6B	297.989	-0.012	0.168	0.941	0.963	297.989	-0.010	0.167	0.954	0.977	297.989	0.003	0.194	0.989	0.994
DNAJC9	697.984	-0.694	0.392	0.076	0.160	697.984	-1.090	0.392	0.005	0.297	697.984	-0.396	0.453	0.382	0.611
SIK2	594.026	-0.380	0.278	0.172	0.292	594.026	0.208	0.277	0.453	0.680	594.026	0.588	0.321	0.067	0.219
PLCB1	151.162	-0.340	0.284	0.231	0.364	151.162	0.136	0.281	0.627	0.796	151.162	0.477	0.327	0.146	0.352
PHLPP1	200.381	-0.375	0.154	0.015	0.047	200.381	-0.026	0.150	0.864	0.931	200.381	0.349	0.178	0.049	0.181
TMEM131L	400.023	-0.805	0.207	0.000	0.001	400.023	-0.207	0.204	0.312	0.575	400.023	0.599	0.239	0.012	0.074
PACS2	843.543	0.474	0.111	0.000	0.000	843.543	0.066	0.111	0.551	0.743	843.543	-0.407	0.128	0.001	0.018
COBL	18.152	0.517	0.380	0.174	0.295	18.152	0.611	0.370	0.099	0.394	18.152	0.094	0.426	0.826	0.914
ANKRD28	1426.069	-0.871	0.220	0.000	0.001	1426.069	-0.061	0.219	0.781	0.884	1426.069	0.810	0.253	0.001	0.017
PDS5A	3174.649	-0.811	0.166	0.000	0.000	3174.649	-0.251	0.165	0.130	0.422	3174.649	0.560	0.191	0.003	0.032
ASTN2	19.192	0.459	0.510	0.368	0.509	19.192	0.577	0.505	0.252	0.526	19.192	0.118	0.583	0.839	0.922
BOP1	3133.327	-0.748	0.386	0.053	0.120	3133.327	-0.780	0.386	0.043	0.331	3133.327	-0.032	0.446	0.943	0.974
KIAA0556	267.647	-0.170	0.165	0.304	0.443	267.647	0.231	0.162	0.155	0.447	267.647	0.400	0.190	0.035	0.145
RPRD2	975.235	-0.571	0.098	0.000	0.000	975.235	-0.189	0.096	0.050	0.337	975.235	0.382	0.113	0.001	0.010
ATP11A	463.186	-0.052	0.286	0.856	0.906	463.186	-0.426	0.286	0.137	0.428	463.186	-0.374	0.331	0.259	0.493
MINAR1	26.016	0.335	0.397	0.399	0.538	26.016	0.168	0.397	0.671	0.822	26.016	-0.166	0.459	0.717	0.853
OTUD3	179.496	0.143	0.215	0.506	0.635	179.496	0.040	0.214	0.853	0.925	179.496	-0.103	0.249	0.678	0.829
ANKRD12	646.755	0.873	0.243	0.000	0.002	646.755	0.209	0.244	0.392	0.637	646.755	-0.664	0.281	0.018	0.096
KAZN	278.433	0.064	0.226	0.778	0.853	278.433	-0.201	0.227	0.377	0.624	278.433	-0.264	0.263	0.314	0.550
MTCL1	258.433	0.380	0.207	0.067	0.144	258.433	-0.129	0.209	0.538	0.735	258.433	-0.508	0.241	0.035	0.144
SCFD1	980.081	-0.615	0.184	0.001	0.005	980.081	-0.370	0.183	0.043	0.331	980.081	0.245	0.212	0.248	0.479
DENND5A	2190.582	-0.264	0.151	0.082	0.168	2190.582	0.063	0.151	0.676	0.825	2190.582	0.327	0.175	0.061	0.208
DDHD2	1331.897	-0.300	0.255	0.239	0.372	1331.897	0.319	0.255	0.210	0.491	1331.897	0.620	0.295	0.035	0.147
CAMTA1	782.735	0.831	0.185	0.000	0.000	782.735	0.123	0.186	0.509	0.718	782.735	-0.708	0.214	0.001	0.013
PPIP5K2	244.552	-0.633	0.242	0.009	0.031	244.552	-0.013	0.238	0.955	0.978	244.552	0.620	0.279	0.026	0.120
ZC3H7B	2635.004	-0.622	0.204	0.002	0.011	2635.004	-0.272	0.204	0.181	0.469	2635.004	0.350	0.236	0.138	0.340
EXOC7	1183.555	0.204	0.138	0.139	0.250	1183.555	0.134	0.138	0.330	0.590	1183.555	-0.070	0.159	0.661	0.819
ADGRL2	869.038	-0.516	0.201	0.010	0.035	869.038	0.046	0.200	0.820	0.906	869.038	0.562	0.232	0.016	0.087
DNMBP	463.478	-0.835	0.204	0.000	0.000	463.478	0.232	0.201	0.247	0.522	463.478	1.067	0.235	0.000	0.000
MGA	353.255	-0.673	0.237	0.005	0.018	353.255	-0.229	0.235	0.330	0.590	353.255	0.444	0.274	0.105	0.288
TSPYL4	268.872	-0.347	0.212	0.102	0.198	268.872	-0.264	0.211	0.212	0.492	268.872	0.083	0.246	0.734	0.863
CAMSAP2	1839.051	-1.113	0.231	0.000	0.000	1839.051	-0.386	0.230	0.094	0.388	1839.051	0.727	0.267	0.006	0.049
TASOR	1437.412	-0.437	0.200	0.028	0.075	1437.412	0.020	0.199	0.919	0.959	1437.412	0.458	0.230	0.047	0.175
CLEC16A	517.254	-0.035	0.196	0.859	0.908	517.254	-0.392	0.197	0.046	0.335	517.254	-0.357	0.227	0.117	0.306
POFUT2	534.712	0.600	0.218	0.006	0.023	534.712	0.012	0.220	0.956	0.978	534.712	-0.588	0.253	0.020	0.102
KLHL18	566.914	-1.055	0.410	0.010	0.034	566.914	-1.100	0.410	0.007	0.297	566.914	-0.044	0.474	0.925	0.965
CLUH	1589.453	-0.549	0.238	0.021	0.060	1589.453	-0.366	0.238	0.124	0.416	1589.453	0.183	0.275	0.507	0.713
NUP160	1145.914	-0.833	0.209	0.000	0.001	1145.914	-0.312	0.208	0.134	0.426	1145.914	0.521	0.241	0.031	0.134
CSTF2T	573.526	-0.153	0.180	0.396	0.535	573.526	-0.313	0.180	0.082	0.374	573.526	-0.160	0.208	0.443	0.663
ADGRL3	461.829	-0.104	0.355	0.770	0.847	461.829	0.403	0.354	0.254	0.528	461.829	0.507	0.409	0.215	0.441
KIAA1107	54.950	-0.132	0.432	0.760	0.841	54.950	0.428	0.426	0.315	0.577	54.950	0.560	0.496	0.259	0.494

WWC1	1488.091	0.390	0.263	0.139	0.249	1488.091	-0.537	0.264	0.042	0.330	1488.091	-0.926	0.304	0.002	0.024
AGTPBP1	196.175	0.181	0.316	0.567	0.687	196.175	0.321	0.314	0.307	0.572	196.175	0.140	0.364	0.699	0.843
IQCE	667.236	0.346	0.148	0.019	0.056	667.236	-0.077	0.149	0.604	0.781	667.236	-0.423	0.172	0.014	0.080
FMO4	21.365	0.944	0.464	0.042	0.101	21.365	0.610	0.468	0.192	0.477	21.365	-0.334	0.530	0.529	0.729
FBXW11	1096.153	-0.803	0.259	0.002	0.010	1096.153	-0.024	0.258	0.925	0.962	1096.153	0.779	0.298	0.009	0.061
SMG6	344.247	-0.005	0.163	0.975	0.985	344.247	0.189	0.161	0.239	0.516	344.247	0.194	0.187	0.299	0.534
ANKS1A	514.909	-0.844	0.244	0.001	0.004	514.909	-0.133	0.242	0.583	0.766	514.909	0.712	0.281	0.011	0.071
MGRN1	855.307	0.173	0.126	0.170	0.291	855.307	0.045	0.126	0.720	0.850	855.307	-0.128	0.146	0.380	0.610
BICD2	1443.252	-0.346	0.146	0.018	0.053	1443.252	-0.135	0.145	0.354	0.608	1443.252	0.211	0.168	0.211	0.436
FMO5	62.623	-0.453	0.387	0.241	0.374	62.623	-0.036	0.380	0.924	0.961	62.623	0.417	0.446	0.350	0.583
ATMIN	671.198	-0.337	0.123	0.006	0.023	671.198	-0.218	0.122	0.073	0.362	671.198	0.119	0.142	0.404	0.631
EHBP1	516.679	-0.356	0.133	0.007	0.027	516.679	-0.280	0.132	0.034	0.322	516.679	0.076	0.154	0.621	0.792
WSCD1	23.233	-0.305	0.518	0.556	0.677	23.233	0.453	0.500	0.365	0.617	23.233	0.758	0.591	0.200	0.423
KIF13B	156.338	-0.215	0.215	0.316	0.456	156.338	0.262	0.210	0.212	0.492	156.338	0.478	0.247	0.053	0.189
UBR2	846.047	-0.411	0.143	0.004	0.017	846.047	0.009	0.141	0.951	0.976	846.047	0.420	0.165	0.011	0.069
NEMP1	538.712	-0.277	0.246	0.260	0.396	538.712	-0.311	0.246	0.206	0.488	538.712	-0.034	0.284	0.905	0.956
FKBP15	974.846	0.187	0.109	0.086	0.174	974.846	-0.120	0.109	0.273	0.544	974.846	-0.307	0.126	0.015	0.086
ICOSLG	75.427	0.359	0.274	0.190	0.315	75.427	0.162	0.274	0.554	0.746	75.427	-0.197	0.316	0.534	0.733
SIN3B	1571.693	0.001	0.163	0.993	0.996	1571.693	0.262	0.163	0.108	0.401	1571.693	0.260	0.188	0.167	0.382
NCAPD3	1122.567	-0.721	0.299	0.016	0.048	1122.567	-0.618	0.298	0.038	0.327	1122.567	0.103	0.345	0.765	0.881
DMXL2	248.461	-0.323	0.273	0.237	0.370	248.461	0.193	0.271	0.477	0.697	248.461	0.516	0.315	0.101	0.283
KIAA0930	1854.329	0.625	0.125	0.000	0.000	1854.329	-0.056	0.126	0.655	0.812	1854.329	-0.681	0.145	0.000	0.000
SATB2	335.985	-0.501	0.159	0.002	0.008	335.985	-0.072	0.156	0.641	0.805	335.985	0.428	0.183	0.019	0.099
SLC9A8	281.823	-0.275	0.151	0.070	0.149	281.823	-0.209	0.150	0.163	0.454	281.823	0.065	0.176	0.709	0.849
CUX2	7.204	-0.782	0.670	0.243	0.376	7.204	0.295	0.600	0.623	0.792	7.204	1.077	0.752	0.152	0.361
DNAJC13	1204.544	-0.670	0.216	0.002	0.010	1204.544	-0.179	0.215	0.404	0.646	1204.544	0.491	0.249	0.049	0.181
TUT4	892.924	-0.850	0.334	0.011	0.036	892.924	0.325	0.333	0.329	0.589	892.924	1.174	0.385	0.002	0.024
FMR1	1181.967	-0.234	0.191	0.221	0.352	1181.967	0.079	0.191	0.680	0.827	1181.967	0.313	0.221	0.156	0.368
TRIM2	373.666	-0.781	0.269	0.004	0.016	373.666	-0.242	0.267	0.365	0.617	373.666	0.539	0.310	0.082	0.249
RPGRIP1L	218.728	-0.446	0.219	0.042	0.101	218.728	-0.072	0.216	0.739	0.861	218.728	0.374	0.253	0.139	0.343
MAN2B2	521.911	0.405	0.227	0.075	0.157	521.911	0.263	0.227	0.248	0.522	521.911	-0.143	0.263	0.587	0.768
WASHC4	1085.072	-0.322	0.198	0.105	0.202	1085.072	-0.132	0.198	0.504	0.715	1085.072	0.190	0.229	0.407	0.634
USP22	2373.605	-0.425	0.131	0.001	0.007	2373.605	-0.100	0.131	0.446	0.677	2373.605	0.325	0.151	0.032	0.137
NEDD4L	941.651	-0.776	0.311	0.013	0.040	941.651	-0.734	0.311	0.018	0.306	941.651	0.042	0.359	0.906	0.957
SASH1	427.832	0.006	0.273	0.983	0.990	427.832	0.262	0.272	0.336	0.595	427.832	0.256	0.315	0.417	0.642
TBC1D30	14.512	0.417	0.432	0.334	0.475	14.512	0.155	0.435	0.721	0.850	14.512	-0.261	0.500	0.601	0.779
TTC28	631.526	0.116	0.181	0.521	0.648	631.526	0.154	0.180	0.393	0.637	631.526	0.038	0.208	0.856	0.931
CLASP1	1823.401	-0.352	0.236	0.136	0.246	1823.401	0.253	0.235	0.282	0.552	1823.401	0.605	0.272	0.026	0.121
DPY19L1	1058.730	0.048	0.165	0.772	0.849	1058.730	-0.249	0.165	0.131	0.423	1058.730	-0.297	0.191	0.120	0.311
SZT2	583.423	-0.066	0.154	0.668	0.769	583.423	0.019	0.153	0.901	0.950	583.423	0.085	0.177	0.632	0.800
WDR7	174.161	-0.624	0.249	0.012	0.040	174.161	-0.482	0.247	0.051	0.338	174.161	0.142	0.289	0.623	0.794
SYNM	89.660	0.704	0.335	0.036	0.090	89.660	-0.009	0.339	0.980	0.989	89.660	-0.712	0.389	0.067	0.219
JADE2	1425.927	-0.811	0.232	0.000	0.003	1425.927	-0.080	0.231	0.730	0.855	1425.927	0.731	0.268	0.006	0.048
VPS39	991.217	-0.103	0.145	0.479	0.613	991.217	-0.002	0.145	0.987	0.992	991.217	0.100	0.167	0.549	0.744
AFF2	797.791	-0.513	0.484	0.289	0.427	797.791	0.380	0.484	0.433	0.667	797.791	0.893	0.559	0.110	0.296
DNAJC16	320.176	-0.169	0.206	0.412	0.550	320.176	-0.147	0.206	0.476	0.697	320.176	0.023	0.239	0.925	0.965
ESYT1	3937.672	-0.476	0.178	0.007	0.027	3937.672	0.061	0.178	0.731	0.856	3937.672	0.537	0.205	0.009	0.060
SYNE1	371.270	-0.287	0.327	0.380	0.520	371.270	0.347	0.325	0.287	0.555	371.270	0.634	0.377	0.093	0.267
SMCHD1	700.859	-0.794	0.235	0.001	0.005	700.859	-0.542	0.234	0.021	0.312	700.859	0.252	0.272	0.354	0.587
DOCK9	739.174	-0.612	0.216	0.005	0.018	739.174	0.073	0.215	0.734	0.857	739.174	0.685	0.249	0.006	0.046
FN1	60582.062	1.009	0.304	0.001	0.005	60582.062	0.491	0.304	0.106	0.398	60582.062	-0.518	0.351	0.140	0.344
U2SURP	2223.098	-0.919	0.249	0.000	0.002	2223.098	-0.589	0.249	0.018	0.306	2223.098	0.330	0.288	0.252	0.484
KHNYN	1249.702	-0.123	0.106	0.247	0.380	1249.702	0.026	0.105	0.802	0.896	1249.702	0.149	0.122	0.223	0.450
UBR4	3954.801	-0.316	0.165	0.055	0.124	3954.801	-0.175	0.165	0.289	0.557	3954.801	0.142	0.190	0.457	0.675
SUN1	1594.872	0.116	0.153	0.450	0.586	1594.872	-0.058	0.153	0.705	0.842	1594.872	-0.174	0.177	0.327	0.562
HAUS5	287.889	0.038	0.244	0.877	0.921	287.889	-0.075	0.244	0.759	0.872	287.889	-0.112	0.282	0.690	0.837
VPS8	415.612	-0.100	0.164	0.542	0.666	415.612	-0.081	0.164	0.620	0.791	415.612	0.019	0.190	0.920	0.963
ANGEL1	378.713	-0.600	0.213	0.005	0.019	378.713	-0.018	0.211	0.933	0.966	378.713	0.583	0.246	0.018	0.094
USP24	1532.935	-0.819	0.212	0.000	0.001	1532.935	-0.341	0.211	0.106	0.398	1532.935	0.479	0.244	0.050	0.183
FAM189A1	16.177	0.683	0.397	0.085	0.173	16.177	0.179	0.409	0.661	0.815	16.177	-0.504	0.462	0.276	0.510
FNBP4	914.507	-0.035	0.239	0.882	0.923	914.507	0.264	0.238	0.269	0.540	914.507	0.299	0.276	0.278	0.512
ZNF629	491.825	0.121	0.167	0.470	0.604	491.825	0.013	0.167	0.939	0.969	491.825	-0.108	0.193	0.576	0.761
PSD3	941.525	-1.056	0.292	0.000	0.002	941.525	0.034	0.291	0.907	0.953	941.525	1.090	0.337	0.001	0.015
OBSL1	911.051	0.650	0.327	0.047	0.110	911.051	0.532	0.327	0.104	0.397	911.051	-0.118	0.377	0.754	0.874
ARHGEF12	2252.064	-0.334	0.240	0.164	0.283	2252.064	-0.185	0.240	0.440	0.672	2252.064	0.149	0.277	0.591	0.771
KIAA0895	233.562	-0.594	0.314	0.058	0.130	233.562	-0.138	0.311	0.658	0.814	233.562	0.456	0.362	0.208	0.433
LARP1	5509.919	-0.535	0.130	0.000	0.000	5509.919	-0.176	0.130	0.175	0.464	5509.919	0.360	0.150	0.016	0.090

PPP1R13B	509.180	0.413	0.132	0.002	0.009	509.180	-0.078	0.133	0.559	0.749	509.180	-0.491	0.153	0.001	0.017
PUM2	1850.679	-0.605	0.177	0.001	0.004	1850.679	-0.045	0.176	0.799	0.895	1850.679	0.561	0.204	0.006	0.046
ARHGEF18	874.552	-0.212	0.156	0.173	0.294	874.552	-0.015	0.155	0.925	0.962	874.552	0.198	0.180	0.272	0.507
TNS2	2009.504	0.608	0.332	0.067	0.144	2009.504	0.619	0.332	0.062	0.350	2009.504	0.011	0.383	0.977	0.988
CRTC1	430.644	0.164	0.338	0.628	0.738	430.644	0.673	0.337	0.046	0.335	430.644	0.509	0.390	0.191	0.413
UFL1	956.117	-0.578	0.210	0.006	0.023	956.117	0.078	0.209	0.711	0.845	956.117	0.656	0.242	0.007	0.051
RRP8	389.854	-0.431	0.242	0.074	0.157	389.854	-0.305	0.241	0.204	0.487	389.854	0.126	0.279	0.653	0.813
ICE1	1394.126	-1.050	0.292	0.000	0.002	1394.126	-0.530	0.291	0.069	0.357	1394.126	0.520	0.337	0.123	0.315
SRGAP2	986.938	-0.347	0.176	0.048	0.112	986.938	0.004	0.175	0.982	0.989	986.938	0.351	0.203	0.083	0.250
SMG5	1362.181	-1.098	0.361	0.002	0.011	1362.181	-0.829	0.360	0.021	0.314	1362.181	0.269	0.417	0.519	0.722
AHCYL2	150.336	0.011	0.300	0.970	0.981	150.336	0.323	0.297	0.278	0.548	150.336	0.311	0.345	0.367	0.599
MAU2	936.188	-0.324	0.160	0.043	0.104	936.188	0.158	0.159	0.322	0.583	936.188	0.481	0.185	0.009	0.062
SPECC1L	443.962	-0.664	0.261	0.011	0.037	443.962	-0.525	0.260	0.044	0.331	443.962	0.138	0.302	0.647	0.809
NCSTN	1589.048	0.116	0.211	0.584	0.701	1589.048	0.294	0.211	0.163	0.454	1589.048	0.178	0.244	0.464	0.680
NUDCD3	1115.250	-0.215	0.153	0.161	0.280	1115.250	-0.108	0.153	0.481	0.700	1115.250	0.107	0.177	0.546	0.741
SIK3	344.352	-0.641	0.205	0.002	0.009	344.352	-0.036	0.202	0.859	0.928	344.352	0.606	0.237	0.010	0.068
MED13L	1398.856	-0.664	0.188	0.000	0.003	1398.856	-0.291	0.187	0.120	0.412	1398.856	0.373	0.217	0.086	0.255
FNTA	2114.725	0.033	0.106	0.754	0.836	2114.725	0.181	0.106	0.087	0.380	2114.725	0.148	0.122	0.227	0.455
ZDHHC17	305.776	-0.053	0.236	0.824	0.885	305.776	0.214	0.235	0.362	0.614	305.776	0.267	0.272	0.328	0.562
ECPAS	3637.111	-0.516	0.099	0.000	0.000	3637.111	-0.067	0.099	0.495	0.709	3637.111	0.449	0.114	0.000	0.002
ADNP	1512.466	-0.766	0.140	0.000	0.000	1512.466	-0.058	0.139	0.678	0.826	1512.466	0.708	0.162	0.000	0.000
LARS2	634.343	-0.551	0.262	0.035	0.089	634.343	-0.438	0.262	0.094	0.388	634.343	0.113	0.303	0.709	0.849
PIP5K1C	753.781	0.570	0.122	0.000	0.000	753.781	-0.113	0.123	0.358	0.611	753.781	-0.683	0.142	0.000	0.000
NCAPH	660.066	-0.660	0.298	0.027	0.072	660.066	-0.363	0.298	0.223	0.501	660.066	0.297	0.344	0.388	0.617
PPWD1	572.974	-0.581	0.136	0.000	0.000	572.974	-0.230	0.134	0.085	0.376	572.974	0.350	0.157	0.025	0.118
CTDNEP1	1390.832	-0.065	0.142	0.645	0.751	1390.832	0.192	0.141	0.175	0.464	1390.832	0.257	0.164	0.116	0.305
ATP13A2	659.871	0.385	0.223	0.084	0.172	659.871	-0.267	0.224	0.234	0.511	659.871	-0.652	0.258	0.012	0.072
FRAT2	325.496	-0.218	0.200	0.276	0.413	325.496	-0.052	0.199	0.795	0.893	325.496	0.166	0.231	0.472	0.686
FBXO46	406.529	0.490	0.244	0.044	0.105	406.529	0.370	0.243	0.129	0.421	406.529	-0.121	0.281	0.668	0.824
EXOSC2	569.413	-0.459	0.217	0.034	0.087	569.413	-0.385	0.216	0.075	0.364	569.413	0.074	0.251	0.768	0.882
DICER1	403.682	-0.377	0.265	0.155	0.271	403.682	-0.150	0.264	0.570	0.756	403.682	0.227	0.306	0.458	0.676
COTL1	5332.602	-0.138	0.277	0.617	0.728	5332.602	-0.318	0.277	0.251	0.525	5332.602	-0.179	0.320	0.575	0.761
SIRT5	350.985	0.084	0.213	0.695	0.790	350.985	0.222	0.212	0.297	0.564	350.985	0.138	0.246	0.575	0.761
SIRT4	21.132	1.186	0.908	0.191	0.316	21.132	0.659	0.913	0.470	0.693	21.132	-0.527	1.044	0.614	0.787
SIRT3	372.945	0.511	0.203	0.012	0.039	372.945	0.428	0.203	0.035	0.322	372.945	-0.083	0.234	0.724	0.857
SIRT1	433.260	-0.702	0.162	0.000	0.000	433.260	-0.130	0.158	0.412	0.651	433.260	0.573	0.187	0.002	0.023
COMMD3	303.012	0.758	0.243	0.002	0.009	303.012	0.317	0.244	0.194	0.478	303.012	-0.440	0.281	0.117	0.306
NCS1	1949.011	0.185	0.082	0.024	0.067	1949.011	-0.113	0.082	0.169	0.458	1949.011	-0.298	0.095	0.002	0.019
ZFPM2	175.334	-0.253	0.183	0.167	0.287	175.334	-0.070	0.180	0.699	0.838	175.334	0.184	0.212	0.386	0.615
KCNH3	15.840	1.702	0.492	0.001	0.004	15.840	0.073	0.529	0.890	0.945	15.840	-1.629	0.582	0.005	0.042
MLYCD	123.949	0.159	0.250	0.526	0.652	123.949	0.234	0.248	0.344	0.600	123.949	0.076	0.288	0.792	0.896
FNTB	36.001	-0.998	0.435	0.022	0.062	36.001	0.101	0.409	0.805	0.898	36.001	1.099	0.496	0.027	0.122
NOMO1	3600.805	-0.405	0.231	0.080	0.165	3600.805	0.006	0.231	0.981	0.989	3600.805	0.410	0.266	0.124	0.317
ITGB3BP	956.934	0.055	0.229	0.811	0.876	956.934	0.201	0.229	0.378	0.626	956.934	0.147	0.264	0.579	0.764
TMED3	1402.434	0.149	0.155	0.336	0.477	1402.434	-0.133	0.155	0.389	0.635	1402.434	-0.282	0.179	0.115	0.304
TDRD7	212.788	0.407	0.185	0.028	0.075	212.788	-0.025	0.187	0.892	0.946	212.788	-0.432	0.215	0.045	0.170
SLC7A8	98.186	1.410	0.349	0.000	0.001	98.186	0.269	0.355	0.448	0.677	98.186	-1.141	0.405	0.005	0.040
RYBP	1602.700	-0.351	0.266	0.188	0.312	1602.700	0.067	0.266	0.800	0.896	1602.700	0.418	0.307	0.174	0.390
AP4E1	138.636	-0.244	0.349	0.485	0.617	138.636	-1.029	0.353	0.004	0.297	138.636	-0.785	0.408	0.054	0.193
GPR161	304.306	0.372	0.267	0.163	0.283	304.306	0.144	0.267	0.590	0.772	304.306	-0.228	0.308	0.459	0.676
RHOQ	966.674	0.933	0.110	0.000	0.000	966.674	-0.140	0.112	0.213	0.493	966.674	-1.073	0.128	0.000	0.000
TARDBP	2410.870	-0.208	0.209	0.319	0.459	2410.870	-0.514	0.209	0.014	0.306	2410.870	-0.306	0.241	0.204	0.428
HARS2	566.165	-0.126	0.099	0.202	0.329	566.165	-0.072	0.098	0.459	0.685	566.165	0.054	0.114	0.638	0.803
SLC35A3	399.719	0.005	0.136	0.971	0.982	399.719	0.050	0.135	0.712	0.846	399.719	0.045	0.157	0.776	0.886
SLC44A1	3702.609	-0.323	0.263	0.220	0.350	3702.609	0.169	0.263	0.521	0.725	3702.609	0.492	0.304	0.105	0.289
SF3B3	4197.603	-0.388	0.097	0.000	0.001	4197.603	0.060	0.097	0.537	0.735	4197.603	0.448	0.112	0.000	0.002
SF3B1	3579.265	-0.489	0.159	0.002	0.010	3579.265	-0.223	0.158	0.159	0.451	3579.265	0.266	0.183	0.147	0.354
ABCB10	510.043	-0.646	0.257	0.012	0.039	510.043	-0.428	0.257	0.095	0.389	510.043	0.218	0.298	0.465	0.681
ABCB9	920.057	-0.031	0.105	0.769	0.847	920.057	0.200	0.104	0.054	0.342	920.057	0.231	0.121	0.056	0.196
FOLH1	32.608	-0.346	0.416	0.407	0.545	32.608	0.455	0.401	0.256	0.529	32.608	0.801	0.474	0.091	0.265
ABCA6	235.987	-1.450	0.235	0.000	0.000	235.987	-0.009	0.224	0.966	0.982	235.987	1.440	0.269	0.000	0.000
HEY1	101.468	1.029	0.241	0.000	0.000	101.468	0.018	0.257	0.946	0.973	101.468	-1.011	0.285	0.000	0.007
ICMT	1973.243	0.074	0.326	0.821	0.882	1973.243	-1.022	0.327	0.002	0.297	1973.243	-1.096	0.377	0.004	0.033
GCAT	464.959	0.130	0.215	0.546	0.670	464.959	-0.147	0.215	0.494	0.709	464.959	-0.277	0.249	0.265	0.500
CBX6	1857.074	0.569	0.166	0.001	0.004	1857.074	-0.091	0.167	0.585	0.768	1857.074	-0.660	0.192	0.001	0.009
NPTXR	44.034	2.230	0.441	0.000	0.000	44.034	0.511	0.458	0.265	0.537	44.034	-1.719	0.511	0.001	0.011
CBX5	1712.081	-0.087	0.176	0.622	0.732	1712.081	0.054	0.175	0.757	0.871	1712.081	0.141	0.203	0.487	0.698

PHF3	2393.297	-0.467	0.252	0.064	0.139	2393.297	0.197	0.252	0.434	0.667	2393.297	0.665	0.291	0.022	0.109
TRAM1	4262.716	-0.008	0.189	0.967	0.980	4262.716	-0.039	0.189	0.837	0.916	4262.716	-0.031	0.218	0.887	0.946
CAPN7	746.053	-0.507	0.162	0.002	0.009	746.053	-0.102	0.161	0.526	0.729	746.053	0.405	0.187	0.030	0.132
ETHE1	841.192	0.338	0.279	0.226	0.358	841.192	0.592	0.279	0.034	0.322	841.192	0.254	0.322	0.430	0.653
QPRT	34.106	0.899	0.305	0.003	0.014	34.106	0.394	0.312	0.206	0.488	34.106	-0.505	0.351	0.150	0.359
BRD4	2006.637	-0.231	0.197	0.242	0.375	2006.637	-0.203	0.197	0.303	0.569	2006.637	0.028	0.228	0.903	0.954
SEC11A	3243.480	0.373	0.191	0.051	0.117	3243.480	0.191	0.191	0.318	0.580	3243.480	-0.182	0.221	0.410	0.636
ISCU	809.631	-0.084	0.135	0.533	0.659	809.631	-0.004	0.134	0.974	0.986	809.631	0.079	0.155	0.609	0.784
SEC61G	1523.813	0.770	0.210	0.000	0.002	1523.813	0.297	0.211	0.159	0.451	1523.813	-0.474	0.243	0.051	0.186
PES1	3241.970	-0.296	0.246	0.229	0.360	3241.970	-0.316	0.245	0.198	0.481	3241.970	-0.020	0.284	0.943	0.974
TGDS	246.546	-0.693	0.256	0.007	0.025	246.546	-0.391	0.253	0.122	0.414	246.546	0.302	0.296	0.308	0.543
LEPROTL1	739.421	-0.439	0.236	0.063	0.137	739.421	-0.389	0.236	0.099	0.393	739.421	0.050	0.273	0.854	0.930
CES3	18.211	0.898	0.937	0.338	0.478	18.211	0.350	0.941	0.710	0.844	18.211	-0.547	1.082	0.613	0.787
CBX7	121.729	0.752	0.304	0.013	0.042	121.729	0.480	0.304	0.114	0.406	121.729	-0.272	0.350	0.437	0.658
TNFRSF13B	28.749	0.678	0.296	0.022	0.062	28.749	0.244	0.302	0.419	0.657	28.749	-0.434	0.343	0.206	0.430
MACF1	2503.963	-0.701	0.185	0.000	0.001	2503.963	-0.284	0.185	0.125	0.416	2503.963	0.417	0.214	0.051	0.186
DAAM2	8.272	2.115	0.552	0.000	0.001	8.272	0.995	0.589	0.091	0.385	8.272	-1.120	0.613	0.068	0.220
ZFYVE26	251.908	-0.243	0.213	0.254	0.388	251.908	-0.310	0.213	0.145	0.436	251.908	-0.067	0.247	0.786	0.893
TMEM131	998.709	-0.766	0.208	0.000	0.002	998.709	-0.229	0.207	0.269	0.540	998.709	0.537	0.240	0.025	0.117
BICRAL	360.431	-0.574	0.196	0.003	0.015	360.431	0.316	0.192	0.100	0.394	360.431	0.891	0.225	0.000	0.002
LRRC8B	217.251	-0.358	0.265	0.177	0.299	217.251	-0.605	0.266	0.023	0.315	217.251	-0.246	0.308	0.424	0.648
TTC9	7.550	0.084	0.575	0.883	0.924	7.550	0.091	0.563	0.872	0.935	7.550	0.006	0.663	0.992	0.996
POFUT1	1507.203	-0.337	0.150	0.024	0.067	1507.203	-0.282	0.149	0.059	0.349	1507.203	0.055	0.173	0.750	0.872
KCTD2	1757.344	0.991	0.207	0.000	0.000	1757.344	0.358	0.207	0.084	0.376	1757.344	-0.633	0.239	0.008	0.057
NUP188	1241.759	-0.748	0.326	0.022	0.061	1241.759	-0.926	0.326	0.004	0.297	1241.759	-0.178	0.377	0.636	0.802
SUZ12	1170.778	-0.819	0.217	0.000	0.001	1170.778	-0.508	0.216	0.019	0.306	1170.778	0.312	0.250	0.213	0.439
SCRIB	3257.957	-0.042	0.251	0.868	0.914	3257.957	-0.413	0.251	0.100	0.394	3257.957	-0.371	0.289	0.200	0.423
SPIDR	850.114	0.199	0.123	0.106	0.205	850.114	0.164	0.123	0.181	0.469	850.114	-0.035	0.142	0.807	0.903
MORC3	556.680	-0.614	0.230	0.007	0.027	556.680	0.041	0.228	0.859	0.928	556.680	0.655	0.265	0.013	0.079
SLC39A14	5810.494	-0.112	0.281	0.689	0.785	5810.494	0.032	0.281	0.911	0.955	5810.494	0.144	0.324	0.657	0.817
MTREX	3668.775	-0.727	0.145	0.000	0.000	3668.775	-0.392	0.145	0.007	0.297	3668.775	0.334	0.168	0.046	0.174
R3HDM1	927.941	-0.773	0.164	0.000	0.000	927.941	-0.263	0.163	0.107	0.399	927.941	0.510	0.190	0.007	0.053
FOLR3	12.056	3.171	0.655	0.000	0.000	12.056	-1.740	0.961	0.070	0.359	12.056	-4.910	0.994	0.000	0.000
ANP32C	18.109	-0.260	0.400	0.515	0.643	18.109	-0.252	0.392	0.521	0.725	18.109	0.008	0.468	0.986	0.992
RPL13A	129546.627	0.222	0.326	0.496	0.628	129546.627	0.536	0.326	0.100	0.394	129546.627	0.314	0.377	0.404	0.631
KAT6B	646.447	-0.523	0.249	0.036	0.090	646.447	0.101	0.248	0.684	0.829	646.447	0.624	0.287	0.030	0.132
CABIN1	1499.474	0.095	0.152	0.532	0.657	1499.474	0.160	0.152	0.294	0.561	1499.474	0.064	0.176	0.715	0.852
SRRM2	4960.201	0.052	0.196	0.793	0.863	4960.201	0.183	0.196	0.351	0.605	4960.201	0.131	0.227	0.562	0.753
ARHGAP45	1192.066	0.564	0.375	0.133	0.241	1192.066	0.628	0.375	0.094	0.388	1192.066	0.064	0.433	0.882	0.943
ACAP2	1044.506	-0.635	0.236	0.007	0.026	1044.506	-0.154	0.235	0.512	0.719	1044.506	0.481	0.272	0.077	0.239
ZNF281	520.517	-0.086	0.198	0.666	0.767	520.517	-0.404	0.199	0.042	0.330	520.517	-0.318	0.230	0.167	0.382
CLCF1	63.848	-0.188	0.394	0.633	0.741	63.848	-0.290	0.392	0.460	0.686	63.848	-0.102	0.458	0.824	0.913
FOS	277.120	0.693	0.227	0.002	0.011	277.120	0.492	0.228	0.031	0.322	277.120	-0.201	0.260	0.441	0.661
NNT	3296.376	-0.562	0.248	0.023	0.065	3296.376	0.030	0.248	0.902	0.951	3296.376	0.593	0.286	0.038	0.154
MMD	80.545	1.102	0.223	0.000	0.000	80.545	-0.173	0.237	0.466	0.690	80.545	-1.275	0.265	0.000	0.000
TNPO3	1238.736	-0.514	0.179	0.004	0.017	1238.736	-0.227	0.179	0.203	0.486	1238.736	0.287	0.207	0.166	0.381
ADAT1	233.709	-0.638	0.344	0.063	0.138	233.709	-0.911	0.344	0.008	0.297	233.709	-0.273	0.399	0.494	0.703
FOSB	42.489	-1.001	0.365	0.006	0.023	42.489	0.241	0.316	0.445	0.676	42.489	1.243	0.407	0.002	0.024
SEC14L2	645.029	0.285	0.318	0.369	0.510	645.029	0.330	0.317	0.298	0.566	645.029	0.045	0.367	0.903	0.955
RBFOX2	7495.303	-0.753	0.288	0.009	0.031	7495.303	0.282	0.287	0.327	0.588	7495.303	1.035	0.332	0.002	0.020
ATP6V0A2	334.233	-0.325	0.298	0.276	0.413	334.233	-0.537	0.298	0.072	0.361	334.233	-0.212	0.345	0.539	0.736
TTC33	469.808	-0.264	0.211	0.210	0.340	469.808	0.197	0.209	0.346	0.602	469.808	0.461	0.243	0.058	0.200
DNPEP	959.729	0.081	0.120	0.501	0.631	959.729	0.103	0.120	0.392	0.636	959.729	0.022	0.139	0.876	0.940
FOSL2	5923.560	-0.035	0.252	0.891	0.929	5923.560	0.256	0.252	0.311	0.575	5923.560	0.290	0.291	0.319	0.554
PSD4	150.676	0.418	0.354	0.238	0.371	150.676	0.326	0.354	0.357	0.611	150.676	-0.092	0.408	0.821	0.912
RASD2	14.074	1.236	0.857	0.149	0.263	14.074	1.021	0.857	0.234	0.511	14.074	-0.215	0.980	0.826	0.914
CDK20	61.427	0.097	0.333	0.770	0.847	61.427	0.278	0.329	0.398	0.642	61.427	0.180	0.383	0.638	0.803
TSPAN15	16.244	0.741	0.382	0.052	0.120	16.244	0.416	0.388	0.283	0.553	16.244	-0.325	0.437	0.456	0.675
PIGN	714.158	-0.497	0.187	0.008	0.028	714.158	-0.065	0.186	0.726	0.854	714.158	0.432	0.215	0.045	0.171
SNAPIN	531.414	0.173	0.161	0.282	0.419	531.414	0.171	0.160	0.286	0.555	531.414	-0.002	0.186	0.991	0.995
WBP2	2511.846	1.119	0.227	0.000	0.000	2511.846	0.380	0.228	0.095	0.389	2511.846	-0.738	0.263	0.005	0.041
WBP1	317.048	0.260	0.398	0.514	0.642	317.048	0.626	0.397	0.114	0.406	317.048	0.366	0.458	0.424	0.648
FPGS	1667.014	0.156	0.205	0.446	0.581	1667.014	-0.159	0.205	0.438	0.670	1667.014	-0.316	0.237	0.183	0.402
GTPBP4	1784.133	-1.346	0.367	0.000	0.002	1784.133	-0.895	0.366	0.015	0.306	1784.133	0.452	0.423	0.286	0.521
DDAH2	777.323	1.378	0.327	0.000	0.000	777.323	0.327	0.328	0.319	0.580	777.323	-1.051	0.378	0.005	0.043
LPAR3	11.681	1.434	0.717	0.046	0.107	11.681	0.574	0.741	0.438	0.671	11.681	-0.860	0.823	0.296	0.532
ZNF346	288.682	-0.194	0.175	0.267	0.403	288.682	-0.025	0.173	0.885	0.943	288.682	0.169	0.202	0.402	0.629

ARL2BP	1072.278	-0.275	0.178	0.123	0.228	1072.278	0.044	0.177	0.803	0.897	1072.278	0.319	0.206	0.121	0.312
DDAH1	152.903	0.628	0.215	0.004	0.015	152.903	-0.578	0.223	0.009	0.297	152.903	-1.206	0.254	0.000	0.000
CDC42EP4	1328.003	-0.454	0.170	0.008	0.027	1328.003	-0.085	0.169	0.616	0.788	1328.003	0.369	0.196	0.060	0.205
CASP14	12.018	-2.860	1.425	0.045	0.106	12.018	0.978	1.169	0.403	0.645	12.018	3.838	1.570	0.015	0.083
CCNDBP1	645.613	-0.200	0.194	0.301	0.439	645.613	0.231	0.192	0.231	0.508	645.613	0.431	0.223	0.053	0.191
SMUG1	430.839	0.112	0.152	0.461	0.596	430.839	-0.062	0.152	0.681	0.827	430.839	-0.174	0.176	0.321	0.556
TMEM50A	1857.635	0.384	0.193	0.047	0.110	1857.635	-0.327	0.194	0.091	0.386	1857.635	-0.712	0.224	0.001	0.018
DDX58	274.570	1.622	0.159	0.000	0.000	274.570	0.068	0.168	0.687	0.831	274.570	-1.554	0.187	0.000	0.000
ELP5	517.047	-0.474	0.122	0.000	0.001	517.047	-0.007	0.119	0.956	0.978	517.047	0.468	0.140	0.001	0.012
KLHDC2	616.100	-0.159	0.113	0.161	0.279	616.100	-0.044	0.112	0.698	0.838	616.100	0.115	0.131	0.378	0.609
CARHSP1	914.945	1.050	0.279	0.000	0.001	914.945	0.511	0.280	0.067	0.356	914.945	-0.538	0.322	0.095	0.271
PDSS1	189.308	-0.735	0.409	0.072	0.153	189.308	-0.780	0.408	0.056	0.344	189.308	-0.045	0.473	0.925	0.965
LEMD3	508.855	-0.703	0.240	0.003	0.015	508.855	-0.126	0.238	0.595	0.775	508.855	0.576	0.276	0.037	0.151
HEBP2	907.286	0.606	0.202	0.003	0.012	907.286	0.277	0.202	0.171	0.460	907.286	-0.330	0.233	0.158	0.370
ORC6	310.709	-0.494	0.394	0.210	0.339	310.709	-0.970	0.395	0.014	0.306	310.709	-0.476	0.457	0.297	0.532
ORC3	661.389	-0.032	0.234	0.890	0.929	661.389	-0.285	0.234	0.224	0.503	661.389	-0.252	0.270	0.351	0.584
OPN3	1822.431	-0.197	0.227	0.386	0.525	1822.431	0.141	0.227	0.535	0.734	1822.431	0.338	0.262	0.198	0.421
ACOT9	774.463	-0.316	0.219	0.149	0.263	774.463	-0.401	0.219	0.067	0.354	774.463	-0.085	0.253	0.735	0.864
PATZ1	487.174	-0.377	0.157	0.017	0.050	487.174	0.073	0.155	0.638	0.803	487.174	0.450	0.181	0.013	0.078
AMACR	169.939	-0.362	0.197	0.066	0.143	169.939	-0.258	0.195	0.186	0.473	169.939	0.104	0.229	0.648	0.810
CORO1C	3587.753	-0.272	0.108	0.012	0.038	3587.753	-0.186	0.108	0.083	0.375	3587.753	0.086	0.124	0.491	0.701
DAPK2	9.184	1.601	0.557	0.004	0.017	9.184	0.670	0.586	0.253	0.527	9.184	-0.931	0.635	0.143	0.348
CD2AP	995.535	-0.750	0.225	0.001	0.005	995.535	-0.161	0.224	0.471	0.693	995.535	0.588	0.260	0.023	0.112
MKRN1	2095.644	0.020	0.220	0.928	0.955	2095.644	0.282	0.220	0.200	0.483	2095.644	0.262	0.254	0.303	0.538
MKRN2	1546.289	-0.131	0.100	0.189	0.313	1546.289	-0.032	0.099	0.745	0.864	1546.289	0.099	0.115	0.391	0.620
PHLDA3	1711.170	0.973	0.321	0.002	0.011	1711.170	0.466	0.321	0.147	0.438	1711.170	-0.507	0.371	0.172	0.389
ZMYND8	3072.284	-0.144	0.252	0.568	0.688	3072.284	0.358	0.252	0.155	0.447	3072.284	0.502	0.291	0.084	0.252
SH3BP1	306.999	0.402	0.190	0.034	0.087	306.999	0.000	0.191	0.998	0.999	306.999	-0.402	0.220	0.067	0.219
TSSK2	15.409	0.098	0.469	0.835	0.891	15.409	-0.328	0.478	0.494	0.709	15.409	-0.425	0.555	0.443	0.663
BACE1	929.830	-0.084	0.189	0.658	0.761	929.830	0.081	0.189	0.667	0.819	929.830	0.165	0.219	0.451	0.670
RUSC1	517.861	0.049	0.166	0.767	0.846	517.861	-0.056	0.165	0.736	0.859	517.861	-0.105	0.191	0.584	0.766
FAM89B	534.399	1.197	0.258	0.000	0.000	534.399	0.481	0.259	0.064	0.350	534.399	-0.717	0.298	0.016	0.089
CA14	10.157	0.012	0.534	0.983	0.990	10.157	-0.206	0.536	0.701	0.839	10.157	-0.217	0.628	0.729	0.859
KPNA6	1614.089	0.384	0.136	0.005	0.019	1614.089	0.028	0.136	0.837	0.916	1614.089	-0.356	0.157	0.023	0.111
SSBP2	1157.649	-0.454	0.228	0.047	0.110	1157.649	0.206	0.227	0.364	0.616	1157.649	0.660	0.263	0.012	0.074
NUP62	1745.183	-0.056	0.108	0.603	0.716	1745.183	-0.067	0.107	0.533	0.733	1745.183	-0.011	0.125	0.929	0.968
RABGAP1	824.377	-0.384	0.240	0.110	0.210	824.377	0.024	0.240	0.920	0.960	824.377	0.408	0.277	0.141	0.345
HSPBP1	1021.382	-0.134	0.299	0.653	0.757	1021.382	-0.409	0.299	0.172	0.461	1021.382	-0.274	0.346	0.427	0.651
SNHG1	1315.432	-1.380	0.184	0.000	0.000	1315.432	0.237	0.181	0.192	0.477	1315.432	1.617	0.212	0.000	0.000
LY96	61.140	1.973	0.318	0.000	0.000	61.140	0.664	0.344	0.054	0.342	61.140	-1.310	0.363	0.000	0.006
EDC4	1240.669	-0.373	0.169	0.027	0.073	1240.669	-0.124	0.168	0.461	0.687	1240.669	0.249	0.195	0.202	0.425
PPP1R15A	3404.595	-0.633	0.402	0.115	0.217	3404.595	-0.895	0.402	0.026	0.316	3404.595	-0.262	0.464	0.572	0.760
PLD3	3652.810	0.787	0.260	0.002	0.011	3652.810	0.470	0.260	0.070	0.359	3652.810	-0.317	0.300	0.290	0.526
ARFIP2	1048.638	0.109	0.172	0.529	0.655	1048.638	-0.185	0.173	0.283	0.552	1048.638	-0.294	0.199	0.141	0.345
SSBP3	468.747	0.559	0.187	0.003	0.012	468.747	-0.124	0.188	0.511	0.718	468.747	-0.683	0.216	0.002	0.019
POLA2	297.522	-0.540	0.413	0.192	0.316	297.522	-0.958	0.414	0.021	0.312	297.522	-0.418	0.479	0.383	0.612
TRIM29	1516.458	-0.402	0.720	0.577	0.695	1516.458	0.594	0.719	0.409	0.649	1516.458	0.996	0.831	0.231	0.459
PLXNB2	6531.614	0.152	0.178	0.394	0.532	6531.614	0.346	0.178	0.052	0.341	6531.614	0.194	0.206	0.347	0.581
SLC7A11	2876.397	-2.091	0.305	0.000	0.000	2876.397	-0.729	0.304	0.016	0.306	2876.397	1.362	0.352	0.000	0.003
LSM5	367.780	0.409	0.208	0.050	0.115	367.780	-0.211	0.209	0.314	0.577	367.780	-0.619	0.241	0.010	0.067
PLA2G15	277.132	0.639	0.205	0.002	0.009	277.132	0.189	0.206	0.359	0.611	277.132	-0.450	0.237	0.058	0.200
ZKSCAN5	236.455	-0.527	0.168	0.002	0.009	236.455	-0.315	0.165	0.056	0.344	236.455	0.212	0.195	0.276	0.511
UBBP4	649.253	-0.331	0.473	0.484	0.617	649.253	0.768	0.473	0.104	0.397	649.253	1.099	0.546	0.044	0.169
CEMIP2	667.442	0.128	0.166	0.442	0.578	667.442	0.012	0.166	0.941	0.970	667.442	-0.115	0.192	0.548	0.742
STX12	628.144	0.140	0.160	0.383	0.523	628.144	0.074	0.160	0.643	0.805	628.144	-0.066	0.185	0.723	0.856
SH3BP4	1046.971	-0.293	0.137	0.032	0.082	1046.971	-0.151	0.136	0.268	0.539	1046.971	0.142	0.158	0.367	0.600
SGK3	68.932	0.650	0.252	0.010	0.034	68.932	-0.120	0.260	0.645	0.806	68.932	-0.770	0.296	0.009	0.062
PRKD3	832.496	-0.694	0.259	0.007	0.027	832.496	0.042	0.257	0.869	0.933	832.496	0.736	0.298	0.014	0.080
KCNE4	182.732	0.515	0.375	0.170	0.290	182.732	0.399	0.375	0.286	0.555	182.732	-0.115	0.432	0.789	0.895
CADM1	399.100	-0.701	0.223	0.002	0.008	399.100	0.161	0.219	0.462	0.688	399.100	0.862	0.256	0.001	0.011
GABARAPL1	652.844	0.486	0.234	0.038	0.093	652.844	0.447	0.234	0.056	0.343	652.844	-0.039	0.270	0.884	0.945
SHPK	208.939	-0.540	0.213	0.011	0.037	208.939	-0.028	0.209	0.894	0.947	208.939	0.512	0.245	0.037	0.151
TMEM245	1909.189	-0.080	0.260	0.759	0.840	1909.189	0.226	0.259	0.384	0.630	1909.189	0.305	0.300	0.308	0.544
EID1	2518.314	0.124	0.114	0.278	0.415	2518.314	-0.095	0.114	0.404	0.646	2518.314	-0.219	0.132	0.097	0.275
SDF2L1	607.268	0.391	0.487	0.422	0.559	607.268	-1.046	0.489	0.032	0.322	607.268	-1.437	0.564	0.011	0.069
PPIL2	478.369	-0.179	0.272	0.511	0.640	478.369	-0.487	0.272	0.073	0.362	478.369	-0.309	0.315	0.327	0.561
PITPNB	1759.435	-1.008	0.281	0.000	0.002	1759.435	-0.578	0.281	0.040	0.327	1759.435	0.430	0.325	0.186	0.406

PISD	631.551	-0.170	0.140	0.223	0.354	631.551	-0.128	0.139	0.358	0.611	631.551	0.042	0.162	0.794	0.896
MAFF	1000.719	-0.280	0.335	0.404	0.542	1000.719	-0.609	0.335	0.069	0.357	1000.719	-0.330	0.387	0.394	0.622
IL17RA	657.862	0.310	0.124	0.013	0.040	657.862	0.159	0.124	0.200	0.483	657.862	-0.151	0.144	0.292	0.528
FLRT3	81.398	-1.983	0.390	0.000	0.000	81.398	0.120	0.362	0.740	0.861	81.398	2.103	0.443	0.000	0.000
FLRT2	538.967	-0.363	0.197	0.065	0.141	538.967	0.162	0.195	0.405	0.646	538.967	0.525	0.227	0.020	0.103
FLRT1	51.226	-1.501	0.486	0.002	0.010	51.226	0.616	0.454	0.175	0.464	51.226	2.117	0.551	0.000	0.003
FKBP8	6711.481	0.718	0.288	0.013	0.040	6711.481	0.567	0.288	0.049	0.337	6711.481	-0.152	0.332	0.648	0.810
BRD1	697.495	-0.405	0.126	0.001	0.007	697.495	-0.186	0.125	0.136	0.427	697.495	0.219	0.146	0.134	0.333
APOL2	1284.856	1.006	0.393	0.010	0.035	1284.856	0.304	0.393	0.439	0.671	1284.856	-0.702	0.454	0.122	0.313
BCL2L13	1480.067	-0.497	0.099	0.000	0.000	1480.067	-0.068	0.098	0.486	0.703	1480.067	0.429	0.114	0.000	0.004
MTCH1	2907.863	0.203	0.108	0.060	0.132	2907.863	0.045	0.108	0.678	0.826	2907.863	-0.158	0.125	0.204	0.428
MTCH2	1729.281	0.059	0.244	0.810	0.875	1729.281	-0.513	0.245	0.036	0.324	1729.281	-0.572	0.283	0.043	0.166
ALOX12	30.971	0.330	0.461	0.474	0.608	30.971	-0.031	0.466	0.946	0.973	30.971	-0.362	0.537	0.500	0.707
FXN	127.109	-0.089	0.256	0.729	0.816	127.109	-0.043	0.254	0.865	0.931	127.109	0.046	0.296	0.877	0.941
KIF4A	989.528	-0.856	0.171	0.000	0.000	989.528	0.037	0.170	0.830	0.912	989.528	0.893	0.198	0.000	0.000
IFIT5	230.627	0.222	0.149	0.136	0.246	230.627	-0.283	0.151	0.061	0.350	230.627	-0.504	0.174	0.004	0.034
EML2	1213.480	0.428	0.151	0.005	0.018	1213.480	0.266	0.151	0.078	0.369	1213.480	-0.162	0.174	0.352	0.585
FTSJ1	834.993	-0.476	0.250	0.057	0.127	834.993	-0.391	0.250	0.117	0.409	834.993	0.085	0.289	0.768	0.882
NAA80	268.298	0.740	0.247	0.003	0.013	268.298	0.495	0.247	0.046	0.334	268.298	-0.245	0.285	0.389	0.618
TFIP11	680.908	-0.205	0.181	0.257	0.391	680.908	-0.353	0.181	0.051	0.338	680.908	-0.148	0.209	0.480	0.693
PANX1	1305.918	-0.259	0.319	0.416	0.554	1305.918	-0.326	0.319	0.306	0.572	1305.918	-0.067	0.368	0.856	0.931
CLDN15	74.330	0.735	0.239	0.002	0.010	74.330	0.255	0.243	0.294	0.561	74.330	-0.480	0.277	0.083	0.250
FJX1	1657.101	-0.543	0.149	0.000	0.002	1657.101	-0.180	0.149	0.226	0.504	1657.101	0.363	0.172	0.035	0.146
PRPF6	1519.883	0.355	0.171	0.038	0.094	1519.883	-0.238	0.172	0.165	0.455	1519.883	-0.593	0.198	0.003	0.027
ZNF318	593.855	-0.337	0.276	0.222	0.353	593.855	0.181	0.275	0.511	0.719	593.855	0.518	0.319	0.104	0.287
TP53TG3	12.528	0.202	1.051	0.848	0.900	12.528	1.134	1.034	0.273	0.544	12.528	0.932	1.201	0.438	0.659
FRK	8.973	0.728	0.548	0.184	0.308	8.973	0.515	0.549	0.349	0.604	8.973	-0.213	0.626	0.734	0.862
SPDYA	60.129	0.726	0.356	0.042	0.101	60.129	-0.195	0.365	0.593	0.774	60.129	-0.921	0.417	0.027	0.123
CNPY4	79.788	1.041	0.291	0.000	0.003	79.788	-0.277	0.303	0.361	0.613	79.788	-1.318	0.343	0.000	0.003
ATP6V1C2	1996.557	0.349	0.182	0.055	0.124	1996.557	-0.109	0.182	0.551	0.744	1996.557	-0.457	0.210	0.030	0.131
TXLNGY	21.020	0.116	0.403	0.774	0.850	21.020	0.250	0.393	0.525	0.728	21.020	0.134	0.461	0.771	0.883
CNOT6L	459.053	-0.583	0.207	0.005	0.019	459.053	-0.024	0.205	0.907	0.954	459.053	0.559	0.239	0.019	0.099
CDC26	279.148	-0.016	0.200	0.934	0.959	279.148	0.003	0.199	0.986	0.992	279.148	0.020	0.231	0.931	0.969
RNASEH1	744.320	-0.694	0.315	0.028	0.074	744.320	-0.812	0.315	0.010	0.298	744.320	-0.118	0.365	0.746	0.869
AFG1L	150.862	-0.439	0.278	0.114	0.216	150.862	0.382	0.273	0.161	0.452	150.862	0.822	0.319	0.010	0.066
STAC3	10.585	1.806	0.622	0.004	0.016	10.585	1.300	0.630	0.039	0.327	10.585	-0.506	0.694	0.466	0.681
PELI3	210.029	0.597	0.206	0.004	0.016	210.029	0.149	0.207	0.473	0.695	210.029	-0.448	0.238	0.060	0.205
POLR2J2	106.845	0.287	0.375	0.445	0.580	106.845	-0.196	0.377	0.604	0.781	106.845	-0.482	0.435	0.267	0.502
ALOX15B	10.585	-0.611	1.171	0.602	0.715	10.585	1.196	1.133	0.291	0.559	10.585	1.807	1.334	0.176	0.393
MTOR	998.073	-0.147	0.256	0.566	0.686	998.073	-0.699	0.257	0.007	0.297	998.073	-0.552	0.297	0.063	0.211
FRG1	650.813	0.330	0.329	0.317	0.457	650.813	-0.486	0.330	0.141	0.432	650.813	-0.816	0.381	0.032	0.138
CENPI	133.771	-0.636	0.297	0.032	0.083	133.771	-0.354	0.294	0.228	0.506	133.771	0.282	0.343	0.411	0.637
NR5A2	15.394	0.704	0.995	0.479	0.613	15.394	0.953	0.979	0.330	0.590	15.394	0.250	1.125	0.824	0.914
FTH1	241154.464	1.090	0.242	0.000	0.000	241154.464	0.557	0.242	0.021	0.314	241154.464	-0.534	0.279	0.056	0.197
ABL1	4790.306	0.002	0.125	0.988	0.993	4790.306	0.118	0.125	0.343	0.600	4790.306	0.117	0.144	0.419	0.643
FTL	81451.594	1.304	0.173	0.000	0.000	81451.594	0.040	0.173	0.819	0.905	81451.594	-1.264	0.200	0.000	0.000
FUCA1	241.120	1.223	0.270	0.000	0.000	241.120	0.600	0.272	0.027	0.320	241.120	-0.623	0.312	0.046	0.172
FUCA2	2488.557	-0.020	0.163	0.901	0.936	2488.557	-0.129	0.163	0.428	0.663	2488.557	-0.109	0.188	0.563	0.754
FUS	4997.191	-0.393	0.399	0.324	0.464	4997.191	-1.152	0.399	0.004	0.297	4997.191	-0.759	0.461	0.099	0.279
FUT1	71.552	-2.163	0.447	0.000	0.000	71.552	-0.350	0.419	0.404	0.646	71.552	1.813	0.510	0.000	0.007
FUT2	11.147	0.626	0.568	0.270	0.407	11.147	-0.019	0.586	0.974	0.986	11.147	-0.645	0.666	0.333	0.567
FUT4	38.213	-0.023	0.269	0.932	0.957	38.213	0.111	0.260	0.671	0.822	38.213	0.133	0.309	0.666	0.823
TMEM9	1116.119	0.558	0.228	0.014	0.045	1116.119	0.410	0.228	0.072	0.361	1116.119	-0.148	0.263	0.574	0.761
NEIL2	242.476	-0.172	0.262	0.511	0.640	242.476	-0.170	0.261	0.515	0.721	242.476	0.002	0.303	0.994	0.996
STXBP4	160.138	-0.037	0.230	0.873	0.919	160.138	-0.202	0.230	0.379	0.626	160.138	-0.166	0.267	0.535	0.733
FUT8	325.934	-0.028	0.188	0.884	0.925	325.934	0.119	0.187	0.523	0.726	325.934	0.147	0.217	0.499	0.706
HCG27	15.616	2.621	1.024	0.011	0.035	15.616	0.967	1.039	0.352	0.606	15.616	-1.654	1.177	0.160	0.373
CUTALP	242.078	-0.448	0.242	0.064	0.140	242.078	0.355	0.238	0.136	0.427	242.078	0.803	0.278	0.004	0.034
KDSR	1599.289	-0.113	0.182	0.535	0.660	1599.289	-0.291	0.182	0.111	0.405	1599.289	-0.177	0.211	0.401	0.628
PRR14L	344.553	-0.340	0.189	0.072	0.152	344.553	-0.269	0.188	0.153	0.445	344.553	0.071	0.219	0.744	0.869
EPHX4	33.200	-0.004	0.368	0.991	0.995	33.200	-0.100	0.366	0.786	0.888	33.200	-0.095	0.427	0.823	0.913
SERHL2	23.021	0.198	0.426	0.642	0.749	23.021	-0.426	0.437	0.330	0.590	23.021	-0.624	0.503	0.215	0.441
RICTOR	689.297	-0.157	0.199	0.430	0.566	689.297	-0.056	0.199	0.779	0.883	689.297	0.102	0.230	0.659	0.817
FYN	1000.984	0.207	0.172	0.229	0.360	1000.984	0.366	0.171	0.033	0.322	1000.984	0.159	0.198	0.422	0.646
IPMK	703.681	-0.193	0.269	0.474	0.607	703.681	0.160	0.269	0.552	0.745	703.681	0.353	0.311	0.257	0.491
ZBTB38	1417.388	-1.247	0.246	0.000	0.000	1417.388	-0.078	0.245	0.749	0.866	1417.388	1.168	0.284	0.000	0.001
FZD2	103.983	1.047	0.496	0.035	0.088	103.983	0.629	0.497	0.206	0.488	103.983	-0.418	0.572	0.465	0.681

SLC25A30	300.160	-0.657	0.337	0.051	0.117	300.160	0.264	0.334	0.429	0.664	300.160	0.921	0.388	0.018	0.094
LCLAT1	168.092	-0.143	0.171	0.401	0.540	168.092	-0.249	0.170	0.143	0.435	168.092	-0.106	0.199	0.595	0.774
CADM2	58.869	0.579	0.781	0.458	0.593	58.869	0.433	0.781	0.579	0.764	58.869	-0.146	0.901	0.871	0.938
GPATCH11	234.566	-0.674	0.223	0.002	0.011	234.566	-0.617	0.221	0.005	0.297	234.566	0.058	0.259	0.823	0.913
ZNF620	11.935	0.715	0.518	0.168	0.288	11.935	0.479	0.520	0.357	0.611	11.935	-0.236	0.592	0.690	0.837
ANKRD18A	23.827	0.234	0.447	0.601	0.715	23.827	-0.279	0.454	0.539	0.736	23.827	-0.513	0.524	0.327	0.562
IFI6	409.714	1.661	0.281	0.000	0.000	409.714	0.525	0.282	0.063	0.350	409.714	-1.136	0.324	0.000	0.007
MMS22L	362.820	-0.965	0.255	0.000	0.001	362.820	-0.526	0.253	0.038	0.327	362.820	0.440	0.295	0.136	0.338
TTC41P	10.657	-0.134	0.644	0.835	0.891	10.657	0.179	0.626	0.775	0.881	10.657	0.313	0.738	0.672	0.825
WASHC2C	710.276	0.237	0.203	0.243	0.376	710.276	0.155	0.203	0.444	0.675	710.276	-0.082	0.234	0.727	0.859
WDR27	171.552	0.037	0.273	0.893	0.931	171.552	0.514	0.270	0.057	0.346	171.552	0.477	0.314	0.128	0.324
CERS6	2027.632	-0.655	0.236	0.006	0.021	2027.632	0.006	0.235	0.981	0.989	2027.632	0.660	0.272	0.015	0.086
MSRB3	862.801	0.149	0.197	0.450	0.586	862.801	0.010	0.197	0.958	0.978	862.801	-0.138	0.227	0.543	0.740
ZDHHC20	945.556	-0.295	0.217	0.174	0.295	945.556	0.013	0.216	0.954	0.977	945.556	0.307	0.250	0.219	0.446
G6PD	3227.114	0.758	0.254	0.003	0.013	3227.114	-0.331	0.254	0.194	0.478	3227.114	-1.088	0.294	0.000	0.004
YTHDF3	1153.090	-0.676	0.231	0.003	0.015	1153.090	-0.172	0.230	0.455	0.682	1153.090	0.504	0.267	0.059	0.202
RALGAPA1	348.957	-0.737	0.229	0.001	0.007	348.957	-0.228	0.227	0.314	0.577	348.957	0.509	0.264	0.054	0.193
KCTD13	316.007	0.651	0.188	0.001	0.004	316.007	0.082	0.190	0.667	0.819	316.007	-0.569	0.218	0.009	0.061
ASPHD1	1133.966	0.891	0.175	0.000	0.000	1133.966	0.101	0.176	0.565	0.753	1133.966	-0.790	0.202	0.000	0.002
ETFBKMT	29.186	0.373	0.305	0.221	0.352	29.186	0.115	0.307	0.708	0.843	29.186	-0.258	0.354	0.465	0.681
METAP1D	87.685	-0.530	0.205	0.010	0.034	87.685	0.558	0.188	0.003	0.297	87.685	1.088	0.230	0.000	0.000
UBN2	145.085	0.532	0.244	0.030	0.078	145.085	0.409	0.244	0.093	0.388	145.085	-0.122	0.281	0.664	0.821
BRWD3	504.261	-0.722	0.306	0.018	0.054	504.261	0.176	0.304	0.563	0.751	504.261	0.898	0.353	0.011	0.070
ZNRD2-AS1	9.794	0.002	0.508	0.997	0.998	9.794	-0.155	0.506	0.759	0.872	9.794	-0.158	0.597	0.791	0.896
EHBP1L1	1667.643	0.203	0.157	0.195	0.321	1667.643	-0.076	0.157	0.629	0.797	1667.643	-0.278	0.181	0.124	0.317
SNX32	2309.066	0.245	0.223	0.272	0.408	2309.066	-0.371	0.223	0.096	0.391	2309.066	-0.616	0.258	0.017	0.092
NIFK-AS1	702.309	-0.431	0.235	0.067	0.144	702.309	-0.472	0.234	0.044	0.332	702.309	-0.042	0.272	0.877	0.941
CXorf58	8.271	-0.306	0.668	0.648	0.753	8.271	-0.280	0.659	0.670	0.822	8.271	0.025	0.779	0.974	0.987
FBXO33	239.435	-0.148	0.166	0.372	0.513	239.435	-0.256	0.165	0.122	0.413	239.435	-0.108	0.193	0.576	0.761
SLC37A4	874.437	0.150	0.103	0.146	0.259	874.437	0.179	0.102	0.080	0.371	874.437	0.029	0.119	0.804	0.902
RNF169	604.876	-0.308	0.212	0.146	0.259	604.876	0.274	0.210	0.192	0.477	604.876	0.582	0.244	0.017	0.092
CALHM5	67.445	0.658	0.305	0.031	0.081	67.445	-0.238	0.314	0.447	0.677	67.445	-0.896	0.358	0.012	0.075
LCORL	456.361	0.048	0.152	0.754	0.836	456.361	0.072	0.151	0.635	0.801	456.361	0.024	0.175	0.890	0.948
AKNAD1	13.623	0.392	0.571	0.492	0.624	13.623	0.588	0.548	0.283	0.552	13.623	0.196	0.638	0.759	0.877
ZDHHC24	410.209	0.882	0.191	0.000	0.000	410.209	0.356	0.192	0.063	0.350	410.209	-0.525	0.220	0.017	0.092
MCM9	169.292	-0.841	0.185	0.000	0.000	169.292	-0.319	0.178	0.073	0.362	169.292	0.523	0.213	0.014	0.082
PROSER2	693.284	-0.567	0.163	0.001	0.003	693.284	-0.275	0.162	0.091	0.385	693.284	0.292	0.189	0.121	0.313
SLC41A1	1073.776	-0.519	0.224	0.020	0.058	1073.776	-0.081	0.223	0.718	0.849	1073.776	0.438	0.258	0.090	0.262
LPCAT4	497.641	0.958	0.178	0.000	0.000	497.641	0.329	0.179	0.067	0.355	497.641	-0.629	0.206	0.002	0.024
NUDT8	81.156	0.646	0.285	0.023	0.065	81.156	-0.134	0.294	0.649	0.808	81.156	-0.780	0.335	0.020	0.101
XRCC6	6755.217	-0.569	0.230	0.013	0.042	6755.217	-0.516	0.230	0.025	0.316	6755.217	0.053	0.265	0.842	0.923
GAA	3072.364	0.752	0.385	0.051	0.117	3072.364	0.580	0.385	0.132	0.424	3072.364	-0.172	0.445	0.699	0.843
NAALADL2	15.867	-0.409	0.953	0.668	0.769	15.867	0.263	0.941	0.780	0.884	15.867	0.672	1.097	0.540	0.737
TMEM256	83.633	1.308	0.429	0.002	0.011	83.633	0.818	0.431	0.058	0.347	83.633	-0.490	0.492	0.320	0.555
ZDHHC23	66.251	0.179	0.315	0.570	0.689	66.251	0.005	0.315	0.987	0.992	66.251	-0.174	0.365	0.633	0.801
LOC254896	59.015	0.538	0.603	0.372	0.512	59.015	0.529	0.602	0.380	0.626	59.015	-0.009	0.694	0.989	0.994
GAB1	249.289	-0.259	0.385	0.501	0.631	249.289	0.258	0.384	0.500	0.713	249.289	0.518	0.444	0.244	0.475
GABBR1	496.548	1.216	0.338	0.000	0.002	496.548	0.507	0.339	0.135	0.426	496.548	-0.709	0.391	0.070	0.225
LINC00957	11.021	0.973	0.720	0.176	0.298	11.021	0.943	0.716	0.188	0.474	11.021	-0.030	0.816	0.970	0.986
TMEM86B	68.213	1.039	0.344	0.003	0.012	68.213	0.364	0.351	0.300	0.566	68.213	-0.675	0.398	0.090	0.262
CBARP	322.183	0.362	0.298	0.224	0.355	322.183	-0.235	0.300	0.433	0.667	322.183	-0.597	0.345	0.084	0.251
CASC2	86.298	-0.669	0.302	0.027	0.072	86.298	-0.095	0.295	0.748	0.866	86.298	0.574	0.348	0.099	0.278
GABPA	825.572	-0.356	0.138	0.010	0.035	825.572	-0.141	0.138	0.305	0.571	825.572	0.215	0.160	0.180	0.398
TMCO4	240.426	0.087	0.153	0.570	0.689	240.426	0.021	0.152	0.890	0.945	240.426	-0.066	0.177	0.710	0.849
IGFBP7-AS1	156.727	1.969	0.359	0.000	0.000	156.727	-0.463	0.369	0.209	0.490	156.727	-2.432	0.420	0.000	0.000
LINC01018	231.917	-0.897	0.407	0.027	0.073	231.917	-1.206	0.407	0.003	0.297	231.917	-0.309	0.472	0.513	0.718
GOLGA2P11	13.544	2.643	0.762	0.001	0.003	13.544	0.911	0.788	0.248	0.522	13.544	-1.732	0.872	0.047	0.176
MCOLN2	135.745	0.839	0.185	0.000	0.000	135.745	0.078	0.189	0.681	0.827	135.745	-0.761	0.215	0.000	0.007
LRRC57	326.036	-0.207	0.163	0.206	0.334	326.036	-0.229	0.163	0.160	0.451	326.036	-0.022	0.190	0.907	0.957
GABPB1	371.856	-1.249	0.334	0.000	0.002	371.856	-0.872	0.332	0.009	0.297	371.856	0.377	0.386	0.329	0.563
EIF2S3B	294.941	-0.499	0.237	0.035	0.089	294.941	0.409	0.233	0.079	0.370	294.941	0.909	0.272	0.001	0.012
EPGN	95.155	-2.952	0.744	0.000	0.001	95.155	-2.966	0.742	0.000	0.092	95.155	-0.014	0.868	0.987	0.993
MBLAC1	28.261	0.115	0.365	0.753	0.835	28.261	-0.217	0.370	0.557	0.747	28.261	-0.332	0.429	0.439	0.660
TCP11L2	83.242	-0.552	0.526	0.294	0.432	83.242	0.407	0.515	0.429	0.664	83.242	0.959	0.603	0.112	0.299
RNF144B	1081.932	-0.193	0.211	0.361	0.501	1081.932	-0.505	0.211	0.017	0.306	1081.932	-0.312	0.244	0.201	0.425
TOLLIP-AS1	31.476	0.685	0.428	0.109	0.209	31.476	0.162	0.434	0.709	0.844	31.476	-0.524	0.496	0.291	0.526
ELMOD2	349.450	-0.321	0.242	0.185	0.309	349.450	-0.021	0.241	0.931	0.965	349.450	0.300	0.279	0.283	0.517

GABRA3	158.053	-0.083	0.363	0.819	0.882	158.053	0.343	0.361	0.342	0.599	158.053	0.426	0.418	0.308	0.544
PCSK9	52.396	5.878	0.465	0.000	0.000	52.396	-0.233	0.636	0.714	0.847	52.396	-6.111	0.618	0.000	0.000
NPNT	16.794	-0.028	0.430	0.949	0.969	16.794	0.643	0.397	0.105	0.398	16.794	0.671	0.479	0.161	0.375
TCTEX1D2	61.493	0.019	0.247	0.939	0.962	61.493	0.254	0.242	0.293	0.561	61.493	0.235	0.283	0.407	0.634
INAFM1	314.816	1.185	0.252	0.000	0.000	314.816	0.520	0.253	0.040	0.327	314.816	-0.665	0.290	0.022	0.108
SDHAP1	286.210	0.381	0.383	0.320	0.460	286.210	0.453	0.382	0.236	0.513	286.210	0.072	0.442	0.871	0.938
CNEP1R1	194.968	0.283	0.154	0.067	0.144	194.968	0.166	0.154	0.281	0.551	194.968	-0.117	0.178	0.512	0.717
PAN3	396.302	-0.383	0.204	0.061	0.134	396.302	0.161	0.202	0.424	0.660	396.302	0.544	0.235	0.021	0.103
ZNF549	88.809	-0.604	0.283	0.033	0.085	88.809	-0.249	0.277	0.369	0.619	88.809	0.355	0.328	0.279	0.513
GABRB2	8.496	0.057	0.633	0.928	0.955	8.496	-0.471	0.648	0.468	0.691	8.496	-0.528	0.750	0.482	0.694
SYCE2	68.263	0.208	0.229	0.363	0.504	68.263	-0.363	0.236	0.123	0.416	68.263	-0.571	0.271	0.035	0.147
HMCN2	10.936	-0.347	0.889	0.696	0.790	10.936	0.280	0.871	0.748	0.866	10.936	0.627	1.021	0.539	0.736
GABRB3	60.875	-0.674	0.439	0.125	0.231	60.875	0.556	0.425	0.191	0.477	60.875	1.230	0.502	0.014	0.082
NUDT14	293.747	1.483	0.433	0.001	0.004	293.747	0.833	0.434	0.055	0.342	293.747	-0.650	0.499	0.193	0.415
NATD1	113.862	0.783	0.529	0.139	0.249	113.862	0.894	0.528	0.090	0.384	113.862	0.112	0.609	0.854	0.930
CCDC110	24.042	0.458	0.698	0.512	0.641	24.042	0.873	0.691	0.206	0.488	24.042	0.416	0.799	0.603	0.780
LMNTD2	53.872	1.092	0.586	0.062	0.137	53.872	1.307	0.583	0.025	0.316	53.872	0.215	0.671	0.748	0.871
GK5	96.635	0.221	0.261	0.397	0.535	96.635	-0.438	0.266	0.100	0.394	96.635	-0.659	0.306	0.031	0.136
EML3	1411.335	0.081	0.106	0.448	0.584	1411.335	0.220	0.106	0.038	0.327	1411.335	0.139	0.123	0.256	0.490
GABRE	59.292	0.675	0.318	0.034	0.086	59.292	-0.022	0.324	0.945	0.973	59.292	-0.697	0.371	0.060	0.205
ST6GALNAC3	272.140	-1.629	0.333	0.000	0.000	272.140	-0.484	0.328	0.140	0.431	272.140	1.145	0.384	0.003	0.028
MFSD8	108.605	-0.162	0.207	0.432	0.569	108.605	-0.264	0.206	0.200	0.483	108.605	-0.101	0.242	0.675	0.828
GABRG1	23.082	-0.812	0.445	0.068	0.146	23.082	0.208	0.411	0.613	0.786	23.082	1.020	0.504	0.043	0.166
LYSMD2	59.828	0.739	0.217	0.001	0.004	59.828	0.487	0.219	0.026	0.316	59.828	-0.252	0.248	0.308	0.544
GABRG2	88.456	-0.930	0.307	0.002	0.012	88.456	0.123	0.295	0.676	0.825	88.456	1.053	0.352	0.003	0.027
BCLAF3	132.199	-0.027	0.212	0.897	0.934	132.199	-0.021	0.211	0.919	0.960	132.199	0.006	0.246	0.980	0.990
MAMDC2	21.536	-0.792	0.750	0.291	0.429	21.536	0.126	0.733	0.863	0.930	21.536	0.918	0.861	0.286	0.521
MAP7D2	58.302	-0.403	1.369	0.769	0.847	58.302	0.506	1.367	0.711	0.845	58.302	0.909	1.580	0.565	0.755
WDR72	1294.231	-0.625	0.276	0.024	0.065	1294.231	0.149	0.275	0.587	0.769	1294.231	0.774	0.318	0.015	0.085
NPB	77.042	1.789	0.251	0.000	0.000	77.042	-0.218	0.275	0.428	0.663	77.042	-2.007	0.302	0.000	0.000
KANK3	54.825	0.055	0.348	0.874	0.919	54.825	0.469	0.341	0.170	0.459	54.825	0.413	0.398	0.299	0.534
SERINC5	283.761	-0.509	0.207	0.014	0.043	283.761	0.102	0.203	0.617	0.789	283.761	0.611	0.238	0.010	0.067
ALX3	48.060	0.319	0.232	0.170	0.290	48.060	0.305	0.229	0.182	0.469	48.060	-0.014	0.266	0.958	0.981
FRMD3	398.794	-1.588	0.411	0.000	0.001	398.794	-1.169	0.409	0.004	0.297	398.794	0.419	0.475	0.378	0.609
PLCXD2	6.400	0.255	0.673	0.705	0.797	6.400	0.316	0.662	0.633	0.800	6.400	0.061	0.770	0.937	0.972
GCSAM	54.243	0.608	0.719	0.398	0.537	54.243	0.744	0.718	0.300	0.567	54.243	0.137	0.829	0.869	0.937
RNF214	338.261	0.090	0.204	0.658	0.761	338.261	0.332	0.203	0.101	0.394	338.261	0.242	0.235	0.304	0.539
C9orf43	167.233	-0.995	0.405	0.014	0.044	167.233	-0.517	0.401	0.197	0.481	167.233	0.478	0.467	0.307	0.542
CFAP126	89.310	-0.541	0.380	0.154	0.270	89.310	0.348	0.372	0.349	0.604	89.310	0.890	0.436	0.041	0.162
NEGR1	171.304	-0.282	0.259	0.276	0.413	171.304	0.356	0.255	0.163	0.454	171.304	0.638	0.298	0.032	0.138
SHPRH	272.374	-0.972	0.253	0.000	0.001	272.374	-0.229	0.249	0.358	0.611	272.374	0.744	0.291	0.011	0.069
UTS2B	344.733	-0.610	0.194	0.002	0.009	344.733	-0.142	0.191	0.459	0.685	344.733	0.468	0.224	0.036	0.149
SNX33	1770.706	0.242	0.313	0.438	0.574	1770.706	0.462	0.312	0.139	0.430	1770.706	0.220	0.361	0.542	0.739
LOC257396	78.320	-0.552	0.296	0.062	0.136	78.320	0.162	0.286	0.572	0.758	78.320	0.713	0.339	0.035	0.146
TAB3	737.476	-0.097	0.171	0.571	0.690	737.476	-0.160	0.171	0.351	0.606	737.476	-0.062	0.198	0.753	0.874
FAM133B	278.674	0.374	0.273	0.171	0.292	278.674	-0.178	0.274	0.515	0.721	278.674	-0.552	0.316	0.081	0.246
HYPK	24.326	0.688	0.381	0.071	0.151	24.326	0.557	0.380	0.142	0.434	24.326	-0.131	0.433	0.763	0.880
PRPF40B	214.173	0.315	0.181	0.081	0.167	214.173	0.027	0.181	0.880	0.939	214.173	-0.288	0.209	0.169	0.385
TBC1D22A	754.543	0.035	0.236	0.880	0.922	754.543	0.296	0.235	0.208	0.490	754.543	0.260	0.272	0.339	0.573
C22orf24	15.524	0.312	0.568	0.583	0.700	15.524	-0.237	0.582	0.684	0.829	15.524	-0.549	0.669	0.411	0.637
CBY1	396.814	0.409	0.210	0.052	0.118	396.814	-0.234	0.212	0.269	0.540	396.814	-0.643	0.244	0.008	0.058
SUN2	3853.120	-0.245	0.167	0.141	0.253	3853.120	0.294	0.167	0.077	0.368	3853.120	0.539	0.192	0.005	0.041
DSTYK	601.760	0.333	0.169	0.049	0.114	601.760	0.062	0.170	0.717	0.848	601.760	-0.271	0.196	0.166	0.381
RAB3GAP2	1341.344	-0.381	0.167	0.022	0.063	1341.344	0.060	0.166	0.718	0.849	1341.344	0.441	0.192	0.022	0.108
DGCR11	15.119	-0.788	0.671	0.240	0.373	15.119	-0.707	0.663	0.286	0.555	15.119	0.081	0.783	0.918	0.961
RAD54B	116.825	-0.977	0.317	0.002	0.010	116.825	-0.620	0.312	0.047	0.336	116.825	0.358	0.367	0.330	0.564
CFAP45	19.563	0.451	0.394	0.253	0.387	19.563	-0.935	0.460	0.042	0.330	19.563	-1.386	0.511	0.007	0.050
CIZ1	2699.430	-0.140	0.152	0.358	0.498	2699.430	0.323	0.152	0.033	0.322	2699.430	0.463	0.175	0.008	0.058
FBXO7	1642.460	-0.338	0.118	0.004	0.017	1642.460	-0.051	0.117	0.663	0.817	1642.460	0.287	0.136	0.035	0.145
PGLS	958.347	0.751	0.302	0.013	0.041	958.347	0.485	0.302	0.108	0.400	958.347	-0.265	0.348	0.446	0.665
QPCT	22.124	0.109	0.400	0.785	0.858	22.124	0.057	0.396	0.885	0.943	22.124	-0.052	0.463	0.911	0.959
BRI3	1449.820	1.447	0.273	0.000	0.000	1449.820	0.348	0.274	0.204	0.487	1449.820	-1.099	0.316	0.001	0.008
ZNF324	170.433	-0.123	0.205	0.549	0.672	170.433	-0.110	0.204	0.590	0.772	170.433	0.013	0.238	0.955	0.979
GAK	1098.816	-0.126	0.246	0.607	0.720	1098.816	-0.357	0.246	0.146	0.438	1098.816	-0.231	0.284	0.416	0.642
SLC39A6	1338.442	-0.600	0.186	0.001	0.007	1338.442	-0.165	0.185	0.371	0.620	1338.442	0.435	0.214	0.043	0.165
GCA	78.900	0.929	0.268	0.001	0.004	78.900	0.040	0.275	0.885	0.942	78.900	-0.889	0.313	0.004	0.038
SVIP	230.005	-0.112	0.235	0.635	0.743	230.005	-0.390	0.235	0.098	0.392	230.005	-0.278	0.273	0.307	0.543

LMOD1	7.792	1.165	1.024	0.255	0.389	7.792	0.591	1.032	0.567	0.754	7.792	-0.575	1.178	0.626	0.795
LSM4	2240.183	0.170	0.286	0.552	0.674	2240.183	-0.442	0.287	0.123	0.415	2240.183	-0.612	0.331	0.064	0.213
BAMBI	273.348	-1.801	0.899	0.045	0.107	273.348	-2.565	0.901	0.004	0.297	273.348	-0.764	1.042	0.463	0.679
VAX2	17.076	1.328	0.389	0.001	0.004	17.076	0.137	0.417	0.742	0.862	17.076	-1.191	0.458	0.009	0.062
RHBDD3	361.696	0.368	0.240	0.125	0.230	361.696	0.067	0.240	0.781	0.885	361.696	-0.302	0.277	0.277	0.511
TTLL1	83.445	0.302	0.405	0.455	0.590	83.445	0.760	0.401	0.058	0.348	83.445	0.458	0.464	0.324	0.559
GALC	65.179	0.085	0.278	0.759	0.840	65.179	-0.044	0.278	0.873	0.936	65.179	-0.130	0.323	0.687	0.836
SAMM50	994.231	-0.421	0.162	0.010	0.033	994.231	-0.152	0.162	0.348	0.604	994.231	0.269	0.188	0.151	0.360
ATXN10	1621.273	-0.032	0.119	0.788	0.859	1621.273	0.018	0.119	0.883	0.941	1621.273	0.050	0.138	0.719	0.854
TNFAIP8	533.505	0.721	0.259	0.005	0.021	533.505	0.441	0.259	0.089	0.382	533.505	-0.280	0.299	0.349	0.582
NOCT	219.816	-0.008	0.311	0.978	0.987	219.816	-0.278	0.311	0.372	0.621	219.816	-0.269	0.360	0.455	0.673
GALE	1206.659	0.328	0.251	0.191	0.316	1206.659	-0.249	0.251	0.321	0.582	1206.659	-0.578	0.290	0.046	0.174
ARIH1	1608.736	-0.747	0.166	0.000	0.000	1608.736	-0.177	0.165	0.286	0.555	1608.736	0.571	0.192	0.003	0.028
MTO1	490.728	-0.854	0.180	0.000	0.000	490.728	-0.261	0.178	0.141	0.432	490.728	0.593	0.208	0.004	0.037
DNAJB5	85.946	1.004	0.182	0.000	0.000	85.946	-0.278	0.199	0.161	0.452	85.946	-1.282	0.220	0.000	0.000
PRDX5	5806.600	1.200	0.296	0.000	0.001	5806.600	0.467	0.296	0.115	0.406	5806.600	-0.733	0.342	0.032	0.138
BACE2	408.226	0.021	0.138	0.877	0.920	408.226	0.042	0.137	0.758	0.871	408.226	0.021	0.160	0.895	0.951
FBXL2	128.853	0.040	0.246	0.871	0.917	128.853	0.049	0.244	0.841	0.917	128.853	0.009	0.284	0.974	0.987
TXN2	925.775	0.000	0.134	0.999	1.000	925.775	0.044	0.133	0.739	0.861	925.775	0.045	0.155	0.773	0.885
TMEM184B	1135.503	-0.006	0.232	0.981	0.988	1135.503	-0.354	0.232	0.127	0.419	1135.503	-0.349	0.268	0.193	0.416
B4GALNT1	434.456	0.581	0.341	0.089	0.179	434.456	-0.766	0.344	0.026	0.316	434.456	-1.348	0.396	0.001	0.010
HECTD1	1674.703	-0.819	0.208	0.000	0.001	1674.703	-0.099	0.208	0.634	0.800	1674.703	0.720	0.241	0.003	0.027
NBPF14	785.928	-0.011	0.270	0.968	0.980	785.928	0.050	0.270	0.854	0.925	785.928	0.061	0.312	0.846	0.926
NIPBL	1899.364	-0.671	0.157	0.000	0.000	1899.364	-0.115	0.156	0.462	0.687	1899.364	0.556	0.181	0.002	0.022
RAB26	123.580	1.051	0.624	0.092	0.184	123.580	0.849	0.624	0.174	0.463	123.580	-0.202	0.720	0.779	0.888
COG4	1319.553	-0.090	0.190	0.635	0.743	1319.553	0.322	0.190	0.089	0.382	1319.553	0.413	0.219	0.060	0.205
GALK1	671.725	0.654	0.162	0.000	0.001	671.725	0.182	0.163	0.263	0.536	671.725	-0.472	0.188	0.012	0.073
METTL7A	1262.773	0.320	0.798	0.688	0.785	1262.773	0.607	0.798	0.447	0.677	1262.773	0.286	0.921	0.756	0.875
ABTB2	1475.342	-0.749	0.251	0.003	0.013	1475.342	-0.318	0.251	0.204	0.487	1475.342	0.430	0.290	0.138	0.341
ASF1A	391.582	-0.098	0.188	0.602	0.715	391.582	-0.311	0.188	0.098	0.393	391.582	-0.213	0.218	0.328	0.562
MOB4	435.945	-0.299	0.178	0.094	0.187	435.945	-0.484	0.178	0.007	0.297	435.945	-0.185	0.207	0.373	0.604
YIPF3	1519.963	0.233	0.172	0.176	0.298	1519.963	0.171	0.172	0.320	0.581	1519.963	-0.062	0.199	0.756	0.875
ANAPC13	813.755	-0.099	0.180	0.581	0.698	813.755	-0.407	0.180	0.024	0.316	813.755	-0.308	0.208	0.140	0.343
PARM1	63.373	0.342	0.300	0.254	0.388	63.373	0.143	0.300	0.634	0.800	63.373	-0.199	0.347	0.566	0.756
GALK2	717.161	-0.168	0.101	0.098	0.193	717.161	-0.027	0.100	0.784	0.886	717.161	0.140	0.117	0.231	0.460
ZNF345	20.138	0.368	0.386	0.341	0.481	20.138	0.372	0.381	0.330	0.590	20.138	0.004	0.441	0.993	0.996
TECPR1	272.540	0.111	0.173	0.522	0.649	272.540	-0.097	0.173	0.573	0.758	272.540	-0.208	0.200	0.299	0.534
ARMC8	635.796	-0.391	0.176	0.027	0.072	635.796	-0.360	0.176	0.041	0.329	635.796	0.032	0.204	0.877	0.941
DCAF12	1204.410	-0.467	0.137	0.001	0.004	1204.410	-0.264	0.137	0.053	0.341	1204.410	0.202	0.159	0.202	0.426
BRMS1	687.804	0.183	0.364	0.616	0.727	687.804	-0.774	0.365	0.034	0.322	687.804	-0.956	0.421	0.023	0.111
WHRN	265.554	0.357	0.367	0.330	0.471	265.554	0.626	0.366	0.087	0.380	265.554	0.269	0.423	0.525	0.726
USP49	148.834	0.186	0.255	0.466	0.600	148.834	-0.145	0.256	0.572	0.757	148.834	-0.331	0.296	0.264	0.498
ABHD14A	287.541	-0.050	0.197	0.799	0.868	287.541	0.442	0.195	0.023	0.316	287.541	0.492	0.227	0.030	0.132
PRKD2	490.334	0.699	0.176	0.000	0.001	490.334	0.220	0.177	0.214	0.493	490.334	-0.480	0.203	0.018	0.096
SUMF2	1607.917	-0.154	0.152	0.313	0.453	1607.917	0.235	0.152	0.121	0.413	1607.917	0.389	0.176	0.027	0.123
NEPRO	880.585	-0.535	0.168	0.001	0.008	880.585	0.046	0.167	0.785	0.888	880.585	0.581	0.194	0.003	0.027
RPL36	11140.322	0.638	0.272	0.019	0.055	11140.322	0.423	0.272	0.120	0.412	11140.322	-0.215	0.314	0.494	0.702
MPC2	1540.556	0.143	0.224	0.522	0.650	1540.556	0.265	0.224	0.236	0.513	1540.556	0.122	0.258	0.637	0.803
LETMD1	1499.718	-0.156	0.372	0.675	0.774	1499.718	0.667	0.371	0.072	0.361	1499.718	0.823	0.429	0.055	0.195
DCAF13	997.786	-0.851	0.276	0.002	0.010	997.786	-0.708	0.275	0.010	0.299	997.786	0.144	0.319	0.652	0.813
GALNS	537.293	0.383	0.144	0.008	0.028	537.293	0.281	0.144	0.051	0.338	537.293	-0.103	0.166	0.537	0.735
TMEM186	223.399	-0.133	0.161	0.409	0.548	223.399	-0.125	0.159	0.433	0.667	223.399	0.008	0.187	0.967	0.985
POLR1A	2926.271	-0.928	0.292	0.001	0.008	2926.271	-0.675	0.292	0.021	0.312	2926.271	0.253	0.337	0.454	0.672
POC1A	277.060	-0.491	0.264	0.063	0.137	277.060	-0.172	0.262	0.511	0.719	277.060	0.319	0.305	0.296	0.531
ZNF473	224.764	-0.811	0.186	0.000	0.000	224.764	-0.292	0.181	0.106	0.398	224.764	0.519	0.214	0.015	0.087
GALNT1	1468.586	-0.370	0.188	0.049	0.113	1468.586	-0.120	0.187	0.523	0.726	1468.586	0.250	0.217	0.248	0.480
ABI3BP	775.314	-0.685	0.444	0.123	0.229	775.314	0.375	0.443	0.397	0.641	775.314	1.060	0.513	0.039	0.155
PAMR1	19.534	-0.440	0.919	0.632	0.741	19.534	0.583	0.904	0.519	0.724	19.534	1.023	1.055	0.332	0.567
TRIM58	638.565	-0.435	0.293	0.138	0.248	638.565	-0.808	0.293	0.006	0.297	638.565	-0.373	0.339	0.271	0.506
PLEKHG4	303.107	-0.172	0.189	0.365	0.505	303.107	0.309	0.187	0.098	0.392	303.107	0.481	0.218	0.027	0.123
EEF1AKMT3	439.762	0.146	0.477	0.759	0.840	439.762	0.805	0.476	0.091	0.385	439.762	0.659	0.550	0.231	0.460
INTS7	713.702	-0.823	0.205	0.000	0.001	713.702	-0.214	0.204	0.293	0.561	713.702	0.609	0.237	0.010	0.066
RNF19A	613.389	-0.158	0.225	0.482	0.616	613.389	-0.056	0.225	0.802	0.896	613.389	0.102	0.260	0.696	0.841
RCHY1	392.607	-0.835	0.205	0.000	0.001	392.607	-0.188	0.202	0.351	0.605	392.607	0.647	0.236	0.006	0.047
GALNT2	6782.030	-0.365	0.146	0.012	0.040	6782.030	-0.044	0.146	0.761	0.873	6782.030	0.320	0.168	0.057	0.199
IFFO1	269.046	1.021	0.193	0.000	0.000	269.046	0.087	0.197	0.657	0.813	269.046	-0.933	0.225	0.000	0.001
CCDC28A	425.820	0.091	0.222	0.682	0.779	425.820	0.394	0.221	0.074	0.364	425.820	0.303	0.256	0.236	0.465

MTHFD1L	988.647	-1.614	0.407	0.000	0.001	988.647	-0.761	0.406	0.061	0.350	988.647	0.854	0.470	0.069	0.225
CNOT10	703.004	-0.619	0.186	0.001	0.005	703.004	-0.393	0.185	0.034	0.322	703.004	0.226	0.215	0.294	0.530
ANAPC15	412.387	0.387	0.253	0.126	0.232	412.387	-0.147	0.254	0.564	0.752	412.387	-0.533	0.293	0.068	0.222
TMEM158	1025.988	0.319	0.286	0.265	0.401	1025.988	-0.166	0.287	0.562	0.750	1025.988	-0.485	0.331	0.142	0.348
AHCTF1	1889.606	-0.897	0.257	0.000	0.003	1889.606	-0.672	0.256	0.009	0.297	1889.606	0.226	0.296	0.446	0.666
GALNT3	21.992	0.635	0.316	0.044	0.106	21.992	-0.194	0.340	0.569	0.756	21.992	-0.829	0.381	0.029	0.130
DPCD	728.268	0.036	0.241	0.881	0.923	728.268	0.572	0.240	0.017	0.306	728.268	0.536	0.277	0.054	0.191
C1orf43	5304.533	-0.270	0.145	0.062	0.136	5304.533	0.179	0.144	0.216	0.495	5304.533	0.448	0.167	0.007	0.053
POT1	384.987	-0.532	0.196	0.007	0.024	384.987	0.070	0.193	0.718	0.849	384.987	0.601	0.225	0.008	0.054
RTTN	406.586	-0.989	0.228	0.000	0.000	406.586	-0.352	0.225	0.117	0.409	406.586	0.637	0.263	0.015	0.087
NDUFAF3	564.336	0.855	0.288	0.003	0.013	564.336	-0.205	0.290	0.479	0.699	564.336	-1.060	0.334	0.001	0.018
THUMPD3	959.012	-0.605	0.196	0.002	0.010	959.012	-0.402	0.195	0.039	0.327	959.012	0.204	0.226	0.367	0.600
ALS2CL	254.530	1.210	0.432	0.005	0.020	254.530	0.971	0.432	0.025	0.316	254.530	-0.239	0.498	0.631	0.799
GALT	145.282	0.310	0.283	0.274	0.410	145.282	0.661	0.280	0.018	0.306	145.282	0.352	0.325	0.279	0.513
NELFB	2020.279	-0.200	0.140	0.152	0.267	2020.279	-0.151	0.140	0.280	0.549	2020.279	0.049	0.161	0.760	0.878
ZDHHC5	1639.194	-0.064	0.206	0.756	0.838	1639.194	-0.438	0.206	0.033	0.322	1639.194	-0.374	0.238	0.116	0.305
HSPA12A	211.479	-0.375	0.225	0.096	0.190	211.479	-0.181	0.223	0.416	0.654	211.479	0.193	0.260	0.457	0.675
ATL3	1659.950	0.508	0.197	0.010	0.034	1659.950	0.369	0.197	0.061	0.350	1659.950	-0.139	0.227	0.540	0.737
SGMS1	493.397	-0.890	0.167	0.000	0.000	493.397	-0.233	0.164	0.155	0.448	493.397	0.657	0.193	0.001	0.010
NALCN	258.464	-0.755	0.297	0.011	0.037	258.464	0.221	0.294	0.452	0.680	258.464	0.976	0.342	0.004	0.037
NOL11	752.002	-1.118	0.270	0.000	0.000	752.002	-0.661	0.269	0.014	0.306	752.002	0.457	0.312	0.143	0.348
ASPM	1471.144	-0.439	0.285	0.124	0.229	1471.144	0.083	0.285	0.772	0.879	1471.144	0.522	0.329	0.113	0.301
CNRIP1	28.266	0.666	0.408	0.103	0.200	28.266	0.449	0.409	0.273	0.544	28.266	-0.217	0.468	0.643	0.806
BOD1L1	926.534	0.013	0.164	0.936	0.959	926.534	-0.038	0.163	0.815	0.903	926.534	-0.051	0.189	0.786	0.893
GEMIN5	749.459	-1.181	0.250	0.000	0.000	749.459	-0.460	0.249	0.064	0.353	749.459	0.722	0.289	0.012	0.076
GAMT	869.152	0.629	0.285	0.027	0.073	869.152	0.382	0.285	0.179	0.467	869.152	-0.246	0.328	0.454	0.672
PTPN23	1125.353	0.206	0.096	0.031	0.081	1125.353	0.049	0.096	0.610	0.785	1125.353	-0.157	0.111	0.155	0.367
CLIC4	8684.239	-0.659	0.206	0.001	0.007	8684.239	-0.091	0.206	0.657	0.813	8684.239	0.567	0.238	0.017	0.092
NIPSNAP3A	380.881	0.211	0.208	0.311	0.450	380.881	-0.070	0.208	0.737	0.859	380.881	-0.281	0.241	0.243	0.474
NSL1	462.994	0.028	0.139	0.839	0.894	462.994	-0.142	0.139	0.305	0.571	462.994	-0.170	0.161	0.289	0.525
WWTR1	727.168	-0.286	0.239	0.231	0.363	727.168	0.246	0.237	0.301	0.567	727.168	0.532	0.275	0.053	0.191
HEATR5A	326.529	-0.659	0.297	0.027	0.071	326.529	-0.739	0.297	0.013	0.305	326.529	-0.081	0.344	0.814	0.908
SAMHD1	336.284	0.477	0.259	0.065	0.141	336.284	-0.577	0.261	0.027	0.319	336.284	-1.054	0.301	0.000	0.007
FAM98A	916.684	-0.816	0.317	0.010	0.034	916.684	-0.801	0.317	0.012	0.305	916.684	0.015	0.366	0.967	0.985
TPGS2	973.042	0.135	0.157	0.388	0.527	973.042	0.112	0.157	0.475	0.696	973.042	-0.023	0.181	0.897	0.951
SIN3A	1242.575	-0.561	0.134	0.000	0.000	1242.575	0.034	0.133	0.798	0.895	1242.575	0.595	0.155	0.000	0.003
C20orf194	250.870	-0.185	0.331	0.576	0.694	250.870	0.527	0.328	0.108	0.400	250.870	0.712	0.381	0.061	0.208
NECTIN3	411.067	-0.867	0.252	0.001	0.004	411.067	-0.551	0.250	0.028	0.321	411.067	0.316	0.291	0.278	0.512
ZNF385A	56.121	1.611	0.445	0.000	0.002	56.121	0.436	0.467	0.350	0.605	56.121	-1.175	0.516	0.023	0.110
KBTBD2	444.097	-0.240	0.168	0.154	0.270	444.097	-0.285	0.168	0.089	0.383	444.097	-0.045	0.195	0.818	0.910
SYF2	1359.202	0.683	0.239	0.004	0.018	1359.202	0.260	0.239	0.278	0.548	1359.202	-0.423	0.276	0.126	0.320
GANC	287.018	-0.523	0.157	0.001	0.005	287.018	-0.065	0.153	0.669	0.820	287.018	0.457	0.180	0.011	0.070
RWDD3	84.394	0.859	0.276	0.002	0.009	84.394	0.218	0.280	0.435	0.668	84.394	-0.641	0.319	0.045	0.170
PNKD	543.625	1.135	0.203	0.000	0.000	543.625	0.214	0.204	0.294	0.561	543.625	-0.921	0.235	0.000	0.002
SEC31B	55.360	1.744	0.589	0.003	0.014	55.360	1.464	0.590	0.013	0.305	55.360	-0.280	0.674	0.678	0.830
PNISR	930.253	0.859	0.236	0.000	0.002	930.253	0.143	0.236	0.546	0.740	930.253	-0.716	0.272	0.008	0.059
KANK2	1686.854	0.396	0.316	0.210	0.339	1686.854	0.353	0.316	0.263	0.536	1686.854	-0.043	0.365	0.907	0.957
ADGRA2	721.252	0.903	0.243	0.000	0.002	721.252	0.333	0.244	0.171	0.460	721.252	-0.570	0.281	0.042	0.165
NUDT13	30.488	-0.678	0.383	0.077	0.161	30.488	0.052	0.362	0.886	0.943	30.488	0.730	0.438	0.095	0.272
VIRMA	1049.764	-0.458	0.165	0.006	0.021	1049.764	0.108	0.164	0.509	0.718	1049.764	0.567	0.191	0.003	0.028
TMEM87A	812.777	-0.466	0.112	0.000	0.000	812.777	-0.157	0.111	0.156	0.448	812.777	0.309	0.130	0.017	0.092
C2CD2	473.282	0.252	0.167	0.132	0.240	473.282	0.207	0.167	0.216	0.495	473.282	-0.045	0.193	0.814	0.907
GAPDH	206095.682	0.532	0.412	0.197	0.323	206095.682	0.711	0.412	0.084	0.376	206095.682	0.179	0.476	0.706	0.847
SH2B1	664.970	0.581	0.170	0.001	0.004	664.970	0.320	0.170	0.061	0.350	664.970	-0.261	0.196	0.184	0.404
UNC50	319.926	-0.237	0.177	0.181	0.304	319.926	-0.122	0.175	0.488	0.705	319.926	0.115	0.205	0.573	0.760
PARS2	117.148	-0.323	0.272	0.235	0.368	117.148	-0.100	0.269	0.711	0.845	117.148	0.224	0.315	0.477	0.691
MMACHC	2867.128	0.681	0.314	0.030	0.078	2867.128	-0.529	0.314	0.092	0.387	2867.128	-1.210	0.362	0.001	0.012
TIPARP	1498.769	-1.084	0.175	0.000	0.000	1498.769	-0.247	0.173	0.154	0.446	1498.769	0.837	0.201	0.000	0.001
NECAP1	490.202	-0.602	0.226	0.008	0.028	490.202	-0.410	0.225	0.069	0.357	490.202	0.193	0.262	0.461	0.678
CHMP2B	942.385	0.003	0.146	0.981	0.989	942.385	-0.294	0.146	0.044	0.332	942.385	-0.297	0.169	0.078	0.242
DHRS7B	139.552	0.285	0.266	0.283	0.421	139.552	-0.588	0.271	0.030	0.322	139.552	-0.873	0.311	0.005	0.041
AAR2	443.663	-0.165	0.136	0.223	0.354	443.663	-0.249	0.135	0.066	0.354	443.663	-0.083	0.158	0.596	0.775
DNAH1	20.228	0.706	0.469	0.132	0.241	20.228	0.403	0.472	0.394	0.638	20.228	-0.303	0.539	0.574	0.761
NGDN	381.656	-0.389	0.242	0.108	0.207	381.656	-0.447	0.242	0.065	0.353	381.656	-0.058	0.280	0.837	0.921
KRT23	15.971	3.285	0.566	0.000	0.000	15.971	0.623	0.621	0.316	0.578	15.971	-2.663	0.656	0.000	0.001
TSKU	1376.596	0.657	0.122	0.000	0.000	1376.596	0.020	0.123	0.869	0.933	1376.596	-0.637	0.142	0.000	0.000
HINFP	513.194	0.033	0.190	0.860	0.908	513.194	0.377	0.189	0.046	0.334	513.194	0.343	0.219	0.116	0.306

ULK3	456.056	0.439	0.199	0.027	0.073	456.056	-0.107	0.200	0.594	0.775	456.056	-0.545	0.231	0.018	0.095
SNED1	53.256	0.096	0.316	0.761	0.841	53.256	0.501	0.307	0.103	0.395	53.256	0.405	0.360	0.260	0.494
HIGD1A	987.943	-0.295	0.319	0.355	0.495	987.943	-0.791	0.319	0.013	0.305	987.943	-0.496	0.369	0.178	0.397
REXO2	812.200	-0.104	0.237	0.662	0.764	812.200	-0.430	0.238	0.070	0.359	812.200	-0.326	0.275	0.235	0.465
IBTK	1318.811	-0.878	0.226	0.000	0.001	1318.811	-0.385	0.225	0.087	0.380	1318.811	0.493	0.261	0.059	0.202
CLIP3	39.860	1.196	0.476	0.012	0.039	39.860	0.452	0.483	0.349	0.604	39.860	-0.744	0.549	0.176	0.393
TBC1D10B	1209.180	0.251	0.164	0.126	0.232	1209.180	0.043	0.164	0.793	0.892	1209.180	-0.208	0.190	0.273	0.508
RNF167	814.849	0.343	0.161	0.033	0.085	814.849	0.123	0.161	0.445	0.675	814.849	-0.220	0.186	0.237	0.466
MOXD1	15.062	1.710	0.444	0.000	0.001	15.062	-0.054	0.487	0.911	0.956	15.062	-1.764	0.531	0.001	0.012
GORASP2	1227.726	-0.473	0.192	0.014	0.043	1227.726	-0.368	0.192	0.055	0.342	1227.726	0.105	0.222	0.637	0.803
C2CD3	276.137	-0.494	0.271	0.068	0.147	276.137	-0.520	0.270	0.055	0.342	276.137	-0.026	0.314	0.934	0.970
TKFC	1204.455	-0.197	0.248	0.426	0.563	1204.455	0.240	0.247	0.333	0.592	1204.455	0.437	0.286	0.127	0.322
ZZZ3	1149.390	-0.827	0.166	0.000	0.000	1149.390	-0.218	0.164	0.185	0.472	1149.390	0.609	0.191	0.001	0.017
SPATS2L	1288.796	-0.422	0.118	0.000	0.003	1288.796	-0.295	0.117	0.012	0.305	1288.796	0.127	0.136	0.353	0.586
NSMF	1878.675	0.169	0.131	0.198	0.324	1878.675	-0.099	0.132	0.452	0.680	1878.675	-0.268	0.152	0.078	0.241
L3MBTL1	16.595	0.884	0.519	0.089	0.179	16.595	0.417	0.525	0.427	0.663	16.595	-0.467	0.598	0.434	0.656
RPAP1	918.704	-0.726	0.165	0.000	0.000	918.704	0.230	0.163	0.157	0.449	918.704	0.956	0.190	0.000	0.000
FAM32A	853.455	0.292	0.125	0.020	0.058	853.455	-0.087	0.126	0.488	0.705	853.455	-0.379	0.145	0.009	0.062
LRIG1	287.413	-0.230	0.184	0.210	0.339	287.413	0.116	0.181	0.523	0.726	287.413	0.346	0.212	0.102	0.284
UPF2	1016.143	-0.545	0.166	0.001	0.006	1016.143	-0.385	0.166	0.020	0.310	1016.143	0.160	0.192	0.405	0.632
LRP10	2160.804	0.285	0.241	0.237	0.370	2160.804	0.415	0.241	0.085	0.376	2160.804	0.130	0.278	0.640	0.805
TMEM98	51.632	0.500	0.283	0.077	0.161	51.632	-0.194	0.291	0.506	0.716	51.632	-0.694	0.333	0.037	0.151
PTCD1	570.910	0.192	0.270	0.477	0.611	570.910	-0.389	0.271	0.151	0.443	570.910	-0.580	0.312	0.063	0.211
ACOT11	87.300	0.233	0.297	0.434	0.570	87.300	0.373	0.295	0.206	0.488	87.300	0.140	0.342	0.682	0.832
NSUN5P2	253.149	0.947	0.241	0.000	0.001	253.149	0.172	0.243	0.480	0.699	253.149	-0.775	0.278	0.005	0.043
PLEKHG3	714.654	-0.170	0.092	0.064	0.139	714.654	0.122	0.090	0.176	0.464	714.654	0.292	0.105	0.006	0.045
OSBPL3	549.340	-0.547	0.270	0.043	0.102	549.340	-0.669	0.269	0.013	0.305	549.340	-0.122	0.312	0.695	0.840
SUSD5	655.157	-0.681	0.234	0.004	0.015	655.157	-0.192	0.233	0.410	0.649	655.157	0.489	0.270	0.070	0.226
ATRNL1	17.497	1.709	0.400	0.000	0.000	17.497	0.146	0.441	0.740	0.861	17.497	-1.563	0.475	0.001	0.013
IPCEF1	14.062	0.822	0.661	0.214	0.343	14.062	0.913	0.656	0.164	0.454	14.062	0.091	0.753	0.904	0.955
GLCE	301.931	-0.328	0.202	0.104	0.202	301.931	-0.330	0.201	0.101	0.394	301.931	-0.002	0.234	0.993	0.996
ZNF451	855.640	-0.842	0.186	0.000	0.000	855.640	-0.018	0.185	0.924	0.962	855.640	0.824	0.215	0.000	0.003
SIPA1L1	843.935	-0.315	0.147	0.032	0.084	843.935	-0.245	0.147	0.096	0.390	843.935	0.070	0.170	0.680	0.831
CHD5	20.009	-0.306	0.729	0.674	0.773	20.009	0.791	0.654	0.227	0.505	20.009	1.098	0.805	0.173	0.390
SS18L1	151.279	0.066	0.234	0.777	0.852	151.279	-0.254	0.235	0.281	0.551	151.279	-0.320	0.272	0.240	0.471
SETBP1	263.128	0.058	0.341	0.865	0.913	263.128	0.154	0.340	0.651	0.810	263.128	0.096	0.393	0.807	0.903
MAGI3	436.682	-0.989	0.223	0.000	0.000	436.682	-0.236	0.220	0.282	0.552	436.682	0.753	0.257	0.003	0.031
UBXN7	1651.179	-0.176	0.186	0.344	0.485	1651.179	0.111	0.186	0.549	0.743	1651.179	0.287	0.215	0.181	0.400
LTN1	729.697	-0.610	0.259	0.018	0.054	729.697	-0.198	0.258	0.444	0.675	729.697	0.413	0.299	0.168	0.382
CNTNAP2	18.489	-2.422	0.854	0.005	0.018	18.489	-2.345	0.844	0.005	0.297	18.489	0.077	1.009	0.939	0.973
ZNF500	171.972	-0.067	0.162	0.677	0.776	171.972	0.173	0.158	0.275	0.545	171.972	0.240	0.186	0.196	0.419
FAM169A	197.582	-0.790	0.230	0.001	0.004	197.582	-0.493	0.227	0.030	0.322	197.582	0.297	0.266	0.265	0.499
SLITRK5	24.306	3.507	0.807	0.000	0.000	24.306	0.598	0.839	0.476	0.697	24.306	-2.909	0.934	0.002	0.020
DNM3	70.799	0.056	0.278	0.841	0.896	70.799	0.134	0.275	0.628	0.796	70.799	0.078	0.321	0.808	0.904
SENP6	1464.647	-0.830	0.158	0.000	0.000	1464.647	-0.134	0.157	0.393	0.637	1464.647	0.696	0.183	0.000	0.003
RAB11FIP5	1757.756	0.183	0.210	0.384	0.524	1757.756	0.231	0.210	0.272	0.543	1757.756	0.048	0.242	0.843	0.924
ANKRD17	1675.027	-0.448	0.181	0.013	0.042	1675.027	-0.177	0.181	0.326	0.586	1675.027	0.270	0.209	0.196	0.418
GIGYF2	1277.715	-0.564	0.217	0.009	0.032	1277.715	-0.189	0.216	0.381	0.627	1277.715	0.374	0.250	0.135	0.335
APPL1	676.312	-0.111	0.364	0.761	0.841	676.312	0.467	0.363	0.198	0.482	676.312	0.578	0.420	0.169	0.384
HACL1	426.623	-0.624	0.222	0.005	0.019	426.623	-0.275	0.220	0.213	0.493	426.623	0.350	0.256	0.172	0.389
DECR2	501.359	0.703	0.221	0.001	0.008	501.359	-0.054	0.222	0.809	0.900	501.359	-0.756	0.255	0.003	0.029
RAI14	2037.210	-0.490	0.220	0.026	0.071	2037.210	-0.476	0.220	0.031	0.322	2037.210	0.014	0.255	0.956	0.980
LSM14A	6267.540	-0.383	0.107	0.000	0.003	6267.540	-0.028	0.107	0.796	0.893	6267.540	0.355	0.124	0.004	0.035
MYRF-AS1	11.799	1.351	0.548	0.014	0.043	11.799	0.170	0.580	0.769	0.877	11.799	-1.181	0.642	0.066	0.217
RTL8A	919.280	0.824	0.195	0.000	0.000	919.280	0.071	0.196	0.716	0.848	919.280	-0.753	0.226	0.001	0.012
POLDIP2	2497.198	-0.171	0.183	0.349	0.489	2497.198	-0.325	0.183	0.075	0.364	2497.198	-0.154	0.211	0.466	0.682
FAM230C	57.434	-0.862	0.445	0.053	0.120	57.434	-0.248	0.436	0.569	0.756	57.434	0.614	0.513	0.232	0.461
ARHGEF26	159.164	-0.074	0.185	0.689	0.785	159.164	-0.140	0.184	0.447	0.677	159.164	-0.066	0.214	0.759	0.877
GPSM1	620.392	0.335	0.148	0.023	0.065	620.392	0.191	0.148	0.196	0.479	620.392	-0.144	0.170	0.399	0.626
GGA1	601.764	-0.048	0.133	0.720	0.810	601.764	-0.141	0.133	0.288	0.556	601.764	-0.093	0.154	0.544	0.740
ABHD12	679.771	0.543	0.149	0.000	0.002	679.771	-0.119	0.150	0.426	0.662	679.771	-0.662	0.172	0.000	0.003
HERC4	1069.593	-0.273	0.165	0.098	0.193	1069.593	0.064	0.164	0.697	0.837	1069.593	0.337	0.190	0.077	0.238
TOR1AIP1	767.107	-0.078	0.115	0.496	0.627	767.107	0.021	0.114	0.855	0.926	767.107	0.099	0.132	0.455	0.673
CCDC9	515.539	-0.642	0.248	0.010	0.033	515.539	-0.206	0.247	0.404	0.646	515.539	0.437	0.287	0.128	0.323
DCAF4	247.127	-0.298	0.198	0.132	0.241	247.127	-0.518	0.198	0.009	0.297	247.127	-0.220	0.231	0.341	0.575
CHTOP	1506.054	-0.344	0.234	0.142	0.253	1506.054	-0.388	0.234	0.098	0.392	1506.054	-0.043	0.270	0.872	0.939
EDRF1	253.858	-0.801	0.198	0.000	0.001	253.858	-0.063	0.193	0.745	0.864	253.858	0.738	0.228	0.001	0.015

SZRD1	3830.287	-0.035	0.105	0.736	0.822	3830.287	-0.016	0.104	0.875	0.937	3830.287	0.019	0.121	0.876	0.941
WIPI2	1220.642	0.128	0.104	0.218	0.348	1220.642	-0.088	0.104	0.398	0.642	1220.642	-0.215	0.120	0.073	0.231
PYGO1	197.721	-0.046	0.197	0.816	0.879	197.721	-0.124	0.197	0.530	0.731	197.721	-0.078	0.229	0.735	0.863
CCDC69	209.916	0.696	0.534	0.193	0.318	209.916	0.888	0.534	0.096	0.391	209.916	0.193	0.616	0.755	0.875
TANC2	2128.541	-0.670	0.233	0.004	0.017	2128.541	0.088	0.232	0.705	0.842	2128.541	0.758	0.269	0.005	0.040
WSB1	726.581	0.593	0.271	0.029	0.076	726.581	0.476	0.271	0.079	0.371	726.581	-0.117	0.313	0.707	0.848
LDLRAP1	250.328	0.248	0.215	0.248	0.382	250.328	-0.467	0.218	0.032	0.322	250.328	-0.715	0.251	0.004	0.037
PRPF31	5108.069	0.098	0.291	0.737	0.823	5108.069	-0.388	0.291	0.182	0.469	5108.069	-0.485	0.336	0.148	0.356
EPC2	425.999	-0.957	0.196	0.000	0.000	425.999	-0.151	0.192	0.431	0.665	425.999	0.806	0.225	0.000	0.006
TCTN3	567.355	-0.422	0.145	0.004	0.016	567.355	-0.068	0.144	0.636	0.801	567.355	0.354	0.168	0.035	0.145
FGFR1OP2	620.390	-0.203	0.142	0.152	0.267	620.390	-0.102	0.141	0.467	0.691	620.390	0.101	0.164	0.539	0.736
KIFBP	544.499	-0.226	0.217	0.298	0.437	544.499	-0.590	0.218	0.007	0.297	544.499	-0.364	0.252	0.149	0.357
GAPVD1	847.297	-0.650	0.167	0.000	0.001	847.297	-0.308	0.166	0.063	0.350	847.297	0.341	0.193	0.076	0.238
TRPC4AP	1239.202	-0.183	0.122	0.133	0.241	1239.202	0.043	0.121	0.724	0.852	1239.202	0.226	0.141	0.108	0.293
RALGAPA1P1	28.885	-0.511	0.397	0.198	0.324	28.885	-0.375	0.389	0.335	0.594	28.885	0.136	0.462	0.768	0.882
SERBP1	15807.504	-0.798	0.162	0.000	0.000	15807.504	-0.259	0.162	0.109	0.401	15807.504	0.539	0.187	0.004	0.035
TES	33.349	0.463	0.331	0.161	0.279	33.349	-0.018	0.337	0.958	0.978	33.349	-0.481	0.386	0.213	0.438
ZBTB20	182.381	1.836	0.429	0.000	0.000	182.381	0.019	0.434	0.964	0.981	182.381	-1.816	0.498	0.000	0.005
TTLL3	49.798	2.261	0.459	0.000	0.000	49.798	1.410	0.464	0.002	0.297	49.798	-0.851	0.522	0.103	0.285
IRF2BP1	863.236	0.033	0.196	0.866	0.913	863.236	0.000	0.196	0.999	1.000	863.236	-0.033	0.226	0.883	0.944
TRAF3IP1	306.011	0.050	0.174	0.773	0.849	306.011	-0.358	0.176	0.042	0.330	306.011	-0.408	0.203	0.045	0.170
PHF19	621.827	-0.360	0.149	0.016	0.048	621.827	0.109	0.148	0.461	0.687	621.827	0.469	0.172	0.006	0.048
ZNF658	27.682	0.001	0.372	0.999	1.000	27.682	-0.117	0.370	0.751	0.867	27.682	-0.118	0.432	0.785	0.892
NAT9	361.379	0.086	0.265	0.746	0.830	361.379	-0.404	0.266	0.129	0.420	361.379	-0.490	0.307	0.111	0.297
ZNF337	381.237	0.300	0.221	0.174	0.295	381.237	0.310	0.220	0.159	0.451	381.237	0.010	0.255	0.968	0.985
ABCA12	40.587	0.023	0.500	0.963	0.978	40.587	0.689	0.490	0.160	0.451	40.587	0.665	0.571	0.244	0.475
NOC2L	4632.336	-0.501	0.315	0.111	0.211	4632.336	-0.636	0.315	0.043	0.331	4632.336	-0.134	0.363	0.711	0.850
RSL1D1	5951.565	-0.334	0.142	0.019	0.055	5951.565	-0.008	0.142	0.955	0.978	5951.565	0.326	0.164	0.047	0.175
GIMAP2	35.358	1.091	0.587	0.063	0.138	35.358	0.570	0.590	0.334	0.593	35.358	-0.521	0.676	0.442	0.662
IFT172	324.487	-0.230	0.206	0.265	0.401	324.487	0.054	0.205	0.790	0.891	324.487	0.285	0.238	0.232	0.461
MTG2	456.707	-0.435	0.246	0.077	0.160	456.707	-0.631	0.246	0.010	0.299	456.707	-0.196	0.285	0.492	0.702
PCDHB5	64.635	0.578	0.246	0.019	0.054	64.635	0.230	0.248	0.354	0.608	64.635	-0.348	0.284	0.220	0.447
SENP3	417.353	-0.949	0.374	0.011	0.037	417.353	-0.815	0.373	0.029	0.322	417.353	0.134	0.432	0.757	0.876
GARS1	4356.536	-1.358	0.321	0.000	0.000	4356.536	-0.629	0.321	0.050	0.337	4356.536	0.729	0.371	0.049	0.181
TIPRL	1111.670	-0.385	0.162	0.018	0.052	1111.670	-0.287	0.162	0.076	0.366	1111.670	0.098	0.188	0.601	0.779
STEAP2	495.431	-0.213	0.235	0.364	0.505	495.431	-0.288	0.235	0.220	0.499	495.431	-0.075	0.272	0.783	0.891
INTS1	3660.338	-0.232	0.158	0.142	0.253	3660.338	-0.166	0.157	0.291	0.559	3660.338	0.065	0.182	0.719	0.854
NPHP4	340.179	0.138	0.285	0.628	0.738	340.179	0.363	0.284	0.202	0.485	340.179	0.225	0.329	0.495	0.703
TMEM251	240.676	-0.107	0.384	0.781	0.855	240.676	-0.614	0.386	0.111	0.405	240.676	-0.507	0.446	0.255	0.489
GART	3460.870	-0.947	0.319	0.003	0.013	3460.870	-0.634	0.319	0.047	0.335	3460.870	0.314	0.368	0.394	0.622
GAS1	1035.386	0.078	0.355	0.827	0.886	1035.386	0.166	0.354	0.640	0.803	1035.386	0.088	0.409	0.829	0.916
FBXW2	933.079	-0.237	0.141	0.094	0.187	933.079	-0.093	0.141	0.510	0.718	933.079	0.144	0.163	0.378	0.609
PTPN22	12.063	-0.723	0.545	0.184	0.308	12.063	0.111	0.504	0.826	0.909	12.063	0.834	0.619	0.177	0.395
AMD1	1751.021	-0.721	0.268	0.007	0.026	1751.021	-0.484	0.268	0.071	0.360	1751.021	0.237	0.310	0.444	0.664
GAS2	23.995	-0.545	0.390	0.162	0.281	23.995	0.183	0.366	0.617	0.789	23.995	0.728	0.443	0.101	0.282
GMEB2	491.635	-0.780	0.211	0.000	0.002	491.635	-0.289	0.209	0.167	0.456	491.635	0.492	0.244	0.044	0.168
PITPNC1	215.893	0.799	0.211	0.000	0.001	215.893	-0.053	0.216	0.808	0.899	215.893	-0.851	0.246	0.001	0.009
GAS6	1144.570	1.278	0.231	0.000	0.000	1144.570	0.354	0.232	0.126	0.419	1144.570	-0.924	0.267	0.001	0.008
GAS8	274.972	0.452	0.196	0.021	0.060	274.972	0.254	0.196	0.195	0.479	274.972	-0.197	0.226	0.383	0.612
FBXL3	1028.257	-0.626	0.234	0.007	0.027	1028.257	-0.024	0.232	0.918	0.959	1028.257	0.602	0.269	0.025	0.119
ARL5A	777.509	-0.707	0.145	0.000	0.000	777.509	-0.075	0.143	0.602	0.779	777.509	0.633	0.167	0.000	0.003
PHGDH	4502.695	-0.643	0.196	0.001	0.006	4502.695	0.349	0.196	0.075	0.364	4502.695	0.992	0.227	0.000	0.000
B3GAT3	733.931	0.820	0.197	0.000	0.000	733.931	-0.164	0.198	0.409	0.649	733.931	-0.983	0.228	0.000	0.001
TIAM2	46.162	0.050	0.278	0.857	0.907	46.162	0.039	0.275	0.888	0.944	46.162	-0.012	0.322	0.971	0.986
LRRC29	16.114	0.184	0.588	0.755	0.837	16.114	0.460	0.575	0.424	0.660	16.114	0.276	0.671	0.681	0.831
FBXO2	41.892	2.400	0.449	0.000	0.000	41.892	1.063	0.495	0.032	0.322	41.892	-1.336	0.490	0.006	0.048
FBXL6	598.117	-0.123	0.377	0.745	0.829	598.117	-0.693	0.378	0.067	0.354	598.117	-0.570	0.436	0.191	0.413
FBXL5	475.982	-0.065	0.104	0.534	0.659	475.982	-0.048	0.103	0.643	0.806	475.982	0.017	0.120	0.888	0.946
FBXL4	560.158	-0.392	0.176	0.026	0.070	560.158	0.156	0.174	0.372	0.621	560.158	0.548	0.203	0.007	0.052
GATA2	794.286	-0.487	0.251	0.053	0.120	794.286	-0.467	0.251	0.063	0.350	794.286	0.020	0.291	0.944	0.974
FAM50B	16.322	0.791	0.525	0.132	0.240	16.322	1.107	0.514	0.031	0.322	16.322	0.315	0.589	0.592	0.772
KLHL3	31.652	0.268	0.726	0.712	0.803	31.652	0.803	0.720	0.265	0.537	31.652	0.535	0.834	0.521	0.724
GATA3	126.711	0.195	0.180	0.281	0.418	126.711	-0.247	0.183	0.178	0.466	126.711	-0.441	0.211	0.037	0.151
KCNG2	11.449	0.386	0.590	0.513	0.641	11.449	0.267	0.588	0.650	0.809	11.449	-0.119	0.679	0.860	0.933
CABYR	27.696	1.023	0.444	0.021	0.060	27.696	-0.079	0.461	0.864	0.931	27.696	-1.102	0.521	0.035	0.144
BLOC1S6	1154.150	-0.439	0.200	0.028	0.074	1154.150	0.001	0.199	0.996	0.998	1154.150	0.440	0.231	0.056	0.197
FBXW8	360.057	-0.220	0.221	0.319	0.459	360.057	0.033	0.220	0.881	0.940	360.057	0.253	0.255	0.321	0.556

FBXO25	705.810	0.229	0.216	0.287	0.426	705.810	0.273	0.215	0.205	0.488	705.810	0.044	0.249	0.861	0.933
TSPAN17	489.047	0.393	0.303	0.195	0.321	489.047	-0.421	0.304	0.167	0.456	489.047	-0.814	0.351	0.020	0.103
FBXO22	674.163	-0.174	0.246	0.478	0.612	674.163	-0.490	0.246	0.046	0.335	674.163	-0.316	0.284	0.267	0.502
SLC13A4	8.057	0.766	0.879	0.383	0.523	8.057	0.121	0.893	0.892	0.946	8.057	-0.644	1.020	0.528	0.728
FBXO10	105.183	-0.096	0.325	0.768	0.847	105.183	0.494	0.320	0.123	0.414	105.183	0.590	0.373	0.114	0.302
FBXO9	906.299	-0.155	0.106	0.143	0.256	906.299	0.093	0.105	0.374	0.622	906.299	0.248	0.122	0.042	0.164
FBXO8	299.472	-0.022	0.157	0.887	0.927	299.472	0.000	0.156	0.998	0.999	299.472	0.023	0.182	0.901	0.953
GATA6	288.237	-0.610	0.221	0.006	0.022	288.237	-0.296	0.219	0.177	0.465	288.237	0.314	0.256	0.220	0.446
FBXO6	151.136	1.604	0.236	0.000	0.000	151.136	0.426	0.243	0.079	0.370	151.136	-1.178	0.273	0.000	0.001
FBXO5	434.665	-1.156	0.204	0.000	0.000	434.665	-0.086	0.200	0.667	0.819	434.665	1.070	0.235	0.000	0.000
FBXO4	178.852	-0.398	0.194	0.040	0.098	178.852	-0.033	0.190	0.861	0.929	178.852	0.365	0.224	0.103	0.285
FBXO3	476.762	-0.305	0.153	0.047	0.110	476.762	-0.133	0.152	0.382	0.629	476.762	0.172	0.177	0.333	0.568
HIBCH	259.962	-0.354	0.180	0.049	0.114	259.962	-0.141	0.178	0.429	0.664	259.962	0.213	0.208	0.305	0.540
VPS33B	46.587	0.866	0.374	0.021	0.059	46.587	0.113	0.381	0.766	0.877	46.587	-0.752	0.435	0.084	0.251
TINF2	362.673	-0.409	0.197	0.038	0.094	362.673	0.019	0.195	0.924	0.962	362.673	0.428	0.228	0.060	0.206
SACS	575.622	-1.519	0.369	0.000	0.000	575.622	-0.954	0.368	0.009	0.297	575.622	0.565	0.427	0.185	0.405
GATM	7.702	1.263	0.588	0.032	0.082	7.702	0.030	0.658	0.964	0.981	7.702	-1.233	0.708	0.081	0.247
ERAL1	799.884	-0.189	0.159	0.234	0.367	799.884	-0.179	0.158	0.259	0.531	799.884	0.010	0.183	0.956	0.979
ARFGAP3	1136.169	-0.054	0.185	0.771	0.848	1136.169	0.010	0.184	0.955	0.978	1136.169	0.064	0.213	0.764	0.880
GBA	1275.565	0.462	0.178	0.009	0.032	1275.565	-0.081	0.178	0.651	0.810	1275.565	-0.543	0.205	0.008	0.057
FGF21	30.328	-4.007	0.686	0.000	0.000	30.328	-0.292	0.485	0.546	0.741	30.328	3.715	0.743	0.000	0.000
MYCBP	166.540	-0.708	0.309	0.022	0.062	166.540	-0.753	0.309	0.015	0.306	166.540	-0.046	0.359	0.899	0.952
SERGEF	348.398	0.883	0.319	0.006	0.022	348.398	0.546	0.319	0.087	0.380	348.398	-0.337	0.368	0.360	0.593
EHF	40.043	0.579	0.325	0.074	0.156	40.043	0.458	0.323	0.157	0.449	40.043	-0.121	0.371	0.744	0.868
GBAP1	186.661	0.595	0.208	0.004	0.017	186.661	-0.146	0.212	0.491	0.707	186.661	-0.741	0.242	0.002	0.023
NIPSNAP2	976.750	-0.321	0.234	0.171	0.291	976.750	0.264	0.234	0.258	0.530	976.750	0.586	0.270	0.030	0.133
GBE1	4951.074	0.022	0.289	0.941	0.963	4951.074	0.402	0.288	0.163	0.454	4951.074	0.381	0.333	0.253	0.486
GBP1	355.640	0.600	0.201	0.003	0.013	355.640	0.086	0.202	0.672	0.822	355.640	-0.515	0.233	0.027	0.123
GAPDHS	36.608	-0.430	0.320	0.179	0.301	36.608	-0.162	0.311	0.602	0.780	36.608	0.268	0.370	0.468	0.683
GBP2	830.516	1.119	0.497	0.024	0.067	830.516	0.584	0.497	0.240	0.516	830.516	-0.535	0.574	0.351	0.584
GBP3	41.778	0.154	0.291	0.597	0.711	41.778	-0.059	0.292	0.840	0.917	41.778	-0.213	0.339	0.530	0.730
HSPB8	1332.014	0.565	0.122	0.000	0.000	1332.014	-0.132	0.123	0.283	0.552	1332.014	-0.696	0.141	0.000	0.000
GNL3	2742.991	-0.517	0.166	0.002	0.010	2742.991	-0.248	0.166	0.135	0.426	2742.991	0.268	0.192	0.162	0.376
FAM162A	5072.764	1.205	0.446	0.007	0.025	5072.764	0.654	0.446	0.143	0.434	5072.764	-0.551	0.515	0.284	0.519
GCDH	358.644	-0.292	0.226	0.197	0.324	358.644	-0.358	0.226	0.113	0.405	358.644	-0.067	0.262	0.799	0.900
GCH1	192.771	0.174	0.380	0.648	0.754	192.771	-0.899	0.385	0.019	0.306	192.771	-1.073	0.444	0.016	0.087
GCHFR	170.093	0.495	0.388	0.202	0.329	170.093	0.642	0.387	0.097	0.392	170.093	0.147	0.447	0.743	0.868
PTPN18	509.584	0.276	0.155	0.074	0.156	509.584	0.265	0.154	0.085	0.378	509.584	-0.011	0.178	0.950	0.977
BLOC1S1	150.372	0.611	0.318	0.055	0.124	150.372	0.424	0.318	0.183	0.470	150.372	-0.187	0.367	0.611	0.785
SEZ6L2	832.446	1.263	0.494	0.011	0.036	832.446	0.856	0.494	0.083	0.375	832.446	-0.407	0.570	0.476	0.689
NUPR1	2981.611	1.440	0.320	0.000	0.000	2981.611	0.646	0.320	0.044	0.331	2981.611	-0.794	0.369	0.031	0.136
PPP1R14B	4454.666	0.669	0.279	0.016	0.050	4454.666	-0.028	0.279	0.921	0.960	4454.666	-0.697	0.322	0.031	0.133
KAT2A	894.805	0.328	0.187	0.079	0.165	894.805	-0.069	0.187	0.714	0.847	894.805	-0.397	0.216	0.067	0.218
NR6A1	50.738	0.330	0.273	0.227	0.359	50.738	0.129	0.274	0.638	0.803	50.738	-0.201	0.316	0.525	0.726
PLEK2	797.775	-0.177	0.209	0.397	0.535	797.775	-0.049	0.209	0.813	0.902	797.775	0.128	0.242	0.597	0.776
GCNT1	92.284	-0.060	0.221	0.786	0.858	92.284	0.102	0.217	0.639	0.803	92.284	0.162	0.254	0.525	0.726
NARF	793.623	0.484	0.187	0.010	0.033	793.623	0.310	0.187	0.097	0.392	793.623	-0.174	0.216	0.420	0.644
SLC17A5	612.262	0.859	0.245	0.000	0.003	612.262	0.388	0.246	0.114	0.406	612.262	-0.470	0.283	0.097	0.275
CNNM4	392.124	-0.642	0.241	0.008	0.028	392.124	-0.149	0.239	0.534	0.733	392.124	0.493	0.279	0.077	0.239
CNNM3	579.664	0.120	0.111	0.280	0.418	579.664	-0.001	0.111	0.994	0.997	579.664	-0.121	0.129	0.348	0.582
MYOF	7813.864	-0.662	0.238	0.005	0.021	7813.864	0.144	0.238	0.543	0.739	7813.864	0.806	0.275	0.003	0.031
GCNT2	490.253	-0.100	0.256	0.696	0.791	490.253	-0.500	0.257	0.052	0.339	490.253	-0.400	0.297	0.178	0.396
CHIC2	271.993	-0.756	0.389	0.052	0.120	271.993	-0.781	0.389	0.044	0.333	271.993	-0.026	0.451	0.955	0.979
INTS6	885.624	-0.713	0.174	0.000	0.000	885.624	-0.309	0.173	0.075	0.364	885.624	0.404	0.201	0.044	0.170
TIMM10B	254.560	-0.330	0.272	0.224	0.355	254.560	-0.585	0.272	0.031	0.322	254.560	-0.255	0.315	0.418	0.643
TIMM13	1428.181	-0.299	0.303	0.325	0.465	1428.181	-0.402	0.303	0.184	0.471	1428.181	-0.104	0.350	0.767	0.881
TIMM10	295.215	-0.568	0.347	0.102	0.198	295.215	-0.624	0.347	0.072	0.361	295.215	-0.056	0.402	0.889	0.947
TIMM9	683.326	0.004	0.170	0.980	0.988	683.326	-0.038	0.169	0.821	0.906	683.326	-0.043	0.196	0.828	0.915
TIMM8B	342.395	0.342	0.280	0.221	0.352	342.395	-0.353	0.281	0.209	0.490	342.395	-0.695	0.324	0.032	0.138
AGO1	610.329	-0.154	0.183	0.399	0.538	610.329	0.001	0.183	0.995	0.997	610.329	0.156	0.212	0.462	0.679
LATS2	357.677	-0.265	0.209	0.206	0.333	357.677	-0.043	0.208	0.835	0.915	357.677	0.222	0.242	0.359	0.591
DAZAP1	4215.903	-0.437	0.203	0.032	0.082	4215.903	-0.210	0.203	0.300	0.567	4215.903	0.226	0.235	0.335	0.569
GCSH	366.433	-0.043	0.279	0.879	0.921	366.433	-0.452	0.280	0.106	0.398	366.433	-0.410	0.323	0.205	0.429
GDF1	16.441	1.266	0.553	0.022	0.062	16.441	0.259	0.583	0.657	0.813	16.441	-1.007	0.645	0.119	0.309
AATF	4284.689	-0.279	0.199	0.159	0.277	4284.689	-0.083	0.198	0.677	0.825	4284.689	0.197	0.229	0.391	0.619
RGS17	387.591	0.077	0.481	0.873	0.918	387.591	-1.464	0.486	0.003	0.297	387.591	-1.541	0.560	0.006	0.046
PCOLCE2	685.472	0.430	0.150	0.004	0.017	685.472	0.112	0.150	0.456	0.683	685.472	-0.318	0.173	0.066	0.216

OSTF1	386.975	0.631	0.129	0.000	0.000	386.975	-0.005	0.131	0.969	0.984	386.975	-0.636	0.150	0.000	0.001
MYEOV	365.743	-0.339	0.277	0.222	0.353	365.743	-0.174	0.277	0.528	0.729	365.743	0.164	0.321	0.608	0.783
BSCL2	155.265	-0.180	0.255	0.481	0.615	155.265	0.213	0.251	0.397	0.641	155.265	0.392	0.293	0.181	0.399
CKAP2	1349.317	-0.588	0.261	0.024	0.067	1349.317	0.164	0.261	0.528	0.729	1349.317	0.752	0.301	0.013	0.076
MRPL46	147.524	-0.618	0.265	0.020	0.057	147.524	-0.359	0.262	0.171	0.460	147.524	0.259	0.307	0.399	0.627
TBL2	799.135	-0.135	0.172	0.431	0.568	799.135	0.133	0.171	0.434	0.667	799.135	0.268	0.198	0.175	0.392
GDF9	209.990	0.745	0.266	0.005	0.020	209.990	-0.049	0.269	0.856	0.927	209.990	-0.794	0.308	0.010	0.066
ELP4	285.871	0.318	0.182	0.081	0.167	285.871	0.108	0.183	0.553	0.745	285.871	-0.210	0.211	0.320	0.555
GDI1	2060.112	0.119	0.118	0.311	0.451	2060.112	0.088	0.118	0.452	0.680	2060.112	-0.031	0.136	0.821	0.911
GDI2	6695.884	-0.434	0.177	0.014	0.044	6695.884	0.110	0.176	0.532	0.732	6695.884	0.544	0.204	0.008	0.055
BRD3OS	812.541	0.292	0.198	0.140	0.251	812.541	0.295	0.198	0.135	0.426	812.541	0.004	0.228	0.988	0.993
HS6ST3	130.969	-1.760	0.325	0.000	0.000	130.969	0.082	0.304	0.788	0.889	130.969	1.842	0.370	0.000	0.000
MDGA1	16.784	3.779	0.758	0.000	0.000	16.784	0.719	0.813	0.376	0.624	16.784	-3.059	0.874	0.000	0.008
MAGEA2B	1008.150	0.028	0.227	0.901	0.937	1008.150	-0.330	0.228	0.147	0.439	1008.150	-0.358	0.263	0.173	0.390
RGL4	30.932	0.796	0.501	0.112	0.213	30.932	0.253	0.505	0.617	0.789	30.932	-0.543	0.579	0.348	0.582
NAP1L5	195.127	-0.117	0.197	0.553	0.675	195.127	-0.502	0.199	0.011	0.305	195.127	-0.385	0.230	0.094	0.270
GEM	97.548	1.508	0.324	0.000	0.000	97.548	-0.045	0.340	0.896	0.948	97.548	-1.552	0.381	0.000	0.001
PIPSL	20.194	-0.976	0.467	0.036	0.091	20.194	-0.566	0.447	0.205	0.488	20.194	0.410	0.542	0.450	0.669
AMFR	2505.092	0.063	0.122	0.607	0.719	2505.092	0.156	0.122	0.200	0.483	2505.092	0.093	0.141	0.508	0.713
GFAP	21.286	-0.033	0.543	0.951	0.970	21.286	0.633	0.523	0.226	0.505	21.286	0.667	0.616	0.279	0.514
PGBD2	59.700	-0.167	0.277	0.547	0.671	59.700	-0.639	0.283	0.024	0.316	59.700	-0.472	0.330	0.152	0.361
GFER	605.921	0.228	0.138	0.098	0.193	605.921	0.172	0.137	0.212	0.492	605.921	-0.056	0.159	0.724	0.857
GFI1	46.928	-0.418	0.429	0.330	0.470	46.928	0.300	0.418	0.474	0.695	46.928	0.717	0.492	0.145	0.351
GFPT1	1585.687	-0.720	0.180	0.000	0.001	1585.687	-0.162	0.179	0.365	0.617	1585.687	0.558	0.208	0.007	0.053
NUFIP1	176.239	-0.966	0.391	0.014	0.043	176.239	-0.693	0.389	0.075	0.364	176.239	0.273	0.453	0.547	0.742
RPS6KC1	341.048	0.258	0.184	0.159	0.277	341.048	-0.249	0.185	0.179	0.468	341.048	-0.507	0.214	0.018	0.094
SH3YL1	158.684	-0.019	0.398	0.961	0.977	158.684	0.362	0.396	0.360	0.612	158.684	0.382	0.459	0.405	0.632
SNORA73B	13.245	0.186	0.524	0.723	0.812	13.245	-0.450	0.542	0.407	0.648	13.245	-0.635	0.624	0.309	0.544
GGCX	1423.200	-0.017	0.107	0.876	0.920	1423.200	-0.063	0.106	0.555	0.746	1423.200	-0.046	0.123	0.708	0.848
GGT1	50.997	1.213	0.279	0.000	0.000	50.997	0.116	0.299	0.697	0.837	50.997	-1.097	0.329	0.001	0.012
AMH	256.610	2.098	0.455	0.000	0.000	256.610	1.100	0.456	0.016	0.306	256.610	-0.998	0.524	0.057	0.199
GGTA1P	6.606	-0.828	1.176	0.481	0.615	6.606	-0.211	1.146	0.854	0.925	6.606	0.617	1.355	0.649	0.811
B4GALT1	6267.256	-0.106	0.211	0.616	0.727	6267.256	0.291	0.211	0.167	0.457	6267.256	0.397	0.243	0.103	0.286
SNORD3B-1	17.220	0.317	0.739	0.668	0.769	17.220	-1.624	0.792	0.040	0.328	17.220	-1.941	0.900	0.031	0.135
GGT7	38.022	0.795	0.507	0.117	0.220	38.022	0.520	0.508	0.306	0.571	38.022	-0.275	0.583	0.637	0.803
RNVU1-18	42.858	1.503	0.638	0.019	0.054	42.858	0.950	0.652	0.146	0.438	42.858	-0.554	0.716	0.439	0.660
GGT5	181.735	1.577	0.405	0.000	0.001	181.735	1.256	0.406	0.002	0.297	181.735	-0.321	0.465	0.490	0.700
STEAP1	462.746	0.122	0.313	0.698	0.792	462.746	-0.617	0.314	0.050	0.337	462.746	-0.739	0.363	0.042	0.164
OPLAH	255.490	0.575	0.176	0.001	0.006	255.490	0.244	0.177	0.168	0.457	255.490	-0.331	0.204	0.104	0.288
GHR	20.837	-0.757	0.570	0.184	0.308	20.837	0.026	0.550	0.963	0.980	20.837	0.783	0.654	0.231	0.460
RANBP6	254.829	-0.290	0.250	0.247	0.381	254.829	-0.563	0.251	0.025	0.316	254.829	-0.273	0.291	0.347	0.581
COPG2	540.168	-0.040	0.211	0.848	0.901	540.168	0.213	0.210	0.311	0.575	540.168	0.253	0.243	0.298	0.534
HBP1	1001.948	-0.025	0.311	0.936	0.960	1001.948	0.339	0.310	0.274	0.545	1001.948	0.364	0.359	0.310	0.545
GIPR	22.810	1.634	0.940	0.082	0.169	22.810	0.811	0.947	0.392	0.637	22.810	-0.823	1.082	0.447	0.666
NBEA	108.439	-0.561	0.407	0.169	0.289	108.439	0.181	0.402	0.653	0.811	108.439	0.742	0.469	0.114	0.302
GJA1	3164.317	-0.548	0.249	0.028	0.074	3164.317	-0.241	0.249	0.333	0.592	3164.317	0.307	0.288	0.286	0.521
CHORDC1	1199.726	-0.490	0.385	0.203	0.330	1199.726	-1.170	0.385	0.002	0.297	1199.726	-0.679	0.445	0.127	0.322
ZNF285	48.760	0.066	0.301	0.828	0.887	48.760	0.509	0.292	0.081	0.372	48.760	0.444	0.342	0.195	0.417
SEC22A	359.057	-0.167	0.145	0.247	0.381	359.057	-0.071	0.143	0.620	0.791	359.057	0.096	0.167	0.564	0.754
AP3M1	901.874	-0.506	0.201	0.012	0.038	901.874	-0.276	0.200	0.167	0.457	901.874	0.230	0.232	0.322	0.556
PABPC1	35400.136	-0.128	0.183	0.484	0.617	35400.136	0.217	0.183	0.236	0.513	35400.136	0.344	0.211	0.103	0.285
AKAP8L	1236.822	0.644	0.202	0.001	0.008	1236.822	0.257	0.203	0.204	0.487	1236.822	-0.387	0.234	0.098	0.276
RNF11	1542.626	0.054	0.128	0.672	0.772	1542.626	-0.017	0.128	0.896	0.948	1542.626	-0.071	0.148	0.632	0.799
TRUB2	730.629	-0.156	0.156	0.318	0.457	730.629	-0.094	0.155	0.544	0.739	730.629	0.062	0.180	0.732	0.861
GPR160	123.059	0.326	0.470	0.488	0.621	123.059	0.822	0.467	0.078	0.370	123.059	0.496	0.540	0.358	0.591
CYFIP2	903.310	-1.148	0.497	0.021	0.059	903.310	0.559	0.496	0.259	0.531	903.310	1.708	0.574	0.003	0.028
ABL2	1380.158	-0.075	0.186	0.686	0.783	1380.158	-0.317	0.186	0.088	0.381	1380.158	-0.242	0.215	0.260	0.495
GJA3	11.825	0.708	0.549	0.197	0.323	11.825	0.769	0.538	0.153	0.445	11.825	0.061	0.612	0.921	0.963
DNAJC2	653.360	-0.091	0.309	0.769	0.847	653.360	-0.542	0.309	0.080	0.371	653.360	-0.451	0.357	0.207	0.431
USP21	359.517	-0.221	0.132	0.092	0.185	359.517	0.202	0.128	0.116	0.408	359.517	0.423	0.151	0.005	0.041
TPK1	140.554	0.087	0.228	0.702	0.795	140.554	-0.201	0.229	0.380	0.627	140.554	-0.288	0.265	0.277	0.511
KCNV1	14.975	0.972	0.370	0.009	0.030	14.975	-0.117	0.407	0.774	0.879	14.975	-1.089	0.447	0.015	0.085
CNPPD1	775.446	0.238	0.115	0.039	0.096	775.446	0.096	0.115	0.405	0.646	775.446	-0.142	0.133	0.287	0.522
BEX3	121.189	0.505	0.425	0.234	0.367	121.189	0.370	0.425	0.384	0.630	121.189	-0.135	0.490	0.782	0.891
NPTN	1202.123	0.347	0.110	0.002	0.008	1202.123	-0.098	0.110	0.374	0.622	1202.123	-0.445	0.127	0.000	0.008
FOXD3	12.080	0.913	0.681	0.180	0.303	12.080	0.348	0.690	0.614	0.787	12.080	-0.565	0.786	0.472	0.686
MLH3	95.303	-0.061	0.199	0.760	0.841	95.303	0.234	0.192	0.222	0.501	95.303	0.295	0.227	0.194	0.416

NPHP3	75.502	1.304	0.419	0.002	0.009	75.502	0.888	0.420	0.035	0.322	75.502	-0.416	0.481	0.387	0.615
ATP2C1	3251.823	0.028	0.172	0.870	0.916	3251.823	0.211	0.172	0.220	0.499	3251.823	0.183	0.199	0.358	0.591
ACAD8	137.414	0.514	0.175	0.003	0.014	137.414	-0.233	0.181	0.198	0.482	137.414	-0.747	0.206	0.000	0.005
NOX1	12.080	0.574	0.465	0.217	0.347	12.080	-0.454	0.505	0.369	0.619	12.080	-1.028	0.567	0.070	0.225
TRMT2A	593.378	-0.283	0.331	0.393	0.532	593.378	-0.620	0.331	0.061	0.350	593.378	-0.338	0.383	0.377	0.609
PKD2L2	48.902	-0.730	0.343	0.033	0.085	48.902	-0.358	0.330	0.278	0.548	48.902	0.372	0.397	0.349	0.582
UTP25	777.709	-0.595	0.178	0.001	0.005	777.709	-0.053	0.177	0.763	0.874	777.709	0.542	0.206	0.008	0.058
PELP1	1020.312	0.042	0.220	0.850	0.902	1020.312	-0.180	0.220	0.414	0.652	1020.312	-0.222	0.255	0.384	0.613
SND1	7844.849	-0.256	0.168	0.128	0.235	7844.849	0.196	0.168	0.244	0.519	7844.849	0.452	0.194	0.020	0.102
GJB2	116.494	1.362	0.333	0.000	0.000	116.494	0.538	0.339	0.112	0.405	116.494	-0.824	0.384	0.032	0.137
ANKRD1	32.669	0.276	0.464	0.552	0.674	32.669	-0.713	0.478	0.136	0.427	32.669	-0.989	0.548	0.071	0.228
NSG1	34.899	-1.486	0.387	0.000	0.001	34.899	-0.675	0.359	0.060	0.350	34.899	0.811	0.446	0.069	0.223
STAU2	439.059	-0.036	0.184	0.847	0.900	439.059	0.084	0.183	0.646	0.807	439.059	0.120	0.213	0.573	0.760
PPA2	659.023	-0.287	0.155	0.064	0.139	659.023	0.136	0.153	0.375	0.622	659.023	0.423	0.178	0.018	0.094
GHITM	5759.219	-0.527	0.161	0.001	0.006	5759.219	-0.291	0.161	0.071	0.360	5759.219	0.237	0.186	0.204	0.428
GJB3	106.996	0.870	0.257	0.001	0.005	106.996	0.870	0.256	0.001	0.258	106.996	0.000	0.292	1.000	1.000
VPS41	546.673	-0.209	0.246	0.396	0.535	546.673	0.055	0.245	0.824	0.908	546.673	0.263	0.284	0.354	0.587
TSPAN13	754.753	-0.556	0.100	0.000	0.000	754.753	-0.126	0.097	0.194	0.478	754.753	0.430	0.115	0.000	0.004
LYPD3	21.753	1.661	0.613	0.007	0.025	21.753	0.963	0.633	0.128	0.420	21.753	-0.698	0.686	0.308	0.544
B9D1	270.350	0.242	0.221	0.274	0.410	270.350	0.369	0.220	0.093	0.388	270.350	0.127	0.254	0.617	0.790
RPUSD2	264.906	-0.171	0.269	0.526	0.652	264.906	-0.340	0.269	0.206	0.488	264.906	-0.170	0.312	0.586	0.767
MTBP	216.987	-0.644	0.259	0.013	0.041	216.987	-0.182	0.255	0.476	0.697	216.987	0.462	0.298	0.122	0.313
FOXP1	500.871	0.009	0.164	0.955	0.973	500.871	-0.208	0.164	0.206	0.488	500.871	-0.217	0.190	0.254	0.486
UQCRQ	1116.749	0.834	0.275	0.002	0.011	1116.749	-0.143	0.276	0.605	0.781	1116.749	-0.977	0.318	0.002	0.023
GJB5	40.642	0.933	0.476	0.050	0.116	40.642	1.061	0.473	0.025	0.316	40.642	0.127	0.541	0.814	0.907
ST6GALNAC4	992.494	-0.128	0.174	0.460	0.595	992.494	0.119	0.173	0.492	0.707	992.494	0.247	0.201	0.217	0.444
KCNMB3	149.795	0.075	0.141	0.594	0.709	149.795	0.370	0.135	0.006	0.297	149.795	0.295	0.160	0.064	0.213
TRAPPC3	897.092	0.440	0.176	0.012	0.040	897.092	-0.209	0.177	0.239	0.515	897.092	-0.649	0.204	0.001	0.018
TAF5L	538.741	-0.602	0.202	0.003	0.013	538.741	-0.312	0.201	0.120	0.412	538.741	0.290	0.234	0.216	0.441
AMPD2	1845.771	-0.189	0.204	0.354	0.495	1845.771	0.336	0.203	0.098	0.392	1845.771	0.525	0.235	0.026	0.119
GK	92.240	1.212	0.300	0.000	0.001	92.240	-0.194	0.316	0.540	0.737	92.240	-1.406	0.355	0.000	0.002
CACYBP	2372.007	-0.214	0.274	0.435	0.571	2372.007	-0.549	0.274	0.045	0.334	2372.007	-0.335	0.317	0.290	0.526
EIF2AK1	4759.719	0.023	0.099	0.812	0.877	4759.719	-0.041	0.099	0.678	0.826	4759.719	-0.064	0.114	0.572	0.760
ARRDC2	1383.203	0.665	0.300	0.027	0.071	1383.203	0.498	0.300	0.096	0.391	1383.203	-0.166	0.346	0.631	0.799
ZBTB11	686.330	-0.784	0.218	0.000	0.002	686.330	-0.354	0.216	0.102	0.394	686.330	0.430	0.251	0.087	0.257
DMAC2L	111.323	0.067	0.237	0.777	0.852	111.323	-0.153	0.238	0.519	0.724	111.323	-0.220	0.276	0.424	0.648
SDCBP2	59.115	0.834	0.407	0.040	0.098	59.115	0.508	0.410	0.215	0.494	59.115	-0.327	0.467	0.484	0.696
BBC3	589.432	0.333	0.200	0.095	0.189	589.432	0.171	0.200	0.391	0.635	589.432	-0.161	0.230	0.483	0.696
PDE7B	7.422	-0.711	0.874	0.416	0.553	7.422	0.124	0.835	0.882	0.940	7.422	0.835	0.999	0.403	0.630
DKK3	5242.295	-0.149	0.339	0.660	0.762	5242.295	0.270	0.339	0.426	0.662	5242.295	0.419	0.391	0.284	0.519
INPP5J	47.095	0.630	0.265	0.017	0.052	47.095	0.218	0.269	0.418	0.656	47.095	-0.413	0.307	0.178	0.396
AFF4	2203.490	-0.780	0.297	0.009	0.030	2203.490	-0.144	0.296	0.628	0.796	2203.490	0.637	0.343	0.063	0.211
CYTH4	13.257	1.519	0.713	0.033	0.085	13.257	-0.139	0.791	0.860	0.929	13.257	-1.659	0.861	0.054	0.192
INVS	437.876	-0.497	0.167	0.003	0.013	437.876	0.021	0.164	0.900	0.950	437.876	0.518	0.192	0.007	0.052
SNX5	1780.395	-0.572	0.091	0.000	0.000	1780.395	-0.025	0.090	0.786	0.888	1780.395	0.548	0.105	0.000	0.000
CPNE7	810.720	0.748	0.159	0.000	0.000	810.720	0.225	0.160	0.159	0.451	810.720	-0.523	0.184	0.005	0.038
TJP3	26.955	0.879	0.493	0.074	0.156	26.955	0.482	0.497	0.331	0.591	26.955	-0.397	0.566	0.484	0.696
CIDEB	310.962	-0.394	0.271	0.146	0.259	310.962	-0.468	0.270	0.084	0.375	310.962	-0.074	0.314	0.813	0.907
PALD1	393.446	-0.360	0.152	0.018	0.053	393.446	-0.065	0.150	0.664	0.817	393.446	0.295	0.176	0.093	0.267
FILIP1	22.076	0.943	0.811	0.245	0.378	22.076	0.599	0.813	0.461	0.687	22.076	-0.344	0.934	0.712	0.850
FAM184B	27.963	1.576	0.353	0.000	0.000	27.963	0.763	0.365	0.037	0.324	27.963	-0.812	0.401	0.043	0.165
DENND2A	86.429	-0.483	0.337	0.152	0.267	86.429	0.316	0.329	0.337	0.595	86.429	0.799	0.386	0.039	0.155
STK36	121.296	0.128	0.235	0.584	0.701	121.296	0.153	0.233	0.513	0.720	121.296	0.024	0.271	0.929	0.967
CPAMD8	907.482	-0.111	0.667	0.868	0.915	907.482	0.748	0.667	0.262	0.535	907.482	0.858	0.770	0.265	0.499
INTU	148.726	-0.092	0.275	0.738	0.823	148.726	0.038	0.273	0.890	0.945	148.726	0.130	0.317	0.682	0.832
ZNF777	501.141	-0.360	0.240	0.133	0.242	501.141	-0.486	0.240	0.043	0.330	501.141	-0.126	0.278	0.651	0.812
BRPF3	501.570	-0.389	0.191	0.042	0.101	501.570	-0.078	0.190	0.683	0.828	501.570	0.312	0.221	0.158	0.370
NDOR1	165.050	-0.585	0.457	0.201	0.328	165.050	-0.885	0.458	0.053	0.342	165.050	-0.300	0.530	0.572	0.760
AGO2	1277.875	-1.015	0.315	0.001	0.007	1277.875	-0.679	0.315	0.031	0.322	1277.875	0.336	0.364	0.356	0.589
NAAA	186.136	0.176	0.141	0.210	0.339	186.136	0.218	0.139	0.115	0.406	186.136	0.042	0.162	0.794	0.896
GLS2	12.851	0.602	0.507	0.235	0.368	12.851	0.234	0.514	0.649	0.808	12.851	-0.368	0.586	0.530	0.729
PRELID1	3241.924	-0.211	0.283	0.457	0.592	3241.924	-0.375	0.283	0.186	0.473	3241.924	-0.164	0.327	0.617	0.790
GLA	470.069	0.169	0.298	0.570	0.689	470.069	-0.585	0.299	0.051	0.338	470.069	-0.754	0.345	0.029	0.128
SLC39A1	2968.881	0.365	0.097	0.000	0.001	2968.881	0.206	0.097	0.034	0.322	2968.881	-0.160	0.112	0.154	0.363
TUBG2	213.357	0.681	0.205	0.001	0.005	213.357	0.513	0.205	0.012	0.305	213.357	-0.169	0.235	0.474	0.688
VPS4A	2588.001	-0.235	0.165	0.155	0.271	2588.001	-0.181	0.165	0.273	0.544	2588.001	0.054	0.191	0.778	0.888
DISC1	197.460	1.491	0.226	0.000	0.000	197.460	0.232	0.230	0.313	0.576	197.460	-1.258	0.262	0.000	0.000

AMPD3	152.084	0.140	0.293	0.632	0.740	152.084	0.452	0.289	0.117	0.409	152.084	0.312	0.336	0.353	0.586
GLB1	1161.999	0.440	0.139	0.002	0.008	1161.999	0.068	0.139	0.623	0.793	1161.999	-0.371	0.160	0.020	0.103
C5AR2	101.988	-0.109	0.375	0.770	0.847	101.988	0.794	0.365	0.030	0.322	101.988	0.903	0.428	0.035	0.145
TUBGCP4	1172.846	-0.144	0.108	0.182	0.305	1172.846	-0.081	0.108	0.450	0.679	1172.846	0.063	0.125	0.614	0.787
SERP1	4920.757	-0.313	0.158	0.047	0.110	4920.757	-0.180	0.157	0.253	0.527	4920.757	0.134	0.182	0.463	0.679
COQ2	314.272	-0.144	0.237	0.543	0.668	314.272	-0.221	0.236	0.351	0.605	314.272	-0.077	0.274	0.780	0.889
ARFIP1	480.079	-0.353	0.157	0.024	0.066	480.079	0.014	0.155	0.926	0.962	480.079	0.368	0.181	0.042	0.163
ARHGEF16	532.938	0.744	0.340	0.029	0.076	532.938	0.738	0.340	0.030	0.322	532.938	-0.005	0.392	0.989	0.994
GPKOW	411.705	-0.345	0.177	0.051	0.117	411.705	-0.161	0.175	0.358	0.611	411.705	0.183	0.204	0.369	0.601
BBS9	115.340	0.058	0.378	0.879	0.921	115.340	0.542	0.375	0.148	0.441	115.340	0.484	0.435	0.266	0.500
TNFRSF21	59.194	-0.608	0.380	0.109	0.209	59.194	-0.891	0.381	0.019	0.306	59.194	-0.283	0.446	0.526	0.727
CHMP2A	1903.737	0.930	0.249	0.000	0.002	1903.737	0.381	0.249	0.126	0.418	1903.737	-0.550	0.287	0.055	0.196
SESN1	522.967	-0.249	0.455	0.584	0.700	522.967	0.397	0.454	0.382	0.628	522.967	0.647	0.525	0.218	0.445
AHDC1	956.335	0.563	0.372	0.130	0.238	956.335	0.514	0.372	0.167	0.456	956.335	-0.049	0.429	0.910	0.959
RNF115	556.571	-0.018	0.174	0.919	0.949	556.571	-0.365	0.175	0.037	0.324	556.571	-0.348	0.202	0.085	0.255
NFU1	395.191	0.365	0.203	0.072	0.152	395.191	0.299	0.202	0.140	0.431	395.191	-0.067	0.234	0.775	0.886
ERLEC1	477.679	-0.115	0.187	0.538	0.663	477.679	-0.001	0.186	0.997	0.999	477.679	0.114	0.216	0.596	0.775
MMADHC	1682.423	-0.166	0.105	0.115	0.216	1682.423	-0.041	0.105	0.699	0.838	1682.423	0.125	0.121	0.302	0.537
PDCD4	3969.887	-0.645	0.370	0.081	0.167	3969.887	0.354	0.369	0.338	0.596	3969.887	0.999	0.427	0.019	0.099
KLHL20	317.154	-0.764	0.168	0.000	0.000	317.154	-0.281	0.165	0.088	0.380	317.154	0.482	0.194	0.013	0.078
PCDH17	216.030	-0.206	0.374	0.583	0.700	216.030	0.264	0.372	0.479	0.699	216.030	0.469	0.431	0.277	0.511
CSDC2	205.339	0.494	0.234	0.035	0.088	205.339	0.551	0.233	0.018	0.306	205.339	0.056	0.269	0.834	0.919
LSM1	480.120	0.135	0.268	0.615	0.726	480.120	-0.510	0.269	0.058	0.347	480.120	-0.644	0.310	0.038	0.153
LSM3	932.266	0.185	0.177	0.296	0.434	932.266	-0.132	0.178	0.459	0.685	932.266	-0.317	0.205	0.122	0.314
SRPX2	53.344	-0.304	0.310	0.326	0.467	53.344	-0.124	0.303	0.682	0.827	53.344	0.180	0.358	0.616	0.789
RND1	7.738	1.386	0.659	0.035	0.089	7.738	0.857	0.675	0.204	0.487	7.738	-0.529	0.738	0.474	0.688
GCLC	386.470	-0.346	0.201	0.085	0.173	386.470	-0.458	0.201	0.022	0.315	386.470	-0.112	0.233	0.631	0.799
R3HCC1L	305.164	-0.664	0.214	0.002	0.010	305.164	-0.322	0.212	0.129	0.421	305.164	0.343	0.248	0.167	0.381
DIMT1	1682.009	-0.573	0.215	0.008	0.027	1682.009	-0.454	0.214	0.034	0.322	1682.009	0.119	0.248	0.631	0.799
SMPDL3B	31.383	1.474	0.399	0.000	0.002	31.383	0.526	0.412	0.202	0.485	31.383	-0.947	0.460	0.039	0.157
PDLIM3	91.536	0.457	0.351	0.193	0.318	91.536	-0.317	0.356	0.373	0.622	91.536	-0.774	0.409	0.059	0.202
TP53TG5	10.765	0.414	0.605	0.494	0.626	10.765	0.704	0.584	0.228	0.506	10.765	0.290	0.679	0.669	0.824
CRCP	436.897	-0.717	0.197	0.000	0.002	436.897	-0.345	0.195	0.078	0.369	436.897	0.372	0.228	0.103	0.285
GCLM	1105.869	-0.188	0.285	0.510	0.639	1105.869	-0.611	0.285	0.032	0.322	1105.869	-0.424	0.329	0.198	0.421
ZNF544	301.681	0.005	0.226	0.983	0.990	301.681	-0.639	0.228	0.005	0.297	301.681	-0.644	0.263	0.014	0.083
APEX2	414.737	-0.345	0.262	0.188	0.313	414.737	-0.336	0.261	0.199	0.482	414.737	0.009	0.303	0.977	0.988
RBMS3	402.005	0.219	0.265	0.407	0.546	402.005	0.619	0.264	0.019	0.306	402.005	0.400	0.305	0.189	0.411
MOCS3	158.832	-0.280	0.208	0.177	0.299	158.832	-0.174	0.205	0.396	0.640	158.832	0.106	0.241	0.659	0.818
ZNF330	1210.207	-0.270	0.153	0.077	0.161	1210.207	-0.296	0.153	0.053	0.341	1210.207	-0.026	0.177	0.885	0.945
GLDC	23.847	-0.555	0.491	0.258	0.393	23.847	0.964	0.457	0.035	0.322	23.847	1.520	0.551	0.006	0.045
RAB30	110.183	1.154	0.451	0.011	0.036	110.183	0.499	0.453	0.271	0.542	110.183	-0.655	0.521	0.209	0.433
PGAP2	205.569	-0.033	0.254	0.897	0.934	205.569	0.380	0.251	0.130	0.422	205.569	0.413	0.292	0.157	0.368
RBMX	5714.091	-0.376	0.158	0.017	0.051	5714.091	0.149	0.157	0.342	0.599	5714.091	0.526	0.182	0.004	0.034
TNRC6A	790.070	-0.339	0.184	0.066	0.143	790.070	-0.194	0.184	0.292	0.559	790.070	0.145	0.213	0.496	0.704
GLE1	805.835	-0.399	0.130	0.002	0.010	805.835	0.092	0.128	0.475	0.696	805.835	0.491	0.150	0.001	0.014
ZNF638	1254.478	-0.068	0.140	0.625	0.735	1254.478	-0.074	0.140	0.595	0.775	1254.478	-0.006	0.162	0.971	0.986
GOLIM4	1841.506	-0.233	0.147	0.114	0.215	1841.506	-0.164	0.147	0.264	0.536	1841.506	0.069	0.170	0.687	0.835
EIF3K	2323.067	0.156	0.130	0.232	0.364	2323.067	0.145	0.130	0.265	0.537	2323.067	-0.011	0.151	0.944	0.974
HTATSF1	1187.651	-0.197	0.168	0.241	0.374	1187.651	-0.202	0.168	0.230	0.507	1187.651	-0.005	0.195	0.981	0.990
UBE2S	6345.201	-0.038	0.250	0.878	0.921	6345.201	-0.217	0.250	0.386	0.632	6345.201	-0.178	0.288	0.536	0.734
PRPF19	2839.132	-0.393	0.269	0.144	0.256	2839.132	-0.519	0.269	0.054	0.342	2839.132	-0.125	0.311	0.687	0.835
GLG1	3174.833	0.088	0.203	0.665	0.767	3174.833	0.341	0.203	0.094	0.388	3174.833	0.253	0.235	0.282	0.516
UTP20	445.342	-1.332	0.361	0.000	0.002	445.342	-0.845	0.360	0.019	0.306	445.342	0.487	0.418	0.244	0.475
RRP7A	824.026	-0.605	0.458	0.186	0.310	824.026	-1.257	0.458	0.006	0.297	824.026	-0.652	0.529	0.218	0.444
RABGEF1	666.744	0.106	0.122	0.386	0.525	666.744	0.050	0.121	0.683	0.828	666.744	-0.056	0.141	0.690	0.837
POLL	645.859	-0.125	0.235	0.595	0.710	645.859	0.443	0.234	0.058	0.348	645.859	0.568	0.271	0.036	0.148
KCNMB4	66.126	1.963	0.281	0.000	0.000	66.126	0.640	0.302	0.034	0.322	66.126	-1.323	0.322	0.000	0.001
TMEM97	968.403	0.888	0.117	0.000	0.000	968.403	0.158	0.118	0.182	0.469	968.403	-0.730	0.136	0.000	0.000
STK39	409.671	-0.728	0.247	0.003	0.014	409.671	-0.524	0.246	0.033	0.322	409.671	0.204	0.286	0.476	0.689
TOR1B	522.625	-0.033	0.115	0.777	0.852	522.625	-0.054	0.115	0.637	0.802	522.625	-0.021	0.134	0.873	0.939
MCAT	413.647	-0.370	0.219	0.091	0.182	413.647	-0.311	0.218	0.155	0.447	413.647	0.060	0.253	0.814	0.907
GLI1	233.551	-0.608	0.221	0.006	0.023	233.551	0.043	0.217	0.843	0.918	233.551	0.651	0.254	0.010	0.068
APOBEC3C	568.006	0.550	0.282	0.051	0.118	568.006	0.444	0.282	0.116	0.408	568.006	-0.106	0.326	0.745	0.869
DESI1	1129.152	-0.070	0.252	0.781	0.855	1129.152	-0.418	0.252	0.098	0.392	1129.152	-0.348	0.291	0.233	0.462
SGSM3	572.422	0.134	0.157	0.395	0.533	572.422	0.040	0.157	0.801	0.896	572.422	-0.094	0.182	0.604	0.781
GLI3	271.887	-0.137	0.227	0.546	0.670	271.887	0.081	0.225	0.718	0.849	271.887	0.218	0.262	0.404	0.631
GLI4	98.062	0.946	0.288	0.001	0.006	98.062	0.214	0.292	0.464	0.689	98.062	-0.732	0.334	0.028	0.126

GLO1	2856.350	0.184	0.184	0.318	0.458	2856.350	-0.347	0.184	0.059	0.350	2856.350	-0.531	0.213	0.013	0.076
BIN1	829.550	0.232	0.118	0.050	0.116	829.550	0.177	0.118	0.134	0.426	829.550	-0.055	0.137	0.687	0.836
HTRA2	1036.325	0.013	0.159	0.933	0.958	1036.325	-0.012	0.159	0.938	0.968	1036.325	-0.026	0.184	0.889	0.947
GLRB	62.913	-0.459	0.384	0.232	0.364	62.913	0.582	0.372	0.117	0.409	62.913	1.041	0.438	0.017	0.094
MAT2B	1509.390	-0.402	0.153	0.008	0.030	1509.390	-0.019	0.152	0.901	0.950	1509.390	0.384	0.176	0.029	0.130
TOR2A	77.495	0.480	0.294	0.102	0.199	77.495	-0.113	0.298	0.706	0.842	77.495	-0.592	0.343	0.084	0.251
POLM	156.647	0.267	0.213	0.210	0.339	156.647	0.558	0.210	0.008	0.297	156.647	0.291	0.244	0.233	0.462
EML4	655.651	-0.659	0.247	0.008	0.028	655.651	-0.514	0.247	0.037	0.326	655.651	0.145	0.286	0.613	0.786
GLS	1711.379	-0.503	0.230	0.029	0.076	1711.379	-0.272	0.230	0.236	0.513	1711.379	0.231	0.265	0.385	0.614
HDHD5	1521.094	-0.246	0.128	0.055	0.124	1521.094	0.119	0.127	0.348	0.604	1521.094	0.365	0.148	0.013	0.079
CECR2	22.887	-0.181	0.337	0.592	0.707	22.887	0.233	0.316	0.461	0.687	22.887	0.413	0.382	0.279	0.514
GLRX	2366.771	1.152	0.411	0.005	0.020	2366.771	0.847	0.411	0.039	0.327	2366.771	-0.305	0.474	0.520	0.723
GLUD1	3205.265	-0.164	0.088	0.062	0.136	3205.265	-0.042	0.087	0.635	0.801	3205.265	0.122	0.101	0.228	0.456
GLUD2	30.123	-0.744	0.392	0.057	0.128	30.123	-0.058	0.369	0.874	0.936	30.123	0.686	0.448	0.126	0.321
GLUD1P3	8.186	-0.359	0.591	0.544	0.668	8.186	-0.506	0.589	0.390	0.635	8.186	-0.148	0.700	0.833	0.919
GLUL	13566.077	0.150	0.508	0.768	0.847	13566.077	0.614	0.508	0.227	0.506	13566.077	0.464	0.587	0.429	0.652
GM2A	983.493	1.183	0.378	0.002	0.009	983.493	0.588	0.378	0.120	0.412	983.493	-0.595	0.437	0.173	0.390
GMDS	523.333	0.579	0.124	0.000	0.000	523.333	0.117	0.125	0.351	0.605	523.333	-0.463	0.144	0.001	0.016
GMFB	1091.070	-0.807	0.209	0.000	0.001	1091.070	-0.417	0.209	0.046	0.335	1091.070	0.391	0.242	0.106	0.291
GMPR	166.287	1.128	0.486	0.020	0.058	166.287	0.476	0.487	0.328	0.588	166.287	-0.653	0.561	0.244	0.475
GNA11	1195.135	-0.036	0.182	0.842	0.896	1195.135	-0.278	0.182	0.127	0.419	1195.135	-0.242	0.211	0.251	0.483
GNA12	1628.455	-0.239	0.214	0.263	0.398	1628.455	-0.133	0.214	0.534	0.733	1628.455	0.107	0.247	0.666	0.822
GNA15	1434.067	0.274	0.192	0.154	0.270	1434.067	-0.080	0.192	0.677	0.825	1434.067	-0.353	0.222	0.111	0.298
GNAI1	683.542	-0.487	0.233	0.037	0.091	683.542	0.091	0.231	0.696	0.836	683.542	0.577	0.268	0.032	0.136
GNAI2	4578.075	0.387	0.143	0.007	0.025	4578.075	0.155	0.143	0.277	0.548	4578.075	-0.232	0.165	0.158	0.370
GNAI3	2651.487	-0.529	0.170	0.002	0.009	2651.487	-0.171	0.169	0.311	0.575	2651.487	0.358	0.196	0.068	0.221
GNAL	63.694	0.718	0.285	0.012	0.038	63.694	-0.143	0.294	0.627	0.795	63.694	-0.861	0.334	0.010	0.066
GNAO1	8.833	-0.180	0.817	0.826	0.885	8.833	0.023	0.806	0.977	0.987	8.833	0.203	0.943	0.830	0.916
GNAQ	1818.926	-0.160	0.167	0.338	0.478	1818.926	-0.004	0.167	0.981	0.989	1818.926	0.156	0.193	0.418	0.643
GNAS	14977.226	0.860	0.208	0.000	0.000	14977.226	0.174	0.208	0.402	0.644	14977.226	-0.686	0.240	0.004	0.037
AMY1C	9.737	2.513	1.119	0.025	0.068	9.737	2.058	1.124	0.067	0.355	9.737	-0.455	1.258	0.717	0.853
GNAT2	14.570	-0.873	0.534	0.102	0.198	14.570	-0.183	0.498	0.714	0.847	14.570	0.691	0.612	0.259	0.493
GNAZ	8.788	0.593	0.498	0.234	0.367	8.788	0.450	0.497	0.365	0.617	8.788	-0.143	0.568	0.801	0.900
GNB1	10390.163	-0.650	0.164	0.000	0.001	10390.163	-0.080	0.164	0.625	0.794	10390.163	0.570	0.189	0.003	0.026
GNB2	3797.602	0.272	0.138	0.049	0.113	3797.602	0.192	0.138	0.163	0.454	3797.602	-0.080	0.159	0.617	0.790
GNB3	29.373	1.022	0.464	0.028	0.073	29.373	0.547	0.467	0.242	0.518	29.373	-0.474	0.533	0.373	0.605
GNG3	14.480	0.032	0.692	0.963	0.978	14.480	0.860	0.668	0.198	0.481	14.480	0.828	0.784	0.291	0.526
GNG4	19.937	1.019	0.390	0.009	0.032	19.937	-0.298	0.426	0.483	0.701	19.937	-1.317	0.472	0.005	0.042
GNG5	1039.366	0.632	0.219	0.004	0.016	1039.366	0.010	0.220	0.962	0.980	1039.366	-0.622	0.253	0.014	0.082
GNG7	54.358	0.073	1.089	0.946	0.967	54.358	0.825	1.086	0.448	0.677	54.358	0.751	1.256	0.550	0.744
GNG10	359.311	-0.362	0.189	0.055	0.124	359.311	-0.260	0.188	0.165	0.455	359.311	0.101	0.218	0.643	0.806
GNG11	421.353	-0.243	0.254	0.340	0.480	421.353	-0.287	0.254	0.259	0.531	421.353	-0.044	0.294	0.881	0.943
GNGT1	98.509	0.489	0.214	0.022	0.063	98.509	-0.209	0.220	0.343	0.600	98.509	-0.697	0.252	0.006	0.044
GNL1	6501.637	-0.008	0.207	0.968	0.980	6501.637	0.260	0.207	0.209	0.490	6501.637	0.269	0.239	0.262	0.496
GNS	2377.441	0.346	0.222	0.118	0.222	2377.441	0.134	0.222	0.545	0.740	2377.441	-0.212	0.256	0.408	0.634
ABO	21.502	0.457	0.590	0.438	0.574	21.502	1.700	0.568	0.003	0.297	21.502	1.243	0.661	0.060	0.205
AMY2B	44.489	1.610	0.617	0.009	0.032	44.489	1.131	0.619	0.068	0.356	44.489	-0.479	0.707	0.498	0.706
GOLGA1	308.417	-0.233	0.235	0.320	0.460	308.417	-0.393	0.235	0.094	0.388	308.417	-0.160	0.272	0.557	0.749
GOLGA2	1542.962	0.356	0.188	0.059	0.130	1542.962	0.120	0.188	0.526	0.728	1542.962	-0.236	0.217	0.277	0.512
GOLGA3	867.179	-0.259	0.227	0.254	0.388	867.179	-0.375	0.227	0.099	0.394	867.179	-0.116	0.263	0.660	0.818
GOLGA4	2586.588	-0.196	0.195	0.316	0.456	2586.588	-0.227	0.195	0.245	0.520	2586.588	-0.031	0.225	0.891	0.948
GOLGB1	1993.924	-0.088	0.280	0.752	0.835	1993.924	0.179	0.280	0.522	0.726	1993.924	0.267	0.323	0.408	0.635
GOT1	759.059	-0.358	0.329	0.277	0.414	759.059	-0.760	0.329	0.021	0.313	759.059	-0.402	0.381	0.291	0.526
GOT2	2328.120	-0.326	0.239	0.174	0.295	2328.120	-0.494	0.239	0.039	0.327	2328.120	-0.169	0.276	0.542	0.739
SELENOH	1088.102	0.702	0.172	0.000	0.000	1088.102	0.159	0.172	0.356	0.610	1088.102	-0.543	0.199	0.006	0.048
SFN	3220.585	1.227	0.257	0.000	0.000	3220.585	-0.265	0.257	0.304	0.570	3220.585	-1.491	0.297	0.000	0.000
GPC1	4952.788	0.770	0.211	0.000	0.002	4952.788	-0.201	0.211	0.339	0.597	4952.788	-0.971	0.243	0.000	0.002
GPD2	10.822	0.267	0.481	0.578	0.696	10.822	0.164	0.477	0.731	0.856	10.822	-0.103	0.555	0.853	0.930
GPI	22826.244	-0.368	0.460	0.424	0.561	22826.244	0.703	0.460	0.126	0.419	22826.244	1.071	0.531	0.044	0.168
GPLD1	61.193	0.228	0.275	0.407	0.545	61.193	0.234	0.272	0.390	0.635	61.193	0.006	0.316	0.984	0.991
PPP2R3B	541.283	0.285	0.206	0.166	0.285	541.283	0.222	0.206	0.279	0.549	541.283	-0.063	0.238	0.791	0.896
GPM6A	115.239	-0.365	0.596	0.540	0.665	115.239	0.799	0.592	0.177	0.466	115.239	1.164	0.687	0.090	0.263
SLCO4A1	6158.899	1.278	0.141	0.000	0.000	6158.899	0.337	0.141	0.017	0.306	6158.899	-0.940	0.163	0.000	0.000
SLCO3A1	255.900	0.683	0.168	0.000	0.001	255.900	-0.151	0.171	0.379	0.626	255.900	-0.834	0.196	0.000	0.001
SLCO1B3	21.274	0.494	0.465	0.288	0.426	21.274	0.501	0.461	0.277	0.547	21.274	0.007	0.531	0.990	0.994
GPM6B	738.713	-0.159	0.267	0.552	0.674	738.713	0.115	0.267	0.666	0.819	738.713	0.274	0.308	0.374	0.606
GPR1	64.807	-0.370	0.224	0.099	0.194	64.807	-0.203	0.219	0.352	0.606	64.807	0.167	0.260	0.521	0.724

CCR10	159.782	-0.011	0.267	0.966	0.979	159.782	0.643	0.262	0.014	0.306	159.782	0.654	0.306	0.033	0.139
AQP11	14.556	0.967	0.395	0.014	0.045	14.556	-0.717	0.463	0.121	0.413	14.556	-1.683	0.505	0.001	0.012
GPR3	26.534	-0.399	0.702	0.570	0.689	26.534	-1.813	0.732	0.013	0.305	26.534	-1.414	0.842	0.093	0.268
OR51B5	421.408	-0.746	0.222	0.001	0.005	421.408	0.300	0.218	0.170	0.459	421.408	1.046	0.255	0.000	0.001
POC1B	269.757	-0.342	0.140	0.015	0.046	269.757	-0.036	0.137	0.793	0.892	269.757	0.306	0.162	0.059	0.202
ZNF311	186.620	-0.008	0.266	0.976	0.985	186.620	0.330	0.263	0.210	0.491	186.620	0.338	0.306	0.269	0.504
FUOM	153.106	-0.035	0.340	0.918	0.948	153.106	-0.704	0.342	0.040	0.327	153.106	-0.669	0.395	0.091	0.263
STK32C	511.789	-0.111	0.183	0.545	0.669	511.789	-0.062	0.183	0.734	0.858	511.789	0.049	0.212	0.817	0.909
BLOC1S2	430.446	-0.361	0.255	0.157	0.273	430.446	-0.518	0.255	0.042	0.330	430.446	-0.157	0.295	0.595	0.774
ANG	130.433	1.494	0.853	0.080	0.165	130.433	1.164	0.853	0.172	0.461	130.433	-0.330	0.983	0.737	0.865
LOC283038	12.158	0.151	0.664	0.820	0.882	12.158	0.908	0.638	0.155	0.447	12.158	0.757	0.748	0.312	0.547
ZMIZ1-AS1	36.974	2.065	0.644	0.001	0.007	36.974	1.002	0.652	0.124	0.416	36.974	-1.063	0.739	0.150	0.359
MKX	18.821	-2.611	0.618	0.000	0.000	18.821	-0.754	0.519	0.147	0.438	18.821	1.858	0.696	0.008	0.054
SBF2-AS1	175.647	-0.406	0.253	0.109	0.209	175.647	0.257	0.249	0.301	0.568	175.647	0.663	0.291	0.023	0.110
CSNK2A3	238.730	-1.362	0.299	0.000	0.000	238.730	-0.232	0.293	0.428	0.663	238.730	1.129	0.344	0.001	0.014
H19	2991.964	0.282	1.122	0.801	0.869	2991.964	0.994	1.122	0.376	0.623	2991.964	0.712	1.295	0.583	0.765
SLC25A45	29.013	0.730	0.423	0.084	0.172	29.013	0.269	0.430	0.532	0.732	29.013	-0.461	0.489	0.346	0.580
NEAT1	4749.792	1.600	0.346	0.000	0.000	4749.792	0.347	0.346	0.316	0.578	4749.792	-1.253	0.399	0.002	0.019
BCL9L	4814.309	-0.265	0.288	0.357	0.497	4814.309	0.376	0.288	0.191	0.477	4814.309	0.641	0.332	0.054	0.191
P4HA3	40.661	1.337	0.374	0.000	0.003	40.661	-0.465	0.405	0.251	0.525	40.661	-1.802	0.451	0.000	0.002
PGM2L1	207.707	0.512	0.299	0.087	0.177	207.707	-0.488	0.304	0.108	0.400	207.707	-1.000	0.349	0.004	0.036
KCTD21	235.028	0.339	0.176	0.054	0.123	235.028	0.348	0.175	0.047	0.335	235.028	0.009	0.203	0.963	0.983
CRACR2B	51.035	0.299	0.295	0.311	0.450	51.035	0.268	0.293	0.360	0.612	51.035	-0.031	0.339	0.927	0.967
TMEM80	148.967	0.456	0.294	0.121	0.226	148.967	0.246	0.295	0.403	0.645	148.967	-0.210	0.340	0.537	0.735
CCDC88B	139.062	1.213	0.352	0.001	0.004	139.062	0.651	0.353	0.065	0.354	139.062	-0.561	0.405	0.166	0.381
TTC9C	463.322	0.222	0.177	0.208	0.337	463.322	0.272	0.176	0.123	0.414	463.322	0.049	0.204	0.809	0.904
HARBI1	142.491	-0.922	0.312	0.003	0.014	142.491	-0.564	0.308	0.067	0.354	142.491	0.358	0.361	0.321	0.556
LINC00294	144.593	1.177	0.393	0.003	0.012	144.593	0.620	0.394	0.115	0.408	144.593	-0.556	0.453	0.219	0.446
C1RL-AS1	148.865	0.305	0.376	0.418	0.555	148.865	0.775	0.374	0.038	0.327	148.865	0.471	0.432	0.276	0.511
CD163L1	781.341	-0.027	0.203	0.893	0.931	781.341	0.070	0.203	0.729	0.855	781.341	0.097	0.235	0.678	0.829
LOC283335	3684.886	-0.962	0.384	0.012	0.039	3684.886	0.441	0.383	0.250	0.523	3684.886	1.404	0.443	0.002	0.018
ZNF740	765.899	-0.537	0.186	0.004	0.016	765.899	-0.116	0.185	0.531	0.731	765.899	0.422	0.215	0.049	0.181
RPL13P5	9.479	0.588	0.506	0.245	0.378	9.479	0.076	0.521	0.884	0.942	9.479	-0.512	0.591	0.386	0.615
RASSF3	696.822	0.097	0.117	0.407	0.545	696.822	-0.204	0.117	0.082	0.373	696.822	-0.301	0.136	0.026	0.121
ANKRD52	1259.725	-0.432	0.304	0.156	0.272	1259.725	-0.648	0.304	0.033	0.322	1259.725	-0.216	0.352	0.539	0.736
SPRYD4	159.795	0.089	0.199	0.654	0.758	159.795	-0.502	0.203	0.013	0.305	159.795	-0.591	0.234	0.012	0.072
TRHDE-AS1	105.354	-0.567	0.293	0.053	0.121	105.354	-0.091	0.288	0.752	0.867	105.354	0.476	0.338	0.160	0.372
GAS2L3	432.037	-0.318	0.351	0.366	0.506	432.037	0.319	0.350	0.362	0.614	432.037	0.637	0.405	0.116	0.305
ITFG2-AS1	55.391	-1.122	0.375	0.003	0.012	55.391	-0.411	0.360	0.254	0.527	55.391	0.712	0.432	0.099	0.279
HECTD4	471.025	-0.425	0.279	0.128	0.234	471.025	0.044	0.278	0.873	0.936	471.025	0.469	0.322	0.145	0.351
GATC	2241.820	0.231	0.154	0.134	0.244	2241.820	-0.035	0.154	0.823	0.908	2241.820	-0.266	0.178	0.136	0.338
C12orf40	11.718	0.747	0.863	0.387	0.526	11.718	0.408	0.866	0.638	0.803	11.718	-0.340	0.995	0.733	0.862
GXYLT1	331.544	-0.677	0.281	0.016	0.049	331.544	-0.479	0.280	0.088	0.380	331.544	0.198	0.326	0.543	0.739
LINC00346	9.894	0.526	0.551	0.340	0.480	9.894	0.198	0.557	0.722	0.851	9.894	-0.328	0.638	0.607	0.783
CHAMP1	362.034	-0.801	0.226	0.000	0.003	362.034	-0.437	0.224	0.051	0.338	362.034	0.365	0.261	0.163	0.377
SLC46A3	131.507	0.863	0.179	0.000	0.000	131.507	-0.236	0.188	0.210	0.491	131.507	-1.099	0.212	0.000	0.000
LINC01588	38.987	0.511	0.590	0.386	0.525	38.987	0.050	0.597	0.934	0.967	38.987	-0.461	0.685	0.501	0.708
GPR137C	10.250	0.258	0.663	0.697	0.791	10.250	-0.609	0.688	0.376	0.624	10.250	-0.867	0.789	0.272	0.507
TMED8	476.383	-0.856	0.254	0.001	0.005	476.383	-0.436	0.252	0.084	0.376	476.383	0.421	0.293	0.152	0.361
C14orf178	11.244	-1.784	0.632	0.005	0.019	11.244	-0.616	0.547	0.261	0.533	11.244	1.169	0.720	0.104	0.288
SNHG10	84.325	-0.584	0.430	0.175	0.296	84.325	-0.717	0.430	0.095	0.390	84.325	-0.133	0.500	0.791	0.896
LINC00641	65.236	-0.424	0.309	0.170	0.291	65.236	-0.141	0.303	0.641	0.804	65.236	0.283	0.357	0.429	0.651
TSSK4	6.842	-0.465	0.733	0.526	0.652	6.842	0.735	0.661	0.266	0.538	6.842	1.199	0.814	0.141	0.345
FAM177A1	579.422	0.167	0.225	0.458	0.593	579.422	-0.436	0.226	0.054	0.342	579.422	-0.603	0.261	0.021	0.104
CEP170B	1539.513	-0.158	0.248	0.523	0.651	1539.513	-0.400	0.248	0.107	0.399	1539.513	-0.242	0.287	0.400	0.627
TEDC1	273.446	-0.337	0.469	0.471	0.606	273.446	-0.988	0.470	0.035	0.324	273.446	-0.651	0.543	0.231	0.459
HMGN2P46	12.323	0.994	0.537	0.064	0.140	12.323	-0.798	0.631	0.206	0.488	12.323	-1.792	0.689	0.009	0.062
PRTG	63.957	-0.655	0.272	0.016	0.049	63.957	-0.335	0.264	0.205	0.487	63.957	0.319	0.315	0.311	0.546
ST20-AS1	92.558	-0.237	0.256	0.354	0.494	92.558	-0.085	0.251	0.735	0.858	92.558	0.152	0.296	0.607	0.783
FAM98B	927.407	-0.553	0.212	0.009	0.032	927.407	-0.545	0.212	0.010	0.299	927.407	0.008	0.245	0.973	0.987
HERC2P3	98.257	0.060	0.422	0.888	0.927	98.257	0.288	0.420	0.493	0.708	98.257	0.228	0.486	0.639	0.804
LOC283788	111.866	0.589	0.260	0.024	0.065	111.866	0.258	0.261	0.324	0.584	111.866	-0.331	0.300	0.271	0.506
NOMO2	6754.606	0.207	0.160	0.196	0.322	6754.606	-0.119	0.160	0.457	0.683	6754.606	-0.326	0.185	0.078	0.241
EXOC3L1	20.812	0.525	0.513	0.306	0.445	20.812	0.594	0.508	0.242	0.518	20.812	0.069	0.586	0.906	0.957
BRICD5	83.411	0.986	0.324	0.002	0.011	83.411	0.128	0.330	0.698	0.838	83.411	-0.858	0.376	0.023	0.109
PGP	513.977	0.149	0.492	0.762	0.842	513.977	-1.411	0.494	0.004	0.297	513.977	-1.560	0.570	0.006	0.048
LINC00921	22.803	-0.084	0.372	0.822	0.884	22.803	0.043	0.358	0.905	0.952	22.803	0.126	0.428	0.768	0.882

IL21R-AS1	11.297	1.757	0.544	0.001	0.007	11.297	-0.603	0.627	0.336	0.594	11.297	-2.360	0.679	0.001	0.008
INO80E	937.275	0.422	0.215	0.049	0.114	937.275	-0.060	0.215	0.779	0.883	937.275	-0.483	0.248	0.052	0.187
LOC283922	47.005	-0.640	0.411	0.120	0.224	47.005	-0.010	0.399	0.980	0.989	47.005	0.630	0.473	0.183	0.402
NUDT7	76.728	1.025	0.369	0.005	0.021	76.728	0.434	0.371	0.243	0.519	76.728	-0.592	0.425	0.164	0.379
FBXL19-AS1	60.641	-0.752	0.345	0.029	0.077	60.641	-0.027	0.334	0.934	0.967	60.641	0.725	0.396	0.067	0.220
C16orf91	198.463	-0.321	0.303	0.289	0.427	198.463	-0.501	0.303	0.098	0.392	198.463	-0.180	0.351	0.608	0.783
PDXDC2P-NPIPB14P	79.524	0.714	0.486	0.142	0.254	79.524	0.604	0.486	0.214	0.493	79.524	-0.110	0.560	0.844	0.924
HID1	20.537	0.372	0.452	0.411	0.549	20.537	0.150	0.454	0.741	0.862	20.537	-0.222	0.523	0.671	0.825
TSEN54	357.236	0.156	0.223	0.483	0.616	357.236	-0.235	0.224	0.294	0.561	357.236	-0.391	0.258	0.130	0.327
UBALD2	2076.970	0.066	0.221	0.765	0.844	2076.970	0.175	0.221	0.428	0.663	2076.970	0.109	0.256	0.669	0.824
ANGPT1	38.915	0.106	0.475	0.824	0.885	38.915	0.354	0.470	0.451	0.679	38.915	0.249	0.546	0.648	0.811
CCDC57	335.262	0.469	0.194	0.016	0.048	335.262	0.231	0.194	0.235	0.513	335.262	-0.238	0.224	0.288	0.523
HEXD	262.326	0.469	0.263	0.074	0.157	262.326	0.641	0.262	0.014	0.306	262.326	0.172	0.303	0.570	0.759
C17orf58	193.383	0.372	0.214	0.082	0.168	193.383	0.176	0.214	0.409	0.649	193.383	-0.195	0.247	0.428	0.651
MILR1	28.440	-0.364	0.415	0.381	0.521	28.440	0.731	0.381	0.055	0.343	28.440	1.095	0.464	0.018	0.096
RNF227	92.526	0.880	0.386	0.023	0.064	92.526	0.687	0.386	0.075	0.364	92.526	-0.193	0.444	0.664	0.821
LINC00324	9.166	0.461	0.992	0.642	0.749	9.166	0.296	0.992	0.766	0.876	9.166	-0.165	1.144	0.885	0.945
CCDC144B	25.911	0.009	0.615	0.988	0.993	25.911	0.128	0.611	0.834	0.915	25.911	0.119	0.710	0.867	0.936
KANSL1	1190.580	-0.394	0.150	0.008	0.030	1190.580	0.103	0.149	0.488	0.705	1190.580	0.497	0.173	0.004	0.035
FAM171A2	89.503	2.342	0.341	0.000	0.000	89.503	0.522	0.352	0.138	0.429	89.503	-1.820	0.395	0.000	0.000
MEIOC	117.662	-0.679	0.213	0.001	0.008	117.662	-0.186	0.206	0.367	0.618	117.662	0.493	0.246	0.045	0.171
PICART1	19.700	0.665	0.563	0.237	0.370	19.700	0.896	0.556	0.107	0.399	19.700	0.231	0.640	0.718	0.854
NEK8	86.262	0.251	0.267	0.348	0.489	86.262	-0.052	0.268	0.848	0.921	86.262	-0.302	0.310	0.330	0.564
PIGW	502.889	-1.214	0.396	0.002	0.010	502.889	-1.025	0.395	0.009	0.297	502.889	0.189	0.458	0.679	0.830
CISD3	2650.716	0.314	0.237	0.185	0.308	2650.716	-0.125	0.237	0.596	0.775	2650.716	-0.439	0.273	0.108	0.293
TMEM102	148.793	1.008	0.321	0.002	0.009	148.793	0.431	0.323	0.182	0.469	148.793	-0.577	0.370	0.119	0.309
CAVIN1	8871.702	0.242	0.100	0.015	0.047	8871.702	-0.034	0.100	0.736	0.859	8871.702	-0.275	0.115	0.017	0.092
SLC26A11	215.124	0.805	0.168	0.000	0.000	215.124	0.286	0.170	0.092	0.387	215.124	-0.519	0.194	0.007	0.054
ENDOV	133.004	0.709	0.366	0.053	0.121	133.004	0.372	0.367	0.312	0.575	133.004	-0.338	0.423	0.424	0.648
GDPD1	85.896	-1.161	0.327	0.000	0.003	85.896	-0.174	0.314	0.579	0.763	85.896	0.986	0.375	0.009	0.059
NDUFAF8	270.611	0.408	0.334	0.222	0.353	270.611	-0.480	0.337	0.154	0.446	270.611	-0.889	0.388	0.022	0.108
LINC00482	9.263	0.436	1.015	0.667	0.768	9.263	1.172	0.991	0.237	0.514	9.263	0.736	1.150	0.522	0.724
METRNL	1093.066	1.239	0.226	0.000	0.000	1093.066	0.053	0.227	0.815	0.903	1093.066	-1.186	0.262	0.000	0.000
LAMA1	769.170	0.485	0.304	0.111	0.211	769.170	0.221	0.304	0.467	0.690	769.170	-0.264	0.351	0.452	0.671
KCTD1	412.435	-0.757	0.142	0.000	0.000	412.435	0.106	0.137	0.443	0.674	412.435	0.862	0.163	0.000	0.000
ZADH2	803.000	-0.455	0.147	0.002	0.010	803.000	-0.083	0.146	0.571	0.757	803.000	0.372	0.170	0.029	0.129
ZNF547	27.274	0.256	0.293	0.384	0.523	27.274	-0.079	0.299	0.792	0.892	27.274	-0.334	0.345	0.333	0.568
ZIK1	6.819	-1.535	0.813	0.059	0.131	6.819	-0.523	0.725	0.471	0.693	6.819	1.012	0.929	0.276	0.511
ZNF776	104.939	-0.136	0.201	0.499	0.630	104.939	-0.286	0.200	0.154	0.446	104.939	-0.150	0.235	0.522	0.724
ZNF780A	127.961	-0.331	0.232	0.154	0.270	127.961	-0.019	0.228	0.935	0.967	127.961	0.313	0.268	0.243	0.474
C19orf54	623.350	-0.061	0.133	0.645	0.751	623.350	0.177	0.132	0.180	0.468	623.350	0.238	0.153	0.120	0.311
PRR19	186.597	0.650	0.190	0.001	0.004	186.597	0.022	0.193	0.907	0.954	186.597	-0.627	0.221	0.004	0.038
TMEM145	66.245	1.039	0.511	0.042	0.101	66.245	1.028	0.510	0.044	0.331	66.245	-0.011	0.585	0.985	0.992
ZNF575	586.612	0.177	0.277	0.525	0.651	586.612	0.594	0.277	0.032	0.322	586.612	0.418	0.320	0.191	0.413
ZNF283	110.623	-0.378	0.197	0.055	0.124	110.623	0.021	0.190	0.914	0.957	110.623	0.399	0.227	0.078	0.242
PPP1R37	583.159	0.339	0.154	0.028	0.074	583.159	0.003	0.154	0.984	0.991	583.159	-0.335	0.178	0.060	0.204
MAMSTR	17.355	0.300	0.449	0.504	0.633	17.355	0.126	0.449	0.779	0.883	17.355	-0.174	0.519	0.737	0.865
EMC10	1342.741	0.527	0.140	0.000	0.001	1342.741	0.105	0.140	0.456	0.683	1342.741	-0.423	0.162	0.009	0.061
ZNF615	137.519	-0.424	0.281	0.131	0.239	137.519	0.272	0.275	0.323	0.584	137.519	0.696	0.322	0.031	0.134
ZNF841	114.124	0.017	0.272	0.950	0.970	114.124	0.074	0.270	0.783	0.886	114.124	0.057	0.314	0.855	0.930
ZNF561-AS1	69.901	0.383	0.234	0.101	0.198	69.901	0.042	0.236	0.858	0.928	69.901	-0.340	0.272	0.210	0.436
WDR62	243.284	-0.390	0.434	0.369	0.509	243.284	-1.181	0.436	0.007	0.297	243.284	-0.790	0.504	0.117	0.306
ZNF790-AS1	22.806	-0.226	0.444	0.610	0.723	22.806	-0.547	0.449	0.223	0.501	22.806	-0.321	0.524	0.541	0.738
LOC284412	16.873	-0.182	0.514	0.723	0.812	16.873	0.468	0.487	0.337	0.595	16.873	0.650	0.581	0.264	0.498
NWD1	31.653	-2.320	1.523	0.128	0.235	31.653	0.442	1.497	0.768	0.877	31.653	2.761	1.751	0.115	0.304
SLC25A42	53.445	0.322	0.579	0.578	0.696	53.445	0.939	0.573	0.101	0.394	53.445	0.617	0.664	0.352	0.586
LINC00663	9.419	-0.161	1.027	0.876	0.919	9.419	1.129	0.993	0.255	0.529	9.419	1.290	1.165	0.268	0.503
ZNF493	11.684	1.005	0.581	0.084	0.171	11.684	0.634	0.587	0.280	0.550	11.684	-0.371	0.663	0.576	0.761
LOC284454	261.081	0.919	0.201	0.000	0.000	261.081	0.116	0.204	0.570	0.757	261.081	-0.803	0.234	0.001	0.009
ZNF875	154.726	-0.231	0.201	0.250	0.384	154.726	-0.213	0.199	0.286	0.555	154.726	0.019	0.233	0.937	0.972
C1orf167	57.379	0.776	0.293	0.008	0.029	57.379	0.351	0.296	0.236	0.513	57.379	-0.425	0.338	0.208	0.433
NBPF15	359.679	0.170	0.183	0.354	0.494	359.679	0.093	0.183	0.612	0.786	359.679	-0.077	0.212	0.716	0.853
LOC284581	60.735	0.428	0.298	0.150	0.265	60.735	-0.323	0.306	0.291	0.559	60.735	-0.751	0.351	0.032	0.138
FAM102B	171.051	0.998	0.394	0.011	0.037	171.051	0.643	0.394	0.103	0.396	171.051	-0.355	0.454	0.435	0.656
CYB561D1	159.749	-0.269	0.199	0.176	0.297	159.749	-0.030	0.196	0.879	0.939	159.749	0.240	0.230	0.297	0.532
ANKRD34A	73.918	0.185	0.263	0.480	0.614	73.918	0.148	0.261	0.570	0.757	73.918	-0.037	0.303	0.902	0.954
RUSC1-AS1	2224.610	1.569	0.200	0.000	0.000	2224.610	-0.231	0.201	0.249	0.523	2224.610	-1.800	0.231	0.000	0.000

SMG7-AS1	9.058	-2.022	0.656	0.002	0.010	9.058	-0.151	0.501	0.763	0.874	9.058	1.871	0.723	0.010	0.064
SNHG28	92.344	2.795	0.346	0.000	0.000	92.344	0.744	0.387	0.055	0.342	92.344	-2.051	0.396	0.000	0.000
ZNF326	472.196	-0.313	0.218	0.150	0.264	472.196	-0.511	0.218	0.019	0.306	472.196	-0.198	0.252	0.433	0.655
LINC01270	23.277	0.277	0.398	0.485	0.618	23.277	0.397	0.390	0.308	0.573	23.277	0.120	0.454	0.792	0.896
C20orf197	47.206	-0.709	0.333	0.033	0.084	47.206	-0.074	0.319	0.817	0.904	47.206	0.635	0.382	0.096	0.273
FRG1BP	136.363	0.010	0.198	0.960	0.976	136.363	0.264	0.195	0.175	0.464	136.363	0.254	0.227	0.264	0.498
LINC00319	9.954	0.562	0.824	0.495	0.627	9.954	0.837	0.812	0.303	0.569	9.954	0.275	0.937	0.769	0.882
AATBC	11.948	0.310	0.900	0.730	0.817	11.948	0.586	0.892	0.512	0.719	11.948	0.275	1.033	0.790	0.895
MIF-AS1	28910.174	0.922	0.282	0.001	0.006	28910.174	0.498	0.282	0.077	0.368	28910.174	-0.423	0.325	0.193	0.415
TTC28-AS1	228.360	-0.320	0.194	0.099	0.194	228.360	0.222	0.190	0.243	0.519	228.360	0.542	0.223	0.015	0.085
LOC284930	94.491	0.908	0.364	0.012	0.040	94.491	0.290	0.367	0.430	0.664	94.491	-0.618	0.420	0.141	0.346
RPL23AP82	123.027	-0.092	0.249	0.711	0.802	123.027	0.356	0.243	0.142	0.433	123.027	0.449	0.285	0.115	0.304
NT5DC4	130.345	-1.186	0.299	0.000	0.001	130.345	-0.110	0.289	0.705	0.841	130.345	1.076	0.343	0.002	0.019
CCDC150	25.639	0.300	0.330	0.363	0.504	25.639	0.274	0.326	0.401	0.644	25.639	-0.026	0.379	0.945	0.975
RNF149	968.428	0.302	0.108	0.005	0.020	968.428	0.113	0.108	0.296	0.564	968.428	-0.189	0.125	0.129	0.325
STARD7-AS1	462.609	-0.963	0.346	0.005	0.021	462.609	-0.322	0.344	0.350	0.605	462.609	0.642	0.399	0.108	0.293
LOC285074	497.985	-0.844	0.134	0.000	0.000	497.985	0.166	0.128	0.194	0.478	497.985	1.009	0.153	0.000	0.000
NKIRAS2	852.853	-0.043	0.087	0.625	0.735	852.853	-0.056	0.087	0.519	0.724	852.853	-0.013	0.101	0.896	0.951
AHCTF1P1	192.608	-1.343	0.323	0.000	0.000	192.608	-0.850	0.319	0.008	0.297	192.608	0.492	0.374	0.188	0.408
NKIRAS1	362.489	0.125	0.145	0.390	0.529	362.489	0.096	0.144	0.506	0.716	362.489	-0.029	0.168	0.863	0.935
CDH19	25.723	-0.984	0.374	0.009	0.030	25.723	0.444	0.326	0.174	0.463	25.723	1.427	0.416	0.001	0.009
DLL1	1077.759	0.560	0.990	0.571	0.690	1077.759	1.013	0.989	0.306	0.572	1077.759	0.453	1.143	0.692	0.838
ERICH2	122.807	0.277	0.298	0.352	0.493	122.807	0.498	0.295	0.091	0.385	122.807	0.222	0.341	0.516	0.720
IAH1	805.555	0.565	0.174	0.001	0.007	805.555	0.295	0.175	0.091	0.385	805.555	-0.270	0.201	0.180	0.398
FAM126B	242.198	0.278	0.188	0.139	0.249	242.198	-0.263	0.190	0.166	0.456	242.198	-0.540	0.219	0.013	0.079
RGPD4	37.240	-0.974	0.431	0.024	0.065	37.240	-0.540	0.417	0.195	0.479	37.240	0.434	0.499	0.385	0.614
DUSP28	46.728	0.176	0.270	0.515	0.643	46.728	0.086	0.268	0.747	0.866	46.728	-0.089	0.312	0.775	0.886
SLC9A9	256.461	-0.200	0.518	0.699	0.792	256.461	0.617	0.517	0.232	0.510	256.461	0.817	0.597	0.171	0.388
GPER1	16.050	-0.170	0.762	0.823	0.885	16.050	0.692	0.736	0.347	0.602	16.050	0.862	0.866	0.319	0.555
EOGT	334.845	-0.227	0.228	0.319	0.459	334.845	-0.075	0.227	0.740	0.861	334.845	0.152	0.264	0.564	0.755
C3orf38	146.577	-0.457	0.177	0.010	0.033	146.577	-0.408	0.174	0.019	0.306	146.577	0.050	0.206	0.810	0.905
ENTPD3-AS1	28.432	0.207	0.360	0.566	0.686	28.432	0.458	0.350	0.191	0.476	28.432	0.252	0.409	0.539	0.736
ZNF619	65.231	-0.581	0.280	0.038	0.094	65.231	-0.182	0.272	0.504	0.715	65.231	0.400	0.323	0.216	0.442
ZNF621	401.129	-0.486	0.149	0.001	0.006	401.129	-0.005	0.147	0.971	0.985	401.129	0.481	0.172	0.005	0.042
RABL3	546.578	-0.566	0.190	0.003	0.013	546.578	-0.168	0.189	0.374	0.622	546.578	0.398	0.219	0.070	0.225
C3orf33	33.114	0.094	0.345	0.785	0.858	33.114	0.142	0.341	0.676	0.825	33.114	0.048	0.398	0.904	0.956
CCDC66	223.311	-0.433	0.217	0.046	0.108	223.311	-0.416	0.216	0.054	0.342	223.311	0.017	0.252	0.946	0.975
TCAIM	889.380	0.001	0.337	0.998	0.999	889.380	0.348	0.337	0.301	0.568	889.380	0.347	0.389	0.372	0.604
ZNF852	31.352	0.005	0.333	0.989	0.994	31.352	0.027	0.328	0.934	0.967	31.352	0.023	0.385	0.953	0.978
KIF9-AS1	54.535	0.014	0.414	0.973	0.983	54.535	0.449	0.408	0.271	0.542	54.535	0.435	0.475	0.360	0.593
SUMF1	622.536	0.332	0.212	0.117	0.220	622.536	0.283	0.211	0.181	0.469	622.536	-0.049	0.244	0.842	0.923
RPUSD3	522.490	-0.194	0.200	0.331	0.471	522.490	-0.087	0.199	0.662	0.816	522.490	0.107	0.231	0.643	0.806
PRRT3	76.170	0.096	0.243	0.694	0.789	76.170	0.227	0.238	0.340	0.598	76.170	0.132	0.279	0.637	0.803
DPH3	349.420	-0.422	0.253	0.095	0.189	349.420	-0.520	0.253	0.040	0.327	349.420	-0.098	0.293	0.738	0.866
TPRG1	62.578	-0.378	0.343	0.271	0.408	62.578	0.091	0.335	0.786	0.888	62.578	0.469	0.395	0.235	0.465
ALG1L9P	69.230	-0.659	0.412	0.110	0.210	69.230	-1.090	0.415	0.009	0.297	69.230	-0.431	0.483	0.373	0.604
CYP4V2	218.782	0.445	0.145	0.002	0.010	218.782	0.025	0.147	0.867	0.932	218.782	-0.421	0.169	0.013	0.077
CTBP1-AS	1005.781	0.568	0.197	0.004	0.016	1005.781	-0.004	0.197	0.986	0.992	1005.781	-0.571	0.228	0.012	0.074
FAM198B-AS1	79.956	-0.146	0.323	0.650	0.755	79.956	-0.072	0.321	0.821	0.907	79.956	0.074	0.373	0.843	0.924
FAM13A-AS1	158.007	-0.110	0.472	0.816	0.879	158.007	0.531	0.468	0.257	0.530	158.007	0.641	0.543	0.238	0.468
COX18	246.106	-0.163	0.143	0.253	0.388	246.106	-0.158	0.142	0.265	0.537	246.106	0.005	0.166	0.975	0.987
YIPF7	18.908	-2.166	0.601	0.000	0.002	18.908	-0.372	0.523	0.476	0.697	18.908	1.793	0.678	0.008	0.057
FRYL	427.402	-0.699	0.230	0.002	0.011	427.402	-0.322	0.228	0.158	0.451	427.402	0.377	0.266	0.156	0.367
RNF175	25.862	2.192	0.451	0.000	0.000	25.862	-0.654	0.595	0.272	0.543	25.862	-2.846	0.611	0.000	0.000
SEPSECS-AS1	16.586	1.029	0.398	0.010	0.033	16.586	-0.082	0.425	0.847	0.921	16.586	-1.111	0.473	0.019	0.098
FAM200B	188.011	0.232	0.209	0.266	0.402	188.011	0.019	0.209	0.929	0.964	188.011	-0.214	0.242	0.377	0.609
SH3PXD2B	1379.932	-0.306	0.142	0.032	0.082	1379.932	-0.011	0.142	0.935	0.967	1379.932	0.294	0.164	0.073	0.231
FAM153A	18.729	1.904	0.952	0.045	0.107	18.729	1.544	0.954	0.106	0.398	18.729	-0.360	1.082	0.739	0.866
ARL10	215.246	-0.465	0.353	0.188	0.312	215.246	-0.447	0.353	0.205	0.488	215.246	0.018	0.409	0.965	0.984
KIAA0825	14.290	-0.336	0.565	0.552	0.675	14.290	0.078	0.547	0.886	0.943	14.290	0.414	0.649	0.523	0.725
DTWD2	124.334	0.098	0.324	0.763	0.843	124.334	0.209	0.322	0.517	0.722	124.334	0.111	0.374	0.766	0.881
RELL2	185.844	0.523	0.327	0.109	0.209	185.844	-0.593	0.332	0.074	0.364	185.844	-1.116	0.382	0.003	0.032
LINC02159	16.898	0.233	0.591	0.693	0.788	16.898	0.103	0.589	0.862	0.930	16.898	-0.130	0.683	0.848	0.927
C5orf51	800.743	0.082	0.130	0.530	0.656	800.743	-0.091	0.130	0.487	0.703	800.743	-0.172	0.151	0.253	0.485
KIF4B	7.434	-1.986	0.738	0.007	0.026	7.434	-0.030	0.577	0.958	0.978	7.434	1.956	0.813	0.016	0.089
RNF180	85.693	-0.691	0.300	0.021	0.060	85.693	-0.112	0.292	0.700	0.839	85.693	0.579	0.345	0.093	0.268
SREK1IP1	782.067	0.099	0.197	0.616	0.727	782.067	-0.003	0.197	0.990	0.994	782.067	-0.101	0.227	0.656	0.816

RGMB	293.272	-1.119	0.267	0.000	0.000	293.272	-0.366	0.263	0.164	0.454	293.272	0.753	0.308	0.014	0.083
LINC00491	28.933	-0.701	0.363	0.054	0.122	28.933	-0.424	0.352	0.228	0.506	28.933	0.276	0.422	0.513	0.718
CEP57L1	256.151	-0.877	0.192	0.000	0.000	256.151	-0.281	0.188	0.135	0.426	256.151	0.596	0.222	0.007	0.053
PPIL6	115.490	-0.187	0.335	0.576	0.694	115.490	0.396	0.330	0.229	0.507	115.490	0.583	0.384	0.129	0.325
DCBLD1	1397.977	-0.022	0.190	0.907	0.941	1397.977	-0.399	0.190	0.036	0.324	1397.977	-0.377	0.220	0.086	0.256
HLA-F-AS1	604.381	1.409	0.568	0.013	0.042	604.381	0.757	0.569	0.183	0.470	604.381	-0.652	0.656	0.321	0.555
RPL7L1	4190.612	-0.564	0.135	0.000	0.000	4190.612	-0.234	0.135	0.084	0.376	4190.612	0.331	0.156	0.034	0.144
GPR35	16.738	0.352	0.447	0.431	0.567	16.738	0.263	0.443	0.553	0.745	16.738	-0.089	0.513	0.863	0.935
INTS4P1	23.778	-1.212	0.461	0.008	0.030	23.778	-0.302	0.428	0.481	0.700	23.778	0.910	0.527	0.084	0.252
LINC00174	35.669	0.687	0.466	0.141	0.252	35.669	0.631	0.465	0.174	0.464	35.669	-0.056	0.534	0.916	0.961
HOXA-AS2	17.154	0.749	0.546	0.170	0.290	17.154	0.628	0.544	0.248	0.522	17.154	-0.122	0.620	0.844	0.925
SNHG15	416.292	-0.632	0.343	0.065	0.142	416.292	-0.546	0.342	0.111	0.405	416.292	0.086	0.397	0.828	0.915
WEE2-AS1	44.446	0.338	0.519	0.515	0.643	44.446	1.051	0.510	0.039	0.327	44.446	0.712	0.591	0.228	0.457
TCAF2	740.266	0.310	0.282	0.272	0.408	740.266	0.352	0.282	0.212	0.492	740.266	0.042	0.326	0.899	0.952
ZNF775	110.211	0.307	0.239	0.198	0.324	110.211	0.063	0.239	0.791	0.891	110.211	-0.244	0.276	0.378	0.609
ZNF789	46.605	0.405	0.259	0.118	0.221	46.605	-0.089	0.265	0.736	0.859	46.605	-0.495	0.304	0.104	0.287
ANK1	38.224	-1.065	0.446	0.017	0.051	38.224	-0.018	0.427	0.966	0.982	38.224	1.046	0.511	0.040	0.160
FAM86B3P	18.578	0.136	0.388	0.726	0.814	18.578	0.465	0.373	0.212	0.492	18.578	0.330	0.439	0.453	0.672
XKR6	68.316	-0.521	0.276	0.059	0.130	68.316	0.044	0.262	0.868	0.932	68.316	0.565	0.316	0.073	0.232
NSMCE2	415.010	0.083	0.140	0.555	0.677	415.010	0.086	0.139	0.537	0.735	415.010	0.003	0.162	0.983	0.991
ZNF707	258.062	0.188	0.241	0.435	0.571	258.062	-0.339	0.243	0.162	0.453	258.062	-0.527	0.280	0.060	0.204
FAM83H	2864.139	0.105	0.130	0.418	0.555	2864.139	-0.123	0.130	0.343	0.600	2864.139	-0.228	0.150	0.128	0.324
ZNF252P	197.186	-0.816	0.205	0.000	0.001	197.186	-0.368	0.200	0.066	0.354	197.186	0.448	0.237	0.059	0.202
ZFP41	164.678	-0.585	0.219	0.008	0.027	164.678	-0.475	0.217	0.028	0.321	164.678	0.109	0.255	0.667	0.823
SCARA5	35.958	-3.190	1.599	0.046	0.109	35.958	1.108	1.528	0.468	0.691	35.958	4.298	1.824	0.018	0.097
RNF5P1	13.678	0.279	0.528	0.597	0.711	13.678	1.204	0.488	0.014	0.306	13.678	0.925	0.578	0.109	0.295
TRIQK	567.144	-0.051	0.324	0.874	0.919	567.144	0.502	0.323	0.120	0.412	567.144	0.553	0.373	0.139	0.342
DPY19L4	668.335	-0.222	0.271	0.412	0.550	668.335	0.125	0.270	0.644	0.806	668.335	0.347	0.313	0.267	0.502
LACTB2-AS1	117.177	0.346	0.171	0.044	0.105	117.177	-0.248	0.176	0.160	0.451	117.177	-0.594	0.202	0.003	0.031
CRB2	63.226	1.707	0.297	0.000	0.000	63.226	0.645	0.307	0.036	0.324	63.226	-1.062	0.342	0.002	0.021
SCAI	140.414	0.057	0.312	0.856	0.906	140.414	0.548	0.309	0.076	0.367	140.414	0.491	0.359	0.171	0.388
CFAP157	187.478	0.117	0.440	0.790	0.861	187.478	-1.335	0.445	0.003	0.297	187.478	-1.453	0.513	0.005	0.039
PAXX	395.362	0.355	0.333	0.286	0.424	395.362	-0.366	0.334	0.273	0.544	395.362	-0.722	0.385	0.061	0.208
TPRN	361.525	0.479	0.225	0.033	0.085	361.525	0.295	0.225	0.191	0.477	361.525	-0.185	0.260	0.478	0.691
GPR39	49.269	1.170	0.294	0.000	0.001	49.269	-0.121	0.312	0.698	0.838	49.269	-1.291	0.349	0.000	0.004
TUSC1	157.841	0.401	0.198	0.043	0.102	157.841	0.083	0.199	0.678	0.826	157.841	-0.318	0.229	0.165	0.380
FAM225A	36.655	0.122	0.347	0.725	0.813	36.655	0.147	0.343	0.669	0.821	36.655	0.024	0.399	0.951	0.978
FAM78A	40.745	0.733	0.338	0.030	0.078	40.745	0.270	0.342	0.429	0.664	40.745	-0.463	0.390	0.235	0.465
LURAP1L	146.549	-1.127	0.378	0.003	0.013	146.549	-0.954	0.375	0.011	0.302	146.549	0.172	0.439	0.695	0.840
ATP11C	620.845	-0.949	0.280	0.001	0.004	620.845	-0.225	0.278	0.419	0.656	620.845	0.724	0.323	0.025	0.117
LINC00632	169.713	-0.950	0.316	0.003	0.012	169.713	-0.351	0.311	0.259	0.531	169.713	0.599	0.365	0.101	0.282
YIPF6	759.439	-0.086	0.123	0.483	0.616	759.439	-0.026	0.122	0.834	0.915	759.439	0.061	0.142	0.669	0.824
FIRRE	34.753	-0.108	0.456	0.812	0.877	34.753	0.018	0.451	0.968	0.983	34.753	0.127	0.526	0.810	0.904
UBE2E4P	12.729	-0.753	0.558	0.177	0.300	12.729	-0.416	0.536	0.438	0.671	12.729	0.337	0.648	0.603	0.780
TTC3P1	44.003	-0.221	0.311	0.477	0.611	44.003	-0.360	0.311	0.246	0.521	44.003	-0.139	0.364	0.703	0.846
FAM133A	125.429	0.237	0.312	0.448	0.583	125.429	0.006	0.313	0.984	0.990	125.429	-0.231	0.361	0.523	0.725
FFAR3	18.613	0.137	0.576	0.812	0.876	18.613	-1.504	0.696	0.031	0.322	18.613	-1.642	0.776	0.034	0.144
P2RY8	20.599	-4.220	1.309	0.001	0.007	20.599	1.805	0.614	0.003	0.297	20.599	6.025	1.330	0.000	0.000
ILDR1	10.209	-0.362	0.679	0.594	0.709	10.209	0.303	0.648	0.640	0.804	10.209	0.665	0.774	0.390	0.618
GRK4	89.468	0.356	0.224	0.113	0.214	89.468	-0.032	0.227	0.887	0.944	89.468	-0.388	0.262	0.138	0.341
LIN9	239.541	-0.684	0.233	0.003	0.014	239.541	-0.416	0.231	0.071	0.360	239.541	0.268	0.270	0.320	0.555
TRIM59	1142.069	-0.116	0.246	0.638	0.745	1142.069	-0.096	0.246	0.696	0.837	1142.069	0.020	0.284	0.945	0.975
KRT6C	36.756	-0.103	0.465	0.824	0.885	36.756	0.126	0.460	0.784	0.887	36.756	0.229	0.536	0.669	0.824
GRK5	150.236	-0.312	0.387	0.420	0.557	150.236	0.222	0.384	0.563	0.751	150.236	0.534	0.446	0.231	0.460
ANK2	225.646	-0.214	0.257	0.405	0.543	225.646	0.168	0.255	0.510	0.718	225.646	0.382	0.296	0.197	0.420
GRK6	1187.848	-0.170	0.206	0.408	0.547	1187.848	-0.149	0.206	0.469	0.692	1187.848	0.021	0.238	0.929	0.967
MKNK2	8798.034	0.281	0.219	0.199	0.325	8798.034	0.447	0.219	0.041	0.329	8798.034	0.166	0.253	0.511	0.716
GPS1	1803.091	-0.104	0.270	0.700	0.793	1803.091	-0.356	0.270	0.188	0.474	1803.091	-0.252	0.312	0.420	0.644
GPS2	3960.505	-0.064	0.303	0.833	0.890	3960.505	-0.541	0.303	0.074	0.364	3960.505	-0.477	0.350	0.173	0.390
GPT	10.004	1.025	0.860	0.233	0.366	10.004	0.112	0.882	0.899	0.949	10.004	-0.914	1.001	0.361	0.594
GPX1	8541.035	0.725	0.213	0.001	0.004	8541.035	0.329	0.213	0.121	0.413	8541.035	-0.396	0.245	0.107	0.291
GPX3	68.282	0.756	0.267	0.005	0.019	68.282	0.015	0.274	0.958	0.978	68.282	-0.741	0.312	0.017	0.094
GPX4	5257.576	0.889	0.226	0.000	0.001	5257.576	0.280	0.226	0.215	0.494	5257.576	-0.608	0.261	0.020	0.101
ANK3	522.435	-0.203	0.334	0.544	0.669	522.435	0.260	0.334	0.437	0.670	522.435	0.462	0.386	0.231	0.460
GRB2	2068.193	-0.351	0.156	0.025	0.068	2068.193	-0.256	0.156	0.101	0.394	2068.193	0.095	0.180	0.598	0.777
GRB10	4434.929	-0.022	0.180	0.903	0.938	4434.929	-0.129	0.180	0.473	0.695	4434.929	-0.107	0.208	0.606	0.782
RAPGEF1	2025.872	-0.509	0.224	0.023	0.064	2025.872	0.088	0.224	0.693	0.834	2025.872	0.598	0.259	0.021	0.104

GRID1	10.271	0.389	0.598	0.515	0.643	10.271	0.875	0.577	0.129	0.421	10.271	0.486	0.672	0.469	0.684
TRIB2	165.062	-0.291	0.167	0.081	0.167	165.062	0.257	0.157	0.103	0.395	165.062	0.548	0.190	0.004	0.034
CCDC22	314.904	0.241	0.174	0.165	0.285	314.904	0.281	0.173	0.104	0.397	314.904	0.040	0.200	0.842	0.923
DEXI	515.966	0.355	0.134	0.008	0.029	515.966	0.000	0.135	1.000	1.000	515.966	-0.355	0.156	0.023	0.109
LAMTOR2	338.480	0.657	0.187	0.000	0.003	338.480	0.028	0.189	0.883	0.941	338.480	-0.629	0.217	0.004	0.034
MRPS28	256.517	-0.057	0.232	0.805	0.872	256.517	-0.346	0.233	0.138	0.429	256.517	-0.289	0.270	0.285	0.520
COA3	466.161	0.278	0.182	0.126	0.232	466.161	-0.004	0.182	0.981	0.989	466.161	-0.282	0.210	0.179	0.398
GRN	7078.815	1.772	0.333	0.000	0.000	7078.815	0.622	0.333	0.062	0.350	7078.815	-1.150	0.385	0.003	0.027
DCPS	452.232	-0.273	0.137	0.047	0.110	452.232	0.018	0.136	0.897	0.948	452.232	0.291	0.158	0.067	0.218
OSTM1	784.020	0.377	0.313	0.229	0.360	784.020	-0.891	0.314	0.005	0.297	784.020	-1.268	0.363	0.000	0.008
GIT1	1436.126	0.078	0.165	0.636	0.743	1436.126	0.330	0.165	0.046	0.334	1436.126	0.251	0.191	0.188	0.408
SNX24	209.981	0.107	0.225	0.634	0.742	209.981	-0.208	0.226	0.357	0.611	209.981	-0.315	0.261	0.228	0.456
BZW2	3484.602	-0.555	0.157	0.000	0.003	3484.602	-0.274	0.157	0.081	0.372	3484.602	0.281	0.182	0.122	0.313
C11orf54	173.741	0.227	0.247	0.358	0.498	173.741	0.356	0.245	0.146	0.438	173.741	0.129	0.284	0.650	0.812
AAMDC	191.653	1.324	0.314	0.000	0.000	191.653	0.523	0.316	0.098	0.393	191.653	-0.801	0.363	0.027	0.123
SPCS1	1128.273	0.088	0.193	0.647	0.753	1128.273	-0.219	0.193	0.255	0.529	1128.273	-0.307	0.223	0.168	0.382
MRPS18B	5738.276	-0.173	0.123	0.161	0.280	5738.276	0.108	0.123	0.381	0.627	5738.276	0.281	0.143	0.049	0.179
C19orf53	1168.661	0.714	0.248	0.004	0.016	1168.661	-0.012	0.248	0.961	0.980	1168.661	-0.726	0.286	0.011	0.070
ACAD9	806.754	-0.331	0.195	0.090	0.180	806.754	-0.422	0.195	0.030	0.322	806.754	-0.091	0.226	0.688	0.836
MRPL42	1648.174	-0.260	0.164	0.112	0.213	1648.174	-0.311	0.164	0.058	0.347	1648.174	-0.051	0.189	0.789	0.895
TMEM14A	334.972	0.323	0.182	0.076	0.159	334.972	0.018	0.183	0.920	0.960	334.972	-0.305	0.211	0.148	0.356
IFT81	326.482	-0.302	0.260	0.245	0.378	326.482	0.201	0.258	0.436	0.669	326.482	0.503	0.300	0.093	0.268
FLVCR1	336.354	0.343	0.254	0.177	0.299	336.354	-0.439	0.256	0.086	0.378	336.354	-0.782	0.295	0.008	0.056
MCTS1	490.509	-0.092	0.186	0.620	0.730	490.509	-0.323	0.186	0.083	0.374	490.509	-0.230	0.216	0.285	0.521
MAGEH1	133.139	0.457	0.176	0.010	0.033	133.139	-0.235	0.182	0.197	0.480	133.139	-0.692	0.208	0.001	0.012
NOB1	2947.887	-0.180	0.144	0.211	0.341	2947.887	0.197	0.144	0.171	0.460	2947.887	0.377	0.166	0.023	0.112
DBNL	1073.614	-0.241	0.317	0.446	0.581	1073.614	-0.775	0.317	0.015	0.306	1073.614	-0.533	0.366	0.146	0.352
NTMT1	719.796	-0.179	0.393	0.649	0.755	719.796	-0.995	0.394	0.012	0.305	719.796	-0.816	0.455	0.073	0.231
ASTE1	236.511	0.609	0.219	0.006	0.021	236.511	0.142	0.221	0.522	0.725	236.511	-0.467	0.254	0.066	0.216
COMMD5	341.335	-0.014	0.354	0.968	0.980	341.335	-0.829	0.355	0.020	0.306	341.335	-0.815	0.410	0.047	0.176
MACROD1	902.957	0.823	0.258	0.001	0.008	902.957	0.460	0.258	0.075	0.364	902.957	-0.363	0.298	0.222	0.450
HIPK2	2881.405	-0.654	0.156	0.000	0.000	2881.405	-0.304	0.156	0.050	0.337	2881.405	0.349	0.180	0.052	0.188
MRPL13	440.393	-0.308	0.224	0.169	0.289	440.393	-0.531	0.224	0.018	0.306	440.393	-0.223	0.260	0.392	0.620
KLF15	358.462	-1.074	0.192	0.000	0.000	358.462	0.183	0.186	0.325	0.586	358.462	1.256	0.220	0.000	0.000
ABR	2391.996	-0.041	0.175	0.812	0.877	2391.996	-0.266	0.175	0.129	0.420	2391.996	-0.224	0.202	0.267	0.502
ANPEP	9199.063	0.124	0.156	0.429	0.565	9199.063	0.287	0.156	0.066	0.354	9199.063	0.164	0.180	0.365	0.597
ATAD2	957.120	-1.121	0.309	0.000	0.002	957.120	-0.682	0.308	0.027	0.319	957.120	0.440	0.357	0.218	0.444
C16orf72	1765.403	-0.625	0.204	0.002	0.011	1765.403	-0.339	0.204	0.096	0.391	1765.403	0.286	0.236	0.226	0.455
TMEM230	1491.352	0.225	0.132	0.088	0.178	1491.352	-0.130	0.132	0.323	0.584	1491.352	-0.355	0.152	0.020	0.101
GRIN2D	110.786	1.005	0.251	0.000	0.001	110.786	0.543	0.254	0.032	0.322	110.786	-0.462	0.289	0.110	0.295
WDR91	75.822	0.272	0.211	0.198	0.324	75.822	-0.075	0.214	0.724	0.852	75.822	-0.347	0.247	0.159	0.372
ZCCHC4	126.500	-0.832	0.218	0.000	0.001	126.500	-0.508	0.212	0.016	0.306	126.500	0.324	0.253	0.200	0.423
ZC3H7A	1051.275	-0.638	0.136	0.000	0.000	1051.275	-0.035	0.134	0.793	0.892	1051.275	0.603	0.157	0.000	0.003
ZBTB44	625.071	-0.131	0.151	0.386	0.525	625.071	0.012	0.150	0.937	0.968	625.071	0.143	0.174	0.413	0.638
GRINA	6651.178	0.257	0.386	0.506	0.635	6651.178	0.632	0.386	0.101	0.394	6651.178	0.375	0.446	0.400	0.627
CCDC113	99.884	-0.145	0.220	0.509	0.638	99.884	0.103	0.215	0.633	0.800	99.884	0.248	0.253	0.326	0.561
C1GALT1C1	236.323	0.679	0.184	0.000	0.002	236.323	0.099	0.186	0.595	0.775	236.323	-0.580	0.213	0.007	0.049
SETD2	1545.262	-0.575	0.147	0.000	0.001	1545.262	-0.166	0.147	0.259	0.531	1545.262	0.410	0.170	0.016	0.089
MRPL18	1125.548	0.725	0.291	0.013	0.041	1125.548	-0.330	0.292	0.258	0.531	1125.548	-1.055	0.337	0.002	0.020
NDUFAF4	374.984	-0.730	0.373	0.050	0.116	374.984	-0.759	0.373	0.042	0.330	374.984	-0.029	0.432	0.946	0.975
MED4	440.656	0.376	0.147	0.010	0.035	440.656	-0.283	0.149	0.057	0.347	440.656	-0.659	0.171	0.000	0.003
NR3C1	2812.978	-0.952	0.197	0.000	0.000	2812.978	-0.407	0.196	0.038	0.327	2812.978	0.545	0.227	0.016	0.090
CCDC59	704.470	0.068	0.320	0.831	0.889	704.470	-0.464	0.320	0.147	0.438	704.470	-0.533	0.370	0.149	0.358
METTL5	1546.452	0.354	0.238	0.136	0.246	1546.452	-0.211	0.238	0.375	0.623	1546.452	-0.566	0.275	0.040	0.158
CHMP4A	95.523	0.368	0.279	0.186	0.310	95.523	0.184	0.279	0.510	0.718	95.523	-0.185	0.322	0.566	0.756
GTPBP8	321.571	-0.704	0.190	0.000	0.002	321.571	-0.015	0.186	0.938	0.968	321.571	0.689	0.218	0.002	0.019
PHPT1	1520.194	0.789	0.322	0.014	0.044	1520.194	0.490	0.322	0.128	0.420	1520.194	-0.299	0.371	0.420	0.645
BABAM1	1098.866	0.115	0.169	0.498	0.629	1098.866	-0.148	0.169	0.381	0.627	1098.866	-0.263	0.196	0.179	0.397
THYN1	282.528	0.512	0.209	0.014	0.045	282.528	0.067	0.210	0.749	0.866	282.528	-0.445	0.242	0.067	0.218
MRPL15	730.440	-0.552	0.221	0.012	0.040	730.440	-0.279	0.220	0.204	0.487	730.440	0.273	0.255	0.284	0.520
UBE2T	423.750	0.043	0.340	0.898	0.934	423.750	-0.610	0.341	0.074	0.363	423.750	-0.653	0.394	0.097	0.275
ARHGAP35	4658.794	-0.404	0.225	0.073	0.154	4658.794	0.094	0.225	0.677	0.825	4658.794	0.498	0.260	0.055	0.195
TIMM21	560.749	-0.218	0.225	0.332	0.472	560.749	-0.418	0.225	0.064	0.350	560.749	-0.199	0.261	0.444	0.664
LINC00339	227.349	0.267	0.158	0.091	0.183	227.349	0.357	0.157	0.023	0.315	227.349	0.089	0.182	0.622	0.793
MRPL22	729.201	0.479	0.261	0.066	0.143	729.201	-0.137	0.261	0.601	0.779	729.201	-0.615	0.301	0.041	0.162
LGALSL	204.312	0.713	0.142	0.000	0.000	204.312	0.063	0.146	0.666	0.818	204.312	-0.650	0.166	0.000	0.002
ORMDL2	540.824	0.314	0.230	0.173	0.293	540.824	-0.251	0.231	0.277	0.547	540.824	-0.565	0.267	0.034	0.143

CNIH4	1662.893	0.402	0.149	0.007	0.025	1662.893	0.106	0.149	0.477	0.698	1662.893	-0.296	0.172	0.085	0.253
RANGRF	159.912	0.193	0.217	0.374	0.514	159.912	-0.023	0.217	0.915	0.958	159.912	-0.216	0.251	0.390	0.618
COMMD9	258.419	0.527	0.203	0.009	0.032	258.419	-0.095	0.205	0.642	0.805	258.419	-0.622	0.235	0.008	0.057
SLC25A4	624.055	0.410	0.156	0.009	0.031	624.055	0.040	0.157	0.800	0.896	624.055	-0.370	0.181	0.041	0.161
TMEM208	428.758	0.480	0.290	0.098	0.193	428.758	-0.446	0.292	0.127	0.419	428.758	-0.925	0.336	0.006	0.046
SSU72	1787.041	0.094	0.221	0.670	0.770	1787.041	-0.237	0.221	0.285	0.554	1787.041	-0.331	0.256	0.195	0.418
DROSHA	1501.996	-0.651	0.188	0.001	0.004	1501.996	-0.267	0.188	0.155	0.447	1501.996	0.384	0.217	0.077	0.240
DNAJC15	527.305	0.504	0.236	0.033	0.084	527.305	0.006	0.236	0.980	0.989	527.305	-0.498	0.273	0.068	0.221
N6AMT1	119.910	-0.353	0.243	0.146	0.260	119.910	-0.519	0.243	0.033	0.322	119.910	-0.166	0.283	0.558	0.749
CFAP20	506.375	-0.100	0.169	0.555	0.677	506.375	-0.354	0.170	0.037	0.324	506.375	-0.254	0.196	0.195	0.418
NXT1	330.193	-0.468	0.375	0.212	0.341	330.193	-0.836	0.375	0.026	0.316	330.193	-0.368	0.434	0.397	0.625
FHOD1	1309.063	-0.007	0.185	0.970	0.981	1309.063	-0.064	0.184	0.727	0.854	1309.063	-0.057	0.213	0.788	0.894
TBK1	690.973	-0.110	0.184	0.552	0.674	690.973	-0.320	0.184	0.083	0.374	690.973	-0.210	0.213	0.325	0.559
C6orf15	26.320	0.597	0.564	0.290	0.428	26.320	0.420	0.564	0.456	0.683	26.320	-0.176	0.649	0.786	0.893
SAP30BP	1107.207	0.117	0.227	0.606	0.719	1107.207	-0.361	0.228	0.113	0.405	1107.207	-0.479	0.263	0.069	0.223
MYLIP	303.473	-1.054	0.277	0.000	0.001	303.473	0.503	0.270	0.063	0.350	303.473	1.557	0.317	0.000	0.000
BRD7	1424.019	-0.408	0.152	0.007	0.026	1424.019	-0.296	0.151	0.050	0.337	1424.019	0.111	0.175	0.525	0.726
CLEC2D	105.153	0.770	0.323	0.017	0.051	105.153	0.364	0.324	0.262	0.534	105.153	-0.407	0.372	0.275	0.510
ANKRD11	4188.847	-0.540	0.207	0.009	0.031	4188.847	-0.338	0.207	0.102	0.394	4188.847	0.202	0.239	0.398	0.625
CD274	66.347	-1.875	0.659	0.004	0.018	66.347	-1.862	0.657	0.005	0.297	66.347	0.013	0.767	0.986	0.993
RACGAP1	1569.967	-1.051	0.312	0.001	0.005	1569.967	0.044	0.311	0.888	0.944	1569.967	1.095	0.360	0.002	0.024
UHRF1	819.749	-0.462	0.444	0.299	0.437	819.749	-1.315	0.445	0.003	0.297	819.749	-0.854	0.513	0.096	0.274
SLC25A5	10298.042	-0.249	0.176	0.159	0.276	10298.042	0.195	0.176	0.268	0.539	10298.042	0.444	0.204	0.029	0.130
CXCL2	466.498	-0.858	0.748	0.251	0.385	466.498	-2.475	0.750	0.001	0.294	466.498	-1.617	0.866	0.062	0.209
CXCL3	214.770	-1.336	0.747	0.074	0.156	214.770	-2.521	0.753	0.001	0.279	214.770	-1.185	0.871	0.174	0.390
PDIA3	9237.970	0.170	0.163	0.296	0.434	9237.970	-0.051	0.163	0.755	0.869	9237.970	-0.221	0.188	0.240	0.470
GRSF1	2889.351	-0.455	0.105	0.000	0.000	2889.351	-0.013	0.104	0.898	0.948	2889.351	0.442	0.121	0.000	0.005
SLC25A6	46604.498	0.346	0.274	0.206	0.334	46604.498	0.445	0.274	0.104	0.396	46604.498	0.099	0.316	0.754	0.874
GSK3A	1296.722	-0.241	0.135	0.074	0.157	1296.722	-0.131	0.135	0.332	0.592	1296.722	0.110	0.156	0.480	0.693
GSK3B	1511.870	-0.443	0.168	0.008	0.030	1511.870	-0.197	0.167	0.240	0.516	1511.870	0.246	0.194	0.205	0.428
GSN	680.192	1.084	0.481	0.024	0.067	680.192	0.696	0.482	0.148	0.441	680.192	-0.388	0.556	0.484	0.696
GSPT1	3616.804	-0.808	0.239	0.001	0.005	3616.804	-0.420	0.239	0.079	0.370	3616.804	0.388	0.277	0.161	0.374
GSR	1358.905	-0.198	0.207	0.339	0.479	1358.905	-0.215	0.207	0.300	0.567	1358.905	-0.016	0.240	0.946	0.975
GSS	698.346	0.259	0.166	0.118	0.222	698.346	-0.087	0.166	0.601	0.779	698.346	-0.346	0.192	0.071	0.228
GSTA4	439.934	0.505	0.391	0.196	0.322	439.934	0.604	0.391	0.122	0.413	439.934	0.099	0.451	0.826	0.914
GSTM3	53.255	0.497	0.236	0.035	0.089	53.255	0.132	0.240	0.583	0.766	53.255	-0.365	0.274	0.183	0.401
GSTM4	89.859	0.961	0.332	0.004	0.016	89.859	0.376	0.335	0.262	0.534	89.859	-0.585	0.383	0.127	0.322
GSTP1	10512.589	0.355	0.229	0.121	0.226	10512.589	0.247	0.229	0.281	0.551	10512.589	-0.108	0.264	0.683	0.832
GSTT1	161.770	0.168	0.294	0.569	0.688	161.770	0.370	0.292	0.205	0.488	161.770	0.203	0.338	0.549	0.744
GSTT2	164.413	1.748	0.246	0.000	0.000	164.413	0.360	0.253	0.155	0.447	164.413	-1.388	0.285	0.000	0.000
GSTZ1	335.165	0.130	0.352	0.711	0.802	335.165	0.405	0.351	0.248	0.522	335.165	0.275	0.405	0.497	0.705
MSH6	1268.727	-0.185	0.142	0.192	0.316	1268.727	-0.267	0.141	0.059	0.350	1268.727	-0.082	0.164	0.618	0.790
GTF2A1	463.732	-0.800	0.215	0.000	0.002	463.732	-0.276	0.213	0.195	0.478	463.732	0.525	0.248	0.034	0.144
GTF2A2	899.850	0.024	0.283	0.934	0.958	899.850	-0.457	0.284	0.107	0.399	899.850	-0.481	0.328	0.142	0.347
GTF2B	414.373	0.429	0.126	0.001	0.004	414.373	-0.112	0.128	0.382	0.629	414.373	-0.540	0.147	0.000	0.004
GTF2E1	300.482	-0.421	0.250	0.093	0.185	300.482	-0.591	0.250	0.018	0.306	300.482	-0.170	0.291	0.559	0.750
GTF2E2	943.479	-0.435	0.190	0.022	0.062	943.479	-0.255	0.190	0.179	0.467	943.479	0.180	0.220	0.413	0.638
GTF2F1	2754.460	0.042	0.143	0.770	0.847	2754.460	0.128	0.143	0.370	0.619	2754.460	0.086	0.165	0.602	0.779
GTF2F2	685.517	-0.248	0.149	0.096	0.190	685.517	-0.164	0.148	0.268	0.540	685.517	0.084	0.172	0.627	0.797
GTF2H1	1025.803	-0.730	0.183	0.000	0.001	1025.803	-0.335	0.182	0.066	0.354	1025.803	0.395	0.212	0.062	0.210
GTF2H2	325.984	-0.949	0.328	0.004	0.016	325.984	-0.679	0.327	0.038	0.327	325.984	0.271	0.379	0.475	0.689
GTF2H3	1064.276	-1.035	0.222	0.000	0.000	1064.276	-0.509	0.221	0.021	0.313	1064.276	0.526	0.256	0.040	0.158
GTF2H4	181.371	-0.036	0.317	0.910	0.942	181.371	-0.097	0.316	0.760	0.872	181.371	-0.061	0.366	0.868	0.937
GTF2I	3345.748	-0.129	0.150	0.387	0.526	3345.748	0.111	0.149	0.457	0.683	3345.748	0.241	0.173	0.163	0.377
GTF2IP1	4683.306	0.078	0.154	0.614	0.725	4683.306	0.100	0.154	0.516	0.722	4683.306	0.022	0.177	0.900	0.953
GTF3A	1386.515	-0.074	0.239	0.758	0.839	1386.515	-0.202	0.238	0.396	0.640	1386.515	-0.129	0.276	0.640	0.805
BRF1	673.884	0.043	0.196	0.828	0.887	673.884	-0.144	0.196	0.464	0.689	673.884	-0.186	0.227	0.412	0.638
GTF3C1	1643.284	-0.250	0.099	0.011	0.037	1643.284	0.006	0.098	0.955	0.978	1643.284	0.256	0.114	0.025	0.116
GTF3C2	846.616	-0.569	0.213	0.008	0.028	846.616	-0.411	0.213	0.054	0.342	846.616	0.158	0.247	0.521	0.724
USP25	913.787	-0.711	0.118	0.000	0.000	913.787	0.093	0.116	0.419	0.656	913.787	0.805	0.136	0.000	0.000
PACSIN3	405.995	0.364	0.176	0.038	0.095	405.995	0.215	0.176	0.221	0.501	405.995	-0.149	0.203	0.463	0.679
TMOD3	1569.386	-0.349	0.155	0.024	0.066	1569.386	-0.177	0.154	0.251	0.525	1569.386	0.172	0.179	0.334	0.569
GUCY1A2	55.948	1.073	0.353	0.002	0.011	55.948	-0.364	0.366	0.320	0.581	55.948	-1.438	0.416	0.001	0.009
CARD10	324.452	0.031	0.226	0.890	0.929	324.452	-0.382	0.227	0.093	0.387	324.452	-0.413	0.262	0.116	0.305
ABT1	619.002	0.149	0.138	0.278	0.416	619.002	-0.122	0.138	0.379	0.626	619.002	-0.271	0.160	0.090	0.263
PARVB	822.643	0.002	0.155	0.989	0.994	822.643	-0.076	0.155	0.626	0.795	822.643	-0.078	0.180	0.665	0.822
NCAPH2	1272.073	-0.068	0.231	0.768	0.846	1272.073	-0.303	0.231	0.189	0.475	1272.073	-0.235	0.267	0.378	0.609

OLA1	3707.074	0.023	0.133	0.865	0.912	3707.074	0.106	0.133	0.426	0.662	3707.074	0.083	0.154	0.589	0.769
GUCA1B	15.662	1.199	0.588	0.041	0.100	15.662	-0.177	0.631	0.779	0.883	15.662	-1.376	0.702	0.050	0.182
UQCR10	590.875	0.579	0.282	0.040	0.098	590.875	-0.414	0.284	0.145	0.436	590.875	-0.993	0.327	0.002	0.025
C2orf27A	97.445	-0.536	0.177	0.002	0.011	97.445	-0.253	0.169	0.135	0.426	97.445	0.283	0.205	0.168	0.383
YPEL1	87.964	0.655	0.299	0.029	0.076	87.964	0.154	0.302	0.609	0.784	87.964	-0.500	0.346	0.149	0.357
ZDHHC1	155.499	1.135	0.364	0.002	0.009	155.499	0.681	0.365	0.062	0.350	155.499	-0.453	0.419	0.279	0.514
ZDHHC8	768.859	0.263	0.177	0.136	0.246	768.859	-0.190	0.177	0.283	0.552	768.859	-0.453	0.204	0.027	0.122
REPIN1	2586.338	0.306	0.084	0.000	0.002	2586.338	0.045	0.084	0.596	0.775	2586.338	-0.261	0.097	0.007	0.053
GRHL1	8.410	1.402	0.680	0.039	0.096	8.410	-0.484	0.744	0.515	0.721	8.410	-1.886	0.824	0.022	0.108
TFCP2L1	7.583	-0.810	0.917	0.377	0.517	7.583	0.289	0.867	0.738	0.860	7.583	1.099	1.043	0.292	0.527
SENP1	324.558	-0.660	0.288	0.022	0.062	324.558	-0.464	0.287	0.106	0.398	324.558	0.196	0.333	0.557	0.749
TFPT	1156.370	0.249	0.232	0.284	0.421	1156.370	-0.353	0.232	0.128	0.420	1156.370	-0.602	0.268	0.025	0.116
UBN1	2103.076	-0.552	0.237	0.020	0.058	2103.076	0.231	0.237	0.328	0.588	2103.076	0.783	0.274	0.004	0.037
GUK1	3856.410	0.790	0.194	0.000	0.001	3856.410	0.167	0.195	0.392	0.636	3856.410	-0.623	0.225	0.006	0.044
ALG5	509.845	-0.348	0.143	0.015	0.047	509.845	-0.022	0.141	0.878	0.939	509.845	0.326	0.165	0.048	0.179
ANAPC2	500.979	0.249	0.141	0.077	0.161	500.979	-0.030	0.141	0.834	0.915	500.979	-0.279	0.163	0.088	0.259
CNOT7	1530.154	-0.546	0.116	0.000	0.000	1530.154	-0.182	0.115	0.113	0.405	1530.154	0.364	0.134	0.007	0.049
SNX8	1141.072	0.288	0.169	0.089	0.179	1141.072	-0.214	0.170	0.208	0.490	1141.072	-0.502	0.196	0.010	0.068
SNX10	774.060	1.476	0.160	0.000	0.000	774.060	0.493	0.162	0.002	0.297	774.060	-0.982	0.185	0.000	0.000
STRN4	2161.526	-0.022	0.113	0.845	0.898	2161.526	-0.131	0.113	0.248	0.522	2161.526	-0.108	0.131	0.407	0.634
GNL2	2050.979	-0.222	0.288	0.442	0.577	2050.979	-0.563	0.289	0.051	0.338	2050.979	-0.341	0.333	0.306	0.541
RBM15B	1621.640	-0.212	0.093	0.023	0.063	1621.640	-0.064	0.092	0.486	0.703	1621.640	0.147	0.107	0.169	0.385
PSMC3IP	506.088	-0.143	0.359	0.690	0.786	506.088	-0.648	0.359	0.071	0.360	506.088	-0.505	0.415	0.224	0.451
CPSF1	1129.647	-0.159	0.120	0.184	0.308	1129.647	-0.042	0.119	0.725	0.853	1129.647	0.117	0.138	0.397	0.625
TRA2A	653.211	-0.425	0.302	0.159	0.276	653.211	-0.697	0.302	0.021	0.313	653.211	-0.271	0.349	0.437	0.658
GPSM2	1769.123	-0.413	0.228	0.070	0.149	1769.123	0.240	0.227	0.290	0.558	1769.123	0.653	0.263	0.013	0.078
GUSB	594.322	0.237	0.145	0.102	0.198	594.322	0.106	0.145	0.465	0.689	594.322	-0.131	0.167	0.433	0.655
SAC3D1	334.436	0.318	0.216	0.141	0.253	334.436	-0.125	0.217	0.565	0.752	334.436	-0.443	0.250	0.077	0.239
FAM216A	621.171	-0.128	0.147	0.383	0.523	621.171	0.208	0.145	0.152	0.444	621.171	0.337	0.169	0.047	0.175
CCDC106	393.558	0.405	0.238	0.088	0.178	393.558	0.212	0.238	0.371	0.620	393.558	-0.193	0.274	0.481	0.694
EEF2K	754.826	-0.227	0.133	0.089	0.180	754.826	0.038	0.132	0.777	0.881	754.826	0.264	0.154	0.086	0.255
HOOK2	1708.031	0.607	0.103	0.000	0.000	1708.031	0.191	0.103	0.065	0.353	1708.031	-0.416	0.119	0.000	0.008
UBIAD1	456.326	-1.044	0.321	0.001	0.006	456.326	-0.692	0.320	0.031	0.322	456.326	0.352	0.372	0.343	0.577
HCFC2	243.064	-0.317	0.246	0.196	0.322	243.064	0.056	0.243	0.818	0.905	243.064	0.373	0.283	0.187	0.407
SNX11	279.216	-0.333	0.313	0.287	0.425	279.216	-0.662	0.313	0.035	0.322	279.216	-0.329	0.363	0.365	0.598
RMC1	345.362	0.848	0.354	0.017	0.050	345.362	-0.540	0.357	0.130	0.422	345.362	-1.388	0.411	0.001	0.011
GYG1	744.517	0.206	0.250	0.410	0.548	744.517	-0.316	0.251	0.207	0.489	744.517	-0.522	0.290	0.071	0.228
PYCR2	518.284	0.493	0.318	0.121	0.225	518.284	-0.479	0.319	0.133	0.425	518.284	-0.971	0.368	0.008	0.057
NME7	493.716	-0.139	0.139	0.315	0.454	493.716	-0.080	0.138	0.560	0.749	493.716	0.059	0.160	0.713	0.850
HILPDA	1873.703	1.138	0.758	0.133	0.242	1873.703	0.959	0.758	0.206	0.488	1873.703	-0.179	0.875	0.838	0.922
EPN1	3080.073	0.329	0.159	0.039	0.095	3080.073	0.124	0.159	0.438	0.671	3080.073	-0.206	0.184	0.263	0.497
GMPPB	443.969	-0.072	0.358	0.841	0.896	443.969	-0.529	0.359	0.140	0.431	443.969	-0.457	0.414	0.270	0.505
GMPPA	548.018	0.455	0.183	0.013	0.041	548.018	-0.031	0.184	0.866	0.932	548.018	-0.486	0.212	0.022	0.108
SEC61A1	6920.567	-0.184	0.177	0.298	0.437	6920.567	-0.109	0.177	0.539	0.736	6920.567	0.076	0.205	0.712	0.850
TIMM22	644.979	0.279	0.286	0.328	0.468	644.979	-0.296	0.286	0.301	0.567	644.979	-0.576	0.330	0.082	0.247
ALG6	182.410	0.166	0.216	0.443	0.578	182.410	-0.381	0.218	0.081	0.372	182.410	-0.547	0.252	0.030	0.132
SNX12	1060.210	-0.102	0.131	0.435	0.571	1060.210	-0.077	0.130	0.557	0.747	1060.210	0.026	0.151	0.865	0.935
NENF	749.422	0.992	0.278	0.000	0.003	749.422	0.461	0.278	0.097	0.392	749.422	-0.531	0.320	0.098	0.277
DSE	2649.899	-0.603	0.284	0.034	0.086	2649.899	-0.265	0.284	0.349	0.604	2649.899	0.338	0.328	0.303	0.538
PKN3	138.225	0.606	0.220	0.006	0.022	138.225	0.005	0.223	0.983	0.990	138.225	-0.602	0.256	0.019	0.097
ANAPC4	269.639	-0.550	0.171	0.001	0.007	269.639	-0.477	0.169	0.005	0.297	269.639	0.072	0.198	0.716	0.853
SERTAD3	307.273	0.770	0.167	0.000	0.000	307.273	0.259	0.168	0.124	0.416	307.273	-0.511	0.193	0.008	0.057
OSGIN1	285.611	0.977	0.169	0.000	0.000	285.611	0.413	0.171	0.016	0.306	285.611	-0.564	0.194	0.004	0.034
GYPC	934.075	0.780	0.271	0.004	0.017	934.075	0.513	0.271	0.059	0.349	934.075	-0.267	0.313	0.393	0.621
SERTAD1	359.409	0.491	0.331	0.138	0.248	359.409	-0.422	0.335	0.208	0.490	359.409	-0.913	0.385	0.018	0.095
DPP7	2366.041	1.025	0.215	0.000	0.000	2366.041	0.334	0.216	0.121	0.413	2366.041	-0.691	0.249	0.005	0.044
TRHDE	149.831	-0.496	0.279	0.076	0.158	149.831	-0.194	0.276	0.483	0.701	149.831	0.302	0.322	0.349	0.582
POMT2	302.943	-0.044	0.202	0.827	0.886	302.943	-0.237	0.202	0.241	0.517	302.943	-0.193	0.234	0.410	0.636
CERS2	5310.624	0.087	0.090	0.335	0.475	5310.624	-0.007	0.090	0.941	0.970	5310.624	-0.094	0.104	0.369	0.601
SLC25A24	1042.577	-0.720	0.266	0.007	0.025	1042.577	-0.157	0.265	0.553	0.745	1042.577	0.563	0.307	0.067	0.218
DMGDH	11.023	-0.924	0.887	0.298	0.436	11.023	0.517	0.835	0.536	0.734	11.023	1.441	1.006	0.152	0.361
NRBP1	1249.067	0.272	0.139	0.050	0.115	1249.067	-0.028	0.139	0.840	0.917	1249.067	-0.300	0.161	0.061	0.208
MRM2	33.027	-0.927	0.478	0.052	0.120	33.027	-0.661	0.469	0.158	0.451	33.027	0.266	0.556	0.632	0.800
PRICKLE4	132.529	0.936	0.336	0.005	0.021	132.529	0.760	0.336	0.024	0.316	132.529	-0.176	0.386	0.649	0.811
CDIP1	26.756	-0.535	0.490	0.275	0.412	26.756	-0.567	0.486	0.244	0.519	26.756	-0.032	0.573	0.955	0.979
STRN3	443.528	-0.572	0.206	0.005	0.021	443.528	-0.105	0.204	0.607	0.783	443.528	0.467	0.237	0.049	0.181
LRP12	451.073	-0.460	0.180	0.011	0.036	451.073	-0.123	0.178	0.489	0.705	451.073	0.337	0.208	0.105	0.289

PSAT1	5024.714	-2.171	0.295	0.000	0.000	5024.714	-0.333	0.295	0.259	0.531	5024.714	1.838	0.341	0.000	0.000
MDFIC	527.226	-0.327	0.221	0.140	0.251	527.226	-0.035	0.220	0.875	0.937	527.226	0.292	0.256	0.253	0.485
GYS1	3203.356	0.264	0.370	0.476	0.609	3203.356	0.543	0.370	0.142	0.434	3203.356	0.279	0.427	0.514	0.719
SCHIP1	20.713	0.482	0.342	0.159	0.277	20.713	-0.536	0.368	0.145	0.437	20.713	-1.018	0.416	0.014	0.083
UBQLN2	773.998	-0.332	0.145	0.022	0.062	773.998	-0.064	0.144	0.654	0.811	773.998	0.268	0.168	0.110	0.296
UBQLN1	3633.543	-0.906	0.222	0.000	0.000	3633.543	-0.337	0.222	0.129	0.421	3633.543	0.569	0.256	0.026	0.121
DONSON	389.473	-0.505	0.283	0.074	0.156	389.473	-0.539	0.282	0.056	0.344	389.473	-0.034	0.327	0.918	0.962
NRBF2	691.417	-0.614	0.237	0.010	0.033	691.417	-0.500	0.237	0.035	0.322	691.417	0.113	0.274	0.680	0.830
RHOD	14.528	0.297	0.515	0.564	0.685	14.528	0.470	0.504	0.351	0.606	14.528	0.173	0.586	0.768	0.882
SLC39A3	509.514	0.511	0.398	0.199	0.325	509.514	-0.732	0.399	0.067	0.354	509.514	-1.243	0.460	0.007	0.052
SLC2A8	175.603	0.354	0.217	0.102	0.198	175.603	-0.116	0.218	0.595	0.775	175.603	-0.470	0.252	0.062	0.208
BAZ2B	124.597	-0.143	0.251	0.569	0.688	124.597	0.248	0.247	0.315	0.578	124.597	0.391	0.289	0.175	0.393
LMCD1	42.085	0.989	0.432	0.022	0.062	42.085	-0.033	0.442	0.941	0.970	42.085	-1.022	0.504	0.043	0.165
NOP53	11821.378	0.595	0.325	0.068	0.145	11821.378	0.553	0.325	0.089	0.383	11821.378	-0.042	0.376	0.911	0.959
BICRA	317.364	0.098	0.175	0.576	0.694	317.364	0.179	0.174	0.303	0.569	317.364	0.081	0.202	0.687	0.836
ACAA1	698.673	0.516	0.142	0.000	0.002	698.673	-0.005	0.143	0.970	0.984	698.673	-0.522	0.165	0.002	0.018
TNPO2	1363.032	-0.597	0.134	0.000	0.000	1363.032	-0.042	0.133	0.752	0.867	1363.032	0.555	0.155	0.000	0.006
ERO1A	3312.948	0.525	0.230	0.023	0.063	3312.948	0.280	0.230	0.224	0.503	3312.948	-0.245	0.266	0.357	0.590
EFEMP2	54.600	2.612	0.739	0.000	0.003	54.600	2.284	0.739	0.002	0.297	54.600	-0.329	0.841	0.696	0.841
SH3KBP1	2200.858	-1.140	0.280	0.000	0.001	2200.858	-0.619	0.279	0.027	0.318	2200.858	0.521	0.323	0.106	0.291
H1-0	8277.351	-1.200	0.136	0.000	0.000	8277.351	-0.030	0.135	0.823	0.908	8277.351	1.170	0.157	0.000	0.000
H1-2	678.130	0.677	0.790	0.391	0.530	678.130	0.853	0.790	0.280	0.550	678.130	0.176	0.912	0.847	0.926
H1-4	10.710	-0.111	0.644	0.863	0.911	10.710	-0.367	0.648	0.571	0.757	10.710	-0.256	0.758	0.735	0.864
ANXA1	5099.515	0.092	0.187	0.623	0.733	5099.515	-0.345	0.187	0.066	0.354	5099.515	-0.437	0.216	0.043	0.167
H2AC8	26.638	0.523	0.477	0.273	0.410	26.638	0.623	0.469	0.184	0.471	26.638	0.100	0.541	0.853	0.930
H2AX	2121.948	0.405	0.206	0.049	0.114	2121.948	-0.052	0.206	0.800	0.896	2121.948	-0.458	0.238	0.055	0.194
H2AZ1	5722.678	-0.430	0.264	0.103	0.200	5722.678	-0.607	0.264	0.021	0.314	5722.678	-0.177	0.305	0.562	0.753
H2BC5	350.859	0.617	0.746	0.408	0.546	350.859	0.691	0.745	0.354	0.608	350.859	0.074	0.861	0.931	0.969
ANXA2	32384.924	0.190	0.133	0.154	0.270	32384.924	-0.094	0.133	0.480	0.699	32384.924	-0.284	0.154	0.065	0.214
H3-3A	55.450	0.160	0.275	0.562	0.683	55.450	0.359	0.269	0.183	0.469	55.450	0.199	0.315	0.527	0.727
H3-3B	8640.708	0.068	0.147	0.643	0.749	8640.708	-0.294	0.147	0.046	0.335	8640.708	-0.362	0.170	0.033	0.141
HSD17B10	1394.461	0.054	0.257	0.833	0.890	1394.461	-0.336	0.257	0.192	0.477	1394.461	-0.390	0.297	0.189	0.410
HAGH	524.570	1.361	0.243	0.000	0.000	524.570	0.193	0.246	0.432	0.666	524.570	-1.168	0.282	0.000	0.001
HADHA	4696.719	-0.186	0.163	0.253	0.387	4696.719	0.162	0.163	0.319	0.581	4696.719	0.349	0.188	0.064	0.213
HADHB	2466.331	-0.235	0.267	0.378	0.518	2466.331	0.315	0.267	0.238	0.515	2466.331	0.550	0.308	0.074	0.234
HADH	803.485	-0.124	0.166	0.454	0.589	803.485	0.205	0.165	0.215	0.494	803.485	0.329	0.191	0.086	0.255
HAL	33.832	-1.125	1.209	0.352	0.492	33.832	1.238	1.190	0.298	0.565	33.832	2.363	1.388	0.089	0.261
HARS1	1439.479	0.118	0.245	0.629	0.738	1439.479	-0.432	0.245	0.078	0.369	1439.479	-0.550	0.283	0.052	0.187
HAS3	395.438	-0.058	0.187	0.756	0.837	395.438	-0.182	0.187	0.331	0.590	395.438	-0.123	0.216	0.569	0.758
ANXA2P2	1587.245	0.129	0.131	0.323	0.463	1587.245	-0.069	0.131	0.598	0.777	1587.245	-0.198	0.151	0.190	0.411
HBA2	15.852	-1.588	1.799	0.377	0.517	15.852	-2.716	1.951	0.164	0.454	15.852	-1.128	2.281	0.621	0.792
HBE1	781.207	0.414	0.400	0.300	0.439	781.207	0.811	0.399	0.042	0.330	781.207	0.397	0.461	0.389	0.618
HBG1	6.043	-0.126	1.040	0.904	0.938	6.043	0.235	1.020	0.818	0.905	6.043	0.361	1.194	0.763	0.879
HBG2	94.933	0.129	0.322	0.689	0.785	94.933	0.397	0.319	0.213	0.493	94.933	0.268	0.370	0.469	0.684
HCCS	433.401	-0.177	0.292	0.544	0.668	433.401	-0.629	0.293	0.032	0.322	433.401	-0.452	0.338	0.182	0.400
HCFC1	1304.674	-0.620	0.224	0.006	0.022	1304.674	-0.314	0.224	0.160	0.452	1304.674	0.306	0.259	0.237	0.467
HCLS1	17.109	-0.793	0.462	0.086	0.174	17.109	-0.775	0.452	0.087	0.380	17.109	0.018	0.548	0.974	0.987
ANXA3	185.061	0.116	0.174	0.504	0.634	185.061	-0.226	0.175	0.196	0.480	185.061	-0.342	0.202	0.091	0.264
HCRTR1	120.663	-0.024	0.253	0.923	0.951	120.663	-0.241	0.254	0.343	0.600	120.663	-0.216	0.295	0.463	0.679
HTT	1259.985	0.221	0.118	0.061	0.134	1259.985	-0.157	0.118	0.184	0.470	1259.985	-0.378	0.137	0.006	0.044
HDAC1	2857.863	0.002	0.099	0.980	0.988	2857.863	-0.118	0.099	0.234	0.511	2857.863	-0.120	0.114	0.293	0.528
HDAC2	3377.828	-0.434	0.098	0.000	0.000	3377.828	-0.130	0.098	0.185	0.472	3377.828	0.305	0.114	0.007	0.053
HDGF	10677.938	-0.220	0.144	0.127	0.234	10677.938	-0.144	0.144	0.319	0.581	10677.938	0.076	0.166	0.647	0.809
HDLBP	13079.827	-0.411	0.187	0.028	0.073	13079.827	0.104	0.186	0.579	0.763	13079.827	0.515	0.215	0.017	0.092
ANXA4	1202.374	0.584	0.169	0.001	0.004	1202.374	0.020	0.169	0.905	0.952	1202.374	-0.563	0.195	0.004	0.035
HELLS	265.421	-0.554	0.483	0.251	0.385	265.421	-1.155	0.484	0.017	0.306	265.421	-0.601	0.559	0.282	0.517
HEXA	1119.448	1.256	0.234	0.000	0.000	1119.448	0.419	0.234	0.073	0.362	1119.448	-0.836	0.270	0.002	0.021
HEXB	2874.589	0.328	0.144	0.022	0.063	2874.589	-0.032	0.144	0.824	0.908	2874.589	-0.360	0.166	0.030	0.132
CFH	826.412	-0.386	0.374	0.301	0.440	826.412	0.503	0.373	0.177	0.466	826.412	0.889	0.431	0.039	0.156
HFE	220.647	0.543	0.228	0.017	0.052	220.647	0.332	0.228	0.145	0.437	220.647	-0.210	0.263	0.424	0.648
CFHR1	11.763	1.170	0.659	0.076	0.159	11.763	0.756	0.666	0.256	0.529	11.763	-0.414	0.749	0.581	0.764
ANXA5	4694.163	0.465	0.230	0.043	0.104	4694.163	-0.496	0.230	0.031	0.322	4694.163	-0.961	0.266	0.000	0.005
ST6GALNAC6	875.725	0.375	0.225	0.095	0.189	875.725	0.381	0.225	0.090	0.384	875.725	0.005	0.259	0.983	0.991
ADGRE2	159.305	-1.623	0.403	0.000	0.001	159.305	-0.036	0.393	0.928	0.964	159.305	1.587	0.463	0.001	0.009
KCNIP3	48.706	0.816	0.278	0.003	0.015	48.706	-0.811	0.307	0.008	0.297	48.706	-1.627	0.343	0.000	0.000
KCNIP2	10.072	0.769	0.557	0.168	0.288	10.072	0.811	0.550	0.141	0.431	10.072	0.042	0.628	0.947	0.975
HGF	12.908	0.927	0.539	0.086	0.174	12.908	0.667	0.542	0.219	0.498	12.908	-0.260	0.612	0.670	0.824

CXXC1	708.642	-0.277	0.176	0.116	0.219	708.642	-0.057	0.175	0.744	0.863	708.642	0.219	0.203	0.280	0.515
ZNF354C	35.199	-0.201	0.447	0.653	0.757	35.199	0.136	0.439	0.757	0.871	35.199	0.337	0.515	0.512	0.718
NT5C	574.700	0.203	0.217	0.350	0.490	574.700	0.052	0.217	0.811	0.900	574.700	-0.151	0.251	0.548	0.742
ZNRD1	1434.513	0.196	0.233	0.401	0.539	1434.513	0.036	0.233	0.876	0.937	1434.513	-0.159	0.269	0.554	0.747
DNTTIP2	1145.262	-0.255	0.285	0.372	0.513	1145.262	-0.697	0.286	0.015	0.306	1145.262	-0.443	0.330	0.180	0.398
SOCS7	1460.345	-0.501	0.158	0.002	0.008	1460.345	0.032	0.157	0.840	0.917	1460.345	0.533	0.183	0.003	0.032
NRG1	20.570	-1.231	0.551	0.025	0.069	20.570	-1.346	0.549	0.014	0.306	20.570	-0.115	0.653	0.860	0.933
EHD4	1203.116	-0.323	0.240	0.180	0.302	1203.116	-0.527	0.240	0.028	0.321	1203.116	-0.205	0.278	0.462	0.678
EHD3	67.734	0.485	0.302	0.108	0.207	67.734	-0.081	0.306	0.791	0.891	67.734	-0.566	0.351	0.107	0.291
EHD2	7439.425	0.426	0.231	0.066	0.142	7439.425	0.333	0.231	0.150	0.443	7439.425	-0.093	0.267	0.728	0.859
PIK3R4	448.163	-0.455	0.241	0.059	0.130	448.163	-0.662	0.241	0.006	0.297	448.163	-0.207	0.279	0.460	0.677
CDR2L	744.653	0.174	0.206	0.400	0.538	744.653	-0.347	0.207	0.094	0.388	744.653	-0.520	0.239	0.030	0.130
TAX1BP3	288.600	0.320	0.179	0.073	0.155	288.600	0.051	0.179	0.774	0.880	288.600	-0.268	0.207	0.194	0.416
HHEX	30.363	-0.698	0.338	0.039	0.095	30.363	-0.919	0.339	0.007	0.297	30.363	-0.220	0.405	0.586	0.767
ANXA6	748.031	0.841	0.125	0.000	0.000	748.031	-0.033	0.127	0.793	0.892	748.031	-0.874	0.145	0.000	0.000
HIC1	242.397	0.597	0.180	0.001	0.005	242.397	-0.046	0.183	0.800	0.896	242.397	-0.644	0.209	0.002	0.022
HIF1A	9957.509	-0.738	0.303	0.015	0.046	9957.509	-0.714	0.303	0.018	0.306	9957.509	0.024	0.350	0.945	0.975
HIP1	751.344	-0.243	0.194	0.208	0.337	751.344	0.199	0.193	0.300	0.567	751.344	0.443	0.223	0.047	0.176
UBE2K	2064.714	-0.529	0.171	0.002	0.010	2064.714	-0.472	0.171	0.006	0.297	2064.714	0.057	0.198	0.775	0.886
HINT1	3806.244	0.442	0.185	0.017	0.051	3806.244	0.322	0.185	0.082	0.373	3806.244	-0.120	0.214	0.576	0.762
HIVEP1	361.632	-0.725	0.213	0.001	0.004	361.632	-0.099	0.210	0.637	0.802	361.632	0.626	0.246	0.011	0.069
STOML2	2316.895	-0.353	0.162	0.029	0.076	2316.895	-0.132	0.162	0.415	0.653	2316.895	0.222	0.187	0.236	0.465
HIVEP2	1185.359	-0.180	0.235	0.443	0.579	1185.359	-0.356	0.235	0.130	0.422	1185.359	-0.176	0.271	0.517	0.721
HK1	10969.825	-0.353	0.127	0.005	0.021	10969.825	0.140	0.127	0.270	0.542	10969.825	0.493	0.147	0.001	0.011
HK2	4285.260	0.327	0.276	0.236	0.369	4285.260	0.376	0.276	0.174	0.463	4285.260	0.049	0.319	0.879	0.942
ACACA	5393.935	0.021	0.165	0.898	0.934	5393.935	-0.120	0.165	0.467	0.690	5393.935	-0.141	0.191	0.458	0.676
ANXA7	1656.696	-0.163	0.300	0.587	0.703	1656.696	-0.687	0.301	0.022	0.315	1656.696	-0.524	0.347	0.131	0.329
ZBTB48	203.642	0.404	0.252	0.109	0.208	203.642	-0.181	0.254	0.476	0.697	203.642	-0.585	0.293	0.045	0.172
HLA-A	58849.007	0.594	0.239	0.013	0.041	58849.007	0.474	0.239	0.047	0.336	58849.007	-0.120	0.276	0.664	0.821
HLA-B	14367.318	1.033	0.353	0.003	0.015	14367.318	0.579	0.353	0.101	0.394	14367.318	-0.454	0.408	0.266	0.500
HLA-C	11109.572	0.815	0.262	0.002	0.009	11109.572	0.484	0.262	0.064	0.353	11109.572	-0.330	0.302	0.275	0.510
HLA-DMA	172.284	0.832	0.318	0.009	0.031	172.284	1.102	0.317	0.000	0.253	172.284	0.271	0.365	0.458	0.675
ANXA11	3112.884	0.448	0.127	0.000	0.003	3112.884	0.087	0.127	0.495	0.709	3112.884	-0.361	0.147	0.014	0.080
HLA-DOB	105.584	1.874	0.420	0.000	0.000	105.584	0.428	0.426	0.314	0.577	105.584	-1.445	0.486	0.003	0.028
HLF	48.252	-1.201	0.666	0.071	0.151	48.252	0.410	0.650	0.528	0.729	48.252	1.611	0.764	0.035	0.145
HLA-E	27943.599	0.729	0.333	0.028	0.075	27943.599	0.556	0.333	0.095	0.389	27943.599	-0.173	0.384	0.653	0.813
HLA-F	1715.759	1.838	0.483	0.000	0.001	1715.759	0.840	0.484	0.083	0.374	1715.759	-0.999	0.558	0.074	0.232
HLA-H	1073.790	0.648	0.270	0.016	0.049	1073.790	0.399	0.270	0.140	0.431	1073.790	-0.250	0.312	0.423	0.647
HLA-J	113.598	0.107	0.485	0.825	0.885	113.598	0.432	0.483	0.372	0.620	113.598	0.324	0.559	0.562	0.753
HLA-L	136.981	0.157	0.277	0.571	0.690	136.981	-0.152	0.278	0.585	0.768	136.981	-0.309	0.322	0.337	0.572
AOC2	146.941	1.066	0.510	0.036	0.091	146.941	1.229	0.509	0.016	0.306	146.941	0.163	0.585	0.781	0.890
MR1	587.499	0.366	0.382	0.338	0.478	587.499	0.497	0.382	0.193	0.477	587.499	0.132	0.441	0.765	0.881
HLCS	471.886	-0.780	0.211	0.000	0.002	471.886	0.015	0.208	0.943	0.971	471.886	0.795	0.243	0.001	0.014
HLX	10.771	0.070	0.553	0.899	0.935	10.771	-0.437	0.574	0.446	0.677	10.771	-0.507	0.665	0.446	0.665
HMBS	814.836	-0.289	0.287	0.315	0.454	814.836	-0.358	0.287	0.213	0.493	814.836	-0.069	0.332	0.836	0.921
HMGB1	6873.138	-0.106	0.285	0.710	0.801	6873.138	-0.728	0.285	0.011	0.299	6873.138	-0.622	0.329	0.059	0.202
HMGB2	3967.118	0.476	0.248	0.055	0.124	3967.118	0.075	0.248	0.764	0.875	3967.118	-0.401	0.287	0.161	0.375
HMGB3	864.555	0.280	0.186	0.133	0.241	864.555	-0.236	0.187	0.206	0.488	864.555	-0.516	0.216	0.017	0.091
HMGN1	3576.469	0.024	0.113	0.830	0.888	3576.469	-0.142	0.113	0.209	0.490	3576.469	-0.166	0.130	0.203	0.426
HMGN2	7014.193	0.052	0.168	0.755	0.837	7014.193	-0.031	0.168	0.855	0.926	7014.193	-0.083	0.194	0.669	0.824
HMGCL	969.092	0.447	0.385	0.245	0.379	969.092	0.693	0.385	0.072	0.361	969.092	0.246	0.444	0.581	0.764
HMGCR	1531.235	1.545	0.221	0.000	0.000	1531.235	-0.186	0.222	0.402	0.645	1531.235	-1.731	0.256	0.000	0.000
HMGCS1	2016.868	3.817	0.227	0.000	0.000	2016.868	-0.617	0.232	0.008	0.297	2016.868	-4.434	0.265	0.000	0.000
HMGA1	10370.493	-0.318	0.201	0.115	0.217	10370.493	-0.237	0.201	0.240	0.516	10370.493	0.081	0.233	0.728	0.859
AOX1	2134.524	-0.406	0.172	0.018	0.054	2134.524	-0.088	0.172	0.607	0.783	2134.524	0.318	0.199	0.109	0.295
HMMR	1193.400	-0.776	0.170	0.000	0.000	1193.400	0.062	0.169	0.714	0.847	1193.400	0.837	0.196	0.000	0.001
HMOX1	8484.155	1.443	0.187	0.000	0.000	8484.155	-0.194	0.188	0.302	0.568	8484.155	-1.637	0.217	0.000	0.000
HMOX2	1379.846	0.007	0.178	0.967	0.980	1379.846	-0.188	0.178	0.289	0.557	1379.846	-0.196	0.205	0.340	0.575
NR4A1	310.347	0.148	0.272	0.585	0.702	310.347	-0.322	0.273	0.238	0.515	310.347	-0.470	0.315	0.136	0.337
FOXA1	64.542	-0.300	0.236	0.204	0.331	64.542	0.050	0.226	0.827	0.910	64.542	0.349	0.271	0.197	0.420
APAF1	417.230	-0.097	0.241	0.688	0.784	417.230	0.151	0.240	0.527	0.729	417.230	0.248	0.278	0.371	0.603
FOXA2	34.617	-1.155	0.768	0.132	0.241	34.617	-0.898	0.760	0.237	0.514	34.617	0.257	0.891	0.773	0.885
HNF4A	9.554	0.743	0.481	0.122	0.228	9.554	0.268	0.494	0.588	0.769	9.554	-0.475	0.556	0.394	0.621
HNF4G	102.329	-0.515	0.258	0.046	0.108	102.329	-0.149	0.253	0.555	0.746	102.329	0.366	0.298	0.219	0.446
NOP14-AS1	830.518	-0.643	0.249	0.010	0.034	830.518	-0.430	0.249	0.084	0.376	830.518	0.213	0.288	0.460	0.677
EIF4E3	433.689	0.028	0.293	0.924	0.952	433.689	0.364	0.293	0.213	0.493	433.689	0.337	0.338	0.320	0.555
FAM149B1	664.876	-0.357	0.217	0.100	0.195	664.876	-0.410	0.217	0.059	0.349	664.876	-0.052	0.251	0.835	0.920

RFESD	26.695	-0.401	0.360	0.266	0.402	26.695	0.044	0.343	0.899	0.949	26.695	0.445	0.413	0.281	0.516
SLC29A2	488.452	0.627	0.136	0.000	0.000	488.452	0.112	0.137	0.412	0.651	488.452	-0.515	0.157	0.001	0.014
MESTIT1	23.960	-0.698	0.417	0.094	0.188	23.960	-0.101	0.397	0.799	0.896	23.960	0.597	0.479	0.212	0.438
CCDC85C	1721.011	-0.193	0.172	0.260	0.396	1721.011	-0.351	0.172	0.041	0.329	1721.011	-0.158	0.199	0.428	0.651
DDX51	449.278	-0.566	0.318	0.075	0.158	449.278	-0.557	0.318	0.080	0.371	449.278	0.009	0.368	0.980	0.990
HNRNPA1	48942.401	0.115	0.209	0.581	0.698	48942.401	0.215	0.209	0.303	0.569	48942.401	0.100	0.242	0.679	0.830
NUDT2	304.272	0.169	0.221	0.445	0.580	304.272	0.509	0.219	0.020	0.310	304.272	0.340	0.254	0.181	0.399
HNRNPA2B1	21480.567	-0.131	0.239	0.583	0.700	21480.567	-0.538	0.239	0.024	0.316	21480.567	-0.407	0.276	0.141	0.345
HNRNPAB	5825.463	-0.316	0.408	0.439	0.575	5825.463	-1.071	0.408	0.009	0.297	5825.463	-0.755	0.471	0.109	0.295
HNRNPC	13050.370	0.095	0.207	0.647	0.753	13050.370	-0.320	0.207	0.121	0.413	13050.370	-0.415	0.239	0.082	0.248
HNRNPD	6594.104	-0.262	0.259	0.311	0.451	6594.104	-0.499	0.259	0.054	0.342	6594.104	-0.237	0.299	0.428	0.651
HNRNPF	4710.035	-0.383	0.182	0.036	0.090	4710.035	-0.248	0.182	0.174	0.463	4710.035	0.135	0.211	0.522	0.724
HNRNPH1	5456.722	-0.843	0.173	0.000	0.000	5456.722	-0.103	0.172	0.551	0.744	5456.722	0.740	0.199	0.000	0.004
HNRNPH2	1320.355	-0.145	0.149	0.331	0.471	1320.355	-0.370	0.149	0.013	0.305	1320.355	-0.225	0.173	0.193	0.415
HNRNPH3	4025.125	-0.319	0.118	0.007	0.026	4025.125	-0.164	0.118	0.164	0.454	4025.125	0.155	0.137	0.257	0.491
HNRNPK	15930.846	0.053	0.065	0.422	0.558	15930.846	-0.111	0.065	0.090	0.384	15930.846	-0.163	0.075	0.031	0.133
HNRNPL	7909.671	-0.286	0.216	0.186	0.310	7909.671	-0.346	0.216	0.109	0.401	7909.671	-0.060	0.249	0.810	0.905
HNRNPU	12372.597	-0.736	0.267	0.006	0.022	12372.597	-0.729	0.267	0.006	0.297	12372.597	0.007	0.308	0.982	0.990
HOXA1	38.436	0.215	0.357	0.548	0.671	38.436	0.325	0.353	0.357	0.611	38.436	0.110	0.410	0.788	0.894
ACACB	127.302	0.444	0.397	0.264	0.400	127.302	0.401	0.397	0.312	0.575	127.302	-0.042	0.458	0.927	0.967
HOXA3	79.421	0.139	0.196	0.476	0.609	79.421	0.400	0.189	0.035	0.322	79.421	0.261	0.222	0.241	0.472
HOXA4	78.129	1.004	0.428	0.019	0.055	78.129	0.508	0.431	0.239	0.516	78.129	-0.496	0.492	0.313	0.549
HOXA5	76.677	0.721	0.500	0.150	0.264	76.677	0.668	0.500	0.181	0.469	76.677	-0.053	0.575	0.927	0.967
HOXA6	21.021	-0.583	0.508	0.251	0.385	21.021	0.751	0.468	0.109	0.401	21.021	1.334	0.569	0.019	0.099
HOXA7	11.780	0.747	0.476	0.117	0.220	11.780	0.552	0.477	0.247	0.522	11.780	-0.195	0.541	0.719	0.854
HOXA9	52.687	0.066	0.270	0.808	0.874	52.687	0.237	0.264	0.369	0.619	52.687	0.172	0.310	0.580	0.764
HOXA10	299.106	0.033	0.142	0.817	0.880	299.106	0.041	0.141	0.770	0.878	299.106	0.008	0.164	0.960	0.981
HOXA11	19.514	0.454	0.387	0.241	0.374	19.514	-0.012	0.397	0.975	0.986	19.514	-0.466	0.454	0.304	0.540
HPCA	65.767	1.757	0.271	0.000	0.000	65.767	0.070	0.289	0.810	0.900	65.767	-1.687	0.319	0.000	0.000
HOXA13	91.957	0.676	0.483	0.162	0.281	91.957	0.600	0.483	0.214	0.493	91.957	-0.076	0.557	0.891	0.948
APBA2	1410.306	0.336	0.135	0.013	0.041	1410.306	-0.158	0.135	0.242	0.518	1410.306	-0.494	0.156	0.002	0.018
HOXB3	45.958	0.401	0.290	0.167	0.287	45.958	0.175	0.291	0.547	0.741	45.958	-0.225	0.335	0.501	0.708
HOXB6	46.071	0.726	0.350	0.038	0.094	46.071	0.323	0.353	0.361	0.613	46.071	-0.403	0.404	0.318	0.554
HOXB7	38.714	0.981	0.328	0.003	0.013	38.714	0.278	0.338	0.411	0.651	38.714	-0.703	0.381	0.065	0.214
HOXB8	22.990	0.501	0.311	0.108	0.207	22.990	0.235	0.314	0.454	0.681	22.990	-0.266	0.359	0.459	0.677
HOXB9	96.572	0.379	0.243	0.118	0.221	96.572	0.082	0.244	0.736	0.859	96.572	-0.297	0.281	0.291	0.526
APBB1	28.794	0.790	0.391	0.043	0.103	28.794	0.844	0.387	0.029	0.322	28.794	0.054	0.443	0.902	0.954
HOXC4	220.250	-0.172	0.181	0.344	0.485	220.250	0.075	0.179	0.676	0.825	220.250	0.246	0.209	0.239	0.469
HOXC5	19.867	0.467	0.454	0.304	0.443	19.867	-0.009	0.462	0.985	0.991	19.867	-0.476	0.530	0.369	0.601
HOXC6	218.720	0.733	0.243	0.003	0.012	218.720	-0.078	0.246	0.753	0.868	218.720	-0.810	0.282	0.004	0.036
HOXC8	147.579	-0.480	0.338	0.155	0.271	147.579	-0.798	0.338	0.018	0.306	147.579	-0.317	0.393	0.419	0.643
HOXC9	120.189	0.730	0.300	0.015	0.046	120.189	0.202	0.303	0.504	0.715	120.189	-0.528	0.347	0.129	0.325
HOXC10	1392.691	0.066	0.311	0.831	0.888	1392.691	0.482	0.310	0.120	0.412	1392.691	0.416	0.359	0.246	0.477
HOXC11	89.079	0.011	0.237	0.962	0.977	89.079	-0.081	0.235	0.731	0.856	89.079	-0.092	0.275	0.737	0.865
HOXC13	396.115	0.175	0.149	0.240	0.374	396.115	-0.244	0.150	0.104	0.397	396.115	-0.419	0.173	0.016	0.088
APBB2	828.212	-1.215	0.224	0.000	0.000	828.212	-0.368	0.223	0.098	0.393	828.212	0.847	0.259	0.001	0.014
HOXD8	69.856	0.810	0.246	0.001	0.006	69.856	0.201	0.251	0.422	0.659	69.856	-0.609	0.285	0.033	0.140
HOXD9	17.686	2.636	0.776	0.001	0.004	17.686	1.090	0.802	0.174	0.463	17.686	-1.546	0.882	0.080	0.244
HOXD10	33.905	1.832	0.610	0.003	0.012	33.905	0.835	0.618	0.177	0.466	33.905	-0.997	0.700	0.154	0.364
HOXD11	35.624	1.135	0.592	0.055	0.124	35.624	0.834	0.594	0.160	0.451	35.624	-0.301	0.678	0.657	0.816
APC	399.911	-0.975	0.258	0.000	0.001	399.911	-0.214	0.256	0.403	0.645	399.911	0.761	0.298	0.011	0.069
HP	18.095	5.629	0.837	0.000	0.000	18.095	2.505	0.897	0.005	0.297	18.095	-3.124	0.810	0.000	0.003
HPCAL1	4722.107	0.050	0.193	0.796	0.865	4722.107	0.240	0.192	0.212	0.492	4722.107	0.190	0.222	0.391	0.620
HPD	11.970	-0.491	0.664	0.460	0.594	11.970	0.116	0.641	0.857	0.927	11.970	0.607	0.761	0.425	0.649
HPGD	27.937	-0.037	0.349	0.916	0.947	27.937	0.342	0.331	0.301	0.568	27.937	0.379	0.395	0.338	0.572
HPRT1	987.402	0.241	0.242	0.318	0.458	987.402	-0.342	0.242	0.158	0.450	987.402	-0.584	0.280	0.037	0.151
HPS1	1207.570	0.519	0.127	0.000	0.000	1207.570	0.146	0.128	0.254	0.527	1207.570	-0.374	0.147	0.011	0.070
HRAS	2153.091	0.453	0.289	0.117	0.220	2153.091	-0.344	0.289	0.234	0.512	2153.091	-0.797	0.334	0.017	0.092
MMAB	614.115	1.214	0.175	0.000	0.000	614.115	0.304	0.177	0.085	0.378	614.115	-0.911	0.203	0.000	0.000
AGFG1	1208.822	-0.558	0.236	0.018	0.053	1208.822	-0.347	0.235	0.141	0.431	1208.822	0.211	0.272	0.438	0.659
AGFG2	1093.088	-0.095	0.153	0.535	0.660	1093.088	0.241	0.153	0.114	0.406	1093.088	0.336	0.177	0.057	0.199
HRH1	586.041	-0.010	0.342	0.977	0.986	586.041	-0.694	0.343	0.043	0.331	586.041	-0.684	0.396	0.084	0.252
APEH	2567.406	-0.487	0.199	0.014	0.044	2567.406	-0.275	0.199	0.166	0.456	2567.406	0.212	0.230	0.356	0.590
PRMT2	1372.790	0.259	0.345	0.452	0.587	1372.790	0.514	0.345	0.136	0.427	1372.790	0.255	0.398	0.522	0.724
PRMT1	5620.135	-0.247	0.227	0.277	0.414	5620.135	-0.300	0.227	0.187	0.473	5620.135	-0.053	0.263	0.840	0.923
APEX1	4556.475	-0.663	0.118	0.000	0.000	4556.475	-0.081	0.117	0.489	0.705	4556.475	0.582	0.136	0.000	0.001
HES1	1450.159	1.297	0.301	0.000	0.000	1450.159	0.335	0.302	0.267	0.538	1450.159	-0.962	0.348	0.006	0.045

HSBP1	2157.496	0.328	0.111	0.003	0.014	2157.496	-0.026	0.111	0.816	0.904	2157.496	-0.354	0.128	0.006	0.046
BIRC2	1242.849	-0.480	0.167	0.004	0.017	1242.849	-0.281	0.166	0.091	0.385	1242.849	0.199	0.193	0.302	0.537
HSD11B1	298.491	2.886	0.242	0.000	0.000	298.491	1.376	0.255	0.000	0.001	298.491	-1.510	0.266	0.000	0.000
HSD17B1	609.298	-0.277	0.128	0.031	0.080	609.298	-0.129	0.127	0.310	0.574	609.298	0.147	0.148	0.319	0.554
HSD17B4	3147.717	0.054	0.205	0.792	0.862	3147.717	0.176	0.205	0.390	0.635	3147.717	0.122	0.237	0.607	0.783
HSF1	1875.439	-0.023	0.185	0.903	0.938	1875.439	-0.166	0.185	0.369	0.619	1875.439	-0.143	0.213	0.502	0.708
HSF2	310.620	-0.264	0.137	0.054	0.122	310.620	0.043	0.134	0.750	0.867	310.620	0.307	0.158	0.052	0.187
HSF4	94.351	2.270	0.511	0.000	0.000	94.351	1.358	0.513	0.008	0.297	94.351	-0.912	0.586	0.120	0.311
ACADL	32.267	0.626	0.695	0.368	0.508	32.267	0.420	0.695	0.546	0.740	32.267	-0.206	0.801	0.797	0.898
BIRC3	6399.766	0.239	0.188	0.204	0.331	6399.766	-0.126	0.188	0.503	0.715	6399.766	-0.366	0.218	0.093	0.268
DNAJB2	1150.009	0.877	0.300	0.003	0.015	1150.009	0.570	0.300	0.057	0.347	1150.009	-0.307	0.346	0.376	0.607
DNAJA1	2716.301	0.287	0.209	0.170	0.290	2716.301	-0.650	0.210	0.002	0.297	2716.301	-0.937	0.242	0.000	0.003
HSPA1A	4328.338	1.440	0.149	0.000	0.000	4328.338	0.097	0.149	0.514	0.720	4328.338	-1.343	0.172	0.000	0.000
HSPA1B	3767.125	1.272	0.134	0.000	0.000	3767.125	0.066	0.135	0.626	0.794	3767.125	-1.207	0.155	0.000	0.000
HSPA1L	42.870	0.868	0.446	0.052	0.118	42.870	0.173	0.454	0.703	0.840	42.870	-0.694	0.517	0.179	0.398
HSPA2	35.829	1.609	0.410	0.000	0.001	35.829	-0.236	0.437	0.590	0.771	35.829	-1.845	0.487	0.000	0.003
HSPA4	3794.772	-0.499	0.243	0.040	0.097	3794.772	-0.667	0.243	0.006	0.297	3794.772	-0.169	0.280	0.547	0.742
HSPA5	10472.772	-0.470	0.324	0.147	0.260	10472.772	-0.403	0.324	0.214	0.493	10472.772	0.068	0.374	0.857	0.931
XIAP	711.628	-0.229	0.165	0.165	0.284	711.628	-0.303	0.165	0.065	0.354	711.628	-0.074	0.191	0.697	0.841
HSPA6	14.978	2.112	0.612	0.001	0.004	14.978	-0.321	0.720	0.656	0.813	14.978	-2.433	0.763	0.001	0.017
HSPA8	23354.315	-0.401	0.322	0.213	0.342	23354.315	0.377	0.322	0.242	0.518	23354.315	0.778	0.372	0.036	0.149
HSPA9	9744.375	-1.079	0.242	0.000	0.000	9744.375	-0.469	0.241	0.052	0.341	9744.375	0.610	0.279	0.029	0.128
HSPB1	5916.847	1.107	0.333	0.001	0.005	5916.847	0.325	0.333	0.330	0.590	5916.847	-0.782	0.385	0.042	0.164
BIRC5	2000.823	-0.495	0.235	0.035	0.089	2000.823	-0.072	0.235	0.759	0.872	2000.823	0.423	0.272	0.119	0.310
HSP90AA1	18343.477	-0.143	0.385	0.711	0.802	18343.477	-1.233	0.385	0.001	0.297	18343.477	-1.090	0.444	0.014	0.082
IGSF3	578.569	-0.330	0.270	0.223	0.353	578.569	0.142	0.269	0.598	0.777	578.569	0.472	0.312	0.131	0.328
HSP90AB1	45716.734	-0.172	0.099	0.082	0.169	45716.734	0.112	0.099	0.255	0.529	45716.734	0.284	0.114	0.013	0.077
HSPD1	14165.942	-0.573	0.152	0.000	0.001	14165.942	-0.319	0.152	0.036	0.324	14165.942	0.255	0.175	0.146	0.353
APLP1	24.509	1.167	0.427	0.006	0.023	24.509	0.705	0.435	0.105	0.397	24.509	-0.462	0.483	0.339	0.574
HSPE1	1144.138	0.600	0.340	0.078	0.161	1144.138	-0.756	0.341	0.026	0.318	1144.138	-1.355	0.393	0.001	0.009
DNAJB1	2693.718	0.029	0.132	0.827	0.886	2693.718	0.051	0.131	0.699	0.838	2693.718	0.022	0.152	0.884	0.945
DNAJC4	282.239	0.868	0.476	0.068	0.147	282.239	0.696	0.476	0.144	0.435	282.239	-0.172	0.549	0.754	0.874
HSPG2	9253.848	0.448	0.458	0.328	0.468	9253.848	0.696	0.458	0.129	0.420	9253.848	0.248	0.529	0.639	0.804
PPM1J	18.618	0.527	0.401	0.189	0.313	18.618	0.170	0.407	0.677	0.825	18.618	-0.357	0.465	0.443	0.663
APLP2	7449.388	0.002	0.137	0.989	0.994	7449.388	0.096	0.137	0.483	0.701	7449.388	0.094	0.158	0.551	0.745
NDST1	1534.111	-0.237	0.204	0.245	0.379	1534.111	-0.052	0.204	0.798	0.895	1534.111	0.185	0.236	0.433	0.655
FOXN2	360.470	-0.662	0.242	0.006	0.023	360.470	-0.279	0.240	0.244	0.519	360.470	0.383	0.279	0.170	0.386
HTR1D	8.892	1.271	0.573	0.027	0.071	8.892	0.787	0.583	0.177	0.466	8.892	-0.484	0.648	0.455	0.673
HTR2C	55.241	-0.966	0.504	0.056	0.125	55.241	0.466	0.488	0.339	0.597	55.241	1.432	0.577	0.013	0.078
HTR3A	18.942	3.245	0.755	0.000	0.000	18.942	1.918	0.790	0.015	0.306	18.942	-1.327	0.790	0.093	0.268
HTR7	51.876	-3.665	0.817	0.000	0.000	51.876	-1.895	0.756	0.012	0.305	51.876	1.770	0.936	0.059	0.202
HUS1	220.569	-0.118	0.196	0.547	0.671	220.569	-0.079	0.194	0.686	0.831	220.569	0.039	0.226	0.862	0.934
TNC	3373.346	-0.030	0.281	0.915	0.946	3373.346	-0.664	0.281	0.018	0.306	3373.346	-0.635	0.324	0.050	0.184
IARS1	5890.079	-1.401	0.275	0.000	0.000	5890.079	-0.277	0.275	0.313	0.576	5890.079	1.123	0.318	0.000	0.007
UBAC2	1042.013	0.113	0.144	0.433	0.569	1042.013	0.164	0.144	0.254	0.528	1042.013	0.051	0.166	0.760	0.878
H2BC20P	214.894	0.401	0.502	0.425	0.562	214.894	0.483	0.502	0.336	0.595	214.894	0.082	0.580	0.887	0.946
CHSY3	74.020	-0.070	0.227	0.758	0.839	74.020	-0.197	0.226	0.382	0.629	74.020	-0.127	0.264	0.629	0.798
FAM151A	28.790	0.797	0.510	0.118	0.221	28.790	0.511	0.511	0.318	0.580	28.790	-0.286	0.587	0.626	0.795
ICA1	59.680	0.695	0.315	0.028	0.073	59.680	0.659	0.314	0.036	0.324	59.680	-0.036	0.360	0.920	0.963
ICAM1	4357.152	0.260	0.236	0.270	0.407	4357.152	-0.212	0.236	0.368	0.618	4357.152	-0.472	0.272	0.083	0.250
RAB7B	35.122	-0.518	0.874	0.554	0.675	35.122	0.282	0.867	0.745	0.864	35.122	0.800	1.007	0.427	0.650
ANO9	22.426	1.189	0.461	0.010	0.034	22.426	-0.119	0.488	0.808	0.899	22.426	-1.307	0.546	0.017	0.091
HCAR2	80.223	2.859	0.628	0.000	0.000	80.223	1.169	0.637	0.066	0.354	80.223	-1.689	0.721	0.019	0.099
ICAM3	232.880	0.344	0.201	0.087	0.176	232.880	0.185	0.201	0.357	0.611	232.880	-0.159	0.232	0.493	0.702
ICAM4	12.453	1.779	0.556	0.001	0.007	12.453	-2.521	1.138	0.027	0.319	12.453	-4.299	1.154	0.000	0.004
KRTAP5-AS1	11.590	1.637	0.661	0.013	0.042	11.590	1.181	0.671	0.079	0.370	11.590	-0.456	0.733	0.533	0.732
CCDC84	2133.327	1.048	0.375	0.005	0.021	2133.327	0.518	0.376	0.168	0.457	2133.327	-0.530	0.434	0.222	0.449
VSIG10L2	55.797	2.301	0.565	0.000	0.001	55.797	0.805	0.572	0.159	0.451	55.797	-1.496	0.651	0.021	0.106
ANKRD13D	411.935	0.752	0.206	0.000	0.002	411.935	0.122	0.208	0.556	0.747	411.935	-0.629	0.239	0.008	0.058
ANKRD42	173.454	-0.031	0.182	0.864	0.911	173.454	0.124	0.179	0.487	0.704	173.454	0.155	0.209	0.458	0.675
B4GALNT4	537.142	0.917	0.343	0.008	0.027	537.142	0.640	0.343	0.062	0.350	537.142	-0.276	0.396	0.485	0.697
ATP2B1-AS1	29.178	0.132	0.344	0.701	0.794	29.178	-0.757	0.366	0.039	0.327	29.178	-0.889	0.419	0.034	0.143
C1QL4	9.838	1.402	0.550	0.011	0.036	9.838	0.956	0.558	0.086	0.379	9.838	-0.446	0.617	0.470	0.684
LINC01089	53.458	0.568	0.456	0.213	0.342	53.458	0.848	0.453	0.061	0.350	53.458	0.280	0.522	0.592	0.772
TAFA2	37.301	-0.226	0.360	0.531	0.656	37.301	-0.090	0.355	0.800	0.896	37.301	0.136	0.417	0.744	0.869
WHAMMP3	135.168	-0.031	0.289	0.915	0.946	135.168	-0.044	0.287	0.878	0.939	135.168	-0.013	0.334	0.969	0.985
PKD1P1	2726.529	-0.248	0.329	0.452	0.587	2726.529	0.367	0.329	0.265	0.537	2726.529	0.614	0.380	0.106	0.290

PRSS53	166.891	1.584	0.440	0.000	0.002	166.891	0.836	0.442	0.059	0.349	166.891	-0.749	0.506	0.139	0.343
RAB43	97.174	0.040	0.351	0.909	0.942	97.174	0.027	0.349	0.938	0.968	97.174	-0.013	0.405	0.975	0.987
JMJD8	2064.682	0.230	0.201	0.253	0.387	2064.682	-0.129	0.202	0.522	0.726	2064.682	-0.359	0.233	0.123	0.315
METTL2A	531.694	-0.417	0.268	0.119	0.222	531.694	-0.511	0.267	0.056	0.344	531.694	-0.094	0.310	0.762	0.879
LOC339192	60.512	-0.025	0.373	0.946	0.967	60.512	0.620	0.365	0.089	0.382	60.512	0.646	0.426	0.130	0.327
ASB16-AS1	48.033	0.487	0.270	0.071	0.151	48.033	0.329	0.269	0.222	0.501	48.033	-0.158	0.310	0.611	0.785
C17orf67	44.131	0.282	0.328	0.390	0.530	44.131	-0.282	0.335	0.401	0.644	44.131	-0.564	0.386	0.144	0.350
OXLD1	442.932	0.369	0.270	0.172	0.292	442.932	-0.018	0.270	0.948	0.974	442.932	-0.387	0.312	0.216	0.441
CCDC137	547.869	-0.079	0.314	0.801	0.869	547.869	-0.551	0.314	0.079	0.370	547.869	-0.472	0.363	0.193	0.416
ARL16	200.082	0.073	0.174	0.677	0.775	200.082	0.045	0.173	0.796	0.894	200.082	-0.028	0.201	0.890	0.947
LOC339260	291.142	-0.865	0.311	0.005	0.021	291.142	-0.094	0.308	0.761	0.873	291.142	0.771	0.359	0.032	0.136
LINC02693	207.927	-1.747	0.478	0.000	0.002	207.927	-1.399	0.476	0.003	0.297	207.927	0.348	0.554	0.530	0.729
MSL1	2057.218	-0.448	0.178	0.012	0.039	2057.218	0.187	0.178	0.293	0.561	2057.218	0.634	0.206	0.002	0.022
LINC00667	255.904	-0.006	0.143	0.966	0.979	255.904	-0.002	0.141	0.991	0.995	255.904	0.005	0.165	0.978	0.989
ZNF181	109.705	0.078	0.203	0.702	0.795	109.705	0.018	0.202	0.928	0.964	109.705	-0.059	0.235	0.800	0.900
ZNF260	251.293	-0.365	0.177	0.039	0.096	251.293	-0.144	0.175	0.410	0.650	251.293	0.221	0.205	0.281	0.515
ZNF546	58.207	0.150	0.344	0.663	0.765	58.207	0.489	0.338	0.148	0.441	58.207	0.339	0.394	0.389	0.617
MYPOP	174.916	0.320	0.189	0.090	0.181	174.916	0.362	0.187	0.053	0.342	174.916	0.042	0.217	0.847	0.926
ADAMTSL5	114.637	0.760	0.248	0.002	0.010	114.637	0.409	0.249	0.101	0.394	114.637	-0.352	0.285	0.218	0.444
C1orf174	507.058	-0.699	0.307	0.023	0.063	507.058	-0.634	0.306	0.038	0.327	507.058	0.066	0.355	0.853	0.930
KLHL17	298.889	0.659	0.242	0.007	0.024	298.889	0.344	0.243	0.156	0.448	298.889	-0.315	0.279	0.260	0.494
TMEM240	34.240	1.295	0.511	0.011	0.037	34.240	0.923	0.514	0.073	0.362	34.240	-0.372	0.583	0.523	0.725
TMEM52	23.396	-0.302	0.640	0.638	0.745	23.396	0.245	0.625	0.695	0.836	23.396	0.546	0.734	0.457	0.675
ZBTB8OS	252.056	0.495	0.266	0.062	0.137	252.056	-0.505	0.269	0.061	0.350	252.056	-1.001	0.310	0.001	0.016
TFAP2E	13.565	2.347	0.729	0.001	0.007	13.565	0.310	0.779	0.691	0.833	13.565	-2.037	0.852	0.017	0.092
ZNF678	46.257	-0.622	0.305	0.041	0.100	46.257	-0.011	0.289	0.971	0.984	46.257	0.612	0.349	0.080	0.244
ZFP69	86.154	-0.377	0.219	0.085	0.173	86.154	-0.405	0.217	0.062	0.350	86.154	-0.027	0.256	0.915	0.960
MRPL58	420.391	-0.039	0.234	0.867	0.914	420.391	-0.261	0.234	0.265	0.537	420.391	-0.222	0.271	0.413	0.638
SLC35E4	304.362	-0.073	0.323	0.820	0.882	304.362	-0.670	0.325	0.039	0.327	304.362	-0.597	0.375	0.112	0.298
ID1	4495.133	0.911	0.279	0.001	0.006	4495.133	-0.251	0.279	0.367	0.618	4495.133	-1.162	0.322	0.000	0.006
SPOPL	316.047	-0.996	0.329	0.002	0.011	316.047	-0.583	0.327	0.075	0.364	316.047	0.413	0.380	0.277	0.512
MAP3K20-AS1	113.446	-1.124	0.390	0.004	0.016	113.446	-0.139	0.382	0.715	0.847	113.446	0.984	0.448	0.028	0.126
LINC00299	5.477	3.504	1.023	0.001	0.004	5.477	1.643	1.120	0.143	0.434	5.477	-1.861	1.055	0.078	0.241
ID2	1026.390	2.307	0.311	0.000	0.000	1026.390	0.372	0.313	0.234	0.512	1026.390	-1.935	0.360	0.000	0.000
LOC339803	178.194	-0.274	0.204	0.180	0.303	178.194	0.217	0.200	0.277	0.548	178.194	0.491	0.235	0.036	0.149
C2orf74	146.102	0.483	0.296	0.103	0.200	146.102	-0.046	0.298	0.877	0.938	146.102	-0.529	0.344	0.123	0.316
CCDC39	36.336	0.209	0.309	0.500	0.630	36.336	-0.123	0.313	0.693	0.835	36.336	-0.332	0.362	0.359	0.591
ID3	4683.317	0.671	0.312	0.031	0.081	4683.317	-0.470	0.312	0.132	0.424	4683.317	-1.141	0.360	0.002	0.018
EHHADH-AS1	17.620	0.181	0.386	0.640	0.747	17.620	-0.235	0.394	0.550	0.743	17.620	-0.416	0.456	0.361	0.594
LPP-AS2	24.207	-0.263	0.366	0.472	0.606	24.207	-0.508	0.368	0.167	0.457	24.207	-0.245	0.433	0.571	0.759
H1FX-AS1	2721.338	0.474	0.255	0.063	0.138	2721.338	0.132	0.255	0.603	0.781	2721.338	-0.341	0.295	0.247	0.478
NAT8L	795.688	-0.212	0.312	0.496	0.628	795.688	-0.586	0.313	0.061	0.350	795.688	-0.373	0.361	0.302	0.537
ACADM	875.304	-0.707	0.199	0.000	0.003	875.304	-0.298	0.198	0.132	0.424	875.304	0.409	0.230	0.075	0.235
PRR7-AS1	12.108	0.265	0.499	0.595	0.710	12.108	0.007	0.503	0.988	0.993	12.108	-0.258	0.582	0.658	0.817
STING1	487.960	1.782	0.459	0.000	0.001	487.960	0.729	0.460	0.113	0.405	487.960	-1.052	0.530	0.047	0.175
MYLK4	9.626	0.226	0.700	0.747	0.831	9.626	0.519	0.683	0.447	0.677	9.626	0.294	0.797	0.713	0.850
IFITM4P	26.419	0.307	0.731	0.674	0.773	26.419	1.373	0.715	0.055	0.342	26.419	1.066	0.830	0.199	0.422
TREML3P	8.945	-1.471	1.263	0.244	0.378	8.945	0.570	1.124	0.612	0.786	8.945	2.040	1.413	0.149	0.357
ZNF680	46.582	0.362	0.254	0.155	0.270	46.582	-0.157	0.260	0.546	0.740	46.582	-0.519	0.299	0.083	0.249
COL28A1	258.874	-0.898	0.799	0.261	0.397	258.874	0.810	0.797	0.309	0.574	258.874	1.709	0.922	0.064	0.213
CTAGE6	15.348	0.842	0.479	0.079	0.163	15.348	0.616	0.480	0.200	0.483	15.348	-0.226	0.544	0.678	0.829
AGBL3	12.203	-0.829	0.478	0.083	0.170	12.203	-0.150	0.433	0.729	0.855	12.203	0.678	0.546	0.214	0.439
NRBP2	363.916	0.394	0.238	0.098	0.193	363.916	0.135	0.238	0.570	0.756	363.916	-0.259	0.275	0.347	0.581
ZNF517	98.524	0.955	0.281	0.001	0.004	98.524	0.528	0.284	0.063	0.350	98.524	-0.427	0.324	0.187	0.407
RSPO2	17.170	0.465	0.805	0.564	0.685	17.170	0.749	0.795	0.346	0.602	17.170	0.284	0.919	0.757	0.876
ZDHHC21	95.711	-0.404	0.265	0.128	0.235	95.711	-0.300	0.262	0.253	0.527	95.711	0.104	0.308	0.736	0.864
ACER2	18.225	1.661	0.544	0.002	0.011	18.225	0.074	0.576	0.897	0.948	18.225	-1.587	0.640	0.013	0.078
MORF4L2-AS1	17.033	-0.477	0.560	0.394	0.533	17.033	0.484	0.525	0.357	0.611	17.033	0.962	0.633	0.129	0.325
SATL1	57.444	-0.323	0.262	0.218	0.348	57.444	0.130	0.251	0.605	0.782	57.444	0.452	0.300	0.131	0.329
CA5BP1	459.971	0.047	0.165	0.775	0.850	459.971	-0.032	0.164	0.846	0.921	459.971	-0.079	0.190	0.678	0.830
VWA2	26.901	0.726	0.372	0.051	0.117	26.901	0.739	0.368	0.045	0.333	26.901	0.012	0.420	0.977	0.988
NANOS1	68.174	-0.265	0.473	0.575	0.693	68.174	0.166	0.467	0.723	0.852	68.174	0.431	0.544	0.429	0.651
APOC1	309.350	1.848	0.462	0.000	0.001	309.350	0.681	0.464	0.142	0.433	309.350	-1.166	0.533	0.029	0.128
IDE	731.806	-0.719	0.288	0.013	0.040	731.806	-0.646	0.287	0.025	0.316	731.806	0.072	0.333	0.828	0.915
IDH1	1211.508	0.628	0.249	0.012	0.038	1211.508	0.222	0.249	0.373	0.621	1211.508	-0.406	0.287	0.157	0.369
IDH2	1133.609	0.723	0.219	0.001	0.006	1133.609	0.250	0.219	0.254	0.527	1133.609	-0.473	0.253	0.062	0.209
IDH3A	1127.074	-0.728	0.284	0.010	0.035	1127.074	-0.702	0.283	0.013	0.305	1127.074	0.026	0.328	0.937	0.972

IDH3B	1133.196	-0.201	0.127	0.113	0.215	1133.196	-0.021	0.126	0.867	0.932	1133.196	0.180	0.146	0.220	0.446
GLDN	43.803	-0.368	0.745	0.621	0.732	43.803	0.854	0.736	0.246	0.521	43.803	1.222	0.856	0.153	0.363
IDH3G	1030.875	0.542	0.190	0.004	0.018	1030.875	0.256	0.190	0.179	0.467	1030.875	-0.285	0.220	0.194	0.416
ZNF774	20.700	1.251	0.367	0.001	0.004	20.700	0.483	0.384	0.209	0.490	20.700	-0.769	0.422	0.068	0.222
IDI1	874.658	2.068	0.227	0.000	0.000	874.658	-0.626	0.231	0.007	0.297	874.658	-2.694	0.264	0.000	0.000
IDS	1400.877	0.150	0.278	0.591	0.706	1400.877	0.312	0.278	0.262	0.535	1400.877	0.162	0.321	0.614	0.787
ZKSCAN2	135.946	-0.723	0.180	0.000	0.001	135.946	-0.021	0.169	0.900	0.950	135.946	0.702	0.206	0.001	0.010
ATXN1L	670.628	-0.107	0.167	0.523	0.651	670.628	0.054	0.166	0.747	0.865	670.628	0.160	0.193	0.406	0.633
PKD1L3	12.963	0.495	0.670	0.460	0.595	12.963	0.239	0.673	0.723	0.852	12.963	-0.256	0.774	0.741	0.867
CFI	117.383	3.376	0.616	0.000	0.000	117.383	0.646	0.624	0.300	0.567	117.383	-2.730	0.712	0.000	0.003
STAC2	347.903	2.246	0.840	0.008	0.027	347.903	0.716	0.841	0.395	0.639	347.903	-1.530	0.970	0.115	0.303
IFI16	3409.599	0.215	0.113	0.057	0.128	3409.599	0.056	0.113	0.621	0.791	3409.599	-0.159	0.131	0.223	0.451
ZNF850	25.034	-0.240	0.356	0.500	0.631	25.034	0.538	0.331	0.104	0.397	25.034	0.778	0.399	0.052	0.186
IFI27	49.960	2.325	0.443	0.000	0.000	49.960	0.447	0.465	0.336	0.595	49.960	-1.878	0.515	0.000	0.005
ZNF404	13.432	1.321	0.766	0.084	0.172	13.432	-0.874	0.824	0.289	0.557	13.432	-2.196	0.925	0.018	0.094
ZNF284	77.259	0.342	0.303	0.259	0.395	77.259	0.367	0.301	0.223	0.502	77.259	0.025	0.348	0.943	0.974
RFPL4A	8.233	1.273	0.706	0.071	0.152	8.233	1.029	0.708	0.146	0.438	8.233	-0.244	0.795	0.759	0.877
ZSCAN22	86.795	-0.146	0.226	0.517	0.644	86.795	0.319	0.216	0.140	0.431	86.795	0.465	0.257	0.070	0.226
IFI35	272.322	0.671	0.221	0.002	0.011	272.322	-0.059	0.224	0.791	0.891	272.322	-0.730	0.257	0.004	0.038
CCDC18	300.589	-0.207	0.189	0.273	0.410	300.589	-0.426	0.189	0.024	0.316	300.589	-0.219	0.220	0.319	0.554
SP110	247.943	0.235	0.172	0.171	0.292	247.943	0.014	0.172	0.937	0.968	247.943	-0.221	0.199	0.266	0.500
OR2W3	53.301	0.091	0.326	0.779	0.853	53.301	-0.368	0.330	0.265	0.537	53.301	-0.460	0.382	0.229	0.457
IFIT2	629.783	3.289	0.710	0.000	0.000	629.783	1.128	0.712	0.113	0.405	629.783	-2.162	0.819	0.008	0.058
IFIT1	361.454	2.821	0.739	0.000	0.001	361.454	0.757	0.741	0.307	0.572	361.454	-2.063	0.853	0.016	0.087
IFIT3	868.401	3.881	0.436	0.000	0.000	868.401	0.865	0.439	0.049	0.337	868.401	-3.016	0.503	0.000	0.000
PRORSD1P	11.707	-0.262	0.544	0.630	0.739	11.707	0.438	0.509	0.389	0.635	11.707	0.700	0.613	0.254	0.487
SH3RF3	476.251	-0.196	0.163	0.228	0.359	476.251	0.198	0.161	0.218	0.497	476.251	0.395	0.187	0.035	0.146
DUBR	158.331	-0.396	0.270	0.143	0.254	158.331	-0.577	0.270	0.033	0.322	158.331	-0.181	0.314	0.565	0.755
COL6A4P1	10.222	-0.733	0.676	0.279	0.416	10.222	0.116	0.637	0.856	0.926	10.222	0.848	0.771	0.271	0.506
NMRAL2P	60.643	0.341	0.493	0.489	0.622	60.643	-0.952	0.507	0.060	0.350	60.643	-1.293	0.582	0.026	0.121
IFNAR1	1119.802	0.016	0.166	0.924	0.952	1119.802	0.101	0.166	0.542	0.738	1119.802	0.085	0.192	0.657	0.817
IFNAR2	385.543	0.728	0.154	0.000	0.000	385.543	-0.175	0.157	0.265	0.537	385.543	-0.903	0.179	0.000	0.000
FBLL1	8.625	1.100	0.696	0.114	0.216	8.625	0.374	0.719	0.603	0.781	8.625	-0.726	0.804	0.367	0.599
MCIDAS	62.379	-0.515	0.351	0.142	0.254	62.379	-0.803	0.352	0.023	0.315	62.379	-0.288	0.411	0.484	0.696
FAM174A	169.573	-0.080	0.184	0.664	0.766	169.573	0.147	0.180	0.413	0.652	169.573	0.227	0.211	0.282	0.516
MTX3	361.672	-0.774	0.179	0.000	0.000	361.672	-0.004	0.175	0.983	0.990	361.672	0.770	0.206	0.000	0.004
IFNGR1	3078.433	0.011	0.130	0.935	0.959	3078.433	-0.212	0.130	0.103	0.396	3078.433	-0.223	0.150	0.138	0.341
IFNGR2	1565.247	0.904	0.178	0.000	0.000	1565.247	0.113	0.179	0.528	0.729	1565.247	-0.791	0.206	0.000	0.003
EYS	31.046	-0.218	0.432	0.614	0.725	31.046	0.190	0.420	0.650	0.809	31.046	0.408	0.495	0.410	0.636
ZNF391	114.232	-0.461	0.283	0.103	0.200	114.232	0.238	0.276	0.390	0.635	114.232	0.699	0.325	0.031	0.135
ZFP57	43.736	0.679	0.548	0.215	0.345	43.736	-0.113	0.554	0.839	0.916	43.736	-0.792	0.636	0.213	0.438
MACC1	12.374	0.383	0.455	0.399	0.537	12.374	0.197	0.455	0.665	0.818	12.374	-0.186	0.525	0.722	0.856
KIF24	179.410	-0.484	0.345	0.161	0.280	179.410	-0.663	0.345	0.055	0.342	179.410	-0.179	0.400	0.654	0.815
CAVIN4	13.865	0.515	0.467	0.270	0.406	13.865	-0.615	0.532	0.247	0.522	13.865	-1.130	0.592	0.056	0.197
ZNF81	42.323	-0.541	0.377	0.151	0.265	42.323	-0.852	0.379	0.025	0.316	42.323	-0.311	0.444	0.484	0.696
IFRD1	1017.197	-0.682	0.249	0.006	0.023	1017.197	-0.463	0.248	0.063	0.350	1017.197	0.220	0.288	0.445	0.665
IGBP1	1312.170	0.243	0.348	0.485	0.617	1312.170	0.415	0.348	0.233	0.510	1312.170	0.172	0.401	0.669	0.824
TUBB2B	494.370	1.161	0.298	0.000	0.001	494.370	-0.885	0.302	0.003	0.297	494.370	-2.046	0.347	0.000	0.000
SLC35B2	1603.325	-0.119	0.146	0.415	0.552	1603.325	-0.002	0.145	0.991	0.995	1603.325	0.117	0.168	0.486	0.697
SERINC2	2805.555	1.329	0.287	0.000	0.000	2805.555	0.622	0.288	0.031	0.322	2805.555	-0.707	0.332	0.033	0.140
NME9	7.151	1.332	0.788	0.091	0.182	7.151	0.379	0.818	0.644	0.806	7.151	-0.954	0.912	0.296	0.531
GATD1	490.545	0.500	0.182	0.006	0.023	490.545	0.076	0.183	0.679	0.827	490.545	-0.424	0.211	0.044	0.169
AMIGO2	211.649	-1.174	0.592	0.047	0.111	211.649	-1.492	0.593	0.012	0.305	211.649	-0.318	0.686	0.643	0.806
EP400P1	89.331	0.097	0.369	0.792	0.863	89.331	-0.566	0.373	0.129	0.421	89.331	-0.663	0.430	0.123	0.316
APOE	195.609	1.947	1.046	0.063	0.137	195.609	0.252	1.048	0.810	0.900	195.609	-1.695	1.208	0.161	0.374
IGF1R	558.642	0.151	0.212	0.477	0.610	558.642	0.036	0.212	0.865	0.931	558.642	-0.115	0.245	0.639	0.804
TMEM255B	112.733	1.211	0.494	0.014	0.044	112.733	0.858	0.499	0.085	0.378	112.733	-0.352	0.555	0.526	0.726
ANKDD1A	53.657	1.688	0.300	0.000	0.000	53.657	0.519	0.313	0.097	0.392	53.657	-1.169	0.346	0.001	0.011
ARPIN	380.160	0.226	0.205	0.270	0.406	380.160	0.052	0.205	0.799	0.895	380.160	-0.174	0.237	0.463	0.679
CTU2	371.081	-0.099	0.388	0.798	0.867	371.081	-0.648	0.389	0.096	0.391	371.081	-0.549	0.450	0.222	0.450
IGF2R	4109.091	0.177	0.165	0.283	0.421	4109.091	0.073	0.165	0.660	0.814	4109.091	-0.105	0.191	0.584	0.766
SKA2	1139.617	-0.221	0.221	0.318	0.458	1139.617	-0.184	0.221	0.406	0.647	1139.617	0.037	0.255	0.884	0.945
MCRIP1	318.484	0.384	0.203	0.058	0.130	318.484	-0.043	0.204	0.832	0.913	318.484	-0.427	0.235	0.069	0.224
ZNF530	17.337	-1.129	0.498	0.023	0.065	17.337	-0.573	0.469	0.222	0.501	17.337	0.556	0.577	0.335	0.570
FAM131C	64.690	1.359	0.509	0.008	0.027	64.690	0.800	0.512	0.118	0.410	64.690	-0.559	0.584	0.338	0.573
IGFBP2	280.514	0.207	0.169	0.220	0.351	280.514	-0.348	0.170	0.041	0.329	280.514	-0.555	0.197	0.005	0.040
IGFBP3	635.209	1.035	0.742	0.163	0.282	635.209	0.655	0.743	0.378	0.625	635.209	-0.381	0.856	0.657	0.816

C22orf34	104.994	-0.763	0.176	0.000	0.000	104.994	0.097	0.160	0.543	0.739	104.994	0.860	0.200	0.000	0.001
GEN1	145.835	-0.406	0.259	0.118	0.221	145.835	-0.509	0.259	0.049	0.337	145.835	-0.103	0.301	0.733	0.862
IGFBP4	16955.445	0.435	0.322	0.177	0.299	16955.445	0.766	0.322	0.018	0.306	16955.445	0.330	0.372	0.375	0.607
FTCDNL1	14.562	0.028	0.414	0.947	0.967	14.562	0.213	0.400	0.595	0.775	14.562	0.185	0.474	0.696	0.841
WDR53	175.160	-0.213	0.348	0.540	0.665	175.160	-0.484	0.349	0.166	0.456	175.160	-0.270	0.404	0.503	0.710
IGFBP5	49.593	0.443	1.441	0.759	0.840	49.593	0.477	1.440	0.740	0.861	49.593	0.035	1.663	0.983	0.991
LNP1	47.293	-0.437	0.319	0.170	0.290	47.293	0.272	0.304	0.371	0.620	47.293	0.710	0.363	0.051	0.185
NPHP3-AS1	36.648	-0.500	0.534	0.349	0.489	36.648	0.325	0.518	0.531	0.731	36.648	0.825	0.611	0.177	0.394
IGFBP6	3017.171	1.124	0.362	0.002	0.010	3017.171	0.548	0.362	0.131	0.423	3017.171	-0.576	0.418	0.168	0.383
FAM86EP	56.415	-1.077	0.325	0.001	0.005	56.415	-0.459	0.311	0.140	0.431	56.415	0.618	0.376	0.100	0.280
NUP43	592.883	-0.553	0.244	0.023	0.064	592.883	-0.466	0.243	0.055	0.343	592.883	0.087	0.282	0.758	0.877
IGFBP7	541.345	1.952	0.269	0.000	0.000	541.345	-0.250	0.273	0.359	0.611	541.345	-2.202	0.313	0.000	0.000
ZNF713	14.862	1.072	0.533	0.044	0.105	14.862	0.618	0.540	0.252	0.526	14.862	-0.454	0.610	0.457	0.675
CCN1	1582.237	1.443	0.231	0.000	0.000	1582.237	-0.163	0.232	0.482	0.700	1582.237	-1.606	0.267	0.000	0.000
LINC00265	22.470	0.186	0.411	0.651	0.756	22.470	-0.132	0.414	0.750	0.867	22.470	-0.318	0.480	0.507	0.713
WDR86	146.438	0.083	0.233	0.723	0.812	146.438	0.052	0.232	0.822	0.907	146.438	-0.031	0.270	0.910	0.959
DPY19L2P2	20.048	-0.888	0.740	0.230	0.363	20.048	0.611	0.709	0.389	0.635	20.048	1.499	0.843	0.075	0.236
NMNAT3	102.451	0.342	0.397	0.389	0.528	102.451	0.574	0.395	0.146	0.438	102.451	0.233	0.457	0.611	0.785
RTN4RL2	162.657	1.090	0.520	0.036	0.090	162.657	0.944	0.520	0.069	0.358	162.657	-0.146	0.598	0.807	0.903
ACADS	161.458	0.455	0.345	0.187	0.312	161.458	0.551	0.344	0.109	0.401	161.458	0.096	0.398	0.810	0.904
IGHMBP2	326.747	-0.375	0.178	0.035	0.089	326.747	-0.242	0.177	0.172	0.460	326.747	0.134	0.206	0.517	0.721
APP	21342.732	-0.054	0.223	0.810	0.875	21342.732	0.226	0.223	0.311	0.575	21342.732	0.280	0.257	0.277	0.512
RBPJ	1522.654	-0.073	0.186	0.693	0.788	1522.654	0.119	0.185	0.520	0.725	1522.654	0.193	0.214	0.369	0.601
DNAAF3	51.325	0.433	0.311	0.164	0.283	51.325	0.490	0.308	0.111	0.405	51.325	0.057	0.356	0.873	0.939
CASTOR3	12.421	0.904	0.531	0.089	0.179	12.421	-0.131	0.563	0.816	0.904	12.421	-1.035	0.631	0.101	0.282
HLA-V	390.405	0.903	0.352	0.010	0.035	390.405	0.810	0.352	0.021	0.314	390.405	-0.093	0.406	0.818	0.910
C6orf58	9.652	-1.095	0.578	0.058	0.129	9.652	-1.120	0.568	0.049	0.337	9.652	-0.025	0.695	0.972	0.986
APRT	1740.170	0.368	0.217	0.090	0.181	1740.170	0.138	0.217	0.524	0.727	1740.170	-0.229	0.251	0.360	0.593
RAET1G	26.307	0.475	0.706	0.501	0.631	26.307	0.576	0.702	0.412	0.651	26.307	0.101	0.811	0.901	0.953
RILPL1	572.867	0.684	0.189	0.000	0.002	572.867	0.138	0.190	0.469	0.691	572.867	-0.546	0.219	0.013	0.076
ZACN	363.106	1.022	0.204	0.000	0.000	363.106	0.195	0.206	0.343	0.600	363.106	-0.827	0.236	0.000	0.007
ZNF445	255.327	-0.331	0.165	0.045	0.107	255.327	-0.260	0.164	0.113	0.405	255.327	0.071	0.192	0.709	0.849
ANKRD37	498.970	0.950	0.423	0.025	0.068	498.970	0.816	0.423	0.053	0.342	498.970	-0.133	0.487	0.784	0.892
POLN	10.673	0.980	0.570	0.086	0.174	10.673	1.291	0.553	0.019	0.306	10.673	0.311	0.624	0.618	0.790
BMP8A	7.383	1.626	0.755	0.031	0.081	7.383	-0.429	0.851	0.614	0.787	7.383	-2.055	0.927	0.027	0.122
IGSF1	24.784	-0.136	0.553	0.806	0.872	24.784	0.272	0.543	0.616	0.788	24.784	0.408	0.634	0.520	0.723
FAS	424.690	-0.366	0.156	0.019	0.056	424.690	0.005	0.154	0.976	0.987	424.690	0.370	0.180	0.040	0.158
IK	3039.288	0.239	0.154	0.120	0.224	3039.288	-0.030	0.154	0.845	0.920	3039.288	-0.269	0.178	0.130	0.327
IKBKB	888.711	-0.129	0.115	0.261	0.396	888.711	-0.014	0.114	0.904	0.952	888.711	0.115	0.133	0.384	0.613
IL1A	1478.189	-1.836	0.538	0.001	0.004	1478.189	-1.684	0.538	0.002	0.297	1478.189	0.152	0.622	0.807	0.903
IL1B	188.752	-1.307	0.593	0.027	0.073	188.752	-2.066	0.595	0.001	0.253	188.752	-0.759	0.689	0.271	0.505
IL1R1	1034.696	0.459	0.176	0.009	0.032	1034.696	0.000	0.176	0.998	0.999	1034.696	-0.458	0.203	0.024	0.115
IL1RAP	712.722	-1.187	0.278	0.000	0.000	712.722	-0.387	0.276	0.161	0.452	712.722	0.800	0.321	0.013	0.076
IL2RG	55.122	2.375	0.556	0.000	0.000	55.122	1.744	0.565	0.002	0.297	55.122	-0.631	0.608	0.299	0.534
IL3RA	47.082	2.011	0.573	0.000	0.003	47.082	1.210	0.578	0.036	0.324	47.082	-0.800	0.656	0.222	0.450
IL4R	1896.326	-0.078	0.245	0.751	0.834	1896.326	-0.239	0.245	0.330	0.590	1896.326	-0.161	0.283	0.569	0.758
IL6	135.697	0.735	0.876	0.402	0.540	135.697	-2.081	0.890	0.019	0.306	135.697	-2.816	1.023	0.006	0.046
SHROOM2	99.046	0.111	0.214	0.605	0.718	99.046	0.272	0.210	0.197	0.480	99.046	0.161	0.245	0.512	0.717
IL6R	1243.525	-0.163	0.263	0.537	0.662	1243.525	0.401	0.263	0.127	0.419	1243.525	0.564	0.304	0.063	0.212
IL6ST	7316.321	-0.464	0.220	0.035	0.088	7316.321	-0.281	0.220	0.201	0.484	7316.321	0.183	0.254	0.471	0.685
IL7	100.240	-0.241	0.232	0.298	0.437	100.240	0.207	0.225	0.357	0.611	100.240	0.448	0.266	0.091	0.265
IL7R	538.337	-0.975	0.462	0.035	0.088	538.337	-1.532	0.463	0.001	0.294	538.337	-0.557	0.536	0.298	0.534
CXCL8	5060.255	-2.998	1.163	0.010	0.034	5060.255	-5.013	1.164	0.000	0.040	5060.255	-2.015	1.344	0.134	0.333
IL9R	140.103	0.490	0.426	0.249	0.383	140.103	0.318	0.426	0.456	0.682	140.103	-0.173	0.491	0.725	0.858
IL10RB	86.166	-0.127	0.349	0.716	0.806	86.166	0.261	0.345	0.449	0.678	86.166	0.388	0.402	0.334	0.569
IL11RA	20.197	1.057	0.598	0.077	0.161	20.197	1.028	0.596	0.085	0.376	20.197	-0.029	0.681	0.966	0.984
IL12A	19.136	-1.924	0.686	0.005	0.020	19.136	-1.316	0.658	0.045	0.334	19.136	0.608	0.798	0.446	0.666
IL13RA1	1883.652	0.275	0.123	0.025	0.068	1883.652	0.046	0.123	0.707	0.842	1883.652	-0.229	0.142	0.106	0.290
RFLNB	101.903	0.069	0.312	0.824	0.885	101.903	-0.412	0.314	0.190	0.475	101.903	-0.481	0.363	0.185	0.404
IRF2BP2	2633.928	0.289	0.108	0.008	0.027	2633.928	0.041	0.108	0.705	0.841	2633.928	-0.248	0.125	0.047	0.176
ACADSB	341.890	-0.514	0.245	0.036	0.090	341.890	0.014	0.243	0.953	0.977	341.890	0.529	0.283	0.062	0.208
AQP3	392.468	0.593	0.837	0.479	0.612	392.468	0.987	0.836	0.238	0.515	392.468	0.394	0.966	0.683	0.833
IL15	56.600	0.529	0.348	0.128	0.235	56.600	0.594	0.345	0.085	0.378	56.600	0.065	0.398	0.870	0.938
ZBTB41	368.176	-0.464	0.261	0.076	0.159	368.176	0.238	0.259	0.357	0.611	368.176	0.702	0.301	0.020	0.100
IL15RA	581.397	0.291	0.195	0.136	0.246	581.397	-0.075	0.196	0.700	0.839	581.397	-0.366	0.226	0.105	0.289
FKBP9P1	62.179	-0.071	0.321	0.824	0.885	62.179	0.249	0.314	0.429	0.664	62.179	0.320	0.368	0.385	0.614
TMPRSS9	24.152	0.182	0.370	0.623	0.733	24.152	0.080	0.368	0.827	0.910	24.152	-0.102	0.428	0.812	0.906

IL16	31.325	0.489	0.508	0.336	0.476	31.325	0.644	0.504	0.201	0.484	31.325	0.155	0.582	0.790	0.895
TNFRSF9	118.702	-1.714	0.569	0.003	0.012	118.702	-1.424	0.564	0.012	0.305	118.702	0.290	0.660	0.660	0.818
IL18	1483.800	0.187	0.170	0.273	0.409	1483.800	0.193	0.170	0.257	0.530	1483.800	0.006	0.196	0.975	0.987
FOXK2	1610.641	-0.162	0.147	0.271	0.407	1610.641	-0.158	0.147	0.281	0.551	1610.641	0.004	0.170	0.982	0.990
ILF2	2783.813	-0.500	0.276	0.070	0.149	2783.813	-0.522	0.276	0.058	0.348	2783.813	-0.022	0.319	0.945	0.975
ILF3	8809.949	-0.796	0.177	0.000	0.000	8809.949	-0.291	0.177	0.100	0.394	8809.949	0.505	0.205	0.014	0.080
ILK	2509.167	0.128	0.097	0.186	0.311	2509.167	-0.032	0.097	0.743	0.863	2509.167	-0.160	0.112	0.154	0.364
IMPA1	576.016	-0.483	0.138	0.000	0.003	576.016	-0.422	0.137	0.002	0.297	576.016	0.061	0.160	0.702	0.845
IMPA2	1334.550	-0.444	0.370	0.229	0.361	1334.550	0.462	0.369	0.210	0.491	1334.550	0.907	0.427	0.033	0.141
IMPDH1	1941.847	0.210	0.198	0.290	0.428	1941.847	-0.349	0.199	0.079	0.370	1941.847	-0.558	0.229	0.015	0.085
IMPDH2	7023.454	-0.621	0.163	0.000	0.001	7023.454	0.299	0.163	0.066	0.354	7023.454	0.920	0.188	0.000	0.000
INCENP	1082.631	-0.753	0.199	0.000	0.001	1082.631	-0.024	0.198	0.902	0.950	1082.631	0.729	0.229	0.001	0.018
ING1	459.007	0.017	0.175	0.925	0.952	459.007	0.156	0.174	0.370	0.619	459.007	0.139	0.202	0.490	0.700
ING2	273.635	0.464	0.270	0.085	0.174	273.635	0.373	0.269	0.166	0.456	273.635	-0.091	0.311	0.770	0.883
INHA	12.473	0.847	0.880	0.336	0.476	12.473	1.301	0.870	0.135	0.426	12.473	0.454	1.000	0.650	0.812
CXCL10	28.629	3.390	0.557	0.000	0.000	28.629	0.779	0.601	0.194	0.478	28.629	-2.611	0.640	0.000	0.001
INPP1	311.469	0.453	0.168	0.007	0.026	311.469	0.098	0.169	0.563	0.751	311.469	-0.355	0.195	0.069	0.223
INPP4A	307.351	-0.224	0.189	0.236	0.369	307.351	-0.136	0.188	0.470	0.692	307.351	0.088	0.219	0.686	0.835
INPP5A	220.957	-0.342	0.166	0.039	0.095	220.957	-0.151	0.163	0.355	0.609	220.957	0.192	0.192	0.317	0.553
INPP5B	186.735	-0.591	0.188	0.002	0.008	186.735	-0.037	0.182	0.840	0.917	186.735	0.554	0.216	0.010	0.067
INPPL1	2555.363	-0.154	0.124	0.213	0.342	2555.363	0.054	0.123	0.660	0.814	2555.363	0.208	0.143	0.144	0.351
INSIG1	1849.160	2.842	0.386	0.000	0.000	1849.160	0.497	0.387	0.199	0.482	1849.160	-2.344	0.446	0.000	0.000
INSR	629.318	0.408	0.218	0.061	0.134	629.318	0.355	0.217	0.102	0.395	629.318	-0.053	0.251	0.834	0.919
EIF3E	11583.808	0.049	0.253	0.846	0.899	11583.808	0.330	0.253	0.192	0.477	11583.808	0.281	0.292	0.336	0.570
IPP	204.041	-0.483	0.219	0.027	0.073	204.041	0.146	0.214	0.497	0.711	204.041	0.629	0.251	0.012	0.075
IRAK1	5959.590	-0.311	0.317	0.327	0.467	5959.590	-0.448	0.317	0.158	0.450	5959.590	-0.137	0.366	0.708	0.849
ITGA6	3661.990	-0.980	0.311	0.002	0.008	3661.990	-0.278	0.311	0.371	0.620	3661.990	0.702	0.359	0.050	0.184
IRAK2	687.133	-0.443	0.342	0.194	0.320	687.133	-0.556	0.341	0.104	0.396	687.133	-0.113	0.395	0.776	0.886
IREB2	1186.398	-0.834	0.218	0.000	0.001	1186.398	-0.200	0.217	0.356	0.610	1186.398	0.633	0.252	0.012	0.073
IRF1	625.348	0.831	0.169	0.000	0.000	625.348	-0.200	0.171	0.242	0.518	625.348	-1.032	0.197	0.000	0.000
AQP9	684.461	3.178	0.260	0.000	0.000	684.461	0.309	0.285	0.278	0.548	684.461	-2.869	0.306	0.000	0.000
IRF2	723.613	0.754	0.159	0.000	0.000	723.613	0.222	0.159	0.164	0.454	723.613	-0.532	0.183	0.004	0.034
IRF3	841.892	0.419	0.274	0.127	0.233	841.892	-0.274	0.275	0.318	0.579	841.892	-0.693	0.317	0.029	0.128
IRF5	16.959	2.657	0.429	0.000	0.000	16.959	0.629	0.483	0.193	0.478	16.959	-2.029	0.495	0.000	0.001
IRF6	75.997	2.297	0.577	0.000	0.001	75.997	0.324	0.585	0.580	0.764	75.997	-1.973	0.668	0.003	0.030
IRF7	287.610	0.857	0.274	0.002	0.009	287.610	0.209	0.276	0.447	0.677	287.610	-0.648	0.316	0.041	0.161
IRS1	90.119	0.328	0.217	0.130	0.237	90.119	-0.122	0.220	0.578	0.763	90.119	-0.450	0.253	0.075	0.235
ISG20	246.406	2.329	0.358	0.000	0.000	246.406	0.859	0.364	0.018	0.306	246.406	-1.470	0.411	0.000	0.006
AR	1566.197	-0.433	0.325	0.183	0.307	1566.197	0.183	0.325	0.574	0.759	1566.197	0.616	0.375	0.101	0.282
ISLR	71.850	2.631	0.689	0.000	0.001	71.850	0.588	0.695	0.398	0.641	71.850	-2.043	0.795	0.010	0.067
ITGA1	152.092	0.722	0.287	0.012	0.039	152.092	0.434	0.287	0.130	0.422	152.092	-0.288	0.330	0.384	0.613
ITGA2	3448.583	-1.328	0.338	0.000	0.001	3448.583	-0.671	0.338	0.047	0.336	3448.583	0.657	0.391	0.092	0.267
ITGA3	10378.116	0.677	0.106	0.000	0.000	10378.116	0.130	0.106	0.221	0.500	10378.116	-0.548	0.122	0.000	0.000
ITGA5	11661.590	0.229	0.135	0.090	0.181	11661.590	0.278	0.135	0.040	0.327	11661.590	0.049	0.156	0.755	0.875
ITGA7	22.868	1.095	0.543	0.044	0.104	22.868	0.349	0.554	0.528	0.729	22.868	-0.746	0.627	0.235	0.464
ABCC6	20.872	1.376	0.750	0.067	0.144	20.872	0.906	0.754	0.229	0.507	20.872	-0.469	0.860	0.585	0.767
ITGA9	20.858	0.200	0.599	0.738	0.823	20.858	0.723	0.588	0.218	0.498	20.858	0.523	0.684	0.444	0.664
ITGAE	121.500	-0.810	0.256	0.002	0.008	121.500	-0.438	0.250	0.080	0.371	121.500	0.372	0.296	0.210	0.435
ITGAL	20.224	-0.603	0.586	0.303	0.442	20.224	1.299	0.545	0.017	0.306	20.224	1.902	0.656	0.004	0.034
ITGAV	1820.432	-0.364	0.274	0.184	0.308	1820.432	-0.258	0.274	0.346	0.602	1820.432	0.106	0.317	0.738	0.865
ITGB1	11933.208	-0.321	0.150	0.033	0.084	11933.208	-0.191	0.150	0.204	0.487	11933.208	0.130	0.174	0.453	0.672
ARAF	862.002	-0.553	0.196	0.005	0.019	862.002	0.067	0.194	0.731	0.856	862.002	0.620	0.226	0.006	0.046
ITGB3	188.935	0.868	0.407	0.033	0.084	188.935	-0.173	0.409	0.673	0.823	188.935	-1.041	0.471	0.027	0.123
ITGB4	14458.380	0.818	0.393	0.037	0.092	14458.380	0.781	0.393	0.047	0.336	14458.380	-0.037	0.454	0.934	0.970
EIF6	2071.363	0.010	0.272	0.969	0.981	2071.363	-0.299	0.272	0.271	0.542	2071.363	-0.310	0.314	0.324	0.558
ITGB5	2399.339	0.177	0.341	0.604	0.717	2399.339	-0.449	0.341	0.188	0.474	2399.339	-0.626	0.394	0.112	0.299
ITGB8	46.832	1.892	0.359	0.000	0.000	46.832	-0.114	0.402	0.777	0.882	46.832	-2.006	0.434	0.000	0.000
ACADVL	3316.854	0.421	0.233	0.071	0.150	3316.854	0.617	0.233	0.008	0.297	3316.854	0.196	0.269	0.465	0.681
STT3A	3021.175	0.040	0.167	0.811	0.876	3021.175	-0.141	0.167	0.398	0.642	3021.175	-0.181	0.193	0.348	0.582
ITPA	736.859	0.059	0.261	0.820	0.882	736.859	-0.378	0.261	0.148	0.440	736.859	-0.437	0.302	0.147	0.355
ITPK1	2853.207	0.107	0.176	0.542	0.667	2853.207	-0.015	0.176	0.931	0.965	2853.207	-0.123	0.204	0.546	0.742
ITPKA	137.675	0.279	0.387	0.471	0.606	137.675	1.093	0.380	0.004	0.297	137.675	0.814	0.441	0.065	0.214
ITPKB	92.243	0.447	0.269	0.097	0.191	92.243	0.287	0.269	0.286	0.555	92.243	-0.160	0.310	0.606	0.783
ITPR1	1423.746	-0.466	0.280	0.096	0.190	1423.746	0.105	0.279	0.707	0.842	1423.746	0.571	0.323	0.077	0.239
ITPR2	322.602	0.097	0.202	0.632	0.740	322.602	-0.159	0.203	0.432	0.666	322.602	-0.256	0.235	0.274	0.509
ITPR3	6171.555	0.039	0.170	0.819	0.882	6171.555	0.086	0.170	0.612	0.786	6171.555	0.047	0.197	0.810	0.904
IVD	1115.025	-0.275	0.150	0.066	0.143	1115.025	0.015	0.149	0.917	0.959	1115.025	0.291	0.173	0.093	0.267

JAG2	3758.985	0.056	0.142	0.696	0.790	3758.985	0.077	0.142	0.590	0.772	3758.985	0.021	0.165	0.898	0.952
JAK1	2762.194	-0.306	0.192	0.111	0.211	2762.194	0.046	0.192	0.811	0.901	2762.194	0.352	0.222	0.112	0.300
JAK2	53.343	0.161	0.302	0.595	0.710	53.343	-0.484	0.309	0.118	0.409	53.343	-0.645	0.357	0.071	0.226
ARCN1	2698.113	-0.223	0.147	0.128	0.235	2698.113	-0.216	0.147	0.141	0.432	2698.113	0.008	0.169	0.963	0.983
JARID2	322.969	-0.379	0.198	0.056	0.125	322.969	-0.184	0.196	0.348	0.604	322.969	0.195	0.229	0.395	0.623
JUN	5003.355	1.295	0.160	0.000	0.000	5003.355	0.119	0.160	0.456	0.683	5003.355	-1.176	0.185	0.000	0.000
JUNB	5296.526	0.704	0.179	0.000	0.001	5296.526	0.037	0.179	0.835	0.915	5296.526	-0.667	0.207	0.001	0.016
JUND	2069.418	1.449	0.224	0.000	0.000	2069.418	-0.014	0.225	0.951	0.976	2069.418	-1.463	0.259	0.000	0.000
JUP	4247.872	0.570	0.522	0.275	0.412	4247.872	0.769	0.522	0.141	0.431	4247.872	0.200	0.603	0.740	0.867
TRIM23	354.414	-0.459	0.202	0.023	0.064	354.414	-0.192	0.200	0.336	0.595	354.414	0.266	0.234	0.254	0.487
GSTK1	681.091	0.689	0.194	0.000	0.003	681.091	0.305	0.194	0.117	0.409	681.091	-0.384	0.224	0.087	0.256
CD82	3413.233	0.710	0.168	0.000	0.000	3413.233	0.323	0.168	0.054	0.342	3413.233	-0.387	0.194	0.046	0.173
KARS1	5236.266	-0.190	0.139	0.172	0.292	5236.266	-0.163	0.139	0.241	0.517	5236.266	0.027	0.160	0.866	0.936
USP50	24.680	1.110	0.461	0.016	0.049	24.680	-0.334	0.488	0.494	0.709	24.680	-1.443	0.548	0.008	0.059
DND1	225.250	0.310	0.193	0.108	0.207	225.250	-0.051	0.194	0.791	0.891	225.250	-0.361	0.224	0.107	0.291
AREG	102.459	-2.728	0.899	0.002	0.011	102.459	-2.901	0.899	0.001	0.297	102.459	-0.173	1.045	0.869	0.937
NDUFS7	1903.484	0.322	0.233	0.166	0.286	1903.484	0.183	0.232	0.431	0.665	1903.484	-0.139	0.268	0.606	0.782
NHLRC2	369.156	-0.411	0.148	0.005	0.021	369.156	-0.181	0.146	0.215	0.494	369.156	0.231	0.171	0.177	0.394
GALNT18	1843.484	-0.111	0.185	0.548	0.671	1843.484	-0.153	0.185	0.408	0.649	1843.484	-0.042	0.214	0.845	0.925
NCR3LG1	456.308	-0.953	0.453	0.035	0.089	456.308	-0.645	0.452	0.154	0.446	456.308	0.308	0.523	0.557	0.749
FAM111B	220.440	-0.283	0.348	0.417	0.554	220.440	-0.768	0.350	0.028	0.321	220.440	-0.485	0.404	0.230	0.459
TMEM179B	487.573	0.205	0.229	0.370	0.511	487.573	0.014	0.229	0.951	0.976	487.573	-0.191	0.264	0.470	0.685
LOC374443	59.336	-0.615	0.226	0.007	0.025	59.336	-0.075	0.211	0.721	0.851	59.336	0.540	0.259	0.037	0.152
PTPRQ	30.796	0.975	0.903	0.280	0.418	30.796	0.408	0.905	0.652	0.810	30.796	-0.566	1.042	0.587	0.768
TEX9	170.877	-0.150	0.224	0.502	0.631	170.877	0.148	0.221	0.504	0.715	170.877	0.298	0.258	0.248	0.479
GOLGA6L5P	50.272	1.241	0.468	0.008	0.029	50.272	0.220	0.476	0.644	0.806	50.272	-1.021	0.542	0.060	0.205
KIF7	212.163	0.230	0.295	0.435	0.571	212.163	-0.355	0.296	0.231	0.509	212.163	-0.584	0.342	0.087	0.258
ZNF710	501.640	-0.180	0.124	0.148	0.262	501.640	0.062	0.123	0.615	0.788	501.640	0.241	0.143	0.092	0.266
HDDC3	202.029	-0.226	0.199	0.257	0.391	202.029	0.055	0.197	0.780	0.884	202.029	0.281	0.230	0.221	0.449
WASH3P	410.303	0.474	0.179	0.008	0.028	410.303	0.441	0.178	0.013	0.305	410.303	-0.033	0.206	0.873	0.939
EFCAB5	26.828	0.021	0.340	0.951	0.970	26.828	-0.422	0.349	0.227	0.506	26.828	-0.443	0.406	0.276	0.511
LRRC37A3	36.740	0.907	0.439	0.039	0.095	36.740	0.298	0.445	0.503	0.714	36.740	-0.609	0.508	0.230	0.459
ANKRD30B	13.714	0.300	0.443	0.498	0.629	13.714	0.286	0.436	0.512	0.719	13.714	-0.014	0.507	0.978	0.989
ATP9B	190.032	-0.098	0.171	0.568	0.688	190.032	-0.120	0.170	0.481	0.700	190.032	-0.022	0.199	0.911	0.959
HSD11B1L	120.382	1.036	0.479	0.030	0.079	120.382	0.721	0.479	0.132	0.424	120.382	-0.314	0.551	0.568	0.758
ZNF699	60.825	0.041	0.261	0.874	0.919	60.825	0.210	0.256	0.411	0.651	60.825	0.169	0.300	0.573	0.760
TMEM205	1284.452	0.424	0.210	0.043	0.104	1284.452	0.480	0.210	0.022	0.315	1284.452	0.056	0.242	0.816	0.908
YJEFN3	45.291	1.925	0.411	0.000	0.000	45.291	0.972	0.418	0.020	0.310	45.291	-0.953	0.469	0.042	0.164
SBSN	10.929	4.757	0.781	0.000	0.000	10.929	0.604	1.045	0.564	0.752	10.929	-4.153	0.925	0.000	0.000
ZNF829	11.794	0.622	0.460	0.177	0.299	11.794	0.564	0.455	0.216	0.495	11.794	-0.058	0.520	0.911	0.959
ZNF568	23.414	-0.369	0.404	0.362	0.502	23.414	-0.309	0.397	0.436	0.669	23.414	0.060	0.471	0.899	0.953
ZSWIM9	327.164	0.584	0.248	0.019	0.055	327.164	0.356	0.249	0.152	0.444	327.164	-0.228	0.287	0.426	0.649
ZNF773	137.261	-0.675	0.264	0.011	0.036	137.261	-0.558	0.262	0.033	0.322	137.261	0.117	0.307	0.703	0.845
SPATA21	1068.405	-0.253	0.199	0.204	0.332	1068.405	0.038	0.199	0.850	0.923	1068.405	0.291	0.230	0.206	0.431
SVBP	185.642	0.087	0.214	0.685	0.782	185.642	0.343	0.212	0.105	0.398	185.642	0.256	0.246	0.298	0.533
MIGA1	512.497	-0.559	0.257	0.029	0.077	512.497	0.107	0.255	0.675	0.825	512.497	0.666	0.296	0.024	0.115
ARF1	8155.318	0.063	0.116	0.589	0.704	8155.318	-0.027	0.116	0.819	0.905	8155.318	-0.089	0.134	0.505	0.712
PEAR1	88.699	-0.249	0.341	0.465	0.599	88.699	0.254	0.335	0.448	0.677	88.699	0.503	0.391	0.199	0.422
SFT2D2	395.587	0.427	0.277	0.123	0.228	395.587	-0.259	0.278	0.352	0.606	395.587	-0.686	0.321	0.033	0.139
MIA3	1362.381	-0.276	0.152	0.070	0.149	1362.381	-0.190	0.152	0.210	0.491	1362.381	0.086	0.176	0.627	0.796
FAM89A	47.304	-0.182	0.479	0.704	0.797	47.304	-0.502	0.481	0.297	0.565	47.304	-0.320	0.560	0.568	0.758
PI4KAP2	270.852	0.214	0.207	0.301	0.440	270.852	0.155	0.206	0.453	0.680	270.852	-0.059	0.239	0.805	0.903
FAM228B	105.208	0.488	0.345	0.157	0.274	105.208	0.485	0.344	0.159	0.451	105.208	-0.003	0.397	0.993	0.996
ANKRD36	108.618	0.486	0.331	0.142	0.253	108.618	-0.210	0.335	0.531	0.731	108.618	-0.696	0.385	0.071	0.226
WASH2P	217.354	0.446	0.279	0.110	0.209	217.354	0.362	0.279	0.194	0.478	217.354	-0.084	0.322	0.794	0.896
RBM43	207.103	-0.251	0.454	0.580	0.698	207.103	0.554	0.452	0.220	0.500	207.103	0.805	0.523	0.124	0.317
LINC01116	45.383	0.206	0.308	0.504	0.633	45.383	-0.251	0.312	0.421	0.658	45.383	-0.457	0.360	0.205	0.428
KCNE1	21.739	3.636	0.687	0.000	0.000	21.739	2.179	0.702	0.002	0.297	21.739	-1.457	0.754	0.053	0.190
C3orf62	82.869	1.165	0.402	0.004	0.016	82.869	0.623	0.405	0.124	0.416	82.869	-0.542	0.463	0.241	0.472
STIMATE	22.560	-0.045	0.519	0.931	0.956	22.560	-0.352	0.522	0.500	0.713	22.560	-0.307	0.606	0.612	0.786
C5orf34	111.766	-0.925	0.354	0.009	0.031	111.766	-0.852	0.352	0.016	0.306	111.766	0.073	0.411	0.859	0.933
MAST4	1033.953	-0.045	0.208	0.828	0.887	1033.953	0.010	0.207	0.961	0.980	1033.953	0.055	0.240	0.818	0.910
SIMC1	129.974	0.038	0.179	0.830	0.888	129.974	-0.091	0.178	0.611	0.785	129.974	-0.129	0.208	0.534	0.733
KCNG1	1315.314	-1.123	0.262	0.000	0.000	1315.314	-0.140	0.261	0.593	0.774	1315.314	0.984	0.303	0.001	0.015
GJB7	9.110	-0.668	0.573	0.244	0.377	9.110	-0.232	0.532	0.663	0.817	9.110	0.436	0.661	0.510	0.715
KCP	400.815	0.829	0.212	0.000	0.001	400.815	0.064	0.214	0.764	0.875	400.815	-0.765	0.245	0.002	0.020
WASH5P	47.682	0.073	0.318	0.817	0.880	47.682	0.123	0.315	0.695	0.836	47.682	0.050	0.367	0.892	0.949

PTAR1	585.951	-0.680	0.279	0.015	0.045	585.951	-0.219	0.277	0.430	0.664	585.951	0.461	0.322	0.152	0.361
ERCC6L2	239.844	-0.523	0.186	0.005	0.020	239.844	-0.127	0.183	0.489	0.705	239.844	0.396	0.215	0.065	0.216
SWI5	310.660	-0.125	0.138	0.364	0.505	310.660	0.074	0.136	0.585	0.768	310.660	0.199	0.159	0.210	0.435
PNPLA7	10.913	0.453	0.686	0.509	0.638	10.913	0.911	0.666	0.171	0.460	10.913	0.458	0.772	0.553	0.747
AGRN	12757.131	0.658	0.313	0.036	0.089	12757.131	0.515	0.313	0.100	0.394	12757.131	-0.143	0.362	0.692	0.838
RAB15	37.936	0.188	0.242	0.437	0.573	37.936	-0.173	0.247	0.483	0.701	37.936	-0.361	0.286	0.207	0.431
KCNJ8	11.180	0.022	0.575	0.970	0.981	11.180	-0.229	0.578	0.691	0.834	11.180	-0.251	0.673	0.709	0.849
SLC27A1	456.277	0.664	0.525	0.206	0.334	456.277	1.000	0.525	0.057	0.345	456.277	0.335	0.606	0.580	0.764
ZC3H6	202.765	0.458	0.390	0.241	0.374	202.765	0.549	0.390	0.159	0.451	202.765	0.091	0.450	0.840	0.923
ARF3	3340.321	0.236	0.141	0.094	0.187	3340.321	-0.274	0.141	0.053	0.341	3340.321	-0.511	0.163	0.002	0.020
KCNJ14	26.587	1.640	0.476	0.001	0.004	26.587	0.580	0.496	0.242	0.518	26.587	-1.061	0.548	0.053	0.189
KLHL30	80.323	-0.240	0.418	0.566	0.686	80.323	0.906	0.409	0.027	0.318	80.323	1.146	0.478	0.016	0.090
KCNK1	315.832	0.203	0.246	0.410	0.548	315.832	-0.164	0.247	0.506	0.716	315.832	-0.367	0.285	0.198	0.421
CA13	101.711	-0.362	0.235	0.123	0.229	101.711	-0.609	0.236	0.010	0.298	101.711	-0.247	0.276	0.371	0.603
KCNK3	13.453	1.769	0.972	0.069	0.147	13.453	-0.363	1.061	0.733	0.857	13.453	-2.132	1.171	0.069	0.223
KCNMA1	1434.372	-1.465	0.353	0.000	0.000	1434.372	0.265	0.351	0.451	0.679	1434.372	1.730	0.407	0.000	0.001
ARF4	2881.488	0.283	0.254	0.264	0.399	2881.488	-0.442	0.254	0.081	0.372	2881.488	-0.726	0.293	0.013	0.079
TRIM74	10.948	1.445	0.589	0.014	0.044	10.948	1.124	0.594	0.059	0.348	10.948	-0.321	0.651	0.622	0.793
KCNN4	26.411	0.558	0.623	0.371	0.511	26.411	0.493	0.620	0.426	0.662	26.411	-0.065	0.713	0.927	0.967
KCNQ1	12.137	0.176	0.582	0.763	0.842	12.137	0.502	0.563	0.373	0.621	12.137	0.327	0.660	0.621	0.792
KCNQ3	173.027	0.102	0.436	0.815	0.878	173.027	-0.617	0.438	0.159	0.451	173.027	-0.719	0.505	0.155	0.365
RN7SL2	1592.062	-0.424	0.220	0.053	0.121	1592.062	0.280	0.219	0.201	0.484	1592.062	0.704	0.253	0.005	0.043
RN7SL3	66.116	-0.414	0.378	0.274	0.411	66.116	-0.236	0.375	0.529	0.730	66.116	0.178	0.438	0.684	0.833
CENPS	70.896	-0.224	0.329	0.495	0.627	70.896	0.060	0.323	0.853	0.925	70.896	0.284	0.379	0.453	0.672
LINC-PINT	55.986	0.169	0.292	0.561	0.682	55.986	-0.366	0.300	0.222	0.501	55.986	-0.536	0.346	0.122	0.314
PICSAR	557.558	0.289	0.164	0.078	0.162	557.558	0.026	0.164	0.873	0.936	557.558	-0.263	0.190	0.166	0.381
MALAT1	5478.570	3.128	0.448	0.000	0.000	5478.570	0.683	0.448	0.128	0.420	5478.570	-2.445	0.517	0.000	0.000
ARL4D	613.380	0.212	0.199	0.287	0.425	613.380	0.135	0.199	0.495	0.710	613.380	-0.076	0.230	0.739	0.866
KCNS3	55.371	1.903	0.295	0.000	0.000	55.371	-0.123	0.326	0.707	0.842	55.371	-2.026	0.354	0.000	0.000
PSMA3-AS1	184.534	-0.088	0.254	0.729	0.816	184.534	-0.126	0.253	0.619	0.790	184.534	-0.038	0.293	0.897	0.951
KDR	67.248	-1.349	1.060	0.203	0.330	67.248	0.424	1.052	0.687	0.831	67.248	1.774	1.222	0.147	0.353
KHK	132.675	0.911	0.220	0.000	0.000	132.675	0.327	0.223	0.144	0.435	132.675	-0.584	0.255	0.022	0.107
KIF2A	1026.974	-1.254	0.359	0.000	0.003	1026.974	-0.359	0.358	0.316	0.578	1026.974	0.894	0.415	0.031	0.134
KIF3C	134.394	1.221	0.394	0.002	0.010	134.394	0.164	0.397	0.680	0.827	134.394	-1.058	0.455	0.020	0.102
KIF5B	3657.654	-0.807	0.168	0.000	0.000	3657.654	-0.400	0.168	0.017	0.306	3657.654	0.407	0.194	0.036	0.148
ACAT1	1116.340	-0.312	0.121	0.010	0.033	1116.340	-0.022	0.120	0.857	0.927	1116.340	0.291	0.139	0.037	0.151
KIFC3	2459.556	-0.389	0.202	0.054	0.122	2459.556	-0.120	0.201	0.550	0.743	2459.556	0.268	0.233	0.249	0.481
KIR2DL1	141.147	0.240	0.458	0.600	0.714	141.147	1.043	0.455	0.022	0.314	141.147	0.803	0.526	0.127	0.322
KIR2DL3	6.186	-0.393	1.574	0.803	0.870	6.186	-1.568	1.617	0.332	0.592	6.186	-1.176	1.865	0.528	0.729
KIR2DL4	48.354	1.212	0.640	0.058	0.130	48.354	1.027	0.641	0.109	0.401	48.354	-0.185	0.735	0.801	0.900
KIR2DS4	108.991	-0.578	0.574	0.314	0.454	108.991	0.968	0.566	0.088	0.380	108.991	1.545	0.659	0.019	0.099
ARF5	1332.093	1.018	0.236	0.000	0.000	1332.093	0.252	0.236	0.286	0.555	1332.093	-0.766	0.272	0.005	0.040
KIR2DS5	143.224	0.501	0.437	0.252	0.386	143.224	1.371	0.433	0.002	0.297	143.224	0.871	0.500	0.082	0.248
KIR3DS1	34.436	-0.273	0.475	0.566	0.686	34.436	-0.491	0.476	0.303	0.569	34.436	-0.218	0.555	0.695	0.841
KIT	30.398	0.683	0.362	0.060	0.132	30.398	0.441	0.364	0.225	0.504	30.398	-0.242	0.415	0.561	0.752
KLK1	9.740	-0.043	0.536	0.936	0.960	9.740	0.369	0.505	0.465	0.689	9.740	0.412	0.605	0.496	0.704
ARF6	3383.476	-0.450	0.178	0.011	0.038	3383.476	-0.331	0.178	0.063	0.350	3383.476	0.120	0.206	0.560	0.752
KLRC2	63.818	0.215	0.604	0.722	0.811	63.818	0.793	0.600	0.186	0.473	63.818	0.578	0.694	0.405	0.632
KLRC3	69.274	0.383	0.660	0.562	0.683	69.274	0.650	0.658	0.323	0.584	69.274	0.267	0.761	0.725	0.858
KLRD1	95.717	4.695	0.450	0.000	0.000	95.717	1.107	0.479	0.021	0.312	95.717	-3.588	0.512	0.000	0.000
KLC1	1915.203	-0.506	0.259	0.051	0.117	1915.203	-0.561	0.259	0.031	0.322	1915.203	-0.055	0.300	0.855	0.930
KIF11	1341.430	-0.676	0.220	0.002	0.010	1341.430	-0.193	0.219	0.378	0.626	1341.430	0.483	0.254	0.057	0.199
KIFC1	3022.407	0.136	0.193	0.481	0.614	3022.407	0.281	0.193	0.146	0.438	3022.407	0.145	0.223	0.517	0.721
KIF22	1216.358	-0.354	0.245	0.150	0.263	1216.358	-0.104	0.245	0.673	0.823	1216.358	0.250	0.283	0.377	0.609
KPNA1	1224.141	-0.578	0.265	0.029	0.077	1224.141	-0.545	0.265	0.040	0.327	1224.141	0.033	0.306	0.913	0.960
KPNB1	10766.462	-0.613	0.117	0.000	0.000	10766.462	-0.161	0.116	0.166	0.456	10766.462	0.452	0.135	0.001	0.011
KPNA2	3465.193	-0.185	0.205	0.367	0.507	3465.193	-0.461	0.205	0.025	0.316	3465.193	-0.276	0.237	0.244	0.475
KPNA3	1393.098	-0.791	0.271	0.003	0.015	1393.098	-0.577	0.270	0.033	0.322	1393.098	0.214	0.313	0.493	0.702
ARG2	1148.930	-0.218	0.141	0.121	0.226	1148.930	0.399	0.139	0.004	0.297	1148.930	0.617	0.162	0.000	0.003
KPNA4	2485.542	-0.914	0.323	0.005	0.019	2485.542	-0.816	0.323	0.012	0.305	2485.542	0.099	0.373	0.792	0.896
KPNA5	119.663	-0.259	0.297	0.384	0.524	119.663	0.386	0.291	0.185	0.472	119.663	0.645	0.341	0.059	0.202
TNPO1	6763.997	-0.926	0.236	0.000	0.001	6763.997	-0.190	0.236	0.421	0.658	6763.997	0.736	0.272	0.007	0.051
IPO5	4864.209	-0.934	0.154	0.000	0.000	4864.209	-0.101	0.153	0.511	0.719	4864.209	0.834	0.177	0.000	0.000
KRAS	792.424	-0.286	0.204	0.161	0.280	792.424	-0.323	0.204	0.112	0.405	792.424	-0.038	0.236	0.872	0.939
KRT5	316.375	0.001	0.907	0.999	1.000	316.375	0.642	0.907	0.479	0.699	316.375	0.641	1.047	0.541	0.737
KRT6A	646.427	0.803	0.360	0.026	0.070	646.427	0.034	0.360	0.926	0.962	646.427	-0.769	0.416	0.064	0.213
KRT6B	11.901	-0.084	0.547	0.878	0.921	11.901	0.512	0.522	0.326	0.587	11.901	0.596	0.619	0.336	0.570

KRT7	55622.549	0.389	0.280	0.165	0.284	55622.549	-0.255	0.280	0.362	0.614	55622.549	-0.644	0.323	0.046	0.174
KRT8	10670.295	0.999	0.188	0.000	0.000	10670.295	0.230	0.188	0.223	0.501	10670.295	-0.769	0.217	0.000	0.007
KRT10	975.865	-0.064	0.238	0.789	0.860	975.865	0.034	0.237	0.885	0.942	975.865	0.098	0.275	0.721	0.855
KRT14	18.307	0.467	0.512	0.362	0.503	18.307	0.306	0.511	0.550	0.743	18.307	-0.161	0.589	0.785	0.892
CHKB-CPT1B	13.299	1.740	0.615	0.005	0.019	13.299	0.715	0.633	0.259	0.531	13.299	-1.024	0.705	0.146	0.353
SAP30L-AS1	98.518	-0.734	0.529	0.165	0.284	98.518	-0.500	0.527	0.342	0.599	98.518	0.234	0.611	0.702	0.845
KRT16	17.178	1.786	0.810	0.027	0.073	17.178	1.658	0.809	0.040	0.328	17.178	-0.127	0.919	0.890	0.947
RHOA	12952.413	-0.011	0.121	0.927	0.954	12952.413	0.000	0.121	0.999	1.000	12952.413	0.011	0.139	0.938	0.972
ZKSCAN4	123.786	0.094	0.311	0.762	0.842	123.786	0.394	0.308	0.201	0.484	123.786	0.300	0.358	0.402	0.629
GUSBP2	11.583	-0.440	0.548	0.422	0.559	11.583	-0.333	0.537	0.535	0.734	11.583	0.107	0.638	0.867	0.936
SNHG5	101.468	-2.261	1.009	0.025	0.068	101.468	-1.422	1.002	0.156	0.448	101.468	0.839	1.166	0.472	0.686
CENPW	343.750	0.529	0.381	0.164	0.284	343.750	-0.348	0.382	0.363	0.615	343.750	-0.877	0.441	0.047	0.175
CEP85L	100.978	-0.214	0.229	0.351	0.491	100.978	-0.100	0.226	0.659	0.814	100.978	0.115	0.265	0.666	0.822
KRT17	230.273	4.642	0.743	0.000	0.000	230.273	1.924	0.746	0.010	0.298	230.273	-2.718	0.854	0.001	0.017
C6orf120	636.255	-0.851	0.214	0.000	0.001	636.255	-0.378	0.213	0.076	0.364	636.255	0.473	0.247	0.056	0.196
KRTAP5-1	9.168	1.506	0.848	0.076	0.159	9.168	0.187	0.881	0.832	0.913	9.168	-1.319	0.989	0.182	0.401
NSUN4	669.509	-0.273	0.108	0.012	0.038	669.509	-0.134	0.107	0.209	0.490	669.509	0.139	0.125	0.268	0.502
RASL11A	14.509	1.624	0.445	0.000	0.002	14.509	-0.222	0.505	0.661	0.815	14.509	-1.846	0.545	0.001	0.010
KRT18	5967.175	0.752	0.187	0.000	0.001	5967.175	0.002	0.187	0.989	0.994	5967.175	-0.750	0.216	0.001	0.008
GPR153	57.491	-0.165	0.336	0.624	0.735	57.491	0.150	0.328	0.648	0.808	57.491	0.315	0.386	0.415	0.641
TMEM189	674.687	1.118	0.241	0.000	0.000	674.687	0.493	0.242	0.042	0.330	674.687	-0.625	0.278	0.025	0.116
FGF7P6	26.876	-0.577	0.418	0.167	0.287	26.876	0.496	0.387	0.200	0.483	26.876	1.073	0.471	0.023	0.109
SKIDA1	37.292	-0.112	0.440	0.799	0.868	37.292	-0.002	0.436	0.997	0.999	37.292	0.110	0.508	0.828	0.915
WASHC2A	1011.702	0.189	0.153	0.217	0.347	1011.702	0.134	0.153	0.380	0.626	1011.702	-0.055	0.176	0.756	0.876
C10orf99	17.800	0.980	1.678	0.559	0.681	17.800	-0.095	1.684	0.955	0.978	17.800	-1.075	1.941	0.580	0.764
C10orf143	8.840	0.461	0.620	0.458	0.593	8.840	1.035	0.590	0.079	0.371	8.840	0.574	0.687	0.403	0.630
GVINP1	21.269	-0.571	0.466	0.220	0.351	21.269	0.075	0.446	0.866	0.932	21.269	0.646	0.534	0.226	0.454
INSC	119.078	-2.227	0.545	0.000	0.000	119.078	-1.230	0.535	0.021	0.314	119.078	0.997	0.629	0.113	0.301
FIBIN	127.208	-0.103	0.492	0.833	0.890	127.208	0.514	0.489	0.294	0.561	127.208	0.617	0.567	0.276	0.511
C11orf96	58.967	1.048	0.506	0.038	0.095	58.967	-0.362	0.542	0.504	0.715	58.967	-1.410	0.607	0.020	0.102
LIPT2	26.002	0.233	0.489	0.634	0.742	26.002	0.338	0.484	0.484	0.702	26.002	0.105	0.562	0.851	0.928
RPL13AP20	16.010	0.988	0.567	0.081	0.167	16.010	0.488	0.576	0.397	0.641	16.010	-0.500	0.651	0.442	0.662
REP15	9.903	0.471	0.572	0.410	0.548	9.903	-0.631	0.616	0.306	0.572	9.903	-1.102	0.696	0.114	0.302
C12orf75	449.095	0.237	0.335	0.479	0.613	449.095	-0.705	0.336	0.036	0.324	449.095	-0.942	0.388	0.015	0.086
CFAP73	671.730	0.324	0.205	0.115	0.217	671.730	0.107	0.205	0.602	0.780	671.730	-0.217	0.237	0.361	0.594
KMT5A	750.315	-1.041	0.235	0.000	0.000	750.315	-0.458	0.234	0.050	0.337	750.315	0.583	0.271	0.031	0.136
LINC00944	94.859	0.364	0.301	0.226	0.358	94.859	0.155	0.302	0.608	0.784	94.859	-0.210	0.348	0.547	0.742
NHLRC3	103.792	0.727	0.237	0.002	0.010	103.792	0.084	0.241	0.726	0.854	103.792	-0.643	0.275	0.019	0.100
TOMM20L	19.519	-0.008	0.461	0.986	0.992	19.519	-0.098	0.458	0.831	0.913	19.519	-0.090	0.536	0.867	0.936
RHOB	3840.541	0.432	0.254	0.088	0.178	3840.541	0.296	0.254	0.244	0.519	3840.541	-0.137	0.293	0.641	0.805
KRT19	238.108	0.366	0.193	0.058	0.129	238.108	0.236	0.193	0.221	0.500	238.108	-0.130	0.223	0.558	0.750
LINC01550	20.185	0.488	0.671	0.468	0.602	20.185	0.454	0.670	0.498	0.711	20.185	-0.034	0.773	0.965	0.984
CCDC9B	420.297	0.573	0.181	0.002	0.008	420.297	-0.282	0.183	0.122	0.414	420.297	-0.855	0.210	0.000	0.001
TNFAIP8L3	268.452	2.209	0.373	0.000	0.000	268.452	-0.883	0.389	0.023	0.316	268.452	-3.092	0.441	0.000	0.000
GOLGA2P7	303.211	-0.551	0.191	0.004	0.016	303.211	0.053	0.187	0.776	0.881	303.211	0.604	0.219	0.006	0.046
UBE2Q2P1	12.403	0.814	0.516	0.115	0.216	12.403	-0.143	0.544	0.792	0.892	12.403	-0.957	0.612	0.118	0.307
C16orf87	332.365	-0.232	0.137	0.092	0.184	332.365	-0.167	0.136	0.220	0.499	332.365	0.065	0.159	0.685	0.834
LOC388282	83.283	-1.196	0.372	0.001	0.007	83.283	-0.265	0.360	0.462	0.687	83.283	0.931	0.428	0.030	0.131
GLTPD2	8.355	1.089	0.646	0.092	0.184	8.355	0.208	0.674	0.758	0.871	8.355	-0.881	0.754	0.243	0.474
INCA1	23.699	-0.169	0.436	0.699	0.792	23.699	0.620	0.415	0.135	0.426	23.699	0.789	0.493	0.109	0.295
C17orf100	7.939	-0.286	0.665	0.668	0.769	7.939	0.128	0.631	0.839	0.916	7.939	0.414	0.760	0.586	0.767
LRRC75A	3797.263	0.305	0.361	0.398	0.536	3797.263	0.497	0.361	0.168	0.457	3797.263	0.192	0.416	0.644	0.807
CCDC103	89.453	-0.680	0.296	0.022	0.061	89.453	0.174	0.284	0.541	0.738	89.453	0.853	0.338	0.012	0.072
YPEL2	84.448	1.333	0.454	0.003	0.014	84.448	0.480	0.459	0.295	0.563	84.448	-0.853	0.524	0.104	0.286
KRT34	28.701	-0.071	0.997	0.943	0.965	28.701	-3.409	1.067	0.001	0.297	28.701	-3.338	1.214	0.006	0.046
RGS9BP	8.060	1.313	0.569	0.021	0.060	8.060	0.541	0.598	0.366	0.617	8.060	-0.773	0.651	0.235	0.465
ZNF790	59.341	-0.774	0.279	0.006	0.021	59.341	-0.777	0.276	0.005	0.297	59.341	-0.003	0.329	0.992	0.996
BLOC1S3	152.754	0.146	0.256	0.568	0.688	152.754	0.036	0.255	0.887	0.943	152.754	-0.110	0.296	0.711	0.850
ZNF808	27.749	-0.022	0.373	0.954	0.972	27.749	0.138	0.364	0.704	0.841	27.749	0.160	0.428	0.709	0.849
ZNF888	87.215	-0.060	0.276	0.827	0.886	87.215	0.276	0.271	0.308	0.573	87.215	0.336	0.317	0.288	0.524
ZNF761	151.490	0.159	0.139	0.254	0.388	151.490	-0.005	0.139	0.971	0.985	151.490	-0.164	0.162	0.312	0.547
TMEM238	73.451	0.864	0.327	0.008	0.029	73.451	0.893	0.326	0.006	0.297	73.451	0.029	0.373	0.938	0.972
ZNF470	100.043	0.198	0.291	0.498	0.629	100.043	-0.081	0.292	0.783	0.886	100.043	-0.278	0.338	0.411	0.637
ZNF749	115.539	-0.468	0.224	0.037	0.092	115.539	-0.219	0.219	0.318	0.579	115.539	0.248	0.259	0.338	0.573
ZNF324B	146.159	-0.563	0.181	0.002	0.009	146.159	-0.204	0.175	0.245	0.520	146.159	0.359	0.209	0.086	0.255
RNF207	81.540	1.137	0.305	0.000	0.002	81.540	-0.025	0.314	0.938	0.968	81.540	-1.162	0.357	0.001	0.014
TRNP1	701.035	0.849	0.208	0.000	0.000	701.035	0.222	0.208	0.288	0.556	701.035	-0.627	0.240	0.009	0.061

TRABD2B	18.785	0.900	0.430	0.036	0.091	18.785	0.366	0.439	0.404	0.646	18.785	-0.534	0.496	0.281	0.516
DIPK1A	400.348	0.317	0.236	0.180	0.302	400.348	0.091	0.236	0.699	0.838	400.348	-0.226	0.273	0.408	0.635
NOTCH2NLA	31.223	-0.169	0.427	0.692	0.788	31.223	-0.180	0.424	0.671	0.822	31.223	-0.011	0.496	0.982	0.990
LINC01138	186.460	0.447	0.223	0.045	0.107	186.460	0.425	0.222	0.056	0.343	186.460	-0.022	0.257	0.932	0.969
LYSMD1	164.779	-0.453	0.226	0.045	0.107	164.779	0.323	0.220	0.142	0.434	164.779	0.775	0.259	0.003	0.027
HRNR	30.029	-0.232	0.642	0.718	0.808	30.029	0.115	0.636	0.857	0.927	30.029	0.347	0.740	0.639	0.804
C1orf53	35.700	0.370	0.363	0.308	0.447	35.700	-0.691	0.383	0.071	0.360	35.700	-1.061	0.436	0.015	0.085
TMEM81	31.115	0.759	0.481	0.114	0.216	31.115	-0.227	0.503	0.651	0.810	31.115	-0.987	0.569	0.083	0.250
COA6	743.066	0.338	0.284	0.233	0.366	743.066	-0.366	0.285	0.198	0.481	743.066	-0.705	0.328	0.032	0.137
SMIM26	400.420	0.706	0.241	0.003	0.015	400.420	0.209	0.242	0.387	0.633	400.420	-0.497	0.278	0.074	0.233
SNHG17	462.612	-0.798	0.397	0.044	0.106	462.612	-0.480	0.396	0.225	0.504	462.612	0.318	0.459	0.488	0.699
LOC388813	18.103	2.591	0.516	0.000	0.000	18.103	1.155	0.543	0.034	0.322	18.103	-1.437	0.577	0.013	0.077
MIR99AHG	34.282	-0.152	0.490	0.756	0.837	34.282	0.400	0.478	0.403	0.645	34.282	0.552	0.561	0.324	0.559
OGFRP1	55.768	-0.102	0.232	0.661	0.763	55.768	-0.311	0.233	0.182	0.469	55.768	-0.209	0.273	0.444	0.664
MFSD2B	7.598	1.344	0.804	0.095	0.188	7.598	1.113	0.806	0.167	0.457	7.598	-0.231	0.903	0.798	0.899
BOLA3	373.494	0.263	0.346	0.447	0.582	373.494	-0.384	0.347	0.269	0.540	373.494	-0.647	0.401	0.106	0.291
C2orf81	65.023	0.655	0.325	0.044	0.105	65.023	0.548	0.325	0.091	0.386	65.023	-0.107	0.373	0.774	0.885
C2orf68	940.384	-0.104	0.175	0.550	0.673	940.384	0.127	0.174	0.466	0.690	940.384	0.231	0.202	0.251	0.484
RHOC	2414.662	0.792	0.200	0.000	0.001	2414.662	-0.083	0.200	0.677	0.825	2414.662	-0.875	0.231	0.000	0.003
SP5	33.182	0.878	0.382	0.021	0.061	33.182	0.452	0.385	0.240	0.517	33.182	-0.426	0.439	0.332	0.567
PLEKHM3	115.128	-0.626	0.204	0.002	0.010	115.128	-0.115	0.196	0.560	0.749	115.128	0.511	0.235	0.030	0.131
SNORC	27.574	1.438	0.493	0.004	0.015	27.574	0.450	0.505	0.373	0.622	27.574	-0.988	0.569	0.083	0.249
INKA1	100.236	0.292	0.322	0.364	0.505	100.236	0.051	0.323	0.874	0.936	100.236	-0.241	0.373	0.518	0.721
VGLL3	949.567	-0.081	0.234	0.729	0.816	949.567	-0.054	0.233	0.816	0.904	949.567	0.027	0.270	0.921	0.963
ANKUB1	158.442	-0.706	0.283	0.013	0.040	158.442	-0.081	0.277	0.770	0.878	158.442	0.625	0.326	0.055	0.195
LOC389199	21.684	-0.546	0.678	0.421	0.558	21.684	-0.345	0.671	0.607	0.783	21.684	0.201	0.785	0.798	0.899
KRT86	15.799	-0.690	0.550	0.210	0.339	15.799	-0.119	0.524	0.821	0.906	15.799	0.572	0.632	0.366	0.598
SMIM20	460.852	-0.082	0.212	0.698	0.792	460.852	0.341	0.211	0.105	0.398	460.852	0.423	0.244	0.083	0.250
LOC389247	9.697	-1.065	0.719	0.138	0.249	9.697	-0.856	0.692	0.216	0.496	9.697	0.209	0.847	0.805	0.902
ANXA2R	35.954	-0.062	0.428	0.885	0.925	35.954	0.595	0.417	0.153	0.445	35.954	0.658	0.489	0.178	0.397
PROB1	75.366	0.814	0.566	0.150	0.264	75.366	0.950	0.564	0.092	0.387	75.366	0.136	0.650	0.835	0.920
PSMG4	294.393	0.720	0.240	0.003	0.012	294.393	0.089	0.242	0.713	0.846	294.393	-0.631	0.278	0.023	0.112
SAMD5	265.987	0.179	0.172	0.300	0.438	265.987	0.055	0.172	0.749	0.866	265.987	-0.124	0.199	0.535	0.733
KTN1	4716.013	-0.278	0.135	0.039	0.096	4716.013	-0.046	0.135	0.735	0.859	4716.013	0.233	0.156	0.136	0.337
GTF2IRD2B	272.512	0.262	0.424	0.536	0.661	272.512	0.651	0.423	0.124	0.416	272.512	0.388	0.489	0.427	0.650
CCZ1P-OR7E38P	92.384	0.188	0.279	0.500	0.631	92.384	-0.405	0.284	0.154	0.447	92.384	-0.593	0.328	0.070	0.226
LAMTOR4	557.076	0.629	0.204	0.002	0.010	557.076	-0.057	0.206	0.781	0.885	557.076	-0.686	0.237	0.004	0.034
LOC389641	126.974	-0.170	0.221	0.441	0.577	126.974	0.328	0.214	0.126	0.418	126.974	0.498	0.252	0.048	0.179
ASNSP1	23.109	-1.625	0.448	0.000	0.002	23.109	0.293	0.371	0.430	0.664	23.109	1.918	0.496	0.000	0.003
XKR9	17.294	0.449	0.547	0.412	0.550	17.294	0.393	0.544	0.470	0.692	17.294	-0.056	0.628	0.929	0.968
RBM12B	327.925	-0.302	0.209	0.148	0.262	327.925	-0.012	0.208	0.956	0.978	327.925	0.291	0.242	0.228	0.457
L1CAM	121.998	2.203	1.032	0.033	0.084	121.998	1.177	1.034	0.255	0.529	121.998	-1.026	1.189	0.389	0.617
GLIDR	39.153	-0.340	0.307	0.268	0.405	39.153	-0.261	0.301	0.386	0.632	39.153	0.079	0.358	0.825	0.914
LOC389765	26.103	-0.174	0.477	0.715	0.805	26.103	0.680	0.456	0.136	0.427	26.103	0.854	0.540	0.114	0.302
IER5L	682.858	1.829	0.254	0.000	0.000	682.858	-0.077	0.257	0.764	0.875	682.858	-1.905	0.294	0.000	0.000
LAD1	44.598	0.525	0.352	0.135	0.245	44.598	0.321	0.352	0.363	0.615	44.598	-0.205	0.405	0.613	0.787
LOC389831	105.234	0.396	0.233	0.089	0.180	105.234	0.194	0.233	0.406	0.647	105.234	-0.202	0.269	0.453	0.672
LOC389834	46.500	-0.470	0.363	0.196	0.322	46.500	-0.322	0.358	0.367	0.618	46.500	0.148	0.422	0.726	0.858
MAP3K15	204.017	-0.109	0.150	0.465	0.600	204.017	-0.139	0.149	0.349	0.604	204.017	-0.030	0.174	0.864	0.935
USP27X	50.559	0.101	0.340	0.767	0.846	50.559	0.435	0.332	0.191	0.476	50.559	0.335	0.388	0.389	0.617
CSAG3	735.662	1.238	0.310	0.000	0.001	735.662	0.601	0.310	0.052	0.341	735.662	-0.637	0.357	0.075	0.235
LOC389906	418.065	-0.287	0.155	0.064	0.139	418.065	0.028	0.152	0.855	0.926	418.065	0.315	0.178	0.078	0.241
C1QL3	13.899	-0.488	0.423	0.248	0.382	13.899	0.282	0.376	0.453	0.680	13.899	0.770	0.473	0.103	0.286
ACAT2	1442.403	1.204	0.183	0.000	0.000	1442.403	-0.275	0.184	0.135	0.426	1442.403	-1.479	0.212	0.000	0.000
RND3	1081.895	0.046	0.308	0.881	0.923	1081.895	-0.648	0.309	0.036	0.324	1081.895	-0.694	0.356	0.051	0.186
LAIR1	24.599	3.320	1.452	0.022	0.062	24.599	0.806	1.465	0.582	0.766	24.599	-2.514	1.676	0.133	0.333
UBAP1L	17.879	2.718	0.517	0.000	0.000	17.879	1.935	0.532	0.000	0.211	17.879	-0.783	0.539	0.146	0.353
SKOR1	12.700	0.838	0.734	0.253	0.388	12.700	-0.022	0.753	0.976	0.987	12.700	-0.861	0.857	0.315	0.551
GDPGP1	303.037	0.497	0.205	0.015	0.047	303.037	-0.195	0.208	0.348	0.604	303.037	-0.692	0.239	0.004	0.034
CES1P2	15.434	1.686	0.709	0.017	0.052	15.434	-3.190	0.998	0.001	0.297	15.434	-4.876	1.069	0.000	0.000
LAMA2	13.491	-0.591	0.461	0.200	0.326	13.491	-0.401	0.443	0.366	0.617	13.491	0.190	0.538	0.724	0.857
LAMA3	452.959	0.335	0.369	0.364	0.505	452.959	0.297	0.369	0.421	0.658	452.959	-0.038	0.426	0.929	0.968
NUDT19	536.080	-0.348	0.179	0.052	0.119	536.080	-0.115	0.178	0.519	0.724	536.080	0.233	0.207	0.259	0.494
ZNF805	81.961	0.310	0.320	0.333	0.473	81.961	-0.188	0.324	0.560	0.749	81.961	-0.498	0.373	0.181	0.400
RHOG	645.408	0.824	0.191	0.000	0.000	645.408	0.231	0.192	0.229	0.507	645.408	-0.594	0.221	0.007	0.052
FRRS1	123.726	-0.357	0.372	0.337	0.477	123.726	0.189	0.368	0.607	0.783	123.726	0.546	0.428	0.202	0.426
LAMA5	2527.787	0.623	0.391	0.111	0.212	2527.787	0.668	0.391	0.088	0.380	2527.787	0.045	0.452	0.921	0.963

LAMB1	4423.063	-0.165	0.149	0.268	0.405	4423.063	0.541	0.149	0.000	0.211	4423.063	0.706	0.172	0.000	0.001
LAMB2	5818.324	0.801	0.485	0.099	0.194	5818.324	0.708	0.485	0.144	0.436	5818.324	-0.092	0.560	0.869	0.937
PTRHD1	312.220	0.345	0.293	0.239	0.372	312.220	0.372	0.293	0.204	0.487	312.220	0.027	0.338	0.937	0.972
LAMB3	6503.774	0.853	0.258	0.001	0.006	6503.774	0.125	0.258	0.628	0.796	6503.774	-0.728	0.298	0.015	0.083
LAMC1	3590.332	0.168	0.181	0.354	0.494	3590.332	0.061	0.181	0.737	0.860	3590.332	-0.107	0.209	0.608	0.784
LAMP1	3522.320	0.323	0.194	0.097	0.191	3522.320	-0.191	0.195	0.326	0.586	3522.320	-0.514	0.225	0.022	0.108
LAMC2	532.380	0.762	0.239	0.001	0.008	532.380	-0.284	0.241	0.238	0.515	532.380	-1.047	0.277	0.000	0.003
ARHGAP1	589.639	0.081	0.102	0.423	0.560	589.639	-0.015	0.101	0.881	0.940	589.639	-0.097	0.118	0.412	0.637
LAMP2	2576.628	0.209	0.200	0.296	0.434	2576.628	0.105	0.200	0.600	0.778	2576.628	-0.104	0.231	0.652	0.813
RPSA	29858.285	0.351	0.233	0.132	0.241	29858.285	0.426	0.233	0.068	0.356	29858.285	0.075	0.269	0.780	0.889
SMPD5	10.408	1.272	0.596	0.033	0.084	10.408	0.526	0.613	0.390	0.635	10.408	-0.745	0.685	0.277	0.511
STMN1	3490.076	0.205	0.196	0.295	0.433	3490.076	-0.197	0.196	0.315	0.578	3490.076	-0.402	0.226	0.076	0.237
NCBP2L	62.000	-1.270	0.492	0.010	0.034	62.000	0.590	0.467	0.206	0.488	62.000	1.860	0.559	0.001	0.012
ELFN1	21.801	-0.823	0.383	0.032	0.082	21.801	0.258	0.338	0.445	0.675	21.801	1.081	0.429	0.012	0.072
LASP1	5062.974	0.158	0.151	0.296	0.434	5062.974	0.227	0.151	0.132	0.424	5062.974	0.070	0.174	0.689	0.837
ARHGAP4	10.914	2.326	0.901	0.010	0.034	10.914	0.357	0.946	0.706	0.842	10.914	-1.970	1.049	0.060	0.206
LBR	1223.858	-0.783	0.235	0.001	0.005	1223.858	-0.579	0.234	0.014	0.306	1223.858	0.204	0.271	0.451	0.670
LCAT	111.563	1.761	0.327	0.000	0.000	111.563	0.481	0.334	0.150	0.442	111.563	-1.280	0.378	0.001	0.010
LCN2	111.515	2.050	0.381	0.000	0.000	111.515	0.873	0.388	0.024	0.316	111.515	-1.177	0.437	0.007	0.052
LCP1	1798.612	0.374	0.272	0.169	0.289	1798.612	-0.543	0.273	0.046	0.335	1798.612	-0.917	0.315	0.004	0.032
LDHA	50054.284	-0.095	0.158	0.548	0.671	50054.284	0.213	0.158	0.178	0.466	50054.284	0.307	0.182	0.091	0.265
ARHGAP5	1852.947	-0.528	0.188	0.005	0.019	1852.947	-0.202	0.187	0.279	0.549	1852.947	0.325	0.217	0.133	0.332
LDHB	4736.998	-0.456	0.122	0.000	0.002	4736.998	-0.058	0.122	0.634	0.800	4736.998	0.398	0.141	0.005	0.040
LDLR	2137.353	1.320	0.221	0.000	0.000	2137.353	-0.356	0.222	0.109	0.401	2137.353	-1.676	0.256	0.000	0.000
LEPR	171.167	-0.369	0.547	0.500	0.631	171.167	0.686	0.544	0.207	0.489	171.167	1.055	0.630	0.094	0.270
LETM1	1142.885	-0.432	0.328	0.188	0.312	1142.885	-0.739	0.328	0.024	0.316	1142.885	-0.308	0.379	0.416	0.641
LFNG	250.247	0.961	0.374	0.010	0.035	250.247	0.240	0.375	0.522	0.725	250.247	-0.720	0.432	0.096	0.272
LGALS1	23596.550	0.909	0.305	0.003	0.013	23596.550	0.598	0.305	0.050	0.337	23596.550	-0.311	0.352	0.377	0.609
LGALS3	154.309	0.475	0.175	0.007	0.025	154.309	-0.048	0.180	0.791	0.891	154.309	-0.522	0.206	0.011	0.070
LGALS3BP	7105.684	0.921	0.157	0.000	0.000	7105.684	0.278	0.158	0.078	0.369	7105.684	-0.644	0.182	0.000	0.007
ARHGDIA	6480.407	0.063	0.273	0.817	0.880	6480.407	-0.581	0.273	0.033	0.322	6480.407	-0.644	0.315	0.041	0.161
LGALS8	1297.271	0.487	0.099	0.000	0.000	1297.271	-0.017	0.100	0.868	0.933	1297.271	-0.504	0.115	0.000	0.000
ARHGDIB	166.409	0.155	0.395	0.695	0.789	166.409	-0.544	0.397	0.171	0.460	166.409	-0.699	0.459	0.128	0.323
LIF	173.729	-1.576	0.641	0.014	0.043	173.729	-1.791	0.641	0.005	0.297	173.729	-0.214	0.743	0.773	0.885
LIFR	1363.927	-0.636	0.437	0.146	0.259	1363.927	0.246	0.437	0.573	0.758	1363.927	0.882	0.505	0.080	0.245
LIG1	476.238	-0.215	0.321	0.502	0.632	476.238	-0.462	0.321	0.149	0.442	476.238	-0.247	0.371	0.505	0.712
LIG3	449.436	-0.424	0.300	0.157	0.274	449.436	-0.495	0.299	0.098	0.393	449.436	-0.071	0.346	0.838	0.921
LIG4	176.864	-0.532	0.241	0.027	0.073	176.864	-0.465	0.239	0.052	0.341	176.864	0.067	0.280	0.811	0.906
ABLIM1	1518.091	-0.538	0.226	0.017	0.051	1518.091	0.112	0.225	0.619	0.790	1518.091	0.650	0.261	0.013	0.076
LIMK1	935.631	0.517	0.224	0.021	0.060	935.631	-0.482	0.225	0.032	0.322	935.631	-0.998	0.259	0.000	0.003
LIMK2	890.643	0.211	0.279	0.449	0.585	890.643	0.309	0.279	0.268	0.540	890.643	0.098	0.322	0.762	0.879
LIMS1	2992.722	0.029	0.113	0.797	0.867	2992.722	0.093	0.113	0.408	0.649	2992.722	0.064	0.131	0.622	0.793
LIPA	1083.654	0.622	0.294	0.034	0.087	1083.654	0.433	0.294	0.140	0.431	1083.654	-0.189	0.339	0.578	0.763
LIPE	47.935	0.208	0.435	0.633	0.741	47.935	0.270	0.433	0.533	0.733	47.935	0.062	0.502	0.902	0.954
FADS1	2386.747	0.630	0.118	0.000	0.000	2386.747	0.035	0.118	0.767	0.877	2386.747	-0.595	0.136	0.000	0.000
LLGL2	114.376	0.756	0.200	0.000	0.001	114.376	0.296	0.203	0.144	0.436	114.376	-0.460	0.231	0.047	0.175
SPRED3	30.013	0.318	0.485	0.513	0.641	30.013	-0.689	0.508	0.175	0.464	30.013	-1.006	0.581	0.083	0.250
TMEM200B	202.811	1.516	0.290	0.000	0.000	202.811	0.297	0.294	0.313	0.576	202.811	-1.220	0.335	0.000	0.005
FADS3	2281.266	1.338	0.187	0.000	0.000	2281.266	0.242	0.188	0.197	0.481	2281.266	-1.096	0.216	0.000	0.000
SLC25A35	174.842	0.371	0.210	0.077	0.160	174.842	0.015	0.211	0.943	0.971	174.842	-0.356	0.243	0.143	0.348
LLGL1	391.949	-0.189	0.174	0.279	0.416	391.949	0.157	0.172	0.362	0.614	391.949	0.346	0.201	0.085	0.253
MEX3D	1218.461	0.166	0.157	0.289	0.427	1218.461	0.240	0.157	0.126	0.418	1218.461	0.073	0.181	0.685	0.834
FAM102A	774.160	0.169	0.171	0.323	0.463	774.160	0.134	0.171	0.435	0.668	774.160	-0.036	0.198	0.856	0.931
HEATR4	27.957	0.425	0.323	0.188	0.313	27.957	-0.571	0.348	0.101	0.394	27.957	-0.996	0.394	0.011	0.071
MYO18A	605.937	0.000	0.204	1.000	1.000	605.937	0.166	0.203	0.414	0.652	605.937	0.166	0.235	0.480	0.693
MIR1915HG	187.993	0.376	0.399	0.347	0.487	187.993	0.458	0.399	0.251	0.525	187.993	0.082	0.460	0.859	0.932
LINC00999	9.335	-0.256	0.565	0.650	0.755	9.335	-0.061	0.543	0.911	0.955	9.335	0.195	0.652	0.765	0.881
BMS1P1	21.389	-0.773	0.419	0.065	0.142	21.389	0.031	0.389	0.936	0.968	21.389	0.804	0.478	0.092	0.266
LMAN1	5352.784	-0.554	0.283	0.050	0.115	5352.784	0.230	0.282	0.415	0.654	5352.784	0.784	0.326	0.016	0.089
LOC399815	31.599	-0.523	0.336	0.120	0.224	31.599	-0.158	0.322	0.624	0.793	31.599	0.365	0.388	0.347	0.581
EEF1AKMT2	212.331	-0.294	0.164	0.074	0.155	212.331	0.227	0.159	0.154	0.446	212.331	0.521	0.188	0.006	0.044
EDRF1-DT	14.733	0.242	0.511	0.635	0.743	14.733	0.681	0.492	0.167	0.456	14.733	0.439	0.576	0.447	0.666
LINC01002	9.568	-0.213	0.597	0.721	0.810	9.568	-0.360	0.595	0.545	0.740	9.568	-0.147	0.701	0.834	0.919
PCNX3	687.045	-0.351	0.342	0.304	0.443	687.045	-0.810	0.342	0.018	0.306	687.045	-0.458	0.395	0.246	0.477
MIR100HG	611.339	-0.698	0.144	0.000	0.000	611.339	-0.122	0.141	0.386	0.632	611.339	0.576	0.166	0.001	0.008
GSEC	79.978	0.689	0.448	0.124	0.230	79.978	0.433	0.449	0.335	0.594	79.978	-0.256	0.517	0.620	0.792
SNX19	502.799	-0.536	0.298	0.072	0.152	502.799	-0.582	0.297	0.050	0.337	502.799	-0.046	0.344	0.893	0.949

ARL1	1022.635	0.013	0.098	0.892	0.930	1022.635	-0.259	0.098	0.008	0.297	1022.635	-0.272	0.114	0.017	0.091
LMNA	14224.268	0.324	0.126	0.010	0.035	14224.268	0.147	0.126	0.246	0.521	14224.268	-0.177	0.146	0.225	0.453
LINC00938	229.728	-0.120	0.164	0.466	0.601	229.728	-0.038	0.162	0.817	0.904	229.728	0.082	0.190	0.666	0.822
LINC02381	173.876	0.531	0.178	0.003	0.013	173.876	0.237	0.179	0.185	0.472	173.876	-0.293	0.206	0.153	0.363
C12orf76	340.787	-0.119	0.304	0.695	0.790	340.787	0.395	0.303	0.191	0.477	340.787	0.514	0.351	0.142	0.347
LMNB1	1236.907	-0.067	0.231	0.773	0.849	1236.907	-0.347	0.231	0.133	0.424	1236.907	-0.281	0.267	0.293	0.528
FLJ22447	103.194	-0.189	0.261	0.470	0.604	103.194	-0.158	0.259	0.543	0.738	103.194	0.031	0.303	0.918	0.962
FOXN3-AS1	13.877	-0.632	0.636	0.321	0.460	13.877	0.234	0.600	0.697	0.837	13.877	0.866	0.724	0.232	0.461
DICER1-AS1	15.974	0.853	0.608	0.161	0.279	15.974	0.667	0.609	0.273	0.544	15.974	-0.186	0.695	0.789	0.895
HERC2P2	215.225	0.694	0.344	0.043	0.104	215.225	0.500	0.344	0.146	0.438	215.225	-0.194	0.396	0.625	0.795
ST20	13.218	1.115	0.518	0.031	0.081	13.218	0.616	0.530	0.245	0.520	13.218	-0.498	0.589	0.398	0.625
FAM174B	10.093	0.026	0.556	0.963	0.978	10.093	0.448	0.524	0.392	0.637	10.093	0.422	0.626	0.500	0.707
LOC400464	11.536	1.443	0.673	0.032	0.083	11.536	0.607	0.689	0.379	0.626	11.536	-0.836	0.773	0.280	0.514
LMO2	34.032	1.514	0.399	0.000	0.001	34.032	0.149	0.423	0.725	0.853	34.032	-1.365	0.469	0.004	0.033
KNOP1	634.083	-0.177	0.303	0.559	0.681	634.083	-0.546	0.304	0.072	0.361	634.083	-0.369	0.351	0.293	0.528
CRYM-AS1	16.412	-1.670	0.663	0.012	0.039	16.412	-0.932	0.627	0.137	0.428	16.412	0.738	0.768	0.337	0.571
NSMCE1-DT	8.381	-0.975	0.682	0.153	0.268	8.381	0.597	0.590	0.311	0.575	8.381	1.572	0.755	0.037	0.152
LOC400541	359.671	-0.089	0.254	0.728	0.815	359.671	0.169	0.253	0.503	0.715	359.671	0.258	0.293	0.379	0.610
C17orf97	8.410	0.191	0.520	0.713	0.804	8.410	-1.107	0.592	0.061	0.350	8.410	-1.298	0.667	0.052	0.187
MED11	72.031	0.286	0.209	0.171	0.291	72.031	0.140	0.209	0.501	0.713	72.031	-0.146	0.242	0.546	0.741
TOB1-AS1	31.127	0.269	0.654	0.681	0.779	31.127	0.626	0.648	0.334	0.593	31.127	0.357	0.751	0.634	0.801
LINC00511	9.331	1.069	0.558	0.055	0.125	9.331	1.394	0.535	0.009	0.297	9.331	0.325	0.596	0.585	0.767
LOC400627	25.486	1.661	0.505	0.001	0.006	25.486	0.232	0.535	0.665	0.818	25.486	-1.430	0.591	0.016	0.087
TEX19	16.305	0.092	0.739	0.901	0.937	16.305	0.426	0.729	0.559	0.748	16.305	0.334	0.848	0.693	0.840
LINC00909	97.171	-0.567	0.262	0.030	0.079	97.171	-0.345	0.257	0.180	0.468	97.171	0.222	0.304	0.464	0.680
C18orf65	7.301	-0.140	0.670	0.834	0.890	7.301	1.082	0.609	0.076	0.365	7.301	1.222	0.738	0.098	0.277
VMAC	9.270	0.661	0.497	0.183	0.307	9.270	0.470	0.497	0.345	0.601	9.270	-0.191	0.565	0.735	0.864
LOC400684	9.803	0.145	0.614	0.813	0.877	9.803	0.373	0.598	0.533	0.733	9.803	0.228	0.700	0.745	0.869
PRICKLE3	2269.215	0.744	0.261	0.004	0.018	2269.215	0.331	0.261	0.204	0.487	2269.215	-0.413	0.301	0.170	0.386
ZNF772	85.551	-0.572	0.253	0.024	0.066	85.551	0.202	0.242	0.405	0.646	85.551	0.773	0.289	0.008	0.054
SH2D5	262.829	0.133	0.726	0.854	0.905	262.829	-1.777	0.731	0.015	0.306	262.829	-1.910	0.843	0.023	0.112
LINC01144	6.844	-0.688	0.638	0.281	0.418	6.844	-0.354	0.603	0.557	0.747	6.844	0.334	0.741	0.652	0.813
GBP1P1	8.110	1.647	0.594	0.006	0.021	8.110	0.262	0.638	0.681	0.827	8.110	-1.385	0.696	0.047	0.174
LRRC8C-DT	38.752	-0.793	0.545	0.146	0.259	38.752	-0.487	0.539	0.366	0.617	38.752	0.306	0.631	0.628	0.797
C1orf226	12.865	-0.073	0.600	0.904	0.938	12.865	0.198	0.585	0.735	0.858	12.865	0.271	0.688	0.694	0.840
LMO7	559.984	-0.017	0.297	0.954	0.972	559.984	0.330	0.296	0.266	0.537	559.984	0.347	0.343	0.311	0.546
NBPF9	718.452	-0.131	0.237	0.580	0.698	718.452	0.192	0.236	0.418	0.656	718.452	0.323	0.274	0.238	0.468
LOC400867	680.112	-0.062	0.323	0.847	0.900	680.112	0.226	0.323	0.484	0.701	680.112	0.288	0.373	0.440	0.660
CHCHD10	1390.438	0.575	0.270	0.033	0.084	1390.438	-0.387	0.270	0.152	0.444	1390.438	-0.963	0.312	0.002	0.022
TPTEP2	24.757	0.313	0.438	0.475	0.608	24.757	0.139	0.438	0.751	0.867	24.757	-0.174	0.506	0.731	0.861
MIRLET7BHG	60.939	1.842	0.378	0.000	0.000	60.939	-0.004	0.396	0.993	0.996	60.939	-1.845	0.443	0.000	0.001
LINC00898	6.494	-0.177	1.047	0.865	0.913	6.494	0.242	1.029	0.814	0.902	6.494	0.419	1.203	0.727	0.859
LINC01376	25.840	-0.562	0.451	0.213	0.342	25.840	0.283	0.429	0.509	0.718	25.840	0.845	0.513	0.100	0.280
EML6	27.165	0.845	0.445	0.058	0.128	27.165	0.454	0.448	0.311	0.575	27.165	-0.391	0.512	0.445	0.665
PCBP1-AS1	6782.397	0.576	0.155	0.000	0.002	6782.397	0.017	0.155	0.911	0.955	6782.397	-0.558	0.179	0.002	0.020
PAIP2B	99.470	0.386	0.567	0.496	0.627	99.470	0.836	0.564	0.138	0.430	99.470	0.450	0.652	0.490	0.700
ANKRD36C	61.762	0.478	0.320	0.136	0.245	61.762	-0.219	0.327	0.503	0.715	61.762	-0.697	0.375	0.063	0.211
FLJ42351	16.582	-0.160	0.382	0.674	0.773	16.582	0.131	0.362	0.717	0.849	16.582	0.291	0.436	0.504	0.711
LINC00960	145.885	0.224	0.157	0.152	0.267	145.885	0.281	0.154	0.067	0.356	145.885	0.057	0.179	0.749	0.871
MBNL1-AS1	278.708	-0.875	0.285	0.002	0.010	278.708	-0.127	0.282	0.652	0.810	278.708	0.748	0.329	0.023	0.110
C4orf48	217.895	1.151	0.347	0.001	0.005	217.895	-0.126	0.351	0.721	0.850	217.895	-1.276	0.403	0.002	0.018
LINC01061	31.902	0.407	0.326	0.211	0.341	31.902	-0.128	0.334	0.701	0.839	31.902	-0.536	0.383	0.162	0.375
C4orf3	1545.357	0.147	0.522	0.778	0.852	1545.357	0.749	0.522	0.151	0.443	1545.357	0.602	0.602	0.317	0.553
RGS7BP	11.573	-1.003	0.818	0.220	0.351	11.573	0.242	0.775	0.755	0.869	11.573	1.245	0.932	0.181	0.400
LNPEP	546.261	-0.396	0.225	0.079	0.163	546.261	-0.001	0.224	0.995	0.997	546.261	0.395	0.260	0.129	0.325
C5orf63	41.173	0.131	0.296	0.657	0.760	41.173	0.332	0.289	0.251	0.525	41.173	0.201	0.338	0.553	0.747
LINC01011	36.878	0.812	0.614	0.186	0.310	36.878	1.147	0.609	0.060	0.350	36.878	0.335	0.702	0.633	0.801
HTATSF1P2	56.585	-1.312	0.353	0.000	0.002	56.585	-0.242	0.331	0.464	0.689	56.585	1.070	0.404	0.008	0.057
LINC00243	11.981	0.592	0.710	0.404	0.542	11.981	0.722	0.700	0.302	0.569	11.981	0.130	0.806	0.871	0.939
MCCD1	36.321	1.348	0.611	0.027	0.073	36.321	0.869	0.614	0.157	0.449	36.321	-0.479	0.702	0.495	0.703
SAPCD1	32.908	2.376	0.671	0.000	0.003	32.908	0.704	0.682	0.302	0.568	32.908	-1.672	0.773	0.031	0.134
TRAM2-AS1	104.527	-0.311	0.281	0.268	0.404	104.527	0.329	0.274	0.231	0.508	104.527	0.640	0.322	0.047	0.175
VWA5A	45.198	2.464	0.395	0.000	0.000	45.198	0.520	0.416	0.212	0.492	45.198	-1.945	0.458	0.000	0.001
ZNF815P	29.891	0.365	0.409	0.372	0.512	29.891	0.313	0.407	0.442	0.673	29.891	-0.052	0.470	0.911	0.959
GARS-DT	388.860	-1.101	0.311	0.000	0.003	388.860	-0.541	0.309	0.080	0.371	388.860	0.561	0.359	0.119	0.309
LINC00997	61.690	0.774	0.335	0.021	0.060	61.690	0.059	0.342	0.864	0.931	61.690	-0.715	0.390	0.067	0.219
RASA4CP	19.807	0.807	0.646	0.211	0.340	19.807	0.678	0.645	0.293	0.561	19.807	-0.129	0.740	0.861	0.933

LINC01446	160.518	-0.742	0.210	0.000	0.003	160.518	0.021	0.202	0.919	0.960	160.518	0.762	0.241	0.002	0.018
SMIM30	681.865	0.666	0.216	0.002	0.010	681.865	-0.168	0.217	0.441	0.672	681.865	-0.834	0.250	0.001	0.012
POT1-AS1	8.109	0.729	0.599	0.224	0.355	8.109	-0.224	0.631	0.723	0.852	8.109	-0.953	0.712	0.181	0.399
ATP6V0E2-AS1	12.063	1.217	0.442	0.006	0.022	12.063	-0.413	0.498	0.407	0.648	12.063	-1.631	0.545	0.003	0.027
RBIS	573.960	0.625	0.174	0.000	0.002	573.960	0.202	0.175	0.249	0.523	573.960	-0.423	0.201	0.036	0.147
GASAL1	13.910	1.049	0.498	0.035	0.089	13.910	-0.900	0.573	0.116	0.409	13.910	-1.949	0.631	0.002	0.022
SAMD12	18.063	1.071	0.429	0.012	0.040	18.063	-0.343	0.462	0.457	0.683	18.063	-1.415	0.515	0.006	0.047
VLDLR-AS1	57.442	0.011	0.552	0.984	0.991	57.442	0.338	0.548	0.537	0.735	57.442	0.327	0.635	0.607	0.783
LOX	6438.587	0.399	0.716	0.578	0.695	6438.587	0.881	0.716	0.219	0.498	6438.587	0.482	0.827	0.560	0.751
TOMM5	797.473	-0.084	0.336	0.802	0.869	797.473	-0.676	0.336	0.044	0.332	797.473	-0.592	0.388	0.128	0.323
ZNF658B	12.388	-0.675	0.544	0.215	0.345	12.388	-0.233	0.518	0.653	0.810	12.388	0.442	0.628	0.482	0.694
CENPP	312.307	-0.099	0.251	0.692	0.788	312.307	-0.555	0.252	0.028	0.321	312.307	-0.455	0.291	0.118	0.307
C9orf152	13.321	0.665	0.450	0.140	0.251	13.321	0.206	0.461	0.656	0.812	13.321	-0.459	0.523	0.380	0.610
SNX30	210.016	0.136	0.279	0.627	0.737	210.016	0.159	0.278	0.569	0.756	210.016	0.023	0.322	0.943	0.974
WDR38	586.467	1.105	0.376	0.003	0.014	586.467	0.589	0.377	0.118	0.410	586.467	-0.516	0.434	0.235	0.464
C9orf139	8.203	0.817	0.605	0.177	0.299	8.203	0.844	0.597	0.157	0.449	8.203	0.027	0.678	0.968	0.985
FAM166A	427.903	1.350	0.296	0.000	0.000	427.903	-0.110	0.298	0.713	0.846	427.903	-1.460	0.343	0.000	0.001
CD99P1	152.462	0.524	0.298	0.079	0.163	152.462	0.438	0.297	0.141	0.432	152.462	-0.086	0.343	0.801	0.900
ZNF674-AS1	49.297	-0.676	0.351	0.054	0.123	49.297	-0.658	0.347	0.058	0.348	49.297	0.019	0.411	0.964	0.983
LOXL1	369.900	1.972	0.365	0.000	0.000	369.900	0.614	0.367	0.094	0.389	369.900	-1.358	0.422	0.001	0.016
SLC25A53	26.391	-0.462	0.361	0.200	0.327	26.391	-0.143	0.345	0.679	0.827	26.391	0.320	0.417	0.443	0.663
GOLGA7B	180.100	1.823	0.823	0.027	0.072	180.100	1.047	0.825	0.204	0.487	180.100	-0.776	0.949	0.414	0.639
LOXL2	5307.790	0.626	0.413	0.130	0.237	5307.790	0.711	0.413	0.085	0.377	5307.790	0.085	0.477	0.859	0.932
ZNF833P	11.106	-0.361	0.488	0.459	0.594	11.106	0.503	0.442	0.256	0.529	11.106	0.863	0.544	0.113	0.301
RNF223	23.167	0.633	0.834	0.448	0.583	23.167	1.409	0.825	0.087	0.380	23.167	0.776	0.952	0.415	0.640
LDLRAD2	2429.296	2.246	0.463	0.000	0.000	2429.296	0.860	0.464	0.064	0.350	2429.296	-1.386	0.535	0.010	0.064
ARL2	682.418	0.820	0.237	0.001	0.004	682.418	0.186	0.238	0.434	0.667	682.418	-0.634	0.274	0.021	0.103
SRRD	503.443	-0.354	0.215	0.100	0.195	503.443	-0.321	0.214	0.134	0.426	503.443	0.033	0.248	0.895	0.950
LPO	28.369	-0.452	0.325	0.164	0.283	28.369	-0.320	0.315	0.309	0.574	28.369	0.132	0.379	0.728	0.859
C7orf61	15.572	3.018	0.651	0.000	0.000	15.572	1.285	0.718	0.073	0.362	15.572	-1.732	0.700	0.013	0.079
LPP	1913.004	0.632	0.163	0.000	0.001	1913.004	0.013	0.163	0.937	0.968	1913.004	-0.619	0.188	0.001	0.013
UFSP1	22.655	-1.137	0.624	0.068	0.147	22.655	-0.901	0.611	0.140	0.431	22.655	0.235	0.729	0.747	0.870
IFITM10	33.678	3.831	0.812	0.000	0.000	33.678	0.486	0.878	0.580	0.764	33.678	-3.345	0.947	0.000	0.007
ARL3	522.100	0.032	0.217	0.883	0.924	522.100	0.368	0.216	0.089	0.383	522.100	0.336	0.251	0.180	0.398
FLJ31356	9.817	-0.515	0.744	0.489	0.622	9.817	1.253	0.677	0.064	0.353	9.817	1.768	0.826	0.032	0.138
LOC403312	46.038	-0.878	0.433	0.043	0.103	46.038	-0.035	0.418	0.934	0.967	46.038	0.843	0.497	0.090	0.263
PLPP6	71.411	-0.470	0.216	0.030	0.077	71.411	-0.027	0.206	0.894	0.947	71.411	0.442	0.248	0.074	0.234
ZBTB34	109.463	-0.136	0.271	0.616	0.727	109.463	0.170	0.267	0.523	0.726	109.463	0.306	0.312	0.326	0.560
LRCH4	295.678	0.642	0.170	0.000	0.001	295.678	0.097	0.172	0.574	0.759	295.678	-0.545	0.197	0.006	0.045
LRP1	7186.159	0.715	0.911	0.433	0.569	7186.159	0.862	0.911	0.344	0.600	7186.159	0.147	1.052	0.889	0.947
LRP3	396.297	0.843	0.314	0.007	0.026	396.297	-0.751	0.317	0.018	0.306	396.297	-1.594	0.365	0.000	0.000
LRP4	600.882	-0.612	0.256	0.017	0.051	600.882	0.078	0.255	0.759	0.872	600.882	0.690	0.296	0.019	0.100
LRP6	572.369	-0.678	0.229	0.003	0.013	572.369	-0.250	0.227	0.271	0.542	572.369	0.428	0.264	0.105	0.289
CUEDC1	379.272	0.913	0.202	0.000	0.000	379.272	0.298	0.203	0.144	0.435	379.272	-0.615	0.234	0.008	0.058
LRP5	3503.823	0.380	0.138	0.006	0.022	3503.823	0.176	0.138	0.200	0.483	3503.823	-0.204	0.159	0.200	0.423
WDFY3-AS2	81.380	-0.737	0.306	0.016	0.049	81.380	0.342	0.293	0.242	0.518	81.380	1.079	0.349	0.002	0.022
CTXN1	488.409	0.502	0.261	0.055	0.123	488.409	0.091	0.262	0.728	0.855	488.409	-0.411	0.302	0.174	0.390
C6orf201	1020.373	-0.104	0.200	0.603	0.716	1020.373	0.269	0.199	0.176	0.465	1020.373	0.373	0.230	0.105	0.289
HOXB-AS3	41.357	0.505	0.302	0.095	0.188	41.357	0.197	0.305	0.518	0.723	41.357	-0.308	0.350	0.379	0.609
YY2	48.504	-0.278	0.391	0.477	0.611	48.504	-0.476	0.391	0.224	0.502	48.504	-0.198	0.456	0.664	0.821
LRPAP1	1375.198	0.615	0.116	0.000	0.000	1375.198	0.028	0.116	0.811	0.900	1375.198	-0.587	0.134	0.000	0.000
C16orf74	447.186	0.878	0.417	0.035	0.089	447.186	0.823	0.417	0.048	0.337	447.186	-0.054	0.481	0.910	0.959
LSP1	21.947	-0.248	0.869	0.776	0.851	21.947	1.083	0.848	0.201	0.484	21.947	1.331	0.991	0.180	0.398
DENND10	620.417	0.146	0.167	0.381	0.521	620.417	0.113	0.167	0.499	0.713	620.417	-0.034	0.193	0.861	0.933
LINC01194	45.193	-0.442	0.296	0.135	0.245	45.193	-0.800	0.300	0.008	0.297	45.193	-0.359	0.353	0.309	0.544
CACTIN-AS1	296.365	-0.011	0.235	0.962	0.977	296.365	-0.149	0.235	0.527	0.729	296.365	-0.138	0.272	0.613	0.787
GTF2H5	620.385	0.539	0.174	0.002	0.010	620.385	0.088	0.175	0.615	0.788	620.385	-0.451	0.201	0.025	0.117
LSS	4254.701	1.768	0.177	0.000	0.000	4254.701	0.177	0.177	0.317	0.579	4254.701	-1.591	0.204	0.000	0.000
ANKHD1-EIF4EBP3	43.473	1.805	0.432	0.000	0.000	43.473	0.582	0.444	0.190	0.476	43.473	-1.224	0.498	0.014	0.081
LTA4H	3046.375	-0.215	0.268	0.421	0.558	3046.375	0.391	0.267	0.143	0.435	3046.375	0.607	0.309	0.049	0.181
ARNT	1339.485	-0.401	0.220	0.068	0.146	1339.485	0.289	0.219	0.187	0.473	1339.485	0.690	0.253	0.006	0.049
LTBP1	43.074	1.013	0.319	0.001	0.008	43.074	0.132	0.330	0.689	0.832	43.074	-0.881	0.372	0.018	0.095
LTBP2	41.813	-0.467	0.329	0.156	0.273	41.813	-0.393	0.324	0.226	0.505	41.813	0.074	0.383	0.847	0.926
LTBP3	3369.682	0.643	0.312	0.040	0.097	3369.682	0.590	0.312	0.059	0.349	3369.682	-0.052	0.361	0.885	0.945
LTBR	4918.430	0.614	0.159	0.000	0.001	4918.430	0.143	0.159	0.369	0.619	4918.430	-0.471	0.184	0.010	0.067
BCAM	48.608	0.555	0.399	0.164	0.284	48.608	0.670	0.396	0.091	0.385	48.608	0.115	0.457	0.801	0.900
ARNTL	614.690	-1.377	0.221	0.000	0.000	614.690	-0.212	0.218	0.331	0.590	614.690	1.165	0.255	0.000	0.000

LY6E	3217.329	0.292	0.190	0.124	0.230	3217.329	0.267	0.190	0.160	0.451	3217.329	-0.025	0.219	0.908	0.958
LYN	1164.965	0.472	0.299	0.114	0.216	1164.965	-0.600	0.299	0.045	0.334	1164.965	-1.072	0.345	0.002	0.021
MIR21	19.836	2.596	0.664	0.000	0.001	19.836	0.078	0.738	0.916	0.958	19.836	-2.519	0.793	0.001	0.018
MIR210	12.386	2.065	0.919	0.025	0.068	12.386	0.539	0.953	0.571	0.757	12.386	-1.526	1.062	0.151	0.360
TACSTD2	24.230	0.430	0.369	0.244	0.377	24.230	0.233	0.370	0.528	0.729	24.230	-0.197	0.426	0.643	0.806
TM4SF1	404.232	1.663	0.206	0.000	0.000	404.232	-0.280	0.213	0.190	0.475	404.232	-1.943	0.242	0.000	0.000
EPCAM	262.221	0.546	0.191	0.004	0.017	262.221	0.294	0.191	0.125	0.416	262.221	-0.252	0.220	0.252	0.485
M6PR	2291.877	-0.215	0.228	0.347	0.487	2291.877	-0.416	0.228	0.069	0.357	2291.877	-0.201	0.264	0.446	0.666
CAPRIN1	6961.751	-0.733	0.138	0.000	0.000	6961.751	-0.081	0.137	0.558	0.748	6961.751	0.653	0.159	0.000	0.001
NBR1	2806.488	-0.252	0.159	0.113	0.214	2806.488	0.124	0.158	0.432	0.666	2806.488	0.376	0.183	0.040	0.159
MIR17HG	8.867	0.408	0.761	0.592	0.707	8.867	0.457	0.753	0.544	0.739	8.867	0.049	0.871	0.955	0.979
ARRB1	44.314	1.061	0.273	0.000	0.001	44.314	0.018	0.296	0.951	0.976	44.314	-1.043	0.326	0.001	0.017
NOMO3	89.540	-1.030	0.468	0.028	0.074	89.540	0.213	0.459	0.642	0.805	89.540	1.243	0.538	0.021	0.104
MARCKS	3432.385	0.211	0.139	0.130	0.238	3432.385	0.066	0.139	0.636	0.801	3432.385	-0.145	0.161	0.367	0.600
FNDC9	78.752	-1.034	0.552	0.061	0.134	78.752	0.651	0.540	0.228	0.506	78.752	1.685	0.633	0.008	0.055
MXD1	845.402	-0.859	0.278	0.002	0.010	845.402	-0.608	0.278	0.028	0.321	845.402	0.251	0.322	0.435	0.657
MAD2L1	980.098	-1.081	0.291	0.000	0.002	980.098	-0.314	0.290	0.279	0.549	980.098	0.767	0.336	0.022	0.109
SMAD1	347.377	-0.743	0.233	0.001	0.008	347.377	-0.116	0.230	0.615	0.788	347.377	0.628	0.269	0.019	0.100
SMAD2	1284.239	-0.296	0.133	0.026	0.071	1284.239	-0.041	0.132	0.758	0.871	1284.239	0.255	0.154	0.097	0.274
SMAD3	2086.598	-0.429	0.180	0.017	0.050	2086.598	-0.077	0.179	0.667	0.819	2086.598	0.352	0.207	0.089	0.262
SMAD4	808.684	-0.718	0.169	0.000	0.000	808.684	-0.085	0.167	0.611	0.786	808.684	0.633	0.194	0.001	0.014
ARRB2	494.791	-0.194	0.157	0.218	0.349	494.791	0.194	0.156	0.214	0.493	494.791	0.387	0.181	0.033	0.139
SMAD5	1072.511	-0.504	0.168	0.003	0.013	1072.511	-0.147	0.168	0.379	0.626	1072.511	0.356	0.194	0.067	0.219
SMAD6	202.034	0.900	0.259	0.001	0.003	202.034	-0.066	0.262	0.800	0.896	202.034	-0.966	0.301	0.001	0.016
SMAD7	410.768	-0.165	0.147	0.261	0.396	410.768	0.121	0.145	0.403	0.645	410.768	0.287	0.169	0.091	0.264
SMAD9	751.876	-0.262	0.177	0.139	0.249	751.876	-0.127	0.177	0.471	0.693	751.876	0.135	0.205	0.509	0.715
MAF	230.528	1.412	1.154	0.221	0.352	230.528	1.033	1.154	0.371	0.620	230.528	-0.379	1.332	0.776	0.887
MAFG	1169.330	-0.239	0.285	0.401	0.539	1169.330	-0.504	0.285	0.076	0.367	1169.330	-0.265	0.329	0.420	0.644
ASIC1	61.814	0.611	0.226	0.007	0.025	61.814	0.461	0.225	0.041	0.328	61.814	-0.150	0.258	0.560	0.752
ARSA	385.411	0.814	0.232	0.000	0.003	385.411	0.527	0.233	0.024	0.316	385.411	-0.287	0.268	0.283	0.518
MAGEA1	12.259	-0.910	0.494	0.066	0.142	12.259	-0.406	0.460	0.378	0.625	12.259	0.504	0.572	0.378	0.609
MAGEA2	81.393	-0.630	0.350	0.072	0.152	81.393	-0.346	0.345	0.316	0.578	81.393	0.284	0.405	0.483	0.695
MAGEA3	672.191	-0.292	0.282	0.300	0.438	672.191	-0.736	0.282	0.009	0.297	672.191	-0.444	0.326	0.173	0.390
MAGEA6	1350.960	-0.361	0.324	0.266	0.402	1350.960	-0.788	0.324	0.015	0.306	1350.960	-0.428	0.375	0.253	0.486
ARSB	174.019	0.031	0.296	0.916	0.947	174.019	-0.715	0.299	0.017	0.306	174.019	-0.746	0.345	0.031	0.133
MAGEA11	9.185	0.462	0.561	0.410	0.549	9.185	0.597	0.549	0.277	0.547	9.185	0.135	0.635	0.831	0.918
MAGEA12	1050.999	-0.247	0.208	0.234	0.367	1050.999	-0.422	0.208	0.042	0.330	1050.999	-0.174	0.240	0.468	0.684
MAGOH	875.531	0.506	0.329	0.124	0.229	875.531	-0.667	0.330	0.043	0.331	875.531	-1.172	0.380	0.002	0.022
MAK	36.102	0.369	0.441	0.403	0.541	36.102	0.368	0.438	0.401	0.644	36.102	-0.002	0.507	0.997	0.998
STS	11.639	-0.129	0.438	0.769	0.847	11.639	-0.275	0.436	0.528	0.729	11.639	-0.146	0.518	0.778	0.888
MAN1A1	343.883	0.591	0.281	0.035	0.089	343.883	-0.737	0.286	0.010	0.298	343.883	-1.328	0.328	0.000	0.001
MAN2A2	458.857	0.411	0.139	0.003	0.013	458.857	0.234	0.139	0.092	0.387	458.857	-0.178	0.160	0.267	0.502
MAN2C1	379.519	0.491	0.207	0.018	0.052	379.519	0.414	0.207	0.045	0.334	379.519	-0.077	0.239	0.746	0.870
MAN2A1	586.508	-0.447	0.264	0.090	0.181	586.508	0.053	0.263	0.840	0.917	586.508	0.500	0.305	0.100	0.281
MAN2B1	4692.149	0.323	0.264	0.220	0.350	4692.149	0.467	0.264	0.076	0.366	4692.149	0.144	0.304	0.637	0.803
MANBA	931.756	0.195	0.362	0.589	0.705	931.756	0.561	0.362	0.121	0.413	931.756	0.366	0.418	0.381	0.611
MAOA	4844.429	-0.106	0.364	0.771	0.848	4844.429	0.478	0.364	0.189	0.475	4844.429	0.584	0.420	0.165	0.380
MAP1A	20.868	1.692	0.579	0.003	0.015	20.868	-0.017	0.616	0.978	0.988	20.868	-1.709	0.684	0.012	0.075
MAP1B	649.555	0.997	0.369	0.007	0.025	649.555	-0.772	0.370	0.037	0.325	649.555	-1.769	0.427	0.000	0.001
MAP2	672.442	0.142	0.333	0.670	0.770	672.442	0.328	0.333	0.325	0.586	672.442	0.186	0.385	0.629	0.798
MAP4	11313.401	-0.059	0.153	0.700	0.793	11313.401	0.110	0.153	0.472	0.694	11313.401	0.169	0.177	0.339	0.574
MAPT	976.212	0.541	0.673	0.421	0.558	976.212	0.455	0.672	0.499	0.713	976.212	-0.086	0.776	0.912	0.960
MARK1	262.778	-0.237	0.172	0.168	0.288	262.778	-0.239	0.171	0.162	0.453	262.778	-0.002	0.199	0.992	0.996
ARSD	14.237	-0.338	0.497	0.496	0.628	14.237	0.199	0.469	0.671	0.822	14.237	0.538	0.566	0.342	0.576
MARK3	2154.284	-0.938	0.283	0.001	0.005	2154.284	-0.583	0.283	0.040	0.327	2154.284	0.355	0.327	0.278	0.512
TBC1D3B	13.427	2.143	0.757	0.005	0.019	13.427	1.637	0.764	0.032	0.322	13.427	-0.506	0.840	0.547	0.742
TBC1D3C	260.616	1.424	0.826	0.084	0.172	260.616	0.777	0.826	0.347	0.603	260.616	-0.647	0.953	0.497	0.705
MARS1	4009.594	-1.489	0.334	0.000	0.000	4009.594	-0.482	0.333	0.148	0.441	4009.594	1.007	0.385	0.009	0.061
ACBD7	53.946	0.337	0.568	0.553	0.675	53.946	0.567	0.566	0.316	0.578	53.946	0.230	0.654	0.725	0.858
CCNYL2	93.317	0.044	0.225	0.845	0.898	93.317	0.160	0.222	0.472	0.694	93.317	0.116	0.259	0.655	0.815
ZNF32-AS1	32.999	1.141	0.368	0.002	0.010	32.999	0.331	0.379	0.383	0.629	32.999	-0.810	0.426	0.058	0.200
ZNF32-AS2	23.238	0.342	0.404	0.397	0.535	23.238	0.079	0.406	0.846	0.920	23.238	-0.263	0.468	0.574	0.761
AGAP12P	13.381	1.339	0.600	0.026	0.070	13.381	0.387	0.618	0.531	0.732	13.381	-0.952	0.694	0.170	0.386
C10orf55	63.057	0.693	0.252	0.006	0.023	63.057	-0.191	0.262	0.465	0.689	63.057	-0.884	0.298	0.003	0.028
SHLD2P1	148.901	-0.043	0.197	0.828	0.887	148.901	-0.317	0.198	0.109	0.401	148.901	-0.274	0.230	0.233	0.462
DNAJC9-AS1	199.021	0.232	0.332	0.485	0.618	199.021	-0.465	0.335	0.164	0.455	199.021	-0.697	0.386	0.071	0.227
IDNK	40.661	0.123	0.313	0.695	0.790	40.661	0.251	0.308	0.416	0.654	40.661	0.128	0.360	0.723	0.856

MAT2A	2323.242	-0.181	0.214	0.397	0.535	2323.242	-0.063	0.214	0.769	0.878	2323.242	0.118	0.247	0.631	0.799
MATN1	19.199	1.053	0.416	0.011	0.037	19.199	0.829	0.418	0.047	0.336	19.199	-0.224	0.462	0.627	0.797
MATN2	17.184	1.354	0.599	0.024	0.066	17.184	1.029	0.602	0.087	0.380	17.184	-0.325	0.682	0.634	0.801
HCG14	6.673	-0.412	3.019	0.892	0.930	6.673	-0.395	3.017	0.896	0.948	6.673	0.017	3.489	0.996	0.998
HCG15	26.539	-0.624	0.662	0.345	0.486	26.539	0.614	0.643	0.340	0.598	26.539	1.238	0.756	0.102	0.283
BMS1P18	25.004	-0.267	0.401	0.505	0.635	25.004	0.073	0.386	0.850	0.923	25.004	0.340	0.460	0.460	0.677
HCG25	704.059	0.710	0.214	0.001	0.005	704.059	0.261	0.215	0.224	0.503	704.059	-0.449	0.247	0.069	0.225
HCG18	1897.516	-1.242	0.212	0.000	0.000	1897.516	-0.519	0.211	0.014	0.306	1897.516	0.724	0.244	0.003	0.029
MAX	1168.055	0.181	0.206	0.378	0.518	1168.055	-0.292	0.206	0.156	0.448	1168.055	-0.474	0.238	0.046	0.174
DENND6B	54.531	1.664	0.660	0.012	0.038	54.531	0.887	0.663	0.181	0.469	54.531	-0.778	0.759	0.306	0.541
C8orf82	850.297	-0.005	0.163	0.973	0.983	850.297	0.297	0.162	0.067	0.354	850.297	0.302	0.188	0.107	0.292
ARSL	33.584	-0.388	0.376	0.302	0.441	33.584	0.164	0.360	0.649	0.808	33.584	0.552	0.430	0.199	0.422
MAZ	4995.573	0.233	0.146	0.111	0.212	4995.573	0.193	0.146	0.186	0.473	4995.573	-0.039	0.169	0.815	0.908
MB	9.150	-0.295	0.767	0.701	0.794	9.150	0.894	0.718	0.214	0.493	9.150	1.189	0.861	0.167	0.382
PIM3	2916.693	0.682	0.176	0.000	0.001	2916.693	0.035	0.176	0.842	0.918	2916.693	-0.647	0.203	0.001	0.018
MBD1	973.174	-1.028	0.289	0.000	0.003	973.174	-0.591	0.288	0.040	0.327	973.174	0.437	0.333	0.190	0.412
MBNL1	3974.285	-0.862	0.217	0.000	0.001	3974.285	-0.105	0.217	0.628	0.796	3974.285	0.757	0.250	0.002	0.025
MBP	15.226	0.351	0.488	0.472	0.606	15.226	0.382	0.482	0.428	0.663	15.226	0.031	0.558	0.956	0.979
MC1R	22.022	1.869	0.448	0.000	0.000	22.022	0.831	0.463	0.072	0.361	22.022	-1.037	0.510	0.042	0.164
MCAM	135.214	2.237	0.224	0.000	0.000	135.214	-0.184	0.239	0.442	0.673	135.214	-2.421	0.266	0.000	0.000
MCC	623.539	-0.035	0.291	0.904	0.938	623.539	0.276	0.291	0.343	0.600	623.539	0.311	0.336	0.355	0.589
MCL1	13851.976	-0.878	0.256	0.001	0.004	13851.976	-0.409	0.256	0.110	0.403	13851.976	0.469	0.296	0.113	0.301
MCM2	2718.371	-0.930	0.374	0.013	0.041	2718.371	-0.982	0.374	0.009	0.297	2718.371	-0.052	0.432	0.904	0.956
MCM3	1866.659	-0.478	0.408	0.241	0.374	1866.659	-1.132	0.409	0.006	0.297	1866.659	-0.654	0.472	0.166	0.381
MCM4	1600.031	-0.612	0.416	0.141	0.253	1600.031	-1.279	0.416	0.002	0.297	1600.031	-0.667	0.481	0.165	0.380
MCM5	1242.462	-0.581	0.502	0.247	0.380	1242.462	-1.329	0.502	0.008	0.297	1242.462	-0.747	0.580	0.197	0.420
MCM6	782.306	-0.725	0.466	0.120	0.224	782.306	-1.427	0.467	0.002	0.297	782.306	-0.703	0.539	0.192	0.415
MCM7	2835.298	-0.522	0.353	0.140	0.251	2835.298	-0.828	0.354	0.019	0.306	2835.298	-0.306	0.408	0.454	0.672
CD46	3211.821	0.450	0.277	0.105	0.202	3211.821	0.386	0.277	0.164	0.455	3211.821	-0.065	0.320	0.840	0.923
MDFI	77.465	-0.322	0.507	0.525	0.652	77.465	-1.454	0.515	0.005	0.297	77.465	-1.132	0.594	0.057	0.198
DNAJB9	561.027	-0.422	0.323	0.191	0.316	561.027	0.185	0.320	0.564	0.752	561.027	0.607	0.372	0.103	0.285
MDH1	1870.040	0.040	0.121	0.742	0.826	1870.040	-0.089	0.121	0.461	0.687	1870.040	-0.129	0.140	0.357	0.590
MDH2	3673.824	-0.360	0.140	0.010	0.034	3673.824	-0.006	0.139	0.967	0.982	3673.824	0.355	0.161	0.028	0.126
MDK	27.336	1.329	0.491	0.007	0.025	27.336	0.592	0.501	0.237	0.514	27.336	-0.736	0.564	0.192	0.414
MDM2	945.532	-0.126	0.233	0.589	0.705	945.532	-0.293	0.233	0.208	0.490	945.532	-0.167	0.269	0.535	0.733
MDM4	258.586	0.055	0.274	0.842	0.896	258.586	-0.097	0.274	0.724	0.852	258.586	-0.151	0.317	0.633	0.800
ME1	2197.355	-0.220	0.123	0.074	0.156	2197.355	-0.069	0.123	0.576	0.761	2197.355	0.151	0.142	0.288	0.524
ME2	946.161	-0.804	0.274	0.003	0.014	946.161	-0.583	0.273	0.033	0.322	946.161	0.221	0.316	0.486	0.697
MEA1	1270.520	0.436	0.168	0.009	0.033	1270.520	0.128	0.168	0.448	0.677	1270.520	-0.309	0.194	0.112	0.299
MECP2	1459.767	0.070	0.175	0.689	0.785	1459.767	0.167	0.175	0.339	0.596	1459.767	0.097	0.202	0.631	0.799
MEF2A	1393.951	-0.521	0.249	0.036	0.090	1393.951	0.147	0.248	0.554	0.746	1393.951	0.668	0.287	0.020	0.101
MEF2C	51.244	0.580	0.283	0.040	0.097	51.244	0.611	0.280	0.029	0.322	51.244	0.031	0.322	0.924	0.964
MEF2D	1470.750	-0.288	0.161	0.074	0.155	1470.750	0.230	0.160	0.152	0.444	1470.750	0.518	0.186	0.005	0.043
ARVCF	95.120	1.294	0.405	0.001	0.007	95.120	0.800	0.407	0.049	0.337	95.120	-0.494	0.465	0.288	0.524
MEFV	142.390	0.018	0.209	0.930	0.956	142.390	0.913	0.195	0.000	0.014	142.390	0.895	0.233	0.000	0.003
MEIS1	173.945	-0.252	0.214	0.240	0.373	173.945	0.186	0.210	0.375	0.623	173.945	0.438	0.246	0.075	0.235
MEIS2	220.694	-0.180	0.166	0.280	0.418	220.694	-0.041	0.164	0.805	0.898	220.694	0.139	0.192	0.469	0.684
MAP3K1	351.903	-0.268	0.224	0.232	0.365	351.903	0.068	0.222	0.760	0.872	351.903	0.335	0.258	0.194	0.416
MAP3K3	401.138	0.191	0.197	0.332	0.472	401.138	-0.337	0.198	0.088	0.380	401.138	-0.528	0.228	0.021	0.103
MAP3K4	658.782	-0.639	0.189	0.001	0.004	658.782	-0.006	0.187	0.974	0.986	658.782	0.633	0.218	0.004	0.033
MAP3K5	469.772	0.274	0.240	0.255	0.389	469.772	-0.054	0.240	0.823	0.908	469.772	-0.327	0.278	0.238	0.468
RAB8A	1116.919	0.141	0.217	0.515	0.643	1116.919	-0.288	0.217	0.184	0.471	1116.919	-0.429	0.250	0.087	0.257
MEN1	949.505	0.131	0.107	0.219	0.349	949.505	0.008	0.107	0.941	0.970	949.505	-0.123	0.123	0.317	0.553
MEST	570.929	0.001	0.104	0.991	0.995	570.929	-0.048	0.104	0.644	0.806	570.929	-0.049	0.121	0.684	0.833
MET	6182.639	-0.525	0.237	0.027	0.073	6182.639	-0.096	0.237	0.686	0.831	6182.639	0.429	0.274	0.118	0.308
METTL1	272.606	-0.028	0.284	0.922	0.950	272.606	-0.445	0.285	0.118	0.409	272.606	-0.417	0.329	0.205	0.428
MFAP1	791.606	-0.437	0.128	0.001	0.004	791.606	-0.198	0.126	0.117	0.409	791.606	0.238	0.148	0.106	0.291
MFAP3	261.026	-0.996	0.400	0.013	0.041	261.026	-0.335	0.397	0.398	0.642	261.026	0.660	0.461	0.152	0.361
MFAP4	8.281	1.527	0.757	0.044	0.104	8.281	-0.044	0.825	0.957	0.978	8.281	-1.571	0.904	0.082	0.248
MFGE8	4515.877	0.316	0.283	0.265	0.402	4515.877	0.586	0.283	0.039	0.327	4515.877	0.270	0.327	0.409	0.635
MELTF	69.222	1.375	0.447	0.002	0.010	69.222	0.358	0.452	0.428	0.663	69.222	-1.017	0.517	0.049	0.181
MGAT1	2371.589	0.039	0.172	0.823	0.884	2371.589	0.031	0.172	0.859	0.928	2371.589	-0.008	0.199	0.968	0.985
MGAT2	1015.518	-0.178	0.099	0.071	0.152	1015.518	-0.111	0.098	0.257	0.530	1015.518	0.067	0.114	0.558	0.750
MGAT5	926.110	-0.563	0.229	0.014	0.043	926.110	-0.224	0.228	0.325	0.586	926.110	0.339	0.264	0.200	0.423
MIA2	491.541	-1.294	0.285	0.000	0.000	491.541	-0.454	0.282	0.108	0.400	491.541	0.840	0.329	0.011	0.069
KITLG	83.371	0.020	0.577	0.972	0.983	83.371	-2.053	0.595	0.001	0.253	83.371	-2.073	0.683	0.002	0.025
MGMT	12.219	0.109	0.467	0.815	0.879	12.219	0.470	0.443	0.289	0.557	12.219	0.360	0.525	0.493	0.702

MGP	36.178	1.514	0.664	0.023	0.063	36.178	0.448	0.682	0.511	0.719	36.178	-1.066	0.768	0.165	0.379
MGST1	5677.352	0.367	0.156	0.019	0.054	5677.352	0.066	0.156	0.671	0.822	5677.352	-0.301	0.180	0.095	0.271
MGST2	189.563	1.299	0.277	0.000	0.000	189.563	0.348	0.280	0.214	0.493	189.563	-0.951	0.320	0.003	0.029
MGST3	1131.677	1.359	0.259	0.000	0.000	1131.677	0.392	0.260	0.131	0.423	1131.677	-0.967	0.299	0.001	0.015
CIITA	490.354	0.330	0.134	0.014	0.043	490.354	-0.031	0.135	0.819	0.905	490.354	-0.360	0.155	0.020	0.102
CD99	8066.570	0.593	0.238	0.013	0.041	8066.570	0.060	0.238	0.801	0.896	8066.570	-0.533	0.275	0.053	0.188
ASAH1	618.195	0.601	0.134	0.000	0.000	618.195	0.048	0.135	0.725	0.853	618.195	-0.554	0.156	0.000	0.006
MICB	759.166	0.547	0.180	0.002	0.011	759.166	-0.140	0.181	0.437	0.670	759.166	-0.687	0.208	0.001	0.013
MID1	233.451	0.190	0.182	0.297	0.436	233.451	0.171	0.181	0.345	0.602	233.451	-0.019	0.210	0.929	0.968
MIF	23445.536	0.852	0.270	0.002	0.008	23445.536	0.463	0.270	0.086	0.379	23445.536	-0.389	0.312	0.212	0.437
MIP	24.540	-0.512	0.371	0.167	0.287	24.540	-0.609	0.368	0.098	0.392	24.540	-0.097	0.439	0.826	0.914
MIPEP	341.178	-0.603	0.216	0.005	0.020	341.178	-0.360	0.214	0.092	0.387	341.178	0.243	0.249	0.329	0.564
MITF	95.562	-0.026	0.299	0.931	0.956	95.562	-0.469	0.302	0.120	0.412	95.562	-0.443	0.349	0.205	0.429
ATXN3	291.536	0.366	0.170	0.031	0.081	291.536	0.131	0.170	0.442	0.673	291.536	-0.235	0.197	0.232	0.461
MKI67	4366.270	-0.928	0.198	0.000	0.000	4366.270	-0.140	0.198	0.480	0.700	4366.270	0.789	0.229	0.001	0.009
MKLN1	969.570	-0.343	0.180	0.057	0.128	969.570	-0.043	0.179	0.810	0.900	969.570	0.299	0.208	0.150	0.359
ASCL1	9.532	0.844	0.740	0.254	0.388	9.532	1.223	0.727	0.093	0.387	9.532	0.378	0.834	0.650	0.812
MLF1	651.331	0.234	0.126	0.063	0.138	651.331	-0.151	0.126	0.232	0.509	651.331	-0.385	0.146	0.008	0.058
MLH1	704.470	-0.679	0.237	0.004	0.017	704.470	-0.381	0.236	0.107	0.399	704.470	0.299	0.273	0.275	0.510
MAP3K9	333.279	0.077	0.257	0.765	0.844	333.279	0.086	0.257	0.737	0.859	333.279	0.010	0.297	0.974	0.987
MAP3K10	443.850	0.700	0.180	0.000	0.001	443.850	0.076	0.181	0.677	0.825	443.850	-0.624	0.208	0.003	0.027
MAP3K11	1034.653	0.244	0.117	0.036	0.091	1034.653	-0.004	0.117	0.973	0.985	1034.653	-0.248	0.135	0.066	0.217
KMT2A	1632.317	-0.329	0.238	0.168	0.288	1632.317	0.112	0.238	0.639	0.803	1632.317	0.440	0.275	0.110	0.295
MLLT1	2666.431	0.067	0.122	0.582	0.699	2666.431	-0.060	0.122	0.621	0.791	2666.431	-0.127	0.141	0.366	0.598
AFF1	909.473	0.243	0.344	0.481	0.614	909.473	0.319	0.344	0.354	0.608	909.473	0.076	0.397	0.848	0.926
ASCL2	55.423	0.584	0.231	0.011	0.037	55.423	0.219	0.234	0.350	0.605	55.423	-0.365	0.267	0.171	0.388
MLLT3	33.499	0.308	0.327	0.347	0.488	33.499	0.148	0.327	0.650	0.809	33.499	-0.160	0.378	0.673	0.827
AFDN	802.114	-0.614	0.187	0.001	0.006	802.114	-0.090	0.186	0.629	0.797	802.114	0.524	0.216	0.015	0.087
MLLT6	4920.748	0.111	0.169	0.510	0.640	4920.748	0.199	0.169	0.239	0.516	4920.748	0.088	0.195	0.653	0.813
FOXO4	371.130	-0.011	0.382	0.978	0.987	371.130	0.515	0.381	0.177	0.465	371.130	0.526	0.441	0.233	0.462
NR3C2	89.661	-0.155	0.275	0.574	0.693	89.661	-0.007	0.272	0.980	0.989	89.661	0.148	0.318	0.642	0.806
MME	9705.175	0.855	0.390	0.028	0.075	9705.175	0.402	0.390	0.303	0.569	9705.175	-0.453	0.451	0.315	0.550
MMP2	1732.637	1.913	0.226	0.000	0.000	1732.637	0.397	0.227	0.080	0.371	1732.637	-1.516	0.261	0.000	0.000
LHX8	31.258	-2.563	0.563	0.000	0.000	31.258	-1.278	0.510	0.012	0.305	31.258	1.285	0.646	0.047	0.175
MMP11	35.133	1.578	0.504	0.002	0.009	35.133	1.335	0.505	0.008	0.297	35.133	-0.243	0.572	0.671	0.825
MMP14	512.091	0.904	0.358	0.012	0.038	512.091	0.734	0.358	0.040	0.328	512.091	-0.170	0.413	0.680	0.831
MMP15	377.265	1.898	0.225	0.000	0.000	377.265	-0.222	0.230	0.336	0.594	377.265	-2.119	0.262	0.000	0.000
MMP16	53.259	-0.964	0.374	0.010	0.034	53.259	-0.325	0.363	0.371	0.620	53.259	0.639	0.431	0.138	0.340
MMP17	263.401	0.857	0.261	0.001	0.006	263.401	-0.076	0.264	0.774	0.880	263.401	-0.933	0.303	0.002	0.022
MMP19	174.481	0.513	0.249	0.039	0.096	174.481	0.488	0.248	0.049	0.337	174.481	-0.025	0.287	0.931	0.968
ALDH6A1	432.359	-0.249	0.560	0.656	0.760	432.359	0.708	0.559	0.206	0.488	432.359	0.957	0.646	0.139	0.342
MN1	90.940	-0.656	0.534	0.219	0.350	90.940	0.824	0.524	0.115	0.407	90.940	1.480	0.612	0.016	0.087
MNAT1	373.670	-0.209	0.182	0.253	0.387	373.670	-0.327	0.182	0.072	0.361	373.670	-0.119	0.211	0.575	0.761
MNT	387.062	0.014	0.219	0.950	0.969	387.062	0.341	0.217	0.117	0.409	387.062	0.327	0.252	0.195	0.417
MOCS1	199.157	0.057	0.165	0.729	0.816	199.157	0.084	0.164	0.607	0.783	199.157	0.027	0.191	0.888	0.946
MOCS2	799.754	0.502	0.237	0.034	0.086	799.754	-0.304	0.238	0.200	0.483	799.754	-0.807	0.274	0.003	0.030
MOV10	1234.608	0.296	0.251	0.239	0.372	1234.608	0.369	0.251	0.142	0.433	1234.608	0.073	0.290	0.801	0.900
ASL	344.913	-0.310	0.253	0.221	0.352	344.913	-0.220	0.252	0.383	0.629	344.913	0.090	0.293	0.760	0.878
MPG	1625.214	0.719	0.209	0.001	0.004	1625.214	0.449	0.209	0.032	0.322	1625.214	-0.270	0.242	0.263	0.498
MPI	1345.456	0.110	0.311	0.724	0.812	1345.456	0.508	0.311	0.102	0.394	1345.456	0.398	0.359	0.267	0.501
MPP1	646.109	-0.050	0.128	0.694	0.788	646.109	0.020	0.127	0.876	0.937	646.109	0.070	0.147	0.634	0.801
MPP2	14.783	0.605	0.392	0.122	0.228	14.783	0.017	0.405	0.966	0.982	14.783	-0.588	0.459	0.201	0.424
MPP3	86.614	0.394	0.393	0.317	0.457	86.614	-0.031	0.395	0.938	0.968	86.614	-0.425	0.456	0.351	0.585
MPST	1868.327	0.105	0.174	0.548	0.671	1868.327	0.344	0.174	0.048	0.337	1868.327	0.239	0.201	0.235	0.464
MPV17	931.372	0.336	0.194	0.084	0.171	931.372	-0.017	0.194	0.931	0.965	931.372	-0.352	0.224	0.116	0.305
MRE11	606.772	-0.453	0.137	0.001	0.006	606.772	-0.218	0.136	0.108	0.400	606.772	0.234	0.158	0.139	0.343
ABCC1	2825.220	-0.256	0.129	0.047	0.111	2825.220	-0.091	0.129	0.479	0.699	2825.220	0.165	0.149	0.269	0.504
GET3	1637.753	0.436	0.156	0.005	0.021	1637.753	-0.017	0.156	0.916	0.958	1637.753	-0.452	0.180	0.012	0.074
MXRA7	2599.810	0.353	0.220	0.108	0.208	2599.810	0.349	0.220	0.112	0.405	2599.810	-0.004	0.254	0.988	0.994
THAP7-AS1	51.153	-0.335	0.369	0.364	0.505	51.153	-0.833	0.375	0.026	0.318	51.153	-0.498	0.436	0.253	0.486
PRKCQ-AS1	14.191	-1.531	0.592	0.010	0.033	14.191	-0.666	0.545	0.222	0.501	14.191	0.865	0.681	0.204	0.428
SHLD2P3	38.555	-1.149	0.347	0.001	0.005	38.555	-0.417	0.323	0.197	0.480	38.555	0.732	0.399	0.067	0.218
LINC00857	54.534	-0.441	0.273	0.106	0.204	54.534	-0.014	0.263	0.956	0.978	54.534	0.426	0.313	0.174	0.390
LINC00863	41.144	-0.166	0.313	0.597	0.711	41.144	0.088	0.304	0.773	0.879	41.144	0.254	0.360	0.481	0.694
ASNS	90.477	-2.050	0.286	0.000	0.000	90.477	0.140	0.245	0.568	0.755	90.477	2.190	0.319	0.000	0.000
TMEM41B	322.859	0.314	0.210	0.136	0.246	322.859	-0.395	0.213	0.063	0.350	322.859	-0.709	0.245	0.004	0.034
LINC02709	14.347	-0.750	0.621	0.227	0.359	14.347	0.149	0.568	0.793	0.892	14.347	0.899	0.703	0.201	0.424

SLC22A20P	8.031	0.730	0.823	0.375	0.516	8.031	0.225	0.837	0.788	0.889	8.031	-0.504	0.953	0.597	0.775
DDX12P	75.078	0.210	0.262	0.423	0.560	75.078	0.033	0.262	0.900	0.950	75.078	-0.177	0.303	0.560	0.752
TMEM198B	82.212	0.578	0.365	0.113	0.214	82.212	0.639	0.363	0.078	0.370	82.212	0.061	0.419	0.884	0.945
ALG11	107.993	-0.762	0.328	0.020	0.058	107.993	-0.637	0.325	0.050	0.337	107.993	0.124	0.381	0.745	0.869
MZT1	933.378	-0.545	0.122	0.000	0.000	933.378	-0.109	0.120	0.367	0.617	933.378	0.437	0.140	0.002	0.021
LOC440173	198.222	0.054	0.357	0.879	0.922	198.222	0.486	0.355	0.171	0.460	198.222	0.432	0.411	0.294	0.529
CCDC88C	503.390	-0.271	0.240	0.259	0.394	503.390	-0.050	0.239	0.835	0.915	503.390	0.221	0.277	0.425	0.648
HERC2P9	207.373	0.707	0.268	0.008	0.029	207.373	0.477	0.268	0.075	0.364	207.373	-0.230	0.309	0.455	0.674
WHAMMP2	141.073	0.266	0.356	0.454	0.589	141.073	0.103	0.355	0.771	0.878	141.073	-0.163	0.411	0.692	0.838
GOLGA8B	249.504	1.483	0.500	0.003	0.013	249.504	0.501	0.502	0.318	0.579	249.504	-0.982	0.578	0.089	0.261
EIF2AK4	1847.692	-0.281	0.113	0.013	0.041	1847.692	-0.007	0.112	0.948	0.974	1847.692	0.273	0.130	0.036	0.148
CATSPER2P1	1270.467	-0.448	0.229	0.051	0.117	1270.467	-0.130	0.229	0.569	0.756	1270.467	0.317	0.265	0.231	0.459
UBL7-AS1	41.457	-0.018	0.324	0.956	0.973	41.457	0.461	0.313	0.141	0.431	41.457	0.479	0.368	0.194	0.416
GOLGA6L9	97.872	0.497	0.415	0.231	0.363	97.872	0.321	0.415	0.440	0.672	97.872	-0.176	0.479	0.713	0.850
LOC440300	14.048	-0.015	0.410	0.970	0.981	14.048	-0.297	0.414	0.474	0.696	14.048	-0.281	0.486	0.563	0.753
LOC440311	15.318	0.861	0.510	0.091	0.182	15.318	0.205	0.526	0.697	0.837	15.318	-0.656	0.593	0.268	0.503
NPIPB4	788.486	1.964	0.370	0.000	0.000	788.486	0.223	0.371	0.548	0.742	788.486	-1.741	0.427	0.000	0.001
NPIPB12	353.637	1.501	0.345	0.000	0.000	353.637	0.000	0.348	1.000	1.000	353.637	-1.501	0.400	0.000	0.004
SMG1P2	367.847	-0.836	0.303	0.006	0.022	367.847	0.137	0.299	0.647	0.808	367.847	0.973	0.348	0.005	0.042
YBX3P1	62.824	-0.172	0.265	0.515	0.643	62.824	0.363	0.253	0.152	0.444	62.824	0.536	0.301	0.075	0.236
RNASEK	93.638	0.630	0.185	0.001	0.004	93.638	0.242	0.188	0.197	0.481	93.638	-0.388	0.214	0.069	0.224
SUZ12P1	145.983	0.361	0.197	0.068	0.145	145.983	0.163	0.197	0.408	0.649	145.983	-0.197	0.228	0.387	0.615
NPEPPSP1	990.424	-0.339	0.434	0.435	0.571	990.424	0.682	0.434	0.116	0.408	990.424	1.021	0.501	0.042	0.163
PLEKHM1P1	92.181	0.736	0.274	0.007	0.027	92.181	0.403	0.276	0.144	0.435	92.181	-0.333	0.316	0.292	0.528
BAIAP2-DT	215.980	0.542	0.577	0.348	0.488	215.980	0.628	0.577	0.276	0.547	215.980	0.086	0.666	0.897	0.951
HSBP1L1	317.219	0.976	0.311	0.002	0.009	317.219	0.461	0.312	0.139	0.430	317.219	-0.515	0.359	0.151	0.360
ZNF506	34.608	-0.283	0.309	0.361	0.502	34.608	-0.509	0.311	0.102	0.394	34.608	-0.226	0.367	0.538	0.735
UQCRHL	90.585	0.351	0.275	0.201	0.328	90.585	0.250	0.274	0.362	0.614	90.585	-0.101	0.316	0.749	0.871
MICOS10	155.666	0.423	0.235	0.072	0.153	155.666	-0.140	0.238	0.556	0.747	155.666	-0.562	0.273	0.040	0.158
SLC2A1-AS1	10.058	0.782	0.629	0.214	0.344	10.058	1.295	0.607	0.033	0.322	10.058	0.514	0.696	0.460	0.677
FAM183A	12.251	0.391	0.554	0.480	0.614	12.251	-0.182	0.567	0.748	0.866	12.251	-0.573	0.651	0.378	0.609
RBM15-AS1	7.752	-0.324	0.669	0.628	0.738	7.752	0.036	0.641	0.955	0.978	7.752	0.360	0.768	0.639	0.804
BCL2L15	10.674	0.111	0.550	0.840	0.895	10.674	0.016	0.546	0.977	0.987	10.674	-0.095	0.638	0.882	0.943
NUDT4B	574.330	-1.522	0.282	0.000	0.000	574.330	-0.192	0.278	0.491	0.707	574.330	1.330	0.325	0.000	0.001
ODF3B	257.149	1.062	0.253	0.000	0.000	257.149	0.751	0.253	0.003	0.297	257.149	-0.311	0.291	0.285	0.520
LINC01123	238.676	-0.209	0.179	0.242	0.376	238.676	0.015	0.176	0.934	0.967	238.676	0.224	0.206	0.278	0.513
H3P6	870.199	0.571	0.225	0.011	0.037	870.199	-0.051	0.226	0.820	0.906	870.199	-0.622	0.260	0.017	0.092
THUMPD3-AS1	318.337	-0.007	0.142	0.960	0.976	318.337	-0.156	0.142	0.272	0.543	318.337	-0.149	0.165	0.368	0.600
EIF1B-AS1	20.639	0.428	0.513	0.404	0.542	20.639	1.080	0.499	0.030	0.322	20.639	0.653	0.579	0.260	0.494
SMIM4	153.507	0.237	0.200	0.237	0.369	153.507	-0.359	0.204	0.078	0.369	153.507	-0.596	0.235	0.011	0.070
LOC440982	41.442	0.344	0.260	0.185	0.309	41.442	0.321	0.257	0.211	0.492	41.442	-0.023	0.297	0.937	0.972
MIR570HG	226.506	1.578	0.425	0.000	0.002	226.506	0.596	0.427	0.162	0.453	226.506	-0.981	0.490	0.045	0.172
MTHFD2L	186.395	-0.409	0.208	0.049	0.114	186.395	-0.145	0.204	0.478	0.698	186.395	0.264	0.240	0.272	0.506
C4orf47	52.217	0.987	0.824	0.231	0.363	52.217	0.823	0.824	0.318	0.580	52.217	-0.165	0.948	0.862	0.934
LOC441086	6.577	-1.911	0.756	0.011	0.038	6.577	-0.408	0.619	0.510	0.718	6.577	1.503	0.848	0.076	0.238
CRSP8P	14.759	-0.573	0.576	0.320	0.460	14.759	-0.658	0.573	0.250	0.524	14.759	-0.085	0.677	0.900	0.953
NR2F1-AS1	17.374	1.818	0.484	0.000	0.001	17.374	0.390	0.513	0.448	0.677	17.374	-1.428	0.563	0.011	0.070
IRF1-AS1	158.670	0.279	0.154	0.070	0.149	158.670	-0.260	0.158	0.100	0.394	158.670	-0.539	0.182	0.003	0.029
GTF2IP20	40.990	0.338	0.416	0.417	0.554	40.990	0.309	0.414	0.455	0.682	40.990	-0.028	0.479	0.953	0.978
C6orf226	36.590	0.794	0.332	0.017	0.050	36.590	-0.094	0.346	0.785	0.888	36.590	-0.888	0.391	0.023	0.111
OOEP	47.483	0.050	0.330	0.880	0.922	47.483	0.616	0.319	0.053	0.342	47.483	0.566	0.375	0.131	0.329
FLJ46906	76.076	-0.661	0.355	0.063	0.137	76.076	0.076	0.345	0.825	0.909	76.076	0.738	0.408	0.070	0.226
RNF216P1	307.903	-0.525	0.297	0.077	0.161	307.903	-0.610	0.297	0.040	0.327	307.903	-0.084	0.344	0.806	0.903
PMS2CL	361.078	0.037	0.192	0.848	0.900	361.078	0.054	0.191	0.776	0.881	361.078	0.018	0.221	0.937	0.972
ZNRF2P1	27.229	-0.400	0.423	0.344	0.485	27.229	-0.216	0.414	0.601	0.779	27.229	0.183	0.490	0.708	0.849
RP9P	72.630	0.383	0.298	0.198	0.324	72.630	-0.019	0.301	0.949	0.975	72.630	-0.402	0.346	0.245	0.476
LOC441242	32.448	-0.133	0.430	0.757	0.838	32.448	0.075	0.422	0.859	0.928	32.448	0.208	0.495	0.674	0.827
TYW1B	17.839	-0.495	0.421	0.240	0.373	17.839	-0.089	0.399	0.823	0.908	17.839	0.405	0.484	0.402	0.629
DTX2P1-UPK3BP1-PMS2P11	93.526	0.498	0.263	0.058	0.129	93.526	0.039	0.266	0.883	0.941	93.526	-0.459	0.305	0.133	0.331
SPDYE3	64.064	-0.271	0.318	0.394	0.533	64.064	-0.241	0.316	0.446	0.677	64.064	0.031	0.369	0.934	0.970
CTAGE15	12.099	0.898	0.461	0.051	0.118	12.099	0.588	0.466	0.207	0.489	12.099	-0.310	0.522	0.552	0.746
OR2A9P	78.291	0.193	0.345	0.577	0.695	78.291	0.322	0.343	0.348	0.604	78.291	0.129	0.397	0.745	0.869
HRAT92	13.377	1.655	0.754	0.028	0.075	13.377	0.155	0.791	0.844	0.919	13.377	-1.500	0.882	0.089	0.261
LRRC24	167.878	0.465	0.214	0.030	0.078	167.878	-0.036	0.217	0.866	0.932	167.878	-0.502	0.249	0.044	0.168
SUGT1P1	9.576	0.141	0.487	0.772	0.848	9.576	0.285	0.471	0.546	0.740	9.576	0.144	0.555	0.796	0.898
ANKRD20A2	10.448	0.665	0.507	0.190	0.315	10.448	-0.018	0.527	0.973	0.985	10.448	-0.683	0.597	0.252	0.485
NRARP	196.227	0.463	0.200	0.020	0.059	196.227	0.274	0.200	0.170	0.460	196.227	-0.189	0.230	0.412	0.638

PGAM4	248.295	0.032	0.148	0.831	0.888	248.295	0.126	0.146	0.389	0.635	248.295	0.094	0.171	0.581	0.764
CDNF	54.667	-1.170	0.292	0.000	0.001	54.667	0.066	0.262	0.799	0.896	54.667	1.237	0.330	0.000	0.004
ANKRD65	34.928	0.241	1.177	0.838	0.893	34.928	0.762	1.174	0.516	0.722	34.928	0.521	1.357	0.701	0.844
ZFAS1	4085.523	0.139	0.358	0.697	0.791	4085.523	0.704	0.358	0.049	0.337	4085.523	0.565	0.413	0.172	0.388
EMC3-AS1	94.614	0.849	0.337	0.012	0.039	94.614	0.081	0.342	0.814	0.902	94.614	-0.769	0.391	0.050	0.182
UQCRBP1	8.458	1.870	0.659	0.005	0.018	8.458	0.494	0.723	0.494	0.709	8.458	-1.376	0.764	0.072	0.229
DPY19L2P3	25.210	-1.181	0.573	0.039	0.096	25.210	-0.505	0.552	0.360	0.612	25.210	0.677	0.661	0.306	0.542
STAG3L3	108.429	0.343	0.190	0.070	0.150	108.429	-0.084	0.192	0.663	0.817	108.429	-0.427	0.221	0.054	0.192
STAG3L2	79.328	0.119	0.199	0.549	0.672	79.328	0.171	0.196	0.384	0.630	79.328	0.051	0.229	0.823	0.913
MYO1B	2307.072	-0.930	0.329	0.005	0.019	2307.072	-0.730	0.329	0.027	0.318	2307.072	0.200	0.381	0.600	0.778
MSH2	364.952	-0.360	0.284	0.205	0.333	364.952	-0.589	0.284	0.038	0.327	364.952	-0.228	0.329	0.488	0.698
MSH3	683.193	-0.266	0.168	0.113	0.215	683.193	0.101	0.167	0.543	0.739	683.193	0.367	0.194	0.058	0.201
MSH5	113.607	0.561	0.428	0.190	0.315	113.607	0.575	0.428	0.179	0.467	113.607	0.014	0.493	0.978	0.989
ASPH	9354.418	-0.651	0.207	0.002	0.008	9354.418	-0.087	0.206	0.673	0.823	9354.418	0.564	0.238	0.018	0.095
ASS1	15247.056	0.311	0.453	0.493	0.625	15247.056	0.746	0.453	0.099	0.394	15247.056	0.436	0.523	0.405	0.631
ARHGEF35	582.500	0.525	0.132	0.000	0.001	582.500	0.120	0.133	0.366	0.617	582.500	-0.405	0.153	0.008	0.057
CBWD3	114.280	0.342	0.406	0.398	0.537	114.280	-0.383	0.410	0.350	0.605	114.280	-0.726	0.472	0.125	0.318
C9orf129	25.275	0.003	0.880	0.997	0.999	25.275	-0.488	0.883	0.581	0.764	25.275	-0.491	1.021	0.631	0.799
PALM2AKAP2	5916.286	-0.245	0.305	0.421	0.558	5916.286	-0.733	0.305	0.016	0.306	5916.286	-0.487	0.353	0.167	0.382
MSN	12597.403	0.094	0.144	0.513	0.642	12597.403	-0.043	0.144	0.763	0.874	12597.403	-0.137	0.166	0.408	0.634
MSRA	76.722	0.879	0.237	0.000	0.002	76.722	0.312	0.242	0.197	0.481	76.722	-0.568	0.273	0.038	0.153
MST1	53.044	1.191	0.561	0.034	0.086	53.044	1.357	0.559	0.015	0.306	53.044	0.166	0.641	0.796	0.898
MST1R	25.476	0.695	0.310	0.025	0.068	25.476	0.276	0.317	0.384	0.630	25.476	-0.420	0.358	0.241	0.472
MSX1	69.422	0.303	0.357	0.397	0.535	69.422	-0.550	0.366	0.133	0.424	69.422	-0.853	0.420	0.042	0.165
MSX2	32.915	0.852	0.278	0.002	0.010	32.915	-0.126	0.303	0.677	0.825	32.915	-0.978	0.335	0.004	0.032
MT1E	1853.076	1.571	0.239	0.000	0.000	1853.076	0.355	0.240	0.139	0.430	1853.076	-1.216	0.277	0.000	0.000
MT1G	59.561	1.183	0.865	0.171	0.292	59.561	1.206	0.863	0.162	0.453	59.561	0.023	0.991	0.981	0.990
GGNBP1	90.029	-0.912	0.468	0.051	0.117	90.029	-0.644	0.464	0.165	0.455	90.029	0.268	0.541	0.621	0.792
MT1X	14407.157	2.398	0.340	0.000	0.000	14407.157	0.898	0.340	0.008	0.297	14407.157	-1.500	0.392	0.000	0.003
MT2A	40272.278	1.543	0.259	0.000	0.000	40272.278	0.157	0.259	0.545	0.739	40272.278	-1.386	0.299	0.000	0.000
MTAP	21.912	0.603	0.346	0.081	0.168	21.912	0.169	0.354	0.632	0.799	21.912	-0.434	0.402	0.281	0.515
MTCP1	20.334	0.321	0.464	0.490	0.623	20.334	0.050	0.466	0.915	0.958	20.334	-0.271	0.538	0.615	0.789
MTF1	372.759	-0.582	0.288	0.043	0.103	372.759	-0.538	0.287	0.061	0.350	372.759	0.044	0.333	0.894	0.950
NUDT1	367.348	0.272	0.283	0.336	0.477	367.348	-0.223	0.283	0.431	0.665	367.348	-0.495	0.327	0.130	0.327
MTHFD1	3108.608	-0.690	0.255	0.007	0.025	3108.608	-0.671	0.255	0.008	0.297	3108.608	0.020	0.294	0.947	0.975
MTHFR	220.591	0.842	0.208	0.000	0.001	220.591	0.155	0.211	0.461	0.687	220.591	-0.687	0.241	0.004	0.037
MTIF2	1170.478	-0.407	0.134	0.002	0.011	1170.478	-0.199	0.133	0.134	0.426	1170.478	0.208	0.154	0.178	0.397
MTM1	108.078	-0.553	0.255	0.030	0.079	108.078	-0.124	0.249	0.618	0.790	108.078	0.429	0.294	0.145	0.352
MYO1F	15.633	1.080	0.624	0.084	0.171	15.633	1.345	0.615	0.029	0.322	15.633	0.265	0.700	0.705	0.847
MTR	1097.003	-0.152	0.142	0.284	0.422	1097.003	-0.010	0.142	0.941	0.970	1097.003	0.142	0.164	0.387	0.616
RNR1	21218.425	2.255	0.331	0.000	0.000	21218.425	0.478	0.331	0.149	0.442	21218.425	-1.777	0.383	0.000	0.000
RNR2	123823.053	1.765	0.345	0.000	0.000	123823.053	0.525	0.345	0.129	0.421	123823.053	-1.240	0.399	0.002	0.021
MTRR	1083.708	-1.231	0.309	0.000	0.001	1083.708	-0.656	0.308	0.033	0.322	1083.708	0.575	0.356	0.107	0.291
MTX1	1059.994	0.531	0.222	0.017	0.050	1059.994	0.012	0.222	0.956	0.978	1059.994	-0.519	0.256	0.043	0.165
MUC1	236.898	1.772	0.760	0.020	0.057	236.898	1.233	0.761	0.105	0.398	236.898	-0.538	0.877	0.539	0.736
MUC4	14.114	0.381	2.092	0.856	0.906	14.114	0.363	2.089	0.862	0.930	14.114	-0.018	2.413	0.994	0.996
MUC5AC	13.057	0.693	0.474	0.144	0.257	13.057	0.023	0.492	0.963	0.981	13.057	-0.670	0.557	0.229	0.457
MUC6	11.334	1.632	0.523	0.002	0.009	11.334	0.371	0.562	0.509	0.718	11.334	-1.261	0.609	0.039	0.155
TRIM37	974.603	-1.028	0.287	0.000	0.003	974.603	-0.431	0.287	0.133	0.424	974.603	0.596	0.332	0.072	0.230
MMUT	697.571	-0.223	0.147	0.129	0.236	697.571	0.079	0.145	0.585	0.768	697.571	0.302	0.169	0.074	0.234
MUTYH	152.645	-0.008	0.221	0.972	0.983	152.645	-0.200	0.221	0.365	0.617	152.645	-0.193	0.257	0.453	0.672
MVD	916.201	2.030	0.129	0.000	0.000	916.201	-0.018	0.133	0.895	0.948	916.201	-2.048	0.151	0.000	0.000
MVK	594.294	1.759	0.123	0.000	0.000	594.294	0.072	0.128	0.572	0.758	594.294	-1.687	0.144	0.000	0.000
MX1	90.511	1.318	0.277	0.000	0.000	90.511	0.549	0.281	0.050	0.338	90.511	-0.768	0.319	0.016	0.088
MX2	14.216	2.974	0.458	0.000	0.000	14.216	0.404	0.532	0.447	0.677	14.216	-2.570	0.542	0.000	0.000
MXI1	2741.295	0.114	0.361	0.752	0.835	2741.295	0.441	0.360	0.221	0.500	2741.295	0.327	0.416	0.432	0.654
MYB	27.271	-0.146	0.299	0.625	0.735	27.271	-0.598	0.309	0.053	0.341	27.271	-0.452	0.361	0.211	0.436
MYBL1	116.367	-0.361	0.279	0.195	0.320	116.367	-0.048	0.275	0.863	0.930	116.367	0.314	0.322	0.329	0.563
MYBPC1	550.825	-0.288	0.713	0.686	0.783	550.825	0.980	0.712	0.168	0.458	550.825	1.268	0.822	0.123	0.316
MYBL2	1923.912	-0.593	0.461	0.198	0.324	1923.912	-1.078	0.461	0.019	0.306	1923.912	-0.485	0.533	0.363	0.595
MYC	12871.394	-1.026	0.216	0.000	0.000	12871.394	-0.179	0.216	0.406	0.647	12871.394	0.846	0.250	0.001	0.010
MYD88	448.480	0.466	0.133	0.000	0.003	448.480	-0.169	0.135	0.210	0.491	448.480	-0.635	0.155	0.000	0.001
GADD45B	2947.039	0.775	0.217	0.000	0.003	2947.039	0.084	0.217	0.697	0.837	2947.039	-0.690	0.250	0.006	0.045
MYH3	17.698	1.347	0.461	0.003	0.015	17.698	0.076	0.489	0.876	0.937	17.698	-1.271	0.542	0.019	0.099
MYH9	39010.047	-0.084	0.088	0.341	0.482	39010.047	0.030	0.088	0.735	0.859	39010.047	0.113	0.101	0.264	0.498
MYH10	1181.946	-0.263	0.184	0.154	0.270	1181.946	0.025	0.184	0.893	0.946	1181.946	0.287	0.213	0.176	0.394
MYH11	574.357	-0.249	0.164	0.128	0.235	574.357	0.108	0.162	0.506	0.716	574.357	0.357	0.189	0.058	0.201

ZFHX3	1763.508	0.000	0.178	1.000	1.000	1763.508	0.117	0.177	0.508	0.717	1763.508	0.117	0.205	0.567	0.757
MYL5	604.303	1.009	0.248	0.000	0.001	604.303	0.125	0.249	0.618	0.789	604.303	-0.884	0.287	0.002	0.022
MYL6	7227.971	0.813	0.173	0.000	0.000	7227.971	0.090	0.173	0.603	0.781	7227.971	-0.723	0.200	0.000	0.005
MYLK	40.075	1.425	0.313	0.000	0.000	40.075	0.421	0.326	0.196	0.480	40.075	-1.005	0.363	0.006	0.044
MYO1C	290.074	0.420	0.168	0.012	0.040	290.074	0.289	0.167	0.085	0.376	290.074	-0.131	0.193	0.498	0.706
MYO1D	1801.086	0.365	0.247	0.139	0.249	1801.086	0.382	0.247	0.121	0.413	1801.086	0.017	0.285	0.952	0.978
MYO1E	1848.677	-0.084	0.197	0.668	0.769	1848.677	-0.287	0.197	0.146	0.438	1848.677	-0.202	0.228	0.374	0.606
MYO5A	35.341	-0.008	0.293	0.979	0.987	35.341	-0.057	0.290	0.844	0.919	35.341	-0.049	0.340	0.885	0.945
MYO5B	64.796	0.246	0.198	0.214	0.344	64.796	0.209	0.195	0.285	0.554	64.796	-0.037	0.228	0.870	0.937
MYO6	935.754	0.146	0.120	0.224	0.355	935.754	-0.247	0.120	0.040	0.328	935.754	-0.392	0.139	0.005	0.040
MYO9A	567.211	-0.435	0.217	0.045	0.106	567.211	-0.043	0.216	0.841	0.917	567.211	0.392	0.251	0.117	0.307
MYO9B	1795.245	0.004	0.120	0.972	0.983	1795.245	0.072	0.120	0.547	0.741	1795.245	0.068	0.138	0.624	0.794
MYO10	3407.303	-0.689	0.166	0.000	0.000	3407.303	-0.223	0.166	0.178	0.466	3407.303	0.465	0.192	0.015	0.086
PPP1R12A	1072.372	-0.268	0.168	0.111	0.211	1072.372	-0.233	0.168	0.165	0.455	1072.372	0.035	0.194	0.857	0.931
ATF1	625.793	-0.894	0.190	0.000	0.000	625.793	-0.247	0.188	0.188	0.474	625.793	0.647	0.219	0.003	0.030
PPP1R12B	212.028	-0.261	0.389	0.503	0.632	212.028	0.262	0.387	0.499	0.712	212.028	0.523	0.449	0.244	0.475
NAB1	1363.429	-0.642	0.146	0.000	0.000	1363.429	-0.147	0.145	0.313	0.576	1363.429	0.496	0.169	0.003	0.031
NAB2	1103.960	0.533	0.207	0.010	0.035	1103.960	0.325	0.208	0.117	0.409	1103.960	-0.208	0.239	0.385	0.614
NACA	11242.138	0.870	0.279	0.002	0.009	11242.138	0.364	0.279	0.192	0.477	11242.138	-0.506	0.322	0.116	0.305
NAGA	396.828	0.190	0.161	0.239	0.372	396.828	-0.120	0.162	0.456	0.683	396.828	-0.310	0.187	0.097	0.275
NAGLU	564.198	0.228	0.139	0.101	0.197	564.198	0.293	0.138	0.034	0.322	564.198	0.065	0.160	0.686	0.835
ATF3	456.341	-0.400	0.491	0.415	0.553	456.341	-1.469	0.493	0.003	0.297	456.341	-1.069	0.570	0.061	0.206
HNRNPM	6894.681	-0.436	0.257	0.090	0.181	6894.681	-0.644	0.257	0.012	0.305	6894.681	-0.208	0.297	0.484	0.696
NAIP	376.145	0.551	0.430	0.200	0.327	376.145	0.479	0.430	0.265	0.537	376.145	-0.072	0.496	0.885	0.945
NAP1L1	22339.428	0.253	0.153	0.098	0.194	22339.428	0.146	0.153	0.341	0.598	22339.428	-0.107	0.177	0.545	0.740
NAP1L2	15.774	0.740	0.455	0.104	0.201	15.774	-0.636	0.499	0.203	0.486	15.774	-1.376	0.558	0.014	0.080
NAP1L3	53.194	0.185	0.307	0.547	0.670	53.194	-0.007	0.308	0.982	0.989	53.194	-0.192	0.356	0.589	0.770
NAP1L4	4327.262	-0.134	0.187	0.474	0.608	4327.262	-0.318	0.187	0.089	0.383	4327.262	-0.184	0.216	0.394	0.622
NARS1	3494.747	-0.880	0.271	0.001	0.006	3494.747	-0.633	0.271	0.019	0.306	3494.747	0.247	0.313	0.430	0.652
NASP	1706.286	-0.072	0.291	0.804	0.871	1706.286	-0.622	0.291	0.033	0.322	1706.286	-0.550	0.337	0.102	0.285
ATF4	9559.722	-0.422	0.225	0.060	0.133	9559.722	-0.197	0.225	0.380	0.626	9559.722	0.225	0.260	0.387	0.615
NBL1	375.927	1.058	0.211	0.000	0.000	375.927	0.214	0.214	0.315	0.578	375.927	-0.844	0.245	0.001	0.009
NUBP1	323.958	0.620	0.183	0.001	0.004	323.958	-0.203	0.186	0.274	0.545	323.958	-0.824	0.213	0.000	0.003
NBN	712.263	-0.991	0.159	0.000	0.000	712.263	-0.389	0.157	0.013	0.305	712.263	0.603	0.183	0.001	0.013
NCAM1	354.888	0.046	0.214	0.830	0.888	354.888	0.185	0.213	0.385	0.631	354.888	0.139	0.246	0.573	0.760
NCAM2	268.398	-0.631	0.365	0.084	0.171	268.398	0.345	0.362	0.340	0.598	268.398	0.977	0.420	0.020	0.102
NCBP1	1004.639	-1.014	0.334	0.002	0.011	1004.639	-0.889	0.334	0.008	0.297	1004.639	0.125	0.386	0.746	0.869
NCF2	36.379	4.759	0.541	0.000	0.000	36.379	0.218	0.758	0.774	0.879	36.379	-4.541	0.692	0.000	0.000
NCK1	1324.693	-0.430	0.184	0.019	0.056	1324.693	0.133	0.183	0.466	0.690	1324.693	0.564	0.212	0.008	0.056
NCL	18405.478	-0.514	0.264	0.052	0.118	18405.478	-0.552	0.264	0.037	0.324	18405.478	-0.038	0.305	0.901	0.954
NDUFA1	880.810	1.039	0.244	0.000	0.000	880.810	0.139	0.245	0.570	0.756	880.810	-0.899	0.282	0.001	0.017
NDUFA2	883.183	1.138	0.239	0.000	0.000	883.183	0.280	0.239	0.241	0.517	883.183	-0.858	0.276	0.002	0.021
NDUFA3	1275.249	1.368	0.372	0.000	0.002	1275.249	0.056	0.373	0.881	0.940	1275.249	-1.312	0.430	0.002	0.024
NDUFA4	2640.774	0.502	0.204	0.014	0.043	2640.774	-0.025	0.204	0.901	0.950	2640.774	-0.527	0.235	0.025	0.117
NDUFA5	1268.808	-0.001	0.133	0.993	0.996	1268.808	-0.350	0.133	0.009	0.297	1268.808	-0.348	0.154	0.023	0.112
ACLY	6309.651	0.456	0.176	0.009	0.033	6309.651	-0.309	0.176	0.079	0.370	6309.651	-0.765	0.203	0.000	0.003
NDUFA6	3601.975	0.626	0.249	0.012	0.039	3601.975	-0.256	0.249	0.304	0.570	3601.975	-0.882	0.288	0.002	0.023
NDUFA7	608.140	0.893	0.290	0.002	0.010	608.140	0.108	0.291	0.711	0.845	608.140	-0.785	0.335	0.019	0.099
NDUFA8	1259.090	0.313	0.198	0.114	0.216	1259.090	-0.049	0.198	0.804	0.897	1259.090	-0.362	0.229	0.114	0.302
NEB	8.193	-0.104	0.781	0.894	0.931	8.193	1.086	0.709	0.125	0.417	8.193	1.190	0.859	0.166	0.381
NDUFA9	1142.030	-0.265	0.207	0.201	0.328	1142.030	-0.406	0.207	0.050	0.337	1142.030	-0.142	0.239	0.554	0.747
NDUFA10	1006.054	-0.039	0.106	0.716	0.806	1006.054	-0.082	0.106	0.438	0.671	1006.054	-0.043	0.122	0.724	0.857
NDUFAB1	977.131	-0.431	0.227	0.058	0.129	977.131	-0.373	0.227	0.100	0.394	977.131	0.058	0.262	0.825	0.914
NDUFB1	349.046	0.902	0.273	0.001	0.006	349.046	-0.049	0.275	0.858	0.928	349.046	-0.951	0.316	0.003	0.026
NDUFB2	1114.282	0.402	0.182	0.027	0.073	1114.282	-0.149	0.183	0.413	0.652	1114.282	-0.551	0.211	0.009	0.061
NDUFB3	489.125	0.564	0.212	0.008	0.028	489.125	-0.240	0.214	0.261	0.534	489.125	-0.804	0.246	0.001	0.014
ATIC	2081.767	-0.144	0.176	0.413	0.551	2081.767	-0.188	0.176	0.285	0.554	2081.767	-0.044	0.203	0.828	0.915
NDUFB4	1810.309	0.790	0.251	0.002	0.009	1810.309	0.317	0.252	0.208	0.490	1810.309	-0.473	0.290	0.103	0.286
NDUFB5	1961.882	-0.458	0.160	0.004	0.017	1961.882	0.048	0.159	0.763	0.874	1961.882	0.506	0.185	0.006	0.047
NDUFB6	756.558	0.343	0.245	0.162	0.281	756.558	-0.384	0.246	0.119	0.411	756.558	-0.726	0.284	0.010	0.068
NDUFB7	978.381	0.519	0.210	0.013	0.042	978.381	0.208	0.210	0.322	0.583	978.381	-0.312	0.242	0.198	0.421
NDUFB8	96.334	0.673	0.261	0.010	0.034	96.334	-0.065	0.267	0.807	0.899	96.334	-0.739	0.304	0.015	0.086
NDUFB9	2181.213	0.318	0.196	0.104	0.201	2181.213	-0.050	0.196	0.800	0.896	2181.213	-0.368	0.226	0.104	0.287
NDUFB10	1727.102	0.510	0.241	0.034	0.087	1727.102	0.062	0.241	0.796	0.894	1727.102	-0.448	0.278	0.107	0.292
NDUFC1	165.640	0.829	0.252	0.001	0.006	165.640	0.534	0.253	0.035	0.322	165.640	-0.295	0.290	0.308	0.544
NDUFC2	241.644	0.432	0.206	0.036	0.090	241.644	-0.050	0.208	0.810	0.900	241.644	-0.482	0.239	0.044	0.169
NDUFS1	1287.946	-0.542	0.179	0.002	0.011	1287.946	-0.483	0.179	0.007	0.297	1287.946	0.058	0.207	0.779	0.888

ATM	455.529	-0.190	0.244	0.436	0.572	455.529	0.170	0.243	0.482	0.701	455.529	0.361	0.281	0.200	0.423
NDUFS2	1726.768	-0.246	0.135	0.068	0.146	1726.768	0.037	0.135	0.786	0.888	1726.768	0.283	0.156	0.069	0.225
NDUFS3	871.666	0.394	0.200	0.049	0.114	871.666	-0.090	0.201	0.652	0.810	871.666	-0.484	0.232	0.036	0.149
NDUFV1	2890.974	-0.131	0.129	0.310	0.449	2890.974	0.175	0.129	0.176	0.465	2890.974	0.306	0.149	0.040	0.160
NDUFS4	1087.698	0.303	0.187	0.105	0.202	1087.698	0.269	0.187	0.150	0.442	1087.698	-0.034	0.216	0.874	0.939
NDUFS5	2645.189	1.083	0.260	0.000	0.000	2645.189	-0.117	0.261	0.653	0.811	2645.189	-1.200	0.301	0.000	0.002
NDUFS6	2185.967	0.731	0.312	0.019	0.056	2185.967	-0.406	0.312	0.193	0.478	2185.967	-1.137	0.361	0.002	0.019
NDUFS8	962.516	0.658	0.295	0.026	0.070	962.516	-0.040	0.296	0.893	0.946	962.516	-0.698	0.341	0.041	0.161
NDUFV2	1380.331	0.665	0.225	0.003	0.014	1380.331	0.058	0.226	0.796	0.894	1380.331	-0.606	0.260	0.020	0.101
RERE	2350.421	-0.227	0.126	0.071	0.151	2350.421	0.081	0.125	0.521	0.725	2350.421	0.308	0.145	0.034	0.144
NDUFV3	726.481	0.220	0.179	0.220	0.351	726.481	-0.067	0.180	0.709	0.844	726.481	-0.287	0.207	0.167	0.382
DRG1	1428.373	-0.150	0.107	0.163	0.282	1428.373	-0.013	0.107	0.902	0.951	1428.373	0.137	0.124	0.270	0.505
NEDD4	2284.202	-1.426	0.265	0.000	0.000	2284.202	-0.386	0.265	0.144	0.436	2284.202	1.040	0.306	0.001	0.010
SEPTIN2	4800.761	-0.390	0.153	0.011	0.036	4800.761	-0.328	0.153	0.032	0.322	4800.761	0.063	0.177	0.724	0.857
RPL10A	25934.891	-0.173	0.282	0.540	0.665	25934.891	0.542	0.282	0.055	0.342	25934.891	0.715	0.326	0.028	0.126
NEDD8	322.293	0.598	0.307	0.052	0.118	322.293	-0.055	0.309	0.858	0.928	322.293	-0.653	0.356	0.066	0.218
NEDD9	76.182	1.338	0.323	0.000	0.000	76.182	0.053	0.337	0.875	0.937	76.182	-1.285	0.378	0.001	0.010
LRRC37A2	114.358	0.716	0.211	0.001	0.004	114.358	0.045	0.216	0.833	0.914	114.358	-0.670	0.246	0.007	0.049
SPIN2B	102.198	0.349	0.257	0.176	0.297	102.198	0.001	0.259	0.997	0.999	102.198	-0.347	0.299	0.245	0.476
F8A2	494.578	0.844	0.208	0.000	0.001	494.578	0.276	0.209	0.186	0.473	494.578	-0.568	0.240	0.018	0.095
ATOX1	408.748	0.480	0.248	0.053	0.121	408.748	-0.046	0.249	0.854	0.925	408.748	-0.526	0.288	0.067	0.219
NEK1	170.920	-0.403	0.224	0.072	0.152	170.920	0.052	0.220	0.813	0.902	170.920	0.455	0.258	0.077	0.240
NEK2	858.966	-0.341	0.299	0.254	0.388	858.966	0.153	0.298	0.608	0.783	858.966	0.494	0.345	0.152	0.361
NEK3	82.668	-0.196	0.229	0.393	0.532	82.668	-0.297	0.228	0.194	0.478	82.668	-0.101	0.267	0.706	0.847
NELL2	21.406	-0.281	0.360	0.436	0.572	21.406	0.003	0.346	0.992	0.996	21.406	0.284	0.414	0.493	0.702
NEO1	469.258	-0.120	0.258	0.642	0.749	469.258	0.450	0.256	0.079	0.370	469.258	0.570	0.297	0.055	0.194
NEU1	3331.327	1.821	0.111	0.000	0.000	3331.327	0.419	0.112	0.000	0.202	3331.327	-1.402	0.129	0.000	0.000
ATP1A1	12042.394	0.125	0.075	0.094	0.187	12042.394	0.135	0.075	0.071	0.359	12042.394	0.010	0.086	0.909	0.958
NF1	1768.186	-0.422	0.203	0.037	0.092	1768.186	-0.026	0.202	0.898	0.949	1768.186	0.396	0.234	0.090	0.263
ATP1A2	181.612	-2.295	1.155	0.047	0.110	181.612	0.399	1.150	0.728	0.855	181.612	2.694	1.332	0.043	0.167
NF2	749.481	0.307	0.245	0.211	0.340	749.481	-0.486	0.246	0.048	0.337	749.481	-0.793	0.284	0.005	0.042
NFATC1	37.589	0.596	0.294	0.043	0.103	37.589	0.506	0.293	0.084	0.376	37.589	-0.090	0.335	0.788	0.894
NFATC2	9.597	-0.271	0.623	0.663	0.765	9.597	0.278	0.583	0.633	0.800	9.597	0.550	0.706	0.436	0.657
NFIA	677.928	0.520	0.225	0.021	0.059	677.928	0.149	0.225	0.509	0.718	677.928	-0.371	0.260	0.153	0.363
NFATC3	664.740	-0.511	0.142	0.000	0.002	664.740	-0.175	0.141	0.213	0.493	664.740	0.336	0.164	0.041	0.160
NFE2	11.094	3.172	0.801	0.000	0.001	11.094	1.583	0.826	0.055	0.343	11.094	-1.589	0.893	0.075	0.236
NFE2L1	7624.110	-0.160	0.131	0.222	0.353	7624.110	0.015	0.131	0.907	0.954	7624.110	0.175	0.151	0.247	0.478
NFE2L2	2053.644	-1.101	0.156	0.000	0.000	2053.644	-0.190	0.155	0.220	0.499	2053.644	0.911	0.180	0.000	0.000
NFIB	369.882	-0.095	0.188	0.612	0.724	369.882	0.059	0.187	0.751	0.867	369.882	0.154	0.217	0.476	0.690
NFIC	4379.669	0.396	0.217	0.067	0.145	4379.669	0.271	0.216	0.210	0.491	4379.669	-0.125	0.250	0.618	0.790
NFIL3	586.392	0.465	0.266	0.081	0.167	586.392	0.261	0.267	0.328	0.588	586.392	-0.204	0.308	0.506	0.712
NFIX	1554.444	-0.354	0.152	0.020	0.058	1554.444	-0.064	0.152	0.674	0.824	1554.444	0.290	0.176	0.099	0.278
NFKB1	2186.337	-0.891	0.273	0.001	0.006	2186.337	-0.501	0.272	0.066	0.354	2186.337	0.390	0.315	0.215	0.441
NFKB2	2394.246	0.087	0.285	0.761	0.841	2394.246	-0.473	0.285	0.097	0.392	2394.246	-0.560	0.329	0.089	0.261
NFKBIA	4848.529	0.698	0.127	0.000	0.000	4848.529	0.214	0.127	0.092	0.387	4848.529	-0.484	0.147	0.001	0.013
NFKBIB	539.228	-0.316	0.446	0.479	0.612	539.228	-0.835	0.446	0.061	0.350	539.228	-0.519	0.516	0.314	0.550
NFKBIE	719.859	0.616	0.268	0.021	0.060	719.859	-0.340	0.269	0.206	0.488	719.859	-0.956	0.310	0.002	0.022
NFKBIL1	4032.477	0.477	0.255	0.061	0.134	4032.477	0.605	0.254	0.018	0.306	4032.477	0.127	0.294	0.665	0.821
TONSL	362.652	-0.681	0.381	0.074	0.156	362.652	-0.819	0.381	0.032	0.322	362.652	-0.138	0.441	0.755	0.875
NFRKB	320.598	-0.077	0.202	0.702	0.795	320.598	0.109	0.201	0.586	0.768	320.598	0.187	0.233	0.423	0.647
NFX1	1044.093	-0.614	0.185	0.001	0.005	1044.093	-0.292	0.184	0.112	0.405	1044.093	0.322	0.213	0.132	0.330
ACO1	2306.506	-0.115	0.266	0.666	0.767	2306.506	-0.360	0.266	0.176	0.464	2306.506	-0.245	0.307	0.424	0.648
NFYA	481.478	-0.667	0.251	0.008	0.028	481.478	-0.727	0.251	0.004	0.297	481.478	-0.059	0.291	0.839	0.922
NFYB	580.347	0.001	0.229	0.997	0.999	580.347	0.032	0.229	0.891	0.945	580.347	0.031	0.265	0.908	0.958
NFYC	743.287	-0.069	0.151	0.648	0.754	743.287	-0.060	0.150	0.689	0.832	743.287	0.009	0.174	0.961	0.982
NGF	14.020	-1.976	0.765	0.010	0.034	14.020	-2.165	0.766	0.005	0.297	14.020	-0.189	0.918	0.837	0.921
NGFR	171.902	0.885	0.882	0.315	0.455	171.902	0.930	0.882	0.291	0.559	171.902	0.045	1.018	0.965	0.984
SNU13	1663.186	0.098	0.233	0.674	0.773	1663.186	-0.323	0.234	0.166	0.456	1663.186	-0.422	0.270	0.118	0.308
ATP1B1	458.664	0.323	0.126	0.010	0.035	458.664	0.007	0.126	0.958	0.978	458.664	-0.316	0.146	0.030	0.132
NHS	155.394	-0.404	0.264	0.125	0.232	155.394	0.083	0.260	0.750	0.867	155.394	0.487	0.304	0.109	0.294
NID1	9.673	0.328	0.943	0.728	0.815	9.673	0.385	0.939	0.682	0.827	9.673	0.057	1.086	0.958	0.981
NINJ1	1221.704	0.581	0.187	0.002	0.009	1221.704	0.365	0.187	0.051	0.338	1221.704	-0.216	0.216	0.316	0.552
NIT1	627.529	-0.137	0.112	0.221	0.352	627.529	0.071	0.111	0.520	0.724	627.529	0.209	0.129	0.107	0.291
NKTR	590.776	0.362	0.265	0.171	0.292	590.776	0.220	0.265	0.407	0.648	590.776	-0.142	0.305	0.641	0.805
NKX3-1	257.200	0.241	0.256	0.347	0.487	257.200	-0.426	0.260	0.101	0.394	257.200	-0.667	0.299	0.026	0.119
NKX6-1	74.465	0.515	0.233	0.027	0.073	74.465	-0.059	0.238	0.806	0.898	74.465	-0.573	0.273	0.035	0.147
NMB	24.533	0.402	0.361	0.266	0.402	24.533	-0.176	0.374	0.638	0.802	24.533	-0.578	0.428	0.176	0.394

ATP1B3	2275.513	-0.086	0.295	0.770	0.847	2275.513	-0.693	0.295	0.019	0.306	2275.513	-0.607	0.341	0.075	0.235
NME1	1267.167	0.436	0.373	0.242	0.376	1267.167	-0.464	0.374	0.214	0.493	1267.167	-0.901	0.431	0.037	0.151
NME2	7480.464	0.150	0.191	0.434	0.570	7480.464	0.283	0.191	0.139	0.430	7480.464	0.133	0.221	0.547	0.742
NME3	609.179	0.773	0.254	0.002	0.011	609.179	0.317	0.254	0.212	0.493	609.179	-0.455	0.293	0.120	0.311
NME4	1081.425	0.783	0.232	0.001	0.005	1081.425	0.109	0.233	0.639	0.803	1081.425	-0.674	0.268	0.012	0.074
NQO2	754.057	0.286	0.136	0.036	0.090	754.057	0.006	0.137	0.968	0.983	754.057	-0.281	0.158	0.075	0.236
NMT1	1769.507	0.199	0.129	0.123	0.229	1769.507	-0.244	0.130	0.060	0.350	1769.507	-0.443	0.150	0.003	0.029
NNMT	1963.688	0.637	0.202	0.002	0.008	1963.688	-0.025	0.202	0.901	0.950	1963.688	-0.662	0.233	0.004	0.038
NOP2	242.049	-1.273	0.395	0.001	0.007	242.049	-0.935	0.393	0.017	0.306	242.049	0.338	0.457	0.460	0.677
NONO	10553.553	-0.349	0.162	0.031	0.081	10553.553	0.142	0.162	0.382	0.629	10553.553	0.491	0.187	0.009	0.060
CNOT2	1068.923	-0.087	0.160	0.588	0.703	1068.923	-0.209	0.160	0.191	0.477	1068.923	-0.122	0.185	0.509	0.714
CNOT3	3972.008	-0.159	0.160	0.320	0.460	3972.008	-0.065	0.160	0.686	0.831	3972.008	0.095	0.185	0.609	0.784
CNOT4	387.491	-0.492	0.154	0.001	0.008	387.491	-0.119	0.152	0.431	0.665	387.491	0.373	0.178	0.036	0.149
NOTCH1	2097.123	-0.609	0.338	0.072	0.152	2097.123	0.245	0.338	0.468	0.691	2097.123	0.854	0.390	0.029	0.128
NOTCH2	2855.582	-0.447	0.257	0.081	0.167	2855.582	-0.028	0.256	0.913	0.957	2855.582	0.419	0.296	0.157	0.369
NOTCH3	2060.297	-0.705	0.887	0.427	0.563	2060.297	0.812	0.887	0.360	0.612	2060.297	1.517	1.024	0.138	0.341
NOTCH4	31.102	0.823	0.514	0.109	0.209	31.102	0.917	0.511	0.073	0.362	31.102	0.093	0.588	0.874	0.939
CCN3	276.599	0.627	0.438	0.153	0.268	276.599	0.628	0.438	0.151	0.444	276.599	0.002	0.505	0.998	0.998
NOVA1	83.904	1.028	0.380	0.007	0.025	83.904	0.710	0.381	0.062	0.350	83.904	-0.318	0.437	0.467	0.682
NOVA2	268.862	0.832	0.490	0.089	0.180	268.862	0.702	0.490	0.152	0.444	268.862	-0.130	0.565	0.819	0.910
PNP	750.799	-0.245	0.354	0.489	0.622	750.799	-0.768	0.355	0.030	0.322	750.799	-0.523	0.410	0.202	0.425
NPAS1	34.737	0.925	0.365	0.011	0.037	34.737	-0.078	0.384	0.840	0.917	34.737	-1.003	0.431	0.020	0.102
NPAS2	22.087	2.565	0.354	0.000	0.000	22.087	0.467	0.403	0.246	0.521	22.087	-2.098	0.415	0.000	0.000
NPAT	131.635	-0.619	0.254	0.015	0.045	131.635	-0.507	0.251	0.044	0.331	131.635	0.112	0.295	0.703	0.846
NPC1	1415.920	1.040	0.202	0.000	0.000	1415.920	-0.279	0.203	0.169	0.458	1415.920	-1.319	0.233	0.000	0.000
NPHP1	83.377	0.689	0.388	0.076	0.159	83.377	0.565	0.388	0.145	0.438	83.377	-0.124	0.447	0.781	0.890
NPM1	54351.453	0.069	0.164	0.674	0.773	54351.453	0.033	0.164	0.840	0.917	54351.453	-0.036	0.189	0.849	0.927
ATP2A1	43.389	1.449	0.360	0.000	0.001	43.389	0.287	0.373	0.442	0.673	43.389	-1.162	0.418	0.005	0.043
ATP2A2	4488.825	-0.706	0.223	0.002	0.008	4488.825	-0.468	0.223	0.036	0.324	4488.825	0.238	0.257	0.356	0.590
NPR1	109.620	3.006	0.419	0.000	0.000	109.620	0.907	0.427	0.034	0.322	109.620	-2.099	0.482	0.000	0.000
NPR3	713.308	-0.168	0.297	0.571	0.690	713.308	-0.690	0.298	0.020	0.311	713.308	-0.522	0.344	0.129	0.325
NPTX1	152.568	-1.398	0.305	0.000	0.000	152.568	-0.573	0.297	0.053	0.342	152.568	0.825	0.352	0.019	0.099
NPTX2	1184.611	-0.220	0.150	0.142	0.253	1184.611	-0.031	0.149	0.834	0.914	1184.611	0.189	0.173	0.275	0.510
SLC11A2	1085.444	-0.139	0.182	0.445	0.580	1085.444	-0.149	0.182	0.413	0.651	1085.444	-0.010	0.210	0.963	0.982
NRAS	1545.769	-0.366	0.141	0.009	0.033	1545.769	-0.264	0.141	0.060	0.350	1545.769	0.101	0.163	0.534	0.732
NRCAM	1928.598	-0.499	0.261	0.056	0.125	1928.598	-0.088	0.260	0.736	0.859	1928.598	0.411	0.301	0.172	0.389
NRDC	3794.130	-0.353	0.128	0.006	0.022	3794.130	0.034	0.128	0.791	0.891	3794.130	0.387	0.148	0.009	0.060
NRF1	313.694	-0.776	0.185	0.000	0.000	313.694	-0.240	0.181	0.184	0.471	313.694	0.536	0.213	0.012	0.074
ATP2B1	1566.378	-0.747	0.287	0.009	0.032	1566.378	-0.664	0.287	0.021	0.312	1566.378	0.083	0.332	0.803	0.902
NRGN	34.695	0.563	0.461	0.222	0.352	34.695	-0.426	0.472	0.367	0.618	34.695	-0.988	0.540	0.067	0.220
NRL	34.422	-0.964	0.326	0.003	0.014	34.422	-0.073	0.295	0.805	0.898	34.422	0.891	0.370	0.016	0.089
NRTN	107.953	0.455	0.389	0.243	0.376	107.953	0.956	0.385	0.013	0.305	107.953	0.502	0.445	0.260	0.494
YBX1	32816.118	0.256	0.171	0.135	0.245	32816.118	-0.061	0.171	0.721	0.851	32816.118	-0.317	0.198	0.109	0.294
NSF	1788.239	-0.402	0.168	0.017	0.050	1788.239	-0.252	0.167	0.132	0.424	1788.239	0.150	0.194	0.440	0.660
NT5E	917.073	0.312	0.380	0.411	0.549	917.073	-0.627	0.380	0.099	0.394	917.073	-0.939	0.439	0.032	0.138
NTF4	9.792	0.792	1.175	0.500	0.631	9.792	1.136	1.167	0.330	0.590	9.792	0.345	1.345	0.798	0.899
NTHL1	246.987	0.138	0.245	0.574	0.693	246.987	-0.389	0.246	0.114	0.406	246.987	-0.527	0.284	0.064	0.213
ROR1	315.855	-0.901	0.294	0.002	0.010	315.855	-0.747	0.293	0.011	0.300	315.855	0.154	0.340	0.651	0.813
ROR2	7.884	5.600	0.895	0.000	0.000	7.884	1.999	1.033	0.053	0.342	7.884	-3.601	0.815	0.000	0.000
DDR2	8.222	0.928	0.759	0.222	0.352	8.222	0.802	0.757	0.289	0.558	8.222	-0.125	0.862	0.884	0.945
IGIP	56.100	0.621	0.403	0.124	0.229	56.100	0.511	0.402	0.204	0.487	56.100	-0.110	0.463	0.812	0.906
NUCB1	3158.522	0.799	0.213	0.000	0.001	3158.522	0.372	0.213	0.080	0.371	3158.522	-0.428	0.246	0.082	0.247
NUCB2	700.299	0.904	0.219	0.000	0.000	700.299	0.050	0.220	0.820	0.906	700.299	-0.854	0.253	0.001	0.011
NUMA1	5617.676	-0.239	0.237	0.313	0.453	5617.676	0.268	0.237	0.258	0.530	5617.676	0.506	0.273	0.064	0.213
NUP88	646.946	-0.726	0.239	0.002	0.011	646.946	-0.452	0.238	0.058	0.348	646.946	0.274	0.276	0.322	0.556
NUP98	1644.927	-0.624	0.196	0.001	0.008	1644.927	-0.314	0.196	0.109	0.401	1644.927	0.310	0.227	0.172	0.389
NR4A2	49.383	-0.400	0.269	0.136	0.246	49.383	-0.086	0.256	0.737	0.859	49.383	0.314	0.310	0.310	0.545
ATP2B4	2355.682	0.266	0.323	0.410	0.548	2355.682	0.149	0.323	0.644	0.806	2355.682	-0.117	0.373	0.753	0.874
NVL	379.969	-0.878	0.214	0.000	0.000	379.969	-0.315	0.211	0.135	0.426	379.969	0.563	0.246	0.022	0.109
GPR143	9.910	0.429	0.676	0.526	0.652	9.910	-0.867	0.752	0.249	0.523	9.910	-1.296	0.846	0.125	0.320
COA5	407.480	0.415	0.177	0.019	0.056	407.480	0.095	0.178	0.594	0.774	407.480	-0.320	0.205	0.119	0.309
GS1-124K5.11	131.623	0.041	0.355	0.908	0.941	131.623	-0.579	0.359	0.106	0.399	131.623	-0.620	0.414	0.134	0.334
OAS1	30.393	1.560	0.328	0.000	0.000	30.393	0.893	0.337	0.008	0.297	30.393	-0.667	0.370	0.071	0.228
CISD2	758.618	0.078	0.120	0.515	0.643	758.618	-0.096	0.120	0.425	0.661	758.618	-0.175	0.140	0.211	0.436
CCDC73	545.229	0.589	0.278	0.034	0.087	545.229	0.074	0.279	0.792	0.891	545.229	-0.516	0.321	0.109	0.294
GPX8	981.048	-0.082	0.143	0.568	0.688	981.048	-0.171	0.143	0.233	0.510	981.048	-0.089	0.166	0.592	0.772
OAS2	67.571	3.113	0.375	0.000	0.000	67.571	1.051	0.389	0.007	0.297	67.571	-2.062	0.428	0.000	0.000

TMEM9B-AS1	31.508	-0.111	0.544	0.838	0.893	31.508	0.092	0.539	0.864	0.931	31.508	0.204	0.628	0.746	0.869
PHOSPHO2	13.608	-0.663	0.483	0.170	0.290	13.608	-0.220	0.454	0.629	0.797	13.608	0.444	0.557	0.426	0.649
OAS3	717.080	0.071	0.197	0.719	0.808	717.080	-0.468	0.198	0.018	0.306	717.080	-0.539	0.228	0.018	0.096
RBMXL1	410.578	-0.235	0.248	0.343	0.484	410.578	-0.433	0.248	0.081	0.373	410.578	-0.197	0.287	0.492	0.702
CHAC2	138.602	-1.034	0.525	0.049	0.113	138.602	-1.692	0.528	0.001	0.297	138.602	-0.658	0.611	0.281	0.516
OAT	1796.227	-0.315	0.131	0.016	0.050	1796.227	-0.232	0.131	0.076	0.367	1796.227	0.083	0.152	0.585	0.767
TBC1D25	462.249	-0.393	0.158	0.013	0.041	462.249	0.215	0.155	0.165	0.455	462.249	0.608	0.182	0.001	0.011
TYMSOS	174.255	-0.563	0.202	0.005	0.020	174.255	-0.354	0.199	0.074	0.364	174.255	0.209	0.234	0.372	0.604
WEE2	7.156	1.677	0.917	0.068	0.145	7.156	2.272	0.896	0.011	0.304	7.156	0.595	0.996	0.550	0.745
OAZ1	8377.243	0.382	0.181	0.035	0.088	8377.243	0.037	0.181	0.838	0.916	8377.243	-0.345	0.209	0.098	0.277
OAZ2	1413.031	0.454	0.206	0.028	0.074	1413.031	0.179	0.206	0.385	0.631	1413.031	-0.275	0.238	0.248	0.479
OCRL	605.765	-0.481	0.164	0.003	0.015	605.765	0.071	0.162	0.663	0.817	605.765	0.551	0.189	0.004	0.032
ODC1	5349.834	-1.494	0.361	0.000	0.000	5349.834	-0.959	0.360	0.008	0.297	5349.834	0.535	0.416	0.199	0.422
ODF2	1197.618	-0.138	0.144	0.338	0.477	1197.618	-0.097	0.144	0.500	0.713	1197.618	0.041	0.166	0.804	0.902
OGDH	1668.517	-0.026	0.118	0.825	0.885	1668.517	-0.015	0.117	0.897	0.948	1668.517	0.011	0.136	0.936	0.972
OGG1	258.673	-0.348	0.180	0.054	0.122	258.673	0.009	0.177	0.959	0.979	258.673	0.357	0.208	0.086	0.255
BDNF-AS	33.402	0.163	0.450	0.718	0.808	33.402	0.786	0.439	0.073	0.362	33.402	0.623	0.512	0.224	0.451
OLR1	74.064	1.531	0.238	0.000	0.000	74.064	0.063	0.252	0.803	0.897	74.064	-1.468	0.280	0.000	0.000
OPA1	2168.435	-0.987	0.181	0.000	0.000	2168.435	-0.313	0.181	0.083	0.375	2168.435	0.674	0.209	0.001	0.016
C18orf32	153.383	0.114	0.201	0.571	0.690	153.383	-0.050	0.201	0.805	0.898	153.383	-0.164	0.234	0.484	0.696
ATP5F1A	6606.696	-0.786	0.168	0.000	0.000	6606.696	-0.080	0.168	0.632	0.799	6606.696	0.706	0.194	0.000	0.005
OPHN1	155.537	-0.248	0.320	0.438	0.574	155.537	-0.003	0.318	0.992	0.996	155.537	0.245	0.369	0.507	0.713
ZBTB21	762.288	-0.705	0.256	0.006	0.022	762.288	-0.380	0.255	0.137	0.428	762.288	0.326	0.296	0.271	0.506
SCAPER	353.413	-0.211	0.373	0.572	0.691	353.413	0.482	0.372	0.195	0.478	353.413	0.693	0.430	0.107	0.292
WRAP73	443.596	0.115	0.161	0.475	0.608	443.596	-0.031	0.161	0.847	0.921	443.596	-0.146	0.186	0.433	0.655
OR1F1	31.120	-0.287	0.294	0.328	0.468	31.120	0.135	0.276	0.626	0.794	31.120	0.422	0.335	0.207	0.431
ORC1	395.343	-1.007	0.240	0.000	0.000	395.343	0.289	0.236	0.221	0.500	395.343	1.296	0.276	0.000	0.000
ORC2	562.142	-0.991	0.243	0.000	0.001	562.142	-0.516	0.242	0.033	0.322	562.142	0.475	0.281	0.091	0.264
ACO2	2216.630	-0.292	0.144	0.043	0.102	2216.630	-0.218	0.144	0.129	0.421	2216.630	0.074	0.166	0.658	0.817
ORC4	553.441	-0.529	0.189	0.005	0.020	553.441	-0.386	0.188	0.040	0.327	553.441	0.143	0.218	0.513	0.718
ORC5	389.254	-1.222	0.300	0.000	0.001	389.254	-0.628	0.298	0.035	0.322	389.254	0.594	0.346	0.087	0.257
SLC22A18	406.163	1.927	0.423	0.000	0.000	406.163	0.764	0.424	0.071	0.360	406.163	-1.163	0.488	0.017	0.092
SLC22A18AS	47.667	1.343	0.389	0.001	0.004	47.667	0.774	0.393	0.049	0.337	47.667	-0.569	0.445	0.201	0.424
ORM1	15.606	7.266	1.105	0.000	0.000	15.606	5.617	1.130	0.000	0.005	15.606	-1.648	0.883	0.062	0.209
OSBP	1854.045	-0.350	0.238	0.141	0.253	1854.045	0.145	0.237	0.541	0.737	1854.045	0.495	0.274	0.071	0.228
ALDH7A1	73.292	0.467	0.261	0.074	0.156	73.292	0.224	0.262	0.392	0.637	73.292	-0.242	0.302	0.422	0.646
OTX1	39.123	0.218	0.294	0.459	0.593	39.123	0.131	0.292	0.654	0.811	39.123	-0.087	0.339	0.799	0.899
OVGP1	16.161	1.260	0.610	0.039	0.096	16.161	-0.092	0.648	0.887	0.943	16.161	-1.352	0.723	0.061	0.208
OXA1L	1702.140	0.213	0.109	0.050	0.116	1702.140	0.072	0.109	0.508	0.717	1702.140	-0.141	0.126	0.262	0.496
OXCT1	1756.333	-0.767	0.261	0.003	0.014	1756.333	-0.481	0.261	0.065	0.353	1756.333	0.286	0.301	0.344	0.577
OXTR	19.125	0.750	0.378	0.047	0.110	19.125	0.378	0.383	0.323	0.584	19.125	-0.371	0.434	0.392	0.620
P2RX4	56.719	0.969	0.296	0.001	0.006	56.719	1.027	0.293	0.000	0.253	56.719	0.058	0.333	0.861	0.933
P2RX5	52.344	-0.007	0.454	0.987	0.993	52.344	-0.520	0.458	0.257	0.529	52.344	-0.512	0.530	0.333	0.568
P2RX7	21.422	0.417	0.885	0.637	0.745	21.422	1.178	0.875	0.178	0.467	21.422	0.761	1.013	0.453	0.672
P2RY1	62.481	-0.131	0.259	0.612	0.724	62.481	-0.193	0.257	0.452	0.680	62.481	-0.062	0.301	0.836	0.921
P2RY2	9.579	1.000	0.640	0.118	0.221	9.579	0.857	0.639	0.180	0.468	9.579	-0.143	0.724	0.844	0.924
P2RY11	473.500	0.864	0.275	0.002	0.009	473.500	0.323	0.276	0.242	0.518	473.500	-0.542	0.317	0.088	0.259
P4HA1	5065.569	-0.441	0.696	0.526	0.653	5065.569	0.706	0.696	0.310	0.574	5065.569	1.147	0.803	0.153	0.363
P4HB	19861.402	0.382	0.152	0.012	0.039	19861.402	0.146	0.152	0.337	0.595	19861.402	-0.236	0.176	0.180	0.398
A1BG-AS1	65.119	0.553	0.238	0.020	0.059	65.119	-0.197	0.248	0.427	0.663	65.119	-0.750	0.282	0.008	0.056
SPRN	50.774	0.292	0.304	0.337	0.477	50.774	-0.203	0.310	0.512	0.719	50.774	-0.495	0.356	0.165	0.380
PA2G4	6526.881	-0.472	0.316	0.135	0.245	6526.881	-0.698	0.316	0.027	0.319	6526.881	-0.227	0.365	0.534	0.733
DUXAP8	505.309	-0.161	0.135	0.233	0.365	505.309	-0.104	0.134	0.441	0.672	505.309	0.058	0.156	0.712	0.850
DUXAP9	442.726	0.361	0.205	0.078	0.162	442.726	0.128	0.205	0.534	0.733	442.726	-0.233	0.237	0.324	0.558
DUXAP10	642.981	-0.152	0.157	0.332	0.472	642.981	-0.125	0.156	0.424	0.660	642.981	0.027	0.181	0.881	0.943
LOH12CR2	10.310	0.063	0.470	0.893	0.931	10.310	-0.458	0.489	0.349	0.604	10.310	-0.521	0.567	0.358	0.591
PEBP1	3891.640	0.520	0.186	0.005	0.020	3891.640	0.001	0.186	0.995	0.997	3891.640	-0.519	0.215	0.016	0.088
PABPC3	152.144	-0.010	0.207	0.962	0.977	152.144	0.407	0.202	0.044	0.333	152.144	0.416	0.237	0.079	0.242
FURIN	1878.424	-0.016	0.193	0.933	0.958	1878.424	-0.211	0.193	0.273	0.544	1878.424	-0.195	0.223	0.381	0.611
PCSK6	417.366	-0.523	0.145	0.000	0.002	417.366	0.023	0.142	0.870	0.934	417.366	0.546	0.167	0.001	0.014
PAFAH1B1	1199.345	-0.807	0.206	0.000	0.001	1199.345	-0.197	0.205	0.337	0.595	1199.345	0.610	0.238	0.010	0.067
RRM2B	604.654	-0.348	0.234	0.137	0.248	604.654	-0.209	0.234	0.372	0.621	604.654	0.140	0.271	0.606	0.783
SMARCAL1	390.446	-0.305	0.135	0.024	0.065	390.446	0.130	0.132	0.324	0.585	390.446	0.435	0.155	0.005	0.041
MINK1	1458.817	0.065	0.214	0.762	0.842	1458.817	0.333	0.214	0.119	0.411	1458.817	0.268	0.247	0.277	0.511
PAFAH1B2	999.123	-0.521	0.171	0.002	0.011	999.123	-0.344	0.171	0.044	0.332	999.123	0.178	0.198	0.369	0.601
PAFAH1B3	442.703	0.960	0.206	0.000	0.000	442.703	0.078	0.208	0.707	0.842	442.703	-0.882	0.238	0.000	0.004
DUOX2	8.815	1.574	1.022	0.123	0.229	8.815	2.068	1.006	0.040	0.327	8.815	0.494	1.135	0.663	0.821

COL5A3		138.875	0.434	0.824	0.598	0.712	138.875	0.369	0.823	0.654	0.811	138.875	-0.065	0.951	0.945	0.975
PAFAH2		192.676	0.085	0.146	0.561	0.682	192.676	0.012	0.145	0.936	0.968	192.676	-0.073	0.169	0.665	0.822
SYCP3		189.826	0.736	0.354	0.038	0.093	189.826	0.194	0.356	0.587	0.768	189.826	-0.543	0.410	0.185	0.404
PODXL2		987.178	0.301	0.216	0.163	0.282	987.178	-0.215	0.216	0.321	0.582	987.178	-0.516	0.250	0.039	0.155
	Dec-01	109.792	-0.758	0.414	0.067	0.144	109.792	-0.548	0.411	0.183	0.470	109.792	0.210	0.479	0.660	0.819
CHST11		759.899	0.013	0.173	0.940	0.963	759.899	-0.127	0.173	0.465	0.689	759.899	-0.140	0.200	0.486	0.697
PRDX1		9955.649	0.232	0.288	0.420	0.557	9955.649	-0.654	0.288	0.023	0.316	9955.649	-0.886	0.333	0.008	0.055
SERPINE1		24118.542	0.312	0.177	0.078	0.162	24118.542	-0.137	0.177	0.438	0.671	24118.542	-0.450	0.204	0.028	0.125
PAK1		1202.694	-0.178	0.094	0.057	0.127	1202.694	-0.031	0.093	0.736	0.859	1202.694	0.147	0.108	0.174	0.390
ATP5F1B		16763.930	-0.737	0.170	0.000	0.000	16763.930	-0.196	0.170	0.250	0.524	16763.930	0.541	0.197	0.006	0.046
IL21R		25.170	1.431	0.347	0.000	0.000	25.170	-0.751	0.401	0.061	0.350	25.170	-2.182	0.437	0.000	0.000
ITSN2		509.147	-0.432	0.204	0.034	0.087	509.147	-0.450	0.203	0.027	0.319	509.147	-0.018	0.236	0.938	0.972
DEF6		350.147	0.248	0.189	0.190	0.315	350.147	0.182	0.189	0.336	0.595	350.147	-0.066	0.218	0.761	0.879
PAK2		2120.674	-0.863	0.140	0.000	0.000	2120.674	-0.237	0.139	0.087	0.380	2120.674	0.626	0.161	0.000	0.002
CYHR1		529.071	0.623	0.175	0.000	0.003	529.071	0.217	0.176	0.217	0.497	529.071	-0.406	0.202	0.044	0.170
GEMIN4		495.871	-0.681	0.238	0.004	0.017	495.871	-0.112	0.236	0.636	0.801	495.871	0.569	0.274	0.038	0.154
ANO7		74.835	0.133	0.333	0.690	0.786	74.835	0.276	0.329	0.403	0.645	74.835	0.143	0.383	0.709	0.849
PNPLA8		742.585	-0.047	0.152	0.757	0.838	742.585	-0.197	0.152	0.196	0.480	742.585	-0.149	0.176	0.397	0.625
ARHGEF4		155.524	-0.379	0.228	0.097	0.191	155.524	0.169	0.223	0.447	0.677	155.524	0.548	0.262	0.036	0.149
ARHGEF3		106.660	0.926	0.227	0.000	0.000	106.660	0.070	0.233	0.764	0.875	106.660	-0.856	0.265	0.001	0.015
SLC45A1		24.439	1.600	0.616	0.009	0.033	24.439	1.480	0.616	0.016	0.306	24.439	-0.119	0.694	0.864	0.935
PAM		1875.127	0.065	0.364	0.859	0.908	1875.127	0.601	0.364	0.098	0.393	1875.127	0.536	0.420	0.202	0.425
PRKN		21.551	1.248	1.072	0.244	0.378	21.551	1.152	1.071	0.282	0.552	21.551	-0.096	1.232	0.938	0.972
DCAF8		1077.553	-0.121	0.181	0.501	0.631	1077.553	0.234	0.180	0.193	0.478	1077.553	0.355	0.208	0.088	0.259
PARN		1299.430	-0.169	0.206	0.413	0.551	1299.430	-0.378	0.206	0.067	0.355	1299.430	-0.209	0.238	0.381	0.610
PAWR		882.801	-0.710	0.231	0.002	0.010	882.801	-0.515	0.231	0.026	0.316	882.801	0.195	0.267	0.466	0.682
PAX6		15.021	0.635	0.467	0.174	0.295	15.021	0.825	0.457	0.071	0.360	15.021	0.189	0.525	0.718	0.854
ASAP1		1152.709	-0.551	0.258	0.033	0.084	1152.709	-0.290	0.258	0.261	0.534	1152.709	0.261	0.298	0.382	0.611
AK3		634.993	-0.339	0.243	0.163	0.282	634.993	0.265	0.242	0.274	0.545	634.993	0.604	0.280	0.031	0.135
HP1BP3		2540.978	-0.348	0.236	0.140	0.251	2540.978	0.197	0.235	0.402	0.644	2540.978	0.545	0.272	0.045	0.171
HDGFL3		836.330	-0.357	0.209	0.088	0.178	836.330	-0.263	0.209	0.207	0.489	836.330	0.094	0.242	0.698	0.841
COPS7A		1164.521	0.066	0.142	0.640	0.747	1164.521	-0.117	0.142	0.410	0.650	1164.521	-0.183	0.164	0.264	0.498
NSDHL		550.162	0.946	0.150	0.000	0.000	550.162	-0.185	0.153	0.228	0.506	550.162	-1.131	0.175	0.000	0.000
PDCL		419.357	0.150	0.130	0.248	0.382	419.357	0.019	0.129	0.886	0.943	419.357	-0.131	0.150	0.383	0.611
PAX9		14.136	0.131	0.527	0.804	0.871	14.136	0.305	0.516	0.554	0.746	14.136	0.174	0.603	0.773	0.884
F11R		139.675	0.503	0.574	0.381	0.521	139.675	0.507	0.574	0.377	0.624	139.675	0.004	0.662	0.995	0.997
VILL		8.740	0.506	0.520	0.331	0.471	8.740	-0.220	0.550	0.689	0.832	8.740	-0.726	0.622	0.243	0.474
SNHG32		26545.248	0.327	0.332	0.324	0.464	26545.248	0.532	0.332	0.109	0.401	26545.248	0.205	0.383	0.593	0.773
PARD6A		14.749	0.152	0.548	0.781	0.855	14.749	0.474	0.531	0.372	0.621	14.749	0.321	0.623	0.606	0.782
SPOCK3		10.515	0.435	0.851	0.609	0.722	10.515	-0.075	0.860	0.931	0.965	10.515	-0.510	0.990	0.607	0.783
STMN3		203.418	0.334	0.162	0.039	0.095	203.418	0.087	0.162	0.594	0.774	203.418	-0.248	0.188	0.186	0.407
RNF141		447.270	-0.164	0.256	0.523	0.651	447.270	0.126	0.255	0.622	0.792	447.270	0.289	0.296	0.328	0.562
HEBP1		1116.607	0.051	0.183	0.778	0.853	1116.607	0.331	0.182	0.069	0.357	1116.607	0.280	0.211	0.184	0.404
PBX1		454.215	-0.294	0.510	0.564	0.685	454.215	0.414	0.510	0.417	0.655	454.215	0.708	0.589	0.229	0.458
PBX2		5236.963	0.145	0.207	0.483	0.616	5236.963	0.407	0.207	0.049	0.337	5236.963	0.262	0.239	0.273	0.508
ATP5F1C		3498.058	0.197	0.149	0.188	0.313	3498.058	-0.053	0.150	0.722	0.851	3498.058	-0.250	0.173	0.148	0.356
PBX3		326.032	0.146	0.301	0.628	0.738	326.032	0.443	0.300	0.140	0.431	326.032	0.297	0.347	0.393	0.621
PC		3166.856	0.176	0.275	0.524	0.651	3166.856	0.496	0.275	0.072	0.361	3166.856	0.320	0.318	0.314	0.549
PCBD1		597.512	0.563	0.200	0.005	0.019	597.512	0.294	0.200	0.142	0.433	597.512	-0.270	0.231	0.242	0.473
PCBP1		6605.988	0.614	0.155	0.000	0.001	6605.988	0.015	0.155	0.922	0.961	6605.988	-0.598	0.179	0.001	0.011
CDON		235.952	0.690	0.460	0.133	0.242	235.952	0.467	0.460	0.310	0.575	235.952	-0.223	0.531	0.674	0.827
PCBP2		13466.964	0.016	0.139	0.911	0.943	13466.964	0.225	0.139	0.106	0.398	13466.964	0.209	0.160	0.192	0.415
FOXP3		13.338	0.398	0.592	0.501	0.631	13.338	0.460	0.585	0.431	0.666	13.338	0.062	0.677	0.927	0.967
PCCA		287.257	0.165	0.204	0.419	0.556	287.257	0.334	0.203	0.099	0.394	287.257	0.169	0.235	0.472	0.686
PCCB		1361.434	-0.495	0.152	0.001	0.006	1361.434	-0.228	0.151	0.131	0.423	1361.434	0.266	0.175	0.129	0.325
PCDH1		55.290	-0.429	0.653	0.512	0.641	55.290	-1.010	0.658	0.125	0.417	55.290	-0.581	0.762	0.446	0.665
PCDHGC3		209.643	0.485	0.330	0.142	0.253	209.643	-0.251	0.332	0.450	0.678	209.643	-0.736	0.383	0.054	0.193
PCDH7		691.145	0.940	0.283	0.001	0.005	691.145	-0.027	0.284	0.924	0.961	691.145	-0.967	0.327	0.003	0.029
TMED5		862.203	-0.454	0.232	0.050	0.116	862.203	-0.144	0.231	0.532	0.732	862.203	0.310	0.267	0.247	0.478
ACOX1		408.900	0.331	0.118	0.005	0.019	408.900	-0.053	0.119	0.656	0.812	408.900	-0.384	0.137	0.005	0.041
SLC35B3		252.781	-0.479	0.184	0.009	0.032	252.781	-0.241	0.181	0.182	0.469	252.781	0.238	0.213	0.264	0.498
MTERF3		276.026	-0.462	0.334	0.166	0.286	276.026	-0.783	0.334	0.019	0.306	276.026	-0.321	0.387	0.407	0.633
TPRKB		368.971	0.177	0.257	0.490	0.623	368.971	-0.369	0.258	0.153	0.445	368.971	-0.546	0.297	0.067	0.218
MED31		85.176	0.713	0.206	0.001	0.004	85.176	-0.033	0.214	0.878	0.938	85.176	-0.746	0.242	0.002	0.022
COQ6		218.328	-0.027	0.151	0.858	0.908	218.328	-0.117	0.150	0.434	0.667	218.328	-0.091	0.175	0.605	0.782
AMDHD2		221.804	0.612	0.259	0.018	0.053	221.804	-0.270	0.263	0.305	0.571	221.804	-0.882	0.302	0.003	0.032
SLC35C2		576.235	-0.086	0.235	0.714	0.805	576.235	-0.424	0.236	0.072	0.361	576.235	-0.338	0.272	0.214	0.440

ASCC1	633.401	-0.314	0.146	0.031	0.081	633.401	0.138	0.144	0.338	0.596	633.401	0.451	0.168	0.007	0.052
DERL2	265.934	0.373	0.186	0.045	0.107	265.934	-0.109	0.188	0.562	0.751	265.934	-0.482	0.217	0.026	0.121
PCDH9	618.617	-1.144	0.272	0.000	0.000	618.617	0.035	0.270	0.896	0.948	618.617	1.180	0.314	0.000	0.004
EXOSC3	526.099	-0.160	0.332	0.631	0.740	526.099	-0.922	0.333	0.006	0.297	526.099	-0.763	0.385	0.048	0.177
FAHD2A	332.350	0.432	0.175	0.013	0.042	332.350	-0.087	0.176	0.621	0.791	332.350	-0.519	0.203	0.010	0.068
PRELID3B	1428.724	-0.513	0.173	0.003	0.013	1428.724	-0.262	0.172	0.128	0.420	1428.724	0.251	0.200	0.209	0.434
EXOSC1	331.146	0.209	0.230	0.365	0.505	331.146	-0.236	0.231	0.306	0.572	331.146	-0.445	0.267	0.095	0.272
TMED7	1217.709	0.011	0.126	0.930	0.956	1217.709	-0.230	0.126	0.068	0.357	1217.709	-0.241	0.146	0.098	0.277
ISOC1	603.443	-0.182	0.142	0.200	0.327	603.443	-0.335	0.142	0.019	0.306	603.443	-0.153	0.165	0.355	0.588
EMC9	398.550	0.160	0.241	0.506	0.636	398.550	0.227	0.240	0.346	0.602	398.550	0.066	0.278	0.811	0.906
RRP15	337.725	-0.887	0.350	0.011	0.037	337.725	-0.927	0.349	0.008	0.297	337.725	-0.041	0.405	0.920	0.963
WASHC3	345.223	0.940	0.347	0.007	0.025	345.223	0.449	0.348	0.197	0.481	345.223	-0.491	0.401	0.220	0.447
HDDC2	1100.493	0.127	0.217	0.556	0.678	1100.493	-0.024	0.217	0.913	0.957	1100.493	-0.151	0.250	0.546	0.741
MRPS16	1260.751	-0.236	0.264	0.371	0.512	1260.751	-0.386	0.264	0.144	0.435	1260.751	-0.150	0.305	0.623	0.794
GLRX2	396.498	-0.003	0.346	0.994	0.997	396.498	-0.580	0.347	0.095	0.389	396.498	-0.577	0.401	0.150	0.359
MRPS18C	552.996	-0.092	0.126	0.464	0.598	552.996	-0.115	0.125	0.358	0.611	552.996	-0.023	0.146	0.874	0.940
FIS1	643.692	0.159	0.197	0.418	0.556	643.692	-0.097	0.197	0.621	0.791	643.692	-0.257	0.228	0.260	0.494
PAM16	459.574	0.215	0.319	0.501	0.631	459.574	-0.212	0.319	0.506	0.716	459.574	-0.427	0.369	0.247	0.478
GOLT1B	511.121	-0.455	0.278	0.102	0.198	511.121	-0.574	0.278	0.039	0.327	511.121	-0.119	0.322	0.712	0.850
BOLA1	118.455	0.680	0.257	0.008	0.029	118.455	0.439	0.257	0.088	0.380	118.455	-0.241	0.295	0.414	0.639
VPS36	265.688	0.138	0.167	0.410	0.549	265.688	-0.252	0.169	0.135	0.426	265.688	-0.390	0.195	0.046	0.172
DESI2	1641.861	-0.928	0.181	0.000	0.000	1641.861	-0.339	0.180	0.060	0.350	1641.861	0.589	0.209	0.005	0.040
TVP23B	556.493	0.102	0.146	0.482	0.615	556.493	0.068	0.145	0.638	0.803	556.493	-0.034	0.168	0.839	0.923
GLOD4	558.239	-0.129	0.163	0.429	0.566	558.239	-0.373	0.163	0.022	0.314	558.239	-0.245	0.189	0.195	0.417
UBXN1	2094.625	0.517	0.183	0.005	0.019	2094.625	0.184	0.183	0.317	0.579	2094.625	-0.334	0.212	0.115	0.304
ZNF593	386.974	0.009	0.352	0.979	0.987	386.974	-0.474	0.353	0.179	0.467	386.974	-0.483	0.408	0.236	0.465
ZBTB7B	1447.042	-0.116	0.157	0.460	0.594	1447.042	0.305	0.157	0.051	0.339	1447.042	0.422	0.182	0.020	0.102
GMNN	608.228	-0.607	0.350	0.083	0.169	608.228	-0.829	0.350	0.018	0.306	608.228	-0.222	0.405	0.583	0.766
PLEKHA8P1	94.120	0.162	0.372	0.663	0.765	94.120	0.322	0.370	0.384	0.630	94.120	0.160	0.428	0.710	0.849
LAP3	1122.900	-0.800	0.287	0.005	0.021	1122.900	-0.246	0.287	0.391	0.636	1122.900	0.554	0.332	0.095	0.271
WDPCP	75.070	-0.060	0.251	0.811	0.876	75.070	-0.051	0.249	0.837	0.916	75.070	0.009	0.291	0.976	0.987
ZNF691	201.934	0.031	0.210	0.885	0.925	201.934	0.172	0.208	0.409	0.649	201.934	0.142	0.242	0.559	0.750
PCK2	1524.293	-1.587	0.238	0.000	0.000	1524.293	0.168	0.236	0.475	0.696	1524.293	1.755	0.274	0.000	0.000
TXNDC12	1593.121	0.028	0.154	0.856	0.906	1593.121	-0.096	0.154	0.532	0.732	1593.121	-0.124	0.178	0.486	0.697
TXNDC11	713.679	-0.179	0.178	0.315	0.455	713.679	-0.077	0.177	0.663	0.817	713.679	0.101	0.206	0.622	0.793
ATL1	18.688	-0.428	0.426	0.316	0.456	18.688	-0.674	0.429	0.116	0.409	18.688	-0.246	0.509	0.628	0.797
CALHM2	46.407	0.879	0.412	0.033	0.084	46.407	-0.086	0.424	0.839	0.916	46.407	-0.965	0.482	0.045	0.171
RPS27L	674.962	1.279	0.242	0.000	0.000	674.962	0.306	0.244	0.209	0.491	674.962	-0.972	0.280	0.001	0.008
YARS2	554.327	-0.037	0.198	0.851	0.903	554.327	-0.294	0.199	0.138	0.429	554.327	-0.257	0.230	0.263	0.497
NMD3	2766.208	-0.710	0.169	0.000	0.000	2766.208	-0.018	0.169	0.917	0.959	2766.208	0.692	0.195	0.000	0.007
MRPL2	859.870	0.211	0.239	0.376	0.516	859.870	-0.116	0.239	0.629	0.797	859.870	-0.327	0.276	0.236	0.465
NOSIP	1848.594	0.572	0.174	0.001	0.006	1848.594	0.055	0.174	0.752	0.868	1848.594	-0.517	0.201	0.010	0.067
DERA	534.213	0.206	0.148	0.165	0.284	534.213	-0.011	0.148	0.939	0.969	534.213	-0.217	0.171	0.205	0.429
MEMO1	847.464	0.038	0.150	0.801	0.869	847.464	-0.162	0.150	0.281	0.550	847.464	-0.200	0.174	0.250	0.482
MRPL4	1597.807	-0.388	0.300	0.195	0.321	1597.807	-0.456	0.300	0.128	0.420	1597.807	-0.068	0.346	0.843	0.924
APIP	623.935	-0.070	0.136	0.606	0.718	623.935	-0.231	0.136	0.090	0.384	623.935	-0.160	0.158	0.309	0.545
TMX2	1166.156	-0.674	0.225	0.003	0.012	1166.156	-0.347	0.224	0.122	0.413	1166.156	0.327	0.260	0.208	0.432
CUTC	479.561	-0.585	0.204	0.004	0.017	479.561	-0.420	0.202	0.038	0.327	479.561	0.165	0.236	0.484	0.696
FCF1	780.084	-0.716	0.217	0.001	0.006	780.084	-0.394	0.216	0.068	0.356	780.084	0.322	0.250	0.198	0.421
THAP4	1078.711	-0.134	0.191	0.483	0.617	1078.711	-0.147	0.191	0.441	0.672	1078.711	-0.013	0.221	0.952	0.978
NDUFA13	319.159	1.520	0.363	0.000	0.000	319.159	0.441	0.364	0.227	0.505	319.159	-1.079	0.419	0.010	0.066
PCM1	1794.496	-0.792	0.186	0.000	0.000	1794.496	0.028	0.185	0.880	0.939	1794.496	0.820	0.214	0.000	0.003
MRPS7	924.311	-0.527	0.250	0.035	0.089	924.311	-0.417	0.250	0.095	0.389	924.311	0.110	0.289	0.704	0.846
POLR1D	2752.425	0.068	0.253	0.786	0.858	2752.425	0.371	0.252	0.142	0.434	2752.425	0.302	0.292	0.300	0.535
GAL	1688.816	-0.703	0.318	0.027	0.073	1688.816	0.406	0.317	0.201	0.484	1688.816	1.108	0.367	0.003	0.026
CRYL1	193.678	0.708	0.367	0.054	0.122	193.678	0.482	0.368	0.190	0.475	193.678	-0.226	0.424	0.593	0.773
MLXIPL	128.403	1.000	0.601	0.096	0.190	128.403	0.909	0.601	0.130	0.422	128.403	-0.091	0.693	0.895	0.951
KLHL5	1324.330	-0.496	0.219	0.024	0.065	1324.330	-0.275	0.219	0.209	0.490	1324.330	0.221	0.253	0.382	0.611
PLLP	1002.162	-0.162	0.170	0.341	0.482	1002.162	0.142	0.169	0.403	0.645	1002.162	0.303	0.196	0.122	0.313
SEPSECS	209.474	-0.901	0.227	0.000	0.001	209.474	-0.203	0.222	0.362	0.614	209.474	0.699	0.262	0.008	0.055
SIDT2	350.037	0.256	0.358	0.473	0.607	350.037	0.511	0.357	0.152	0.445	350.037	0.254	0.412	0.537	0.735
RRNAD1	660.889	0.464	0.185	0.012	0.039	660.889	0.459	0.185	0.013	0.305	660.889	-0.005	0.213	0.981	0.990
ADIPOR1	1509.814	0.205	0.087	0.019	0.056	1509.814	-0.125	0.088	0.155	0.448	1509.814	-0.329	0.101	0.001	0.015
TRNT1	410.354	-0.304	0.230	0.186	0.311	410.354	-0.529	0.230	0.022	0.314	410.354	-0.225	0.266	0.398	0.626
UTP18	1224.465	-0.518	0.236	0.028	0.075	1224.465	-0.378	0.236	0.109	0.401	1224.465	0.140	0.273	0.607	0.783
SCCPDH	771.118	0.414	0.185	0.026	0.070	771.118	0.198	0.185	0.286	0.555	771.118	-0.216	0.214	0.314	0.549
IFT52	231.689	-0.064	0.192	0.737	0.823	231.689	0.243	0.189	0.199	0.482	231.689	0.308	0.221	0.163	0.377

ABHD5	454.626	-0.569	0.342	0.096	0.190	454.626	-0.815	0.342	0.017	0.306	454.626	-0.246	0.396	0.535	0.733
PCMT1	1889.108	-0.263	0.183	0.152	0.266	1889.108	-0.327	0.183	0.075	0.364	1889.108	-0.064	0.212	0.763	0.880
SH3GLB1	1796.423	-0.113	0.172	0.511	0.640	1796.423	0.063	0.172	0.713	0.846	1796.423	0.177	0.199	0.375	0.606
ZC2HC1A	146.221	-0.389	0.223	0.081	0.167	146.221	-0.291	0.221	0.188	0.474	146.221	0.098	0.259	0.704	0.846
MECR	445.926	-0.264	0.200	0.187	0.311	445.926	-0.269	0.200	0.179	0.467	445.926	-0.004	0.232	0.985	0.992
NDUFAF1	259.300	-0.043	0.273	0.875	0.919	259.300	-0.494	0.274	0.071	0.361	259.300	-0.451	0.317	0.155	0.365
ABHD17B	210.502	-0.487	0.181	0.007	0.026	210.502	0.163	0.174	0.349	0.604	210.502	0.650	0.207	0.002	0.019
PHF20L1	654.399	-0.669	0.244	0.006	0.023	654.399	-0.666	0.244	0.006	0.297	654.399	0.004	0.282	0.990	0.994
TFB1M	426.462	-0.268	0.162	0.099	0.194	426.462	-0.238	0.161	0.141	0.432	426.462	0.030	0.188	0.873	0.939
APH1A	1889.184	0.091	0.114	0.427	0.563	1889.184	0.111	0.114	0.329	0.590	1889.184	0.020	0.132	0.877	0.941
METTL9	1645.954	-0.161	0.158	0.306	0.445	1645.954	0.107	0.157	0.496	0.711	1645.954	0.268	0.182	0.140	0.344
RDH11	1625.683	0.722	0.187	0.000	0.001	1625.683	-0.439	0.188	0.020	0.306	1625.683	-1.161	0.217	0.000	0.000
PCNA	1107.755	0.012	0.409	0.976	0.986	1107.755	-1.117	0.410	0.006	0.297	1107.755	-1.130	0.473	0.017	0.092
LACTB2	249.134	0.518	0.159	0.001	0.006	249.134	-0.251	0.162	0.122	0.414	249.134	-0.769	0.186	0.000	0.001
KMT5B	612.678	-0.776	0.145	0.000	0.000	612.678	-0.141	0.142	0.322	0.583	612.678	0.635	0.167	0.000	0.003
TRAPPC12	576.852	0.358	0.232	0.123	0.228	576.852	-0.264	0.233	0.256	0.529	576.852	-0.622	0.268	0.020	0.103
ZDHHC9	1682.770	0.248	0.180	0.169	0.289	1682.770	0.270	0.180	0.133	0.425	1682.770	0.022	0.208	0.915	0.960
RMDN1	634.820	-0.364	0.160	0.023	0.063	634.820	-0.006	0.159	0.968	0.983	634.820	0.358	0.184	0.052	0.188
MRPS2	1291.221	-0.183	0.134	0.171	0.292	1291.221	0.039	0.133	0.769	0.877	1291.221	0.223	0.155	0.150	0.359
COQ4	470.034	0.056	0.197	0.776	0.852	470.034	0.149	0.196	0.448	0.677	470.034	0.093	0.227	0.682	0.832
UTP11	666.316	-0.236	0.314	0.453	0.587	666.316	-0.800	0.315	0.011	0.302	666.316	-0.564	0.364	0.121	0.313
SBDS	1399.106	-0.226	0.263	0.391	0.530	1399.106	-0.586	0.263	0.026	0.318	1399.106	-0.360	0.304	0.237	0.466
RPL26L1	451.161	0.294	0.299	0.327	0.467	451.161	-0.386	0.300	0.199	0.482	451.161	-0.679	0.346	0.050	0.183
COMMD2	500.567	-0.482	0.191	0.011	0.038	500.567	-0.555	0.190	0.004	0.297	500.567	-0.072	0.221	0.743	0.868
ZNF706	825.522	0.357	0.277	0.198	0.324	825.522	-0.493	0.278	0.076	0.366	825.522	-0.850	0.321	0.008	0.057
IER3IP1	1001.894	0.222	0.161	0.168	0.288	1001.894	-0.287	0.162	0.076	0.364	1001.894	-0.509	0.187	0.006	0.048
GOLGA7	1160.837	-0.010	0.081	0.902	0.937	1160.837	-0.093	0.080	0.250	0.524	1160.837	-0.083	0.094	0.377	0.609
NAA20	1239.661	0.396	0.255	0.121	0.225	1239.661	-0.230	0.255	0.368	0.618	1239.661	-0.626	0.295	0.034	0.142
SAR1B	823.804	-0.127	0.264	0.631	0.740	823.804	-0.689	0.264	0.009	0.297	823.804	-0.562	0.305	0.066	0.216
ANGPTL4	7951.407	2.974	0.524	0.000	0.000	7951.407	1.151	0.524	0.028	0.321	7951.407	-1.823	0.605	0.003	0.026
ASB3	52.115	-0.423	0.288	0.142	0.254	52.115	0.039	0.276	0.887	0.944	52.115	0.462	0.330	0.162	0.375
PHF11	219.175	0.964	0.218	0.000	0.000	219.175	0.190	0.221	0.390	0.635	219.175	-0.774	0.252	0.002	0.023
RLIM	1221.229	-0.699	0.191	0.000	0.002	1221.229	-0.351	0.190	0.065	0.354	1221.229	0.348	0.221	0.115	0.303
KCTD3	1487.758	-0.607	0.145	0.000	0.000	1487.758	-0.060	0.145	0.679	0.827	1487.758	0.547	0.168	0.001	0.014
CEP83	280.411	-0.775	0.216	0.000	0.002	280.411	-0.266	0.213	0.211	0.491	280.411	0.509	0.250	0.041	0.163
IRAK4	240.158	0.494	0.131	0.000	0.001	240.158	0.390	0.131	0.003	0.297	240.158	-0.104	0.150	0.491	0.701
RNFT1	39.538	0.355	0.309	0.250	0.385	39.538	-0.341	0.320	0.288	0.556	39.538	-0.696	0.367	0.058	0.201
COPS4	599.811	-0.598	0.172	0.001	0.003	599.811	-0.009	0.170	0.960	0.979	599.811	0.590	0.199	0.003	0.029
INSIG2	963.000	0.844	0.387	0.029	0.077	963.000	0.473	0.387	0.221	0.501	963.000	-0.371	0.446	0.406	0.633
CHCHD2	4375.015	0.104	0.190	0.585	0.701	4375.015	-0.103	0.190	0.586	0.768	4375.015	-0.207	0.219	0.345	0.579
DYNC1LI1	1170.872	-0.518	0.118	0.000	0.000	1170.872	-0.281	0.117	0.016	0.306	1170.872	0.236	0.136	0.082	0.248
HSD17B12	2219.900	-0.086	0.089	0.329	0.469	2219.900	-0.123	0.088	0.163	0.453	2219.900	-0.037	0.102	0.718	0.854
ING4	352.580	0.205	0.375	0.584	0.701	352.580	0.614	0.374	0.100	0.394	352.580	0.409	0.432	0.344	0.578
CERCAM	969.592	1.137	0.259	0.000	0.000	969.592	0.576	0.259	0.026	0.318	969.592	-0.562	0.298	0.060	0.205
MRNIP	17928.977	1.801	0.209	0.000	0.000	17928.977	-0.157	0.209	0.453	0.680	17928.977	-1.958	0.242	0.000	0.000
SDF4	4009.503	0.391	0.156	0.012	0.040	4009.503	-0.112	0.156	0.475	0.696	4009.503	-0.502	0.180	0.005	0.043
MRTO4	1393.538	-0.488	0.373	0.191	0.315	1393.538	-0.735	0.373	0.049	0.337	1393.538	-0.247	0.431	0.566	0.756
JPT1	3290.802	0.244	0.216	0.258	0.393	3290.802	-0.143	0.216	0.509	0.718	3290.802	-0.387	0.249	0.121	0.313
ZNF580	544.030	0.183	0.198	0.356	0.496	544.030	0.384	0.197	0.051	0.339	544.030	0.201	0.228	0.378	0.609
PCNT	824.752	-0.306	0.125	0.015	0.045	824.752	-0.039	0.124	0.752	0.868	824.752	0.267	0.144	0.065	0.215
VPS28	909.912	0.492	0.180	0.006	0.024	909.912	0.333	0.180	0.064	0.352	909.912	-0.158	0.208	0.447	0.666
C3orf18	161.933	1.021	0.393	0.009	0.032	161.933	0.671	0.393	0.088	0.380	161.933	-0.349	0.452	0.440	0.660
EGFL7	18.176	1.003	0.593	0.090	0.182	18.176	0.379	0.608	0.532	0.732	18.176	-0.624	0.684	0.362	0.594
DBR1	474.970	-0.303	0.175	0.084	0.172	474.970	-0.316	0.175	0.071	0.359	474.970	-0.013	0.203	0.947	0.975
DCTN4	2231.472	-0.252	0.234	0.282	0.420	2231.472	0.204	0.234	0.383	0.629	2231.472	0.457	0.271	0.092	0.265
AADAT	27.318	-0.498	0.431	0.248	0.382	27.318	-0.639	0.430	0.138	0.429	27.318	-0.141	0.506	0.781	0.890
CYB5R4	384.038	-0.443	0.174	0.011	0.037	384.038	-0.234	0.173	0.175	0.464	384.038	0.209	0.202	0.300	0.535
HSD17B11	523.969	-0.037	0.372	0.920	0.949	523.969	0.405	0.371	0.275	0.545	523.969	0.443	0.429	0.302	0.537
NAGPA	177.738	0.597	0.181	0.001	0.006	177.738	-0.029	0.184	0.876	0.937	177.738	-0.625	0.211	0.003	0.029
TUBD1	117.224	-0.099	0.194	0.608	0.720	117.224	-0.092	0.192	0.632	0.799	117.224	0.008	0.225	0.973	0.987
TUBE1	318.192	-1.716	0.359	0.000	0.000	318.192	-0.714	0.354	0.044	0.332	318.192	1.002	0.414	0.015	0.087
LEF1	131.330	-0.418	0.253	0.098	0.193	131.330	-0.292	0.250	0.243	0.519	131.330	0.126	0.293	0.667	0.823
PLEKHO1	600.652	1.011	0.238	0.000	0.000	600.652	-0.198	0.241	0.410	0.649	600.652	-1.210	0.276	0.000	0.000
PCOLCE	77.400	2.036	0.402	0.000	0.000	77.400	0.123	0.413	0.766	0.876	77.400	-1.913	0.468	0.000	0.001
DCXR	1264.887	0.329	0.203	0.105	0.202	1264.887	0.132	0.203	0.514	0.720	1264.887	-0.197	0.234	0.401	0.628
HSPA14	665.755	-0.824	0.327	0.012	0.039	665.755	-0.871	0.327	0.008	0.297	665.755	-0.047	0.379	0.901	0.953
GPN3	702.867	-0.693	0.212	0.001	0.006	702.867	-0.350	0.211	0.097	0.392	702.867	0.343	0.245	0.162	0.375

CRBN	611.738	0.045	0.182	0.804	0.870	611.738	0.191	0.181	0.290	0.558	611.738	0.146	0.210	0.485	0.697
TCEAL9	402.703	1.267	0.265	0.000	0.000	402.703	0.278	0.267	0.299	0.566	402.703	-0.990	0.307	0.001	0.016
RSL24D1	5097.113	-0.458	0.151	0.002	0.011	5097.113	-0.068	0.151	0.654	0.811	5097.113	0.391	0.174	0.025	0.117
SS18L2	280.050	0.121	0.161	0.450	0.586	280.050	-0.231	0.162	0.152	0.445	280.050	-0.353	0.187	0.059	0.204
CHMP1A	1047.853	0.091	0.170	0.593	0.708	1047.853	-0.305	0.170	0.073	0.362	1047.853	-0.395	0.196	0.044	0.169
HERC5	39.968	2.883	0.412	0.000	0.000	39.968	0.823	0.443	0.063	0.350	39.968	-2.060	0.470	0.000	0.000
CKLF	207.636	-0.091	0.208	0.663	0.765	207.636	0.036	0.207	0.861	0.930	207.636	0.127	0.240	0.598	0.776
ZNF639	855.454	-0.716	0.169	0.000	0.000	855.454	-0.255	0.168	0.129	0.421	855.454	0.461	0.196	0.018	0.096
IPO11	550.720	-1.805	0.464	0.000	0.001	550.720	-0.791	0.462	0.087	0.380	550.720	1.014	0.535	0.058	0.201
RAPGEFL1	37.192	0.319	0.389	0.412	0.550	37.192	-0.096	0.394	0.807	0.899	37.192	-0.415	0.454	0.361	0.593
PLCE1	131.132	0.072	0.236	0.761	0.841	131.132	-0.631	0.240	0.009	0.297	131.132	-0.703	0.277	0.011	0.070
NIN	472.063	-0.693	0.278	0.013	0.041	472.063	-0.505	0.278	0.069	0.357	472.063	0.188	0.322	0.558	0.750
CPA4	64.015	0.043	0.389	0.912	0.944	64.015	0.293	0.384	0.446	0.677	64.015	0.250	0.447	0.577	0.762
ZDHHC2	386.910	0.336	0.167	0.044	0.105	386.910	-0.074	0.168	0.658	0.813	386.910	-0.410	0.193	0.034	0.142
DDX47	762.468	-0.211	0.153	0.168	0.288	762.468	-0.107	0.152	0.482	0.701	762.468	0.104	0.177	0.557	0.749
NUSAP1	2080.462	-0.180	0.222	0.418	0.555	2080.462	-0.025	0.222	0.912	0.957	2080.462	0.156	0.257	0.544	0.740
TACO1	422.131	-0.185	0.274	0.500	0.630	422.131	-0.463	0.274	0.091	0.386	422.131	-0.278	0.317	0.381	0.610
ACP6	115.942	0.228	0.226	0.313	0.452	115.942	0.434	0.222	0.051	0.338	115.942	0.206	0.258	0.425	0.648
GLRX5	1291.045	-0.057	0.263	0.827	0.886	1291.045	-0.442	0.264	0.093	0.388	1291.045	-0.385	0.304	0.206	0.430
PCSK1	205.975	-0.347	0.468	0.459	0.594	205.975	0.653	0.465	0.160	0.451	205.975	0.999	0.539	0.064	0.212
ZNF219	490.018	0.713	0.207	0.001	0.004	490.018	0.234	0.207	0.260	0.532	490.018	-0.479	0.239	0.045	0.170
COPZ2	112.678	1.515	0.327	0.000	0.000	112.678	0.892	0.329	0.007	0.297	112.678	-0.623	0.375	0.096	0.274
PIGP	343.526	0.059	0.296	0.841	0.896	343.526	0.443	0.294	0.132	0.424	343.526	0.384	0.341	0.259	0.494
GLTP	1036.257	0.366	0.145	0.012	0.038	1036.257	0.043	0.145	0.766	0.877	1036.257	-0.323	0.168	0.054	0.192
PHF20	918.076	-0.051	0.178	0.775	0.850	918.076	0.150	0.178	0.398	0.642	918.076	0.201	0.206	0.328	0.562
VRK3	337.419	0.031	0.135	0.820	0.882	337.419	0.139	0.133	0.298	0.565	337.419	0.108	0.155	0.487	0.698
CRIM1	2933.726	-0.459	0.231	0.047	0.110	2933.726	-0.102	0.231	0.658	0.814	2933.726	0.357	0.267	0.180	0.399
EMC4	1503.877	0.097	0.176	0.581	0.699	1503.877	-0.241	0.176	0.172	0.461	1503.877	-0.338	0.203	0.097	0.275
HGH1	357.733	-0.743	0.385	0.053	0.121	357.733	-0.725	0.384	0.059	0.349	357.733	0.019	0.445	0.966	0.984
ANKRD39	13.988	0.163	0.576	0.777	0.852	13.988	0.264	0.567	0.642	0.805	13.988	0.101	0.661	0.879	0.942
COX16	346.625	0.190	0.181	0.294	0.432	346.625	-0.201	0.182	0.271	0.542	346.625	-0.391	0.210	0.063	0.212
CCDC174	461.559	-0.341	0.204	0.095	0.189	461.559	-0.282	0.203	0.165	0.455	461.559	0.058	0.236	0.805	0.902
SHISA5	3150.161	0.233	0.134	0.080	0.166	3150.161	-0.034	0.134	0.801	0.896	3150.161	-0.267	0.154	0.083	0.251
PAIP2	2766.976	-0.300	0.130	0.021	0.061	2766.976	0.053	0.130	0.682	0.827	2766.976	0.353	0.150	0.019	0.098
PDZD11	635.955	0.475	0.162	0.003	0.014	635.955	0.218	0.162	0.180	0.468	635.955	-0.258	0.187	0.168	0.383
TMEM69	604.818	0.221	0.170	0.193	0.319	604.818	0.020	0.170	0.907	0.954	604.818	-0.201	0.196	0.306	0.541
MTRES1	194.960	0.635	0.223	0.004	0.018	194.960	-0.065	0.226	0.773	0.879	194.960	-0.700	0.260	0.007	0.052
NT5C3A	565.649	-0.284	0.135	0.035	0.088	565.649	-0.019	0.133	0.887	0.944	565.649	0.265	0.156	0.088	0.259
MRPL37	2299.205	-0.433	0.139	0.002	0.010	2299.205	-0.028	0.139	0.839	0.916	2299.205	0.405	0.161	0.012	0.073
RNF181	1609.287	0.987	0.290	0.001	0.004	1609.287	0.550	0.290	0.058	0.348	1609.287	-0.437	0.335	0.191	0.413
TBC1D7	166.850	-0.251	0.372	0.500	0.631	166.850	-0.871	0.375	0.020	0.310	166.850	-0.621	0.434	0.152	0.361
MARCHF2	173.620	1.838	0.201	0.000	0.000	173.620	0.204	0.211	0.334	0.593	173.620	-1.634	0.235	0.000	0.000
MRPL51	1737.685	0.280	0.159	0.079	0.163	1737.685	0.112	0.159	0.484	0.701	1737.685	-0.168	0.184	0.360	0.593
TMEM216	79.836	-0.408	0.265	0.125	0.230	79.836	-0.113	0.259	0.661	0.815	79.836	0.294	0.306	0.337	0.571
PBDC1	294.089	-0.114	0.302	0.706	0.798	294.089	-0.745	0.304	0.014	0.306	294.089	-0.631	0.351	0.072	0.229
MRPL30	929.167	-0.301	0.134	0.025	0.068	929.167	-0.274	0.134	0.040	0.328	929.167	0.027	0.155	0.864	0.935
MRPL27	575.417	0.370	0.267	0.165	0.284	575.417	-0.266	0.268	0.319	0.581	575.417	-0.637	0.309	0.039	0.156
CDKL3	43.013	0.133	0.338	0.694	0.789	43.013	0.383	0.330	0.247	0.521	43.013	0.250	0.386	0.518	0.721
CDK16	2417.682	-0.127	0.091	0.164	0.284	2417.682	0.205	0.091	0.024	0.316	2417.682	0.331	0.105	0.002	0.019
UBAP1	1948.049	-0.421	0.138	0.002	0.011	1948.049	-0.142	0.137	0.299	0.566	1948.049	0.279	0.159	0.080	0.244
BET1L	840.442	0.043	0.096	0.652	0.756	840.442	-0.009	0.096	0.927	0.963	840.442	-0.052	0.111	0.639	0.804
KLF3	988.528	-0.623	0.152	0.000	0.000	988.528	0.057	0.151	0.705	0.842	988.528	0.680	0.176	0.000	0.003
MAPKAPK5-AS1	419.500	-0.429	0.139	0.002	0.010	419.500	0.067	0.136	0.625	0.794	419.500	0.495	0.160	0.002	0.022
ZNF571	24.076	0.749	0.371	0.043	0.104	24.076	-0.501	0.404	0.214	0.493	24.076	-1.251	0.452	0.006	0.044
DNAJC27	38.509	-0.250	0.327	0.443	0.579	38.509	0.537	0.308	0.082	0.373	38.509	0.787	0.369	0.033	0.139
IER5	973.371	0.752	0.255	0.003	0.014	973.371	-0.101	0.256	0.693	0.834	973.371	-0.853	0.295	0.004	0.034
C1RL	986.018	1.083	0.367	0.003	0.014	986.018	0.794	0.367	0.031	0.322	986.018	-0.290	0.424	0.494	0.703
CDK17	686.039	-0.998	0.191	0.000	0.000	686.039	-0.405	0.189	0.032	0.322	686.039	0.593	0.220	0.007	0.052
GOLM1	2704.719	-0.435	0.139	0.002	0.009	2704.719	-0.214	0.138	0.122	0.413	2704.719	0.221	0.160	0.167	0.381
ANKMY1	216.819	-0.420	0.186	0.024	0.066	216.819	-0.028	0.182	0.880	0.940	216.819	0.392	0.215	0.067	0.220
SCAND1	868.588	1.011	0.236	0.000	0.000	868.588	0.150	0.237	0.526	0.728	868.588	-0.860	0.273	0.002	0.019
BFAR	3090.115	-0.048	0.113	0.669	0.770	3090.115	0.031	0.113	0.785	0.887	3090.115	0.079	0.131	0.545	0.740
COA4	809.115	-0.137	0.249	0.581	0.699	809.115	-0.257	0.249	0.301	0.567	809.115	-0.120	0.288	0.676	0.828
CDK18	1224.057	1.090	1.049	0.299	0.437	1224.057	0.784	1.049	0.455	0.682	1224.057	-0.306	1.211	0.800	0.900
ERGIC2	895.034	-0.096	0.200	0.631	0.740	895.034	-0.351	0.200	0.079	0.371	895.034	-0.255	0.231	0.270	0.505
GMIP	254.215	0.029	0.154	0.851	0.902	254.215	0.316	0.152	0.037	0.326	254.215	0.287	0.177	0.105	0.289
GMPR2	1342.001	-0.138	0.174	0.426	0.563	1342.001	0.274	0.173	0.113	0.405	1342.001	0.412	0.200	0.039	0.158

CD320	1914.399	-0.222	0.255	0.384	0.523	1914.399	-0.283	0.255	0.266	0.538	1914.399	-0.061	0.294	0.835	0.920
PCDH12	8.571	1.454	0.816	0.075	0.157	8.571	1.172	0.819	0.152	0.445	8.571	-0.282	0.919	0.759	0.878
ECSIT	1166.687	0.113	0.186	0.544	0.668	1166.687	0.309	0.185	0.095	0.389	1166.687	0.197	0.214	0.359	0.591
SLC15A3	89.698	2.303	0.362	0.000	0.000	89.698	0.578	0.376	0.124	0.416	89.698	-1.725	0.418	0.000	0.001
NRN1	2994.959	0.628	1.160	0.589	0.704	2994.959	1.024	1.160	0.377	0.625	2994.959	0.397	1.340	0.767	0.881
ATP5F1D	2358.620	0.690	0.251	0.006	0.023	2358.620	0.183	0.251	0.466	0.690	2358.620	-0.507	0.290	0.080	0.245
PCYT1A	1100.553	-0.180	0.152	0.238	0.371	1100.553	-0.246	0.152	0.105	0.398	1100.553	-0.066	0.176	0.706	0.847
TIMMDC1	1802.234	-0.416	0.219	0.057	0.128	1802.234	-0.327	0.218	0.135	0.426	1802.234	0.089	0.252	0.725	0.858
CYP39A1	45.934	-0.841	0.580	0.147	0.260	45.934	0.247	0.564	0.661	0.815	45.934	1.089	0.665	0.101	0.283
FKBP11	533.609	-0.158	0.234	0.500	0.631	533.609	-0.317	0.234	0.176	0.465	533.609	-0.159	0.271	0.558	0.749
ZDHHC3	958.394	-0.049	0.169	0.772	0.848	958.394	-0.361	0.169	0.033	0.322	958.394	-0.312	0.195	0.110	0.296
FAM13B	323.390	-0.383	0.251	0.127	0.233	323.390	-0.204	0.249	0.412	0.651	323.390	0.178	0.290	0.538	0.736
FAM53C	1124.376	-0.422	0.115	0.000	0.002	1124.376	-0.153	0.114	0.180	0.469	1124.376	0.269	0.133	0.043	0.165
REEP2	8.200	0.953	0.660	0.149	0.263	8.200	0.123	0.685	0.857	0.927	8.200	-0.830	0.771	0.282	0.516
SLC25A37	2794.698	0.589	0.208	0.005	0.019	2794.698	0.302	0.208	0.148	0.440	2794.698	-0.287	0.240	0.232	0.461
GASK1B	357.711	-0.044	0.221	0.841	0.895	357.711	0.025	0.220	0.910	0.955	357.711	0.069	0.255	0.786	0.893
KRCC1	176.684	0.695	0.449	0.121	0.226	176.684	0.455	0.449	0.311	0.575	176.684	-0.241	0.518	0.642	0.806
PLAC8	143.826	0.196	0.338	0.561	0.682	143.826	0.444	0.336	0.186	0.473	143.826	0.248	0.389	0.524	0.725
PHF21A	1479.385	0.024	0.315	0.940	0.963	1479.385	0.468	0.315	0.137	0.428	1479.385	0.445	0.364	0.222	0.449
MRPL35	1187.884	-0.608	0.294	0.038	0.094	1187.884	-0.701	0.294	0.017	0.306	1187.884	-0.093	0.339	0.784	0.892
RSRC1	954.643	-0.173	0.129	0.181	0.305	954.643	-0.181	0.129	0.160	0.451	954.643	-0.008	0.150	0.955	0.979
MEX3C	1120.255	-0.910	0.127	0.000	0.000	1120.255	-0.077	0.125	0.535	0.734	1120.255	0.833	0.146	0.000	0.000
AMZ2	1354.519	-0.110	0.248	0.656	0.759	1354.519	0.501	0.247	0.043	0.331	1354.519	0.611	0.286	0.033	0.139
WAC	3073.635	-0.815	0.147	0.000	0.000	3073.635	-0.178	0.147	0.224	0.503	3073.635	0.636	0.170	0.000	0.004
SPG21	1291.259	0.156	0.107	0.146	0.259	1291.259	0.008	0.107	0.941	0.970	1291.259	-0.148	0.124	0.232	0.461
ARL17A	231.009	0.398	0.217	0.066	0.143	231.009	0.158	0.217	0.467	0.691	231.009	-0.241	0.250	0.337	0.571
ARL6IP4	58.583	1.237	0.336	0.000	0.002	58.583	0.301	0.345	0.382	0.628	58.583	-0.935	0.389	0.016	0.090
TNFRSF12A	2605.196	-0.399	0.730	0.585	0.701	2605.196	-1.759	0.730	0.016	0.306	2605.196	-1.360	0.843	0.107	0.291
ZNF771	399.909	-0.505	0.310	0.103	0.200	399.909	-0.611	0.310	0.049	0.337	399.909	-0.106	0.359	0.768	0.882
PRR16	67.027	0.116	0.262	0.659	0.762	67.027	0.426	0.255	0.095	0.389	67.027	0.310	0.299	0.299	0.534
NGRN	647.119	-0.469	0.231	0.043	0.102	647.119	-0.495	0.231	0.032	0.322	647.119	-0.026	0.268	0.924	0.965
THEM6	604.719	0.085	0.206	0.679	0.777	604.719	-0.084	0.206	0.683	0.828	604.719	-0.170	0.239	0.477	0.690
PDCD2	1271.563	-0.050	0.183	0.786	0.858	1271.563	-0.181	0.183	0.322	0.583	1271.563	-0.131	0.211	0.535	0.733
CRNKL1	505.449	-0.526	0.145	0.000	0.002	505.449	-0.446	0.144	0.002	0.297	505.449	0.079	0.168	0.637	0.803
ZBTB7A	1819.160	0.390	0.145	0.007	0.026	1819.160	0.072	0.145	0.619	0.790	1819.160	-0.318	0.167	0.057	0.199
FZR1	1329.636	0.169	0.180	0.348	0.488	1329.636	0.325	0.180	0.071	0.359	1329.636	0.156	0.208	0.453	0.672
TAOK3	443.439	0.082	0.185	0.658	0.761	443.439	-0.214	0.185	0.248	0.522	443.439	-0.296	0.214	0.167	0.382
MBTPS2	497.098	-0.910	0.287	0.002	0.008	497.098	-0.077	0.285	0.786	0.888	497.098	0.833	0.330	0.012	0.073
HOOK1	23.936	0.365	0.354	0.302	0.441	23.936	0.601	0.342	0.078	0.370	23.936	0.236	0.398	0.553	0.746
CDC40	835.054	-0.748	0.214	0.000	0.003	835.054	0.028	0.212	0.897	0.948	835.054	0.776	0.246	0.002	0.019
CHST15	310.711	-0.054	0.154	0.727	0.815	310.711	0.189	0.151	0.210	0.491	310.711	0.243	0.177	0.169	0.385
ZMYND10	11.758	0.668	0.691	0.333	0.473	11.758	0.472	0.691	0.495	0.710	11.758	-0.196	0.791	0.804	0.902
UBR5	2574.502	-0.823	0.245	0.001	0.005	2574.502	-0.009	0.245	0.970	0.984	2574.502	0.813	0.283	0.004	0.036
POP5	197.519	0.076	0.391	0.846	0.899	197.519	-0.732	0.393	0.063	0.350	197.519	-0.808	0.454	0.075	0.235
TEX264	1049.608	0.647	0.209	0.002	0.010	1049.608	0.365	0.209	0.080	0.371	1049.608	-0.282	0.241	0.242	0.473
POMP	1941.267	0.348	0.241	0.148	0.262	1941.267	-0.480	0.241	0.046	0.335	1941.267	-0.828	0.278	0.003	0.028
TMA7	1880.937	0.678	0.261	0.009	0.032	1880.937	-0.026	0.261	0.921	0.960	1880.937	-0.704	0.301	0.019	0.100
MRPS17	94.401	0.538	0.207	0.009	0.033	94.401	-0.117	0.213	0.582	0.766	94.401	-0.655	0.243	0.007	0.052
ATRAID	1030.870	0.265	0.166	0.111	0.212	1030.870	0.230	0.166	0.167	0.456	1030.870	-0.035	0.192	0.854	0.930
SNX7	1531.436	-0.228	0.237	0.337	0.477	1531.436	0.171	0.237	0.471	0.693	1531.436	0.398	0.274	0.146	0.353
UCHL5	1566.840	-0.739	0.300	0.014	0.043	1566.840	-0.810	0.300	0.007	0.297	1566.840	-0.071	0.346	0.838	0.921
ANGPT4	5.451	-5.472	1.378	0.000	0.001	5.451	-0.163	0.772	0.833	0.914	5.451	5.309	1.452	0.000	0.005
CRLF3	297.075	-0.551	0.194	0.004	0.018	297.075	-0.448	0.192	0.020	0.306	297.075	0.103	0.224	0.646	0.809
PDE2A	313.762	0.335	0.829	0.686	0.783	313.762	1.057	0.827	0.202	0.485	313.762	0.721	0.956	0.450	0.669
CSAD	71.033	0.402	0.311	0.196	0.322	71.033	0.210	0.311	0.499	0.712	71.033	-0.192	0.359	0.593	0.773
ATP6V1D	967.756	0.043	0.146	0.768	0.847	967.756	-0.378	0.146	0.010	0.298	967.756	-0.421	0.169	0.013	0.077
ZNF589	146.557	-0.568	0.198	0.004	0.017	146.557	-0.080	0.192	0.675	0.825	146.557	0.488	0.228	0.032	0.138
EIF3L	6752.922	-0.412	0.209	0.048	0.112	6752.922	0.276	0.209	0.185	0.472	6752.922	0.689	0.241	0.004	0.037
NIP7	1063.457	-0.608	0.306	0.047	0.111	1063.457	-0.737	0.306	0.016	0.306	1063.457	-0.130	0.354	0.715	0.852
RWDD1	998.416	0.552	0.223	0.013	0.042	998.416	-0.038	0.223	0.863	0.930	998.416	-0.591	0.257	0.022	0.107
PDE3A	6.704	-1.818	0.704	0.010	0.034	6.704	-0.811	0.603	0.178	0.467	6.704	1.007	0.807	0.212	0.437
AIG1	1215.545	0.658	0.190	0.001	0.004	1215.545	0.286	0.190	0.132	0.424	1215.545	-0.372	0.219	0.090	0.263
COMMD10	280.189	0.079	0.170	0.640	0.747	280.189	-0.161	0.170	0.343	0.600	280.189	-0.241	0.197	0.222	0.449
TRAPPC4	1061.762	-0.127	0.227	0.576	0.694	1061.762	-0.380	0.227	0.095	0.389	1061.762	-0.252	0.263	0.336	0.571
ATP5F1E	1209.408	0.611	0.188	0.001	0.006	1209.408	0.085	0.188	0.652	0.810	1209.408	-0.526	0.217	0.015	0.087
PDE3B	58.517	-0.548	0.334	0.101	0.197	58.517	-0.026	0.325	0.937	0.968	58.517	0.522	0.384	0.174	0.390
PPME1	1543.826	0.279	0.194	0.151	0.265	1543.826	0.149	0.194	0.442	0.673	1543.826	-0.130	0.224	0.563	0.754

NOL7	2078.487	-0.427	0.212	0.044	0.105	2078.487	-0.301	0.212	0.156	0.448	2078.487	0.126	0.245	0.606	0.783
HEMK1	235.445	0.220	0.174	0.205	0.333	235.445	0.235	0.173	0.174	0.463	235.445	0.014	0.200	0.943	0.974
PDE4A	1175.679	-0.007	0.201	0.971	0.982	1175.679	0.369	0.200	0.066	0.354	1175.679	0.376	0.232	0.104	0.288
PDE4B	66.027	-0.446	0.418	0.285	0.423	66.027	0.525	0.408	0.198	0.481	66.027	0.972	0.478	0.042	0.164
AMOTL2	3423.855	0.100	0.191	0.601	0.715	3423.855	0.135	0.191	0.480	0.699	3423.855	0.035	0.220	0.874	0.939
PRKAG2	1799.822	-0.457	0.165	0.006	0.022	1799.822	0.170	0.164	0.300	0.567	1799.822	0.628	0.191	0.001	0.013
POLK	625.325	-0.731	0.182	0.000	0.001	625.325	-0.301	0.180	0.095	0.390	625.325	0.430	0.210	0.041	0.160
ZNF107	52.706	0.046	0.293	0.876	0.920	52.706	-0.062	0.292	0.833	0.914	52.706	-0.107	0.340	0.752	0.873
DDX41	1843.032	0.536	0.193	0.006	0.021	1843.032	0.306	0.193	0.113	0.405	1843.032	-0.230	0.223	0.303	0.538
SNX9	1503.090	0.158	0.086	0.066	0.143	1503.090	-0.091	0.086	0.292	0.559	1503.090	-0.248	0.100	0.013	0.076
SUCO	500.261	-0.339	0.229	0.139	0.249	500.261	-0.198	0.228	0.385	0.631	500.261	0.141	0.265	0.594	0.774
ANAPC5	1790.287	-0.379	0.225	0.092	0.185	1790.287	-0.423	0.225	0.060	0.350	1790.287	-0.044	0.260	0.867	0.936
ANAPC7	629.565	-0.089	0.279	0.751	0.834	629.565	-0.604	0.280	0.031	0.322	629.565	-0.516	0.323	0.111	0.297
SCARA3	519.314	0.572	0.233	0.014	0.044	519.314	-0.104	0.234	0.656	0.813	519.314	-0.676	0.270	0.012	0.075
FAM8A1	664.429	-0.269	0.308	0.383	0.523	664.429	0.288	0.308	0.349	0.604	664.429	0.557	0.356	0.117	0.307
PDE4D	145.730	-1.581	0.419	0.000	0.001	145.730	-0.891	0.413	0.031	0.322	145.730	0.690	0.484	0.154	0.364
YTHDF2	1512.221	-0.148	0.130	0.254	0.388	1512.221	-0.336	0.130	0.010	0.298	1512.221	-0.188	0.150	0.211	0.436
RNF138	690.725	-0.965	0.178	0.000	0.000	690.725	-0.268	0.176	0.127	0.420	690.725	0.697	0.205	0.001	0.010
IP6K2	1270.538	0.117	0.274	0.670	0.770	1270.538	0.472	0.273	0.084	0.376	1270.538	0.355	0.316	0.260	0.495
PCYOX1	982.449	-0.023	0.461	0.960	0.976	982.449	0.428	0.460	0.353	0.607	982.449	0.451	0.532	0.397	0.625
PDE6A	8.471	-1.092	1.114	0.327	0.467	8.471	0.465	1.060	0.661	0.815	8.471	1.557	1.268	0.219	0.446
LCMT1	413.807	0.231	0.149	0.123	0.228	413.807	0.100	0.149	0.502	0.714	413.807	-0.130	0.173	0.450	0.669
GULP1	12.135	0.730	0.457	0.110	0.209	12.135	0.300	0.468	0.521	0.725	12.135	-0.431	0.527	0.414	0.639
REV1	435.025	-0.497	0.208	0.017	0.051	435.025	-0.110	0.206	0.594	0.774	435.025	0.387	0.240	0.107	0.292
SFMBT1	86.224	-0.614	0.340	0.071	0.151	86.224	-0.450	0.336	0.181	0.469	86.224	0.164	0.394	0.678	0.829
GPR89B	194.330	-0.063	0.209	0.763	0.842	194.330	-0.099	0.208	0.634	0.800	194.330	-0.036	0.242	0.882	0.943
UBE2J1	1476.699	-0.260	0.116	0.025	0.068	1476.699	-0.251	0.116	0.030	0.322	1476.699	0.009	0.134	0.947	0.975
EVL	632.044	1.559	0.183	0.000	0.000	632.044	0.139	0.185	0.452	0.680	632.044	-1.419	0.212	0.000	0.000
PDE6D	209.434	0.572	0.151	0.000	0.001	209.434	-0.134	0.155	0.388	0.634	209.434	-0.705	0.177	0.000	0.002
LIMA1	3663.457	-0.518	0.235	0.028	0.074	3663.457	-0.439	0.235	0.062	0.350	3663.457	0.079	0.272	0.771	0.883
ISYNA1	121.700	2.068	0.335	0.000	0.000	121.700	0.634	0.341	0.063	0.350	121.700	-1.434	0.386	0.000	0.004
HSD17B7	126.314	1.902	0.190	0.000	0.000	126.314	-0.346	0.212	0.102	0.395	126.314	-2.247	0.231	0.000	0.000
ANKFY1	431.694	-0.284	0.230	0.217	0.347	431.694	-0.304	0.230	0.185	0.472	431.694	-0.020	0.266	0.940	0.973
SPOUT1	631.841	0.000	0.242	0.999	1.000	631.841	-0.363	0.243	0.135	0.426	631.841	-0.363	0.280	0.196	0.418
NOP16	1631.000	0.117	0.230	0.609	0.721	1631.000	-0.140	0.230	0.543	0.738	1631.000	-0.257	0.265	0.332	0.567
RTCB	1202.555	-0.740	0.282	0.009	0.030	1202.555	-0.537	0.281	0.056	0.344	1202.555	0.203	0.325	0.533	0.732
HACD3	343.129	-0.773	0.297	0.009	0.032	343.129	-0.738	0.297	0.013	0.305	343.129	0.034	0.344	0.921	0.963
CTDSPL2	748.807	-0.709	0.216	0.001	0.006	748.807	-0.129	0.215	0.549	0.743	748.807	0.580	0.250	0.020	0.102
NELFCD	1180.223	-0.323	0.137	0.018	0.054	1180.223	-0.125	0.136	0.360	0.612	1180.223	0.198	0.158	0.211	0.436
TRIAP1	502.796	-0.162	0.187	0.387	0.527	502.796	-0.262	0.187	0.161	0.452	502.796	-0.100	0.217	0.643	0.806
ATP5PB	3394.778	-0.225	0.140	0.108	0.207	3394.778	-0.188	0.140	0.178	0.467	3394.778	0.037	0.162	0.820	0.911
PDE7A	457.126	-0.156	0.132	0.238	0.371	457.126	-0.047	0.131	0.719	0.849	457.126	0.109	0.153	0.476	0.689
HIKESHI	271.979	-0.111	0.173	0.522	0.649	271.979	-0.160	0.173	0.355	0.609	271.979	-0.049	0.201	0.808	0.904
CWC15	666.051	0.722	0.219	0.001	0.006	666.051	0.085	0.220	0.699	0.838	666.051	-0.637	0.254	0.012	0.074
TRMT112	2790.432	0.939	0.260	0.000	0.002	2790.432	-0.091	0.260	0.727	0.854	2790.432	-1.029	0.300	0.001	0.009
UFC1	942.798	0.461	0.241	0.055	0.124	942.798	0.344	0.240	0.153	0.445	942.798	-0.117	0.278	0.673	0.827
RTF2	1479.767	0.110	0.117	0.349	0.489	1479.767	-0.012	0.117	0.916	0.958	1479.767	-0.122	0.136	0.367	0.600
PDE8A	545.082	-0.344	0.184	0.062	0.135	545.082	-0.279	0.183	0.129	0.420	545.082	0.065	0.213	0.759	0.877
CHMP5	950.077	0.136	0.114	0.234	0.366	950.077	0.083	0.114	0.467	0.691	950.077	-0.053	0.132	0.686	0.835
GTSE1	665.495	-0.386	0.151	0.010	0.035	665.495	-0.211	0.150	0.158	0.450	665.495	0.175	0.174	0.315	0.551
DTL	661.798	-1.542	0.457	0.001	0.005	661.798	-1.301	0.457	0.004	0.297	661.798	0.241	0.529	0.649	0.811
NCKIPSD	829.098	0.136	0.260	0.601	0.714	829.098	0.468	0.260	0.072	0.361	829.098	0.332	0.300	0.270	0.505
LARS1	4665.853	-0.946	0.213	0.000	0.000	4665.853	-0.287	0.213	0.178	0.466	4665.853	0.659	0.246	0.007	0.053
TMEM14C	2548.520	0.474	0.183	0.010	0.033	2548.520	0.233	0.183	0.203	0.486	2548.520	-0.241	0.211	0.254	0.487
CXXC5	529.570	0.910	0.318	0.004	0.017	529.570	0.529	0.318	0.097	0.391	529.570	-0.381	0.367	0.299	0.534
TMEM138	405.953	-0.441	0.279	0.114	0.215	405.953	-0.573	0.279	0.040	0.327	405.953	-0.132	0.323	0.682	0.832
OSER1	917.802	0.058	0.191	0.761	0.842	917.802	0.133	0.190	0.484	0.701	917.802	0.075	0.220	0.732	0.861
GSKIP	288.469	-0.505	0.253	0.046	0.108	288.469	-0.657	0.253	0.009	0.297	288.469	-0.152	0.293	0.605	0.782
JKAMP	480.008	-0.383	0.150	0.011	0.036	480.008	-0.211	0.149	0.156	0.448	480.008	0.172	0.174	0.322	0.557
ANAPC11	1233.401	0.579	0.225	0.010	0.035	1233.401	0.013	0.226	0.953	0.977	1233.401	-0.565	0.261	0.030	0.132
ZC3HC1	255.705	-0.478	0.248	0.054	0.123	255.705	-0.435	0.247	0.078	0.370	255.705	0.043	0.288	0.882	0.943
TRMO	104.392	-0.181	0.289	0.531	0.656	104.392	-0.227	0.287	0.429	0.664	104.392	-0.046	0.335	0.891	0.948
PHF7	86.132	-0.244	0.365	0.504	0.634	86.132	0.247	0.360	0.494	0.709	86.132	0.491	0.420	0.243	0.474
VTA1	1159.186	-0.438	0.138	0.002	0.008	1159.186	-0.376	0.138	0.006	0.297	1159.186	0.062	0.160	0.698	0.841
PPHLN1	1030.127	-0.356	0.170	0.037	0.091	1030.127	-0.441	0.170	0.010	0.298	1030.127	-0.085	0.197	0.666	0.822
MTFP1	751.552	-0.175	0.241	0.467	0.601	751.552	-0.155	0.241	0.519	0.724	751.552	0.020	0.279	0.943	0.974
ZCCHC17	634.919	0.463	0.219	0.035	0.088	634.919	-0.195	0.220	0.376	0.624	634.919	-0.658	0.254	0.010	0.063

PDGFA	1179.705	0.882	0.224	0.000	0.001	1179.705	0.027	0.225	0.903	0.951	1179.705	-0.855	0.259	0.001	0.013
SCLY	161.020	-1.243	0.438	0.005	0.018	161.020	-1.045	0.437	0.017	0.306	161.020	0.198	0.508	0.696	0.841
VPS54	291.293	-0.212	0.133	0.111	0.211	291.293	-0.269	0.132	0.042	0.330	291.293	-0.057	0.155	0.714	0.851
ZNF581	902.893	0.386	0.409	0.345	0.485	902.893	0.751	0.408	0.066	0.354	902.893	0.365	0.471	0.439	0.660
SIRT7	641.028	0.409	0.164	0.012	0.040	641.028	0.015	0.164	0.929	0.964	641.028	-0.394	0.189	0.037	0.152
SIRT6	742.449	0.678	0.220	0.002	0.010	742.449	-0.177	0.221	0.424	0.660	742.449	-0.854	0.254	0.001	0.011
PDGFB	9.247	-0.244	0.580	0.674	0.773	9.247	0.366	0.546	0.503	0.714	9.247	0.610	0.657	0.353	0.586
CINP	530.036	0.185	0.225	0.411	0.549	530.036	-0.122	0.225	0.587	0.769	530.036	-0.307	0.260	0.238	0.467
RAB14	1872.942	-0.481	0.153	0.002	0.009	1872.942	0.067	0.152	0.659	0.814	1872.942	0.548	0.177	0.002	0.021
ACKR4	14.782	0.483	0.629	0.442	0.578	14.782	-0.738	0.655	0.260	0.532	14.782	-1.221	0.748	0.102	0.285
NT5DC3	111.916	-0.277	0.293	0.344	0.484	111.916	-0.194	0.291	0.505	0.716	111.916	0.084	0.339	0.805	0.903
PDGFRA	889.121	-1.566	0.296	0.000	0.000	889.121	0.120	0.292	0.682	0.828	889.121	1.686	0.341	0.000	0.000
RAB6B	108.599	1.403	0.303	0.000	0.000	108.599	0.210	0.310	0.498	0.712	108.599	-1.193	0.352	0.001	0.010
MBIP	200.530	0.059	0.167	0.725	0.813	200.530	0.047	0.166	0.775	0.881	200.530	-0.011	0.193	0.953	0.978
HDAC7	2091.306	0.365	0.103	0.000	0.003	2091.306	0.129	0.103	0.210	0.491	2091.306	-0.235	0.119	0.048	0.178
ARMCX3	1047.730	0.188	0.317	0.553	0.675	1047.730	0.362	0.317	0.253	0.527	1047.730	0.174	0.366	0.635	0.802
TDP2	765.725	-0.112	0.200	0.577	0.695	765.725	-0.317	0.200	0.113	0.405	765.725	-0.205	0.231	0.375	0.606
UFM1	986.364	-0.047	0.164	0.776	0.851	986.364	-0.176	0.164	0.284	0.553	986.364	-0.129	0.189	0.496	0.704
PDGFRL	29.892	2.747	0.376	0.000	0.000	29.892	1.056	0.412	0.010	0.299	29.892	-1.691	0.417	0.000	0.001
FAM49B	906.091	-0.566	0.281	0.044	0.105	906.091	-0.583	0.281	0.038	0.327	906.091	-0.017	0.325	0.958	0.981
GDE1	936.424	-0.041	0.103	0.692	0.787	936.424	-0.079	0.102	0.441	0.673	936.424	-0.038	0.119	0.749	0.871
LARP7	599.362	0.837	0.259	0.001	0.007	599.362	-0.061	0.260	0.815	0.903	599.362	-0.898	0.299	0.003	0.027
ESF1	615.311	0.396	0.268	0.139	0.250	615.311	-0.301	0.268	0.263	0.535	615.311	-0.697	0.310	0.024	0.115
AZIN1	3458.532	-0.627	0.131	0.000	0.000	3458.532	-0.151	0.130	0.248	0.522	3458.532	0.476	0.151	0.002	0.019
PCF11	564.885	-0.133	0.220	0.546	0.670	564.885	0.027	0.220	0.901	0.950	564.885	0.160	0.254	0.528	0.729
MED15	2675.049	0.145	0.180	0.421	0.558	2675.049	0.313	0.180	0.083	0.374	2675.049	0.167	0.208	0.421	0.646
PIAS4	492.302	0.383	0.129	0.003	0.013	492.302	-0.049	0.130	0.706	0.842	492.302	-0.432	0.150	0.004	0.034
PDGFRB	57.877	0.413	0.700	0.556	0.677	57.877	0.494	0.699	0.480	0.699	57.877	0.081	0.807	0.920	0.963
TRIM33	1003.599	0.127	0.161	0.429	0.566	1003.599	-0.034	0.161	0.833	0.914	1003.599	-0.161	0.186	0.386	0.615
SRRT	1735.650	-0.138	0.342	0.687	0.784	1735.650	-0.860	0.343	0.012	0.305	1735.650	-0.723	0.396	0.068	0.221
NBAS	1117.016	-0.564	0.309	0.068	0.146	1117.016	0.406	0.308	0.187	0.474	1117.016	0.970	0.356	0.006	0.049
CUTA	5036.666	0.971	0.219	0.000	0.000	5036.666	0.136	0.219	0.534	0.733	5036.666	-0.835	0.253	0.001	0.013
LSR	91.714	0.391	0.264	0.139	0.250	91.714	0.456	0.262	0.082	0.373	91.714	0.065	0.303	0.829	0.916
ATP5MC1	1472.427	0.458	0.324	0.157	0.274	1472.427	-0.573	0.325	0.077	0.369	1472.427	-1.031	0.375	0.006	0.046
PDHA1	2152.893	0.122	0.174	0.484	0.617	2152.893	-0.262	0.174	0.132	0.424	2152.893	-0.384	0.201	0.056	0.197
LIPT1	57.136	-0.120	0.279	0.667	0.768	57.136	-0.107	0.276	0.698	0.837	57.136	0.013	0.323	0.968	0.985
NOP58	1947.530	-0.515	0.259	0.047	0.110	1947.530	-0.594	0.259	0.022	0.314	1947.530	-0.078	0.299	0.794	0.896
EEF1AKNMT	855.351	-0.616	0.227	0.007	0.025	855.351	-0.273	0.226	0.227	0.506	855.351	0.343	0.262	0.190	0.411
PIGT	916.312	0.074	0.116	0.524	0.651	916.312	0.153	0.115	0.183	0.470	916.312	0.079	0.133	0.551	0.745
TRMT6	650.742	-0.916	0.391	0.019	0.055	650.742	-1.098	0.391	0.005	0.297	650.742	-0.182	0.452	0.688	0.836
ATP6V1H	702.416	0.265	0.096	0.006	0.023	702.416	-0.095	0.097	0.328	0.588	702.416	-0.360	0.112	0.001	0.016
GET4	947.531	0.195	0.246	0.427	0.563	947.531	-0.315	0.246	0.200	0.483	947.531	-0.510	0.284	0.073	0.230
DPH5	770.236	-0.158	0.105	0.133	0.241	770.236	-0.003	0.104	0.978	0.988	770.236	0.155	0.121	0.201	0.424
ERGIC3	2653.530	0.159	0.257	0.534	0.660	2653.530	0.452	0.257	0.078	0.369	2653.530	0.293	0.296	0.323	0.558
TAF9B	339.398	-0.391	0.138	0.005	0.018	339.398	-0.194	0.136	0.153	0.445	339.398	0.197	0.159	0.216	0.442
UBE2D4	146.241	0.226	0.249	0.364	0.505	146.241	-0.269	0.251	0.284	0.553	146.241	-0.495	0.290	0.088	0.259
PDHB	1025.571	0.308	0.141	0.029	0.077	1025.571	-0.198	0.142	0.163	0.454	1025.571	-0.506	0.164	0.002	0.022
KLF13	6160.601	-0.971	0.350	0.006	0.021	6160.601	0.288	0.350	0.411	0.650	6160.601	1.259	0.404	0.002	0.020
CCZ1	1444.938	-0.138	0.268	0.607	0.719	1444.938	-0.507	0.268	0.058	0.348	1444.938	-0.370	0.309	0.232	0.461
DYNC2LI1	109.443	-0.012	0.246	0.962	0.977	109.443	0.279	0.242	0.248	0.522	109.443	0.291	0.282	0.303	0.538
SLC25A39	2405.950	0.560	0.251	0.026	0.070	2405.950	-0.372	0.252	0.139	0.430	2405.950	-0.933	0.291	0.001	0.016
PDK1	782.505	-0.332	0.270	0.218	0.348	782.505	0.165	0.269	0.538	0.735	782.505	0.498	0.311	0.110	0.295
LUC7L2	743.106	-0.904	0.179	0.000	0.000	743.106	-0.209	0.177	0.239	0.515	743.106	0.695	0.207	0.001	0.011
OTUD6B	301.448	-1.519	0.423	0.000	0.002	301.448	-1.182	0.421	0.005	0.297	301.448	0.337	0.489	0.491	0.701
RBMX2	338.372	0.770	0.247	0.002	0.009	338.372	0.151	0.248	0.543	0.738	338.372	-0.619	0.286	0.030	0.133
DHRS7	284.861	0.951	0.156	0.000	0.000	284.861	0.266	0.159	0.094	0.389	284.861	-0.685	0.181	0.000	0.003
RTRAF	1574.832	0.340	0.172	0.048	0.112	1574.832	-0.162	0.172	0.348	0.603	1574.832	-0.502	0.199	0.012	0.072
SF3B6	1129.640	0.154	0.157	0.325	0.465	1129.640	-0.098	0.157	0.531	0.732	1129.640	-0.253	0.181	0.164	0.378
PDK2	236.358	0.030	0.387	0.938	0.961	236.358	0.440	0.386	0.254	0.527	236.358	0.410	0.446	0.359	0.591
MRPL48	368.484	0.437	0.200	0.029	0.077	368.484	-0.106	0.201	0.600	0.778	368.484	-0.543	0.232	0.019	0.100
TMBIM4	546.715	0.397	0.166	0.017	0.051	546.715	0.017	0.167	0.918	0.959	546.715	-0.380	0.193	0.048	0.179
PPIL1	631.423	-0.509	0.295	0.084	0.172	631.423	-0.634	0.295	0.032	0.322	631.423	-0.125	0.341	0.715	0.852
YPEL5	977.285	0.564	0.376	0.134	0.244	977.285	0.585	0.376	0.120	0.412	977.285	0.021	0.434	0.961	0.982
CIAO2B	1204.003	0.601	0.212	0.005	0.018	1204.003	-0.001	0.213	0.998	0.999	1204.003	-0.602	0.245	0.014	0.082
MRPS23	904.675	-0.269	0.288	0.351	0.491	904.675	-0.408	0.288	0.157	0.449	904.675	-0.140	0.333	0.675	0.828
PDK3	307.419	0.398	0.454	0.381	0.521	307.419	0.556	0.454	0.220	0.500	307.419	0.159	0.524	0.762	0.879
MRPS33	732.135	0.383	0.172	0.026	0.071	732.135	0.029	0.173	0.864	0.931	732.135	-0.354	0.199	0.076	0.238

PTRH2	886.893	-0.248	0.173	0.152	0.267	886.893	-0.350	0.173	0.043	0.331	886.893	-0.102	0.201	0.611	0.785
CHMP3	1545.783	0.053	0.144	0.715	0.805	1545.783	-0.057	0.144	0.692	0.834	1545.783	-0.109	0.166	0.510	0.715
CDK5RAP1	271.192	-0.160	0.325	0.623	0.734	271.192	-0.820	0.326	0.012	0.305	271.192	-0.661	0.377	0.080	0.244
STYXL1	299.300	0.177	0.139	0.202	0.329	299.300	0.116	0.138	0.399	0.643	299.300	-0.061	0.160	0.704	0.846
GINS2	226.199	-0.425	0.484	0.379	0.519	226.199	-0.945	0.485	0.051	0.338	226.199	-0.520	0.560	0.353	0.586
PDK4	277.184	-1.135	0.599	0.058	0.129	277.184	0.606	0.596	0.310	0.574	277.184	1.741	0.691	0.012	0.073
MPC1	408.246	0.079	0.272	0.772	0.848	408.246	-0.579	0.273	0.034	0.322	408.246	-0.658	0.315	0.037	0.151
FKBP7	278.188	0.229	0.261	0.380	0.520	278.188	0.248	0.260	0.341	0.599	278.188	0.019	0.301	0.949	0.976
ZFR	2659.301	-1.150	0.183	0.000	0.000	2659.301	-0.189	0.183	0.300	0.567	2659.301	0.961	0.212	0.000	0.000
ASB1	680.630	-0.886	0.355	0.013	0.041	680.630	-0.776	0.355	0.029	0.322	680.630	0.110	0.410	0.789	0.895
NUB1	984.662	-0.278	0.158	0.078	0.162	984.662	-0.254	0.157	0.107	0.399	984.662	0.025	0.182	0.892	0.949
HSPB11	454.805	0.603	0.147	0.000	0.000	454.805	0.065	0.149	0.662	0.816	454.805	-0.538	0.171	0.002	0.019
SARAF	3619.115	-0.121	0.212	0.567	0.687	3619.115	0.354	0.211	0.093	0.388	3619.115	0.476	0.244	0.051	0.186
ENPP1	204.790	0.118	0.221	0.592	0.707	204.790	-0.006	0.221	0.980	0.989	204.790	-0.124	0.256	0.628	0.797
ASB2	11.327	3.513	0.691	0.000	0.000	11.327	1.733	0.763	0.023	0.316	11.327	-1.779	0.687	0.010	0.064
MPP6	309.042	-1.102	0.303	0.000	0.002	309.042	-0.752	0.301	0.013	0.305	309.042	0.351	0.351	0.318	0.553
ENPP2	418.082	-1.289	0.217	0.000	0.000	418.082	0.150	0.211	0.477	0.698	418.082	1.439	0.249	0.000	0.000
SUFU	387.575	-0.002	0.295	0.995	0.998	387.575	0.355	0.294	0.227	0.506	387.575	0.357	0.340	0.294	0.530
OAZ3	252.024	-0.786	0.218	0.000	0.002	252.024	-0.162	0.214	0.448	0.677	252.024	0.624	0.252	0.013	0.079
LSM7	1299.781	0.840	0.233	0.000	0.002	1299.781	-0.090	0.234	0.700	0.839	1299.781	-0.930	0.269	0.001	0.009
LSM8	301.967	0.039	0.156	0.800	0.868	301.967	-0.165	0.156	0.289	0.558	301.967	-0.204	0.181	0.258	0.492
CPSF3	745.437	-0.123	0.230	0.592	0.707	745.437	-0.375	0.230	0.104	0.397	745.437	-0.251	0.266	0.346	0.580
TRAPPC2L	546.220	0.385	0.182	0.035	0.088	546.220	0.198	0.182	0.278	0.548	546.220	-0.187	0.210	0.374	0.606
HECA	473.592	-0.151	0.189	0.426	0.562	473.592	0.138	0.188	0.463	0.688	473.592	0.289	0.218	0.186	0.406
VPS29	1052.382	0.404	0.196	0.040	0.097	1052.382	-0.269	0.197	0.172	0.461	1052.382	-0.673	0.227	0.003	0.029
ATP5MC2	3550.893	0.665	0.284	0.019	0.056	3550.893	0.412	0.284	0.146	0.438	3550.893	-0.253	0.328	0.440	0.660
PDPK1	1138.337	-0.623	0.189	0.001	0.006	1138.337	-0.176	0.188	0.349	0.604	1138.337	0.447	0.219	0.041	0.161
CYB5R2	8.284	1.715	0.588	0.004	0.015	8.284	0.108	0.664	0.871	0.935	8.284	-1.607	0.705	0.023	0.109
NLK	197.205	-0.393	0.245	0.108	0.207	197.205	-0.599	0.245	0.015	0.306	197.205	-0.206	0.285	0.471	0.685
PADI3	11.685	0.257	1.247	0.837	0.892	11.685	0.137	1.247	0.913	0.957	11.685	-0.120	1.440	0.933	0.970
ACSL5	335.849	0.654	0.279	0.019	0.056	335.849	0.167	0.280	0.550	0.743	335.849	-0.486	0.322	0.131	0.329
GPRC5B	96.932	0.629	0.159	0.000	0.001	96.932	0.044	0.164	0.787	0.889	96.932	-0.585	0.186	0.002	0.019
CYB5R1	557.588	0.052	0.191	0.786	0.858	557.588	0.363	0.190	0.056	0.344	557.588	0.311	0.221	0.158	0.370
ZNF44	65.775	0.071	0.242	0.771	0.848	65.775	-0.210	0.244	0.388	0.634	65.775	-0.281	0.283	0.321	0.555
SELENOT	1785.556	-0.517	0.202	0.010	0.035	1785.556	-0.404	0.202	0.045	0.334	1785.556	0.113	0.233	0.629	0.798
RAB23	602.109	-0.310	0.263	0.238	0.372	602.109	-0.626	0.263	0.017	0.306	602.109	-0.317	0.304	0.297	0.533
CES1P1	1425.720	1.570	0.294	0.000	0.000	1425.720	-0.367	0.295	0.213	0.493	1425.720	-1.937	0.340	0.000	0.000
CAB39	1736.354	-1.005	0.238	0.000	0.000	1736.354	-0.462	0.238	0.052	0.340	1736.354	0.543	0.275	0.048	0.179
UIMC1	845.357	-0.201	0.127	0.114	0.216	845.357	-0.037	0.127	0.768	0.877	845.357	0.164	0.147	0.265	0.499
DNAJB11	1700.874	-0.100	0.394	0.799	0.868	1700.874	-0.900	0.394	0.022	0.315	1700.874	-0.800	0.455	0.079	0.242
CMPK1	3621.001	0.036	0.102	0.726	0.814	3621.001	-0.124	0.102	0.224	0.503	3621.001	-0.160	0.118	0.175	0.393
POLR3K	384.486	-0.662	0.419	0.114	0.215	384.486	-0.983	0.419	0.019	0.306	384.486	-0.321	0.485	0.508	0.714
WBP11	1921.842	-0.412	0.209	0.049	0.114	1921.842	-0.461	0.209	0.028	0.321	1921.842	-0.048	0.242	0.842	0.923
MSRB1	293.812	0.284	0.232	0.222	0.353	293.812	-0.260	0.234	0.265	0.537	293.812	-0.544	0.270	0.044	0.168
RAPGEF6	387.305	-0.854	0.268	0.001	0.008	387.305	-0.257	0.266	0.333	0.592	387.305	0.597	0.309	0.053	0.191
WWOX	129.602	0.452	0.301	0.133	0.242	129.602	0.370	0.301	0.218	0.498	129.602	-0.082	0.347	0.813	0.907
ARID4B	788.471	0.186	0.149	0.212	0.341	788.471	0.040	0.149	0.786	0.888	788.471	-0.145	0.172	0.398	0.625
LUC7L3	1147.790	0.942	0.299	0.002	0.008	1147.790	0.029	0.300	0.922	0.960	1147.790	-0.912	0.346	0.008	0.058
PECAM1	23.435	-0.650	1.056	0.538	0.663	23.435	0.784	1.013	0.439	0.672	23.435	1.434	1.201	0.233	0.461
RTEL1	27.527	-2.281	0.539	0.000	0.000	27.527	-1.377	0.501	0.006	0.297	27.527	0.904	0.624	0.147	0.355
ERAP1	1090.896	-0.008	0.187	0.964	0.978	1090.896	0.113	0.187	0.546	0.741	1090.896	0.121	0.216	0.575	0.761
TMEM8B	128.483	0.982	0.436	0.024	0.067	128.483	0.631	0.437	0.148	0.441	128.483	-0.351	0.503	0.485	0.697
CDK12	909.676	-1.090	0.276	0.000	0.001	909.676	-0.603	0.275	0.029	0.321	909.676	0.487	0.319	0.127	0.322
C9orf78	867.709	-0.039	0.234	0.867	0.914	867.709	-0.492	0.234	0.036	0.324	867.709	-0.453	0.271	0.094	0.270
SERPINF1	20.013	0.749	0.734	0.308	0.447	20.013	0.226	0.739	0.760	0.872	20.013	-0.523	0.849	0.538	0.736
SYT17	50.223	1.862	0.566	0.001	0.006	50.223	0.863	0.576	0.134	0.426	50.223	-0.999	0.649	0.124	0.316
RAB8B	341.581	0.310	0.463	0.503	0.633	341.581	0.397	0.462	0.390	0.635	341.581	0.088	0.534	0.869	0.937
INPP5K	240.506	0.382	0.187	0.041	0.100	240.506	-0.041	0.189	0.826	0.909	240.506	-0.423	0.217	0.051	0.186
STK26	22.081	0.102	0.364	0.780	0.853	22.081	0.295	0.353	0.402	0.645	22.081	0.193	0.416	0.641	0.805
TM7SF3	760.776	-0.232	0.230	0.312	0.452	760.776	0.043	0.229	0.853	0.925	760.776	0.275	0.265	0.300	0.535
RSF1	1617.891	-0.038	0.165	0.816	0.879	1617.891	0.116	0.165	0.480	0.699	1617.891	0.155	0.190	0.416	0.642
MAP3K20	1845.018	-0.246	0.187	0.188	0.312	1845.018	0.067	0.186	0.718	0.849	1845.018	0.313	0.216	0.146	0.353
MYOZ2	66.218	2.510	0.236	0.000	0.000	66.218	0.008	0.272	0.975	0.986	66.218	-2.501	0.287	0.000	0.000
KDM3B	1492.632	-0.350	0.147	0.017	0.051	1492.632	-0.012	0.147	0.934	0.967	1492.632	0.338	0.170	0.046	0.174
ATP5MC3	2447.401	0.084	0.208	0.686	0.783	2447.401	-0.324	0.208	0.120	0.412	2447.401	-0.408	0.241	0.090	0.262
SIX4	243.926	-0.589	0.196	0.003	0.012	243.926	-0.391	0.193	0.043	0.331	243.926	0.198	0.227	0.382	0.611
COQ3	243.496	-0.421	0.309	0.173	0.293	243.496	-0.580	0.309	0.060	0.350	243.496	-0.158	0.358	0.658	0.817

TUBA8	8.820	0.539	0.807	0.504	0.634	8.820	0.381	0.806	0.637	0.802	8.820	-0.158	0.926	0.864	0.935
PHAX	661.306	-0.259	0.113	0.023	0.063	661.306	-0.302	0.113	0.007	0.297	661.306	-0.044	0.132	0.740	0.867
GALNT7	362.229	0.030	0.189	0.875	0.919	362.229	-0.472	0.190	0.013	0.305	362.229	-0.502	0.220	0.023	0.109
PEPD	1055.067	0.384	0.153	0.012	0.039	1055.067	-0.136	0.153	0.376	0.624	1055.067	-0.520	0.177	0.003	0.031
PER1	1651.021	0.018	0.239	0.941	0.963	1651.021	0.509	0.239	0.033	0.322	1651.021	0.491	0.276	0.075	0.236
GATB	355.057	-0.156	0.207	0.451	0.586	355.057	-0.013	0.206	0.951	0.976	355.057	0.143	0.239	0.549	0.744
PEX1	224.336	-0.211	0.202	0.296	0.434	224.336	0.161	0.199	0.420	0.657	224.336	0.372	0.233	0.110	0.296
PEX6	952.195	0.477	0.413	0.248	0.382	952.195	0.656	0.413	0.112	0.405	952.195	0.180	0.476	0.706	0.847
PEX7	129.429	-0.156	0.212	0.461	0.596	129.429	0.277	0.207	0.182	0.469	129.429	0.433	0.243	0.075	0.236
PEX10	866.789	0.236	0.144	0.101	0.197	866.789	0.094	0.144	0.512	0.719	866.789	-0.141	0.166	0.394	0.622
PEX12	175.294	-0.421	0.202	0.037	0.092	175.294	0.086	0.197	0.662	0.816	175.294	0.507	0.232	0.029	0.128
PEX13	549.851	0.268	0.106	0.011	0.038	549.851	0.056	0.106	0.597	0.776	549.851	-0.212	0.123	0.084	0.252
PEX14	710.624	0.031	0.150	0.837	0.893	710.624	-0.088	0.150	0.559	0.748	710.624	-0.119	0.174	0.495	0.703
PFAS	545.386	-0.906	0.399	0.023	0.064	545.386	-0.813	0.399	0.041	0.330	545.386	0.093	0.461	0.840	0.923
ACP1	1384.494	-0.379	0.125	0.002	0.011	1384.494	-0.124	0.124	0.318	0.579	1384.494	0.255	0.144	0.077	0.240
PFDN1	886.174	0.395	0.190	0.038	0.093	886.174	-0.059	0.190	0.758	0.871	886.174	-0.453	0.220	0.039	0.156
PFDN2	1907.516	0.257	0.413	0.533	0.659	1907.516	-0.554	0.413	0.180	0.468	1907.516	-0.811	0.477	0.089	0.261
PFDN4	361.952	0.258	0.200	0.199	0.325	361.952	-0.161	0.201	0.424	0.660	361.952	-0.419	0.233	0.072	0.229
PFDN5	2512.615	0.981	0.286	0.001	0.004	2512.615	0.412	0.286	0.150	0.442	2512.615	-0.569	0.330	0.085	0.253
ATP8B1	83.680	0.842	0.225	0.000	0.001	83.680	0.269	0.229	0.239	0.516	83.680	-0.572	0.260	0.028	0.125
PFKFB2	125.816	0.237	0.268	0.375	0.516	125.816	-0.292	0.270	0.279	0.549	125.816	-0.530	0.312	0.089	0.261
PFKFB3	1417.154	-0.567	0.270	0.036	0.090	1417.154	-0.218	0.270	0.419	0.656	1417.154	0.349	0.312	0.264	0.498
ATP5ME	630.581	1.027	0.253	0.000	0.001	630.581	0.099	0.254	0.696	0.837	630.581	-0.927	0.293	0.002	0.018
PFKFB4	1022.655	1.183	1.030	0.251	0.385	1022.655	0.916	1.030	0.374	0.622	1022.655	-0.267	1.189	0.823	0.913
PFKL	6320.636	0.060	0.274	0.826	0.886	6320.636	0.501	0.274	0.068	0.357	6320.636	0.440	0.317	0.165	0.379
PFKM	3381.272	-0.383	0.163	0.019	0.055	3381.272	0.115	0.163	0.479	0.699	3381.272	0.499	0.189	0.008	0.057
PFKP	8175.240	-0.101	0.158	0.524	0.651	8175.240	0.067	0.158	0.670	0.821	8175.240	0.168	0.183	0.357	0.590
PFN1	9428.904	0.189	0.231	0.414	0.552	9428.904	-0.165	0.231	0.475	0.697	9428.904	-0.354	0.267	0.185	0.404
PFN2	3153.858	-0.034	0.136	0.803	0.870	3153.858	0.014	0.136	0.916	0.958	3153.858	0.048	0.157	0.758	0.877
CDK14	212.727	-0.002	0.202	0.992	0.995	212.727	-0.133	0.201	0.510	0.718	212.727	-0.131	0.234	0.576	0.762
ATP5PF	1521.469	0.411	0.196	0.036	0.090	1521.469	-0.094	0.196	0.633	0.800	1521.469	-0.504	0.226	0.026	0.120
PGAM1	12865.585	-0.452	0.169	0.008	0.027	12865.585	-0.007	0.169	0.968	0.983	12865.585	0.445	0.195	0.023	0.110
PGAM2	11.057	1.358	0.852	0.111	0.211	11.057	1.229	0.851	0.149	0.441	11.057	-0.129	0.963	0.893	0.949
PGD	3660.320	0.656	0.155	0.000	0.000	3660.320	-0.288	0.155	0.063	0.350	3660.320	-0.944	0.179	0.000	0.000
PGF	1706.057	0.972	1.108	0.380	0.520	1706.057	0.877	1.108	0.428	0.663	1706.057	-0.095	1.279	0.941	0.973
PGGT1B	406.204	-0.531	0.184	0.004	0.016	406.204	-0.256	0.182	0.161	0.452	406.204	0.276	0.213	0.196	0.418
ATP6V1A	1064.625	0.004	0.144	0.979	0.987	1064.625	0.037	0.144	0.797	0.894	1064.625	0.033	0.167	0.842	0.923
PGK1	26305.986	-0.106	0.337	0.753	0.835	26305.986	0.548	0.337	0.105	0.397	26305.986	0.654	0.390	0.093	0.268
PGM1	3364.584	0.238	0.381	0.532	0.657	3364.584	0.538	0.381	0.158	0.450	3364.584	0.300	0.440	0.496	0.704
PGM3	940.046	-0.292	0.181	0.107	0.205	940.046	-0.094	0.180	0.604	0.781	940.046	0.199	0.209	0.342	0.576
ABCB1	85.695	-0.060	0.230	0.793	0.863	85.695	-0.297	0.231	0.197	0.481	85.695	-0.237	0.269	0.378	0.609
PHB	4061.106	-0.195	0.253	0.440	0.576	4061.106	-0.445	0.253	0.079	0.370	4061.106	-0.249	0.292	0.394	0.622
ATP6V1B1	16.569	1.663	0.607	0.006	0.023	16.569	0.082	0.632	0.896	0.948	16.569	-1.581	0.709	0.026	0.120
SLC25A3	9296.933	-0.299	0.097	0.002	0.010	9296.933	-0.019	0.097	0.842	0.918	9296.933	0.280	0.112	0.012	0.075
PHEX	78.324	0.915	0.332	0.006	0.022	78.324	0.246	0.337	0.465	0.689	78.324	-0.669	0.384	0.081	0.247
PHF1	1069.406	1.034	0.203	0.000	0.000	1069.406	0.448	0.204	0.028	0.321	1069.406	-0.586	0.234	0.012	0.075
PHF2	578.294	0.066	0.129	0.611	0.723	578.294	-0.159	0.129	0.217	0.496	578.294	-0.225	0.149	0.132	0.330
PHKA1	466.602	-0.613	0.239	0.010	0.035	466.602	0.064	0.237	0.788	0.889	466.602	0.676	0.276	0.014	0.082
PHKA2	326.108	-0.127	0.194	0.511	0.640	326.108	0.219	0.192	0.253	0.527	326.108	0.347	0.223	0.120	0.311
PHKB	1354.840	-0.714	0.239	0.003	0.013	1354.840	0.015	0.238	0.948	0.974	1354.840	0.729	0.276	0.008	0.057
ATP6V1B2	1748.143	-0.213	0.173	0.218	0.348	1748.143	-0.057	0.172	0.741	0.862	1748.143	0.156	0.200	0.434	0.656
PHKG1	189.672	0.832	0.273	0.002	0.011	189.672	0.262	0.275	0.340	0.598	189.672	-0.570	0.316	0.071	0.227
PHKG2	369.339	0.611	0.243	0.012	0.039	369.339	-0.371	0.245	0.130	0.422	369.339	-0.981	0.282	0.000	0.008
PHYH	472.021	0.849	0.213	0.000	0.001	472.021	0.259	0.214	0.225	0.504	472.021	-0.589	0.246	0.017	0.091
SERPINA1	638.958	1.616	0.459	0.000	0.003	638.958	0.846	0.460	0.066	0.354	638.958	-0.769	0.530	0.146	0.353
PI3	2424.421	1.984	0.464	0.000	0.000	2424.421	0.206	0.476	0.665	0.818	2424.421	-1.778	0.539	0.001	0.013
SERPINB5	810.133	-0.544	0.250	0.030	0.078	810.133	-0.160	0.249	0.521	0.725	810.133	0.384	0.289	0.183	0.402
SERPINB6	2001.853	0.464	0.192	0.016	0.049	2001.853	0.326	0.192	0.090	0.384	2001.853	-0.137	0.222	0.536	0.734
ATP6V0C	28.177	0.431	0.373	0.248	0.382	28.177	-0.065	0.383	0.864	0.931	28.177	-0.496	0.438	0.257	0.492
SERPINE2	152.533	1.032	0.382	0.007	0.025	152.533	-0.365	0.402	0.364	0.616	152.533	-1.397	0.453	0.002	0.022
SERPINB8	191.373	-0.770	0.338	0.023	0.063	191.373	-0.777	0.337	0.021	0.314	191.373	-0.006	0.393	0.987	0.993
SERPINI1	99.511	0.248	0.366	0.498	0.629	99.511	0.327	0.364	0.369	0.619	99.511	0.079	0.421	0.851	0.928
PIGA	213.412	-0.224	0.234	0.340	0.480	213.412	-0.237	0.233	0.309	0.574	213.412	-0.014	0.271	0.960	0.981
PIGC	419.218	0.491	0.125	0.000	0.001	419.218	-0.072	0.127	0.569	0.756	419.218	-0.563	0.146	0.000	0.003
ATP6V1C1	755.726	-0.094	0.212	0.657	0.760	755.726	-0.326	0.212	0.123	0.416	755.726	-0.232	0.245	0.343	0.577
PIGF	460.787	0.312	0.144	0.030	0.078	460.787	-0.006	0.144	0.968	0.983	460.787	-0.318	0.166	0.056	0.197
PIGH	311.999	0.054	0.136	0.689	0.785	311.999	0.069	0.134	0.607	0.783	311.999	0.015	0.157	0.925	0.965

PIK3C2A	1460.644	-0.447	0.263	0.090	0.180	1460.644	-0.505	0.263	0.055	0.342	1460.644	-0.058	0.304	0.848	0.926
PIK3C2B	136.459	0.293	0.477	0.538	0.663	136.459	0.551	0.476	0.246	0.521	136.459	0.258	0.550	0.639	0.804
PIK3C3	638.740	-0.046	0.199	0.819	0.881	638.740	0.174	0.198	0.379	0.626	638.740	0.220	0.230	0.338	0.573
ATP6V1E1	1098.884	0.533	0.232	0.022	0.061	1098.884	-0.369	0.233	0.113	0.405	1098.884	-0.901	0.268	0.001	0.011
PIK3CA	864.725	-0.763	0.178	0.000	0.000	864.725	-0.083	0.176	0.636	0.801	864.725	0.680	0.205	0.001	0.012
PIK3CB	860.111	-0.866	0.249	0.000	0.003	860.111	-0.223	0.248	0.367	0.617	860.111	0.643	0.287	0.025	0.117
PIM1	620.170	0.631	0.200	0.002	0.008	620.170	0.321	0.201	0.109	0.401	620.170	-0.310	0.231	0.180	0.398
PIK3CD	202.989	1.112	0.364	0.002	0.011	202.989	0.713	0.364	0.050	0.337	202.989	-0.398	0.419	0.342	0.576
PIK3R1	6583.994	-0.508	0.216	0.019	0.054	6583.994	-0.280	0.216	0.194	0.478	6583.994	0.228	0.249	0.360	0.593
PIK3R2	3917.191	0.426	0.327	0.193	0.318	3917.191	0.452	0.327	0.166	0.456	3917.191	0.027	0.377	0.944	0.974
PI4KA	1690.867	-0.302	0.172	0.079	0.164	1690.867	-0.011	0.171	0.950	0.976	1690.867	0.291	0.198	0.142	0.348
PI4KB	1062.774	-0.231	0.145	0.112	0.212	1062.774	0.065	0.144	0.652	0.810	1062.774	0.296	0.167	0.077	0.240
ACP2	328.883	-0.096	0.182	0.598	0.712	328.883	-0.245	0.182	0.178	0.466	328.883	-0.149	0.211	0.480	0.693
PIN1	1210.408	0.163	0.154	0.290	0.428	1210.408	-0.007	0.154	0.966	0.982	1210.408	-0.170	0.178	0.341	0.575
PIN4	232.797	1.286	0.308	0.000	0.000	232.797	0.272	0.311	0.381	0.627	232.797	-1.014	0.356	0.004	0.038
PIP4K2A	697.076	-0.317	0.213	0.137	0.247	697.076	-0.407	0.213	0.056	0.344	697.076	-0.090	0.247	0.717	0.853
PITPNA	922.721	-0.259	0.205	0.208	0.336	922.721	-0.305	0.205	0.138	0.429	922.721	-0.046	0.237	0.847	0.926
PITX1	1130.752	-0.088	0.298	0.766	0.846	1130.752	-0.182	0.298	0.542	0.738	1130.752	-0.093	0.344	0.787	0.894
PITX2	74.877	-0.245	0.195	0.209	0.338	74.877	0.158	0.185	0.393	0.637	74.877	0.403	0.222	0.070	0.226
PKD1	2986.374	-0.869	0.190	0.000	0.000	2986.374	-0.018	0.189	0.926	0.962	2986.374	0.851	0.219	0.000	0.002
PKD2	255.682	-0.051	0.213	0.813	0.877	255.682	-0.125	0.213	0.556	0.747	255.682	-0.075	0.247	0.762	0.879
PKM	59029.108	0.039	0.129	0.761	0.842	59029.108	0.186	0.129	0.150	0.443	59029.108	0.146	0.149	0.326	0.560
PKNOX1	290.171	-0.613	0.190	0.001	0.007	290.171	-0.307	0.187	0.101	0.394	290.171	0.306	0.220	0.164	0.379
PKP2	119.433	-0.213	0.240	0.375	0.515	119.433	0.374	0.233	0.107	0.400	119.433	0.587	0.274	0.032	0.138
PLA2G4A	136.082	-0.372	0.242	0.125	0.231	136.082	-0.295	0.240	0.220	0.499	136.082	0.077	0.281	0.784	0.892
PLAG1	227.526	-0.236	0.278	0.396	0.535	227.526	0.256	0.276	0.354	0.608	227.526	0.492	0.321	0.125	0.319
PLAGL1	284.749	0.395	0.334	0.236	0.369	284.749	0.575	0.333	0.084	0.376	284.749	0.179	0.385	0.641	0.805
PLAGL2	340.852	-0.367	0.225	0.103	0.200	340.852	-0.297	0.224	0.186	0.472	340.852	0.070	0.261	0.788	0.894
PLAT	3033.490	0.089	0.132	0.501	0.631	3033.490	0.007	0.132	0.955	0.978	3033.490	-0.081	0.152	0.593	0.773
PLAU	208.750	1.186	0.223	0.000	0.000	208.750	-0.166	0.228	0.466	0.690	208.750	-1.353	0.260	0.000	0.000
PLAUR	390.749	1.397	0.174	0.000	0.000	390.749	0.690	0.176	0.000	0.101	390.749	-0.706	0.199	0.000	0.007
ATP6V0B	1894.757	-0.115	0.391	0.769	0.847	1894.757	-0.918	0.391	0.019	0.306	1894.757	-0.803	0.452	0.075	0.236
PLCB3	1363.120	0.157	0.113	0.168	0.288	1363.120	-0.013	0.113	0.910	0.955	1363.120	-0.169	0.131	0.196	0.419
PLCB4	10.295	0.802	0.461	0.082	0.168	10.295	0.662	0.459	0.149	0.442	10.295	-0.141	0.517	0.786	0.893
PLCD1	73.361	0.464	0.492	0.346	0.487	73.361	0.747	0.490	0.127	0.420	73.361	0.283	0.566	0.616	0.789
BCL11A	15.314	0.576	0.721	0.424	0.561	15.314	1.171	0.707	0.098	0.392	15.314	0.595	0.817	0.467	0.682
BTBD1	1514.996	-0.789	0.218	0.000	0.002	1514.996	-0.238	0.217	0.274	0.545	1514.996	0.551	0.252	0.029	0.127
SPA17	193.886	0.369	0.218	0.090	0.182	193.886	0.076	0.219	0.727	0.854	193.886	-0.293	0.252	0.246	0.477
IL17D	47.839	0.244	0.277	0.378	0.519	47.839	0.401	0.271	0.138	0.429	47.839	0.158	0.315	0.617	0.789
NUDT9	398.156	0.137	0.178	0.441	0.577	398.156	-0.344	0.179	0.055	0.343	398.156	-0.481	0.207	0.020	0.102
CHIC1	130.876	-0.183	0.214	0.394	0.532	130.876	0.074	0.210	0.724	0.852	130.876	0.257	0.247	0.297	0.533
TM6SF1	34.605	-0.681	0.316	0.031	0.081	34.605	0.267	0.289	0.355	0.609	34.605	0.948	0.357	0.008	0.056
ZFYVE1	495.379	0.101	0.299	0.734	0.821	495.379	0.530	0.298	0.075	0.364	495.379	0.428	0.344	0.213	0.439
PLCG1	1297.339	-0.038	0.146	0.797	0.866	1297.339	0.055	0.145	0.706	0.842	1297.339	0.092	0.168	0.582	0.765
LRP1B	219.545	-0.526	0.558	0.346	0.486	219.545	0.374	0.556	0.501	0.713	219.545	0.900	0.643	0.162	0.375
PANK1	103.658	-0.169	0.281	0.548	0.671	103.658	0.147	0.277	0.595	0.775	103.658	0.316	0.324	0.329	0.563
SHC3	22.670	0.795	0.781	0.309	0.449	22.670	-0.089	0.793	0.910	0.955	22.670	-0.884	0.909	0.331	0.565
PLCG2	163.102	5.227	0.332	0.000	0.000	163.102	1.183	0.357	0.001	0.294	163.102	-4.044	0.375	0.000	0.000
PLD1	34.987	0.286	0.380	0.451	0.586	34.987	0.447	0.375	0.233	0.511	34.987	0.160	0.435	0.713	0.850
NUP54	432.825	-0.585	0.173	0.001	0.004	432.825	-0.197	0.170	0.249	0.523	432.825	0.389	0.199	0.051	0.186
TPCN1	1345.520	-0.090	0.177	0.610	0.722	1345.520	0.133	0.177	0.453	0.680	1345.520	0.223	0.204	0.275	0.510
PLD2	321.209	0.502	0.379	0.185	0.309	321.209	0.630	0.379	0.096	0.391	321.209	0.128	0.437	0.770	0.883
PLEC	10001.487	-0.064	0.143	0.652	0.756	10001.487	-0.016	0.143	0.913	0.957	10001.487	0.049	0.165	0.767	0.881
ATP6V1G2	214.532	1.073	0.434	0.013	0.042	214.532	1.805	0.432	0.000	0.056	214.532	0.732	0.498	0.141	0.346
STX18	542.223	-0.257	0.200	0.198	0.324	542.223	-0.395	0.200	0.048	0.337	542.223	-0.138	0.231	0.551	0.745
SERPINF2	24.630	2.065	0.732	0.005	0.019	24.630	0.982	0.742	0.186	0.472	24.630	-1.083	0.839	0.196	0.419
PLIN1	14.884	1.055	0.820	0.198	0.324	14.884	0.971	0.818	0.235	0.513	14.884	-0.084	0.935	0.929	0.967
PLK1	2060.466	-0.924	0.231	0.000	0.001	2060.466	0.073	0.230	0.752	0.867	2060.466	0.997	0.266	0.000	0.004
FXYD3	14.602	1.077	0.450	0.017	0.050	14.602	0.466	0.465	0.316	0.578	14.602	-0.611	0.517	0.237	0.467
ATP6V0A1	631.408	0.225	0.100	0.024	0.066	631.408	0.087	0.100	0.380	0.627	631.408	-0.137	0.115	0.233	0.462
PLOD1	7332.672	0.188	0.304	0.535	0.660	7332.672	0.505	0.303	0.096	0.391	7332.672	0.316	0.350	0.367	0.599
PLOD2	370.893	-0.056	0.238	0.816	0.879	370.893	-0.403	0.239	0.092	0.386	370.893	-0.347	0.276	0.209	0.434
PLP2	5385.253	0.528	0.246	0.032	0.082	5385.253	0.425	0.246	0.084	0.376	5385.253	-0.103	0.284	0.716	0.853
PLRG1	1307.249	-0.750	0.162	0.000	0.000	1307.249	-0.253	0.161	0.116	0.409	1307.249	0.497	0.187	0.008	0.056
PLS1	685.538	-0.899	0.216	0.000	0.000	685.538	-0.327	0.214	0.127	0.420	685.538	0.572	0.249	0.022	0.107
PLS3	3513.507	-0.274	0.190	0.149	0.263	3513.507	-0.145	0.190	0.447	0.677	3513.507	0.130	0.220	0.555	0.748
PLSCR1	918.947	0.342	0.143	0.017	0.050	918.947	-0.219	0.143	0.127	0.420	918.947	-0.560	0.165	0.001	0.010

PLTP	137.049	2.277	0.340	0.000	0.000	137.049	0.195	0.352	0.580	0.764	137.049	-2.082	0.396	0.000	0.000
PLXNA1	2095.052	-0.276	0.172	0.108	0.207	2095.052	-0.137	0.171	0.422	0.659	2095.052	0.138	0.198	0.485	0.697
MBD3	2183.615	0.172	0.165	0.297	0.436	2183.615	-0.108	0.165	0.511	0.719	2183.615	-0.280	0.190	0.141	0.346
ADAM22	91.087	0.042	0.415	0.918	0.948	91.087	0.132	0.413	0.750	0.867	91.087	0.089	0.479	0.852	0.929
PLXNA2	1753.948	-1.224	0.347	0.000	0.003	1753.948	-0.741	0.347	0.033	0.322	1753.948	0.483	0.401	0.229	0.457
PTOV1	1762.790	0.983	0.231	0.000	0.000	1762.790	0.300	0.231	0.195	0.479	1762.790	-0.684	0.267	0.010	0.068
S1PR5	42.827	1.189	0.759	0.117	0.220	42.827	0.357	0.763	0.639	0.803	42.827	-0.832	0.877	0.343	0.577
PLXNB1	2003.855	0.301	0.324	0.353	0.494	2003.855	0.457	0.324	0.159	0.451	2003.855	0.156	0.374	0.677	0.829
PLXNB3	481.038	0.495	0.568	0.384	0.523	481.038	1.067	0.568	0.060	0.350	481.038	0.573	0.656	0.382	0.611
PMAIP1	510.334	-0.019	0.435	0.966	0.979	510.334	-1.220	0.438	0.005	0.297	510.334	-1.201	0.505	0.017	0.094
PMCH	105.504	-0.623	0.290	0.032	0.081	105.504	-0.368	0.286	0.198	0.482	105.504	0.255	0.335	0.447	0.666
ATP6AP1	1759.855	0.665	0.163	0.000	0.001	1759.855	0.212	0.163	0.193	0.478	1759.855	-0.452	0.188	0.016	0.090
PML	1922.859	0.897	0.254	0.000	0.003	1922.859	0.550	0.254	0.031	0.322	1922.859	-0.347	0.294	0.237	0.466
PMM1	656.646	0.319	0.198	0.107	0.206	656.646	0.458	0.198	0.021	0.312	656.646	0.139	0.229	0.543	0.740
PMM2	338.337	-0.864	0.309	0.005	0.020	338.337	-0.831	0.309	0.007	0.297	338.337	0.033	0.358	0.927	0.966
PMP22	295.873	0.876	0.217	0.000	0.001	295.873	0.487	0.218	0.026	0.316	295.873	-0.389	0.250	0.120	0.312
PMS1	240.252	-0.717	0.247	0.004	0.016	240.252	-0.518	0.245	0.035	0.322	240.252	0.199	0.286	0.488	0.698
PMS2P1	128.601	-0.255	0.200	0.202	0.329	128.601	-0.237	0.198	0.230	0.508	128.601	0.018	0.232	0.939	0.973
ATP7A	195.733	-0.275	0.385	0.475	0.608	195.733	0.024	0.383	0.950	0.976	195.733	0.299	0.444	0.501	0.708
PMS2P2	46.897	-0.945	0.349	0.007	0.025	46.897	0.033	0.328	0.921	0.960	46.897	0.978	0.398	0.014	0.082
PMS2P4	18.372	-0.429	0.405	0.290	0.428	18.372	-0.248	0.391	0.527	0.729	18.372	0.181	0.471	0.701	0.844
FXYD5	1391.370	1.186	0.250	0.000	0.000	1391.370	0.301	0.251	0.230	0.508	1391.370	-0.885	0.289	0.002	0.023
PMS2P5	14.524	-0.755	0.464	0.104	0.201	14.524	-0.223	0.432	0.606	0.782	14.524	0.532	0.534	0.319	0.554
IL20RA	58.204	-0.094	0.407	0.818	0.880	58.204	0.611	0.399	0.126	0.418	58.204	0.705	0.466	0.130	0.327
IL20RB	2376.591	-0.692	0.259	0.008	0.027	2376.591	0.395	0.259	0.126	0.419	2376.591	1.087	0.299	0.000	0.005
FGFRL1	632.384	-0.249	0.118	0.034	0.087	632.384	0.040	0.116	0.731	0.856	632.384	0.289	0.136	0.033	0.140
GPR87	157.381	-0.605	0.284	0.033	0.084	157.381	-0.303	0.281	0.281	0.551	157.381	0.302	0.328	0.357	0.590
C11orf24	708.364	0.030	0.349	0.931	0.956	708.364	-0.859	0.350	0.014	0.306	708.364	-0.889	0.404	0.028	0.125
TRIM34	8.187	-0.043	0.665	0.948	0.968	8.187	0.672	0.631	0.287	0.555	8.187	0.715	0.749	0.340	0.574
CLDN22	30.393	0.539	0.417	0.196	0.322	30.393	-0.180	0.427	0.672	0.823	30.393	-0.720	0.488	0.140	0.344
PMS2P3	43.791	0.338	0.343	0.325	0.465	43.791	0.155	0.343	0.652	0.810	43.791	-0.183	0.396	0.645	0.807
ATP5PO	2987.447	0.233	0.177	0.188	0.313	2987.447	0.105	0.177	0.552	0.745	2987.447	-0.128	0.205	0.532	0.732
DUOX1	9.992	1.003	0.618	0.105	0.202	9.992	-0.186	0.655	0.776	0.881	9.992	-1.189	0.734	0.105	0.289
RAB24	417.456	0.261	0.178	0.143	0.255	417.456	0.240	0.178	0.178	0.466	417.456	-0.021	0.206	0.917	0.961
PELO	1304.323	-0.181	0.214	0.398	0.536	1304.323	-0.154	0.214	0.470	0.692	1304.323	0.026	0.247	0.915	0.960
EXOSC9	486.271	-0.014	0.281	0.959	0.975	486.271	-0.437	0.281	0.120	0.412	486.271	-0.423	0.325	0.194	0.416
PPIL3	468.653	0.522	0.198	0.008	0.030	468.653	0.273	0.199	0.170	0.459	468.653	-0.250	0.229	0.276	0.511
EXOSC10	1382.643	-0.092	0.144	0.525	0.652	1382.643	-0.188	0.144	0.193	0.478	1382.643	-0.096	0.167	0.565	0.755
CSNK1G1	396.389	-0.635	0.224	0.005	0.018	396.389	-0.129	0.222	0.561	0.750	396.389	0.506	0.258	0.050	0.183
A4GALT	914.953	1.131	0.193	0.000	0.000	914.953	0.353	0.194	0.069	0.357	914.953	-0.778	0.223	0.000	0.008
PMS2	772.802	-0.479	0.142	0.001	0.004	772.802	0.048	0.140	0.734	0.858	772.802	0.527	0.163	0.001	0.016
CPSF2	1202.164	-0.853	0.361	0.018	0.053	1202.164	-1.045	0.361	0.004	0.297	1202.164	-0.192	0.417	0.644	0.807
ATP7B	119.283	0.161	0.220	0.465	0.600	119.283	0.518	0.215	0.016	0.306	119.283	0.358	0.251	0.154	0.364
BRWD1	746.875	-0.348	0.169	0.039	0.096	746.875	0.013	0.168	0.937	0.968	746.875	0.362	0.195	0.064	0.212
SLC37A1	97.401	0.853	0.188	0.000	0.000	97.401	-0.002	0.196	0.991	0.995	97.401	-0.856	0.221	0.000	0.003
PCBP3	814.667	1.141	0.356	0.001	0.007	814.667	0.780	0.356	0.029	0.321	814.667	-0.361	0.411	0.380	0.610
C21orf58	112.805	-0.015	0.342	0.964	0.978	112.805	0.141	0.341	0.680	0.827	112.805	0.156	0.395	0.693	0.839
YBEY	255.200	0.286	0.300	0.340	0.480	255.200	0.509	0.298	0.088	0.380	255.200	0.223	0.345	0.518	0.721
C21orf62-AS1	13.913	0.368	0.861	0.669	0.769	13.913	0.857	0.850	0.313	0.576	13.913	0.489	0.984	0.619	0.792
MIS18A	442.592	-0.250	0.284	0.378	0.518	442.592	-0.342	0.284	0.228	0.506	442.592	-0.091	0.328	0.781	0.890
SETD4	167.267	-0.255	0.250	0.307	0.446	167.267	-0.299	0.249	0.230	0.507	167.267	-0.044	0.289	0.880	0.943
RIPK4	2563.445	0.189	0.413	0.647	0.753	2563.445	0.550	0.413	0.183	0.470	2563.445	0.361	0.477	0.449	0.669
GSAP	7.005	-0.075	0.856	0.930	0.956	7.005	0.343	0.830	0.679	0.827	7.005	0.418	0.977	0.669	0.824
POLE3	1237.825	-0.753	0.302	0.013	0.041	1237.825	-0.672	0.302	0.026	0.318	1237.825	0.081	0.349	0.816	0.908
CHRAC1	490.537	0.088	0.151	0.562	0.683	490.537	-0.030	0.151	0.842	0.918	490.537	-0.118	0.175	0.501	0.708
PNN	2007.970	-0.472	0.267	0.078	0.161	2007.970	-0.603	0.267	0.024	0.316	2007.970	-0.131	0.309	0.671	0.825
UBL3	767.484	-0.892	0.232	0.000	0.001	767.484	0.083	0.230	0.717	0.848	767.484	0.975	0.267	0.000	0.005
SEPTIN4	17.323	1.829	1.008	0.070	0.149	17.323	1.080	1.013	0.287	0.555	17.323	-0.750	1.157	0.517	0.721
LINC00308	8.662	0.016	0.711	0.982	0.989	8.662	0.221	0.698	0.751	0.867	8.662	0.205	0.816	0.802	0.901
H2BS1	9.445	-2.324	1.156	0.044	0.105	9.445	0.036	0.869	0.967	0.982	9.445	2.361	1.263	0.062	0.209
LURAP1	27.268	-0.215	0.396	0.587	0.703	27.268	-0.042	0.387	0.913	0.957	27.268	0.172	0.457	0.706	0.847
MIR4435-2HG	747.516	0.065	0.088	0.458	0.593	747.516	0.007	0.087	0.935	0.967	747.516	-0.058	0.101	0.567	0.757
IL6-AS1	36.060	0.118	0.747	0.874	0.919	36.060	-1.798	0.780	0.021	0.314	36.060	-1.916	0.893	0.032	0.137
LOC541473	13.240	1.809	0.719	0.012	0.039	13.240	1.131	0.731	0.122	0.413	13.240	-0.678	0.812	0.403	0.630
MRPL39	515.000	-0.144	0.178	0.418	0.555	515.000	-0.269	0.178	0.130	0.422	515.000	-0.125	0.206	0.545	0.741
C21orf91	263.580	-0.727	0.217	0.001	0.005	263.580	-0.181	0.214	0.398	0.642	263.580	0.547	0.251	0.029	0.130
C8orf58	275.753	0.879	0.425	0.039	0.095	275.753	0.649	0.425	0.127	0.419	275.753	-0.230	0.490	0.639	0.804

CXorf40B	377.078	-0.485	0.212	0.022	0.062	377.078	-0.277	0.210	0.187	0.473	377.078	0.208	0.245	0.396	0.624
DCUN1D1	776.784	-0.320	0.171	0.061	0.135	776.784	-0.267	0.171	0.118	0.409	776.784	0.053	0.198	0.788	0.894
NANS	1675.554	0.149	0.279	0.594	0.709	1675.554	-0.356	0.279	0.202	0.486	1675.554	-0.505	0.322	0.118	0.307
PODXL	108.928	2.130	0.285	0.000	0.000	108.928	0.477	0.295	0.105	0.398	108.928	-1.653	0.330	0.000	0.000
CYCS	3165.811	-0.652	0.443	0.141	0.252	3165.811	-1.420	0.443	0.001	0.297	3165.811	-0.768	0.511	0.133	0.332
ERRFI1	6178.802	0.222	0.191	0.245	0.379	6178.802	-0.088	0.191	0.644	0.806	6178.802	-0.310	0.220	0.160	0.372
TREM2	192.737	-3.044	1.068	0.004	0.018	192.737	0.556	1.058	0.599	0.777	192.737	3.600	1.230	0.003	0.032
TREM1	1537.740	-1.296	0.422	0.002	0.010	1537.740	0.981	0.420	0.019	0.306	1537.740	2.277	0.487	0.000	0.000
POLA1	510.309	-0.844	0.312	0.007	0.025	510.309	-0.928	0.311	0.003	0.297	510.309	-0.084	0.361	0.815	0.908
POLB	267.642	-0.186	0.192	0.335	0.475	267.642	-0.362	0.192	0.060	0.350	267.642	-0.176	0.223	0.430	0.653
POLD1	1483.772	-0.631	0.272	0.020	0.058	1483.772	-0.362	0.271	0.182	0.469	1483.772	0.269	0.314	0.391	0.620
POLD2	3234.502	-0.206	0.187	0.271	0.408	3234.502	-0.167	0.187	0.373	0.621	3234.502	0.039	0.216	0.856	0.931
POLE	760.370	-0.439	0.249	0.078	0.161	760.370	-0.392	0.248	0.115	0.407	760.370	0.048	0.288	0.869	0.937
POLE2	73.414	-1.032	0.551	0.061	0.134	73.414	-1.415	0.553	0.010	0.299	73.414	-0.383	0.642	0.550	0.745
POLG	1497.158	-0.286	0.199	0.152	0.267	1497.158	-0.198	0.199	0.319	0.581	1497.158	0.087	0.230	0.704	0.847
POLH	313.802	-0.448	0.203	0.028	0.074	313.802	0.114	0.200	0.570	0.756	313.802	0.561	0.234	0.016	0.090
POLR2A	1855.997	-0.230	0.178	0.198	0.324	1855.997	0.106	0.178	0.551	0.743	1855.997	0.336	0.206	0.103	0.286
POLR2B	1558.086	-0.570	0.164	0.000	0.003	1558.086	-0.317	0.163	0.052	0.340	1558.086	0.253	0.189	0.181	0.400
POLR2C	1087.844	-0.202	0.199	0.310	0.450	1087.844	-0.227	0.199	0.254	0.527	1087.844	-0.025	0.230	0.914	0.960
GPR173	46.629	0.678	0.512	0.186	0.310	46.629	0.801	0.510	0.116	0.409	46.629	0.123	0.588	0.834	0.919
GPR85	13.656	1.405	0.393	0.000	0.003	13.656	-0.301	0.445	0.500	0.713	13.656	-1.705	0.483	0.000	0.007
POLR2D	691.980	-0.579	0.213	0.007	0.025	691.980	-0.372	0.213	0.080	0.371	691.980	0.207	0.247	0.400	0.627
GNG2	138.379	0.971	0.369	0.009	0.030	138.379	-0.253	0.374	0.500	0.713	138.379	-1.223	0.429	0.004	0.037
GDAP1	156.979	-1.089	0.346	0.002	0.009	156.979	-0.735	0.343	0.032	0.322	156.979	0.354	0.401	0.377	0.609
POLR2E	4498.451	-0.055	0.205	0.787	0.859	4498.451	-0.274	0.205	0.181	0.469	4498.451	-0.218	0.236	0.356	0.589
DPM3	501.152	1.162	0.263	0.000	0.000	501.152	0.418	0.264	0.114	0.405	501.152	-0.743	0.304	0.014	0.083
POLR2F	753.210	0.447	0.293	0.127	0.234	753.210	-0.311	0.294	0.290	0.558	753.210	-0.758	0.339	0.025	0.118
POLR2G	694.782	0.163	0.166	0.326	0.466	694.782	0.067	0.166	0.684	0.829	694.782	-0.096	0.191	0.617	0.790
WNT4	18.069	-0.644	0.966	0.505	0.635	18.069	0.666	0.946	0.481	0.700	18.069	1.310	1.107	0.237	0.466
POLR2H	1156.307	0.307	0.193	0.113	0.214	1156.307	0.073	0.193	0.706	0.842	1156.307	-0.234	0.223	0.295	0.531
POLR2I	468.113	0.523	0.284	0.065	0.141	468.113	0.129	0.284	0.649	0.809	468.113	-0.394	0.328	0.229	0.458
TERF2IP	1731.567	0.048	0.120	0.690	0.786	1731.567	-0.042	0.120	0.727	0.854	1731.567	-0.090	0.139	0.518	0.721
POLR2J	484.270	0.836	0.269	0.002	0.009	484.270	0.147	0.270	0.587	0.769	484.270	-0.689	0.311	0.027	0.122
POLR2K	483.773	0.272	0.282	0.334	0.475	483.773	-0.558	0.283	0.049	0.337	483.773	-0.830	0.327	0.011	0.070
SLC38A2	9586.873	-0.719	0.253	0.005	0.018	9586.873	-0.546	0.253	0.031	0.322	9586.873	0.172	0.293	0.556	0.749
POLR2L	2497.046	0.456	0.183	0.013	0.041	2497.046	-0.216	0.184	0.240	0.516	2497.046	-0.671	0.212	0.002	0.018
NLGN3	8.056	1.820	0.672	0.007	0.025	8.056	1.576	0.675	0.019	0.306	8.056	-0.244	0.725	0.736	0.864
SIAE	1085.504	-0.105	0.240	0.662	0.765	1085.504	0.349	0.239	0.144	0.436	1085.504	0.454	0.277	0.101	0.282
POLRMT	1241.721	-0.386	0.322	0.231	0.363	1241.721	-0.613	0.322	0.057	0.347	1241.721	-0.226	0.373	0.543	0.740
DNAJC10	1210.550	-0.304	0.270	0.260	0.395	1210.550	-0.632	0.270	0.019	0.306	1210.550	-0.328	0.312	0.293	0.528
YIPF1	232.450	0.518	0.185	0.005	0.020	232.450	-0.128	0.188	0.496	0.710	232.450	-0.646	0.215	0.003	0.027
GAR1	596.544	-0.571	0.350	0.103	0.200	596.544	-0.712	0.350	0.042	0.330	596.544	-0.141	0.405	0.728	0.859
SSH1	1842.348	-0.790	0.242	0.001	0.006	1842.348	-0.385	0.242	0.111	0.405	1842.348	0.405	0.279	0.147	0.355
HCG4	309.436	0.717	0.337	0.034	0.086	309.436	0.878	0.337	0.009	0.297	309.436	0.161	0.388	0.679	0.830
SH3TC1	781.461	0.298	0.192	0.121	0.225	781.461	-0.010	0.192	0.958	0.978	781.461	-0.308	0.222	0.165	0.380
GFOD1	233.508	-0.222	0.212	0.295	0.434	233.508	-0.359	0.212	0.091	0.385	233.508	-0.137	0.246	0.579	0.764
RBM27	753.362	-0.719	0.235	0.002	0.010	753.362	-0.527	0.234	0.024	0.316	753.362	0.192	0.271	0.478	0.691
STAG3L1	93.541	-0.065	0.262	0.804	0.871	93.541	-0.011	0.259	0.967	0.983	93.541	0.054	0.303	0.858	0.932
KCTD5	737.978	-0.282	0.238	0.236	0.369	737.978	-0.559	0.238	0.019	0.306	737.978	-0.277	0.275	0.314	0.549
ANLN	1891.697	-0.565	0.256	0.027	0.073	1891.697	-0.422	0.256	0.099	0.393	1891.697	0.142	0.296	0.631	0.799
PON2	2104.434	-0.007	0.191	0.972	0.982	2104.434	0.186	0.191	0.330	0.590	2104.434	0.193	0.221	0.382	0.611
RIN2	920.039	-0.491	0.189	0.009	0.032	920.039	-0.273	0.188	0.147	0.439	920.039	0.218	0.218	0.319	0.554
ATAD2B	121.170	-0.596	0.308	0.053	0.120	121.170	-0.344	0.304	0.259	0.531	121.170	0.252	0.356	0.479	0.692
FBXO42	726.980	-0.224	0.168	0.183	0.307	726.980	-0.018	0.167	0.916	0.958	726.980	0.206	0.194	0.288	0.523
PRR13	694.773	0.537	0.209	0.010	0.035	694.773	-0.042	0.210	0.840	0.917	694.773	-0.579	0.242	0.017	0.091
MRPS21	1109.405	0.575	0.201	0.004	0.018	1109.405	0.027	0.202	0.895	0.948	1109.405	-0.548	0.233	0.018	0.096
FBXW5	2932.915	0.438	0.144	0.002	0.011	2932.915	0.254	0.144	0.078	0.369	2932.915	-0.184	0.166	0.269	0.504
CCSER2	965.999	-0.462	0.217	0.033	0.084	965.999	-0.132	0.216	0.541	0.737	965.999	0.330	0.250	0.187	0.407
RETREG1	64.850	1.957	0.443	0.000	0.000	64.850	0.411	0.453	0.364	0.615	64.850	-1.546	0.512	0.003	0.026
XRN1	762.562	-0.532	0.257	0.038	0.094	762.562	-0.025	0.256	0.921	0.960	762.562	0.507	0.297	0.088	0.258
ETAA1	266.998	-0.463	0.234	0.048	0.112	266.998	-0.129	0.232	0.578	0.763	266.998	0.334	0.270	0.216	0.442
SPIN2A	64.559	0.285	0.329	0.386	0.525	64.559	0.329	0.326	0.313	0.576	64.559	0.044	0.378	0.907	0.957
ANKIB1	919.535	-0.385	0.218	0.078	0.162	919.535	-0.021	0.217	0.921	0.960	919.535	0.363	0.252	0.149	0.357
MIOS	1603.189	-0.758	0.179	0.000	0.000	1603.189	0.001	0.179	0.994	0.997	1603.189	0.759	0.207	0.000	0.005
ZFAND6	879.173	-0.328	0.132	0.013	0.041	879.173	-0.007	0.131	0.957	0.978	879.173	0.321	0.152	0.035	0.145
POR	1816.861	0.946	0.125	0.000	0.000	1816.861	0.135	0.126	0.282	0.552	1816.861	-0.811	0.144	0.000	0.000
ARMCX6	390.921	0.575	0.208	0.006	0.022	390.921	0.458	0.208	0.028	0.321	390.921	-0.117	0.240	0.624	0.794

MIEF1	1307.281	-0.951	0.228	0.000	0.000	1307.281	-0.372	0.227	0.101	0.394	1307.281	0.578	0.263	0.028	0.125
TOLLIP	1161.475	0.559	0.147	0.000	0.001	1161.475	0.275	0.147	0.061	0.350	1161.475	-0.284	0.169	0.093	0.268
KRT20	33.397	0.208	0.437	0.634	0.742	33.397	0.537	0.428	0.210	0.491	33.397	0.329	0.499	0.510	0.715
NLE1	1015.606	-0.746	0.112	0.000	0.000	1015.606	-0.019	0.110	0.862	0.930	1015.606	0.727	0.129	0.000	0.000
RNF216	1209.983	-0.256	0.169	0.130	0.238	1209.983	-0.270	0.169	0.109	0.401	1209.983	-0.014	0.195	0.941	0.973
PLEKHA5	191.600	0.557	0.146	0.000	0.001	191.600	0.242	0.146	0.099	0.394	191.600	-0.316	0.168	0.060	0.206
PIMREG	295.616	-0.213	0.208	0.308	0.447	295.616	0.071	0.207	0.731	0.856	295.616	0.284	0.240	0.238	0.468
CHPF2	655.886	1.011	0.274	0.000	0.002	655.886	-0.522	0.278	0.060	0.350	655.886	-1.533	0.319	0.000	0.000
TRMT13	238.036	-0.779	0.218	0.000	0.003	238.036	-0.491	0.216	0.023	0.315	238.036	0.289	0.253	0.254	0.486
DGCR8	344.050	-0.280	0.335	0.402	0.540	344.050	-1.195	0.338	0.000	0.243	344.050	-0.914	0.390	0.019	0.098
OTULINL	11.611	-0.293	0.550	0.594	0.709	11.611	0.112	0.529	0.832	0.914	11.611	0.405	0.630	0.520	0.723
NEURL1B	49.741	0.246	0.856	0.774	0.850	49.741	0.541	0.854	0.526	0.729	49.741	0.296	0.987	0.765	0.881
C11orf71	47.164	-0.072	0.273	0.791	0.862	47.164	0.076	0.267	0.775	0.881	47.164	0.148	0.314	0.637	0.803
TMX3	1019.044	-0.610	0.174	0.000	0.003	1019.044	-0.367	0.173	0.034	0.322	1019.044	0.243	0.201	0.226	0.454
PRMT7	464.754	-0.095	0.249	0.702	0.795	464.754	-0.388	0.249	0.119	0.411	464.754	-0.293	0.288	0.309	0.544
HEATR5B	401.321	-0.768	0.233	0.001	0.006	401.321	0.254	0.230	0.268	0.539	401.321	1.022	0.268	0.000	0.003
SMOX	745.397	-0.919	0.272	0.001	0.004	745.397	-0.183	0.270	0.497	0.711	745.397	0.735	0.313	0.019	0.098
TMCO1	1789.138	-0.033	0.091	0.714	0.804	1789.138	-0.172	0.091	0.058	0.348	1789.138	-0.138	0.105	0.187	0.407
ATR	566.886	-0.814	0.337	0.016	0.048	566.886	-0.877	0.336	0.009	0.297	566.886	-0.063	0.389	0.872	0.939
RBM47	187.689	-0.159	0.285	0.577	0.695	187.689	0.028	0.283	0.922	0.960	187.689	0.187	0.329	0.570	0.759
ZDHHC13	120.344	0.132	0.222	0.552	0.674	120.344	-0.181	0.223	0.418	0.656	120.344	-0.312	0.258	0.226	0.454
CPVL	33.330	0.771	0.293	0.009	0.030	33.330	-0.618	0.331	0.062	0.350	33.330	-1.389	0.366	0.000	0.003
DHX29	860.062	-0.584	0.223	0.009	0.031	860.062	-0.517	0.223	0.020	0.310	860.062	0.067	0.258	0.795	0.897
ADAMTSL4	1214.730	1.113	0.322	0.001	0.004	1214.730	0.771	0.322	0.016	0.306	1214.730	-0.342	0.371	0.357	0.590
RHOF	384.431	0.206	0.126	0.104	0.201	384.431	0.046	0.126	0.716	0.848	384.431	-0.160	0.146	0.275	0.510
POU2F1	400.758	0.566	0.223	0.011	0.037	400.758	0.114	0.224	0.611	0.786	400.758	-0.452	0.258	0.079	0.244
PCDH18	37.551	-0.582	0.518	0.261	0.397	37.551	0.200	0.507	0.693	0.834	37.551	0.782	0.595	0.189	0.409
EXOSC4	527.239	-0.215	0.258	0.405	0.543	527.239	-0.297	0.258	0.250	0.523	527.239	-0.082	0.298	0.784	0.892
MTRF1L	694.247	-0.254	0.183	0.165	0.284	694.247	-0.549	0.183	0.003	0.297	694.247	-0.295	0.212	0.164	0.379
PUS7	378.378	-0.509	0.277	0.066	0.143	378.378	-0.627	0.277	0.024	0.316	378.378	-0.118	0.321	0.713	0.851
POU2F2	9.249	1.081	0.654	0.098	0.193	9.249	0.438	0.674	0.516	0.721	9.249	-0.643	0.752	0.393	0.621
CCDC93	702.074	-0.485	0.238	0.042	0.100	702.074	-0.328	0.237	0.167	0.456	702.074	0.157	0.275	0.568	0.758
WDR44	155.788	-0.398	0.265	0.133	0.241	155.788	-0.111	0.262	0.672	0.823	155.788	0.288	0.306	0.347	0.581
ANKRD16	183.496	0.021	0.217	0.923	0.951	183.496	0.373	0.214	0.081	0.372	183.496	0.352	0.249	0.158	0.370
ASNSD1	491.037	-0.393	0.177	0.026	0.071	491.037	-0.164	0.175	0.351	0.605	491.037	0.229	0.204	0.262	0.497
MIER2	573.483	0.226	0.157	0.149	0.262	573.483	-0.074	0.157	0.640	0.804	573.483	-0.300	0.182	0.098	0.277
USP53	3149.115	-0.688	0.225	0.002	0.011	3149.115	-0.510	0.225	0.023	0.316	3149.115	0.179	0.260	0.492	0.701
MRPL50	549.040	-0.096	0.169	0.571	0.690	549.040	-0.215	0.169	0.203	0.486	549.040	-0.119	0.196	0.542	0.739
CCHCR1	1170.547	0.700	0.227	0.002	0.010	1170.547	0.326	0.227	0.152	0.444	1170.547	-0.374	0.262	0.154	0.364
EXOC6	179.320	-0.712	0.287	0.013	0.042	179.320	-0.553	0.286	0.053	0.341	179.320	0.158	0.333	0.634	0.801
SHLD2	1180.056	-1.064	0.270	0.000	0.001	1180.056	-0.277	0.269	0.302	0.568	1180.056	0.787	0.311	0.011	0.071
NDUFB11	1382.967	0.215	0.193	0.265	0.401	1382.967	0.236	0.193	0.221	0.500	1382.967	0.021	0.223	0.924	0.965
FAM193B	320.218	1.088	0.328	0.001	0.005	320.218	0.543	0.329	0.099	0.393	320.218	-0.546	0.378	0.149	0.357
DDIT4	42870.233	-0.332	0.143	0.020	0.059	42870.233	0.232	0.143	0.106	0.398	42870.233	0.564	0.166	0.001	0.010
RC3H2	1013.897	-0.989	0.225	0.000	0.000	1013.897	-0.092	0.223	0.680	0.827	1013.897	0.897	0.259	0.001	0.008
TOMM7	1615.634	0.142	0.254	0.577	0.695	1615.634	0.498	0.254	0.050	0.337	1615.634	0.356	0.293	0.225	0.453
MTMR12	717.583	-0.723	0.254	0.004	0.018	717.583	-0.246	0.253	0.332	0.592	717.583	0.478	0.294	0.104	0.286
POU3F3	712.341	0.431	0.321	0.179	0.302	712.341	0.459	0.321	0.153	0.445	712.341	0.028	0.371	0.940	0.973
NECAB2	10.788	-0.575	0.532	0.280	0.417	10.788	0.236	0.482	0.624	0.793	10.788	0.811	0.599	0.176	0.393
GNL3L	678.196	-0.191	0.280	0.495	0.627	678.196	-0.468	0.280	0.094	0.388	678.196	-0.278	0.323	0.390	0.619
MIR4453HG	30.720	-1.140	0.479	0.017	0.052	30.720	-1.056	0.473	0.026	0.316	30.720	0.084	0.561	0.881	0.943
WDR5B	143.113	-0.642	0.276	0.020	0.058	143.113	-0.435	0.274	0.112	0.405	143.113	0.206	0.320	0.519	0.722
DDX49	782.971	0.028	0.195	0.886	0.926	782.971	-0.001	0.195	0.997	0.999	782.971	-0.029	0.226	0.899	0.952
ING3	231.923	-0.852	0.187	0.000	0.000	231.923	-0.073	0.181	0.688	0.831	231.923	0.779	0.215	0.000	0.005
SGTB	780.533	-0.130	0.143	0.364	0.505	780.533	-0.200	0.143	0.163	0.454	780.533	-0.070	0.166	0.675	0.828
SPATA6	99.292	-0.329	0.338	0.330	0.470	99.292	0.511	0.331	0.122	0.413	99.292	0.841	0.387	0.030	0.132
EPB41L4B	668.181	-0.204	0.135	0.132	0.240	668.181	0.059	0.134	0.663	0.817	668.181	0.263	0.156	0.093	0.267
UGT1A7	25.959	4.174	0.706	0.000	0.000	25.959	-0.631	0.883	0.475	0.696	25.959	-4.805	0.917	0.000	0.000
UGT1A6	10.671	0.933	0.843	0.268	0.405	10.671	0.474	0.849	0.576	0.762	10.671	-0.459	0.971	0.636	0.803
SCAND2P	323.531	-0.536	0.224	0.017	0.050	323.531	-0.308	0.222	0.165	0.455	323.531	0.228	0.259	0.378	0.609
EGLN1	3709.693	-0.048	0.121	0.692	0.787	3709.693	-0.021	0.120	0.861	0.929	3709.693	0.027	0.139	0.848	0.926
GNB1L	193.363	0.441	0.257	0.086	0.174	193.363	-0.213	0.259	0.411	0.650	193.363	-0.654	0.298	0.028	0.126
LZTFL1	278.342	-0.362	0.212	0.088	0.178	278.342	0.172	0.209	0.410	0.650	278.342	0.534	0.244	0.029	0.128
MXRA8	20.730	1.202	0.408	0.003	0.014	20.730	0.346	0.424	0.415	0.654	20.730	-0.857	0.473	0.070	0.225
ATRX	1676.639	0.043	0.241	0.857	0.907	1676.639	0.226	0.241	0.347	0.603	1676.639	0.183	0.278	0.511	0.716
POU5F1	286.832	2.947	0.579	0.000	0.000	286.832	1.245	0.580	0.032	0.322	286.832	-1.702	0.667	0.011	0.069
NDFIP2	256.302	-0.161	0.171	0.346	0.486	256.302	-0.330	0.171	0.054	0.342	256.302	-0.168	0.199	0.398	0.625

DDX56	861.439	-0.297	0.286	0.298	0.437	861.439	-0.484	0.286	0.090	0.384	861.439	-0.187	0.330	0.570	0.759
INO80	857.073	-1.122	0.217	0.000	0.000	857.073	-0.404	0.216	0.061	0.350	857.073	0.718	0.251	0.004	0.037
CCNJ	197.700	-1.093	0.424	0.010	0.034	197.700	-1.125	0.423	0.008	0.297	197.700	-0.032	0.492	0.947	0.975
FBXL19	905.452	0.248	0.129	0.055	0.124	905.452	0.170	0.129	0.187	0.473	905.452	-0.078	0.149	0.602	0.780
VSIG10	296.675	0.043	0.156	0.783	0.856	296.675	0.100	0.155	0.520	0.724	296.675	0.057	0.180	0.753	0.874
ARL15	97.032	0.502	0.249	0.043	0.104	97.032	-0.048	0.252	0.849	0.922	97.032	-0.550	0.289	0.057	0.199
PAF1	842.828	0.044	0.202	0.827	0.886	842.828	-0.152	0.202	0.452	0.680	842.828	-0.197	0.234	0.400	0.627
PARP14	1227.963	0.691	0.231	0.003	0.013	1227.963	0.271	0.231	0.241	0.517	1227.963	-0.420	0.267	0.116	0.305
MINDY2	579.451	-0.223	0.241	0.356	0.496	579.451	0.103	0.240	0.668	0.819	579.451	0.326	0.279	0.242	0.472
POU6F1	149.219	0.493	0.299	0.099	0.194	149.219	0.472	0.298	0.113	0.405	149.219	-0.020	0.344	0.953	0.978
PPA1	6567.874	-0.025	0.120	0.835	0.891	6567.874	0.105	0.120	0.380	0.626	6567.874	0.130	0.139	0.347	0.581
PPARA	313.589	0.248	0.456	0.587	0.703	313.589	0.468	0.456	0.304	0.570	313.589	0.220	0.526	0.675	0.828
PCDHB17P	8.049	0.703	0.767	0.359	0.499	8.049	1.212	0.744	0.103	0.396	8.049	0.509	0.856	0.553	0.746
TBC1D13	410.030	0.075	0.170	0.661	0.764	410.030	0.040	0.170	0.813	0.902	410.030	-0.034	0.197	0.861	0.933
WDR74	1401.170	-0.472	0.298	0.113	0.214	1401.170	-0.420	0.298	0.159	0.451	1401.170	0.052	0.344	0.879	0.942
TMEM106B	1084.262	-0.202	0.190	0.289	0.427	1084.262	0.066	0.190	0.728	0.855	1084.262	0.268	0.220	0.223	0.451
RSBN1	451.539	0.252	0.290	0.385	0.524	451.539	0.267	0.290	0.357	0.611	451.539	0.015	0.335	0.965	0.984
PPARD	893.994	-0.068	0.254	0.788	0.860	893.994	-0.593	0.254	0.020	0.308	893.994	-0.524	0.294	0.075	0.235
CRLS1	714.158	0.015	0.111	0.893	0.931	714.158	0.012	0.110	0.911	0.956	714.158	-0.003	0.128	0.984	0.991
GTPBP2	1941.977	-0.721	0.193	0.000	0.002	1941.977	-0.140	0.193	0.466	0.690	1941.977	0.581	0.223	0.009	0.062
CROT	192.461	0.021	0.300	0.945	0.966	192.461	-0.040	0.299	0.893	0.946	192.461	-0.061	0.346	0.860	0.933
PPARG	483.279	0.146	0.185	0.432	0.568	483.279	0.078	0.185	0.675	0.825	483.279	-0.068	0.214	0.750	0.872
ZNHIT6	606.631	-1.136	0.339	0.001	0.005	606.631	-0.804	0.338	0.017	0.306	606.631	0.331	0.392	0.398	0.626
P4HTM	566.811	0.795	0.304	0.009	0.031	566.811	0.601	0.304	0.048	0.337	566.811	-0.194	0.351	0.581	0.764
MANSC1	81.370	1.125	0.196	0.000	0.000	81.370	0.090	0.208	0.667	0.819	81.370	-1.035	0.231	0.000	0.000
MED1	1259.417	-1.049	0.253	0.000	0.000	1259.417	-0.459	0.252	0.069	0.357	1259.417	0.590	0.292	0.044	0.168
RRN3	1578.174	-0.706	0.217	0.001	0.006	1578.174	-0.635	0.217	0.003	0.297	1578.174	0.072	0.251	0.775	0.886
PDP1	272.866	-0.391	0.350	0.264	0.400	272.866	-0.898	0.351	0.011	0.299	272.866	-0.507	0.406	0.212	0.437
GPN2	473.207	0.318	0.131	0.015	0.047	473.207	0.066	0.131	0.618	0.790	473.207	-0.253	0.152	0.096	0.273
MARCHF5	985.640	-0.298	0.136	0.028	0.075	985.640	-0.221	0.135	0.104	0.396	985.640	0.078	0.157	0.621	0.792
PPAT	872.503	-0.881	0.174	0.000	0.000	872.503	-0.191	0.172	0.267	0.538	872.503	0.689	0.201	0.001	0.009
SLC6A20	19.114	0.451	0.409	0.270	0.406	19.114	0.218	0.411	0.596	0.775	19.114	-0.233	0.472	0.621	0.792
BTN2A3P	44.105	0.551	0.451	0.222	0.353	44.105	0.330	0.452	0.466	0.689	44.105	-0.221	0.520	0.671	0.825
OTUD4	1429.917	-0.721	0.226	0.001	0.008	1429.917	-0.389	0.226	0.084	0.376	1429.917	0.331	0.261	0.205	0.429
TMED9	3100.886	0.406	0.230	0.078	0.162	3100.886	-0.319	0.230	0.166	0.456	3100.886	-0.724	0.266	0.006	0.049
SLC35F2	653.124	-0.528	0.274	0.054	0.123	653.124	-0.457	0.274	0.095	0.389	653.124	0.071	0.317	0.823	0.913
MPHOSPH8	643.252	0.806	0.196	0.000	0.000	643.252	0.081	0.197	0.680	0.827	643.252	-0.725	0.226	0.001	0.016
LEPROT	1068.621	0.372	0.217	0.087	0.176	1068.621	0.134	0.217	0.537	0.735	1068.621	-0.238	0.250	0.342	0.576
LY6K	901.815	-0.069	0.288	0.810	0.876	901.815	-0.375	0.288	0.193	0.478	901.815	-0.306	0.333	0.358	0.591
EPDR1	28.485	0.224	0.309	0.470	0.604	28.485	-0.143	0.315	0.650	0.809	28.485	-0.367	0.364	0.314	0.549
PPEF1	16.515	-1.076	0.606	0.076	0.159	16.515	0.545	0.548	0.320	0.581	16.515	1.621	0.680	0.017	0.093
FBLIM1	675.423	0.223	0.113	0.049	0.113	675.423	0.079	0.113	0.487	0.704	675.423	-0.145	0.131	0.270	0.505
FNDC8	47.099	-0.093	0.544	0.865	0.912	47.099	0.530	0.537	0.324	0.584	47.099	0.622	0.625	0.319	0.554
IL17RD	16.370	0.266	0.380	0.484	0.617	16.370	0.428	0.366	0.242	0.518	16.370	0.162	0.430	0.705	0.847
KLHDC4	328.720	-0.177	0.202	0.381	0.521	328.720	-0.049	0.201	0.808	0.899	328.720	0.128	0.233	0.583	0.765
CTSA	1490.880	1.399	0.121	0.000	0.000	1490.880	0.024	0.122	0.848	0.921	1490.880	-1.375	0.140	0.000	0.000
PCSK4	19.765	1.146	0.758	0.131	0.239	19.765	1.204	0.756	0.111	0.405	19.765	0.057	0.866	0.947	0.975
ZRANB1	716.798	-0.609	0.174	0.000	0.003	716.798	-0.300	0.173	0.082	0.373	716.798	0.308	0.201	0.125	0.319
TRIM44	2784.705	-0.362	0.151	0.017	0.050	2784.705	-0.199	0.151	0.187	0.474	2784.705	0.163	0.174	0.350	0.583
PPP1R12C	969.508	0.297	0.160	0.064	0.140	969.508	-0.015	0.160	0.927	0.963	969.508	-0.311	0.185	0.093	0.267
CFAP46	9.996	-0.827	0.497	0.096	0.190	9.996	-0.700	0.477	0.143	0.434	9.996	0.127	0.588	0.829	0.916
RNF111	528.155	-0.816	0.268	0.002	0.011	528.155	-0.215	0.266	0.419	0.656	528.155	0.601	0.309	0.052	0.186
PPIA	20952.998	0.757	0.188	0.000	0.001	20952.998	0.020	0.188	0.916	0.958	20952.998	-0.737	0.217	0.001	0.010
NSMCE4A	377.126	-0.243	0.349	0.486	0.619	377.126	-0.532	0.349	0.128	0.420	377.126	-0.289	0.404	0.474	0.688
ALKBH4	180.107	0.512	0.224	0.022	0.062	180.107	0.210	0.225	0.350	0.605	180.107	-0.302	0.259	0.243	0.474
BORCS6	174.837	0.711	0.293	0.015	0.047	174.837	0.539	0.293	0.066	0.354	174.837	-0.173	0.337	0.609	0.784
DNAJB12	571.501	-0.076	0.202	0.706	0.798	571.501	-0.261	0.202	0.198	0.481	571.501	-0.185	0.234	0.431	0.653
PPIB	5765.500	0.719	0.243	0.003	0.014	5765.500	-0.047	0.243	0.846	0.920	5765.500	-0.767	0.281	0.006	0.048
TET2	233.664	-0.706	0.323	0.029	0.076	233.664	-0.374	0.321	0.243	0.519	233.664	0.332	0.373	0.374	0.606
KCTD9	1226.479	-0.597	0.187	0.001	0.008	1226.479	-0.171	0.187	0.359	0.611	1226.479	0.426	0.216	0.049	0.181
TRPM4	377.418	1.303	0.265	0.000	0.000	377.418	0.479	0.266	0.072	0.361	377.418	-0.824	0.306	0.007	0.052
BNC2	35.984	0.854	0.333	0.010	0.035	35.984	0.463	0.336	0.168	0.457	35.984	-0.391	0.382	0.306	0.542
MED18	254.485	-0.046	0.151	0.761	0.842	254.485	-0.115	0.150	0.441	0.673	254.485	-0.070	0.175	0.690	0.837
MBTD1	263.082	-0.485	0.317	0.126	0.232	263.082	0.467	0.314	0.137	0.428	263.082	0.952	0.365	0.009	0.061
PPIC	209.369	1.280	0.211	0.000	0.000	209.369	-0.324	0.219	0.138	0.429	209.369	-1.604	0.248	0.000	0.000
KLHL24	785.308	0.410	0.313	0.190	0.315	785.308	0.441	0.313	0.158	0.451	785.308	0.031	0.361	0.931	0.969
HAUS6	2497.260	0.148	0.197	0.453	0.588	2497.260	0.096	0.197	0.627	0.795	2497.260	-0.052	0.227	0.819	0.910

TRIT1	568.095	0.082	0.144	0.569	0.688	568.095	-0.073	0.144	0.613	0.787	568.095	-0.154	0.166	0.353	0.586
CNNM2	689.783	0.429	0.194	0.027	0.073	689.783	0.203	0.194	0.296	0.564	689.783	-0.226	0.224	0.314	0.550
AHI1	303.266	-0.563	0.146	0.000	0.001	303.266	-0.164	0.143	0.251	0.525	303.266	0.399	0.169	0.018	0.096
ZNF586	155.463	-0.755	0.171	0.000	0.000	155.463	-0.181	0.163	0.267	0.538	155.463	0.574	0.197	0.004	0.032
DYM	982.559	-0.403	0.212	0.058	0.128	982.559	0.245	0.211	0.246	0.521	982.559	0.648	0.244	0.008	0.057
SAMD9	366.477	0.345	0.288	0.231	0.363	366.477	0.019	0.288	0.947	0.974	366.477	-0.326	0.333	0.328	0.562
PPID	760.776	-0.272	0.273	0.319	0.459	760.776	-0.700	0.274	0.011	0.299	760.776	-0.428	0.316	0.176	0.394
ZNF562	375.769	-0.401	0.225	0.075	0.157	375.769	-0.508	0.225	0.024	0.316	375.769	-0.107	0.261	0.682	0.832
AFTPH	533.214	-0.212	0.141	0.133	0.241	533.214	-0.181	0.140	0.198	0.482	533.214	0.032	0.163	0.846	0.926
KLHL28	259.320	-0.224	0.274	0.414	0.552	259.320	0.208	0.272	0.444	0.675	259.320	0.432	0.316	0.171	0.388
QPCTL	566.225	0.992	0.188	0.000	0.000	566.225	0.377	0.189	0.046	0.335	566.225	-0.615	0.217	0.005	0.039
GATAD2A	1443.566	-0.464	0.290	0.110	0.210	1443.566	-0.559	0.290	0.054	0.342	1443.566	-0.095	0.336	0.778	0.888
ZNF280D	425.820	-0.266	0.260	0.307	0.446	425.820	0.474	0.258	0.067	0.355	425.820	0.740	0.300	0.014	0.080
ZCCHC10	223.896	-0.132	0.193	0.495	0.627	223.896	-0.294	0.193	0.128	0.420	223.896	-0.162	0.224	0.470	0.684
NDE1	3483.280	-0.743	0.119	0.000	0.000	3483.280	0.112	0.118	0.344	0.600	3483.280	0.855	0.137	0.000	0.000
ERCC6L	135.680	-0.586	0.239	0.014	0.044	135.680	-0.628	0.237	0.008	0.297	135.680	-0.042	0.278	0.881	0.943
TRPM7	827.325	-0.403	0.248	0.104	0.201	827.325	-0.337	0.247	0.172	0.462	827.325	0.066	0.286	0.819	0.910
SWT1	59.219	-0.004	0.292	0.990	0.994	59.219	0.441	0.283	0.119	0.411	59.219	0.445	0.333	0.181	0.399
CDHR2	8.327	2.137	0.748	0.004	0.018	8.327	-0.090	0.837	0.914	0.957	8.327	-2.227	0.902	0.014	0.080
GIN1	72.975	-1.025	0.265	0.000	0.001	72.975	-0.114	0.247	0.643	0.805	72.975	0.911	0.302	0.003	0.026
BCAS3	609.928	-0.437	0.306	0.154	0.269	609.928	0.468	0.305	0.124	0.416	609.928	0.905	0.353	0.010	0.067
NUP62CL	39.791	-0.236	0.345	0.494	0.626	39.791	0.127	0.336	0.706	0.842	39.791	0.363	0.396	0.360	0.593
BEST2	49.964	-2.012	1.208	0.096	0.190	49.964	0.630	1.187	0.596	0.775	49.964	2.641	1.388	0.057	0.199
VPS13C	666.674	-0.049	0.280	0.861	0.909	666.674	0.247	0.279	0.376	0.624	666.674	0.296	0.323	0.359	0.591
GDAP2	206.006	-0.198	0.280	0.479	0.612	206.006	-0.510	0.281	0.069	0.357	206.006	-0.312	0.325	0.338	0.572
BSPRY	13.387	0.466	0.440	0.289	0.427	13.387	-0.009	0.451	0.984	0.991	13.387	-0.475	0.515	0.357	0.590
WBP1L	555.034	0.026	0.164	0.874	0.919	555.034	0.120	0.163	0.462	0.687	555.034	0.094	0.189	0.619	0.791
LRRC49	239.718	-0.750	0.248	0.003	0.012	239.718	-0.154	0.243	0.527	0.729	239.718	0.596	0.286	0.037	0.151
APTX	649.141	-0.303	0.204	0.139	0.249	649.141	-0.416	0.204	0.042	0.330	649.141	-0.113	0.237	0.633	0.800
BIVM	225.431	-0.019	0.196	0.923	0.951	225.431	0.265	0.193	0.170	0.460	225.431	0.284	0.225	0.207	0.432
MFSD6	191.908	0.188	0.213	0.379	0.519	191.908	0.140	0.213	0.509	0.718	191.908	-0.047	0.246	0.848	0.926
SYTL2	36.928	0.643	0.327	0.049	0.113	36.928	0.408	0.328	0.213	0.493	36.928	-0.235	0.375	0.530	0.730
ESRP1	30.819	0.362	0.293	0.217	0.347	30.819	0.512	0.285	0.073	0.362	30.819	0.150	0.331	0.651	0.812
DEF8	711.312	0.364	0.222	0.102	0.198	711.312	-0.355	0.223	0.112	0.405	711.312	-0.719	0.258	0.005	0.043
FBXL12	288.966	0.431	0.181	0.017	0.051	288.966	0.020	0.182	0.911	0.956	288.966	-0.411	0.209	0.050	0.182
ANKRD49	104.606	0.416	0.190	0.028	0.075	104.606	-0.111	0.194	0.568	0.755	104.606	-0.527	0.223	0.018	0.095
PAQR5	1640.263	-0.929	0.166	0.000	0.000	1640.263	-0.027	0.164	0.869	0.933	1640.263	0.902	0.191	0.000	0.000
WDR55	699.101	0.295	0.161	0.067	0.144	699.101	-0.131	0.161	0.417	0.655	699.101	-0.425	0.186	0.022	0.108
TENT5C	20.487	2.762	0.579	0.000	0.000	20.487	0.737	0.607	0.225	0.503	20.487	-2.025	0.666	0.002	0.024
GON4L	729.325	-0.403	0.157	0.010	0.035	729.325	-0.263	0.156	0.093	0.388	729.325	0.140	0.182	0.441	0.661
PGPEP1	600.040	1.044	0.469	0.026	0.070	600.040	0.829	0.469	0.077	0.368	600.040	-0.215	0.541	0.691	0.838
ELP6	429.979	-0.264	0.196	0.179	0.302	429.979	-0.190	0.195	0.330	0.590	429.979	0.073	0.227	0.747	0.870
CKMT1A	139.763	-0.050	0.313	0.873	0.918	139.763	0.182	0.311	0.559	0.748	139.763	0.232	0.361	0.520	0.723
SNRK	376.236	-0.980	0.341	0.004	0.017	376.236	-0.781	0.340	0.022	0.314	376.236	0.199	0.394	0.613	0.787
CC2D1A	685.904	0.237	0.166	0.153	0.268	685.904	-0.064	0.166	0.702	0.840	685.904	-0.300	0.192	0.117	0.307
TOR4A	1010.218	-0.008	0.126	0.951	0.970	1010.218	0.112	0.125	0.369	0.619	1010.218	0.120	0.145	0.408	0.635
POLR2J3	172.664	0.051	0.285	0.858	0.907	172.664	-0.155	0.285	0.586	0.768	172.664	-0.206	0.330	0.532	0.731
DNAJC25	259.957	0.133	0.242	0.582	0.699	259.957	-0.280	0.244	0.252	0.525	259.957	-0.413	0.282	0.143	0.348
GPATCH4	788.862	-0.987	0.453	0.029	0.077	788.862	-1.312	0.453	0.004	0.297	788.862	-0.325	0.523	0.535	0.733
TMEM214	828.488	0.047	0.244	0.849	0.901	828.488	-0.398	0.245	0.104	0.397	828.488	-0.444	0.282	0.116	0.305
TMEM104	161.300	0.224	0.393	0.568	0.688	161.300	-1.006	0.399	0.012	0.305	161.300	-1.230	0.459	0.007	0.054
EPS8L1	253.630	0.836	0.412	0.043	0.102	253.630	0.359	0.413	0.386	0.631	253.630	-0.477	0.476	0.316	0.552
QRICH1	916.718	-0.176	0.125	0.158	0.275	916.718	0.026	0.124	0.833	0.914	916.718	0.202	0.144	0.160	0.373
PIGG	421.214	0.063	0.147	0.668	0.769	421.214	-0.284	0.148	0.055	0.342	421.214	-0.347	0.171	0.043	0.165
PALMD	67.411	1.561	0.440	0.000	0.003	67.411	0.084	0.448	0.851	0.924	67.411	-1.477	0.511	0.004	0.034
FNBP1L	154.706	-0.213	0.532	0.689	0.785	154.706	0.622	0.529	0.240	0.516	154.706	0.835	0.613	0.173	0.390
CNTLN	217.330	-0.487	0.238	0.041	0.099	217.330	-0.012	0.235	0.960	0.979	217.330	0.475	0.275	0.084	0.251
DCAF16	486.753	-0.250	0.172	0.146	0.259	486.753	0.051	0.171	0.763	0.874	486.753	0.302	0.199	0.129	0.324
ZCCHC2	624.015	0.025	0.251	0.922	0.950	624.015	0.241	0.251	0.336	0.594	624.015	0.216	0.290	0.456	0.674
DPP8	556.464	-0.227	0.164	0.166	0.286	556.464	-0.305	0.164	0.063	0.350	556.464	-0.077	0.190	0.685	0.834
ST7L	272.825	-0.338	0.139	0.015	0.046	272.825	-0.298	0.137	0.029	0.322	272.825	0.040	0.161	0.806	0.903
BCOR	1085.448	-0.352	0.233	0.132	0.240	1085.448	0.143	0.233	0.537	0.735	1085.448	0.495	0.269	0.066	0.217
TEX10	921.583	-0.483	0.150	0.001	0.007	921.583	-0.197	0.149	0.186	0.473	921.583	0.286	0.173	0.099	0.277
ANKHD1	143.163	-0.821	0.321	0.010	0.035	143.163	-0.239	0.316	0.449	0.678	143.163	0.582	0.370	0.116	0.305
CWC25	603.650	-0.078	0.243	0.749	0.833	603.650	-0.357	0.243	0.142	0.433	603.650	-0.280	0.281	0.320	0.555
RETSAT	3655.153	0.179	0.265	0.498	0.629	3655.153	0.386	0.265	0.144	0.436	3655.153	0.207	0.306	0.498	0.706
TBC1D8B	252.665	-0.132	0.389	0.734	0.821	252.665	0.226	0.388	0.559	0.748	252.665	0.359	0.449	0.424	0.648

UHRF1BP1	892.733	-1.268	0.265	0.000	0.000	892.733	-0.577	0.263	0.028	0.321	892.733	0.691	0.306	0.024	0.114
NSUN2	2809.890	-1.088	0.367	0.003	0.013	2809.890	-1.099	0.367	0.003	0.297	2809.890	-0.011	0.424	0.980	0.990
ALKBH5	1160.173	0.454	0.192	0.018	0.053	1160.173	0.329	0.192	0.086	0.379	1160.173	-0.125	0.222	0.573	0.760
INO80D	184.889	-0.147	0.212	0.488	0.621	184.889	-0.256	0.212	0.227	0.506	184.889	-0.108	0.247	0.660	0.818
NCAPG2	1114.865	-0.830	0.208	0.000	0.001	1114.865	-0.286	0.207	0.168	0.457	1114.865	0.544	0.240	0.024	0.113
MTMR10	656.583	-0.473	0.187	0.011	0.038	656.583	-0.107	0.186	0.566	0.753	656.583	0.366	0.216	0.090	0.263
RNF43	240.329	0.003	0.233	0.990	0.995	240.329	0.374	0.231	0.105	0.398	240.329	0.371	0.268	0.166	0.381
SLC66A1	248.226	0.883	0.194	0.000	0.000	248.226	-0.321	0.201	0.111	0.405	248.226	-1.204	0.229	0.000	0.000
CASZ1	64.847	-1.609	0.639	0.012	0.039	64.847	0.609	0.619	0.325	0.586	64.847	2.218	0.731	0.002	0.025
PXK	1303.746	-1.215	0.184	0.000	0.000	1303.746	-0.206	0.182	0.259	0.531	1303.746	1.009	0.212	0.000	0.000
AUH	187.202	0.010	0.219	0.962	0.977	187.202	0.137	0.217	0.529	0.730	187.202	0.126	0.253	0.618	0.790
CDKAL1	203.152	-0.001	0.177	0.997	0.999	203.152	-0.216	0.177	0.223	0.501	203.152	-0.215	0.205	0.295	0.531
TTC19	597.456	-0.571	0.203	0.005	0.020	597.456	0.184	0.201	0.360	0.612	597.456	0.755	0.234	0.001	0.016
MKS1	214.519	-0.031	0.152	0.839	0.894	214.519	-0.062	0.151	0.679	0.826	214.519	-0.032	0.176	0.857	0.931
NSD3	1642.306	-1.015	0.229	0.000	0.000	1642.306	-0.180	0.229	0.430	0.665	1642.306	0.835	0.265	0.002	0.019
TASOR2	1147.883	-1.446	0.333	0.000	0.000	1147.883	-0.823	0.332	0.013	0.305	1147.883	0.623	0.385	0.105	0.289
SPDL1	942.091	-1.110	0.313	0.000	0.003	942.091	-0.621	0.312	0.047	0.335	942.091	0.489	0.362	0.176	0.394
SEMA4C	570.594	0.025	0.133	0.850	0.902	570.594	0.272	0.132	0.040	0.327	570.594	0.246	0.153	0.108	0.294
FOCAD	30.487	-0.105	0.296	0.722	0.811	30.487	-0.302	0.296	0.309	0.573	30.487	-0.196	0.349	0.574	0.761
YTHDF1	804.436	-0.053	0.168	0.753	0.835	804.436	-0.412	0.168	0.014	0.306	804.436	-0.360	0.194	0.064	0.213
TMEM260	239.407	-0.151	0.134	0.261	0.397	239.407	0.118	0.131	0.365	0.617	239.407	0.269	0.154	0.081	0.247
CMTM6	809.727	0.369	0.175	0.035	0.088	809.727	-0.311	0.176	0.077	0.368	809.727	-0.679	0.202	0.001	0.011
DNAAF5	1370.673	-0.759	0.199	0.000	0.001	1370.673	-0.197	0.198	0.318	0.580	1370.673	0.561	0.229	0.014	0.083
DUS2	374.935	-0.334	0.201	0.097	0.192	374.935	-0.106	0.200	0.596	0.775	374.935	0.228	0.232	0.327	0.562
RASIP1	275.713	0.055	0.212	0.796	0.865	275.713	-0.112	0.212	0.596	0.775	275.713	-0.167	0.245	0.495	0.703
LIME1	65.827	1.666	0.421	0.000	0.001	65.827	0.284	0.431	0.510	0.718	65.827	-1.382	0.488	0.005	0.039
ZSCAN32	208.404	-0.660	0.194	0.001	0.004	208.404	-0.260	0.190	0.171	0.460	208.404	0.399	0.224	0.075	0.236
UBE2R2	2711.069	0.126	0.219	0.565	0.686	2711.069	0.348	0.219	0.113	0.405	2711.069	0.222	0.253	0.381	0.611
CHCHD3	1309.052	-0.509	0.206	0.013	0.042	1309.052	-0.378	0.205	0.065	0.354	1309.052	0.131	0.238	0.582	0.765
IMPAD1	2539.021	-0.128	0.192	0.505	0.635	2539.021	-0.260	0.192	0.176	0.465	2539.021	-0.132	0.222	0.553	0.746
TMEM161A	947.536	0.463	0.183	0.011	0.037	947.536	-0.114	0.183	0.536	0.734	947.536	-0.576	0.211	0.006	0.049
PPL	268.419	-0.870	0.945	0.357	0.497	268.419	0.965	0.943	0.306	0.572	268.419	1.835	1.090	0.092	0.267
HAUS4	764.110	-0.209	0.218	0.338	0.478	764.110	0.430	0.217	0.048	0.337	764.110	0.639	0.252	0.011	0.070
TRMT10C	449.770	-0.317	0.173	0.067	0.145	449.770	-0.338	0.173	0.050	0.337	449.770	-0.021	0.201	0.916	0.961
EXD3	266.870	0.890	0.366	0.015	0.046	266.870	0.662	0.366	0.070	0.359	266.870	-0.228	0.421	0.589	0.770
RHBDL2	65.601	1.737	0.490	0.000	0.003	65.601	1.043	0.493	0.034	0.322	65.601	-0.694	0.562	0.217	0.443
KANSL2	413.125	-0.921	0.186	0.000	0.000	413.125	-0.304	0.183	0.096	0.391	413.125	0.618	0.215	0.004	0.035
DUSP23	840.870	1.290	0.273	0.000	0.000	840.870	0.056	0.275	0.837	0.916	840.870	-1.234	0.316	0.000	0.002
ADPRHL2	663.155	0.563	0.182	0.002	0.010	663.155	-0.129	0.183	0.482	0.701	663.155	-0.692	0.211	0.001	0.014
SOHLH2	13.153	0.691	0.602	0.251	0.385	13.153	1.164	0.587	0.047	0.336	13.153	0.473	0.676	0.485	0.696
SARS2	330.013	-0.127	0.168	0.450	0.586	330.013	0.121	0.166	0.468	0.691	330.013	0.247	0.193	0.201	0.424
COMMD4	522.994	0.511	0.308	0.097	0.192	522.994	-0.266	0.309	0.390	0.635	522.994	-0.777	0.357	0.029	0.130
PPM1A	720.953	-0.702	0.185	0.000	0.001	720.953	-0.251	0.183	0.171	0.460	720.953	0.451	0.213	0.035	0.145
OCIAD1	1474.177	0.284	0.150	0.058	0.130	1474.177	0.102	0.150	0.495	0.709	1474.177	-0.181	0.173	0.295	0.530
RNF125	56.316	0.117	0.334	0.727	0.814	56.316	-0.411	0.338	0.224	0.503	56.316	-0.528	0.391	0.177	0.394
ABITRAM	399.522	0.038	0.181	0.835	0.891	399.522	-0.234	0.181	0.196	0.480	399.522	-0.272	0.210	0.195	0.417
DNAJC28	14.416	1.108	0.597	0.064	0.139	14.416	0.674	0.604	0.264	0.536	14.416	-0.433	0.682	0.525	0.726
LINC01521	30.127	-0.174	0.360	0.629	0.738	30.127	0.214	0.347	0.537	0.735	30.127	0.389	0.412	0.346	0.580
SLC41A3	2316.089	-0.325	0.352	0.357	0.497	2316.089	0.546	0.352	0.121	0.412	2316.089	0.871	0.407	0.032	0.138
LPCAT2	171.386	0.561	0.200	0.005	0.020	171.386	-0.560	0.207	0.007	0.297	171.386	-1.121	0.236	0.000	0.000
MRPL16	532.016	0.125	0.202	0.536	0.661	532.016	-0.134	0.202	0.509	0.718	532.016	-0.259	0.234	0.268	0.503
SDHAF2	274.451	-0.267	0.216	0.216	0.346	274.451	-0.483	0.216	0.025	0.316	274.451	-0.216	0.251	0.389	0.617
PPM1B	126.311	-0.252	0.186	0.175	0.296	126.311	-0.037	0.181	0.838	0.916	126.311	0.215	0.214	0.316	0.552
COMMD8	296.712	-0.388	0.215	0.071	0.151	296.712	-0.301	0.214	0.159	0.451	296.712	0.087	0.249	0.727	0.859
TRNAU1AP	297.882	0.418	0.177	0.018	0.053	297.882	-0.121	0.178	0.497	0.711	297.882	-0.539	0.205	0.009	0.059
ODR4	424.136	0.076	0.170	0.654	0.758	424.136	-0.171	0.171	0.317	0.579	424.136	-0.247	0.198	0.211	0.436
FAM120C	116.591	0.433	0.216	0.045	0.107	116.591	0.052	0.218	0.810	0.900	116.591	-0.380	0.251	0.129	0.326
C1orf109	362.363	-1.422	0.250	0.000	0.000	362.363	-0.445	0.245	0.070	0.358	362.363	0.977	0.288	0.001	0.010
PARP16	198.800	0.143	0.369	0.698	0.792	198.800	0.712	0.367	0.052	0.341	198.800	0.569	0.425	0.181	0.399
TXNL4B	407.803	-0.729	0.232	0.002	0.009	407.803	-0.370	0.230	0.108	0.400	407.803	0.359	0.268	0.180	0.398
TMEM160	283.860	1.065	0.344	0.002	0.010	283.860	-0.357	0.347	0.304	0.570	283.860	-1.422	0.399	0.000	0.006
PPM1G	2920.963	-0.235	0.275	0.392	0.530	2920.963	-0.531	0.275	0.053	0.342	2920.963	-0.295	0.318	0.352	0.586
GEMIN8	188.886	0.207	0.251	0.410	0.549	188.886	0.506	0.249	0.042	0.330	188.886	0.299	0.289	0.301	0.536
SSH3	314.458	0.771	0.272	0.005	0.018	314.458	0.229	0.273	0.401	0.644	314.458	-0.542	0.314	0.084	0.252
TIPIN	175.922	-0.818	0.388	0.035	0.089	175.922	-0.960	0.388	0.013	0.305	175.922	-0.141	0.451	0.754	0.874
UCKL1	511.483	-0.002	0.109	0.988	0.993	511.483	0.211	0.107	0.048	0.337	511.483	0.212	0.125	0.089	0.261
C1orf56	269.396	0.616	0.190	0.001	0.007	269.396	0.102	0.192	0.596	0.776	269.396	-0.515	0.221	0.020	0.100

PIGX	455.508	-0.063	0.211	0.766	0.845	455.508	0.011	0.211	0.957	0.978	455.508	0.074	0.244	0.761	0.878
TMEM70	582.654	-0.347	0.294	0.238	0.372	582.654	-0.641	0.294	0.029	0.322	582.654	-0.294	0.340	0.387	0.616
HPF1	342.636	-0.141	0.204	0.490	0.623	342.636	-0.475	0.205	0.020	0.310	342.636	-0.334	0.237	0.158	0.371
TTC12	68.048	-0.766	0.276	0.006	0.021	68.048	-0.029	0.263	0.912	0.956	68.048	0.737	0.317	0.020	0.101
BANP	364.208	-0.390	0.216	0.071	0.151	364.208	0.095	0.213	0.655	0.812	364.208	0.485	0.249	0.051	0.186
TMEM132A	4625.195	1.053	0.128	0.000	0.000	4625.195	0.338	0.128	0.009	0.297	4625.195	-0.716	0.148	0.000	0.000
INTS11	1687.634	-0.028	0.163	0.863	0.911	1687.634	-0.008	0.163	0.960	0.979	1687.634	0.020	0.188	0.916	0.961
THG1L	750.335	0.068	0.252	0.788	0.859	750.335	0.346	0.251	0.169	0.458	750.335	0.278	0.291	0.338	0.573
C20orf27	835.811	-0.067	0.219	0.758	0.840	835.811	-0.223	0.219	0.308	0.573	835.811	-0.155	0.253	0.538	0.736
SLC25A38	1220.462	-0.515	0.122	0.000	0.000	1220.462	0.042	0.121	0.726	0.854	1220.462	0.557	0.141	0.000	0.002
SLC35F6	847.370	-0.086	0.344	0.803	0.870	847.370	-0.596	0.345	0.084	0.376	847.370	-0.510	0.398	0.200	0.424
PPOX	239.643	0.179	0.162	0.269	0.405	239.643	0.357	0.160	0.025	0.316	239.643	0.178	0.186	0.336	0.571
C2orf42	95.710	-0.677	0.196	0.001	0.004	95.710	-0.432	0.189	0.022	0.315	95.710	0.245	0.229	0.284	0.519
NMRK1	197.545	0.622	0.472	0.187	0.311	197.545	0.601	0.471	0.202	0.486	197.545	-0.022	0.544	0.968	0.985
CLN6	524.063	-0.403	0.339	0.234	0.367	524.063	-0.841	0.339	0.013	0.305	524.063	-0.438	0.392	0.264	0.498
PINX1	123.843	-0.518	0.478	0.278	0.416	123.843	-0.948	0.480	0.048	0.337	123.843	-0.429	0.556	0.440	0.660
HCFC1R1	651.311	0.824	0.331	0.013	0.041	651.311	0.612	0.331	0.064	0.353	651.311	-0.212	0.382	0.578	0.763
ULK4	41.892	-0.756	0.312	0.015	0.047	41.892	0.179	0.288	0.535	0.734	41.892	0.934	0.353	0.008	0.057
CZIB	477.495	0.529	0.211	0.012	0.039	477.495	0.239	0.211	0.257	0.530	477.495	-0.290	0.244	0.234	0.462
ZNF770	1333.852	-0.661	0.126	0.000	0.000	1333.852	-0.214	0.125	0.086	0.378	1333.852	0.446	0.145	0.002	0.023
PPP1CA	2875.602	0.221	0.165	0.180	0.303	2875.602	-0.081	0.165	0.624	0.793	2875.602	-0.302	0.191	0.113	0.301
C1orf159	204.259	0.388	0.309	0.209	0.337	204.259	-0.510	0.312	0.101	0.394	204.259	-0.898	0.359	0.012	0.075
ZSCAN2	105.474	-0.089	0.267	0.737	0.823	105.474	0.217	0.262	0.407	0.649	105.474	0.307	0.307	0.317	0.553
GID8	849.275	-0.086	0.139	0.535	0.660	849.275	0.040	0.138	0.773	0.879	849.275	0.126	0.160	0.432	0.654
OXSM	365.081	-0.262	0.158	0.099	0.194	365.081	0.080	0.156	0.609	0.784	365.081	0.341	0.182	0.061	0.208
MARC2	311.257	0.309	0.154	0.045	0.106	311.257	0.132	0.154	0.392	0.636	311.257	-0.177	0.178	0.320	0.555
AURKAIP1	1595.698	0.237	0.315	0.452	0.587	1595.698	-0.507	0.315	0.107	0.400	1595.698	-0.744	0.364	0.041	0.161
AUP1	1811.278	0.163	0.194	0.402	0.540	1811.278	-0.151	0.194	0.436	0.669	1811.278	-0.314	0.224	0.161	0.375
PPP1CB	3478.495	-0.288	0.097	0.003	0.013	3478.495	-0.113	0.097	0.241	0.517	3478.495	0.175	0.112	0.118	0.307
TUG1	1481.168	-0.374	0.179	0.037	0.092	1481.168	-0.247	0.179	0.168	0.457	1481.168	0.127	0.207	0.540	0.737
TMCO3	464.788	0.182	0.348	0.601	0.715	464.788	0.308	0.348	0.375	0.623	464.788	0.126	0.402	0.753	0.874
PAK1IP1	531.800	-0.888	0.426	0.037	0.092	531.800	-1.247	0.427	0.003	0.297	531.800	-0.359	0.493	0.466	0.682
LAMTOR1	775.635	0.165	0.192	0.392	0.531	775.635	-0.203	0.193	0.291	0.559	775.635	-0.368	0.222	0.098	0.277
RMND1	735.493	-0.278	0.161	0.084	0.172	735.493	0.187	0.159	0.242	0.518	735.493	0.465	0.185	0.012	0.075
TRMT61B	269.454	-0.535	0.174	0.002	0.010	269.454	-0.266	0.171	0.120	0.412	269.454	0.269	0.201	0.180	0.398
FAM118A	206.425	-0.817	0.300	0.006	0.024	206.425	-0.632	0.298	0.034	0.322	206.425	0.185	0.348	0.594	0.774
HERC6	162.833	0.294	0.248	0.237	0.370	162.833	0.099	0.248	0.690	0.832	162.833	-0.195	0.287	0.497	0.705
FAM174C	743.954	0.216	0.231	0.349	0.489	743.954	0.110	0.231	0.635	0.801	743.954	-0.107	0.267	0.688	0.836
PPP1CC	5585.400	-0.620	0.179	0.001	0.004	5585.400	0.181	0.179	0.312	0.575	5585.400	0.801	0.207	0.000	0.003
PARPBP	370.067	-1.008	0.307	0.001	0.006	370.067	-0.522	0.305	0.087	0.380	370.067	0.486	0.354	0.170	0.386
PIH1D1	1227.863	0.204	0.163	0.212	0.341	1227.863	0.058	0.163	0.722	0.851	1227.863	-0.146	0.189	0.439	0.660
UBA6-AS1	248.460	-0.567	0.198	0.004	0.017	248.460	-0.100	0.194	0.606	0.782	248.460	0.468	0.228	0.040	0.160
PPP2R3C	292.320	-0.981	0.245	0.000	0.001	292.320	-0.263	0.240	0.274	0.545	292.320	0.718	0.282	0.011	0.069
MCUB	338.670	0.427	0.154	0.006	0.022	338.670	0.108	0.155	0.488	0.705	338.670	-0.320	0.179	0.073	0.232
STX17	575.259	-0.466	0.236	0.048	0.113	575.259	0.140	0.235	0.550	0.743	575.259	0.606	0.272	0.026	0.120
PRPF39	777.382	0.411	0.208	0.048	0.112	777.382	0.095	0.208	0.647	0.808	777.382	-0.316	0.240	0.189	0.410
MARCHF1	6.538	0.048	0.785	0.952	0.970	6.538	0.484	0.759	0.524	0.727	6.538	0.436	0.893	0.625	0.795
C14orf119	509.085	0.100	0.240	0.676	0.775	509.085	-0.277	0.240	0.249	0.523	509.085	-0.377	0.277	0.174	0.391
TTC38	294.474	0.145	0.177	0.414	0.552	294.474	-0.127	0.178	0.476	0.697	294.474	-0.272	0.206	0.186	0.407
PID1	752.364	-1.549	0.322	0.000	0.000	752.364	-0.403	0.319	0.206	0.488	752.364	1.146	0.371	0.002	0.022
PHIP	1404.360	-0.121	0.229	0.596	0.711	1404.360	-0.261	0.229	0.253	0.527	1404.360	-0.140	0.264	0.596	0.775
TMEM255A	25.248	-0.047	0.809	0.953	0.972	25.248	0.956	0.796	0.230	0.507	25.248	1.003	0.926	0.279	0.513
HEATR3	262.968	-0.727	0.330	0.027	0.073	262.968	-0.673	0.329	0.041	0.328	262.968	0.054	0.382	0.888	0.946
C17orf80	438.263	-0.582	0.135	0.000	0.000	438.263	-0.146	0.132	0.269	0.540	438.263	0.436	0.156	0.005	0.042
FBXO34	401.262	-0.144	0.123	0.241	0.374	401.262	-0.163	0.122	0.181	0.469	401.262	-0.019	0.143	0.894	0.950
USP47	1207.911	-0.550	0.148	0.000	0.002	1207.911	-0.078	0.147	0.598	0.776	1207.911	0.473	0.171	0.006	0.044
SLC35A5	730.597	-0.457	0.174	0.009	0.030	730.597	0.017	0.173	0.923	0.961	730.597	0.474	0.201	0.018	0.096
FKBP14	429.282	-0.525	0.269	0.051	0.118	429.282	-0.580	0.269	0.031	0.322	429.282	-0.055	0.312	0.859	0.932
MOCOS	695.555	-1.056	0.281	0.000	0.001	695.555	-0.298	0.280	0.288	0.556	695.555	0.758	0.325	0.020	0.100
NOL8	870.076	-0.374	0.303	0.216	0.346	870.076	-0.758	0.303	0.012	0.305	870.076	-0.384	0.350	0.273	0.508
CCDC40	37.135	0.778	0.339	0.022	0.061	37.135	-0.385	0.359	0.283	0.553	37.135	-1.164	0.405	0.004	0.036
PTCD3	2248.359	-0.547	0.124	0.000	0.000	2248.359	-0.121	0.123	0.325	0.586	2248.359	0.426	0.143	0.003	0.028
CDCA4	1073.537	-0.383	0.249	0.124	0.229	1073.537	-0.351	0.248	0.157	0.449	1073.537	0.031	0.287	0.914	0.960
TRMT12	166.752	-0.473	0.283	0.095	0.189	166.752	-0.773	0.284	0.007	0.297	166.752	-0.301	0.331	0.363	0.595
PPP1R2	931.390	-0.114	0.116	0.324	0.464	931.390	-0.157	0.115	0.174	0.463	931.390	-0.043	0.134	0.750	0.872
PLEKHB2	1566.906	-0.703	0.275	0.011	0.036	1566.906	-0.450	0.275	0.102	0.394	1566.906	0.253	0.318	0.426	0.649
VPS37C	602.995	-0.239	0.224	0.284	0.422	602.995	-0.344	0.223	0.124	0.416	602.995	-0.104	0.259	0.687	0.835

REX1BD	420.740	0.616	0.237	0.009	0.033	420.740	0.090	0.238	0.705	0.841	420.740	-0.526	0.275	0.055	0.195
NRDE2	1382.593	-0.067	0.225	0.765	0.844	1382.593	-0.339	0.225	0.132	0.424	1382.593	-0.272	0.260	0.295	0.531
MRPL20	775.628	-0.301	0.299	0.314	0.454	775.628	-0.532	0.299	0.075	0.364	775.628	-0.231	0.346	0.505	0.712
ATG16L1	257.618	-0.280	0.223	0.210	0.339	257.618	-0.343	0.223	0.124	0.416	257.618	-0.063	0.259	0.808	0.904
ZWILCH	686.969	-1.018	0.413	0.014	0.043	686.969	-1.029	0.413	0.013	0.305	686.969	-0.011	0.478	0.982	0.990
GABPB1-IT1	232.903	0.260	0.205	0.205	0.333	232.903	0.224	0.204	0.273	0.544	232.903	-0.036	0.236	0.880	0.943
CRYBG2	88.303	1.577	0.300	0.000	0.000	88.303	0.278	0.308	0.366	0.617	88.303	-1.299	0.348	0.000	0.004
WIPI1	415.341	1.247	0.171	0.000	0.000	415.341	0.131	0.175	0.454	0.681	415.341	-1.117	0.199	0.000	0.000
ZCWPW1	49.906	-0.013	0.316	0.967	0.980	49.906	0.383	0.306	0.210	0.491	49.906	0.396	0.360	0.271	0.506
CCDC157	105.873	-0.520	0.294	0.077	0.160	105.873	0.016	0.288	0.956	0.978	105.873	0.536	0.338	0.113	0.301
SPATA6L	20.916	0.220	0.403	0.585	0.701	20.916	0.391	0.394	0.321	0.582	20.916	0.171	0.460	0.711	0.850
NBDY	361.687	0.309	0.199	0.120	0.224	361.687	0.246	0.198	0.215	0.494	361.687	-0.063	0.229	0.783	0.891
SLC52A1	12.166	0.912	0.794	0.250	0.384	12.166	1.100	0.788	0.162	0.453	12.166	0.188	0.904	0.835	0.920
PDPR	889.838	-0.191	0.263	0.467	0.602	889.838	0.158	0.263	0.547	0.741	889.838	0.350	0.304	0.250	0.482
ENOX1	43.203	-0.889	0.383	0.020	0.058	43.203	-0.402	0.370	0.278	0.548	43.203	0.488	0.442	0.270	0.505
TMEM248	1043.643	0.149	0.146	0.308	0.447	1043.643	-0.145	0.146	0.320	0.581	1043.643	-0.294	0.169	0.082	0.247
PPP1R3C	910.288	-0.124	0.366	0.734	0.821	910.288	0.380	0.366	0.298	0.566	910.288	0.504	0.422	0.233	0.462
DET1	99.483	-0.760	0.223	0.001	0.004	99.483	-0.050	0.211	0.812	0.901	99.483	0.710	0.255	0.005	0.043
C9orf40	326.190	-0.024	0.223	0.915	0.946	326.190	-0.312	0.224	0.164	0.454	326.190	-0.288	0.259	0.267	0.501
RNF31	618.229	-0.246	0.137	0.073	0.154	618.229	0.008	0.136	0.952	0.976	618.229	0.254	0.158	0.108	0.293
LRRC37A4P	363.678	0.075	0.261	0.775	0.850	363.678	0.061	0.260	0.814	0.903	363.678	-0.013	0.301	0.964	0.983
OXR1	858.530	-0.371	0.212	0.080	0.165	858.530	0.156	0.211	0.459	0.685	858.530	0.527	0.244	0.031	0.134
UACA	570.827	-0.066	0.223	0.767	0.846	570.827	0.233	0.222	0.294	0.561	570.827	0.299	0.257	0.244	0.475
TMEM45A	2109.301	0.330	0.791	0.677	0.775	2109.301	0.799	0.791	0.313	0.576	2109.301	0.469	0.914	0.607	0.783
TAPBPL	227.252	1.051	0.211	0.000	0.000	227.252	0.263	0.214	0.220	0.499	227.252	-0.788	0.245	0.001	0.016
IFT57	683.180	0.164	0.158	0.298	0.437	683.180	-0.142	0.158	0.370	0.619	683.180	-0.306	0.183	0.094	0.269
ARGLU1	570.555	-0.209	0.259	0.419	0.556	570.555	-0.176	0.259	0.498	0.711	570.555	0.034	0.299	0.910	0.959
KIF26B	25.069	1.886	0.519	0.000	0.002	25.069	-0.561	0.576	0.330	0.590	25.069	-2.447	0.633	0.000	0.003
RADX	155.167	-0.325	0.381	0.394	0.533	155.167	0.166	0.379	0.661	0.815	155.167	0.491	0.439	0.264	0.498
CCDC186	300.596	-0.840	0.261	0.001	0.007	300.596	-0.113	0.257	0.659	0.814	300.596	0.727	0.300	0.015	0.087
SLC38A4	20.753	-1.068	0.398	0.007	0.026	20.753	-0.552	0.371	0.137	0.429	20.753	0.516	0.461	0.263	0.498
PPP1R3D	66.953	0.057	0.280	0.839	0.894	66.953	0.091	0.277	0.744	0.863	66.953	0.034	0.323	0.916	0.961
MED9	121.495	0.123	0.193	0.521	0.649	121.495	-0.044	0.192	0.818	0.905	121.495	-0.168	0.224	0.453	0.672
TMEM51	484.454	0.918	0.162	0.000	0.000	484.454	0.058	0.165	0.728	0.854	484.454	-0.860	0.188	0.000	0.000
WDYHV1	182.869	-0.373	0.177	0.035	0.089	182.869	0.121	0.172	0.483	0.701	182.869	0.494	0.203	0.015	0.086
GPATCH1	199.897	-0.185	0.170	0.276	0.413	199.897	-0.298	0.169	0.078	0.370	199.897	-0.113	0.198	0.568	0.758
SAMD4B	1619.714	-0.097	0.136	0.475	0.609	1619.714	0.055	0.135	0.686	0.830	1619.714	0.151	0.156	0.333	0.568
PPP1R7	893.945	0.483	0.138	0.000	0.003	893.945	0.008	0.139	0.954	0.977	893.945	-0.475	0.160	0.003	0.029
WDR70	672.058	-0.148	0.129	0.250	0.384	672.058	-0.032	0.128	0.805	0.898	672.058	0.117	0.149	0.433	0.655
DMAC2	785.323	0.034	0.135	0.799	0.868	785.323	0.063	0.135	0.637	0.802	785.323	0.029	0.156	0.852	0.929
ATG2B	364.397	-0.590	0.264	0.026	0.070	364.397	-0.542	0.264	0.040	0.327	364.397	0.048	0.306	0.876	0.941
RALGPS2	1728.922	-0.659	0.246	0.007	0.027	1728.922	-0.206	0.245	0.402	0.644	1728.922	0.453	0.284	0.111	0.297
GPATCH2	210.528	-1.076	0.343	0.002	0.009	210.528	-0.608	0.340	0.073	0.362	210.528	0.467	0.396	0.238	0.468
SLFN12	144.755	-0.636	0.325	0.051	0.117	144.755	0.199	0.320	0.534	0.733	144.755	0.835	0.374	0.026	0.119
BSDC1	666.895	0.058	0.167	0.729	0.816	666.895	0.229	0.166	0.170	0.459	666.895	0.171	0.193	0.376	0.608
AGGF1	718.818	-0.295	0.136	0.030	0.079	718.818	-0.180	0.135	0.183	0.470	718.818	0.115	0.157	0.466	0.681
PPP1R8	855.766	-0.250	0.135	0.064	0.139	855.766	-0.264	0.135	0.050	0.337	855.766	-0.014	0.156	0.930	0.968
MAGOHB	365.640	-0.073	0.414	0.859	0.908	365.640	-0.985	0.415	0.018	0.306	365.640	-0.912	0.480	0.057	0.199
PLEKHJ1	770.832	-0.012	0.230	0.959	0.975	770.832	-0.168	0.230	0.465	0.689	770.832	-0.156	0.266	0.557	0.749
WDR60	484.885	0.554	0.394	0.160	0.278	484.885	0.670	0.394	0.089	0.382	484.885	0.116	0.455	0.798	0.899
XKR8	70.128	2.062	0.240	0.000	0.000	70.128	0.522	0.260	0.044	0.332	70.128	-1.540	0.278	0.000	0.000
ARHGAP17	340.185	0.475	0.124	0.000	0.001	340.185	-0.082	0.126	0.514	0.720	340.185	-0.558	0.145	0.000	0.003
TMEM39B	494.815	0.275	0.134	0.040	0.097	494.815	0.328	0.133	0.013	0.305	494.815	0.053	0.154	0.728	0.859
SLC6A15	19.247	0.902	0.679	0.184	0.308	19.247	-0.064	0.720	0.930	0.965	19.247	-0.966	0.805	0.231	0.459
CRTAC1	11.634	0.299	0.882	0.735	0.821	11.634	0.933	0.855	0.275	0.545	11.634	0.634	0.997	0.525	0.726
PRPF38B	1420.106	0.135	0.241	0.576	0.694	1420.106	-0.450	0.242	0.063	0.350	1420.106	-0.585	0.279	0.036	0.148
FANCL	326.312	0.021	0.155	0.893	0.931	326.312	-0.196	0.155	0.207	0.489	326.312	-0.216	0.180	0.228	0.457
AKIRIN2	1809.319	-0.588	0.179	0.001	0.006	1809.319	-0.322	0.178	0.071	0.360	1809.319	0.266	0.206	0.198	0.421
CEP192	312.898	-0.703	0.287	0.014	0.044	312.898	-0.188	0.285	0.508	0.717	312.898	0.514	0.331	0.120	0.312
HEATR1	1679.517	-0.768	0.251	0.002	0.010	1679.517	-0.481	0.250	0.054	0.342	1679.517	0.287	0.289	0.321	0.556
TRIM68	155.794	-0.193	0.206	0.349	0.489	155.794	-0.117	0.204	0.567	0.755	155.794	0.076	0.238	0.749	0.871
ANO10	486.251	0.676	0.119	0.000	0.000	486.251	-0.066	0.122	0.589	0.770	486.251	-0.742	0.139	0.000	0.000
ARMC4	79.830	-0.632	0.379	0.095	0.189	79.830	0.252	0.370	0.496	0.711	79.830	0.884	0.435	0.042	0.164
RBM28	666.200	-0.734	0.318	0.021	0.059	666.200	-0.723	0.317	0.023	0.315	666.200	0.011	0.367	0.976	0.987
LARP1B	450.309	-0.942	0.261	0.000	0.002	450.309	-0.423	0.259	0.102	0.394	450.309	0.519	0.301	0.085	0.254
SRBD1	295.638	-0.616	0.221	0.005	0.021	295.638	-0.591	0.220	0.007	0.297	295.638	0.025	0.256	0.921	0.963
WRAP53	203.914	-0.205	0.177	0.245	0.379	203.914	-0.364	0.177	0.039	0.327	203.914	-0.159	0.206	0.441	0.661

FIGN	153.589	-0.657	0.222	0.003	0.014	153.589	-0.206	0.218	0.343	0.600	153.589	0.451	0.257	0.079	0.242
ANKZF1	896.080	0.466	0.222	0.036	0.090	896.080	0.325	0.222	0.144	0.435	896.080	-0.141	0.257	0.582	0.765
PPP1R10	4357.722	-0.069	0.192	0.719	0.809	4357.722	0.193	0.192	0.315	0.578	4357.722	0.262	0.222	0.238	0.467
ELP3	916.975	-0.388	0.086	0.000	0.000	916.975	-0.047	0.084	0.574	0.760	916.975	0.340	0.099	0.001	0.009
HAUS2	476.982	-0.644	0.266	0.015	0.047	476.982	-0.613	0.265	0.021	0.312	476.982	0.032	0.307	0.918	0.962
CDCA8	644.358	-0.402	0.196	0.041	0.099	644.358	0.060	0.195	0.759	0.872	644.358	0.462	0.227	0.042	0.163
LRRC8D	1247.362	-0.758	0.197	0.000	0.001	1247.362	-0.399	0.197	0.042	0.330	1247.362	0.359	0.228	0.116	0.305
THAP1	260.085	-0.862	0.196	0.000	0.000	260.085	-0.136	0.191	0.476	0.697	260.085	0.726	0.226	0.001	0.016
ZDHHC4	711.474	0.093	0.153	0.546	0.670	711.474	0.121	0.153	0.428	0.663	711.474	0.028	0.177	0.872	0.939
RBM23	654.196	-0.344	0.147	0.019	0.055	654.196	-0.151	0.146	0.298	0.565	654.196	0.192	0.169	0.256	0.490
UBR7	1399.655	-0.392	0.190	0.038	0.095	1399.655	-0.338	0.189	0.074	0.363	1399.655	0.054	0.219	0.805	0.903
MTPAP	535.436	-0.473	0.211	0.025	0.068	535.436	-0.314	0.210	0.135	0.426	535.436	0.159	0.244	0.513	0.718
PPP2CA	2750.456	-0.433	0.236	0.066	0.143	2750.456	-0.663	0.236	0.005	0.297	2750.456	-0.230	0.273	0.400	0.627
C19orf73	20.168	-0.426	0.459	0.354	0.494	20.168	-1.210	0.482	0.012	0.305	20.168	-0.784	0.560	0.162	0.375
TMEM38B	778.715	-0.339	0.112	0.003	0.012	778.715	-0.172	0.111	0.120	0.412	778.715	0.166	0.130	0.200	0.423
DALRD3	961.802	0.478	0.188	0.011	0.036	961.802	0.348	0.187	0.063	0.350	961.802	-0.130	0.216	0.547	0.742
SDAD1	615.686	-0.517	0.252	0.040	0.098	615.686	-0.502	0.252	0.046	0.335	615.686	0.015	0.291	0.960	0.981
MSTO1	432.897	-0.468	0.356	0.188	0.313	432.897	-0.787	0.356	0.027	0.319	432.897	-0.319	0.412	0.439	0.659
ARMC1	681.621	-0.650	0.216	0.003	0.012	681.621	-0.486	0.216	0.024	0.316	681.621	0.164	0.250	0.513	0.718
DARS2	602.331	-0.736	0.263	0.005	0.020	602.331	-0.526	0.262	0.045	0.334	602.331	0.210	0.304	0.489	0.699
RFWD3	764.136	-0.659	0.320	0.039	0.096	764.136	-0.733	0.320	0.022	0.314	764.136	-0.073	0.370	0.843	0.924
PPP2CB	1611.610	-0.415	0.149	0.005	0.021	1611.610	-0.171	0.148	0.248	0.522	1611.610	0.244	0.172	0.156	0.367
ARHGEF10L	429.049	1.226	0.172	0.000	0.000	429.049	0.282	0.175	0.106	0.399	429.049	-0.944	0.200	0.000	0.000
TMEM33	2355.327	-0.740	0.160	0.000	0.000	2355.327	-0.125	0.159	0.432	0.667	2355.327	0.615	0.184	0.001	0.012
PNPO	702.448	-0.311	0.166	0.061	0.135	702.448	-0.307	0.166	0.064	0.351	702.448	0.004	0.192	0.985	0.992
SHQ1	498.822	-0.575	0.197	0.003	0.015	498.822	-0.492	0.196	0.012	0.305	498.822	0.083	0.228	0.715	0.852
CEP55	1497.164	-0.422	0.242	0.081	0.167	1497.164	-0.354	0.242	0.143	0.435	1497.164	0.068	0.279	0.808	0.904
CENPQ	206.275	-0.462	0.225	0.040	0.098	206.275	-0.280	0.223	0.209	0.490	206.275	0.181	0.261	0.486	0.697
MSL2	775.944	-1.127	0.215	0.000	0.000	775.944	-0.199	0.213	0.350	0.605	775.944	0.928	0.248	0.000	0.004
MRPS18A	585.302	-0.229	0.231	0.322	0.462	585.302	-0.158	0.231	0.493	0.708	585.302	0.071	0.267	0.791	0.896
PRMT6	427.822	-0.519	0.281	0.065	0.140	427.822	-0.722	0.281	0.010	0.299	427.822	-0.203	0.325	0.532	0.731
TBCCD1	187.462	-0.731	0.239	0.002	0.011	187.462	-0.415	0.235	0.078	0.370	187.462	0.316	0.276	0.253	0.485
DNAAF2	374.454	-0.432	0.229	0.059	0.130	374.454	-0.352	0.228	0.122	0.413	374.454	0.080	0.265	0.763	0.880
MRPS10	1005.464	-0.412	0.297	0.165	0.285	1005.464	-0.709	0.297	0.017	0.306	1005.464	-0.297	0.343	0.387	0.616
INTS10	1080.187	-0.227	0.145	0.117	0.220	1080.187	0.167	0.144	0.247	0.522	1080.187	0.394	0.167	0.018	0.096
KLHL11	186.595	0.213	0.210	0.311	0.451	186.595	0.016	0.211	0.940	0.970	186.595	-0.197	0.244	0.418	0.643
SEC61A2	336.752	0.581	0.286	0.042	0.101	336.752	-0.406	0.288	0.159	0.451	336.752	-0.986	0.332	0.003	0.028
RMDN3	808.059	-0.671	0.274	0.014	0.045	808.059	-0.551	0.274	0.044	0.332	808.059	0.120	0.317	0.704	0.846
MRM3	195.499	-0.121	0.201	0.547	0.671	195.499	-0.036	0.200	0.856	0.927	195.499	0.085	0.233	0.715	0.852
FAIM	220.984	0.264	0.215	0.219	0.350	220.984	-0.202	0.217	0.352	0.606	220.984	-0.466	0.250	0.062	0.210
PPP2R1A	4682.852	0.222	0.102	0.030	0.078	4682.852	0.182	0.102	0.075	0.364	4682.852	-0.040	0.118	0.733	0.862
LINS1	139.035	-0.717	0.248	0.004	0.016	139.035	-0.453	0.244	0.063	0.350	139.035	0.263	0.287	0.359	0.592
SMG8	351.749	-0.256	0.171	0.134	0.244	351.749	0.054	0.169	0.751	0.867	351.749	0.309	0.197	0.116	0.305
RNF220	1437.175	0.263	0.170	0.123	0.228	1437.175	-0.174	0.171	0.306	0.572	1437.175	-0.437	0.197	0.026	0.121
RIF1	980.515	-1.223	0.291	0.000	0.000	980.515	-0.693	0.290	0.017	0.306	980.515	0.530	0.337	0.115	0.304
DZANK1	15.159	-0.164	0.565	0.772	0.848	15.159	-0.208	0.560	0.710	0.844	15.159	-0.045	0.657	0.946	0.975
SLC25A36	2044.753	-0.268	0.266	0.315	0.454	2044.753	0.255	0.266	0.337	0.595	2044.753	0.523	0.307	0.089	0.261
VPS13D	862.964	-0.307	0.189	0.104	0.201	862.964	-0.200	0.189	0.290	0.558	862.964	0.108	0.218	0.621	0.792
RIC8B	230.199	-0.077	0.151	0.608	0.720	230.199	0.197	0.147	0.181	0.469	230.199	0.274	0.173	0.113	0.300
PPP2R1B	329.091	-0.390	0.235	0.097	0.191	329.091	-0.404	0.234	0.084	0.376	329.091	-0.015	0.272	0.957	0.980
NUDT11	412.345	-0.154	0.162	0.344	0.484	412.345	0.039	0.161	0.810	0.900	412.345	0.192	0.187	0.304	0.539
NADSYN1	698.319	0.803	0.174	0.000	0.000	698.319	0.183	0.175	0.297	0.565	698.319	-0.620	0.201	0.002	0.022
DNAJC17	365.333	0.131	0.165	0.425	0.562	365.333	0.057	0.164	0.729	0.855	365.333	-0.074	0.190	0.696	0.841
PBRM1	774.952	-0.781	0.204	0.000	0.001	774.952	-0.403	0.203	0.047	0.336	774.952	0.378	0.236	0.109	0.294
EVA1B	179.799	0.941	0.302	0.002	0.009	179.799	0.660	0.303	0.029	0.322	179.799	-0.281	0.347	0.418	0.642
RESF1	760.303	-0.416	0.271	0.124	0.230	760.303	-0.553	0.271	0.041	0.329	760.303	-0.137	0.313	0.662	0.820
RPRD1A	1380.370	-0.721	0.191	0.000	0.001	1380.370	-0.191	0.191	0.317	0.579	1380.370	0.530	0.221	0.016	0.090
APPL2	219.712	-0.528	0.224	0.019	0.055	219.712	-0.001	0.221	0.997	0.999	219.712	0.527	0.258	0.041	0.163
FAM86C1	127.228	-1.017	0.425	0.017	0.050	127.228	-1.072	0.424	0.012	0.305	127.228	-0.055	0.494	0.912	0.960
PPP2R2A	1064.459	-0.966	0.266	0.000	0.002	1064.459	-0.478	0.265	0.072	0.361	1064.459	0.488	0.308	0.112	0.300
PLEKHG6	20.444	0.208	0.394	0.597	0.711	20.444	0.325	0.386	0.400	0.643	20.444	0.117	0.450	0.795	0.897
MAP1S	1088.462	0.683	0.166	0.000	0.000	1088.462	0.217	0.166	0.191	0.477	1088.462	-0.465	0.192	0.015	0.086
GOLPH3L	441.262	-0.323	0.227	0.155	0.271	441.262	0.195	0.225	0.388	0.634	441.262	0.518	0.262	0.048	0.178
ZNF532	864.422	-0.529	0.176	0.003	0.012	864.422	-0.196	0.175	0.264	0.536	864.422	0.333	0.203	0.102	0.284
SBNO1	1406.370	-0.180	0.150	0.229	0.360	1406.370	-0.234	0.149	0.117	0.409	1406.370	-0.054	0.173	0.755	0.875
ARL8B	1882.100	-0.088	0.155	0.570	0.689	1882.100	-0.015	0.155	0.921	0.960	1882.100	0.073	0.179	0.685	0.834
DCUN1D2	131.533	-0.289	0.203	0.155	0.271	131.533	-0.228	0.200	0.253	0.526	131.533	0.061	0.236	0.798	0.899

SETD5	1809.132	-0.125	0.154	0.416	0.554	1809.132	-0.084	0.153	0.585	0.768	1809.132	0.041	0.177	0.817	0.909
ATAD3A	1371.927	-0.859	0.482	0.075	0.157	1371.927	-1.280	0.482	0.008	0.297	1371.927	-0.422	0.557	0.449	0.668
BBS7	330.830	-0.603	0.205	0.003	0.014	330.830	-0.142	0.202	0.483	0.701	330.830	0.461	0.236	0.051	0.185
RCBTB1	337.186	-0.111	0.170	0.514	0.643	337.186	-0.129	0.169	0.448	0.677	337.186	-0.018	0.197	0.928	0.967
P3H2	2713.141	-0.198	0.148	0.180	0.303	2713.141	0.010	0.147	0.945	0.973	2713.141	0.208	0.170	0.222	0.450
FANCI	1189.824	-0.742	0.231	0.001	0.007	1189.824	-0.382	0.231	0.098	0.392	1189.824	0.360	0.267	0.177	0.394
NKAPD1	496.218	-0.304	0.124	0.014	0.044	496.218	0.019	0.122	0.874	0.937	496.218	0.323	0.143	0.024	0.113
TMLHE	293.850	-0.256	0.291	0.379	0.519	293.850	0.111	0.290	0.701	0.839	293.850	0.368	0.336	0.274	0.509
EXD2	508.682	-0.326	0.218	0.134	0.243	508.682	0.220	0.216	0.309	0.573	508.682	0.546	0.251	0.029	0.130
MACO1	503.405	0.440	0.176	0.013	0.041	503.405	-0.086	0.178	0.628	0.796	503.405	-0.526	0.205	0.010	0.067
LRRC20	103.189	0.394	0.288	0.171	0.291	103.189	-0.361	0.292	0.217	0.496	103.189	-0.755	0.336	0.025	0.116
TRIM62	59.242	0.384	0.238	0.106	0.204	59.242	0.049	0.241	0.837	0.916	59.242	-0.335	0.277	0.226	0.455
ETNK2	132.984	-0.257	0.681	0.706	0.798	132.984	0.767	0.678	0.258	0.531	132.984	1.024	0.785	0.192	0.414
RAVER2	667.389	-0.182	0.297	0.541	0.665	667.389	0.317	0.297	0.285	0.554	667.389	0.499	0.343	0.146	0.353
NAT10	1995.343	-0.574	0.214	0.007	0.027	1995.343	-0.391	0.214	0.068	0.356	1995.343	0.182	0.247	0.461	0.678
LRRC1	198.474	-0.369	0.255	0.147	0.260	198.474	-0.499	0.254	0.050	0.337	198.474	-0.129	0.296	0.662	0.819
PANK4	874.750	0.110	0.112	0.326	0.466	874.750	-0.010	0.112	0.929	0.964	874.750	-0.120	0.129	0.354	0.587
PPP2R3A	451.521	-0.202	0.233	0.384	0.524	451.521	0.051	0.232	0.825	0.909	451.521	0.254	0.269	0.345	0.579
USP40	423.328	-0.120	0.175	0.493	0.625	423.328	-0.267	0.175	0.127	0.420	423.328	-0.147	0.203	0.469	0.684
MOB1A	1849.693	-0.083	0.119	0.485	0.618	1849.693	-0.272	0.119	0.022	0.314	1849.693	-0.189	0.137	0.167	0.382
SMU1	1333.515	-0.325	0.149	0.029	0.077	1333.515	-0.325	0.149	0.029	0.322	1333.515	0.000	0.172	1.000	1.000
UBA6	883.292	-0.520	0.269	0.054	0.122	883.292	-0.692	0.269	0.010	0.299	883.292	-0.172	0.311	0.581	0.764
SLC38A7	246.657	0.875	0.305	0.004	0.017	246.657	-0.316	0.309	0.307	0.572	246.657	-1.191	0.355	0.001	0.011
OGFOD1	1034.956	-0.443	0.289	0.126	0.232	1034.956	-0.761	0.290	0.009	0.297	1034.956	-0.318	0.335	0.342	0.576
PTPA	2795.878	-0.147	0.120	0.219	0.349	2795.878	0.047	0.120	0.697	0.837	2795.878	0.194	0.138	0.161	0.374
STEAP3	2961.219	1.119	0.204	0.000	0.000	2961.219	0.473	0.205	0.021	0.312	2961.219	-0.646	0.236	0.006	0.047
KIRREL1	4120.465	-0.543	0.266	0.041	0.099	4120.465	0.181	0.265	0.495	0.710	4120.465	0.723	0.307	0.018	0.096
UQCC1	496.852	-0.028	0.117	0.810	0.875	496.852	0.094	0.116	0.415	0.653	496.852	0.123	0.135	0.364	0.596
CCDC25	1316.463	-0.116	0.178	0.515	0.643	1316.463	0.127	0.178	0.473	0.695	1316.463	0.243	0.205	0.237	0.466
NEIL3	192.321	-0.369	0.370	0.319	0.459	192.321	-0.144	0.369	0.696	0.837	192.321	0.225	0.428	0.599	0.777
PACC1	256.114	-0.494	0.382	0.196	0.322	256.114	-0.976	0.384	0.011	0.302	256.114	-0.482	0.444	0.277	0.512
YY1AP1	1212.060	-0.570	0.163	0.000	0.003	1212.060	0.071	0.162	0.660	0.814	1212.060	0.641	0.188	0.001	0.010
PPP2R5A	573.444	-0.154	0.158	0.330	0.470	573.444	0.196	0.157	0.211	0.492	573.444	0.350	0.182	0.055	0.194
ELP2	761.950	-0.446	0.124	0.000	0.002	761.950	-0.101	0.123	0.412	0.651	761.950	0.345	0.144	0.016	0.090
PCMTD2	583.071	-0.389	0.212	0.066	0.143	583.071	0.071	0.210	0.735	0.858	583.071	0.460	0.244	0.059	0.203
ASXL2	486.284	-0.282	0.263	0.284	0.422	486.284	-0.271	0.263	0.302	0.568	486.284	0.011	0.304	0.972	0.986
TYW1	569.439	-0.889	0.267	0.001	0.005	569.439	-0.627	0.266	0.019	0.306	569.439	0.262	0.309	0.397	0.624
TMEM39A	685.739	-0.224	0.195	0.251	0.385	685.739	0.088	0.194	0.652	0.810	685.739	0.312	0.225	0.166	0.381
WDR41	1252.126	-0.651	0.155	0.000	0.000	1252.126	0.040	0.153	0.793	0.892	1252.126	0.691	0.178	0.000	0.003
ADI1	3269.391	-0.046	0.146	0.752	0.835	3269.391	0.059	0.146	0.688	0.831	3269.391	0.105	0.169	0.535	0.733
MRGBP	779.631	0.305	0.155	0.049	0.114	779.631	0.208	0.155	0.181	0.469	779.631	-0.098	0.179	0.586	0.767
PPP2R5B	473.764	0.929	0.234	0.000	0.001	473.764	0.240	0.236	0.308	0.573	473.764	-0.689	0.271	0.011	0.069
TMEM143	84.091	0.650	0.261	0.013	0.041	84.091	0.570	0.260	0.028	0.321	84.091	-0.079	0.299	0.792	0.896
MAP11	94.427	-0.097	0.354	0.783	0.856	94.427	-0.669	0.357	0.061	0.350	94.427	-0.571	0.413	0.166	0.381
TMEM19	254.144	-0.019	0.199	0.922	0.951	254.144	-0.050	0.198	0.802	0.896	254.144	-0.030	0.230	0.895	0.951
ECHDC2	81.989	1.165	0.441	0.008	0.029	81.989	0.504	0.443	0.256	0.529	81.989	-0.661	0.508	0.193	0.416
PSPC1	667.327	-0.707	0.301	0.019	0.055	667.327	-0.724	0.301	0.016	0.306	667.327	-0.016	0.348	0.962	0.982
PPP2R5C	1792.818	-0.173	0.120	0.148	0.262	1792.818	0.111	0.119	0.351	0.605	1792.818	0.284	0.138	0.039	0.157
NUDT15	467.350	-0.296	0.193	0.126	0.232	467.350	-0.212	0.193	0.271	0.542	467.350	0.084	0.224	0.708	0.849
IMP3	1443.386	0.133	0.145	0.358	0.498	1443.386	0.024	0.145	0.866	0.931	1443.386	-0.109	0.167	0.516	0.720
TMEM100	155.749	-0.381	0.340	0.262	0.397	155.749	0.459	0.335	0.170	0.460	155.749	0.840	0.390	0.031	0.136
PHF10	946.942	-0.078	0.178	0.663	0.765	946.942	0.179	0.178	0.314	0.577	946.942	0.257	0.206	0.212	0.437
VPS53	332.211	0.553	0.226	0.015	0.045	332.211	-0.469	0.230	0.041	0.329	332.211	-1.022	0.264	0.000	0.003
PGM2	1177.727	-0.703	0.231	0.002	0.011	1177.727	-0.093	0.231	0.686	0.831	1177.727	0.610	0.267	0.022	0.109
FGGY	117.532	0.007	0.424	0.986	0.992	117.532	0.561	0.421	0.182	0.469	117.532	0.553	0.488	0.256	0.490
QRSL1	536.453	-0.787	0.229	0.001	0.004	536.453	-0.248	0.228	0.275	0.545	536.453	0.539	0.265	0.042	0.164
ZNF654	212.854	-0.238	0.304	0.434	0.570	212.854	-0.112	0.302	0.711	0.845	212.854	0.125	0.351	0.721	0.855
PPP2R5D	1525.263	-0.207	0.135	0.124	0.229	1525.263	0.113	0.134	0.401	0.644	1525.263	0.320	0.155	0.039	0.158
CWF19L1	298.111	-0.459	0.308	0.137	0.247	298.111	-0.489	0.308	0.112	0.405	298.111	-0.031	0.357	0.931	0.969
TMEM140	199.492	-0.465	0.216	0.031	0.081	199.492	-0.326	0.214	0.127	0.420	199.492	0.138	0.250	0.581	0.764
MCOLN3	11.767	-0.047	0.491	0.923	0.951	11.767	-0.432	0.501	0.389	0.635	11.767	-0.385	0.585	0.511	0.716
UBE2W	391.482	-0.387	0.133	0.004	0.015	391.482	-0.211	0.131	0.108	0.400	391.482	0.176	0.154	0.253	0.486
RBM41	253.978	-0.558	0.189	0.003	0.014	253.978	-0.002	0.185	0.993	0.997	253.978	0.556	0.217	0.011	0.068
TMEM40	193.371	1.035	0.518	0.046	0.108	193.371	0.768	0.519	0.139	0.430	193.371	-0.267	0.598	0.655	0.815
RHOT1	1131.759	-0.275	0.152	0.071	0.151	1131.759	0.067	0.151	0.657	0.813	1131.759	0.342	0.176	0.051	0.186
ATXN7L3B	1562.900	-0.277	0.155	0.074	0.155	1562.900	-0.001	0.154	0.996	0.998	1562.900	0.276	0.179	0.122	0.314
PPP2R5E	963.665	-0.731	0.216	0.001	0.004	963.665	-0.511	0.215	0.018	0.306	963.665	0.220	0.250	0.378	0.609

BRF2	376.130	0.563	0.186	0.002	0.011	376.130	0.201	0.186	0.280	0.549	376.130	-0.362	0.214	0.092	0.265
BOLA2	590.293	0.517	0.222	0.020	0.057	590.293	-0.064	0.222	0.772	0.879	590.293	-0.581	0.256	0.023	0.112
PPP6R3	1159.456	-0.829	0.233	0.000	0.003	1159.456	-0.498	0.233	0.032	0.322	1159.456	0.331	0.269	0.219	0.446
UEVLD	382.752	-0.289	0.202	0.152	0.267	382.752	-0.396	0.201	0.049	0.337	382.752	-0.107	0.234	0.646	0.809
FBXW7	220.382	-0.168	0.165	0.311	0.450	220.382	-0.286	0.165	0.083	0.375	220.382	-0.118	0.193	0.540	0.737
KLHL26	355.516	-0.173	0.237	0.465	0.599	355.516	0.514	0.234	0.028	0.321	355.516	0.688	0.272	0.012	0.072
TBC1D19	128.697	-0.576	0.288	0.046	0.108	128.697	0.219	0.281	0.436	0.669	128.697	0.795	0.331	0.016	0.090
CCDC91	104.363	-0.165	0.182	0.366	0.507	104.363	-0.345	0.182	0.059	0.348	104.363	-0.180	0.214	0.400	0.627
RNF121	333.957	-0.311	0.154	0.044	0.104	333.957	-0.197	0.152	0.196	0.480	333.957	0.114	0.178	0.524	0.725
BRIX1	1209.120	-0.597	0.318	0.061	0.134	1209.120	-0.840	0.319	0.008	0.297	1209.120	-0.243	0.368	0.510	0.715
PPP3CA	1884.156	-0.530	0.189	0.005	0.020	1884.156	-0.043	0.188	0.819	0.905	1884.156	0.487	0.218	0.026	0.119
PI4K2B	605.515	-0.773	0.218	0.000	0.003	605.515	-0.344	0.217	0.113	0.405	605.515	0.430	0.252	0.088	0.259
OLAH	80.677	-2.759	0.454	0.000	0.000	80.677	0.058	0.359	0.872	0.935	80.677	2.817	0.499	0.000	0.000
SPTLC3	309.295	0.463	0.565	0.412	0.550	309.295	0.800	0.564	0.156	0.448	309.295	0.337	0.651	0.605	0.782
DDX19A	744.904	-0.206	0.282	0.465	0.600	744.904	-0.704	0.283	0.013	0.305	744.904	-0.498	0.327	0.128	0.323
PPP4C	2188.014	0.229	0.238	0.336	0.476	2188.014	-0.357	0.238	0.135	0.426	2188.014	-0.586	0.275	0.033	0.141
MIR3936HG	31.321	0.167	0.325	0.608	0.721	31.321	0.384	0.316	0.225	0.503	31.321	0.218	0.371	0.557	0.749
ZNF444	304.336	0.338	0.233	0.147	0.261	304.336	0.036	0.234	0.879	0.939	304.336	-0.302	0.270	0.262	0.496
PEF1	807.160	0.284	0.191	0.136	0.246	807.160	-0.192	0.191	0.316	0.578	807.160	-0.476	0.221	0.031	0.135
RFK	490.959	-0.498	0.310	0.107	0.206	490.959	-0.698	0.310	0.024	0.316	490.959	-0.200	0.358	0.577	0.762
CPPED1	164.167	-0.099	0.166	0.550	0.672	164.167	-0.057	0.164	0.728	0.855	164.167	0.042	0.192	0.826	0.914
TMEM144	89.263	0.353	0.203	0.083	0.170	89.263	0.095	0.204	0.642	0.805	89.263	-0.258	0.236	0.274	0.510
SLC29A3	58.192	0.125	0.296	0.674	0.773	58.192	-0.510	0.304	0.093	0.388	58.192	-0.635	0.350	0.070	0.226
RSAD1	487.035	-0.414	0.205	0.044	0.104	487.035	-0.188	0.204	0.357	0.611	487.035	0.226	0.237	0.340	0.575
AP5S1	172.580	-0.347	0.258	0.179	0.301	172.580	-0.272	0.257	0.289	0.557	172.580	0.075	0.299	0.802	0.901
TMA16	537.445	-0.381	0.158	0.016	0.048	537.445	-0.365	0.157	0.020	0.311	537.445	0.016	0.183	0.930	0.968
PPP3CB	692.537	-0.628	0.112	0.000	0.000	692.537	-0.168	0.110	0.127	0.419	692.537	0.460	0.130	0.000	0.007
MIS18BP1	696.116	-0.849	0.226	0.000	0.001	696.116	-0.065	0.225	0.773	0.879	696.116	0.784	0.261	0.003	0.026
TMEM74B	19.942	1.916	0.641	0.003	0.013	19.942	1.084	0.664	0.102	0.395	19.942	-0.832	0.715	0.245	0.476
C5orf22	497.954	-0.346	0.213	0.105	0.203	497.954	-0.510	0.213	0.017	0.306	497.954	-0.164	0.247	0.507	0.713
LARP6	949.733	-0.523	0.199	0.009	0.031	949.733	0.063	0.198	0.750	0.867	949.733	0.586	0.230	0.011	0.069
ABCF3	995.270	-0.249	0.131	0.057	0.128	995.270	-0.130	0.130	0.317	0.579	995.270	0.118	0.151	0.434	0.656
UFSP2	677.333	0.010	0.283	0.972	0.983	677.333	0.429	0.282	0.128	0.420	677.333	0.420	0.327	0.199	0.422
AGPAT5	1653.236	-0.612	0.161	0.000	0.001	1653.236	-0.208	0.160	0.194	0.478	1653.236	0.404	0.186	0.030	0.132
LIN7C	459.167	-0.074	0.189	0.696	0.791	459.167	-0.265	0.189	0.161	0.452	459.167	-0.191	0.219	0.382	0.611
MNS1	164.180	-0.215	0.211	0.308	0.447	164.180	0.162	0.207	0.435	0.668	164.180	0.377	0.243	0.120	0.311
PPP3CC	530.101	-0.132	0.124	0.288	0.426	530.101	0.053	0.123	0.664	0.818	530.101	0.185	0.143	0.196	0.419
BLOC1S4	337.777	0.174	0.173	0.315	0.455	337.777	0.140	0.172	0.416	0.654	337.777	-0.033	0.200	0.867	0.936
ACER3	344.692	0.177	0.239	0.459	0.594	344.692	-0.714	0.242	0.003	0.297	344.692	-0.891	0.279	0.001	0.017
DRAM1	2167.990	0.596	0.149	0.000	0.001	2167.990	-0.178	0.150	0.235	0.513	2167.990	-0.773	0.172	0.000	0.000
SYNJ2BP	443.822	-0.275	0.197	0.163	0.282	443.822	-0.120	0.196	0.541	0.738	443.822	0.155	0.228	0.496	0.704
SLC39A9	1500.607	-0.098	0.134	0.466	0.600	1500.607	-0.236	0.134	0.079	0.370	1500.607	-0.138	0.155	0.373	0.605
NIPSNAP3B	8.058	1.252	0.771	0.104	0.202	8.058	0.652	0.783	0.405	0.646	8.058	-0.600	0.883	0.497	0.705
FBXL8	95.746	0.622	0.240	0.009	0.032	95.746	0.310	0.241	0.198	0.482	95.746	-0.312	0.276	0.258	0.492
SHFL	546.729	0.861	0.222	0.000	0.001	546.729	0.432	0.222	0.052	0.340	546.729	-0.428	0.256	0.094	0.270
WDR33	1065.682	-0.195	0.094	0.038	0.093	1065.682	-0.009	0.093	0.921	0.960	1065.682	0.186	0.109	0.086	0.256
PPP3R1	733.617	-0.492	0.181	0.007	0.025	733.617	-0.327	0.180	0.070	0.358	733.617	0.165	0.210	0.430	0.653
LSG1	869.072	-0.804	0.401	0.045	0.107	869.072	-1.064	0.401	0.008	0.297	869.072	-0.259	0.464	0.576	0.762
STRBP	421.814	-0.528	0.242	0.029	0.076	421.814	-0.431	0.241	0.073	0.362	421.814	0.097	0.279	0.729	0.859
SLC35C1	726.931	1.161	0.163	0.000	0.000	726.931	-0.010	0.165	0.951	0.976	726.931	-1.171	0.190	0.000	0.000
PLCXD1	262.817	0.288	0.230	0.211	0.340	262.817	0.106	0.230	0.644	0.806	262.817	-0.182	0.266	0.494	0.703
ZGRF1	79.409	-0.444	0.273	0.104	0.201	79.409	-0.330	0.270	0.221	0.500	79.409	0.114	0.317	0.719	0.854
TCP11L1	304.588	0.117	0.249	0.637	0.745	304.588	0.178	0.249	0.474	0.695	304.588	0.061	0.288	0.833	0.919
ABHD10	459.741	-0.499	0.132	0.000	0.001	459.741	-0.195	0.130	0.134	0.426	459.741	0.304	0.153	0.047	0.175
CHDH	8.693	0.723	0.569	0.204	0.331	8.693	0.690	0.562	0.220	0.499	8.693	-0.033	0.639	0.959	0.981
COPRS	586.994	0.372	0.341	0.276	0.413	586.994	-0.583	0.342	0.089	0.382	586.994	-0.955	0.395	0.016	0.088
LAPTM4B	2409.196	-0.412	0.233	0.078	0.162	2409.196	-0.438	0.233	0.060	0.350	2409.196	-0.026	0.270	0.922	0.963
HJURP	574.621	-0.372	0.178	0.037	0.092	574.621	0.087	0.177	0.624	0.793	574.621	0.459	0.206	0.026	0.120
SLC22A15	402.706	-0.142	0.191	0.457	0.592	402.706	0.025	0.190	0.897	0.948	402.706	0.167	0.221	0.450	0.669
TBC1D2	989.617	1.074	0.159	0.000	0.000	989.617	0.485	0.160	0.002	0.297	989.617	-0.589	0.184	0.001	0.016
PPP5C	2921.195	-0.344	0.104	0.001	0.006	2921.195	-0.137	0.104	0.188	0.474	2921.195	0.207	0.120	0.084	0.252
PI4K2A	918.050	0.296	0.139	0.033	0.084	918.050	-0.258	0.140	0.065	0.353	918.050	-0.554	0.161	0.001	0.009
TMEM63B	1104.179	0.134	0.239	0.575	0.694	1104.179	0.165	0.238	0.489	0.705	1104.179	0.031	0.275	0.909	0.959
IMPACT	727.466	-0.786	0.224	0.000	0.003	727.466	-0.064	0.222	0.773	0.879	727.466	0.722	0.258	0.005	0.042
LGR4	587.909	0.569	0.398	0.153	0.268	587.909	0.409	0.398	0.304	0.570	587.909	-0.160	0.460	0.728	0.859
PIDD1	173.872	0.840	0.272	0.002	0.010	173.872	0.493	0.273	0.071	0.360	173.872	-0.347	0.313	0.268	0.502
PPP6C	989.982	-0.103	0.117	0.378	0.518	989.982	-0.131	0.116	0.260	0.532	989.982	-0.028	0.135	0.835	0.920

PPP4R1L	109.209	0.199	0.302	0.510	0.639	109.209	0.449	0.299	0.133	0.424	109.209	0.250	0.347	0.471	0.685
TMCO6	209.946	0.007	0.167	0.968	0.980	209.946	0.328	0.164	0.046	0.334	209.946	0.321	0.192	0.094	0.269
LRRC59	4732.595	-1.099	0.456	0.016	0.048	4732.595	-1.324	0.456	0.004	0.297	4732.595	-0.225	0.526	0.669	0.824
PPT1	5151.303	0.225	0.138	0.104	0.201	5151.303	-0.164	0.138	0.236	0.513	5151.303	-0.389	0.160	0.015	0.085
MCM10	259.273	-1.372	0.549	0.012	0.040	259.273	-1.968	0.551	0.000	0.231	259.273	-0.596	0.637	0.349	0.583
JPX	610.905	-0.141	0.160	0.377	0.517	610.905	0.044	0.159	0.784	0.886	610.905	0.185	0.185	0.317	0.552
LOC554206	8.308	1.395	0.584	0.017	0.051	8.308	0.437	0.615	0.478	0.698	8.308	-0.958	0.675	0.156	0.368
NCBP3	365.031	-0.263	0.132	0.046	0.109	365.031	-0.172	0.130	0.187	0.474	365.031	0.091	0.153	0.551	0.745
ZNF331	32.669	1.184	0.361	0.001	0.006	32.669	0.122	0.374	0.745	0.864	32.669	-1.062	0.421	0.012	0.072
GPALPP1	334.390	-0.386	0.132	0.003	0.015	334.390	-0.015	0.129	0.910	0.955	334.390	0.371	0.152	0.014	0.083
FBXO48	26.575	-0.637	0.357	0.074	0.156	26.575	0.306	0.324	0.345	0.601	26.575	0.943	0.401	0.019	0.098
FAM72C	610.306	-0.819	0.205	0.000	0.001	610.306	-0.076	0.203	0.711	0.845	610.306	0.743	0.237	0.002	0.019
H4C15	459.001	-0.268	0.706	0.704	0.797	459.001	0.622	0.705	0.378	0.626	459.001	0.890	0.815	0.275	0.510
YOD1	233.368	-0.129	0.230	0.574	0.692	233.368	-0.204	0.229	0.373	0.622	233.368	-0.075	0.266	0.779	0.888
AP1AR	625.327	-1.021	0.232	0.000	0.000	625.327	-0.444	0.230	0.054	0.342	625.327	0.577	0.268	0.031	0.135
STRADB	297.866	-0.170	0.182	0.351	0.491	297.866	-0.156	0.181	0.390	0.635	297.866	0.014	0.211	0.947	0.975
DHRS4-AS1	546.567	-0.143	0.188	0.446	0.581	546.567	0.139	0.187	0.456	0.682	546.567	0.282	0.216	0.192	0.414
CAMK2N1	222.463	0.449	0.303	0.138	0.249	222.463	-0.154	0.305	0.612	0.786	222.463	-0.603	0.351	0.086	0.255
CSGALNACT2	488.451	-1.017	0.181	0.000	0.000	488.451	-0.032	0.177	0.857	0.927	488.451	0.985	0.208	0.000	0.000
PRCC	1485.814	-0.167	0.165	0.311	0.451	1485.814	-0.120	0.164	0.465	0.689	1485.814	0.047	0.190	0.806	0.903
PRCP	1582.556	0.365	0.407	0.371	0.511	1582.556	0.515	0.407	0.206	0.488	1582.556	0.151	0.470	0.749	0.871
NDUFAF7	269.670	-0.100	0.128	0.434	0.571	269.670	0.036	0.126	0.772	0.879	269.670	0.137	0.148	0.355	0.589
RBM12B-AS1	10.337	-0.379	0.756	0.617	0.727	10.337	0.196	0.731	0.788	0.889	10.337	0.575	0.866	0.507	0.713
PARL	156.165	0.446	0.289	0.123	0.228	156.165	0.107	0.290	0.713	0.846	156.165	-0.340	0.335	0.310	0.545
PREP	2034.739	-0.305	0.085	0.000	0.002	2034.739	-0.084	0.084	0.321	0.582	2034.739	0.222	0.098	0.024	0.113
ETNK1	636.635	-0.183	0.150	0.222	0.352	636.635	0.035	0.149	0.815	0.903	636.635	0.218	0.173	0.207	0.432
CHST12	559.498	0.734	0.233	0.002	0.008	559.498	0.445	0.233	0.056	0.344	559.498	-0.289	0.269	0.283	0.517
HES6	151.223	0.241	0.247	0.329	0.469	151.223	-0.357	0.250	0.153	0.445	151.223	-0.598	0.288	0.038	0.153
TNFRSF19	16.389	0.475	0.484	0.327	0.467	16.389	0.532	0.477	0.265	0.537	16.389	0.058	0.551	0.917	0.961
NOP10	693.181	0.150	0.286	0.601	0.715	693.181	-0.378	0.287	0.187	0.474	693.181	-0.528	0.331	0.111	0.298
MACROH2A2	10.143	0.027	0.584	0.963	0.978	10.143	0.083	0.572	0.885	0.943	10.143	0.056	0.673	0.934	0.970
SLC35E3	275.954	0.061	0.146	0.674	0.773	275.954	-0.067	0.146	0.646	0.807	275.954	-0.128	0.169	0.450	0.669
BATF3	74.262	0.162	0.573	0.777	0.852	74.262	-1.312	0.584	0.025	0.316	74.262	-1.474	0.672	0.028	0.126
DDX43	140.897	-0.097	0.216	0.652	0.756	140.897	0.044	0.213	0.836	0.916	140.897	0.141	0.249	0.570	0.759
SMPD3	14.999	1.622	0.603	0.007	0.026	14.999	0.956	0.612	0.118	0.409	14.999	-0.666	0.686	0.332	0.567
SRGN	1874.947	0.342	0.126	0.007	0.024	1874.947	0.197	0.126	0.118	0.409	1874.947	-0.146	0.145	0.316	0.552
ELAC1	40.697	-0.465	0.362	0.199	0.326	40.697	-0.176	0.354	0.619	0.790	40.697	0.289	0.419	0.490	0.700
TRIM36	84.633	-0.942	0.498	0.059	0.130	84.633	-1.124	0.499	0.024	0.316	84.633	-0.182	0.584	0.755	0.875
DHTKD1	452.964	0.032	0.264	0.902	0.937	452.964	0.316	0.263	0.230	0.507	452.964	0.284	0.305	0.352	0.585
FEM1A	707.709	-0.479	0.233	0.040	0.097	707.709	-0.353	0.233	0.130	0.422	707.709	0.127	0.270	0.639	0.804
PIP4P2	305.462	0.051	0.209	0.808	0.874	305.462	0.115	0.208	0.583	0.766	305.462	0.064	0.241	0.792	0.896
ELMOD1	35.907	-0.868	0.377	0.021	0.061	35.907	-0.250	0.361	0.490	0.706	35.907	0.619	0.434	0.154	0.364
MAML3	291.391	-1.038	0.201	0.000	0.000	291.391	0.046	0.194	0.814	0.902	291.391	1.084	0.231	0.000	0.000
CDCA7L	1212.801	-1.089	0.138	0.000	0.000	1212.801	-0.172	0.136	0.206	0.488	1212.801	0.918	0.159	0.000	0.000
PRH1	65.016	0.368	0.334	0.270	0.406	65.016	-0.168	0.339	0.620	0.791	65.016	-0.536	0.390	0.169	0.384
IL17RB	38.850	0.218	0.243	0.371	0.511	38.850	0.435	0.233	0.062	0.350	38.850	0.217	0.274	0.429	0.651
RBM38	370.389	1.668	0.203	0.000	0.000	370.389	0.139	0.207	0.504	0.715	370.389	-1.529	0.236	0.000	0.000
ZNF823	47.623	0.415	0.294	0.158	0.275	47.623	0.137	0.295	0.642	0.805	47.623	-0.277	0.340	0.415	0.640
ENOSF1	1201.676	0.076	0.155	0.624	0.734	1201.676	-0.299	0.155	0.054	0.342	1201.676	-0.375	0.180	0.037	0.150
PLXNA3	666.648	0.512	0.459	0.265	0.401	666.648	0.630	0.459	0.170	0.459	666.648	0.118	0.530	0.824	0.913
HAUS7	281.200	-0.279	0.440	0.525	0.652	281.200	-0.691	0.440	0.116	0.409	281.200	-0.412	0.509	0.418	0.643
CDC42BPG	34.508	0.348	0.369	0.346	0.486	34.508	0.230	0.368	0.532	0.732	34.508	-0.118	0.426	0.781	0.890
ZNF821	92.952	0.355	0.246	0.148	0.262	92.952	0.401	0.243	0.100	0.394	92.952	0.045	0.282	0.873	0.939
DNAH3	7.890	-0.093	0.597	0.876	0.919	7.890	-0.278	0.597	0.641	0.805	7.890	-0.185	0.702	0.793	0.896
GALNT10	1121.444	-0.236	0.342	0.489	0.622	1121.444	-0.624	0.342	0.068	0.356	1121.444	-0.388	0.395	0.325	0.560
PRIM1	417.952	-1.121	0.261	0.000	0.000	417.952	-0.485	0.259	0.061	0.350	417.952	0.636	0.301	0.035	0.145
CNOT11	572.057	-0.158	0.234	0.500	0.631	572.057	-0.465	0.234	0.047	0.336	572.057	-0.307	0.271	0.258	0.492
FOXRED1	370.630	-0.429	0.298	0.150	0.264	370.630	-0.778	0.298	0.009	0.297	370.630	-0.349	0.345	0.311	0.547
CDV3	5315.849	-0.706	0.217	0.001	0.006	5315.849	-0.325	0.217	0.133	0.425	5315.849	0.381	0.250	0.128	0.324
NAGK	300.015	0.896	0.170	0.000	0.000	300.015	0.126	0.174	0.470	0.692	300.015	-0.771	0.198	0.000	0.002
SUPT20H	745.864	-0.776	0.233	0.001	0.005	745.864	-0.586	0.233	0.012	0.305	745.864	0.190	0.270	0.482	0.694
PRIM2	371.359	-0.501	0.291	0.086	0.174	371.359	-0.444	0.291	0.127	0.419	371.359	0.057	0.337	0.866	0.936
KIF27	113.390	-0.511	0.251	0.042	0.101	113.390	0.158	0.243	0.516	0.721	113.390	0.669	0.288	0.020	0.102
UBE2Q1	1420.486	0.307	0.144	0.033	0.085	1420.486	0.120	0.144	0.407	0.648	1420.486	-0.187	0.167	0.261	0.495
MED29	551.879	0.283	0.140	0.044	0.104	551.879	-0.009	0.140	0.949	0.975	551.879	-0.292	0.162	0.072	0.229
BMP2K	355.667	-0.728	0.310	0.019	0.055	355.667	-0.715	0.310	0.021	0.313	355.667	0.013	0.359	0.972	0.986
VEZT	1137.907	-0.723	0.130	0.000	0.000	1137.907	-0.271	0.129	0.035	0.324	1137.907	0.452	0.150	0.003	0.026

GOLGA2P5	38.465	1.470	0.456	0.001	0.007	38.465	0.529	0.464	0.254	0.527	38.465	-0.941	0.526	0.074	0.232
OTUD5	1180.143	-0.154	0.096	0.106	0.204	1180.143	-0.038	0.095	0.689	0.832	1180.143	0.116	0.110	0.292	0.527
ZCCHC8	603.204	-0.517	0.257	0.045	0.106	603.204	-0.403	0.257	0.116	0.409	603.204	0.113	0.298	0.703	0.846
RNPC3	129.855	0.108	0.186	0.562	0.683	129.855	0.388	0.182	0.033	0.322	129.855	0.281	0.213	0.187	0.407
DDX60	607.420	1.131	0.382	0.003	0.013	607.420	0.506	0.382	0.185	0.472	607.420	-0.625	0.441	0.156	0.367
CDKN2AIP	267.456	-0.547	0.278	0.049	0.114	267.456	-0.521	0.277	0.061	0.350	267.456	0.027	0.323	0.934	0.970
TENT5A	148.064	1.563	0.284	0.000	0.000	148.064	0.388	0.293	0.185	0.472	148.064	-1.175	0.329	0.000	0.006
CARMIL1	15.271	0.373	0.392	0.341	0.482	15.271	0.169	0.393	0.667	0.819	15.271	-0.204	0.453	0.653	0.813
KIF21A	1746.693	-0.784	0.169	0.000	0.000	1746.693	-0.131	0.168	0.435	0.668	1746.693	0.653	0.195	0.001	0.011
PPP1R9A	56.429	-0.144	0.322	0.653	0.757	56.429	0.202	0.315	0.523	0.726	56.429	0.346	0.370	0.349	0.583
ANKRD10	469.870	0.685	0.379	0.071	0.151	469.870	0.701	0.379	0.065	0.353	469.870	0.015	0.437	0.972	0.986
ZNF280C	105.896	-0.620	0.282	0.028	0.074	105.896	-0.278	0.277	0.316	0.578	105.896	0.342	0.325	0.293	0.528
VPS50	152.476	-0.428	0.246	0.082	0.168	152.476	-0.467	0.245	0.056	0.344	152.476	-0.039	0.286	0.891	0.948
OTUB1	1443.833	0.278	0.154	0.072	0.153	1443.833	-0.195	0.155	0.208	0.490	1443.833	-0.473	0.179	0.008	0.057
FERMT1	402.335	-1.078	0.258	0.000	0.000	402.335	-0.390	0.256	0.127	0.420	402.335	0.689	0.298	0.021	0.103
KIF16B	359.551	-0.753	0.181	0.000	0.000	359.551	-0.090	0.178	0.611	0.786	359.551	0.663	0.209	0.002	0.018
PRR5	127.651	0.159	0.401	0.691	0.787	127.651	-0.414	0.404	0.306	0.572	127.651	-0.573	0.467	0.219	0.446
ASAP3	425.680	1.161	0.299	0.000	0.001	425.680	0.621	0.300	0.038	0.327	425.680	-0.540	0.345	0.118	0.307
TASP1	121.672	-0.888	0.197	0.000	0.000	121.672	-0.295	0.187	0.115	0.407	121.672	0.593	0.227	0.009	0.061
DOCK10	395.747	-0.430	0.326	0.187	0.311	395.747	-0.731	0.326	0.025	0.316	395.747	-0.301	0.377	0.426	0.649
PRKAA1	1539.320	-0.747	0.185	0.000	0.001	1539.320	-0.266	0.185	0.150	0.443	1539.320	0.481	0.214	0.025	0.116
STAP2	575.692	0.884	0.323	0.006	0.023	575.692	0.674	0.323	0.037	0.325	575.692	-0.210	0.373	0.572	0.760
TRMT1	881.112	0.107	0.213	0.614	0.725	881.112	-0.076	0.213	0.722	0.852	881.112	-0.183	0.246	0.457	0.675
TTC27	422.568	-0.978	0.239	0.000	0.000	422.568	-0.359	0.236	0.129	0.420	422.568	0.619	0.275	0.024	0.115
THUMPD1	842.907	-0.246	0.105	0.019	0.055	842.907	-0.225	0.104	0.031	0.322	842.907	0.021	0.122	0.864	0.935
POMGNT1	1322.885	-0.017	0.149	0.912	0.944	1322.885	0.049	0.148	0.740	0.861	1322.885	0.066	0.172	0.702	0.845
ZDHHC7	2496.912	0.096	0.147	0.515	0.643	2496.912	0.167	0.147	0.256	0.529	2496.912	0.071	0.170	0.675	0.828
AMBRA1	597.349	-0.043	0.121	0.723	0.812	597.349	-0.006	0.120	0.961	0.980	597.349	0.037	0.139	0.792	0.896
SMPD4	3445.842	0.513	0.190	0.007	0.026	3445.842	0.205	0.190	0.281	0.551	3445.842	-0.308	0.220	0.162	0.375
ZNF407	167.777	-0.877	0.223	0.000	0.001	167.777	-0.259	0.216	0.232	0.509	167.777	0.618	0.257	0.016	0.089
PNRC2	2684.717	-0.217	0.124	0.081	0.167	2684.717	-0.217	0.124	0.080	0.371	2684.717	0.000	0.144	1.000	1.000
SLC39A4	333.273	0.530	0.289	0.066	0.143	333.273	0.035	0.290	0.904	0.952	333.273	-0.495	0.334	0.138	0.341
LRRC40	540.455	-0.719	0.176	0.000	0.001	540.455	-0.385	0.175	0.028	0.321	540.455	0.334	0.204	0.102	0.284
G2E3	575.289	-0.794	0.187	0.000	0.000	575.289	-0.198	0.185	0.284	0.553	575.289	0.596	0.216	0.006	0.045
TBC1D22B	381.117	-0.296	0.156	0.058	0.129	381.117	-0.199	0.155	0.199	0.482	381.117	0.097	0.181	0.592	0.772
KRBOX4	187.247	0.102	0.188	0.587	0.703	187.247	0.125	0.186	0.503	0.715	187.247	0.023	0.217	0.916	0.961
DEPDC1	1110.399	-1.140	0.288	0.000	0.001	1110.399	-0.114	0.286	0.690	0.833	1110.399	1.025	0.332	0.002	0.022
CHD7	934.827	-0.662	0.226	0.003	0.015	934.827	-0.601	0.226	0.008	0.297	934.827	0.061	0.261	0.814	0.907
SYBU	185.418	-1.118	0.205	0.000	0.000	185.418	-0.289	0.197	0.142	0.433	185.418	0.828	0.236	0.000	0.007
PRKAB1	308.868	-0.544	0.267	0.041	0.100	308.868	-0.433	0.265	0.103	0.396	308.868	0.111	0.308	0.719	0.854
FLVCR2	430.611	-0.474	0.300	0.114	0.215	430.611	0.379	0.297	0.202	0.485	430.611	0.853	0.345	0.013	0.079
BTBD2	4506.696	0.382	0.368	0.300	0.438	4506.696	0.673	0.368	0.067	0.356	4506.696	0.291	0.425	0.493	0.702
OSGEP	204.627	-0.236	0.295	0.422	0.559	204.627	-0.645	0.296	0.029	0.322	204.627	-0.408	0.343	0.233	0.462
LYAR	922.518	-0.573	0.439	0.191	0.316	922.518	-1.074	0.439	0.014	0.306	922.518	-0.501	0.507	0.323	0.558
RAB20	408.734	0.684	0.481	0.155	0.270	408.734	0.651	0.480	0.175	0.464	408.734	-0.033	0.554	0.953	0.978
PRKAB2	551.663	-0.673	0.357	0.059	0.132	551.663	0.347	0.355	0.330	0.590	551.663	1.019	0.412	0.013	0.079
PIGV	408.691	-0.324	0.345	0.347	0.487	408.691	0.590	0.343	0.086	0.378	408.691	0.914	0.397	0.021	0.106
NHP2	2493.871	0.103	0.227	0.651	0.756	2493.871	-0.213	0.227	0.348	0.603	2493.871	-0.316	0.262	0.228	0.457
SLC48A1	515.399	0.419	0.240	0.080	0.166	515.399	-0.196	0.241	0.414	0.653	515.399	-0.615	0.277	0.026	0.121
BCAS4	101.153	0.611	0.184	0.001	0.005	101.153	0.399	0.185	0.031	0.322	101.153	-0.212	0.211	0.317	0.552
TMEM127	858.258	0.310	0.108	0.004	0.017	858.258	-0.040	0.108	0.715	0.847	858.258	-0.349	0.125	0.005	0.042
NLRP2	64.372	0.545	0.308	0.078	0.161	64.372	0.112	0.312	0.718	0.849	64.372	-0.432	0.358	0.227	0.455
INTS8	872.647	-0.313	0.216	0.149	0.263	872.647	-0.431	0.216	0.046	0.335	872.647	-0.118	0.250	0.637	0.803
ZNF692	362.400	0.766	0.215	0.000	0.003	362.400	0.524	0.215	0.015	0.306	362.400	-0.242	0.247	0.328	0.562
RNF126	1902.582	-0.172	0.269	0.523	0.651	1902.582	-0.352	0.269	0.191	0.477	1902.582	-0.180	0.311	0.562	0.753
ZNF416	72.557	-0.367	0.250	0.142	0.254	72.557	-0.131	0.244	0.591	0.772	72.557	0.236	0.289	0.414	0.640
PRKACA	717.283	0.847	0.130	0.000	0.000	717.283	0.016	0.131	0.905	0.952	717.283	-0.832	0.151	0.000	0.000
PRPF40A	1967.030	-0.538	0.193	0.005	0.021	1967.030	-0.433	0.193	0.025	0.316	1967.030	0.105	0.223	0.637	0.803
DDX27	890.334	-0.483	0.304	0.112	0.213	890.334	-0.630	0.304	0.038	0.327	890.334	-0.147	0.352	0.676	0.828
HIF1AN	1665.069	0.097	0.084	0.248	0.382	1665.069	-0.040	0.084	0.634	0.800	1665.069	-0.137	0.097	0.159	0.371
ZNF446	171.388	0.356	0.239	0.136	0.246	171.388	0.606	0.236	0.010	0.299	171.388	0.250	0.274	0.360	0.593
CDC37L1	220.159	-0.510	0.177	0.004	0.016	220.159	-0.172	0.173	0.318	0.580	220.159	0.337	0.204	0.099	0.278
URGCP	620.502	-0.003	0.106	0.980	0.988	620.502	-0.093	0.105	0.378	0.626	620.502	-0.090	0.122	0.462	0.679
NPLOC4	1576.806	-0.299	0.331	0.365	0.505	1576.806	-0.731	0.331	0.027	0.319	1576.806	-0.432	0.382	0.258	0.493
DENND4C	656.732	0.040	0.354	0.909	0.942	656.732	0.312	0.353	0.377	0.625	656.732	0.272	0.409	0.506	0.712
GPATCH2L	761.746	-0.333	0.281	0.236	0.369	761.746	-0.010	0.281	0.973	0.985	761.746	0.323	0.325	0.319	0.554
MFN1	645.985	-0.959	0.245	0.000	0.001	645.985	-0.434	0.243	0.075	0.364	645.985	0.525	0.283	0.063	0.211

PRKACB	320.668	0.221	0.304	0.469	0.603	320.668	0.430	0.303	0.156	0.448	320.668	0.210	0.351	0.550	0.744
PEX26	716.676	-0.229	0.245	0.350	0.490	716.676	-0.484	0.245	0.049	0.337	716.676	-0.255	0.284	0.369	0.601
PPP4R3A	1207.751	-0.849	0.234	0.000	0.002	1207.751	-0.418	0.233	0.073	0.362	1207.751	0.431	0.270	0.110	0.296
NBPF1	502.205	-0.673	0.176	0.000	0.001	502.205	-0.127	0.174	0.464	0.689	502.205	0.546	0.203	0.007	0.053
SLC30A6	355.053	-0.678	0.214	0.001	0.008	355.053	-0.347	0.211	0.100	0.394	355.053	0.331	0.247	0.181	0.399
IWS1	683.946	-0.452	0.276	0.102	0.198	683.946	-0.664	0.276	0.016	0.306	683.946	-0.212	0.320	0.506	0.713
RUFY2	1360.552	-0.323	0.116	0.005	0.021	1360.552	-0.136	0.115	0.236	0.514	1360.552	0.186	0.134	0.163	0.377
SCYL2	924.464	-0.880	0.247	0.000	0.003	924.464	-0.503	0.246	0.041	0.329	924.464	0.378	0.285	0.185	0.405
KANSL3	1792.072	-0.573	0.111	0.000	0.000	1792.072	-0.092	0.110	0.402	0.645	1792.072	0.481	0.128	0.000	0.004
RABL6	2275.597	0.099	0.200	0.619	0.730	2275.597	-0.232	0.200	0.246	0.521	2275.597	-0.332	0.231	0.151	0.360
MREG	140.373	0.656	0.243	0.007	0.026	140.373	-0.237	0.248	0.341	0.599	140.373	-0.892	0.284	0.002	0.019
TRMU	495.278	0.390	0.245	0.111	0.212	495.278	-0.294	0.246	0.233	0.510	495.278	-0.684	0.284	0.016	0.089
YEATS2	3282.255	-0.375	0.284	0.187	0.312	3282.255	0.324	0.284	0.255	0.528	3282.255	0.699	0.328	0.033	0.141
PKIA	16.505	0.363	0.382	0.342	0.482	16.505	-0.789	0.421	0.061	0.350	16.505	-1.152	0.475	0.015	0.086
PACS1	1509.155	0.340	0.139	0.014	0.044	1509.155	0.053	0.139	0.702	0.839	1509.155	-0.287	0.161	0.074	0.233
FRMD4A	623.739	-0.594	0.187	0.001	0.008	623.739	-0.257	0.186	0.167	0.456	623.739	0.337	0.216	0.119	0.309
LUC7L	439.325	0.190	0.419	0.651	0.756	439.325	-0.579	0.420	0.168	0.457	439.325	-0.769	0.485	0.113	0.301
KDM4D	18.063	0.012	0.417	0.977	0.986	18.063	-0.380	0.424	0.370	0.619	18.063	-0.392	0.493	0.426	0.649
NSUN5	927.860	-0.485	0.343	0.158	0.275	927.860	-0.716	0.343	0.037	0.325	927.860	-0.231	0.397	0.561	0.752
RBM22	792.432	-0.479	0.124	0.000	0.001	792.432	-0.076	0.122	0.535	0.734	792.432	0.403	0.143	0.005	0.039
VAC14	551.996	0.147	0.244	0.547	0.671	551.996	-0.256	0.244	0.296	0.563	551.996	-0.402	0.282	0.154	0.364
IARS2	3268.797	-0.558	0.143	0.000	0.001	3268.797	0.088	0.143	0.539	0.736	3268.797	0.646	0.165	0.000	0.002
PKIB	23.920	0.423	0.488	0.387	0.526	23.920	0.437	0.482	0.364	0.616	23.920	0.014	0.557	0.979	0.990
MAP7D1	3535.918	-0.399	0.272	0.142	0.254	3535.918	-0.502	0.272	0.065	0.354	3535.918	-0.103	0.314	0.744	0.868
ARHGEF40	276.174	1.262	0.264	0.000	0.000	276.174	0.629	0.266	0.018	0.306	276.174	-0.633	0.305	0.038	0.153
YJU2	356.818	0.191	0.257	0.457	0.592	356.818	-0.219	0.258	0.394	0.639	356.818	-0.411	0.297	0.168	0.382
POLR3B	335.595	-1.732	0.373	0.000	0.000	335.595	-0.953	0.370	0.010	0.298	335.595	0.779	0.431	0.070	0.226
CCDC88A	756.320	-0.062	0.210	0.769	0.847	756.320	-0.044	0.210	0.836	0.916	756.320	0.018	0.243	0.940	0.973
IPO9	1733.754	-0.271	0.192	0.158	0.275	1733.754	-0.377	0.192	0.049	0.337	1733.754	-0.107	0.221	0.629	0.798
NDC1	1383.264	-1.110	0.248	0.000	0.000	1383.264	-0.419	0.247	0.090	0.384	1383.264	0.691	0.286	0.016	0.088
NECAP2	930.121	0.429	0.115	0.000	0.002	930.121	-0.127	0.116	0.274	0.545	930.121	-0.556	0.134	0.000	0.001
KBTBD4	375.600	0.016	0.177	0.930	0.956	375.600	0.098	0.177	0.579	0.764	375.600	0.082	0.205	0.688	0.836
PRKAG1	1353.221	-0.009	0.103	0.929	0.955	1353.221	0.080	0.102	0.431	0.665	1353.221	0.090	0.118	0.449	0.669
FAR2	148.595	0.343	0.258	0.184	0.308	148.595	-0.220	0.261	0.398	0.642	148.595	-0.563	0.300	0.061	0.207
TENM3	223.613	-0.128	0.264	0.628	0.738	223.613	-0.237	0.264	0.369	0.619	223.613	-0.109	0.306	0.721	0.855
DOK4	157.153	0.911	0.283	0.001	0.007	157.153	0.465	0.284	0.102	0.394	157.153	-0.446	0.326	0.170	0.387
LMBR1L	274.136	0.614	0.226	0.006	0.024	274.136	0.545	0.225	0.016	0.306	274.136	-0.070	0.260	0.789	0.895
WDR11	858.792	-0.466	0.187	0.013	0.041	858.792	0.100	0.186	0.593	0.774	858.792	0.565	0.216	0.009	0.061
POLR3E	1293.329	-0.902	0.239	0.000	0.001	1293.329	-0.446	0.238	0.061	0.350	1293.329	0.456	0.276	0.098	0.277
SLF2	323.286	-0.174	0.227	0.442	0.578	323.286	-0.308	0.227	0.175	0.464	323.286	-0.133	0.263	0.612	0.786
TSR1	1520.968	-0.744	0.218	0.001	0.004	1520.968	-0.422	0.217	0.052	0.341	1520.968	0.322	0.252	0.201	0.425
IQCC	50.735	-0.346	0.235	0.141	0.253	50.735	-0.264	0.229	0.250	0.524	50.735	0.082	0.274	0.765	0.881
CEP72	311.884	-1.075	0.372	0.004	0.016	311.884	-0.828	0.370	0.025	0.316	311.884	0.247	0.430	0.566	0.756
ASF1B	511.020	0.384	0.297	0.195	0.321	511.020	-0.534	0.298	0.073	0.362	511.020	-0.918	0.343	0.007	0.054
INTS13	2268.881	-0.159	0.169	0.349	0.489	2268.881	0.167	0.169	0.324	0.584	2268.881	0.325	0.195	0.096	0.273
BTBD7	1005.754	-0.347	0.165	0.036	0.090	1005.754	-0.201	0.165	0.222	0.501	1005.754	0.146	0.191	0.445	0.665
N4BP2	202.043	-1.225	0.229	0.000	0.000	202.043	-0.283	0.220	0.200	0.483	202.043	0.942	0.263	0.000	0.006
ATF7IP	1221.119	-0.144	0.268	0.592	0.707	1221.119	0.141	0.267	0.599	0.777	1221.119	0.284	0.309	0.358	0.591
PRKAR1A	2704.397	-0.151	0.192	0.429	0.566	2704.397	0.019	0.191	0.921	0.960	2704.397	0.170	0.221	0.441	0.661
FAM222B	596.777	0.127	0.117	0.278	0.415	596.777	0.167	0.117	0.151	0.444	596.777	0.040	0.135	0.767	0.881
C1orf112	213.378	-0.617	0.280	0.028	0.074	213.378	-0.501	0.279	0.072	0.361	213.378	0.116	0.324	0.721	0.855
HHAT	36.398	1.089	0.598	0.068	0.147	36.398	0.895	0.598	0.134	0.426	36.398	-0.194	0.686	0.777	0.887
ZFP64	369.619	-0.598	0.194	0.002	0.010	369.619	0.120	0.190	0.528	0.729	369.619	0.718	0.223	0.001	0.016
DNAJC11	1065.161	-0.187	0.275	0.497	0.628	1065.161	-0.644	0.275	0.019	0.306	1065.161	-0.458	0.318	0.150	0.359
VPS35	2882.519	0.019	0.089	0.828	0.887	2882.519	-0.052	0.089	0.554	0.746	2882.519	-0.072	0.103	0.484	0.696
ARFGAP1	902.015	0.286	0.269	0.287	0.425	902.015	-0.408	0.269	0.130	0.422	902.015	-0.694	0.311	0.026	0.119
NAXD	577.495	-0.092	0.115	0.424	0.561	577.495	-0.001	0.114	0.994	0.997	577.495	0.091	0.133	0.493	0.702
ENAH	4627.879	-0.733	0.192	0.000	0.001	4627.879	-0.269	0.192	0.161	0.452	4627.879	0.464	0.222	0.037	0.150
EDEM2	383.504	0.358	0.143	0.012	0.040	383.504	0.193	0.143	0.178	0.467	383.504	-0.165	0.165	0.317	0.552
PARVA	540.250	0.487	0.246	0.048	0.112	540.250	0.204	0.246	0.407	0.648	540.250	-0.283	0.284	0.319	0.554
CHFR	148.034	0.352	0.202	0.081	0.167	148.034	-0.101	0.204	0.619	0.790	148.034	-0.453	0.235	0.053	0.191
COA1	505.558	-0.273	0.128	0.032	0.083	505.558	-0.253	0.127	0.046	0.335	505.558	0.020	0.148	0.891	0.948
AP5M1	465.468	-0.080	0.189	0.674	0.773	465.468	-0.540	0.190	0.005	0.297	465.468	-0.461	0.220	0.037	0.150
NUP133	929.533	-0.980	0.200	0.000	0.000	929.533	-0.260	0.199	0.192	0.477	929.533	0.721	0.231	0.002	0.020
CNDP2	1042.738	-0.405	0.232	0.080	0.165	1042.738	-0.330	0.231	0.154	0.447	1042.738	0.076	0.268	0.777	0.887
CCAR1	1253.093	0.050	0.194	0.799	0.868	1253.093	-0.322	0.194	0.097	0.392	1253.093	-0.372	0.225	0.098	0.277
PRKAR1B	1385.751	0.063	0.198	0.750	0.833	1385.751	-0.441	0.198	0.026	0.316	1385.751	-0.504	0.229	0.028	0.125

AGK	716.980	-0.014	0.174	0.934	0.958	716.980	-0.205	0.174	0.238	0.515	716.980	-0.191	0.201	0.343	0.577
TMEM184C	589.310	-0.543	0.159	0.001	0.004	589.310	-0.181	0.158	0.251	0.525	589.310	0.362	0.184	0.049	0.181
SEPTIN11	1269.519	-0.604	0.243	0.013	0.041	1269.519	-0.492	0.243	0.043	0.330	1269.519	0.112	0.281	0.689	0.836
TMEM30A	1957.639	-0.169	0.168	0.315	0.454	1957.639	-0.070	0.168	0.678	0.826	1957.639	0.099	0.194	0.609	0.784
CDK5RAP2	1118.545	-0.471	0.108	0.000	0.000	1118.545	-0.184	0.108	0.088	0.380	1118.545	0.287	0.125	0.022	0.107
INTS9	279.806	-0.388	0.226	0.086	0.174	279.806	-0.286	0.225	0.203	0.486	279.806	0.102	0.261	0.696	0.841
UGGT2	355.209	-0.121	0.151	0.423	0.560	355.209	-0.027	0.149	0.855	0.926	355.209	0.093	0.174	0.591	0.772
RCOR3	658.090	-0.343	0.154	0.026	0.070	658.090	0.079	0.152	0.601	0.779	658.090	0.423	0.177	0.017	0.092
WDR12	871.857	-0.635	0.236	0.007	0.026	871.857	-0.394	0.235	0.094	0.389	871.857	0.241	0.273	0.376	0.607
PRKAR2A	1392.536	-0.450	0.240	0.061	0.134	1392.536	-0.684	0.240	0.004	0.297	1392.536	-0.234	0.278	0.399	0.627
DHX32	959.645	-0.209	0.145	0.151	0.266	959.645	-0.238	0.145	0.101	0.394	959.645	-0.029	0.168	0.863	0.935
TTC17	1095.335	-0.210	0.143	0.142	0.253	1095.335	0.046	0.142	0.748	0.866	1095.335	0.256	0.165	0.121	0.313
ZNF701	33.406	-0.088	0.305	0.773	0.849	33.406	-0.027	0.299	0.928	0.964	33.406	0.061	0.352	0.863	0.935
EXOC1	513.198	-0.214	0.128	0.095	0.188	513.198	-0.012	0.126	0.923	0.961	513.198	0.202	0.148	0.172	0.389
IFT122	428.966	-0.029	0.196	0.882	0.923	428.966	0.253	0.195	0.193	0.478	428.966	0.282	0.226	0.211	0.436
INAVA	18.243	0.276	0.438	0.529	0.655	18.243	0.567	0.423	0.180	0.469	18.243	0.292	0.495	0.555	0.748
H2AJ	750.000	1.013	0.219	0.000	0.000	750.000	0.229	0.220	0.299	0.566	750.000	-0.785	0.254	0.002	0.021
NGLY1	1243.099	-0.740	0.221	0.001	0.005	1243.099	0.059	0.220	0.789	0.890	1243.099	0.799	0.255	0.002	0.019
ZNF83	67.412	0.036	0.247	0.885	0.925	67.412	-0.239	0.248	0.335	0.594	67.412	-0.275	0.289	0.341	0.576
EXOC2	673.926	-0.221	0.168	0.188	0.313	673.926	-0.352	0.168	0.036	0.324	673.926	-0.131	0.195	0.501	0.708
PRR11	1973.462	-0.669	0.273	0.014	0.044	1973.462	0.221	0.272	0.417	0.655	1973.462	0.890	0.315	0.005	0.039
TBC1D23	470.101	-0.268	0.229	0.242	0.375	470.101	0.056	0.227	0.805	0.898	470.101	0.324	0.264	0.220	0.446
TDP1	263.824	-0.506	0.215	0.018	0.054	263.824	-0.337	0.213	0.113	0.405	263.824	0.169	0.248	0.497	0.705
SAYSD1	98.757	0.457	0.195	0.019	0.056	98.757	0.110	0.197	0.576	0.761	98.757	-0.347	0.226	0.125	0.320
MBD5	199.117	-0.277	0.395	0.482	0.616	199.117	0.515	0.392	0.189	0.475	199.117	0.793	0.455	0.081	0.247
ZNF839	145.092	-0.126	0.218	0.563	0.683	145.092	0.123	0.215	0.565	0.753	145.092	0.249	0.251	0.320	0.555
CFAP44	65.004	1.510	0.442	0.001	0.004	65.004	0.493	0.448	0.271	0.542	65.004	-1.017	0.510	0.046	0.173
PRKCA	1630.961	-1.380	0.295	0.000	0.000	1630.961	0.187	0.294	0.525	0.728	1630.961	1.566	0.340	0.000	0.000
ERMARD	440.547	-0.392	0.208	0.059	0.131	440.547	-0.145	0.207	0.482	0.701	440.547	0.247	0.240	0.304	0.539
RIOK2	659.344	-0.843	0.205	0.000	0.000	659.344	-0.510	0.204	0.012	0.305	659.344	0.333	0.237	0.160	0.373
CMTR2	255.725	-1.086	0.336	0.001	0.007	255.725	-0.582	0.334	0.081	0.372	255.725	0.504	0.389	0.195	0.417
MCTP2	366.514	0.119	0.230	0.607	0.719	366.514	0.286	0.229	0.213	0.493	366.514	0.167	0.266	0.529	0.729
FGD6	388.049	-0.190	0.184	0.301	0.439	388.049	-0.207	0.184	0.259	0.531	388.049	-0.017	0.213	0.937	0.972
TXLNG	1077.475	-1.116	0.197	0.000	0.000	1077.475	-0.504	0.196	0.010	0.299	1077.475	0.611	0.228	0.007	0.053
LMBRD1	590.082	-0.110	0.329	0.738	0.823	590.082	0.296	0.328	0.367	0.617	590.082	0.406	0.380	0.284	0.520
DEPDC1B	593.405	-0.516	0.209	0.014	0.043	593.405	-0.189	0.208	0.363	0.615	593.405	0.327	0.241	0.176	0.393
LRIF1	317.183	0.023	0.164	0.888	0.928	317.183	-0.249	0.165	0.131	0.423	317.183	-0.272	0.191	0.155	0.365
MINDY1	207.390	-0.250	0.406	0.538	0.663	207.390	0.559	0.403	0.166	0.456	207.390	0.809	0.468	0.084	0.251
DDX28	232.237	-0.443	0.302	0.143	0.255	232.237	-0.519	0.302	0.085	0.377	232.237	-0.077	0.350	0.827	0.915
PCID2	592.756	-0.667	0.262	0.011	0.036	592.756	-0.472	0.261	0.071	0.360	592.756	0.195	0.303	0.520	0.723
METTL2B	608.625	-0.426	0.270	0.115	0.218	608.625	-0.573	0.270	0.034	0.322	608.625	-0.148	0.313	0.637	0.803
CACNA2D3	23.501	-0.120	0.443	0.786	0.858	23.501	0.532	0.425	0.211	0.492	23.501	0.652	0.503	0.195	0.417
AXL	2982.301	-0.336	0.287	0.242	0.376	2982.301	0.407	0.287	0.156	0.448	2982.301	0.743	0.331	0.025	0.117
PRKCD	932.874	0.040	0.238	0.867	0.914	932.874	0.371	0.238	0.119	0.412	932.874	0.331	0.275	0.229	0.458
DCP1A	707.954	-0.066	0.132	0.617	0.727	707.954	0.049	0.131	0.711	0.845	707.954	0.115	0.152	0.451	0.670
LRP2BP	22.630	0.534	0.375	0.154	0.270	22.630	-0.013	0.388	0.972	0.985	22.630	-0.548	0.441	0.214	0.440
HR	866.473	-0.230	0.656	0.726	0.814	866.473	0.623	0.656	0.342	0.599	866.473	0.853	0.758	0.260	0.494
TRERF1	884.549	-0.305	0.192	0.113	0.214	884.549	0.135	0.192	0.481	0.700	884.549	0.440	0.222	0.047	0.176
PRKCE	81.641	-0.250	0.256	0.329	0.470	81.641	-0.105	0.252	0.676	0.825	81.641	0.144	0.296	0.626	0.795
FOXJ2	651.002	-0.671	0.164	0.000	0.000	651.002	-0.038	0.161	0.812	0.901	651.002	0.632	0.189	0.001	0.011
SPATA7	130.960	-0.072	0.276	0.794	0.864	130.960	0.392	0.272	0.148	0.441	130.960	0.464	0.317	0.142	0.348
UTP6	1041.633	-0.770	0.316	0.015	0.046	1041.633	-0.624	0.316	0.048	0.337	1041.633	0.147	0.365	0.688	0.836
BDP1	3506.583	-0.840	0.247	0.001	0.004	3506.583	-0.368	0.247	0.136	0.427	3506.583	0.471	0.286	0.099	0.278
TSNAXIP1	150.215	-0.136	0.192	0.478	0.612	150.215	-0.079	0.190	0.676	0.825	150.215	0.057	0.222	0.798	0.899
KDM3A	2753.715	0.059	0.378	0.876	0.920	2753.715	0.465	0.377	0.218	0.498	2753.715	0.406	0.436	0.352	0.585
RNF130	812.703	0.446	0.246	0.070	0.149	812.703	0.471	0.246	0.056	0.343	812.703	0.024	0.284	0.932	0.969
VPS11	1030.423	0.486	0.174	0.005	0.021	1030.423	0.215	0.174	0.218	0.498	1030.423	-0.271	0.201	0.177	0.395
PAG1	706.396	0.968	0.333	0.004	0.015	706.396	0.261	0.333	0.434	0.667	706.396	-0.707	0.384	0.065	0.216
PECR	287.536	-0.059	0.272	0.828	0.887	287.536	0.419	0.270	0.121	0.412	287.536	0.479	0.313	0.127	0.322
DCAF6	2127.237	-0.042	0.229	0.854	0.905	2127.237	0.177	0.229	0.438	0.671	2127.237	0.219	0.264	0.406	0.633
SELENOS	740.066	-0.031	0.288	0.915	0.946	740.066	-0.454	0.289	0.116	0.409	740.066	-0.423	0.334	0.205	0.429
PRKCH	753.745	-0.730	0.221	0.001	0.006	753.745	-0.330	0.220	0.134	0.426	753.745	0.400	0.256	0.117	0.307
GLT8D1	828.742	-0.173	0.102	0.088	0.178	828.742	0.108	0.100	0.283	0.552	828.742	0.281	0.117	0.016	0.090
EMC3	1491.046	0.298	0.146	0.042	0.101	1491.046	0.177	0.146	0.225	0.504	1491.046	-0.120	0.169	0.476	0.689
CAND1	2769.355	-0.756	0.201	0.000	0.001	2769.355	-0.077	0.200	0.701	0.839	2769.355	0.680	0.232	0.003	0.031
UBAP2	753.652	-0.199	0.138	0.148	0.262	753.652	-0.266	0.138	0.053	0.342	753.652	-0.066	0.160	0.678	0.829
CENPJ	182.263	-0.405	0.217	0.062	0.136	182.263	-0.370	0.215	0.086	0.378	182.263	0.035	0.252	0.889	0.947

EAPP	426.541	-0.255	0.144	0.078	0.162	426.541	-0.166	0.143	0.246	0.521	426.541	0.088	0.167	0.596	0.775
CENPN	674.734	-0.564	0.211	0.007	0.027	674.734	-0.422	0.210	0.045	0.333	674.734	0.142	0.243	0.560	0.752
PRKCI	1910.349	-0.418	0.204	0.041	0.099	1910.349	-0.081	0.204	0.691	0.833	1910.349	0.337	0.236	0.153	0.363
EAF2	34.111	-0.099	0.343	0.773	0.849	34.111	-0.396	0.346	0.252	0.526	34.111	-0.297	0.403	0.461	0.678
WWC3	459.024	-0.142	0.163	0.385	0.524	459.024	-0.307	0.163	0.059	0.349	459.024	-0.166	0.189	0.381	0.610
PPP2R2D	569.207	-0.167	0.203	0.410	0.549	569.207	-0.305	0.203	0.133	0.424	569.207	-0.138	0.235	0.557	0.749
BRK1	4582.878	0.283	0.195	0.147	0.261	4582.878	0.299	0.195	0.125	0.417	4582.878	0.017	0.225	0.942	0.973
ITFG2	634.337	0.032	0.238	0.893	0.931	634.337	-0.157	0.238	0.511	0.719	634.337	-0.189	0.276	0.493	0.702
CISD1	519.914	-0.244	0.139	0.080	0.166	519.914	-0.175	0.138	0.207	0.489	519.914	0.069	0.161	0.668	0.824
PLGRKT	116.239	-0.147	0.289	0.612	0.724	116.239	-0.139	0.288	0.630	0.797	116.239	0.008	0.335	0.981	0.990
PKN1	5866.190	0.474	0.200	0.017	0.052	5866.190	0.463	0.199	0.020	0.310	5866.190	-0.011	0.230	0.962	0.982
USE1	408.043	0.621	0.229	0.007	0.025	408.043	0.324	0.229	0.158	0.450	408.043	-0.297	0.264	0.260	0.494
PSENEN	201.382	0.824	0.277	0.003	0.013	201.382	0.046	0.280	0.868	0.933	201.382	-0.777	0.321	0.015	0.087
TEX2	979.634	-0.529	0.228	0.020	0.058	979.634	-0.432	0.227	0.057	0.347	979.634	0.096	0.263	0.714	0.851
IDI2-AS1	97.480	1.577	0.389	0.000	0.001	97.480	-0.719	0.405	0.076	0.365	97.480	-2.295	0.459	0.000	0.000
ZC3H15	2027.814	-0.349	0.196	0.074	0.156	2027.814	-0.365	0.195	0.062	0.350	2027.814	-0.016	0.226	0.943	0.974
DENND10P1	89.952	0.032	0.252	0.900	0.935	89.952	0.141	0.249	0.572	0.758	89.952	0.109	0.291	0.708	0.849
ACOT13	778.647	0.550	0.266	0.038	0.095	778.647	-0.045	0.266	0.866	0.932	778.647	-0.595	0.307	0.053	0.189
KIZ	1233.883	-0.177	0.431	0.681	0.779	1233.883	0.576	0.431	0.181	0.469	1233.883	0.753	0.498	0.130	0.327
TMEM165	1389.810	0.551	0.170	0.001	0.007	1389.810	-0.127	0.171	0.455	0.682	1389.810	-0.678	0.197	0.001	0.009
BEX1	163.462	2.470	0.289	0.000	0.000	163.462	-0.578	0.304	0.057	0.347	163.462	-3.048	0.342	0.000	0.000
PKN2	1020.832	-0.694	0.197	0.000	0.003	1020.832	-0.235	0.196	0.230	0.508	1020.832	0.459	0.228	0.044	0.168
ACTR10	623.004	-0.218	0.157	0.163	0.282	623.004	-0.206	0.156	0.187	0.473	623.004	0.013	0.181	0.945	0.975
DBNDD2	77.267	0.376	0.229	0.101	0.198	77.267	0.486	0.226	0.031	0.322	77.267	0.111	0.262	0.672	0.826
ECHDC1	922.362	-0.747	0.242	0.002	0.010	922.362	-0.451	0.241	0.061	0.350	922.362	0.296	0.279	0.289	0.525
TMEM126B	486.617	0.169	0.274	0.538	0.663	486.617	-0.679	0.276	0.014	0.306	486.617	-0.848	0.318	0.008	0.055
HDAC8	401.370	0.508	0.179	0.005	0.019	401.370	-0.054	0.180	0.764	0.875	401.370	-0.562	0.208	0.007	0.051
PRKD1	253.225	-0.296	0.246	0.228	0.360	253.225	0.124	0.244	0.612	0.786	253.225	0.420	0.283	0.138	0.341
ASH1L	1150.595	-0.862	0.299	0.004	0.016	1150.595	0.010	0.298	0.973	0.985	1150.595	0.872	0.345	0.011	0.071
CBWD1	473.119	-0.130	0.190	0.496	0.627	473.119	-0.358	0.191	0.060	0.350	473.119	-0.229	0.221	0.301	0.536
PBK	814.250	-0.873	0.272	0.001	0.007	814.250	-0.345	0.272	0.204	0.487	814.250	0.528	0.315	0.093	0.268
GSDMB	33.283	2.656	0.467	0.000	0.000	33.283	1.012	0.484	0.037	0.324	33.283	-1.644	0.533	0.002	0.022
WSB2	2186.721	-0.297	0.100	0.003	0.014	2186.721	0.068	0.100	0.493	0.708	2186.721	0.365	0.116	0.002	0.019
PRKCSH	9494.204	0.554	0.244	0.023	0.064	9494.204	0.461	0.244	0.058	0.348	9494.204	-0.093	0.281	0.742	0.868
GPRC5C	12.451	0.248	0.598	0.678	0.776	12.451	0.596	0.580	0.304	0.570	12.451	0.348	0.678	0.608	0.783
MYNN	483.126	-0.871	0.214	0.000	0.001	483.126	-0.375	0.212	0.077	0.368	483.126	0.496	0.248	0.045	0.171
ZNF395	3521.077	0.312	0.730	0.670	0.770	3521.077	0.711	0.730	0.330	0.590	3521.077	0.399	0.843	0.636	0.802
UNC45A	1396.839	0.116	0.130	0.374	0.514	1396.839	0.154	0.130	0.237	0.514	1396.839	0.038	0.150	0.800	0.900
PRKCZ	98.462	0.549	0.296	0.063	0.138	98.462	0.042	0.300	0.890	0.945	98.462	-0.507	0.344	0.140	0.344
ZNF302	160.632	0.353	0.173	0.042	0.100	160.632	-0.070	0.175	0.690	0.832	160.632	-0.423	0.202	0.036	0.148
ACSS2	804.741	2.315	0.162	0.000	0.000	804.741	0.229	0.165	0.167	0.456	804.741	-2.086	0.188	0.000	0.000
KMT2E	855.287	0.125	0.182	0.493	0.625	855.287	0.010	0.182	0.958	0.978	855.287	-0.116	0.211	0.583	0.766
RNF114	1623.518	0.080	0.108	0.463	0.598	1623.518	0.042	0.108	0.695	0.836	1623.518	-0.037	0.125	0.767	0.881
ZC4H2	230.344	0.198	0.189	0.296	0.435	230.344	0.203	0.189	0.281	0.551	230.344	0.005	0.218	0.981	0.990
CMAS	874.294	0.217	0.158	0.168	0.288	874.294	-0.133	0.158	0.401	0.644	874.294	-0.350	0.183	0.055	0.195
ANGPTL8	31.372	-0.096	0.565	0.865	0.913	31.372	0.558	0.553	0.313	0.576	31.372	0.654	0.646	0.312	0.547
BIN3	292.235	0.495	0.174	0.004	0.018	292.235	-0.318	0.178	0.073	0.362	292.235	-0.814	0.204	0.000	0.002
PRKDC	6529.728	-1.181	0.263	0.000	0.000	6529.728	-0.543	0.263	0.039	0.327	6529.728	0.638	0.303	0.035	0.147
ERBIN	1459.854	-0.540	0.152	0.000	0.003	1459.854	-0.242	0.151	0.109	0.401	1459.854	0.297	0.175	0.089	0.262
LANCL2	199.178	-0.625	0.174	0.000	0.002	199.178	-0.376	0.171	0.028	0.321	199.178	0.250	0.202	0.217	0.444
NXT2	237.651	-0.428	0.207	0.038	0.095	237.651	-0.040	0.204	0.845	0.919	237.651	0.388	0.239	0.104	0.286
CTTNBP2NL	636.774	-0.847	0.240	0.000	0.003	636.774	-0.478	0.239	0.046	0.334	636.774	0.369	0.278	0.184	0.403
PRKG1	77.884	-0.904	0.582	0.121	0.225	77.884	0.661	0.571	0.247	0.522	77.884	1.564	0.668	0.019	0.099
RCC2	3103.057	-0.193	0.187	0.302	0.441	3103.057	-0.211	0.187	0.260	0.532	3103.057	-0.018	0.216	0.935	0.970
NKRF	715.322	-0.750	0.130	0.000	0.000	715.322	-0.101	0.127	0.427	0.663	715.322	0.649	0.150	0.000	0.001
INKA2	11.551	0.181	0.520	0.727	0.815	11.551	0.482	0.500	0.335	0.594	11.551	0.301	0.588	0.608	0.784
DMAP1	380.422	0.040	0.165	0.810	0.875	380.422	0.183	0.164	0.263	0.535	380.422	0.144	0.190	0.449	0.669
PRKG2	36.392	-1.255	0.471	0.008	0.028	36.392	-0.173	0.448	0.699	0.838	36.392	1.082	0.539	0.045	0.170
MYO5C	19.939	0.762	0.447	0.088	0.178	19.939	0.653	0.445	0.143	0.434	19.939	-0.109	0.507	0.829	0.916
APOM	1021.634	-0.966	0.324	0.003	0.013	1021.634	-0.039	0.323	0.903	0.951	1021.634	0.927	0.374	0.013	0.079
MAPK1	2543.288	-0.380	0.165	0.021	0.060	2543.288	-0.249	0.165	0.131	0.423	2543.288	0.131	0.191	0.492	0.702
MAPK3	701.507	0.739	0.428	0.084	0.172	701.507	0.664	0.428	0.121	0.413	701.507	-0.075	0.494	0.879	0.942
ZMAT5	248.232	0.618	0.263	0.019	0.055	248.232	0.407	0.263	0.122	0.413	248.232	-0.211	0.303	0.487	0.697
LIN37	185.760	0.314	0.242	0.196	0.322	185.760	-0.005	0.243	0.984	0.991	185.760	-0.318	0.281	0.257	0.491
KLHL9	99.860	-0.348	0.221	0.115	0.217	99.860	-0.292	0.218	0.181	0.469	99.860	0.057	0.257	0.826	0.914
AJAP1	165.759	-1.115	0.576	0.053	0.121	165.759	-2.125	0.581	0.000	0.211	165.759	-1.011	0.672	0.132	0.331
NDUFA12	857.161	0.531	0.244	0.029	0.077	857.161	-0.147	0.244	0.549	0.743	857.161	-0.677	0.282	0.016	0.090

NSFL1C	1163.022	0.190	0.151	0.206	0.334	1163.022	-0.128	0.151	0.394	0.639	1163.022	-0.319	0.174	0.067	0.219
RAB5IF	573.707	-0.197	0.442	0.656	0.760	573.707	-1.143	0.444	0.010	0.298	573.707	-0.946	0.512	0.065	0.214
MAPK6	1068.301	-1.147	0.271	0.000	0.000	1068.301	-0.577	0.270	0.033	0.322	1068.301	0.569	0.313	0.069	0.223
GNG12	3595.653	-0.992	0.235	0.000	0.000	3595.653	-0.376	0.235	0.109	0.401	3595.653	0.616	0.271	0.023	0.111
BAIAP2L1	915.626	-0.370	0.083	0.000	0.000	915.626	0.036	0.081	0.654	0.811	915.626	0.406	0.096	0.000	0.001
SLC25A40	219.516	-0.585	0.260	0.025	0.067	219.516	-0.167	0.257	0.516	0.721	219.516	0.417	0.300	0.164	0.379
BCAP29	406.618	-0.048	0.156	0.759	0.840	406.618	-0.371	0.157	0.018	0.306	406.618	-0.323	0.182	0.076	0.238
SLC50A1	512.278	0.580	0.149	0.000	0.001	512.278	0.274	0.149	0.066	0.354	512.278	-0.306	0.171	0.075	0.235
KLHL7	247.123	0.312	0.163	0.055	0.124	247.123	0.149	0.163	0.359	0.611	247.123	-0.163	0.188	0.385	0.614
MAPK7	212.133	0.866	0.281	0.002	0.010	212.133	0.336	0.283	0.236	0.513	212.133	-0.531	0.325	0.102	0.285
MAPK8	514.780	-0.696	0.221	0.002	0.008	514.780	-0.343	0.220	0.118	0.410	514.780	0.353	0.255	0.167	0.381
MAPK11	65.488	1.432	0.380	0.000	0.001	65.488	-0.370	0.401	0.356	0.610	65.488	-1.802	0.451	0.000	0.002
MYDGF	2998.781	0.072	0.242	0.767	0.846	2998.781	-0.159	0.242	0.511	0.719	2998.781	-0.231	0.280	0.409	0.635
SMG9	313.983	0.148	0.170	0.383	0.523	313.983	-0.042	0.170	0.803	0.897	313.983	-0.191	0.197	0.333	0.568
MAPK9	979.849	-0.425	0.203	0.037	0.091	979.849	-0.388	0.203	0.056	0.344	979.849	0.037	0.235	0.875	0.940
MAPK10	104.588	0.028	0.301	0.925	0.953	104.588	0.282	0.298	0.345	0.601	104.588	0.253	0.347	0.465	0.681
MAPK13	57.626	0.939	0.315	0.003	0.013	57.626	0.751	0.315	0.017	0.306	57.626	-0.188	0.358	0.600	0.778
PDGFC	221.360	-0.001	0.251	0.995	0.998	221.360	0.364	0.249	0.143	0.435	221.360	0.366	0.289	0.205	0.429
MAP2K1	2914.962	-0.177	0.170	0.298	0.436	2914.962	-0.120	0.170	0.480	0.699	2914.962	0.057	0.197	0.771	0.884
MAP2K2	5221.222	0.981	0.167	0.000	0.000	5221.222	0.238	0.167	0.154	0.446	5221.222	-0.743	0.193	0.000	0.003
ALG1	504.807	-0.141	0.302	0.642	0.748	504.807	-0.619	0.303	0.041	0.329	504.807	-0.478	0.350	0.172	0.389
MAP2K3	526.563	-0.063	0.245	0.797	0.866	526.563	-0.509	0.246	0.039	0.327	526.563	-0.445	0.284	0.117	0.307
UBFD1	1647.269	-0.410	0.196	0.036	0.091	1647.269	0.116	0.195	0.554	0.746	1647.269	0.526	0.226	0.020	0.102
TMEM234	94.068	0.687	0.178	0.000	0.001	94.068	0.008	0.186	0.967	0.982	94.068	-0.679	0.210	0.001	0.015
MAP2K5	525.391	-0.384	0.355	0.279	0.417	525.391	0.734	0.353	0.038	0.327	525.391	1.119	0.409	0.006	0.048
MAP2K7	1472.648	-0.348	0.076	0.000	0.000	1472.648	0.053	0.075	0.476	0.697	1472.648	0.401	0.088	0.000	0.000
PCDHGB7	50.666	0.327	0.564	0.562	0.683	50.666	0.379	0.562	0.501	0.713	50.666	0.051	0.650	0.937	0.972
EIF2AK2	789.136	-0.107	0.143	0.452	0.587	789.136	-0.102	0.142	0.475	0.697	789.136	0.006	0.165	0.972	0.986
PCDHGA10	12.773	1.554	0.553	0.005	0.020	12.773	0.171	0.585	0.770	0.878	12.773	-1.383	0.648	0.033	0.139
DNAJC3	1306.267	-0.391	0.184	0.033	0.085	1306.267	0.013	0.183	0.945	0.973	1306.267	0.403	0.212	0.057	0.199
PCDHGA1	8.558	1.067	0.589	0.070	0.150	8.558	0.050	0.623	0.936	0.968	8.558	-1.017	0.694	0.143	0.349
THAP12	1073.407	-0.838	0.205	0.000	0.001	1073.407	-0.324	0.204	0.113	0.405	1073.407	0.514	0.237	0.030	0.132
PCDHB15	9.594	1.734	0.566	0.002	0.010	9.594	1.171	0.582	0.044	0.332	9.594	-0.563	0.618	0.362	0.594
PCDHB14	66.446	0.857	0.528	0.104	0.202	66.446	0.322	0.530	0.544	0.739	66.446	-0.535	0.609	0.380	0.610
PCDHB13	12.420	1.432	0.581	0.014	0.043	12.420	0.605	0.598	0.312	0.575	12.420	-0.828	0.667	0.214	0.440
PCDHB12	18.385	1.174	0.406	0.004	0.016	18.385	0.188	0.427	0.661	0.815	18.385	-0.986	0.475	0.038	0.153
PCDHB11	9.458	0.320	0.854	0.708	0.800	9.458	0.742	0.837	0.375	0.623	9.458	0.422	0.972	0.664	0.821
PCDHB10	14.942	1.977	0.429	0.000	0.000	14.942	-0.124	0.500	0.804	0.897	14.942	-2.101	0.528	0.000	0.002
PCDHB9	20.973	1.591	0.340	0.000	0.000	20.973	0.365	0.370	0.324	0.584	20.973	-1.226	0.397	0.002	0.022
PCDHB8	10.138	1.471	0.870	0.091	0.182	10.138	-0.305	0.908	0.737	0.860	10.138	-1.775	1.025	0.083	0.250
PRKX	869.259	-0.189	0.209	0.366	0.506	869.259	0.192	0.209	0.357	0.611	869.259	0.382	0.242	0.114	0.303
PCDHB6	15.147	1.154	0.409	0.005	0.019	15.147	0.219	0.436	0.615	0.788	15.147	-0.935	0.479	0.051	0.186
PCDHB4	42.207	0.962	0.438	0.028	0.075	42.207	0.668	0.439	0.128	0.420	42.207	-0.293	0.503	0.560	0.751
PCDHB3	11.658	0.691	0.593	0.244	0.378	11.658	-0.084	0.615	0.891	0.945	11.658	-0.775	0.698	0.267	0.501
PCDHB2	171.419	0.731	0.252	0.004	0.016	171.419	0.244	0.253	0.336	0.594	171.419	-0.487	0.291	0.094	0.270
NSMCE3	676.085	0.842	0.237	0.000	0.003	676.085	-0.161	0.238	0.499	0.713	676.085	-1.003	0.274	0.000	0.005
GSDMC	51.652	0.616	0.396	0.119	0.223	51.652	-0.364	0.405	0.369	0.619	51.652	-0.980	0.464	0.035	0.144
DNAH7	13.349	-0.064	0.627	0.918	0.948	13.349	0.565	0.607	0.352	0.606	13.349	0.630	0.714	0.378	0.609
ANKH	2146.312	-0.387	0.102	0.000	0.001	2146.312	0.081	0.101	0.422	0.659	2146.312	0.469	0.118	0.000	0.002
MOSPD1	160.113	1.190	0.223	0.000	0.000	160.113	-0.219	0.232	0.346	0.602	160.113	-1.409	0.262	0.000	0.000
MTFR1L	735.442	-0.033	0.138	0.814	0.878	735.442	0.120	0.137	0.381	0.628	735.442	0.153	0.159	0.338	0.572
FAM214A	99.362	0.289	0.328	0.378	0.518	99.362	0.344	0.326	0.292	0.559	99.362	0.055	0.377	0.884	0.945
PRNP	5387.023	-0.738	0.182	0.000	0.001	5387.023	-0.352	0.181	0.052	0.341	5387.023	0.386	0.210	0.066	0.216
PSPN	12.667	2.242	0.784	0.004	0.017	12.667	1.289	0.805	0.109	0.401	12.667	-0.953	0.877	0.277	0.512
SUSD2	13.728	1.805	0.811	0.026	0.070	13.728	1.509	0.813	0.063	0.350	13.728	-0.295	0.919	0.748	0.871
KIAA1217	649.298	-0.156	0.283	0.581	0.699	649.298	0.484	0.282	0.086	0.379	649.298	0.639	0.326	0.050	0.182
MRAP	125.028	0.321	0.265	0.227	0.359	125.028	-0.121	0.267	0.651	0.810	125.028	-0.442	0.308	0.152	0.361
PRODH	17.821	1.317	1.015	0.194	0.320	17.821	1.121	1.015	0.269	0.541	17.821	-0.196	1.165	0.866	0.936
YLPM1	1069.522	-0.351	0.148	0.017	0.052	1069.522	-0.117	0.147	0.428	0.663	1069.522	0.234	0.170	0.169	0.385
RNF20	1255.511	-0.749	0.195	0.000	0.001	1255.511	0.038	0.194	0.846	0.920	1255.511	0.787	0.225	0.000	0.008
TMX4	738.337	-0.622	0.201	0.002	0.010	738.337	0.114	0.200	0.569	0.756	738.337	0.736	0.232	0.002	0.018
SERTAD4	53.766	1.626	0.233	0.000	0.000	53.766	0.182	0.251	0.468	0.691	53.766	-1.444	0.274	0.000	0.000
MEPCE	975.299	-0.543	0.136	0.000	0.001	975.299	0.165	0.134	0.217	0.497	975.299	0.708	0.156	0.000	0.000
CTNNBL1	726.854	0.086	0.175	0.622	0.732	726.854	-0.187	0.175	0.284	0.553	726.854	-0.273	0.202	0.176	0.394
C8orf44	38.093	0.826	0.366	0.024	0.066	38.093	0.508	0.368	0.168	0.457	38.093	-0.318	0.420	0.449	0.668
GPCPD1	257.855	-1.069	0.383	0.005	0.021	257.855	-0.961	0.382	0.012	0.305	257.855	0.108	0.444	0.808	0.904
LRRC8A	953.876	0.015	0.141	0.915	0.946	953.876	-0.180	0.141	0.201	0.485	953.876	-0.196	0.164	0.232	0.461

KYAT3	355.201	-0.396	0.209	0.057	0.128	355.201	0.296	0.205	0.150	0.442	355.201	0.692	0.240	0.004	0.035
PROS1	2286.223	1.152	0.296	0.000	0.001	2286.223	0.377	0.296	0.204	0.487	2286.223	-0.775	0.342	0.023	0.112
WDR45B	2714.428	0.202	0.205	0.326	0.466	2714.428	0.234	0.205	0.253	0.527	2714.428	0.033	0.237	0.890	0.947
PARD3	996.096	-0.961	0.135	0.000	0.000	996.096	0.026	0.133	0.843	0.918	996.096	0.987	0.156	0.000	0.000
AZGP1	22.236	-0.960	0.745	0.197	0.323	22.236	2.255	0.634	0.000	0.236	22.236	3.215	0.809	0.000	0.002
PRPS1	1303.207	-0.318	0.220	0.149	0.262	1303.207	-0.486	0.220	0.027	0.319	1303.207	-0.169	0.254	0.507	0.713
METTL3	593.224	-0.281	0.247	0.256	0.391	593.224	-0.335	0.247	0.175	0.464	593.224	-0.055	0.286	0.848	0.926
PRPS2	1231.389	-0.435	0.119	0.000	0.002	1231.389	0.052	0.118	0.656	0.812	1231.389	0.487	0.137	0.000	0.006
PPAN	415.456	0.116	0.185	0.531	0.656	415.456	0.237	0.184	0.197	0.481	415.456	0.121	0.213	0.569	0.758
PRPSAP1	548.617	0.174	0.103	0.092	0.184	548.617	0.133	0.103	0.194	0.478	548.617	-0.041	0.119	0.733	0.862
PRPSAP2	269.557	-0.371	0.128	0.004	0.015	269.557	-0.181	0.125	0.147	0.439	269.557	0.191	0.148	0.196	0.419
PRRG1	392.020	-0.348	0.145	0.016	0.049	392.020	-0.016	0.142	0.908	0.954	392.020	0.332	0.167	0.047	0.175
PRRG2	11.202	0.707	0.544	0.194	0.320	11.202	0.641	0.538	0.234	0.511	11.202	-0.066	0.611	0.914	0.960
LGMN	903.855	0.289	0.112	0.010	0.034	903.855	0.058	0.112	0.607	0.783	903.855	-0.231	0.130	0.075	0.235
LTB4R2	11.538	-0.256	0.546	0.639	0.746	11.538	-0.161	0.535	0.763	0.874	11.538	0.095	0.634	0.881	0.943
PRSS1	51.371	0.767	0.263	0.003	0.015	51.371	0.217	0.269	0.419	0.657	51.371	-0.550	0.305	0.071	0.228
PRSS2	118.301	0.419	0.276	0.129	0.236	118.301	0.171	0.276	0.537	0.735	118.301	-0.248	0.319	0.436	0.657
PRSS3	615.063	0.036	0.221	0.870	0.916	615.063	-0.010	0.220	0.964	0.981	615.063	-0.046	0.255	0.857	0.931
CTPS2	367.148	-0.168	0.233	0.472	0.606	367.148	0.119	0.232	0.608	0.783	367.148	0.287	0.269	0.286	0.521
EIF4ENIF1	364.618	-0.476	0.171	0.005	0.021	364.618	-0.302	0.170	0.075	0.364	364.618	0.174	0.198	0.382	0.611
KCNQ5	405.603	-1.007	0.253	0.000	0.001	405.603	-0.436	0.251	0.083	0.374	405.603	0.571	0.293	0.051	0.185
MASP1	46.376	0.418	0.666	0.531	0.656	46.376	0.622	0.664	0.349	0.604	46.376	0.204	0.767	0.790	0.895
TMPRSS15	5983.798	-1.010	0.251	0.000	0.001	5983.798	0.271	0.251	0.280	0.550	5983.798	1.282	0.290	0.000	0.000
PRSS8	31.477	0.832	0.398	0.036	0.091	31.477	0.491	0.401	0.220	0.500	31.477	-0.341	0.456	0.455	0.673
DNAJC12	228.048	0.249	0.213	0.243	0.376	228.048	-0.062	0.214	0.772	0.879	228.048	-0.311	0.247	0.208	0.433
KLK6	42.517	0.310	0.278	0.266	0.402	42.517	-0.084	0.283	0.768	0.877	42.517	-0.393	0.326	0.228	0.456
HTRA1	1580.342	1.255	0.242	0.000	0.000	1580.342	0.271	0.242	0.264	0.536	1580.342	-0.984	0.279	0.000	0.007
CHST7	373.564	-0.141	0.179	0.432	0.568	373.564	-0.053	0.178	0.767	0.877	373.564	0.088	0.207	0.670	0.824
KLK10	20.531	0.490	0.388	0.207	0.335	20.531	0.300	0.388	0.441	0.672	20.531	-0.190	0.446	0.669	0.824
PRTN3	335.746	1.365	1.158	0.238	0.372	335.746	1.219	1.158	0.292	0.560	335.746	-0.146	1.336	0.913	0.960
PSAP	40369.650	0.492	0.333	0.140	0.251	40369.650	0.477	0.333	0.153	0.445	40369.650	-0.015	0.385	0.969	0.985
CYP26B1	37.141	1.862	0.352	0.000	0.000	37.141	-0.046	0.382	0.905	0.952	37.141	-1.908	0.419	0.000	0.000
ERO1B	149.348	-0.011	0.290	0.969	0.981	149.348	0.419	0.286	0.143	0.435	149.348	0.430	0.333	0.196	0.419
SLC2A9	964.046	-0.951	0.183	0.000	0.000	964.046	0.079	0.181	0.665	0.818	964.046	1.029	0.211	0.000	0.000
DIABLO	222.625	-0.227	0.227	0.317	0.457	222.625	-0.220	0.226	0.332	0.591	222.625	0.008	0.263	0.977	0.988
PSD	127.770	-0.174	0.211	0.408	0.546	127.770	-0.195	0.209	0.351	0.605	127.770	-0.020	0.245	0.933	0.970
INPP5E	254.914	0.309	0.172	0.072	0.152	254.914	0.122	0.172	0.476	0.697	254.914	-0.187	0.198	0.347	0.581
ASAH2	91.741	-1.323	0.371	0.000	0.003	91.741	-0.853	0.364	0.019	0.306	91.741	0.469	0.430	0.275	0.510
PSEN1	1093.391	-0.364	0.169	0.031	0.081	1093.391	-0.315	0.168	0.061	0.350	1093.391	0.049	0.195	0.803	0.902
PSEN2	246.262	0.626	0.308	0.042	0.101	246.262	-0.537	0.312	0.085	0.376	246.262	-1.164	0.358	0.001	0.015
BCCIP	1524.075	-0.373	0.329	0.258	0.392	1524.075	-0.771	0.329	0.019	0.306	1524.075	-0.398	0.380	0.295	0.530
EIF5A2	335.234	-0.590	0.412	0.152	0.268	335.234	-1.045	0.413	0.011	0.305	335.234	-0.455	0.477	0.340	0.575
CLDND1	1594.398	-0.063	0.213	0.768	0.847	1594.398	0.257	0.213	0.227	0.506	1594.398	0.320	0.246	0.194	0.416
LINC00470	20.016	0.211	0.334	0.528	0.654	20.016	-0.453	0.352	0.198	0.481	20.016	-0.664	0.403	0.100	0.280
TWNK	1175.521	-0.782	0.206	0.000	0.001	1175.521	-0.344	0.205	0.094	0.388	1175.521	0.438	0.238	0.065	0.216
NPDC1	1138.282	1.166	0.268	0.000	0.000	1138.282	0.450	0.269	0.094	0.388	1138.282	-0.716	0.310	0.021	0.104
POLE4	461.601	0.367	0.186	0.048	0.112	461.601	-0.059	0.187	0.753	0.868	461.601	-0.426	0.215	0.048	0.177
TRIM39	1195.528	-0.749	0.200	0.000	0.001	1195.528	-0.350	0.199	0.079	0.370	1195.528	0.399	0.231	0.084	0.252
PANX2	144.648	1.609	0.362	0.000	0.000	144.648	-0.749	0.383	0.050	0.338	144.648	-2.358	0.432	0.000	0.000
MUC13	11.788	0.543	0.560	0.332	0.472	11.788	0.638	0.548	0.244	0.519	11.788	0.095	0.631	0.880	0.943
AKIP1	487.934	0.298	0.185	0.107	0.206	487.934	0.122	0.185	0.510	0.718	487.934	-0.176	0.214	0.410	0.636
TMEM9B	433.404	-0.198	0.234	0.397	0.536	433.404	-0.416	0.234	0.075	0.364	433.404	-0.218	0.271	0.421	0.645
NRIP3	84.487	0.300	0.262	0.253	0.387	84.487	-0.284	0.267	0.287	0.556	84.487	-0.584	0.307	0.057	0.199
SAR1A	2379.338	0.518	0.108	0.000	0.000	2379.338	0.038	0.108	0.723	0.852	2379.338	-0.480	0.125	0.000	0.003
CFAP298	424.609	0.083	0.285	0.772	0.848	424.609	-0.395	0.286	0.167	0.457	424.609	-0.478	0.330	0.148	0.356
B2M	29433.516	0.675	0.204	0.001	0.006	29433.516	0.236	0.204	0.249	0.523	29433.516	-0.439	0.236	0.062	0.210
JPH1	495.010	-0.696	0.248	0.005	0.020	495.010	-0.414	0.247	0.094	0.388	495.010	0.282	0.287	0.325	0.559
PSG4	180.888	3.621	0.344	0.000	0.000	180.888	-0.362	0.364	0.321	0.582	180.888	-3.983	0.407	0.000	0.000
SLC2A4RG	1680.049	0.850	0.229	0.000	0.002	1680.049	0.192	0.230	0.402	0.645	1680.049	-0.658	0.265	0.013	0.078
FMN2	699.456	0.062	0.211	0.769	0.847	699.456	-0.178	0.211	0.399	0.642	699.456	-0.240	0.244	0.325	0.560
PSKH1	446.328	-0.123	0.189	0.515	0.643	446.328	0.110	0.187	0.558	0.748	446.328	0.232	0.218	0.285	0.520
PSMA1	3063.539	0.258	0.158	0.103	0.199	3063.539	-0.279	0.158	0.078	0.369	3063.539	-0.537	0.183	0.003	0.031
ZC3HAV1	1139.737	1.542	0.320	0.000	0.000	1139.737	0.480	0.321	0.135	0.426	1139.737	-1.062	0.370	0.004	0.036
PSMA2	115.767	0.560	0.313	0.073	0.154	115.767	-0.262	0.318	0.409	0.649	115.767	-0.823	0.365	0.024	0.114
SLAMF8	117.100	2.818	0.414	0.000	0.000	117.100	1.062	0.440	0.016	0.306	117.100	-1.756	0.466	0.000	0.003
GPR137	1163.262	0.843	0.223	0.000	0.001	1163.262	0.347	0.224	0.121	0.413	1163.262	-0.496	0.258	0.054	0.193
PSMA3	1305.481	0.099	0.329	0.763	0.843	1305.481	-0.665	0.330	0.044	0.331	1305.481	-0.764	0.381	0.045	0.170

SPHK2	318.638	-0.120	0.177	0.496	0.627	318.638	-0.189	0.176	0.283	0.553	318.638	-0.069	0.205	0.737	0.865
PSMA4	2650.777	0.084	0.291	0.772	0.848	2650.777	-0.696	0.291	0.017	0.306	2650.777	-0.781	0.336	0.020	0.102
GRIPAP1	503.062	0.042	0.118	0.719	0.808	503.062	-0.054	0.117	0.644	0.806	503.062	-0.097	0.136	0.479	0.692
EMC7	1026.411	0.022	0.236	0.924	0.952	1026.411	-0.296	0.236	0.210	0.491	1026.411	-0.318	0.272	0.243	0.474
RAD18	421.849	-1.249	0.310	0.000	0.001	421.849	-0.879	0.309	0.004	0.297	421.849	0.370	0.359	0.302	0.537
PSMA5	1798.566	0.001	0.307	0.998	0.999	1798.566	-0.623	0.307	0.042	0.330	1798.566	-0.624	0.355	0.078	0.242
PSMA6	2303.643	-0.195	0.160	0.222	0.353	2303.643	-0.029	0.160	0.855	0.926	2303.643	0.166	0.185	0.368	0.600
PSMA7	4299.421	0.042	0.266	0.876	0.920	4299.421	-0.478	0.266	0.072	0.361	4299.421	-0.519	0.307	0.091	0.264
CDC42SE1	1437.984	-0.265	0.223	0.234	0.367	1437.984	-0.449	0.223	0.044	0.332	1437.984	-0.183	0.258	0.477	0.690
UGGT1	1286.679	-0.405	0.247	0.101	0.197	1286.679	-0.390	0.247	0.114	0.405	1286.679	0.015	0.285	0.959	0.981
KCMF1	5544.793	-0.475	0.157	0.002	0.011	5544.793	0.032	0.157	0.837	0.916	5544.793	0.507	0.181	0.005	0.041
TM9SF3	3867.426	-0.457	0.157	0.004	0.016	3867.426	-0.223	0.157	0.156	0.448	3867.426	0.234	0.182	0.199	0.422
PSMB1	2441.767	0.285	0.199	0.152	0.267	2441.767	-0.324	0.199	0.104	0.396	2441.767	-0.609	0.230	0.008	0.057
MDM1	102.043	-0.515	0.245	0.036	0.090	102.043	-0.604	0.244	0.013	0.306	102.043	-0.089	0.287	0.755	0.875
UBQLN4	1245.231	-0.552	0.212	0.009	0.032	1245.231	-0.280	0.211	0.185	0.472	1245.231	0.273	0.244	0.265	0.499
AGPAT3	2409.696	-0.141	0.112	0.206	0.334	2409.696	-0.015	0.112	0.892	0.946	2409.696	0.126	0.129	0.329	0.563
AGPAT4	1020.754	0.215	0.267	0.420	0.557	1020.754	0.185	0.267	0.489	0.705	1020.754	-0.031	0.308	0.921	0.963
WRNIP1	1855.042	0.120	0.116	0.303	0.441	1855.042	0.033	0.116	0.779	0.883	1855.042	-0.087	0.134	0.516	0.720
BDH2	278.092	0.817	0.274	0.003	0.013	278.092	0.454	0.274	0.098	0.393	278.092	-0.363	0.316	0.251	0.483
ANKS1B	337.689	-0.154	0.355	0.664	0.766	337.689	0.475	0.353	0.178	0.467	337.689	0.629	0.409	0.124	0.317
PSMB2	2172.283	-0.050	0.256	0.846	0.899	2172.283	-0.470	0.256	0.066	0.354	2172.283	-0.420	0.296	0.155	0.366
TMEM167B	831.347	-0.289	0.189	0.125	0.231	831.347	0.152	0.188	0.418	0.656	831.347	0.441	0.218	0.043	0.165
NDUFA4L2	121.904	-0.231	0.335	0.491	0.623	121.904	0.927	0.326	0.004	0.297	121.904	1.158	0.382	0.002	0.025
PNO1	514.904	-1.167	0.457	0.011	0.036	514.904	-1.350	0.457	0.003	0.297	514.904	-0.182	0.528	0.730	0.860
SH3GLB2	879.296	0.025	0.186	0.892	0.930	879.296	0.139	0.185	0.452	0.680	879.296	0.114	0.214	0.594	0.774
C15orf39	649.796	0.071	0.112	0.523	0.650	649.796	0.073	0.111	0.510	0.718	649.796	0.002	0.129	0.989	0.994
THAP10	71.251	-0.465	0.256	0.070	0.149	71.251	-0.166	0.248	0.502	0.714	71.251	0.298	0.296	0.313	0.549
SPIRE1	1329.404	-1.027	0.203	0.000	0.000	1329.404	-0.347	0.202	0.086	0.378	1329.404	0.680	0.234	0.004	0.033
PSMB3	3328.308	0.613	0.336	0.068	0.146	3328.308	-0.488	0.336	0.147	0.438	3328.308	-1.101	0.388	0.005	0.038
STARD7	2181.978	-0.655	0.220	0.003	0.013	2181.978	-0.324	0.220	0.141	0.432	2181.978	0.331	0.254	0.193	0.415
MAP3K7CL	24.918	1.575	0.484	0.001	0.006	24.918	0.727	0.495	0.142	0.433	24.918	-0.849	0.554	0.126	0.320
IFT46	280.419	-0.275	0.166	0.098	0.194	280.419	0.004	0.164	0.980	0.989	280.419	0.279	0.192	0.146	0.353
C1GALT1	1470.445	0.342	0.153	0.025	0.069	1470.445	-0.166	0.154	0.279	0.549	1470.445	-0.509	0.177	0.004	0.036
EXOSC5	1230.392	0.236	0.185	0.202	0.329	1230.392	0.365	0.185	0.048	0.337	1230.392	0.129	0.214	0.546	0.741
SMARCAD1	444.261	-0.704	0.189	0.000	0.002	444.261	-0.223	0.187	0.232	0.509	444.261	0.481	0.218	0.028	0.125
MEIS3	236.684	1.857	0.389	0.000	0.000	236.684	0.782	0.391	0.046	0.334	236.684	-1.075	0.448	0.016	0.090
DHX33	641.111	-0.976	0.196	0.000	0.000	641.111	-0.370	0.194	0.056	0.344	641.111	0.606	0.226	0.007	0.054
PSMB4	3897.586	0.434	0.190	0.022	0.062	3897.586	0.042	0.190	0.827	0.910	3897.586	-0.393	0.219	0.073	0.231
MCCC1	1006.813	-0.496	0.300	0.099	0.194	1006.813	0.450	0.299	0.132	0.424	1006.813	0.946	0.346	0.006	0.048
LXN	64.906	-1.780	0.470	0.000	0.001	64.906	-1.176	0.459	0.010	0.299	64.906	0.604	0.545	0.268	0.502
NCLN	2633.113	-0.181	0.294	0.539	0.663	2633.113	-0.595	0.294	0.043	0.331	2633.113	-0.414	0.340	0.223	0.450
GPR108	1450.785	0.221	0.166	0.184	0.308	1450.785	0.351	0.166	0.034	0.322	1450.785	0.130	0.192	0.497	0.705
SPPL2B	657.368	0.618	0.134	0.000	0.000	657.368	0.260	0.135	0.054	0.342	657.368	-0.358	0.155	0.021	0.103
FEM1C	982.302	-0.092	0.290	0.752	0.834	982.302	0.360	0.289	0.214	0.493	982.302	0.452	0.335	0.177	0.394
PSMB5	1636.486	-0.233	0.171	0.174	0.295	1636.486	-0.241	0.171	0.159	0.451	1636.486	-0.008	0.198	0.968	0.985
DUS3L	874.811	-0.521	0.231	0.024	0.067	874.811	-0.312	0.231	0.176	0.464	874.811	0.209	0.267	0.434	0.656
SMCO4	100.356	0.981	0.278	0.000	0.003	100.356	-0.030	0.285	0.917	0.959	100.356	-1.011	0.324	0.002	0.020
PMEPA1	347.123	0.091	0.167	0.587	0.703	347.123	-0.167	0.168	0.320	0.581	347.123	-0.257	0.194	0.185	0.404
ARNTL2	262.245	-0.354	0.495	0.475	0.609	262.245	-1.211	0.497	0.015	0.306	262.245	-0.857	0.574	0.136	0.337
PSMB6	684.830	0.241	0.238	0.313	0.452	684.830	-0.255	0.239	0.285	0.554	684.830	-0.496	0.276	0.072	0.229
DUSP22	256.896	0.619	0.121	0.000	0.000	256.896	0.082	0.124	0.506	0.716	256.896	-0.536	0.141	0.000	0.003
HMCES	1073.354	0.088	0.120	0.465	0.600	1073.354	-0.033	0.120	0.782	0.886	1073.354	-0.121	0.139	0.384	0.613
CMC2	235.110	0.164	0.314	0.602	0.715	235.110	-0.419	0.316	0.185	0.472	235.110	-0.582	0.364	0.110	0.296
ENY2	1301.971	0.436	0.174	0.012	0.040	1301.971	-0.111	0.175	0.526	0.729	1301.971	-0.547	0.202	0.007	0.050
OLFML3	10.167	1.075	0.467	0.021	0.060	10.167	1.276	0.453	0.005	0.297	10.167	0.201	0.505	0.691	0.837
MRPS22	973.081	-0.166	0.199	0.405	0.543	973.081	-0.189	0.199	0.342	0.599	973.081	-0.023	0.230	0.919	0.962
EMSY	310.428	-0.664	0.205	0.001	0.007	310.428	-0.623	0.204	0.002	0.297	310.428	0.041	0.238	0.864	0.935
MFF	974.579	0.170	0.137	0.216	0.346	974.579	0.106	0.137	0.440	0.672	974.579	-0.064	0.159	0.688	0.836
SDR39U1	662.254	0.338	0.135	0.012	0.040	662.254	0.037	0.135	0.787	0.888	662.254	-0.302	0.156	0.054	0.191
XAB2	1621.179	0.150	0.142	0.292	0.430	1621.179	0.226	0.142	0.111	0.404	1621.179	0.076	0.164	0.641	0.805
PSMB7	4107.063	0.225	0.177	0.204	0.331	4107.063	-0.120	0.177	0.496	0.711	4107.063	-0.345	0.204	0.091	0.265
SMYD2	606.820	-0.294	0.300	0.328	0.468	606.820	0.392	0.299	0.189	0.475	606.820	0.686	0.346	0.047	0.176
C5orf15	960.583	0.294	0.190	0.121	0.225	960.583	0.096	0.190	0.614	0.787	960.583	-0.198	0.219	0.365	0.597
PRTFDC1	21.257	0.688	0.360	0.056	0.126	21.257	0.225	0.369	0.542	0.738	21.257	-0.462	0.418	0.269	0.503
NT5M	23.728	1.183	0.436	0.007	0.025	23.728	-0.020	0.457	0.965	0.982	23.728	-1.203	0.513	0.019	0.099
NIT2	706.494	0.386	0.158	0.015	0.045	706.494	0.133	0.158	0.402	0.644	706.494	-0.254	0.183	0.165	0.380
LHX9	19.595	1.330	0.445	0.003	0.013	19.595	0.687	0.454	0.130	0.422	19.595	-0.642	0.508	0.206	0.430

OTUD7B	715.888	-0.690	0.264	0.009	0.032	715.888	-0.431	0.264	0.102	0.394	715.888	0.259	0.306	0.398	0.625
PSMB8	8400.001	0.468	0.217	0.031	0.081	8400.001	0.146	0.217	0.500	0.713	8400.001	-0.322	0.251	0.200	0.423
PARP6	499.244	0.394	0.273	0.149	0.263	499.244	0.609	0.272	0.025	0.316	499.244	0.215	0.314	0.495	0.703
C14orf132	2226.121	0.051	0.530	0.923	0.951	2226.121	0.556	0.529	0.293	0.561	2226.121	0.505	0.611	0.408	0.635
ATXN7L3	1149.482	-0.145	0.118	0.220	0.351	1149.482	-0.140	0.118	0.237	0.514	1149.482	0.005	0.137	0.969	0.985
CEACAM19	60.879	2.705	0.527	0.000	0.000	60.879	1.110	0.538	0.039	0.327	60.879	-1.594	0.602	0.008	0.057
FAM20C	20.730	1.600	0.486	0.001	0.006	20.730	0.223	0.515	0.665	0.818	20.730	-1.377	0.568	0.015	0.087
STOX2	15.179	0.607	0.609	0.319	0.459	15.179	0.450	0.608	0.460	0.686	15.179	-0.157	0.699	0.823	0.913
PRDM8	7.283	-0.509	0.648	0.432	0.568	7.283	0.369	0.579	0.524	0.727	7.283	0.878	0.724	0.225	0.453
PSMB9	5331.655	0.511	0.275	0.063	0.138	5331.655	0.392	0.275	0.154	0.446	5331.655	-0.119	0.318	0.708	0.848
PRDM10	181.102	-0.584	0.255	0.022	0.062	181.102	-0.242	0.252	0.336	0.594	181.102	0.342	0.294	0.246	0.477
PRDM11	176.513	-0.259	0.268	0.333	0.473	176.513	0.202	0.264	0.445	0.676	176.513	0.461	0.308	0.135	0.335
POGLUT1	371.084	-0.172	0.150	0.252	0.386	371.084	-0.112	0.149	0.452	0.680	371.084	0.060	0.174	0.730	0.860
PSMG2	660.988	0.123	0.182	0.497	0.628	660.988	-0.103	0.182	0.572	0.758	660.988	-0.226	0.210	0.282	0.516
ADPRM	138.760	-0.166	0.188	0.376	0.516	138.760	0.217	0.182	0.232	0.509	138.760	0.384	0.215	0.074	0.234
DTWD1	370.370	-0.096	0.131	0.464	0.598	370.370	-0.038	0.130	0.771	0.878	370.370	0.058	0.151	0.701	0.844
BBX	2688.429	-0.447	0.195	0.022	0.062	2688.429	-0.069	0.195	0.724	0.852	2688.429	0.378	0.225	0.093	0.268
PSMB10	284.569	1.113	0.223	0.000	0.000	284.569	0.107	0.226	0.635	0.801	284.569	-1.006	0.258	0.000	0.002
CDC42SE2	919.990	-0.590	0.180	0.001	0.006	919.990	0.088	0.178	0.620	0.791	919.990	0.679	0.207	0.001	0.014
KIF15	512.335	-0.739	0.125	0.000	0.000	512.335	-0.206	0.122	0.092	0.387	512.335	0.533	0.144	0.000	0.004
TOMM22	1078.235	-0.490	0.235	0.037	0.091	1078.235	-0.427	0.234	0.068	0.357	1078.235	0.063	0.271	0.817	0.909
CHPT1	1532.500	-0.122	0.124	0.325	0.465	1532.500	0.131	0.123	0.285	0.554	1532.500	0.253	0.143	0.076	0.237
TULP4	423.198	-0.305	0.218	0.163	0.281	423.198	-0.236	0.218	0.278	0.548	423.198	0.069	0.253	0.785	0.892
SLC12A9	342.323	0.645	0.126	0.000	0.000	342.323	0.048	0.128	0.710	0.844	342.323	-0.597	0.147	0.000	0.001
COQ8A	1193.799	-0.169	0.281	0.547	0.671	1193.799	0.627	0.280	0.025	0.316	1193.799	0.796	0.324	0.014	0.081
CTNNBIP1	293.921	0.129	0.344	0.708	0.799	293.921	0.353	0.343	0.305	0.571	293.921	0.223	0.397	0.574	0.761
ADAMTS9	1287.917	-0.058	0.409	0.887	0.927	1287.917	-0.810	0.410	0.048	0.337	1287.917	-0.751	0.473	0.112	0.299
PSMC1	2427.993	-0.342	0.218	0.117	0.220	2427.993	-0.370	0.218	0.090	0.383	2427.993	-0.028	0.252	0.911	0.959
GSN-AS1	18.741	0.128	0.643	0.842	0.896	18.741	0.700	0.629	0.266	0.538	18.741	0.571	0.733	0.436	0.657
SDHAF3	333.261	-0.385	0.195	0.049	0.113	333.261	-0.372	0.195	0.056	0.343	333.261	0.013	0.226	0.954	0.979
YAE1	128.628	0.411	0.244	0.092	0.184	128.628	-0.017	0.246	0.944	0.972	128.628	-0.429	0.283	0.130	0.327
CCDC47	4341.442	-0.634	0.179	0.000	0.003	4341.442	-0.146	0.179	0.413	0.652	4341.442	0.487	0.207	0.018	0.096
ACKR3	30.962	5.111	0.894	0.000	0.000	30.962	0.695	0.954	0.467	0.690	30.962	-4.417	1.034	0.000	0.001
PSMC2	1614.873	-0.047	0.267	0.860	0.908	1614.873	-0.595	0.267	0.026	0.316	1614.873	-0.548	0.308	0.075	0.236
COQ9	1206.199	-0.320	0.152	0.036	0.090	1206.199	-0.033	0.152	0.826	0.910	1206.199	0.287	0.176	0.103	0.285
CCNL1	601.104	0.477	0.301	0.113	0.213	601.104	-0.071	0.301	0.814	0.902	601.104	-0.548	0.348	0.115	0.304
CIAPIN1	1021.102	-0.516	0.279	0.064	0.140	1021.102	-0.548	0.279	0.049	0.337	1021.102	-0.032	0.322	0.921	0.963
PSMC3	2904.650	-0.088	0.305	0.774	0.849	2904.650	-0.559	0.305	0.067	0.356	2904.650	-0.471	0.353	0.182	0.400
VPS35L	668.379	0.027	0.106	0.801	0.869	668.379	0.056	0.105	0.597	0.776	668.379	0.029	0.122	0.813	0.907
PDXP	270.566	-0.785	0.404	0.052	0.119	270.566	-0.805	0.403	0.046	0.335	270.566	-0.020	0.468	0.966	0.984
SLC17A7	46.506	0.384	1.131	0.734	0.821	46.506	0.949	1.126	0.399	0.643	46.506	0.566	1.301	0.664	0.821
RSRP1	466.955	0.798	0.270	0.003	0.014	466.955	0.497	0.270	0.066	0.354	466.955	-0.301	0.312	0.334	0.569
ANKMY2	454.524	-0.394	0.277	0.155	0.271	454.524	0.174	0.275	0.528	0.729	454.524	0.568	0.319	0.075	0.236
RARS2	694.849	-0.268	0.116	0.021	0.059	694.849	-0.038	0.115	0.739	0.861	694.849	0.230	0.134	0.086	0.255
PSMC4	1762.328	0.048	0.333	0.885	0.925	1762.328	-0.830	0.333	0.013	0.305	1762.328	-0.878	0.385	0.023	0.109
TWSG1	759.454	0.349	0.206	0.090	0.182	759.454	0.086	0.206	0.677	0.825	759.454	-0.264	0.238	0.269	0.504
PLSCR3	10.630	1.423	0.629	0.024	0.066	10.630	0.205	0.663	0.757	0.871	10.630	-1.218	0.736	0.098	0.277
PSMC5	2332.870	0.096	0.231	0.678	0.776	2332.870	-0.314	0.231	0.175	0.464	2332.870	-0.410	0.267	0.125	0.319
UTP3	558.191	-0.021	0.208	0.919	0.949	558.191	-0.450	0.209	0.031	0.322	558.191	-0.428	0.241	0.076	0.237
PSMC6	1116.475	-0.463	0.178	0.009	0.032	1116.475	-0.283	0.177	0.111	0.404	1116.475	0.181	0.206	0.380	0.610
PCBP4	911.571	0.343	0.244	0.161	0.279	911.571	-0.401	0.245	0.102	0.394	911.571	-0.743	0.283	0.009	0.059
DDX24	3525.089	-0.057	0.212	0.789	0.860	3525.089	-0.437	0.212	0.039	0.327	3525.089	-0.380	0.245	0.120	0.311
PSMD1	1410.891	-0.600	0.427	0.161	0.279	1410.891	-1.386	0.428	0.001	0.297	1410.891	-0.787	0.494	0.111	0.298
PSMD2	6268.492	-1.007	0.361	0.005	0.021	6268.492	-0.944	0.361	0.009	0.297	6268.492	0.063	0.417	0.880	0.943
KNL1	461.502	-0.962	0.316	0.002	0.011	461.502	-0.350	0.315	0.266	0.538	461.502	0.613	0.365	0.093	0.268
AGTRAP	1369.322	0.882	0.197	0.000	0.000	1369.322	0.298	0.198	0.132	0.424	1369.322	-0.584	0.228	0.010	0.067
PLSCR4	304.155	0.351	0.493	0.477	0.610	304.155	0.504	0.492	0.306	0.572	304.155	0.153	0.568	0.788	0.894
ENTPD7	178.022	-0.618	0.400	0.123	0.228	178.022	-0.825	0.400	0.039	0.327	178.022	-0.207	0.464	0.655	0.815
PSMD3	3683.505	-0.147	0.233	0.528	0.655	3683.505	-0.480	0.233	0.039	0.327	3683.505	-0.334	0.269	0.215	0.441
PCNP	3626.426	-0.530	0.160	0.001	0.005	3626.426	-0.060	0.160	0.708	0.843	3626.426	0.470	0.185	0.011	0.069
PITHD1	1045.662	0.081	0.254	0.749	0.832	1045.662	-0.592	0.255	0.020	0.310	1045.662	-0.673	0.294	0.022	0.108
PARP11	142.885	-0.436	0.200	0.029	0.077	142.885	-0.294	0.197	0.135	0.426	142.885	0.142	0.232	0.540	0.737
AVEN	295.687	-0.023	0.243	0.923	0.951	295.687	-0.225	0.243	0.355	0.609	295.687	-0.201	0.281	0.474	0.688
BACH1	312.526	-0.021	0.254	0.933	0.958	312.526	-0.252	0.255	0.322	0.583	312.526	-0.231	0.295	0.433	0.655
PSMD4	3317.062	0.002	0.168	0.989	0.993	3317.062	-0.143	0.168	0.396	0.641	3317.062	-0.145	0.195	0.455	0.674
C12orf4	246.266	-0.630	0.319	0.048	0.112	246.266	-0.751	0.318	0.018	0.306	246.266	-0.121	0.369	0.744	0.869
TIGAR	187.221	-0.666	0.518	0.198	0.324	187.221	-1.386	0.520	0.008	0.297	187.221	-0.719	0.601	0.232	0.461

PNPLA2	2611.648	0.259	0.188	0.168	0.288	2611.648	0.386	0.188	0.040	0.327	2611.648	0.127	0.217	0.559	0.750
NAT14	504.237	0.743	0.175	0.000	0.000	504.237	0.098	0.176	0.577	0.762	504.237	-0.645	0.202	0.001	0.017
PDSS2	267.354	-0.799	0.255	0.002	0.009	267.354	0.110	0.251	0.661	0.815	267.354	0.909	0.294	0.002	0.021
REXO4	1378.198	-0.727	0.228	0.001	0.008	1378.198	-0.313	0.228	0.169	0.459	1378.198	0.414	0.264	0.116	0.305
PSMD5	969.262	-0.304	0.099	0.002	0.010	969.262	-0.102	0.098	0.297	0.565	969.262	0.203	0.114	0.076	0.236
RAB25	36.243	0.244	0.296	0.410	0.549	36.243	0.147	0.294	0.616	0.789	36.243	-0.097	0.342	0.778	0.888
ZNF695	74.707	-0.359	0.219	0.101	0.197	74.707	0.011	0.210	0.957	0.978	74.707	0.371	0.252	0.141	0.345
INTS12	531.628	-0.772	0.223	0.001	0.004	531.628	-0.478	0.222	0.031	0.322	531.628	0.294	0.258	0.254	0.487
CAMK1D	11.827	0.818	0.535	0.126	0.233	11.827	1.151	0.519	0.027	0.318	11.827	0.333	0.592	0.574	0.761
GOPC	1558.987	-0.550	0.165	0.001	0.005	1558.987	-0.401	0.165	0.015	0.306	1558.987	0.149	0.191	0.435	0.657
NUP107	838.785	-0.440	0.323	0.172	0.293	838.785	-0.898	0.323	0.005	0.297	838.785	-0.458	0.373	0.220	0.447
LYRM4	717.152	0.349	0.155	0.024	0.067	717.152	0.073	0.155	0.641	0.804	717.152	-0.277	0.179	0.122	0.314
MRPL47	759.381	0.115	0.364	0.752	0.834	759.381	-0.619	0.365	0.090	0.384	759.381	-0.734	0.421	0.081	0.247
PSMD7	1439.262	-0.370	0.300	0.217	0.347	1439.262	-0.691	0.300	0.021	0.314	1439.262	-0.320	0.347	0.356	0.589
ATP13A1	1515.787	0.310	0.196	0.114	0.216	1515.787	-0.097	0.196	0.621	0.791	1515.787	-0.407	0.227	0.073	0.230
CHMP1B	1252.339	0.037	0.094	0.695	0.789	1252.339	-0.031	0.093	0.744	0.863	1252.339	-0.067	0.108	0.534	0.733
APMAP	2105.201	-0.133	0.202	0.509	0.638	2105.201	-0.264	0.202	0.191	0.477	2105.201	-0.130	0.233	0.576	0.762
RGL3	19.136	0.208	0.407	0.610	0.722	19.136	-0.326	0.419	0.437	0.670	19.136	-0.534	0.483	0.269	0.504
PSMD8	3023.401	0.283	0.172	0.099	0.194	3023.401	-0.164	0.172	0.340	0.598	3023.401	-0.447	0.198	0.024	0.114
RNPEPL1	820.644	0.601	0.160	0.000	0.001	820.644	0.250	0.160	0.118	0.410	820.644	-0.351	0.184	0.057	0.199
RTN4	5994.030	-0.128	0.201	0.523	0.650	5994.030	-0.415	0.201	0.039	0.327	5994.030	-0.286	0.232	0.217	0.443
ADCK1	58.742	-0.088	0.391	0.822	0.884	58.742	-0.819	0.398	0.039	0.327	58.742	-0.732	0.459	0.111	0.298
TMEM159	774.779	0.454	0.316	0.151	0.266	774.779	0.517	0.316	0.102	0.394	774.779	0.062	0.365	0.864	0.935
SCYL3	194.792	-0.173	0.145	0.233	0.366	194.792	-0.240	0.144	0.097	0.391	194.792	-0.067	0.169	0.694	0.840
RALGAPB	744.627	-0.655	0.169	0.000	0.001	744.627	-0.355	0.168	0.034	0.322	744.627	0.301	0.195	0.123	0.316
LYRM1	673.385	-0.818	0.110	0.000	0.000	673.385	-0.053	0.106	0.616	0.788	673.385	0.765	0.126	0.000	0.000
PSMD9	695.889	-0.360	0.196	0.067	0.143	695.889	-0.194	0.195	0.319	0.581	695.889	0.165	0.226	0.466	0.681
SMIM8	106.502	0.030	0.293	0.920	0.949	106.502	-0.170	0.294	0.563	0.751	106.502	-0.200	0.340	0.557	0.749
SLC44A2	535.862	0.660	0.377	0.080	0.166	535.862	0.453	0.377	0.231	0.508	535.862	-0.208	0.436	0.634	0.801
SMURF1	593.140	-0.596	0.201	0.003	0.014	593.140	-0.232	0.200	0.246	0.521	593.140	0.364	0.233	0.117	0.307
TMEM63C	203.198	-0.582	0.510	0.254	0.388	203.198	0.501	0.507	0.323	0.584	203.198	1.083	0.588	0.066	0.216
PHTF2	408.567	-0.670	0.189	0.000	0.003	408.567	-0.200	0.187	0.284	0.554	408.567	0.470	0.218	0.031	0.135
JPH2	222.828	2.488	1.163	0.032	0.084	222.828	1.059	1.164	0.363	0.615	222.828	-1.429	1.343	0.287	0.522
TRIM54	12.431	1.959	0.616	0.001	0.008	12.431	1.592	0.622	0.010	0.299	12.431	-0.367	0.669	0.583	0.766
PSMD10	720.365	0.361	0.109	0.001	0.006	720.365	-0.095	0.110	0.389	0.635	720.365	-0.456	0.127	0.000	0.006
PELI2	17.318	1.030	0.475	0.030	0.079	17.318	0.802	0.477	0.093	0.388	17.318	-0.228	0.532	0.669	0.824
PELI1	244.527	-0.192	0.125	0.126	0.232	244.527	-0.015	0.122	0.902	0.950	244.527	0.177	0.145	0.222	0.450
GJC2	48.252	1.602	0.502	0.001	0.008	48.252	-0.212	0.589	0.719	0.849	48.252	-1.814	0.625	0.004	0.033
ASPHD2	80.694	-0.135	0.350	0.700	0.793	80.694	-0.514	0.352	0.144	0.435	80.694	-0.380	0.409	0.353	0.586
ZNFX1	1360.087	0.199	0.135	0.140	0.251	1360.087	-0.139	0.135	0.304	0.570	1360.087	-0.337	0.156	0.030	0.133
PSMD11	1261.049	-0.255	0.357	0.474	0.608	1261.049	-0.792	0.357	0.027	0.318	1261.049	-0.536	0.412	0.193	0.416
DOLPP1	182.811	-0.143	0.225	0.526	0.652	182.811	-0.180	0.224	0.422	0.659	182.811	-0.037	0.261	0.887	0.946
CORO1B	1695.008	1.065	0.211	0.000	0.000	1695.008	0.252	0.211	0.233	0.510	1695.008	-0.813	0.243	0.001	0.011
VARS2	1382.844	0.166	0.190	0.381	0.521	1382.844	0.385	0.189	0.042	0.330	1382.844	0.219	0.219	0.316	0.552
ZMIZ1	1843.098	-0.148	0.273	0.588	0.704	1843.098	0.257	0.273	0.346	0.602	1843.098	0.405	0.315	0.199	0.422
KIAA1191	1945.267	-0.075	0.152	0.620	0.730	1945.267	0.157	0.151	0.299	0.566	1945.267	0.232	0.175	0.185	0.404
PSMD12	1781.208	-0.395	0.324	0.223	0.354	1781.208	-0.915	0.324	0.005	0.297	1781.208	-0.520	0.375	0.165	0.380
ACTR3B	210.230	-0.584	0.144	0.000	0.001	210.230	-0.034	0.137	0.806	0.898	210.230	0.551	0.165	0.001	0.012
SLC39A10	501.725	-0.777	0.224	0.001	0.004	501.725	-0.393	0.223	0.078	0.369	501.725	0.384	0.259	0.138	0.341
ANKRD50	1061.426	-1.110	0.281	0.000	0.001	1061.426	-0.539	0.280	0.054	0.342	1061.426	0.571	0.324	0.078	0.241
FAM219B	408.972	0.451	0.180	0.012	0.040	408.972	0.240	0.180	0.181	0.469	408.972	-0.210	0.207	0.311	0.547
NIPAL3	2544.918	-0.438	0.199	0.028	0.073	2544.918	0.178	0.199	0.371	0.620	2544.918	0.616	0.230	0.007	0.053
RALGAPA2	849.526	-0.314	0.353	0.373	0.513	849.526	0.280	0.352	0.427	0.662	849.526	0.594	0.407	0.145	0.351
THOC2	1249.887	0.018	0.165	0.913	0.945	1249.887	-0.172	0.165	0.298	0.565	1249.887	-0.190	0.191	0.319	0.554
DENND11	804.055	-0.351	0.220	0.111	0.211	804.055	-0.128	0.220	0.561	0.750	804.055	0.224	0.254	0.379	0.610
PSMD13	2165.330	-0.110	0.283	0.696	0.790	2165.330	-0.607	0.283	0.032	0.322	2165.330	-0.497	0.327	0.128	0.324
SELENON	1639.671	0.286	0.182	0.117	0.220	1639.671	0.139	0.182	0.445	0.675	1639.671	-0.147	0.211	0.486	0.697
MCOLN1	240.378	0.469	0.250	0.061	0.135	240.378	-0.361	0.254	0.154	0.447	240.378	-0.830	0.292	0.004	0.038
ATP8B2	846.103	0.277	0.142	0.050	0.116	846.103	0.012	0.142	0.932	0.966	846.103	-0.265	0.164	0.105	0.289
BAD	872.034	0.956	0.247	0.000	0.001	872.034	0.373	0.247	0.131	0.423	872.034	-0.584	0.285	0.040	0.160
PSME1	2476.210	0.646	0.201	0.001	0.007	2476.210	0.283	0.201	0.160	0.451	2476.210	-0.364	0.232	0.117	0.307
ATP10D	267.783	-0.616	0.237	0.009	0.032	267.783	-0.168	0.234	0.472	0.694	267.783	0.448	0.274	0.102	0.283
ZNF248	142.600	0.069	0.332	0.836	0.892	142.600	0.265	0.330	0.422	0.658	142.600	0.197	0.382	0.607	0.783
PSME2	1937.926	0.639	0.273	0.019	0.056	1937.926	-0.387	0.274	0.157	0.449	1937.926	-1.026	0.316	0.001	0.015
SLC45A4	92.381	0.110	0.204	0.591	0.706	92.381	-0.439	0.210	0.036	0.324	92.381	-0.548	0.242	0.024	0.113
ADGRG6	873.511	-1.606	0.230	0.000	0.000	873.511	-0.058	0.227	0.799	0.895	873.511	1.548	0.265	0.000	0.000
TP73-AS1	77.775	1.484	0.539	0.006	0.022	77.775	0.836	0.541	0.123	0.414	77.775	-0.649	0.621	0.296	0.532

SPRYD7	631.221	-0.287	0.118	0.015	0.046	631.221	-0.141	0.116	0.226	0.504	631.221	0.146	0.136	0.284	0.519
CEMIP	94.626	0.370	0.245	0.131	0.239	94.626	0.270	0.244	0.268	0.539	94.626	-0.100	0.282	0.724	0.857
THAP11	625.603	-0.066	0.155	0.673	0.772	625.603	0.258	0.154	0.094	0.388	625.603	0.323	0.179	0.071	0.227
TTC7A	410.243	-0.018	0.131	0.891	0.929	410.243	-0.079	0.131	0.547	0.741	410.243	-0.061	0.152	0.689	0.837
ARFGEF3	454.078	0.196	0.812	0.809	0.874	454.078	0.864	0.811	0.287	0.555	454.078	0.668	0.937	0.476	0.689
ERGIC1	9081.744	-0.300	0.191	0.115	0.217	9081.744	0.296	0.190	0.120	0.412	9081.744	0.597	0.220	0.007	0.050
PPP4R3B	1875.858	-0.556	0.152	0.000	0.002	1875.858	-0.147	0.151	0.332	0.592	1875.858	0.410	0.175	0.019	0.099
NHSL1	379.454	-1.019	0.382	0.008	0.028	379.454	-0.666	0.381	0.080	0.371	379.454	0.353	0.441	0.424	0.648
LYRM2	545.785	-0.087	0.133	0.514	0.642	545.785	-0.242	0.133	0.070	0.358	545.785	-0.155	0.155	0.317	0.553
SMAGP	205.432	-0.119	0.268	0.656	0.759	205.432	-0.101	0.267	0.706	0.842	205.432	0.019	0.310	0.951	0.978
PSPH	439.717	-0.570	0.188	0.002	0.011	439.717	0.088	0.185	0.633	0.800	439.717	0.658	0.217	0.002	0.025
SNX14	827.118	-0.291	0.181	0.107	0.206	827.118	-0.025	0.180	0.889	0.945	827.118	0.266	0.209	0.203	0.426
LINC00869	456.349	0.399	0.192	0.037	0.093	456.349	0.090	0.192	0.639	0.803	456.349	-0.309	0.222	0.163	0.378
PTBP1	9225.850	-0.291	0.088	0.001	0.006	9225.850	-0.089	0.088	0.311	0.575	9225.850	0.202	0.102	0.047	0.176
PTCH1	204.958	-0.111	0.401	0.782	0.856	204.958	0.559	0.398	0.161	0.452	204.958	0.670	0.462	0.147	0.354
PTEN	1918.845	-0.247	0.158	0.118	0.222	1918.845	0.124	0.158	0.433	0.667	1918.845	0.371	0.183	0.042	0.165
DANCR	1911.563	-0.135	0.213	0.527	0.653	1911.563	0.226	0.213	0.290	0.558	1911.563	0.361	0.246	0.143	0.349
BAG1	1158.495	-0.190	0.186	0.305	0.445	1158.495	-0.213	0.185	0.250	0.524	1158.495	-0.023	0.215	0.914	0.960
KAT14	165.630	-0.655	0.191	0.001	0.004	165.630	0.074	0.183	0.685	0.830	165.630	0.730	0.219	0.001	0.012
PBXIP1	751.681	0.867	0.524	0.098	0.193	751.681	0.729	0.524	0.164	0.454	751.681	-0.138	0.605	0.820	0.911
CBX8	226.793	0.602	0.306	0.049	0.113	226.793	0.403	0.306	0.188	0.474	226.793	-0.200	0.352	0.571	0.759
RCN3	1836.725	1.696	0.243	0.000	0.000	1836.725	0.092	0.244	0.707	0.842	1836.725	-1.604	0.281	0.000	0.000
ZNF286A	187.824	0.007	0.170	0.966	0.979	187.824	0.193	0.167	0.248	0.522	187.824	0.186	0.195	0.341	0.575
SENP7	202.614	-0.672	0.308	0.029	0.077	202.614	0.289	0.303	0.341	0.599	202.614	0.961	0.354	0.007	0.050
PTGER4	185.352	0.722	0.305	0.018	0.053	185.352	-0.547	0.310	0.078	0.370	185.352	-1.268	0.356	0.000	0.006
ZNF304	146.244	-0.378	0.224	0.091	0.183	146.244	0.185	0.218	0.396	0.640	146.244	0.564	0.257	0.028	0.127
PTGFR	586.902	-0.596	0.255	0.020	0.057	586.902	0.246	0.254	0.332	0.592	586.902	0.842	0.294	0.004	0.037
PTGFRN	2118.561	0.060	0.186	0.749	0.832	2118.561	-0.225	0.186	0.228	0.506	2118.561	-0.285	0.215	0.186	0.407
MRS2	538.084	-0.072	0.118	0.545	0.670	538.084	-0.153	0.118	0.194	0.478	538.084	-0.082	0.137	0.552	0.745
CLTRN	10.308	0.117	0.905	0.897	0.933	10.308	0.825	0.885	0.351	0.605	10.308	0.708	1.031	0.492	0.702
CLK4	334.325	-0.437	0.376	0.245	0.379	334.325	0.737	0.373	0.048	0.337	334.325	1.174	0.433	0.007	0.050
S100A14	94.843	0.614	0.212	0.004	0.016	94.843	0.270	0.214	0.208	0.490	94.843	-0.344	0.245	0.160	0.373
RAB22A	514.884	0.223	0.141	0.114	0.215	514.884	-0.155	0.142	0.274	0.545	514.884	-0.378	0.164	0.021	0.104
SERTAD4-AS1	33.526	2.001	0.383	0.000	0.000	33.526	0.779	0.396	0.049	0.337	33.526	-1.222	0.439	0.005	0.043
CYP20A1	134.495	-0.278	0.230	0.225	0.357	134.495	-0.086	0.226	0.704	0.841	134.495	0.192	0.265	0.468	0.683
SPC25	315.306	-0.347	0.411	0.400	0.538	315.306	-0.842	0.412	0.041	0.329	315.306	-0.496	0.476	0.298	0.534
ABHD6	206.361	0.047	0.237	0.845	0.898	206.361	-0.608	0.240	0.011	0.305	206.361	-0.655	0.278	0.018	0.096
NMRAL1	1321.106	0.291	0.194	0.135	0.244	1321.106	0.124	0.194	0.525	0.728	1321.106	-0.167	0.225	0.457	0.675
MIF4GD	270.899	0.158	0.181	0.383	0.522	270.899	0.259	0.179	0.148	0.441	270.899	0.102	0.208	0.625	0.795
SCYL1	1404.887	0.134	0.197	0.494	0.626	1404.887	-0.166	0.197	0.399	0.643	1404.887	-0.300	0.227	0.187	0.407
AS3MT	8.308	1.566	0.611	0.010	0.035	8.308	0.166	0.657	0.801	0.896	8.308	-1.400	0.719	0.052	0.186
RHBDD2	574.521	0.124	0.204	0.544	0.668	574.521	-0.377	0.205	0.065	0.354	574.521	-0.501	0.236	0.034	0.144
C3orf14	175.358	0.237	0.248	0.340	0.481	175.358	-0.386	0.251	0.124	0.416	175.358	-0.623	0.289	0.031	0.136
WDR18	774.995	0.133	0.264	0.614	0.725	774.995	-0.146	0.264	0.580	0.764	774.995	-0.279	0.305	0.360	0.593
PTGS1	419.813	-0.305	0.903	0.735	0.821	419.813	0.478	0.902	0.596	0.776	419.813	0.783	1.042	0.452	0.671
PTGS2	175.614	-1.157	0.501	0.021	0.060	175.614	-1.078	0.496	0.030	0.322	175.614	0.079	0.587	0.893	0.949
ADAMTSL4-AS1	9.486	2.980	0.692	0.000	0.000	9.486	1.188	0.740	0.108	0.401	9.486	-1.792	0.771	0.020	0.102
MIR497	201.938	0.467	0.247	0.059	0.130	201.938	0.447	0.247	0.070	0.359	201.938	-0.021	0.285	0.942	0.974
NDRG3	766.027	0.177	0.151	0.241	0.374	766.027	0.168	0.151	0.266	0.538	766.027	-0.009	0.174	0.957	0.980
BIRC6	854.545	-0.547	0.252	0.030	0.078	854.545	-0.334	0.251	0.183	0.470	854.545	0.213	0.291	0.464	0.680
PLEKHG5	166.707	-0.288	0.311	0.354	0.494	166.707	0.302	0.307	0.325	0.585	166.707	0.590	0.357	0.099	0.278
PTH1R	15.001	1.564	0.958	0.103	0.199	15.001	1.603	0.956	0.093	0.388	15.001	0.039	1.088	0.971	0.986
TENM2	18.848	0.301	0.702	0.668	0.769	18.848	0.827	0.693	0.233	0.510	18.848	0.526	0.804	0.513	0.718
REXO1	593.381	0.056	0.288	0.846	0.899	593.381	-0.470	0.289	0.104	0.396	593.381	-0.526	0.334	0.115	0.304
KIAA1143	959.475	-0.189	0.218	0.385	0.524	959.475	0.253	0.217	0.244	0.519	959.475	0.442	0.251	0.079	0.242
GATAD2B	1710.148	-0.100	0.094	0.288	0.426	1710.148	0.045	0.094	0.633	0.800	1710.148	0.145	0.109	0.183	0.401
PPM1H	34.426	1.272	0.364	0.000	0.003	34.426	-0.481	0.394	0.222	0.501	34.426	-1.753	0.439	0.000	0.002
ISY1	377.299	0.759	0.234	0.001	0.007	377.299	-0.007	0.236	0.976	0.987	377.299	-0.767	0.271	0.005	0.040
MYORG	115.384	0.254	0.350	0.468	0.602	115.384	0.379	0.348	0.276	0.546	115.384	0.126	0.403	0.755	0.875
STRIP2	208.031	0.770	0.450	0.087	0.176	208.031	-1.213	0.457	0.008	0.297	208.031	-1.983	0.525	0.000	0.003
TBC1D24	275.049	-0.227	0.181	0.210	0.340	275.049	-0.199	0.180	0.268	0.540	275.049	0.028	0.210	0.896	0.951
SCAF4	725.240	0.008	0.230	0.971	0.982	725.240	-0.172	0.230	0.455	0.682	725.240	-0.180	0.266	0.498	0.705
PTK2	2435.270	-0.689	0.137	0.000	0.000	2435.270	-0.102	0.136	0.455	0.682	2435.270	0.588	0.158	0.000	0.004
LRRC47	1103.793	-0.581	0.158	0.000	0.002	1103.793	-0.024	0.157	0.878	0.939	1103.793	0.557	0.182	0.002	0.023
CNOT6	987.646	-0.528	0.144	0.000	0.002	987.646	-0.233	0.143	0.103	0.396	987.646	0.296	0.166	0.075	0.235
ZNF512B	514.863	-0.166	0.131	0.204	0.332	514.863	0.100	0.129	0.441	0.673	514.863	0.266	0.151	0.078	0.241
ZNF490	37.193	0.405	0.461	0.379	0.519	37.193	0.374	0.459	0.415	0.654	37.193	-0.031	0.530	0.953	0.978

PLEKHH1	147.800	0.453	0.347	0.192	0.317	147.800	0.511	0.346	0.140	0.431	147.800	0.057	0.400	0.886	0.945
GRAMD1B	128.766	-0.417	0.362	0.249	0.383	128.766	-0.009	0.358	0.980	0.989	128.766	0.408	0.417	0.328	0.562
USP31	1248.191	-0.113	0.234	0.629	0.738	1248.191	0.237	0.233	0.309	0.573	1248.191	0.350	0.270	0.194	0.416
PRR12	783.109	0.050	0.111	0.653	0.757	783.109	0.025	0.111	0.823	0.908	783.109	-0.025	0.129	0.844	0.925
CRACD	91.236	-0.413	0.443	0.351	0.491	91.236	-0.509	0.443	0.251	0.524	91.236	-0.095	0.513	0.853	0.930
RNF150	69.669	1.300	0.679	0.056	0.125	69.669	0.694	0.681	0.308	0.573	69.669	-0.606	0.783	0.439	0.660
NLN	2043.066	-0.811	0.151	0.000	0.000	2043.066	-0.260	0.150	0.083	0.374	2043.066	0.551	0.174	0.002	0.018
ESYT2	2786.127	-0.414	0.284	0.145	0.257	2786.127	0.340	0.283	0.230	0.507	2786.127	0.754	0.327	0.021	0.105
ODF2L	391.150	0.263	0.181	0.147	0.260	391.150	-0.284	0.182	0.119	0.412	391.150	-0.547	0.210	0.009	0.062
AHRR	85.215	-0.699	0.392	0.075	0.157	85.215	0.076	0.384	0.843	0.918	85.215	0.775	0.451	0.086	0.255
ARID1B	1559.538	-0.578	0.232	0.013	0.041	1559.538	0.230	0.232	0.321	0.582	1559.538	0.808	0.268	0.003	0.026
HEG1	1762.440	0.823	0.233	0.000	0.003	1762.440	-0.035	0.233	0.882	0.940	1762.440	-0.858	0.269	0.001	0.017
RIMKLB	160.019	-0.831	0.339	0.014	0.045	160.019	-0.684	0.337	0.042	0.330	160.019	0.147	0.393	0.709	0.849
MRTFB	483.846	-0.839	0.279	0.003	0.012	483.846	-0.135	0.277	0.626	0.794	483.846	0.704	0.322	0.029	0.128
KIDINS220	1328.416	-0.554	0.194	0.004	0.018	1328.416	-0.048	0.193	0.806	0.898	1328.416	0.506	0.224	0.024	0.113
KIAA1257	117.002	0.080	0.218	0.715	0.805	117.002	-0.526	0.223	0.018	0.306	117.002	-0.606	0.257	0.018	0.096
MTA3	651.234	-0.030	0.257	0.907	0.941	651.234	0.315	0.256	0.219	0.498	651.234	0.345	0.297	0.244	0.475
AARS2	385.106	-0.295	0.198	0.137	0.246	385.106	-0.256	0.198	0.196	0.479	385.106	0.040	0.229	0.863	0.935
MAVS	1530.977	0.265	0.219	0.227	0.359	1530.977	0.317	0.219	0.149	0.441	1530.977	0.052	0.253	0.838	0.922
ZNF608	10.722	0.580	0.641	0.365	0.505	10.722	0.905	0.625	0.147	0.440	10.722	0.325	0.721	0.652	0.813
INTS2	219.215	-0.265	0.149	0.075	0.158	219.215	0.062	0.145	0.671	0.822	219.215	0.326	0.171	0.057	0.198
MTUS1	124.041	1.581	0.237	0.000	0.000	124.041	0.149	0.254	0.557	0.747	124.041	-1.432	0.279	0.000	0.000
XPO5	1680.360	-0.973	0.330	0.003	0.014	1680.360	-0.694	0.330	0.035	0.324	1680.360	0.279	0.381	0.463	0.680
COG6	308.285	-0.048	0.232	0.837	0.893	308.285	0.277	0.231	0.230	0.507	308.285	0.325	0.268	0.225	0.453
GPR158	259.427	-0.825	0.215	0.000	0.001	259.427	-0.165	0.210	0.433	0.667	259.427	0.660	0.247	0.008	0.054
CASKIN2	371.154	-0.122	0.176	0.490	0.623	371.154	-0.303	0.176	0.086	0.378	371.154	-0.181	0.205	0.375	0.607
ARHGAP31	264.857	0.754	0.172	0.000	0.000	264.857	-0.141	0.176	0.424	0.660	264.857	-0.895	0.201	0.000	0.000
SERINC1	3725.488	-0.208	0.185	0.263	0.398	3725.488	0.030	0.185	0.873	0.936	3725.488	0.237	0.214	0.268	0.502
STARD9	188.198	0.106	0.292	0.715	0.806	188.198	0.368	0.290	0.204	0.487	188.198	0.262	0.336	0.435	0.657
HECW2	69.767	-0.251	0.341	0.461	0.596	69.767	0.130	0.335	0.697	0.837	69.767	0.381	0.392	0.331	0.565
RPTOR	1385.263	-0.010	0.155	0.950	0.969	1385.263	0.127	0.154	0.409	0.649	1385.263	0.137	0.178	0.443	0.663
SRGAP1	842.185	-0.509	0.214	0.017	0.052	842.185	-0.213	0.214	0.320	0.581	842.185	0.297	0.247	0.231	0.459
CASKIN1	713.779	1.449	0.283	0.000	0.000	713.779	0.151	0.284	0.596	0.775	713.779	-1.298	0.327	0.000	0.002
KCTD16	82.529	-1.059	0.324	0.001	0.006	82.529	-0.196	0.311	0.528	0.729	82.529	0.863	0.372	0.021	0.103
PTK6	24.403	0.837	0.394	0.034	0.086	24.403	0.948	0.388	0.014	0.306	24.403	0.111	0.441	0.800	0.900
CGN	15.717	0.486	0.446	0.276	0.413	15.717	0.266	0.447	0.551	0.744	15.717	-0.219	0.513	0.669	0.824
HACE1	346.168	-0.363	0.312	0.245	0.378	346.168	0.308	0.310	0.320	0.581	346.168	0.671	0.360	0.062	0.209
NUFIP2	2354.318	-0.389	0.203	0.055	0.124	2354.318	-0.206	0.203	0.310	0.575	2354.318	0.183	0.234	0.434	0.656
TBC1D14	1129.496	-0.682	0.172	0.000	0.001	1129.496	0.043	0.171	0.800	0.896	1129.496	0.725	0.198	0.000	0.005
MIB1	1124.590	-0.624	0.290	0.031	0.081	1124.590	-0.021	0.289	0.942	0.971	1124.590	0.602	0.334	0.072	0.229
KIAA1324	14.908	0.258	0.598	0.665	0.767	14.908	0.535	0.587	0.362	0.614	14.908	0.276	0.682	0.686	0.834
KIAA1328	36.553	-0.382	0.370	0.303	0.441	36.553	0.258	0.355	0.468	0.691	36.553	0.640	0.423	0.130	0.327
SORCS2	23.785	0.014	0.696	0.984	0.991	23.785	0.803	0.683	0.240	0.516	23.785	0.790	0.796	0.321	0.556
WDR35	241.176	-1.250	0.276	0.000	0.000	241.176	-0.580	0.272	0.033	0.322	241.176	0.670	0.319	0.036	0.148
PTK7	5189.711	0.755	0.278	0.007	0.025	5189.711	0.431	0.278	0.121	0.413	5189.711	-0.324	0.321	0.313	0.549
ZNF398	232.596	-0.963	0.262	0.000	0.002	232.596	-0.531	0.258	0.040	0.327	232.596	0.432	0.303	0.153	0.363
KLHL42	394.374	-0.057	0.167	0.734	0.821	394.374	-0.303	0.167	0.069	0.357	394.374	-0.247	0.193	0.202	0.425
TXNDC16	238.519	0.063	0.295	0.830	0.888	238.519	0.192	0.294	0.514	0.720	238.519	0.128	0.340	0.706	0.847
CC2D2A	184.287	-0.791	0.255	0.002	0.010	184.287	0.095	0.249	0.702	0.840	184.287	0.887	0.293	0.002	0.025
PDP2	355.628	0.028	0.183	0.878	0.921	355.628	-0.031	0.183	0.865	0.931	355.628	-0.059	0.212	0.780	0.889
ZNF624	11.306	0.034	0.462	0.941	0.963	11.306	-0.114	0.461	0.805	0.898	11.306	-0.148	0.541	0.784	0.892
IGSF9	42.524	0.864	0.403	0.032	0.083	42.524	0.511	0.407	0.209	0.490	42.524	-0.352	0.462	0.445	0.665
TAOK1	1483.127	-0.450	0.271	0.097	0.192	1483.127	-0.395	0.271	0.145	0.437	1483.127	0.055	0.313	0.861	0.933
NCEH1	417.868	-0.805	0.323	0.013	0.041	417.868	-0.830	0.323	0.010	0.299	417.868	-0.026	0.374	0.945	0.975
MICAL3	1280.239	-0.177	0.163	0.278	0.415	1280.239	-0.201	0.163	0.217	0.496	1280.239	-0.024	0.189	0.898	0.952
NLGN2	590.773	0.843	0.429	0.049	0.114	590.773	0.679	0.429	0.113	0.405	590.773	-0.164	0.495	0.741	0.867
SEMA6A	11.750	1.044	0.572	0.068	0.146	11.750	0.936	0.568	0.100	0.394	11.750	-0.109	0.632	0.864	0.935
USP35	161.386	1.076	0.243	0.000	0.000	161.386	0.196	0.247	0.428	0.663	161.386	-0.880	0.281	0.002	0.020
STAMBPL1	229.536	-1.534	0.434	0.000	0.003	229.536	-0.888	0.430	0.039	0.327	229.536	0.646	0.501	0.197	0.420
TWF1	2707.206	-0.252	0.129	0.050	0.116	2707.206	-0.309	0.129	0.017	0.306	2707.206	-0.056	0.149	0.705	0.847
IFT80	265.704	-0.688	0.206	0.001	0.005	265.704	-0.200	0.203	0.323	0.584	265.704	0.487	0.238	0.040	0.160
ARRDC3	1148.084	0.025	0.199	0.898	0.934	1148.084	0.115	0.199	0.563	0.751	1148.084	0.090	0.230	0.697	0.841
CEP126	119.852	0.017	0.304	0.954	0.972	119.852	0.284	0.301	0.346	0.602	119.852	0.267	0.350	0.446	0.665
KLHL8	605.517	-0.697	0.192	0.000	0.002	605.517	-0.394	0.191	0.039	0.327	605.517	0.302	0.222	0.174	0.391
ZNF319	514.818	-0.008	0.176	0.964	0.978	514.818	-0.106	0.175	0.547	0.741	514.818	-0.098	0.203	0.631	0.799
SIPA1L2	242.474	0.359	0.424	0.396	0.535	242.474	-0.878	0.427	0.040	0.327	242.474	-1.238	0.492	0.012	0.073
PTMA	28443.189	0.552	0.184	0.003	0.012	28443.189	0.038	0.184	0.838	0.916	28443.189	-0.515	0.212	0.015	0.086

TRMT5	560.085	-0.528	0.154	0.001	0.004	560.085	-0.105	0.152	0.488	0.705	560.085	0.423	0.178	0.017	0.094
DOCK6	422.563	-0.013	0.295	0.966	0.979	422.563	0.665	0.293	0.023	0.316	422.563	0.678	0.340	0.046	0.173
MARCHF4	61.503	-0.821	0.832	0.324	0.464	61.503	-2.699	0.849	0.001	0.297	61.503	-1.878	0.978	0.055	0.194
KIF17	21.121	1.560	0.556	0.005	0.020	21.121	1.105	0.562	0.049	0.337	21.121	-0.454	0.624	0.466	0.682
CCDC191	51.056	0.595	0.431	0.168	0.288	51.056	0.761	0.429	0.076	0.365	51.056	0.166	0.494	0.737	0.865
UNC79	8.910	0.269	0.785	0.731	0.818	8.910	-1.213	0.873	0.165	0.455	8.910	-1.482	0.986	0.133	0.331
FAM135A	287.777	-0.493	0.191	0.010	0.034	287.777	-0.085	0.188	0.653	0.811	287.777	0.408	0.220	0.064	0.213
PREX1	945.836	-0.626	0.185	0.001	0.004	945.836	-0.351	0.184	0.057	0.346	945.836	0.275	0.214	0.199	0.422
TMEM181	895.147	-0.597	0.241	0.013	0.042	895.147	-0.621	0.241	0.010	0.298	895.147	-0.025	0.279	0.929	0.968
ARHGAP21	1208.912	-0.346	0.247	0.161	0.280	1208.912	-0.473	0.247	0.056	0.343	1208.912	-0.126	0.286	0.658	0.817
CRAMP1	437.035	-0.535	0.160	0.001	0.005	437.035	-0.332	0.158	0.036	0.324	437.035	0.203	0.185	0.272	0.507
CFAP97	1436.303	-0.990	0.240	0.000	0.000	1436.303	-0.155	0.239	0.518	0.723	1436.303	0.835	0.277	0.003	0.026
RIC1	347.275	-0.725	0.310	0.019	0.056	347.275	0.134	0.307	0.662	0.816	347.275	0.859	0.357	0.016	0.089
WDFY1	840.736	-0.127	0.244	0.601	0.715	840.736	0.168	0.243	0.491	0.707	840.736	0.295	0.281	0.294	0.530
MRTFA	724.343	0.106	0.189	0.573	0.691	724.343	-0.034	0.189	0.858	0.927	724.343	-0.140	0.218	0.520	0.723
ZNF687	677.952	-0.197	0.145	0.175	0.297	677.952	0.067	0.144	0.643	0.805	677.952	0.264	0.168	0.116	0.305
EBF4	27.401	1.410	0.689	0.041	0.099	27.401	0.591	0.697	0.396	0.640	27.401	-0.819	0.794	0.302	0.537
HOMEZ	365.489	0.674	0.243	0.005	0.021	365.489	0.390	0.243	0.108	0.401	365.489	-0.284	0.280	0.310	0.546
PDZD4	11.233	2.022	0.924	0.029	0.076	11.233	1.593	0.935	0.089	0.382	11.233	-0.429	1.003	0.669	0.824
BEGAIN	14.806	0.586	0.495	0.236	0.369	14.806	-0.248	0.521	0.634	0.800	14.806	-0.834	0.590	0.158	0.370
BAHCC1	641.771	0.421	0.323	0.193	0.318	641.771	0.445	0.323	0.168	0.457	641.771	0.024	0.373	0.948	0.976
WDR48	927.293	-0.579	0.147	0.000	0.001	927.293	-0.081	0.145	0.577	0.762	927.293	0.499	0.169	0.003	0.030
ADGRB2	97.516	1.343	0.260	0.000	0.000	97.516	-0.134	0.272	0.623	0.792	97.516	-1.476	0.306	0.000	0.000
FNIP2	373.576	0.488	0.253	0.054	0.122	373.576	0.041	0.254	0.873	0.936	373.576	-0.447	0.293	0.127	0.322
USP36	1390.927	-0.913	0.276	0.001	0.006	1390.927	-0.562	0.276	0.042	0.330	1390.927	0.351	0.319	0.272	0.506
TRMT9B	9.111	0.479	0.639	0.453	0.588	9.111	-0.152	0.656	0.817	0.904	9.111	-0.631	0.751	0.400	0.627
PITPNM2	525.966	0.212	0.153	0.167	0.286	525.966	0.052	0.153	0.736	0.859	525.966	-0.160	0.177	0.365	0.597
SLAIN2	1255.971	-0.666	0.221	0.003	0.012	1255.971	-0.358	0.220	0.104	0.397	1255.971	0.307	0.255	0.228	0.457
JCAD	381.323	0.858	0.399	0.032	0.082	381.323	0.288	0.400	0.471	0.693	381.323	-0.570	0.461	0.216	0.443
DIP2B	702.440	-0.539	0.262	0.040	0.097	702.440	-0.192	0.261	0.463	0.689	702.440	0.347	0.303	0.251	0.484
RANBP10	471.438	-0.444	0.192	0.021	0.059	471.438	0.096	0.190	0.612	0.786	471.438	0.540	0.221	0.014	0.083
FAM234B	290.440	-0.337	0.285	0.238	0.371	290.440	0.359	0.283	0.204	0.487	290.440	0.696	0.329	0.034	0.144
RELCH	308.081	-0.488	0.217	0.024	0.067	308.081	-0.169	0.215	0.432	0.666	308.081	0.319	0.251	0.203	0.426
TSHZ3	288.697	-0.129	0.158	0.412	0.550	288.697	0.181	0.155	0.243	0.518	288.697	0.310	0.181	0.087	0.257
VPS18	466.123	0.536	0.154	0.001	0.003	466.123	-0.208	0.157	0.185	0.472	466.123	-0.744	0.180	0.000	0.001
SHROOM3	278.833	-0.405	0.207	0.051	0.117	278.833	0.001	0.205	0.998	0.999	278.833	0.406	0.239	0.090	0.262
STIM2	244.312	-0.269	0.159	0.090	0.180	244.312	-0.493	0.159	0.002	0.297	244.312	-0.224	0.186	0.227	0.455
ZBTB2	473.555	-0.986	0.311	0.002	0.008	473.555	-0.620	0.310	0.045	0.334	473.555	0.366	0.359	0.308	0.544
ZFAT	185.165	-0.339	0.175	0.053	0.121	185.165	-0.343	0.174	0.048	0.337	185.165	-0.004	0.204	0.986	0.992
PTMS	5404.983	1.094	0.303	0.000	0.002	5404.983	0.338	0.303	0.263	0.536	5404.983	-0.755	0.349	0.031	0.134
SH3RF1	115.422	-0.735	0.391	0.060	0.133	115.422	-0.687	0.390	0.078	0.369	115.422	0.047	0.454	0.917	0.961
EP400	1350.071	-0.648	0.184	0.000	0.003	1350.071	-0.330	0.183	0.072	0.361	1350.071	0.318	0.212	0.134	0.333
ARHGAP23	2112.073	0.402	0.247	0.103	0.200	2112.073	0.286	0.247	0.246	0.521	2112.073	-0.116	0.285	0.684	0.833
CCDC146	9.714	1.627	0.653	0.013	0.041	9.714	0.365	0.684	0.594	0.774	9.714	-1.262	0.758	0.096	0.273
PTN	72.727	1.689	0.421	0.000	0.001	72.727	-0.122	0.431	0.777	0.881	72.727	-1.812	0.491	0.000	0.004
ZSWIM5	23.235	0.704	0.565	0.213	0.342	23.235	0.903	0.559	0.106	0.398	23.235	0.199	0.643	0.757	0.876
MYH7B	11.451	1.971	0.497	0.000	0.001	11.451	-0.389	0.613	0.526	0.728	11.451	-2.360	0.639	0.000	0.004
POGK	1009.514	-0.818	0.243	0.001	0.005	1009.514	-0.522	0.242	0.031	0.322	1009.514	0.296	0.281	0.291	0.526
USP28	319.305	-0.397	0.238	0.095	0.189	319.305	0.031	0.236	0.896	0.948	319.305	0.428	0.275	0.119	0.309
DHX37	1049.626	-0.721	0.218	0.001	0.006	1049.626	-0.405	0.217	0.062	0.350	1049.626	0.316	0.252	0.210	0.435
KIAA1522	1779.785	0.131	0.330	0.691	0.787	1779.785	0.467	0.330	0.157	0.449	1779.785	0.336	0.381	0.378	0.609
PHF12	628.553	-0.039	0.104	0.708	0.800	628.553	-0.127	0.104	0.223	0.502	628.553	-0.088	0.121	0.469	0.684
CIP2A	403.659	-1.019	0.353	0.004	0.016	403.659	-0.876	0.353	0.013	0.305	403.659	0.143	0.409	0.726	0.858
UVSSA	99.639	-0.630	0.370	0.088	0.178	99.639	-0.512	0.367	0.163	0.454	99.639	0.118	0.428	0.783	0.891
GRAMD1A	2158.687	0.744	0.182	0.000	0.000	2158.687	0.309	0.182	0.089	0.383	2158.687	-0.435	0.210	0.038	0.154
HCN3	72.484	1.548	0.308	0.000	0.000	72.484	0.236	0.321	0.462	0.688	72.484	-1.312	0.359	0.000	0.005
CALCOCO1	796.727	0.379	0.485	0.434	0.570	796.727	0.699	0.484	0.149	0.442	796.727	0.320	0.559	0.568	0.758
ZBTB4	1158.954	0.154	0.305	0.613	0.724	1158.954	0.298	0.305	0.328	0.588	1158.954	0.144	0.352	0.683	0.832
PHRF1	2784.411	-0.042	0.160	0.795	0.865	2784.411	-0.339	0.160	0.035	0.322	2784.411	-0.297	0.185	0.109	0.294
PLEKHA4	590.057	1.114	0.253	0.000	0.000	590.057	0.519	0.254	0.041	0.328	590.057	-0.595	0.292	0.042	0.163
RDH14	327.589	0.638	0.187	0.001	0.004	327.589	0.104	0.189	0.584	0.767	327.589	-0.535	0.217	0.014	0.081
FBRSL1	1294.055	-0.304	0.230	0.187	0.312	1294.055	-0.326	0.230	0.156	0.448	1294.055	-0.023	0.266	0.932	0.969
EPB41L5	175.291	0.018	0.281	0.948	0.968	175.291	0.212	0.280	0.449	0.678	175.291	0.194	0.324	0.551	0.745
KIAA1549	213.650	-0.196	0.183	0.285	0.422	213.650	-0.285	0.182	0.118	0.409	213.650	-0.090	0.213	0.673	0.827
BEND3	73.553	-0.454	0.423	0.283	0.421	73.553	-1.223	0.430	0.004	0.297	73.553	-0.769	0.498	0.123	0.315
RNF213	2022.349	0.055	0.203	0.786	0.858	2022.349	0.045	0.203	0.823	0.908	2022.349	-0.010	0.235	0.966	0.984
GPAM	715.045	-1.136	0.313	0.000	0.002	715.045	0.283	0.310	0.361	0.613	715.045	1.419	0.360	0.000	0.002

ALS2	344.084	-0.428	0.237	0.071	0.151	344.084	-0.254	0.236	0.282	0.552	344.084	0.174	0.274	0.524	0.726
QSOX1	3614.567	0.018	0.167	0.914	0.946	3614.567	0.266	0.167	0.111	0.405	3614.567	0.248	0.193	0.199	0.422
CHD8	1073.514	-0.608	0.160	0.000	0.001	1073.514	-0.321	0.160	0.044	0.333	1073.514	0.287	0.185	0.122	0.313
ZDBF2	218.754	-0.573	0.353	0.105	0.202	218.754	0.072	0.350	0.836	0.916	218.754	0.646	0.407	0.113	0.301
ZBTB26	93.819	-0.692	0.186	0.000	0.002	93.819	-0.261	0.176	0.139	0.430	93.819	0.432	0.215	0.045	0.170
CACHD1	204.377	-0.024	0.274	0.931	0.956	204.377	0.538	0.271	0.047	0.336	204.377	0.562	0.315	0.074	0.234
ZSWIM6	508.760	-0.114	0.179	0.524	0.651	508.760	-0.208	0.178	0.242	0.518	508.760	-0.095	0.207	0.647	0.810
TNRC6C	224.232	-0.224	0.243	0.357	0.497	224.232	0.184	0.240	0.443	0.674	224.232	0.408	0.279	0.145	0.351
KIAA1586	213.328	-0.183	0.189	0.333	0.473	213.328	-0.241	0.188	0.200	0.483	213.328	-0.058	0.219	0.791	0.896
MAGEE1	8.962	0.829	0.571	0.147	0.260	8.962	-0.578	0.623	0.353	0.607	8.962	-1.406	0.696	0.043	0.167
ZNF317	576.136	-0.495	0.172	0.004	0.017	576.136	-0.138	0.170	0.416	0.654	576.136	0.356	0.198	0.072	0.230
USP37	305.859	-0.536	0.319	0.093	0.186	305.859	0.065	0.317	0.838	0.916	305.859	0.601	0.368	0.103	0.285
DDX55	567.741	-0.077	0.295	0.795	0.865	567.741	-0.409	0.295	0.166	0.456	567.741	-0.333	0.341	0.330	0.564
FANCM	104.380	-1.004	0.389	0.010	0.034	104.380	-1.038	0.387	0.007	0.297	104.380	-0.034	0.452	0.939	0.973
SHTN1	352.642	0.025	0.155	0.874	0.919	352.642	0.080	0.154	0.603	0.780	352.642	0.056	0.179	0.756	0.875
ADGRB3	27.999	-1.220	0.598	0.041	0.100	27.999	0.640	0.563	0.256	0.529	27.999	1.859	0.678	0.006	0.047
PTPN1	2405.973	-0.412	0.250	0.100	0.195	2405.973	-0.389	0.250	0.120	0.412	2405.973	0.023	0.289	0.937	0.972
FAM160B1	162.884	0.018	0.274	0.946	0.967	162.884	0.176	0.272	0.517	0.722	162.884	0.158	0.316	0.617	0.790
NCKAP5L	552.611	0.097	0.153	0.524	0.651	552.611	-0.150	0.153	0.327	0.587	552.611	-0.247	0.177	0.163	0.376
CWC22	488.339	-0.663	0.236	0.005	0.020	488.339	-0.590	0.235	0.012	0.305	488.339	0.072	0.273	0.791	0.896
GBA2	536.462	-0.058	0.181	0.747	0.830	536.462	-0.197	0.181	0.275	0.545	536.462	-0.139	0.209	0.507	0.713
DENND1A	734.836	-0.028	0.167	0.869	0.915	734.836	0.237	0.166	0.155	0.447	734.836	0.264	0.193	0.170	0.387
MEAK7	566.213	0.631	0.174	0.000	0.002	566.213	0.456	0.174	0.009	0.297	566.213	-0.174	0.201	0.385	0.614
MIER1	958.723	-0.503	0.134	0.000	0.001	958.723	-0.184	0.133	0.166	0.456	958.723	0.319	0.154	0.039	0.155
PTPN2	690.391	-0.113	0.139	0.414	0.552	690.391	-0.034	0.138	0.808	0.899	690.391	0.080	0.161	0.619	0.792
KIAA1614	42.403	-0.165	0.251	0.511	0.640	42.403	-0.191	0.247	0.439	0.671	42.403	-0.026	0.293	0.929	0.967
ZNF529	97.512	-0.317	0.195	0.104	0.201	97.512	-0.094	0.189	0.619	0.790	97.512	0.223	0.226	0.322	0.557
SEMA4G	115.773	0.407	0.329	0.216	0.346	115.773	0.622	0.327	0.057	0.346	115.773	0.215	0.378	0.569	0.758
PRX	112.471	0.583	0.548	0.287	0.426	112.471	0.781	0.547	0.153	0.445	112.471	0.198	0.631	0.753	0.874
PPP4R4	531.145	-0.601	0.182	0.001	0.006	531.145	-0.141	0.180	0.433	0.667	531.145	0.460	0.210	0.028	0.127
ANO8	547.508	0.335	0.267	0.209	0.337	547.508	0.473	0.266	0.075	0.364	547.508	0.138	0.307	0.654	0.814
GPR107	1604.076	-0.268	0.202	0.184	0.308	1604.076	0.031	0.202	0.880	0.939	1604.076	0.299	0.233	0.200	0.423
METTL14	348.251	-0.292	0.144	0.042	0.102	348.251	-0.106	0.142	0.457	0.683	348.251	0.187	0.166	0.262	0.496
EPG5	657.483	-0.473	0.327	0.148	0.262	657.483	-0.646	0.327	0.048	0.337	657.483	-0.173	0.378	0.646	0.809
NCOA5	362.730	-0.424	0.183	0.020	0.058	362.730	-0.344	0.182	0.058	0.348	362.730	0.080	0.212	0.706	0.847
WDR19	185.864	0.274	0.184	0.137	0.247	185.864	0.134	0.184	0.466	0.690	185.864	-0.140	0.213	0.510	0.716
ANKRD36B	70.923	0.372	0.330	0.259	0.394	70.923	0.019	0.331	0.955	0.978	70.923	-0.354	0.382	0.355	0.588
SPTBN4	10.507	1.279	0.668	0.056	0.125	10.507	0.843	0.676	0.212	0.492	10.507	-0.436	0.758	0.565	0.755
ZFYVE28	247.137	0.253	0.260	0.332	0.472	247.137	0.488	0.259	0.059	0.350	247.137	0.235	0.300	0.432	0.655
PTPN3	178.264	-0.169	0.296	0.567	0.687	178.264	0.351	0.293	0.231	0.508	178.264	0.520	0.340	0.126	0.321
PTPN4	172.427	-0.448	0.262	0.087	0.176	172.427	-0.040	0.258	0.876	0.937	172.427	0.408	0.302	0.177	0.394
TRIB3	2894.300	-2.089	0.447	0.000	0.000	2894.300	-0.876	0.446	0.049	0.337	2894.300	1.212	0.516	0.019	0.097
ANKRA2	279.637	0.046	0.190	0.807	0.873	279.637	0.396	0.187	0.035	0.322	279.637	0.349	0.218	0.108	0.294
PTPN6	13.430	0.427	0.440	0.333	0.473	13.430	0.022	0.449	0.961	0.980	13.430	-0.404	0.515	0.432	0.654
RBAK	452.737	-0.437	0.206	0.034	0.087	452.737	-0.272	0.205	0.184	0.471	452.737	0.165	0.238	0.490	0.700
MARK4	888.709	0.785	0.340	0.021	0.059	888.709	0.510	0.340	0.133	0.425	888.709	-0.275	0.392	0.483	0.695
SUGP1	435.015	0.271	0.158	0.087	0.176	435.015	-0.076	0.159	0.633	0.800	435.015	-0.346	0.183	0.059	0.202
GATAD1	813.600	0.136	0.145	0.349	0.489	813.600	0.090	0.145	0.533	0.733	813.600	-0.046	0.168	0.785	0.892
RAB40C	2746.547	0.753	0.417	0.071	0.151	2746.547	0.737	0.417	0.077	0.368	2746.547	-0.016	0.481	0.973	0.987
BAK1	617.038	-0.412	0.370	0.266	0.402	617.038	-0.691	0.370	0.062	0.350	617.038	-0.279	0.428	0.514	0.718
PTPN9	784.562	-0.550	0.154	0.000	0.003	784.562	-0.170	0.152	0.265	0.537	784.562	0.380	0.177	0.032	0.138
HES4	564.556	1.085	0.287	0.000	0.001	564.556	-0.359	0.289	0.214	0.493	564.556	-1.444	0.332	0.000	0.000
POLD4	563.264	1.005	0.267	0.000	0.001	563.264	0.724	0.267	0.007	0.297	563.264	-0.281	0.307	0.360	0.593
CCAR2	1909.305	-0.146	0.179	0.415	0.552	1909.305	-0.313	0.179	0.080	0.371	1909.305	-0.167	0.206	0.418	0.643
PTPN11	5280.488	-0.983	0.207	0.000	0.000	5280.488	-0.376	0.206	0.068	0.357	5280.488	0.607	0.238	0.011	0.069
LSM2	2793.407	0.175	0.188	0.354	0.494	2793.407	0.171	0.188	0.364	0.616	2793.407	-0.004	0.217	0.986	0.992
PTPN12	1663.966	-0.416	0.167	0.013	0.041	1663.966	-0.300	0.167	0.071	0.360	1663.966	0.115	0.193	0.549	0.744
CCNB1IP1	1536.888	-0.308	0.145	0.034	0.086	1536.888	0.134	0.144	0.352	0.606	1536.888	0.442	0.167	0.008	0.057
GRHL3	24.063	-0.781	0.353	0.027	0.073	24.063	0.258	0.312	0.409	0.649	24.063	1.039	0.396	0.009	0.059
RAP2C	385.452	-0.651	0.235	0.005	0.021	385.452	-0.178	0.232	0.443	0.674	385.452	0.473	0.271	0.080	0.245
C6orf47	1660.237	0.290	0.173	0.094	0.187	1660.237	-0.231	0.173	0.182	0.469	1660.237	-0.521	0.200	0.009	0.062
PTPN13	1061.223	-1.114	0.267	0.000	0.000	1061.223	0.004	0.265	0.987	0.992	1061.223	1.118	0.308	0.000	0.005
CYP4F11	25.727	1.326	0.382	0.001	0.003	25.727	0.825	0.388	0.033	0.322	25.727	-0.501	0.433	0.247	0.478
SLC4A5	2139.020	-1.037	0.205	0.000	0.000	2139.020	-0.119	0.204	0.561	0.750	2139.020	0.919	0.237	0.000	0.003
PTPN14	3826.377	-0.766	0.292	0.009	0.030	3826.377	0.212	0.292	0.468	0.691	3826.377	0.978	0.337	0.004	0.033
PTPRA	1246.444	0.182	0.122	0.135	0.245	1246.444	0.104	0.122	0.393	0.637	1246.444	-0.078	0.141	0.579	0.763
ZNF410	698.914	-0.917	0.318	0.004	0.016	698.914	-0.657	0.317	0.038	0.327	698.914	0.260	0.367	0.479	0.692

CADM3	147.423	2.069	0.318	0.000	0.000	147.423	0.250	0.325	0.442	0.673	147.423	-1.819	0.369	0.000	0.000
PTPRB	31.791	2.085	0.376	0.000	0.000	31.791	-0.032	0.423	0.939	0.969	31.791	-2.117	0.453	0.000	0.000
PTPRD	109.642	-0.586	0.361	0.104	0.202	109.642	0.374	0.354	0.291	0.559	109.642	0.960	0.414	0.021	0.103
PTPRE	274.854	0.280	0.129	0.030	0.079	274.854	-0.064	0.131	0.623	0.793	274.854	-0.344	0.151	0.022	0.109
PTPRF	6895.856	-0.135	0.225	0.549	0.672	6895.856	0.250	0.225	0.267	0.538	6895.856	0.385	0.260	0.139	0.342
PTPRG	798.712	-0.227	0.220	0.301	0.440	798.712	-0.147	0.219	0.502	0.714	798.712	0.080	0.254	0.753	0.874
PTPRH	352.035	0.321	0.370	0.387	0.526	352.035	0.795	0.369	0.031	0.322	352.035	0.474	0.427	0.266	0.501
PTPRJ	351.615	0.063	0.135	0.642	0.748	351.615	-0.112	0.135	0.409	0.649	351.615	-0.175	0.157	0.266	0.500
PTPRK	968.568	-0.373	0.190	0.049	0.114	968.568	-0.202	0.189	0.286	0.555	968.568	0.171	0.219	0.434	0.656
PTPRM	912.439	-0.330	0.335	0.325	0.465	912.439	0.589	0.335	0.078	0.370	912.439	0.920	0.387	0.017	0.094
PTPRN	106.865	0.146	1.576	0.926	0.953	106.865	1.183	1.574	0.452	0.680	106.865	1.037	1.818	0.568	0.758
ACTA1	273.896	0.415	1.197	0.729	0.816	273.896	-0.392	1.197	0.743	0.863	273.896	-0.807	1.382	0.559	0.751
BARD1	177.807	-0.803	0.245	0.001	0.006	177.807	-0.609	0.243	0.012	0.305	177.807	0.194	0.284	0.495	0.703
PTPRR	22.966	2.542	0.445	0.000	0.000	22.966	-0.306	0.577	0.596	0.775	22.966	-2.848	0.583	0.000	0.000
PTPRS	2349.554	-0.218	0.278	0.434	0.570	2349.554	0.425	0.278	0.126	0.419	2349.554	0.643	0.321	0.045	0.172
PTS	180.949	0.116	0.348	0.739	0.823	180.949	-0.582	0.350	0.097	0.391	180.949	-0.698	0.404	0.084	0.252
PTX3	852.809	-3.185	1.002	0.001	0.008	852.809	-3.430	1.002	0.001	0.257	852.809	-0.245	1.159	0.832	0.918
BAX	920.580	0.366	0.364	0.314	0.454	920.580	-0.761	0.365	0.037	0.325	920.580	-1.127	0.421	0.007	0.054
RAD1	862.553	-0.751	0.289	0.009	0.032	862.553	-0.755	0.289	0.009	0.297	862.553	-0.004	0.334	0.990	0.995
PURA	691.013	0.316	0.153	0.039	0.095	691.013	0.099	0.153	0.519	0.724	691.013	-0.217	0.177	0.219	0.446
PURB	1116.286	-0.243	0.227	0.284	0.422	1116.286	-0.487	0.227	0.032	0.322	1116.286	-0.244	0.262	0.353	0.586
PTBP2	143.241	-0.008	0.249	0.976	0.985	143.241	-0.168	0.249	0.500	0.713	143.241	-0.160	0.289	0.579	0.764
NFE4	163.457	1.159	0.278	0.000	0.000	163.457	0.433	0.281	0.123	0.415	163.457	-0.726	0.320	0.024	0.113
PVR	3125.516	-0.412	0.380	0.278	0.415	3125.516	-0.913	0.380	0.016	0.306	3125.516	-0.501	0.439	0.254	0.486
NECTIN1	4335.097	0.282	0.348	0.418	0.555	4335.097	0.434	0.348	0.213	0.493	4335.097	0.151	0.402	0.707	0.848
NECTIN2	1161.073	0.603	0.139	0.000	0.000	1161.073	-0.055	0.140	0.694	0.835	1161.073	-0.658	0.161	0.000	0.001
CTDSP1	1065.527	0.119	0.142	0.401	0.540	1065.527	-0.037	0.142	0.795	0.893	1065.527	-0.156	0.164	0.342	0.576
CXCL16	26.526	0.737	0.360	0.040	0.098	26.526	0.245	0.367	0.504	0.715	26.526	-0.492	0.416	0.238	0.467
BBS1	288.616	0.382	0.394	0.332	0.472	288.616	0.459	0.394	0.243	0.519	288.616	0.077	0.455	0.865	0.935
PVT1	18390.768	-0.108	0.108	0.318	0.457	18390.768	0.216	0.108	0.046	0.334	18390.768	0.325	0.125	0.009	0.063
PWP2	398.007	-0.368	0.371	0.322	0.462	398.007	-0.730	0.372	0.050	0.337	398.007	-0.362	0.430	0.399	0.627
PEX19	830.634	-0.282	0.254	0.266	0.402	830.634	0.204	0.253	0.421	0.658	830.634	0.486	0.293	0.097	0.275
ABCD3	603.390	-0.715	0.268	0.008	0.028	603.390	-0.160	0.267	0.549	0.743	603.390	0.555	0.310	0.073	0.232
ABCD4	247.823	0.372	0.218	0.087	0.177	247.823	0.366	0.217	0.091	0.385	247.823	-0.006	0.251	0.982	0.990
PXMP2	382.955	-0.109	0.198	0.582	0.699	382.955	0.027	0.197	0.892	0.946	382.955	0.136	0.229	0.553	0.747
PEX2	507.599	-0.123	0.136	0.368	0.509	507.599	0.028	0.135	0.834	0.914	507.599	0.151	0.157	0.337	0.571
PXN	8876.146	0.222	0.219	0.311	0.451	8876.146	0.396	0.219	0.070	0.359	8876.146	0.174	0.253	0.490	0.700
BBS2	726.945	-0.158	0.147	0.283	0.421	726.945	0.145	0.146	0.321	0.582	726.945	0.303	0.170	0.074	0.234
PEX5	2401.239	-0.200	0.178	0.260	0.395	2401.239	0.300	0.177	0.090	0.384	2401.239	0.500	0.205	0.015	0.084
PYCR1	2759.963	-0.563	0.236	0.017	0.051	2759.963	-0.166	0.236	0.481	0.700	2759.963	0.397	0.272	0.145	0.351
ALDH18A1	3263.321	-0.175	0.158	0.269	0.405	3263.321	-0.202	0.158	0.202	0.486	3263.321	-0.027	0.183	0.884	0.945
PCYT2	830.490	1.382	0.228	0.000	0.000	830.490	-0.468	0.230	0.042	0.330	830.490	-1.850	0.264	0.000	0.000
PYGB	4527.232	-0.871	0.216	0.000	0.001	4527.232	-0.286	0.216	0.185	0.472	4527.232	0.584	0.250	0.019	0.099
PYGL	9.397	-0.480	0.624	0.442	0.578	9.397	-0.186	0.594	0.754	0.868	9.397	0.294	0.722	0.684	0.833
SQOR	1056.372	0.953	0.308	0.002	0.010	1056.372	-0.222	0.309	0.473	0.695	1056.372	-1.175	0.356	0.001	0.013
TP53INP2	157.912	0.961	0.427	0.025	0.067	157.912	0.440	0.429	0.305	0.570	157.912	-0.521	0.493	0.291	0.526
SRPRB	1854.706	-0.384	0.300	0.200	0.327	1854.706	-0.540	0.300	0.072	0.361	1854.706	-0.156	0.346	0.652	0.813
ENOPH1	1011.194	-0.169	0.198	0.392	0.531	1011.194	-0.378	0.198	0.056	0.344	1011.194	-0.208	0.229	0.362	0.594
RHOU	783.555	-0.503	0.345	0.145	0.258	783.555	0.171	0.344	0.619	0.790	783.555	0.674	0.398	0.091	0.264
TRAPPC1	604.671	1.166	0.218	0.000	0.000	604.671	0.147	0.220	0.505	0.715	604.671	-1.020	0.253	0.000	0.001
ZBED5	350.874	-0.044	0.176	0.802	0.869	350.874	-0.125	0.176	0.478	0.698	350.874	-0.081	0.204	0.693	0.839
CREBZF	411.617	-0.321	0.232	0.166	0.286	411.617	0.138	0.230	0.549	0.743	411.617	0.459	0.267	0.086	0.255
PCTP	367.259	0.137	0.194	0.479	0.612	367.259	0.344	0.192	0.073	0.362	367.259	0.207	0.223	0.352	0.586
ABHD17C	166.800	-0.285	0.200	0.153	0.269	166.800	-0.243	0.198	0.220	0.500	166.800	0.043	0.232	0.854	0.930
RPRD1B	466.508	-1.008	0.251	0.000	0.001	466.508	-0.149	0.248	0.548	0.742	466.508	0.859	0.289	0.003	0.028
ZNF71	160.609	0.831	0.204	0.000	0.001	160.609	0.437	0.206	0.034	0.322	160.609	-0.393	0.235	0.094	0.270
ZNF77	53.385	0.007	0.283	0.981	0.989	53.385	-0.303	0.285	0.289	0.557	53.385	-0.309	0.332	0.351	0.584
INIP	338.782	-0.199	0.127	0.116	0.218	338.782	-0.290	0.126	0.022	0.314	338.782	-0.091	0.147	0.538	0.736
JAM2	158.508	0.869	0.949	0.360	0.500	158.508	0.734	0.949	0.439	0.672	158.508	-0.136	1.094	0.901	0.954
LY6G5B	198.171	0.825	0.353	0.020	0.057	198.171	-0.181	0.356	0.611	0.786	198.171	-1.006	0.410	0.014	0.082
PRUNE1	321.366	-0.408	0.188	0.030	0.078	321.366	-0.006	0.185	0.974	0.986	321.366	0.402	0.217	0.063	0.212
ZNF462	604.844	-0.698	0.205	0.001	0.004	604.844	-0.022	0.203	0.912	0.956	604.844	0.676	0.237	0.004	0.037
BBS4	169.990	-0.521	0.220	0.018	0.054	169.990	0.016	0.216	0.941	0.970	169.990	0.536	0.254	0.034	0.144
ZNF250	158.540	0.304	0.229	0.184	0.308	158.540	0.218	0.228	0.341	0.598	158.540	-0.086	0.264	0.744	0.868
ARHGAP22	834.635	0.238	0.272	0.382	0.522	834.635	0.503	0.272	0.065	0.353	834.635	0.264	0.314	0.400	0.627
OSTC	1297.246	0.283	0.142	0.046	0.109	1297.246	0.100	0.142	0.482	0.701	1297.246	-0.183	0.164	0.265	0.499
SCAF1	2545.361	0.412	0.176	0.019	0.056	2545.361	-0.038	0.176	0.827	0.910	2545.361	-0.450	0.203	0.027	0.122

KMT2C	955.371	-0.240	0.231	0.298	0.437	955.371	-0.003	0.230	0.988	0.993	955.371	0.237	0.266	0.374	0.606
CACTIN	639.822	-0.142	0.214	0.507	0.636	639.822	-0.333	0.215	0.120	0.412	639.822	-0.191	0.248	0.442	0.662
EPS15L1	932.422	-0.091	0.106	0.393	0.532	932.422	0.065	0.106	0.538	0.735	932.422	0.156	0.123	0.204	0.428
SELENOK	799.758	0.631	0.245	0.010	0.034	799.758	-0.363	0.247	0.142	0.433	799.758	-0.994	0.284	0.000	0.008
SINHCAF	4589.206	-0.844	0.158	0.000	0.000	4589.206	-0.196	0.158	0.213	0.493	4589.206	0.647	0.182	0.000	0.007
RBM25	1350.292	0.192	0.242	0.427	0.563	1350.292	-0.398	0.242	0.100	0.394	1350.292	-0.590	0.280	0.035	0.145
WIZ	1280.648	-0.048	0.106	0.651	0.756	1280.648	0.057	0.105	0.585	0.768	1280.648	0.105	0.122	0.389	0.617
MID1IP1	54.016	0.443	0.263	0.092	0.184	54.016	0.581	0.258	0.025	0.316	54.016	0.137	0.299	0.646	0.808
ABRACL	354.772	1.252	0.244	0.000	0.000	354.772	-0.356	0.249	0.152	0.445	354.772	-1.609	0.285	0.000	0.000
RRAGD	120.200	1.722	0.557	0.002	0.010	120.200	0.932	0.562	0.097	0.392	120.200	-0.790	0.640	0.217	0.443
LY6G6D	16.161	0.579	0.529	0.274	0.410	16.161	0.414	0.528	0.434	0.667	16.161	-0.165	0.606	0.785	0.893
SNX6	1760.699	-0.318	0.133	0.017	0.051	1760.699	0.070	0.133	0.596	0.775	1760.699	0.389	0.154	0.011	0.071
QARS1	3469.829	-0.185	0.211	0.382	0.521	3469.829	0.304	0.211	0.151	0.443	3469.829	0.489	0.244	0.045	0.172
BCAT1	3862.478	-0.528	0.147	0.000	0.002	3862.478	-0.180	0.147	0.222	0.501	3862.478	0.348	0.170	0.040	0.160
QDPR	523.589	0.087	0.219	0.690	0.786	523.589	-0.393	0.220	0.074	0.364	523.589	-0.480	0.254	0.059	0.202
RAB1A	3215.420	-0.016	0.109	0.885	0.925	3215.420	-0.174	0.109	0.111	0.404	3215.420	-0.158	0.126	0.210	0.435
RAB2A	2386.103	-0.113	0.125	0.366	0.507	2386.103	-0.325	0.125	0.009	0.297	2386.103	-0.212	0.145	0.144	0.349
RGL2	3678.831	0.158	0.199	0.427	0.563	3678.831	0.516	0.199	0.009	0.297	3678.831	0.358	0.230	0.119	0.310
RAB3A	57.129	1.473	0.592	0.013	0.041	57.129	0.910	0.594	0.125	0.417	57.129	-0.563	0.681	0.409	0.635
RAB3B	10.877	0.946	0.500	0.059	0.130	10.877	0.027	0.531	0.960	0.979	10.877	-0.919	0.591	0.120	0.311
RAB3IL1	439.578	0.758	0.311	0.015	0.045	439.578	-0.509	0.314	0.105	0.397	439.578	-1.267	0.361	0.000	0.007
RAB4A	886.370	-0.196	0.288	0.497	0.628	886.370	0.368	0.287	0.201	0.484	886.370	0.563	0.333	0.090	0.263
RAB5A	1374.262	-0.415	0.177	0.019	0.056	1374.262	-0.230	0.177	0.194	0.478	1374.262	0.186	0.205	0.365	0.597
RAB5B	1489.249	0.793	0.302	0.009	0.030	1489.249	0.371	0.302	0.219	0.498	1489.249	-0.422	0.349	0.226	0.454
BCAT2	1417.260	0.590	0.148	0.000	0.001	1417.260	0.290	0.149	0.051	0.338	1417.260	-0.300	0.171	0.080	0.244
RAB6A	2994.442	-0.348	0.157	0.027	0.072	2994.442	0.038	0.157	0.808	0.899	2994.442	0.386	0.182	0.033	0.141
MAP4K2	213.758	0.036	0.193	0.851	0.903	213.758	-0.166	0.193	0.391	0.635	213.758	-0.202	0.224	0.367	0.600
RAB13	3727.592	0.935	0.310	0.003	0.012	3727.592	0.516	0.310	0.096	0.391	3727.592	-0.419	0.358	0.241	0.472
RAB27A	671.263	-0.123	0.124	0.321	0.460	671.263	-0.216	0.124	0.081	0.372	671.263	-0.093	0.144	0.519	0.722
RAB27B	254.476	-0.311	0.334	0.352	0.492	254.476	-0.399	0.333	0.231	0.508	254.476	-0.089	0.386	0.818	0.910
RABGGTA	366.869	0.108	0.130	0.404	0.542	366.869	0.147	0.129	0.254	0.528	366.869	0.038	0.150	0.798	0.899
RABGGTB	2659.274	-0.299	0.116	0.010	0.034	2659.274	0.002	0.116	0.983	0.990	2659.274	0.301	0.134	0.024	0.115
RABIF	118.558	0.244	0.341	0.473	0.607	118.558	-0.425	0.344	0.217	0.496	118.558	-0.670	0.397	0.092	0.265
RAB5C	2299.139	-0.093	0.207	0.655	0.759	2299.139	-0.413	0.207	0.046	0.335	2299.139	-0.321	0.240	0.181	0.399
RAC1	7111.199	0.278	0.174	0.111	0.212	7111.199	-0.027	0.174	0.876	0.937	7111.199	-0.305	0.201	0.130	0.327
RAC2	2106.450	0.887	0.234	0.000	0.001	2106.450	0.390	0.234	0.096	0.391	2106.450	-0.497	0.270	0.066	0.216
RAC3	293.740	0.882	0.257	0.001	0.004	293.740	0.673	0.257	0.009	0.297	293.740	-0.208	0.296	0.481	0.694
RAD9A	464.159	1.010	0.213	0.000	0.000	464.159	0.113	0.215	0.598	0.777	464.159	-0.896	0.247	0.000	0.005
RAD17	1188.043	-0.551	0.124	0.000	0.000	1188.043	0.024	0.123	0.847	0.921	1188.043	0.575	0.143	0.000	0.002
RAD21	5166.898	-0.608	0.101	0.000	0.000	5166.898	-0.069	0.101	0.492	0.707	5166.898	0.539	0.117	0.000	0.000
RAD23A	5088.759	0.018	0.194	0.928	0.955	5088.759	-0.140	0.194	0.470	0.693	5088.759	-0.158	0.224	0.482	0.694
RAD23B	6523.753	-0.634	0.151	0.000	0.000	6523.753	-0.229	0.151	0.129	0.421	6523.753	0.405	0.174	0.020	0.102
RAD51	317.951	-0.811	0.407	0.046	0.109	317.951	-0.838	0.407	0.039	0.327	317.951	-0.027	0.471	0.955	0.979
RAD51C	312.372	-0.307	0.254	0.227	0.358	312.372	-0.332	0.254	0.191	0.476	312.372	-0.025	0.294	0.933	0.970
RAD51B	76.336	-0.046	0.267	0.863	0.911	76.336	0.226	0.262	0.388	0.634	76.336	0.272	0.307	0.375	0.607
MOK	274.783	-0.025	0.178	0.888	0.927	274.783	-0.220	0.178	0.218	0.498	274.783	-0.194	0.207	0.348	0.581
RAD51D	204.186	-0.253	0.228	0.267	0.404	204.186	-0.431	0.228	0.059	0.349	204.186	-0.177	0.265	0.503	0.710
RAD52	140.956	-0.141	0.276	0.608	0.720	140.956	0.368	0.272	0.175	0.464	140.956	0.509	0.317	0.108	0.293
RAF1	2697.621	-0.223	0.099	0.024	0.067	2697.621	0.102	0.099	0.301	0.568	2697.621	0.325	0.114	0.004	0.038
RAG2	19.872	1.023	0.346	0.003	0.014	19.872	0.575	0.354	0.104	0.397	19.872	-0.448	0.393	0.254	0.487
RALA	796.795	0.180	0.147	0.221	0.352	796.795	-0.297	0.148	0.045	0.333	796.795	-0.478	0.171	0.005	0.042
IL22RA1	55.235	0.449	0.453	0.321	0.461	55.235	0.391	0.452	0.387	0.633	55.235	-0.058	0.522	0.911	0.959
PGAP6	768.426	0.377	0.322	0.243	0.376	768.426	-0.558	0.323	0.084	0.376	768.426	-0.935	0.373	0.012	0.075
RALB	910.088	0.121	0.126	0.338	0.478	910.088	0.104	0.126	0.407	0.648	910.088	-0.017	0.146	0.910	0.959
ACTA2	278.089	0.381	0.396	0.336	0.476	278.089	0.888	0.395	0.025	0.316	278.089	0.506	0.457	0.267	0.502
BCHE	56.994	0.109	0.547	0.842	0.896	56.994	0.463	0.544	0.395	0.639	56.994	0.354	0.630	0.574	0.761
RALGDS	763.758	0.804	0.414	0.052	0.119	763.758	0.489	0.414	0.237	0.514	763.758	-0.315	0.478	0.510	0.716
RAN	10540.150	-0.310	0.176	0.078	0.162	10540.150	-0.263	0.176	0.135	0.426	10540.150	0.047	0.203	0.816	0.908
RANBP1	1987.725	-0.905	0.383	0.018	0.053	1987.725	-0.941	0.383	0.014	0.306	1987.725	-0.036	0.442	0.936	0.971
RANBP2	2312.253	-0.756	0.246	0.002	0.010	2312.253	-0.523	0.246	0.033	0.322	2312.253	0.233	0.284	0.412	0.637
RANGAP1	3596.022	-0.822	0.404	0.042	0.101	3596.022	-0.988	0.404	0.014	0.306	3596.022	-0.166	0.466	0.722	0.856
RAP1A	1345.457	-0.212	0.133	0.112	0.213	1345.457	-0.187	0.133	0.159	0.451	1345.457	0.025	0.154	0.873	0.939
RAP1B	2052.231	-0.586	0.271	0.031	0.080	2052.231	-0.663	0.271	0.014	0.306	2052.231	-0.078	0.313	0.804	0.902
ENPP5	19.351	1.381	0.578	0.017	0.051	19.351	0.810	0.585	0.166	0.456	19.351	-0.571	0.661	0.388	0.616
RAP1GAP	11.656	1.316	0.548	0.016	0.049	11.656	1.062	0.551	0.054	0.342	11.656	-0.255	0.599	0.670	0.824
RAP1GDS1	874.542	-0.570	0.136	0.000	0.000	874.542	0.099	0.134	0.458	0.685	874.542	0.669	0.156	0.000	0.001
RAP2A	771.636	-0.514	0.371	0.166	0.286	771.636	0.121	0.370	0.744	0.863	771.636	0.635	0.428	0.138	0.341

RAP2B	1275.487	-0.101	0.126	0.424	0.561	1275.487	-0.277	0.126	0.028	0.321	1275.487	-0.177	0.146	0.225	0.453
RARA	1336.306	0.841	0.231	0.000	0.002	1336.306	0.517	0.231	0.025	0.316	1336.306	-0.324	0.266	0.224	0.451
RARG	277.966	0.626	0.236	0.008	0.028	277.966	0.334	0.236	0.157	0.449	277.966	-0.293	0.272	0.282	0.516
RARS1	1796.950	-0.584	0.164	0.000	0.003	1796.950	-0.225	0.164	0.170	0.459	1796.950	0.360	0.190	0.058	0.201
RARRES2	9.933	1.210	0.590	0.040	0.098	9.933	-0.020	0.644	0.976	0.986	9.933	-1.229	0.705	0.081	0.247
PLAAT4	78.389	3.121	0.475	0.000	0.000	78.389	1.049	0.485	0.031	0.322	78.389	-2.073	0.545	0.000	0.003
RASA1	921.438	-1.267	0.278	0.000	0.000	921.438	-0.121	0.276	0.662	0.816	921.438	1.146	0.320	0.000	0.006
RASA2	243.751	-0.280	0.194	0.149	0.263	243.751	-0.203	0.192	0.291	0.559	243.751	0.077	0.224	0.733	0.862
RASGRF2	27.491	0.811	0.318	0.011	0.036	27.491	-0.036	0.336	0.916	0.958	27.491	-0.846	0.377	0.025	0.116
RB1	798.366	-0.416	0.165	0.011	0.038	798.366	-0.321	0.164	0.051	0.338	798.366	0.095	0.190	0.616	0.789
ARID4A	325.369	0.229	0.154	0.137	0.247	325.369	0.228	0.153	0.135	0.426	325.369	0.000	0.177	0.999	1.000
HIVEP3	55.794	1.093	0.225	0.000	0.000	55.794	-0.055	0.241	0.818	0.905	55.794	-1.149	0.267	0.000	0.001
KDM5A	1174.663	-0.291	0.149	0.051	0.117	1174.663	-0.027	0.149	0.854	0.925	1174.663	0.264	0.173	0.126	0.320
EVA1C	104.105	0.631	0.449	0.160	0.278	104.105	0.795	0.447	0.076	0.364	104.105	0.164	0.516	0.751	0.872
ACE2	28.420	0.088	0.396	0.825	0.885	28.420	0.232	0.389	0.550	0.743	28.420	0.145	0.454	0.750	0.872
TLNRD1	481.049	-0.265	0.236	0.261	0.396	481.049	-0.277	0.235	0.239	0.516	481.049	-0.012	0.273	0.965	0.984
NTN4	665.895	-0.501	0.327	0.126	0.232	665.895	-0.028	0.326	0.931	0.965	665.895	0.473	0.378	0.210	0.436
RBBP4	2432.577	0.147	0.131	0.264	0.400	2432.577	-0.155	0.131	0.239	0.515	2432.577	-0.301	0.152	0.047	0.176
CACNG8	218.593	0.739	0.225	0.001	0.006	218.593	0.006	0.228	0.979	0.988	218.593	-0.732	0.261	0.005	0.041
UBL5	1447.007	0.855	0.251	0.001	0.004	1447.007	-0.139	0.252	0.579	0.764	1447.007	-0.994	0.290	0.001	0.009
RBBP5	440.327	-0.586	0.244	0.016	0.049	440.327	-0.596	0.243	0.014	0.306	440.327	-0.011	0.283	0.970	0.985
BCKDHA	1914.988	0.095	0.429	0.825	0.885	1914.988	0.808	0.429	0.060	0.350	1914.988	0.713	0.495	0.150	0.359
RBBP6	1053.442	0.525	0.205	0.010	0.035	1053.442	0.123	0.205	0.547	0.741	1053.442	-0.401	0.237	0.090	0.262
SIGIRR	141.783	1.077	0.311	0.001	0.004	141.783	0.185	0.315	0.556	0.747	141.783	-0.892	0.360	0.013	0.079
RBBP7	2975.925	-0.520	0.189	0.006	0.022	2975.925	-0.241	0.188	0.201	0.484	2975.925	0.279	0.218	0.200	0.424
RBBP8	1351.569	-1.067	0.341	0.002	0.009	1351.569	-0.832	0.341	0.015	0.306	1351.569	0.235	0.394	0.551	0.745
RBL1	222.591	-0.769	0.317	0.015	0.047	222.591	-0.485	0.315	0.124	0.416	222.591	0.284	0.366	0.438	0.659
PLEKHA1	843.158	-0.514	0.139	0.000	0.002	843.158	-0.152	0.138	0.271	0.542	843.158	0.362	0.161	0.024	0.115
PLEKHA2	1894.411	-0.236	0.269	0.381	0.521	1894.411	0.254	0.269	0.344	0.600	1894.411	0.490	0.311	0.115	0.303
RBL2	1491.258	-0.206	0.276	0.455	0.590	1491.258	0.311	0.275	0.258	0.530	1491.258	0.517	0.318	0.104	0.287
TRPV4	93.092	-0.044	0.428	0.918	0.948	93.092	0.626	0.423	0.139	0.430	93.092	0.670	0.491	0.173	0.390
SCPEP1	1156.344	1.024	0.156	0.000	0.000	1156.344	0.144	0.157	0.359	0.611	1156.344	-0.881	0.180	0.000	0.000
SENP2	677.342	-0.673	0.188	0.000	0.003	677.342	-0.183	0.187	0.327	0.588	677.342	0.490	0.217	0.024	0.114
ALOXE3	30.652	0.054	0.406	0.894	0.931	30.652	-0.166	0.408	0.684	0.829	30.652	-0.220	0.474	0.642	0.806
GNB4	1554.133	-0.684	0.232	0.003	0.014	1554.133	-0.472	0.232	0.042	0.330	1554.133	0.212	0.268	0.430	0.652
ZNF350	92.908	0.345	0.228	0.131	0.240	92.908	0.512	0.224	0.023	0.315	92.908	0.167	0.260	0.521	0.724
KLHL12	610.037	-0.530	0.147	0.000	0.002	610.037	-0.066	0.145	0.647	0.808	610.037	0.464	0.169	0.006	0.047
RBM3	3003.257	-0.195	0.148	0.187	0.311	3003.257	-0.137	0.148	0.355	0.608	3003.257	0.059	0.171	0.732	0.861
RXFP1	30.849	0.621	0.694	0.371	0.511	30.849	0.718	0.691	0.299	0.566	30.849	0.097	0.797	0.903	0.955
RBM4	506.959	-0.465	0.193	0.016	0.049	506.959	-0.209	0.192	0.278	0.548	506.959	0.257	0.224	0.251	0.483
RBMS1	525.526	0.028	0.317	0.929	0.955	525.526	-0.717	0.318	0.024	0.316	525.526	-0.745	0.367	0.042	0.165
RBMS2	1127.528	0.312	0.134	0.020	0.057	1127.528	0.179	0.134	0.181	0.469	1127.528	-0.133	0.154	0.390	0.618
BCKDHB	256.686	-0.879	0.317	0.006	0.021	256.686	0.208	0.313	0.505	0.715	256.686	1.088	0.365	0.003	0.028
SNORA33	9.311	1.147	0.619	0.064	0.139	9.311	-0.432	0.679	0.524	0.727	9.311	-1.579	0.752	0.036	0.148
ZFAT-AS1	16.325	-0.559	0.484	0.248	0.382	16.325	-0.975	0.495	0.049	0.337	16.325	-0.416	0.585	0.477	0.690
CCND1	5917.484	-0.075	0.112	0.500	0.631	5917.484	0.029	0.112	0.797	0.895	5917.484	0.104	0.129	0.420	0.644
SMG1P5	186.614	-0.477	0.330	0.149	0.262	186.614	-0.244	0.328	0.458	0.684	186.614	0.233	0.381	0.541	0.738
PGM5P2	25.984	0.370	0.342	0.279	0.416	25.984	0.033	0.346	0.925	0.962	25.984	-0.338	0.398	0.396	0.624
RCN1	7728.998	0.178	0.131	0.173	0.294	7728.998	0.064	0.131	0.623	0.792	7728.998	-0.114	0.151	0.451	0.670
RCN2	1177.509	-0.340	0.168	0.043	0.104	1177.509	-0.291	0.168	0.084	0.375	1177.509	0.049	0.195	0.800	0.900
BCL2	126.985	-0.420	0.238	0.077	0.161	126.985	-0.172	0.234	0.462	0.688	126.985	0.248	0.275	0.367	0.600
RDX	1099.423	-0.715	0.170	0.000	0.000	1099.423	-0.362	0.169	0.032	0.322	1099.423	0.353	0.196	0.072	0.229
RECQL	881.197	-0.540	0.259	0.037	0.092	881.197	-0.613	0.259	0.018	0.306	881.197	-0.073	0.299	0.807	0.903
REL	311.095	-0.258	0.243	0.289	0.427	311.095	-0.282	0.242	0.244	0.519	311.095	-0.025	0.281	0.930	0.968
RELA	2288.116	0.111	0.148	0.454	0.589	2288.116	0.136	0.148	0.357	0.611	2288.116	0.025	0.171	0.882	0.943
RELB	1040.373	0.305	0.184	0.098	0.193	1040.373	0.007	0.184	0.972	0.985	1040.373	-0.298	0.213	0.162	0.375
UPF1	2474.261	-0.331	0.167	0.048	0.112	2474.261	-0.258	0.167	0.121	0.413	2474.261	0.072	0.193	0.708	0.849
DPF2	1158.205	-0.130	0.137	0.344	0.485	1158.205	0.191	0.137	0.162	0.453	1158.205	0.321	0.158	0.043	0.165
REST	704.746	0.029	0.131	0.825	0.885	704.746	-0.108	0.131	0.408	0.649	704.746	-0.137	0.152	0.365	0.598
BCL2L1	5113.180	-0.021	0.103	0.839	0.894	5113.180	0.080	0.103	0.440	0.672	5113.180	0.101	0.119	0.399	0.626
REV3L	1170.269	-0.401	0.275	0.144	0.257	1170.269	0.009	0.274	0.974	0.986	1170.269	0.410	0.317	0.196	0.419
RFC1	1881.850	-0.548	0.155	0.000	0.003	1881.850	-0.076	0.154	0.623	0.792	1881.850	0.472	0.178	0.008	0.057
RFC2	624.042	-0.688	0.388	0.076	0.159	624.042	-0.991	0.388	0.011	0.299	624.042	-0.303	0.448	0.499	0.706
RFC3	443.988	-1.152	0.390	0.003	0.014	443.988	-1.179	0.389	0.002	0.297	443.988	-0.027	0.451	0.952	0.978
RFC4	789.392	-0.377	0.278	0.175	0.297	789.392	-0.199	0.278	0.474	0.695	789.392	0.178	0.321	0.580	0.764
RFC5	627.533	-0.203	0.237	0.391	0.530	627.533	-0.500	0.238	0.035	0.324	627.533	-0.296	0.275	0.281	0.515
RFNG	444.544	0.574	0.273	0.036	0.089	444.544	0.677	0.272	0.013	0.305	444.544	0.103	0.314	0.744	0.868

TRIM27	5472.086	-0.178	0.184	0.335	0.475	5472.086	-0.081	0.184	0.658	0.814	5472.086	0.096	0.213	0.651	0.812
RFX1	391.977	0.188	0.179	0.295	0.434	391.977	0.178	0.179	0.320	0.581	391.977	-0.010	0.207	0.961	0.982
BCL2L2	571.544	-0.345	0.215	0.108	0.207	571.544	0.165	0.214	0.440	0.672	571.544	0.510	0.248	0.040	0.158
RFX2	652.733	-0.149	0.214	0.485	0.618	652.733	0.307	0.213	0.149	0.442	652.733	0.456	0.247	0.064	0.214
RFX3	65.708	-0.865	0.299	0.004	0.016	65.708	0.320	0.280	0.254	0.527	65.708	1.184	0.340	0.000	0.008
RFX5	1013.748	-0.275	0.139	0.048	0.111	1013.748	-0.097	0.138	0.483	0.701	1013.748	0.178	0.160	0.267	0.501
RFXAP	131.116	-0.930	0.214	0.000	0.000	131.116	-0.711	0.210	0.001	0.258	131.116	0.219	0.250	0.380	0.610
RGS2	181.016	-0.466	0.295	0.114	0.216	181.016	0.085	0.291	0.770	0.878	181.016	0.551	0.340	0.105	0.289
RGS3	320.744	0.187	0.193	0.331	0.471	320.744	0.229	0.192	0.232	0.509	320.744	0.042	0.222	0.850	0.927
ACTB	52861.801	0.795	0.305	0.009	0.032	52861.801	-0.622	0.305	0.042	0.330	52861.801	-1.417	0.353	0.000	0.002
RGS10	390.086	0.551	0.258	0.033	0.084	390.086	0.135	0.258	0.600	0.778	390.086	-0.415	0.298	0.163	0.377
RGS12	121.570	0.141	0.266	0.597	0.711	121.570	0.326	0.263	0.216	0.495	121.570	0.185	0.306	0.545	0.740
RGS16	64.180	-1.102	0.472	0.020	0.057	64.180	-0.126	0.459	0.784	0.886	64.180	0.976	0.543	0.072	0.230
RHCE	38.666	1.562	0.358	0.000	0.000	38.666	-0.309	0.391	0.429	0.664	38.666	-1.871	0.431	0.000	0.000
RHD	12.326	0.946	0.561	0.092	0.184	12.326	0.170	0.581	0.770	0.878	12.326	-0.776	0.654	0.235	0.465
RHEB	2356.832	0.340	0.155	0.029	0.076	2356.832	-0.184	0.156	0.238	0.515	2356.832	-0.524	0.180	0.004	0.032
RING1	4349.686	-0.319	0.262	0.223	0.354	4349.686	-0.221	0.262	0.398	0.642	4349.686	0.098	0.303	0.747	0.870
RIT1	478.341	0.266	0.146	0.067	0.145	478.341	-0.016	0.146	0.913	0.957	478.341	-0.282	0.169	0.094	0.270
RLF	725.240	-0.032	0.224	0.888	0.927	725.240	0.240	0.224	0.282	0.552	725.240	0.272	0.259	0.293	0.528
BCL3	1007.953	0.816	0.302	0.007	0.026	1007.953	-0.118	0.303	0.697	0.837	1007.953	-0.934	0.349	0.008	0.054
RMRP	17.415	0.493	0.509	0.333	0.473	17.415	-0.300	0.526	0.569	0.756	17.415	-0.792	0.601	0.187	0.407
RN7SL1	961.162	-0.267	0.207	0.197	0.323	961.162	0.397	0.206	0.053	0.342	961.162	0.664	0.238	0.005	0.043
AFAP1	1092.325	-0.133	0.189	0.482	0.615	1092.325	-0.078	0.189	0.679	0.827	1092.325	0.055	0.218	0.801	0.901
GPBP1L1	1468.970	-0.307	0.110	0.005	0.021	1468.970	0.008	0.109	0.943	0.971	1468.970	0.314	0.127	0.013	0.079
C12orf10	783.937	0.430	0.216	0.047	0.110	783.937	0.102	0.217	0.639	0.803	783.937	-0.328	0.250	0.190	0.411
FAM3A	632.132	0.477	0.165	0.004	0.016	632.132	0.141	0.166	0.396	0.640	632.132	-0.337	0.191	0.078	0.241
AVPI1	1489.860	0.411	0.285	0.149	0.263	1489.860	0.583	0.285	0.041	0.329	1489.860	0.172	0.329	0.602	0.780
RNASE4	348.914	0.778	0.963	0.419	0.556	348.914	1.069	0.963	0.267	0.538	348.914	0.291	1.111	0.793	0.896
SLC25A19	257.844	-1.003	0.512	0.050	0.116	257.844	-1.223	0.512	0.017	0.306	257.844	-0.221	0.592	0.710	0.849
BCL6	2558.962	0.518	0.247	0.036	0.090	2558.962	0.378	0.247	0.125	0.417	2558.962	-0.140	0.285	0.624	0.794
EDA2R	25.560	-1.508	0.339	0.000	0.000	25.560	-0.221	0.275	0.422	0.658	25.560	1.286	0.379	0.001	0.010
RNASEL	144.398	0.007	0.268	0.980	0.988	144.398	0.067	0.267	0.801	0.896	144.398	0.061	0.310	0.845	0.925
EXOC4	1136.541	-0.684	0.345	0.047	0.110	1136.541	0.361	0.344	0.294	0.562	1136.541	1.045	0.398	0.009	0.059
TGIF2	540.272	-0.356	0.125	0.004	0.018	540.272	0.090	0.123	0.465	0.689	540.272	0.445	0.144	0.002	0.022
RNF2	337.029	0.313	0.146	0.032	0.082	337.029	0.176	0.146	0.228	0.506	337.029	-0.137	0.168	0.415	0.640
BRD2	19084.019	-0.078	0.142	0.582	0.699	19084.019	0.042	0.142	0.768	0.877	19084.019	0.120	0.164	0.464	0.680
RNF4	981.826	-0.427	0.187	0.022	0.063	981.826	-0.227	0.186	0.222	0.501	981.826	0.199	0.216	0.356	0.589
RNF5	5504.511	0.411	0.225	0.067	0.145	5504.511	0.177	0.225	0.430	0.664	5504.511	-0.233	0.259	0.368	0.600
ELOVL5	3067.737	-0.300	0.211	0.155	0.271	3067.737	-0.072	0.211	0.733	0.857	3067.737	0.228	0.244	0.350	0.583
SAV1	718.258	0.409	0.122	0.001	0.005	718.258	-0.054	0.123	0.657	0.813	718.258	-0.464	0.141	0.001	0.014
TRMT11	384.046	-0.319	0.148	0.031	0.080	384.046	-0.241	0.147	0.100	0.394	384.046	0.078	0.171	0.650	0.811
MRPS35	1147.335	-0.502	0.159	0.002	0.008	1147.335	-0.171	0.158	0.280	0.550	1147.335	0.331	0.184	0.071	0.229
APOBEC3G	61.633	0.607	0.438	0.166	0.286	61.633	0.357	0.439	0.416	0.654	61.633	-0.250	0.505	0.621	0.792
RNF6	767.587	-0.785	0.187	0.000	0.000	767.587	-0.462	0.186	0.013	0.305	767.587	0.323	0.216	0.135	0.336
PPCDC	124.641	-0.794	0.296	0.007	0.027	124.641	-0.265	0.290	0.361	0.613	124.641	0.528	0.341	0.122	0.313
NIF3L1	192.539	-0.147	0.233	0.528	0.654	192.539	-0.365	0.234	0.118	0.409	192.539	-0.218	0.271	0.421	0.645
CCDC90B	357.629	-0.037	0.159	0.817	0.880	357.629	-0.026	0.158	0.872	0.935	357.629	0.011	0.183	0.951	0.978
FASTKD5	308.543	-0.115	0.195	0.554	0.676	308.543	-0.553	0.196	0.005	0.297	308.543	-0.438	0.227	0.054	0.193
AASDHPPT	589.855	-0.397	0.154	0.010	0.033	589.855	-0.289	0.153	0.059	0.348	589.855	0.109	0.178	0.541	0.738
BCL7A	331.323	0.304	0.174	0.080	0.166	331.323	0.002	0.175	0.991	0.995	331.323	-0.302	0.202	0.134	0.333
RNH1	6447.725	0.089	0.168	0.596	0.711	6447.725	0.013	0.168	0.941	0.970	6447.725	-0.077	0.194	0.693	0.839
AGBL5	831.581	0.063	0.211	0.767	0.846	831.581	0.355	0.211	0.092	0.387	831.581	0.292	0.244	0.230	0.459
RNPEP	2179.138	0.268	0.119	0.024	0.067	2179.138	0.038	0.119	0.749	0.866	2179.138	-0.230	0.137	0.094	0.270
LDAH	372.660	-0.289	0.152	0.058	0.129	372.660	-0.028	0.151	0.852	0.924	372.660	0.261	0.176	0.138	0.340
ELAC2	808.661	-0.274	0.258	0.290	0.428	808.661	-0.444	0.258	0.086	0.378	808.661	-0.170	0.299	0.569	0.758
GUF1	754.930	-1.252	0.241	0.000	0.000	754.930	-0.466	0.239	0.051	0.338	754.930	0.787	0.278	0.005	0.039
SPCS3	997.833	-0.306	0.261	0.241	0.374	997.833	-0.546	0.261	0.036	0.324	997.833	-0.241	0.302	0.425	0.649
NAA35	780.859	-0.797	0.164	0.000	0.000	780.859	-0.170	0.163	0.295	0.562	780.859	0.626	0.190	0.001	0.013
RINT1	334.557	-0.701	0.237	0.003	0.014	334.557	-0.331	0.235	0.159	0.451	334.557	0.370	0.274	0.177	0.394
ABCE1	2473.791	-0.879	0.223	0.000	0.001	2473.791	-0.491	0.222	0.027	0.319	2473.791	0.388	0.257	0.131	0.329
SCOC	733.182	-0.199	0.128	0.121	0.225	733.182	0.044	0.127	0.731	0.856	733.182	0.242	0.148	0.101	0.282
NBEAP1	187.891	-0.693	0.435	0.111	0.211	187.891	0.342	0.431	0.427	0.663	187.891	1.035	0.501	0.039	0.155
DHX35	135.485	-0.411	0.341	0.229	0.360	135.485	-0.450	0.341	0.187	0.473	135.485	-0.039	0.396	0.922	0.964
RIC8A	1944.117	-0.097	0.175	0.580	0.698	1944.117	-0.297	0.175	0.089	0.383	1944.117	-0.200	0.202	0.322	0.556
CYB5RL	93.628	-0.312	0.244	0.202	0.329	93.628	-0.353	0.243	0.146	0.438	93.628	-0.041	0.284	0.885	0.945
SNORD68	24.458	1.215	0.569	0.033	0.084	24.458	0.154	0.585	0.792	0.892	24.458	-1.061	0.662	0.109	0.295
UBE2MP1	26.131	-0.433	0.363	0.233	0.365	26.131	-0.865	0.372	0.020	0.310	26.131	-0.432	0.439	0.325	0.559

RNU2-1	24.435	-1.440	0.662	0.030	0.077	24.435	-0.951	0.642	0.139	0.430	24.435	0.489	0.769	0.525	0.726
MIIP	267.600	0.552	0.313	0.078	0.162	267.600	-0.288	0.315	0.360	0.612	267.600	-0.840	0.363	0.021	0.103
ATG101	789.138	-0.663	0.407	0.104	0.201	789.138	-0.937	0.407	0.021	0.314	789.138	-0.274	0.471	0.560	0.752
GAS5	10363.201	0.325	0.315	0.302	0.440	10363.201	0.619	0.315	0.049	0.337	10363.201	0.294	0.364	0.419	0.643
PAPPA2	44.725	-1.063	0.416	0.011	0.035	44.725	1.248	0.366	0.001	0.257	44.725	2.311	0.460	0.000	0.000
EEFSEC	517.221	0.029	0.136	0.831	0.889	517.221	-0.008	0.136	0.952	0.976	517.221	-0.037	0.158	0.813	0.907
FKBP10	8611.651	0.503	0.284	0.076	0.159	8611.651	0.572	0.284	0.044	0.331	8611.651	0.069	0.328	0.833	0.919
SMAP1	859.469	0.283	0.258	0.273	0.410	859.469	0.395	0.258	0.126	0.418	859.469	0.112	0.298	0.708	0.848
TRAPPC11	602.416	-0.646	0.192	0.001	0.005	602.416	0.015	0.190	0.938	0.968	602.416	0.660	0.221	0.003	0.028
ZFAND3	2128.954	-0.272	0.172	0.115	0.217	2128.954	-0.004	0.172	0.980	0.989	2128.954	0.267	0.199	0.179	0.397
C14orf93	232.911	0.229	0.362	0.526	0.653	232.911	0.662	0.360	0.066	0.354	232.911	0.433	0.417	0.299	0.534
BCL9	162.251	-0.143	0.222	0.517	0.645	162.251	-0.198	0.221	0.370	0.620	162.251	-0.054	0.257	0.833	0.919
ROBO1	2209.345	-0.339	0.295	0.250	0.384	2209.345	0.246	0.294	0.404	0.646	2209.345	0.585	0.340	0.086	0.255
ROCK1	1388.594	-0.653	0.166	0.000	0.001	1388.594	-0.131	0.166	0.428	0.663	1388.594	0.522	0.192	0.007	0.049
ROM1	83.895	0.739	0.319	0.020	0.059	83.895	0.583	0.319	0.068	0.356	83.895	-0.157	0.366	0.668	0.824
RORA	36.745	0.043	0.407	0.916	0.947	36.745	0.243	0.397	0.541	0.737	36.745	0.200	0.466	0.668	0.824
RORC	20.199	-0.545	1.084	0.615	0.726	20.199	0.807	1.063	0.448	0.677	20.199	1.353	1.242	0.276	0.511
ROS1	2140.544	0.180	0.456	0.693	0.788	2140.544	0.432	0.456	0.343	0.600	2140.544	0.252	0.527	0.632	0.800
HCN2	39.652	1.202	0.334	0.000	0.002	39.652	0.325	0.354	0.358	0.611	39.652	-0.877	0.389	0.024	0.114
RP9	141.161	0.084	0.399	0.833	0.890	141.161	-0.463	0.401	0.248	0.522	141.161	-0.548	0.464	0.237	0.467
RP2	595.925	0.270	0.171	0.114	0.216	595.925	0.073	0.171	0.667	0.819	595.925	-0.196	0.197	0.319	0.555
RPGR	522.807	-1.180	0.400	0.003	0.014	522.807	-1.057	0.400	0.008	0.297	522.807	0.124	0.463	0.789	0.895
OPN1SW	31.509	0.154	0.350	0.659	0.762	31.509	-0.308	0.358	0.390	0.635	31.509	-0.463	0.414	0.264	0.499
RPA1	1655.582	-0.691	0.187	0.000	0.002	1655.582	-0.118	0.186	0.525	0.728	1655.582	0.573	0.216	0.008	0.056
RPA2	689.303	-0.190	0.195	0.331	0.471	689.303	-0.371	0.195	0.058	0.347	689.303	-0.181	0.226	0.423	0.647
RPA3	413.090	-0.007	0.216	0.974	0.984	413.090	-0.178	0.216	0.409	0.649	413.090	-0.171	0.249	0.493	0.702
RPE	989.869	-0.330	0.179	0.065	0.141	989.869	-0.442	0.179	0.013	0.305	989.869	-0.112	0.207	0.588	0.769
RPL3	76690.658	0.289	0.265	0.275	0.412	76690.658	0.425	0.265	0.108	0.401	76690.658	0.136	0.305	0.656	0.816
RPL3L	10.376	0.268	0.674	0.691	0.787	10.376	0.753	0.654	0.249	0.523	10.376	0.485	0.763	0.525	0.726
RPL4	64029.279	0.363	0.269	0.178	0.300	64029.279	0.365	0.269	0.175	0.464	64029.279	0.002	0.311	0.994	0.996
RPL5	37477.928	0.399	0.266	0.134	0.243	37477.928	0.368	0.266	0.168	0.457	37477.928	-0.032	0.308	0.918	0.962
RPL6	31097.253	0.227	0.223	0.308	0.448	31097.253	0.295	0.223	0.186	0.473	31097.253	0.068	0.257	0.793	0.896
RPL7	32999.500	0.089	0.231	0.700	0.793	32999.500	0.317	0.231	0.170	0.459	32999.500	0.228	0.267	0.393	0.621
BCR	1186.695	-0.498	0.288	0.084	0.171	1186.695	-0.539	0.288	0.061	0.350	1186.695	-0.040	0.333	0.903	0.955
RPL7A	49049.653	0.584	0.282	0.038	0.094	49049.653	0.413	0.282	0.143	0.434	49049.653	-0.171	0.326	0.600	0.778
NPIPB13	226.073	1.672	0.339	0.000	0.000	226.073	0.166	0.343	0.628	0.796	226.073	-1.506	0.393	0.000	0.003
RPL8	63526.266	0.276	0.212	0.192	0.317	63526.266	0.329	0.212	0.120	0.412	63526.266	0.052	0.244	0.830	0.917
RPL9	16530.141	0.611	0.275	0.027	0.071	16530.141	0.383	0.275	0.165	0.455	16530.141	-0.228	0.318	0.473	0.687
RPL10	34763.611	0.513	0.293	0.080	0.165	34763.611	0.510	0.293	0.081	0.373	34763.611	-0.003	0.338	0.993	0.996
RPL11	24724.530	0.705	0.279	0.011	0.038	24724.530	0.527	0.279	0.059	0.348	24724.530	-0.178	0.322	0.580	0.764
RPL12	30739.003	0.632	0.331	0.056	0.126	30739.003	0.500	0.331	0.131	0.423	30739.003	-0.132	0.382	0.729	0.859
RPL13	2798.579	0.749	0.313	0.017	0.050	2798.579	0.457	0.313	0.144	0.435	2798.579	-0.292	0.361	0.419	0.643
RPL15	29544.921	0.267	0.247	0.281	0.419	29544.921	0.353	0.247	0.153	0.445	29544.921	0.087	0.286	0.762	0.879
RPL17	4574.039	-0.374	0.211	0.076	0.160	4574.039	0.321	0.211	0.128	0.420	4574.039	0.696	0.244	0.004	0.037
RPL18	24298.994	0.787	0.286	0.006	0.023	24298.994	0.469	0.286	0.101	0.394	24298.994	-0.318	0.330	0.335	0.570
RPL18A	39973.836	0.481	0.329	0.143	0.256	39973.836	0.606	0.329	0.065	0.354	39973.836	0.125	0.379	0.741	0.867
RPL19	24713.303	0.864	0.265	0.001	0.006	24713.303	0.388	0.265	0.144	0.435	24713.303	-0.476	0.306	0.120	0.312
RPL21	15205.555	-0.102	0.265	0.700	0.793	15205.555	0.467	0.265	0.078	0.369	15205.555	0.569	0.306	0.063	0.211
RPL22	20681.327	0.389	0.263	0.139	0.250	20681.327	0.422	0.263	0.109	0.401	20681.327	0.033	0.304	0.914	0.960
RPL23A	18737.248	0.182	0.248	0.463	0.598	18737.248	0.416	0.248	0.093	0.388	18737.248	0.235	0.286	0.412	0.638
MRPL23	1572.137	0.911	0.259	0.000	0.003	1572.137	0.130	0.259	0.616	0.788	1572.137	-0.781	0.299	0.009	0.061
RPL24	21314.104	0.978	0.299	0.001	0.006	21314.104	0.500	0.299	0.095	0.389	21314.104	-0.479	0.345	0.166	0.381
RPL26	3622.262	0.482	0.230	0.036	0.090	3622.262	0.527	0.230	0.022	0.314	3622.262	0.045	0.265	0.866	0.936
RPL27	26065.385	0.345	0.251	0.170	0.291	26065.385	0.461	0.251	0.067	0.354	26065.385	0.116	0.290	0.688	0.836
RPL30	14476.112	0.166	0.242	0.494	0.626	14476.112	0.424	0.242	0.080	0.371	14476.112	0.258	0.280	0.355	0.589
RPL27A	21153.551	0.839	0.290	0.004	0.016	21153.551	0.418	0.290	0.150	0.442	21153.551	-0.421	0.335	0.208	0.433
RPL28	27160.923	0.769	0.326	0.018	0.054	27160.923	0.533	0.326	0.102	0.394	27160.923	-0.236	0.376	0.531	0.730
RPL29	28723.520	0.812	0.309	0.009	0.030	28723.520	0.475	0.309	0.124	0.416	28723.520	-0.337	0.357	0.346	0.580
RPL31	19794.653	0.800	0.298	0.007	0.027	19794.653	0.428	0.298	0.151	0.444	19794.653	-0.372	0.344	0.280	0.514
RPL32	17308.839	0.701	0.298	0.019	0.054	17308.839	0.453	0.298	0.128	0.420	17308.839	-0.248	0.344	0.470	0.684
RPL34	9376.988	0.731	0.289	0.011	0.038	9376.988	0.454	0.289	0.117	0.409	9376.988	-0.278	0.334	0.406	0.633
RPL35A	17242.336	0.430	0.263	0.102	0.198	17242.336	0.461	0.263	0.080	0.371	17242.336	0.031	0.304	0.919	0.962
RPL36AL	3036.574	0.988	0.253	0.000	0.001	3036.574	0.126	0.253	0.618	0.790	3036.574	-0.862	0.292	0.003	0.030
RPL37	24203.224	0.505	0.323	0.118	0.221	24203.224	0.514	0.323	0.112	0.405	24203.224	0.009	0.373	0.981	0.990
RPL37A	15727.018	0.810	0.328	0.014	0.043	15727.018	0.564	0.328	0.086	0.379	15727.018	-0.246	0.379	0.516	0.720
RPL38	9196.205	0.797	0.284	0.005	0.020	9196.205	0.420	0.284	0.140	0.431	9196.205	-0.377	0.328	0.251	0.483
BCS1L	595.693	-0.410	0.225	0.068	0.145	595.693	-0.492	0.224	0.028	0.321	595.693	-0.082	0.260	0.754	0.874

RPL39	8369.890	0.558	0.288	0.053	0.120	8369.890	0.422	0.288	0.143	0.434	8369.890	-0.136	0.332	0.683	0.832
RPL41	25583.574	0.071	0.280	0.801	0.869	25583.574	0.529	0.280	0.059	0.349	25583.574	0.459	0.324	0.157	0.368
RPL36A	1443.595	1.039	0.309	0.001	0.005	1443.595	0.413	0.309	0.181	0.469	1443.595	-0.626	0.356	0.079	0.242
RPLP0	68080.114	0.055	0.266	0.837	0.893	68080.114	0.470	0.266	0.077	0.368	68080.114	0.415	0.307	0.176	0.394
RPLP1	31452.931	0.712	0.269	0.008	0.029	31452.931	0.460	0.269	0.088	0.380	31452.931	-0.253	0.311	0.417	0.642
BCYRN1	1724.479	0.806	0.490	0.100	0.196	1724.479	-0.083	0.490	0.865	0.931	1724.479	-0.889	0.566	0.116	0.305
RPLP2	15456.021	0.853	0.279	0.002	0.011	15456.021	0.434	0.279	0.119	0.412	15456.021	-0.419	0.322	0.194	0.416
MRPL12	271.938	0.640	0.370	0.084	0.172	271.938	-0.052	0.371	0.889	0.945	271.938	-0.691	0.428	0.106	0.291
MRPS12	643.858	-0.237	0.238	0.320	0.460	643.858	-0.417	0.238	0.081	0.371	643.858	-0.179	0.276	0.515	0.719
RPN1	6101.557	0.031	0.231	0.894	0.931	6101.557	-0.453	0.231	0.050	0.337	6101.557	-0.484	0.267	0.070	0.225
RPN2	3019.851	-0.257	0.135	0.057	0.128	3019.851	-0.070	0.135	0.605	0.781	3019.851	0.187	0.156	0.231	0.459
RPS2	81615.107	0.332	0.286	0.245	0.379	81615.107	0.489	0.286	0.087	0.380	81615.107	0.157	0.330	0.634	0.801
RPS3	68108.732	0.531	0.269	0.049	0.114	68108.732	0.438	0.269	0.104	0.397	68108.732	-0.093	0.311	0.766	0.881
RPS3A	28764.676	0.310	0.273	0.256	0.391	28764.676	0.389	0.273	0.154	0.446	28764.676	0.079	0.315	0.802	0.901
RPS4X	42518.890	0.207	0.217	0.342	0.482	42518.890	0.324	0.217	0.136	0.427	42518.890	0.118	0.251	0.639	0.804
SERINC4	141.122	0.003	0.272	0.991	0.995	141.122	0.320	0.269	0.235	0.513	141.122	0.317	0.313	0.312	0.547
FAM229B	121.596	0.564	0.334	0.091	0.182	121.596	0.542	0.333	0.103	0.396	121.596	-0.022	0.384	0.954	0.979
ZNF704	129.458	1.093	0.329	0.001	0.005	129.458	0.282	0.331	0.395	0.639	129.458	-0.811	0.380	0.033	0.139
RPS5	17466.439	-0.093	0.235	0.694	0.788	17466.439	0.386	0.235	0.101	0.394	17466.439	0.478	0.271	0.078	0.241
RPS6	46916.231	0.628	0.279	0.024	0.067	46916.231	0.457	0.279	0.101	0.394	46916.231	-0.171	0.322	0.596	0.775
FAM85A	32.717	0.212	0.493	0.666	0.767	32.717	0.553	0.486	0.255	0.528	32.717	0.341	0.564	0.546	0.741
RPS6KA1	1513.483	0.524	0.125	0.000	0.000	1513.483	-0.008	0.125	0.947	0.974	1513.483	-0.532	0.145	0.000	0.004
RPS6KA2	418.216	-1.420	0.257	0.000	0.000	418.216	0.093	0.252	0.711	0.845	418.216	1.513	0.295	0.000	0.000
RPS6KA3	540.295	-0.500	0.218	0.022	0.061	540.295	-0.364	0.217	0.093	0.388	540.295	0.135	0.252	0.591	0.771
RPS6KB1	726.025	-0.626	0.182	0.001	0.004	726.025	-0.373	0.181	0.039	0.327	726.025	0.252	0.210	0.230	0.459
RPS6KB2	543.613	0.092	0.263	0.726	0.814	543.613	-0.324	0.263	0.219	0.498	543.613	-0.416	0.304	0.172	0.388
RPS7	11670.548	0.522	0.235	0.027	0.072	11670.548	0.336	0.235	0.154	0.446	11670.548	-0.187	0.272	0.493	0.702
RPS8	36291.154	0.724	0.290	0.013	0.041	36291.154	0.439	0.290	0.130	0.422	36291.154	-0.284	0.335	0.396	0.624
RPS9	177632.201	0.547	0.295	0.063	0.138	177632.201	0.572	0.295	0.052	0.341	177632.201	0.025	0.340	0.942	0.974
RPS10	397.018	0.812	0.314	0.010	0.034	397.018	0.620	0.314	0.049	0.337	397.018	-0.192	0.362	0.596	0.775
RPS11	45507.933	0.685	0.298	0.021	0.061	45507.933	0.476	0.298	0.110	0.403	45507.933	-0.209	0.344	0.544	0.740
RPS12	23369.734	0.427	0.281	0.129	0.236	23369.734	0.493	0.281	0.080	0.371	23369.734	0.066	0.325	0.839	0.922
RPS13	13029.435	0.282	0.235	0.230	0.363	13029.435	0.359	0.235	0.127	0.419	13029.435	0.077	0.272	0.777	0.887
RPS14	30934.852	0.716	0.293	0.015	0.045	30934.852	0.497	0.293	0.090	0.384	30934.852	-0.219	0.339	0.518	0.721
RPS15	15669.591	0.800	0.251	0.001	0.008	15669.591	0.407	0.251	0.105	0.398	15669.591	-0.393	0.290	0.176	0.393
RPS15A	12602.656	0.713	0.303	0.019	0.055	12602.656	0.484	0.303	0.110	0.403	12602.656	-0.229	0.350	0.513	0.718
RPS16	34009.828	0.593	0.291	0.042	0.101	34009.828	0.505	0.291	0.083	0.375	34009.828	-0.089	0.336	0.792	0.896
RPS17	27866.434	0.259	0.244	0.288	0.426	27866.434	0.445	0.244	0.068	0.357	27866.434	0.186	0.282	0.509	0.715
BDH1	993.397	0.334	0.149	0.026	0.069	993.397	-0.137	0.150	0.362	0.614	993.397	-0.470	0.173	0.007	0.049
RPS18	239237.825	0.923	0.328	0.005	0.019	239237.825	0.525	0.328	0.109	0.401	239237.825	-0.398	0.378	0.293	0.528
RPS19	18643.648	0.671	0.283	0.018	0.052	18643.648	0.511	0.283	0.071	0.360	18643.648	-0.160	0.327	0.624	0.794
RPS20	19973.091	0.749	0.276	0.007	0.025	19973.091	0.449	0.276	0.104	0.396	19973.091	-0.300	0.318	0.346	0.581
RPS21	9176.212	0.781	0.261	0.003	0.012	9176.212	0.474	0.261	0.069	0.357	9176.212	-0.307	0.301	0.308	0.543
RPS23	29525.607	0.509	0.275	0.065	0.140	29525.607	0.469	0.275	0.088	0.381	29525.607	-0.039	0.318	0.901	0.954
RPS24	19121.394	0.715	0.281	0.011	0.037	19121.394	0.300	0.281	0.286	0.555	19121.394	-0.415	0.325	0.201	0.425
BDKRB1	572.815	1.107	0.396	0.005	0.020	572.815	-0.719	0.400	0.072	0.361	572.815	-1.827	0.460	0.000	0.002
RPS25	23825.061	0.759	0.310	0.014	0.044	23825.061	0.461	0.310	0.137	0.428	23825.061	-0.298	0.357	0.404	0.631
RPS26	6300.260	0.468	0.209	0.026	0.069	6300.260	0.265	0.209	0.206	0.488	6300.260	-0.203	0.242	0.401	0.628
RPS27	12943.995	0.646	0.304	0.034	0.086	12943.995	0.543	0.304	0.074	0.364	12943.995	-0.103	0.351	0.770	0.883
RPS27A	11739.264	-0.043	0.199	0.830	0.888	11739.264	0.349	0.199	0.080	0.371	11739.264	0.391	0.230	0.089	0.261
RPS28	10297.330	0.603	0.296	0.042	0.101	10297.330	0.557	0.296	0.060	0.350	10297.330	-0.046	0.342	0.894	0.950
RPS29	6369.837	0.402	0.290	0.167	0.286	6369.837	0.533	0.290	0.066	0.354	6369.837	0.132	0.335	0.695	0.840
RRAD	36.116	3.664	0.570	0.000	0.000	36.116	1.092	0.622	0.079	0.370	36.116	-2.572	0.640	0.000	0.002
RRAS	2070.318	1.013	0.309	0.001	0.006	2070.318	0.576	0.309	0.063	0.350	2070.318	-0.438	0.357	0.220	0.447
RRBP1	2647.383	0.865	0.195	0.000	0.000	2647.383	-0.330	0.196	0.091	0.386	2647.383	-1.196	0.226	0.000	0.000
RREB1	822.883	-0.442	0.203	0.030	0.078	822.883	0.172	0.202	0.393	0.638	822.883	0.614	0.234	0.009	0.060
BDKRB2	440.253	0.255	0.305	0.403	0.542	440.253	0.341	0.304	0.263	0.535	440.253	0.086	0.352	0.806	0.903
RRM1	2229.857	-0.721	0.246	0.003	0.014	2229.857	-0.473	0.245	0.054	0.342	2229.857	0.249	0.284	0.381	0.610
RRM2	1962.832	-1.052	0.336	0.002	0.009	1962.832	-0.807	0.336	0.016	0.306	1962.832	0.246	0.388	0.527	0.727
RTKN	258.546	0.197	0.225	0.381	0.521	258.546	0.229	0.224	0.305	0.571	258.546	0.033	0.259	0.900	0.953
CLIP1	2516.306	-0.664	0.201	0.001	0.006	2516.306	-0.065	0.201	0.744	0.863	2516.306	0.598	0.232	0.010	0.066
RSU1	900.921	0.193	0.225	0.390	0.529	900.921	-0.485	0.225	0.032	0.322	900.921	-0.678	0.260	0.009	0.062
RTN1	625.307	-1.731	0.169	0.000	0.000	625.307	-0.200	0.162	0.215	0.494	625.307	1.531	0.193	0.000	0.000
RTN2	158.594	0.560	0.294	0.057	0.127	158.594	0.465	0.294	0.113	0.405	158.594	-0.095	0.339	0.778	0.888
RXRA	5190.042	-0.128	0.257	0.617	0.728	5190.042	0.357	0.257	0.164	0.454	5190.042	0.486	0.296	0.101	0.283
RXRB	4833.729	0.063	0.163	0.700	0.793	4833.729	0.330	0.163	0.043	0.330	4833.729	0.267	0.188	0.155	0.367
RYK	1074.057	-0.296	0.124	0.017	0.050	1074.057	-0.061	0.123	0.619	0.790	1074.057	0.235	0.143	0.101	0.281

RYR2	41.374	-1.084	0.579	0.061	0.134	41.374	0.437	0.557	0.433	0.667	41.374	1.521	0.661	0.021	0.106
RYR3	8.742	0.251	0.683	0.714	0.805	8.742	-0.019	0.688	0.978	0.988	8.742	-0.270	0.796	0.735	0.863
BDNF	13.731	0.409	0.417	0.327	0.467	13.731	0.249	0.416	0.550	0.743	13.731	-0.160	0.480	0.739	0.866
S100A1	21.824	0.741	0.444	0.095	0.189	21.824	0.766	0.439	0.081	0.372	21.824	0.025	0.502	0.960	0.981
SORT1	892.098	1.053	0.389	0.007	0.025	892.098	0.522	0.389	0.180	0.468	892.098	-0.532	0.449	0.237	0.466
S100A2	3650.895	0.550	0.209	0.009	0.030	3650.895	0.010	0.209	0.963	0.980	3650.895	-0.540	0.242	0.025	0.118
S100A3	108.334	1.607	0.311	0.000	0.000	108.334	0.596	0.319	0.062	0.350	108.334	-1.011	0.358	0.005	0.040
S100A4	288.037	0.739	0.426	0.083	0.170	288.037	0.360	0.427	0.399	0.642	288.037	-0.378	0.492	0.442	0.662
S100A6	10632.953	1.268	0.277	0.000	0.000	10632.953	0.359	0.277	0.196	0.479	10632.953	-0.909	0.320	0.004	0.038
S100A8	11.922	2.660	0.902	0.003	0.014	11.922	0.224	0.998	0.822	0.907	11.922	-2.435	1.068	0.023	0.109
S100A9	96.512	2.358	0.450	0.000	0.000	96.512	1.662	0.454	0.000	0.211	96.512	-0.696	0.509	0.172	0.388
S100A10	5292.186	1.049	0.287	0.000	0.002	5292.186	0.371	0.287	0.196	0.480	5292.186	-0.679	0.331	0.040	0.160
S100A11	3406.346	1.417	0.284	0.000	0.000	3406.346	-0.122	0.284	0.667	0.819	3406.346	-1.539	0.328	0.000	0.000
S100A13	828.027	0.210	0.157	0.179	0.302	828.027	0.033	0.157	0.834	0.915	828.027	-0.178	0.181	0.327	0.561
S100P	110.942	0.015	0.250	0.954	0.972	110.942	0.328	0.245	0.180	0.468	110.942	0.314	0.286	0.273	0.508
SAA1	16398.229	2.503	0.396	0.000	0.000	16398.229	0.947	0.396	0.017	0.306	16398.229	-1.556	0.458	0.001	0.010
SAA2	4077.006	4.016	0.461	0.000	0.000	4077.006	1.810	0.461	0.000	0.101	4077.006	-2.205	0.531	0.000	0.001
CFB	11905.199	2.156	0.508	0.000	0.000	11905.199	1.162	0.508	0.022	0.315	11905.199	-0.993	0.587	0.091	0.263
SAA4	22.975	3.365	0.688	0.000	0.000	22.975	0.649	0.734	0.376	0.624	22.975	-2.715	0.795	0.001	0.010
VPS52	2507.188	-0.068	0.129	0.599	0.712	2507.188	0.117	0.129	0.363	0.615	2507.188	0.185	0.149	0.214	0.440
SAFB	3228.277	0.086	0.168	0.608	0.720	3228.277	-0.056	0.168	0.740	0.861	3228.277	-0.142	0.194	0.465	0.681
ACSM3	159.616	-0.032	0.373	0.932	0.957	159.616	0.389	0.371	0.294	0.561	159.616	0.421	0.430	0.327	0.562
MAPK12	210.183	-0.002	0.292	0.993	0.996	210.183	-0.515	0.293	0.079	0.370	210.183	-0.513	0.339	0.130	0.327
SARS1	4472.586	-0.928	0.206	0.000	0.000	4472.586	-0.102	0.206	0.618	0.790	4472.586	0.825	0.238	0.001	0.008
TSPAN31	2028.586	0.612	0.185	0.001	0.006	2028.586	0.069	0.185	0.712	0.845	2028.586	-0.544	0.214	0.011	0.070
SLC22A23	177.124	0.746	0.197	0.000	0.001	177.124	0.200	0.199	0.315	0.578	177.124	-0.546	0.228	0.016	0.090
SAT1	1289.004	0.261	0.353	0.460	0.594	1289.004	-0.295	0.354	0.405	0.646	1289.004	-0.556	0.408	0.173	0.390
BCORL1	147.752	0.293	0.373	0.432	0.569	147.752	0.716	0.370	0.053	0.342	147.752	0.424	0.428	0.323	0.557
SATB1	805.908	-0.582	0.386	0.131	0.240	805.908	0.496	0.385	0.198	0.481	805.908	1.078	0.445	0.015	0.087
SBF1	2508.715	-0.125	0.100	0.212	0.341	2508.715	0.039	0.100	0.697	0.837	2508.715	0.164	0.116	0.156	0.367
MSMO1	1398.705	3.014	0.138	0.000	0.000	1398.705	0.016	0.143	0.910	0.955	1398.705	-2.997	0.162	0.000	0.000
SC5D	752.120	1.065	0.149	0.000	0.000	752.120	-0.223	0.152	0.143	0.435	752.120	-1.288	0.174	0.000	0.000
BFSP1	19.943	0.426	0.496	0.391	0.530	19.943	0.433	0.491	0.378	0.625	19.943	0.008	0.567	0.989	0.994
ATXN1	307.830	0.161	0.313	0.607	0.720	307.830	0.318	0.312	0.309	0.574	307.830	0.157	0.361	0.664	0.821
ATXN2	1092.872	-0.328	0.146	0.025	0.068	1092.872	-0.150	0.146	0.302	0.568	1092.872	0.177	0.169	0.294	0.529
ATXN7	444.711	-0.544	0.189	0.004	0.017	444.711	-0.243	0.187	0.194	0.478	444.711	0.301	0.218	0.169	0.384
SERPINB3	29.318	2.497	0.685	0.000	0.002	29.318	-2.950	1.069	0.006	0.297	29.318	-5.447	1.122	0.000	0.000
SERPINB4	62.761	4.239	0.579	0.000	0.000	62.761	-0.296	0.704	0.674	0.824	62.761	-4.535	0.729	0.000	0.000
SCD	13723.594	1.258	0.259	0.000	0.000	13723.594	0.150	0.259	0.564	0.752	13723.594	-1.109	0.299	0.000	0.004
CLEC11A	24.278	1.131	0.343	0.001	0.006	24.278	0.602	0.355	0.090	0.384	24.278	-0.529	0.388	0.172	0.389
SCML1	341.144	-0.146	0.187	0.436	0.572	341.144	-0.162	0.186	0.384	0.630	341.144	-0.017	0.216	0.938	0.972
SCN1B	24.357	2.323	0.321	0.000	0.000	24.357	0.031	0.385	0.937	0.968	24.357	-2.292	0.395	0.000	0.000
SCN3A	13.069	4.923	0.706	0.000	0.000	13.069	1.282	0.819	0.118	0.409	13.069	-3.641	0.760	0.000	0.000
SCN9A	20.743	0.199	0.630	0.752	0.835	20.743	0.325	0.625	0.603	0.781	20.743	0.126	0.725	0.862	0.934
SCNN1A	1639.743	-0.027	0.438	0.951	0.970	1639.743	0.638	0.438	0.146	0.438	1639.743	0.665	0.506	0.189	0.410
SCNN1B	921.857	-0.848	0.971	0.383	0.522	921.857	1.202	0.970	0.215	0.494	921.857	2.050	1.121	0.067	0.220
SCNN1D	10.644	1.195	0.773	0.122	0.227	10.644	0.472	0.787	0.549	0.743	10.644	-0.723	0.891	0.417	0.642
CEACAM1	10.733	2.552	0.557	0.000	0.000	10.733	0.233	0.643	0.717	0.848	10.733	-2.319	0.667	0.001	0.008
SCNN1G	910.325	-0.779	0.859	0.365	0.505	910.325	1.206	0.858	0.160	0.451	910.325	1.985	0.991	0.045	0.171
SCO1	405.059	-0.545	0.243	0.025	0.068	405.059	-0.406	0.242	0.093	0.388	405.059	0.139	0.281	0.620	0.792
SCP2	1225.560	0.225	0.200	0.260	0.396	1225.560	0.004	0.200	0.983	0.990	1225.560	-0.221	0.231	0.339	0.573
CCL5	1150.183	5.963	0.711	0.000	0.000	1150.183	0.983	0.715	0.169	0.459	1150.183	-4.980	0.821	0.000	0.000
BICD1	424.728	-0.920	0.302	0.002	0.011	424.728	-0.680	0.301	0.024	0.316	424.728	0.240	0.349	0.492	0.702
CCL20	122.902	-0.117	1.012	0.908	0.941	122.902	-2.028	1.030	0.049	0.337	122.902	-1.911	1.186	0.107	0.292
BID	1465.879	0.348	0.171	0.041	0.099	1465.879	-0.192	0.171	0.263	0.535	1465.879	-0.540	0.197	0.006	0.048
CXCL11	23.754	3.932	0.525	0.000	0.000	23.754	1.292	0.573	0.024	0.316	23.754	-2.640	0.579	0.000	0.000
CXCL5	121.134	0.477	0.228	0.037	0.091	121.134	0.480	0.227	0.034	0.322	121.134	0.004	0.262	0.989	0.994
BIK	28.773	1.302	0.337	0.000	0.001	28.773	0.675	0.350	0.054	0.342	28.773	-0.627	0.382	0.100	0.281
SDC1	3867.729	0.278	0.208	0.182	0.305	3867.729	0.028	0.208	0.892	0.946	3867.729	-0.250	0.241	0.298	0.534
SRR	497.798	-0.164	0.188	0.382	0.522	497.798	-0.263	0.187	0.161	0.452	497.798	-0.099	0.217	0.649	0.811
BCAN	265.972	-0.341	0.208	0.101	0.197	265.972	-0.124	0.206	0.549	0.743	265.972	0.218	0.240	0.365	0.598
SDC2	1239.943	0.453	0.176	0.010	0.035	1239.943	0.190	0.177	0.281	0.551	1239.943	-0.263	0.204	0.198	0.421
SDC4	11894.689	1.070	0.191	0.000	0.000	11894.689	0.379	0.191	0.047	0.335	11894.689	-0.690	0.220	0.002	0.019
SDCBP	5483.967	0.136	0.179	0.449	0.584	5483.967	0.084	0.179	0.638	0.803	5483.967	-0.051	0.207	0.804	0.902
CXCL12	41.348	2.818	0.448	0.000	0.000	41.348	-1.879	0.562	0.001	0.279	41.348	-4.697	0.606	0.000	0.000
ABHD4	530.375	0.770	0.367	0.036	0.090	530.375	0.601	0.367	0.102	0.394	530.375	-0.170	0.423	0.689	0.836
MRPL17	1264.249	0.212	0.284	0.455	0.590	1264.249	-0.493	0.284	0.083	0.374	1264.249	-0.705	0.328	0.032	0.137

PKNOX2	2929.660	-0.258	0.088	0.003	0.014	2929.660	-0.047	0.087	0.593	0.774	2929.660	0.211	0.101	0.037	0.151
FAM204A	602.837	-0.168	0.217	0.440	0.576	602.837	-0.210	0.217	0.333	0.592	602.837	-0.042	0.251	0.867	0.936
SDF2	454.876	0.328	0.118	0.005	0.021	454.876	-0.165	0.120	0.169	0.459	454.876	-0.493	0.138	0.000	0.006
SDHA	6583.336	-0.306	0.107	0.004	0.017	6583.336	0.019	0.107	0.860	0.929	6583.336	0.325	0.123	0.008	0.058
RNF123	812.476	-0.299	0.142	0.034	0.087	812.476	-0.003	0.141	0.985	0.991	812.476	0.297	0.163	0.069	0.224
THADA	452.474	-0.750	0.165	0.000	0.000	452.474	-0.280	0.162	0.085	0.376	452.474	0.471	0.190	0.013	0.079
UBE2O	1407.832	0.408	0.169	0.016	0.049	1407.832	0.227	0.169	0.181	0.469	1407.832	-0.182	0.196	0.353	0.586
VIPAS39	424.602	-0.501	0.208	0.016	0.048	424.602	0.163	0.205	0.426	0.662	424.602	0.664	0.239	0.005	0.043
HEATR6	465.220	-0.457	0.111	0.000	0.000	465.220	0.062	0.108	0.567	0.754	465.220	0.519	0.127	0.000	0.001
SH2D4A	266.507	-0.582	0.222	0.009	0.030	266.507	-0.472	0.220	0.032	0.322	266.507	0.110	0.257	0.669	0.824
NSUN3	224.572	-0.391	0.139	0.005	0.019	224.572	-0.124	0.135	0.357	0.611	224.572	0.267	0.160	0.096	0.273
PRDM1	224.621	0.474	0.281	0.092	0.184	224.621	0.162	0.282	0.565	0.753	224.621	-0.312	0.325	0.337	0.572
SDHB	1447.511	-0.306	0.179	0.088	0.178	1447.511	-0.260	0.179	0.146	0.438	1447.511	0.046	0.207	0.825	0.914
FAM111A	772.047	0.026	0.139	0.853	0.904	772.047	0.130	0.139	0.349	0.604	772.047	0.104	0.161	0.517	0.721
MANBAL	850.844	0.016	0.144	0.912	0.944	850.844	-0.238	0.144	0.099	0.393	850.844	-0.253	0.166	0.128	0.324
GPATCH3	238.217	0.110	0.173	0.525	0.652	238.217	-0.175	0.174	0.313	0.576	238.217	-0.285	0.201	0.156	0.368
NAPB	145.066	-0.145	0.243	0.550	0.673	145.066	-0.092	0.241	0.703	0.840	145.066	0.053	0.281	0.850	0.927
SDHC	1671.328	0.008	0.150	0.955	0.973	1671.328	-0.014	0.150	0.923	0.961	1671.328	-0.023	0.173	0.895	0.950
SLC17A9	508.882	0.740	0.296	0.012	0.040	508.882	-0.194	0.297	0.514	0.720	508.882	-0.934	0.343	0.006	0.049
BLOC1S5	214.990	-0.216	0.182	0.234	0.367	214.990	0.317	0.177	0.073	0.362	214.990	0.533	0.208	0.010	0.068
ELMO2	239.450	0.722	0.157	0.000	0.000	239.450	-0.122	0.161	0.449	0.678	239.450	-0.844	0.183	0.000	0.000
GALNT11	381.209	0.094	0.176	0.596	0.711	381.209	0.097	0.176	0.581	0.764	381.209	0.004	0.204	0.986	0.992
SDHD	598.905	0.301	0.188	0.109	0.209	598.905	-0.140	0.189	0.459	0.685	598.905	-0.441	0.218	0.043	0.166
ZBED8	70.636	0.125	0.313	0.690	0.786	70.636	0.155	0.311	0.618	0.790	70.636	0.030	0.361	0.933	0.970
CHTF18	348.536	0.200	0.292	0.494	0.625	348.536	-0.196	0.292	0.501	0.714	348.536	-0.396	0.337	0.240	0.471
CIDEC	25.929	0.339	1.012	0.738	0.823	25.929	0.400	1.010	0.692	0.834	25.929	0.061	1.167	0.958	0.981
ZNF335	285.627	-0.376	0.192	0.050	0.116	285.627	-0.175	0.190	0.358	0.611	285.627	0.202	0.222	0.364	0.596
ANKEF1	184.715	-0.096	0.230	0.675	0.774	184.715	0.160	0.228	0.481	0.700	184.715	0.257	0.265	0.332	0.567
XPNPEP3	233.825	0.335	0.144	0.020	0.058	233.825	0.045	0.145	0.755	0.869	233.825	-0.290	0.167	0.083	0.250
MRPS14	317.936	-0.226	0.139	0.103	0.200	317.936	-0.050	0.137	0.716	0.848	317.936	0.177	0.160	0.270	0.505
CXorf56	472.098	-0.342	0.146	0.019	0.056	472.098	-0.215	0.145	0.138	0.429	472.098	0.128	0.169	0.450	0.669
MCUR1	525.841	0.030	0.200	0.881	0.923	525.841	-0.348	0.200	0.083	0.374	525.841	-0.378	0.232	0.103	0.286
PCIF1	679.328	-0.099	0.127	0.438	0.574	679.328	0.144	0.126	0.253	0.527	679.328	0.243	0.147	0.098	0.277
FAM217B	132.394	-0.288	0.221	0.192	0.317	132.394	-0.101	0.217	0.643	0.805	132.394	0.187	0.255	0.463	0.679
GPSM3	30.460	0.595	0.394	0.131	0.239	30.460	-0.122	0.407	0.763	0.874	30.460	-0.717	0.463	0.121	0.313
NECAB3	247.951	0.532	0.164	0.001	0.007	247.951	0.353	0.164	0.032	0.322	247.951	-0.179	0.189	0.344	0.578
FKBPL	406.181	-0.044	0.171	0.799	0.868	406.181	0.101	0.170	0.554	0.745	406.181	0.144	0.198	0.465	0.681
SEC13	1630.842	0.097	0.243	0.690	0.786	1630.842	-0.466	0.243	0.055	0.343	1630.842	-0.563	0.281	0.045	0.171
CLSPN	246.047	-0.115	0.594	0.847	0.899	246.047	-1.955	0.599	0.001	0.297	246.047	-1.840	0.690	0.008	0.055
SEC14L1	3229.755	-0.565	0.162	0.000	0.003	3229.755	-0.278	0.162	0.086	0.378	3229.755	0.287	0.187	0.125	0.319
KIF13A	22.584	0.856	0.501	0.087	0.177	22.584	0.049	0.514	0.924	0.962	22.584	-0.808	0.584	0.167	0.382
PRDM15	150.026	-0.137	0.248	0.580	0.698	150.026	-0.129	0.246	0.601	0.779	150.026	0.008	0.287	0.977	0.988
FIGNL1	112.344	-0.799	0.344	0.020	0.058	112.344	-0.684	0.341	0.045	0.334	112.344	0.116	0.399	0.772	0.884
SECTM1	899.267	0.317	0.174	0.068	0.146	899.267	0.235	0.173	0.176	0.465	899.267	-0.082	0.200	0.682	0.832
TRAPPC2	274.314	0.093	0.181	0.607	0.720	274.314	0.333	0.179	0.064	0.350	274.314	0.240	0.209	0.250	0.483
SEL1L	1551.590	-0.676	0.283	0.017	0.050	1551.590	-0.045	0.282	0.873	0.936	1551.590	0.631	0.326	0.053	0.190
SEMA3F	1939.092	0.578	0.402	0.150	0.264	1939.092	0.638	0.402	0.112	0.405	1939.092	0.059	0.464	0.898	0.952
TSPYL2	317.512	-0.505	0.443	0.254	0.388	317.512	0.329	0.441	0.455	0.682	317.512	0.834	0.511	0.102	0.285
RBM26	692.772	-0.665	0.207	0.001	0.007	692.772	-0.413	0.206	0.045	0.334	692.772	0.252	0.239	0.292	0.527
PRSS22	13.734	0.672	0.444	0.130	0.238	13.734	0.383	0.448	0.393	0.638	13.734	-0.290	0.509	0.569	0.758
PERP	4806.995	0.265	0.269	0.324	0.464	4806.995	0.254	0.269	0.346	0.602	4806.995	-0.012	0.311	0.970	0.985
CDH23	9.321	0.559	0.991	0.573	0.692	9.321	1.192	0.976	0.222	0.501	9.321	0.634	1.128	0.574	0.761
C19orf33	932.349	0.848	0.332	0.011	0.036	932.349	0.556	0.332	0.094	0.388	932.349	-0.292	0.383	0.446	0.665
LHPP	307.240	0.716	0.443	0.106	0.204	307.240	0.560	0.443	0.206	0.488	307.240	-0.155	0.511	0.761	0.879
RBKS	50.374	0.731	0.285	0.010	0.035	50.374	0.533	0.286	0.062	0.350	50.374	-0.198	0.326	0.545	0.741
PBLD	55.814	0.094	0.321	0.769	0.847	55.814	0.271	0.317	0.393	0.637	55.814	0.176	0.369	0.633	0.800
GOLPH3	2676.936	-0.242	0.145	0.096	0.190	2676.936	-0.312	0.145	0.032	0.322	2676.936	-0.070	0.168	0.675	0.828
MCCC2	2055.899	-0.418	0.165	0.011	0.037	2055.899	-0.300	0.164	0.068	0.356	2055.899	0.118	0.190	0.535	0.733
SNX16	175.617	0.042	0.181	0.817	0.880	175.617	-0.028	0.180	0.876	0.937	175.617	-0.070	0.209	0.739	0.866
GAL3ST2	7.577	0.459	0.808	0.570	0.689	7.577	1.038	0.782	0.184	0.471	7.577	0.579	0.908	0.524	0.725
SAMSN1	994.086	0.195	0.194	0.315	0.454	994.086	-0.107	0.194	0.582	0.766	994.086	-0.302	0.224	0.178	0.396
SMOC1	982.847	-0.723	0.200	0.000	0.002	982.847	-0.216	0.199	0.276	0.547	982.847	0.507	0.231	0.028	0.126
EPB41L4A	89.626	0.502	0.254	0.049	0.113	89.626	0.328	0.254	0.198	0.481	89.626	-0.174	0.293	0.553	0.746
BLM	183.454	-1.061	0.451	0.019	0.054	183.454	-1.438	0.452	0.001	0.297	183.454	-0.377	0.523	0.471	0.685
CENPK	330.806	-0.918	0.348	0.008	0.030	330.806	-0.650	0.347	0.061	0.350	330.806	0.268	0.403	0.506	0.712
RTP4	45.335	2.129	0.534	0.000	0.001	45.335	0.436	0.548	0.426	0.662	45.335	-1.693	0.618	0.006	0.047
CRLF2	246.070	1.839	0.736	0.012	0.040	246.070	1.410	0.736	0.055	0.343	246.070	-0.429	0.846	0.612	0.786

MAGEF1	552.481	0.322	0.205	0.117	0.220	552.481	-0.157	0.206	0.447	0.677	552.481	-0.479	0.238	0.044	0.169
MOAP1	910.305	-0.210	0.158	0.183	0.307	910.305	-0.010	0.157	0.949	0.974	910.305	0.200	0.182	0.272	0.507
TMBIM1	2192.788	0.738	0.116	0.000	0.000	2192.788	0.212	0.116	0.069	0.357	2192.788	-0.526	0.134	0.000	0.002
VSIR	2087.452	-0.452	0.477	0.343	0.483	2087.452	0.597	0.476	0.210	0.491	2087.452	1.050	0.550	0.056	0.198
SLC39A8	8739.922	1.817	0.179	0.000	0.000	8739.922	0.214	0.179	0.231	0.508	8739.922	-1.603	0.206	0.000	0.000
DUS1L	1058.266	0.299	0.338	0.376	0.516	1058.266	-0.459	0.338	0.175	0.464	1058.266	-0.758	0.391	0.052	0.188
RRAGC	883.725	0.758	0.160	0.000	0.000	883.725	0.065	0.161	0.686	0.831	883.725	-0.693	0.186	0.000	0.004
FN3K	207.532	1.654	0.818	0.043	0.103	207.532	0.790	0.819	0.335	0.594	207.532	-0.864	0.944	0.360	0.593
NOD2	70.352	1.659	0.688	0.016	0.048	70.352	0.534	0.698	0.444	0.675	70.352	-1.124	0.794	0.157	0.368
TINAGL1	1317.810	1.038	0.165	0.000	0.000	1317.810	0.147	0.166	0.375	0.623	1317.810	-0.891	0.191	0.000	0.000
SMG1P1	223.327	-0.706	0.341	0.038	0.094	223.327	-0.097	0.337	0.774	0.879	223.327	0.609	0.393	0.121	0.312
LIN7B	40.921	1.488	0.368	0.000	0.001	40.921	-0.322	0.394	0.413	0.652	40.921	-1.811	0.439	0.000	0.001
XYLT1	8.343	-0.964	0.629	0.126	0.232	8.343	-0.817	0.606	0.178	0.466	8.343	0.147	0.746	0.844	0.924
XYLT2	803.095	0.500	0.162	0.002	0.010	803.095	0.371	0.162	0.022	0.314	803.095	-0.129	0.186	0.488	0.698
ZNF674	61.610	-0.801	0.309	0.010	0.033	61.610	-0.232	0.296	0.433	0.667	61.610	0.569	0.356	0.110	0.295
IFIH1	313.789	0.954	0.225	0.000	0.000	313.789	0.301	0.227	0.185	0.472	313.789	-0.653	0.260	0.012	0.074
SLC7A11-AS1	273.513	-3.114	0.593	0.000	0.000	273.513	-0.863	0.583	0.139	0.430	273.513	2.251	0.683	0.001	0.013
ACOT1	202.418	1.004	0.248	0.000	0.001	202.418	0.230	0.251	0.360	0.612	202.418	-0.774	0.288	0.007	0.052
SELENOP	296.790	-0.466	0.603	0.440	0.576	296.790	0.583	0.602	0.333	0.592	296.790	1.049	0.696	0.132	0.330
RBSN	611.947	-0.967	0.244	0.000	0.001	611.947	-0.251	0.242	0.299	0.566	611.947	0.716	0.281	0.011	0.070
PDF	155.674	-1.157	0.416	0.005	0.021	155.674	-0.719	0.412	0.081	0.372	155.674	0.437	0.481	0.363	0.595
TSC22D1-AS1	93.323	-0.617	0.530	0.244	0.377	93.323	0.366	0.524	0.485	0.702	93.323	0.984	0.610	0.107	0.291
KIF9	167.560	-0.841	0.237	0.000	0.003	167.560	-0.372	0.232	0.109	0.401	167.560	0.469	0.274	0.087	0.257
C17orf75	295.586	-0.409	0.147	0.005	0.021	295.586	-0.053	0.144	0.714	0.847	295.586	0.356	0.169	0.035	0.147
SELENOW	432.355	0.907	0.213	0.000	0.000	432.355	-0.119	0.215	0.581	0.765	432.355	-1.026	0.247	0.000	0.001
NCAPG	1173.421	-0.681	0.194	0.000	0.003	1173.421	-0.256	0.194	0.186	0.473	1173.421	0.424	0.224	0.059	0.202
KC6	51.834	-0.355	0.359	0.322	0.462	51.834	0.283	0.349	0.418	0.655	51.834	0.638	0.411	0.120	0.312
LEF1-AS1	22.092	-0.607	0.506	0.230	0.362	22.092	-0.538	0.499	0.281	0.551	22.092	0.069	0.590	0.907	0.957
MAP2K4	251.595	-0.298	0.151	0.049	0.114	251.595	-0.194	0.149	0.195	0.479	251.595	0.104	0.176	0.552	0.746
SNHG6	2739.771	0.683	0.336	0.042	0.101	2739.771	0.549	0.336	0.102	0.395	2739.771	-0.134	0.388	0.729	0.859
TMEM91	53.893	1.090	0.492	0.027	0.072	53.893	0.432	0.496	0.384	0.630	53.893	-0.658	0.568	0.246	0.478
ERAP2	1079.542	0.733	0.193	0.000	0.001	1079.542	0.112	0.193	0.561	0.750	1079.542	-0.620	0.223	0.005	0.043
NECAB1	280.640	-0.037	0.158	0.814	0.878	280.640	0.081	0.157	0.607	0.783	280.640	0.118	0.183	0.519	0.722
CARD9	42.631	0.448	0.416	0.282	0.420	42.631	0.107	0.419	0.799	0.895	42.631	-0.341	0.482	0.479	0.692
OSGEPL1	142.075	-0.411	0.181	0.023	0.064	142.075	-0.070	0.176	0.689	0.832	142.075	0.341	0.208	0.102	0.284
P3H1	1189.755	0.396	0.120	0.001	0.006	1189.755	0.034	0.120	0.778	0.882	1189.755	-0.362	0.139	0.009	0.061
SET	12775.152	-0.413	0.141	0.003	0.014	12775.152	-0.195	0.141	0.166	0.456	12775.152	0.218	0.162	0.179	0.397
SETMAR	159.960	-0.477	0.193	0.013	0.042	159.960	-0.178	0.188	0.343	0.600	159.960	0.298	0.222	0.180	0.398
SEPTIN7P2	101.153	-0.375	0.326	0.250	0.384	101.153	-0.258	0.322	0.424	0.660	101.153	0.117	0.378	0.756	0.875
BLMH	722.987	-0.311	0.219	0.155	0.271	722.987	-0.339	0.219	0.122	0.413	722.987	-0.027	0.253	0.915	0.960
IRF2BPL	2620.353	-0.295	0.130	0.023	0.064	2620.353	0.128	0.130	0.323	0.583	2620.353	0.424	0.150	0.005	0.040
POPDC3	103.123	0.774	0.243	0.001	0.008	103.123	0.349	0.246	0.156	0.448	103.123	-0.425	0.280	0.130	0.327
SFPQ	4279.258	0.138	0.327	0.674	0.773	4279.258	-0.778	0.327	0.017	0.306	4279.258	-0.916	0.378	0.015	0.086
MMS19	1253.280	-0.338	0.133	0.011	0.037	1253.280	0.162	0.132	0.219	0.498	1253.280	0.500	0.153	0.001	0.014
DNAJC1	1294.540	0.343	0.204	0.093	0.185	1294.540	0.330	0.204	0.106	0.398	1294.540	-0.013	0.236	0.955	0.979
TFB2M	452.610	-1.546	0.344	0.000	0.000	452.610	-0.751	0.342	0.028	0.321	452.610	0.795	0.397	0.046	0.172
PJA1	309.989	-0.445	0.256	0.082	0.168	309.989	-0.121	0.254	0.635	0.801	309.989	0.325	0.296	0.272	0.507
ROBO3	18.831	0.586	0.451	0.194	0.319	18.831	0.247	0.455	0.586	0.768	18.831	-0.338	0.521	0.516	0.720
TOR3A	633.576	-0.686	0.388	0.077	0.160	633.576	-0.736	0.388	0.058	0.347	633.576	-0.050	0.448	0.911	0.959
MLST8	1225.715	-0.023	0.164	0.890	0.929	1225.715	0.015	0.163	0.928	0.964	1225.715	0.037	0.189	0.843	0.924
HERPUD2	449.847	-0.414	0.142	0.004	0.015	449.847	-0.087	0.140	0.532	0.732	449.847	0.326	0.164	0.047	0.175
ATL2	702.247	-0.555	0.164	0.001	0.005	702.247	-0.239	0.163	0.143	0.435	702.247	0.316	0.190	0.096	0.274
MIR4500HG	35.880	-0.180	0.384	0.639	0.746	35.880	0.486	0.370	0.189	0.475	35.880	0.667	0.437	0.127	0.322
PDLIM2	431.020	0.906	0.414	0.029	0.075	431.020	0.761	0.413	0.066	0.354	431.020	-0.144	0.477	0.762	0.879
LOC642361	117.626	-0.215	0.208	0.300	0.439	117.626	-0.070	0.204	0.733	0.857	117.626	0.145	0.240	0.545	0.740
GOLGA6L17P	10.336	0.589	0.734	0.422	0.559	10.336	-0.274	0.758	0.718	0.849	10.336	-0.863	0.864	0.318	0.554
MROH6	81.731	0.490	0.369	0.184	0.308	81.731	-0.449	0.376	0.232	0.510	81.731	-0.939	0.432	0.030	0.130
AGAP9	52.013	1.081	0.470	0.022	0.061	52.013	-0.135	0.480	0.779	0.883	52.013	-1.216	0.548	0.026	0.121
MIR205HG	81.307	1.644	0.544	0.003	0.012	81.307	0.794	0.547	0.147	0.438	81.307	-0.851	0.627	0.175	0.393
SRSF1	3541.186	-1.081	0.414	0.009	0.031	3541.186	-1.235	0.414	0.003	0.297	3541.186	-0.154	0.478	0.747	0.870
SCX	22.298	3.217	0.727	0.000	0.000	22.298	1.367	0.750	0.068	0.357	22.298	-1.851	0.821	0.024	0.115
SRSF2	1712.372	-0.562	0.466	0.228	0.359	1712.372	-1.292	0.466	0.006	0.297	1712.372	-0.729	0.538	0.175	0.393
NPIPA2	75.654	0.243	0.358	0.497	0.628	75.654	0.392	0.355	0.269	0.541	75.654	0.149	0.412	0.717	0.853
SRSF3	5397.439	-0.678	0.286	0.018	0.053	5397.439	-0.719	0.286	0.012	0.305	5397.439	-0.041	0.330	0.900	0.953
ZNF487	15.863	0.098	0.567	0.862	0.910	15.863	0.370	0.555	0.505	0.715	15.863	0.271	0.649	0.676	0.828
TENT4B	383.036	-1.029	0.167	0.000	0.000	383.036	-0.257	0.162	0.112	0.405	383.036	0.772	0.192	0.000	0.002
ARHGEF28	789.928	-0.736	0.296	0.013	0.041	789.928	-0.635	0.295	0.031	0.322	789.928	0.101	0.342	0.768	0.882

RAB17	17.369	0.492	0.432	0.255	0.389	17.369	0.681	0.420	0.105	0.398	17.369	0.189	0.485	0.697	0.841
CPSF4L	34.196	0.456	0.368	0.215	0.345	34.196	0.262	0.368	0.476	0.697	34.196	-0.193	0.424	0.648	0.810
LOC642846	80.505	0.001	0.312	0.998	0.999	80.505	-0.026	0.310	0.933	0.967	80.505	-0.027	0.361	0.941	0.973
RHBDF1	362.245	0.656	0.170	0.000	0.001	362.245	-0.017	0.172	0.923	0.961	362.245	-0.673	0.197	0.001	0.010
LINC00205	174.791	-0.016	0.215	0.940	0.963	174.791	0.011	0.214	0.959	0.979	174.791	0.027	0.249	0.914	0.960
ZSCAN31	174.638	0.025	0.321	0.939	0.962	174.638	0.211	0.319	0.508	0.718	174.638	0.186	0.370	0.614	0.787
SRSF4	2875.979	-0.140	0.168	0.405	0.543	2875.979	-0.136	0.168	0.416	0.654	2875.979	0.003	0.194	0.987	0.993
CERNA2	176.242	0.099	0.902	0.913	0.945	176.242	0.125	0.902	0.890	0.945	176.242	0.027	1.042	0.980	0.990
FLVCR1-DT	37.461	0.822	0.307	0.007	0.027	37.461	-0.586	0.332	0.078	0.369	37.461	-1.408	0.373	0.000	0.003
CXCR5	1258.736	0.665	0.274	0.015	0.047	1258.736	0.577	0.274	0.035	0.324	1258.736	-0.088	0.317	0.782	0.890
SRSF5	1483.808	0.359	0.223	0.107	0.205	1483.808	-0.154	0.223	0.489	0.705	1483.808	-0.513	0.257	0.046	0.173
LOC643072	15.927	-0.218	0.483	0.652	0.757	15.927	0.060	0.465	0.897	0.948	15.927	0.278	0.555	0.617	0.789
SRSF6	984.829	-0.621	0.322	0.054	0.122	984.829	-0.853	0.322	0.008	0.297	984.829	-0.233	0.373	0.532	0.732
ZC3H11B	49.724	0.218	0.295	0.459	0.594	49.724	-0.170	0.299	0.569	0.756	49.724	-0.389	0.345	0.260	0.494
SMIM15	2011.583	-0.595	0.176	0.001	0.005	2011.583	-0.421	0.176	0.017	0.306	2011.583	0.174	0.204	0.393	0.621
NOC3L	732.998	-1.384	0.354	0.000	0.001	732.998	-0.845	0.352	0.017	0.306	732.998	0.539	0.408	0.187	0.407
CCT6P3	35.545	0.510	0.468	0.276	0.413	35.545	-0.123	0.476	0.797	0.894	35.545	-0.632	0.546	0.247	0.478
FBRS	1500.004	-0.008	0.113	0.941	0.963	1500.004	0.099	0.113	0.382	0.628	1500.004	0.107	0.131	0.412	0.638
SRSF7	1741.109	-1.048	0.419	0.012	0.040	1741.109	-1.181	0.419	0.005	0.297	1741.109	-0.133	0.484	0.783	0.891
RNF25	215.658	0.000	0.349	1.000	1.000	215.658	-0.567	0.350	0.106	0.398	215.658	-0.567	0.404	0.161	0.375
NSD1	1402.665	-0.694	0.186	0.000	0.002	1402.665	-0.203	0.185	0.272	0.543	1402.665	0.491	0.214	0.022	0.108
MAP1LC3B2	54.115	-0.078	0.276	0.776	0.852	54.115	-0.425	0.280	0.129	0.420	54.115	-0.347	0.326	0.288	0.524
CCT6P1	55.754	0.064	0.396	0.871	0.916	55.754	0.520	0.387	0.179	0.467	55.754	0.455	0.452	0.314	0.549
COP1	536.694	-0.566	0.222	0.011	0.037	536.694	-0.356	0.221	0.108	0.400	536.694	0.209	0.257	0.415	0.641
LMBR1	811.309	-0.836	0.199	0.000	0.000	811.309	-0.260	0.198	0.190	0.475	811.309	0.577	0.230	0.012	0.075
XPO4	393.251	-1.073	0.334	0.001	0.007	393.251	-0.943	0.333	0.005	0.297	393.251	0.129	0.387	0.738	0.865
SFSWAP	804.102	-0.031	0.207	0.880	0.922	804.102	-0.088	0.207	0.669	0.821	804.102	-0.057	0.239	0.811	0.906
NFKBIZ	520.451	0.202	0.265	0.448	0.583	520.451	-0.327	0.266	0.219	0.499	520.451	-0.529	0.308	0.086	0.255
C15orf62	18.679	1.060	0.574	0.064	0.140	18.679	0.704	0.578	0.223	0.502	18.679	-0.357	0.654	0.586	0.767
TMEM253	19.743	0.398	0.461	0.388	0.527	19.743	0.467	0.456	0.306	0.571	19.743	0.068	0.527	0.897	0.951
TRA2B	2677.723	-0.952	0.310	0.002	0.010	2677.723	-0.859	0.310	0.006	0.297	2677.723	0.093	0.358	0.795	0.897
HS1BP3	749.297	0.875	0.197	0.000	0.000	749.297	0.309	0.197	0.117	0.409	749.297	-0.565	0.227	0.013	0.077
AZI2	1796.500	-0.314	0.172	0.067	0.145	1796.500	0.215	0.171	0.208	0.490	1796.500	0.530	0.198	0.008	0.054
LOC643542	8.072	-1.589	0.783	0.042	0.102	8.072	-1.363	0.751	0.070	0.358	8.072	0.226	0.932	0.808	0.904
NXN	1249.531	-0.179	0.176	0.309	0.448	1249.531	0.219	0.175	0.213	0.493	1249.531	0.398	0.203	0.050	0.183
ZNF862	139.580	0.667	0.438	0.128	0.235	139.580	0.801	0.437	0.067	0.355	139.580	0.134	0.504	0.790	0.895
LINC00842	29.057	-0.299	0.353	0.397	0.535	29.057	0.441	0.334	0.187	0.474	29.057	0.740	0.400	0.064	0.214
GOLGA8N	22.641	0.308	0.780	0.693	0.788	22.641	0.329	0.778	0.672	0.823	22.641	0.021	0.899	0.981	0.990
GOLGA6L4	36.300	0.976	0.651	0.134	0.243	36.300	0.068	0.658	0.918	0.959	36.300	-0.908	0.755	0.229	0.457
LYPLAL1-DT	45.655	0.185	0.265	0.487	0.619	45.655	-0.125	0.268	0.642	0.805	45.655	-0.309	0.311	0.319	0.554
SIL1	828.740	0.251	0.142	0.078	0.163	828.740	0.089	0.142	0.534	0.733	828.740	-0.162	0.164	0.325	0.559
TRAF3IP2-AS1	37.979	-0.587	0.284	0.039	0.095	37.979	0.278	0.254	0.274	0.545	37.979	0.866	0.319	0.007	0.050
IKZF4	73.810	-0.054	0.205	0.794	0.864	73.810	-0.041	0.202	0.838	0.916	73.810	0.012	0.238	0.959	0.981
IKZF5	335.540	-0.967	0.212	0.000	0.000	335.540	-0.163	0.207	0.432	0.666	335.540	0.804	0.244	0.001	0.013
USP46-AS1	12.513	0.154	0.431	0.720	0.810	12.513	-0.109	0.434	0.801	0.896	12.513	-0.264	0.504	0.601	0.779
CNTNAP3P2	58.674	-0.719	0.348	0.039	0.096	58.674	0.014	0.335	0.966	0.982	58.674	0.734	0.400	0.066	0.218
ZFP62	221.547	-0.144	0.145	0.320	0.460	221.547	0.244	0.141	0.083	0.374	221.547	0.388	0.166	0.019	0.099
LINC01128	350.243	-0.127	0.166	0.445	0.581	350.243	-0.233	0.166	0.159	0.451	350.243	-0.106	0.193	0.581	0.764
TMPPE	19.069	-0.409	0.404	0.311	0.451	19.069	-1.381	0.438	0.002	0.297	19.069	-0.972	0.507	0.055	0.196
MMP25	36.537	0.669	0.329	0.042	0.101	36.537	0.372	0.331	0.262	0.534	36.537	-0.298	0.378	0.431	0.653
SPDYE10P	29.060	-0.030	0.357	0.933	0.958	29.060	-0.706	0.371	0.057	0.347	29.060	-0.676	0.429	0.115	0.304
CBLN3	74.247	-0.605	0.317	0.056	0.126	74.247	0.106	0.306	0.729	0.855	74.247	0.711	0.363	0.050	0.183
CRNDE	186.251	-0.028	0.256	0.914	0.946	186.251	0.536	0.252	0.033	0.322	186.251	0.563	0.293	0.055	0.194
ZMAT3	508.940	-0.019	0.269	0.944	0.965	508.940	0.237	0.268	0.376	0.624	508.940	0.256	0.310	0.408	0.635
GMCL1	397.767	-0.701	0.219	0.001	0.007	397.767	-0.025	0.216	0.907	0.953	397.767	0.675	0.252	0.007	0.054
ZNF106	1347.800	-0.679	0.198	0.001	0.004	1347.800	-0.120	0.198	0.545	0.739	1347.800	0.560	0.229	0.015	0.083
MPP5	820.823	-0.923	0.242	0.000	0.001	820.823	-0.258	0.241	0.285	0.554	820.823	0.665	0.280	0.017	0.094
FNDC10	8.990	2.026	0.663	0.002	0.011	8.990	0.166	0.753	0.826	0.909	8.990	-1.860	0.793	0.019	0.099
HHIP	33.281	-3.345	0.743	0.000	0.000	33.281	-1.108	0.671	0.099	0.393	33.281	2.237	0.843	0.008	0.056
BLVRA	741.804	0.549	0.167	0.001	0.006	741.804	0.063	0.168	0.709	0.844	741.804	-0.486	0.193	0.012	0.073
AKTIP	505.134	0.469	0.193	0.015	0.047	505.134	0.260	0.194	0.180	0.468	505.134	-0.209	0.223	0.350	0.583
CBWD6	465.523	-0.144	0.425	0.735	0.821	465.523	-1.298	0.428	0.002	0.297	465.523	-1.155	0.493	0.019	0.099
CDH24	367.349	0.135	0.129	0.297	0.435	367.349	-0.014	0.129	0.915	0.958	367.349	-0.148	0.149	0.321	0.555
LINC01529	8.579	-0.117	0.736	0.874	0.919	8.579	0.598	0.706	0.397	0.641	8.579	0.715	0.835	0.392	0.620
RPS14P3	33.294	0.654	0.342	0.056	0.126	33.294	0.333	0.345	0.335	0.594	33.294	-0.321	0.394	0.416	0.641
SDHAF1	323.528	0.318	0.159	0.045	0.107	323.528	0.127	0.159	0.423	0.660	323.528	-0.191	0.184	0.299	0.534
KLHL25	142.170	1.392	0.173	0.000	0.000	142.170	0.512	0.178	0.004	0.297	142.170	-0.880	0.199	0.000	0.000

ARAP3	534.822	0.173	0.162	0.283	0.421	534.822	-0.129	0.162	0.426	0.662	534.822	-0.302	0.187	0.106	0.291
GZF1	362.547	-0.950	0.304	0.002	0.009	362.547	-0.685	0.302	0.024	0.316	362.547	0.265	0.352	0.452	0.671
RPL23AP53	33.275	-1.063	0.425	0.012	0.040	33.275	-1.031	0.419	0.014	0.306	33.275	0.032	0.500	0.949	0.976
LOC644135	20.132	2.390	0.684	0.000	0.003	20.132	-2.481	1.334	0.063	0.350	20.132	-4.871	1.335	0.000	0.005
TMEM267	317.034	-0.655	0.207	0.002	0.008	317.034	-0.461	0.205	0.025	0.316	317.034	0.195	0.240	0.416	0.642
MAPK8IP1P2	28.791	-0.478	0.731	0.513	0.642	28.791	0.331	0.719	0.645	0.806	28.791	0.809	0.840	0.335	0.570
TMEM168	315.032	-0.574	0.200	0.004	0.017	315.032	-0.325	0.198	0.101	0.394	315.032	0.248	0.232	0.284	0.519
MTMR14	594.901	0.004	0.152	0.978	0.987	594.901	-0.151	0.152	0.323	0.584	594.901	-0.155	0.176	0.381	0.610
SUSD1	18.810	0.239	0.438	0.585	0.702	18.810	-0.131	0.445	0.769	0.877	18.810	-0.370	0.514	0.472	0.686
DCLRE1C	235.415	-0.597	0.210	0.004	0.018	235.415	-0.392	0.208	0.060	0.350	235.415	0.206	0.244	0.398	0.626
ATG3	1291.466	-0.376	0.221	0.089	0.179	1291.466	-0.438	0.221	0.047	0.336	1291.466	-0.062	0.256	0.809	0.904
INF2	3424.915	0.508	0.127	0.000	0.001	3424.915	0.335	0.127	0.008	0.297	3424.915	-0.173	0.147	0.239	0.469
LINC00622	28.723	3.134	0.732	0.000	0.000	28.723	1.426	0.752	0.058	0.348	28.723	-1.708	0.827	0.039	0.156
KANSL1-AS1	26.107	1.181	0.544	0.030	0.078	26.107	0.275	0.555	0.620	0.791	26.107	-0.906	0.631	0.151	0.360
POLR1E	922.755	-0.552	0.104	0.000	0.000	922.755	-0.086	0.102	0.401	0.644	922.755	0.466	0.120	0.000	0.002
SUDS3	723.946	-0.027	0.127	0.830	0.888	723.946	-0.031	0.127	0.810	0.900	723.946	-0.003	0.147	0.982	0.990
TTC31	186.672	-0.511	0.236	0.031	0.080	186.672	-0.280	0.234	0.232	0.509	186.672	0.232	0.273	0.397	0.625
CIAO3	403.642	0.172	0.170	0.311	0.450	403.642	-0.128	0.170	0.452	0.680	403.642	-0.300	0.197	0.127	0.322
ZDHHC6	494.797	-0.198	0.223	0.373	0.513	494.797	-0.471	0.223	0.034	0.322	494.797	-0.273	0.258	0.290	0.525
SGCB	699.463	0.158	0.204	0.440	0.576	699.463	0.111	0.204	0.588	0.770	699.463	-0.047	0.236	0.841	0.923
PCNX4	1010.481	-0.466	0.136	0.001	0.004	1010.481	-0.243	0.135	0.071	0.360	1010.481	0.222	0.157	0.156	0.367
ACTR6	498.862	-0.448	0.152	0.003	0.014	498.862	-0.274	0.151	0.069	0.358	498.862	0.174	0.176	0.323	0.558
MRPS25	851.784	0.369	0.204	0.070	0.149	851.784	-0.254	0.204	0.214	0.493	851.784	-0.623	0.236	0.008	0.057
NOM1	383.319	-0.399	0.274	0.145	0.258	383.319	-0.671	0.274	0.014	0.306	383.319	-0.272	0.317	0.392	0.620
SGK1	80.377	0.801	0.589	0.174	0.295	80.377	-1.044	0.601	0.082	0.374	80.377	-1.844	0.689	0.007	0.054
INTS4P2	13.480	-0.578	0.444	0.193	0.318	13.480	0.259	0.402	0.518	0.723	13.480	0.838	0.499	0.093	0.268
LOC644634	195.008	0.965	0.283	0.001	0.004	195.008	0.705	0.283	0.013	0.305	195.008	-0.260	0.325	0.424	0.647
LOC644656	11.749	0.726	0.457	0.112	0.212	11.749	0.299	0.467	0.523	0.726	11.749	-0.428	0.527	0.417	0.642
RAD21-AS1	131.799	-1.401	0.428	0.001	0.006	131.799	-0.054	0.419	0.898	0.948	131.799	1.347	0.492	0.006	0.048
SCG5	69.815	1.292	0.335	0.000	0.001	69.815	0.605	0.340	0.075	0.364	69.815	-0.687	0.385	0.075	0.235
LIMD1-AS1	621.218	-0.510	0.186	0.006	0.023	621.218	0.058	0.184	0.753	0.868	621.218	0.568	0.214	0.008	0.057
CSMD1	156.961	-0.782	0.243	0.001	0.007	156.961	-0.217	0.237	0.360	0.612	156.961	0.566	0.280	0.043	0.167
LINC02604	10.132	1.226	0.583	0.035	0.089	10.132	0.886	0.588	0.132	0.424	10.132	-0.340	0.656	0.605	0.782
SGSH	624.725	0.549	0.155	0.000	0.003	624.725	0.072	0.156	0.645	0.806	624.725	-0.477	0.179	0.008	0.056
FAM83G	570.716	-0.245	0.157	0.117	0.220	570.716	0.034	0.155	0.824	0.908	570.716	0.280	0.181	0.121	0.313
LINC01184	338.349	-0.088	0.165	0.595	0.710	338.349	0.077	0.164	0.636	0.801	338.349	0.165	0.191	0.386	0.615
SGTA	2184.518	-0.111	0.256	0.665	0.767	2184.518	-0.506	0.256	0.048	0.337	2184.518	-0.395	0.296	0.182	0.400
GCC2-AS1	39.898	-0.149	0.293	0.612	0.723	39.898	0.088	0.284	0.758	0.871	39.898	0.236	0.336	0.483	0.695
LINC02315	7.834	0.033	0.566	0.954	0.972	7.834	-0.243	0.571	0.670	0.822	7.834	-0.276	0.666	0.679	0.830
LINC00235	25.529	-0.428	0.389	0.271	0.408	25.529	-0.546	0.387	0.159	0.451	25.529	-0.117	0.458	0.798	0.899
RASSF10	9.286	0.533	0.615	0.386	0.525	9.286	0.244	0.619	0.694	0.835	9.286	-0.289	0.709	0.684	0.833
TPSB2	164.040	0.996	1.002	0.320	0.460	164.040	1.583	0.984	0.108	0.400	164.040	0.587	1.127	0.602	0.780
BLVRB	2416.754	0.775	0.295	0.009	0.030	2416.754	0.601	0.295	0.042	0.330	2416.754	-0.174	0.341	0.609	0.784
SH3BGR	12.675	0.595	0.539	0.269	0.406	12.675	0.466	0.537	0.386	0.632	12.675	-0.129	0.616	0.834	0.919
EVPLL	15.592	0.410	0.982	0.676	0.775	15.592	1.380	0.968	0.154	0.446	15.592	0.969	1.121	0.387	0.616
SH3BGRL	1156.193	0.062	0.345	0.857	0.907	1156.193	0.424	0.345	0.219	0.498	1156.193	0.362	0.399	0.364	0.596
CCNI2	27.971	1.392	0.616	0.024	0.066	27.971	0.571	0.624	0.360	0.612	27.971	-0.822	0.710	0.247	0.478
LSP1P5	68.314	1.116	0.309	0.000	0.002	68.314	0.181	0.316	0.568	0.755	68.314	-0.935	0.359	0.009	0.062
SH3BP2	618.142	-0.307	0.199	0.123	0.228	618.142	0.421	0.197	0.032	0.322	618.142	0.728	0.229	0.001	0.017
EIF3J-DT	172.525	-0.376	0.268	0.162	0.280	172.525	0.245	0.264	0.353	0.607	172.525	0.620	0.308	0.044	0.169
ITSN1	2087.672	-0.931	0.188	0.000	0.000	2087.672	-0.071	0.187	0.705	0.842	2087.672	0.860	0.217	0.000	0.002
FAM86C2P	57.206	-0.673	0.232	0.004	0.016	57.206	0.073	0.211	0.731	0.856	57.206	0.745	0.263	0.005	0.039
FUT8-AS1	10.985	-0.197	0.587	0.737	0.823	10.985	0.523	0.540	0.333	0.592	10.985	0.720	0.656	0.272	0.507
CEP170P1	77.307	-1.488	0.350	0.000	0.000	77.307	-0.245	0.332	0.459	0.685	77.307	1.243	0.401	0.002	0.021
SH3GL1	5147.633	0.107	0.115	0.353	0.493	5147.633	0.097	0.115	0.397	0.641	5147.633	-0.009	0.133	0.943	0.974
LOC645513	54.441	-0.285	0.272	0.296	0.434	54.441	-0.209	0.267	0.434	0.668	54.441	0.076	0.316	0.811	0.906
IGFL2-AS1	131.164	3.635	0.359	0.000	0.000	131.164	0.422	0.448	0.346	0.602	131.164	-3.213	0.431	0.000	0.000
WFDC21P	886.010	0.322	0.164	0.049	0.114	886.010	0.297	0.164	0.069	0.357	886.010	-0.025	0.189	0.896	0.951
ASH1L-AS1	29.579	-0.398	0.387	0.304	0.443	29.579	-0.375	0.383	0.327	0.587	29.579	0.023	0.452	0.959	0.981
SH3GL3	106.336	0.276	0.296	0.350	0.490	106.336	0.532	0.291	0.067	0.355	106.336	0.256	0.338	0.449	0.668
GPR135	10.604	0.295	0.635	0.643	0.749	10.604	0.152	0.634	0.811	0.900	10.604	-0.143	0.734	0.846	0.925
SVIL2P	12.897	-1.641	0.739	0.026	0.071	12.897	0.328	0.653	0.616	0.788	12.897	1.969	0.828	0.017	0.094
RPS18P9	18.287	1.189	0.513	0.021	0.059	18.287	0.164	0.533	0.759	0.872	18.287	-1.026	0.599	0.087	0.257
LOC645967	33.883	-1.457	0.616	0.018	0.053	33.883	-0.374	0.594	0.529	0.730	33.883	1.083	0.708	0.126	0.320
MOSPD3	181.898	0.980	0.472	0.038	0.093	181.898	0.834	0.472	0.077	0.368	181.898	-0.147	0.544	0.787	0.894
GIGYF1	618.290	0.316	0.202	0.118	0.221	618.290	0.287	0.202	0.155	0.447	618.290	-0.029	0.233	0.902	0.954
BNC1	1105.761	-1.514	0.231	0.000	0.000	1105.761	-0.306	0.229	0.182	0.469	1105.761	1.208	0.266	0.000	0.000

VPS16	382.885	0.487	0.136	0.000	0.002	382.885	-0.045	0.137	0.745	0.864	382.885	-0.532	0.158	0.001	0.011
SHB	754.793	-0.130	0.247	0.598	0.712	754.793	-0.190	0.247	0.442	0.673	754.793	-0.060	0.286	0.835	0.920
PDCD6IPP2	18.132	0.518	0.539	0.337	0.477	18.132	-0.995	0.581	0.087	0.380	18.132	-1.513	0.657	0.021	0.106
SHC1	11563.784	0.215	0.201	0.287	0.425	11563.784	0.379	0.201	0.060	0.350	11563.784	0.164	0.232	0.480	0.693
MFSD14A	782.496	-0.041	0.232	0.858	0.908	782.496	0.194	0.232	0.402	0.645	782.496	0.236	0.268	0.379	0.610
ARIH2OS	93.685	-1.057	0.341	0.002	0.010	93.685	-0.635	0.334	0.057	0.347	93.685	0.422	0.395	0.286	0.521
LOC646471	44.507	-0.628	0.341	0.066	0.142	44.507	0.372	0.318	0.243	0.519	44.507	1.000	0.386	0.010	0.064
CSRNP1	694.867	-0.335	0.436	0.443	0.578	694.867	-1.108	0.437	0.011	0.304	694.867	-0.774	0.505	0.126	0.320
HHIP-AS1	12.634	-3.212	0.950	0.001	0.004	12.634	-1.266	0.820	0.123	0.414	12.634	1.946	1.075	0.070	0.226
IGF2BP2-AS1	9.508	-2.653	0.835	0.001	0.008	9.508	-0.843	0.666	0.206	0.488	9.508	1.810	0.935	0.053	0.189
LOC646626	35.741	-0.702	0.366	0.055	0.124	35.741	-0.180	0.348	0.605	0.781	35.741	0.522	0.421	0.215	0.441
LYPD8	36.742	0.113	0.512	0.825	0.885	36.742	0.497	0.505	0.325	0.586	36.742	0.384	0.587	0.513	0.718
NIPBL-DT	226.790	-0.572	0.172	0.001	0.005	226.790	-0.112	0.167	0.501	0.713	226.790	0.460	0.198	0.020	0.102
LOC646762	5.894	-2.418	0.988	0.014	0.045	5.894	-0.488	0.793	0.539	0.736	5.894	1.930	1.101	0.079	0.244
FBXW4	722.343	0.627	0.257	0.015	0.046	722.343	0.330	0.257	0.200	0.483	722.343	-0.297	0.297	0.317	0.553
SETSIP	13.538	-0.561	0.508	0.269	0.406	13.538	-0.139	0.484	0.774	0.880	13.538	0.422	0.585	0.470	0.684
ANAPC1	1625.520	-1.084	0.252	0.000	0.000	1625.520	-0.415	0.252	0.099	0.394	1625.520	0.669	0.291	0.022	0.106
FAM227A	11.955	1.262	0.488	0.010	0.033	11.955	0.163	0.519	0.753	0.868	11.955	-1.099	0.573	0.055	0.195
GORASP1	616.830	0.034	0.214	0.876	0.919	616.830	-0.415	0.215	0.053	0.342	616.830	-0.449	0.248	0.070	0.226
HRCT1	125.713	0.241	0.491	0.623	0.733	125.713	0.520	0.489	0.287	0.556	125.713	0.279	0.565	0.622	0.793
TMPRSS3	17.019	1.497	0.524	0.004	0.018	17.019	1.391	0.523	0.008	0.297	17.019	-0.106	0.583	0.855	0.930
SHMT1	366.576	0.120	0.125	0.338	0.478	366.576	-0.016	0.125	0.898	0.949	366.576	-0.136	0.145	0.349	0.582
C6orf132	24.828	1.210	0.319	0.000	0.001	24.828	-0.025	0.348	0.942	0.971	24.828	-1.236	0.381	0.001	0.015
PA2G4P4	81.410	-0.040	0.373	0.915	0.946	81.410	-0.514	0.376	0.172	0.461	81.410	-0.474	0.435	0.276	0.511
GOLGA6L10	61.309	0.770	0.563	0.171	0.292	61.309	0.496	0.563	0.379	0.626	61.309	-0.274	0.648	0.673	0.826
LOC647070	28.301	-1.072	0.539	0.047	0.110	28.301	-0.777	0.529	0.142	0.434	28.301	0.295	0.627	0.638	0.803
COPS7B	384.715	-0.330	0.263	0.209	0.338	384.715	-0.518	0.263	0.049	0.337	384.715	-0.188	0.304	0.536	0.734
STMP1	572.201	0.201	0.207	0.331	0.471	572.201	0.138	0.207	0.506	0.716	572.201	-0.064	0.239	0.790	0.895
NUCKS1	8657.073	-0.194	0.201	0.335	0.475	8657.073	0.097	0.201	0.631	0.798	8657.073	0.291	0.232	0.211	0.436
SRGAP2B	996.511	-0.173	0.138	0.211	0.340	996.511	0.010	0.137	0.943	0.971	996.511	0.183	0.159	0.252	0.484
SERPINE3	46.188	-0.826	0.297	0.005	0.021	46.188	-0.584	0.288	0.042	0.330	46.188	0.242	0.346	0.485	0.696
UNKL	644.122	-0.029	0.149	0.846	0.899	644.122	0.133	0.148	0.367	0.618	644.122	0.162	0.172	0.345	0.579
SHMT2	8354.028	-0.601	0.128	0.000	0.000	8354.028	0.091	0.128	0.475	0.696	8354.028	0.692	0.148	0.000	0.000
LINC02026	15.050	-0.357	0.466	0.444	0.580	15.050	0.601	0.426	0.159	0.451	15.050	0.957	0.520	0.066	0.216
SHOX2	202.487	0.500	0.380	0.189	0.313	202.487	0.434	0.380	0.253	0.527	202.487	-0.066	0.439	0.881	0.943
WDR13	3075.865	0.810	0.229	0.000	0.003	3075.865	0.475	0.229	0.038	0.327	3075.865	-0.335	0.265	0.205	0.429
SMAP2	2104.894	-0.497	0.097	0.000	0.000	2104.894	0.120	0.096	0.213	0.493	2104.894	0.617	0.112	0.000	0.000
METTL17	398.197	-0.224	0.221	0.310	0.450	398.197	-0.198	0.220	0.369	0.619	398.197	0.026	0.255	0.918	0.962
ACBD3	977.098	-0.368	0.228	0.106	0.204	977.098	-0.121	0.227	0.594	0.774	977.098	0.247	0.263	0.348	0.582
MFSD1	761.030	-0.003	0.167	0.987	0.993	761.030	-0.415	0.168	0.013	0.305	761.030	-0.412	0.194	0.034	0.141
PLPPR2	833.339	0.500	0.185	0.007	0.025	833.339	0.483	0.185	0.009	0.297	833.339	-0.017	0.213	0.936	0.971
SMURF2	595.354	-0.622	0.127	0.000	0.000	595.354	-0.143	0.124	0.251	0.525	595.354	0.479	0.146	0.001	0.014
SMYD3	262.346	0.222	0.196	0.257	0.391	262.346	0.189	0.196	0.333	0.592	262.346	-0.033	0.226	0.884	0.945
C16orf58	1248.950	-0.011	0.139	0.935	0.959	1248.950	0.272	0.139	0.050	0.337	1248.950	0.283	0.160	0.077	0.240
ATPAF1	974.959	-0.312	0.139	0.025	0.068	974.959	0.031	0.138	0.822	0.908	974.959	0.343	0.161	0.033	0.139
MARC1	26.426	0.768	0.400	0.055	0.124	26.426	0.183	0.409	0.654	0.811	26.426	-0.585	0.465	0.208	0.433
TNS3	2106.157	-0.152	0.242	0.530	0.656	2106.157	0.148	0.241	0.539	0.736	2106.157	0.300	0.279	0.282	0.516
FAM160B2	582.602	0.750	0.226	0.001	0.005	582.602	0.369	0.226	0.103	0.395	582.602	-0.381	0.261	0.143	0.349
PARP12	545.268	0.341	0.199	0.087	0.176	545.268	-0.276	0.200	0.168	0.457	545.268	-0.618	0.231	0.008	0.054
GAREM1	141.550	-0.998	0.283	0.000	0.003	141.550	-0.309	0.276	0.264	0.536	141.550	0.690	0.326	0.034	0.144
ZNF574	442.166	0.096	0.119	0.422	0.558	442.166	0.119	0.118	0.313	0.576	442.166	0.023	0.137	0.865	0.935
CREB3L2	1241.669	-0.563	0.162	0.001	0.003	1241.669	0.023	0.161	0.888	0.944	1241.669	0.586	0.187	0.002	0.019
S100PBP	433.547	-0.507	0.161	0.002	0.009	433.547	-0.090	0.159	0.573	0.758	433.547	0.417	0.186	0.025	0.116
IPPK	342.287	-0.262	0.249	0.292	0.430	342.287	-0.676	0.250	0.007	0.297	342.287	-0.414	0.289	0.152	0.361
MEAF6	785.297	-0.052	0.125	0.678	0.776	785.297	-0.083	0.125	0.504	0.715	785.297	-0.031	0.144	0.828	0.915
SIAH1	458.199	-0.173	0.151	0.252	0.387	458.199	-0.192	0.151	0.202	0.486	458.199	-0.019	0.175	0.914	0.960
CCDC14	491.312	-0.038	0.192	0.845	0.898	491.312	0.120	0.191	0.532	0.732	491.312	0.157	0.222	0.479	0.692
ILRUN	2672.450	-0.141	0.125	0.256	0.391	2672.450	0.042	0.124	0.737	0.860	2672.450	0.183	0.144	0.203	0.426
ENGASE	377.978	0.531	0.199	0.008	0.027	377.978	0.136	0.200	0.495	0.709	377.978	-0.395	0.230	0.086	0.255
PCED1A	283.372	0.362	0.239	0.130	0.238	283.372	0.580	0.238	0.015	0.306	283.372	0.219	0.275	0.426	0.649
C11orf1	77.510	0.743	0.307	0.016	0.048	77.510	0.567	0.307	0.065	0.354	77.510	-0.176	0.352	0.617	0.790
RMND5B	1840.462	0.130	0.233	0.577	0.695	1840.462	-0.238	0.234	0.309	0.573	1840.462	-0.368	0.270	0.173	0.389
FNDC3B	2125.921	-0.482	0.296	0.103	0.200	2125.921	-0.474	0.296	0.109	0.401	2125.921	0.008	0.342	0.981	0.990
MTHFSD	243.975	-0.205	0.154	0.183	0.306	243.975	0.208	0.150	0.167	0.456	243.975	0.413	0.177	0.019	0.100
SIAH2	1535.865	-0.671	0.243	0.006	0.022	1535.865	-0.063	0.242	0.795	0.893	1535.865	0.608	0.280	0.030	0.132
MICAL1	521.960	0.732	0.158	0.000	0.000	521.960	-0.028	0.160	0.859	0.928	521.960	-0.760	0.184	0.000	0.001
CERK	1053.914	-0.381	0.231	0.100	0.195	1053.914	0.116	0.230	0.616	0.788	1053.914	0.497	0.267	0.063	0.211

AEN	368.153	-1.724	0.731	0.018	0.054	368.153	-1.855	0.730	0.011	0.302	368.153	-0.131	0.845	0.877	0.941
RBM15	241.316	-0.196	0.231	0.396	0.534	241.316	-0.273	0.231	0.237	0.514	241.316	-0.076	0.268	0.776	0.886
CRTC3	583.070	-0.401	0.133	0.002	0.011	583.070	-0.090	0.131	0.492	0.708	583.070	0.311	0.153	0.042	0.163
GINS3	159.161	-0.684	0.326	0.036	0.090	159.161	-0.665	0.325	0.040	0.328	159.161	0.019	0.378	0.961	0.982
OCLNP1	15.750	0.928	0.552	0.093	0.186	15.750	0.888	0.549	0.106	0.398	15.750	-0.040	0.626	0.949	0.976
TBC1D15	973.825	-0.629	0.207	0.002	0.011	973.825	-0.177	0.206	0.390	0.635	973.825	0.452	0.238	0.058	0.201
EPS8L2	110.236	0.437	0.210	0.037	0.092	110.236	0.305	0.209	0.144	0.436	110.236	-0.132	0.241	0.584	0.766
LMF1	39.569	0.624	0.487	0.199	0.326	39.569	0.395	0.487	0.417	0.655	39.569	-0.229	0.560	0.682	0.832
EXO5	21.427	-0.359	0.396	0.364	0.505	21.427	-0.539	0.396	0.173	0.463	21.427	-0.179	0.467	0.701	0.844
IFT22	251.783	0.200	0.173	0.246	0.380	251.783	0.186	0.172	0.280	0.549	251.783	-0.014	0.199	0.942	0.974
CEP85	437.022	-0.314	0.184	0.088	0.177	437.022	-0.045	0.183	0.807	0.899	437.022	0.269	0.212	0.204	0.428
DDX31	356.391	-0.739	0.236	0.002	0.009	356.391	-0.417	0.234	0.075	0.364	356.391	0.322	0.273	0.239	0.469
MIR924HG	43.655	-0.732	0.314	0.020	0.057	43.655	-0.054	0.297	0.856	0.926	43.655	0.678	0.359	0.059	0.203
RMND5A	3394.076	-0.651	0.108	0.000	0.000	3394.076	-0.184	0.108	0.087	0.380	3394.076	0.467	0.125	0.000	0.004
NORAD	3979.541	-0.174	0.247	0.481	0.615	3979.541	0.135	0.247	0.584	0.767	3979.541	0.309	0.285	0.279	0.513
DEPTOR	88.942	0.167	0.400	0.676	0.775	88.942	0.466	0.396	0.239	0.516	88.942	0.299	0.459	0.516	0.720
IQCH	30.782	0.243	0.287	0.396	0.535	30.782	0.599	0.275	0.029	0.322	30.782	0.356	0.323	0.270	0.505
BMI1	776.415	-0.575	0.139	0.000	0.000	776.415	-0.106	0.137	0.439	0.672	776.415	0.470	0.160	0.003	0.031
ST6GAL1	710.459	-1.760	0.363	0.000	0.000	710.459	-0.600	0.361	0.096	0.391	710.459	1.160	0.419	0.006	0.044
ARV1	343.521	0.578	0.212	0.006	0.024	343.521	-0.162	0.214	0.450	0.678	343.521	-0.740	0.246	0.003	0.026
NMNAT1	237.417	0.079	0.130	0.543	0.668	237.417	-0.043	0.130	0.738	0.860	237.417	-0.123	0.151	0.418	0.643
ST3GAL1	977.585	-0.906	0.355	0.011	0.036	977.585	-0.812	0.355	0.022	0.315	977.585	0.094	0.410	0.819	0.910
ST3GAL2	816.247	0.452	0.110	0.000	0.000	816.247	0.098	0.110	0.373	0.622	816.247	-0.354	0.127	0.005	0.043
ELOVL1	1957.300	0.221	0.196	0.260	0.395	1957.300	-0.232	0.196	0.238	0.515	1957.300	-0.452	0.227	0.046	0.173
KLC2	1071.608	-0.057	0.321	0.859	0.908	1071.608	-0.769	0.321	0.017	0.306	1071.608	-0.712	0.371	0.055	0.194
FBXL17	467.998	-0.419	0.379	0.269	0.406	467.998	0.343	0.378	0.365	0.616	467.998	0.762	0.437	0.082	0.247
ST3GAL4	389.063	-0.112	0.215	0.601	0.715	389.063	-0.178	0.215	0.407	0.648	389.063	-0.066	0.249	0.792	0.896
PORCN	313.787	0.552	0.181	0.002	0.011	313.787	0.340	0.181	0.060	0.350	313.787	-0.212	0.208	0.308	0.543
GNPNAT1	1392.439	-1.270	0.257	0.000	0.000	1392.439	-0.601	0.256	0.019	0.306	1392.439	0.669	0.297	0.024	0.115
ISL2	74.668	-0.824	0.324	0.011	0.037	74.668	-0.305	0.315	0.334	0.593	74.668	0.519	0.374	0.165	0.380
MARCHF7	842.306	-0.526	0.167	0.002	0.008	842.306	-0.293	0.166	0.077	0.368	842.306	0.233	0.193	0.227	0.455
SPATA20	635.349	0.866	0.325	0.008	0.028	635.349	0.609	0.325	0.061	0.350	635.349	-0.258	0.375	0.492	0.702
YTHDC2	554.598	-0.799	0.213	0.000	0.001	554.598	-0.330	0.212	0.119	0.412	554.598	0.470	0.246	0.056	0.198
TUT1	533.350	-0.395	0.221	0.074	0.156	533.350	-0.401	0.221	0.069	0.357	533.350	-0.006	0.256	0.980	0.990
AIDA	1299.651	-0.108	0.106	0.305	0.444	1299.651	-0.156	0.105	0.139	0.430	1299.651	-0.048	0.122	0.697	0.841
USP46	272.667	-0.533	0.204	0.009	0.032	272.667	-0.051	0.201	0.800	0.896	272.667	0.482	0.235	0.041	0.160
NIBAN2	6177.561	-0.098	0.157	0.533	0.658	6177.561	-0.015	0.157	0.926	0.963	6177.561	0.084	0.182	0.645	0.808
VWA1	72.877	0.557	0.988	0.573	0.692	72.877	0.829	0.986	0.401	0.644	72.877	0.272	1.139	0.811	0.906
PLEKHG2	402.867	-0.345	0.141	0.014	0.044	402.867	0.018	0.138	0.897	0.948	402.867	0.363	0.162	0.025	0.117
DCLRE1B	260.335	-0.755	0.354	0.033	0.084	260.335	-0.863	0.354	0.015	0.306	260.335	-0.108	0.410	0.792	0.896
NABP1	300.020	0.144	0.232	0.535	0.660	300.020	-0.250	0.233	0.282	0.552	300.020	-0.394	0.269	0.142	0.348
ARMCX5	98.739	-0.600	0.236	0.011	0.037	98.739	-0.234	0.230	0.310	0.574	98.739	0.366	0.273	0.180	0.398
METTL4	155.214	-1.164	0.277	0.000	0.000	155.214	-0.430	0.270	0.112	0.405	155.214	0.735	0.319	0.021	0.106
RFX7	1076.754	-0.801	0.298	0.007	0.026	1076.754	0.038	0.297	0.897	0.948	1076.754	0.840	0.344	0.015	0.083
CDCP1	3041.588	-0.678	0.246	0.006	0.022	3041.588	-0.239	0.245	0.329	0.589	3041.588	0.439	0.284	0.122	0.313
ST3GAL3	51.492	-0.092	0.300	0.759	0.840	51.492	0.540	0.288	0.061	0.350	51.492	0.632	0.341	0.064	0.212
PPP1R3G	11.692	0.022	1.052	0.983	0.990	11.692	0.481	1.040	0.644	0.806	11.692	0.459	1.208	0.704	0.846
PCDH20	49.880	0.123	0.368	0.739	0.823	49.880	-0.280	0.371	0.449	0.678	49.880	-0.403	0.429	0.347	0.581
PAPOLG	200.188	0.181	0.159	0.255	0.390	200.188	-0.039	0.160	0.805	0.898	200.188	-0.221	0.185	0.233	0.462
C12orf43	230.055	-0.066	0.288	0.820	0.882	230.055	-0.474	0.289	0.101	0.394	230.055	-0.408	0.335	0.223	0.450
LOC648987	24.206	-0.053	0.371	0.885	0.926	24.206	-0.113	0.367	0.757	0.871	24.206	-0.060	0.432	0.890	0.947
BMP1	1979.702	0.769	0.280	0.006	0.023	1979.702	0.485	0.280	0.083	0.375	1979.702	-0.284	0.323	0.379	0.610
PMEL	213.880	-0.310	0.214	0.148	0.262	213.880	-0.218	0.213	0.306	0.571	213.880	0.092	0.248	0.712	0.850
LPIN3	198.524	0.715	0.419	0.088	0.178	198.524	0.711	0.418	0.089	0.382	198.524	-0.004	0.482	0.994	0.996
RANBP17	40.130	-0.091	0.311	0.770	0.847	40.130	0.130	0.303	0.667	0.819	40.130	0.222	0.358	0.536	0.734
STIL	395.457	-0.959	0.300	0.001	0.008	395.457	-0.513	0.299	0.086	0.379	395.457	0.446	0.347	0.198	0.421
BCL11B	66.039	1.574	0.258	0.000	0.000	66.039	0.102	0.275	0.711	0.845	66.039	-1.473	0.303	0.000	0.000
CASD1	115.370	-0.110	0.273	0.687	0.784	115.370	0.116	0.270	0.667	0.819	115.370	0.226	0.315	0.472	0.686
SLC30A5	1540.846	-0.445	0.184	0.016	0.048	1540.846	-0.390	0.184	0.034	0.322	1540.846	0.055	0.213	0.796	0.898
CCDC71	357.237	-0.120	0.211	0.568	0.688	357.237	-0.265	0.211	0.209	0.490	357.237	-0.145	0.244	0.554	0.747
TTC23	320.970	-0.069	0.156	0.658	0.761	320.970	0.192	0.154	0.213	0.493	320.970	0.261	0.180	0.146	0.353
MRPL14	1303.453	0.796	0.251	0.001	0.008	1303.453	-0.288	0.252	0.253	0.527	1303.453	-1.084	0.290	0.000	0.004
SIM2	367.292	-1.114	0.154	0.000	0.000	367.292	-0.017	0.147	0.907	0.954	367.292	1.096	0.176	0.000	0.000
YWHAEP1	16.607	-0.530	0.395	0.179	0.302	16.607	-0.530	0.386	0.170	0.460	16.607	0.000	0.467	1.000	1.000
SIPA1	682.242	0.748	0.229	0.001	0.006	682.242	-0.331	0.231	0.152	0.444	682.242	-1.079	0.266	0.000	0.001
STAG3L4	128.393	-0.126	0.216	0.558	0.680	128.393	-0.053	0.214	0.805	0.898	128.393	0.074	0.250	0.768	0.882
NT5DC2	1587.141	-0.489	0.230	0.034	0.086	1587.141	-0.371	0.230	0.106	0.398	1587.141	0.118	0.266	0.657	0.817

DLGAP1-AS1	14.078	1.007	0.441	0.022	0.062	14.078	-0.039	0.473	0.935	0.967	14.078	-1.046	0.524	0.046	0.173
CENPH	742.970	0.120	0.197	0.543	0.667	742.970	0.018	0.197	0.929	0.964	742.970	-0.102	0.228	0.653	0.813
MRPS26	737.711	0.450	0.300	0.133	0.242	737.711	-0.296	0.301	0.324	0.585	737.711	-0.747	0.347	0.031	0.136
SIX1	240.339	-0.236	0.227	0.300	0.438	240.339	-0.424	0.227	0.062	0.350	240.339	-0.189	0.264	0.475	0.688
MRPS24	726.592	0.096	0.152	0.529	0.655	726.592	0.103	0.152	0.498	0.712	726.592	0.007	0.176	0.968	0.985
MRPS15	1055.102	0.564	0.242	0.020	0.057	1055.102	-0.120	0.243	0.620	0.791	1055.102	-0.685	0.280	0.014	0.083
MRPS11	405.521	-0.023	0.230	0.921	0.950	405.521	-0.348	0.231	0.131	0.423	405.521	-0.325	0.267	0.223	0.451
MRPS9	843.459	-0.304	0.132	0.021	0.060	843.459	-0.200	0.131	0.128	0.420	843.459	0.104	0.153	0.495	0.703
MRPS6	760.022	-0.019	0.189	0.921	0.950	760.022	0.114	0.188	0.543	0.738	760.022	0.133	0.218	0.541	0.737
MRPS5	1444.530	0.235	0.168	0.161	0.280	1444.530	0.063	0.168	0.707	0.842	1444.530	-0.172	0.194	0.375	0.607
SKI	1804.504	-0.138	0.092	0.135	0.245	1804.504	0.168	0.091	0.067	0.355	1804.504	0.305	0.106	0.004	0.036
MRPL41	1126.009	0.735	0.375	0.050	0.116	1126.009	-0.515	0.376	0.171	0.460	1126.009	-1.250	0.434	0.004	0.035
MRPL40	479.520	0.400	0.246	0.104	0.202	479.520	-0.119	0.247	0.630	0.797	479.520	-0.519	0.285	0.069	0.223
MRPL38	472.553	-0.175	0.255	0.492	0.624	472.553	-0.234	0.255	0.359	0.611	472.553	-0.059	0.295	0.842	0.923
MRPL36	1023.217	-0.075	0.314	0.812	0.877	1023.217	-0.452	0.314	0.150	0.443	1023.217	-0.377	0.363	0.299	0.534
SKIL	584.503	-0.143	0.266	0.590	0.706	584.503	-0.442	0.266	0.097	0.392	584.503	-0.298	0.308	0.332	0.567
MRPL34	866.223	0.475	0.254	0.061	0.135	866.223	-0.218	0.254	0.392	0.636	866.223	-0.693	0.294	0.018	0.096
MRPL32	936.764	0.554	0.198	0.005	0.020	936.764	-0.043	0.198	0.826	0.910	936.764	-0.598	0.229	0.009	0.060
SKIV2L	3519.015	0.099	0.154	0.519	0.647	3519.015	0.000	0.154	0.998	0.999	3519.015	-0.100	0.178	0.575	0.761
BMP2	118.351	0.499	0.286	0.081	0.166	118.351	-0.081	0.290	0.779	0.883	118.351	-0.580	0.333	0.081	0.247
SKP1	2534.346	-0.181	0.139	0.192	0.317	2534.346	-0.084	0.138	0.546	0.741	2534.346	0.097	0.160	0.543	0.739
MRPL11	673.527	0.135	0.217	0.533	0.658	673.527	-0.075	0.217	0.729	0.855	673.527	-0.211	0.251	0.401	0.628
MRPL9	1052.430	-0.257	0.165	0.118	0.222	1052.430	-0.097	0.164	0.557	0.747	1052.430	0.161	0.190	0.398	0.626
MRPL1	538.089	-0.304	0.256	0.236	0.369	538.089	-0.288	0.256	0.260	0.532	538.089	0.015	0.296	0.959	0.981
NDRG4	16.978	1.568	0.467	0.001	0.005	16.978	0.284	0.498	0.569	0.756	16.978	-1.285	0.546	0.019	0.097
SLC26A6	425.670	1.319	0.306	0.000	0.000	425.670	0.611	0.307	0.046	0.335	425.670	-0.707	0.353	0.045	0.171
SLC26A10	143.395	1.130	0.333	0.001	0.004	143.395	-0.469	0.341	0.169	0.458	143.395	-1.599	0.389	0.000	0.001
PINK1	2375.932	0.654	0.205	0.001	0.008	2375.932	-0.115	0.205	0.575	0.760	2375.932	-0.770	0.237	0.001	0.015
SKP2	1296.957	-0.248	0.149	0.095	0.188	1296.957	-0.092	0.148	0.533	0.733	1296.957	0.156	0.172	0.364	0.596
SLC1A1	107.180	0.378	0.266	0.155	0.271	107.180	0.176	0.266	0.508	0.717	107.180	-0.202	0.307	0.511	0.716
GPBP1	1906.771	-0.307	0.146	0.035	0.089	1906.771	0.062	0.146	0.670	0.821	1906.771	0.369	0.169	0.028	0.127
ACD	536.592	-0.263	0.260	0.312	0.452	536.592	-0.302	0.260	0.245	0.520	536.592	-0.039	0.300	0.896	0.951
RAPH1	676.394	-0.984	0.235	0.000	0.000	676.394	-0.317	0.233	0.174	0.463	676.394	0.667	0.271	0.014	0.081
TMEM237	440.922	-0.332	0.183	0.069	0.148	440.922	-0.314	0.182	0.085	0.376	440.922	0.018	0.212	0.932	0.969
BEND3P3	61.474	-0.596	0.385	0.122	0.226	61.474	-0.081	0.377	0.830	0.912	61.474	0.515	0.444	0.246	0.477
NBEAL1	873.783	0.434	0.260	0.095	0.188	873.783	0.325	0.259	0.210	0.491	873.783	-0.109	0.300	0.717	0.853
GAS6-AS1	295.015	0.406	0.266	0.126	0.233	295.015	0.211	0.266	0.426	0.662	295.015	-0.195	0.307	0.526	0.726
SLC1A3	1757.933	0.075	0.284	0.792	0.862	1757.933	0.301	0.283	0.288	0.556	1757.933	0.227	0.327	0.489	0.699
CFLAR-AS1	95.254	0.076	0.227	0.738	0.823	95.254	-0.222	0.228	0.331	0.591	95.254	-0.298	0.265	0.261	0.495
RTN4R	14.633	2.882	0.512	0.000	0.000	14.633	0.701	0.574	0.222	0.501	14.633	-2.181	0.588	0.000	0.004
MIPEPP3	16.554	-0.161	0.453	0.722	0.811	16.554	0.034	0.438	0.938	0.968	16.554	0.195	0.521	0.708	0.849
SLC4A3	66.531	1.562	0.318	0.000	0.000	66.531	0.058	0.331	0.861	0.930	66.531	-1.504	0.372	0.000	0.001
MRPL44	419.903	-0.248	0.159	0.118	0.221	419.903	0.114	0.157	0.465	0.689	419.903	0.363	0.183	0.047	0.176
VPS33A	363.971	-0.057	0.207	0.783	0.856	363.971	-0.359	0.208	0.084	0.376	363.971	-0.302	0.241	0.210	0.435
NOL6	1118.147	-1.247	0.428	0.004	0.015	1118.147	-1.212	0.428	0.005	0.297	1118.147	0.035	0.494	0.944	0.974
TMEM135	601.313	-0.019	0.343	0.956	0.973	601.313	0.446	0.343	0.193	0.478	601.313	0.465	0.396	0.240	0.471
SLC1A4	337.591	-0.919	0.182	0.000	0.000	337.591	-0.138	0.177	0.438	0.671	337.591	0.782	0.210	0.000	0.004
JMJD4	320.987	-0.517	0.310	0.095	0.189	320.987	-0.721	0.310	0.020	0.310	320.987	-0.204	0.359	0.571	0.759
KRI1	987.877	-0.126	0.214	0.555	0.677	987.877	-0.162	0.214	0.449	0.678	987.877	-0.036	0.248	0.886	0.945
SLC1A5	10256.515	-0.840	0.171	0.000	0.000	10256.515	-0.078	0.171	0.647	0.807	10256.515	0.761	0.197	0.000	0.003
MARCKSL1	825.378	0.472	0.156	0.003	0.012	825.378	-0.068	0.157	0.665	0.818	825.378	-0.541	0.181	0.003	0.028
UPF3B	535.073	0.427	0.226	0.059	0.131	535.073	-0.111	0.227	0.625	0.794	535.073	-0.538	0.262	0.040	0.159
UPF3A	647.594	0.211	0.181	0.243	0.377	647.594	-0.024	0.181	0.896	0.948	647.594	-0.235	0.209	0.262	0.496
RSRC2	1553.102	-0.514	0.223	0.021	0.061	1553.102	-0.443	0.223	0.047	0.336	1553.102	0.071	0.258	0.782	0.890
INTS3	1990.453	0.071	0.183	0.697	0.791	1990.453	0.284	0.183	0.121	0.413	1990.453	0.213	0.211	0.315	0.550
SOWAHC	1235.451	-0.582	0.343	0.090	0.181	1235.451	-0.865	0.343	0.012	0.305	1235.451	-0.283	0.397	0.476	0.689
WNK1	3567.617	-0.592	0.175	0.001	0.004	3567.617	-0.188	0.175	0.283	0.552	3567.617	0.404	0.202	0.045	0.172
SLC2A1	5666.450	0.246	0.234	0.294	0.432	5666.450	0.324	0.234	0.166	0.456	5666.450	0.078	0.271	0.773	0.884
ZNF192P1	21.047	-0.394	0.434	0.364	0.505	21.047	0.119	0.413	0.773	0.879	21.047	0.513	0.496	0.301	0.536
LOC651337	59.596	-0.597	0.365	0.101	0.197	59.596	-0.216	0.358	0.546	0.740	59.596	0.381	0.421	0.365	0.597
FLJ38576	7.101	0.269	0.740	0.717	0.807	7.101	-0.353	0.760	0.642	0.805	7.101	-0.622	0.874	0.477	0.690
SLC2A3	3569.051	1.836	0.428	0.000	0.000	3569.051	0.871	0.428	0.042	0.330	3569.051	-0.965	0.495	0.051	0.185
SLC2A4	19.840	0.779	0.573	0.174	0.295	19.840	1.507	0.559	0.007	0.297	19.840	0.727	0.643	0.258	0.492
ANKRD33B	448.442	-0.732	0.228	0.001	0.007	448.442	-0.079	0.225	0.727	0.854	448.442	0.654	0.263	0.013	0.077
SLC2A5	13.181	1.490	0.950	0.117	0.220	13.181	0.218	0.974	0.823	0.908	13.181	-1.272	1.103	0.249	0.481
SLC3A1	303.912	-0.963	0.203	0.000	0.000	303.912	-0.031	0.198	0.873	0.936	303.912	0.932	0.233	0.000	0.002
BMP4	202.616	0.370	0.201	0.065	0.141	202.616	0.326	0.200	0.103	0.396	202.616	-0.044	0.231	0.848	0.926

SLC3A2	9690.467	-1.235	0.272	0.000	0.000	9690.467	-0.440	0.271	0.105	0.398	9690.467	0.795	0.314	0.011	0.070
SLC4A2	2502.568	-0.501	0.118	0.000	0.000	2502.568	-0.096	0.117	0.412	0.651	2502.568	0.404	0.136	0.003	0.028
NADK	1635.013	-0.177	0.201	0.378	0.519	1635.013	-0.349	0.201	0.083	0.374	1635.013	-0.172	0.233	0.461	0.678
LOC652276	90.054	-0.955	0.289	0.001	0.006	90.054	-0.493	0.279	0.078	0.369	90.054	0.462	0.334	0.167	0.382
SLC5A2	596.522	0.441	0.196	0.025	0.067	596.522	0.290	0.196	0.140	0.431	596.522	-0.151	0.227	0.504	0.711
ZFP69B	52.418	-2.086	0.464	0.000	0.000	52.418	-1.212	0.444	0.006	0.297	52.418	0.874	0.537	0.103	0.286
SPATS2	783.456	-0.243	0.093	0.009	0.031	783.456	0.019	0.091	0.837	0.916	783.456	0.262	0.107	0.014	0.083
ZSWIM4	302.964	0.177	0.131	0.177	0.299	302.964	0.107	0.130	0.409	0.649	302.964	-0.069	0.151	0.648	0.810
SMTN	639.629	-0.094	0.198	0.633	0.742	639.629	0.249	0.196	0.205	0.488	639.629	0.343	0.228	0.132	0.330
CPLANE1	183.010	-0.018	0.271	0.949	0.968	183.010	-0.153	0.271	0.574	0.759	183.010	-0.135	0.314	0.667	0.823
ZNF649	118.936	-0.688	0.279	0.014	0.043	118.936	-0.492	0.276	0.074	0.364	118.936	0.196	0.324	0.545	0.741
MPPE1	181.146	0.364	0.160	0.023	0.064	181.146	-0.161	0.163	0.322	0.583	181.146	-0.525	0.187	0.005	0.041
SLC5A3	1822.206	0.000	0.342	0.999	1.000	1822.206	0.024	0.342	0.943	0.971	1822.206	0.024	0.395	0.951	0.978
COA7	937.740	-0.489	0.268	0.069	0.147	937.740	-0.660	0.268	0.014	0.306	937.740	-0.172	0.310	0.580	0.764
PYCR3	392.179	-0.746	0.481	0.121	0.226	392.179	-0.991	0.482	0.040	0.327	392.179	-0.245	0.557	0.660	0.818
UBE2Z	2624.798	-0.174	0.163	0.286	0.424	2624.798	0.100	0.163	0.542	0.738	2624.798	0.274	0.189	0.146	0.353
C8orf33	920.068	-0.978	0.250	0.000	0.001	920.068	-0.562	0.249	0.024	0.316	920.068	0.416	0.289	0.150	0.359
WNK4	29.598	1.733	0.488	0.000	0.003	29.598	0.786	0.497	0.114	0.406	29.598	-0.947	0.559	0.090	0.263
SNORA48	9.146	0.193	0.563	0.732	0.819	9.146	-0.399	0.588	0.498	0.711	9.146	-0.592	0.677	0.382	0.611
CASTOR1	142.317	0.861	0.594	0.148	0.261	142.317	0.504	0.595	0.397	0.641	142.317	-0.357	0.686	0.603	0.780
LRRC37A5P	20.971	0.452	0.689	0.512	0.641	20.971	0.121	0.692	0.862	0.930	20.971	-0.331	0.797	0.678	0.829
XAGE1B	1233.389	0.794	0.240	0.001	0.005	1233.389	0.034	0.240	0.888	0.944	1233.389	-0.760	0.277	0.006	0.047
GOLGA8T	11.897	-0.926	0.589	0.116	0.218	11.897	-0.230	0.551	0.677	0.825	11.897	0.696	0.676	0.303	0.538
GUSBP16	64.582	0.031	0.384	0.937	0.960	64.582	0.665	0.377	0.078	0.370	64.582	0.634	0.439	0.149	0.357
ZDHHC11B	12.097	1.642	0.708	0.020	0.058	12.097	0.753	0.723	0.297	0.565	12.097	-0.888	0.811	0.273	0.508
ZBTB8A	193.841	0.236	0.171	0.167	0.287	193.841	0.142	0.170	0.405	0.646	193.841	-0.094	0.197	0.633	0.800
ANXA8	4345.135	0.402	0.302	0.182	0.306	4345.135	0.073	0.302	0.809	0.900	4345.135	-0.330	0.348	0.344	0.578
LOC653160	26.689	1.345	0.559	0.016	0.049	26.689	0.669	0.566	0.237	0.514	26.689	-0.676	0.642	0.292	0.528
RPSAP9	3826.154	-0.632	0.254	0.013	0.041	3826.154	0.299	0.254	0.238	0.515	3826.154	0.932	0.293	0.001	0.018
GUSBP3	36.975	0.271	0.389	0.487	0.620	36.975	1.061	0.376	0.005	0.297	36.975	0.790	0.439	0.072	0.229
XAGE1A	450.450	0.641	0.284	0.024	0.066	450.450	0.109	0.285	0.702	0.840	450.450	-0.532	0.328	0.105	0.289
AGAP10P	36.864	0.946	0.399	0.018	0.052	36.864	0.094	0.409	0.818	0.905	36.864	-0.852	0.464	0.067	0.218
GTF2H2B	313.200	-0.587	0.238	0.014	0.043	313.200	-0.353	0.237	0.136	0.427	313.200	0.234	0.276	0.397	0.625
AGAP7P	36.616	1.242	0.426	0.004	0.015	36.616	0.296	0.436	0.498	0.712	36.616	-0.947	0.493	0.055	0.195
SLC6A6	3097.175	0.109	0.182	0.551	0.673	3097.175	0.200	0.182	0.272	0.543	3097.175	0.091	0.210	0.664	0.821
ASAH2B	148.999	-0.977	0.357	0.006	0.023	148.999	-0.793	0.355	0.026	0.316	148.999	0.184	0.414	0.656	0.816
FAM153CP	78.796	2.050	0.457	0.000	0.000	78.796	1.234	0.461	0.007	0.297	78.796	-0.815	0.522	0.118	0.308
KIAA0895L	266.274	1.002	0.353	0.004	0.018	266.274	0.583	0.353	0.099	0.394	266.274	-0.420	0.407	0.302	0.537
FAM86B2	125.410	-1.291	0.300	0.000	0.000	125.410	-0.356	0.290	0.219	0.499	125.410	0.935	0.345	0.007	0.050
SORD2P	398.717	-0.579	0.164	0.000	0.003	398.717	-0.024	0.161	0.881	0.940	398.717	0.555	0.188	0.003	0.030
SRGAP2C	751.934	-0.097	0.113	0.388	0.527	751.934	0.039	0.112	0.731	0.856	751.934	0.136	0.130	0.296	0.532
MRPL45P2	71.449	-0.026	0.334	0.938	0.961	71.449	-0.122	0.333	0.713	0.846	71.449	-0.097	0.388	0.803	0.902
RGPD3	53.422	-0.466	0.370	0.208	0.337	53.422	-0.465	0.368	0.206	0.488	53.422	0.001	0.431	0.998	0.999
SLC6A8	7575.807	0.301	0.168	0.072	0.153	7575.807	0.215	0.168	0.199	0.482	7575.807	-0.086	0.193	0.656	0.816
LOC653513	25.979	0.864	0.418	0.039	0.095	25.979	0.269	0.426	0.528	0.729	25.979	-0.595	0.484	0.219	0.446
GPR89A	285.480	-0.050	0.188	0.790	0.861	285.480	-0.133	0.187	0.477	0.698	285.480	-0.083	0.218	0.703	0.845
PHLDB3	394.183	0.495	0.512	0.333	0.473	394.183	0.821	0.511	0.108	0.401	394.183	0.326	0.590	0.581	0.764
SLC6A9	746.531	-1.340	0.288	0.000	0.000	746.531	-0.298	0.285	0.296	0.564	746.531	1.042	0.332	0.002	0.019
WASH7P	326.283	0.780	0.261	0.003	0.013	326.283	0.405	0.261	0.121	0.412	326.283	-0.374	0.301	0.214	0.439
TBC1D3P1-DHX40P1	44.875	-0.385	0.594	0.517	0.645	44.875	0.480	0.584	0.411	0.651	44.875	0.865	0.682	0.205	0.428
LOC653653	36.289	-0.940	0.305	0.002	0.010	36.289	0.236	0.256	0.356	0.610	36.289	1.177	0.338	0.000	0.008
TMEM183B	107.299	0.133	0.254	0.601	0.715	107.299	-0.207	0.256	0.419	0.656	107.299	-0.340	0.296	0.251	0.484
SEC1P	19.835	1.632	0.606	0.007	0.026	19.835	0.832	0.620	0.179	0.467	19.835	-0.799	0.691	0.247	0.478
GSTT2B	90.577	2.120	0.305	0.000	0.000	90.577	0.817	0.313	0.009	0.297	90.577	-1.304	0.350	0.000	0.004
TCAF2P1	130.519	0.504	0.340	0.138	0.248	130.519	0.635	0.338	0.060	0.350	130.519	0.131	0.390	0.736	0.864
MZT2A	1473.723	0.717	0.262	0.006	0.023	1473.723	0.424	0.262	0.105	0.398	1473.723	-0.293	0.302	0.333	0.568
FAM72B	525.048	-0.794	0.213	0.000	0.002	525.048	-0.054	0.211	0.799	0.896	525.048	0.740	0.246	0.003	0.026
BMP6	327.477	1.287	0.369	0.000	0.003	327.477	-0.169	0.372	0.649	0.808	327.477	-1.457	0.428	0.001	0.010
SLC7A1	2530.174	-1.491	0.257	0.000	0.000	2530.174	-0.490	0.256	0.055	0.343	2530.174	1.000	0.296	0.001	0.011
SLC7A2	4853.312	-1.467	0.304	0.000	0.000	4853.312	-0.427	0.303	0.159	0.451	4853.312	1.039	0.351	0.003	0.029
LSP1P4	358.075	0.620	0.213	0.004	0.015	358.075	0.491	0.213	0.021	0.313	358.075	-0.129	0.245	0.600	0.778
PAX8-AS1	69.707	0.586	0.399	0.142	0.254	69.707	0.677	0.393	0.085	0.376	69.707	0.092	0.452	0.839	0.922
SNHG20	70.087	0.116	0.244	0.635	0.743	70.087	-0.093	0.245	0.705	0.842	70.087	-0.209	0.285	0.463	0.679
FGF7P3	262.183	-0.286	0.263	0.277	0.414	262.183	0.454	0.259	0.080	0.371	262.183	0.740	0.302	0.014	0.083
SLC8A1	11.034	-1.222	0.520	0.019	0.055	11.034	-0.786	0.484	0.105	0.397	11.034	0.437	0.609	0.473	0.687
SLC9A1	1303.691	-0.044	0.234	0.852	0.903	1303.691	-0.260	0.234	0.266	0.538	1303.691	-0.216	0.270	0.423	0.647
MFF-DT	150.796	-0.369	0.157	0.019	0.056	150.796	0.016	0.151	0.913	0.957	150.796	0.385	0.181	0.033	0.140

SLC9A2	135.036	-0.381	0.306	0.213	0.343	135.036	-0.285	0.305	0.349	0.604	135.036	0.096	0.355	0.786	0.893
SLC9A3	514.859	0.561	0.282	0.047	0.110	514.859	0.550	0.282	0.051	0.338	514.859	-0.011	0.325	0.972	0.986
SLC9A5	396.199	-0.340	0.186	0.067	0.145	396.199	0.104	0.184	0.572	0.758	396.199	0.444	0.214	0.038	0.154
SLC12A2	377.667	-0.427	0.201	0.034	0.086	377.667	-0.242	0.200	0.225	0.504	377.667	0.185	0.233	0.426	0.649
BMP8B	43.069	0.981	0.293	0.001	0.005	43.069	0.160	0.304	0.599	0.777	43.069	-0.821	0.342	0.016	0.090
SLC12A4	1418.281	0.278	0.182	0.126	0.232	1418.281	0.150	0.182	0.409	0.649	1418.281	-0.128	0.210	0.541	0.738
SLC14A1	55.423	-1.784	0.489	0.000	0.002	55.423	-1.472	0.479	0.002	0.297	55.423	0.311	0.571	0.586	0.767
SLC15A2	22.352	-0.120	0.481	0.803	0.870	22.352	0.449	0.465	0.334	0.593	22.352	0.569	0.548	0.299	0.534
SLC16A1	5898.674	-0.135	0.139	0.331	0.472	5898.674	0.016	0.138	0.906	0.953	5898.674	0.151	0.160	0.346	0.580
SLC16A2	10.422	2.663	0.506	0.000	0.000	10.422	1.306	0.546	0.017	0.306	10.422	-1.357	0.543	0.012	0.076
BMPR1A	591.120	-0.569	0.154	0.000	0.002	591.120	-0.168	0.152	0.270	0.541	591.120	0.402	0.177	0.024	0.113
SLC18A2	158.868	-0.106	0.251	0.672	0.772	158.868	0.340	0.247	0.168	0.457	158.868	0.447	0.288	0.121	0.313
SLC19A1	1203.524	0.119	0.272	0.663	0.765	1203.524	-0.610	0.273	0.025	0.316	1203.524	-0.728	0.315	0.021	0.103
SLC20A1	2626.431	-0.375	0.355	0.291	0.429	2626.431	-0.772	0.355	0.030	0.322	2626.431	-0.397	0.411	0.333	0.568
SLC20A2	455.682	-0.644	0.156	0.000	0.000	455.682	-0.097	0.153	0.524	0.727	455.682	0.547	0.179	0.002	0.024
SLC25A1	2414.444	1.268	0.221	0.000	0.000	2414.444	0.338	0.222	0.128	0.420	2414.444	-0.930	0.256	0.000	0.005
SLCO2A1	115.149	-0.276	0.574	0.631	0.740	115.149	0.384	0.571	0.501	0.713	115.149	0.660	0.662	0.318	0.554
SLCO1A2	70.984	-1.167	0.590	0.048	0.112	70.984	0.531	0.578	0.358	0.611	70.984	1.697	0.677	0.012	0.075
BMPR1B	43.921	-0.532	0.327	0.104	0.201	43.921	0.107	0.312	0.732	0.856	43.921	0.639	0.374	0.088	0.259
SLC22A4	18.697	0.429	0.481	0.372	0.512	18.697	0.390	0.475	0.412	0.651	18.697	-0.039	0.549	0.943	0.974
SLC22A5	128.448	0.568	0.164	0.001	0.004	128.448	0.377	0.165	0.022	0.315	128.448	-0.192	0.189	0.310	0.545
BMPR2	630.540	-0.081	0.208	0.699	0.792	630.540	-0.044	0.208	0.834	0.914	630.540	0.037	0.240	0.878	0.942
SLPI	512.701	1.631	0.477	0.001	0.004	512.701	1.365	0.477	0.004	0.297	512.701	-0.266	0.550	0.628	0.797
SNAI2	1030.570	-0.321	0.160	0.045	0.106	1030.570	-0.423	0.160	0.008	0.297	1030.570	-0.102	0.185	0.582	0.765
SMARCA1	208.849	-0.241	0.284	0.396	0.534	208.849	0.471	0.280	0.092	0.387	208.849	0.713	0.326	0.029	0.128
SMARCA2	1212.617	-0.339	0.202	0.093	0.186	1212.617	0.201	0.202	0.320	0.581	1212.617	0.540	0.233	0.021	0.103
HLTF	359.553	-0.052	0.343	0.878	0.921	359.553	0.151	0.342	0.660	0.814	359.553	0.203	0.395	0.608	0.783
SMARCA4	4063.960	0.201	0.172	0.244	0.377	4063.960	-0.179	0.172	0.299	0.566	4063.960	-0.379	0.199	0.056	0.197
PLEKHA3	307.178	-0.416	0.188	0.027	0.073	307.178	-0.233	0.186	0.211	0.491	307.178	0.183	0.218	0.402	0.629
PHACTR4	742.972	0.038	0.091	0.677	0.775	742.972	-0.018	0.090	0.842	0.918	742.972	-0.056	0.105	0.595	0.774
SMARCB1	3134.936	-0.095	0.203	0.639	0.746	3134.936	-0.044	0.202	0.830	0.912	3134.936	0.051	0.234	0.826	0.914
BRD9	1657.957	0.134	0.203	0.510	0.640	1657.957	-0.245	0.203	0.228	0.506	1657.957	-0.379	0.235	0.107	0.291
CAPRIN2	176.024	-0.344	0.339	0.310	0.450	176.024	-0.517	0.339	0.127	0.420	176.024	-0.173	0.393	0.660	0.818
GRAMD2B	16.242	1.795	0.447	0.000	0.001	16.242	0.487	0.491	0.321	0.582	16.242	-1.309	0.517	0.011	0.071
AACS	1316.702	0.506	0.220	0.021	0.060	1316.702	0.255	0.220	0.246	0.521	1316.702	-0.251	0.254	0.322	0.557
ZBTB10	479.400	0.158	0.188	0.400	0.539	479.400	0.039	0.188	0.836	0.916	479.400	-0.119	0.217	0.583	0.766
KCTD14	83.295	0.577	0.192	0.003	0.012	83.295	-0.052	0.200	0.794	0.893	83.295	-0.629	0.227	0.006	0.044
ZNF747	191.179	-0.112	0.188	0.552	0.674	191.179	0.183	0.185	0.321	0.582	191.179	0.295	0.216	0.172	0.389
DLK2	291.682	0.397	0.296	0.180	0.302	291.682	0.601	0.295	0.042	0.330	291.682	0.204	0.341	0.550	0.744
SMARCC1	2833.663	-0.825	0.216	0.000	0.001	2833.663	-0.394	0.216	0.068	0.356	2833.663	0.431	0.250	0.085	0.253
ANTKMT	293.425	0.653	0.330	0.048	0.112	293.425	-0.293	0.333	0.379	0.626	293.425	-0.946	0.383	0.014	0.080
FUNDC2	705.836	0.098	0.121	0.421	0.558	705.836	0.071	0.121	0.556	0.747	705.836	-0.026	0.140	0.850	0.928
DDRGK1	1049.353	0.354	0.178	0.047	0.110	1049.353	0.040	0.178	0.821	0.906	1049.353	-0.314	0.206	0.127	0.322
MRPS34	1775.203	0.232	0.230	0.312	0.452	1775.203	-0.236	0.230	0.306	0.571	1775.203	-0.468	0.266	0.078	0.241
CENPBD1P1	845.553	-0.036	0.127	0.775	0.850	845.553	0.227	0.126	0.071	0.360	845.553	0.263	0.146	0.072	0.229
RASL11B	131.862	0.748	0.263	0.004	0.018	131.862	0.308	0.265	0.244	0.519	131.862	-0.440	0.304	0.148	0.356
C11orf95	586.074	-0.365	0.291	0.209	0.338	586.074	0.448	0.289	0.122	0.413	586.074	0.813	0.335	0.015	0.086
CHID1	895.655	0.106	0.181	0.557	0.679	895.655	-0.235	0.181	0.196	0.479	895.655	-0.341	0.209	0.104	0.286
TRAK2	452.626	-0.191	0.197	0.331	0.471	452.626	-0.549	0.197	0.005	0.297	452.626	-0.358	0.229	0.118	0.307
SMARCC2	1728.767	-0.251	0.101	0.013	0.042	1728.767	0.023	0.101	0.822	0.907	1728.767	0.274	0.117	0.019	0.099
SMARCD1	870.006	-0.008	0.194	0.968	0.980	870.006	-0.415	0.194	0.032	0.322	870.006	-0.408	0.224	0.069	0.224
SMARCD2	2569.389	-0.113	0.260	0.663	0.765	2569.389	0.387	0.260	0.137	0.428	2569.389	0.500	0.300	0.096	0.272
SLC2A11	159.279	0.767	0.367	0.037	0.091	159.279	0.632	0.367	0.085	0.377	159.279	-0.135	0.422	0.749	0.871
MTMR9	303.477	-0.541	0.291	0.064	0.139	303.477	-0.725	0.292	0.013	0.305	303.477	-0.185	0.338	0.585	0.767
SMARCD3	586.197	1.323	0.218	0.000	0.000	586.197	0.344	0.220	0.117	0.409	586.197	-0.979	0.252	0.000	0.002
SMARCE1	1213.092	-0.052	0.134	0.698	0.791	1213.092	0.053	0.133	0.692	0.834	1213.092	0.105	0.154	0.497	0.705
SMN1	142.092	0.025	0.432	0.954	0.972	142.092	-0.680	0.434	0.118	0.409	142.092	-0.704	0.501	0.160	0.373
SMN2	791.544	0.540	0.354	0.127	0.233	791.544	-0.179	0.354	0.613	0.787	791.544	-0.719	0.409	0.079	0.242
SMPD1	331.817	1.089	0.160	0.000	0.000	331.817	0.122	0.164	0.457	0.683	331.817	-0.967	0.186	0.000	0.000
POLR3D	1023.253	-0.790	0.186	0.000	0.000	1023.253	-0.170	0.184	0.356	0.610	1023.253	0.620	0.214	0.004	0.034
SMPD2	187.994	0.171	0.223	0.444	0.580	187.994	-0.066	0.224	0.767	0.877	187.994	-0.237	0.259	0.360	0.593
SMS	4220.324	0.155	0.126	0.219	0.350	4220.324	-0.126	0.127	0.321	0.582	4220.324	-0.281	0.146	0.054	0.193
SUMO3	2100.497	-0.082	0.143	0.567	0.687	2100.497	0.026	0.143	0.857	0.927	2100.497	0.108	0.165	0.514	0.719
SUMO2	4417.199	0.098	0.157	0.531	0.657	4417.199	0.129	0.157	0.412	0.651	4417.199	0.030	0.181	0.867	0.936
SNAI1	67.478	0.987	0.246	0.000	0.001	67.478	0.500	0.250	0.046	0.335	67.478	-0.487	0.282	0.084	0.252
SNAPC1	2052.406	-0.994	0.421	0.018	0.054	2052.406	-1.108	0.421	0.008	0.297	2052.406	-0.115	0.486	0.814	0.907
SNAPC2	638.046	0.262	0.175	0.134	0.244	638.046	0.372	0.174	0.033	0.322	638.046	0.110	0.202	0.586	0.767

SNAPC3	881.288	-0.162	0.163	0.320	0.460	881.288	-0.062	0.162	0.703	0.841	881.288	0.100	0.188	0.594	0.774
BNIP1	196.280	-0.413	0.214	0.053	0.121	196.280	-0.127	0.211	0.548	0.742	196.280	0.287	0.247	0.246	0.477
SNAPC4	351.960	-0.015	0.254	0.952	0.970	351.960	-0.440	0.255	0.084	0.376	351.960	-0.425	0.294	0.149	0.358
SNCA	454.627	1.065	0.300	0.000	0.003	454.627	0.222	0.301	0.460	0.687	454.627	-0.842	0.347	0.015	0.086
FSCN1	15206.321	0.299	0.155	0.053	0.121	15206.321	0.253	0.155	0.102	0.394	15206.321	-0.046	0.179	0.797	0.898
SNRNP70	4030.904	0.327	0.304	0.283	0.421	4030.904	-0.428	0.305	0.160	0.451	4030.904	-0.755	0.352	0.032	0.137
SNRPA	1810.724	0.457	0.231	0.048	0.112	1810.724	0.033	0.231	0.887	0.944	1810.724	-0.424	0.267	0.112	0.299
SNRPA1	899.632	-0.126	0.256	0.622	0.732	899.632	-0.489	0.256	0.056	0.344	899.632	-0.363	0.296	0.221	0.448
SNRPB	3988.884	0.091	0.301	0.762	0.842	3988.884	-0.571	0.301	0.058	0.348	3988.884	-0.662	0.348	0.057	0.199
SNRPB2	930.740	0.364	0.262	0.164	0.284	930.740	-0.351	0.262	0.181	0.469	930.740	-0.714	0.303	0.018	0.096
BNIP2	976.271	-0.644	0.197	0.001	0.006	976.271	-0.350	0.196	0.074	0.364	976.271	0.293	0.228	0.198	0.421
SNRPC	984.770	0.159	0.263	0.546	0.670	984.770	-0.337	0.263	0.201	0.484	984.770	-0.496	0.304	0.103	0.285
SNRPD1	886.617	0.016	0.304	0.959	0.975	886.617	-0.562	0.305	0.065	0.353	886.617	-0.578	0.352	0.100	0.281
SNRPD2	4025.428	0.579	0.193	0.003	0.012	4025.428	0.147	0.193	0.447	0.677	4025.428	-0.433	0.223	0.052	0.188
SNRPD3	702.078	-0.205	0.112	0.068	0.146	702.078	-0.183	0.111	0.100	0.394	702.078	0.021	0.130	0.869	0.937
SNRPE	1206.166	0.233	0.180	0.194	0.319	1206.166	-0.120	0.180	0.505	0.715	1206.166	-0.353	0.208	0.089	0.261
SNRPF	1053.117	0.618	0.288	0.032	0.082	1053.117	-0.235	0.288	0.416	0.654	1053.117	-0.852	0.333	0.010	0.068
SNRPG	845.846	0.365	0.347	0.293	0.431	845.846	-0.684	0.348	0.050	0.337	845.846	-1.049	0.402	0.009	0.061
SNRPN	42.000	-1.262	0.474	0.008	0.028	42.000	-0.546	0.452	0.227	0.505	42.000	0.716	0.547	0.191	0.413
BNIP3	7461.662	-0.011	0.663	0.987	0.993	7461.662	0.773	0.663	0.244	0.519	7461.662	0.784	0.766	0.306	0.542
SNTA1	618.312	0.750	0.379	0.048	0.112	618.312	0.567	0.379	0.134	0.426	618.312	-0.183	0.437	0.676	0.828
SNTB1	799.339	-0.173	0.287	0.547	0.670	799.339	0.409	0.286	0.153	0.445	799.339	0.582	0.331	0.079	0.243
SNX1	1473.291	-0.204	0.099	0.040	0.098	1473.291	0.020	0.099	0.843	0.918	1473.291	0.224	0.115	0.051	0.186
SNX2	1458.741	-0.132	0.135	0.330	0.470	1458.741	0.005	0.135	0.972	0.985	1458.741	0.137	0.156	0.382	0.611
SNTB2	658.360	0.467	0.207	0.024	0.065	658.360	0.365	0.206	0.077	0.368	658.360	-0.103	0.238	0.667	0.823
SOAT1	778.522	0.108	0.234	0.646	0.752	778.522	-0.426	0.235	0.070	0.358	778.522	-0.534	0.271	0.049	0.181
SOD1	3666.377	0.726	0.235	0.002	0.010	3666.377	-0.152	0.235	0.518	0.723	3666.377	-0.878	0.271	0.001	0.015
HNRNPA1P10	1401.584	-0.185	0.179	0.300	0.438	1401.584	0.193	0.178	0.278	0.548	1401.584	0.378	0.206	0.066	0.218
SOD2	100095.250	1.066	0.201	0.000	0.000	100095.250	-0.077	0.201	0.702	0.839	100095.250	-1.144	0.232	0.000	0.000
BNIP3L	3734.316	0.439	0.492	0.372	0.512	3734.316	0.680	0.491	0.166	0.456	3734.316	0.242	0.568	0.670	0.824
CAPN15	1869.253	-0.067	0.226	0.768	0.846	1869.253	-0.256	0.226	0.258	0.530	1869.253	-0.189	0.261	0.469	0.684
SON	2986.362	-0.259	0.143	0.069	0.148	2986.362	-0.305	0.143	0.033	0.322	2986.362	-0.046	0.165	0.782	0.891
SORD	2008.501	-0.975	0.174	0.000	0.000	2008.501	-0.208	0.173	0.230	0.507	2008.501	0.767	0.201	0.000	0.003
SORL1	533.027	-0.899	0.464	0.053	0.120	533.027	0.409	0.462	0.377	0.624	533.027	1.308	0.535	0.015	0.083
SOS1	623.565	-0.314	0.252	0.213	0.342	623.565	0.103	0.251	0.680	0.827	623.565	0.417	0.291	0.151	0.360
SOS2	340.932	-0.501	0.195	0.010	0.034	340.932	-0.082	0.192	0.668	0.820	340.932	0.419	0.224	0.062	0.209
SOX4	1241.480	0.684	0.194	0.000	0.003	1241.480	0.171	0.194	0.378	0.626	1241.480	-0.513	0.224	0.022	0.107
BOK	365.040	0.273	0.161	0.090	0.181	365.040	-0.036	0.162	0.825	0.909	365.040	-0.309	0.187	0.099	0.277
SOX5	67.703	-0.139	0.433	0.748	0.832	67.703	0.335	0.428	0.434	0.668	67.703	0.474	0.498	0.342	0.576
SOX9	77.621	0.607	0.303	0.045	0.107	77.621	0.128	0.307	0.676	0.825	77.621	-0.479	0.352	0.173	0.390
SOX12	1814.470	0.117	0.220	0.595	0.710	1814.470	0.385	0.220	0.080	0.371	1814.470	0.268	0.254	0.292	0.527
SP1	1501.662	-0.244	0.189	0.198	0.324	1501.662	0.201	0.189	0.287	0.556	1501.662	0.445	0.218	0.042	0.163
SP2	296.993	-0.850	0.196	0.000	0.000	296.993	-0.183	0.191	0.340	0.598	296.993	0.667	0.226	0.003	0.029
DST	4320.167	-0.323	0.237	0.172	0.292	4320.167	-0.049	0.236	0.835	0.915	4320.167	0.274	0.273	0.316	0.552
SP3	1113.100	-0.667	0.120	0.000	0.000	1113.100	-0.098	0.118	0.410	0.650	1113.100	0.569	0.138	0.000	0.001
SP4	91.575	-0.969	0.219	0.000	0.000	91.575	-0.112	0.203	0.580	0.764	91.575	0.857	0.250	0.001	0.009
SP100	1345.720	0.141	0.096	0.143	0.254	1345.720	0.057	0.096	0.554	0.746	1345.720	-0.084	0.111	0.448	0.668
SPAG1	153.202	-0.100	0.248	0.688	0.784	153.202	-0.476	0.249	0.056	0.344	153.202	-0.376	0.289	0.193	0.415
UAP1	2235.521	-0.349	0.362	0.336	0.476	2235.521	-0.926	0.363	0.011	0.299	2235.521	-0.577	0.419	0.168	0.383
SPAG4	438.717	0.886	0.835	0.288	0.426	438.717	1.055	0.834	0.206	0.488	438.717	0.168	0.963	0.861	0.933
SPARC	4543.551	0.492	0.279	0.079	0.163	4543.551	0.083	0.279	0.766	0.876	4543.551	-0.408	0.323	0.206	0.430
SPAST	217.853	-0.714	0.162	0.000	0.000	217.853	-0.164	0.156	0.294	0.561	217.853	0.550	0.187	0.003	0.030
SPG7	703.958	0.425	0.171	0.013	0.041	703.958	-0.249	0.172	0.148	0.441	703.958	-0.674	0.198	0.001	0.010
BPGM	140.563	0.394	0.185	0.033	0.084	140.563	-0.305	0.190	0.107	0.400	140.563	-0.699	0.217	0.001	0.016
SPINT1	96.252	0.491	0.228	0.031	0.081	96.252	0.496	0.226	0.028	0.321	96.252	0.005	0.260	0.986	0.992
SPOCK1	609.932	0.286	0.254	0.260	0.395	609.932	0.258	0.253	0.308	0.573	609.932	-0.028	0.293	0.925	0.965
SPR	413.543	0.686	0.228	0.003	0.012	413.543	0.012	0.229	0.957	0.978	413.543	-0.674	0.264	0.011	0.069
BPHL	188.337	0.637	0.187	0.001	0.004	188.337	0.235	0.189	0.214	0.493	188.337	-0.402	0.217	0.064	0.212
SPTA1	11.035	1.121	0.402	0.005	0.021	11.035	-0.713	0.484	0.141	0.431	11.035	-1.834	0.521	0.000	0.007
SPTAN1	5341.276	-0.429	0.151	0.004	0.018	5341.276	0.252	0.150	0.094	0.388	5341.276	0.681	0.174	0.000	0.002
SPTB	48.965	-0.184	0.299	0.537	0.662	48.965	0.139	0.291	0.631	0.799	48.965	0.324	0.343	0.345	0.579
SPTBN1	6043.120	-0.707	0.148	0.000	0.000	6043.120	-0.083	0.148	0.573	0.759	6043.120	0.623	0.171	0.000	0.005
SPTBN2	1788.094	-0.268	0.170	0.116	0.218	1788.094	0.207	0.170	0.223	0.501	1788.094	0.475	0.196	0.016	0.088
SQLE	2659.945	1.341	0.142	0.000	0.000	2659.945	-0.357	0.143	0.013	0.305	2659.945	-1.698	0.165	0.000	0.000
SRC	1132.646	0.267	0.116	0.021	0.060	1132.646	0.068	0.116	0.559	0.748	1132.646	-0.199	0.134	0.136	0.338
SRD5A1	676.509	0.205	0.185	0.268	0.404	676.509	0.169	0.185	0.359	0.611	676.509	-0.036	0.214	0.868	0.936
SRI	1296.953	-0.167	0.156	0.286	0.424	1296.953	-0.010	0.156	0.948	0.974	1296.953	0.157	0.181	0.386	0.615

BRCA1	564.127	-1.155	0.397	0.004	0.015	564.127	-0.626	0.396	0.114	0.406	564.127	0.529	0.459	0.249	0.481
SREBF1	1962.682	0.186	0.235	0.429	0.566	1962.682	0.166	0.235	0.481	0.700	1962.682	-0.020	0.271	0.940	0.973
SREBF2	5325.355	0.469	0.191	0.014	0.043	5325.355	0.063	0.191	0.742	0.863	5325.355	-0.406	0.220	0.065	0.215
SRF	1413.190	-0.459	0.209	0.028	0.075	1413.190	-0.383	0.209	0.067	0.354	1413.190	0.076	0.241	0.753	0.874
SRM	2196.932	-0.145	0.382	0.706	0.798	2196.932	-0.702	0.383	0.066	0.354	2196.932	-0.558	0.442	0.207	0.431
SRP9	3312.005	-0.330	0.116	0.004	0.018	3312.005	-0.223	0.116	0.053	0.342	3312.005	0.107	0.134	0.426	0.649
SRP14	4660.271	0.833	0.232	0.000	0.002	4660.271	-0.004	0.232	0.987	0.993	4660.271	-0.837	0.268	0.002	0.020
SRP19	607.781	-0.102	0.319	0.750	0.833	607.781	-0.706	0.320	0.027	0.319	607.781	-0.605	0.370	0.102	0.284
SRP54	1005.382	-0.007	0.228	0.974	0.984	1005.382	-0.438	0.229	0.055	0.343	1005.382	-0.430	0.264	0.103	0.286
BRAF	545.274	-0.833	0.277	0.003	0.012	545.274	0.071	0.275	0.796	0.894	545.274	0.904	0.320	0.005	0.039
SRP68	2420.129	-0.517	0.134	0.000	0.001	2420.129	-0.025	0.134	0.850	0.923	2420.129	0.491	0.155	0.002	0.018
SRP72	2947.518	-0.796	0.151	0.000	0.000	2947.518	-0.095	0.150	0.528	0.729	2947.518	0.702	0.174	0.000	0.001
SRPK1	1987.203	-0.891	0.262	0.001	0.004	1987.203	-0.522	0.261	0.046	0.335	1987.203	0.369	0.302	0.222	0.449
SRPK2	789.338	0.066	0.120	0.580	0.697	789.338	-0.050	0.120	0.675	0.824	789.338	-0.117	0.139	0.400	0.627
SRPRA	2988.811	-0.273	0.284	0.337	0.477	2988.811	-0.631	0.284	0.026	0.318	2988.811	-0.358	0.328	0.275	0.510
TRIM21	295.827	0.666	0.190	0.000	0.003	295.827	0.189	0.191	0.324	0.584	295.827	-0.478	0.220	0.030	0.131
RO60	721.975	-0.395	0.152	0.009	0.032	721.975	-0.225	0.151	0.137	0.429	721.975	0.171	0.176	0.332	0.567
SSB	2877.556	0.057	0.256	0.825	0.885	2877.556	-0.494	0.256	0.054	0.342	2877.556	-0.551	0.296	0.063	0.211
SSBP1	3255.559	0.246	0.261	0.346	0.487	3255.559	-0.365	0.261	0.162	0.453	3255.559	-0.611	0.302	0.043	0.166
ITPRID2	1230.558	-1.014	0.221	0.000	0.000	1230.558	-0.076	0.220	0.731	0.856	1230.558	0.939	0.255	0.000	0.005
SSR1	3187.436	-0.315	0.153	0.040	0.097	3187.436	-0.151	0.153	0.324	0.584	3187.436	0.164	0.177	0.353	0.586
SSR2	3581.234	0.396	0.226	0.079	0.164	3581.234	0.318	0.226	0.159	0.451	3581.234	-0.078	0.261	0.763	0.880
SSR3	3890.593	-0.294	0.204	0.149	0.263	3890.593	-0.307	0.204	0.131	0.423	3890.593	-0.013	0.235	0.955	0.979
SSR4	2157.438	0.751	0.211	0.000	0.003	2157.438	0.287	0.211	0.173	0.463	2157.438	-0.464	0.243	0.057	0.199
SSRP1	6651.025	-0.509	0.155	0.001	0.006	6651.025	-0.162	0.155	0.296	0.564	6651.025	0.347	0.179	0.053	0.190
BRCA2	148.762	-1.335	0.377	0.000	0.003	148.762	-0.829	0.372	0.026	0.316	148.762	0.506	0.436	0.245	0.476
SS18	937.785	-0.255	0.093	0.006	0.023	937.785	-0.119	0.092	0.194	0.478	937.785	0.135	0.107	0.208	0.432
DENND2B	693.797	0.556	0.174	0.001	0.007	693.797	-0.039	0.174	0.821	0.906	693.797	-0.596	0.201	0.003	0.029
ST13	6609.539	-0.212	0.154	0.169	0.289	6609.539	0.062	0.154	0.689	0.832	6609.539	0.273	0.178	0.124	0.317
ST14	159.152	0.532	0.231	0.021	0.060	159.152	0.186	0.232	0.421	0.658	159.152	-0.346	0.267	0.195	0.417
STAC	576.173	-0.567	0.236	0.016	0.049	576.173	0.220	0.234	0.348	0.603	576.173	0.787	0.272	0.004	0.034
ZFP36L1	6220.184	0.328	0.154	0.033	0.084	6220.184	-0.038	0.154	0.805	0.898	6220.184	-0.366	0.178	0.039	0.157
STAR	46.372	-0.229	0.577	0.691	0.787	46.372	0.493	0.570	0.387	0.633	46.372	0.722	0.663	0.276	0.511
STAT1	1575.559	-0.016	0.202	0.935	0.959	1575.559	-0.258	0.202	0.202	0.485	1575.559	-0.241	0.233	0.301	0.536
STAT2	599.631	1.096	0.397	0.006	0.022	599.631	0.618	0.398	0.120	0.412	599.631	-0.478	0.459	0.297	0.533
STAT3	4449.727	0.297	0.127	0.019	0.055	4449.727	0.091	0.127	0.470	0.692	4449.727	-0.205	0.146	0.160	0.373
STAT4	10.673	1.141	0.663	0.086	0.174	10.673	0.436	0.680	0.522	0.725	10.673	-0.705	0.765	0.357	0.590
STAT5A	62.650	0.638	0.462	0.167	0.287	62.650	0.093	0.464	0.842	0.918	62.650	-0.545	0.534	0.307	0.543
STAT5B	776.753	-0.234	0.176	0.184	0.307	776.753	0.190	0.175	0.277	0.548	776.753	0.424	0.203	0.037	0.150
SCARNA2	17.695	1.658	0.577	0.004	0.017	17.695	0.456	0.617	0.460	0.687	17.695	-1.202	0.669	0.072	0.229
STAT6	2537.128	0.134	0.124	0.281	0.418	2537.128	0.091	0.124	0.464	0.689	2537.128	-0.043	0.144	0.763	0.880
SNORD1C	8.453	-0.706	0.638	0.268	0.405	8.453	0.536	0.552	0.331	0.591	8.453	1.242	0.705	0.078	0.241
ZFP36L2	2108.640	-0.403	0.264	0.127	0.233	2108.640	0.532	0.263	0.043	0.331	2108.640	0.935	0.305	0.002	0.023
STAU1	2341.357	-0.321	0.156	0.039	0.096	2341.357	-0.045	0.155	0.770	0.878	2341.357	0.276	0.180	0.125	0.319
STC1	1775.554	1.607	0.842	0.056	0.126	1775.554	0.896	0.843	0.287	0.556	1775.554	-0.711	0.973	0.465	0.681
HSPA13	1371.536	-1.264	0.256	0.000	0.000	1371.536	-0.382	0.255	0.134	0.425	1371.536	0.882	0.295	0.003	0.028
SULT1E1	98.921	1.192	0.261	0.000	0.000	98.921	0.283	0.267	0.288	0.557	98.921	-0.909	0.303	0.003	0.027
ELOVL4	38.806	0.463	0.542	0.393	0.532	38.806	-0.800	0.558	0.152	0.444	38.806	-1.263	0.639	0.048	0.178
STIM1	840.531	-0.004	0.171	0.983	0.990	840.531	0.245	0.170	0.149	0.442	840.531	0.249	0.197	0.206	0.430
CD27-AS1	29.418	1.125	0.486	0.021	0.059	29.418	0.534	0.493	0.278	0.548	29.418	-0.591	0.559	0.291	0.526
NEK4	356.715	-0.484	0.252	0.055	0.124	356.715	-0.469	0.251	0.062	0.350	356.715	0.015	0.292	0.960	0.981
STK3	348.260	-0.338	0.196	0.085	0.173	348.260	-0.217	0.195	0.265	0.537	348.260	0.121	0.227	0.594	0.774
STK4	726.931	-0.577	0.194	0.003	0.013	726.931	-0.353	0.194	0.068	0.357	726.931	0.224	0.225	0.318	0.553
AURKA	1104.446	-0.661	0.215	0.002	0.010	1104.446	-0.353	0.215	0.100	0.394	1104.446	0.309	0.248	0.214	0.440
CDKL5	268.351	-0.841	0.223	0.000	0.001	268.351	0.120	0.217	0.580	0.764	268.351	0.961	0.256	0.000	0.004
STK10	645.608	-0.316	0.283	0.264	0.401	645.608	-0.673	0.284	0.018	0.306	645.608	-0.357	0.328	0.277	0.511
STK11	1993.235	0.508	0.152	0.001	0.005	1993.235	0.119	0.152	0.434	0.667	1993.235	-0.389	0.175	0.026	0.121
STRN	530.098	-0.625	0.259	0.016	0.049	530.098	-0.105	0.258	0.683	0.829	530.098	0.519	0.299	0.083	0.250
STX1A	133.955	-0.227	0.253	0.371	0.511	133.955	-0.214	0.252	0.394	0.639	133.955	0.013	0.294	0.966	0.984
STX3	611.605	1.030	0.181	0.000	0.000	611.605	-0.272	0.184	0.140	0.431	611.605	-1.302	0.210	0.000	0.000
STX4	955.468	0.882	0.148	0.000	0.000	955.468	0.267	0.149	0.074	0.363	955.468	-0.615	0.172	0.000	0.006
STX5	564.423	0.075	0.161	0.641	0.748	564.423	-0.123	0.161	0.447	0.677	564.423	-0.198	0.187	0.289	0.525
STXBP1	533.117	1.248	0.406	0.002	0.010	533.117	0.419	0.407	0.303	0.569	533.117	-0.829	0.469	0.077	0.239
STXBP2	475.007	0.530	0.266	0.046	0.108	475.007	0.638	0.265	0.016	0.306	475.007	0.107	0.306	0.726	0.858
STXBP3	633.796	0.355	0.114	0.002	0.009	633.796	-0.126	0.115	0.275	0.545	633.796	-0.481	0.133	0.000	0.005
STYX	423.436	-0.640	0.159	0.000	0.001	423.436	-0.355	0.157	0.024	0.316	423.436	0.286	0.184	0.120	0.311
SULT1A3	26.842	-0.066	0.558	0.906	0.940	26.842	-0.035	0.555	0.949	0.975	26.842	0.031	0.646	0.962	0.982

BSG	21952.165	0.976	0.228	0.000	0.000	21952.165	0.244	0.228	0.285	0.554	21952.165	-0.733	0.263	0.005	0.043
SUOX	116.281	0.500	0.255	0.050	0.116	116.281	0.224	0.256	0.381	0.628	116.281	-0.276	0.295	0.349	0.582
SUPT4H1	442.520	0.056	0.125	0.654	0.758	442.520	-0.169	0.126	0.178	0.467	442.520	-0.225	0.146	0.122	0.314
SUPT5H	3350.653	-0.044	0.108	0.687	0.784	3350.653	0.113	0.108	0.295	0.563	3350.653	0.156	0.125	0.210	0.435
SUPT6H	2460.384	-0.453	0.118	0.000	0.001	2460.384	-0.170	0.118	0.149	0.441	2460.384	0.283	0.137	0.039	0.155
SUPV3L1	556.873	-0.975	0.262	0.000	0.002	556.873	-0.526	0.261	0.044	0.331	556.873	0.449	0.303	0.139	0.342
SURF1	543.325	0.381	0.200	0.058	0.128	543.325	0.085	0.201	0.674	0.824	543.325	-0.296	0.232	0.201	0.425
SURF2	341.681	0.003	0.369	0.993	0.996	341.681	-0.540	0.370	0.144	0.435	341.681	-0.543	0.427	0.203	0.427
SURF4	4643.760	-0.242	0.194	0.212	0.342	4643.760	-0.202	0.194	0.296	0.563	4643.760	0.039	0.224	0.861	0.933
MED22	468.443	-0.949	0.260	0.000	0.002	468.443	-0.530	0.258	0.040	0.327	468.443	0.420	0.300	0.162	0.375
SURF6	999.404	-0.260	0.258	0.314	0.454	999.404	-0.339	0.258	0.188	0.474	999.404	-0.080	0.298	0.788	0.895
SUV39H1	249.542	-0.527	0.262	0.045	0.106	249.542	-0.400	0.261	0.126	0.418	249.542	0.127	0.304	0.676	0.828
BST2	4007.981	0.314	0.178	0.077	0.161	4007.981	0.336	0.178	0.059	0.349	4007.981	0.022	0.205	0.916	0.961
SVIL	1550.363	-0.650	0.217	0.003	0.012	1550.363	0.030	0.216	0.888	0.944	1550.363	0.681	0.250	0.006	0.049
VAMP1	68.689	0.707	0.398	0.076	0.159	68.689	0.432	0.399	0.279	0.549	68.689	-0.275	0.458	0.549	0.744
VAMP2	317.205	0.426	0.368	0.247	0.381	317.205	0.550	0.367	0.134	0.426	317.205	0.124	0.424	0.771	0.883
VAMP7	1404.793	-0.461	0.152	0.002	0.011	1404.793	-0.185	0.151	0.221	0.500	1404.793	0.276	0.175	0.115	0.303
BTC	11.622	1.207	0.509	0.018	0.052	11.622	1.157	0.505	0.022	0.314	11.622	-0.050	0.564	0.930	0.968
SYK	26.673	0.442	0.333	0.184	0.308	26.673	0.478	0.327	0.144	0.435	26.673	0.036	0.378	0.923	0.964
SYP	8.035	1.259	0.635	0.048	0.111	8.035	0.045	0.676	0.948	0.974	8.035	-1.214	0.750	0.105	0.289
SYPL1	1502.270	-0.168	0.174	0.333	0.473	1502.270	0.099	0.174	0.571	0.757	1502.270	0.267	0.201	0.184	0.404
SYT1	13.154	-0.413	0.423	0.329	0.469	13.154	-0.575	0.422	0.173	0.463	13.154	-0.162	0.503	0.747	0.870
BTD	443.737	-0.442	0.202	0.029	0.076	443.737	0.242	0.199	0.225	0.504	443.737	0.684	0.232	0.003	0.030
TACC1	652.593	-0.556	0.314	0.077	0.160	652.593	-0.747	0.315	0.018	0.306	652.593	-0.190	0.364	0.601	0.778
ADAM17	894.089	-0.151	0.143	0.290	0.428	894.089	0.046	0.142	0.745	0.864	894.089	0.197	0.165	0.232	0.461
KLF9	4162.037	-0.930	0.228	0.000	0.001	4162.037	0.105	0.228	0.644	0.806	4162.037	1.036	0.264	0.000	0.002
TADA2A	467.234	-0.531	0.148	0.000	0.003	467.234	-0.188	0.146	0.200	0.483	467.234	0.343	0.171	0.045	0.171
TAF1	635.422	-0.679	0.215	0.002	0.008	635.422	-0.004	0.213	0.987	0.992	635.422	0.676	0.248	0.006	0.048
TAF2	501.277	-0.853	0.292	0.003	0.015	501.277	-0.466	0.291	0.109	0.401	501.277	0.387	0.337	0.251	0.484
TAF4	474.724	-0.388	0.187	0.038	0.093	474.724	-0.131	0.185	0.478	0.698	474.724	0.256	0.216	0.235	0.464
TAF4B	442.059	-0.586	0.163	0.000	0.002	442.059	-0.205	0.161	0.202	0.486	442.059	0.381	0.188	0.043	0.167
TAGLN	147.308	1.159	0.474	0.014	0.045	147.308	-0.746	0.480	0.120	0.412	147.308	-1.905	0.551	0.001	0.009
TAF5	344.345	-0.117	0.238	0.624	0.734	344.345	-0.205	0.238	0.388	0.634	344.345	-0.088	0.276	0.748	0.871
TAF6	1003.855	0.161	0.158	0.308	0.447	1003.855	-0.192	0.158	0.226	0.505	1003.855	-0.353	0.183	0.054	0.191
TAF7	899.840	-0.273	0.109	0.012	0.039	899.840	0.018	0.107	0.866	0.931	899.840	0.291	0.125	0.020	0.101
KLF5	1463.284	-0.726	0.215	0.001	0.005	1463.284	-0.499	0.215	0.020	0.311	1463.284	0.227	0.249	0.362	0.594
TAF9	3714.485	-0.079	0.175	0.650	0.756	3714.485	-0.331	0.175	0.058	0.348	3714.485	-0.252	0.202	0.212	0.438
TAF10	2854.320	0.205	0.109	0.060	0.133	2854.320	-0.055	0.109	0.614	0.787	2854.320	-0.260	0.126	0.039	0.157
TAF11	412.050	0.122	0.195	0.532	0.658	412.050	-0.160	0.196	0.415	0.653	412.050	-0.282	0.226	0.213	0.439
TAF12	453.874	0.772	0.217	0.000	0.003	453.874	0.112	0.218	0.609	0.784	453.874	-0.660	0.251	0.009	0.059
TAF13	427.109	-0.190	0.359	0.597	0.711	427.109	-0.842	0.360	0.019	0.306	427.109	-0.652	0.416	0.117	0.306
MAP3K7	1077.927	-0.701	0.198	0.000	0.003	1077.927	-0.355	0.197	0.072	0.361	1077.927	0.345	0.229	0.131	0.329
TALDO1	4006.832	0.369	0.218	0.090	0.181	4006.832	-0.153	0.218	0.483	0.701	4006.832	-0.522	0.252	0.038	0.153
BTF3	18414.890	0.278	0.179	0.120	0.224	18414.890	0.199	0.179	0.265	0.537	18414.890	-0.079	0.206	0.703	0.846
TAP1	11525.709	0.368	0.176	0.036	0.090	11525.709	0.307	0.176	0.081	0.372	11525.709	-0.061	0.203	0.762	0.879
TAP2	6573.223	-0.852	0.236	0.000	0.002	6573.223	-0.410	0.235	0.082	0.373	6573.223	0.442	0.272	0.104	0.287
TAPBP	13431.850	1.315	0.207	0.000	0.000	13431.850	0.523	0.207	0.011	0.305	13431.850	-0.793	0.239	0.001	0.012
TARBP1	430.754	0.057	0.184	0.757	0.839	430.754	-0.081	0.184	0.659	0.814	430.754	-0.138	0.213	0.517	0.720
TARBP2	369.048	-0.062	0.189	0.744	0.828	369.048	-0.101	0.188	0.591	0.772	369.048	-0.039	0.219	0.857	0.931
TARS1	8391.126	-0.890	0.239	0.000	0.002	8391.126	-0.478	0.239	0.045	0.334	8391.126	0.412	0.276	0.135	0.336
TAZ	362.025	0.616	0.192	0.001	0.007	362.025	0.143	0.193	0.459	0.685	362.025	-0.473	0.222	0.034	0.142
TBCA	2853.752	0.578	0.198	0.003	0.015	2853.752	0.028	0.198	0.889	0.945	2853.752	-0.550	0.228	0.016	0.088
TBCC	266.025	0.323	0.175	0.065	0.141	266.025	-0.066	0.176	0.710	0.844	266.025	-0.389	0.203	0.056	0.196
TBCD	1858.887	0.073	0.161	0.652	0.756	1858.887	-0.211	0.162	0.192	0.477	1858.887	-0.284	0.187	0.128	0.324
TBCE	1262.432	-0.260	0.125	0.037	0.092	1262.432	-0.173	0.124	0.164	0.455	1262.432	0.087	0.144	0.545	0.741
TBL1X	1506.640	-0.635	0.145	0.000	0.000	1506.640	-0.071	0.144	0.621	0.791	1506.640	0.564	0.168	0.001	0.011
TBP	248.046	-0.505	0.234	0.031	0.080	248.046	-0.368	0.232	0.114	0.405	248.046	0.138	0.271	0.612	0.786
TBX2	112.729	1.116	0.283	0.000	0.001	112.729	-0.550	0.295	0.063	0.350	112.729	-1.665	0.335	0.000	0.000
TBX5	92.808	-0.005	0.265	0.984	0.991	92.808	0.295	0.259	0.255	0.528	92.808	0.301	0.303	0.322	0.556
TBX6	34.522	0.818	0.501	0.102	0.199	34.522	0.740	0.500	0.139	0.430	34.522	-0.078	0.574	0.892	0.948
TBXAS1	121.425	-0.546	0.236	0.021	0.059	121.425	-0.049	0.229	0.830	0.912	121.425	0.497	0.272	0.067	0.219
TCEA1	3204.542	-0.701	0.130	0.000	0.000	3204.542	-0.068	0.130	0.599	0.777	3204.542	0.633	0.150	0.000	0.001
TCEA2	640.380	0.922	0.299	0.002	0.010	640.380	0.617	0.300	0.040	0.327	640.380	-0.305	0.345	0.377	0.609
TCEA3	9.724	0.524	0.575	0.362	0.503	9.724	-0.199	0.600	0.741	0.862	9.724	-0.723	0.682	0.289	0.525
SNORD17	18.360	-2.169	0.703	0.002	0.010	18.360	-0.867	0.652	0.184	0.471	18.360	1.302	0.806	0.106	0.291
MSMP	222.591	-0.422	0.196	0.031	0.081	222.591	0.101	0.192	0.598	0.776	222.591	0.524	0.225	0.020	0.102
FAM86DP	236.899	-0.413	0.227	0.068	0.146	236.899	-0.054	0.224	0.810	0.900	236.899	0.360	0.262	0.169	0.385

ELOC	1202.854	0.124	0.223	0.577	0.695	1202.854	-0.285	0.223	0.202	0.485	1202.854	-0.409	0.258	0.113	0.300
SNORA57	23.054	1.563	0.510	0.002	0.010	23.054	0.444	0.526	0.399	0.643	23.054	-1.119	0.590	0.058	0.200
SNORD89	12.158	1.081	0.467	0.021	0.059	12.158	0.258	0.492	0.600	0.778	12.158	-0.823	0.545	0.131	0.328
SNORD104	29.338	1.272	0.356	0.000	0.003	29.338	0.152	0.376	0.687	0.831	29.338	-1.120	0.418	0.007	0.053
LMNTD2-AS1	30.895	1.002	0.695	0.149	0.263	30.895	1.234	0.692	0.074	0.364	30.895	0.232	0.795	0.770	0.883
ELOB	2633.839	0.825	0.246	0.001	0.005	2633.839	0.310	0.246	0.208	0.490	2633.839	-0.515	0.284	0.070	0.225
PPAN-P2RY11	20.137	-1.350	0.559	0.016	0.048	20.137	-0.879	0.536	0.101	0.394	20.137	0.470	0.651	0.470	0.684
ELOA	1514.922	-0.841	0.241	0.000	0.003	1514.922	-0.576	0.241	0.017	0.306	1514.922	0.266	0.278	0.340	0.575
TCF4	190.572	0.045	0.268	0.867	0.914	190.572	-0.093	0.268	0.728	0.855	190.572	-0.138	0.310	0.656	0.816
TBX3	506.967	-0.089	0.210	0.671	0.771	506.967	-0.030	0.209	0.884	0.942	506.967	0.059	0.243	0.809	0.904
HNF1A	28.634	0.713	0.390	0.068	0.145	28.634	0.578	0.389	0.138	0.429	28.634	-0.136	0.445	0.761	0.878
HNF1B	10.855	0.367	0.583	0.529	0.655	10.855	-0.009	0.592	0.988	0.993	10.855	-0.376	0.681	0.581	0.764
TCF3	2960.039	-0.103	0.105	0.325	0.466	2960.039	-0.060	0.105	0.565	0.753	2960.039	0.043	0.121	0.723	0.856
TCF7	22.801	0.646	0.391	0.099	0.194	22.801	-0.605	0.420	0.150	0.442	22.801	-1.252	0.474	0.008	0.058
TCF7L2	309.205	-0.401	0.135	0.003	0.013	309.205	0.020	0.131	0.877	0.937	309.205	0.421	0.155	0.007	0.050
ZEB1	797.814	-0.942	0.246	0.000	0.001	797.814	-0.344	0.245	0.159	0.451	797.814	0.598	0.284	0.035	0.146
GCFC2	330.388	-0.474	0.205	0.021	0.059	330.388	-0.381	0.204	0.062	0.350	330.388	0.093	0.237	0.695	0.841
TCF12	1152.350	-1.121	0.196	0.000	0.000	1152.350	-0.078	0.195	0.687	0.831	1152.350	1.042	0.226	0.000	0.000
BTG1	4567.925	0.845	0.305	0.006	0.021	4567.925	0.450	0.305	0.140	0.431	4567.925	-0.394	0.352	0.262	0.496
ZNF354A	118.253	0.189	0.239	0.430	0.567	118.253	-0.071	0.240	0.768	0.877	118.253	-0.259	0.278	0.350	0.583
TCF19	1618.116	-0.346	0.380	0.362	0.503	1618.116	-1.031	0.380	0.007	0.297	1618.116	-0.685	0.439	0.119	0.310
TCF20	3516.369	-0.575	0.186	0.002	0.010	3516.369	-0.406	0.186	0.029	0.322	3516.369	0.169	0.215	0.432	0.654
TCF21	7.859	-0.293	0.629	0.642	0.748	7.859	-0.325	0.621	0.601	0.779	7.859	-0.032	0.735	0.965	0.984
VPS72	908.079	0.037	0.223	0.870	0.916	908.079	-0.228	0.223	0.306	0.572	908.079	-0.264	0.257	0.304	0.539
MLX	1212.731	-0.120	0.145	0.407	0.545	1212.731	-0.168	0.145	0.246	0.521	1212.731	-0.048	0.167	0.776	0.886
TCN2	82.946	1.800	0.303	0.000	0.000	82.946	0.810	0.312	0.010	0.298	82.946	-0.990	0.345	0.004	0.036
TCOF1	2584.292	-0.820	0.397	0.039	0.095	2584.292	-1.073	0.397	0.007	0.297	2584.292	-0.253	0.458	0.581	0.764
TCP1	6319.617	-0.557	0.181	0.002	0.010	6319.617	-0.336	0.181	0.063	0.350	6319.617	0.220	0.209	0.292	0.528
TCTA	365.084	-0.262	0.141	0.063	0.137	365.084	0.136	0.138	0.323	0.584	365.084	0.398	0.162	0.014	0.081
BUB1	1174.065	-0.784	0.173	0.000	0.000	1174.065	-0.272	0.172	0.113	0.405	1174.065	0.511	0.199	0.010	0.067
DYNLT3	443.438	0.352	0.125	0.005	0.020	443.438	-0.105	0.127	0.409	0.649	443.438	-0.456	0.146	0.002	0.020
TCTE3	25.311	0.826	0.451	0.067	0.144	25.311	0.045	0.462	0.923	0.961	25.311	-0.782	0.525	0.137	0.339
PPP1R11	3689.046	0.182	0.168	0.279	0.416	3689.046	0.090	0.168	0.592	0.773	3689.046	-0.092	0.194	0.636	0.802
DYNLT1	1784.475	0.543	0.166	0.001	0.006	1784.475	-0.107	0.166	0.521	0.725	1784.475	-0.650	0.192	0.001	0.010
TDG	482.193	-0.348	0.297	0.241	0.374	482.193	-0.944	0.298	0.002	0.297	482.193	-0.597	0.345	0.083	0.251
TDO2	45.918	1.075	0.432	0.013	0.041	45.918	0.919	0.432	0.034	0.322	45.918	-0.156	0.494	0.752	0.873
PRDX2	5691.223	0.458	0.245	0.062	0.136	5691.223	0.413	0.245	0.092	0.386	5691.223	-0.044	0.283	0.875	0.940
TEAD1	2585.372	-0.605	0.268	0.024	0.067	2585.372	-0.061	0.268	0.820	0.906	2585.372	0.544	0.310	0.079	0.243
TEAD4	658.215	-0.588	0.292	0.044	0.105	658.215	-0.472	0.292	0.106	0.398	658.215	0.117	0.338	0.730	0.860
TEAD3	552.252	0.546	0.181	0.003	0.012	552.252	-0.235	0.183	0.198	0.481	552.252	-0.781	0.210	0.000	0.004
TEC	84.250	0.017	0.321	0.959	0.975	84.250	0.129	0.318	0.685	0.830	84.250	0.113	0.370	0.761	0.879
TEF	218.505	0.018	0.443	0.968	0.980	218.505	0.756	0.440	0.086	0.379	218.505	0.738	0.510	0.148	0.355
TMBIM6	12726.413	-0.068	0.079	0.388	0.527	12726.413	0.001	0.079	0.993	0.996	12726.413	0.069	0.091	0.450	0.669
BUB1B	943.261	-0.776	0.271	0.004	0.017	943.261	-0.351	0.271	0.194	0.478	943.261	0.425	0.313	0.175	0.393
TEP1	263.498	0.330	0.190	0.083	0.170	263.498	0.024	0.191	0.898	0.949	263.498	-0.305	0.220	0.165	0.380
TERF1	368.446	-0.280	0.143	0.051	0.117	368.446	-0.323	0.143	0.024	0.316	368.446	-0.042	0.167	0.799	0.899
TERF2	511.560	-0.645	0.176	0.000	0.002	511.560	-0.217	0.174	0.213	0.493	511.560	0.428	0.203	0.035	0.146
TERT	76.002	-1.231	0.316	0.000	0.001	76.002	-0.431	0.303	0.155	0.447	76.002	0.800	0.364	0.028	0.126
TESK1	356.553	0.826	0.203	0.000	0.001	356.553	0.099	0.205	0.630	0.797	356.553	-0.727	0.235	0.002	0.022
TFAM	1011.338	-1.112	0.245	0.000	0.000	1011.338	-0.583	0.245	0.017	0.306	1011.338	0.529	0.284	0.062	0.209
TFAP2A	697.856	0.169	0.260	0.516	0.644	697.856	0.350	0.259	0.177	0.466	697.856	0.182	0.300	0.544	0.740
TFAP2C	112.698	-0.081	0.219	0.710	0.801	112.698	0.406	0.212	0.055	0.343	112.698	0.488	0.249	0.050	0.184
TFAP4	860.947	-0.189	0.138	0.172	0.292	860.947	0.227	0.137	0.099	0.393	860.947	0.416	0.159	0.009	0.061
TFCP2	597.446	-0.498	0.190	0.009	0.031	597.446	-0.377	0.190	0.047	0.335	597.446	0.121	0.220	0.583	0.766
NR2F1	23.730	1.728	0.319	0.000	0.000	23.730	0.432	0.348	0.215	0.494	23.730	-1.296	0.371	0.000	0.008
NR2F2	531.321	-0.104	0.144	0.470	0.604	531.321	0.115	0.143	0.422	0.659	531.321	0.219	0.166	0.188	0.408
TFDP1	2141.122	-0.485	0.196	0.013	0.042	2141.122	-0.528	0.196	0.007	0.297	2141.122	-0.043	0.226	0.848	0.926
TFDP2	1035.614	0.096	0.288	0.739	0.823	1035.614	0.357	0.288	0.215	0.494	1035.614	0.261	0.332	0.433	0.655
TFE3	2049.419	-0.199	0.133	0.134	0.244	2049.419	-0.167	0.132	0.208	0.490	2049.419	0.032	0.153	0.835	0.920
TFF3	12.584	0.732	0.459	0.111	0.211	12.584	0.205	0.474	0.665	0.818	12.584	-0.527	0.533	0.323	0.558
TFPI	1267.101	0.717	0.348	0.039	0.096	1267.101	0.350	0.348	0.315	0.577	1267.101	-0.367	0.402	0.361	0.593
TFRC	7585.877	-0.840	0.329	0.011	0.036	7585.877	-0.508	0.329	0.122	0.413	7585.877	0.332	0.379	0.382	0.611
TG	70.118	0.891	0.721	0.216	0.346	70.118	0.871	0.720	0.227	0.505	70.118	-0.020	0.830	0.981	0.990
TGFA	429.193	-0.329	0.157	0.036	0.090	429.193	-0.050	0.155	0.745	0.864	429.193	0.279	0.181	0.123	0.316
TGFB1	6868.029	0.744	0.179	0.000	0.000	6868.029	0.326	0.179	0.068	0.357	6868.029	-0.418	0.206	0.043	0.167
TGFB1I1	675.008	0.664	0.287	0.021	0.060	675.008	0.518	0.287	0.072	0.361	675.008	-0.146	0.332	0.660	0.818
TGFB2	18.524	0.065	0.493	0.896	0.932	18.524	-0.200	0.495	0.686	0.830	18.524	-0.265	0.575	0.645	0.808

TGFB3	38.525	0.099	0.392	0.800	0.868	38.525	0.777	0.380	0.041	0.329	38.525	0.677	0.445	0.128	0.324
TGFBI	44044.694	-0.358	0.354	0.313	0.452	44044.694	0.443	0.354	0.211	0.492	44044.694	0.801	0.409	0.050	0.183
TGFBR1	773.683	0.839	0.269	0.002	0.009	773.683	0.222	0.269	0.410	0.649	773.683	-0.617	0.310	0.047	0.175
TGFBR2	2446.898	-1.199	0.263	0.000	0.000	2446.898	-0.274	0.262	0.296	0.564	2446.898	0.925	0.303	0.002	0.024
TGFBR3	999.460	-0.639	0.502	0.204	0.331	999.460	0.446	0.502	0.374	0.622	999.460	1.085	0.580	0.061	0.208
BYSL	753.889	-0.622	0.256	0.015	0.047	753.889	-0.464	0.256	0.070	0.358	753.889	0.158	0.296	0.595	0.774
TGIF1	739.771	0.138	0.175	0.430	0.566	739.771	-0.048	0.175	0.782	0.885	739.771	-0.186	0.202	0.357	0.590
TGM1	11.604	1.269	0.571	0.026	0.071	11.604	-0.118	0.615	0.848	0.922	11.604	-1.387	0.681	0.042	0.163
TGM2	1252.260	2.868	0.237	0.000	0.000	1252.260	0.584	0.238	0.014	0.306	1252.260	-2.284	0.273	0.000	0.000
THBD	587.471	-0.932	0.231	0.000	0.001	587.471	-0.256	0.230	0.265	0.537	587.471	0.675	0.267	0.011	0.071
THBS1	1082.102	-1.125	0.318	0.000	0.003	1082.102	-0.619	0.317	0.051	0.339	1082.102	0.507	0.367	0.168	0.383
THBS2	15.990	0.214	0.451	0.635	0.743	15.990	0.296	0.443	0.504	0.715	15.990	0.082	0.516	0.873	0.939
THBS3	185.924	0.411	0.555	0.459	0.593	185.924	0.666	0.555	0.230	0.507	185.924	0.255	0.640	0.691	0.837
TSPO	3963.230	0.623	0.199	0.002	0.009	3963.230	0.257	0.199	0.197	0.481	3963.230	-0.366	0.230	0.111	0.298
THBS4	10.764	1.660	0.700	0.018	0.053	10.764	0.922	0.717	0.198	0.482	10.764	-0.738	0.794	0.352	0.586
THOP1	2161.129	-0.115	0.167	0.492	0.624	2161.129	-0.106	0.167	0.526	0.729	2161.129	0.009	0.193	0.963	0.982
THRA	2022.217	0.038	0.366	0.917	0.948	2022.217	0.557	0.366	0.128	0.420	2022.217	0.519	0.423	0.220	0.446
KLF10	2045.543	-0.616	0.164	0.000	0.001	2045.543	-0.076	0.164	0.643	0.805	2045.543	0.540	0.190	0.004	0.038
TIA1	491.092	-0.320	0.175	0.067	0.144	491.092	-0.283	0.174	0.104	0.396	491.092	0.037	0.202	0.856	0.931
TIAL1	1465.036	-0.464	0.174	0.008	0.028	1465.036	-0.143	0.173	0.410	0.649	1465.036	0.321	0.201	0.110	0.296
TIAM1	8.513	0.642	0.539	0.234	0.366	8.513	0.419	0.541	0.439	0.671	8.513	-0.223	0.616	0.717	0.853
TIMP1	1831.072	1.126	0.242	0.000	0.000	1831.072	0.214	0.242	0.378	0.625	1831.072	-0.912	0.280	0.001	0.014
TIMP2	3939.869	0.962	0.254	0.000	0.001	3939.869	0.350	0.254	0.169	0.459	3939.869	-0.612	0.294	0.037	0.151
TIMP3	940.261	-0.781	0.487	0.109	0.208	940.261	0.687	0.486	0.157	0.450	940.261	1.469	0.562	0.009	0.061
C1QBP	2158.286	-0.231	0.264	0.381	0.521	2158.286	-0.467	0.264	0.077	0.368	2158.286	-0.236	0.305	0.439	0.659
TJP1	1439.496	-0.901	0.255	0.000	0.003	1439.496	-0.606	0.254	0.017	0.306	1439.496	0.295	0.294	0.317	0.552
TK1	1694.927	0.214	0.257	0.406	0.544	1694.927	-0.136	0.257	0.597	0.776	1694.927	-0.349	0.297	0.239	0.469
TK2	758.822	-0.067	0.286	0.814	0.878	758.822	0.273	0.286	0.339	0.597	758.822	0.341	0.330	0.302	0.537
TKT	48468.292	0.436	0.324	0.178	0.300	48468.292	0.382	0.324	0.237	0.514	48468.292	-0.053	0.374	0.887	0.946
ICAM5	295.684	0.073	0.593	0.902	0.937	295.684	1.058	0.591	0.073	0.362	295.684	0.985	0.683	0.149	0.358
TLE1	1071.843	-0.383	0.129	0.003	0.014	1071.843	-0.047	0.128	0.715	0.847	1071.843	0.336	0.149	0.024	0.115
TLE2	621.950	0.217	0.455	0.634	0.742	621.950	0.650	0.455	0.153	0.445	621.950	0.433	0.525	0.410	0.636
TLE3	793.518	-0.110	0.183	0.547	0.671	793.518	-0.216	0.182	0.237	0.514	793.518	-0.106	0.211	0.615	0.789
TLE4	35.585	-0.001	0.315	0.996	0.998	35.585	0.415	0.302	0.170	0.459	35.585	0.416	0.358	0.244	0.475
TLL1	314.423	-0.598	0.239	0.012	0.040	314.423	-0.366	0.238	0.124	0.416	314.423	0.232	0.277	0.402	0.629
TLN1	8620.778	-0.104	0.112	0.353	0.493	8620.778	0.123	0.112	0.270	0.542	8620.778	0.227	0.129	0.079	0.242
SEC62	2781.218	0.245	0.156	0.116	0.219	2781.218	-0.203	0.156	0.193	0.478	2781.218	-0.448	0.180	0.013	0.078
TLR2	1073.764	0.383	0.268	0.153	0.268	1073.764	-0.220	0.269	0.414	0.652	1073.764	-0.603	0.310	0.052	0.187
TLR3	181.457	0.495	0.267	0.064	0.140	181.457	0.360	0.267	0.177	0.466	181.457	-0.134	0.308	0.663	0.820
TLR4	442.769	-0.151	0.328	0.646	0.752	442.769	-0.675	0.329	0.040	0.327	442.769	-0.524	0.380	0.167	0.382
ACTG1	51145.393	0.339	0.144	0.019	0.055	51145.393	0.158	0.144	0.273	0.544	51145.393	-0.181	0.166	0.277	0.511
SERPING1	22.544	2.348	1.554	0.131	0.239	22.544	0.180	1.566	0.908	0.954	22.544	-2.168	1.798	0.228	0.457
TSPAN7	267.950	0.116	0.227	0.608	0.720	267.950	-0.289	0.228	0.205	0.488	267.950	-0.405	0.263	0.124	0.317
TSPAN8	63.681	0.344	0.299	0.251	0.385	63.681	0.512	0.295	0.082	0.374	63.681	0.169	0.342	0.622	0.793
TSPAN6	116.978	-0.172	0.189	0.362	0.503	116.978	0.052	0.184	0.777	0.881	116.978	0.224	0.217	0.303	0.537
TSPAN4	2072.256	0.335	0.182	0.066	0.143	2072.256	0.057	0.182	0.753	0.868	2072.256	-0.278	0.210	0.187	0.407
GPR137B	173.887	1.197	0.206	0.000	0.000	173.887	-0.143	0.218	0.513	0.720	173.887	-1.340	0.244	0.000	0.000
TM7SF2	139.146	1.607	0.477	0.001	0.005	139.146	0.648	0.479	0.177	0.465	139.146	-0.959	0.550	0.081	0.247
TRAPPC10	526.470	-0.645	0.274	0.019	0.055	526.470	-0.750	0.274	0.006	0.297	526.470	-0.105	0.318	0.740	0.867
TMF1	1083.857	-0.396	0.249	0.112	0.213	1083.857	0.036	0.249	0.885	0.943	1083.857	0.432	0.288	0.133	0.333
TMOD1	305.118	-0.121	0.192	0.528	0.655	305.118	-0.114	0.191	0.551	0.744	305.118	0.007	0.223	0.974	0.987
TMPO	2492.943	-0.983	0.262	0.000	0.001	2492.943	-0.652	0.261	0.013	0.305	2492.943	0.331	0.302	0.273	0.508
TMPRSS2	12.765	0.683	0.454	0.133	0.241	12.765	0.491	0.455	0.281	0.550	12.765	-0.192	0.517	0.710	0.849
TMSB4X	13676.195	1.550	0.243	0.000	0.000	13676.195	-0.152	0.243	0.532	0.732	13676.195	-1.703	0.281	0.000	0.000
CLEC3B	7.596	0.176	1.231	0.887	0.927	7.596	1.076	1.210	0.374	0.622	7.596	0.900	1.406	0.522	0.724
TNFAIP1	885.007	0.044	0.137	0.746	0.830	885.007	-0.166	0.137	0.227	0.505	885.007	-0.210	0.159	0.185	0.405
TNFAIP2	3139.557	0.768	0.173	0.000	0.000	3139.557	0.377	0.173	0.029	0.322	3139.557	-0.391	0.200	0.050	0.182
TNFAIP3	2759.282	-0.118	0.288	0.681	0.779	2759.282	-0.384	0.288	0.182	0.469	2759.282	-0.266	0.332	0.424	0.648
TNFAIP6	131.402	-0.732	0.378	0.053	0.120	131.402	-1.000	0.379	0.008	0.297	131.402	-0.268	0.444	0.547	0.742
TNFRSF1A	5306.472	0.319	0.140	0.023	0.063	5306.472	0.125	0.140	0.370	0.619	5306.472	-0.193	0.162	0.232	0.460
TNFRSF1B	899.795	0.667	0.141	0.000	0.000	899.795	0.054	0.142	0.701	0.839	899.795	-0.612	0.163	0.000	0.004
TNNC1	32.821	0.497	0.322	0.123	0.228	32.821	0.152	0.326	0.642	0.805	32.821	-0.346	0.373	0.355	0.588
TNNI3	9.220	1.091	0.656	0.096	0.191	9.220	0.665	0.665	0.317	0.579	9.220	-0.425	0.747	0.569	0.758
TNNT1	1428.899	0.893	0.406	0.028	0.074	1428.899	0.806	0.406	0.047	0.336	1428.899	-0.087	0.468	0.853	0.930
TNNT3	5.627	-4.411	1.773	0.013	0.041	5.627	0.251	1.351	0.853	0.925	5.627	4.663	1.936	0.016	0.089
TNS1	277.958	-0.760	1.221	0.534	0.659	277.958	0.616	1.219	0.613	0.786	277.958	1.376	1.409	0.329	0.563
TNXA	45.218	1.466	1.388	0.291	0.429	45.218	1.977	1.386	0.154	0.446	45.218	0.511	1.597	0.749	0.871

TNXB	292.702	1.297	1.052	0.217	0.347	292.702	1.175	1.052	0.264	0.536	292.702	-0.123	1.214	0.920	0.963
C1R	1453.002	3.446	0.459	0.000	0.000	1453.002	1.488	0.460	0.001	0.297	1453.002	-1.958	0.529	0.000	0.004
TOP1	2837.454	-0.583	0.266	0.028	0.075	2837.454	-0.581	0.266	0.029	0.322	2837.454	0.003	0.307	0.993	0.996
TOP2A	9237.487	-0.243	0.315	0.439	0.575	9237.487	0.219	0.315	0.485	0.702	9237.487	0.463	0.363	0.202	0.426
TOP2B	3062.531	-0.701	0.182	0.000	0.001	3062.531	-0.308	0.182	0.090	0.384	3062.531	0.393	0.210	0.062	0.209
TOP3A	326.201	-0.852	0.338	0.012	0.038	326.201	-0.715	0.337	0.034	0.322	326.201	0.137	0.391	0.726	0.858
TP53	1204.210	0.098	0.215	0.650	0.755	1204.210	0.142	0.215	0.509	0.718	1204.210	0.044	0.249	0.859	0.932
TP53BP1	1038.381	-0.104	0.120	0.387	0.526	1038.381	-0.237	0.120	0.048	0.337	1038.381	-0.134	0.139	0.336	0.571
TP53BP2	429.816	-0.042	0.267	0.875	0.919	429.816	-0.120	0.267	0.655	0.811	429.816	-0.077	0.309	0.802	0.901
C1S	955.002	2.911	0.269	0.000	0.000	955.002	1.231	0.271	0.000	0.020	955.002	-1.680	0.309	0.000	0.000
TP73	671.654	0.069	0.482	0.887	0.927	671.654	0.701	0.482	0.146	0.438	671.654	0.632	0.556	0.256	0.490
TPBG	2359.632	-0.236	0.197	0.232	0.365	2359.632	0.216	0.197	0.273	0.544	2359.632	0.452	0.228	0.047	0.176
TPD52	1798.171	0.060	0.195	0.759	0.840	1798.171	0.193	0.195	0.322	0.583	1798.171	0.133	0.226	0.555	0.747
TPD52L1	124.893	0.540	0.241	0.025	0.069	124.893	0.644	0.239	0.007	0.297	124.893	0.104	0.276	0.705	0.847
TPD52L2	2275.021	-0.334	0.255	0.190	0.315	2275.021	-0.431	0.255	0.091	0.385	2275.021	-0.097	0.294	0.741	0.867
TPI1	17097.078	0.176	0.201	0.381	0.521	17097.078	0.364	0.201	0.071	0.359	17097.078	0.188	0.232	0.420	0.644
TPM1	3143.198	0.286	0.212	0.178	0.300	3143.198	-0.372	0.212	0.080	0.371	3143.198	-0.658	0.245	0.007	0.053
TPM2	7808.798	1.092	0.264	0.000	0.000	7808.798	0.251	0.264	0.343	0.600	7808.798	-0.841	0.305	0.006	0.046
C2	185.832	-0.297	0.433	0.492	0.625	185.832	0.561	0.430	0.192	0.477	185.832	0.858	0.498	0.085	0.254
TPM3	6878.989	-0.211	0.245	0.389	0.528	6878.989	-0.628	0.245	0.010	0.299	6878.989	-0.417	0.282	0.140	0.343
TPM4	12029.993	0.170	0.131	0.196	0.322	12029.993	-0.251	0.131	0.056	0.343	12029.993	-0.421	0.152	0.005	0.044
TPMT	326.280	0.551	0.180	0.002	0.011	326.280	-0.413	0.184	0.025	0.316	326.280	-0.964	0.211	0.000	0.000
TPP2	732.998	-0.491	0.210	0.019	0.056	732.998	-0.410	0.210	0.051	0.338	732.998	0.081	0.243	0.738	0.865
TPR	2676.907	-0.649	0.155	0.000	0.000	2676.907	-0.334	0.155	0.031	0.322	2676.907	0.315	0.179	0.079	0.242
TPSAB1	115.617	2.554	1.181	0.031	0.080	115.617	2.516	1.176	0.032	0.322	115.617	-0.038	1.247	0.976	0.987
TPT1	41064.794	0.657	0.285	0.021	0.060	41064.794	0.432	0.285	0.130	0.422	41064.794	-0.225	0.329	0.494	0.702
C3	68971.584	0.370	0.330	0.262	0.397	68971.584	0.634	0.330	0.055	0.342	68971.584	0.264	0.381	0.488	0.699
NR2C1	257.635	-0.177	0.138	0.198	0.324	257.635	-0.182	0.136	0.183	0.470	257.635	-0.004	0.160	0.978	0.988
NR2C2	926.766	-0.120	0.135	0.372	0.512	926.766	-0.303	0.135	0.025	0.316	926.766	-0.183	0.156	0.242	0.473
HSP90B1	17149.191	-0.207	0.193	0.283	0.421	17149.191	-0.149	0.193	0.441	0.672	17149.191	0.058	0.223	0.794	0.896
TRAF1	40.095	1.262	0.354	0.000	0.003	40.095	-0.107	0.390	0.784	0.887	40.095	-1.369	0.426	0.001	0.016
TRAF2	599.698	0.331	0.218	0.128	0.234	599.698	-0.323	0.219	0.139	0.430	599.698	-0.655	0.252	0.009	0.063
TRAF3	1776.432	-0.951	0.265	0.000	0.002	1776.432	-0.533	0.265	0.044	0.332	1776.432	0.418	0.306	0.173	0.390
TRAF5	155.571	0.196	0.157	0.211	0.340	155.571	-0.268	0.160	0.093	0.388	155.571	-0.465	0.184	0.012	0.073
TRAF6	225.235	-0.152	0.154	0.325	0.465	225.235	-0.073	0.152	0.633	0.800	225.235	0.079	0.179	0.657	0.817
C3AR1	12.034	-1.857	0.991	0.061	0.134	12.034	-1.507	0.907	0.097	0.391	12.034	0.350	1.192	0.769	0.882
CCT3	6753.285	-0.187	0.246	0.446	0.582	6753.285	-0.505	0.246	0.040	0.327	6753.285	-0.318	0.284	0.262	0.496
TRIO	4131.198	-0.690	0.213	0.001	0.007	4131.198	-0.089	0.212	0.674	0.824	4131.198	0.600	0.246	0.014	0.083
TRIP6	2261.786	-0.058	0.144	0.684	0.782	2261.786	0.148	0.143	0.301	0.567	2261.786	0.207	0.166	0.212	0.437
TRO	7.693	1.067	0.850	0.209	0.338	7.693	0.778	0.853	0.362	0.614	7.693	-0.289	0.971	0.766	0.881
TRPC1	246.454	0.071	0.373	0.848	0.901	246.454	0.456	0.372	0.220	0.499	246.454	0.385	0.430	0.371	0.603
TRPC4	165.843	-0.138	0.266	0.604	0.717	165.843	-0.309	0.266	0.244	0.519	165.843	-0.171	0.308	0.578	0.763
TRPM2	38.404	0.575	0.296	0.052	0.120	38.404	0.050	0.304	0.869	0.933	38.404	-0.525	0.346	0.129	0.326
TRPS1	259.805	-0.249	0.200	0.211	0.340	259.805	-0.043	0.197	0.827	0.910	259.805	0.206	0.230	0.370	0.602
MIG7	17.951	-1.324	0.718	0.065	0.141	17.951	-0.859	0.699	0.219	0.499	17.951	0.466	0.833	0.576	0.762
H2AC19	1533.287	0.795	0.652	0.222	0.353	1533.287	0.986	0.652	0.130	0.422	1533.287	0.191	0.752	0.800	0.900
ANP32AP1	11.078	-0.594	0.506	0.241	0.374	11.078	-0.352	0.481	0.464	0.689	11.078	0.242	0.590	0.682	0.832
SNHG4	189.370	-0.158	0.177	0.371	0.512	189.370	-0.096	0.175	0.584	0.767	189.370	0.062	0.205	0.761	0.879
TSN	1228.652	-0.667	0.219	0.002	0.011	1228.652	-0.575	0.218	0.009	0.297	1228.652	0.093	0.253	0.713	0.851
TSC1	506.408	-0.001	0.182	0.995	0.998	506.408	0.116	0.181	0.524	0.727	506.408	0.117	0.210	0.579	0.763
TSC2	1448.067	0.150	0.190	0.432	0.568	1448.067	0.360	0.190	0.058	0.348	1448.067	0.210	0.220	0.338	0.573
C4BPB	8.385	0.626	0.545	0.251	0.385	8.385	-0.303	0.588	0.606	0.782	8.385	-0.929	0.660	0.159	0.372
TSG101	888.926	-0.525	0.164	0.001	0.007	888.926	-0.211	0.163	0.195	0.479	888.926	0.314	0.189	0.096	0.274
TSNAX	357.980	-0.233	0.193	0.228	0.360	357.980	-0.288	0.193	0.135	0.426	357.980	-0.055	0.224	0.805	0.902
TSPYL1	1855.221	-0.921	0.237	0.000	0.001	1855.221	-0.296	0.236	0.209	0.490	1855.221	0.624	0.273	0.022	0.109
CAPN5	18.230	0.669	0.350	0.056	0.125	18.230	-0.049	0.370	0.894	0.947	18.230	-0.719	0.416	0.084	0.251
EIPR1	521.139	-0.238	0.198	0.229	0.361	521.139	-0.062	0.197	0.754	0.868	521.139	0.176	0.228	0.441	0.661
PHLDA2	1775.482	0.241	0.550	0.662	0.764	1775.482	-1.521	0.551	0.006	0.297	1775.482	-1.762	0.636	0.006	0.044
TST	530.483	0.533	0.217	0.014	0.044	530.483	0.396	0.217	0.069	0.357	530.483	-0.138	0.251	0.583	0.766
TSTA3	1232.904	-0.029	0.275	0.917	0.947	1232.904	-0.346	0.275	0.209	0.490	1232.904	-0.317	0.318	0.318	0.554
TTC1	894.040	0.226	0.211	0.284	0.422	894.040	-0.410	0.212	0.053	0.342	894.040	-0.636	0.245	0.009	0.062
DNAJC7	2096.636	-0.185	0.133	0.165	0.285	2096.636	-0.099	0.133	0.458	0.684	2096.636	0.086	0.154	0.576	0.762
TTC3	3234.444	-0.490	0.328	0.136	0.245	3234.444	0.247	0.328	0.451	0.679	3234.444	0.737	0.379	0.052	0.187
TTC4	133.116	-1.145	0.305	0.000	0.001	133.116	-0.712	0.300	0.018	0.306	133.116	0.433	0.354	0.222	0.449
C5	53.172	-0.562	0.629	0.372	0.512	53.172	0.837	0.618	0.175	0.464	53.172	1.399	0.721	0.052	0.188
TTF1	387.519	0.308	0.185	0.096	0.190	387.519	-0.063	0.186	0.735	0.858	387.519	-0.371	0.214	0.084	0.251
TTK	677.514	-0.307	0.203	0.131	0.239	677.514	-0.080	0.203	0.691	0.834	677.514	0.226	0.235	0.335	0.569

TTN	38.478	0.054	0.322	0.868	0.914	38.478	-0.078	0.322	0.807	0.899	38.478	-0.132	0.375	0.724	0.857
CASC8	243.849	0.660	0.347	0.057	0.127	243.849	0.440	0.347	0.205	0.487	243.849	-0.220	0.400	0.583	0.765
LINC00163	26.958	1.877	0.744	0.012	0.038	26.958	1.274	0.750	0.090	0.384	26.958	-0.603	0.846	0.476	0.689
TUBA4A	1721.960	0.728	0.197	0.000	0.002	1721.960	-0.287	0.198	0.146	0.438	1721.960	-1.015	0.228	0.000	0.000
LOC727751	261.722	-0.445	0.219	0.042	0.101	261.722	0.400	0.214	0.061	0.350	261.722	0.845	0.251	0.001	0.011
ROCK1P1	42.225	-0.439	0.256	0.087	0.176	42.225	0.025	0.238	0.917	0.959	42.225	0.464	0.293	0.113	0.301
MAFIP	157.809	0.466	0.196	0.018	0.052	157.809	0.263	0.197	0.181	0.469	157.809	-0.203	0.226	0.370	0.602
RNF208	12.975	2.063	0.585	0.000	0.003	12.975	0.797	0.614	0.194	0.478	12.975	-1.266	0.667	0.058	0.201
RGPD8	259.383	-0.111	0.259	0.668	0.769	259.383	-0.327	0.259	0.207	0.489	259.383	-0.216	0.300	0.472	0.686
FAM156B	393.584	0.003	0.269	0.990	0.994	393.584	-0.223	0.269	0.406	0.647	393.584	-0.227	0.311	0.465	0.681
LOC727896	8.853	0.190	0.641	0.767	0.846	8.853	-0.426	0.661	0.519	0.724	8.853	-0.616	0.761	0.418	0.643
GOLGA8Q	11.338	0.491	0.777	0.528	0.654	11.338	1.672	0.747	0.025	0.316	11.338	1.182	0.869	0.174	0.390
TLCD2	82.121	1.875	0.576	0.001	0.006	82.121	0.752	0.580	0.195	0.478	82.121	-1.123	0.664	0.091	0.264
GXYLT2	306.434	-0.034	0.220	0.878	0.921	306.434	0.252	0.218	0.248	0.522	306.434	0.285	0.253	0.259	0.494
SDHAP2	1082.902	-0.019	0.302	0.949	0.969	1082.902	0.106	0.302	0.726	0.854	1082.902	0.125	0.349	0.720	0.855
MROH1	449.280	0.720	0.176	0.000	0.000	449.280	0.154	0.178	0.387	0.633	449.280	-0.567	0.204	0.005	0.044
LINC01249	8.462	-0.909	0.759	0.231	0.363	8.462	1.025	0.669	0.125	0.417	8.462	1.935	0.839	0.021	0.105
C5AR1	22.376	1.449	0.639	0.023	0.065	22.376	1.034	0.642	0.107	0.400	22.376	-0.416	0.731	0.570	0.758
TUBB2A	494.811	0.830	0.305	0.006	0.024	494.811	-1.051	0.308	0.001	0.257	494.811	-1.881	0.355	0.000	0.000
BMS1P14	79.283	0.401	0.237	0.091	0.182	79.283	0.098	0.239	0.681	0.827	79.283	-0.303	0.275	0.270	0.505
SSR4P1	78.547	-0.431	0.458	0.346	0.487	78.547	0.350	0.451	0.437	0.670	78.547	0.781	0.526	0.137	0.340
GOLGA8O	27.776	1.166	0.711	0.101	0.197	27.776	1.169	0.710	0.100	0.394	27.776	0.003	0.814	0.997	0.998
FAM133DP	21.089	0.370	0.439	0.399	0.538	21.089	-0.595	0.464	0.200	0.483	21.089	-0.965	0.529	0.068	0.221
ANXA8L1	2412.236	0.477	0.290	0.100	0.196	2412.236	0.112	0.290	0.700	0.839	2412.236	-0.365	0.335	0.275	0.510
NUTM2A	30.359	0.191	0.506	0.705	0.798	30.359	-0.104	0.508	0.838	0.916	30.359	-0.295	0.587	0.615	0.788
NUTM2D	15.801	0.198	0.502	0.693	0.788	15.801	0.329	0.493	0.505	0.715	15.801	0.130	0.575	0.820	0.911
LOC728158	79.809	-0.540	0.359	0.132	0.240	79.809	0.760	0.330	0.021	0.314	79.809	1.300	0.402	0.001	0.015
NUTM2A-AS1	212.025	-0.047	0.138	0.735	0.821	212.025	0.011	0.136	0.938	0.968	212.025	0.057	0.159	0.720	0.854
LINC00460	57.941	-0.635	0.228	0.005	0.021	57.941	0.005	0.210	0.982	0.989	57.941	0.640	0.260	0.014	0.081
RSPH10B2	374.709	0.615	0.305	0.044	0.105	374.709	-0.271	0.307	0.378	0.626	374.709	-0.885	0.354	0.012	0.075
PI4KAP1	80.184	0.594	0.338	0.079	0.163	80.184	0.050	0.341	0.883	0.942	80.184	-0.544	0.392	0.165	0.380
FAM157A	27.152	0.742	0.467	0.112	0.213	27.152	0.000	0.478	0.999	1.000	27.152	-0.743	0.545	0.173	0.390
D2HGDH	343.069	0.171	0.297	0.566	0.687	343.069	0.438	0.296	0.139	0.430	343.069	0.268	0.343	0.435	0.657
TUBG1	1161.748	0.153	0.366	0.676	0.775	1161.748	-0.918	0.366	0.012	0.305	1161.748	-1.071	0.423	0.011	0.071
LINC01881	51.346	-0.538	0.448	0.230	0.363	51.346	-0.274	0.442	0.535	0.734	51.346	0.263	0.518	0.611	0.786
ARHGEF34P	713.363	0.391	0.175	0.026	0.070	713.363	0.279	0.175	0.111	0.405	713.363	-0.112	0.202	0.579	0.763
LOC728392	11.170	1.807	0.708	0.011	0.036	11.170	1.414	0.714	0.048	0.336	11.170	-0.393	0.791	0.619	0.791
TUFM	6538.911	0.043	0.183	0.814	0.878	6538.911	-0.131	0.183	0.474	0.696	6538.911	-0.174	0.211	0.410	0.636
PARGP1	18.210	-0.495	0.397	0.212	0.341	18.210	0.058	0.371	0.875	0.937	18.210	0.554	0.453	0.222	0.449
GUSBP1	132.084	-0.479	0.227	0.034	0.087	132.084	-0.193	0.222	0.383	0.629	132.084	0.286	0.262	0.275	0.510
LINC01137	111.946	1.081	0.241	0.000	0.000	111.946	0.185	0.247	0.452	0.680	111.946	-0.896	0.280	0.001	0.016
DNLZ	13.334	1.696	0.552	0.002	0.010	13.334	0.700	0.577	0.225	0.504	13.334	-0.997	0.630	0.114	0.302
SERF1B	266.287	1.002	0.314	0.001	0.008	266.287	0.184	0.316	0.560	0.749	266.287	-0.818	0.363	0.024	0.115
SPDYE8P	10.307	-0.067	0.604	0.912	0.944	10.307	-0.450	0.612	0.462	0.688	10.307	-0.383	0.713	0.591	0.771
LOC728554	1513.677	0.169	0.249	0.498	0.629	1513.677	-0.258	0.249	0.299	0.566	1513.677	-0.427	0.287	0.137	0.340
ENTPD1-AS1	206.681	-1.132	0.361	0.002	0.009	206.681	-0.902	0.359	0.012	0.305	206.681	0.230	0.418	0.583	0.765
C12orf73	156.320	0.174	0.239	0.467	0.601	156.320	-0.056	0.240	0.814	0.902	156.320	-0.230	0.277	0.406	0.633
CNTNAP3B	158.219	-0.484	0.183	0.008	0.029	158.219	0.096	0.176	0.585	0.768	158.219	0.580	0.210	0.006	0.045
CCDC192	11.191	-0.675	0.573	0.239	0.372	11.191	0.052	0.523	0.921	0.960	11.191	0.727	0.650	0.264	0.498
CCDC169	45.316	-1.048	0.429	0.014	0.045	45.316	-0.361	0.415	0.384	0.629	45.316	0.687	0.494	0.164	0.379
TUFT1	406.426	-1.413	0.291	0.000	0.000	406.426	-0.624	0.288	0.030	0.322	406.426	0.789	0.336	0.019	0.098
SDHAP3	15.764	1.214	0.440	0.006	0.022	15.764	0.557	0.453	0.219	0.498	15.764	-0.657	0.504	0.192	0.415
LOC728613	87.825	0.181	0.250	0.468	0.602	87.825	0.159	0.248	0.521	0.725	87.825	-0.022	0.288	0.938	0.972
CCDC30	11.973	0.790	0.751	0.293	0.431	11.973	0.597	0.751	0.426	0.662	11.973	-0.193	0.859	0.822	0.913
CDK11A	1388.261	0.388	0.291	0.182	0.305	1388.261	-0.384	0.291	0.188	0.474	1388.261	-0.772	0.336	0.022	0.107
HNRNPA1P33	40.348	0.538	0.435	0.216	0.346	40.348	-0.702	0.448	0.117	0.409	40.348	-1.240	0.513	0.016	0.087
RPL13AP5	2307.639	0.210	0.332	0.527	0.654	2307.639	0.545	0.332	0.100	0.394	2307.639	0.335	0.383	0.382	0.611
SLC35E2B	1679.649	-0.458	0.219	0.037	0.091	1679.649	0.241	0.219	0.270	0.541	1679.649	0.699	0.253	0.006	0.045
LOC728673	11.939	-0.991	0.493	0.044	0.106	11.939	-0.433	0.447	0.332	0.592	11.939	0.558	0.570	0.328	0.562
EIF3CL	12439.734	-0.262	0.114	0.022	0.062	12439.734	-0.050	0.114	0.661	0.816	12439.734	0.212	0.132	0.109	0.294
ZBED3-AS1	34.371	-1.482	0.378	0.000	0.001	34.371	-0.176	0.337	0.601	0.779	34.371	1.306	0.428	0.002	0.024
MAP4K3-DT	38.043	0.535	0.358	0.135	0.245	38.043	0.531	0.356	0.136	0.427	38.043	-0.004	0.410	0.992	0.995
NPIPB8	21.783	2.265	0.700	0.001	0.007	21.783	1.332	0.709	0.060	0.350	21.783	-0.933	0.795	0.240	0.471
NPIPB6	8.460	1.536	0.814	0.059	0.131	8.460	0.880	0.828	0.288	0.557	8.460	-0.656	0.925	0.478	0.691
LOC728743	29.770	0.772	0.388	0.047	0.110	29.770	0.094	0.397	0.812	0.901	29.770	-0.678	0.452	0.134	0.333
LOC728752	30.783	-0.264	0.397	0.505	0.635	30.783	-0.029	0.389	0.941	0.970	30.783	0.235	0.458	0.607	0.783
LOC728755	10.797	-1.375	0.568	0.015	0.047	10.797	0.103	0.479	0.829	0.912	10.797	1.479	0.633	0.019	0.100

SCAMP1-AS1	100.792	-0.109	0.182	0.547	0.671	100.792	0.151	0.176	0.391	0.635	100.792	0.260	0.208	0.211	0.436
NSFP1	644.373	-0.942	0.481	0.050	0.116	644.373	-0.123	0.480	0.798	0.895	644.373	0.820	0.555	0.140	0.344
C1GALT1C1L	8.185	0.500	0.644	0.437	0.573	8.185	-0.157	0.663	0.813	0.902	8.185	-0.657	0.757	0.386	0.615
FAM72D	595.595	-0.818	0.215	0.000	0.001	595.595	-0.106	0.213	0.620	0.791	595.595	0.712	0.248	0.004	0.036
NBPF8	642.261	0.497	0.231	0.031	0.081	642.261	0.210	0.231	0.363	0.615	642.261	-0.287	0.266	0.282	0.516
LINC00623	601.695	0.309	0.178	0.083	0.170	601.695	0.099	0.178	0.577	0.762	601.695	-0.210	0.206	0.308	0.544
NPIPB11	121.688	2.002	0.449	0.000	0.000	121.688	0.086	0.457	0.851	0.923	121.688	-1.916	0.521	0.000	0.005
TULP3	349.119	0.104	0.204	0.610	0.722	349.119	0.154	0.203	0.448	0.677	349.119	0.050	0.235	0.831	0.917
LOC728975	114.927	-0.062	0.286	0.827	0.886	114.927	0.419	0.281	0.136	0.427	114.927	0.482	0.328	0.142	0.347
HIRA	834.071	-0.967	0.295	0.001	0.006	834.071	-0.609	0.295	0.039	0.327	834.071	0.359	0.341	0.293	0.528
ZBED5-AS1	84.193	0.349	0.428	0.414	0.552	84.193	0.195	0.428	0.648	0.808	84.193	-0.154	0.494	0.755	0.875
OIP5-AS1	1725.260	0.074	0.178	0.680	0.778	1725.260	-0.040	0.178	0.824	0.908	1725.260	-0.113	0.206	0.582	0.765
TWIST1	186.221	1.316	0.355	0.000	0.002	186.221	0.681	0.357	0.056	0.344	186.221	-0.634	0.409	0.121	0.313
GTF2IRD1P1	158.497	0.772	0.192	0.000	0.001	158.497	0.226	0.195	0.247	0.521	158.497	-0.546	0.222	0.014	0.081
FAM239B	460.324	-0.622	0.153	0.000	0.001	460.324	-0.088	0.150	0.558	0.748	460.324	0.535	0.176	0.002	0.024
STXBP5-AS1	50.352	-0.618	0.396	0.118	0.222	50.352	-0.135	0.386	0.726	0.853	50.352	0.483	0.456	0.290	0.525
TNFSF4	7.930	0.964	0.676	0.154	0.270	7.930	0.688	0.679	0.311	0.575	7.930	-0.276	0.769	0.720	0.855
LOC729218	59.929	0.056	0.294	0.849	0.901	59.929	-0.126	0.294	0.668	0.819	59.929	-0.182	0.342	0.594	0.773
FAHD2CP	64.520	0.925	0.384	0.016	0.049	64.520	0.259	0.388	0.504	0.715	64.520	-0.665	0.445	0.135	0.335
NUTM2B	13.456	0.162	0.576	0.778	0.853	13.456	0.052	0.573	0.928	0.964	13.456	-0.110	0.666	0.869	0.937
TP53TG3D	18.344	0.709	0.709	0.317	0.457	18.344	0.604	0.708	0.393	0.637	18.344	-0.105	0.813	0.898	0.952
ZNF286B	28.694	0.817	0.294	0.005	0.021	28.694	0.354	0.301	0.240	0.516	28.694	-0.462	0.338	0.172	0.389
LOC729291	15.171	-0.096	0.399	0.810	0.875	15.171	-0.383	0.404	0.343	0.600	15.171	-0.287	0.474	0.545	0.741
PLIN4	44.778	1.818	0.830	0.028	0.075	44.778	0.584	0.835	0.484	0.702	44.778	-1.235	0.958	0.197	0.420
FAM86HP	14.632	-0.491	0.480	0.306	0.445	14.632	-0.119	0.459	0.795	0.893	14.632	0.372	0.553	0.501	0.708
GAGE12B	7.666	1.080	0.959	0.260	0.396	7.666	0.153	0.981	0.876	0.937	7.666	-0.927	1.115	0.406	0.633
GAGE12E	13.708	1.179	0.681	0.083	0.170	13.708	0.743	0.686	0.279	0.549	13.708	-0.436	0.779	0.575	0.761
CASTOR2	79.971	1.406	0.254	0.000	0.000	79.971	0.144	0.265	0.587	0.768	79.971	-1.262	0.296	0.000	0.001
CCDC61	114.111	-0.785	0.336	0.019	0.056	114.111	-0.350	0.331	0.290	0.558	114.111	0.434	0.388	0.263	0.497
TXN	4254.518	0.896	0.253	0.000	0.003	4254.518	-0.221	0.253	0.381	0.628	4254.518	-1.117	0.292	0.000	0.003
TMEM242	335.243	0.269	0.174	0.122	0.228	335.243	-0.221	0.176	0.207	0.489	335.243	-0.490	0.202	0.015	0.087
FAM72A	604.694	-0.841	0.234	0.000	0.002	604.694	-0.043	0.232	0.851	0.924	604.694	0.798	0.269	0.003	0.029
RGPD6	29.502	-0.830	0.381	0.029	0.077	29.502	-0.077	0.356	0.827	0.910	29.502	0.753	0.435	0.084	0.251
SPDYE6	10.752	1.350	0.479	0.005	0.019	10.752	-0.569	0.567	0.315	0.578	10.752	-1.919	0.610	0.002	0.019
TXNRD1	16276.018	-0.536	0.204	0.009	0.030	16276.018	-0.444	0.204	0.029	0.322	16276.018	0.092	0.235	0.696	0.841
LOC729603	10.918	1.342	0.576	0.020	0.057	10.918	0.262	0.621	0.673	0.823	10.918	-1.080	0.675	0.109	0.295
FLJ37453	122.385	-0.402	0.261	0.124	0.229	122.385	0.086	0.255	0.737	0.859	122.385	0.488	0.300	0.104	0.287
CCDC175	27.506	0.597	0.346	0.085	0.172	27.506	-0.276	0.366	0.450	0.679	27.506	-0.873	0.413	0.035	0.145
LINC00847	474.862	-0.049	0.196	0.803	0.870	474.862	0.308	0.195	0.113	0.405	474.862	0.357	0.226	0.113	0.301
TYK2	1317.647	0.019	0.116	0.873	0.918	1317.647	-0.002	0.116	0.983	0.990	1317.647	-0.021	0.134	0.876	0.940
SEC14L1P1	8.607	-0.185	0.796	0.816	0.879	8.607	-1.243	0.835	0.137	0.428	8.607	-1.058	0.961	0.271	0.506
TYMS	956.642	-0.138	0.205	0.501	0.631	956.642	-0.544	0.205	0.008	0.297	956.642	-0.406	0.237	0.087	0.258
FAM160A1	250.003	0.776	0.462	0.093	0.186	250.003	0.769	0.462	0.096	0.391	250.003	-0.007	0.533	0.990	0.994
UMAD1	347.467	-0.395	0.136	0.004	0.016	347.467	0.023	0.133	0.863	0.930	347.467	0.417	0.157	0.008	0.055
LOC729867	347.445	-0.612	0.250	0.014	0.044	347.445	-0.082	0.247	0.739	0.861	347.445	0.530	0.288	0.066	0.216
TBC1D3	213.575	1.406	0.913	0.123	0.229	213.575	1.922	0.912	0.035	0.323	213.575	0.516	1.052	0.624	0.794
TBC1D3H	567.321	1.525	0.729	0.037	0.091	567.321	1.332	0.729	0.068	0.356	567.321	-0.193	0.842	0.819	0.910
MORN2	131.883	0.668	0.252	0.008	0.029	131.883	0.183	0.254	0.472	0.694	131.883	-0.485	0.292	0.096	0.273
LOC729970	10.385	0.667	0.427	0.118	0.222	10.385	-0.429	0.471	0.363	0.615	10.385	-1.096	0.525	0.037	0.151
NPIPB2	40.223	1.512	0.536	0.005	0.019	40.223	0.463	0.544	0.395	0.639	40.223	-1.049	0.619	0.090	0.263
BORCS8	73.178	-0.099	0.301	0.743	0.827	73.178	-0.230	0.301	0.444	0.675	73.178	-0.131	0.350	0.707	0.848
ZNF814	78.266	0.369	0.270	0.172	0.293	78.266	-0.107	0.273	0.696	0.837	78.266	-0.475	0.315	0.131	0.328
MOSMO	158.850	-0.334	0.225	0.138	0.248	158.850	-0.211	0.222	0.343	0.600	158.850	0.123	0.261	0.636	0.802
TYRO3	1592.436	-0.421	0.128	0.001	0.006	1592.436	0.163	0.126	0.196	0.480	1592.436	0.584	0.147	0.000	0.002
LOC730101	69.865	-0.813	0.268	0.002	0.011	69.865	-0.368	0.259	0.156	0.448	69.865	0.445	0.310	0.151	0.360
CRYZL2P	33.251	0.380	0.502	0.449	0.585	33.251	0.790	0.495	0.111	0.404	33.251	0.410	0.574	0.475	0.688
FAM166B	9.947	0.798	0.688	0.246	0.380	9.947	-0.160	0.717	0.823	0.908	9.947	-0.958	0.811	0.238	0.467
LOC730183	29.137	-1.155	0.370	0.002	0.009	29.137	-0.268	0.335	0.423	0.659	29.137	0.887	0.422	0.035	0.147
LOC730202	18.252	-0.347	0.544	0.524	0.651	18.252	-0.700	0.550	0.202	0.486	18.252	-0.353	0.642	0.582	0.765
LOC730234	23.579	0.642	0.375	0.087	0.176	23.579	-0.410	0.398	0.304	0.570	23.579	-1.052	0.450	0.019	0.100
LOC730268	355.997	-0.321	0.153	0.036	0.090	355.997	0.307	0.149	0.040	0.327	355.997	0.627	0.175	0.000	0.006
GTF2H2C_2	413.982	-1.779	0.451	0.000	0.001	413.982	-1.368	0.449	0.002	0.297	413.982	0.411	0.521	0.430	0.653
TYRP1	24.983	-0.212	0.860	0.805	0.871	24.983	0.979	0.847	0.248	0.522	24.983	1.192	0.986	0.227	0.455
U2AF1	59.250	0.553	0.384	0.150	0.264	59.250	0.472	0.383	0.217	0.497	59.250	-0.081	0.441	0.855	0.930
UBA52	16887.443	0.388	0.227	0.088	0.178	16887.443	0.428	0.227	0.060	0.350	16887.443	0.040	0.263	0.880	0.943
LINC01347	12.665	0.447	0.508	0.379	0.519	12.665	1.011	0.482	0.036	0.324	12.665	0.564	0.562	0.316	0.552
UBB	5614.035	0.338	0.152	0.027	0.072	5614.035	0.068	0.153	0.654	0.811	5614.035	-0.270	0.176	0.126	0.320

UBC	95696.805	0.830	0.172	0.000	0.000	95696.805	0.258	0.172	0.133	0.424	95696.805	-0.572	0.198	0.004	0.035
UBA1	6164.522	-0.306	0.136	0.025	0.068	6164.522	-0.125	0.136	0.358	0.611	6164.522	0.181	0.157	0.251	0.483
UBA7	288.350	1.351	0.392	0.001	0.004	288.350	0.646	0.393	0.100	0.394	288.350	-0.705	0.452	0.119	0.309
UBE2A	1175.729	-0.109	0.147	0.460	0.595	1175.729	-0.202	0.147	0.171	0.460	1175.729	-0.093	0.170	0.586	0.767
UBE2B	1299.908	0.090	0.117	0.444	0.580	1299.908	-0.045	0.117	0.702	0.840	1299.908	-0.135	0.136	0.322	0.556
UBE2D1	341.444	-0.229	0.211	0.279	0.417	341.444	-0.255	0.211	0.226	0.505	341.444	-0.027	0.245	0.914	0.960
UBE2D2	2800.689	-0.117	0.091	0.196	0.322	2800.689	0.031	0.090	0.733	0.857	2800.689	0.148	0.105	0.158	0.370
UBE2D3	5243.245	-0.001	0.122	0.991	0.995	5243.245	-0.037	0.122	0.763	0.874	5243.245	-0.035	0.141	0.802	0.901
UBE2E1	3199.437	0.251	0.111	0.024	0.065	3199.437	-0.054	0.111	0.627	0.795	3199.437	-0.305	0.128	0.017	0.094
UBE2E2	438.288	0.099	0.125	0.429	0.566	438.288	-0.072	0.125	0.563	0.751	438.288	-0.171	0.145	0.238	0.468
UBE2G1	569.581	-0.555	0.176	0.002	0.008	569.581	-0.116	0.174	0.505	0.715	569.581	0.439	0.203	0.031	0.134
UBE2G2	1496.022	-0.159	0.244	0.514	0.643	1496.022	-0.600	0.245	0.014	0.306	1496.022	-0.440	0.282	0.119	0.310
UBE2H	3249.302	-0.225	0.170	0.186	0.310	3249.302	0.041	0.170	0.809	0.900	3249.302	0.266	0.196	0.176	0.393
UBE2I	2740.591	0.166	0.154	0.283	0.421	2740.591	-0.167	0.155	0.281	0.551	2740.591	-0.332	0.179	0.063	0.211
UBE2L3	2314.566	0.235	0.147	0.110	0.210	2314.566	-0.356	0.147	0.016	0.306	2314.566	-0.591	0.170	0.001	0.008
UBE2N	2125.902	0.017	0.230	0.942	0.963	2125.902	-0.513	0.230	0.026	0.316	2125.902	-0.530	0.265	0.046	0.173
UBE2V1	253.771	-0.280	0.166	0.092	0.185	253.771	-0.135	0.164	0.412	0.651	253.771	0.145	0.192	0.451	0.670
UBE2V2	1525.260	0.040	0.201	0.844	0.897	1525.260	-0.463	0.201	0.021	0.314	1525.260	-0.503	0.232	0.030	0.133
UBE3A	1356.211	-0.567	0.169	0.001	0.005	1356.211	-0.309	0.168	0.066	0.354	1356.211	0.257	0.195	0.187	0.407
OSGIN2	393.820	-0.772	0.217	0.000	0.003	393.820	-0.307	0.214	0.151	0.444	393.820	0.465	0.250	0.063	0.211
SUMO1	1731.828	0.335	0.209	0.109	0.209	1731.828	-0.391	0.210	0.062	0.350	1731.828	-0.727	0.242	0.003	0.027
UBP1	1742.240	-0.955	0.272	0.000	0.003	1742.240	-0.638	0.271	0.019	0.306	1742.240	0.317	0.314	0.313	0.548
UBTF	2177.614	-0.504	0.136	0.000	0.002	2177.614	-0.153	0.136	0.259	0.531	2177.614	0.350	0.157	0.026	0.119
UCHL3	75.426	0.421	0.385	0.275	0.411	75.426	-0.209	0.389	0.592	0.773	75.426	-0.629	0.448	0.160	0.373
UCN	60.101	1.840	0.339	0.000	0.000	60.101	0.707	0.349	0.043	0.331	60.101	-1.133	0.389	0.004	0.033
UFD1	1103.360	0.075	0.278	0.788	0.860	1103.360	-0.527	0.278	0.058	0.348	1103.360	-0.601	0.322	0.061	0.208
SLC35A2	396.395	0.011	0.262	0.966	0.979	396.395	-0.315	0.262	0.229	0.507	396.395	-0.326	0.303	0.281	0.515
UGCG	957.756	0.332	0.203	0.101	0.197	957.756	-0.004	0.203	0.985	0.992	957.756	-0.336	0.234	0.152	0.361
UGDH	774.382	-0.617	0.181	0.001	0.004	774.382	-0.149	0.179	0.405	0.646	774.382	0.468	0.209	0.025	0.117
UGP2	1897.535	0.129	0.183	0.479	0.613	1897.535	0.119	0.183	0.515	0.721	1897.535	-0.011	0.211	0.960	0.981
UGT2B7	109.030	0.419	0.245	0.088	0.177	109.030	0.010	0.248	0.969	0.983	109.030	-0.410	0.285	0.151	0.360
UGT8	18.644	0.706	0.353	0.045	0.107	18.644	0.209	0.363	0.565	0.753	18.644	-0.497	0.410	0.225	0.453
UCK2	1991.417	-0.599	0.224	0.008	0.027	1991.417	-0.352	0.224	0.117	0.409	1991.417	0.247	0.259	0.339	0.574
UMPS	729.565	-0.802	0.246	0.001	0.006	729.565	-0.549	0.246	0.025	0.316	729.565	0.253	0.285	0.375	0.607
UNG	604.030	-0.359	0.261	0.169	0.289	604.030	-0.574	0.261	0.028	0.321	604.030	-0.215	0.302	0.477	0.690
USP4	928.700	-0.417	0.155	0.007	0.026	928.700	-0.366	0.154	0.018	0.306	928.700	0.051	0.179	0.775	0.886
NR1H2	2286.386	0.402	0.128	0.002	0.008	2286.386	0.070	0.128	0.582	0.766	2286.386	-0.332	0.147	0.024	0.115
UPP1	1075.845	-0.696	0.663	0.293	0.432	1075.845	-1.588	0.663	0.017	0.306	1075.845	-0.891	0.766	0.245	0.476
UPK2	125.098	-0.901	0.490	0.066	0.143	125.098	0.343	0.484	0.478	0.698	125.098	1.244	0.564	0.027	0.124
VPS51	2478.208	-0.073	0.203	0.719	0.809	2478.208	0.303	0.203	0.135	0.426	2478.208	0.376	0.234	0.109	0.294
UQCRB	3481.502	0.974	0.271	0.000	0.002	3481.502	0.308	0.271	0.256	0.529	3481.502	-0.666	0.313	0.034	0.142
UQCRC1	5050.732	0.040	0.119	0.738	0.823	5050.732	0.196	0.119	0.099	0.394	5050.732	0.156	0.137	0.255	0.488
UQCRC2	4093.653	-0.410	0.126	0.001	0.006	4093.653	0.057	0.125	0.647	0.807	4093.653	0.468	0.145	0.001	0.016
UQCRFS1	1202.404	-0.450	0.194	0.020	0.058	1202.404	-0.235	0.194	0.225	0.504	1202.404	0.215	0.224	0.337	0.571
UQCRH	4897.450	0.547	0.201	0.007	0.025	4897.450	0.236	0.202	0.242	0.518	4897.450	-0.311	0.233	0.181	0.399
UROD	1173.536	0.558	0.154	0.000	0.002	1173.536	0.163	0.154	0.291	0.559	1173.536	-0.396	0.178	0.026	0.120
UROS	710.115	0.000	0.264	0.999	1.000	710.115	-0.463	0.265	0.080	0.371	710.115	-0.463	0.306	0.130	0.327
USF1	543.055	0.409	0.145	0.005	0.019	543.055	0.312	0.145	0.031	0.322	543.055	-0.097	0.167	0.563	0.754
USF2	2303.361	0.609	0.156	0.000	0.001	2303.361	0.127	0.156	0.414	0.653	2303.361	-0.482	0.180	0.007	0.053
USP1	1342.102	-1.463	0.284	0.000	0.000	1342.102	-0.634	0.283	0.025	0.316	1342.102	0.829	0.328	0.011	0.071
MRPL49	696.778	-0.273	0.188	0.145	0.258	696.778	-0.244	0.187	0.192	0.477	696.778	0.029	0.217	0.894	0.949
UTRN	1529.633	-0.425	0.221	0.054	0.122	1529.633	-0.010	0.220	0.963	0.980	1529.633	0.415	0.255	0.103	0.286
KDM6A	258.585	-0.658	0.299	0.028	0.073	258.585	-0.148	0.296	0.617	0.789	258.585	0.510	0.345	0.139	0.342
UVRAG	557.997	-0.266	0.130	0.041	0.099	557.997	-0.136	0.129	0.292	0.559	557.997	0.130	0.150	0.386	0.615
VARS1	16661.355	-0.462	0.306	0.131	0.239	16661.355	-0.485	0.306	0.113	0.405	16661.355	-0.023	0.353	0.948	0.976
VASP	2071.258	0.100	0.219	0.650	0.755	2071.258	-0.289	0.219	0.187	0.474	2071.258	-0.389	0.253	0.125	0.319
ZNHIT2	254.397	-0.346	0.347	0.318	0.458	254.397	-0.586	0.347	0.092	0.386	254.397	-0.239	0.402	0.551	0.745
VAV2	969.529	-0.265	0.116	0.023	0.064	969.529	-0.144	0.116	0.213	0.493	969.529	0.121	0.135	0.368	0.600
VBP1	1068.439	-0.506	0.176	0.004	0.017	1068.439	-0.377	0.176	0.032	0.322	1068.439	0.129	0.204	0.526	0.726
VCL	1455.293	-0.086	0.186	0.643	0.749	1455.293	-0.181	0.186	0.330	0.590	1455.293	-0.095	0.215	0.659	0.817
VCP	4588.393	-0.084	0.253	0.739	0.824	4588.393	-0.599	0.253	0.018	0.306	4588.393	-0.515	0.292	0.078	0.241
VDAC1	10370.053	0.262	0.138	0.058	0.129	10370.053	0.048	0.138	0.729	0.855	10370.053	-0.214	0.160	0.180	0.398
VDAC2	5010.664	0.146	0.169	0.389	0.529	5010.664	0.276	0.169	0.102	0.395	5010.664	0.131	0.195	0.504	0.711
VDAC3	2227.725	0.072	0.160	0.653	0.757	2227.725	-0.253	0.160	0.114	0.405	2227.725	-0.326	0.185	0.079	0.242
VDR	415.081	-0.388	0.175	0.027	0.072	415.081	0.145	0.173	0.400	0.643	415.081	0.533	0.202	0.008	0.057
VEGFA	6948.065	-0.102	0.243	0.673	0.773	6948.065	0.018	0.243	0.942	0.971	6948.065	0.120	0.281	0.669	0.824
VEGFB	922.697	1.107	0.338	0.001	0.006	922.697	0.612	0.338	0.071	0.360	922.697	-0.496	0.390	0.204	0.428

VEGFC	217.482	1.046	0.363	0.004	0.016	217.482	-0.698	0.374	0.062	0.350	217.482	-1.744	0.427	0.000	0.001
VGF	115.281	-0.104	0.487	0.831	0.889	115.281	-0.826	0.492	0.093	0.388	115.281	-0.722	0.568	0.204	0.427
VHL	1825.066	-0.361	0.133	0.007	0.025	1825.066	-0.106	0.133	0.425	0.661	1825.066	0.255	0.154	0.097	0.275
VIL1	154.250	0.040	0.253	0.876	0.920	154.250	0.077	0.251	0.758	0.871	154.250	0.038	0.292	0.897	0.951
EZR	7922.469	-0.231	0.103	0.025	0.069	7922.469	-0.245	0.103	0.018	0.306	7922.469	-0.014	0.119	0.906	0.957
VIM	73501.888	0.415	0.219	0.058	0.129	73501.888	0.381	0.219	0.082	0.373	73501.888	-0.034	0.253	0.892	0.948
VIPR1	45.680	-0.625	0.688	0.363	0.504	45.680	-0.219	0.683	0.749	0.866	45.680	0.407	0.794	0.609	0.784
VLDLR	213.210	0.702	0.420	0.095	0.189	213.210	0.556	0.420	0.186	0.473	213.210	-0.146	0.484	0.763	0.880
BEST1	194521.988	1.165	0.248	0.000	0.000	194521.988	0.555	0.248	0.025	0.316	194521.988	-0.610	0.286	0.033	0.139
TRPV1	14.592	1.927	0.722	0.008	0.027	14.592	1.201	0.730	0.100	0.394	14.592	-0.726	0.820	0.376	0.607
VRK1	478.252	-0.882	0.464	0.057	0.128	478.252	-1.431	0.465	0.002	0.297	478.252	-0.549	0.537	0.307	0.543
VRK2	585.557	-0.382	0.131	0.004	0.015	585.557	-0.343	0.131	0.009	0.297	585.557	0.039	0.152	0.800	0.900
VSNL1	31.162	0.573	0.356	0.107	0.206	31.162	0.057	0.362	0.876	0.937	31.162	-0.517	0.414	0.212	0.437
MYRF	32.117	0.500	0.470	0.288	0.426	32.117	0.155	0.473	0.743	0.863	32.117	-0.344	0.544	0.527	0.727
WARS1	2332.249	-0.489	0.379	0.197	0.323	2332.249	-0.841	0.379	0.027	0.318	2332.249	-0.352	0.438	0.422	0.646
WIPF1	550.386	0.032	0.149	0.831	0.889	550.386	0.018	0.149	0.902	0.950	550.386	-0.013	0.172	0.938	0.972
EIF4H	9808.213	-0.281	0.111	0.011	0.037	9808.213	0.030	0.111	0.786	0.888	9808.213	0.311	0.128	0.015	0.085
TMEM258	1303.240	0.827	0.227	0.000	0.002	1303.240	0.480	0.227	0.035	0.322	1303.240	-0.348	0.262	0.184	0.404
CLIP2	1024.520	-0.058	0.264	0.825	0.885	1024.520	0.305	0.263	0.247	0.522	1024.520	0.363	0.305	0.233	0.462
LAT2	58.495	-0.831	0.453	0.066	0.143	58.495	-0.612	0.448	0.172	0.461	58.495	0.219	0.525	0.677	0.829
CORO2A	128.077	0.824	0.427	0.054	0.122	128.077	0.442	0.428	0.301	0.568	128.077	-0.382	0.492	0.438	0.659
WEE1	809.781	-0.665	0.211	0.002	0.008	809.781	0.318	0.209	0.128	0.420	809.781	0.982	0.243	0.000	0.001
WFS1	200.207	0.921	0.223	0.000	0.000	200.207	-0.136	0.229	0.553	0.745	200.207	-1.056	0.261	0.000	0.001
NSD2	3163.489	-0.754	0.184	0.000	0.000	3163.489	-0.422	0.183	0.022	0.314	3163.489	0.333	0.212	0.117	0.306
NELFA	1013.153	-0.051	0.155	0.743	0.827	1013.153	0.106	0.154	0.492	0.708	1013.153	0.157	0.179	0.380	0.610
DAGLA	187.066	-0.073	0.171	0.670	0.770	187.066	-0.259	0.171	0.130	0.422	187.066	-0.186	0.199	0.350	0.583
WNT3	270.371	0.236	0.274	0.389	0.529	270.371	0.472	0.273	0.083	0.374	270.371	0.237	0.315	0.453	0.672
WNT5A	32.482	0.536	0.494	0.278	0.415	32.482	1.114	0.474	0.019	0.306	32.482	0.578	0.550	0.293	0.528
WNT10B	75.872	-1.608	0.485	0.001	0.005	75.872	-0.856	0.475	0.071	0.361	75.872	0.752	0.560	0.179	0.398
WNT2B	79.731	0.544	0.279	0.051	0.117	79.731	0.219	0.280	0.435	0.668	79.731	-0.325	0.322	0.313	0.548
WNT9A	36.167	1.131	0.428	0.008	0.029	36.167	0.488	0.436	0.264	0.536	36.167	-0.644	0.492	0.191	0.413
GET1	139.626	0.293	0.220	0.183	0.307	139.626	0.167	0.220	0.447	0.677	139.626	-0.126	0.254	0.620	0.792
WRN	332.716	-0.830	0.248	0.001	0.005	332.716	-0.386	0.245	0.116	0.409	332.716	0.445	0.286	0.120	0.311
XBP1	4082.735	-1.530	0.401	0.000	0.001	4082.735	-0.715	0.401	0.074	0.364	4082.735	0.815	0.463	0.079	0.242
XDH	264.915	-0.953	0.270	0.000	0.003	264.915	-0.035	0.266	0.895	0.948	264.915	0.918	0.311	0.003	0.030
XIST	654.691	0.967	0.389	0.013	0.041	654.691	0.441	0.389	0.257	0.529	654.691	-0.525	0.449	0.242	0.473
XK	31.885	-0.025	0.280	0.930	0.956	31.885	0.070	0.273	0.798	0.895	31.885	0.094	0.322	0.769	0.883
XPA	275.471	-0.053	0.165	0.750	0.833	275.471	0.055	0.163	0.734	0.858	275.471	0.108	0.190	0.570	0.759
XPC	813.064	-0.759	0.127	0.000	0.000	813.064	-0.068	0.125	0.587	0.769	813.064	0.691	0.147	0.000	0.000
CSKMT	276.394	-0.749	0.166	0.000	0.000	276.394	0.221	0.159	0.164	0.455	276.394	0.970	0.190	0.000	0.000
XPNPEP1	1255.921	0.052	0.165	0.752	0.835	1255.921	0.097	0.165	0.555	0.746	1255.921	0.045	0.190	0.813	0.907
XPO1	2928.217	-0.491	0.199	0.014	0.044	2928.217	-0.456	0.199	0.022	0.315	2928.217	0.035	0.230	0.880	0.943
XRCC1	894.564	0.292	0.205	0.154	0.270	894.564	0.283	0.205	0.167	0.456	894.564	-0.009	0.236	0.970	0.985
XRCC2	173.323	-1.551	0.463	0.001	0.005	173.323	-1.263	0.461	0.006	0.297	173.323	0.289	0.537	0.591	0.771
XRCC3	405.789	-0.400	0.433	0.356	0.496	405.789	-0.959	0.434	0.027	0.319	405.789	-0.560	0.501	0.264	0.499
XRCC4	160.280	-0.387	0.234	0.098	0.193	160.280	-0.327	0.232	0.159	0.451	160.280	0.060	0.271	0.824	0.914
FMNL1	1062.950	0.700	0.262	0.008	0.027	1062.950	0.595	0.262	0.023	0.316	1062.950	-0.105	0.303	0.728	0.859
XRCC5	4079.461	-0.406	0.246	0.099	0.195	4079.461	-0.641	0.246	0.009	0.297	4079.461	-0.235	0.284	0.409	0.635
CEMP1	62.607	1.713	0.289	0.000	0.000	62.607	0.278	0.311	0.371	0.620	62.607	-1.435	0.338	0.000	0.001
YES1	2106.481	-0.418	0.157	0.008	0.028	2106.481	-0.109	0.157	0.487	0.703	2106.481	0.309	0.182	0.089	0.262
YY1	1984.461	-0.276	0.093	0.003	0.014	1984.461	-0.016	0.093	0.860	0.928	1984.461	0.260	0.108	0.016	0.088
YWHAB	4027.223	0.273	0.247	0.268	0.405	4027.223	-0.377	0.247	0.127	0.420	4027.223	-0.651	0.286	0.023	0.109
YWHAE	13013.424	-0.268	0.113	0.018	0.052	13013.424	-0.182	0.113	0.107	0.399	13013.424	0.086	0.130	0.509	0.715
YWHAG	5727.517	-0.178	0.141	0.206	0.334	5727.517	-0.244	0.141	0.083	0.374	5727.517	-0.066	0.163	0.684	0.834
YWHAH	1960.820	-0.245	0.323	0.448	0.583	1960.820	-0.846	0.323	0.009	0.297	1960.820	-0.601	0.373	0.108	0.293
YWHAZ	11054.607	-0.094	0.082	0.250	0.384	11054.607	-0.170	0.082	0.038	0.327	11054.607	-0.076	0.094	0.422	0.646
SF1	2654.635	-0.525	0.192	0.006	0.024	2654.635	-0.346	0.192	0.071	0.360	2654.635	0.179	0.222	0.420	0.645
ZFP36	1063.047	0.611	0.214	0.004	0.017	1063.047	0.530	0.214	0.013	0.305	1063.047	-0.081	0.246	0.743	0.868
ZFP37	58.632	-0.001	0.293	0.998	0.999	58.632	0.405	0.285	0.156	0.448	58.632	0.405	0.334	0.226	0.454
PTTG1IP	9640.168	0.805	0.467	0.085	0.173	9640.168	0.702	0.467	0.133	0.424	9640.168	-0.103	0.539	0.849	0.927
ZBTB14	158.551	-0.500	0.203	0.014	0.043	158.551	-0.214	0.199	0.280	0.550	158.551	0.285	0.235	0.224	0.451
ZFPL1	600.429	0.506	0.167	0.002	0.011	600.429	0.212	0.167	0.204	0.487	600.429	-0.294	0.193	0.127	0.322
ZFX	493.970	-0.275	0.141	0.051	0.118	493.970	-0.167	0.140	0.231	0.508	493.970	0.107	0.163	0.511	0.716
ZIC1	60.812	0.916	0.433	0.034	0.087	60.812	0.770	0.432	0.075	0.364	60.812	-0.145	0.497	0.770	0.883
ZIC2	268.219	0.044	0.164	0.789	0.860	268.219	0.243	0.161	0.132	0.424	268.219	0.199	0.188	0.290	0.526
ZNF2	90.530	-0.139	0.250	0.578	0.696	90.530	0.165	0.244	0.498	0.711	90.530	0.304	0.287	0.288	0.524
CFAP410	265.790	0.917	0.254	0.000	0.002	265.790	0.432	0.255	0.090	0.385	265.790	-0.485	0.293	0.098	0.277

ZNF3	326.109	-0.001	0.138	0.996	0.998	326.109	0.028	0.137	0.838	0.916	326.109	0.029	0.159	0.858	0.932
ZNF7	303.599	-0.374	0.148	0.011	0.037	303.599	0.059	0.144	0.681	0.827	303.599	0.433	0.170	0.011	0.069
ZNF8	95.591	0.826	0.255	0.001	0.007	95.591	0.503	0.257	0.050	0.337	95.591	-0.323	0.293	0.271	0.506
CNBP	11264.374	-0.036	0.107	0.734	0.821	11264.374	-0.104	0.107	0.334	0.593	11264.374	-0.067	0.124	0.588	0.769
ZNF10	27.902	0.519	0.359	0.148	0.262	27.902	-0.146	0.373	0.695	0.836	27.902	-0.665	0.425	0.117	0.307
ZNF12	421.053	-0.305	0.159	0.055	0.124	421.053	-0.196	0.158	0.215	0.494	421.053	0.109	0.184	0.553	0.747
ZNF14	66.366	-0.660	0.209	0.002	0.008	66.366	-0.192	0.195	0.327	0.587	66.366	0.468	0.241	0.052	0.188
ZNF708	26.495	0.097	0.347	0.781	0.854	26.495	-0.200	0.350	0.569	0.756	26.495	-0.296	0.407	0.467	0.682
ZNF16	109.672	-0.009	0.186	0.961	0.977	109.672	0.025	0.183	0.890	0.945	109.672	0.034	0.215	0.873	0.939
ZNF17	49.559	-0.883	0.320	0.006	0.022	49.559	-0.399	0.306	0.192	0.477	49.559	0.484	0.370	0.190	0.411
ZNF18	36.131	0.306	0.274	0.263	0.399	36.131	-0.081	0.278	0.771	0.878	36.131	-0.387	0.321	0.227	0.455
ZNF19	34.654	-0.141	0.427	0.741	0.826	34.654	0.613	0.413	0.138	0.429	34.654	0.754	0.486	0.121	0.313
ZNF182	100.534	-0.253	0.196	0.196	0.321	100.534	0.075	0.189	0.694	0.835	100.534	0.328	0.225	0.145	0.351
TMEM50B	1092.764	0.905	0.161	0.000	0.000	1092.764	-0.031	0.162	0.847	0.921	1092.764	-0.936	0.187	0.000	0.000
ZNF22	665.205	-0.320	0.110	0.004	0.015	665.205	-0.031	0.108	0.775	0.880	665.205	0.290	0.127	0.022	0.109
ZNF23	259.675	0.241	0.223	0.280	0.418	259.675	-0.110	0.224	0.625	0.794	259.675	-0.351	0.259	0.176	0.393
ZNF24	847.265	-0.415	0.155	0.007	0.027	847.265	-0.200	0.154	0.194	0.478	847.265	0.215	0.179	0.230	0.459
ZNF26	147.070	-0.560	0.233	0.016	0.049	147.070	-0.114	0.227	0.617	0.789	147.070	0.446	0.268	0.096	0.273
ZNF28	114.225	-0.634	0.267	0.018	0.052	114.225	-0.059	0.260	0.821	0.906	114.225	0.575	0.308	0.061	0.208
ZSCAN20	74.368	-0.501	0.318	0.115	0.217	74.368	0.001	0.310	0.997	0.999	74.368	0.502	0.366	0.170	0.385
ZNF32	168.138	0.649	0.210	0.002	0.010	168.138	0.046	0.213	0.827	0.910	168.138	-0.603	0.244	0.014	0.080
ZNF33A	432.131	-0.538	0.241	0.025	0.069	432.131	0.107	0.238	0.653	0.811	432.131	0.645	0.277	0.020	0.101
ZNF33B	481.119	-0.323	0.309	0.296	0.434	481.119	0.414	0.307	0.177	0.466	481.119	0.737	0.356	0.038	0.154
ZNF35	75.076	-0.201	0.406	0.620	0.731	75.076	-0.780	0.410	0.057	0.347	75.076	-0.579	0.475	0.223	0.450
ZKSCAN1	1533.228	0.009	0.263	0.972	0.983	1533.228	0.152	0.263	0.563	0.751	1533.228	0.143	0.304	0.638	0.803
ZNF37A	658.794	-0.290	0.149	0.052	0.118	658.794	-0.243	0.148	0.102	0.394	658.794	0.047	0.172	0.783	0.891
ZSCAN21	126.600	-0.119	0.227	0.599	0.713	126.600	-0.302	0.227	0.182	0.469	126.600	-0.183	0.264	0.488	0.698
ZNF41	188.496	-0.969	0.233	0.000	0.000	188.496	-0.294	0.226	0.194	0.478	188.496	0.675	0.268	0.012	0.073
MZF1	489.052	0.680	0.310	0.028	0.075	489.052	0.663	0.310	0.032	0.322	489.052	-0.017	0.358	0.962	0.982
ZNF45	175.720	-0.771	0.185	0.000	0.000	175.720	-0.012	0.177	0.946	0.973	175.720	0.759	0.212	0.000	0.006
ZBTB25	806.094	0.077	0.151	0.611	0.723	806.094	0.046	0.151	0.761	0.873	806.094	-0.031	0.175	0.859	0.932
CA2	39.143	-0.761	0.448	0.089	0.180	39.143	-0.247	0.432	0.567	0.754	39.143	0.514	0.516	0.320	0.555
ZNF56	11.168	-0.273	0.497	0.583	0.700	11.168	0.211	0.461	0.647	0.808	11.168	0.484	0.563	0.390	0.619
CA3	7.336	0.873	0.828	0.292	0.430	7.336	1.118	0.809	0.167	0.457	7.336	0.245	0.922	0.791	0.896
ZNF70	54.337	-0.091	0.251	0.717	0.807	54.337	-0.129	0.248	0.604	0.781	54.337	-0.038	0.292	0.897	0.951
ZNF74	169.227	-0.245	0.205	0.232	0.365	169.227	-0.079	0.203	0.697	0.837	169.227	0.166	0.237	0.484	0.696
ZNF75D	72.640	0.235	0.364	0.519	0.646	72.640	0.362	0.362	0.317	0.579	72.640	0.127	0.419	0.762	0.879
ZNF75A	129.960	-0.244	0.160	0.127	0.234	129.960	-0.252	0.158	0.110	0.404	129.960	-0.008	0.186	0.967	0.984
ZNF76	358.180	0.244	0.192	0.204	0.331	358.180	0.161	0.192	0.402	0.644	358.180	-0.083	0.221	0.707	0.848
ZNF79	54.744	-0.260	0.276	0.345	0.485	54.744	-0.734	0.282	0.009	0.297	54.744	-0.474	0.329	0.150	0.359
ZNF84	417.274	0.120	0.255	0.637	0.745	417.274	0.206	0.254	0.418	0.656	417.274	0.086	0.294	0.771	0.883
ZNF221	20.003	0.364	0.460	0.429	0.565	20.003	-0.211	0.470	0.653	0.811	20.003	-0.576	0.540	0.286	0.521
ZNF85	34.399	-0.499	0.502	0.320	0.460	34.399	-0.395	0.498	0.427	0.663	34.399	0.104	0.582	0.859	0.932
ZNF91	9.446	0.554	0.521	0.288	0.426	9.446	0.874	0.497	0.079	0.370	9.446	0.320	0.574	0.577	0.762
CA8	5.069	-1.175	1.198	0.327	0.467	5.069	-1.172	1.174	0.318	0.580	5.069	0.002	1.437	0.999	0.999
ZNF222	43.123	0.237	0.278	0.394	0.533	43.123	-0.079	0.281	0.778	0.883	43.123	-0.316	0.325	0.331	0.565
ZNF121	415.939	0.653	0.257	0.011	0.036	415.939	0.071	0.258	0.782	0.885	415.939	-0.582	0.297	0.050	0.183
ZNF124	45.857	-1.056	0.412	0.010	0.035	45.857	-0.650	0.402	0.106	0.398	45.857	0.406	0.478	0.396	0.624
CA9	4023.434	0.478	1.130	0.672	0.772	4023.434	1.137	1.130	0.315	0.577	4023.434	0.658	1.305	0.614	0.787
MKRN3	98.130	-0.580	0.212	0.006	0.023	98.130	-0.060	0.202	0.768	0.877	98.130	0.521	0.244	0.033	0.139
RELL1	169.352	0.377	0.229	0.099	0.194	169.352	-0.405	0.233	0.082	0.373	169.352	-0.782	0.267	0.003	0.032
ZNF131	883.111	-0.519	0.135	0.000	0.001	883.111	-0.117	0.134	0.381	0.627	883.111	0.402	0.156	0.010	0.066
ZNF132	15.097	-0.861	0.461	0.062	0.136	15.097	-0.314	0.430	0.466	0.690	15.097	0.547	0.532	0.303	0.538
ZNF133	252.411	0.073	0.185	0.694	0.789	252.411	0.155	0.184	0.400	0.643	252.411	0.082	0.214	0.702	0.845
ZNF134	280.547	-0.440	0.181	0.015	0.046	280.547	0.027	0.177	0.877	0.937	280.547	0.468	0.208	0.025	0.116
ZNF136	75.141	-0.109	0.228	0.632	0.740	75.141	0.061	0.223	0.784	0.887	75.141	0.170	0.263	0.517	0.720
ZNF138	26.298	0.904	0.329	0.006	0.023	26.298	0.479	0.335	0.152	0.445	26.298	-0.425	0.377	0.259	0.494
ZNF140	177.305	0.002	0.192	0.991	0.995	177.305	-0.106	0.192	0.579	0.764	177.305	-0.108	0.223	0.627	0.796
CA11	282.927	1.199	0.498	0.016	0.049	282.927	0.854	0.498	0.087	0.380	282.927	-0.345	0.574	0.547	0.742
ZNF142	320.688	-0.935	0.286	0.001	0.006	320.688	-0.691	0.285	0.015	0.306	320.688	0.244	0.331	0.461	0.678
ZNF143	360.901	-0.116	0.229	0.614	0.725	360.901	-0.428	0.230	0.063	0.350	360.901	-0.312	0.266	0.242	0.472
PCGF2	2006.052	0.761	0.233	0.001	0.006	2006.052	0.244	0.233	0.296	0.564	2006.052	-0.517	0.269	0.055	0.194
ZBTB16	2400.190	-0.297	0.384	0.439	0.575	2400.190	0.499	0.384	0.194	0.478	2400.190	0.796	0.443	0.072	0.230
ZNF146	1857.845	-0.830	0.172	0.000	0.000	1857.845	-0.364	0.171	0.033	0.322	1857.845	0.465	0.198	0.019	0.098
TRIM25	3812.366	-0.682	0.175	0.000	0.001	3812.366	0.037	0.175	0.831	0.913	3812.366	0.719	0.202	0.000	0.006
ZNF148	940.329	-0.785	0.186	0.000	0.000	940.329	-0.207	0.185	0.263	0.535	940.329	0.578	0.215	0.007	0.052
ZBTB17	653.145	0.304	0.134	0.024	0.065	653.145	-0.018	0.135	0.892	0.946	653.145	-0.322	0.156	0.038	0.155

CA12	10473.139	1.024	0.341	0.003	0.012	10473.139	0.620	0.341	0.069	0.357	10473.139	-0.403	0.394	0.306	0.542
ZNF155	38.722	0.142	0.325	0.663	0.765	38.722	0.025	0.324	0.938	0.968	38.722	-0.117	0.377	0.757	0.876
VEZF1	1320.904	-0.889	0.258	0.001	0.004	1320.904	-0.039	0.257	0.879	0.939	1320.904	0.850	0.298	0.004	0.037
ZNF165	27.884	0.717	0.387	0.064	0.139	27.884	0.429	0.389	0.270	0.541	27.884	-0.288	0.444	0.517	0.720
TRIM26	4462.323	-0.177	0.278	0.525	0.651	4462.323	-0.578	0.278	0.038	0.327	4462.323	-0.401	0.321	0.212	0.437
ZNF174	151.201	0.019	0.178	0.914	0.946	151.201	0.109	0.176	0.534	0.733	151.201	0.090	0.205	0.661	0.819
ZNF175	108.112	-0.149	0.315	0.637	0.744	108.112	0.182	0.311	0.558	0.748	108.112	0.331	0.363	0.361	0.594
ZNF180	90.139	-0.298	0.233	0.201	0.328	90.139	-0.172	0.229	0.452	0.680	90.139	0.126	0.270	0.642	0.806
RNF113A	449.350	0.638	0.245	0.009	0.032	449.350	0.328	0.246	0.182	0.469	449.350	-0.310	0.283	0.274	0.509
ZNF184	157.069	-0.655	0.243	0.007	0.026	157.069	-0.060	0.238	0.800	0.896	157.069	0.595	0.280	0.034	0.142
ZNF185	40.155	-0.981	0.324	0.002	0.011	40.155	0.159	0.295	0.590	0.771	40.155	1.141	0.366	0.002	0.020
ZSCAN26	161.270	-0.192	0.196	0.329	0.469	161.270	-0.089	0.194	0.648	0.808	161.270	0.103	0.227	0.650	0.811
ZNF189	250.419	0.035	0.236	0.883	0.924	250.419	0.124	0.235	0.597	0.776	250.419	0.090	0.272	0.742	0.867
ZKSCAN8	496.437	-0.458	0.268	0.088	0.177	496.437	-0.012	0.267	0.964	0.981	496.437	0.446	0.309	0.150	0.358
ZSCAN9	112.366	0.255	0.221	0.247	0.381	112.366	-0.295	0.225	0.190	0.476	112.366	-0.550	0.259	0.034	0.142
ZNF195	212.377	0.391	0.211	0.064	0.140	212.377	-0.243	0.214	0.256	0.529	212.377	-0.634	0.246	0.010	0.066
ZMYM2	747.665	-0.519	0.206	0.012	0.038	747.665	0.068	0.204	0.741	0.862	747.665	0.587	0.237	0.013	0.079
ZNF200	121.856	-0.776	0.233	0.001	0.005	121.856	-0.431	0.227	0.057	0.347	121.856	0.345	0.270	0.201	0.425
ZNF202	344.590	-0.951	0.344	0.006	0.022	344.590	-0.602	0.343	0.079	0.370	344.590	0.349	0.398	0.380	0.610
ZNF205	418.337	0.458	0.228	0.045	0.106	418.337	0.429	0.228	0.060	0.350	418.337	-0.029	0.263	0.911	0.959
ZNF207	2467.629	-0.352	0.175	0.044	0.105	2467.629	-0.355	0.175	0.042	0.330	2467.629	-0.003	0.202	0.990	0.994
ZNF213	312.295	-0.187	0.128	0.144	0.257	312.295	0.028	0.126	0.825	0.909	312.295	0.215	0.148	0.146	0.353
ZNF214	35.546	-0.083	0.331	0.803	0.870	35.546	0.107	0.324	0.742	0.862	35.546	0.189	0.381	0.619	0.792
ZNF215	207.872	-0.690	0.223	0.002	0.010	207.872	-0.382	0.220	0.083	0.374	207.872	0.308	0.258	0.233	0.462
ZFAND5	1761.702	-0.067	0.116	0.562	0.683	1761.702	-0.154	0.116	0.184	0.471	1761.702	-0.087	0.134	0.517	0.721
ZNF217	617.294	-0.161	0.144	0.262	0.397	617.294	-0.188	0.143	0.191	0.476	617.294	-0.026	0.166	0.875	0.940
ZNF223	10.503	-0.550	0.617	0.372	0.512	10.503	0.119	0.579	0.837	0.916	10.503	0.669	0.703	0.341	0.575
ZNF224	171.718	0.867	0.300	0.004	0.016	171.718	0.564	0.301	0.061	0.350	171.718	-0.304	0.346	0.381	0.610
ZNF225	66.992	-0.108	0.287	0.706	0.798	66.992	0.189	0.281	0.501	0.714	66.992	0.297	0.330	0.367	0.600
ZNF226	146.059	0.191	0.218	0.381	0.521	146.059	0.488	0.215	0.023	0.316	146.059	0.297	0.250	0.236	0.465
ZNF227	285.319	-0.599	0.228	0.009	0.031	285.319	0.065	0.225	0.773	0.879	285.319	0.664	0.263	0.012	0.072
ZNF112	78.378	-0.350	0.277	0.207	0.336	78.378	0.432	0.267	0.106	0.398	78.378	0.782	0.316	0.013	0.079
ZNF230	62.402	0.177	0.242	0.465	0.600	62.402	0.004	0.242	0.986	0.992	62.402	-0.172	0.281	0.539	0.736
ZNF232	28.675	-0.173	0.385	0.654	0.758	28.675	0.224	0.372	0.548	0.742	28.675	0.397	0.441	0.368	0.600
ZNF236	193.674	-0.399	0.186	0.032	0.082	193.674	-0.091	0.182	0.619	0.790	193.674	0.309	0.215	0.150	0.360
SLC30A1	1541.368	-0.997	0.324	0.002	0.010	1541.368	-0.631	0.323	0.051	0.338	1541.368	0.365	0.374	0.328	0.562
SLC30A3	42.397	1.224	0.402	0.002	0.011	42.397	0.594	0.408	0.146	0.438	42.397	-0.630	0.462	0.173	0.390
SLC30A4	60.091	0.807	0.262	0.002	0.010	60.091	0.346	0.266	0.193	0.478	60.091	-0.461	0.302	0.127	0.322
ZP3	147.269	0.944	0.430	0.028	0.075	147.269	0.870	0.430	0.043	0.331	147.269	-0.074	0.495	0.881	0.943
MAP3K12	752.716	1.061	0.239	0.000	0.000	752.716	0.438	0.240	0.068	0.356	752.716	-0.623	0.276	0.024	0.115
ZXDA	102.959	-1.076	0.363	0.003	0.014	102.959	0.111	0.352	0.752	0.867	102.959	1.187	0.417	0.004	0.037
ZYX	4013.767	0.377	0.163	0.020	0.059	4013.767	0.038	0.163	0.815	0.903	4013.767	-0.339	0.188	0.071	0.228
LUZP1	1718.510	-0.482	0.235	0.040	0.098	1718.510	-0.287	0.235	0.221	0.501	1718.510	0.195	0.271	0.472	0.686
PRDM2	503.838	-0.622	0.179	0.001	0.004	503.838	-0.130	0.177	0.463	0.689	503.838	0.492	0.207	0.017	0.094
DDR1	10828.528	0.887	0.407	0.029	0.077	10828.528	0.553	0.407	0.174	0.463	10828.528	-0.333	0.470	0.478	0.691
DNALI1	30.145	1.346	0.452	0.003	0.013	30.145	-0.065	0.476	0.891	0.945	30.145	-1.411	0.533	0.008	0.057
PTP4A1	3141.764	-0.586	0.197	0.003	0.013	3141.764	-0.422	0.197	0.032	0.322	3141.764	0.165	0.228	0.470	0.684
LRP8	561.748	0.233	0.399	0.559	0.681	561.748	-1.026	0.400	0.010	0.299	561.748	-1.259	0.462	0.006	0.049
LAPTM5	4208.429	-0.671	1.007	0.505	0.635	4208.429	0.873	1.007	0.386	0.631	4208.429	1.545	1.162	0.184	0.403
TVP23A	186.259	0.955	0.260	0.000	0.002	186.259	-0.201	0.265	0.447	0.677	186.259	-1.157	0.303	0.000	0.003
SNORD3A	18.523	0.389	0.714	0.586	0.703	18.523	-1.752	0.782	0.025	0.316	18.523	-2.140	0.884	0.015	0.087
SNORD3C	9.718	0.623	1.006	0.536	0.661	9.718	-2.374	1.120	0.034	0.322	9.718	-2.997	1.259	0.017	0.093
SNORD3D	6.857	0.458	0.939	0.625	0.735	6.857	-0.874	0.995	0.379	0.626	6.857	-1.333	1.131	0.239	0.469
CSDE1	17642.991	-0.601	0.188	0.001	0.008	17642.991	0.124	0.188	0.509	0.718	17642.991	0.725	0.218	0.001	0.012
EVI5	306.735	-0.229	0.165	0.165	0.285	306.735	-0.182	0.164	0.267	0.538	306.735	0.047	0.191	0.806	0.903
DAP3	2072.596	-0.028	0.137	0.836	0.892	2072.596	-0.104	0.137	0.448	0.677	2072.596	-0.076	0.159	0.634	0.801
CACNB1	322.956	0.749	0.188	0.000	0.001	322.956	0.181	0.190	0.341	0.599	322.956	-0.568	0.218	0.009	0.061
BTG2	370.906	0.185	0.384	0.629	0.739	370.906	0.399	0.383	0.298	0.565	370.906	0.214	0.443	0.629	0.798
CACNB3	325.443	1.038	0.349	0.003	0.013	325.443	0.611	0.349	0.080	0.371	325.443	-0.426	0.402	0.289	0.524
ALMS1	263.351	-0.366	0.229	0.111	0.212	263.351	-0.248	0.228	0.277	0.547	263.351	0.118	0.265	0.658	0.817
MOGS	1414.668	-0.044	0.142	0.757	0.838	1414.668	-0.044	0.142	0.754	0.868	1414.668	0.000	0.164	0.998	0.999
RNF103	637.411	-0.011	0.167	0.946	0.966	637.411	0.101	0.166	0.541	0.737	637.411	0.113	0.192	0.557	0.749
TUBA1A	2311.611	0.837	0.147	0.000	0.000	2311.611	-0.070	0.147	0.633	0.800	2311.611	-0.908	0.170	0.000	0.000
PAX8	46.083	0.829	0.364	0.023	0.064	46.083	0.510	0.369	0.167	0.457	46.083	-0.320	0.415	0.441	0.661
CACNB4	247.660	0.058	0.401	0.886	0.926	247.660	0.593	0.399	0.137	0.428	247.660	0.535	0.462	0.246	0.478
IL1R2	14.012	0.585	0.636	0.358	0.498	14.012	0.568	0.628	0.366	0.617	14.012	-0.017	0.720	0.981	0.990
MALL	7.972	0.342	0.559	0.541	0.666	7.972	0.366	0.548	0.504	0.715	7.972	0.024	0.637	0.969	0.985

FZD5	93.402	-0.252	0.210	0.229	0.361	93.402	-0.517	0.211	0.014	0.306	93.402	-0.265	0.248	0.285	0.520
BRPF1	491.896	-0.536	0.245	0.029	0.076	491.896	-0.489	0.245	0.046	0.334	491.896	0.047	0.284	0.869	0.937
IFRD2	2214.210	-0.736	0.307	0.016	0.050	2214.210	-0.513	0.307	0.095	0.389	2214.210	0.223	0.354	0.529	0.729
MAPKAPK3	1681.885	-0.245	0.198	0.216	0.345	1681.885	-0.304	0.198	0.124	0.416	1681.885	-0.059	0.229	0.796	0.898
SEMA3B	240.674	1.815	0.589	0.002	0.010	240.674	0.999	0.590	0.091	0.385	240.674	-0.816	0.680	0.230	0.459
SLMAP	1457.217	-0.800	0.212	0.000	0.001	1457.217	-0.359	0.212	0.090	0.384	1457.217	0.441	0.245	0.072	0.229
MANF	1260.402	-0.214	0.406	0.599	0.712	1260.402	-0.832	0.406	0.041	0.328	1260.402	-0.618	0.469	0.188	0.408
USP7	3158.681	-0.659	0.172	0.000	0.001	3158.681	-0.386	0.171	0.024	0.316	3158.681	0.272	0.198	0.170	0.385
RAB7A	7560.440	0.210	0.180	0.241	0.374	7560.440	-0.353	0.180	0.049	0.337	7560.440	-0.564	0.207	0.007	0.049
SLC25A20	147.028	0.181	0.302	0.549	0.672	147.028	-0.314	0.304	0.302	0.568	147.028	-0.495	0.351	0.159	0.371
KCNAB1	16.906	0.540	0.433	0.212	0.342	16.906	-0.050	0.446	0.911	0.956	16.906	-0.590	0.509	0.246	0.477
SLBP	1001.639	-0.354	0.251	0.157	0.274	1001.639	-0.554	0.251	0.027	0.319	1001.639	-0.200	0.290	0.490	0.700
CRELD1	188.090	0.874	0.302	0.004	0.016	188.090	0.392	0.303	0.196	0.480	188.090	-0.482	0.348	0.166	0.381
MRPL57	512.743	0.065	0.227	0.775	0.850	512.743	-0.193	0.228	0.396	0.640	512.743	-0.258	0.263	0.327	0.561
OTUB2	110.162	-0.045	0.321	0.889	0.928	110.162	0.036	0.319	0.910	0.955	110.162	0.081	0.371	0.828	0.915
PCYOX1L	176.949	0.877	0.183	0.000	0.000	176.949	0.025	0.187	0.893	0.946	176.949	-0.852	0.213	0.000	0.002
YIPF2	1354.745	0.767	0.182	0.000	0.000	1354.745	0.101	0.182	0.579	0.763	1354.745	-0.665	0.210	0.002	0.018
PRR14	576.494	0.366	0.183	0.045	0.106	576.494	0.333	0.182	0.068	0.356	576.494	-0.033	0.211	0.875	0.940
HROB	165.291	-0.145	0.199	0.466	0.600	165.291	0.173	0.195	0.374	0.622	165.291	0.318	0.228	0.164	0.378
CYREN	401.623	-0.671	0.249	0.007	0.026	401.623	-0.614	0.249	0.014	0.306	401.623	0.056	0.289	0.845	0.925
LRFN4	1152.063	0.315	0.226	0.164	0.284	1152.063	-0.214	0.227	0.346	0.602	1152.063	-0.529	0.262	0.043	0.167
CAD	1397.128	-0.609	0.309	0.049	0.113	1397.128	-0.657	0.309	0.033	0.322	1397.128	-0.048	0.357	0.892	0.949
AUNIP	241.322	-0.651	0.230	0.005	0.019	241.322	-0.534	0.229	0.020	0.306	241.322	0.117	0.267	0.661	0.819
VKORC1	2251.367	0.477	0.322	0.139	0.249	2251.367	0.615	0.322	0.056	0.344	2251.367	0.138	0.372	0.711	0.850
TRIR	3840.475	0.623	0.224	0.005	0.021	3840.475	0.183	0.224	0.413	0.652	3840.475	-0.440	0.258	0.088	0.260
MIS12	161.746	-0.574	0.220	0.009	0.032	161.746	-0.140	0.215	0.516	0.721	161.746	0.434	0.254	0.088	0.258
CUEDC2	1350.207	0.471	0.178	0.008	0.029	1350.207	0.199	0.178	0.264	0.537	1350.207	-0.272	0.206	0.186	0.405
SCNM1	195.079	-0.005	0.237	0.983	0.990	195.079	-0.111	0.237	0.638	0.803	195.079	-0.106	0.275	0.699	0.843
METRN	1059.600	0.724	0.235	0.002	0.010	1059.600	-0.067	0.235	0.777	0.881	1059.600	-0.790	0.271	0.004	0.032
DBNDD1	163.327	0.946	0.415	0.023	0.063	163.327	0.641	0.416	0.123	0.414	163.327	-0.305	0.478	0.524	0.726
SLX1B	93.820	0.437	0.269	0.105	0.202	93.820	-0.019	0.272	0.943	0.971	93.820	-0.456	0.313	0.145	0.352
DDX50	1522.533	-0.565	0.155	0.000	0.002	1522.533	0.038	0.154	0.804	0.897	1522.533	0.603	0.178	0.001	0.010
CAMKV	16.016	0.030	0.816	0.970	0.982	16.016	1.306	0.757	0.084	0.376	16.016	1.276	0.903	0.157	0.369
LINC01260	11.989	-0.915	1.067	0.391	0.530	11.989	0.506	1.034	0.625	0.794	11.989	1.421	1.220	0.244	0.475
DDA1	1156.825	0.273	0.182	0.134	0.244	1156.825	-0.211	0.183	0.249	0.523	1156.825	-0.483	0.211	0.022	0.107
GGCT	1137.609	0.278	0.233	0.231	0.364	1137.609	-0.195	0.233	0.402	0.644	1137.609	-0.474	0.269	0.078	0.241
GID4	346.672	-0.149	0.212	0.480	0.614	346.672	0.362	0.209	0.084	0.376	346.672	0.511	0.243	0.036	0.147
CENPM	275.103	-0.056	0.416	0.893	0.931	275.103	-0.644	0.417	0.123	0.414	275.103	-0.588	0.482	0.223	0.450
C7orf25	134.355	0.321	0.280	0.252	0.386	134.355	-0.461	0.285	0.105	0.398	134.355	-0.783	0.328	0.017	0.092
TMEM106C	775.383	0.006	0.141	0.965	0.979	775.383	0.004	0.141	0.978	0.988	775.383	-0.002	0.163	0.988	0.994
NUP37	526.734	-0.082	0.180	0.648	0.754	526.734	-0.209	0.180	0.245	0.520	526.734	-0.127	0.208	0.542	0.739
AHNAK	20100.181	-0.427	0.290	0.141	0.253	20100.181	0.263	0.290	0.366	0.617	20100.181	0.690	0.335	0.040	0.158
ZNF655	486.618	-0.416	0.139	0.003	0.013	486.618	-0.047	0.137	0.734	0.858	486.618	0.370	0.160	0.021	0.105
SPATA5L1	341.921	-0.422	0.218	0.054	0.122	341.921	-0.313	0.217	0.150	0.442	341.921	0.109	0.253	0.667	0.823
PDCL3	322.142	0.043	0.230	0.853	0.904	322.142	-0.386	0.231	0.095	0.389	322.142	-0.428	0.267	0.109	0.294
ERI3	1199.513	-0.048	0.185	0.794	0.864	1199.513	-0.184	0.185	0.321	0.582	1199.513	-0.135	0.214	0.527	0.728
C7orf26	469.343	-0.442	0.310	0.153	0.268	469.343	-0.535	0.309	0.084	0.376	469.343	-0.092	0.358	0.797	0.898
NABP2	834.238	-0.387	0.181	0.033	0.084	834.238	-0.191	0.180	0.290	0.558	834.238	0.196	0.209	0.348	0.582
KXD1	1646.159	0.060	0.114	0.598	0.712	1646.159	0.043	0.114	0.706	0.842	1646.159	-0.017	0.132	0.896	0.951
PVRIG	12.903	1.172	0.726	0.106	0.205	12.903	0.971	0.726	0.181	0.469	12.903	-0.202	0.826	0.807	0.903
ZFYVE21	561.606	0.627	0.134	0.000	0.000	561.606	0.004	0.135	0.974	0.986	561.606	-0.623	0.155	0.000	0.002
DDX54	2924.742	-0.204	0.131	0.118	0.222	2924.742	-0.041	0.130	0.751	0.867	2924.742	0.163	0.151	0.281	0.515
TMEM38A	134.224	1.295	0.258	0.000	0.000	134.224	0.785	0.261	0.003	0.297	134.224	-0.510	0.293	0.082	0.248
TSEN34	4269.300	0.617	0.189	0.001	0.006	4269.300	0.237	0.189	0.208	0.490	4269.300	-0.380	0.218	0.081	0.247
KCTD15	9.539	1.125	0.484	0.020	0.058	9.539	0.336	0.512	0.512	0.719	9.539	-0.789	0.562	0.160	0.373
SECISBP2	340.181	-0.440	0.200	0.028	0.075	340.181	-0.268	0.199	0.179	0.467	340.181	0.172	0.232	0.458	0.676
REEP5	1594.742	0.101	0.096	0.289	0.427	1594.742	-0.055	0.096	0.565	0.752	1594.742	-0.156	0.111	0.157	0.369
NOC4L	556.360	-0.259	0.369	0.482	0.616	556.360	-0.822	0.370	0.026	0.318	556.360	-0.562	0.427	0.188	0.409
ALG8	328.049	0.113	0.188	0.550	0.673	328.049	-0.353	0.190	0.063	0.350	328.049	-0.466	0.219	0.034	0.142
PRRG4	247.191	0.788	0.173	0.000	0.000	247.191	-0.286	0.181	0.114	0.405	247.191	-1.073	0.205	0.000	0.000
ASPSCR1	723.822	0.677	0.210	0.001	0.007	723.822	0.158	0.210	0.452	0.680	723.822	-0.519	0.242	0.032	0.138
TMEM223	472.501	0.448	0.218	0.040	0.098	472.501	0.000	0.219	0.999	1.000	472.501	-0.448	0.253	0.076	0.238
ATG9A	1160.794	-0.478	0.277	0.085	0.172	1160.794	-0.090	0.276	0.744	0.863	1160.794	0.387	0.320	0.226	0.454
METTL16	365.058	-0.850	0.202	0.000	0.000	365.058	-0.335	0.199	0.092	0.387	365.058	0.515	0.233	0.027	0.123
FTO	609.816	-0.183	0.127	0.150	0.264	609.816	0.062	0.126	0.626	0.794	609.816	0.245	0.147	0.096	0.272
POGLUT2	304.351	0.095	0.203	0.641	0.748	304.351	-0.249	0.204	0.222	0.501	304.351	-0.344	0.236	0.144	0.351
ELOVL6	1146.276	0.680	0.080	0.000	0.000	1146.276	-0.162	0.082	0.048	0.337	1146.276	-0.842	0.094	0.000	0.000

FASTKD3	188.216	-0.626	0.251	0.013	0.041	188.216	-0.397	0.249	0.111	0.404	188.216	0.230	0.291	0.430	0.652
TMEM109	2330.688	-0.006	0.221	0.980	0.988	2330.688	-0.463	0.222	0.037	0.324	2330.688	-0.458	0.256	0.074	0.233
C2orf49	436.978	-0.208	0.128	0.104	0.201	436.978	-0.023	0.126	0.858	0.927	436.978	0.185	0.147	0.210	0.435
DSCC1	171.788	-1.235	0.357	0.001	0.004	171.788	-0.439	0.352	0.212	0.492	171.788	0.796	0.411	0.053	0.190
DCTPP1	776.994	-0.920	0.383	0.016	0.049	776.994	-1.013	0.383	0.008	0.297	776.994	-0.093	0.443	0.835	0.920
C1orf50	164.694	-0.105	0.191	0.582	0.699	164.694	0.126	0.188	0.502	0.714	164.694	0.231	0.220	0.293	0.528
CCDC86	1409.837	-0.695	0.399	0.082	0.168	1409.837	-0.856	0.399	0.032	0.322	1409.837	-0.162	0.461	0.726	0.858
LBHD1	380.778	0.241	0.207	0.243	0.377	380.778	-0.140	0.208	0.502	0.714	380.778	-0.381	0.240	0.112	0.299
MLPH	16.908	0.276	0.371	0.457	0.592	16.908	0.171	0.368	0.643	0.805	16.908	-0.105	0.428	0.806	0.903
WDR77	714.016	-0.549	0.416	0.186	0.310	714.016	-1.132	0.416	0.007	0.297	714.016	-0.582	0.481	0.226	0.454
SLC25A23	927.395	0.748	0.366	0.041	0.100	927.395	0.453	0.366	0.216	0.495	927.395	-0.295	0.423	0.485	0.697
SMIM7	833.102	-0.205	0.132	0.120	0.224	833.102	-0.100	0.132	0.449	0.678	833.102	0.106	0.153	0.489	0.699
ALG12	525.439	0.228	0.135	0.089	0.180	525.439	0.278	0.134	0.038	0.327	525.439	0.049	0.155	0.751	0.872
ZNF426	196.161	0.053	0.202	0.791	0.862	196.161	0.438	0.198	0.027	0.321	196.161	0.384	0.231	0.097	0.274
TMUB2	504.770	0.263	0.162	0.106	0.204	504.770	0.275	0.162	0.089	0.382	504.770	0.013	0.187	0.947	0.975
TRAPPC6A	284.812	1.225	0.565	0.030	0.079	284.812	0.638	0.566	0.260	0.532	284.812	-0.587	0.653	0.368	0.601
METTL22	194.614	-0.274	0.147	0.062	0.137	194.614	0.058	0.143	0.683	0.829	194.614	0.333	0.169	0.049	0.181
CARD14	74.698	1.604	0.468	0.001	0.004	74.698	0.523	0.474	0.269	0.541	74.698	-1.080	0.540	0.045	0.172
CHAC1	361.367	-4.007	0.734	0.000	0.000	361.367	-2.003	0.724	0.006	0.297	361.367	2.004	0.846	0.018	0.094
C9orf16	713.522	1.408	0.326	0.000	0.000	713.522	0.480	0.327	0.142	0.433	713.522	-0.929	0.376	0.014	0.080
UQCC3	436.795	0.113	0.251	0.653	0.757	436.795	-0.168	0.251	0.504	0.715	436.795	-0.281	0.290	0.334	0.568
C11orf49	293.772	0.514	0.221	0.020	0.058	293.772	0.323	0.221	0.144	0.435	293.772	-0.190	0.255	0.455	0.673
C1orf116	8.645	0.322	0.696	0.643	0.749	8.645	0.068	0.699	0.922	0.961	8.645	-0.254	0.807	0.753	0.874
TAF1D	2944.067	-0.022	0.244	0.927	0.954	2944.067	0.288	0.244	0.238	0.515	2944.067	0.310	0.281	0.271	0.506
RNF26	2199.909	-0.023	0.219	0.917	0.948	2199.909	0.278	0.219	0.203	0.486	2199.909	0.301	0.253	0.233	0.462
MAPKAP1	1737.658	-0.157	0.120	0.192	0.318	1737.658	-0.252	0.120	0.036	0.324	1737.658	-0.095	0.139	0.495	0.703
DEK	3420.762	-0.590	0.180	0.001	0.006	3420.762	-0.401	0.180	0.026	0.316	3420.762	0.189	0.208	0.365	0.597
DHX58	194.408	1.885	0.360	0.000	0.000	194.408	0.543	0.363	0.134	0.426	194.408	-1.341	0.415	0.001	0.015
NDUFAF5	145.213	-0.251	0.295	0.394	0.532	145.213	-0.521	0.295	0.078	0.369	145.213	-0.269	0.343	0.432	0.654
TMEM185B	385.372	0.040	0.182	0.824	0.885	385.372	-0.258	0.183	0.159	0.451	385.372	-0.298	0.211	0.159	0.371
APOO	192.983	0.173	0.291	0.552	0.674	192.983	-0.441	0.293	0.133	0.424	192.983	-0.614	0.339	0.070	0.225
RETREG2	1902.993	0.274	0.289	0.342	0.483	1902.993	0.469	0.288	0.104	0.397	1902.993	0.195	0.333	0.559	0.750
DERL1	1434.862	-0.343	0.138	0.013	0.042	1434.862	-0.099	0.138	0.473	0.695	1434.862	0.244	0.160	0.127	0.322
CCDC28B	256.980	1.190	0.243	0.000	0.000	256.980	0.075	0.246	0.760	0.872	256.980	-1.115	0.281	0.000	0.002
PHF23	584.307	-0.211	0.118	0.073	0.154	584.307	-0.152	0.117	0.194	0.478	584.307	0.059	0.137	0.664	0.821
MBOAT7	7046.179	0.967	0.140	0.000	0.000	7046.179	-0.037	0.140	0.790	0.890	7046.179	-1.004	0.161	0.000	0.000
PPDPF	1190.356	0.826	0.255	0.001	0.007	1190.356	0.382	0.255	0.134	0.426	1190.356	-0.443	0.295	0.132	0.331
CHCHD7	616.532	0.076	0.138	0.581	0.698	616.532	-0.026	0.138	0.853	0.925	616.532	-0.102	0.160	0.524	0.725
FKRP	1148.688	-0.481	0.184	0.009	0.031	1148.688	-0.086	0.183	0.640	0.804	1148.688	0.395	0.212	0.063	0.211
MMP28	227.470	0.260	0.865	0.764	0.843	227.470	1.326	0.864	0.125	0.416	227.470	1.067	0.998	0.285	0.520
ZSCAN5A	24.774	-0.157	0.363	0.665	0.766	24.774	-0.028	0.354	0.936	0.968	24.774	0.129	0.420	0.758	0.877
ALDH5A1	184.377	0.346	0.397	0.384	0.523	184.377	0.444	0.396	0.262	0.535	184.377	0.098	0.458	0.830	0.917
FA2H	14.047	0.453	0.710	0.523	0.651	14.047	0.969	0.683	0.156	0.448	14.047	0.516	0.794	0.516	0.720
GDPD3	21.427	1.105	0.433	0.011	0.036	21.427	0.559	0.442	0.205	0.488	21.427	-0.546	0.496	0.272	0.506
DHRS11	239.015	-0.053	0.363	0.884	0.925	239.015	-0.476	0.364	0.191	0.476	239.015	-0.423	0.421	0.314	0.550
TNIP2	1371.465	0.678	0.263	0.010	0.034	1371.465	-0.375	0.264	0.155	0.447	1371.465	-1.054	0.304	0.001	0.008
PLEKHF1	108.840	2.108	0.258	0.000	0.000	108.840	0.507	0.271	0.061	0.350	108.840	-1.601	0.298	0.000	0.000
MFSD11	1171.181	-0.805	0.412	0.050	0.116	1171.181	-1.140	0.412	0.006	0.297	1171.181	-0.335	0.476	0.481	0.694
GNPTAB	747.675	0.119	0.225	0.596	0.711	747.675	-0.353	0.226	0.119	0.411	747.675	-0.472	0.261	0.070	0.226
NOL12	54.707	-0.308	0.454	0.497	0.628	54.707	-0.846	0.458	0.065	0.353	54.707	-0.538	0.531	0.311	0.546
PRRC2A	31147.959	-0.474	0.117	0.000	0.001	31147.959	-0.068	0.117	0.560	0.749	31147.959	0.406	0.135	0.003	0.026
TMEM243	269.441	-0.033	0.172	0.849	0.901	269.441	-0.213	0.172	0.215	0.494	269.441	-0.180	0.200	0.367	0.599
LENG1	792.070	-0.023	0.370	0.951	0.970	792.070	-0.293	0.370	0.428	0.663	792.070	-0.270	0.427	0.528	0.728
C1orf35	331.030	0.595	0.278	0.032	0.083	331.030	-0.164	0.280	0.559	0.748	331.030	-0.759	0.322	0.019	0.097
BAG6	20422.257	-0.043	0.089	0.630	0.739	20422.257	0.148	0.089	0.097	0.392	20422.257	0.191	0.103	0.064	0.212
PRR15L	20.896	0.438	0.383	0.252	0.386	20.896	0.568	0.374	0.129	0.421	20.896	0.129	0.433	0.765	0.881
RBM42	1615.724	0.285	0.242	0.240	0.373	1615.724	-0.315	0.243	0.194	0.478	1615.724	-0.599	0.280	0.032	0.138
CENPO	880.563	-0.045	0.216	0.835	0.891	880.563	-0.456	0.216	0.035	0.322	880.563	-0.411	0.250	0.100	0.280
C19orf57	42.205	-0.727	0.311	0.020	0.057	42.205	-0.355	0.299	0.235	0.513	42.205	0.372	0.361	0.302	0.537
CRELD2	512.526	0.548	0.366	0.134	0.244	512.526	-0.701	0.368	0.057	0.344	512.526	-1.249	0.424	0.003	0.030
ZNF343	264.326	-0.457	0.158	0.004	0.016	264.326	-0.045	0.154	0.768	0.877	264.326	0.412	0.182	0.024	0.113
FBXL15	479.272	0.183	0.196	0.351	0.491	479.272	0.005	0.196	0.980	0.989	479.272	-0.178	0.227	0.432	0.654
ZNF576	226.656	-0.480	0.159	0.003	0.012	226.656	-0.133	0.155	0.389	0.635	226.656	0.347	0.184	0.059	0.203
THTPA	177.116	0.042	0.155	0.789	0.861	177.116	0.002	0.154	0.991	0.995	177.116	-0.040	0.180	0.825	0.914
GPANK1	2278.272	-0.098	0.196	0.616	0.727	2278.272	-0.009	0.196	0.962	0.980	2278.272	0.089	0.227	0.695	0.840
EFHD2	3691.968	0.510	0.161	0.001	0.008	3691.968	-0.095	0.161	0.553	0.745	3691.968	-0.605	0.186	0.001	0.014
TTPAL	389.851	-0.319	0.209	0.127	0.234	389.851	-0.380	0.209	0.069	0.357	389.851	-0.061	0.242	0.801	0.900

BRCC3	526.776	-0.386	0.232	0.095	0.189	526.776	-0.333	0.231	0.150	0.442	526.776	0.053	0.268	0.842	0.923
FSD1	93.621	0.270	0.328	0.410	0.549	93.621	-0.396	0.332	0.233	0.510	93.621	-0.666	0.382	0.081	0.247
TMEM43	3489.149	-0.432	0.139	0.002	0.010	3489.149	0.164	0.139	0.238	0.515	3489.149	0.596	0.161	0.000	0.004
DDX39B	309.829	-0.108	0.298	0.717	0.807	309.829	-0.095	0.297	0.748	0.866	309.829	0.013	0.344	0.971	0.986
IRX3	761.232	-0.265	0.366	0.469	0.604	761.232	0.435	0.366	0.235	0.512	761.232	0.700	0.423	0.098	0.277
IRX1	94.895	0.126	0.210	0.548	0.671	94.895	0.466	0.204	0.023	0.315	94.895	0.339	0.239	0.156	0.368
ABHD16A	1443.959	0.416	0.138	0.003	0.012	1443.959	0.204	0.138	0.140	0.431	1443.959	-0.212	0.159	0.183	0.402
SLC39A7	10124.157	0.109	0.286	0.702	0.795	10124.157	-0.424	0.286	0.138	0.429	10124.157	-0.534	0.330	0.106	0.291
THOC6	861.070	0.249	0.205	0.225	0.357	861.070	-0.074	0.206	0.720	0.850	861.070	-0.323	0.238	0.174	0.391
HSD17B8	536.125	0.257	0.309	0.406	0.544	536.125	0.418	0.308	0.175	0.464	536.125	0.161	0.356	0.651	0.812
ZNF557	74.711	-0.059	0.234	0.801	0.869	74.711	0.056	0.230	0.808	0.899	74.711	0.115	0.270	0.670	0.824
MMEL1	178.002	1.196	0.330	0.000	0.002	178.002	-0.462	0.337	0.171	0.460	178.002	-1.658	0.385	0.000	0.001
DCAF10	1105.115	0.041	0.127	0.744	0.828	1105.115	-0.016	0.126	0.902	0.950	1105.115	-0.057	0.146	0.697	0.841
OR51B4	47.106	-0.615	0.424	0.147	0.261	47.106	-0.043	0.414	0.916	0.958	47.106	0.571	0.488	0.242	0.473
NELFE	5098.754	-0.149	0.166	0.369	0.510	5098.754	0.134	0.166	0.419	0.656	5098.754	0.284	0.192	0.139	0.343
CPLANE2	77.516	-0.691	0.243	0.004	0.018	77.516	0.118	0.229	0.605	0.782	77.516	0.810	0.277	0.003	0.032
ZXDC	362.915	-0.442	0.177	0.013	0.041	362.915	-0.023	0.175	0.894	0.947	362.915	0.419	0.204	0.040	0.160
BHLHE41	196.574	1.428	0.930	0.125	0.230	196.574	0.938	0.930	0.313	0.577	196.574	-0.490	1.073	0.648	0.810
HMGN5	606.202	0.252	0.316	0.426	0.562	606.202	-0.093	0.316	0.768	0.877	606.202	-0.345	0.365	0.345	0.579
B3GNT4	484.162	0.443	0.200	0.026	0.071	484.162	-0.124	0.201	0.536	0.734	484.162	-0.568	0.232	0.014	0.082
GLB1L	49.935	0.339	0.289	0.242	0.375	49.935	0.466	0.285	0.102	0.394	49.935	0.127	0.331	0.700	0.843
KREMEN2	120.569	0.801	0.223	0.000	0.002	120.569	0.486	0.224	0.030	0.322	120.569	-0.315	0.256	0.219	0.446
LRFN3	231.729	0.808	0.189	0.000	0.000	231.729	0.338	0.191	0.077	0.367	231.729	-0.471	0.218	0.031	0.134
CYBC1	624.984	0.490	0.134	0.000	0.002	624.984	0.198	0.135	0.142	0.434	624.984	-0.292	0.155	0.060	0.205
TFEB	399.741	-0.470	0.144	0.001	0.006	399.741	0.131	0.140	0.351	0.605	399.741	0.601	0.165	0.000	0.005
HAUS3	40.000	-0.198	0.311	0.523	0.650	40.000	-0.009	0.303	0.977	0.987	40.000	0.190	0.358	0.596	0.775
FYCO1	924.821	-0.170	0.184	0.356	0.496	924.821	0.033	0.184	0.857	0.927	924.821	0.203	0.213	0.339	0.574
WDR25	118.547	-0.582	0.262	0.026	0.070	118.547	-0.579	0.260	0.026	0.316	118.547	0.003	0.305	0.992	0.995
PAGR1	220.728	-0.170	0.200	0.395	0.534	220.728	-0.088	0.199	0.658	0.814	220.728	0.082	0.231	0.722	0.856
ULBP3	178.496	0.650	0.167	0.000	0.001	178.496	0.042	0.170	0.803	0.897	178.496	-0.607	0.194	0.002	0.020
RNF217-AS1	57.270	-0.282	0.268	0.293	0.431	57.270	-0.088	0.262	0.737	0.859	57.270	0.194	0.310	0.531	0.731
RIPOR1	1629.122	-0.197	0.196	0.315	0.454	1629.122	-0.310	0.196	0.115	0.406	1629.122	-0.112	0.227	0.621	0.792
MAIP1	154.843	-0.182	0.215	0.397	0.536	154.843	-0.291	0.214	0.174	0.463	154.843	-0.109	0.250	0.661	0.819
EPM2A	63.181	-0.434	0.216	0.045	0.106	63.181	-0.384	0.211	0.069	0.357	63.181	0.051	0.254	0.842	0.923
GCC1	464.275	-0.875	0.277	0.002	0.008	464.275	-0.468	0.275	0.089	0.382	464.275	0.407	0.320	0.203	0.426
ATP13A3	2694.003	-0.438	0.325	0.177	0.299	2694.003	-0.646	0.325	0.047	0.335	2694.003	-0.208	0.375	0.580	0.764
TTC13	235.071	-0.090	0.224	0.688	0.785	235.071	-0.044	0.223	0.844	0.919	235.071	0.046	0.258	0.859	0.932
EPS8L3	13.082	0.323	0.441	0.463	0.598	13.082	0.120	0.442	0.786	0.888	13.082	-0.204	0.511	0.690	0.837
ABHD8	265.125	1.281	0.337	0.000	0.001	265.125	0.773	0.338	0.022	0.315	265.125	-0.508	0.388	0.191	0.412
NKAP	449.109	0.336	0.197	0.088	0.178	449.109	0.187	0.197	0.342	0.599	449.109	-0.149	0.227	0.512	0.717
CDC73	1110.166	-0.967	0.223	0.000	0.000	1110.166	-0.425	0.222	0.055	0.343	1110.166	0.542	0.257	0.035	0.146
SLC52A2	1057.702	0.156	0.382	0.682	0.780	1057.702	-0.788	0.383	0.039	0.327	1057.702	-0.945	0.442	0.033	0.139
SPAG16	220.142	0.532	0.268	0.047	0.111	220.142	0.222	0.269	0.409	0.649	220.142	-0.311	0.310	0.316	0.552
TMEM231	332.872	-0.074	0.168	0.658	0.761	332.872	0.078	0.167	0.639	0.803	332.872	0.153	0.194	0.431	0.653
CORO7	155.808	1.507	0.318	0.000	0.000	155.808	0.129	0.323	0.690	0.832	155.808	-1.378	0.369	0.000	0.004
CHPF	1961.444	1.167	0.140	0.000	0.000	1961.444	-0.012	0.141	0.932	0.966	1961.444	-1.179	0.162	0.000	0.000
CARS2	1565.300	0.324	0.146	0.027	0.071	1565.300	-0.164	0.147	0.264	0.536	1565.300	-0.488	0.169	0.004	0.035
MRPL24	1118.407	0.218	0.235	0.353	0.493	1118.407	-0.028	0.235	0.904	0.952	1118.407	-0.246	0.271	0.363	0.595
ARMH3	456.857	-0.402	0.160	0.012	0.039	456.857	-0.106	0.159	0.502	0.714	456.857	0.296	0.185	0.110	0.295
MUL1	521.389	-0.185	0.248	0.456	0.590	521.389	-0.377	0.248	0.128	0.420	521.389	-0.192	0.287	0.502	0.709
SAP130	414.982	-0.055	0.150	0.715	0.806	414.982	-0.123	0.150	0.412	0.651	414.982	-0.068	0.174	0.696	0.841
OBI1	200.267	-1.231	0.340	0.000	0.002	200.267	-0.946	0.338	0.005	0.297	200.267	0.285	0.394	0.470	0.684
CEP97	268.994	-0.524	0.237	0.027	0.073	268.994	-0.028	0.235	0.905	0.952	268.994	0.496	0.273	0.070	0.225
TCTN1	336.862	0.249	0.249	0.318	0.457	336.862	0.448	0.248	0.071	0.360	336.862	0.199	0.287	0.488	0.698
ADIPOR2	1995.150	-0.051	0.188	0.785	0.858	1995.150	-0.341	0.188	0.069	0.358	1995.150	-0.290	0.217	0.182	0.400
CERS4	573.863	1.361	0.327	0.000	0.000	573.863	0.571	0.327	0.081	0.372	573.863	-0.790	0.377	0.036	0.148
PGBD5	14.590	1.648	0.717	0.022	0.061	14.590	-5.710	1.370	0.000	0.056	14.590	-7.358	1.422	0.000	0.000
FAM118B	198.521	-0.068	0.342	0.841	0.896	198.521	-0.693	0.344	0.044	0.332	198.521	-0.625	0.398	0.116	0.305
VCPKMT	77.092	-0.596	0.286	0.037	0.093	77.092	-0.622	0.284	0.028	0.321	77.092	-0.026	0.335	0.938	0.972
NAA16	111.609	-0.440	0.258	0.088	0.178	111.609	-0.422	0.256	0.100	0.394	111.609	0.019	0.300	0.950	0.977
TANGO6	409.012	-0.642	0.206	0.002	0.009	409.012	-0.293	0.204	0.152	0.444	409.012	0.350	0.238	0.142	0.347
CCNJL	43.460	0.425	0.301	0.158	0.276	43.460	0.085	0.306	0.782	0.885	43.460	-0.340	0.350	0.332	0.567
HMBOX1	214.166	0.513	0.294	0.081	0.168	214.166	0.352	0.294	0.232	0.509	214.166	-0.161	0.339	0.636	0.802
RNASEH2B	375.035	-0.261	0.191	0.171	0.292	375.035	-0.109	0.190	0.564	0.752	375.035	0.152	0.220	0.492	0.701
SNRNP25	421.485	-0.100	0.367	0.784	0.857	421.485	-0.900	0.368	0.014	0.306	421.485	-0.799	0.425	0.060	0.205
GALNT14	787.547	-0.279	0.287	0.332	0.472	787.547	-0.463	0.287	0.107	0.399	787.547	-0.184	0.332	0.578	0.763
ARMT1	337.211	-0.537	0.203	0.008	0.029	337.211	-0.292	0.201	0.146	0.438	337.211	0.245	0.235	0.296	0.532

OGFRL1	3266.059	-0.960	0.222	0.000	0.000	3266.059	0.070	0.222	0.751	0.867	3266.059	1.030	0.257	0.000	0.002
SH3TC2	17.261	0.657	0.489	0.179	0.302	17.261	0.208	0.495	0.674	0.824	17.261	-0.449	0.566	0.428	0.651
OCEL1	413.891	0.907	0.317	0.004	0.017	413.891	0.631	0.317	0.046	0.335	413.891	-0.276	0.365	0.449	0.668
C1orf54	182.241	-0.505	0.249	0.043	0.103	182.241	0.030	0.245	0.903	0.951	182.241	0.535	0.287	0.063	0.210
EFL1	331.063	-0.428	0.212	0.044	0.104	331.063	0.012	0.209	0.954	0.978	331.063	0.440	0.245	0.072	0.229
FAM184A	12.460	0.433	0.475	0.362	0.503	12.460	0.501	0.467	0.284	0.553	12.460	0.068	0.540	0.900	0.953
FAT4	142.607	-0.047	0.452	0.917	0.947	142.607	0.299	0.450	0.506	0.716	142.607	0.346	0.521	0.506	0.712
SCRN3	283.984	-0.309	0.164	0.060	0.133	283.984	-0.033	0.162	0.840	0.917	283.984	0.276	0.190	0.145	0.352
CCDC121	35.950	-0.269	0.399	0.501	0.631	35.950	0.409	0.386	0.289	0.557	35.950	0.677	0.455	0.137	0.339
ARMC7	168.137	0.321	0.200	0.109	0.209	168.137	-0.016	0.201	0.937	0.968	168.137	-0.336	0.232	0.148	0.355
TMEM53	621.054	0.216	0.157	0.171	0.291	621.054	-0.147	0.158	0.353	0.607	621.054	-0.362	0.182	0.047	0.175
C22orf46	431.669	0.122	0.210	0.560	0.681	431.669	0.141	0.209	0.501	0.714	431.669	0.018	0.242	0.940	0.973
ROGDI	150.728	0.963	0.279	0.001	0.004	150.728	0.349	0.281	0.215	0.494	150.728	-0.615	0.322	0.056	0.197
ARSJ	733.699	-0.367	0.201	0.068	0.147	733.699	-0.142	0.200	0.478	0.698	733.699	0.224	0.232	0.334	0.569
CHMP6	228.829	0.129	0.240	0.591	0.706	228.829	-0.541	0.243	0.026	0.316	228.829	-0.670	0.280	0.017	0.092
SRD5A3	167.784	0.317	0.230	0.168	0.288	167.784	0.234	0.230	0.308	0.573	167.784	-0.083	0.265	0.755	0.875
PANK3	2160.581	-0.458	0.283	0.106	0.203	2160.581	-0.345	0.283	0.223	0.502	2160.581	0.113	0.327	0.729	0.859
AKIRIN1	1339.759	-0.329	0.280	0.241	0.374	1339.759	-0.648	0.280	0.021	0.312	1339.759	-0.319	0.324	0.324	0.559
MCPH1	359.539	-0.793	0.238	0.001	0.005	359.539	-0.544	0.237	0.022	0.314	359.539	0.249	0.276	0.367	0.600
MAP7D3	598.320	-0.083	0.177	0.640	0.747	598.320	-0.287	0.177	0.105	0.398	598.320	-0.205	0.205	0.319	0.554
AIMP2	1062.865	-0.768	0.295	0.009	0.032	1062.865	-0.576	0.294	0.050	0.337	1062.865	0.192	0.340	0.574	0.761
USB1	1275.368	0.297	0.296	0.316	0.455	1275.368	-0.620	0.297	0.037	0.324	1275.368	-0.917	0.342	0.007	0.054
RHBDF2	710.970	0.700	0.109	0.000	0.000	710.970	0.165	0.110	0.134	0.425	710.970	-0.535	0.126	0.000	0.001
TMEM204	505.196	0.238	0.165	0.150	0.264	505.196	0.081	0.165	0.624	0.794	505.196	-0.157	0.191	0.411	0.637
HECTD3	966.380	0.480	0.145	0.001	0.006	966.380	0.234	0.145	0.107	0.399	966.380	-0.245	0.168	0.143	0.349
RPAP3	483.994	-0.667	0.219	0.002	0.011	483.994	-0.636	0.218	0.004	0.297	483.994	0.031	0.253	0.902	0.954
ARHGAP10	544.604	-0.486	0.141	0.001	0.004	544.604	0.219	0.138	0.112	0.405	544.604	0.705	0.162	0.000	0.000
DYNC2H1	710.572	-0.805	0.240	0.001	0.005	710.572	-0.048	0.238	0.841	0.917	710.572	0.757	0.277	0.006	0.047
PPP1R3B	334.659	1.334	0.774	0.085	0.173	334.659	1.099	0.775	0.156	0.448	334.659	-0.235	0.894	0.793	0.896
NEIL1	125.426	1.309	0.360	0.000	0.002	125.426	0.747	0.361	0.039	0.327	125.426	-0.561	0.414	0.175	0.393
HSPBAP1	223.271	0.005	0.397	0.990	0.994	223.271	-0.863	0.400	0.031	0.322	223.271	-0.868	0.461	0.060	0.205
ICE2	683.070	-0.558	0.145	0.000	0.001	683.070	-0.169	0.144	0.239	0.515	683.070	0.389	0.168	0.020	0.103
DHX40	1400.427	-0.337	0.299	0.260	0.395	1400.427	0.440	0.299	0.141	0.432	1400.427	0.777	0.345	0.024	0.115
PLEKHF2	298.671	-0.939	0.233	0.000	0.001	298.671	-0.002	0.228	0.994	0.997	298.671	0.938	0.268	0.000	0.008
KLF3-AS1	93.022	-0.781	0.245	0.001	0.008	93.022	0.020	0.230	0.930	0.965	93.022	0.801	0.280	0.004	0.036
PARP8	857.320	-0.537	0.132	0.000	0.001	857.320	-0.034	0.130	0.792	0.891	857.320	0.502	0.152	0.001	0.013
C3orf52	58.722	0.205	0.311	0.511	0.640	58.722	-0.417	0.318	0.190	0.475	58.722	-0.622	0.366	0.090	0.262
TUT7	634.725	-0.140	0.190	0.461	0.596	634.725	-0.058	0.190	0.760	0.872	634.725	0.082	0.220	0.708	0.849
NLRX1	586.588	-0.036	0.283	0.898	0.934	586.588	0.447	0.283	0.114	0.405	586.588	0.483	0.327	0.139	0.343
FN3KRP	636.664	-0.012	0.110	0.913	0.945	636.664	-0.138	0.110	0.212	0.492	636.664	-0.126	0.128	0.327	0.562
ZNF329	68.111	-0.535	0.257	0.037	0.093	68.111	-0.165	0.248	0.506	0.716	68.111	0.371	0.297	0.212	0.437
VEPH1	35.142	-2.316	0.787	0.003	0.014	35.142	-2.471	0.786	0.002	0.297	35.142	-0.155	0.931	0.867	0.936
FASTKD1	253.057	-0.254	0.219	0.245	0.378	253.057	-0.462	0.219	0.035	0.322	253.057	-0.207	0.254	0.414	0.640
OGFOD2	269.162	0.071	0.154	0.646	0.752	269.162	0.089	0.153	0.558	0.748	269.162	0.019	0.178	0.916	0.961
SMC6	662.966	-0.526	0.277	0.057	0.128	662.966	-0.777	0.277	0.005	0.297	662.966	-0.251	0.320	0.434	0.655
RTL10	482.335	-0.297	0.166	0.075	0.157	482.335	-0.226	0.166	0.172	0.462	482.335	0.071	0.193	0.713	0.851
CENPU	254.088	-0.160	0.327	0.625	0.735	254.088	-0.459	0.328	0.161	0.453	254.088	-0.299	0.379	0.431	0.653
ZDHHC14	120.805	0.517	0.386	0.181	0.304	120.805	-0.582	0.393	0.138	0.429	120.805	-1.099	0.451	0.015	0.085
MSANTD2	84.482	0.460	0.254	0.070	0.149	84.482	0.101	0.256	0.692	0.834	84.482	-0.358	0.294	0.223	0.451
SAP30L	567.426	0.068	0.178	0.701	0.794	567.426	0.247	0.177	0.163	0.454	567.426	0.178	0.205	0.384	0.613
QTRT2	493.735	-0.888	0.255	0.000	0.003	493.735	-0.418	0.253	0.099	0.393	493.735	0.470	0.294	0.110	0.296
ZNF322	372.930	-0.181	0.153	0.238	0.371	372.930	-0.083	0.152	0.585	0.768	372.930	0.098	0.177	0.580	0.764
YRDC	555.720	-0.863	0.433	0.046	0.109	555.720	-0.932	0.433	0.031	0.322	555.720	-0.068	0.501	0.892	0.948
MANEA	384.844	-0.619	0.201	0.002	0.010	384.844	-0.139	0.199	0.485	0.702	384.844	0.480	0.232	0.039	0.155
GALNT12	32.136	0.385	0.370	0.298	0.437	32.136	0.173	0.370	0.640	0.803	32.136	-0.211	0.427	0.621	0.792
RIOX1	182.827	-0.295	0.273	0.280	0.417	182.827	-0.690	0.274	0.012	0.305	182.827	-0.395	0.318	0.214	0.440
ZYG11B	724.671	-0.125	0.296	0.674	0.773	724.671	0.189	0.296	0.523	0.726	724.671	0.314	0.342	0.359	0.591
CALCB	51.615	-4.557	0.814	0.000	0.000	51.615	-2.000	0.720	0.005	0.297	51.615	2.557	0.921	0.006	0.044
OGFOD3	717.296	0.153	0.117	0.190	0.315	717.296	-0.038	0.117	0.746	0.865	717.296	-0.191	0.135	0.158	0.370
C11orf80	178.372	0.272	0.312	0.384	0.523	178.372	0.366	0.311	0.240	0.516	178.372	0.094	0.360	0.793	0.896
LRRK1	495.867	-0.104	0.179	0.560	0.681	495.867	-0.006	0.178	0.973	0.985	495.867	0.098	0.206	0.634	0.801
NOL9	329.337	-0.586	0.260	0.024	0.066	329.337	-0.387	0.258	0.134	0.426	329.337	0.199	0.300	0.507	0.713
COLGALT1	6925.814	-0.114	0.264	0.667	0.768	6925.814	-0.340	0.264	0.197	0.481	6925.814	-0.227	0.305	0.457	0.675
MORC4	500.529	-0.684	0.123	0.000	0.000	500.529	-0.212	0.120	0.077	0.369	500.529	0.472	0.142	0.001	0.012
IPO4	1520.761	-1.173	0.244	0.000	0.000	1520.761	-0.257	0.243	0.290	0.558	1520.761	0.916	0.282	0.001	0.015
GTDC1	132.107	-0.669	0.200	0.001	0.005	132.107	-0.177	0.193	0.358	0.611	132.107	0.492	0.231	0.033	0.140
IGFLR1	26.792	0.981	0.432	0.023	0.064	26.792	0.423	0.438	0.334	0.593	26.792	-0.558	0.497	0.262	0.496

CCDC51	1002.059	0.311	0.179	0.082	0.169	1002.059	-0.075	0.179	0.674	0.824	1002.059	-0.386	0.207	0.062	0.209
NPEPL1	134.168	0.472	0.238	0.048	0.112	134.168	0.377	0.238	0.113	0.405	134.168	-0.095	0.274	0.728	0.859
PPCS	573.848	0.816	0.199	0.000	0.000	573.848	0.119	0.201	0.552	0.744	573.848	-0.696	0.231	0.003	0.026
TBL1XR1	1632.658	-0.255	0.160	0.112	0.212	1632.658	-0.096	0.160	0.549	0.743	1632.658	0.159	0.185	0.390	0.619
AAGAB	668.751	-0.427	0.350	0.223	0.353	668.751	-0.670	0.350	0.056	0.343	668.751	-0.243	0.404	0.548	0.743
VPS37B	1531.624	-0.559	0.177	0.002	0.008	1531.624	-0.341	0.176	0.054	0.342	1531.624	0.219	0.204	0.285	0.520
SUV39H2	363.080	-0.646	0.252	0.010	0.035	363.080	-0.464	0.251	0.064	0.353	363.080	0.182	0.292	0.533	0.732
ZNF768	1279.900	0.006	0.154	0.969	0.981	1279.900	0.089	0.153	0.561	0.750	1279.900	0.083	0.177	0.640	0.804
THAP9	57.685	-0.329	0.289	0.254	0.388	57.685	0.250	0.277	0.367	0.617	57.685	0.579	0.329	0.079	0.242
WDR59	826.696	0.037	0.146	0.798	0.867	826.696	0.093	0.145	0.522	0.726	826.696	0.056	0.168	0.741	0.867
PALB2	316.262	-1.140	0.284	0.000	0.001	316.262	-0.599	0.282	0.033	0.322	316.262	0.541	0.329	0.100	0.280
SH3D21	331.399	0.983	0.445	0.027	0.073	331.399	1.037	0.445	0.020	0.306	331.399	0.054	0.513	0.916	0.961
NSUN7	10.865	1.001	0.476	0.035	0.089	10.865	0.524	0.487	0.281	0.551	10.865	-0.477	0.544	0.380	0.610
NARS2	480.037	-0.584	0.188	0.002	0.009	480.037	-0.268	0.186	0.151	0.443	480.037	0.316	0.217	0.146	0.352
E2F8	23.042	-0.502	0.326	0.123	0.229	23.042	-0.348	0.314	0.268	0.539	23.042	0.154	0.380	0.686	0.835
KCTD17	186.410	0.016	0.205	0.938	0.961	186.410	-0.483	0.208	0.020	0.308	186.410	-0.499	0.240	0.038	0.152
TBC1D17	805.334	0.909	0.261	0.001	0.003	805.334	0.504	0.262	0.054	0.342	805.334	-0.406	0.302	0.179	0.397
TEFM	102.050	-0.037	0.236	0.876	0.920	102.050	-0.351	0.237	0.139	0.430	102.050	-0.315	0.275	0.253	0.486
BBS10	252.013	-0.979	0.210	0.000	0.000	252.013	-0.437	0.206	0.034	0.322	252.013	0.542	0.243	0.026	0.120
TTLL7	146.847	0.558	0.249	0.025	0.068	146.847	-0.014	0.252	0.954	0.978	146.847	-0.573	0.289	0.048	0.176
CCDC7	22.638	-0.916	0.373	0.014	0.044	22.638	0.012	0.332	0.972	0.985	22.638	0.928	0.422	0.028	0.125
ZNF419	97.418	-0.186	0.181	0.304	0.443	97.418	-0.104	0.177	0.559	0.748	97.418	0.082	0.210	0.695	0.841
CLIP4	422.061	-0.536	0.325	0.099	0.194	422.061	0.173	0.323	0.592	0.773	422.061	0.709	0.374	0.058	0.201
ECHDC3	469.117	-0.298	0.296	0.314	0.454	469.117	0.389	0.295	0.187	0.473	469.117	0.687	0.341	0.044	0.169
MAFK	3266.441	-0.200	0.224	0.371	0.512	3266.441	-0.314	0.224	0.160	0.451	3266.441	-0.114	0.258	0.658	0.817
ZNF385D	214.259	-0.173	0.151	0.253	0.388	214.259	-0.156	0.149	0.297	0.565	214.259	0.017	0.175	0.924	0.965
SLC25A22	1626.759	0.184	0.136	0.177	0.299	1626.759	0.055	0.136	0.688	0.831	1626.759	-0.129	0.157	0.411	0.637
ZFAND1	1503.611	-0.656	0.158	0.000	0.000	1503.611	0.112	0.157	0.475	0.696	1503.611	0.769	0.182	0.000	0.001
SNIP1	346.156	-0.291	0.189	0.124	0.230	346.156	-0.059	0.188	0.754	0.868	346.156	0.232	0.219	0.288	0.523
ASB13	900.104	-0.068	0.187	0.715	0.805	900.104	0.095	0.186	0.611	0.786	900.104	0.163	0.215	0.450	0.669
DHRS12	211.796	0.015	0.182	0.935	0.959	211.796	0.163	0.180	0.366	0.617	211.796	0.148	0.209	0.480	0.693
ZNF668	302.812	0.328	0.178	0.064	0.140	302.812	-0.037	0.178	0.837	0.916	302.812	-0.365	0.206	0.076	0.238
FZD3	251.774	0.454	0.143	0.002	0.008	251.774	0.086	0.144	0.553	0.745	251.774	-0.368	0.166	0.027	0.122
GEMIN7	260.549	-0.174	0.278	0.531	0.657	260.549	-0.825	0.280	0.003	0.297	260.549	-0.651	0.324	0.045	0.170
C1orf115	177.535	0.492	0.333	0.139	0.250	177.535	0.510	0.332	0.124	0.416	177.535	0.019	0.383	0.961	0.982
ISOC2	558.297	0.426	0.185	0.022	0.061	558.297	0.019	0.186	0.918	0.959	558.297	-0.406	0.214	0.058	0.201
ELMO3	38.782	0.783	0.402	0.051	0.118	38.782	-0.175	0.414	0.672	0.823	38.782	-0.958	0.471	0.042	0.164
KATNBL1	266.539	-0.004	0.201	0.985	0.992	266.539	-0.256	0.202	0.205	0.488	266.539	-0.252	0.234	0.281	0.515
TXNDC15	537.090	0.570	0.143	0.000	0.001	537.090	0.176	0.144	0.221	0.501	537.090	-0.394	0.166	0.017	0.093
MCTP1	856.427	-0.593	0.226	0.009	0.030	856.427	-0.078	0.225	0.728	0.855	856.427	0.515	0.260	0.048	0.178
GRTP1	8.349	0.576	0.583	0.324	0.464	8.349	-0.415	0.626	0.508	0.717	8.349	-0.990	0.706	0.161	0.374
ZFHX4	7.063	0.878	0.747	0.240	0.373	7.063	0.964	0.738	0.192	0.477	7.063	0.086	0.841	0.919	0.962
ACBD4	190.810	0.388	0.299	0.195	0.320	190.810	0.460	0.298	0.122	0.414	190.810	0.073	0.344	0.833	0.918
MICALL2	507.448	1.214	0.215	0.000	0.000	507.448	0.164	0.217	0.449	0.678	507.448	-1.050	0.249	0.000	0.001
MTERF1	116.795	0.240	0.200	0.232	0.364	116.795	-0.474	0.207	0.022	0.314	116.795	-0.714	0.238	0.003	0.026
CCDC82	301.214	-0.308	0.203	0.128	0.235	301.214	-0.497	0.203	0.014	0.306	301.214	-0.189	0.235	0.422	0.646
SUGCT	17.352	-0.027	0.560	0.962	0.977	17.352	0.333	0.548	0.544	0.739	17.352	0.359	0.642	0.576	0.761
MYH14	19.881	0.379	0.374	0.311	0.450	19.881	0.370	0.368	0.315	0.577	19.881	-0.008	0.426	0.984	0.991
KLHL36	1002.391	0.307	0.249	0.218	0.348	1002.391	0.392	0.249	0.115	0.407	1002.391	0.085	0.287	0.767	0.881
CLMN	196.562	0.115	0.297	0.697	0.791	196.562	0.519	0.294	0.078	0.369	196.562	0.404	0.341	0.236	0.466
SEM1	1106.277	1.075	0.265	0.000	0.001	1106.277	0.070	0.265	0.791	0.891	1106.277	-1.005	0.306	0.001	0.013
FBXO31	806.649	-0.476	0.122	0.000	0.001	806.649	0.188	0.120	0.117	0.409	806.649	0.664	0.141	0.000	0.000
GSDMD	2011.232	0.421	0.158	0.008	0.028	2011.232	0.247	0.158	0.117	0.409	2011.232	-0.173	0.182	0.341	0.575
C12orf49	596.905	-0.221	0.225	0.327	0.467	596.905	-0.376	0.225	0.095	0.390	596.905	-0.155	0.261	0.552	0.746
ALG9	171.273	0.175	0.160	0.272	0.409	171.273	-0.098	0.160	0.542	0.738	171.273	-0.273	0.186	0.142	0.347
ZNF408	270.700	-0.219	0.179	0.220	0.351	270.700	-0.063	0.177	0.719	0.849	270.700	0.156	0.206	0.451	0.670
ARMC5	312.479	-0.125	0.185	0.498	0.629	312.479	0.071	0.183	0.697	0.837	312.479	0.196	0.213	0.357	0.590
TFPI2	269.260	-1.207	0.801	0.132	0.240	269.260	-2.318	0.809	0.004	0.297	269.260	-1.111	0.935	0.235	0.464
CARF	51.631	-0.343	0.523	0.512	0.641	51.631	0.530	0.513	0.302	0.568	51.631	0.873	0.599	0.145	0.352
SHCBP1	971.102	-0.949	0.293	0.001	0.007	971.102	-0.376	0.292	0.198	0.481	971.102	0.573	0.338	0.090	0.263
HPS6	511.892	-0.015	0.131	0.909	0.942	511.892	0.010	0.130	0.941	0.970	511.892	0.025	0.151	0.870	0.938
HAND2-AS1	20.371	-0.569	0.513	0.267	0.403	20.371	0.251	0.490	0.608	0.784	20.371	0.820	0.585	0.161	0.374
VASH2	8.743	1.407	0.660	0.033	0.084	8.743	-1.176	0.774	0.129	0.421	8.743	-2.583	0.847	0.002	0.024
GSTCD	223.415	-0.886	0.206	0.000	0.000	223.415	-0.135	0.200	0.499	0.712	223.415	0.751	0.237	0.002	0.018
TTC21B	241.650	-0.695	0.255	0.006	0.024	241.650	0.029	0.251	0.907	0.954	241.650	0.724	0.293	0.014	0.080
PTCD2	499.047	-0.733	0.147	0.000	0.000	499.047	-0.283	0.144	0.050	0.337	499.047	0.450	0.169	0.008	0.056
SLTM	1265.108	0.002	0.150	0.988	0.993	1265.108	-0.116	0.150	0.438	0.671	1265.108	-0.118	0.173	0.495	0.703

EHMT1	977.961	-0.334	0.159	0.036	0.090	977.961	-0.222	0.159	0.162	0.453	977.961	0.112	0.184	0.542	0.739
AGMAT	8.919	1.034	0.709	0.145	0.257	8.919	-0.016	0.753	0.983	0.990	8.919	-1.050	0.839	0.210	0.436
NIPAL2	209.131	0.629	0.380	0.098	0.193	209.131	0.386	0.381	0.311	0.575	209.131	-0.244	0.439	0.579	0.764
TLE6	170.785	0.937	0.408	0.022	0.061	170.785	1.078	0.407	0.008	0.297	170.785	0.141	0.469	0.763	0.880
MOB3B	24.970	-0.873	0.356	0.014	0.044	24.970	0.083	0.314	0.792	0.892	24.970	0.956	0.401	0.017	0.092
ZNF552	52.310	-0.337	0.254	0.185	0.309	52.310	-0.281	0.249	0.259	0.531	52.310	0.056	0.296	0.851	0.928
WDR78	30.753	0.499	0.510	0.327	0.467	30.753	0.680	0.505	0.178	0.467	30.753	0.181	0.583	0.757	0.876
ST7	179.692	-0.392	0.220	0.076	0.159	179.692	-0.497	0.220	0.024	0.316	179.692	-0.105	0.257	0.682	0.832
CAMKMT	93.578	0.083	0.253	0.744	0.828	93.578	-0.229	0.255	0.369	0.619	93.578	-0.311	0.295	0.291	0.526
CLMP	4003.674	-0.716	0.196	0.000	0.002	4003.674	0.214	0.196	0.274	0.545	4003.674	0.930	0.226	0.000	0.001
METTL8	362.421	-0.628	0.151	0.000	0.000	362.421	-0.212	0.148	0.152	0.444	362.421	0.415	0.174	0.017	0.093
NAA40	195.567	-0.117	0.252	0.644	0.750	195.567	-0.365	0.253	0.149	0.441	195.567	-0.248	0.293	0.396	0.624
ZMYM1	197.883	-0.587	0.248	0.018	0.052	197.883	-0.062	0.244	0.801	0.896	197.883	0.526	0.285	0.065	0.216
KDM8	45.084	-0.094	0.315	0.765	0.844	45.084	-0.322	0.316	0.309	0.573	45.084	-0.228	0.369	0.537	0.735
QSER1	1264.812	-0.928	0.227	0.000	0.000	1264.812	-0.194	0.226	0.390	0.635	1264.812	0.734	0.262	0.005	0.041
GEMIN6	190.573	0.385	0.175	0.028	0.074	190.573	-0.095	0.177	0.590	0.771	190.573	-0.480	0.204	0.019	0.097
PEAK1	439.489	-0.723	0.233	0.002	0.010	439.489	-0.143	0.231	0.534	0.733	439.489	0.579	0.268	0.031	0.134
PIP4K2C	318.460	-0.052	0.222	0.816	0.879	318.460	-0.287	0.223	0.197	0.481	318.460	-0.236	0.258	0.361	0.594
TMC5	16.279	0.498	0.416	0.232	0.364	16.279	0.192	0.421	0.649	0.808	16.279	-0.306	0.482	0.525	0.726
CCDC102B	158.377	-0.540	0.319	0.090	0.181	158.377	0.389	0.314	0.215	0.494	158.377	0.929	0.366	0.011	0.070
ARHGEF5	867.317	0.360	0.168	0.032	0.083	867.317	0.239	0.168	0.154	0.446	867.317	-0.120	0.194	0.534	0.733
NHEJ1	269.082	-0.244	0.165	0.140	0.251	269.082	0.083	0.162	0.608	0.784	269.082	0.327	0.190	0.086	0.255
AGBL2	10.759	0.088	0.705	0.901	0.937	10.759	0.999	0.677	0.140	0.431	10.759	0.912	0.795	0.252	0.484
ZBTB3	42.044	-0.317	0.324	0.327	0.467	42.044	0.267	0.310	0.390	0.635	42.044	0.584	0.369	0.114	0.302
ZDHHC11	30.846	2.110	0.693	0.002	0.011	30.846	1.494	0.696	0.032	0.322	30.846	-0.616	0.790	0.436	0.657
RNF122	108.345	1.482	0.406	0.000	0.002	108.345	0.978	0.408	0.017	0.306	108.345	-0.504	0.465	0.279	0.513
CFAP69	26.783	-0.106	0.372	0.775	0.850	26.783	0.076	0.362	0.833	0.914	26.783	0.183	0.428	0.669	0.824
MFSD13A	81.343	-0.216	0.336	0.521	0.648	81.343	-0.653	0.339	0.054	0.342	81.343	-0.437	0.393	0.266	0.500
CSPP1	325.502	0.313	0.228	0.169	0.289	325.502	-0.164	0.229	0.475	0.696	325.502	-0.477	0.264	0.071	0.227
TLCD3A	580.497	0.410	0.148	0.006	0.022	580.497	-0.099	0.150	0.508	0.717	580.497	-0.509	0.173	0.003	0.030
LINC00115	14.364	0.232	0.469	0.621	0.731	14.364	-0.384	0.485	0.429	0.664	14.364	-0.616	0.558	0.270	0.505
SNX22	4437.856	0.832	0.258	0.001	0.007	4437.856	-0.008	0.259	0.974	0.986	4437.856	-0.841	0.298	0.005	0.040
FLJ13224	6.970	-1.230	0.944	0.193	0.318	6.970	-0.112	0.872	0.897	0.948	6.970	1.117	1.075	0.299	0.534
NEK11	37.156	0.604	0.454	0.183	0.307	37.156	0.522	0.453	0.248	0.522	37.156	-0.082	0.521	0.875	0.940
TUBAL3	68.606	0.679	0.232	0.003	0.015	68.606	0.023	0.240	0.925	0.962	68.606	-0.657	0.272	0.016	0.088
ZNF669	52.041	-0.387	0.281	0.168	0.288	52.041	-0.642	0.283	0.023	0.316	52.041	-0.255	0.333	0.445	0.665
RBFA	264.939	0.110	0.193	0.568	0.688	264.939	-0.197	0.194	0.308	0.573	264.939	-0.308	0.224	0.170	0.385
JHY	74.595	0.804	0.223	0.000	0.002	74.595	0.147	0.229	0.521	0.725	74.595	-0.657	0.259	0.011	0.071
BORA	155.193	-0.348	0.175	0.048	0.111	155.193	-0.079	0.171	0.646	0.807	155.193	0.269	0.202	0.184	0.403
TCTN2	254.450	0.007	0.326	0.982	0.989	254.450	0.421	0.324	0.194	0.478	254.450	0.413	0.375	0.271	0.506
ALG13	300.023	-0.383	0.162	0.018	0.053	300.023	-0.025	0.159	0.876	0.937	300.023	0.358	0.186	0.055	0.194
CPSF7	1290.504	-0.689	0.213	0.001	0.007	1290.504	-0.410	0.213	0.054	0.342	1290.504	0.280	0.246	0.257	0.491
RPAP2	404.772	-0.274	0.143	0.055	0.124	404.772	-0.101	0.142	0.477	0.697	404.772	0.174	0.166	0.294	0.529
CBLL1	452.334	-0.279	0.149	0.061	0.134	452.334	-0.173	0.148	0.241	0.517	452.334	0.106	0.172	0.539	0.736
NUDT18	269.875	-0.104	0.196	0.594	0.709	269.875	0.214	0.194	0.269	0.540	269.875	0.318	0.225	0.158	0.370
RABEP2	326.275	0.431	0.164	0.009	0.030	326.275	-0.156	0.166	0.349	0.604	326.275	-0.586	0.191	0.002	0.023
THSD4	12.454	-0.171	0.567	0.763	0.842	12.454	-0.122	0.558	0.827	0.910	12.454	0.050	0.658	0.940	0.973
UBA5	1131.312	-0.851	0.210	0.000	0.001	1131.312	-0.386	0.209	0.064	0.353	1131.312	0.464	0.242	0.055	0.194
DCAKD	532.468	0.301	0.230	0.191	0.315	532.468	0.320	0.230	0.163	0.454	532.468	0.019	0.265	0.942	0.974
CCDC134	27.297	-1.177	0.395	0.003	0.013	27.297	-0.719	0.375	0.055	0.343	27.297	0.458	0.459	0.319	0.554
ZNF212	173.782	-0.174	0.212	0.412	0.550	173.782	-0.179	0.211	0.397	0.641	173.782	-0.005	0.246	0.985	0.992
ZC3H14	1166.433	-0.591	0.174	0.001	0.004	1166.433	-0.331	0.173	0.055	0.343	1166.433	0.260	0.201	0.195	0.417
PODNL1	11.540	1.263	0.587	0.031	0.081	11.540	0.631	0.599	0.292	0.560	11.540	-0.632	0.672	0.347	0.581
MAP9	112.954	0.641	0.286	0.025	0.068	112.954	-0.045	0.289	0.876	0.937	112.954	-0.686	0.332	0.039	0.155
HDAC11	219.826	0.829	0.401	0.038	0.095	219.826	0.688	0.400	0.086	0.378	219.826	-0.141	0.462	0.761	0.878
CAAP1	370.806	-0.625	0.159	0.000	0.001	370.806	-0.364	0.157	0.020	0.312	370.806	0.260	0.185	0.158	0.370
PLBD1	19.365	0.820	0.420	0.051	0.117	19.365	1.076	0.409	0.009	0.297	19.365	0.256	0.466	0.583	0.766
LPCAT1	7074.300	1.375	0.131	0.000	0.000	7074.300	-0.072	0.131	0.583	0.766	7074.300	-1.447	0.151	0.000	0.000
RIN3	255.529	0.297	0.165	0.072	0.152	255.529	0.161	0.165	0.329	0.589	255.529	-0.136	0.191	0.474	0.688
MCMBP	2394.885	-1.085	0.276	0.000	0.001	2394.885	-0.655	0.275	0.017	0.306	2394.885	0.429	0.318	0.177	0.395
GGNBP2	3107.309	-0.394	0.090	0.000	0.000	3107.309	0.033	0.090	0.714	0.847	3107.309	0.427	0.104	0.000	0.001
ZNF672	677.194	0.008	0.147	0.958	0.975	677.194	-0.059	0.146	0.686	0.830	677.194	-0.067	0.170	0.693	0.839
ATP8B4	58.899	-1.432	0.320	0.000	0.000	58.899	-0.351	0.298	0.239	0.516	58.899	1.082	0.366	0.003	0.030
THNSL1	63.119	-0.374	0.239	0.117	0.220	63.119	0.310	0.223	0.164	0.455	63.119	0.684	0.270	0.011	0.071
RPP21	1037.614	0.622	0.232	0.007	0.027	1037.614	0.518	0.232	0.026	0.316	1037.614	-0.104	0.268	0.697	0.841
ZNF613	54.852	0.032	0.252	0.898	0.934	54.852	-0.221	0.253	0.383	0.629	54.852	-0.253	0.295	0.391	0.620
PRR5L	255.547	0.694	0.388	0.073	0.155	255.547	0.303	0.388	0.435	0.668	255.547	-0.391	0.448	0.383	0.612

CYBRD1	698.837	0.412	0.370	0.266	0.402	698.837	0.316	0.370	0.393	0.638	698.837	-0.096	0.428	0.822	0.913
NUP85	583.989	-0.294	0.231	0.203	0.330	583.989	-0.450	0.231	0.052	0.339	583.989	-0.156	0.267	0.561	0.752
NAA60	775.602	-0.038	0.177	0.831	0.889	775.602	-0.235	0.177	0.184	0.471	775.602	-0.197	0.204	0.335	0.570
TMC7	64.043	-0.084	0.248	0.735	0.821	64.043	-0.180	0.247	0.465	0.689	64.043	-0.096	0.289	0.739	0.866
MORN1	67.644	-0.521	0.244	0.033	0.084	67.644	0.059	0.231	0.799	0.896	67.644	0.580	0.279	0.037	0.152
TUSC3	1119.151	-0.088	0.103	0.391	0.530	1119.151	0.014	0.102	0.895	0.948	1119.151	0.102	0.119	0.392	0.620
PYROXD1	724.369	-0.585	0.236	0.013	0.042	724.369	-0.605	0.235	0.010	0.299	724.369	-0.019	0.273	0.943	0.974
ACTR5	118.472	-0.482	0.318	0.129	0.237	118.472	-0.775	0.319	0.015	0.306	118.472	-0.294	0.371	0.429	0.651
ATAD5	107.147	-0.506	0.375	0.177	0.299	107.147	-0.656	0.374	0.080	0.371	107.147	-0.150	0.435	0.730	0.860
MAGIX	9.391	-0.215	0.744	0.772	0.848	9.391	0.272	0.713	0.702	0.840	9.391	0.488	0.848	0.565	0.755
SETD6	695.822	0.209	0.241	0.385	0.524	695.822	-0.087	0.241	0.719	0.849	695.822	-0.296	0.278	0.288	0.523
MAB21L4	8.861	0.539	0.613	0.379	0.519	8.861	0.973	0.591	0.099	0.394	8.861	0.434	0.683	0.525	0.726
TCEAL4	1492.541	0.917	0.173	0.000	0.000	1492.541	-0.005	0.173	0.977	0.987	1492.541	-0.922	0.200	0.000	0.000
MRM1	380.227	-0.415	0.318	0.192	0.317	380.227	-0.433	0.318	0.173	0.463	380.227	-0.018	0.368	0.961	0.982
ADM2	83.201	-1.353	0.375	0.000	0.002	83.201	-0.271	0.358	0.448	0.677	83.201	1.082	0.430	0.012	0.073
MAP6D1	27.404	0.047	0.541	0.931	0.956	27.404	0.010	0.537	0.985	0.991	27.404	-0.036	0.626	0.954	0.979
UBXN8	256.901	-0.478	0.170	0.005	0.020	256.901	-0.168	0.167	0.315	0.578	256.901	0.310	0.197	0.115	0.305
DOK3	60.428	1.569	0.333	0.000	0.000	60.428	0.394	0.348	0.258	0.530	60.428	-1.175	0.386	0.002	0.024
TNIP3	211.828	-0.387	0.294	0.189	0.313	211.828	-0.646	0.294	0.028	0.321	211.828	-0.259	0.341	0.448	0.667
KIAA0319L	967.530	-0.048	0.234	0.837	0.893	967.530	0.365	0.233	0.118	0.409	967.530	0.413	0.270	0.126	0.320
COQ8B	643.423	0.215	0.193	0.266	0.402	643.423	0.463	0.192	0.016	0.306	643.423	0.248	0.223	0.265	0.500
CNTD2	10.727	3.584	1.016	0.000	0.003	10.727	2.412	1.068	0.024	0.316	10.727	-1.173	0.938	0.211	0.436
CNTNAP3	71.916	-0.450	0.276	0.103	0.200	71.916	-0.048	0.267	0.857	0.927	71.916	0.402	0.317	0.206	0.430
SLC35E1	2065.570	0.073	0.218	0.739	0.823	2065.570	0.237	0.217	0.275	0.545	2065.570	0.165	0.251	0.512	0.717
KAT6A	983.791	-0.649	0.176	0.000	0.002	983.791	-0.167	0.174	0.339	0.596	983.791	0.482	0.203	0.017	0.094
LINC00472	150.841	-0.811	0.283	0.004	0.017	150.841	-0.240	0.278	0.387	0.633	150.841	0.571	0.326	0.080	0.244
ZNF696	241.980	-0.536	0.264	0.042	0.101	241.980	-0.477	0.262	0.069	0.357	241.980	0.059	0.305	0.846	0.926
L2HGDH	259.742	-0.787	0.237	0.001	0.005	259.742	-0.124	0.233	0.596	0.775	259.742	0.663	0.273	0.015	0.086
C10orf95	52.326	0.003	0.243	0.991	0.995	52.326	0.194	0.236	0.411	0.650	52.326	0.191	0.278	0.491	0.701
DHDDS	389.601	-0.213	0.260	0.412	0.550	389.601	-0.432	0.260	0.097	0.392	389.601	-0.218	0.301	0.468	0.684
NOL10	683.763	-0.604	0.275	0.028	0.075	683.763	-0.580	0.275	0.035	0.322	683.763	0.024	0.318	0.940	0.973
PDZD7	162.188	1.171	0.293	0.000	0.001	162.188	0.506	0.295	0.086	0.379	162.188	-0.665	0.338	0.049	0.180
ERMP1	742.876	-0.477	0.274	0.081	0.167	742.876	0.187	0.272	0.493	0.708	742.876	0.664	0.316	0.035	0.146
PAQR6	33.315	2.290	0.625	0.000	0.002	33.315	0.696	0.638	0.275	0.545	33.315	-1.593	0.721	0.027	0.123
DENND1C	12.003	0.526	0.503	0.295	0.434	12.003	0.202	0.508	0.692	0.834	12.003	-0.325	0.582	0.577	0.762
CEP76	111.126	-0.608	0.243	0.012	0.040	111.126	-0.371	0.239	0.120	0.412	111.126	0.237	0.282	0.400	0.627
JADE1	832.517	-0.743	0.196	0.000	0.001	832.517	-0.457	0.195	0.019	0.306	832.517	0.285	0.226	0.207	0.431
DNAJC22	46.713	0.781	0.552	0.157	0.274	46.713	0.640	0.552	0.246	0.521	46.713	-0.142	0.635	0.824	0.913
ABCA11P	44.793	-0.790	0.331	0.017	0.050	44.793	-0.317	0.318	0.318	0.580	44.793	0.473	0.382	0.216	0.441
SCD5	311.801	0.529	0.300	0.077	0.161	311.801	0.092	0.300	0.759	0.872	311.801	-0.437	0.346	0.207	0.431
WDR76	283.566	-0.830	0.413	0.045	0.106	283.566	-0.966	0.413	0.019	0.306	283.566	-0.136	0.479	0.776	0.886
ATAT1	448.183	0.015	0.208	0.942	0.964	448.183	0.459	0.206	0.026	0.316	448.183	0.444	0.239	0.063	0.211
ZNF767P	36.085	0.421	0.380	0.268	0.404	36.085	0.580	0.375	0.121	0.413	36.085	0.159	0.434	0.714	0.851
WLS	4770.362	0.012	0.197	0.953	0.972	4770.362	0.148	0.197	0.453	0.680	4770.362	0.136	0.227	0.549	0.744
CPED1	332.441	-0.339	0.495	0.493	0.625	332.441	0.257	0.494	0.603	0.780	332.441	0.597	0.572	0.297	0.532
GRHL2	18.445	0.638	0.398	0.109	0.209	18.445	0.064	0.411	0.876	0.937	18.445	-0.574	0.466	0.218	0.444
TRMT2B	243.143	-0.768	0.204	0.000	0.001	243.143	-0.197	0.200	0.323	0.584	243.143	0.571	0.235	0.015	0.086
DSN1	218.079	-0.320	0.293	0.275	0.412	218.079	-0.504	0.293	0.086	0.379	218.079	-0.183	0.340	0.590	0.771
DNAJB14	486.926	-0.539	0.169	0.001	0.008	486.926	-0.224	0.167	0.181	0.469	486.926	0.316	0.195	0.106	0.290
POF1B	242.109	-0.352	0.375	0.348	0.489	242.109	0.348	0.373	0.351	0.605	242.109	0.700	0.432	0.105	0.289
TTC26	155.586	-0.263	0.169	0.119	0.223	155.586	-0.344	0.168	0.041	0.328	155.586	-0.081	0.197	0.683	0.832
PLEKHH3	1144.288	-0.046	0.251	0.854	0.905	1144.288	0.456	0.251	0.069	0.357	1144.288	0.503	0.290	0.083	0.250
STN1	464.729	-0.126	0.271	0.641	0.748	464.729	0.426	0.270	0.114	0.406	464.729	0.552	0.312	0.077	0.240
ELOVL7	321.252	0.943	0.170	0.000	0.000	321.252	-0.272	0.175	0.120	0.412	321.252	-1.215	0.199	0.000	0.000
LOC79999	16.369	0.729	0.604	0.227	0.359	16.369	1.034	0.591	0.080	0.371	16.369	0.305	0.679	0.653	0.813
CALD1	2626.687	-0.278	0.271	0.306	0.445	2626.687	0.186	0.271	0.492	0.707	2626.687	0.464	0.313	0.138	0.341
PSCA	147.983	0.112	0.391	0.775	0.850	147.983	0.436	0.389	0.263	0.536	147.983	0.324	0.451	0.473	0.687
GREB1L	10.343	-0.433	0.757	0.567	0.687	10.343	-0.396	0.750	0.597	0.776	10.343	0.037	0.880	0.966	0.984
PCNX2	353.741	0.253	0.212	0.232	0.364	353.741	0.309	0.211	0.143	0.435	353.741	0.056	0.244	0.819	0.911
ESRP2	59.374	0.535	0.266	0.044	0.105	59.374	0.156	0.269	0.563	0.751	59.374	-0.380	0.308	0.218	0.445
DOCK5	729.016	-0.133	0.256	0.604	0.717	729.016	-0.211	0.256	0.409	0.649	729.016	-0.078	0.296	0.791	0.896
TRAPPC13	345.652	-0.263	0.197	0.183	0.307	345.652	-0.255	0.197	0.194	0.478	345.652	0.008	0.228	0.973	0.987
C10orf88	132.871	-0.605	0.187	0.001	0.007	132.871	-0.098	0.179	0.585	0.768	132.871	0.508	0.215	0.018	0.096
TMEM156	384.818	-0.737	0.187	0.000	0.001	384.818	-0.056	0.183	0.760	0.872	384.818	0.681	0.215	0.002	0.018
RMI1	384.013	-1.160	0.212	0.000	0.000	384.013	-0.082	0.207	0.692	0.834	384.013	1.078	0.243	0.000	0.000
PSME3IP1	1055.909	0.086	0.213	0.687	0.784	1055.909	-0.391	0.213	0.067	0.354	1055.909	-0.477	0.246	0.053	0.189
PHC3	1235.681	-0.394	0.228	0.084	0.171	1235.681	0.188	0.227	0.407	0.648	1235.681	0.582	0.263	0.027	0.122

MINDY3	339.985	-0.663	0.205	0.001	0.007	339.985	-0.377	0.203	0.063	0.350	339.985	0.286	0.237	0.227	0.455
WWC2	718.141	-1.070	0.263	0.000	0.001	718.141	-0.507	0.262	0.053	0.342	718.141	0.563	0.304	0.064	0.213
DGLUCY	572.828	0.475	0.244	0.052	0.119	572.828	0.247	0.245	0.313	0.576	572.828	-0.228	0.282	0.419	0.643
NAA25	726.777	-0.939	0.211	0.000	0.000	726.777	-0.261	0.210	0.212	0.492	726.777	0.678	0.244	0.005	0.043
UBTD1	204.207	1.626	0.219	0.000	0.000	204.207	0.080	0.228	0.725	0.853	204.207	-1.545	0.256	0.000	0.000
FOXRED2	432.875	0.341	0.121	0.005	0.020	432.875	-0.240	0.123	0.051	0.339	432.875	-0.581	0.142	0.000	0.001
TMEM62	224.300	-0.307	0.248	0.217	0.347	224.300	-0.227	0.247	0.357	0.611	224.300	0.079	0.287	0.783	0.891
MYO15B	21.192	1.358	0.467	0.004	0.015	21.192	1.567	0.459	0.001	0.257	21.192	0.209	0.514	0.684	0.833
NRSN2	189.190	0.529	0.214	0.013	0.043	189.190	-0.195	0.217	0.370	0.619	189.190	-0.723	0.249	0.004	0.033
SLC8B1	701.673	0.033	0.204	0.870	0.916	701.673	-0.208	0.204	0.309	0.574	701.673	-0.241	0.236	0.307	0.543
PANK2	404.715	-0.442	0.205	0.031	0.080	404.715	-0.349	0.204	0.087	0.380	404.715	0.094	0.237	0.693	0.839
FBXL18	574.390	0.177	0.187	0.345	0.486	574.390	-0.264	0.188	0.160	0.451	574.390	-0.441	0.217	0.042	0.165
SEMA6D	141.822	-2.261	0.303	0.000	0.000	141.822	-0.181	0.271	0.504	0.715	141.822	2.080	0.342	0.000	0.000
CSRNP3	50.547	-0.391	0.564	0.488	0.621	50.547	0.178	0.559	0.750	0.867	50.547	0.569	0.650	0.381	0.611
TRPM3	31.831	-0.460	0.517	0.373	0.513	31.831	0.159	0.505	0.753	0.868	31.831	0.619	0.594	0.297	0.532
GPR157	277.580	-0.031	0.218	0.885	0.925	277.580	0.018	0.217	0.933	0.967	277.580	0.050	0.251	0.844	0.924
CEBPA-DT	10.439	0.695	0.543	0.201	0.327	10.439	0.950	0.529	0.073	0.362	10.439	0.255	0.607	0.675	0.828
PGAP1	190.007	-0.278	0.387	0.473	0.607	190.007	0.457	0.384	0.234	0.511	190.007	0.734	0.445	0.099	0.279
LRRTM4	9.790	-1.469	0.868	0.091	0.182	9.790	0.202	0.797	0.800	0.896	9.790	1.671	0.982	0.089	0.261
DCAF17	209.663	-0.565	0.184	0.002	0.010	209.663	-0.378	0.182	0.037	0.326	209.663	0.187	0.214	0.381	0.611
ADAMTS20	15.679	-0.757	0.613	0.217	0.347	15.679	0.404	0.579	0.486	0.703	15.679	1.160	0.696	0.096	0.273
CCDC15	23.801	-1.104	0.390	0.005	0.019	23.801	-0.356	0.359	0.322	0.583	23.801	0.748	0.448	0.095	0.271
TUBA4B	8.939	0.553	0.746	0.458	0.593	8.939	-0.329	0.775	0.671	0.822	8.939	-0.882	0.883	0.317	0.553
MZT2B	3248.358	0.903	0.289	0.002	0.009	3248.358	0.472	0.289	0.103	0.395	3248.358	-0.431	0.334	0.197	0.420
CALM1	2601.273	-0.080	0.192	0.676	0.775	2601.273	-0.456	0.192	0.017	0.306	2601.273	-0.376	0.221	0.089	0.262
ZNF614	169.303	-0.904	0.207	0.000	0.000	169.303	-0.317	0.200	0.113	0.405	169.303	0.587	0.239	0.014	0.081
BICC1	11.988	-0.621	0.535	0.246	0.379	11.988	0.799	0.472	0.090	0.385	11.988	1.419	0.591	0.016	0.090
BAIAP2L2	85.587	-0.194	0.348	0.578	0.696	85.587	-0.453	0.349	0.194	0.478	85.587	-0.260	0.407	0.523	0.725
PIF1	110.800	-0.143	0.289	0.621	0.732	110.800	0.386	0.284	0.175	0.464	110.800	0.528	0.332	0.111	0.298
VCPIP1	443.788	-0.215	0.133	0.108	0.207	443.788	-0.092	0.132	0.485	0.702	443.788	0.122	0.154	0.428	0.651
BBOF1	86.658	0.217	0.555	0.696	0.790	86.658	0.637	0.552	0.248	0.522	86.658	0.420	0.639	0.511	0.716
TRIM46	12.410	0.613	0.571	0.283	0.421	12.410	0.677	0.561	0.228	0.506	12.410	0.063	0.643	0.922	0.963
NR4A3	13.192	0.425	0.491	0.387	0.526	13.192	-0.841	0.550	0.126	0.419	13.192	-1.265	0.617	0.040	0.160
LRRC8E	231.063	0.528	0.250	0.035	0.088	231.063	0.083	0.251	0.741	0.862	231.063	-0.445	0.289	0.124	0.318
RPF1	910.287	-0.922	0.295	0.002	0.009	910.287	-0.627	0.295	0.033	0.322	910.287	0.295	0.341	0.387	0.615
ZNF703	182.410	0.674	0.279	0.016	0.048	182.410	-0.077	0.283	0.786	0.888	182.410	-0.751	0.324	0.021	0.103
PTGES2	1159.832	-0.050	0.335	0.881	0.923	1159.832	-0.413	0.335	0.218	0.498	1159.832	-0.363	0.387	0.349	0.582
SIKE1	561.666	-0.138	0.136	0.308	0.448	561.666	-0.066	0.135	0.623	0.792	561.666	0.072	0.157	0.647	0.809
FRAS1	38.426	0.179	0.346	0.605	0.718	38.426	0.699	0.335	0.037	0.324	38.426	0.520	0.392	0.184	0.404
THOC7	1254.767	0.664	0.214	0.002	0.010	1254.767	0.073	0.215	0.733	0.857	1254.767	-0.591	0.247	0.017	0.092
UXS1	693.831	0.143	0.137	0.296	0.434	693.831	-0.089	0.137	0.517	0.722	693.831	-0.232	0.159	0.143	0.349
SLC66A2	1018.838	0.327	0.205	0.111	0.211	1018.838	-0.171	0.205	0.405	0.646	1018.838	-0.498	0.237	0.036	0.147
ZC3H12A	1552.872	1.518	0.185	0.000	0.000	1552.872	-0.110	0.187	0.557	0.747	1552.872	-1.628	0.214	0.000	0.000
CENPT	659.838	0.206	0.163	0.206	0.334	659.838	0.235	0.162	0.147	0.439	659.838	0.029	0.188	0.875	0.940
EDC3	661.283	-0.683	0.135	0.000	0.000	661.283	-0.227	0.133	0.089	0.382	661.283	0.456	0.156	0.003	0.032
GOLGA2P10	407.908	-0.435	0.200	0.030	0.079	407.908	0.262	0.197	0.184	0.471	407.908	0.696	0.230	0.002	0.026
NAA15	1285.645	-1.134	0.347	0.001	0.006	1285.645	-0.968	0.347	0.005	0.297	1285.645	0.166	0.401	0.680	0.830
ASMTL-AS1	411.764	0.994	0.319	0.002	0.009	411.764	0.362	0.320	0.257	0.530	411.764	-0.631	0.368	0.086	0.256
PGGHG	310.324	1.234	0.262	0.000	0.000	310.324	0.419	0.264	0.112	0.405	310.324	-0.815	0.303	0.007	0.052
PRR36	22.954	1.681	0.839	0.045	0.107	22.954	0.882	0.846	0.297	0.565	22.954	-0.799	0.964	0.407	0.633
ABHD18	195.961	-0.240	0.198	0.226	0.358	195.961	0.174	0.194	0.371	0.620	195.961	0.414	0.228	0.069	0.224
CTC1	138.938	-0.475	0.211	0.024	0.067	138.938	-0.271	0.207	0.190	0.476	138.938	0.204	0.244	0.404	0.631
IFT74	419.024	0.105	0.167	0.530	0.656	419.024	-0.008	0.167	0.964	0.981	419.024	-0.112	0.193	0.560	0.752
DBF4B	213.804	-0.542	0.280	0.052	0.120	213.804	-0.233	0.278	0.402	0.644	213.804	0.310	0.323	0.338	0.572
SPSB1	202.857	1.146	0.189	0.000	0.000	202.857	0.153	0.196	0.436	0.669	202.857	-0.993	0.220	0.000	0.000
TEDC2	112.148	-0.528	0.490	0.282	0.419	112.148	-1.184	0.493	0.016	0.306	112.148	-0.656	0.571	0.251	0.483
MYO19	1646.750	-0.827	0.286	0.004	0.016	1646.750	-0.432	0.286	0.131	0.422	1646.750	0.395	0.331	0.232	0.461
CEP290	515.952	-0.139	0.184	0.450	0.585	515.952	-0.147	0.184	0.425	0.661	515.952	-0.007	0.213	0.972	0.986
TTI2	401.431	-1.192	0.336	0.000	0.003	401.431	-0.845	0.335	0.012	0.305	401.431	0.347	0.389	0.372	0.604
BRD3	1107.488	-0.044	0.098	0.651	0.756	1107.488	-0.096	0.098	0.325	0.586	1107.488	-0.052	0.113	0.648	0.810
TMEM134	451.828	1.299	0.349	0.000	0.002	451.828	0.659	0.350	0.060	0.350	451.828	-0.640	0.403	0.112	0.299
TMEM254	201.550	0.329	0.241	0.172	0.293	201.550	0.289	0.241	0.229	0.507	201.550	-0.040	0.278	0.885	0.945
RNF34	701.461	-0.528	0.224	0.019	0.055	701.461	-0.321	0.224	0.152	0.444	701.461	0.207	0.259	0.424	0.648
MUS81	535.069	-0.300	0.190	0.113	0.215	535.069	-0.250	0.189	0.187	0.473	535.069	0.051	0.219	0.817	0.910
FUZ	449.098	1.194	0.373	0.001	0.007	449.098	0.678	0.373	0.069	0.358	449.098	-0.516	0.430	0.230	0.459
FBXO11	930.768	-0.453	0.129	0.000	0.003	930.768	-0.132	0.127	0.302	0.568	930.768	0.321	0.149	0.030	0.133
CHD9	1145.134	-0.669	0.228	0.003	0.015	1145.134	-0.219	0.228	0.335	0.594	1145.134	0.449	0.264	0.088	0.260

FHOD3	109.645	0.384	0.230	0.094	0.187	109.645	0.197	0.230	0.392	0.637	109.645	-0.188	0.265	0.479	0.692
OPA3	627.977	0.126	0.153	0.409	0.548	627.977	-0.221	0.154	0.151	0.443	627.977	-0.347	0.178	0.051	0.184
SPG11	810.801	-0.478	0.195	0.014	0.045	810.801	0.123	0.194	0.527	0.729	810.801	0.601	0.225	0.008	0.055
PROSER1	727.324	-1.150	0.373	0.002	0.010	727.324	-0.889	0.372	0.017	0.306	727.324	0.261	0.431	0.544	0.740
NUP214	843.810	-0.284	0.102	0.005	0.021	843.810	-0.089	0.101	0.380	0.626	843.810	0.196	0.118	0.097	0.275
ARMC9	135.567	0.229	0.175	0.191	0.316	135.567	-0.159	0.177	0.368	0.618	135.567	-0.388	0.204	0.058	0.200
CCDC92	493.203	1.951	0.493	0.000	0.001	493.203	0.808	0.494	0.102	0.394	493.203	-1.144	0.569	0.045	0.170
TM2D3	473.507	-0.134	0.128	0.293	0.432	473.507	-0.002	0.126	0.990	0.994	473.507	0.132	0.147	0.369	0.601
ALPK1	378.603	-0.062	0.221	0.778	0.852	378.603	0.308	0.219	0.160	0.451	378.603	0.371	0.254	0.145	0.352
CFAP43	8.622	0.325	0.758	0.668	0.769	8.622	1.173	0.725	0.106	0.398	8.622	0.848	0.848	0.317	0.553
NAA50	3462.407	-1.175	0.305	0.000	0.001	3462.407	-0.728	0.305	0.017	0.306	3462.407	0.446	0.352	0.206	0.430
COQ10B	257.347	-0.059	0.298	0.843	0.897	257.347	-0.715	0.300	0.017	0.306	257.347	-0.656	0.347	0.059	0.202
ACSF2	162.546	1.137	0.599	0.058	0.128	162.546	0.895	0.599	0.135	0.426	162.546	-0.242	0.691	0.726	0.858
TARS2	583.268	0.125	0.162	0.438	0.574	583.268	-0.196	0.162	0.227	0.506	583.268	-0.321	0.188	0.086	0.256
RAB11FIP1	741.415	-0.023	0.277	0.934	0.958	741.415	0.310	0.276	0.262	0.535	741.415	0.333	0.319	0.297	0.533
NUBPL	234.923	-0.366	0.243	0.132	0.241	234.923	0.179	0.240	0.456	0.682	234.923	0.546	0.280	0.051	0.186
PAAF1	446.178	-0.055	0.175	0.753	0.835	446.178	0.160	0.174	0.358	0.611	446.178	0.215	0.202	0.287	0.522
ORAI2	643.607	0.673	0.162	0.000	0.000	643.607	-0.090	0.164	0.584	0.767	643.607	-0.762	0.188	0.000	0.001
RUFY1	849.353	0.474	0.125	0.000	0.001	849.353	0.003	0.126	0.981	0.989	849.353	-0.471	0.145	0.001	0.015
WDR26	2012.176	-0.037	0.146	0.801	0.869	2012.176	0.091	0.146	0.532	0.732	2012.176	0.128	0.168	0.448	0.667
FAAP100	533.961	0.100	0.152	0.511	0.640	533.961	-0.134	0.152	0.376	0.624	533.961	-0.234	0.176	0.183	0.402
PIGZ	42.593	0.912	0.548	0.096	0.190	42.593	0.633	0.549	0.249	0.523	42.593	-0.279	0.630	0.658	0.817
ELL3	60.741	0.446	0.282	0.113	0.214	60.741	0.532	0.278	0.056	0.344	60.741	0.085	0.322	0.791	0.896
CEP63	683.186	-0.154	0.128	0.231	0.364	683.186	-0.080	0.128	0.529	0.729	683.186	0.073	0.148	0.622	0.793
SLC35F5	889.723	0.151	0.123	0.219	0.349	889.723	0.131	0.122	0.283	0.553	889.723	-0.020	0.142	0.890	0.947
FAM214B	87.916	0.765	0.261	0.003	0.015	87.916	0.343	0.264	0.193	0.478	87.916	-0.422	0.301	0.161	0.375
C16orf70	251.504	0.018	0.153	0.904	0.939	251.504	-0.218	0.154	0.155	0.448	251.504	-0.237	0.178	0.185	0.404
TRIM45	17.832	0.387	0.413	0.348	0.489	17.832	-0.279	0.428	0.515	0.721	17.832	-0.666	0.490	0.174	0.390
ZNF430	88.240	0.000	0.215	1.000	1.000	88.240	-0.173	0.214	0.420	0.657	88.240	-0.173	0.250	0.489	0.700
EDEM3	623.728	-0.536	0.262	0.041	0.099	623.728	-0.248	0.261	0.343	0.600	623.728	0.288	0.303	0.341	0.575
STAM	586.025	-0.469	0.187	0.012	0.040	586.025	-0.407	0.187	0.029	0.322	586.025	0.062	0.217	0.774	0.885
HSD3B7	152.094	1.606	0.469	0.001	0.004	152.094	0.816	0.472	0.084	0.375	152.094	-0.790	0.539	0.143	0.349
ITPKC	1206.838	0.369	0.129	0.004	0.017	1206.838	0.304	0.128	0.018	0.306	1206.838	-0.065	0.148	0.662	0.819
GRPEL1	1149.692	-0.250	0.236	0.290	0.428	1149.692	-0.381	0.236	0.107	0.399	1149.692	-0.131	0.273	0.632	0.799
CDK5RAP3	661.805	0.176	0.176	0.317	0.457	661.805	0.241	0.176	0.170	0.460	661.805	0.065	0.203	0.750	0.872
MLLT10	673.801	-0.675	0.217	0.002	0.009	673.801	0.006	0.215	0.978	0.988	673.801	0.681	0.250	0.006	0.049
CUBN	30.455	1.011	0.439	0.021	0.060	30.455	0.714	0.440	0.105	0.398	30.455	-0.297	0.502	0.554	0.747
MTERF2	145.108	0.576	0.233	0.014	0.043	145.108	-0.058	0.236	0.807	0.899	145.108	-0.634	0.271	0.019	0.100
CCDC6	1648.247	-0.535	0.142	0.000	0.001	1648.247	0.053	0.141	0.709	0.843	1648.247	0.588	0.163	0.000	0.006
PLEKHO2	277.691	0.606	0.198	0.002	0.011	277.691	0.084	0.200	0.675	0.824	277.691	-0.522	0.230	0.023	0.111
WDCP	146.670	-0.480	0.231	0.037	0.092	146.670	0.082	0.225	0.716	0.848	146.670	0.562	0.265	0.034	0.143
TRABD	1068.764	-0.297	0.228	0.193	0.319	1068.764	-0.307	0.228	0.179	0.467	1068.764	-0.010	0.264	0.971	0.986
MED28	614.036	0.203	0.101	0.045	0.106	614.036	0.032	0.101	0.751	0.867	614.036	-0.171	0.117	0.144	0.350
FER1L4	70.249	2.658	0.696	0.000	0.001	70.249	1.429	0.700	0.041	0.329	70.249	-1.229	0.799	0.124	0.317
FLAD1	530.326	-0.294	0.354	0.407	0.545	530.326	-0.658	0.355	0.064	0.350	530.326	-0.364	0.410	0.374	0.606
NCOA4	2968.365	-0.566	0.191	0.003	0.013	2968.365	-0.060	0.191	0.754	0.868	2968.365	0.506	0.220	0.022	0.107
KLHL15	306.498	-0.886	0.254	0.000	0.003	306.498	-0.042	0.251	0.867	0.932	306.498	0.844	0.293	0.004	0.035
TET1	49.716	-0.145	0.617	0.815	0.878	49.716	0.230	0.613	0.707	0.842	49.716	0.375	0.711	0.598	0.776
LRRC27	134.774	0.212	0.337	0.531	0.656	134.774	0.510	0.335	0.128	0.420	134.774	0.298	0.388	0.442	0.662
EPC1	293.820	-0.372	0.179	0.037	0.092	293.820	-0.042	0.175	0.809	0.900	293.820	0.330	0.206	0.109	0.294
CPEB4	521.093	-1.147	0.229	0.000	0.000	521.093	-0.391	0.226	0.083	0.375	521.093	0.756	0.264	0.004	0.036
ZKSCAN3	97.638	-0.252	0.290	0.384	0.524	97.638	0.474	0.283	0.094	0.388	97.638	0.727	0.332	0.029	0.127
GKAP1	88.244	0.219	0.347	0.527	0.653	88.244	0.445	0.344	0.196	0.480	88.244	0.225	0.398	0.572	0.760
CXXC4	48.614	-0.879	0.627	0.161	0.280	48.614	0.527	0.614	0.391	0.635	48.614	1.406	0.720	0.051	0.184
SP6	15.932	-0.114	0.646	0.860	0.908	15.932	-0.393	0.648	0.545	0.740	15.932	-0.278	0.753	0.712	0.850
CEP70	1148.597	-0.504	0.237	0.033	0.085	1148.597	0.124	0.236	0.600	0.778	1148.597	0.628	0.273	0.022	0.106
CCDC68	93.169	-0.662	0.352	0.060	0.132	93.169	-0.645	0.350	0.065	0.354	93.169	0.017	0.409	0.968	0.985
PUS1	905.283	-0.461	0.364	0.206	0.334	905.283	-0.754	0.365	0.039	0.327	905.283	-0.294	0.421	0.486	0.697
ABTB1	208.994	1.065	0.443	0.016	0.049	208.994	0.476	0.444	0.283	0.553	208.994	-0.589	0.511	0.249	0.481
ULBP2	46.165	1.205	0.260	0.000	0.000	46.165	-0.023	0.285	0.936	0.968	46.165	-1.227	0.312	0.000	0.002
ULBP1	59.613	-0.441	0.266	0.097	0.192	59.613	0.291	0.249	0.242	0.518	59.613	0.732	0.301	0.015	0.086
DNAJC5	1752.316	-0.096	0.097	0.326	0.467	1752.316	-0.237	0.097	0.015	0.306	1752.316	-0.141	0.113	0.212	0.437
KCNIP4	13.734	-1.353	0.591	0.022	0.062	13.734	-0.302	0.538	0.575	0.760	13.734	1.051	0.674	0.119	0.310
WDR82	3177.281	-0.232	0.108	0.031	0.081	3177.281	-0.241	0.108	0.025	0.316	3177.281	-0.009	0.125	0.943	0.974
PABPC1L	46.391	0.429	0.390	0.271	0.408	46.391	0.284	0.390	0.466	0.690	46.391	-0.145	0.450	0.746	0.870
PNPLA3	38.748	0.516	0.322	0.109	0.209	38.748	0.229	0.324	0.478	0.698	38.748	-0.286	0.372	0.441	0.661
SLC25A16	107.826	0.027	0.247	0.912	0.945	107.826	-0.306	0.248	0.218	0.497	107.826	-0.333	0.288	0.247	0.478

TRAF3IP3	60.155	0.180	0.254	0.479	0.613	60.155	-0.063	0.255	0.805	0.898	60.155	-0.243	0.296	0.411	0.637
DCAF11	911.712	-0.104	0.166	0.530	0.656	911.712	0.169	0.166	0.306	0.572	911.712	0.274	0.192	0.153	0.363
ZSCAN16	21.323	0.628	0.358	0.079	0.164	21.323	-0.207	0.380	0.586	0.768	21.323	-0.835	0.428	0.051	0.185
REEP4	1470.049	0.065	0.247	0.792	0.863	1470.049	0.392	0.247	0.112	0.405	1470.049	0.327	0.285	0.252	0.484
COASY	1534.021	-0.014	0.229	0.953	0.971	1534.021	-0.250	0.229	0.276	0.547	1534.021	-0.236	0.265	0.373	0.605
WDR61	518.032	-0.520	0.290	0.072	0.153	518.032	-0.726	0.290	0.012	0.305	518.032	-0.206	0.335	0.540	0.737
TNKS2	1149.514	-0.998	0.248	0.000	0.001	1149.514	-0.236	0.247	0.339	0.597	1149.514	0.762	0.286	0.008	0.055
RNF39	387.960	1.004	0.261	0.000	0.001	387.960	0.225	0.262	0.392	0.637	387.960	-0.780	0.302	0.010	0.065
SHOC2	774.405	-0.633	0.149	0.000	0.000	774.405	-0.365	0.148	0.013	0.305	774.405	0.268	0.172	0.119	0.310
ADAM12	1162.777	-0.503	0.189	0.008	0.027	1162.777	0.011	0.188	0.954	0.977	1162.777	0.514	0.218	0.018	0.096
PDCD1LG2	41.396	-0.272	0.495	0.582	0.700	41.396	-0.993	0.503	0.048	0.337	41.396	-0.720	0.582	0.216	0.441
CD276	2474.466	0.539	0.149	0.000	0.002	2474.466	0.056	0.149	0.707	0.842	2474.466	-0.483	0.172	0.005	0.041
RASSF7	610.709	0.987	0.232	0.000	0.000	610.709	0.388	0.233	0.096	0.390	610.709	-0.599	0.268	0.025	0.117
CALM2	5459.395	0.217	0.135	0.108	0.207	5459.395	-0.158	0.135	0.244	0.519	5459.395	-0.375	0.156	0.016	0.090
PDHX	795.942	-0.122	0.101	0.227	0.359	795.942	-0.200	0.101	0.047	0.336	795.942	-0.078	0.117	0.507	0.713
FOSL1	1469.761	-0.354	0.714	0.619	0.730	1469.761	-1.943	0.714	0.007	0.297	1469.761	-1.589	0.825	0.054	0.192
CUL5	428.662	-0.739	0.238	0.002	0.010	428.662	0.030	0.236	0.898	0.948	428.662	0.770	0.275	0.005	0.041
UBXN6	2699.070	0.641	0.285	0.025	0.067	2699.070	0.636	0.285	0.026	0.316	2699.070	-0.005	0.329	0.987	0.993
TSGA10	23.803	0.544	0.488	0.265	0.401	23.803	0.246	0.490	0.616	0.788	23.803	-0.299	0.563	0.596	0.775
AKNA	404.202	-1.150	0.245	0.000	0.000	404.202	-0.270	0.242	0.263	0.535	404.202	0.880	0.282	0.002	0.020
SLC35G2	101.593	-0.274	0.258	0.288	0.426	101.593	-0.421	0.257	0.102	0.394	101.593	-0.147	0.301	0.625	0.795
ACAD10	279.979	0.047	0.167	0.779	0.853	279.979	0.227	0.165	0.170	0.459	279.979	0.180	0.192	0.349	0.582
SRCIN1	131.101	0.074	0.455	0.871	0.917	131.101	0.598	0.452	0.186	0.472	131.101	0.525	0.523	0.316	0.552
TTYH3	2751.704	0.484	0.140	0.001	0.004	2751.704	0.127	0.140	0.364	0.616	2751.704	-0.357	0.161	0.027	0.122
ARHGAP39	91.749	-0.086	0.338	0.800	0.868	91.749	-0.694	0.344	0.043	0.331	91.749	-0.609	0.398	0.126	0.320
PTP4A2	3893.035	0.046	0.145	0.749	0.832	3893.035	-0.169	0.145	0.244	0.519	3893.035	-0.215	0.167	0.199	0.422
SLC44A4	42.856	0.517	0.471	0.273	0.410	42.856	0.487	0.469	0.299	0.566	42.856	-0.029	0.541	0.957	0.980
VWA7	44.895	0.799	0.619	0.197	0.323	44.895	0.064	0.624	0.919	0.959	44.895	-0.735	0.717	0.305	0.541
LY6G5C	33.222	-0.557	0.613	0.364	0.505	33.222	0.071	0.600	0.905	0.952	33.222	0.628	0.705	0.373	0.605
PRR3	1558.288	-0.414	0.193	0.032	0.083	1558.288	-0.214	0.193	0.267	0.539	1558.288	0.200	0.223	0.370	0.602
THUMPD2	186.418	0.049	0.182	0.787	0.859	186.418	0.009	0.181	0.962	0.980	186.418	-0.041	0.210	0.847	0.926
TSEN2	293.756	-0.300	0.216	0.166	0.286	293.756	-0.363	0.216	0.093	0.388	293.756	-0.063	0.251	0.802	0.901
AARSD1	121.523	-0.340	0.263	0.196	0.322	121.523	-0.426	0.262	0.105	0.397	121.523	-0.086	0.306	0.780	0.889
PRR7	676.562	0.787	0.245	0.001	0.007	676.562	0.321	0.245	0.191	0.476	676.562	-0.466	0.283	0.099	0.279
KHDC1	102.480	-0.238	0.222	0.284	0.422	102.480	-0.094	0.218	0.666	0.818	102.480	0.143	0.256	0.576	0.762
NDFIP1	1583.473	0.088	0.111	0.431	0.568	1583.473	-0.162	0.111	0.146	0.438	1583.473	-0.250	0.129	0.053	0.189
THAP7	297.282	-0.327	0.330	0.322	0.462	297.282	-0.450	0.330	0.173	0.462	297.282	-0.123	0.382	0.748	0.871
STARD5	43.609	0.340	0.417	0.414	0.552	43.609	0.504	0.413	0.222	0.501	43.609	0.164	0.478	0.732	0.861
CPTP	582.868	0.407	0.156	0.009	0.032	582.868	0.035	0.157	0.823	0.908	582.868	-0.371	0.181	0.040	0.159
LIMD2	41.722	1.846	0.322	0.000	0.000	41.722	-0.158	0.354	0.654	0.811	41.722	-2.005	0.387	0.000	0.000
TMEM177	286.142	-0.240	0.354	0.498	0.629	286.142	-0.666	0.354	0.060	0.350	286.142	-0.426	0.410	0.298	0.534
B9D2	153.463	0.648	0.257	0.012	0.038	153.463	0.293	0.258	0.257	0.529	153.463	-0.355	0.297	0.231	0.459
CYB5B	1815.548	0.210	0.159	0.187	0.311	1815.548	-0.394	0.160	0.014	0.306	1815.548	-0.603	0.184	0.001	0.014
ZNF34	63.218	-0.092	0.257	0.721	0.810	63.218	0.196	0.250	0.433	0.667	63.218	0.288	0.295	0.329	0.563
USP5	1348.922	-0.096	0.192	0.616	0.727	1348.922	-0.404	0.192	0.036	0.324	1348.922	-0.307	0.222	0.166	0.381
COL18A1	1648.247	1.388	0.225	0.000	0.000	1648.247	0.134	0.226	0.553	0.745	1648.247	-1.254	0.260	0.000	0.000
INTS5	512.930	-0.259	0.161	0.107	0.205	512.930	-0.100	0.159	0.529	0.730	512.930	0.159	0.186	0.392	0.620
MLF2	2620.298	0.494	0.146	0.001	0.004	2620.298	-0.056	0.146	0.704	0.841	2620.298	-0.550	0.169	0.001	0.014
CMIP	1940.870	-0.119	0.172	0.489	0.622	1940.870	0.099	0.171	0.565	0.752	1940.870	0.217	0.198	0.272	0.507
CALM3	4945.569	0.000	0.137	1.000	1.000	4945.569	0.061	0.137	0.656	0.813	4945.569	0.061	0.158	0.700	0.843
CEP44	234.720	-0.478	0.203	0.019	0.055	234.720	0.021	0.200	0.917	0.959	234.720	0.499	0.234	0.033	0.140
ZNF436	231.771	-0.118	0.221	0.591	0.706	231.771	0.229	0.218	0.293	0.560	231.771	0.348	0.254	0.170	0.387
SSPN	16.026	0.871	0.766	0.256	0.390	16.026	0.484	0.770	0.530	0.730	16.026	-0.387	0.882	0.661	0.819
EEPD1	8.969	1.629	0.554	0.003	0.014	8.969	-0.290	0.636	0.648	0.808	8.969	-1.919	0.684	0.005	0.041
DDHD1	323.913	-0.473	0.238	0.047	0.110	323.913	-0.464	0.237	0.051	0.338	323.913	0.009	0.276	0.973	0.986
DUSP16	743.495	-0.261	0.157	0.097	0.192	743.495	-0.022	0.156	0.886	0.943	743.495	0.238	0.182	0.189	0.410
ZFP91	1467.819	-1.020	0.282	0.000	0.002	1467.819	-0.470	0.282	0.096	0.390	1467.819	0.550	0.326	0.092	0.266
APOL6	1025.553	0.243	0.202	0.230	0.362	1025.553	-0.321	0.203	0.113	0.405	1025.553	-0.564	0.234	0.016	0.089
APOL3	624.896	1.452	0.485	0.003	0.013	624.896	0.721	0.486	0.138	0.429	624.896	-0.731	0.560	0.192	0.414
KMT2D	1166.003	-0.369	0.195	0.059	0.130	1166.003	-0.302	0.195	0.121	0.413	1166.003	0.067	0.225	0.767	0.881
SH3BP5L	531.789	0.138	0.132	0.298	0.437	531.789	-0.055	0.132	0.677	0.825	531.789	-0.193	0.153	0.208	0.433
GRIP2	120.437	2.226	0.409	0.000	0.000	120.437	0.643	0.418	0.124	0.416	120.437	-1.583	0.472	0.001	0.011
KDM7A	396.132	-1.441	0.422	0.001	0.004	396.132	-0.990	0.420	0.018	0.306	396.132	0.451	0.488	0.356	0.590
SETD7	2498.734	-0.796	0.237	0.001	0.005	2498.734	-0.261	0.237	0.271	0.542	2498.734	0.535	0.274	0.051	0.185
LNPK	765.757	0.138	0.311	0.659	0.762	765.757	0.363	0.311	0.244	0.519	765.757	0.225	0.359	0.531	0.731
AAAS	756.032	0.167	0.161	0.298	0.437	756.032	-0.108	0.161	0.503	0.715	756.032	-0.275	0.186	0.139	0.343
ZNRD1ASP	1007.892	0.063	0.146	0.668	0.769	1007.892	0.073	0.146	0.616	0.788	1007.892	0.011	0.169	0.950	0.977

PRRT1	209.082	0.640	0.370	0.084	0.171	209.082	0.559	0.369	0.130	0.422	209.082	-0.081	0.426	0.849	0.927
HCG4B	103.722	0.686	0.524	0.190	0.315	103.722	0.781	0.523	0.135	0.426	103.722	0.095	0.603	0.874	0.940
FXR1	5400.204	-0.067	0.190	0.724	0.813	5400.204	0.213	0.190	0.261	0.534	5400.204	0.281	0.219	0.201	0.425
YEATS4	414.563	-0.551	0.190	0.004	0.016	414.563	-0.208	0.188	0.269	0.540	414.563	0.343	0.219	0.117	0.307
ILKAP	406.816	-0.140	0.317	0.659	0.762	406.816	-0.622	0.318	0.051	0.338	406.816	-0.482	0.367	0.190	0.411
HMGA2	85.283	0.680	0.385	0.078	0.162	85.283	-0.262	0.397	0.509	0.718	85.283	-0.942	0.453	0.037	0.152
CDK2AP1	1360.999	0.772	0.250	0.002	0.010	1360.999	-0.307	0.251	0.222	0.501	1360.999	-1.078	0.290	0.000	0.004
ACTN4	14277.562	0.643	0.140	0.000	0.000	14277.562	-0.133	0.140	0.341	0.598	14277.562	-0.776	0.161	0.000	0.000
IFT88	120.003	-0.559	0.166	0.001	0.005	120.003	0.015	0.156	0.923	0.961	120.003	0.574	0.190	0.002	0.025
SLC25A32	589.929	-1.211	0.356	0.001	0.004	589.929	-1.020	0.355	0.004	0.297	589.929	0.191	0.412	0.643	0.806
CLPTM1L	6098.651	-0.682	0.266	0.010	0.035	6098.651	-0.537	0.266	0.044	0.331	6098.651	0.144	0.307	0.638	0.803
PABPN1	1340.560	0.679	0.254	0.007	0.027	1340.560	-0.083	0.254	0.744	0.863	1340.560	-0.762	0.293	0.009	0.062
CALR	15170.431	0.182	0.271	0.503	0.633	15170.431	-0.451	0.271	0.096	0.391	15170.431	-0.633	0.313	0.043	0.167
ANP32A	3321.015	-0.457	0.168	0.006	0.024	3321.015	-0.370	0.168	0.027	0.321	3321.015	0.088	0.194	0.652	0.813
OR51E2	28.060	0.088	0.319	0.783	0.856	28.060	0.043	0.316	0.892	0.946	28.060	-0.045	0.369	0.903	0.955
CALU	3666.146	-0.305	0.253	0.229	0.360	3666.146	-0.371	0.253	0.143	0.434	3666.146	-0.066	0.292	0.821	0.912
NPRL3	1839.084	0.444	0.178	0.012	0.040	1839.084	0.239	0.178	0.178	0.467	1839.084	-0.205	0.205	0.319	0.554
GAN	96.993	-0.756	0.396	0.056	0.126	96.993	-0.648	0.393	0.099	0.394	96.993	0.108	0.459	0.814	0.908
CAMK4	75.736	0.573	0.290	0.048	0.112	75.736	0.115	0.292	0.694	0.835	75.736	-0.458	0.336	0.172	0.389
SLC7A5	18814.880	-2.047	0.200	0.000	0.000	18814.880	-0.396	0.200	0.048	0.337	18814.880	1.651	0.231	0.000	0.000
TAF15	12844.529	-0.554	0.223	0.013	0.041	12844.529	-0.361	0.223	0.105	0.398	12844.529	0.193	0.257	0.454	0.672
POLR2M	702.196	0.045	0.138	0.744	0.828	702.196	0.061	0.137	0.658	0.813	702.196	0.016	0.159	0.921	0.963
PTDSS2	1043.449	0.546	0.161	0.001	0.004	1043.449	-0.050	0.161	0.757	0.871	1043.449	-0.596	0.186	0.001	0.017
GPR63	73.203	-0.967	0.299	0.001	0.007	73.203	-0.276	0.287	0.337	0.595	73.203	0.691	0.344	0.044	0.170
SYNC	862.309	0.836	0.246	0.001	0.004	862.309	-0.100	0.247	0.686	0.831	862.309	-0.936	0.285	0.001	0.014
HM13	2404.696	0.649	0.164	0.000	0.001	2404.696	-0.073	0.165	0.660	0.815	2404.696	-0.721	0.190	0.000	0.003
MOB2	443.487	0.294	0.167	0.079	0.163	443.487	0.088	0.168	0.598	0.777	443.487	-0.206	0.194	0.287	0.522
ITFG1	666.564	-0.288	0.202	0.153	0.269	666.564	-0.040	0.201	0.843	0.918	666.564	0.248	0.233	0.287	0.522
SGPP1	338.876	-0.212	0.238	0.375	0.515	338.876	-0.328	0.238	0.169	0.459	338.876	-0.116	0.276	0.675	0.828
SLC38A1	10980.233	-0.658	0.181	0.000	0.002	10980.233	-0.262	0.181	0.149	0.441	10980.233	0.396	0.209	0.058	0.201
TMX1	1288.231	-0.780	0.167	0.000	0.000	1288.231	-0.284	0.166	0.088	0.380	1288.231	0.496	0.193	0.010	0.067
GDPD5	74.911	1.725	0.418	0.000	0.000	74.911	0.696	0.423	0.100	0.394	74.911	-1.029	0.481	0.033	0.139
FBXO38	505.088	-0.533	0.127	0.000	0.000	505.088	-0.153	0.125	0.220	0.500	505.088	0.380	0.147	0.010	0.065
TDRD3	223.366	0.228	0.183	0.213	0.342	223.366	0.128	0.182	0.482	0.701	223.366	-0.100	0.211	0.637	0.803
VOPP1	1215.594	-0.093	0.161	0.561	0.682	1215.594	-0.335	0.161	0.038	0.327	1215.594	-0.241	0.186	0.195	0.417
FAM49A	27.716	0.868	0.504	0.085	0.173	27.716	0.351	0.508	0.489	0.705	27.716	-0.516	0.582	0.375	0.607
RCC1L	1313.391	-0.331	0.168	0.049	0.113	1313.391	-0.290	0.168	0.084	0.376	1313.391	0.041	0.194	0.832	0.918
YIPF5	1444.827	-0.494	0.161	0.002	0.010	1444.827	-0.207	0.161	0.199	0.482	1444.827	0.288	0.186	0.123	0.315
INTS14	675.286	-0.698	0.166	0.000	0.000	675.286	-0.158	0.164	0.336	0.594	675.286	0.541	0.191	0.005	0.040
FAM117A	76.896	0.594	0.415	0.152	0.267	76.896	0.520	0.414	0.209	0.490	76.896	-0.074	0.477	0.876	0.941
TRIM11	757.554	0.045	0.188	0.812	0.877	757.554	-0.129	0.188	0.494	0.709	757.554	-0.173	0.217	0.425	0.649
LMAN2L	524.265	0.039	0.189	0.835	0.891	524.265	0.023	0.188	0.901	0.950	524.265	-0.016	0.218	0.942	0.973
C1orf21	1952.899	0.238	0.332	0.474	0.608	1952.899	0.363	0.332	0.275	0.546	1952.899	0.125	0.384	0.746	0.869
NDEL1	499.854	-0.271	0.183	0.139	0.250	499.854	-0.123	0.182	0.499	0.712	499.854	0.148	0.212	0.486	0.697
CSRNP2	445.144	-0.462	0.177	0.009	0.031	445.144	-0.365	0.176	0.037	0.326	445.144	0.097	0.205	0.636	0.802
TXNDC5	566.441	-1.416	0.400	0.000	0.003	566.441	-0.390	0.398	0.326	0.587	566.441	1.025	0.461	0.026	0.121
ACTL8	27.921	-1.602	0.559	0.004	0.017	27.921	-1.504	0.551	0.006	0.297	27.921	0.098	0.658	0.881	0.943
CLPB	518.816	-0.022	0.218	0.918	0.948	518.816	-0.371	0.219	0.090	0.384	518.816	-0.348	0.253	0.169	0.384
MIR600HG	28.869	0.269	0.476	0.572	0.691	28.869	0.159	0.475	0.738	0.860	28.869	-0.110	0.549	0.842	0.923
PDRG1	341.363	-0.370	0.283	0.191	0.316	341.363	-0.679	0.283	0.016	0.306	341.363	-0.309	0.328	0.346	0.580
ANKRD13C	505.185	-0.355	0.160	0.027	0.072	505.185	-0.193	0.159	0.225	0.504	505.185	0.162	0.185	0.382	0.611
APOLD1	193.349	-0.558	0.924	0.546	0.670	193.349	0.603	0.922	0.513	0.720	193.349	1.161	1.067	0.276	0.511
CCDC130	384.903	0.321	0.284	0.259	0.395	384.903	-0.037	0.285	0.898	0.948	384.903	-0.358	0.329	0.277	0.511
GFOD2	443.821	0.160	0.151	0.288	0.426	443.821	-0.172	0.152	0.256	0.529	443.821	-0.333	0.175	0.058	0.200
PLA2G12A	421.355	0.015	0.238	0.950	0.970	421.355	0.230	0.237	0.333	0.592	421.355	0.215	0.274	0.434	0.656
CDADC1	149.260	-0.095	0.172	0.583	0.700	149.260	0.044	0.169	0.795	0.893	149.260	0.138	0.198	0.486	0.697
TRIM8	1851.246	0.092	0.114	0.417	0.555	1851.246	0.050	0.114	0.658	0.814	1851.246	-0.042	0.131	0.749	0.871
URM1	944.698	-0.154	0.138	0.262	0.397	944.698	0.036	0.137	0.792	0.892	944.698	0.191	0.159	0.230	0.459
LBH	250.438	3.171	0.450	0.000	0.000	250.438	0.886	0.454	0.051	0.338	250.438	-2.285	0.519	0.000	0.000
NECTIN4	317.863	0.438	0.494	0.376	0.516	317.863	0.621	0.494	0.208	0.490	317.863	0.184	0.571	0.748	0.870
FIP1L1	551.776	-0.380	0.243	0.118	0.221	551.776	-0.481	0.243	0.048	0.337	551.776	-0.101	0.281	0.721	0.855
SNX27	531.174	0.269	0.112	0.017	0.050	531.174	0.360	0.111	0.001	0.297	531.174	0.091	0.129	0.481	0.694
COIL	384.873	-0.054	0.131	0.681	0.779	384.873	-0.162	0.131	0.214	0.493	384.873	-0.109	0.152	0.475	0.689
FAM83D	569.206	-0.605	0.254	0.017	0.051	569.206	0.129	0.252	0.610	0.785	569.206	0.734	0.293	0.012	0.074
ANP32E	3292.383	-0.608	0.118	0.000	0.000	3292.383	-0.265	0.117	0.024	0.316	3292.383	0.343	0.136	0.011	0.071
NIPA2	1244.413	-0.911	0.349	0.009	0.031	1244.413	-1.052	0.349	0.003	0.297	1244.413	-0.141	0.403	0.727	0.859
CAB39L	64.822	0.048	0.267	0.856	0.906	64.822	0.130	0.263	0.622	0.792	64.822	0.081	0.307	0.791	0.896

ITM2C	604.288	0.263	0.233	0.260	0.395	604.288	0.328	0.233	0.159	0.451	604.288	0.065	0.269	0.809	0.904
TSPAN14	977.484	0.203	0.149	0.175	0.296	977.484	0.072	0.149	0.631	0.798	977.484	-0.131	0.173	0.448	0.668
CDT1	1499.944	-0.023	0.316	0.943	0.965	1499.944	0.590	0.316	0.062	0.350	1499.944	0.613	0.365	0.093	0.268
KAZALD1	140.674	0.499	0.293	0.089	0.179	140.674	0.531	0.292	0.069	0.357	140.674	0.032	0.337	0.925	0.965
UNC93B1	689.997	0.646	0.217	0.003	0.013	689.997	0.366	0.217	0.091	0.386	689.997	-0.280	0.250	0.262	0.496
DIAPH3	730.636	-0.948	0.252	0.000	0.001	730.636	-0.377	0.251	0.133	0.425	730.636	0.571	0.291	0.050	0.182
TRMT1L	532.654	-0.526	0.202	0.009	0.032	532.654	-0.043	0.200	0.831	0.913	532.654	0.484	0.233	0.038	0.153
TSC22D4	1607.791	0.675	0.211	0.001	0.007	1607.791	0.463	0.211	0.028	0.321	1607.791	-0.212	0.243	0.384	0.613
TSSK3	34.816	1.471	0.624	0.018	0.054	34.816	1.212	0.625	0.053	0.341	34.816	-0.259	0.713	0.716	0.853
MAP1LC3B	2029.589	-0.062	0.188	0.742	0.826	2029.589	-0.306	0.188	0.104	0.397	2029.589	-0.244	0.217	0.262	0.496
AKAP1	1526.748	-0.625	0.177	0.000	0.003	1526.748	-0.068	0.177	0.701	0.839	1526.748	0.557	0.205	0.007	0.049
CCNL2	728.161	0.902	0.349	0.010	0.033	728.161	0.043	0.349	0.901	0.950	728.161	-0.859	0.403	0.033	0.140
VMP1	997.669	-0.382	0.200	0.056	0.125	997.669	-0.280	0.199	0.161	0.452	997.669	0.102	0.231	0.658	0.817
C6orf62	1945.899	0.178	0.119	0.137	0.247	1945.899	0.029	0.119	0.811	0.901	1945.899	-0.149	0.138	0.280	0.514
ISCA1	930.676	-0.189	0.112	0.092	0.184	930.676	-0.110	0.112	0.326	0.586	930.676	0.080	0.130	0.540	0.737
REXO5	103.734	-0.885	0.192	0.000	0.000	103.734	-0.148	0.179	0.409	0.649	103.734	0.737	0.219	0.001	0.011
AMN	453.257	0.591	0.189	0.002	0.009	453.257	0.073	0.190	0.700	0.839	453.257	-0.518	0.219	0.018	0.095
CAMK2D	814.580	-0.137	0.201	0.493	0.625	814.580	0.234	0.200	0.243	0.518	814.580	0.371	0.232	0.109	0.295
DOCK8	19.354	-0.243	0.486	0.616	0.727	19.354	-0.018	0.474	0.970	0.984	19.354	0.226	0.560	0.687	0.835
PPP1R14C	640.677	0.918	0.137	0.000	0.000	640.677	0.128	0.139	0.355	0.609	640.677	-0.790	0.159	0.000	0.000
MADCAM1	11.006	0.753	0.533	0.157	0.274	11.006	0.482	0.537	0.369	0.619	11.006	-0.272	0.608	0.655	0.815
SF3A2	2059.927	0.561	0.202	0.006	0.021	2059.927	0.124	0.203	0.539	0.736	2059.927	-0.437	0.234	0.062	0.208
ELL	936.503	-0.055	0.139	0.692	0.787	936.503	-0.038	0.138	0.782	0.885	936.503	0.017	0.160	0.917	0.961
TRIM7	640.948	-0.011	0.267	0.968	0.980	640.948	0.358	0.266	0.178	0.467	640.948	0.369	0.308	0.231	0.459
NUAK2	116.853	0.058	0.265	0.827	0.886	116.853	0.105	0.264	0.691	0.834	116.853	0.047	0.306	0.879	0.942
TIGD6	111.325	-0.013	0.160	0.933	0.958	111.325	-0.108	0.159	0.497	0.711	111.325	-0.095	0.187	0.612	0.786
RNF170	206.004	-0.008	0.266	0.977	0.986	206.004	0.192	0.265	0.468	0.691	206.004	0.200	0.307	0.516	0.720
ADAMTS12	461.572	0.514	0.315	0.102	0.199	461.572	-0.345	0.316	0.275	0.545	461.572	-0.860	0.365	0.018	0.096
ADAMTS10	72.292	1.701	0.630	0.007	0.026	72.292	0.765	0.634	0.227	0.506	72.292	-0.936	0.726	0.198	0.421
SLCO5A1	113.420	-1.308	0.410	0.001	0.008	113.420	-0.653	0.403	0.105	0.398	113.420	0.655	0.473	0.166	0.381
CAMK2G	553.636	0.161	0.247	0.514	0.643	553.636	0.490	0.246	0.046	0.335	553.636	0.329	0.284	0.247	0.478
NETO2	785.542	-0.617	0.233	0.008	0.029	785.542	-0.498	0.233	0.032	0.322	785.542	0.119	0.270	0.659	0.818
NETO1	144.564	-1.171	0.238	0.000	0.000	144.564	-0.248	0.225	0.271	0.542	144.564	0.923	0.273	0.001	0.010
VANGL1	627.503	-0.081	0.199	0.685	0.782	627.503	-0.098	0.198	0.621	0.791	627.503	-0.018	0.230	0.939	0.973
TRIM56	1096.673	0.591	0.216	0.006	0.023	1096.673	0.131	0.216	0.543	0.738	1096.673	-0.459	0.249	0.065	0.215
SBF2	927.412	-0.519	0.248	0.036	0.090	927.412	0.168	0.247	0.494	0.709	927.412	0.687	0.286	0.016	0.089
RNF146	446.361	-0.185	0.111	0.095	0.189	446.361	-0.053	0.109	0.626	0.795	446.361	0.132	0.128	0.303	0.538
SPRY4	1588.901	-0.979	0.545	0.072	0.153	1588.901	-1.488	0.545	0.006	0.297	1588.901	-0.509	0.629	0.419	0.643
TMEM14B	1095.479	0.330	0.178	0.064	0.139	1095.479	-0.063	0.179	0.722	0.852	1095.479	-0.394	0.206	0.056	0.197
ZNF205-AS1	127.805	-0.192	0.204	0.346	0.487	127.805	0.456	0.196	0.020	0.310	127.805	0.648	0.232	0.005	0.043
SFXN3	1233.043	0.730	0.265	0.006	0.022	1233.043	0.424	0.265	0.110	0.402	1233.043	-0.306	0.305	0.316	0.552
ZNF611	50.741	-0.095	0.277	0.733	0.820	50.741	-0.573	0.283	0.043	0.331	50.741	-0.478	0.329	0.146	0.353
MED25	869.678	0.130	0.141	0.355	0.495	869.678	0.113	0.140	0.419	0.656	869.678	-0.017	0.162	0.918	0.962
SHARPIN	942.950	0.307	0.167	0.067	0.144	942.950	0.086	0.168	0.609	0.784	942.950	-0.221	0.193	0.253	0.485
ARPC5L	853.814	-0.475	0.300	0.113	0.214	853.814	-0.601	0.300	0.045	0.334	853.814	-0.125	0.347	0.718	0.853
ISG20L2	786.023	-0.469	0.228	0.040	0.097	786.023	-0.495	0.228	0.030	0.322	786.023	-0.026	0.264	0.920	0.963
RAB1B	3204.977	0.084	0.120	0.484	0.617	3204.977	-0.083	0.120	0.487	0.704	3204.977	-0.167	0.139	0.227	0.456
LAS1L	1143.324	-0.400	0.292	0.171	0.291	1143.324	-0.733	0.292	0.012	0.305	1143.324	-0.333	0.337	0.324	0.558
HYI	115.019	0.733	0.199	0.000	0.002	115.019	0.254	0.202	0.208	0.490	115.019	-0.479	0.230	0.037	0.151
FAHD1	772.208	0.376	0.187	0.044	0.105	772.208	-0.277	0.188	0.140	0.431	772.208	-0.653	0.216	0.003	0.026
SYMPK	2214.630	0.125	0.117	0.284	0.422	2214.630	-0.057	0.117	0.626	0.795	2214.630	-0.183	0.135	0.178	0.395
QTRT1	764.550	0.151	0.169	0.370	0.511	764.550	0.089	0.169	0.597	0.776	764.550	-0.062	0.195	0.750	0.872
SLIRP	718.447	0.220	0.266	0.407	0.546	718.447	-0.235	0.266	0.379	0.626	718.447	-0.455	0.308	0.139	0.343
SLC25A28	356.227	0.745	0.197	0.000	0.001	356.227	0.068	0.198	0.733	0.857	356.227	-0.677	0.228	0.003	0.029
CAMLG	1816.167	0.200	0.251	0.425	0.562	1816.167	0.285	0.251	0.256	0.529	1816.167	0.085	0.290	0.770	0.883
CLPP	1499.264	0.065	0.206	0.751	0.834	1499.264	-0.121	0.206	0.558	0.748	1499.264	-0.186	0.238	0.435	0.656
ABHD17A	1698.138	0.525	0.162	0.001	0.007	1698.138	0.140	0.163	0.390	0.635	1698.138	-0.386	0.188	0.040	0.158
CABLES2	91.629	0.280	0.213	0.188	0.313	91.629	-0.053	0.215	0.807	0.899	91.629	-0.333	0.249	0.181	0.399
SEH1L	834.623	-0.585	0.201	0.004	0.015	834.623	-0.473	0.200	0.018	0.306	834.623	0.112	0.232	0.629	0.798
DPF1	25.678	-0.328	0.494	0.507	0.637	25.678	-0.990	0.506	0.051	0.338	25.678	-0.663	0.588	0.260	0.494
KIF18A	352.323	-0.863	0.193	0.000	0.000	352.323	-0.254	0.189	0.180	0.469	352.323	0.609	0.222	0.006	0.047
HDHD3	206.294	0.936	0.248	0.000	0.001	206.294	0.405	0.249	0.104	0.397	206.294	-0.531	0.286	0.063	0.211
MKKS	663.282	-0.567	0.201	0.005	0.019	663.282	-0.279	0.200	0.162	0.453	663.282	0.288	0.232	0.215	0.440
NCOA3	1325.864	-0.368	0.271	0.175	0.297	1325.864	0.072	0.271	0.790	0.890	1325.864	0.440	0.313	0.160	0.373
NRIP1	587.126	-0.930	0.280	0.001	0.005	587.126	-0.420	0.279	0.132	0.424	587.126	0.509	0.324	0.116	0.305
CHAF1B	384.504	-0.580	0.411	0.158	0.275	384.504	-1.086	0.411	0.008	0.297	384.504	-0.506	0.475	0.287	0.522
GATD3A	1040.515	0.232	0.205	0.257	0.392	1040.515	0.238	0.205	0.244	0.519	1040.515	0.006	0.237	0.979	0.989

CANX	23160.806	-0.381	0.188	0.043	0.104	23160.806	0.077	0.188	0.682	0.827	23160.806	0.458	0.217	0.035	0.146
LZTR1	639.105	-0.343	0.251	0.172	0.293	639.105	-0.439	0.251	0.080	0.371	639.105	-0.097	0.290	0.739	0.866
CLTCL1	347.915	0.127	0.126	0.315	0.455	347.915	0.433	0.124	0.000	0.253	347.915	0.307	0.144	0.034	0.142
CAPG	374.383	1.036	0.279	0.000	0.002	374.383	0.315	0.280	0.262	0.534	374.383	-0.722	0.322	0.025	0.117
ESS2	332.562	0.426	0.210	0.043	0.102	332.562	-0.203	0.212	0.339	0.597	332.562	-0.629	0.244	0.010	0.066
GTPBP6	2519.217	0.110	0.126	0.382	0.521	2519.217	-0.065	0.126	0.605	0.781	2519.217	-0.175	0.146	0.228	0.457
PUDP	80.887	-0.030	0.247	0.904	0.939	80.887	-0.299	0.248	0.229	0.507	80.887	-0.269	0.289	0.352	0.586
AKAP17A	2292.530	0.097	0.287	0.735	0.821	2292.530	-0.158	0.287	0.582	0.765	2292.530	-0.255	0.331	0.441	0.661
PNPLA4	103.903	0.489	0.290	0.092	0.184	103.903	0.371	0.290	0.200	0.483	103.903	-0.117	0.334	0.725	0.858
CAPN1	3670.995	0.301	0.142	0.034	0.086	3670.995	0.182	0.142	0.199	0.482	3670.995	-0.119	0.164	0.469	0.684
ZRSR2	200.706	0.303	0.266	0.255	0.389	200.706	-0.259	0.268	0.334	0.593	200.706	-0.562	0.309	0.069	0.224
USP11	814.822	-0.172	0.113	0.128	0.235	814.822	0.054	0.112	0.629	0.797	814.822	0.226	0.130	0.083	0.250
USP9X	3017.437	-0.303	0.128	0.018	0.053	3017.437	-0.045	0.128	0.726	0.853	3017.437	0.258	0.148	0.080	0.245
CAPN2	5002.621	-0.790	0.258	0.002	0.011	5002.621	-0.577	0.258	0.026	0.316	5002.621	0.213	0.298	0.476	0.689
RBM10	1822.880	-0.432	0.168	0.010	0.034	1822.880	-0.126	0.167	0.452	0.680	1822.880	0.306	0.194	0.115	0.304
KDM5C	2716.525	-0.395	0.146	0.007	0.025	2716.525	-0.007	0.145	0.959	0.979	2716.525	0.388	0.168	0.021	0.104
SMC1A	2001.073	-0.820	0.274	0.003	0.013	2001.073	-0.716	0.274	0.009	0.297	2001.073	0.104	0.317	0.743	0.868
CAPN3	11.041	2.154	0.754	0.004	0.018	11.041	1.321	0.767	0.085	0.377	11.041	-0.834	0.851	0.327	0.562
CAPNS1	4477.898	0.629	0.194	0.001	0.007	4477.898	0.225	0.194	0.247	0.522	4477.898	-0.404	0.224	0.071	0.228
NAA10	781.532	0.330	0.298	0.268	0.404	781.532	-0.279	0.299	0.351	0.605	781.532	-0.609	0.345	0.077	0.240
F8A1	1733.153	0.964	0.222	0.000	0.000	1733.153	0.311	0.222	0.162	0.453	1733.153	-0.653	0.256	0.011	0.069
UBL4A	1237.559	-0.330	0.169	0.050	0.116	1237.559	-0.050	0.168	0.764	0.875	1237.559	0.280	0.195	0.151	0.360
TMEM187	227.943	0.188	0.177	0.288	0.426	227.943	0.054	0.176	0.758	0.871	227.943	-0.133	0.204	0.514	0.718
LAGE3	430.013	0.587	0.214	0.006	0.023	430.013	0.235	0.215	0.273	0.544	430.013	-0.352	0.247	0.154	0.365
SLC10A3	658.924	0.478	0.143	0.001	0.005	658.924	0.103	0.143	0.473	0.695	658.924	-0.375	0.165	0.023	0.111
CAPS	23.446	2.016	0.556	0.000	0.002	23.446	1.212	0.565	0.032	0.322	23.446	-0.804	0.630	0.201	0.425
KDM5D	15.341	0.288	0.503	0.567	0.687	15.341	0.322	0.496	0.517	0.722	15.341	0.033	0.576	0.954	0.979
ARID1A	1429.217	-0.813	0.175	0.000	0.000	1429.217	-0.116	0.174	0.504	0.715	1429.217	0.696	0.202	0.001	0.009
CAPZA1	3275.222	-0.183	0.156	0.241	0.374	3275.222	-0.315	0.156	0.043	0.331	3275.222	-0.132	0.180	0.462	0.679
DYSF	78.989	2.355	0.387	0.000	0.000	78.989	0.852	0.400	0.033	0.322	78.989	-1.503	0.443	0.001	0.010
COLQ	43.330	0.018	0.237	0.939	0.962	43.330	-0.230	0.239	0.335	0.594	43.330	-0.248	0.280	0.376	0.607
SERF1A	272.861	0.316	0.272	0.244	0.377	272.861	-0.349	0.273	0.201	0.484	272.861	-0.666	0.315	0.035	0.145
H4C9	254.861	0.762	0.544	0.162	0.280	254.861	0.647	0.544	0.235	0.513	254.861	-0.115	0.628	0.854	0.930
TRRAP	948.993	-0.757	0.181	0.000	0.000	948.993	-0.236	0.179	0.188	0.474	948.993	0.521	0.208	0.012	0.075
CAPZA2	1566.024	-0.228	0.143	0.111	0.211	1566.024	-0.081	0.143	0.568	0.755	1566.024	0.147	0.165	0.374	0.606
PICALM	2586.800	-0.295	0.215	0.171	0.292	2586.800	-0.479	0.215	0.026	0.318	2586.800	-0.184	0.249	0.459	0.676
SNN	253.899	1.002	0.327	0.002	0.011	253.899	0.278	0.329	0.397	0.641	253.899	-0.723	0.378	0.056	0.196
ACOX2	9.979	-1.474	0.862	0.087	0.177	9.979	-0.398	0.766	0.604	0.781	9.979	1.076	0.982	0.273	0.508
CAST	3300.132	0.035	0.162	0.830	0.888	3300.132	0.135	0.162	0.404	0.646	3300.132	0.100	0.187	0.592	0.772
ACOX3	287.497	0.246	0.176	0.161	0.280	287.497	0.137	0.176	0.435	0.668	287.497	-0.109	0.203	0.591	0.771
AXIN1	1345.086	-0.175	0.180	0.329	0.470	1345.086	-0.093	0.180	0.603	0.781	1345.086	0.082	0.208	0.693	0.839
AXIN2	362.248	0.688	0.270	0.011	0.036	362.248	0.429	0.270	0.112	0.405	362.248	-0.258	0.312	0.407	0.634
BAP1	1746.246	-0.302	0.148	0.042	0.101	1746.246	-0.049	0.148	0.742	0.862	1746.246	0.253	0.171	0.139	0.343
BRAP	458.696	-0.562	0.187	0.003	0.012	458.696	-0.414	0.185	0.026	0.316	458.696	0.148	0.216	0.493	0.702
CDC7	335.125	-0.750	0.327	0.022	0.061	335.125	-0.686	0.326	0.036	0.324	335.125	0.065	0.378	0.864	0.935
CDC45	513.454	-1.533	0.548	0.005	0.020	513.454	-1.631	0.548	0.003	0.297	513.454	-0.098	0.634	0.877	0.941
CAPZB	4633.719	0.339	0.141	0.016	0.050	4633.719	-0.141	0.141	0.317	0.579	4633.719	-0.480	0.163	0.003	0.030
FZD1	221.215	0.780	0.210	0.000	0.002	221.215	0.328	0.211	0.120	0.412	221.215	-0.452	0.242	0.062	0.210
FZD4	344.378	0.970	0.313	0.002	0.010	344.378	0.684	0.313	0.029	0.322	344.378	-0.285	0.361	0.428	0.651
FZD6	751.904	-0.249	0.162	0.124	0.230	751.904	-0.200	0.161	0.214	0.493	751.904	0.048	0.187	0.797	0.898
FZD7	160.765	0.515	0.335	0.124	0.230	160.765	0.563	0.334	0.092	0.387	160.765	0.047	0.386	0.902	0.954
FZD8	414.883	0.316	0.399	0.428	0.565	414.883	0.375	0.398	0.347	0.602	414.883	0.059	0.460	0.898	0.952
H2AC13	18.502	-0.708	0.571	0.215	0.345	18.502	-0.014	0.545	0.979	0.988	18.502	0.693	0.654	0.289	0.524
CARS1	4984.174	-1.085	0.280	0.000	0.001	4984.174	-0.415	0.279	0.137	0.428	4984.174	0.670	0.323	0.038	0.154
H2AC17	12.893	0.439	0.516	0.394	0.533	12.893	0.202	0.517	0.696	0.837	12.893	-0.237	0.595	0.690	0.837
H2AC20	18.226	0.000	0.525	0.999	1.000	18.226	-0.562	0.536	0.294	0.562	18.226	-0.561	0.620	0.365	0.597
H2BC8	14.804	-0.010	0.651	0.988	0.993	14.804	0.657	0.621	0.290	0.558	14.804	0.666	0.735	0.365	0.597
PITPNM3	8.074	-0.395	0.793	0.618	0.729	8.074	0.036	0.765	0.962	0.980	8.074	0.431	0.910	0.636	0.802
CASP1	832.405	0.013	0.153	0.934	0.959	832.405	-0.195	0.153	0.201	0.484	832.405	-0.208	0.177	0.240	0.470
ELOVL3	10.239	0.402	0.559	0.473	0.607	10.239	0.558	0.549	0.309	0.574	10.239	0.157	0.636	0.805	0.903
H2BC15	52.363	0.160	0.551	0.771	0.848	52.363	0.722	0.543	0.184	0.471	52.363	0.562	0.631	0.373	0.605
TCF7L1	18.402	0.979	0.553	0.076	0.160	18.402	0.660	0.557	0.237	0.514	18.402	-0.319	0.628	0.611	0.785
ADPGK	791.214	0.201	0.162	0.216	0.346	791.214	-0.298	0.163	0.068	0.357	791.214	-0.499	0.188	0.008	0.057
SH3BGRL3	2667.633	1.363	0.274	0.000	0.000	2667.633	0.263	0.275	0.338	0.596	2667.633	-1.100	0.317	0.001	0.008
SF3B5	1361.259	0.133	0.200	0.506	0.635	1361.259	-0.137	0.201	0.494	0.709	1361.259	-0.271	0.232	0.243	0.474
INO80B	29.301	0.214	0.375	0.568	0.688	29.301	0.077	0.374	0.838	0.916	29.301	-0.138	0.435	0.751	0.872
PUS7L	283.393	-0.525	0.169	0.002	0.009	283.393	-0.094	0.165	0.569	0.756	283.393	0.431	0.194	0.027	0.122

PMFBP1	226.872	1.025	0.308	0.001	0.005	226.872	-0.049	0.311	0.875	0.937	226.872	-1.074	0.357	0.003	0.026
H2BC9	22.300	-0.578	0.391	0.139	0.250	22.300	-0.137	0.363	0.707	0.842	22.300	0.442	0.450	0.326	0.560
DRC3	14.187	0.391	0.536	0.466	0.600	14.187	0.550	0.526	0.296	0.563	14.187	0.159	0.610	0.794	0.896
ABHD11	143.493	-0.008	0.433	0.986	0.992	143.493	-0.837	0.436	0.055	0.342	143.493	-0.829	0.503	0.099	0.279
RAB33B	255.339	-0.580	0.226	0.010	0.035	255.339	0.117	0.222	0.599	0.777	255.339	0.697	0.260	0.007	0.053
EMC6	326.423	0.093	0.307	0.761	0.842	326.423	-0.287	0.308	0.351	0.605	326.423	-0.381	0.356	0.285	0.520
CDCA3	581.927	0.111	0.190	0.558	0.680	581.927	-0.026	0.190	0.890	0.945	581.927	-0.138	0.220	0.531	0.731
MXD3	1637.879	0.230	0.272	0.397	0.535	1637.879	-0.308	0.272	0.258	0.530	1637.879	-0.538	0.314	0.087	0.257
APH1B	41.564	0.200	0.573	0.726	0.814	41.564	0.229	0.571	0.688	0.831	41.564	0.029	0.660	0.965	0.984
GLT8D2	134.004	0.696	0.200	0.001	0.003	134.004	0.304	0.201	0.131	0.423	134.004	-0.392	0.231	0.090	0.262
H2BC4	21.991	0.335	0.560	0.549	0.672	21.991	0.552	0.552	0.318	0.579	21.991	0.216	0.640	0.735	0.864
KATNAL2	16.151	0.460	0.414	0.267	0.403	16.151	0.231	0.415	0.578	0.763	16.151	-0.229	0.477	0.632	0.800
DOHH	422.091	-0.103	0.330	0.755	0.837	422.091	-0.589	0.331	0.075	0.364	422.091	-0.486	0.382	0.203	0.427
ARHGAP24	230.255	-0.158	0.159	0.318	0.458	230.255	-0.141	0.157	0.370	0.619	230.255	0.017	0.184	0.925	0.965
DDX59	225.712	-0.785	0.181	0.000	0.000	225.712	-0.299	0.175	0.087	0.380	225.712	0.486	0.208	0.020	0.101
H2BC17	10.011	-0.266	0.756	0.725	0.813	10.011	0.664	0.717	0.355	0.608	10.011	0.929	0.854	0.276	0.511
PUS3	202.120	0.334	0.257	0.194	0.319	202.120	-0.055	0.258	0.831	0.913	202.120	-0.389	0.298	0.191	0.413
EPPK1	47.619	2.171	0.486	0.000	0.000	47.619	1.276	0.491	0.009	0.297	47.619	-0.895	0.555	0.107	0.291
H2BC21	89.432	0.243	0.526	0.644	0.750	89.432	1.076	0.520	0.038	0.327	89.432	0.833	0.602	0.167	0.381
CASP2	288.307	0.204	0.146	0.162	0.281	288.307	-0.008	0.146	0.955	0.978	288.307	-0.212	0.169	0.210	0.435
H3C4	17.770	0.724	0.365	0.047	0.110	17.770	0.019	0.389	0.960	0.979	17.770	-0.704	0.433	0.104	0.287
TTC25	31.074	0.013	0.301	0.964	0.978	31.074	0.590	0.283	0.037	0.325	31.074	0.577	0.337	0.087	0.258
NUF2	609.571	-0.060	0.218	0.784	0.857	609.571	0.001	0.217	0.995	0.998	609.571	0.061	0.252	0.809	0.904
FAM110A	364.209	0.272	0.198	0.169	0.289	364.209	0.484	0.196	0.014	0.306	364.209	0.212	0.227	0.351	0.584
AIF1L	13.496	0.596	0.450	0.186	0.310	13.496	0.881	0.435	0.043	0.330	13.496	0.285	0.500	0.568	0.758
DNAL1	171.489	0.046	0.135	0.733	0.820	171.489	-0.032	0.134	0.813	0.902	171.489	-0.078	0.156	0.620	0.792
RILP	193.958	1.499	0.394	0.000	0.001	193.958	0.810	0.396	0.041	0.328	193.958	-0.689	0.454	0.129	0.325
COG3	361.178	-0.514	0.182	0.005	0.019	361.178	0.041	0.179	0.818	0.905	361.178	0.555	0.209	0.008	0.057
UCK1	560.437	0.109	0.126	0.389	0.528	560.437	0.124	0.125	0.321	0.582	560.437	0.016	0.145	0.913	0.960
H3C8	13.793	-0.027	0.617	0.965	0.979	13.793	0.094	0.609	0.878	0.938	13.793	0.121	0.711	0.865	0.935
H3C10	69.817	0.317	0.370	0.391	0.530	69.817	0.138	0.370	0.710	0.844	69.817	-0.180	0.427	0.674	0.827
H3C2	7.675	0.319	0.780	0.682	0.780	7.675	0.582	0.765	0.447	0.677	7.675	0.263	0.888	0.767	0.882
TMUB1	1038.264	0.735	0.221	0.001	0.005	1038.264	0.243	0.222	0.272	0.544	1038.264	-0.491	0.256	0.055	0.194
THAP2	154.226	-0.377	0.303	0.214	0.343	154.226	0.449	0.298	0.132	0.424	154.226	0.826	0.348	0.018	0.094
AKR1E2	39.596	0.269	0.326	0.409	0.548	39.596	0.062	0.327	0.850	0.923	39.596	-0.208	0.378	0.583	0.765
RASSF5	129.055	0.493	0.453	0.276	0.414	129.055	0.571	0.452	0.206	0.488	129.055	0.078	0.521	0.881	0.943
NUDT12	152.502	-0.237	0.233	0.308	0.447	152.502	-0.246	0.232	0.289	0.557	152.502	-0.008	0.270	0.975	0.987
SOX7	381.751	-1.046	0.207	0.000	0.000	381.751	-0.325	0.203	0.110	0.403	381.751	0.721	0.238	0.003	0.026
BCL2L12	668.943	0.066	0.297	0.825	0.885	668.943	-0.440	0.297	0.138	0.429	668.943	-0.506	0.343	0.140	0.344
CASP3	499.612	-0.226	0.214	0.292	0.430	499.612	-0.386	0.214	0.072	0.361	499.612	-0.160	0.248	0.521	0.723
TMEM47	612.766	0.146	0.234	0.533	0.658	612.766	0.066	0.233	0.778	0.882	612.766	-0.080	0.270	0.768	0.882
CCM2	598.206	0.284	0.145	0.051	0.117	598.206	0.105	0.145	0.472	0.693	598.206	-0.179	0.168	0.286	0.521
GUCD1	988.107	0.522	0.159	0.001	0.006	988.107	0.277	0.159	0.081	0.372	988.107	-0.245	0.184	0.183	0.401
AMMECR1L	570.086	-0.950	0.202	0.000	0.000	570.086	-0.427	0.200	0.033	0.322	570.086	0.523	0.234	0.025	0.118
C18orf21	201.817	0.000	0.215	1.000	1.000	201.817	-0.017	0.214	0.935	0.967	201.817	-0.017	0.249	0.944	0.974
H4C12	78.107	0.467	0.294	0.112	0.213	78.107	0.084	0.296	0.777	0.882	78.107	-0.383	0.341	0.261	0.495
H4C11	67.673	0.678	0.293	0.020	0.059	67.673	0.108	0.298	0.718	0.849	67.673	-0.571	0.340	0.093	0.268
C19orf12	401.416	0.098	0.137	0.473	0.607	401.416	0.109	0.136	0.426	0.662	401.416	0.010	0.158	0.949	0.976
ZMIZ2	1107.900	0.420	0.121	0.001	0.003	1107.900	-0.041	0.122	0.733	0.857	1107.900	-0.461	0.140	0.001	0.013
C11orf68	1075.663	-0.110	0.167	0.512	0.641	1075.663	-0.025	0.167	0.881	0.940	1075.663	0.085	0.193	0.662	0.819
H4C3	13.262	-0.590	0.764	0.440	0.576	13.262	-1.038	0.776	0.181	0.469	13.262	-0.448	0.910	0.622	0.793
RAMAC	356.211	0.141	0.216	0.513	0.642	356.211	-0.311	0.218	0.153	0.445	356.211	-0.453	0.251	0.072	0.229
FAM107B	868.075	-1.710	0.173	0.000	0.000	868.075	-0.258	0.169	0.127	0.420	868.075	1.452	0.199	0.000	0.000
SELENOO	614.413	0.263	0.227	0.248	0.382	614.413	-0.080	0.228	0.726	0.854	614.413	-0.342	0.263	0.193	0.415
CCDC3	8.067	0.044	0.599	0.941	0.963	8.067	0.768	0.552	0.164	0.454	8.067	0.724	0.663	0.275	0.510
FAM167A	269.128	-2.187	0.302	0.000	0.000	269.128	-0.160	0.288	0.579	0.764	269.128	2.027	0.345	0.000	0.000
H4C8	49.524	-0.579	0.672	0.389	0.528	49.524	0.229	0.656	0.727	0.854	49.524	0.808	0.771	0.295	0.530
DYNLRB1	417.550	1.330	0.247	0.000	0.000	417.550	0.166	0.249	0.505	0.715	417.550	-1.163	0.286	0.000	0.001
TLN2	171.330	0.394	0.283	0.164	0.283	171.330	0.330	0.282	0.242	0.518	171.330	-0.063	0.326	0.846	0.925
PARP9	564.036	0.575	0.153	0.000	0.001	564.036	-0.022	0.154	0.888	0.944	564.036	-0.597	0.177	0.001	0.011
SESN2	1390.553	-2.324	0.367	0.000	0.000	1390.553	-1.020	0.366	0.005	0.297	1390.553	1.304	0.424	0.002	0.022
CRISPLD1	49.200	-0.016	0.371	0.965	0.979	49.200	0.145	0.367	0.693	0.834	49.200	0.161	0.428	0.706	0.847
CD99L2	822.355	0.355	0.207	0.086	0.175	822.355	0.080	0.207	0.699	0.838	822.355	-0.275	0.239	0.250	0.483
HSDL1	358.729	-0.249	0.183	0.174	0.295	358.729	-0.240	0.182	0.188	0.474	358.729	0.009	0.212	0.966	0.984
RHNO1	373.600	-0.074	0.175	0.672	0.772	373.600	-0.217	0.175	0.214	0.493	373.600	-0.143	0.202	0.480	0.693
TRAPPC9	263.476	0.617	0.189	0.001	0.006	263.476	0.354	0.190	0.063	0.350	263.476	-0.264	0.218	0.227	0.456
SH3BGRL2	148.067	-0.223	0.246	0.365	0.505	148.067	-0.342	0.246	0.165	0.455	148.067	-0.118	0.286	0.679	0.830

CASP4	895.338	0.562	0.134	0.000	0.000	895.338	0.123	0.134	0.359	0.612	895.338	-0.438	0.155	0.005	0.039
JAM3	710.865	0.339	0.128	0.008	0.029	710.865	-0.130	0.129	0.315	0.578	710.865	-0.468	0.149	0.002	0.019
FERMT3	15.852	0.830	0.551	0.132	0.240	15.852	0.233	0.565	0.680	0.827	15.852	-0.597	0.639	0.350	0.583
TRPT1	425.490	1.049	0.318	0.001	0.006	425.490	0.700	0.318	0.028	0.321	425.490	-0.348	0.367	0.342	0.576
NRIP2	11.631	0.196	0.502	0.696	0.790	11.631	0.353	0.490	0.472	0.694	11.631	0.156	0.573	0.785	0.893
ESPN	18.103	-1.179	0.916	0.198	0.324	18.103	0.922	0.873	0.291	0.559	18.103	2.101	1.042	0.044	0.168
CRISPLD2	281.252	-0.258	0.764	0.735	0.821	281.252	0.639	0.762	0.402	0.645	281.252	0.897	0.881	0.309	0.544
YPEL3	160.492	1.113	0.623	0.074	0.156	160.492	0.853	0.623	0.171	0.460	160.492	-0.261	0.718	0.717	0.853
HYAL3	167.776	1.052	0.191	0.000	0.000	167.776	0.256	0.196	0.192	0.477	167.776	-0.796	0.221	0.000	0.006
INHBE	404.558	-2.238	0.284	0.000	0.000	404.558	0.098	0.272	0.718	0.849	404.558	2.336	0.324	0.000	0.000
RIOK1	784.328	-0.990	0.301	0.001	0.006	784.328	-0.676	0.300	0.024	0.316	784.328	0.314	0.348	0.367	0.599
ATG10	26675.311	0.548	0.283	0.053	0.120	26675.311	0.491	0.283	0.082	0.374	26675.311	-0.056	0.326	0.863	0.935
ITCH	837.862	-0.929	0.221	0.000	0.000	837.862	-0.253	0.220	0.251	0.525	837.862	0.676	0.255	0.008	0.057
MARVELD1	718.934	0.566	0.287	0.048	0.112	718.934	0.670	0.286	0.019	0.306	718.934	0.104	0.331	0.754	0.874
GRWD1	978.954	-0.775	0.347	0.025	0.069	978.954	-0.753	0.347	0.030	0.322	978.954	0.022	0.401	0.957	0.980
ZNF484	87.113	-0.487	0.240	0.042	0.102	87.113	-0.120	0.233	0.607	0.783	87.113	0.367	0.276	0.184	0.404
L3MBTL2	332.045	-0.386	0.341	0.257	0.392	332.045	-0.841	0.341	0.014	0.306	332.045	-0.455	0.395	0.249	0.481
LONP2	1647.413	-0.172	0.097	0.077	0.161	1647.413	-0.102	0.097	0.295	0.563	1647.413	0.071	0.113	0.531	0.731
TAS1R3	24.909	1.293	0.455	0.004	0.018	24.909	0.727	0.463	0.117	0.409	24.909	-0.566	0.518	0.275	0.510
RBP5	39.675	0.048	0.420	0.910	0.943	39.675	0.556	0.410	0.176	0.464	39.675	0.508	0.479	0.289	0.524
RBM4B	312.467	0.308	0.149	0.038	0.094	312.467	-0.013	0.149	0.933	0.966	312.467	-0.321	0.173	0.063	0.211
FRMD8	601.134	0.593	0.144	0.000	0.000	601.134	-0.031	0.146	0.830	0.912	601.134	-0.624	0.168	0.000	0.004
ARMC10	1177.292	-0.017	0.121	0.891	0.929	1177.292	0.119	0.121	0.323	0.584	1177.292	0.136	0.140	0.331	0.566
MAD1L1	1154.740	0.606	0.197	0.002	0.010	1154.740	0.005	0.197	0.978	0.988	1154.740	-0.601	0.227	0.008	0.058
SYT15	17.625	1.340	0.603	0.026	0.071	17.625	0.930	0.607	0.126	0.418	17.625	-0.411	0.687	0.550	0.744
ESYT3	22.177	0.468	0.374	0.211	0.340	22.177	-0.247	0.387	0.524	0.727	22.177	-0.714	0.442	0.106	0.290
SETDB2	102.898	-0.266	0.241	0.269	0.406	102.898	0.171	0.235	0.468	0.691	102.898	0.437	0.277	0.114	0.303
ROPN1L	9.527	1.345	0.968	0.165	0.284	9.527	1.949	0.944	0.039	0.327	9.527	0.603	1.064	0.571	0.759
ANGPTL6	146.719	1.338	0.254	0.000	0.000	146.719	0.514	0.258	0.046	0.335	146.719	-0.824	0.293	0.005	0.040
KLF16	1277.439	0.025	0.244	0.919	0.949	1277.439	-0.148	0.244	0.545	0.739	1277.439	-0.172	0.281	0.540	0.737
FSD1L	82.691	-0.568	0.301	0.059	0.131	82.691	-0.227	0.295	0.441	0.673	82.691	0.342	0.348	0.327	0.561
TMTC1	3329.669	-0.167	0.207	0.420	0.557	3329.669	-0.020	0.207	0.923	0.961	3329.669	0.147	0.239	0.538	0.736
ATAD3B	718.650	-1.157	0.530	0.029	0.077	718.650	-1.540	0.531	0.004	0.297	718.650	-0.384	0.613	0.531	0.731
TAF3	300.455	-0.059	0.230	0.798	0.867	300.455	-0.452	0.231	0.051	0.338	300.455	-0.393	0.267	0.142	0.347
RSPH3	562.556	0.334	0.449	0.458	0.593	562.556	0.474	0.449	0.291	0.559	562.556	0.140	0.519	0.787	0.894
TMEM120A	317.066	1.072	0.323	0.001	0.005	317.066	0.388	0.324	0.232	0.510	317.066	-0.685	0.373	0.067	0.218
RAB34	2811.024	0.193	0.128	0.132	0.240	2811.024	0.026	0.128	0.838	0.916	2811.024	-0.167	0.148	0.259	0.494
HMCN1	39.268	-0.115	0.597	0.848	0.900	39.268	0.341	0.592	0.564	0.752	39.268	0.456	0.687	0.507	0.713
TBC1D10A	330.795	0.341	0.274	0.213	0.342	330.795	0.467	0.273	0.087	0.380	330.795	0.126	0.315	0.690	0.837
BCO2	96.808	0.966	0.223	0.000	0.000	96.808	0.192	0.229	0.401	0.644	96.808	-0.774	0.259	0.003	0.028
TM2D2	347.546	0.129	0.137	0.348	0.488	347.546	-0.217	0.138	0.117	0.409	347.546	-0.345	0.160	0.031	0.134
CDCA7	596.156	-0.946	0.351	0.007	0.026	596.156	-0.837	0.351	0.017	0.306	596.156	0.109	0.406	0.788	0.894
TSPAN10	19.771	2.244	0.544	0.000	0.000	19.771	0.774	0.573	0.177	0.465	19.771	-1.470	0.622	0.018	0.096
PRSS27	15.251	1.853	0.708	0.009	0.031	15.251	1.966	0.703	0.005	0.297	15.251	0.113	0.784	0.885	0.945
SPATA9	138.172	-1.047	0.422	0.013	0.041	138.172	0.153	0.415	0.713	0.846	138.172	1.199	0.485	0.013	0.079
SNX25	335.181	-0.006	0.164	0.972	0.982	335.181	0.068	0.163	0.676	0.825	335.181	0.074	0.190	0.696	0.841
KCTD10	655.976	0.194	0.134	0.148	0.262	655.976	-0.056	0.135	0.675	0.824	655.976	-0.251	0.156	0.107	0.292
CASP6	178.001	0.030	0.169	0.857	0.907	178.001	-0.172	0.169	0.308	0.573	178.001	-0.203	0.196	0.302	0.537
HASPIN	85.130	-0.841	0.312	0.007	0.026	85.130	-0.033	0.302	0.912	0.956	85.130	0.808	0.358	0.024	0.114
STARD3NL	238.478	0.084	0.185	0.652	0.756	238.478	-0.370	0.187	0.048	0.337	238.478	-0.454	0.216	0.036	0.148
STK40	1130.600	-0.997	0.315	0.002	0.008	1130.600	-0.728	0.314	0.021	0.312	1130.600	0.268	0.364	0.461	0.678
SPRTN	281.358	-0.503	0.258	0.051	0.117	281.358	-0.607	0.257	0.018	0.306	281.358	-0.104	0.299	0.727	0.859
HDAC10	270.587	1.027	0.294	0.000	0.003	270.587	0.681	0.294	0.021	0.312	270.587	-0.346	0.338	0.307	0.543
RASSF4	356.199	0.493	0.903	0.585	0.702	356.199	0.648	0.903	0.473	0.695	356.199	0.155	1.042	0.882	0.943
LRMDA	26.729	1.390	0.535	0.009	0.032	26.729	1.418	0.532	0.008	0.297	26.729	0.028	0.603	0.962	0.982
EIF2A	4149.281	-0.125	0.199	0.532	0.657	4149.281	0.253	0.199	0.204	0.487	4149.281	0.378	0.230	0.101	0.282
PIP5K1A	1701.571	-0.801	0.205	0.000	0.001	1701.571	-0.448	0.205	0.029	0.322	1701.571	0.353	0.237	0.137	0.339
TATDN1	1387.394	-0.165	0.152	0.278	0.415	1387.394	-0.055	0.152	0.719	0.849	1387.394	0.110	0.176	0.530	0.729
TM2D1	321.629	0.288	0.260	0.268	0.404	321.629	0.474	0.259	0.067	0.356	321.629	0.186	0.300	0.536	0.734
IMMP2L	212.843	0.018	0.358	0.961	0.977	212.843	0.404	0.356	0.257	0.530	212.843	0.386	0.412	0.349	0.583
SLC4A11	44.073	0.914	0.247	0.000	0.002	44.073	-0.155	0.265	0.559	0.749	44.073	-1.069	0.295	0.000	0.005
PIP4K2B	2521.090	-0.139	0.248	0.576	0.694	2521.090	0.231	0.248	0.350	0.605	2521.090	0.370	0.286	0.196	0.419
PLA2G6	427.723	0.003	0.202	0.990	0.994	427.723	-0.059	0.202	0.771	0.878	427.723	-0.061	0.234	0.793	0.896
IFI27L2	986.048	1.084	0.344	0.002	0.008	986.048	0.597	0.344	0.083	0.374	986.048	-0.488	0.397	0.220	0.446
TSSK6	30.347	-0.364	0.293	0.215	0.345	30.347	0.168	0.273	0.539	0.736	30.347	0.532	0.333	0.111	0.297
SPNS1	1053.090	0.482	0.159	0.002	0.011	1053.090	-0.067	0.160	0.676	0.825	1053.090	-0.549	0.184	0.003	0.028
FAM234A	1938.024	0.622	0.180	0.001	0.004	1938.024	0.251	0.181	0.165	0.455	1938.024	-0.371	0.208	0.075	0.235

FAM172A	320.192	-0.262	0.427	0.540	0.664	320.192	0.559	0.426	0.189	0.475	320.192	0.822	0.493	0.095	0.272
BRIP1	190.055	-0.843	0.303	0.005	0.021	190.055	-0.472	0.300	0.116	0.408	190.055	0.371	0.350	0.290	0.525
KREMEN1	852.769	-0.037	0.246	0.882	0.923	852.769	0.197	0.246	0.424	0.660	852.769	0.233	0.284	0.412	0.637
CASP7	470.513	0.081	0.126	0.521	0.649	470.513	-0.067	0.126	0.593	0.774	470.513	-0.148	0.146	0.310	0.546
TMPRSS13	11.681	0.304	0.461	0.509	0.639	11.681	0.455	0.446	0.308	0.573	11.681	0.151	0.521	0.772	0.884
B3GNT5	1229.106	-0.578	0.283	0.041	0.100	1229.106	-0.615	0.283	0.030	0.322	1229.106	-0.037	0.327	0.909	0.959
SLC25A11	565.851	-0.197	0.230	0.394	0.532	565.851	-0.277	0.230	0.229	0.507	565.851	-0.080	0.266	0.764	0.880
OBSCN	35.703	1.301	0.365	0.000	0.003	35.703	0.787	0.371	0.034	0.322	35.703	-0.514	0.414	0.214	0.440
SPOP	1470.023	-0.547	0.150	0.000	0.002	1470.023	-0.209	0.150	0.162	0.453	1470.023	0.338	0.174	0.051	0.186
KATNAL1	438.679	-0.700	0.241	0.004	0.015	438.679	-0.337	0.239	0.159	0.451	438.679	0.363	0.278	0.192	0.414
MND1	228.680	-0.162	0.250	0.517	0.645	228.680	0.130	0.248	0.600	0.778	228.680	0.292	0.288	0.311	0.546
WDR54	1008.432	0.307	0.368	0.403	0.541	1008.432	0.754	0.367	0.040	0.327	1008.432	0.446	0.424	0.293	0.528
ADGRV1	35.824	-1.309	0.475	0.006	0.022	35.824	-0.217	0.446	0.626	0.795	35.824	1.091	0.543	0.045	0.170
SRPX	1239.294	0.676	0.099	0.000	0.000	1239.294	0.344	0.100	0.001	0.253	1239.294	-0.332	0.114	0.004	0.034
RBM48	140.794	-0.265	0.149	0.075	0.158	140.794	0.018	0.143	0.898	0.948	140.794	0.283	0.171	0.098	0.277
MAGT1	1306.181	-0.125	0.153	0.415	0.553	1306.181	-0.243	0.153	0.112	0.405	1306.181	-0.118	0.177	0.503	0.710
DTNBP1	198.917	0.069	0.267	0.797	0.866	198.917	-0.198	0.267	0.459	0.685	198.917	-0.266	0.309	0.388	0.617
HDHD2	485.610	-0.435	0.139	0.002	0.009	485.610	0.131	0.137	0.338	0.596	485.610	0.566	0.160	0.000	0.007
TMEM222	509.654	0.229	0.197	0.243	0.377	509.654	-0.139	0.197	0.480	0.699	509.654	-0.369	0.228	0.106	0.289
FAM160A2	346.357	-0.002	0.172	0.993	0.996	346.357	0.129	0.171	0.449	0.678	346.357	0.131	0.199	0.510	0.715
SLC10A7	99.416	-0.212	0.197	0.281	0.419	99.416	-0.086	0.193	0.655	0.812	99.416	0.126	0.227	0.580	0.764
PLEKHN1	156.787	0.520	0.331	0.116	0.219	156.787	-0.555	0.336	0.098	0.393	156.787	-1.075	0.386	0.005	0.043
TAGLN2	15194.418	0.610	0.118	0.000	0.000	15194.418	0.110	0.118	0.349	0.604	15194.418	-0.500	0.136	0.000	0.005
ARMC2	30.752	-0.313	0.312	0.317	0.457	30.752	-0.035	0.300	0.908	0.954	30.752	0.278	0.360	0.440	0.660
QRICH2	25.255	2.677	0.479	0.000	0.000	25.255	0.422	0.516	0.414	0.652	25.255	-2.256	0.559	0.000	0.001
KBTBD7	170.636	-0.057	0.330	0.862	0.910	170.636	0.267	0.328	0.415	0.654	170.636	0.325	0.381	0.393	0.621
ANKRD27	1325.812	-0.694	0.256	0.007	0.025	1325.812	-0.551	0.256	0.031	0.322	1325.812	0.142	0.296	0.631	0.799
ULK1	1075.080	-0.058	0.206	0.779	0.853	1075.080	0.448	0.205	0.029	0.322	1075.080	0.505	0.237	0.033	0.140
ENKD1	255.734	0.394	0.220	0.074	0.156	255.734	0.256	0.220	0.244	0.519	255.734	-0.137	0.254	0.589	0.770
NSRP1	868.373	-0.218	0.243	0.369	0.509	868.373	-0.256	0.242	0.291	0.559	868.373	-0.038	0.280	0.892	0.949
ZRANB3	280.454	-0.477	0.219	0.029	0.077	280.454	0.302	0.215	0.160	0.451	280.454	0.779	0.251	0.002	0.021
FBXO30	1152.536	-1.101	0.300	0.000	0.002	1152.536	-0.832	0.299	0.005	0.297	1152.536	0.269	0.346	0.438	0.659
UXT	710.796	0.331	0.218	0.129	0.236	710.796	0.372	0.218	0.088	0.380	710.796	0.041	0.251	0.872	0.939
CASP8	865.442	-0.336	0.141	0.017	0.052	865.442	-0.170	0.140	0.226	0.505	865.442	0.166	0.163	0.310	0.546
ARL6	129.596	-0.124	0.176	0.483	0.616	129.596	-0.232	0.175	0.185	0.472	129.596	-0.109	0.206	0.596	0.775
SLC41A2	589.622	0.727	0.244	0.003	0.013	589.622	0.285	0.244	0.243	0.519	589.622	-0.442	0.281	0.116	0.305
PCBD2	68.231	-0.549	0.279	0.049	0.114	68.231	0.155	0.267	0.560	0.749	68.231	0.704	0.319	0.027	0.123
ZIC4	46.529	0.015	0.350	0.967	0.980	46.529	0.359	0.343	0.296	0.564	46.529	0.344	0.401	0.391	0.620
PCGF6	318.397	-0.651	0.261	0.013	0.040	318.397	-0.504	0.260	0.052	0.341	318.397	0.147	0.302	0.626	0.795
EEA1	955.474	-0.636	0.249	0.011	0.035	955.474	-0.473	0.248	0.057	0.345	955.474	0.163	0.287	0.571	0.759
BCAR3	743.669	-1.504	0.410	0.000	0.002	743.669	-0.846	0.409	0.038	0.327	743.669	0.658	0.473	0.164	0.379
ZNF394	268.300	0.178	0.193	0.357	0.497	268.300	-0.369	0.196	0.059	0.349	268.300	-0.547	0.226	0.015	0.086
LRRIQ1	27.905	0.674	0.418	0.107	0.206	27.905	0.001	0.427	0.998	0.999	27.905	-0.672	0.488	0.168	0.383
ATRIP	32.454	-0.699	0.334	0.037	0.091	32.454	-0.498	0.324	0.125	0.416	32.454	0.201	0.390	0.607	0.783
WDR75	867.225	-0.647	0.230	0.005	0.019	867.225	-0.492	0.229	0.032	0.322	867.225	0.155	0.266	0.559	0.750
ACAD11	111.840	-1.197	0.280	0.000	0.000	111.840	-0.111	0.268	0.678	0.826	111.840	1.085	0.321	0.001	0.011
CEP78	454.276	-0.262	0.268	0.329	0.469	454.276	-0.784	0.269	0.004	0.297	454.276	-0.522	0.311	0.093	0.268
USP42	333.814	-0.394	0.212	0.063	0.138	333.814	-0.335	0.211	0.113	0.405	333.814	0.059	0.245	0.809	0.904
ZNRF3	280.720	-0.687	0.203	0.001	0.005	280.720	-0.204	0.200	0.307	0.572	280.720	0.482	0.235	0.040	0.159
TOMM40L	197.645	-0.156	0.344	0.650	0.755	197.645	-0.636	0.345	0.065	0.354	197.645	-0.480	0.399	0.229	0.457
UTP15	566.741	-1.061	0.414	0.010	0.035	566.741	-1.212	0.414	0.003	0.297	566.741	-0.151	0.479	0.752	0.873
SLC7A6OS	664.773	-0.151	0.207	0.467	0.601	664.773	-0.359	0.207	0.083	0.374	664.773	-0.208	0.240	0.384	0.613
FAM161A	88.105	-0.232	0.194	0.233	0.365	88.105	-0.314	0.193	0.103	0.396	88.105	-0.082	0.227	0.718	0.853
EVA1A	8.552	0.245	0.513	0.634	0.742	8.552	-0.735	0.558	0.188	0.474	8.552	-0.979	0.635	0.123	0.315
ABRAXAS1	795.998	-0.294	0.180	0.103	0.200	795.998	0.066	0.179	0.714	0.847	795.998	0.359	0.208	0.084	0.251
SYDE2	99.510	-0.477	0.374	0.202	0.329	99.510	-0.091	0.370	0.806	0.898	99.510	0.386	0.431	0.370	0.602
ZNF644	661.096	-0.629	0.160	0.000	0.001	661.096	-0.254	0.159	0.110	0.403	661.096	0.375	0.185	0.043	0.166
KAT8	559.773	0.449	0.190	0.018	0.054	559.773	0.256	0.190	0.179	0.467	559.773	-0.193	0.220	0.380	0.610
RNASEH2C	749.592	0.211	0.127	0.098	0.193	749.592	-0.028	0.128	0.827	0.910	749.592	-0.239	0.148	0.105	0.289
RPF2	1197.719	-0.314	0.217	0.149	0.262	1197.719	-0.437	0.217	0.044	0.332	1197.719	-0.123	0.251	0.624	0.794
ARID5B	390.973	0.486	0.271	0.073	0.154	390.973	0.261	0.271	0.336	0.594	390.973	-0.225	0.313	0.472	0.686
ANXA9	47.342	0.644	0.466	0.166	0.286	47.342	0.810	0.463	0.080	0.371	47.342	0.166	0.533	0.756	0.875
KIAA1109	675.174	-0.452	0.412	0.273	0.410	675.174	0.251	0.411	0.541	0.738	675.174	0.703	0.475	0.139	0.343
GTF2IRD2	109.475	0.338	0.447	0.449	0.585	109.475	0.586	0.445	0.188	0.474	109.475	0.248	0.514	0.630	0.798
ASCC2	1040.696	-0.062	0.194	0.749	0.832	1040.696	-0.272	0.194	0.160	0.451	1040.696	-0.210	0.224	0.348	0.582
NLRC5	230.126	0.537	0.177	0.002	0.011	230.126	-0.024	0.179	0.894	0.947	230.126	-0.560	0.205	0.006	0.048
C19orf44	575.554	0.133	0.169	0.434	0.570	575.554	-0.076	0.169	0.653	0.811	575.554	-0.209	0.196	0.287	0.522

ANTXR1	2080.542	0.357	0.198	0.071	0.152	2080.542	0.073	0.198	0.714	0.847	2080.542	-0.284	0.229	0.214	0.439
STX7	899.390	0.043	0.172	0.801	0.869	899.390	0.073	0.171	0.670	0.822	899.390	0.030	0.198	0.881	0.943
POLR1B	1363.163	-1.167	0.239	0.000	0.000	1363.163	-0.466	0.238	0.050	0.337	1363.163	0.701	0.276	0.011	0.070
ELMOD3	374.502	0.029	0.166	0.860	0.908	374.502	0.195	0.164	0.235	0.513	374.502	0.166	0.191	0.385	0.614
SLA2	171.553	0.346	0.460	0.452	0.587	171.553	-1.308	0.467	0.005	0.297	171.553	-1.654	0.537	0.002	0.022
SLC49A3	10.511	0.651	0.700	0.352	0.492	10.511	0.067	0.715	0.925	0.962	10.511	-0.584	0.815	0.473	0.687
CHD6	558.472	-0.063	0.276	0.820	0.882	558.472	0.139	0.275	0.613	0.786	558.472	0.202	0.318	0.526	0.726
MINDY4	20.814	0.183	0.461	0.692	0.788	20.814	-0.965	0.489	0.048	0.337	20.814	-1.148	0.559	0.040	0.159
ZCCHC7	1117.400	-0.415	0.146	0.005	0.019	1117.400	0.119	0.145	0.412	0.651	1117.400	0.534	0.169	0.002	0.018
TMEM164	509.535	0.068	0.239	0.777	0.852	509.535	0.311	0.239	0.193	0.478	509.535	0.243	0.276	0.379	0.609
FAR1	1198.430	-0.595	0.256	0.020	0.058	1198.430	-0.583	0.256	0.023	0.315	1198.430	0.012	0.296	0.967	0.985
METTL25	49.026	-0.243	0.231	0.292	0.430	49.026	0.208	0.214	0.332	0.592	49.026	0.451	0.261	0.085	0.253
CIAO2A	678.316	0.263	0.199	0.187	0.312	678.316	-0.165	0.200	0.408	0.649	678.316	-0.428	0.230	0.063	0.212
SETD3	1517.284	-0.500	0.218	0.022	0.062	1517.284	-0.482	0.218	0.027	0.319	1517.284	0.018	0.252	0.944	0.974
USP48	905.223	-0.383	0.132	0.004	0.016	905.223	-0.155	0.131	0.236	0.513	905.223	0.227	0.152	0.136	0.338
POMK	30.381	-0.446	0.483	0.356	0.496	30.381	-0.452	0.480	0.346	0.602	30.381	-0.006	0.562	0.991	0.995
CASP9	143.952	0.239	0.164	0.144	0.257	143.952	0.222	0.162	0.173	0.462	143.952	-0.018	0.189	0.925	0.965
SNHG3	966.890	-0.177	0.186	0.341	0.481	966.890	0.369	0.185	0.046	0.335	966.890	0.546	0.214	0.011	0.069
MEX3B	89.988	0.245	0.363	0.500	0.631	89.988	0.755	0.357	0.034	0.322	89.988	0.511	0.414	0.218	0.444
ANKRD20A1	10.577	0.234	0.517	0.651	0.756	10.577	-0.768	0.555	0.166	0.456	10.577	-1.001	0.632	0.113	0.301
TMEM117	47.031	-0.210	0.349	0.547	0.670	47.031	0.037	0.343	0.914	0.957	47.031	0.247	0.402	0.538	0.736
TBC1D3F	47.151	2.549	0.527	0.000	0.000	47.151	1.830	0.532	0.001	0.253	47.151	-0.719	0.592	0.225	0.452
WDR24	381.805	-0.135	0.146	0.355	0.495	381.805	-0.038	0.145	0.794	0.892	381.805	0.097	0.169	0.564	0.754
RGPD5	821.110	-0.391	0.255	0.124	0.230	821.110	-0.421	0.254	0.098	0.392	821.110	-0.030	0.294	0.918	0.962
SPATC1L	234.600	1.115	0.234	0.000	0.000	234.600	0.815	0.234	0.001	0.253	234.600	-0.300	0.268	0.263	0.497
TMEM191A	12.209	0.918	0.540	0.089	0.180	12.209	0.556	0.548	0.310	0.575	12.209	-0.362	0.614	0.556	0.749
IQCG	168.519	0.586	0.248	0.018	0.054	168.519	0.159	0.250	0.526	0.728	168.519	-0.428	0.287	0.137	0.339
NBPF3	94.492	-0.825	0.282	0.003	0.015	94.492	0.080	0.270	0.767	0.877	94.492	0.905	0.322	0.005	0.041
C2orf16	6.373	-1.427	0.868	0.100	0.196	6.373	0.474	0.731	0.517	0.722	6.373	1.902	0.960	0.048	0.176
LRRC8C	10.885	0.526	0.657	0.424	0.560	10.885	0.050	0.667	0.940	0.970	10.885	-0.476	0.764	0.533	0.732
TRAF7	2743.645	0.300	0.105	0.004	0.017	2743.645	-0.045	0.105	0.665	0.818	2743.645	-0.345	0.121	0.004	0.037
MAF1	2094.779	-0.050	0.155	0.745	0.829	2094.779	-0.109	0.155	0.481	0.700	2094.779	-0.059	0.179	0.743	0.868
TMEM126A	280.269	0.049	0.184	0.788	0.860	280.269	-0.099	0.184	0.590	0.771	280.269	-0.148	0.213	0.486	0.697
RHBDD1	382.328	-1.006	0.164	0.000	0.000	382.328	-0.150	0.159	0.345	0.602	382.328	0.856	0.188	0.000	0.000
ATP13A4	7.918	-1.788	0.994	0.072	0.153	7.918	-0.152	0.908	0.868	0.932	7.918	1.636	1.128	0.147	0.354
ZCCHC9	441.696	0.181	0.228	0.428	0.564	441.696	-0.132	0.229	0.563	0.752	441.696	-0.313	0.264	0.236	0.465
ZDHHC18	357.622	0.614	0.171	0.000	0.002	357.622	-0.071	0.173	0.684	0.829	357.622	-0.684	0.199	0.001	0.009
MRI1	405.637	-0.278	0.150	0.064	0.139	405.637	0.164	0.147	0.265	0.537	405.637	0.441	0.172	0.010	0.067
MED10	1228.018	-0.229	0.185	0.216	0.345	1228.018	-0.219	0.184	0.236	0.513	1228.018	0.010	0.213	0.963	0.982
RTL6	908.729	-0.683	0.245	0.005	0.021	908.729	-0.041	0.244	0.866	0.931	908.729	0.641	0.283	0.024	0.113
FYTTD1	2259.050	-0.781	0.227	0.001	0.004	2259.050	-0.376	0.227	0.097	0.392	2259.050	0.405	0.263	0.123	0.315
LTBP4	2542.805	0.493	0.489	0.313	0.453	2542.805	0.738	0.489	0.131	0.423	2542.805	0.245	0.564	0.664	0.821
SLF1	223.387	-0.221	0.286	0.441	0.577	223.387	-0.434	0.287	0.130	0.422	223.387	-0.213	0.332	0.520	0.723
SGIP1	24.690	0.788	0.493	0.110	0.210	24.690	0.864	0.488	0.077	0.368	24.690	0.075	0.559	0.893	0.949
GARNL3	43.309	0.107	0.448	0.811	0.876	43.309	0.275	0.444	0.536	0.734	43.309	0.168	0.515	0.744	0.869
CAMKK1	260.001	0.125	0.223	0.574	0.693	260.001	0.410	0.221	0.063	0.350	260.001	0.285	0.256	0.266	0.501
SLC37A3	390.200	-0.100	0.184	0.587	0.703	390.200	-0.196	0.184	0.287	0.556	390.200	-0.096	0.213	0.654	0.814
FLYWCH1	2323.343	-0.269	0.105	0.010	0.035	2323.343	0.155	0.104	0.138	0.429	2323.343	0.423	0.121	0.000	0.008
DCUN1D5	985.004	-1.040	0.345	0.003	0.012	985.004	-0.760	0.345	0.027	0.321	985.004	0.281	0.399	0.481	0.694
TCHP	520.388	-0.713	0.289	0.014	0.043	520.388	-0.849	0.289	0.003	0.297	520.388	-0.137	0.334	0.683	0.833
FBXW9	210.573	-0.050	0.155	0.746	0.830	210.573	0.049	0.152	0.749	0.866	210.573	0.099	0.179	0.580	0.764
PSMG3	615.128	-0.195	0.418	0.641	0.748	615.128	-0.944	0.419	0.024	0.316	615.128	-0.749	0.484	0.121	0.313
HSDL2	1281.884	-0.337	0.123	0.006	0.023	1281.884	-0.130	0.122	0.289	0.557	1281.884	0.207	0.142	0.144	0.349
HAGHL	563.993	0.557	0.202	0.006	0.022	563.993	-0.105	0.203	0.604	0.781	563.993	-0.662	0.234	0.005	0.039
POLR3GL	470.139	0.688	0.254	0.007	0.025	470.139	0.368	0.255	0.149	0.442	470.139	-0.320	0.294	0.276	0.511
ALKBH7	669.624	0.784	0.328	0.017	0.050	669.624	0.584	0.328	0.075	0.364	669.624	-0.200	0.378	0.597	0.775
RPAIN	306.495	0.335	0.204	0.100	0.196	306.495	-0.059	0.205	0.773	0.879	306.495	-0.394	0.236	0.095	0.272
CHCHD5	308.304	0.167	0.208	0.420	0.557	308.304	0.228	0.207	0.270	0.541	308.304	0.061	0.240	0.800	0.900
ZNF282	1259.591	-0.384	0.126	0.002	0.011	1259.591	0.113	0.125	0.364	0.616	1259.591	0.498	0.145	0.001	0.009
CARD19	850.610	0.720	0.164	0.000	0.000	850.610	0.144	0.164	0.382	0.629	850.610	-0.577	0.189	0.002	0.024
POLDIP3	1612.405	-0.627	0.205	0.002	0.011	1612.405	0.027	0.204	0.895	0.948	1612.405	0.654	0.237	0.006	0.045
YIPF4	815.653	-0.104	0.095	0.274	0.411	815.653	0.010	0.094	0.915	0.958	815.653	0.114	0.109	0.300	0.534
NOA1	1003.560	-0.164	0.240	0.494	0.626	1003.560	0.357	0.239	0.135	0.426	1003.560	0.521	0.276	0.059	0.204
COQ5	743.941	-0.205	0.183	0.263	0.399	743.941	0.272	0.182	0.134	0.426	743.941	0.477	0.211	0.024	0.113
SLC25A33	285.727	-0.579	0.372	0.120	0.224	285.727	-0.923	0.373	0.013	0.305	285.727	-0.344	0.432	0.426	0.649
NICN1	15.554	0.124	0.545	0.820	0.882	15.554	0.077	0.542	0.887	0.943	15.554	-0.047	0.630	0.941	0.973
DNAJC30	137.679	-0.043	0.234	0.853	0.904	137.679	-0.032	0.233	0.889	0.945	137.679	0.011	0.271	0.968	0.985

MFSD14C	312.095	-0.115	0.167	0.491	0.623	312.095	-0.230	0.167	0.168	0.457	312.095	-0.115	0.194	0.554	0.747
PRADC1	145.462	0.182	0.338	0.591	0.706	145.462	-0.922	0.344	0.007	0.297	145.462	-1.103	0.396	0.005	0.043
STK24	2425.555	-0.691	0.103	0.000	0.000	2425.555	0.074	0.102	0.468	0.691	2425.555	0.765	0.118	0.000	0.000
BTBD10	623.065	-0.442	0.191	0.021	0.059	623.065	-0.425	0.191	0.026	0.316	623.065	0.017	0.221	0.940	0.973
C2orf88	9.195	-0.968	0.514	0.060	0.132	9.195	-0.991	0.503	0.049	0.337	9.195	-0.023	0.619	0.970	0.985
RNF135	298.422	-0.012	0.202	0.954	0.972	298.422	0.362	0.200	0.069	0.358	298.422	0.374	0.232	0.107	0.291
TMEM79	190.096	0.296	0.196	0.130	0.238	190.096	-0.157	0.198	0.427	0.662	190.096	-0.454	0.228	0.047	0.175
NTPCR	761.377	0.619	0.222	0.005	0.020	761.377	0.256	0.222	0.249	0.523	761.377	-0.363	0.256	0.156	0.367
EIF1AD	290.599	0.118	0.234	0.614	0.725	290.599	-0.573	0.236	0.015	0.306	290.599	-0.690	0.273	0.011	0.071
TMEM175	124.279	0.948	0.274	0.001	0.004	124.279	-0.197	0.281	0.482	0.701	124.279	-1.146	0.320	0.000	0.006
ZDHHC16	570.766	-0.029	0.180	0.874	0.919	570.766	-0.269	0.181	0.136	0.427	570.766	-0.241	0.209	0.249	0.481
EFCAB2	80.397	0.664	0.262	0.011	0.037	80.397	0.477	0.262	0.069	0.357	80.397	-0.187	0.300	0.534	0.732
ING5	551.832	0.326	0.199	0.101	0.198	551.832	0.219	0.199	0.271	0.542	551.832	-0.107	0.230	0.641	0.805
WDR83	740.880	0.575	0.204	0.005	0.020	740.880	0.176	0.205	0.391	0.636	740.880	-0.399	0.236	0.091	0.265
PRXL2A	137.501	0.895	0.681	0.189	0.313	137.501	0.265	0.682	0.698	0.837	137.501	-0.630	0.786	0.423	0.647
UTP23	864.332	-0.740	0.168	0.000	0.000	864.332	-0.090	0.166	0.589	0.770	864.332	0.651	0.193	0.001	0.011
PHF6	841.698	-0.643	0.209	0.002	0.010	841.698	-0.193	0.208	0.353	0.607	841.698	0.450	0.241	0.062	0.209
GINS4	271.170	-0.244	0.479	0.611	0.723	271.170	-1.335	0.481	0.005	0.297	271.170	-1.092	0.555	0.049	0.181
LLPH	610.864	-0.382	0.219	0.081	0.167	610.864	-0.637	0.219	0.004	0.297	610.864	-0.256	0.254	0.314	0.549
MIEN1	643.196	0.757	0.232	0.001	0.006	643.196	-0.059	0.233	0.799	0.895	643.196	-0.816	0.269	0.002	0.025
CASP10	66.045	0.475	0.192	0.013	0.042	66.045	0.033	0.196	0.867	0.932	66.045	-0.442	0.225	0.049	0.181
UQCC2	431.307	0.798	0.299	0.008	0.028	431.307	-0.087	0.301	0.773	0.879	431.307	-0.884	0.346	0.011	0.069
DDI2	565.492	-0.804	0.303	0.008	0.029	565.492	-0.519	0.302	0.086	0.379	565.492	0.285	0.350	0.416	0.641
CHCHD6	221.157	0.419	0.212	0.048	0.112	221.157	0.304	0.211	0.150	0.442	221.157	-0.114	0.244	0.639	0.804
NUDT22	706.127	0.312	0.144	0.031	0.080	706.127	0.298	0.144	0.039	0.327	706.127	-0.014	0.166	0.932	0.969
PYM1	449.960	0.668	0.206	0.001	0.007	449.960	-0.109	0.208	0.600	0.778	449.960	-0.777	0.239	0.001	0.015
PDCD2L	221.055	-0.283	0.420	0.500	0.631	221.055	-0.766	0.421	0.069	0.357	221.055	-0.483	0.487	0.322	0.556
ZNF397	193.860	0.039	0.209	0.850	0.902	193.860	0.265	0.207	0.199	0.482	193.860	0.226	0.240	0.347	0.581
NUDT16L1	274.727	0.383	0.152	0.012	0.039	274.727	0.005	0.154	0.974	0.986	274.727	-0.378	0.177	0.033	0.140
C7orf50	1233.168	-0.082	0.343	0.811	0.876	1233.168	-0.549	0.343	0.110	0.403	1233.168	-0.466	0.396	0.239	0.469
MRPL45	1819.405	-0.269	0.178	0.131	0.239	1819.405	0.252	0.177	0.154	0.447	1819.405	0.521	0.205	0.011	0.070
BRMS1L	175.689	-0.510	0.253	0.044	0.105	175.689	-0.352	0.251	0.160	0.451	175.689	0.158	0.293	0.591	0.771
VPS25	489.966	-0.167	0.315	0.596	0.710	489.966	-0.713	0.316	0.024	0.316	489.966	-0.546	0.365	0.134	0.334
TMEM107	147.961	0.360	0.406	0.375	0.516	147.961	0.631	0.404	0.119	0.411	147.961	0.271	0.467	0.562	0.753
MON1A	278.469	-0.327	0.326	0.317	0.457	278.469	-0.501	0.326	0.124	0.416	278.469	-0.175	0.378	0.644	0.807
NAA38	316.468	0.215	0.224	0.339	0.479	316.468	0.012	0.225	0.958	0.978	316.468	-0.203	0.260	0.435	0.656
CCDC115	211.146	0.656	0.303	0.030	0.079	211.146	0.399	0.303	0.188	0.474	211.146	-0.257	0.350	0.462	0.679
CCDC77	327.528	-0.166	0.156	0.287	0.425	327.528	-0.101	0.155	0.514	0.720	327.528	0.065	0.180	0.719	0.854
CMSS1	799.488	-0.120	0.306	0.695	0.789	799.488	-0.610	0.306	0.046	0.335	799.488	-0.490	0.354	0.166	0.381
THOC3	1161.590	-0.070	0.208	0.738	0.823	1161.590	-0.444	0.209	0.033	0.322	1161.590	-0.375	0.241	0.120	0.311
SARNP	35.919	0.616	0.405	0.128	0.235	35.919	-0.173	0.416	0.677	0.825	35.919	-0.789	0.475	0.097	0.274
METTL26	2086.138	0.661	0.288	0.021	0.061	2086.138	0.582	0.288	0.043	0.331	2086.138	-0.079	0.332	0.812	0.906
ZBED3	554.017	-0.649	0.218	0.003	0.013	554.017	0.026	0.217	0.905	0.952	554.017	0.675	0.252	0.007	0.053
LZIC	558.257	-0.162	0.104	0.119	0.222	558.257	-0.052	0.102	0.611	0.786	558.257	0.110	0.120	0.360	0.593
HVCN1	13.438	2.284	0.518	0.000	0.000	13.438	0.842	0.553	0.128	0.420	13.438	-1.442	0.588	0.014	0.082
ZNF414	238.811	0.438	0.280	0.118	0.222	238.811	0.323	0.280	0.249	0.523	238.811	-0.115	0.323	0.722	0.856
MCRIP2	1381.610	0.564	0.194	0.004	0.015	1381.610	0.307	0.194	0.113	0.405	1381.610	-0.257	0.224	0.250	0.483
PCGF5	1337.416	-0.600	0.182	0.001	0.006	1337.416	-0.059	0.181	0.743	0.863	1337.416	0.541	0.210	0.010	0.066
COA8	324.955	0.322	0.225	0.153	0.268	324.955	0.113	0.225	0.615	0.788	324.955	-0.209	0.260	0.422	0.646
AKT1S1	1742.017	-0.167	0.144	0.245	0.379	1742.017	-0.041	0.144	0.775	0.880	1742.017	0.126	0.166	0.448	0.668
TMEM101	923.549	0.033	0.133	0.805	0.872	923.549	0.032	0.132	0.809	0.900	923.549	-0.001	0.153	0.996	0.998
ELOF1	917.450	0.507	0.232	0.029	0.077	917.450	-0.043	0.233	0.853	0.925	917.450	-0.550	0.269	0.041	0.161
RECK	78.552	0.451	0.359	0.209	0.338	78.552	0.252	0.359	0.484	0.701	78.552	-0.199	0.415	0.631	0.799
GFM2	709.211	-0.519	0.176	0.003	0.014	709.211	-0.255	0.175	0.146	0.438	709.211	0.264	0.203	0.194	0.416
COG8	815.296	-0.035	0.119	0.771	0.848	815.296	-0.025	0.119	0.836	0.916	815.296	0.010	0.138	0.941	0.973
HPS3	1587.084	0.481	0.256	0.061	0.134	1587.084	0.325	0.256	0.205	0.488	1587.084	-0.156	0.296	0.598	0.777
CAVIN2	1518.664	-0.084	0.305	0.784	0.857	1518.664	0.209	0.305	0.492	0.708	1518.664	0.293	0.352	0.406	0.632
ARFGAP2	945.458	-0.430	0.178	0.016	0.048	945.458	-0.222	0.178	0.212	0.492	945.458	0.209	0.206	0.311	0.547
NIFK	1441.858	-0.570	0.282	0.043	0.104	1441.858	-0.626	0.282	0.026	0.318	1441.858	-0.056	0.326	0.864	0.935
HOOK3	812.716	0.053	0.153	0.726	0.814	812.716	-0.053	0.153	0.729	0.855	812.716	-0.106	0.176	0.547	0.742
RAD54L	324.403	-0.530	0.321	0.099	0.194	324.403	-0.656	0.321	0.041	0.329	324.403	-0.126	0.372	0.735	0.863
NSMAF	1169.309	-0.573	0.198	0.004	0.016	1169.309	-0.265	0.197	0.179	0.467	1169.309	0.308	0.229	0.179	0.397
NCK2	1398.581	-0.366	0.137	0.008	0.027	1398.581	0.011	0.136	0.938	0.968	1398.581	0.377	0.158	0.017	0.093
CYSTM1	710.848	0.687	0.122	0.000	0.000	710.848	0.027	0.124	0.827	0.910	710.848	-0.660	0.142	0.000	0.000
GNPAT	951.136	-0.251	0.094	0.007	0.027	951.136	0.066	0.092	0.478	0.698	951.136	0.317	0.108	0.003	0.031
ZNF528	28.634	-0.187	0.420	0.655	0.759	28.634	0.396	0.405	0.329	0.589	28.634	0.583	0.479	0.223	0.451
MSANTD4	436.255	-0.989	0.277	0.000	0.003	436.255	-0.554	0.275	0.044	0.332	436.255	0.435	0.320	0.175	0.392

DYRK3	634.326	-0.002	0.122	0.984	0.991	634.326	0.011	0.121	0.927	0.963	634.326	0.014	0.140	0.923	0.964
RAB11FIP4	28.289	0.896	0.321	0.005	0.020	28.289	0.147	0.334	0.659	0.814	28.289	-0.749	0.375	0.046	0.172
MAML2	200.778	-0.364	0.285	0.201	0.328	200.778	0.059	0.282	0.835	0.915	200.778	0.423	0.329	0.198	0.421
DOT1L	1301.228	-0.512	0.244	0.035	0.089	1301.228	-0.410	0.243	0.092	0.387	1301.228	0.102	0.281	0.716	0.853
LZTS2	2217.959	0.767	0.247	0.002	0.010	2217.959	0.433	0.247	0.080	0.371	2217.959	-0.334	0.285	0.241	0.472
BRSK1	201.635	-0.110	0.217	0.611	0.723	201.635	0.194	0.214	0.365	0.617	201.635	0.304	0.249	0.223	0.451
SYVN1	730.337	-0.008	0.147	0.959	0.975	730.337	-0.013	0.147	0.931	0.965	730.337	-0.005	0.170	0.977	0.988
ABLIM2	342.955	3.802	0.306	0.000	0.000	342.955	1.017	0.313	0.001	0.297	342.955	-2.786	0.351	0.000	0.000
ZNF333	136.010	-0.176	0.156	0.259	0.395	136.010	-0.212	0.154	0.170	0.459	136.010	-0.036	0.182	0.845	0.925
DYRK2	425.845	-0.378	0.180	0.035	0.089	425.845	-0.274	0.178	0.125	0.417	425.845	0.104	0.208	0.616	0.789
ZNF512	480.504	-0.619	0.185	0.001	0.005	480.504	-0.223	0.183	0.224	0.503	480.504	0.397	0.213	0.063	0.211
MAP3K21	408.899	-0.625	0.191	0.001	0.006	408.899	-0.063	0.189	0.737	0.860	408.899	0.561	0.221	0.011	0.070
EFCAB7	122.520	0.032	0.319	0.921	0.950	122.520	0.338	0.316	0.285	0.554	122.520	0.306	0.367	0.404	0.631
L3MBTL3	152.924	-0.183	0.227	0.420	0.557	152.924	-0.015	0.225	0.948	0.974	152.924	0.168	0.262	0.521	0.723
LCOR	501.501	-0.583	0.252	0.021	0.059	501.501	-0.268	0.251	0.285	0.554	501.501	0.315	0.291	0.280	0.515
DUSP11	521.905	-0.309	0.174	0.076	0.160	521.905	-0.360	0.174	0.038	0.327	521.905	-0.051	0.202	0.799	0.899
ZMAT1	15.412	1.150	0.734	0.117	0.220	15.412	1.126	0.731	0.124	0.416	15.412	-0.024	0.836	0.977	0.988
NEURL4	212.692	-0.350	0.239	0.143	0.255	212.692	-0.454	0.239	0.057	0.347	212.692	-0.104	0.278	0.709	0.849
SLX4	195.298	-0.328	0.236	0.165	0.284	195.298	-0.169	0.234	0.470	0.692	195.298	0.159	0.273	0.561	0.752
DOC2B	19.296	1.348	0.535	0.012	0.039	19.296	2.258	0.520	0.000	0.040	19.296	0.910	0.587	0.121	0.313
DOC2A	227.613	1.022	0.313	0.001	0.006	227.613	0.129	0.316	0.683	0.829	227.613	-0.893	0.363	0.014	0.081
DHX16	4634.336	-0.825	0.290	0.004	0.018	4634.336	-0.527	0.289	0.069	0.357	4634.336	0.298	0.334	0.373	0.605
FAM120B	1342.894	-0.203	0.208	0.327	0.468	1342.894	0.124	0.207	0.548	0.742	1342.894	0.328	0.240	0.172	0.388
CUL4B	2104.243	0.016	0.369	0.965	0.979	2104.243	0.472	0.369	0.200	0.483	2104.243	0.456	0.426	0.284	0.519
SPIRE2	58.168	0.912	0.261	0.000	0.003	58.168	0.493	0.265	0.062	0.350	58.168	-0.419	0.299	0.161	0.375
ZNF527	47.403	-0.366	0.286	0.201	0.328	47.403	-0.185	0.278	0.507	0.716	47.403	0.181	0.331	0.585	0.767
NKX6-2	27.541	0.649	0.385	0.092	0.184	27.541	0.168	0.391	0.667	0.819	27.541	-0.481	0.446	0.282	0.516
CUL4A	1593.652	-0.528	0.177	0.003	0.013	1593.652	-0.436	0.177	0.014	0.306	1593.652	0.093	0.205	0.651	0.812
PLPP5	155.315	0.446	0.187	0.017	0.051	155.315	0.129	0.189	0.495	0.710	155.315	-0.317	0.217	0.144	0.349
GHDC	428.255	0.729	0.347	0.036	0.090	428.255	0.532	0.347	0.126	0.418	428.255	-0.197	0.401	0.623	0.793
MCM8	104.811	-0.444	0.384	0.248	0.382	104.811	-0.977	0.387	0.012	0.305	104.811	-0.533	0.448	0.234	0.463
DCTN5	1273.861	-0.299	0.182	0.101	0.197	1273.861	-0.386	0.182	0.034	0.322	1273.861	-0.086	0.211	0.682	0.832
CUL3	1210.911	-0.395	0.137	0.004	0.016	1210.911	-0.262	0.136	0.054	0.342	1210.911	0.133	0.158	0.400	0.627
GON7	402.226	-0.134	0.280	0.634	0.742	402.226	-0.375	0.281	0.181	0.469	402.226	-0.242	0.324	0.456	0.675
JAGN1	969.969	0.167	0.177	0.344	0.485	969.969	0.274	0.176	0.121	0.413	969.969	0.106	0.204	0.602	0.780
ZC3H8	411.119	-0.775	0.129	0.000	0.000	411.119	-0.163	0.124	0.190	0.476	411.119	0.613	0.148	0.000	0.001
ZNF559	86.394	0.266	0.277	0.337	0.477	86.394	0.429	0.274	0.118	0.409	86.394	0.163	0.318	0.609	0.784
C15orf41	109.326	0.065	0.180	0.718	0.808	109.326	-0.055	0.179	0.760	0.872	109.326	-0.120	0.209	0.567	0.757
CUL2	640.991	-0.766	0.269	0.004	0.018	640.991	-0.656	0.268	0.014	0.306	640.991	0.109	0.311	0.725	0.858
ACSS1	71.906	0.280	0.213	0.188	0.313	71.906	0.154	0.212	0.466	0.690	71.906	-0.125	0.245	0.610	0.785
LINC01547	31.799	-0.360	0.340	0.289	0.427	31.799	-0.446	0.338	0.186	0.473	31.799	-0.086	0.399	0.830	0.917
CUL1	1517.127	-0.328	0.146	0.025	0.067	1517.127	-0.261	0.146	0.073	0.362	1517.127	0.067	0.169	0.692	0.839
KBTBD8	51.048	-1.122	0.486	0.021	0.060	51.048	-1.100	0.483	0.023	0.315	51.048	0.022	0.569	0.970	0.985
KIAA1841	101.045	0.012	0.317	0.971	0.982	101.045	-0.615	0.321	0.055	0.343	101.045	-0.626	0.370	0.091	0.264
MRPL43	818.871	0.320	0.176	0.069	0.147	818.871	0.124	0.176	0.480	0.699	818.871	-0.196	0.203	0.335	0.569
PGBD1	105.272	-0.181	0.343	0.598	0.712	105.272	-0.756	0.346	0.029	0.322	105.272	-0.575	0.401	0.151	0.361
TMEM185A	295.957	0.102	0.199	0.608	0.721	295.957	0.257	0.197	0.192	0.477	295.957	0.156	0.229	0.497	0.705
MAK16	662.068	-1.447	0.408	0.000	0.003	662.068	-1.040	0.408	0.011	0.300	662.068	0.407	0.472	0.389	0.617
ATRN	1284.405	-0.171	0.250	0.494	0.626	1284.405	0.156	0.250	0.533	0.732	1284.405	0.327	0.289	0.257	0.492
PARD6G	126.185	0.041	0.217	0.850	0.902	126.185	0.043	0.215	0.842	0.918	126.185	0.002	0.250	0.994	0.996
FAXC	43.974	1.094	0.257	0.000	0.000	43.974	-0.248	0.278	0.374	0.622	43.974	-1.341	0.309	0.000	0.000
MAP1LC3A	81.966	1.991	0.382	0.000	0.000	81.966	0.039	0.396	0.921	0.960	81.966	-1.952	0.446	0.000	0.000
SLC12A8	33.938	1.707	0.498	0.001	0.004	33.938	0.375	0.518	0.469	0.691	33.938	-1.332	0.578	0.021	0.105
GNPTG	957.051	1.278	0.288	0.000	0.000	957.051	0.484	0.289	0.093	0.388	957.051	-0.793	0.333	0.017	0.092
TTF2	548.177	-0.226	0.234	0.335	0.475	548.177	-0.520	0.234	0.026	0.318	548.177	-0.295	0.271	0.277	0.511
TPST2	523.535	0.495	0.168	0.003	0.014	523.535	0.035	0.169	0.837	0.916	523.535	-0.460	0.195	0.018	0.095
TPST1	126.777	0.426	0.244	0.081	0.167	126.777	0.164	0.245	0.502	0.714	126.777	-0.261	0.282	0.354	0.588
PARD6B	83.829	0.060	0.312	0.848	0.900	83.829	0.625	0.305	0.040	0.328	83.829	0.565	0.356	0.113	0.300
ZBTB37	95.497	-0.070	0.321	0.828	0.887	95.497	-0.296	0.321	0.357	0.611	95.497	-0.226	0.373	0.544	0.740
TUBB6	4639.448	0.484	0.226	0.032	0.083	4639.448	-0.429	0.226	0.058	0.347	4639.448	-0.913	0.261	0.000	0.008
ZGPAT	82.058	0.051	0.321	0.873	0.918	82.058	-0.666	0.327	0.042	0.330	82.058	-0.717	0.377	0.057	0.200
KLF11	237.637	0.384	0.321	0.232	0.364	237.637	0.387	0.320	0.226	0.505	237.637	0.004	0.370	0.992	0.996
ZNF594	41.794	-0.221	0.329	0.502	0.631	41.794	-0.271	0.327	0.408	0.649	41.794	-0.050	0.383	0.897	0.951
KRBA1	137.296	0.618	0.225	0.006	0.023	137.296	0.070	0.228	0.757	0.871	137.296	-0.548	0.261	0.036	0.148
TNRC18	5456.278	-0.832	0.107	0.000	0.000	5456.278	-0.081	0.106	0.444	0.675	5456.278	0.751	0.123	0.000	0.000
TEAD2	322.517	0.581	0.195	0.003	0.013	322.517	-0.391	0.199	0.049	0.337	322.517	-0.972	0.228	0.000	0.001
KISS1R	34.818	1.609	0.907	0.076	0.159	34.818	1.447	0.907	0.111	0.404	34.818	-0.163	1.038	0.876	0.940

SUPT3H	119.682	-0.442	0.191	0.021	0.059	119.682	0.482	0.178	0.007	0.297	119.682	0.923	0.216	0.000	0.001
USP38	451.081	-0.554	0.213	0.009	0.032	451.081	-0.365	0.212	0.084	0.376	451.081	0.189	0.246	0.443	0.663
MFSD14B	1091.038	0.119	0.247	0.629	0.739	1091.038	-0.617	0.248	0.013	0.305	1091.038	-0.736	0.286	0.010	0.066
C22orf23	257.200	0.670	0.228	0.003	0.015	257.200	0.356	0.229	0.120	0.412	257.200	-0.313	0.263	0.234	0.463
DGAT2	307.668	0.487	0.195	0.012	0.040	307.668	0.352	0.195	0.071	0.359	307.668	-0.135	0.225	0.549	0.744
EBPL	1687.283	-0.011	0.194	0.956	0.973	1687.283	0.325	0.194	0.093	0.388	1687.283	0.336	0.224	0.134	0.333
GLYR1	1230.734	-0.292	0.154	0.058	0.129	1230.734	-0.394	0.154	0.011	0.299	1230.734	-0.102	0.179	0.568	0.758
DPY30	624.032	0.646	0.168	0.000	0.001	624.032	0.219	0.169	0.194	0.478	624.032	-0.427	0.194	0.028	0.126
GLIS2	3168.811	0.529	0.236	0.025	0.068	3168.811	0.475	0.236	0.044	0.331	3168.811	-0.054	0.272	0.844	0.925
MYPN	249.264	-0.363	0.252	0.149	0.262	249.264	-0.565	0.252	0.025	0.316	249.264	-0.202	0.292	0.489	0.700
HES7	340.968	0.132	0.359	0.714	0.805	340.968	-0.089	0.359	0.804	0.897	340.968	-0.221	0.414	0.594	0.774
FAM126A	1258.032	-0.520	0.282	0.066	0.142	1258.032	-0.552	0.282	0.051	0.338	1258.032	-0.032	0.326	0.922	0.964
USP32	1628.700	-0.233	0.177	0.188	0.312	1628.700	-0.043	0.177	0.809	0.900	1628.700	0.191	0.205	0.351	0.585
SMARCA5	1819.946	-0.346	0.171	0.043	0.103	1819.946	-0.353	0.171	0.039	0.327	1819.946	-0.007	0.198	0.972	0.986
CARD6	417.128	0.260	0.153	0.088	0.178	417.128	-0.030	0.153	0.843	0.918	417.128	-0.291	0.177	0.101	0.282
KDM2B	758.535	-0.334	0.184	0.069	0.147	758.535	-0.198	0.183	0.279	0.549	758.535	0.136	0.212	0.520	0.723
SLC9A7	340.812	0.758	0.129	0.000	0.000	340.812	-0.093	0.133	0.484	0.702	340.812	-0.851	0.151	0.000	0.000
ACCS	60.138	0.892	0.501	0.075	0.157	60.138	0.486	0.502	0.333	0.592	60.138	-0.405	0.577	0.483	0.695
HINT2	103.460	0.700	0.343	0.041	0.100	103.460	0.267	0.345	0.440	0.672	103.460	-0.434	0.397	0.274	0.509
PPP1R9B	2644.926	-0.146	0.087	0.092	0.183	2644.926	0.123	0.086	0.153	0.445	2644.926	0.269	0.100	0.007	0.052
CCDC54	17.018	-2.022	0.472	0.000	0.000	17.018	-0.340	0.371	0.360	0.612	17.018	1.683	0.525	0.001	0.016
MCEE	127.622	0.225	0.290	0.439	0.575	127.622	0.022	0.291	0.939	0.969	127.622	-0.203	0.336	0.547	0.742
LOXL3	268.154	0.870	0.207	0.000	0.000	268.154	0.292	0.209	0.163	0.454	268.154	-0.578	0.240	0.016	0.088
ABHD1	17.827	2.089	0.554	0.000	0.001	17.827	0.828	0.579	0.152	0.445	17.827	-1.261	0.631	0.046	0.173
CAPS2	68.163	-0.005	0.557	0.993	0.996	68.163	0.345	0.554	0.533	0.733	68.163	0.350	0.642	0.586	0.767
CAT	1615.495	-0.043	0.285	0.881	0.923	1615.495	0.348	0.284	0.221	0.500	1615.495	0.391	0.329	0.234	0.463
SORBS2	1077.014	-0.290	0.356	0.415	0.553	1077.014	0.389	0.355	0.273	0.544	1077.014	0.679	0.411	0.098	0.277
GTPBP3	420.185	-0.264	0.168	0.115	0.217	420.185	0.080	0.166	0.628	0.796	420.185	0.345	0.193	0.074	0.234
GPT2	1546.756	-1.345	0.299	0.000	0.000	1546.756	-0.440	0.298	0.140	0.431	1546.756	0.904	0.345	0.009	0.060
LNX1	280.830	-0.035	0.171	0.838	0.893	280.830	-0.284	0.171	0.098	0.392	280.830	-0.249	0.199	0.211	0.436
HDGFL2	1492.814	0.695	0.199	0.000	0.003	1492.814	0.166	0.200	0.407	0.648	1492.814	-0.529	0.230	0.022	0.106
PIGO	477.540	-0.679	0.192	0.000	0.003	477.540	0.116	0.189	0.539	0.736	477.540	0.795	0.220	0.000	0.006
PSRC1	717.861	-0.163	0.248	0.511	0.640	717.861	0.464	0.247	0.060	0.350	717.861	0.627	0.286	0.028	0.127
PLEKHA8	384.187	-0.210	0.252	0.405	0.543	384.187	-0.241	0.252	0.338	0.596	384.187	-0.031	0.292	0.914	0.960
PRRC2B	4929.265	-0.557	0.194	0.004	0.017	4929.265	0.115	0.194	0.553	0.745	4929.265	0.672	0.224	0.003	0.027
SPSB2	131.347	0.049	0.307	0.874	0.919	131.347	-0.320	0.308	0.299	0.566	131.347	-0.368	0.356	0.301	0.537
OGT	756.188	-0.006	0.264	0.981	0.988	756.188	0.204	0.264	0.439	0.671	756.188	0.211	0.305	0.490	0.700
CBX2	386.045	0.201	0.194	0.301	0.440	386.045	-0.016	0.194	0.934	0.967	386.045	-0.216	0.224	0.335	0.569
FAM167B	20.341	4.469	0.659	0.000	0.000	20.341	0.775	0.866	0.371	0.620	20.341	-3.695	0.753	0.000	0.000
AFAP1-AS1	176.615	-0.302	0.261	0.247	0.381	176.615	0.030	0.258	0.908	0.954	176.615	0.332	0.301	0.270	0.504
UNC119B	1381.394	-0.805	0.351	0.022	0.061	1381.394	0.280	0.350	0.423	0.659	1381.394	1.086	0.405	0.007	0.053
USP30	200.308	-0.029	0.310	0.925	0.953	200.308	0.343	0.308	0.266	0.538	200.308	0.372	0.357	0.298	0.533
FUT10	100.385	-0.384	0.276	0.165	0.284	100.385	-0.314	0.274	0.252	0.526	100.385	0.070	0.320	0.828	0.915
B3GNT9	244.208	1.113	0.208	0.000	0.000	244.208	-0.022	0.213	0.919	0.960	244.208	-1.134	0.243	0.000	0.000
PCGF1	403.410	0.210	0.210	0.317	0.457	403.410	-0.032	0.211	0.880	0.939	403.410	-0.242	0.243	0.320	0.555
CDC42BPA	1998.000	-0.915	0.193	0.000	0.000	1998.000	-0.169	0.192	0.381	0.628	1998.000	0.746	0.223	0.001	0.011
ZNF577	78.278	0.594	0.275	0.031	0.080	78.278	0.167	0.278	0.547	0.741	78.278	-0.427	0.318	0.180	0.398
CRACR2A	164.067	-1.172	0.355	0.001	0.006	164.067	-0.708	0.351	0.044	0.331	164.067	0.463	0.410	0.259	0.493
MPV17L2	228.819	0.172	0.458	0.707	0.799	228.819	-1.291	0.462	0.005	0.297	228.819	-1.463	0.533	0.006	0.047
ZNF607	19.595	0.199	0.462	0.666	0.767	19.595	0.363	0.453	0.423	0.659	19.595	0.164	0.528	0.756	0.876
DLGAP1-AS2	25.532	0.056	0.311	0.856	0.906	25.532	0.370	0.294	0.209	0.490	25.532	0.314	0.351	0.372	0.604
KMT5C	198.454	0.467	0.258	0.071	0.151	198.454	0.529	0.257	0.040	0.327	198.454	0.062	0.297	0.834	0.919
HIRIP3	690.533	-0.170	0.181	0.348	0.488	690.533	0.014	0.181	0.939	0.969	690.533	0.184	0.209	0.379	0.610
TUBA1C	9229.715	0.023	0.181	0.901	0.936	9229.715	-0.208	0.181	0.251	0.525	9229.715	-0.230	0.209	0.271	0.506
LINC00467	107.950	0.263	0.299	0.379	0.519	107.950	0.274	0.298	0.358	0.611	107.950	0.011	0.345	0.975	0.987
FAM220A	917.849	-0.324	0.186	0.081	0.166	917.849	0.061	0.185	0.740	0.861	917.849	0.385	0.214	0.072	0.229
FOXD2-AS1	216.541	0.577	0.243	0.018	0.052	216.541	0.542	0.243	0.026	0.316	216.541	-0.035	0.280	0.901	0.953
PYROXD2	221.074	0.968	0.476	0.042	0.101	221.074	0.627	0.477	0.188	0.474	221.074	-0.341	0.550	0.535	0.733
C19orf48	1960.030	-0.955	0.248	0.000	0.001	1960.030	-0.319	0.247	0.197	0.481	1960.030	0.636	0.286	0.026	0.120
RAE1	526.891	-0.602	0.260	0.021	0.059	526.891	-0.656	0.260	0.012	0.305	526.891	-0.054	0.301	0.858	0.932
GPAT3	215.933	-0.569	0.287	0.047	0.111	215.933	-0.250	0.285	0.380	0.627	215.933	0.319	0.332	0.336	0.570
MFSD9	207.526	0.011	0.197	0.956	0.973	207.526	-0.056	0.196	0.775	0.880	207.526	-0.067	0.228	0.769	0.882
NFKBID	41.374	0.680	0.393	0.084	0.171	41.374	-0.201	0.407	0.621	0.791	41.374	-0.881	0.463	0.057	0.199
PERM1	40.547	1.379	0.439	0.002	0.009	40.547	-0.675	0.475	0.155	0.447	40.547	-2.055	0.531	0.000	0.003
CROCCP2	414.630	0.286	0.182	0.115	0.218	414.630	0.175	0.182	0.334	0.593	414.630	-0.111	0.210	0.597	0.776
OFD1	385.470	0.470	0.208	0.024	0.066	385.470	-0.045	0.209	0.831	0.913	385.470	-0.514	0.241	0.033	0.139
BUD13	194.856	-0.591	0.224	0.008	0.030	194.856	-0.234	0.221	0.290	0.558	194.856	0.357	0.259	0.168	0.382

PLCD4	52.369	-0.054	0.240	0.823	0.885	52.369	-0.407	0.244	0.096	0.391	52.369	-0.353	0.286	0.216	0.442
MGC12916	35.193	-1.395	0.460	0.002	0.011	35.193	-0.950	0.441	0.031	0.322	35.193	0.445	0.537	0.407	0.633
RTN4IP1	140.079	-0.477	0.368	0.195	0.321	140.079	-0.652	0.368	0.076	0.367	140.079	-0.175	0.427	0.682	0.832
TXNDC17	376.316	0.449	0.180	0.013	0.041	376.316	0.045	0.181	0.803	0.897	376.316	-0.404	0.208	0.053	0.189
IL17RC	228.928	1.192	0.394	0.003	0.012	228.928	0.672	0.395	0.089	0.382	228.928	-0.520	0.455	0.253	0.485
POLR2J4	168.296	0.105	0.197	0.594	0.708	168.296	-0.183	0.198	0.355	0.609	168.296	-0.288	0.229	0.209	0.434
LMNB2	5462.854	-0.509	0.213	0.017	0.050	5462.854	-0.462	0.213	0.030	0.322	5462.854	0.048	0.246	0.847	0.926
FCRLA	21.720	1.343	0.688	0.051	0.117	21.720	0.827	0.692	0.232	0.510	21.720	-0.516	0.789	0.513	0.718
SFT2D3	122.843	-0.271	0.181	0.136	0.246	122.843	-0.043	0.177	0.806	0.898	122.843	0.227	0.209	0.278	0.512
ANKRD36BP1	18.415	-0.603	0.494	0.223	0.354	18.415	-0.150	0.476	0.753	0.868	18.415	0.452	0.570	0.427	0.650
ATP5MD	1704.497	0.741	0.246	0.003	0.012	1704.497	0.000	0.247	0.999	0.999	1704.497	-0.741	0.285	0.009	0.062
ABHD14B	1162.177	0.135	0.222	0.542	0.667	1162.177	0.438	0.222	0.048	0.337	1162.177	0.303	0.256	0.237	0.466
ARHGAP5-AS1	48.146	-0.200	0.369	0.588	0.704	48.146	0.071	0.362	0.844	0.919	48.146	0.271	0.425	0.524	0.725
ZNF496	1266.831	-0.493	0.209	0.018	0.053	1266.831	0.289	0.208	0.163	0.454	1266.831	0.783	0.240	0.001	0.015
PHF5A	529.348	-0.229	0.327	0.484	0.617	529.348	-0.592	0.327	0.070	0.359	529.348	-0.364	0.378	0.336	0.570
TRIM52	154.282	0.593	0.408	0.147	0.260	154.282	0.362	0.409	0.376	0.624	154.282	-0.231	0.471	0.624	0.794
ATP1A1-AS1	5146.184	0.100	0.094	0.286	0.424	5146.184	0.119	0.094	0.204	0.487	5146.184	0.019	0.109	0.861	0.933
LINC00839	434.837	0.075	0.131	0.565	0.686	434.837	0.066	0.130	0.612	0.786	434.837	-0.009	0.151	0.951	0.978
ZNF503	401.881	-0.191	0.307	0.534	0.659	401.881	-0.414	0.308	0.179	0.467	401.881	-0.222	0.356	0.532	0.732
LRCH3	651.807	-0.308	0.097	0.001	0.008	651.807	-0.155	0.095	0.105	0.398	651.807	0.153	0.112	0.171	0.387
KLHL22	197.820	0.765	0.329	0.020	0.057	197.820	0.560	0.329	0.089	0.382	197.820	-0.205	0.379	0.588	0.769
RIOX2	815.964	-0.726	0.246	0.003	0.014	815.964	-0.550	0.245	0.025	0.316	815.964	0.177	0.284	0.534	0.733
CCDC142	105.579	0.001	0.203	0.995	0.997	105.579	0.185	0.198	0.349	0.604	105.579	0.184	0.233	0.428	0.651
TMEM25	124.826	0.626	0.414	0.131	0.239	124.826	0.603	0.414	0.145	0.436	124.826	-0.022	0.477	0.962	0.982
CBR4	397.483	-0.581	0.181	0.001	0.007	397.483	0.130	0.178	0.466	0.690	397.483	0.711	0.208	0.001	0.010
GEMIN2	186.081	-0.956	0.328	0.004	0.015	186.081	-0.594	0.325	0.068	0.356	186.081	0.362	0.379	0.340	0.575
ZC3H10	195.224	-0.409	0.167	0.015	0.045	195.224	-0.164	0.164	0.316	0.578	195.224	0.245	0.193	0.206	0.430
ZNF514	47.416	0.751	0.331	0.023	0.064	47.416	0.076	0.337	0.823	0.908	47.416	-0.676	0.385	0.079	0.242
PARP10	1856.048	0.095	0.338	0.778	0.853	1856.048	0.563	0.338	0.096	0.391	1856.048	0.468	0.390	0.231	0.459
ORAI1	433.646	-0.116	0.347	0.738	0.823	433.646	-0.509	0.347	0.142	0.434	433.646	-0.393	0.401	0.327	0.562
ZBTB45	287.643	0.007	0.186	0.970	0.981	287.643	0.055	0.185	0.766	0.877	287.643	0.048	0.215	0.824	0.913
MFSD2A	196.769	-0.652	0.420	0.121	0.225	196.769	-0.809	0.420	0.054	0.342	196.769	-0.157	0.487	0.747	0.870
RPUSD4	764.292	-0.455	0.161	0.005	0.019	764.292	0.337	0.159	0.034	0.322	764.292	0.792	0.185	0.000	0.001
AIFM2	1678.004	0.152	0.121	0.210	0.339	1678.004	-0.006	0.121	0.962	0.980	1678.004	-0.158	0.140	0.261	0.495
ZDHHC12	421.664	1.034	0.265	0.000	0.001	421.664	0.213	0.266	0.423	0.660	421.664	-0.821	0.306	0.007	0.053
C1orf198	809.293	1.738	0.134	0.000	0.000	809.293	0.168	0.137	0.221	0.501	809.293	-1.571	0.156	0.000	0.000
SPPL2A	552.530	0.030	0.154	0.847	0.900	552.530	-0.142	0.154	0.355	0.609	552.530	-0.172	0.178	0.335	0.569
ADO	620.150	-0.482	0.194	0.013	0.042	620.150	-0.165	0.193	0.392	0.637	620.150	0.317	0.225	0.158	0.370
POMGNT2	206.939	0.384	0.236	0.104	0.201	206.939	-0.206	0.239	0.388	0.634	206.939	-0.590	0.275	0.032	0.137
FBH1	1287.296	-0.232	0.169	0.170	0.290	1287.296	-0.335	0.169	0.047	0.336	1287.296	-0.103	0.196	0.598	0.776
MIGA2	206.637	0.422	0.280	0.131	0.240	206.637	-0.435	0.283	0.124	0.416	206.637	-0.857	0.326	0.009	0.059
ATAD1	1181.256	-0.470	0.154	0.002	0.011	1181.256	-0.337	0.154	0.029	0.322	1181.256	0.134	0.178	0.454	0.672
TBRG1	533.049	-0.280	0.209	0.180	0.303	533.049	-0.217	0.208	0.297	0.565	533.049	0.063	0.241	0.794	0.896
PLXDC2	273.602	1.082	0.412	0.009	0.030	273.602	0.676	0.412	0.101	0.394	273.602	-0.407	0.475	0.392	0.620
TMTC4	216.611	0.008	0.150	0.957	0.974	216.611	0.164	0.147	0.264	0.536	216.611	0.156	0.172	0.364	0.596
RGS5	57.549	-0.347	0.373	0.352	0.492	57.549	0.267	0.363	0.461	0.687	57.549	0.614	0.427	0.150	0.359
RNFT2	32.929	1.215	0.403	0.003	0.012	32.929	0.174	0.415	0.676	0.825	32.929	-1.042	0.469	0.026	0.121
NFATC2IP	564.311	0.213	0.181	0.239	0.372	564.311	-0.357	0.182	0.050	0.337	564.311	-0.571	0.210	0.007	0.050
CEP89	431.458	0.027	0.130	0.834	0.890	431.458	0.071	0.129	0.583	0.766	431.458	0.044	0.150	0.772	0.884
ARHGEF39	2084.396	0.518	0.529	0.328	0.468	2084.396	0.912	0.529	0.085	0.376	2084.396	0.395	0.611	0.518	0.721
ZNF341	59.956	0.407	0.215	0.058	0.129	59.956	-0.091	0.221	0.680	0.827	59.956	-0.498	0.253	0.049	0.180
FAM136A	877.967	-0.411	0.241	0.088	0.178	877.967	-0.464	0.241	0.054	0.342	877.967	-0.052	0.278	0.851	0.928
AOPEP	451.182	-0.512	0.179	0.004	0.017	451.182	-0.041	0.177	0.817	0.904	451.182	0.471	0.206	0.022	0.109
MAP4K3	207.368	-0.306	0.183	0.094	0.187	207.368	-0.128	0.180	0.476	0.697	207.368	0.177	0.211	0.401	0.628
TMEM87B	413.304	0.122	0.169	0.470	0.604	413.304	-0.098	0.169	0.562	0.751	413.304	-0.220	0.196	0.261	0.495
SLC35B4	551.004	-0.585	0.218	0.007	0.026	551.004	-0.389	0.217	0.073	0.362	551.004	0.196	0.252	0.437	0.658
ATOH8	3648.971	-0.260	0.216	0.229	0.361	3648.971	0.172	0.216	0.426	0.662	3648.971	0.432	0.249	0.083	0.250
ZNF587	165.097	-0.426	0.233	0.067	0.144	165.097	-0.044	0.229	0.848	0.922	165.097	0.382	0.268	0.154	0.364
FAM222A	10.527	0.371	0.766	0.628	0.738	10.527	-1.473	0.872	0.091	0.386	10.527	-1.845	0.978	0.059	0.203
UTP4	2222.197	-1.065	0.285	0.000	0.002	2222.197	-0.574	0.285	0.044	0.331	2222.197	0.491	0.329	0.136	0.337
LRP11	1666.968	0.511	0.257	0.047	0.110	1666.968	0.463	0.257	0.071	0.360	1666.968	-0.047	0.297	0.874	0.939
PPP1R15B	1111.105	-0.426	0.159	0.008	0.027	1111.105	-0.249	0.159	0.117	0.409	1111.105	0.177	0.184	0.337	0.572
PRSS12	193.767	0.108	0.250	0.667	0.768	193.767	0.216	0.249	0.386	0.632	193.767	0.108	0.289	0.708	0.848
ALG10	69.988	-0.760	0.302	0.012	0.039	69.988	-0.218	0.292	0.456	0.683	69.988	0.542	0.348	0.119	0.310
FIZ1	301.043	0.042	0.154	0.786	0.858	301.043	-0.105	0.154	0.495	0.709	301.043	-0.147	0.178	0.411	0.637
FAM104A	820.694	-0.030	0.104	0.773	0.849	820.694	-0.160	0.104	0.122	0.413	820.694	-0.130	0.120	0.279	0.513
ZNF566	121.317	-0.386	0.237	0.103	0.200	121.317	0.005	0.231	0.982	0.989	121.317	0.391	0.272	0.151	0.360

SLC49A4	274.162	0.369	0.214	0.085	0.173	274.162	-0.249	0.216	0.248	0.522	274.162	-0.618	0.249	0.013	0.077
SPRYD3	813.586	-0.081	0.225	0.720	0.809	813.586	-0.306	0.225	0.175	0.464	813.586	-0.225	0.261	0.389	0.617
TMEM209	360.990	-0.494	0.258	0.055	0.125	360.990	-0.513	0.258	0.046	0.335	360.990	-0.019	0.299	0.949	0.976
PPM1D	238.309	-0.760	0.240	0.002	0.008	238.309	-0.331	0.236	0.161	0.452	238.309	0.429	0.277	0.122	0.313
MASTL	516.480	-0.883	0.253	0.000	0.003	516.480	-0.433	0.251	0.085	0.376	516.480	0.450	0.292	0.123	0.316
RAB2B	201.544	0.105	0.180	0.559	0.681	201.544	0.141	0.178	0.430	0.664	201.544	0.036	0.207	0.863	0.935
C8orf76	139.595	-0.495	0.323	0.125	0.230	139.595	-0.377	0.321	0.240	0.516	139.595	0.118	0.374	0.751	0.873
RITA1	622.812	-0.191	0.181	0.290	0.428	622.812	-0.286	0.180	0.113	0.405	622.812	-0.095	0.209	0.651	0.812
ZFYVE19	402.464	0.271	0.258	0.293	0.431	402.464	0.389	0.257	0.131	0.423	402.464	0.117	0.297	0.693	0.839
ZNRF1	858.772	0.911	0.172	0.000	0.000	858.772	0.166	0.173	0.337	0.595	858.772	-0.744	0.199	0.000	0.004
ATG4C	260.388	-0.353	0.150	0.018	0.054	260.388	-0.215	0.148	0.146	0.438	260.388	0.139	0.174	0.425	0.649
PWWP3A	457.088	0.173	0.190	0.363	0.503	457.088	-0.267	0.191	0.162	0.453	457.088	-0.440	0.220	0.046	0.173
CORO6	12.820	1.726	0.863	0.045	0.107	12.820	0.824	0.875	0.347	0.602	12.820	-0.902	0.990	0.362	0.594
WDR73	338.282	-0.487	0.224	0.029	0.077	338.282	-0.258	0.222	0.247	0.521	338.282	0.230	0.259	0.374	0.606
ABHD13	200.991	-0.034	0.175	0.847	0.900	200.991	-0.487	0.177	0.006	0.297	200.991	-0.453	0.206	0.028	0.125
LTV1	528.075	-0.594	0.417	0.154	0.270	528.075	-1.113	0.418	0.008	0.297	528.075	-0.519	0.483	0.282	0.517
SERAC1	139.386	-0.040	0.226	0.861	0.909	139.386	-0.001	0.225	0.995	0.998	139.386	0.038	0.262	0.884	0.945
TIGD5	823.974	0.242	0.164	0.140	0.251	823.974	0.147	0.164	0.370	0.619	823.974	-0.095	0.189	0.616	0.789
PPFIBP2	78.450	0.050	0.257	0.845	0.898	78.450	0.344	0.252	0.172	0.462	78.450	0.294	0.295	0.319	0.554
PRPF38A	1226.813	-0.451	0.154	0.003	0.015	1226.813	-0.327	0.153	0.033	0.322	1226.813	0.124	0.178	0.486	0.697
TNS4	119.167	0.178	0.291	0.541	0.666	119.167	0.356	0.288	0.217	0.496	119.167	0.179	0.334	0.593	0.773
CGNL1	98.185	2.730	0.442	0.000	0.000	98.185	0.948	0.449	0.035	0.322	98.185	-1.782	0.508	0.000	0.007
MPND	212.970	0.399	0.225	0.076	0.159	212.970	0.302	0.224	0.178	0.467	212.970	-0.097	0.259	0.708	0.849
NUDCD1	706.512	-0.870	0.291	0.003	0.013	706.512	-0.651	0.291	0.025	0.316	706.512	0.219	0.337	0.515	0.719
RELT	384.518	0.194	0.264	0.462	0.596	384.518	-0.332	0.266	0.212	0.492	384.518	-0.526	0.307	0.086	0.256
SYTL1	292.503	0.337	0.564	0.549	0.672	292.503	1.057	0.563	0.060	0.350	292.503	0.720	0.650	0.268	0.503
UBASH3B	247.073	-0.570	0.428	0.183	0.307	247.073	-0.976	0.429	0.023	0.315	247.073	-0.406	0.496	0.413	0.639
PPFIBP1	1589.545	-1.111	0.234	0.000	0.000	1589.545	-0.024	0.233	0.918	0.959	1589.545	1.087	0.270	0.000	0.002
CCDC183	54.509	-0.070	0.256	0.786	0.858	54.509	0.171	0.249	0.492	0.707	54.509	0.240	0.294	0.413	0.639
FBXL20	354.588	0.315	0.120	0.009	0.031	354.588	0.205	0.120	0.088	0.380	354.588	-0.111	0.139	0.425	0.649
AJUBA	3501.039	-0.761	0.228	0.001	0.005	3501.039	0.207	0.228	0.364	0.616	3501.039	0.967	0.263	0.000	0.005
ALKBH6	225.739	0.949	0.246	0.000	0.001	225.739	0.095	0.248	0.701	0.839	225.739	-0.854	0.285	0.003	0.027
LSM10	383.807	-0.104	0.371	0.779	0.853	383.807	-0.766	0.372	0.039	0.327	383.807	-0.661	0.429	0.123	0.316
TOX2	306.822	-0.713	0.154	0.000	0.000	306.822	0.159	0.148	0.283	0.553	306.822	0.872	0.176	0.000	0.000
PPFIA4	126.711	1.170	1.120	0.296	0.434	126.711	1.297	1.120	0.247	0.521	126.711	0.127	1.292	0.922	0.964
ATG4D	448.479	0.229	0.167	0.170	0.290	448.479	0.092	0.166	0.579	0.764	448.479	-0.136	0.192	0.479	0.692
SNHG7	3061.659	0.128	0.295	0.664	0.766	3061.659	0.608	0.295	0.039	0.327	3061.659	0.480	0.340	0.158	0.370
MFSD5	321.158	0.614	0.185	0.001	0.005	321.158	-0.065	0.187	0.726	0.854	321.158	-0.679	0.215	0.002	0.018
DISP1	108.287	0.346	0.245	0.158	0.275	108.287	0.245	0.245	0.317	0.579	108.287	-0.102	0.283	0.720	0.854
FRMD5	183.182	-0.461	0.186	0.013	0.042	183.182	-0.372	0.184	0.043	0.331	183.182	0.089	0.216	0.681	0.832
RANBP3	1094.541	-0.166	0.193	0.390	0.529	1094.541	-0.391	0.193	0.043	0.330	1094.541	-0.225	0.223	0.313	0.548
MIR22HG	229.515	1.126	0.412	0.006	0.023	229.515	-0.682	0.425	0.109	0.401	229.515	-1.808	0.485	0.000	0.004
FAM222A-AS1	23.206	-1.310	0.544	0.016	0.049	23.206	-1.184	0.535	0.027	0.319	23.206	0.127	0.641	0.843	0.924
CEP19	53.072	0.349	0.241	0.148	0.262	53.072	0.102	0.243	0.674	0.824	53.072	-0.247	0.280	0.378	0.609
ARHGAP19	260.985	-1.052	0.227	0.000	0.000	260.985	-0.090	0.221	0.684	0.829	260.985	0.962	0.261	0.000	0.004
COX14	224.879	0.415	0.249	0.095	0.189	224.879	-0.254	0.251	0.312	0.575	224.879	-0.669	0.289	0.021	0.103
PPP1R16A	1082.903	0.925	0.203	0.000	0.000	1082.903	0.347	0.203	0.088	0.380	1082.903	-0.577	0.234	0.014	0.080
JMJD1C-AS1	60.596	-0.373	0.253	0.140	0.251	60.596	0.041	0.243	0.864	0.931	60.596	0.415	0.290	0.153	0.363
PPFIA2	10.867	0.784	0.618	0.205	0.332	10.867	0.067	0.634	0.916	0.958	10.867	-0.717	0.721	0.320	0.555
RBM17	1426.193	-0.349	0.227	0.123	0.228	1426.193	-0.338	0.226	0.135	0.426	1426.193	0.011	0.262	0.967	0.985
PIGY	1302.420	0.130	0.159	0.415	0.553	1302.420	-0.113	0.160	0.477	0.698	1302.420	-0.243	0.184	0.187	0.407
UBL7	851.226	-0.024	0.184	0.895	0.932	851.226	-0.158	0.184	0.391	0.635	851.226	-0.134	0.213	0.530	0.729
URB1-AS1	66.018	0.151	0.314	0.631	0.740	66.018	0.090	0.313	0.772	0.879	66.018	-0.060	0.363	0.868	0.937
PPFIA1	1219.843	-0.533	0.197	0.007	0.025	1219.843	-0.313	0.197	0.112	0.405	1219.843	0.221	0.228	0.333	0.568
MGC16275	35.835	0.531	0.382	0.165	0.285	35.835	0.548	0.379	0.149	0.441	35.835	0.017	0.437	0.968	0.985
FAM86B1	45.468	0.297	0.322	0.356	0.496	45.468	0.021	0.324	0.949	0.975	45.468	-0.277	0.374	0.459	0.676
PHYKPL	874.986	0.581	0.211	0.006	0.023	874.986	0.146	0.212	0.491	0.707	874.986	-0.435	0.244	0.075	0.235
SLC43A1	233.704	-0.735	0.238	0.002	0.010	233.704	-0.038	0.233	0.872	0.935	233.704	0.697	0.274	0.011	0.069
TCEAL3	198.459	1.085	0.177	0.000	0.000	198.459	-0.100	0.184	0.588	0.769	198.459	-1.185	0.208	0.000	0.000
TMEM128	162.951	0.050	0.160	0.756	0.838	162.951	-0.041	0.159	0.796	0.894	162.951	-0.091	0.186	0.624	0.794
TMEM141	367.516	0.437	0.177	0.014	0.043	367.516	0.004	0.178	0.980	0.989	367.516	-0.432	0.205	0.035	0.146
USP45	317.250	-0.912	0.207	0.000	0.000	317.250	-0.396	0.203	0.052	0.340	317.250	0.516	0.239	0.031	0.134
CFAP300	110.489	0.112	0.391	0.775	0.851	110.489	0.266	0.390	0.494	0.709	110.489	0.155	0.451	0.732	0.861
TMEM241	120.292	-0.330	0.229	0.149	0.263	120.292	-0.249	0.226	0.271	0.542	120.292	0.081	0.265	0.760	0.878
PKP4	1262.187	-0.762	0.117	0.000	0.000	1262.187	-0.242	0.116	0.036	0.324	1262.187	0.520	0.135	0.000	0.003
REPS1	456.824	-0.824	0.204	0.000	0.001	456.824	-0.407	0.202	0.044	0.331	456.824	0.417	0.235	0.076	0.238
TMEM60	141.981	1.068	0.198	0.000	0.000	141.981	-0.052	0.206	0.800	0.896	141.981	-1.121	0.233	0.000	0.000

ARRDC1-AS1	239.914	-0.196	0.274	0.475	0.608	239.914	-0.262	0.273	0.338	0.596	239.914	-0.066	0.317	0.835	0.920
SMIM3	1293.352	0.526	0.493	0.286	0.424	1293.352	0.686	0.493	0.164	0.454	1293.352	0.160	0.569	0.779	0.888
SNHG12	541.192	-0.063	0.282	0.822	0.884	541.192	0.514	0.280	0.067	0.355	541.192	0.577	0.325	0.075	0.236
PIK3R3	384.547	0.404	0.309	0.191	0.316	384.547	0.265	0.309	0.391	0.635	384.547	-0.139	0.357	0.697	0.841
PEX3	1320.826	-0.298	0.191	0.118	0.222	1320.826	-0.209	0.191	0.274	0.545	1320.826	0.090	0.221	0.684	0.833
PARG	469.981	-0.991	0.278	0.000	0.003	469.981	-0.580	0.277	0.036	0.324	469.981	0.411	0.322	0.202	0.425
CNTNAP1	2003.872	0.674	0.186	0.000	0.002	2003.872	0.532	0.186	0.004	0.297	2003.872	-0.143	0.215	0.506	0.712
ENC1	691.198	0.036	0.192	0.853	0.904	691.198	-0.078	0.192	0.683	0.829	691.198	-0.114	0.222	0.608	0.783
NIPSNAP1	1713.207	0.583	0.263	0.027	0.072	1713.207	0.431	0.263	0.101	0.394	1713.207	-0.152	0.303	0.617	0.789
NDST2	382.222	-0.141	0.179	0.431	0.567	382.222	-0.182	0.179	0.310	0.575	382.222	-0.040	0.208	0.847	0.926
MMP23B	5.804	0.825	1.012	0.415	0.553	5.804	0.134	1.031	0.897	0.948	5.804	-0.692	1.175	0.556	0.749
KCNAB2	1652.980	0.771	0.127	0.000	0.000	1652.980	0.210	0.128	0.100	0.394	1652.980	-0.561	0.147	0.000	0.003
ITGA10	46.361	-1.709	0.497	0.001	0.004	46.361	0.574	0.451	0.202	0.486	46.361	2.284	0.559	0.000	0.001
IKBKG	1206.578	0.235	0.220	0.285	0.423	1206.578	-0.430	0.220	0.051	0.338	1206.578	-0.666	0.254	0.009	0.061
ELP1	1031.034	-0.835	0.215	0.000	0.001	1031.034	-0.219	0.214	0.307	0.572	1031.034	0.616	0.248	0.013	0.078
IFITM1	2581.865	1.846	0.366	0.000	0.000	2581.865	0.757	0.366	0.039	0.327	2581.865	-1.089	0.423	0.010	0.066
HAT1	939.605	-0.959	0.363	0.008	0.029	939.605	-0.938	0.363	0.010	0.298	939.605	0.021	0.420	0.961	0.982
GAS7	9.514	0.130	0.506	0.797	0.867	9.514	-0.318	0.521	0.542	0.738	9.514	-0.448	0.603	0.458	0.676
H2BC12	581.583	0.579	0.502	0.249	0.383	581.583	0.613	0.502	0.222	0.501	581.583	0.034	0.580	0.953	0.978
DGKZ	957.841	-0.240	0.302	0.427	0.563	957.841	-0.637	0.302	0.035	0.322	957.841	-0.397	0.349	0.255	0.488
DGKE	99.486	-0.256	0.242	0.290	0.428	99.486	-0.469	0.242	0.053	0.342	99.486	-0.213	0.283	0.451	0.670
DGKD	736.967	-0.380	0.157	0.015	0.047	736.967	-0.006	0.155	0.969	0.984	736.967	0.374	0.181	0.038	0.154
DDO	57.569	0.651	0.505	0.197	0.324	57.569	0.355	0.506	0.483	0.701	57.569	-0.296	0.583	0.611	0.785
CST7	25.574	0.057	0.380	0.881	0.923	25.574	-0.241	0.384	0.531	0.731	25.574	-0.297	0.447	0.506	0.712
COL27A1	218.936	1.216	0.447	0.006	0.024	218.936	0.739	0.448	0.098	0.393	218.936	-0.477	0.515	0.355	0.588
FBF1	156.232	0.328	0.260	0.208	0.337	156.232	-0.462	0.264	0.080	0.371	156.232	-0.790	0.304	0.009	0.062
YBX3	12631.555	0.208	0.116	0.074	0.156	12631.555	0.260	0.116	0.026	0.316	12631.555	0.052	0.134	0.700	0.843
PPIL4	761.401	-0.132	0.151	0.381	0.521	761.401	-0.334	0.151	0.027	0.318	761.401	-0.202	0.175	0.247	0.478
PAQR8	62.025	0.569	0.433	0.189	0.313	62.025	0.709	0.431	0.100	0.394	62.025	0.140	0.497	0.778	0.888
CPZ	13.444	0.648	0.482	0.179	0.302	13.444	0.592	0.478	0.216	0.495	13.444	-0.056	0.547	0.918	0.962
ABCC11	84.275	-1.101	0.384	0.004	0.017	84.275	-0.414	0.376	0.270	0.541	84.275	0.687	0.443	0.121	0.313
COPS3	674.342	-0.637	0.225	0.005	0.019	674.342	-0.417	0.224	0.063	0.350	674.342	0.220	0.260	0.398	0.626
CHST1	4.747	6.177	1.156	0.000	0.000	4.747	1.141	1.630	0.484	0.701	4.747	-5.036	1.491	0.001	0.011
KRT87P	31.632	0.983	0.403	0.015	0.046	31.632	-1.542	0.459	0.001	0.277	31.632	-2.525	0.511	0.000	0.000
CBX4	1476.375	-0.425	0.182	0.019	0.056	1476.375	0.105	0.181	0.561	0.750	1476.375	0.530	0.210	0.011	0.071
SHISAL1	800.968	-0.410	0.204	0.044	0.105	800.968	-0.141	0.203	0.486	0.703	800.968	0.269	0.235	0.253	0.485
SHANK3	22.134	-0.695	0.503	0.167	0.287	22.134	-0.158	0.477	0.740	0.861	22.134	0.536	0.578	0.354	0.587
DGCR6L	433.232	0.603	0.225	0.007	0.027	433.232	0.135	0.226	0.551	0.744	433.232	-0.468	0.261	0.072	0.229
CAMK1	268.683	0.083	0.180	0.642	0.749	268.683	0.106	0.179	0.555	0.746	268.683	0.022	0.207	0.915	0.960
SYDE1	1563.646	0.358	0.157	0.023	0.064	1563.646	0.334	0.157	0.034	0.322	1563.646	-0.024	0.182	0.895	0.950
TRIM5	352.167	0.064	0.244	0.793	0.863	352.167	0.270	0.242	0.265	0.537	352.167	0.206	0.281	0.462	0.679
ZCCHC3	524.204	-0.390	0.097	0.000	0.001	524.204	0.105	0.094	0.261	0.533	524.204	0.495	0.112	0.000	0.000
ALG2	624.610	0.030	0.167	0.858	0.908	624.610	-0.043	0.167	0.795	0.893	624.610	-0.073	0.193	0.705	0.847
STRIP1	340.735	-0.687	0.251	0.006	0.023	340.735	-0.401	0.249	0.108	0.401	340.735	0.287	0.290	0.323	0.558
KIAA1656	10.040	1.177	0.635	0.064	0.139	10.040	1.203	0.629	0.056	0.343	10.040	0.026	0.707	0.970	0.985
RIMBP3	22.728	1.181	0.440	0.007	0.026	22.728	0.712	0.446	0.110	0.403	22.728	-0.470	0.502	0.349	0.583
MICALL1	1282.712	-0.803	0.288	0.005	0.021	1282.712	-0.613	0.287	0.033	0.322	1282.712	0.190	0.332	0.568	0.758
TUBGCP6	310.775	0.490	0.218	0.025	0.068	310.775	0.087	0.219	0.690	0.833	310.775	-0.403	0.252	0.111	0.297
KIAA1671	1217.142	-0.877	0.256	0.001	0.004	1217.142	-0.123	0.255	0.629	0.797	1217.142	0.754	0.295	0.011	0.069
API5	2381.919	-0.681	0.208	0.001	0.006	2381.919	-0.487	0.208	0.019	0.306	2381.919	0.194	0.241	0.420	0.644
FAM207A	970.298	-0.011	0.160	0.945	0.966	970.298	0.094	0.160	0.557	0.747	970.298	0.105	0.185	0.571	0.759
AGPS	1330.374	-0.479	0.235	0.042	0.101	1330.374	-0.605	0.235	0.010	0.299	1330.374	-0.126	0.272	0.644	0.807
EAF1	936.304	-0.587	0.177	0.001	0.005	936.304	-0.085	0.175	0.627	0.796	936.304	0.502	0.204	0.014	0.081
DNAJC14	950.707	-0.150	0.201	0.455	0.590	950.707	-0.381	0.201	0.058	0.348	950.707	-0.231	0.232	0.321	0.555
NKD1	20.336	0.334	0.414	0.421	0.558	20.336	0.209	0.413	0.613	0.787	20.336	-0.125	0.478	0.794	0.896
NKD2	42.092	-0.294	0.556	0.596	0.711	42.092	0.937	0.540	0.083	0.374	42.092	1.231	0.633	0.052	0.187
PPFIA3	193.275	0.726	0.168	0.000	0.000	193.275	0.154	0.170	0.366	0.617	193.275	-0.572	0.194	0.003	0.030
SLC45A3	52.522	-0.176	0.402	0.662	0.764	52.522	-0.383	0.403	0.341	0.599	52.522	-0.208	0.468	0.657	0.817
RHPN2	423.948	-0.521	0.210	0.013	0.042	423.948	-0.066	0.208	0.751	0.867	423.948	0.455	0.242	0.060	0.205
ZIC5	27.464	-0.559	0.421	0.184	0.307	27.464	-0.287	0.408	0.483	0.701	27.464	0.273	0.488	0.576	0.761
CCNB3	472.061	-0.623	0.258	0.016	0.048	472.061	0.242	0.256	0.344	0.601	472.061	0.865	0.297	0.004	0.033
APOL1	4134.340	1.672	0.512	0.001	0.006	4134.340	0.633	0.513	0.217	0.496	4134.340	-1.039	0.592	0.079	0.243
LMO4	805.807	-0.752	0.254	0.003	0.013	805.807	-0.450	0.253	0.075	0.364	805.807	0.302	0.293	0.302	0.537
ZCRB1	1017.900	1.074	0.316	0.001	0.004	1017.900	0.265	0.316	0.402	0.645	1017.900	-0.809	0.365	0.027	0.122
STON2	59.569	0.984	0.291	0.001	0.005	59.569	0.024	0.300	0.936	0.968	59.569	-0.960	0.340	0.005	0.040
PIR	1269.266	0.172	0.222	0.439	0.575	1269.266	0.121	0.222	0.585	0.768	1269.266	-0.050	0.256	0.844	0.924
DOCK7	755.354	-0.631	0.268	0.018	0.054	755.354	0.102	0.267	0.703	0.840	755.354	0.733	0.309	0.018	0.094

HELZ2	664.285	0.187	0.163	0.250	0.384	664.285	0.046	0.163	0.776	0.881	664.285	-0.141	0.188	0.453	0.672
KNDC1	15.164	0.470	0.503	0.350	0.490	15.164	1.232	0.481	0.010	0.299	15.164	0.762	0.560	0.174	0.390
LRRCC1	290.887	-0.170	0.215	0.431	0.567	290.887	-0.206	0.215	0.337	0.595	290.887	-0.037	0.249	0.882	0.944
CNTNAP4	146.311	-0.287	0.458	0.532	0.657	146.311	0.617	0.454	0.175	0.464	146.311	0.904	0.527	0.087	0.257
ZFHX2	13.190	1.049	0.491	0.032	0.084	13.190	0.450	0.505	0.373	0.622	13.190	-0.599	0.564	0.288	0.523
CGGBP1	926.076	-0.686	0.164	0.000	0.000	926.076	-0.206	0.162	0.204	0.487	926.076	0.480	0.189	0.011	0.070
ITPRIP	1342.095	-0.621	0.373	0.096	0.190	1342.095	-0.890	0.373	0.017	0.306	1342.095	-0.269	0.431	0.533	0.732
UNK	403.150	-0.256	0.139	0.065	0.141	403.150	0.042	0.137	0.760	0.872	403.150	0.298	0.160	0.063	0.211
CFAP74	6.063	-1.039	1.333	0.436	0.572	6.063	0.940	1.253	0.453	0.680	6.063	1.979	1.508	0.189	0.411
DISP2	96.551	1.360	0.272	0.000	0.000	96.551	0.562	0.277	0.042	0.330	96.551	-0.797	0.313	0.011	0.069
TNKS1BP1	4759.760	0.208	0.156	0.183	0.307	4759.760	0.265	0.156	0.089	0.382	4759.760	0.057	0.180	0.749	0.871
CIPC	220.513	-0.656	0.252	0.009	0.032	220.513	0.172	0.247	0.486	0.703	220.513	0.828	0.289	0.004	0.037
DIXDC1	281.984	0.047	0.315	0.882	0.924	281.984	0.160	0.314	0.612	0.786	281.984	0.113	0.364	0.756	0.875
CEP295	394.007	0.074	0.166	0.656	0.760	394.007	0.123	0.165	0.455	0.682	394.007	0.049	0.192	0.797	0.898
AP3B1	1512.229	-0.536	0.133	0.000	0.001	1512.229	-0.075	0.132	0.567	0.754	1512.229	0.460	0.153	0.003	0.026
ZNF518B	315.382	-0.235	0.200	0.240	0.373	315.382	-0.118	0.199	0.552	0.745	315.382	0.117	0.231	0.613	0.787
TANC1	440.763	-1.052	0.257	0.000	0.000	440.763	-0.292	0.254	0.251	0.525	440.763	0.760	0.296	0.010	0.067
FHDC1	16.145	1.995	0.437	0.000	0.000	16.145	0.093	0.500	0.852	0.924	16.145	-1.902	0.527	0.000	0.006
ZC3H12C	384.326	-0.868	0.363	0.017	0.051	384.326	-0.394	0.361	0.276	0.547	384.326	0.474	0.420	0.259	0.493
SSH2	216.723	-0.404	0.172	0.019	0.056	216.723	0.142	0.167	0.394	0.639	216.723	0.546	0.198	0.006	0.045
SELENOI	844.995	-1.020	0.336	0.002	0.011	844.995	-0.783	0.336	0.020	0.306	844.995	0.237	0.388	0.542	0.739
LBX2	9.683	0.829	0.774	0.284	0.422	9.683	0.627	0.775	0.419	0.656	9.683	-0.203	0.886	0.819	0.910
GFM1	1020.356	-1.403	0.369	0.000	0.001	1020.356	-1.023	0.369	0.006	0.297	1020.356	0.381	0.427	0.372	0.604
CCDC65	15.383	0.924	0.440	0.036	0.090	15.383	0.187	0.459	0.683	0.829	15.383	-0.736	0.514	0.152	0.361
BLZF1	384.856	-0.539	0.235	0.022	0.062	384.856	-0.454	0.234	0.053	0.342	384.856	0.086	0.272	0.753	0.874
TSLP	20.616	-1.545	0.469	0.001	0.006	20.616	-0.499	0.414	0.228	0.506	20.616	1.046	0.535	0.050	0.184
LGR5	10.712	0.559	0.525	0.286	0.424	10.712	0.495	0.520	0.340	0.598	10.712	-0.064	0.596	0.914	0.960
RPPH1	25.170	-0.364	0.449	0.417	0.555	25.170	-0.143	0.436	0.744	0.863	25.170	0.221	0.519	0.670	0.824
MAPKAPK5	552.872	-0.491	0.184	0.008	0.028	552.872	-0.143	0.183	0.434	0.667	552.872	0.348	0.213	0.102	0.284
INE2	18.672	0.315	0.460	0.494	0.626	18.672	0.556	0.449	0.216	0.495	18.672	0.241	0.522	0.644	0.807
BHLHE40	6911.911	1.864	0.244	0.000	0.000	6911.911	0.494	0.245	0.044	0.331	6911.911	-1.371	0.282	0.000	0.000
PIAS1	619.941	-0.443	0.178	0.013	0.041	619.941	-0.036	0.177	0.839	0.916	619.941	0.407	0.206	0.048	0.177
CDC14B	583.015	-0.037	0.236	0.876	0.920	583.015	0.072	0.236	0.761	0.873	583.015	0.108	0.273	0.691	0.838
CDC14A	40.480	-0.416	0.364	0.254	0.388	40.480	0.044	0.352	0.900	0.950	40.480	0.460	0.418	0.272	0.506
CDK10	428.021	0.424	0.224	0.058	0.129	428.021	-0.046	0.224	0.839	0.916	428.021	-0.470	0.259	0.069	0.225
PRPF18	350.841	-0.625	0.222	0.005	0.019	350.841	-0.495	0.220	0.025	0.316	350.841	0.129	0.257	0.614	0.787
DEGS1	1346.589	0.479	0.225	0.033	0.085	1346.589	-0.541	0.226	0.017	0.306	1346.589	-1.021	0.261	0.000	0.002
DENR	1682.315	-0.748	0.205	0.000	0.002	1682.315	-0.374	0.204	0.067	0.356	1682.315	0.374	0.237	0.114	0.302
THOC5	707.005	-0.878	0.262	0.001	0.005	707.005	-0.543	0.261	0.038	0.327	707.005	0.335	0.303	0.269	0.503
KMO	411.282	0.603	0.530	0.256	0.390	411.282	0.751	0.530	0.156	0.448	411.282	0.149	0.612	0.808	0.904
YARS1	5533.916	-0.955	0.274	0.000	0.003	5533.916	-0.358	0.274	0.192	0.477	5533.916	0.597	0.317	0.059	0.203
PDXK	3323.743	0.467	0.074	0.000	0.000	3323.743	0.137	0.074	0.064	0.350	3323.743	-0.330	0.085	0.000	0.003
MADD	843.013	-0.297	0.197	0.132	0.240	843.013	0.252	0.196	0.199	0.482	843.013	0.549	0.227	0.016	0.088
RRP1	1297.161	-0.237	0.303	0.433	0.569	1297.161	-0.461	0.303	0.127	0.420	1297.161	-0.224	0.350	0.522	0.724
MKNK1	277.685	0.051	0.143	0.721	0.810	277.685	0.278	0.140	0.047	0.336	277.685	0.227	0.164	0.165	0.380
CAV1	4623.846	-0.455	0.169	0.007	0.026	4623.846	0.009	0.169	0.958	0.978	4623.846	0.464	0.195	0.017	0.093
KHSRP	6556.083	-0.215	0.178	0.228	0.359	6556.083	-0.402	0.178	0.024	0.316	6556.083	-0.187	0.206	0.364	0.597
PDLIM4	1211.474	1.156	0.201	0.000	0.000	1211.474	0.131	0.202	0.515	0.721	1211.474	-1.025	0.232	0.000	0.000
CASK	488.625	-0.624	0.145	0.000	0.000	488.625	0.048	0.141	0.734	0.858	488.625	0.671	0.166	0.000	0.001
AKR7A2	964.983	0.109	0.182	0.551	0.674	964.983	0.456	0.182	0.012	0.305	964.983	0.348	0.210	0.098	0.277
PRKRA	619.356	-0.264	0.180	0.143	0.256	619.356	0.194	0.179	0.278	0.548	619.356	0.457	0.208	0.028	0.125
STK16	475.521	0.135	0.179	0.450	0.586	475.521	0.430	0.178	0.015	0.306	475.521	0.295	0.206	0.152	0.361
SCARF1	12.010	2.705	0.887	0.002	0.011	12.010	1.592	0.911	0.081	0.371	12.010	-1.113	0.984	0.258	0.492
CAV2	1006.978	0.068	0.123	0.579	0.697	1006.978	-0.007	0.123	0.954	0.977	1006.978	-0.075	0.142	0.596	0.775
GTPBP10	321.658	-0.883	0.357	0.014	0.043	321.658	-0.968	0.357	0.007	0.297	321.658	-0.085	0.414	0.837	0.921
ACTL6A	1736.001	-0.609	0.266	0.022	0.062	1736.001	-0.638	0.266	0.016	0.306	1736.001	-0.029	0.307	0.926	0.966
RUNX2	81.277	-1.185	0.409	0.004	0.016	81.277	-1.039	0.405	0.010	0.299	81.277	0.146	0.477	0.759	0.878
RGS20	35.214	0.723	0.345	0.036	0.090	35.214	0.497	0.346	0.150	0.443	35.214	-0.226	0.394	0.566	0.756
NOP14	2001.626	-0.780	0.233	0.001	0.005	2001.626	-0.283	0.233	0.224	0.503	2001.626	0.497	0.269	0.065	0.215
FAM193A	552.145	-0.313	0.102	0.002	0.010	552.145	-0.089	0.100	0.372	0.621	552.145	0.224	0.117	0.056	0.197
SLC25A12	377.344	-0.391	0.181	0.031	0.081	377.344	-0.336	0.180	0.063	0.350	377.344	0.055	0.210	0.794	0.896
PLA2G4C	65.482	2.212	0.301	0.000	0.000	65.482	0.399	0.338	0.239	0.515	65.482	-1.813	0.354	0.000	0.000
RUVBL1	2688.838	-0.706	0.302	0.020	0.057	2688.838	-0.690	0.302	0.023	0.315	2688.838	0.016	0.349	0.962	0.982
KLF7	832.901	0.233	0.158	0.140	0.251	832.901	-0.119	0.158	0.453	0.680	832.901	-0.352	0.183	0.054	0.193
RUNX1	613.160	-0.454	0.238	0.056	0.126	613.160	-0.276	0.237	0.244	0.519	613.160	0.178	0.275	0.518	0.722
PLPP1	1143.963	0.654	0.175	0.000	0.002	1143.963	0.099	0.175	0.570	0.756	1143.963	-0.555	0.202	0.006	0.047
PLPP2	716.214	0.773	0.142	0.000	0.000	716.214	0.144	0.143	0.314	0.577	716.214	-0.629	0.164	0.000	0.003

PLPP3	400.337	1.705	0.333	0.000	0.000	400.337	0.421	0.335	0.209	0.490	400.337	-1.284	0.384	0.001	0.012
STC2	4342.913	-1.155	0.476	0.015	0.047	4342.913	-1.319	0.476	0.006	0.297	4342.913	-0.164	0.550	0.766	0.881
USO1	2381.038	-0.657	0.270	0.015	0.046	2381.038	0.168	0.269	0.532	0.732	2381.038	0.825	0.311	0.008	0.057
CADPS	20.353	0.256	0.392	0.514	0.643	20.353	0.116	0.391	0.768	0.877	20.353	-0.140	0.454	0.757	0.876
CDK13	610.337	-0.437	0.191	0.022	0.062	610.337	-0.208	0.190	0.274	0.545	610.337	0.229	0.221	0.299	0.534
PDE8B	61.968	0.123	0.269	0.647	0.753	61.968	0.318	0.264	0.228	0.506	61.968	0.195	0.308	0.527	0.727
ASMTL	2276.870	0.097	0.138	0.480	0.613	2276.870	-0.195	0.138	0.157	0.449	2276.870	-0.292	0.159	0.066	0.218
PSMG1	1565.241	-0.415	0.293	0.156	0.273	1565.241	-0.580	0.293	0.048	0.336	1565.241	-0.165	0.338	0.626	0.796
RFXANK	637.840	0.561	0.194	0.004	0.016	637.840	-0.012	0.195	0.953	0.977	637.840	-0.573	0.225	0.011	0.069
TP63	2032.059	-0.834	0.209	0.000	0.001	2032.059	-0.354	0.208	0.089	0.383	2032.059	0.480	0.241	0.046	0.174
JRK	437.118	-0.439	0.240	0.068	0.145	437.118	0.199	0.238	0.403	0.645	437.118	0.639	0.277	0.021	0.105
HSD17B6	60.040	-0.886	0.345	0.010	0.034	60.040	0.051	0.330	0.877	0.938	60.040	0.937	0.395	0.018	0.094
DNAH17	50.400	1.083	0.321	0.001	0.005	50.400	0.139	0.333	0.675	0.825	50.400	-0.944	0.374	0.012	0.072
UNC5C	126.005	-0.046	0.527	0.931	0.956	126.005	0.651	0.524	0.214	0.493	126.005	0.697	0.607	0.251	0.483
RTCA	887.301	-0.168	0.098	0.086	0.174	887.301	-0.045	0.097	0.642	0.805	887.301	0.123	0.113	0.276	0.511
RNASET2	304.887	1.084	0.402	0.007	0.026	304.887	0.635	0.402	0.114	0.406	304.887	-0.448	0.464	0.334	0.568
SSNA1	1046.405	0.234	0.269	0.384	0.523	1046.405	-0.325	0.270	0.228	0.506	1046.405	-0.559	0.311	0.072	0.230
OASL	1555.544	3.714	0.481	0.000	0.000	1555.544	1.093	0.482	0.023	0.316	1555.544	-2.621	0.555	0.000	0.000
AOC3	170.598	-0.122	0.902	0.892	0.931	170.598	0.937	0.899	0.298	0.565	170.598	1.059	1.040	0.309	0.544
DCHS1	13.340	1.153	0.449	0.010	0.035	13.340	0.721	0.457	0.115	0.406	13.340	-0.432	0.508	0.396	0.624
AKR1C3	2984.114	1.522	0.263	0.000	0.000	2984.114	0.218	0.263	0.408	0.649	2984.114	-1.304	0.303	0.000	0.001
NCOA1	211.895	0.077	0.171	0.651	0.756	211.895	0.076	0.170	0.654	0.811	211.895	-0.001	0.198	0.994	0.997
LAMTOR3	474.130	0.383	0.131	0.003	0.015	474.130	-0.042	0.132	0.752	0.867	474.130	-0.425	0.152	0.005	0.042
CBFB	960.886	-0.279	0.198	0.160	0.279	960.886	-0.520	0.199	0.009	0.297	960.886	-0.242	0.230	0.293	0.528
NUMB	1475.617	-0.411	0.114	0.000	0.002	1475.617	-0.133	0.113	0.240	0.516	1475.617	0.278	0.131	0.035	0.145
SOCS1	204.553	1.461	0.520	0.005	0.020	204.553	0.736	0.521	0.158	0.450	204.553	-0.725	0.600	0.227	0.455
DDX3Y	22.835	0.625	0.359	0.082	0.168	22.835	0.637	0.354	0.072	0.361	22.835	0.012	0.405	0.977	0.988
PDE5A	113.624	0.314	0.406	0.439	0.575	113.624	0.327	0.405	0.420	0.657	113.624	0.013	0.468	0.978	0.989
DYNLL1	2174.855	0.627	0.179	0.000	0.003	2174.855	-0.181	0.179	0.314	0.577	2174.855	-0.808	0.207	0.000	0.002
TNKS	1269.945	-0.656	0.310	0.034	0.087	1269.945	0.335	0.309	0.279	0.549	1269.945	0.992	0.358	0.006	0.044
ALDH4A1	278.451	0.483	0.212	0.023	0.064	278.451	-0.030	0.214	0.889	0.945	278.451	-0.513	0.246	0.037	0.151
SERPINA6	18.190	0.809	0.492	0.100	0.196	18.190	-0.478	0.527	0.364	0.616	18.190	-1.287	0.592	0.030	0.131
IRS2	1080.598	-0.152	0.280	0.587	0.703	1080.598	0.472	0.279	0.090	0.385	1080.598	0.625	0.323	0.053	0.190
EIF3A	10094.872	-1.125	0.174	0.000	0.000	10094.872	-0.351	0.174	0.044	0.331	10094.872	0.774	0.201	0.000	0.003
EIF3B	9756.271	-0.698	0.222	0.002	0.009	9756.271	-0.386	0.222	0.082	0.374	9756.271	0.312	0.256	0.224	0.451
EIF3C	10214.400	-0.055	0.123	0.655	0.758	10214.400	-0.016	0.123	0.894	0.947	10214.400	0.039	0.142	0.786	0.893
EIF3D	5689.525	-0.355	0.112	0.002	0.008	5689.525	0.106	0.112	0.342	0.599	5689.525	0.461	0.129	0.000	0.006
EIF3F	4985.160	0.300	0.227	0.186	0.311	4985.160	0.313	0.227	0.169	0.459	4985.160	0.012	0.263	0.963	0.983
EIF3G	4112.708	0.163	0.152	0.284	0.422	4112.708	0.116	0.152	0.446	0.676	4112.708	-0.047	0.175	0.789	0.895
EIF3H	5965.842	0.005	0.192	0.978	0.986	5965.842	0.254	0.192	0.185	0.472	5965.842	0.249	0.221	0.261	0.495
EIF3I	6194.387	-0.037	0.174	0.832	0.889	6194.387	-0.199	0.174	0.255	0.528	6194.387	-0.162	0.201	0.422	0.646
EIF3J	2157.317	-0.324	0.281	0.249	0.384	2157.317	-0.545	0.281	0.052	0.341	2157.317	-0.222	0.325	0.495	0.703
CBL	1199.869	-0.195	0.177	0.272	0.409	1199.869	-0.052	0.177	0.767	0.877	1199.869	0.142	0.205	0.487	0.698
EIF4G3	1762.290	-0.070	0.128	0.584	0.701	1762.290	-0.222	0.128	0.082	0.373	1762.290	-0.152	0.148	0.303	0.538
VAMP8	41.069	0.755	0.322	0.019	0.056	41.069	0.370	0.326	0.257	0.530	41.069	-0.385	0.371	0.299	0.534
VAMP4	289.777	-0.288	0.211	0.171	0.292	289.777	0.185	0.208	0.372	0.621	289.777	0.474	0.242	0.051	0.184
STX16	773.425	0.248	0.175	0.156	0.273	773.425	-0.094	0.176	0.593	0.774	773.425	-0.342	0.203	0.091	0.265
STX11	91.916	0.510	0.338	0.132	0.241	91.916	-0.420	0.345	0.224	0.503	91.916	-0.929	0.396	0.019	0.098
STX10	1014.236	0.407	0.172	0.018	0.052	1014.236	0.175	0.172	0.310	0.574	1014.236	-0.233	0.198	0.241	0.471
BECN1	973.874	-0.362	0.154	0.018	0.054	973.874	-0.204	0.153	0.181	0.469	973.874	0.157	0.177	0.375	0.607
CBLB	583.672	-0.470	0.204	0.021	0.060	583.672	-0.042	0.203	0.836	0.916	583.672	0.429	0.236	0.069	0.224
JMJD7-PLA2G4B	20.503	0.560	0.509	0.272	0.408	20.503	0.555	0.506	0.273	0.544	20.503	-0.005	0.583	0.993	0.996
PEA15	4182.611	-0.097	0.190	0.611	0.723	4182.611	-0.108	0.190	0.569	0.756	4182.611	-0.011	0.219	0.959	0.981
SRSF9	3530.089	0.139	0.172	0.421	0.558	3530.089	-0.093	0.172	0.590	0.771	3530.089	-0.232	0.199	0.245	0.476
MARCO	26.391	2.401	0.512	0.000	0.000	26.391	1.128	0.529	0.033	0.322	26.391	-1.273	0.578	0.028	0.125
JRKL	213.915	-0.406	0.198	0.040	0.098	213.915	-0.021	0.194	0.914	0.957	213.915	0.385	0.228	0.091	0.265
HYAL2	419.929	0.592	0.231	0.011	0.035	419.929	-0.312	0.234	0.181	0.469	419.929	-0.904	0.269	0.001	0.011
DGAT1	589.759	0.502	0.195	0.010	0.035	589.759	0.075	0.196	0.701	0.839	589.759	-0.426	0.226	0.059	0.203
CDC23	576.798	-0.271	0.156	0.083	0.170	576.798	-0.217	0.156	0.163	0.453	576.798	0.054	0.181	0.767	0.881
S1PR4	11.064	-0.018	0.751	0.980	0.988	11.064	1.835	0.678	0.007	0.297	11.064	1.854	0.818	0.023	0.112
ACTN1	7596.488	0.233	0.091	0.010	0.035	7596.488	-0.111	0.091	0.222	0.501	7596.488	-0.344	0.105	0.001	0.014
DNAH11	285.320	-0.141	0.178	0.428	0.565	285.320	0.039	0.177	0.826	0.909	285.320	0.180	0.206	0.381	0.611
B4GALT4	337.958	0.320	0.137	0.019	0.056	337.958	-0.049	0.138	0.723	0.852	337.958	-0.369	0.159	0.020	0.102
B4GALT3	513.046	-0.268	0.160	0.093	0.186	513.046	0.090	0.158	0.568	0.755	513.046	0.358	0.184	0.051	0.186
B4GALT2	1792.375	0.121	0.207	0.559	0.680	1792.375	-0.117	0.207	0.573	0.759	1792.375	-0.238	0.239	0.320	0.555
B3GALT4	51.383	1.291	0.635	0.042	0.101	51.383	1.043	0.635	0.101	0.394	51.383	-0.248	0.728	0.733	0.862
B3GALNT1	47.777	-0.437	0.266	0.100	0.196	47.777	-0.487	0.263	0.065	0.353	47.777	-0.049	0.313	0.875	0.940

SERPINH1	5057.600	0.693	0.120	0.000	0.000	5057.600	0.105	0.121	0.383	0.629	5057.600	-0.587	0.139	0.000	0.001
TNK1	41.404	0.501	0.319	0.116	0.219	41.404	0.489	0.316	0.122	0.413	41.404	-0.011	0.364	0.975	0.987
ABCC3	3321.564	0.660	0.250	0.008	0.029	3321.564	0.574	0.250	0.022	0.314	3321.564	-0.086	0.288	0.766	0.881
TRADD	525.426	0.808	0.189	0.000	0.000	525.426	0.330	0.189	0.082	0.373	525.426	-0.478	0.218	0.028	0.126
PNPT1	917.361	-0.937	0.363	0.010	0.034	917.361	-0.962	0.363	0.008	0.297	917.361	-0.025	0.419	0.953	0.978
TNFRSF25	31.751	0.995	0.501	0.047	0.110	31.751	0.728	0.502	0.147	0.439	31.751	-0.267	0.573	0.642	0.805
MBTPS1	2706.784	-0.215	0.115	0.061	0.134	2706.784	-0.019	0.114	0.866	0.931	2706.784	0.196	0.133	0.139	0.343
EDF1	3406.368	0.724	0.221	0.001	0.006	3406.368	0.135	0.221	0.540	0.737	3406.368	-0.588	0.255	0.021	0.105
CTSF	179.846	0.822	0.593	0.166	0.285	179.846	0.897	0.593	0.130	0.422	179.846	0.074	0.684	0.913	0.960
SNX4	805.931	-0.482	0.168	0.004	0.017	805.931	-0.201	0.167	0.229	0.507	805.931	0.282	0.194	0.146	0.353
SNX3	4379.006	0.288	0.143	0.044	0.106	4379.006	0.155	0.143	0.280	0.549	4379.006	-0.133	0.166	0.421	0.645
URI1	1446.506	-0.841	0.145	0.000	0.000	1446.506	-0.088	0.144	0.540	0.737	1446.506	0.753	0.168	0.000	0.000
EED	301.650	-0.212	0.214	0.321	0.461	301.650	-0.429	0.214	0.045	0.334	301.650	-0.217	0.249	0.383	0.612
CTNNAL1	1715.661	-0.719	0.171	0.000	0.000	1715.661	-0.162	0.170	0.342	0.599	1715.661	0.558	0.197	0.005	0.039
ADAM19	9.874	1.892	0.593	0.001	0.008	9.874	-0.830	0.695	0.232	0.510	9.874	-2.723	0.753	0.000	0.005
GBF1	1210.516	-0.327	0.200	0.101	0.197	1210.516	-0.367	0.200	0.066	0.354	1210.516	-0.040	0.231	0.863	0.935
CBR1	1552.617	0.443	0.157	0.005	0.019	1552.617	-0.235	0.158	0.135	0.426	1552.617	-0.679	0.182	0.000	0.004
RNMT	1072.128	-0.391	0.254	0.123	0.228	1072.128	-0.225	0.253	0.375	0.623	1072.128	0.167	0.293	0.569	0.758
RNGTT	403.202	-0.760	0.233	0.001	0.006	403.202	-0.436	0.232	0.060	0.350	403.202	0.325	0.269	0.228	0.457
GPAA1	1247.497	-0.279	0.135	0.038	0.094	1247.497	-0.032	0.134	0.814	0.902	1247.497	0.248	0.156	0.111	0.298
MYOM1	29.550	0.073	0.845	0.931	0.956	29.550	0.505	0.840	0.548	0.742	29.550	0.432	0.973	0.657	0.817
RIPK1	342.421	0.015	0.169	0.928	0.955	342.421	-0.235	0.169	0.166	0.456	342.421	-0.250	0.196	0.203	0.427
CRADD	437.721	0.076	0.276	0.782	0.855	437.721	0.506	0.275	0.065	0.354	437.721	0.429	0.318	0.177	0.394
HRK	14.092	5.446	0.755	0.000	0.000	14.092	1.156	0.921	0.209	0.490	14.092	-4.289	0.815	0.000	0.000
CBR3	98.696	0.884	0.300	0.003	0.014	98.696	0.387	0.302	0.200	0.483	98.696	-0.497	0.346	0.151	0.360
TNFSF14	36.482	3.053	0.555	0.000	0.000	36.482	1.062	0.572	0.063	0.350	36.482	-1.991	0.634	0.002	0.019
TNFSF13	20.814	-0.123	0.523	0.814	0.878	20.814	0.302	0.509	0.553	0.745	20.814	0.425	0.599	0.477	0.691
TNFSF12	52.785	1.971	0.419	0.000	0.000	52.785	0.477	0.431	0.268	0.540	52.785	-1.495	0.485	0.002	0.022
TNFSF9	106.630	3.471	0.367	0.000	0.000	106.630	0.610	0.395	0.122	0.414	106.630	-2.861	0.425	0.000	0.000
ADAM21	11.818	0.681	0.587	0.246	0.379	11.818	0.210	0.595	0.724	0.852	11.818	-0.471	0.680	0.488	0.699
CBS	273.734	0.318	0.271	0.240	0.373	273.734	0.619	0.269	0.022	0.314	273.734	0.301	0.311	0.334	0.569
ADAM15	2993.369	0.452	0.130	0.000	0.003	2993.369	0.206	0.130	0.112	0.405	2993.369	-0.246	0.150	0.101	0.282
ADAM9	3092.655	-0.822	0.297	0.006	0.022	3092.655	0.142	0.297	0.632	0.799	3092.655	0.964	0.343	0.005	0.041
CDS2	704.828	0.143	0.188	0.448	0.583	704.828	0.254	0.188	0.177	0.466	704.828	0.111	0.217	0.610	0.785
PABPC4	6227.624	-0.099	0.138	0.473	0.607	6227.624	0.253	0.138	0.066	0.354	6227.624	0.352	0.159	0.027	0.122
CD164	3691.726	-0.122	0.133	0.362	0.503	3691.726	0.004	0.133	0.973	0.985	3691.726	0.126	0.154	0.413	0.639
TNFRSF14	164.155	1.737	0.497	0.000	0.003	164.155	0.932	0.499	0.062	0.350	164.155	-0.805	0.573	0.160	0.373
RAB11A	1762.297	0.405	0.080	0.000	0.000	1762.297	-0.093	0.080	0.245	0.520	1762.297	-0.498	0.092	0.000	0.000
RIPK2	443.006	-0.884	0.193	0.000	0.000	443.006	-0.087	0.189	0.646	0.807	443.006	0.797	0.222	0.000	0.006
FADD	655.715	-0.012	0.203	0.952	0.971	655.715	-0.331	0.204	0.104	0.397	655.715	-0.319	0.235	0.175	0.393
SNAP23	660.399	-0.063	0.127	0.618	0.729	660.399	-0.113	0.127	0.371	0.620	660.399	-0.050	0.147	0.733	0.862
NAPG	269.192	0.038	0.193	0.846	0.899	269.192	-0.307	0.194	0.115	0.406	269.192	-0.344	0.225	0.126	0.321
NAPA	1154.576	0.888	0.252	0.000	0.003	1154.576	-0.581	0.254	0.022	0.314	1154.576	-1.469	0.292	0.000	0.000
MTMR1	738.243	-0.623	0.159	0.000	0.001	738.243	-0.068	0.158	0.665	0.818	738.243	0.555	0.184	0.003	0.026
GGACT	99.896	0.427	0.256	0.095	0.189	99.896	0.342	0.255	0.181	0.469	99.896	-0.085	0.294	0.772	0.884
MPDZ	404.847	-0.518	0.232	0.025	0.069	404.847	-0.135	0.230	0.556	0.747	404.847	0.383	0.267	0.152	0.361
RIOK3	2464.035	-0.203	0.284	0.474	0.608	2464.035	0.424	0.283	0.134	0.426	2464.035	0.627	0.327	0.055	0.195
FPGT	104.584	-0.728	0.290	0.012	0.039	104.584	-0.350	0.285	0.219	0.498	104.584	0.378	0.336	0.260	0.494
TNFRSF11A	218.401	-0.071	0.293	0.808	0.874	218.401	0.155	0.291	0.594	0.774	218.401	0.227	0.338	0.503	0.710
TNFRSF10D	1121.530	-0.484	0.514	0.347	0.487	1121.530	0.750	0.513	0.144	0.435	1121.530	1.234	0.593	0.038	0.153
TNFRSF10B	2734.386	-0.554	0.187	0.003	0.013	2734.386	-0.208	0.187	0.266	0.538	2734.386	0.347	0.216	0.108	0.294
TNFRSF10A	576.929	-0.870	0.293	0.003	0.013	576.929	-0.527	0.292	0.071	0.360	576.929	0.343	0.339	0.311	0.546
DYRK4	204.027	0.548	0.222	0.014	0.043	204.027	0.025	0.224	0.910	0.955	204.027	-0.523	0.258	0.042	0.165
PEX11B	274.536	0.393	0.185	0.034	0.086	274.536	0.139	0.185	0.452	0.680	274.536	-0.253	0.214	0.236	0.465
PEX11A	349.276	0.421	0.439	0.338	0.478	349.276	0.588	0.438	0.180	0.468	349.276	0.167	0.506	0.741	0.867
SUCLG2	2321.262	-0.486	0.271	0.073	0.154	2321.262	0.252	0.270	0.351	0.605	2321.262	0.738	0.313	0.018	0.096
SUCLG1	2805.235	0.242	0.200	0.226	0.357	2805.235	-0.287	0.200	0.152	0.445	2805.235	-0.529	0.231	0.022	0.108
SUCLA2	684.878	-0.608	0.152	0.000	0.001	684.878	-0.193	0.151	0.201	0.484	684.878	0.415	0.176	0.018	0.096
CREG1	2362.608	-0.133	0.282	0.638	0.745	2362.608	0.437	0.282	0.121	0.413	2362.608	0.570	0.326	0.080	0.245
TRIM24	816.737	0.562	0.162	0.001	0.004	816.737	0.027	0.163	0.869	0.933	816.737	-0.535	0.188	0.004	0.038
IL18R1	1762.802	-0.267	0.127	0.036	0.091	1762.802	0.413	0.126	0.001	0.297	1762.802	0.679	0.147	0.000	0.000
CCNK	1305.484	-0.032	0.201	0.874	0.919	1305.484	-0.454	0.201	0.024	0.316	1305.484	-0.422	0.233	0.070	0.225
DPM1	759.892	-0.371	0.246	0.132	0.240	759.892	-0.414	0.246	0.092	0.387	759.892	-0.044	0.285	0.878	0.942
CDKL1	29.652	-0.738	0.335	0.028	0.074	29.652	-0.323	0.318	0.309	0.573	29.652	0.415	0.388	0.285	0.520
BANF1	2632.216	0.717	0.306	0.019	0.055	2632.216	-0.322	0.306	0.292	0.560	2632.216	-1.040	0.353	0.003	0.030
DCAF5	896.199	-0.096	0.157	0.539	0.663	896.199	0.047	0.156	0.765	0.875	896.199	0.143	0.181	0.429	0.652
DPM2	906.094	0.048	0.283	0.865	0.913	906.094	-0.446	0.283	0.115	0.407	906.094	-0.494	0.327	0.131	0.328

SAP30	435.735	0.434	0.209	0.037	0.093	435.735	0.225	0.209	0.280	0.550	435.735	-0.209	0.241	0.386	0.615
INPP4B	552.920	-0.590	0.246	0.016	0.050	552.920	0.136	0.244	0.577	0.762	552.920	0.726	0.284	0.010	0.068
CES2	786.345	-0.102	0.217	0.640	0.747	786.345	0.248	0.216	0.251	0.525	786.345	0.350	0.250	0.162	0.376
LIN7A	57.637	0.776	0.425	0.068	0.145	57.637	0.471	0.426	0.269	0.540	57.637	-0.305	0.489	0.533	0.732
IQGAP1	4575.307	-0.432	0.185	0.020	0.057	4575.307	-0.183	0.185	0.323	0.584	4575.307	0.249	0.214	0.244	0.475
NRP2	66.174	-1.514	0.630	0.016	0.049	66.174	-2.112	0.635	0.001	0.293	66.174	-0.599	0.739	0.418	0.642
NRP1	945.824	-0.929	0.469	0.048	0.111	945.824	-1.293	0.469	0.006	0.297	945.824	-0.365	0.542	0.501	0.708
KYAT1	18.462	-0.284	0.434	0.513	0.641	18.462	0.041	0.419	0.922	0.961	18.462	0.325	0.499	0.515	0.719
SYNGAP1	569.955	0.181	0.341	0.596	0.711	569.955	0.553	0.341	0.105	0.397	569.955	0.372	0.394	0.345	0.579
GMPS	2789.588	-0.430	0.190	0.024	0.065	2789.588	-0.335	0.190	0.077	0.368	2789.588	0.094	0.219	0.668	0.823
TMEM11	306.755	0.098	0.271	0.717	0.807	306.755	-0.487	0.272	0.074	0.363	306.755	-0.585	0.314	0.063	0.211
SOCS2	80.426	-0.536	0.222	0.016	0.048	80.426	0.023	0.211	0.913	0.957	80.426	0.559	0.254	0.028	0.125
GGH	1204.593	0.058	0.159	0.716	0.807	1204.593	-0.042	0.159	0.793	0.892	1204.593	-0.100	0.184	0.589	0.769
CFLAR	1414.218	0.213	0.135	0.116	0.218	1414.218	0.035	0.135	0.797	0.894	1414.218	-0.178	0.156	0.255	0.489
CCN6	19.149	-0.657	0.509	0.197	0.323	19.149	-0.659	0.503	0.191	0.477	19.149	-0.001	0.597	0.998	0.999
CCN5	2735.984	-0.151	1.001	0.880	0.922	2735.984	0.786	1.001	0.432	0.666	2735.984	0.937	1.156	0.418	0.642
CCN4	148.934	0.316	0.305	0.300	0.439	148.934	-0.078	0.306	0.799	0.895	148.934	-0.394	0.353	0.265	0.499
HDAC3	1383.695	0.015	0.188	0.937	0.960	1383.695	0.268	0.187	0.153	0.445	1383.695	0.253	0.217	0.244	0.475
HCAR3	35.121	2.803	0.536	0.000	0.000	35.121	1.046	0.567	0.065	0.353	35.121	-1.757	0.606	0.004	0.034
KSR1	123.823	-0.178	0.312	0.567	0.687	123.823	0.210	0.308	0.496	0.710	123.823	0.388	0.359	0.279	0.514
ANKRD13A	874.843	-0.478	0.179	0.007	0.027	874.843	-0.121	0.178	0.496	0.710	874.843	0.357	0.206	0.083	0.250
ALKBH1	127.216	-0.816	0.281	0.004	0.016	127.216	-0.490	0.276	0.077	0.367	127.216	0.326	0.326	0.317	0.552
DLEU2	101.077	-1.089	0.292	0.000	0.002	101.077	-0.737	0.286	0.010	0.298	101.077	0.352	0.339	0.300	0.535
TSC22D1	2903.034	-0.270	0.161	0.094	0.188	2903.034	-0.392	0.161	0.015	0.306	2903.034	-0.122	0.187	0.515	0.719
KAT2B	263.441	-0.368	0.352	0.296	0.434	263.441	0.162	0.350	0.642	0.805	263.441	0.530	0.405	0.191	0.413
CDK5R1	158.314	-0.502	0.190	0.008	0.029	158.314	-0.078	0.184	0.672	0.823	158.314	0.424	0.219	0.052	0.188
ASAP2	1708.100	-0.899	0.212	0.000	0.000	1708.100	-0.087	0.211	0.681	0.827	1708.100	0.812	0.245	0.001	0.012
ALDH1A2	43.531	2.337	0.590	0.000	0.001	43.531	0.063	0.702	0.929	0.964	43.531	-2.274	0.723	0.002	0.019
FCGBP	11.920	0.011	0.748	0.988	0.993	11.920	2.568	0.629	0.000	0.073	11.920	2.557	0.781	0.001	0.014
STK19	1186.225	0.126	0.153	0.411	0.549	1186.225	0.188	0.152	0.219	0.498	1186.225	0.062	0.176	0.725	0.858
LDB1	1119.575	-0.197	0.328	0.548	0.671	1119.575	0.340	0.328	0.300	0.567	1119.575	0.537	0.379	0.157	0.368
APLN	211.366	1.316	0.384	0.001	0.004	211.366	0.282	0.386	0.466	0.690	211.366	-1.034	0.444	0.020	0.101
PER3	46.871	0.716	0.415	0.084	0.172	46.871	0.172	0.420	0.681	0.827	46.871	-0.544	0.481	0.258	0.492
PER2	118.685	-0.409	0.338	0.226	0.358	118.685	0.074	0.331	0.824	0.908	118.685	0.482	0.389	0.215	0.441
SYNJ1	253.557	-0.889	0.211	0.000	0.000	253.557	-0.129	0.205	0.531	0.731	253.557	0.761	0.242	0.002	0.019
ST3GAL5	48.334	1.975	0.398	0.000	0.000	48.334	0.024	0.432	0.956	0.978	48.334	-1.951	0.472	0.000	0.001
IER3	23436.468	-0.166	0.451	0.714	0.804	23436.468	-0.877	0.452	0.052	0.341	23436.468	-0.712	0.521	0.172	0.389
SYNJ2	1142.188	-0.788	0.196	0.000	0.001	1142.188	-0.584	0.195	0.003	0.297	1142.188	0.203	0.227	0.369	0.601
CDC123	1698.148	-0.542	0.285	0.057	0.128	1698.148	-0.640	0.285	0.025	0.316	1698.148	-0.098	0.329	0.767	0.881
ARHGEF7	542.424	-0.584	0.183	0.001	0.007	542.424	-0.171	0.181	0.343	0.600	542.424	0.412	0.211	0.050	0.183
RRP36	550.423	-0.491	0.369	0.183	0.307	550.423	-0.985	0.369	0.008	0.297	550.423	-0.494	0.427	0.247	0.478
VNN1	17.013	2.663	0.537	0.000	0.000	17.013	-0.350	0.733	0.633	0.800	17.013	-3.013	0.726	0.000	0.001
SPHK1	481.567	-0.027	0.551	0.961	0.977	481.567	-1.604	0.554	0.004	0.297	481.567	-1.578	0.639	0.014	0.080
SQSTM1	35762.946	1.373	0.179	0.000	0.000	35762.946	-0.211	0.179	0.239	0.516	35762.946	-1.584	0.207	0.000	0.000
SGPL1	925.089	-0.795	0.199	0.000	0.001	925.089	-0.213	0.198	0.282	0.551	925.089	0.583	0.229	0.011	0.070
FUBP1	1796.189	-0.350	0.141	0.013	0.042	1796.189	-0.320	0.141	0.023	0.316	1796.189	0.030	0.163	0.855	0.930
CDC16	695.369	-0.440	0.188	0.019	0.056	695.369	-0.320	0.188	0.088	0.380	695.369	0.120	0.218	0.581	0.764
ZPR1	1053.707	-0.978	0.098	0.000	0.000	1053.707	0.034	0.094	0.722	0.851	1053.707	1.011	0.112	0.000	0.000
NAE1	1114.627	-0.199	0.281	0.478	0.612	1114.627	-0.602	0.281	0.032	0.322	1114.627	-0.402	0.325	0.215	0.441
SLC5A6	625.618	-0.354	0.247	0.151	0.265	625.618	-0.386	0.247	0.118	0.409	625.618	-0.031	0.285	0.913	0.960
DDX18	2168.475	-0.756	0.264	0.004	0.017	2168.475	-0.633	0.264	0.016	0.306	2168.475	0.122	0.305	0.688	0.836
TAX1BP1	2072.815	-0.315	0.131	0.016	0.049	2072.815	-0.231	0.131	0.077	0.369	2072.815	0.085	0.151	0.576	0.761
MCM3AP	1327.225	-0.714	0.205	0.000	0.003	1327.225	-0.467	0.204	0.022	0.315	1327.225	0.247	0.237	0.298	0.533
KRIT1	526.838	-0.565	0.150	0.000	0.001	526.838	-0.104	0.148	0.482	0.700	526.838	0.461	0.173	0.008	0.055
EIF2B4	537.711	-0.017	0.174	0.924	0.952	537.711	-0.304	0.174	0.081	0.372	537.711	-0.287	0.201	0.154	0.364
EIF2B3	590.033	-0.523	0.288	0.069	0.147	590.033	-0.684	0.288	0.017	0.306	590.033	-0.161	0.333	0.629	0.798
EIF2B2	648.550	-0.488	0.164	0.003	0.013	648.550	-0.207	0.163	0.205	0.488	648.550	0.281	0.190	0.138	0.341
EIF2B5	1064.115	-0.349	0.171	0.041	0.100	1064.115	-0.214	0.171	0.209	0.491	1064.115	0.135	0.198	0.495	0.703
EIF2S2	3960.442	-0.121	0.243	0.619	0.730	3960.442	-0.307	0.243	0.206	0.488	3960.442	-0.187	0.281	0.506	0.712
CPNE3	1866.116	-0.440	0.259	0.089	0.180	1866.116	0.263	0.258	0.308	0.573	1866.116	0.703	0.299	0.019	0.097
BUD31	972.896	-0.017	0.274	0.951	0.970	972.896	-0.344	0.274	0.209	0.490	972.896	-0.327	0.316	0.301	0.537
MTMR3	490.419	-0.380	0.158	0.016	0.049	490.419	-0.200	0.157	0.202	0.485	490.419	0.180	0.183	0.325	0.559
MTMR2	1585.860	-0.981	0.220	0.000	0.000	1585.860	-0.348	0.219	0.112	0.405	1585.860	0.633	0.254	0.013	0.076
PRPF4B	758.281	-0.547	0.184	0.003	0.013	758.281	-0.158	0.183	0.387	0.633	758.281	0.389	0.212	0.066	0.218
CCNA2	1294.267	-0.697	0.233	0.003	0.013	1294.267	-0.329	0.233	0.158	0.450	1294.267	0.368	0.269	0.171	0.388
CPNE1	1683.848	-0.161	0.246	0.513	0.641	1683.848	0.457	0.245	0.063	0.350	1683.848	0.618	0.284	0.029	0.130
AP1S2	686.550	-0.252	0.164	0.125	0.230	686.550	0.138	0.163	0.396	0.640	686.550	0.390	0.189	0.039	0.156

AP1G2	310.698	0.697	0.270	0.010	0.034	310.698	0.573	0.270	0.034	0.322	310.698	-0.124	0.311	0.689	0.837
AP1M1	1202.774	0.282	0.147	0.054	0.123	1202.774	-0.141	0.147	0.339	0.597	1202.774	-0.423	0.170	0.013	0.076
CCNB1	4190.177	-0.618	0.186	0.001	0.005	4190.177	-0.139	0.186	0.455	0.682	4190.177	0.479	0.215	0.026	0.120
SGCE	810.303	-0.003	0.284	0.993	0.996	810.303	0.429	0.284	0.130	0.422	810.303	0.432	0.328	0.188	0.408
TRIM4	697.108	-0.128	0.222	0.564	0.685	697.108	0.310	0.221	0.160	0.451	697.108	0.438	0.255	0.086	0.256
CACNA1G	27.226	1.271	0.727	0.080	0.166	27.226	0.841	0.732	0.250	0.524	27.226	-0.430	0.830	0.605	0.782
TIMELESS	1127.803	-0.468	0.199	0.019	0.055	1127.803	-0.348	0.199	0.080	0.371	1127.803	0.120	0.230	0.603	0.781
BCL10	435.023	-0.212	0.145	0.144	0.256	435.023	-0.203	0.144	0.158	0.451	435.023	0.009	0.168	0.959	0.981
HERC3	585.316	-0.315	0.261	0.227	0.359	585.316	0.151	0.259	0.560	0.749	585.316	0.466	0.301	0.121	0.313
CCNC	1480.956	-0.490	0.135	0.000	0.002	1480.956	-0.157	0.134	0.242	0.518	1480.956	0.333	0.156	0.032	0.138
HERC2	2398.096	-0.406	0.206	0.049	0.113	2398.096	-0.448	0.206	0.029	0.322	2398.096	-0.043	0.238	0.858	0.932
HERC1	764.705	-0.617	0.207	0.003	0.013	764.705	-0.200	0.206	0.333	0.592	764.705	0.418	0.239	0.081	0.246
BSN	12.274	0.331	0.698	0.635	0.743	12.274	0.831	0.682	0.224	0.502	12.274	0.499	0.793	0.529	0.729
FOXH1	52.158	1.942	0.399	0.000	0.000	52.158	0.308	0.417	0.461	0.687	52.158	-1.635	0.465	0.000	0.007
MBD4	547.338	-0.595	0.241	0.013	0.042	547.338	-0.347	0.240	0.148	0.440	547.338	0.248	0.278	0.372	0.604
MBD2	1752.269	0.445	0.216	0.040	0.097	1752.269	-0.224	0.217	0.302	0.568	1752.269	-0.669	0.250	0.008	0.054
RTL8C	1475.735	1.147	0.208	0.000	0.000	1475.735	0.303	0.209	0.146	0.438	1475.735	-0.843	0.241	0.000	0.007
RAB29	1069.758	-0.426	0.184	0.021	0.059	1069.758	-0.081	0.183	0.658	0.813	1069.758	0.345	0.212	0.105	0.288
SKAP2	843.788	-0.097	0.179	0.587	0.703	843.788	0.105	0.178	0.557	0.747	843.788	0.202	0.207	0.328	0.562
WASF1	1416.489	-0.330	0.163	0.043	0.104	1416.489	0.053	0.163	0.744	0.863	1416.489	0.383	0.188	0.042	0.164
BAIAP3	290.087	1.044	0.283	0.000	0.002	290.087	0.665	0.284	0.019	0.306	290.087	-0.378	0.326	0.245	0.476
FUBP3	1489.365	-0.597	0.202	0.003	0.014	1489.365	-0.381	0.202	0.059	0.349	1489.365	0.216	0.234	0.356	0.590
CCND2	47.844	-0.112	0.406	0.782	0.856	47.844	-0.490	0.409	0.231	0.508	47.844	-0.378	0.474	0.426	0.649
TOP3B	258.099	0.245	0.224	0.274	0.411	258.099	-0.213	0.225	0.343	0.600	258.099	-0.458	0.259	0.078	0.241
KYNU	291.267	1.586	0.187	0.000	0.000	291.267	-0.060	0.196	0.759	0.872	291.267	-1.646	0.220	0.000	0.000
AP3D1	3178.281	-0.215	0.173	0.214	0.344	3178.281	-0.322	0.173	0.063	0.350	3178.281	-0.107	0.200	0.594	0.773
BTRC	276.641	-0.122	0.228	0.592	0.707	276.641	0.112	0.226	0.619	0.790	276.641	0.234	0.263	0.372	0.604
CCND3	2095.325	0.435	0.128	0.001	0.004	2095.325	0.062	0.129	0.632	0.799	2095.325	-0.373	0.148	0.012	0.073
H2AC11	59.545	-0.507	0.565	0.369	0.510	59.545	0.389	0.557	0.485	0.702	59.545	0.896	0.649	0.168	0.382
H2BC11	18.086	-0.905	0.537	0.092	0.184	18.086	0.581	0.469	0.215	0.494	18.086	1.487	0.596	0.013	0.076
H1-10	6613.944	0.552	0.245	0.024	0.067	6613.944	0.158	0.245	0.519	0.724	6613.944	-0.394	0.283	0.165	0.379
MGAM	14.486	-0.647	0.727	0.374	0.514	14.486	-1.853	0.762	0.015	0.306	14.486	-1.205	0.880	0.171	0.387
P4HA2	3088.846	0.610	0.398	0.125	0.231	3088.846	0.592	0.398	0.137	0.428	3088.846	-0.018	0.460	0.968	0.985
USP13	435.593	-0.337	0.200	0.092	0.184	435.593	-0.233	0.199	0.243	0.518	435.593	0.104	0.231	0.653	0.813
WASL	839.717	-0.588	0.171	0.001	0.004	839.717	-0.037	0.170	0.829	0.911	839.717	0.551	0.198	0.005	0.043
SERPINB11	15.461	3.234	0.683	0.000	0.000	15.461	-1.841	1.202	0.126	0.418	15.461	-5.075	1.207	0.000	0.001
HPS4	660.004	-0.130	0.114	0.255	0.389	660.004	0.184	0.113	0.103	0.395	660.004	0.314	0.132	0.017	0.092
LMLN	223.563	0.026	0.316	0.934	0.959	223.563	0.366	0.314	0.245	0.520	223.563	0.340	0.364	0.351	0.584
NAV3	110.660	-2.365	0.562	0.000	0.000	110.660	-1.403	0.552	0.011	0.302	110.660	0.962	0.650	0.138	0.341
NAV1	1974.589	0.428	0.131	0.001	0.006	1974.589	-0.026	0.132	0.845	0.920	1974.589	-0.454	0.152	0.003	0.027
NAV2	1943.939	-0.071	0.158	0.651	0.756	1943.939	-0.032	0.158	0.838	0.916	1943.939	0.039	0.182	0.830	0.917
CCNE1	73.679	-0.281	0.346	0.417	0.554	73.679	-0.942	0.354	0.008	0.297	73.679	-0.661	0.410	0.107	0.291
PPP1R3F	46.092	1.066	0.371	0.004	0.017	46.092	0.801	0.373	0.032	0.322	46.092	-0.265	0.421	0.529	0.729
ULK4P3	7.332	0.789	0.601	0.189	0.314	7.332	0.592	0.601	0.325	0.586	7.332	-0.197	0.683	0.773	0.885
ULK4P1	16.277	0.067	0.402	0.868	0.914	16.277	0.265	0.389	0.497	0.711	16.277	0.198	0.459	0.667	0.823
ARHGAP11B	354.667	-1.109	0.271	0.000	0.000	354.667	-0.051	0.267	0.849	0.922	354.667	1.058	0.312	0.001	0.010
ABCC10	291.463	0.211	0.217	0.331	0.472	291.463	-0.433	0.219	0.048	0.337	291.463	-0.644	0.253	0.011	0.069
FCHSD1	234.034	0.622	0.159	0.000	0.001	234.034	0.187	0.161	0.246	0.521	234.034	-0.435	0.185	0.018	0.096
ATG16L2	138.167	0.452	0.325	0.164	0.283	138.167	0.356	0.324	0.273	0.544	138.167	-0.097	0.375	0.796	0.898
PLOD3	2277.682	0.149	0.195	0.444	0.580	2277.682	-0.168	0.195	0.390	0.635	2277.682	-0.317	0.225	0.160	0.373
MVB12B	15.822	0.641	0.446	0.151	0.265	15.822	0.268	0.452	0.553	0.745	15.822	-0.373	0.516	0.469	0.684
RPS6KA4	1274.189	0.283	0.413	0.493	0.625	1274.189	-0.998	0.413	0.016	0.306	1274.189	-1.280	0.477	0.007	0.053
STBD1	108.450	0.678	0.507	0.182	0.305	108.450	0.466	0.507	0.359	0.611	108.450	-0.212	0.585	0.717	0.853
TRIM15	70.669	-0.419	0.464	0.366	0.507	70.669	0.319	0.457	0.484	0.702	70.669	0.739	0.534	0.166	0.381
SLC25A21	20.589	-0.390	0.482	0.419	0.556	20.589	0.394	0.460	0.392	0.637	20.589	0.784	0.548	0.153	0.362
LHX4	14.155	1.571	0.425	0.000	0.002	14.155	1.239	0.429	0.004	0.297	14.155	-0.331	0.464	0.475	0.689
SLAMF9	23.245	1.354	0.912	0.138	0.248	23.245	1.187	0.912	0.193	0.478	23.245	-0.167	1.025	0.870	0.938
ZNF628	240.967	-0.207	0.184	0.260	0.396	240.967	0.058	0.181	0.748	0.866	240.967	0.265	0.212	0.211	0.436
TRPA1	82.924	0.224	0.371	0.546	0.670	82.924	0.614	0.367	0.095	0.389	82.924	0.390	0.426	0.360	0.593
KBTBD6	295.674	-0.530	0.231	0.022	0.062	295.674	-0.241	0.229	0.294	0.561	295.674	0.289	0.267	0.279	0.514
WDR34	1128.363	-0.030	0.292	0.918	0.948	1128.363	-0.435	0.292	0.137	0.428	1128.363	-0.405	0.338	0.230	0.459
TMEM116	40.281	-0.089	0.363	0.806	0.872	40.281	0.440	0.351	0.210	0.491	40.281	0.530	0.414	0.201	0.424
CCNF	642.149	-0.439	0.155	0.005	0.018	642.149	0.061	0.153	0.692	0.834	642.149	0.500	0.179	0.005	0.042
SELENBP1	271.451	-0.049	0.502	0.923	0.951	271.451	0.500	0.500	0.317	0.579	271.451	0.549	0.579	0.343	0.577
UBE3B	616.074	0.110	0.157	0.483	0.616	616.074	0.120	0.157	0.443	0.674	616.074	0.010	0.181	0.957	0.980
ATP6V0E1	2667.580	0.765	0.136	0.000	0.000	2667.580	-0.141	0.136	0.299	0.566	2667.580	-0.906	0.157	0.000	0.000
BMERB1	72.739	-0.287	0.433	0.508	0.638	72.739	0.359	0.427	0.401	0.644	72.739	0.646	0.498	0.195	0.417

PAPLN	9.679	0.795	0.563	0.158	0.276	9.679	0.194	0.583	0.739	0.861	9.679	-0.601	0.656	0.360	0.593
LIMD1	1053.704	-0.399	0.167	0.017	0.051	1053.704	0.026	0.167	0.876	0.937	1053.704	0.425	0.193	0.028	0.125
RHOT2	1373.883	0.150	0.216	0.487	0.620	1373.883	-0.031	0.216	0.887	0.943	1373.883	-0.181	0.249	0.469	0.684
GLB1L2	32.109	0.404	0.353	0.254	0.388	32.109	-0.319	0.367	0.383	0.629	32.109	-0.723	0.419	0.084	0.252
KLC4	422.671	0.371	0.363	0.307	0.446	422.671	0.697	0.362	0.054	0.342	422.671	0.326	0.418	0.435	0.657
SAPCD2	1409.006	-0.465	0.277	0.093	0.186	1409.006	-0.560	0.277	0.043	0.331	1409.006	-0.095	0.320	0.767	0.881
NOL3	779.365	1.072	0.362	0.003	0.014	779.365	0.607	0.363	0.094	0.388	779.365	-0.465	0.418	0.266	0.501
RSPRY1	650.295	-0.469	0.135	0.001	0.003	650.295	-0.032	0.133	0.809	0.900	650.295	0.436	0.156	0.005	0.042
DPH6	114.183	-0.347	0.211	0.099	0.195	114.183	-0.145	0.207	0.483	0.701	114.183	0.202	0.244	0.407	0.633
NAT1	36.724	0.851	0.386	0.027	0.073	36.724	-0.135	0.401	0.737	0.859	36.724	-0.985	0.454	0.030	0.132
ACVR1	468.225	-0.524	0.174	0.003	0.012	468.225	-0.376	0.173	0.029	0.322	468.225	0.149	0.201	0.460	0.677
CCNG1	3449.120	0.092	0.279	0.743	0.827	3449.120	0.314	0.279	0.261	0.534	3449.120	0.222	0.323	0.491	0.701
MIDN	1295.113	0.317	0.140	0.023	0.065	1295.113	0.152	0.140	0.276	0.547	1295.113	-0.165	0.161	0.307	0.542
SYT8	6.886	0.373	0.733	0.611	0.723	6.886	0.497	0.721	0.490	0.706	6.886	0.124	0.835	0.882	0.943
FLJ20021	71.661	0.528	0.302	0.081	0.167	71.661	0.639	0.300	0.033	0.322	71.661	0.111	0.346	0.748	0.871
UBE3D	122.378	-0.342	0.192	0.075	0.157	122.378	0.047	0.185	0.799	0.896	122.378	0.389	0.221	0.078	0.240
ZNF30	48.231	-0.422	0.269	0.117	0.220	48.231	-0.725	0.272	0.008	0.297	48.231	-0.303	0.320	0.343	0.577
CCNG2	1017.171	0.630	0.734	0.391	0.530	1017.171	0.815	0.734	0.267	0.538	1017.171	0.185	0.847	0.827	0.915
PHLDB2	1573.867	-1.074	0.341	0.002	0.008	1573.867	-0.704	0.341	0.039	0.327	1573.867	0.371	0.394	0.347	0.581
VWA5B2	21.372	1.325	0.501	0.008	0.029	21.372	0.896	0.510	0.079	0.370	21.372	-0.429	0.560	0.443	0.663
TMEM250	1401.595	0.212	0.139	0.126	0.232	1401.595	-0.010	0.139	0.943	0.971	1401.595	-0.222	0.160	0.166	0.381
TSR2	723.947	0.539	0.204	0.008	0.030	723.947	-0.283	0.205	0.169	0.458	723.947	-0.821	0.237	0.001	0.008
TAF1C	515.645	0.413	0.245	0.091	0.183	515.645	0.183	0.245	0.454	0.681	515.645	-0.230	0.283	0.415	0.641
BTBD6	323.879	0.211	0.214	0.324	0.464	323.879	-0.195	0.215	0.364	0.616	323.879	-0.406	0.248	0.102	0.284
TSPAN18	49.564	0.304	0.322	0.346	0.486	49.564	0.235	0.321	0.463	0.688	49.564	-0.068	0.371	0.854	0.930
TAF1B	292.391	-0.525	0.150	0.000	0.003	292.391	-0.330	0.147	0.025	0.316	292.391	0.194	0.174	0.263	0.497
EFCAB11	84.913	0.040	0.231	0.864	0.912	84.913	-0.244	0.233	0.294	0.561	84.913	-0.284	0.270	0.294	0.529
TAF1A	138.714	-1.316	0.447	0.003	0.014	138.714	-0.953	0.444	0.032	0.322	138.714	0.363	0.517	0.483	0.695
SLC25A14	121.674	0.017	0.158	0.915	0.946	121.674	-0.013	0.156	0.933	0.966	121.674	-0.030	0.183	0.870	0.937
EMILIN3	25.701	2.065	0.529	0.000	0.001	25.701	0.790	0.543	0.146	0.438	25.701	-1.275	0.607	0.036	0.148
MPZL1	2915.560	0.108	0.137	0.431	0.567	2915.560	0.166	0.137	0.224	0.503	2915.560	0.058	0.158	0.712	0.850
SYS1	362.666	-0.037	0.157	0.816	0.879	362.666	0.135	0.155	0.382	0.629	362.666	0.172	0.181	0.342	0.576
CCNH	633.963	-0.539	0.185	0.004	0.015	633.963	-0.338	0.184	0.067	0.354	633.963	0.201	0.214	0.348	0.582
MAP3K14	290.197	-0.228	0.243	0.348	0.488	290.197	-0.179	0.242	0.459	0.685	290.197	0.049	0.280	0.862	0.934
SNX21	137.171	0.853	0.287	0.003	0.013	137.171	0.354	0.289	0.221	0.500	137.171	-0.499	0.331	0.132	0.329
ZSWIM1	102.974	0.361	0.194	0.063	0.138	102.974	0.009	0.196	0.964	0.981	102.974	-0.352	0.226	0.119	0.310
SOCS3	499.404	1.741	0.335	0.000	0.000	499.404	-0.004	0.337	0.989	0.994	499.404	-1.745	0.388	0.000	0.000
CLIC3	34.516	2.392	0.710	0.001	0.005	34.516	0.748	0.724	0.302	0.568	34.516	-1.644	0.818	0.045	0.170
UCN2	32.997	1.718	0.501	0.001	0.004	32.997	0.993	0.508	0.051	0.338	32.997	-0.725	0.571	0.204	0.428
KIAA2013	2077.756	0.456	0.167	0.007	0.024	2077.756	0.257	0.168	0.124	0.416	2077.756	-0.198	0.193	0.306	0.541
ZNF551	119.208	-0.944	0.266	0.000	0.003	119.208	-0.213	0.257	0.408	0.649	119.208	0.731	0.306	0.017	0.092
BRSK2	9.310	1.400	0.714	0.050	0.115	9.310	-0.393	0.785	0.617	0.789	9.310	-1.793	0.865	0.038	0.154
RNF8	547.456	0.023	0.125	0.855	0.905	547.456	-0.030	0.125	0.810	0.900	547.456	-0.053	0.145	0.714	0.852
HIP1R	1458.191	0.120	0.166	0.469	0.603	1458.191	-0.055	0.166	0.741	0.862	1458.191	-0.175	0.192	0.361	0.594
OTULIN	879.348	-0.489	0.172	0.004	0.018	879.348	-0.008	0.171	0.962	0.980	879.348	0.481	0.198	0.015	0.087
OLMALINC	218.779	0.332	0.462	0.472	0.606	218.779	0.747	0.460	0.105	0.398	218.779	0.414	0.532	0.436	0.657
RHBDL1	20.822	3.126	0.752	0.000	0.000	20.822	2.148	0.760	0.005	0.297	20.822	-0.978	0.839	0.244	0.475
KLHL13	110.887	-0.338	0.314	0.282	0.420	110.887	-0.004	0.311	0.989	0.994	110.887	0.334	0.363	0.357	0.590
BAZ1B	2359.347	-0.958	0.246	0.000	0.001	2359.347	-0.561	0.246	0.023	0.315	2359.347	0.397	0.285	0.163	0.377
TP53I13	510.139	0.908	0.312	0.004	0.015	510.139	0.520	0.313	0.097	0.391	510.139	-0.388	0.361	0.281	0.516
ZNF616	188.205	-0.102	0.247	0.681	0.779	188.205	0.243	0.244	0.320	0.581	188.205	0.344	0.284	0.226	0.454
ZNF766	340.106	-0.271	0.138	0.050	0.116	340.106	0.076	0.136	0.574	0.759	340.106	0.348	0.159	0.029	0.129
CCDC97	282.539	-0.432	0.214	0.044	0.104	282.539	-0.162	0.212	0.447	0.677	282.539	0.270	0.247	0.274	0.510
THAP3	751.686	0.303	0.209	0.147	0.261	751.686	-0.199	0.209	0.341	0.599	751.686	-0.502	0.242	0.038	0.153
ZNF468	125.334	-0.338	0.204	0.097	0.192	125.334	-0.184	0.200	0.358	0.611	125.334	0.154	0.236	0.514	0.718
ZNF160	313.379	0.125	0.343	0.714	0.805	313.379	0.382	0.342	0.263	0.535	313.379	0.257	0.395	0.515	0.720
CTU1	451.227	0.294	0.275	0.285	0.423	451.227	-0.040	0.276	0.884	0.942	451.227	-0.334	0.318	0.294	0.529
C5orf30	191.423	-0.420	0.145	0.004	0.016	191.423	-0.292	0.141	0.039	0.327	191.423	0.128	0.169	0.449	0.668
FAM110B	23.175	0.595	0.403	0.140	0.251	23.175	-0.277	0.430	0.520	0.724	23.175	-0.872	0.484	0.072	0.229
SEMA5A	204.645	-0.509	0.427	0.233	0.365	204.645	0.126	0.425	0.767	0.877	204.645	0.635	0.492	0.197	0.420
SAMD1	1009.696	0.911	0.189	0.000	0.000	1009.696	0.089	0.190	0.639	0.803	1009.696	-0.822	0.219	0.000	0.004
DCAF15	379.836	0.176	0.244	0.470	0.604	379.836	-0.058	0.244	0.813	0.902	379.836	-0.234	0.282	0.407	0.633
TICRR	302.108	-0.123	0.133	0.352	0.492	302.108	0.023	0.131	0.863	0.930	302.108	0.146	0.153	0.340	0.574
UBA3	960.242	-0.228	0.152	0.133	0.242	960.242	-0.319	0.151	0.035	0.324	960.242	-0.091	0.176	0.604	0.781
MED30	176.536	0.086	0.185	0.642	0.748	176.536	-0.127	0.185	0.491	0.707	176.536	-0.213	0.214	0.320	0.555
CCNT1	766.765	-0.792	0.244	0.001	0.007	766.765	-0.078	0.243	0.748	0.866	766.765	0.714	0.282	0.011	0.071
UBE2M	2389.775	-0.126	0.263	0.631	0.740	2389.775	-0.248	0.263	0.346	0.602	2389.775	-0.121	0.303	0.690	0.837

TMEM41A	462.908	0.058	0.162	0.722	0.811	462.908	-0.205	0.163	0.209	0.490	462.908	-0.262	0.189	0.164	0.379
IFT20	422.952	0.762	0.172	0.000	0.000	422.952	0.233	0.173	0.180	0.468	422.952	-0.530	0.199	0.008	0.055
MCFD2	1938.364	-0.763	0.240	0.001	0.008	1938.364	0.060	0.239	0.803	0.897	1938.364	0.823	0.276	0.003	0.028
CCDC32	281.505	0.435	0.177	0.014	0.044	281.505	0.162	0.178	0.362	0.614	281.505	-0.274	0.205	0.181	0.400
KNSTRN	934.924	-0.569	0.205	0.006	0.021	934.924	-0.263	0.204	0.198	0.481	934.924	0.306	0.237	0.197	0.420
ATP6V1E2	113.800	-0.141	0.244	0.563	0.683	113.800	0.199	0.240	0.407	0.648	113.800	0.340	0.281	0.226	0.454
BMF	155.368	0.826	0.873	0.344	0.484	155.368	0.658	0.873	0.451	0.679	155.368	-0.168	1.007	0.868	0.936
SPAG9	2860.606	-0.361	0.249	0.146	0.260	2860.606	-0.214	0.248	0.390	0.635	2860.606	0.147	0.287	0.607	0.783
BTAF1	638.307	-0.850	0.316	0.007	0.026	638.307	-0.238	0.315	0.450	0.678	638.307	0.612	0.365	0.094	0.269
ZNF622	1609.662	-0.340	0.210	0.105	0.202	1609.662	-0.081	0.209	0.697	0.837	1609.662	0.259	0.242	0.285	0.520
RPL14	36055.084	0.843	0.296	0.004	0.018	36055.084	0.391	0.296	0.187	0.474	36055.084	-0.452	0.342	0.187	0.407
ERI1	394.927	-0.839	0.379	0.027	0.072	394.927	-0.826	0.378	0.029	0.322	394.927	0.013	0.438	0.976	0.987
GADD45GIP1	2545.617	0.672	0.271	0.013	0.042	2545.617	-0.070	0.272	0.797	0.895	2545.617	-0.742	0.313	0.018	0.095
TMEM263	935.072	-0.232	0.268	0.387	0.526	935.072	0.126	0.268	0.637	0.802	935.072	0.358	0.309	0.247	0.478
AIP	1317.626	0.494	0.218	0.024	0.065	1317.626	0.298	0.218	0.172	0.461	1317.626	-0.196	0.252	0.436	0.657
CCNT2	366.346	-0.510	0.218	0.019	0.056	366.346	-0.363	0.217	0.094	0.388	366.346	0.147	0.253	0.561	0.752
PSTPIP2	202.966	0.444	0.145	0.002	0.011	202.966	0.406	0.144	0.005	0.297	202.966	-0.038	0.166	0.819	0.911
LRRC46	7.746	0.799	0.736	0.278	0.415	7.746	0.889	0.726	0.221	0.500	7.746	0.090	0.829	0.913	0.960
SCRN2	344.790	1.052	0.292	0.000	0.002	344.790	0.567	0.293	0.053	0.342	344.790	-0.485	0.337	0.150	0.359
GPRC5A	419.430	1.337	0.271	0.000	0.000	419.430	0.597	0.274	0.029	0.322	419.430	-0.740	0.312	0.018	0.094
YIF1B	2144.095	0.630	0.233	0.007	0.026	2144.095	0.380	0.233	0.104	0.396	2144.095	-0.250	0.269	0.353	0.586
MLIP	8.270	0.673	1.223	0.582	0.699	8.270	-0.067	1.234	0.957	0.978	8.270	-0.740	1.418	0.602	0.780
SHF	11.738	0.552	0.893	0.537	0.662	11.738	0.704	0.886	0.427	0.663	11.738	0.153	1.023	0.881	0.943
STPG1	371.836	-0.250	0.180	0.165	0.284	371.836	-0.160	0.179	0.371	0.620	371.836	0.090	0.208	0.665	0.822
MAP7	72.269	0.656	0.280	0.019	0.056	72.269	0.529	0.280	0.059	0.349	72.269	-0.128	0.321	0.691	0.837
NFS1	414.146	-0.005	0.122	0.970	0.982	414.146	-0.077	0.121	0.526	0.728	414.146	-0.072	0.141	0.608	0.783
PRC1	1620.926	-0.551	0.239	0.021	0.060	1620.926	-0.267	0.239	0.263	0.536	1620.926	0.284	0.276	0.303	0.538
MCU	652.000	-0.133	0.114	0.244	0.378	652.000	-0.067	0.114	0.555	0.746	652.000	0.066	0.132	0.617	0.790
CCDC74A	12.008	0.362	0.513	0.480	0.614	12.008	0.209	0.512	0.683	0.829	12.008	-0.153	0.591	0.795	0.897
SLC7A7	21.692	1.395	0.564	0.013	0.042	21.692	0.723	0.572	0.206	0.488	21.692	-0.672	0.647	0.299	0.534
SLC7A6	810.219	-0.251	0.268	0.348	0.488	810.219	-0.559	0.268	0.037	0.325	810.219	-0.308	0.310	0.320	0.555
ZNF799	39.245	-0.014	0.353	0.967	0.980	39.245	-0.357	0.356	0.317	0.579	39.245	-0.342	0.414	0.408	0.635
TIMM29	198.215	-0.164	0.219	0.453	0.588	198.215	-0.289	0.219	0.187	0.474	198.215	-0.124	0.255	0.626	0.795
ZNF700	82.643	0.444	0.216	0.040	0.097	82.643	0.193	0.217	0.374	0.622	82.643	-0.251	0.250	0.315	0.551
PAPSS2	54.756	0.429	0.224	0.055	0.124	54.756	0.313	0.222	0.159	0.451	54.756	-0.116	0.256	0.652	0.813
PAPSS1	959.460	-0.061	0.163	0.710	0.802	959.460	0.071	0.162	0.663	0.817	959.460	0.131	0.188	0.485	0.697
LYRM7	632.378	-0.872	0.122	0.000	0.000	632.378	-0.282	0.119	0.017	0.306	632.378	0.590	0.140	0.000	0.001
STARD13	305.807	-0.950	0.231	0.000	0.000	305.807	0.091	0.226	0.688	0.831	305.807	1.041	0.265	0.000	0.002
PIAS2	522.604	0.122	0.191	0.524	0.651	522.604	0.108	0.190	0.569	0.756	522.604	-0.013	0.220	0.951	0.978
N4BP2L1	44.267	1.112	0.533	0.037	0.092	44.267	0.902	0.533	0.091	0.385	44.267	-0.210	0.611	0.731	0.861
ZFAND2A	383.369	-0.327	0.235	0.165	0.284	383.369	-0.274	0.234	0.243	0.518	383.369	0.053	0.273	0.846	0.925
COX19	368.260	-0.066	0.154	0.671	0.771	368.260	-0.045	0.153	0.771	0.878	368.260	0.021	0.178	0.906	0.957
MAP3K6	491.251	0.889	0.134	0.000	0.000	491.251	0.148	0.136	0.274	0.544	491.251	-0.741	0.155	0.000	0.000
SYT7	516.514	0.300	0.997	0.763	0.843	516.514	0.956	0.996	0.337	0.595	516.514	0.656	1.151	0.568	0.758
PPP1R3E	84.493	0.681	0.360	0.059	0.130	84.493	0.224	0.362	0.537	0.735	84.493	-0.457	0.416	0.272	0.507
LRSAM1	369.664	0.643	0.152	0.000	0.000	369.664	0.197	0.153	0.197	0.481	369.664	-0.446	0.175	0.011	0.070
ANGPTL1	24.111	0.617	0.390	0.114	0.215	24.111	-0.115	0.404	0.777	0.882	24.111	-0.731	0.459	0.111	0.298
CLDN12	542.676	-0.202	0.181	0.265	0.401	542.676	-0.114	0.180	0.526	0.729	542.676	0.088	0.209	0.675	0.828
CCDC126	60.580	0.352	0.261	0.177	0.299	60.580	-0.400	0.271	0.141	0.432	60.580	-0.752	0.310	0.015	0.087
ASH2L	885.489	-0.773	0.184	0.000	0.000	885.489	-0.207	0.183	0.259	0.531	885.489	0.566	0.213	0.008	0.056
SEC11C	314.413	0.137	0.200	0.493	0.625	314.413	-0.128	0.200	0.522	0.726	314.413	-0.265	0.231	0.253	0.485
FAM104B	224.691	0.365	0.216	0.091	0.183	224.691	0.120	0.216	0.579	0.764	224.691	-0.245	0.250	0.327	0.562
CLDN2	47.935	0.377	0.283	0.183	0.307	47.935	0.263	0.282	0.351	0.605	47.935	-0.114	0.325	0.726	0.858
CLDN1	1130.333	-1.831	0.657	0.005	0.021	1130.333	-1.703	0.657	0.010	0.298	1130.333	0.128	0.759	0.866	0.936
TENM3-AS1	22.531	-1.167	0.422	0.006	0.022	22.531	-0.538	0.393	0.171	0.460	22.531	0.629	0.487	0.197	0.420
PYGO2	573.013	0.161	0.169	0.338	0.478	573.013	-0.134	0.169	0.429	0.664	573.013	-0.295	0.195	0.131	0.328
LDB2	25.828	-0.217	0.675	0.748	0.831	25.828	0.569	0.663	0.391	0.636	25.828	0.786	0.774	0.310	0.545
CEP95	363.700	-0.114	0.178	0.524	0.651	363.700	0.071	0.177	0.690	0.832	363.700	0.184	0.206	0.370	0.602
CCT6A	5523.813	-0.684	0.264	0.010	0.033	5523.813	-0.706	0.264	0.008	0.297	5523.813	-0.022	0.305	0.943	0.974
ANGEL2	402.721	-0.319	0.194	0.099	0.194	402.721	-0.362	0.193	0.061	0.350	402.721	-0.042	0.224	0.851	0.928
PIP4P1	392.783	0.564	0.154	0.000	0.002	392.783	0.041	0.155	0.791	0.891	392.783	-0.523	0.178	0.003	0.031
PRMT9	105.320	-0.553	0.246	0.025	0.068	105.320	-0.197	0.240	0.414	0.652	105.320	0.356	0.284	0.210	0.436
CCDC189	38.240	0.504	0.282	0.074	0.156	38.240	-0.732	0.312	0.019	0.306	38.240	-1.236	0.351	0.000	0.007
TCEAL8	323.184	0.745	0.307	0.015	0.047	323.184	0.147	0.308	0.634	0.800	323.184	-0.598	0.355	0.092	0.267
ZNF598	2237.082	-0.865	0.279	0.002	0.010	2237.082	-0.378	0.279	0.175	0.464	2237.082	0.488	0.322	0.130	0.327
SPOCD1	22.474	-0.169	0.505	0.739	0.823	22.474	-1.136	0.527	0.031	0.322	22.474	-0.968	0.608	0.112	0.298
EIF1AY	13.653	0.037	0.490	0.940	0.963	13.653	-0.234	0.494	0.636	0.801	13.653	-0.270	0.575	0.638	0.803

JPT2	2691.926	-0.400	0.123	0.001	0.006	2691.926	-0.159	0.123	0.195	0.479	2691.926	0.241	0.142	0.090	0.262
SPSB3	515.728	0.764	0.233	0.001	0.006	515.728	0.564	0.233	0.016	0.306	515.728	-0.199	0.269	0.459	0.676
IL33	14.375	1.459	0.597	0.014	0.045	14.375	1.017	0.605	0.093	0.387	14.375	-0.442	0.669	0.509	0.715
TMSB4Y	9.821	0.335	0.482	0.488	0.621	9.821	-0.263	0.507	0.604	0.781	9.821	-0.598	0.579	0.302	0.537
DMAC1	348.792	-0.250	0.169	0.138	0.248	348.792	-0.121	0.167	0.471	0.693	348.792	0.130	0.195	0.506	0.712
ZNF697	752.265	-1.395	0.412	0.001	0.004	752.265	-0.908	0.411	0.027	0.319	752.265	0.488	0.475	0.305	0.540
PKMYT1	852.116	-0.246	0.414	0.552	0.674	852.116	-0.833	0.414	0.044	0.333	852.116	-0.587	0.479	0.220	0.447
PIGQ	444.438	0.583	0.255	0.022	0.062	444.438	-0.260	0.257	0.311	0.575	444.438	-0.843	0.296	0.004	0.037
SART1	1686.542	0.135	0.264	0.609	0.721	1686.542	-0.323	0.264	0.222	0.501	1686.542	-0.458	0.305	0.134	0.333
DNAJA3	3379.942	-0.808	0.238	0.001	0.004	3379.942	-0.362	0.238	0.128	0.420	3379.942	0.446	0.275	0.105	0.289
TRIM41	583.573	-0.070	0.170	0.680	0.777	583.573	0.008	0.169	0.962	0.980	583.573	0.078	0.196	0.690	0.837
UNC119	438.937	1.053	0.254	0.000	0.000	438.937	0.386	0.256	0.131	0.423	438.937	-0.667	0.294	0.023	0.111
TBX19	30.213	0.753	0.688	0.273	0.410	30.213	0.743	0.685	0.279	0.549	30.213	-0.011	0.788	0.989	0.994
ADCK2	578.720	-0.108	0.165	0.513	0.642	578.720	0.274	0.164	0.093	0.388	578.720	0.382	0.190	0.044	0.169
DHX57	273.076	-0.104	0.163	0.524	0.651	273.076	-0.322	0.163	0.049	0.337	273.076	-0.218	0.190	0.250	0.482
TBX18	641.037	-1.311	0.179	0.000	0.000	641.037	-0.050	0.175	0.773	0.879	641.037	1.261	0.206	0.000	0.000
USP14	1245.025	-0.384	0.221	0.082	0.169	1245.025	-0.570	0.221	0.010	0.298	1245.025	-0.186	0.256	0.467	0.682
ZNF251	380.292	0.324	0.393	0.409	0.547	380.292	0.407	0.392	0.299	0.566	380.292	0.083	0.453	0.855	0.930
USP2	55.011	1.063	0.229	0.000	0.000	55.011	-0.106	0.245	0.665	0.818	55.011	-1.169	0.272	0.000	0.001
KIFC2	315.218	1.229	0.263	0.000	0.000	315.218	0.553	0.264	0.036	0.324	315.218	-0.676	0.303	0.026	0.119
CREB3L1	375.971	1.438	0.286	0.000	0.000	375.971	0.433	0.288	0.133	0.424	375.971	-1.005	0.331	0.002	0.024
ACVR1B	213.252	0.039	0.252	0.877	0.920	213.252	0.481	0.249	0.053	0.342	213.252	0.442	0.290	0.127	0.322
USP10	2763.845	-0.898	0.194	0.000	0.000	2763.845	-0.317	0.193	0.100	0.394	2763.845	0.581	0.224	0.009	0.063
USP8	1117.940	-0.523	0.128	0.000	0.000	1117.940	-0.135	0.127	0.286	0.555	1117.940	0.388	0.148	0.008	0.059
FMNL3	383.084	0.046	0.237	0.845	0.898	383.084	-0.373	0.237	0.116	0.409	383.084	-0.419	0.274	0.126	0.321
CERS5	597.016	0.266	0.098	0.007	0.025	597.016	0.038	0.098	0.700	0.839	597.016	-0.228	0.114	0.044	0.170
DPP9	1637.265	0.058	0.263	0.827	0.886	1637.265	-0.573	0.264	0.030	0.322	1637.265	-0.630	0.305	0.039	0.155
CCDC149	23.610	1.073	0.649	0.098	0.193	23.610	0.596	0.653	0.361	0.613	23.610	-0.476	0.746	0.523	0.725
AP5B1	355.837	0.500	0.269	0.063	0.138	355.837	-0.403	0.271	0.137	0.428	355.837	-0.903	0.312	0.004	0.034
CCDC34	447.766	0.693	0.261	0.008	0.029	447.766	0.160	0.262	0.542	0.738	447.766	-0.533	0.302	0.078	0.240
MTMR6	478.402	-0.912	0.291	0.002	0.009	478.402	-0.600	0.289	0.038	0.327	478.402	0.312	0.336	0.353	0.586
MTMR7	108.733	0.579	0.224	0.010	0.034	108.733	-0.092	0.230	0.690	0.832	108.733	-0.671	0.262	0.011	0.068
MTMR4	1890.020	-0.273	0.394	0.488	0.621	1890.020	0.546	0.394	0.165	0.455	1890.020	0.820	0.455	0.072	0.229
TRIM47	975.989	1.075	0.229	0.000	0.000	975.989	-0.099	0.230	0.667	0.819	975.989	-1.174	0.265	0.000	0.000
NMI	215.537	0.179	0.186	0.337	0.477	215.537	-0.159	0.187	0.396	0.640	215.537	-0.338	0.216	0.119	0.309
MTA1	3134.235	0.168	0.179	0.349	0.489	3134.235	0.355	0.179	0.047	0.336	3134.235	0.187	0.207	0.365	0.597
LATS1	622.957	-0.944	0.233	0.000	0.001	622.957	-0.223	0.231	0.333	0.593	622.957	0.720	0.268	0.007	0.053
L3MBTL4	80.249	-0.532	0.196	0.007	0.024	80.249	0.018	0.183	0.920	0.960	80.249	0.551	0.224	0.014	0.081
SLC25A46	1004.919	-0.490	0.226	0.030	0.079	1004.919	-0.286	0.226	0.206	0.488	1004.919	0.205	0.262	0.434	0.656
ATP6V0D1	975.459	0.556	0.302	0.065	0.142	975.459	-0.785	0.303	0.010	0.298	975.459	-1.341	0.350	0.000	0.003
TMEM67	149.914	-0.305	0.240	0.204	0.331	149.914	0.250	0.235	0.287	0.555	149.914	0.555	0.276	0.044	0.168
TIGD7	101.190	-0.258	0.213	0.226	0.357	101.190	0.208	0.206	0.311	0.575	101.190	0.467	0.244	0.056	0.196
SEC22C	1039.142	-0.338	0.165	0.040	0.098	1039.142	-0.078	0.164	0.636	0.801	1039.142	0.261	0.191	0.172	0.389
SCARF2	276.457	0.341	0.292	0.244	0.377	276.457	-0.452	0.294	0.125	0.416	276.457	-0.793	0.339	0.019	0.100
KRT75	11218.833	0.045	0.341	0.895	0.932	11218.833	-0.804	0.341	0.018	0.306	11218.833	-0.849	0.393	0.031	0.134
CD1D	130.895	1.317	0.286	0.000	0.000	130.895	0.863	0.287	0.003	0.297	130.895	-0.454	0.327	0.166	0.381
SLC16A6	93.539	-0.970	0.418	0.020	0.059	93.539	-0.606	0.402	0.131	0.423	93.539	0.364	0.487	0.455	0.673
SLC16A4	10.014	1.027	0.691	0.138	0.248	10.014	0.108	0.722	0.881	0.940	10.014	-0.919	0.809	0.256	0.490
SLC16A3	4322.782	0.775	0.111	0.000	0.000	4322.782	0.211	0.111	0.058	0.347	4322.782	-0.564	0.128	0.000	0.000
PDLIM1	5014.702	0.291	0.246	0.236	0.369	5014.702	0.354	0.246	0.150	0.442	5014.702	0.063	0.284	0.825	0.914
CNOT9	908.897	-0.188	0.224	0.402	0.540	908.897	-0.411	0.224	0.067	0.355	908.897	-0.223	0.259	0.390	0.618
SLC39A13	676.074	1.061	0.204	0.000	0.000	676.074	-0.051	0.206	0.805	0.898	676.074	-1.112	0.237	0.000	0.000
SMC3	1839.712	-0.539	0.140	0.000	0.001	1839.712	-0.342	0.140	0.015	0.306	1839.712	0.197	0.162	0.224	0.451
BOD1	1359.205	-0.509	0.185	0.006	0.023	1359.205	-0.141	0.184	0.443	0.674	1359.205	0.367	0.214	0.085	0.254
PRPF4	1138.644	-1.108	0.279	0.000	0.001	1138.644	-0.512	0.278	0.066	0.354	1138.644	0.596	0.322	0.064	0.213
MSANTD3	847.905	0.226	0.171	0.187	0.312	847.905	-0.062	0.172	0.719	0.850	847.905	-0.288	0.198	0.147	0.354
LMF2	1206.224	0.500	0.153	0.001	0.006	1206.224	0.183	0.153	0.232	0.510	1206.224	-0.317	0.177	0.073	0.232
PRPF3	548.362	-0.034	0.226	0.879	0.921	548.362	-0.379	0.226	0.094	0.388	548.362	-0.345	0.262	0.187	0.407
C12orf29	434.325	-0.748	0.244	0.002	0.011	434.325	-0.583	0.243	0.017	0.306	434.325	0.165	0.283	0.560	0.752
FAM50A	1587.557	1.102	0.207	0.000	0.000	1587.557	-0.105	0.208	0.614	0.787	1587.557	-1.207	0.240	0.000	0.000
R3HDM4	1343.839	0.496	0.176	0.005	0.019	1343.839	0.000	0.176	0.999	0.999	1343.839	-0.496	0.203	0.015	0.083
TMEM259	2612.159	0.203	0.199	0.307	0.446	2612.159	-0.136	0.199	0.496	0.710	2612.159	-0.339	0.230	0.141	0.345
AIFM1	964.163	-0.070	0.278	0.801	0.869	964.163	-0.636	0.279	0.022	0.315	964.163	-0.566	0.322	0.079	0.242
GUSBP11	94.372	1.230	0.360	0.001	0.004	94.372	0.346	0.364	0.342	0.599	94.372	-0.884	0.416	0.033	0.141
DERL3	21.908	1.644	0.534	0.002	0.010	21.908	0.324	0.558	0.561	0.750	21.908	-1.320	0.621	0.034	0.142
CCNB2	1286.159	-0.441	0.244	0.070	0.149	1286.159	0.000	0.243	0.999	0.999	1286.159	0.441	0.281	0.117	0.306
CCNE2	143.782	0.051	0.389	0.897	0.933	143.782	-1.034	0.394	0.009	0.297	143.782	-1.085	0.454	0.017	0.092

RABEP1	981.530	-0.534	0.181	0.003	0.014	981.530	-0.346	0.180	0.055	0.343	981.530	0.188	0.209	0.369	0.601
DDX60L	164.988	0.316	0.262	0.228	0.359	164.988	-0.107	0.263	0.683	0.829	164.988	-0.423	0.303	0.163	0.377
LRP5L	60.891	0.931	0.355	0.009	0.031	60.891	0.272	0.360	0.449	0.678	60.891	-0.659	0.411	0.109	0.294
RRP9	1027.535	-0.388	0.374	0.300	0.438	1027.535	-0.865	0.374	0.021	0.312	1027.535	-0.477	0.433	0.270	0.505
CDKN2AIPNL	886.202	-0.101	0.167	0.543	0.668	886.202	-0.122	0.166	0.463	0.688	886.202	-0.021	0.193	0.914	0.960
ANKRD40	854.802	-0.410	0.209	0.049	0.114	854.802	-0.203	0.208	0.330	0.590	854.802	0.207	0.241	0.390	0.618
LOC91370	9.281	-0.769	0.736	0.296	0.434	9.281	-0.965	0.737	0.190	0.476	9.281	-0.196	0.874	0.822	0.913
UAP1L1	343.143	1.376	0.258	0.000	0.000	343.143	0.492	0.261	0.059	0.349	343.143	-0.884	0.298	0.003	0.029
ARHGEF1	1935.359	0.024	0.241	0.922	0.950	1935.359	-0.321	0.241	0.183	0.469	1935.359	-0.345	0.278	0.215	0.441
CBFA2T2	325.443	0.255	0.237	0.282	0.420	325.443	0.364	0.237	0.124	0.416	325.443	0.109	0.274	0.691	0.838
ZNF502	15.781	0.086	0.466	0.854	0.905	15.781	-0.075	0.465	0.871	0.935	15.781	-0.161	0.543	0.767	0.881
ATG12	864.212	-0.144	0.149	0.335	0.475	864.212	0.083	0.148	0.576	0.761	864.212	0.226	0.172	0.188	0.408
SESTD1	850.245	-0.189	0.232	0.417	0.554	850.245	0.164	0.231	0.478	0.698	850.245	0.353	0.268	0.188	0.408
BTF3L4	1292.292	-0.588	0.133	0.000	0.000	1292.292	-0.145	0.132	0.272	0.543	1292.292	0.443	0.154	0.004	0.034
CCDC74B	11.597	0.899	0.596	0.131	0.240	11.597	0.873	0.592	0.140	0.431	11.597	-0.026	0.675	0.970	0.985
PDCD5	2165.564	0.868	0.269	0.001	0.007	2165.564	-0.095	0.269	0.724	0.852	2165.564	-0.963	0.311	0.002	0.021
ATP23	262.201	0.238	0.247	0.335	0.475	262.201	0.339	0.246	0.168	0.457	262.201	0.101	0.285	0.722	0.856
SYNGR3	29.864	1.561	0.388	0.000	0.001	29.864	0.043	0.426	0.920	0.960	29.864	-1.518	0.463	0.001	0.014
RCCD1	43.490	1.860	0.500	0.000	0.002	43.490	0.484	0.513	0.345	0.601	43.490	-1.376	0.578	0.017	0.093
SYNGR2	2055.325	0.433	0.135	0.001	0.007	2055.325	-0.033	0.135	0.805	0.898	2055.325	-0.466	0.156	0.003	0.027
FAAP24	49.325	0.235	0.275	0.392	0.531	49.325	-0.047	0.277	0.865	0.931	49.325	-0.282	0.320	0.378	0.609
RNF185	903.295	-0.092	0.154	0.551	0.673	903.295	0.006	0.154	0.971	0.985	903.295	0.098	0.178	0.584	0.766
SYNGR1	10.573	0.792	0.588	0.178	0.300	10.573	0.431	0.593	0.467	0.690	10.573	-0.361	0.675	0.593	0.773
ACBD5	860.799	-0.687	0.241	0.004	0.018	860.799	0.021	0.240	0.932	0.966	860.799	0.708	0.278	0.011	0.070
HGS	2720.020	-0.110	0.208	0.596	0.711	2720.020	-0.277	0.208	0.183	0.470	2720.020	-0.167	0.241	0.488	0.699
PKDCC	39.598	0.954	0.248	0.000	0.001	39.598	0.117	0.261	0.655	0.812	39.598	-0.837	0.291	0.004	0.035
NEMF	712.589	0.164	0.221	0.457	0.592	712.589	-0.400	0.221	0.070	0.359	712.589	-0.564	0.255	0.027	0.123
DYRK1B	73.534	0.855	0.400	0.033	0.084	73.534	0.528	0.402	0.189	0.475	73.534	-0.327	0.460	0.478	0.691
CTDP1	971.886	0.012	0.251	0.963	0.978	971.886	-0.414	0.252	0.100	0.394	971.886	-0.426	0.291	0.143	0.348
PCED1B	55.460	0.227	0.342	0.507	0.637	55.460	-0.125	0.344	0.717	0.848	55.460	-0.352	0.398	0.376	0.608
ANKRD44	19.039	0.763	0.446	0.087	0.177	19.039	0.089	0.459	0.847	0.921	19.039	-0.674	0.521	0.195	0.418
RSAD2	184.744	5.651	0.539	0.000	0.000	184.744	1.313	0.562	0.019	0.306	184.744	-4.338	0.613	0.000	0.000
UBXN11	701.365	1.274	0.289	0.000	0.000	701.365	0.131	0.290	0.652	0.810	701.365	-1.143	0.334	0.001	0.009
EXO1	398.720	-1.410	0.430	0.001	0.006	398.720	-0.953	0.429	0.026	0.318	398.720	0.458	0.497	0.357	0.590
C12orf65	575.595	0.492	0.225	0.029	0.076	575.595	-0.100	0.226	0.657	0.813	575.595	-0.592	0.260	0.023	0.110
FIBP	714.289	-0.012	0.207	0.954	0.972	714.289	-0.033	0.206	0.873	0.936	714.289	-0.021	0.239	0.930	0.968
RPS19BP1	788.537	-0.015	0.298	0.961	0.977	788.537	-0.403	0.299	0.177	0.466	788.537	-0.389	0.345	0.260	0.494
PCSK7	277.162	0.081	0.433	0.851	0.903	277.162	-0.913	0.435	0.036	0.324	277.162	-0.994	0.502	0.048	0.176
ZNF830	244.940	-0.363	0.159	0.023	0.064	244.940	0.022	0.155	0.887	0.943	244.940	0.385	0.183	0.036	0.148
SLFN11	859.864	0.114	0.232	0.625	0.735	859.864	0.166	0.232	0.474	0.696	859.864	0.052	0.268	0.845	0.925
RASL10B	11.042	0.622	0.938	0.508	0.637	11.042	0.820	0.932	0.379	0.626	11.042	0.198	1.075	0.854	0.930
CHURC1	382.145	-0.464	0.249	0.063	0.137	382.145	0.124	0.247	0.615	0.788	382.145	0.589	0.287	0.041	0.160
DEPDC7	103.027	0.371	0.214	0.083	0.170	103.027	0.174	0.214	0.417	0.655	103.027	-0.197	0.247	0.425	0.648
DGKI	311.615	0.096	0.406	0.813	0.877	311.615	0.318	0.406	0.434	0.667	311.615	0.222	0.469	0.636	0.803
NEXN	93.850	0.754	0.375	0.044	0.105	93.850	-0.460	0.382	0.229	0.506	93.850	-1.214	0.438	0.006	0.044
ATPAF2	140.940	0.758	0.210	0.000	0.002	140.940	0.139	0.213	0.514	0.720	140.940	-0.619	0.244	0.011	0.070
BOC	26.786	2.008	0.367	0.000	0.000	26.786	0.298	0.408	0.464	0.689	26.786	-1.709	0.433	0.000	0.002
EBAG9	375.067	-0.598	0.126	0.000	0.000	375.067	-0.061	0.121	0.612	0.786	375.067	0.536	0.144	0.000	0.004
ZNF765	147.096	-0.229	0.202	0.258	0.393	147.096	-0.279	0.201	0.165	0.455	147.096	-0.050	0.235	0.831	0.917
MYADM	1608.329	0.644	0.199	0.001	0.007	1608.329	0.390	0.199	0.050	0.337	1608.329	-0.253	0.230	0.270	0.505
ZNF845	151.278	-0.023	0.170	0.894	0.931	151.278	0.129	0.167	0.441	0.673	151.278	0.151	0.196	0.440	0.660
COX7A2L	2935.666	-0.138	0.138	0.316	0.456	2935.666	0.205	0.137	0.135	0.426	2935.666	0.344	0.159	0.031	0.134
TMSB10	67940.278	0.938	0.273	0.001	0.004	67940.278	0.466	0.273	0.088	0.380	67940.278	-0.472	0.315	0.134	0.334
SYT12	126.270	0.724	0.352	0.040	0.097	126.270	0.553	0.352	0.116	0.409	126.270	-0.171	0.405	0.673	0.827
CENPL	280.901	-0.935	0.205	0.000	0.000	280.901	-0.403	0.201	0.046	0.334	280.901	0.533	0.237	0.024	0.115
SMDT1	2418.553	0.490	0.252	0.052	0.118	2418.553	0.396	0.252	0.115	0.407	2418.553	-0.094	0.291	0.747	0.870
SCAF11	1988.234	-0.569	0.216	0.009	0.030	1988.234	-0.162	0.216	0.454	0.681	1988.234	0.407	0.250	0.103	0.286
LONRF1	396.776	-0.045	0.202	0.823	0.885	396.776	-0.180	0.201	0.373	0.621	396.776	-0.135	0.233	0.564	0.754
RRP7BP	119.575	-0.387	0.428	0.366	0.506	119.575	-0.859	0.430	0.046	0.334	119.575	-0.472	0.498	0.342	0.576
LPAR2	60.152	1.902	0.519	0.000	0.002	60.152	0.750	0.525	0.153	0.445	60.152	-1.152	0.598	0.054	0.193
ACY3	9.422	1.876	0.594	0.002	0.008	9.422	0.515	0.646	0.425	0.661	9.422	-1.361	0.687	0.047	0.176
IL1RL1	5818.026	0.191	0.528	0.718	0.808	5818.026	-0.540	0.528	0.307	0.572	5818.026	-0.731	0.610	0.231	0.459
IDI2	41.816	-0.399	0.359	0.266	0.403	41.816	-0.458	0.356	0.199	0.482	41.816	-0.060	0.419	0.887	0.946
YTHDC1	2404.537	-0.399	0.100	0.000	0.001	2404.537	-0.361	0.100	0.000	0.211	2404.537	0.038	0.116	0.741	0.867
ELMSAN1	1317.906	-0.427	0.240	0.075	0.158	1317.906	-0.082	0.240	0.734	0.857	1317.906	0.346	0.277	0.213	0.438
MFSD4B	98.691	0.458	0.240	0.056	0.126	98.691	-0.417	0.248	0.093	0.388	98.691	-0.875	0.284	0.002	0.022
MAP3K13	571.419	0.649	0.190	0.001	0.004	571.419	0.169	0.191	0.377	0.625	571.419	-0.481	0.220	0.029	0.129

LIN52	115.928	-0.031	0.298	0.918	0.948	115.928	-0.531	0.301	0.078	0.369	115.928	-0.500	0.348	0.151	0.360
ZNF804A	143.603	-0.928	0.194	0.000	0.000	143.603	-0.197	0.185	0.286	0.555	143.603	0.731	0.223	0.001	0.014
NEK9	1852.821	-0.224	0.252	0.374	0.514	1852.821	0.250	0.251	0.321	0.582	1852.821	0.474	0.291	0.103	0.286
CABLES1	69.819	0.319	0.398	0.423	0.560	69.819	-0.791	0.407	0.052	0.341	69.819	-1.109	0.468	0.018	0.094
NXPE3	743.281	0.253	0.220	0.251	0.385	743.281	-0.148	0.221	0.502	0.714	743.281	-0.401	0.255	0.115	0.304
CHMP7	843.643	-0.384	0.137	0.005	0.020	843.643	-0.189	0.136	0.166	0.456	843.643	0.195	0.159	0.218	0.445
AP4M1	648.031	0.653	0.185	0.000	0.003	648.031	-0.012	0.186	0.948	0.974	648.031	-0.665	0.214	0.002	0.021
OSMR	5151.064	0.161	0.283	0.571	0.690	5151.064	0.331	0.283	0.242	0.518	5151.064	0.171	0.327	0.601	0.779
ALKBH8	263.951	-1.606	0.296	0.000	0.000	263.951	-0.928	0.292	0.001	0.297	263.951	0.678	0.342	0.047	0.176
ARHGEF2	3769.873	-1.380	0.191	0.000	0.000	3769.873	-0.125	0.190	0.512	0.719	3769.873	1.255	0.220	0.000	0.000
RASSF9	66.813	-0.714	0.464	0.124	0.230	66.813	0.456	0.454	0.315	0.578	66.813	1.170	0.532	0.028	0.126
ZW10	174.667	-0.969	0.341	0.004	0.018	174.667	-0.442	0.337	0.190	0.475	174.667	0.527	0.394	0.181	0.399
WDR20	389.938	-0.427	0.161	0.008	0.028	389.938	-0.198	0.159	0.211	0.492	389.938	0.229	0.186	0.219	0.446
BUB3	2852.130	-0.411	0.130	0.002	0.008	2852.130	-0.200	0.130	0.125	0.417	2852.130	0.211	0.151	0.161	0.374
REPS2	11.894	0.972	0.935	0.298	0.437	11.894	1.058	0.922	0.251	0.525	11.894	0.085	1.045	0.935	0.971
CALML4	186.588	0.262	0.188	0.164	0.284	186.588	-0.098	0.189	0.605	0.782	186.588	-0.359	0.219	0.100	0.281
MARVELD3	13.318	0.574	0.474	0.226	0.358	13.318	0.094	0.485	0.846	0.920	13.318	-0.479	0.553	0.386	0.615
RFT1	750.595	-0.373	0.223	0.095	0.189	750.595	-0.393	0.223	0.078	0.370	750.595	-0.020	0.258	0.939	0.973
SLC24A1	170.199	-0.410	0.330	0.214	0.343	170.199	0.245	0.326	0.453	0.680	170.199	0.655	0.380	0.084	0.252
TTC5	221.400	-0.712	0.271	0.009	0.031	221.400	-0.499	0.270	0.064	0.353	221.400	0.213	0.314	0.497	0.705
DDX21	6365.221	-1.357	0.329	0.000	0.000	6365.221	-0.804	0.328	0.014	0.306	6365.221	0.554	0.379	0.144	0.351
ZBED1	4095.371	-0.084	0.170	0.622	0.733	4095.371	0.062	0.170	0.715	0.847	4095.371	0.146	0.196	0.458	0.676
DEDD	630.558	-0.035	0.105	0.736	0.822	630.558	0.115	0.104	0.265	0.537	630.558	0.151	0.121	0.212	0.438
SLC16A7	689.480	-0.559	0.216	0.010	0.033	689.480	0.013	0.215	0.954	0.977	689.480	0.572	0.249	0.022	0.107
NDUFAF2	1261.763	0.615	0.274	0.025	0.068	1261.763	-0.149	0.275	0.588	0.769	1261.763	-0.764	0.317	0.016	0.088
ARRDC4	469.319	-0.699	0.269	0.009	0.032	469.319	0.245	0.266	0.357	0.611	469.319	0.944	0.310	0.002	0.024
COG7	191.646	0.190	0.169	0.260	0.395	191.646	0.056	0.168	0.737	0.860	191.646	-0.134	0.195	0.494	0.702
CXorf40A	318.877	0.219	0.181	0.227	0.359	318.877	0.120	0.181	0.508	0.717	318.877	-0.099	0.209	0.636	0.802
SLC33A1	878.303	-0.132	0.170	0.438	0.574	878.303	0.130	0.169	0.443	0.674	878.303	0.262	0.196	0.182	0.401
ZNF300	30.769	-0.395	0.402	0.326	0.466	30.769	-0.123	0.393	0.754	0.868	30.769	0.271	0.464	0.559	0.750
TPGS1	387.972	0.656	0.172	0.000	0.001	387.972	0.274	0.173	0.112	0.405	387.972	-0.382	0.198	0.054	0.192
ACVR2A	86.532	-0.279	0.227	0.219	0.350	86.532	-0.152	0.222	0.495	0.709	86.532	0.127	0.263	0.629	0.798
HACD1	364.804	0.811	0.168	0.000	0.000	364.804	0.213	0.170	0.210	0.491	364.804	-0.598	0.194	0.002	0.022
CCNQ	263.672	0.173	0.387	0.654	0.758	263.672	-0.847	0.390	0.030	0.322	263.672	-1.020	0.449	0.023	0.111
DCLK1	10.767	-0.383	1.132	0.735	0.821	10.767	0.728	1.109	0.511	0.719	10.767	1.111	1.296	0.391	0.620
SLC25A51	149.821	-0.068	0.187	0.715	0.805	149.821	-0.227	0.187	0.224	0.503	149.821	-0.159	0.218	0.467	0.682
SNX29	198.916	-0.181	0.244	0.459	0.593	198.916	-0.084	0.242	0.729	0.855	198.916	0.097	0.282	0.731	0.861
ZMYM4	1285.205	-0.638	0.160	0.000	0.001	1285.205	-0.036	0.159	0.821	0.907	1285.205	0.602	0.184	0.001	0.014
ZMYM3	793.228	-0.130	0.406	0.748	0.831	793.228	0.629	0.405	0.120	0.412	793.228	0.760	0.468	0.105	0.288
ZMYM6	311.568	-0.301	0.205	0.143	0.255	311.568	-0.130	0.204	0.523	0.727	311.568	0.171	0.237	0.471	0.685
ZMYM5	141.112	-0.377	0.246	0.126	0.232	141.112	-0.076	0.242	0.754	0.868	141.112	0.301	0.284	0.289	0.525
CTBP1-DT	58.881	-0.807	0.315	0.011	0.035	58.881	0.250	0.298	0.402	0.644	58.881	1.057	0.359	0.003	0.030
LRRFIP1	2831.927	-0.313	0.165	0.058	0.128	2831.927	-0.173	0.165	0.293	0.560	2831.927	0.140	0.190	0.463	0.679
LRRFIP2	676.401	0.269	0.160	0.092	0.184	676.401	0.095	0.160	0.554	0.746	676.401	-0.175	0.185	0.344	0.578
ZC3HAV1L	58.993	-0.448	0.357	0.209	0.338	58.993	0.321	0.344	0.351	0.606	58.993	0.769	0.407	0.059	0.203
TTC30A	39.144	-0.111	0.528	0.834	0.890	39.144	0.037	0.524	0.943	0.971	39.144	0.148	0.609	0.808	0.904
INTS4	302.916	-0.628	0.178	0.000	0.003	302.916	-0.113	0.174	0.518	0.723	302.916	0.516	0.205	0.012	0.073
OXNAD1	247.618	-0.679	0.318	0.033	0.084	247.618	-0.739	0.317	0.020	0.308	247.618	-0.060	0.368	0.871	0.938
AURKB	404.328	0.186	0.220	0.397	0.536	404.328	-0.003	0.220	0.990	0.994	404.328	-0.189	0.254	0.457	0.675
DSEL	426.497	-0.141	0.220	0.522	0.650	426.497	-0.222	0.220	0.314	0.577	426.497	-0.081	0.255	0.752	0.873
XPR1	1178.332	-0.325	0.233	0.163	0.282	1178.332	-0.203	0.233	0.383	0.629	1178.332	0.122	0.269	0.651	0.812
MTDH	5287.038	-0.509	0.131	0.000	0.001	5287.038	-0.185	0.131	0.157	0.449	5287.038	0.323	0.151	0.033	0.139
LARGE1	91.112	-0.021	0.539	0.969	0.981	91.112	0.722	0.535	0.177	0.466	91.112	0.743	0.620	0.231	0.459
MTSS2	3859.111	-0.021	0.306	0.945	0.966	3859.111	0.446	0.306	0.144	0.435	3859.111	0.468	0.353	0.185	0.405
VAPB	843.543	-0.485	0.114	0.000	0.000	843.543	-0.186	0.113	0.097	0.392	843.543	0.298	0.132	0.024	0.113
MTG1	205.376	-0.051	0.239	0.832	0.889	205.376	-0.420	0.240	0.080	0.371	205.376	-0.370	0.278	0.183	0.402
VAPA	2983.156	-0.369	0.093	0.000	0.001	2983.156	-0.139	0.093	0.133	0.425	2983.156	0.230	0.107	0.033	0.139
UBTD2	600.280	-0.253	0.158	0.108	0.208	600.280	-0.102	0.156	0.516	0.722	600.280	0.152	0.182	0.405	0.632
MTA2	2460.428	-0.524	0.230	0.023	0.064	2460.428	-0.537	0.230	0.020	0.306	2460.428	-0.013	0.266	0.960	0.981
TIAF1	8.597	1.459	0.821	0.075	0.158	8.597	0.203	0.849	0.811	0.901	8.597	-1.257	0.955	0.188	0.409
NOLC1	2904.985	-1.011	0.321	0.002	0.008	2904.985	-0.802	0.320	0.012	0.305	2904.985	0.209	0.370	0.573	0.760
CDHR1	7.597	0.961	1.066	0.367	0.508	7.597	0.059	1.082	0.957	0.978	7.597	-0.902	1.237	0.466	0.681
MAGI1	265.320	0.229	0.175	0.192	0.317	265.320	-0.189	0.176	0.284	0.553	265.320	-0.418	0.204	0.040	0.159
RCSD1	7.061	-2.127	1.442	0.140	0.251	7.061	-0.033	1.345	0.980	0.989	7.061	2.094	1.640	0.202	0.425
LINC01278	276.141	0.263	0.186	0.159	0.276	276.141	-0.038	0.187	0.841	0.917	276.141	-0.300	0.216	0.164	0.379
LMBRD2	556.426	-0.759	0.345	0.028	0.074	556.426	0.024	0.344	0.944	0.972	556.426	0.783	0.398	0.049	0.181
MRPS36	589.905	0.446	0.297	0.133	0.242	589.905	-0.164	0.298	0.582	0.766	589.905	-0.610	0.343	0.076	0.237

ATP6AP1L	54.849	0.450	0.556	0.418	0.556	54.849	0.779	0.553	0.158	0.451	54.849	0.330	0.639	0.606	0.782
ZNF461	55.037	-0.150	0.353	0.671	0.771	55.037	-0.011	0.349	0.975	0.986	55.037	0.139	0.408	0.733	0.862
ZNF585B	76.111	0.410	0.222	0.065	0.141	76.111	0.130	0.224	0.561	0.750	76.111	-0.280	0.258	0.277	0.512
GLYATL1	9.077	0.121	0.717	0.866	0.913	9.077	0.324	0.702	0.645	0.806	9.077	0.203	0.820	0.805	0.902
RAB11B	1561.505	0.386	0.159	0.015	0.047	1561.505	0.262	0.159	0.101	0.394	1561.505	-0.125	0.184	0.498	0.706
TMEM129	625.450	0.254	0.194	0.190	0.315	625.450	0.317	0.193	0.101	0.394	625.450	0.063	0.223	0.778	0.888
DLG5	10406.379	-0.564	0.413	0.172	0.292	10406.379	0.482	0.412	0.243	0.518	10406.379	1.045	0.476	0.028	0.126
MEX3A	621.815	-0.234	0.250	0.350	0.490	621.815	0.315	0.249	0.206	0.488	621.815	0.549	0.289	0.057	0.199
PTTG1	1797.125	0.485	0.263	0.065	0.141	1797.125	0.093	0.263	0.724	0.852	1797.125	-0.392	0.304	0.197	0.419
STRADA	354.929	0.241	0.236	0.307	0.446	354.929	-0.187	0.236	0.429	0.663	354.929	-0.428	0.273	0.117	0.306
METTL18	108.462	-0.221	0.207	0.285	0.423	108.462	-0.195	0.204	0.340	0.598	108.462	0.026	0.240	0.914	0.960
GORAB	139.626	-0.259	0.283	0.360	0.500	139.626	-0.357	0.283	0.206	0.488	139.626	-0.098	0.329	0.766	0.881
NAF1	248.153	-1.042	0.287	0.000	0.002	248.153	-0.546	0.284	0.054	0.342	248.153	0.496	0.331	0.135	0.335
IL32	11998.165	1.068	0.266	0.000	0.001	11998.165	-0.036	0.266	0.891	0.946	11998.165	-1.105	0.307	0.000	0.006
CRB3	13.563	0.721	0.530	0.173	0.295	13.563	0.567	0.529	0.284	0.553	13.563	-0.154	0.604	0.798	0.899
CCPG1	377.526	-0.675	0.254	0.008	0.028	377.526	-0.303	0.253	0.231	0.508	377.526	0.373	0.294	0.205	0.428
PXYLP1	708.244	-0.572	0.352	0.104	0.201	708.244	0.007	0.351	0.984	0.991	708.244	0.579	0.407	0.154	0.364
TBRG4	1438.805	-0.153	0.216	0.479	0.612	1438.805	-0.362	0.216	0.094	0.388	1438.805	-0.208	0.250	0.404	0.631
MRRF	675.861	-0.458	0.149	0.002	0.010	675.861	-0.059	0.147	0.690	0.832	675.861	0.399	0.171	0.020	0.101
CD7	15.434	2.584	0.629	0.000	0.000	15.434	0.445	0.721	0.537	0.735	15.434	-2.138	0.740	0.004	0.034
PNMA1	726.095	-0.490	0.228	0.031	0.081	726.095	-0.184	0.227	0.417	0.655	726.095	0.306	0.263	0.245	0.476
RBM18	742.681	-0.907	0.162	0.000	0.000	742.681	-0.292	0.160	0.068	0.357	742.681	0.615	0.187	0.001	0.013
NOG	567.865	0.849	0.235	0.000	0.002	567.865	0.269	0.237	0.255	0.528	567.865	-0.580	0.272	0.033	0.139
MSC	315.700	0.343	0.248	0.167	0.286	315.700	-0.182	0.250	0.465	0.689	315.700	-0.526	0.288	0.068	0.221
CHMP4C	391.041	-0.161	0.111	0.147	0.260	391.041	0.058	0.109	0.591	0.772	391.041	0.219	0.128	0.086	0.255
CRLF1	207.969	-0.155	0.241	0.521	0.649	207.969	0.228	0.237	0.336	0.594	207.969	0.383	0.277	0.167	0.382
GCNT3	18.297	3.290	0.809	0.000	0.001	18.297	0.945	0.967	0.329	0.589	18.297	-2.345	0.893	0.009	0.059
UBE2L6	1312.422	0.618	0.229	0.007	0.025	1312.422	0.440	0.229	0.054	0.342	1312.422	-0.178	0.264	0.499	0.706
BBIP1	547.885	0.461	0.247	0.062	0.136	547.885	0.355	0.247	0.149	0.442	547.885	-0.105	0.285	0.712	0.850
DHRS3	2470.456	0.841	0.202	0.000	0.000	2470.456	0.397	0.202	0.049	0.337	2470.456	-0.444	0.233	0.057	0.199
RPS6KA5	50.982	-0.649	0.275	0.018	0.054	50.982	-0.332	0.266	0.211	0.492	50.982	0.317	0.319	0.321	0.555
SPECC1	441.947	0.196	0.174	0.259	0.394	441.947	0.144	0.174	0.408	0.649	441.947	-0.053	0.201	0.793	0.896
NUMBL	850.711	0.464	0.146	0.001	0.008	850.711	0.292	0.146	0.046	0.334	850.711	-0.172	0.169	0.307	0.543
CACNA2D2	7.841	1.375	0.931	0.140	0.251	7.841	0.466	0.951	0.624	0.794	7.841	-0.909	1.075	0.398	0.625
AIMP1	1069.168	-0.070	0.110	0.524	0.651	1069.168	-0.090	0.110	0.414	0.653	1069.168	-0.019	0.127	0.879	0.942
BICDL1	75.687	0.592	0.505	0.241	0.374	75.687	1.058	0.501	0.035	0.322	75.687	0.466	0.578	0.420	0.644
TSPOAP1	9.591	0.238	0.593	0.688	0.785	9.591	0.086	0.592	0.885	0.943	9.591	-0.153	0.688	0.824	0.914
FANK1	31.456	1.430	0.602	0.018	0.052	31.456	1.084	0.604	0.073	0.362	31.456	-0.346	0.687	0.614	0.787
G6PC3	1081.531	0.565	0.270	0.036	0.090	1081.531	-0.175	0.270	0.518	0.723	1081.531	-0.740	0.312	0.018	0.094
MFHAS1	420.914	-0.088	0.261	0.735	0.821	420.914	-0.277	0.261	0.289	0.558	420.914	-0.188	0.302	0.533	0.732
ASB16	19.513	0.584	0.462	0.206	0.335	19.513	0.147	0.468	0.753	0.868	19.513	-0.437	0.536	0.415	0.641
ZNF764	127.937	-0.130	0.149	0.383	0.522	127.937	-0.188	0.148	0.204	0.487	127.937	-0.057	0.174	0.742	0.868
MOB1B	361.902	-0.280	0.227	0.217	0.347	361.902	-0.148	0.226	0.513	0.720	361.902	0.132	0.262	0.613	0.787
PDLIM7	1122.809	0.654	0.340	0.055	0.123	1122.809	-0.534	0.341	0.117	0.409	1122.809	-1.188	0.393	0.003	0.026
TIMM50	1703.287	-0.279	0.333	0.401	0.540	1703.287	-0.607	0.333	0.068	0.357	1703.287	-0.328	0.384	0.394	0.622
MAPKAPK2	1692.889	0.056	0.093	0.544	0.668	1692.889	-0.044	0.092	0.631	0.798	1692.889	-0.101	0.107	0.347	0.581
TIFA	469.236	-0.533	0.108	0.000	0.000	469.236	0.063	0.103	0.545	0.739	469.236	0.596	0.123	0.000	0.000
STK17B	533.275	-0.498	0.238	0.037	0.091	533.275	-0.442	0.238	0.063	0.350	533.275	0.057	0.276	0.838	0.921
STK17A	807.522	-0.838	0.203	0.000	0.000	807.522	-0.431	0.202	0.032	0.322	807.522	0.407	0.234	0.083	0.249
CYTH3	1580.385	-0.196	0.225	0.384	0.523	1580.385	0.042	0.225	0.852	0.924	1580.385	0.238	0.260	0.359	0.592
MAFG-DT	87.879	-0.432	0.364	0.236	0.369	87.879	-0.243	0.361	0.500	0.713	87.879	0.188	0.421	0.655	0.815
CYTH2	977.914	0.615	0.204	0.003	0.012	977.914	0.333	0.204	0.103	0.396	977.914	-0.283	0.235	0.230	0.459
MGME1	477.945	-0.605	0.188	0.001	0.007	477.945	-0.262	0.187	0.161	0.452	477.945	0.343	0.218	0.115	0.303
CYTH1	583.413	-0.054	0.134	0.687	0.784	583.413	0.126	0.133	0.343	0.600	583.413	0.180	0.155	0.244	0.475
DTD1	1044.313	-0.099	0.148	0.502	0.631	1044.313	0.062	0.147	0.676	0.825	1044.313	0.161	0.170	0.345	0.580
FAM114A1	240.335	1.297	0.350	0.000	0.002	240.335	0.291	0.353	0.410	0.650	240.335	-1.006	0.405	0.013	0.077
ITGB1BP1	1516.155	-0.141	0.120	0.239	0.372	1516.155	-0.125	0.120	0.295	0.563	1516.155	0.016	0.139	0.907	0.957
TMEM183A	1264.233	-0.258	0.194	0.183	0.307	1264.233	-0.261	0.194	0.178	0.467	1264.233	-0.003	0.224	0.991	0.995
ARRDC1	624.664	0.633	0.187	0.001	0.004	624.664	0.194	0.188	0.302	0.568	624.664	-0.439	0.216	0.042	0.165
DPH7	231.278	0.159	0.357	0.656	0.759	231.278	-0.369	0.358	0.303	0.569	231.278	-0.528	0.414	0.202	0.425
DNER	1091.032	-1.025	0.192	0.000	0.000	1091.032	0.387	0.187	0.038	0.327	1091.032	1.413	0.220	0.000	0.000
BCL7C	518.765	0.514	0.227	0.023	0.065	518.765	0.256	0.227	0.258	0.531	518.765	-0.258	0.262	0.325	0.559
SLC38A5	12.322	-0.625	0.814	0.443	0.578	12.322	1.506	0.725	0.038	0.327	12.322	2.131	0.896	0.017	0.094
BCL7B	611.616	-0.277	0.230	0.229	0.361	611.616	-0.438	0.230	0.057	0.346	611.616	-0.161	0.267	0.546	0.741
COPB2	3992.490	-0.349	0.194	0.072	0.152	3992.490	-0.316	0.194	0.103	0.395	3992.490	0.033	0.224	0.884	0.945
WDR46	3428.236	-0.381	0.367	0.299	0.438	3428.236	-0.656	0.367	0.074	0.363	3428.236	-0.275	0.424	0.516	0.720
ZBTB22	1852.155	0.295	0.224	0.188	0.313	1852.155	0.374	0.224	0.095	0.389	1852.155	0.079	0.259	0.760	0.878

HELB	66.415	-0.059	0.262	0.821	0.883	66.415	-0.046	0.259	0.861	0.929	66.415	0.014	0.303	0.964	0.983
SHKBP1	1134.812	0.266	0.204	0.193	0.318	1134.812	-0.220	0.205	0.281	0.551	1134.812	-0.487	0.236	0.039	0.157
CD9	309.765	0.653	0.244	0.007	0.027	309.765	0.197	0.245	0.421	0.658	309.765	-0.456	0.282	0.105	0.289
CENPBD1	106.044	-0.112	0.225	0.620	0.730	106.044	-0.231	0.225	0.303	0.570	106.044	-0.119	0.263	0.649	0.811
H2AW	1067.178	0.529	0.372	0.155	0.271	1067.178	0.674	0.372	0.070	0.359	1067.178	0.145	0.429	0.736	0.864
MED14	908.201	-0.503	0.237	0.034	0.086	908.201	0.090	0.236	0.703	0.840	908.201	0.593	0.273	0.030	0.132
ZNF276	477.036	0.433	0.199	0.029	0.077	477.036	-0.019	0.199	0.923	0.961	477.036	-0.453	0.230	0.049	0.180
GPR37L1	43.563	1.558	0.433	0.000	0.002	43.563	0.283	0.450	0.529	0.730	43.563	-1.275	0.504	0.011	0.071
NPIPA1	56.449	0.955	0.432	0.027	0.073	56.449	0.518	0.434	0.233	0.510	56.449	-0.436	0.498	0.381	0.610
REEP6	108.617	1.342	0.535	0.012	0.039	108.617	0.860	0.536	0.109	0.401	108.617	-0.483	0.616	0.433	0.655
IMP4	1222.517	-0.611	0.272	0.024	0.067	1222.517	-0.436	0.271	0.108	0.400	1222.517	0.175	0.314	0.578	0.762
ADGRG1	165.077	0.440	0.457	0.336	0.476	165.077	0.795	0.456	0.081	0.372	165.077	0.355	0.526	0.500	0.707
CD14	113.188	1.760	0.524	0.001	0.005	113.188	-0.646	0.537	0.229	0.507	113.188	-2.406	0.613	0.000	0.002
HNRNPLL	762.449	-0.973	0.224	0.000	0.000	762.449	-0.181	0.223	0.416	0.654	762.449	0.792	0.259	0.002	0.023
UBE2Q2	751.787	-0.275	0.150	0.067	0.145	751.787	-0.158	0.150	0.292	0.559	751.787	0.117	0.174	0.499	0.706
CCDC102A	484.102	0.610	0.371	0.100	0.196	484.102	0.700	0.371	0.059	0.349	484.102	0.090	0.428	0.834	0.919
MARS2	242.667	-1.083	0.380	0.004	0.018	242.667	-0.847	0.378	0.025	0.316	242.667	0.236	0.439	0.591	0.772
S1PR2	79.500	0.972	0.252	0.000	0.001	79.500	-0.086	0.266	0.746	0.865	79.500	-1.058	0.298	0.000	0.007
SRSF11	2232.969	0.081	0.168	0.630	0.740	2232.969	-0.086	0.168	0.611	0.785	2232.969	-0.167	0.194	0.391	0.620
ATP6V1F	1320.341	0.949	0.231	0.000	0.000	1320.341	0.016	0.231	0.945	0.973	1320.341	-0.933	0.267	0.000	0.008
PEX11G	13.479	0.919	0.629	0.144	0.256	13.479	0.382	0.638	0.550	0.743	13.479	-0.537	0.725	0.459	0.676
MARCHF9	526.645	1.472	0.361	0.000	0.000	526.645	0.654	0.361	0.070	0.359	526.645	-0.818	0.416	0.049	0.181
ZBTB47	741.972	0.313	0.127	0.014	0.043	741.972	-0.049	0.127	0.700	0.839	741.972	-0.362	0.147	0.014	0.080
ACVR2B	224.059	-0.517	0.180	0.004	0.017	224.059	0.108	0.175	0.535	0.734	224.059	0.625	0.207	0.002	0.025
COQ10A	101.594	0.354	0.227	0.118	0.222	101.594	0.474	0.224	0.034	0.322	101.594	0.120	0.259	0.643	0.806
SOCS6	368.123	-1.018	0.198	0.000	0.000	368.123	-0.469	0.195	0.016	0.306	368.123	0.549	0.229	0.017	0.091
CD83	181.508	-0.828	0.429	0.053	0.121	181.508	-0.961	0.428	0.025	0.316	181.508	-0.133	0.497	0.789	0.895
TEX30	118.096	0.087	0.379	0.818	0.881	118.096	-0.911	0.386	0.018	0.306	118.096	-0.998	0.444	0.025	0.116
NEURL3	62.088	3.459	0.753	0.000	0.000	62.088	1.039	0.789	0.188	0.474	62.088	-2.421	0.858	0.005	0.040
DMKN	160.847	2.373	0.190	0.000	0.000	160.847	0.130	0.206	0.528	0.729	160.847	-2.243	0.224	0.000	0.000
ZNF235	137.114	-0.255	0.209	0.221	0.352	137.114	0.097	0.204	0.636	0.801	137.114	0.352	0.240	0.143	0.348
NAPRT	104.337	0.197	0.258	0.446	0.581	104.337	-0.211	0.260	0.416	0.655	104.337	-0.408	0.301	0.175	0.392
TMEM44	253.108	1.214	0.275	0.000	0.000	253.108	0.379	0.277	0.172	0.461	253.108	-0.835	0.317	0.008	0.059
ASIC3	12.625	2.540	0.641	0.000	0.001	12.625	1.233	0.667	0.065	0.353	12.625	-1.307	0.718	0.069	0.223
ORAI3	658.364	1.110	0.495	0.025	0.068	658.364	0.772	0.495	0.119	0.411	658.364	-0.338	0.571	0.554	0.747
ZNF561	511.052	-0.515	0.189	0.006	0.024	511.052	0.034	0.187	0.854	0.925	511.052	0.550	0.217	0.011	0.071
KLF4	806.709	-1.312	0.289	0.000	0.000	806.709	-0.292	0.286	0.307	0.572	806.709	1.020	0.333	0.002	0.023
OLFM2	51.889	0.934	0.501	0.062	0.137	51.889	0.232	0.505	0.646	0.807	51.889	-0.701	0.579	0.226	0.454
NREP	238.827	0.762	0.248	0.002	0.010	238.827	0.149	0.249	0.550	0.743	238.827	-0.613	0.287	0.032	0.139
HTR7P1	26.316	-0.821	0.370	0.026	0.071	26.316	-0.356	0.351	0.310	0.575	26.316	0.465	0.427	0.277	0.511
PTER	244.792	-1.257	0.193	0.000	0.000	244.792	-0.126	0.184	0.492	0.707	244.792	1.131	0.221	0.000	0.000
COPS2	2815.758	-0.163	0.216	0.451	0.586	2815.758	0.172	0.216	0.425	0.662	2815.758	0.335	0.249	0.179	0.398
PIGM	219.341	-0.231	0.169	0.172	0.292	219.341	-0.505	0.170	0.003	0.297	219.341	-0.274	0.198	0.165	0.380
IGSF8	749.821	0.114	0.516	0.825	0.885	749.821	0.819	0.516	0.112	0.405	749.821	0.705	0.596	0.236	0.466
TRIP13	1614.786	-0.727	0.301	0.016	0.048	1614.786	-0.807	0.301	0.007	0.297	1614.786	-0.080	0.348	0.818	0.910
TRIP12	2612.165	-0.948	0.225	0.000	0.000	2612.165	-0.271	0.225	0.228	0.506	2612.165	0.677	0.260	0.009	0.062
TRIP11	657.228	-0.443	0.221	0.045	0.106	657.228	-0.277	0.220	0.208	0.490	657.228	0.166	0.255	0.516	0.720
PGAP3	177.081	0.779	0.315	0.013	0.042	177.081	0.384	0.315	0.223	0.502	177.081	-0.394	0.363	0.278	0.512
TRIP10	2530.599	0.457	0.159	0.004	0.017	2530.599	0.203	0.159	0.203	0.486	2530.599	-0.254	0.183	0.166	0.381
CCDC114	24.043	0.427	0.700	0.542	0.666	24.043	0.941	0.676	0.164	0.454	24.043	0.513	0.786	0.513	0.718
HMGN3	646.509	0.694	0.259	0.007	0.027	646.509	-0.308	0.260	0.236	0.513	646.509	-1.002	0.299	0.001	0.011
TRIP4	267.962	-0.366	0.145	0.011	0.038	267.962	0.004	0.141	0.977	0.987	267.962	0.370	0.167	0.026	0.121
ZNHIT3	865.283	0.119	0.155	0.442	0.577	865.283	-0.044	0.155	0.776	0.881	865.283	-0.163	0.179	0.362	0.594
GTF3C5	1112.802	-0.171	0.129	0.186	0.310	1112.802	-0.162	0.129	0.207	0.489	1112.802	0.009	0.149	0.954	0.979
GTF3C4	974.790	-0.813	0.236	0.001	0.004	974.790	-0.159	0.235	0.500	0.713	974.790	0.654	0.273	0.016	0.090
GTF3C3	601.662	-0.811	0.222	0.000	0.002	601.662	-0.414	0.221	0.061	0.350	601.662	0.397	0.256	0.121	0.313
B4GALT6	119.183	-0.522	0.226	0.021	0.060	119.183	-0.217	0.222	0.328	0.588	119.183	0.305	0.262	0.243	0.474
HAUS8	276.705	-0.396	0.411	0.336	0.476	276.705	-0.786	0.412	0.056	0.344	276.705	-0.390	0.476	0.413	0.638
B4GALT5	1615.558	0.808	0.249	0.001	0.007	1615.558	0.117	0.249	0.639	0.803	1615.558	-0.691	0.287	0.016	0.089
MVB12A	713.473	0.441	0.185	0.018	0.052	713.473	0.194	0.186	0.295	0.563	713.473	-0.246	0.214	0.250	0.483
SP140L	372.854	0.335	0.124	0.007	0.025	372.854	-0.017	0.125	0.891	0.945	372.854	-0.352	0.144	0.014	0.083
CNOT8	1854.210	0.067	0.280	0.812	0.877	1854.210	0.411	0.280	0.142	0.433	1854.210	0.345	0.324	0.287	0.522
TCEAL1	193.525	0.253	0.220	0.251	0.385	193.525	0.121	0.220	0.584	0.767	193.525	-0.132	0.254	0.604	0.781
MMGT1	460.498	-1.103	0.268	0.000	0.000	460.498	-0.164	0.265	0.536	0.735	460.498	0.939	0.309	0.002	0.025
VAMP3	1101.749	0.238	0.169	0.159	0.276	1101.749	0.114	0.169	0.501	0.714	1101.749	-0.124	0.195	0.523	0.725
SNAP29	594.116	0.123	0.109	0.260	0.395	594.116	-0.037	0.109	0.736	0.859	594.116	-0.160	0.127	0.207	0.431
LOC93429	12.692	0.230	0.876	0.792	0.863	12.692	-0.531	0.900	0.555	0.746	12.692	-0.761	1.034	0.462	0.678

EFTUD2	2494.024	-0.401	0.234	0.086	0.175	2494.024	-0.515	0.234	0.028	0.321	2494.024	-0.114	0.270	0.674	0.827
ARMC6	865.707	-0.471	0.274	0.085	0.174	865.707	-0.434	0.274	0.113	0.405	865.707	0.037	0.317	0.907	0.957
TAOK2	987.102	0.109	0.141	0.439	0.575	987.102	-0.279	0.141	0.048	0.337	987.102	-0.388	0.163	0.018	0.094
ZNF670	60.129	-0.661	0.319	0.038	0.094	60.129	-0.648	0.316	0.040	0.327	60.129	0.013	0.373	0.972	0.986
MAPK1IP1L	1058.047	-0.268	0.208	0.198	0.324	1058.047	-0.407	0.208	0.050	0.337	1058.047	-0.139	0.240	0.563	0.754
RPL23	6131.765	1.307	0.358	0.000	0.002	6131.765	0.498	0.358	0.164	0.454	6131.765	-0.808	0.413	0.050	0.184
SLC9A3R2	471.353	0.842	0.232	0.000	0.002	471.353	-0.075	0.234	0.748	0.866	471.353	-0.917	0.269	0.001	0.010
TXNL1	1259.257	0.059	0.140	0.671	0.771	1259.257	0.043	0.140	0.760	0.872	1259.257	-0.017	0.161	0.917	0.961
SLIT2	379.483	-0.748	0.394	0.058	0.128	379.483	-0.765	0.393	0.052	0.340	379.483	-0.018	0.455	0.969	0.985
UBE4A	915.224	-0.559	0.160	0.000	0.003	915.224	-0.217	0.159	0.171	0.460	915.224	0.342	0.185	0.064	0.213
ZFAND4	60.573	-0.526	0.374	0.159	0.277	60.573	-0.605	0.373	0.105	0.397	60.573	-0.079	0.436	0.857	0.931
ITGBL1	7.784	0.777	0.598	0.194	0.319	7.784	0.644	0.597	0.280	0.550	7.784	-0.133	0.679	0.845	0.925
TRMT10A	121.175	-0.905	0.217	0.000	0.000	121.175	-0.118	0.206	0.568	0.755	121.175	0.787	0.249	0.002	0.018
TBC1D31	486.700	-0.841	0.210	0.000	0.001	486.700	-0.329	0.208	0.113	0.405	486.700	0.512	0.242	0.035	0.144
PPIG	1119.351	0.405	0.220	0.066	0.142	1119.351	-0.216	0.220	0.326	0.586	1119.351	-0.621	0.254	0.015	0.083
LONP1	5744.244	-0.324	0.161	0.044	0.105	5744.244	-0.035	0.161	0.828	0.910	5744.244	0.289	0.186	0.120	0.312
FBXO44	196.538	1.102	0.279	0.000	0.001	196.538	0.709	0.280	0.011	0.305	196.538	-0.393	0.321	0.221	0.448
MRFAP1	6372.247	-0.318	0.154	0.039	0.096	6372.247	-0.256	0.154	0.097	0.392	6372.247	0.062	0.178	0.727	0.859
LOC93622	312.823	0.097	0.144	0.500	0.631	312.823	-0.022	0.144	0.877	0.937	312.823	-0.120	0.167	0.474	0.688
TADA2B	449.766	-0.107	0.186	0.565	0.686	449.766	-0.007	0.185	0.968	0.983	449.766	0.100	0.215	0.643	0.806
TBCK	273.521	-0.118	0.196	0.546	0.670	273.521	0.180	0.194	0.351	0.606	273.521	0.299	0.225	0.185	0.405
RAB28	256.538	-0.458	0.221	0.038	0.093	256.538	-0.142	0.218	0.514	0.720	256.538	0.316	0.255	0.215	0.441
TJAP1	692.076	-0.252	0.142	0.075	0.157	692.076	-0.060	0.141	0.671	0.822	692.076	0.193	0.164	0.239	0.469
ST7-AS1	42.014	-0.049	0.315	0.877	0.921	42.014	-0.109	0.313	0.728	0.855	42.014	-0.060	0.366	0.870	0.937
ST7-AS2	30.112	-1.101	0.386	0.004	0.018	30.112	-1.028	0.378	0.006	0.297	30.112	0.073	0.458	0.873	0.939
ARHGAP18	1214.149	-0.845	0.163	0.000	0.000	1214.149	0.016	0.162	0.920	0.960	1214.149	0.861	0.188	0.000	0.000
CADPS2	511.616	-0.059	0.225	0.792	0.862	511.616	0.203	0.224	0.365	0.616	511.616	0.262	0.259	0.311	0.547
RAB9A	1001.360	0.013	0.132	0.921	0.950	1001.360	-0.027	0.132	0.835	0.915	1001.360	-0.040	0.153	0.791	0.896
SLC9A3R1	412.778	0.308	0.210	0.143	0.255	412.778	-0.317	0.211	0.134	0.426	412.778	-0.625	0.244	0.010	0.067
KIF3B	828.146	-0.255	0.216	0.239	0.372	828.146	-0.171	0.216	0.428	0.663	828.146	0.083	0.250	0.738	0.866
ZFYVE9	626.736	-0.589	0.177	0.001	0.005	626.736	-0.064	0.175	0.714	0.847	626.736	0.525	0.204	0.010	0.066
PLAA	1094.778	-0.307	0.147	0.036	0.091	1094.778	-0.309	0.146	0.035	0.322	1094.778	-0.002	0.170	0.992	0.995
PPT2	353.061	0.828	0.225	0.000	0.002	353.061	-0.157	0.228	0.491	0.707	353.061	-0.985	0.261	0.000	0.003
TM9SF2	1690.857	0.033	0.109	0.761	0.841	1690.857	-0.080	0.109	0.461	0.687	1690.857	-0.113	0.126	0.368	0.600
COX5A	2254.047	0.411	0.195	0.034	0.087	2254.047	-0.048	0.195	0.803	0.897	2254.047	-0.460	0.225	0.041	0.161
GRHPR	1816.917	0.135	0.145	0.352	0.492	1816.917	0.162	0.145	0.263	0.535	1816.917	0.027	0.168	0.871	0.938
COG1	743.932	0.315	0.126	0.013	0.041	743.932	0.071	0.127	0.577	0.762	743.932	-0.244	0.146	0.095	0.271
TSIX	431.152	1.546	0.379	0.000	0.001	431.152	0.477	0.380	0.210	0.491	431.152	-1.069	0.438	0.015	0.084
LIPG	40.624	1.435	0.589	0.015	0.046	40.624	-1.518	0.631	0.016	0.306	40.624	-2.953	0.712	0.000	0.001
CD27	8.658	0.834	0.549	0.129	0.236	8.658	0.828	0.541	0.126	0.418	8.658	-0.005	0.612	0.993	0.996
CIAO1	1495.199	-0.115	0.132	0.383	0.523	1495.199	-0.317	0.132	0.017	0.306	1495.199	-0.201	0.153	0.188	0.409
TGFBRAP1	649.431	-0.926	0.234	0.000	0.001	649.431	-0.090	0.232	0.697	0.837	649.431	0.835	0.270	0.002	0.021
HS6ST1	909.502	0.618	0.197	0.002	0.009	909.502	0.219	0.197	0.266	0.538	909.502	-0.398	0.228	0.080	0.244
GCNA	16.939	0.892	0.644	0.166	0.286	16.939	1.061	0.633	0.094	0.388	16.939	0.169	0.721	0.815	0.908
NMT2	911.880	-0.309	0.120	0.010	0.034	911.880	-0.192	0.120	0.108	0.400	911.880	0.117	0.139	0.400	0.627
ACTR8	439.714	-0.582	0.181	0.001	0.007	439.714	-0.198	0.179	0.268	0.539	439.714	0.384	0.209	0.066	0.216
ATP5IF1	1874.191	0.962	0.275	0.000	0.003	1874.191	0.258	0.276	0.349	0.604	1874.191	-0.704	0.318	0.027	0.123
FOXP2	15.602	0.414	0.675	0.540	0.665	15.602	0.460	0.671	0.493	0.708	15.602	0.047	0.775	0.952	0.978
STOML1	238.283	-0.057	0.321	0.859	0.908	238.283	-0.777	0.323	0.016	0.306	238.283	-0.720	0.373	0.054	0.191
RECQL5	353.026	0.011	0.129	0.931	0.956	353.026	0.139	0.128	0.277	0.547	353.026	0.128	0.149	0.391	0.619
PIGS	820.390	-0.399	0.255	0.117	0.220	820.390	-0.068	0.254	0.790	0.890	820.390	0.332	0.294	0.259	0.494
SERHL	136.460	-0.247	0.363	0.496	0.627	136.460	-0.984	0.366	0.007	0.297	136.460	-0.736	0.424	0.082	0.248
RECQL4	555.472	-0.177	0.262	0.498	0.629	555.472	-0.356	0.262	0.174	0.463	555.472	-0.179	0.303	0.554	0.747
TTYH2	13.067	-0.052	0.558	0.926	0.953	13.067	-0.078	0.554	0.888	0.944	13.067	-0.026	0.648	0.968	0.985
GRAP2	17.863	-1.013	0.660	0.125	0.230	17.863	0.670	0.617	0.278	0.548	17.863	1.683	0.746	0.024	0.114
CGB7	9.556	1.626	0.820	0.047	0.111	9.556	1.280	0.824	0.120	0.412	9.556	-0.347	0.926	0.708	0.849
SELENOF	2931.642	-0.372	0.160	0.020	0.058	2931.642	-0.094	0.160	0.558	0.748	2931.642	0.279	0.185	0.132	0.331
HTRA3	2054.347	0.421	0.227	0.064	0.139	2054.347	0.155	0.227	0.495	0.709	2054.347	-0.266	0.263	0.311	0.546
CAMK2N2	28.472	0.681	0.332	0.040	0.097	28.472	0.047	0.343	0.890	0.945	28.472	-0.634	0.389	0.103	0.285
ZNF101	68.554	0.011	0.242	0.965	0.979	68.554	-0.230	0.243	0.343	0.600	68.554	-0.241	0.283	0.395	0.622
LPXN	58.015	-0.126	0.319	0.693	0.788	58.015	-0.660	0.325	0.042	0.330	58.015	-0.534	0.377	0.157	0.368
SYAP1	1627.210	0.116	0.124	0.350	0.490	1627.210	-0.142	0.124	0.254	0.527	1627.210	-0.257	0.143	0.072	0.230
LENG9	1368.419	0.291	0.202	0.149	0.263	1368.419	0.534	0.202	0.008	0.297	1368.419	0.243	0.233	0.297	0.533
ZRANB2	1513.044	-0.989	0.203	0.000	0.000	1513.044	-0.428	0.202	0.034	0.322	1513.044	0.561	0.234	0.017	0.090
SFXN1	1960.988	-0.399	0.132	0.003	0.012	1960.988	-0.180	0.132	0.171	0.460	1960.988	0.219	0.153	0.152	0.361
PEX16	423.309	0.341	0.212	0.108	0.208	423.309	-0.056	0.213	0.794	0.893	423.309	-0.397	0.246	0.107	0.291
SFXN5	160.273	0.198	0.167	0.234	0.367	160.273	-0.086	0.168	0.607	0.783	160.273	-0.285	0.194	0.143	0.348

SNRNP40	907.383	-0.588	0.344	0.087	0.177	907.383	-0.743	0.344	0.031	0.322	907.383	-0.155	0.398	0.697	0.841
ORMDL1	367.857	0.194	0.243	0.424	0.561	367.857	-0.301	0.244	0.218	0.497	367.857	-0.495	0.282	0.079	0.242
ORMDL3	507.044	1.056	0.136	0.000	0.000	507.044	0.023	0.139	0.867	0.932	507.044	-1.033	0.159	0.000	0.000
PAXBP1	375.032	-0.107	0.207	0.607	0.719	375.032	-0.131	0.207	0.527	0.729	375.032	-0.024	0.240	0.920	0.963
TMEM203	775.100	-0.105	0.145	0.471	0.605	775.100	-0.126	0.145	0.383	0.629	775.100	-0.022	0.168	0.897	0.951
ARHGAP29	149.470	0.794	0.209	0.000	0.001	149.470	0.394	0.210	0.061	0.350	149.470	-0.400	0.241	0.097	0.276
MED21	402.843	0.042	0.186	0.820	0.882	402.843	-0.260	0.186	0.163	0.454	402.843	-0.302	0.215	0.161	0.374
SYTL3	306.050	0.268	0.180	0.138	0.248	306.050	0.175	0.180	0.330	0.590	306.050	-0.093	0.208	0.657	0.817
SYTL4	128.278	-0.655	0.276	0.018	0.052	128.278	-0.526	0.273	0.054	0.342	128.278	0.129	0.321	0.687	0.835
ARHGAP12	838.899	-0.679	0.185	0.000	0.002	838.899	-0.058	0.183	0.749	0.867	838.899	0.621	0.213	0.004	0.032
TJP2	2120.768	-0.875	0.228	0.000	0.001	2120.768	-0.366	0.227	0.108	0.400	2120.768	0.509	0.263	0.053	0.189
FADS2	295.476	0.233	0.222	0.294	0.432	295.476	-0.010	0.223	0.964	0.981	295.476	-0.243	0.257	0.344	0.578
DDX23	1847.994	-0.351	0.197	0.076	0.158	1847.994	-0.445	0.197	0.024	0.316	1847.994	-0.094	0.228	0.680	0.830
CRIPT	271.391	0.466	0.137	0.001	0.004	271.391	0.089	0.138	0.519	0.724	271.391	-0.378	0.158	0.017	0.093
ZNF264	205.153	0.444	0.200	0.027	0.072	205.153	0.376	0.200	0.060	0.350	205.153	-0.067	0.230	0.771	0.883
FOXQ1	330.170	-0.636	0.403	0.115	0.216	330.170	-0.879	0.403	0.029	0.322	330.170	-0.243	0.466	0.602	0.780
H2AZ2	1689.807	0.258	0.147	0.080	0.165	1689.807	-0.090	0.147	0.542	0.738	1689.807	-0.348	0.170	0.041	0.161
KCNK6	36.624	1.500	0.496	0.002	0.011	36.624	0.745	0.502	0.138	0.429	36.624	-0.756	0.569	0.184	0.404
TP53INP1	27.503	0.700	0.722	0.332	0.472	27.503	0.337	0.724	0.642	0.805	27.503	-0.364	0.833	0.663	0.820
CDYL	654.604	-1.186	0.241	0.000	0.000	654.604	-0.402	0.239	0.093	0.388	654.604	0.784	0.279	0.005	0.040
ABCG2	28.806	-0.034	0.635	0.958	0.975	28.806	-1.806	0.665	0.007	0.297	28.806	-1.773	0.761	0.020	0.101
CHST2	1248.263	0.139	0.332	0.676	0.775	1248.263	-0.466	0.332	0.161	0.452	1248.263	-0.605	0.384	0.115	0.304
MED23	410.010	-0.222	0.243	0.362	0.502	410.010	0.149	0.242	0.536	0.734	410.010	0.371	0.280	0.185	0.405
MED17	362.678	-0.316	0.153	0.039	0.096	362.678	-0.393	0.153	0.010	0.298	362.678	-0.077	0.178	0.663	0.821
MED26	319.888	-0.069	0.209	0.743	0.827	319.888	-0.197	0.209	0.346	0.602	319.888	-0.128	0.242	0.597	0.776
MED27	308.471	-0.333	0.375	0.374	0.514	308.471	-0.878	0.376	0.019	0.306	308.471	-0.545	0.434	0.210	0.435
MED7	101.279	0.225	0.156	0.148	0.262	101.279	-0.121	0.158	0.445	0.675	101.279	-0.346	0.183	0.059	0.202
QKI	2639.760	-0.611	0.209	0.003	0.015	2639.760	-0.247	0.209	0.237	0.514	2639.760	0.364	0.241	0.132	0.329
ITM2B	1744.184	0.594	0.212	0.005	0.020	1744.184	0.332	0.212	0.117	0.409	1744.184	-0.261	0.245	0.286	0.521
GSTO1	2437.858	0.889	0.182	0.000	0.000	2437.858	0.125	0.183	0.495	0.710	2437.858	-0.764	0.211	0.000	0.005
AIM2	222.381	1.592	0.254	0.000	0.000	222.381	-0.399	0.264	0.132	0.424	222.381	-1.990	0.299	0.000	0.000
MAP4K4	4003.650	0.246	0.186	0.186	0.310	4003.650	-0.440	0.186	0.018	0.306	4003.650	-0.686	0.215	0.001	0.017
CD33	71.758	0.042	0.258	0.871	0.917	71.758	0.343	0.252	0.174	0.463	71.758	0.301	0.296	0.308	0.544
EIF2AK3	682.763	-0.081	0.290	0.779	0.853	682.763	0.125	0.289	0.665	0.818	682.763	0.207	0.335	0.537	0.735
ITM2A	83.260	0.082	0.348	0.814	0.878	83.260	0.256	0.346	0.460	0.686	83.260	0.174	0.401	0.665	0.822
GGPS1	364.000	1.056	0.253	0.000	0.000	364.000	0.474	0.254	0.062	0.350	364.000	-0.582	0.292	0.046	0.174
HOMER3	1261.278	0.854	0.265	0.001	0.007	1261.278	-0.292	0.266	0.272	0.543	1261.278	-1.146	0.307	0.000	0.004
HOMER2	212.725	-0.409	0.399	0.305	0.444	212.725	-1.018	0.401	0.011	0.302	212.725	-0.608	0.463	0.189	0.410
HOMER1	353.986	-0.617	0.279	0.027	0.072	353.986	-0.448	0.277	0.107	0.399	353.986	0.170	0.322	0.599	0.777
ARHGEF6	207.191	0.465	0.436	0.287	0.425	207.191	0.630	0.435	0.148	0.441	207.191	0.165	0.503	0.743	0.868
RASAL2	380.913	-0.183	0.238	0.442	0.577	380.913	-0.112	0.237	0.635	0.801	380.913	0.070	0.275	0.798	0.899
PICK1	294.303	0.299	0.171	0.080	0.166	294.303	-0.048	0.172	0.780	0.883	294.303	-0.347	0.199	0.080	0.245
AKAP7	150.534	-0.461	0.163	0.005	0.018	150.534	0.133	0.155	0.389	0.635	150.534	0.595	0.186	0.001	0.017
IL27RA	487.393	0.418	0.171	0.015	0.046	487.393	0.326	0.171	0.057	0.347	487.393	-0.092	0.198	0.641	0.805
SH3BP5	416.698	0.399	0.147	0.006	0.024	416.698	0.163	0.147	0.266	0.538	416.698	-0.236	0.169	0.164	0.379
CHST3	1250.715	-0.310	0.170	0.069	0.148	1250.715	0.006	0.170	0.972	0.985	1250.715	0.315	0.197	0.109	0.294
EIF4E2	1136.316	0.357	0.093	0.000	0.001	1136.316	-0.116	0.094	0.215	0.494	1136.316	-0.473	0.108	0.000	0.000
AKAP6	25.211	0.115	0.436	0.792	0.862	25.211	0.590	0.425	0.165	0.455	25.211	0.475	0.497	0.339	0.574
THEMIS2	99.338	2.426	0.410	0.000	0.000	99.338	1.064	0.418	0.011	0.301	99.338	-1.362	0.468	0.004	0.033
ATG5	607.189	-0.507	0.118	0.000	0.000	607.189	-0.164	0.116	0.156	0.448	607.189	0.343	0.136	0.012	0.073
ROCK2	1035.446	-0.476	0.227	0.036	0.090	1035.446	-0.201	0.226	0.374	0.622	1035.446	0.274	0.262	0.295	0.530
NAPSA	8.445	0.360	0.863	0.676	0.775	8.445	0.441	0.855	0.606	0.782	8.445	0.080	0.990	0.935	0.971
MED20	449.089	-0.812	0.214	0.000	0.001	449.089	-0.245	0.212	0.248	0.522	449.089	0.567	0.247	0.022	0.107
MAPK8IP1	194.985	0.154	0.169	0.363	0.503	194.985	-0.011	0.169	0.946	0.973	194.985	-0.165	0.196	0.399	0.626
CD36	58.857	2.697	0.493	0.000	0.000	58.857	0.251	0.533	0.638	0.803	58.857	-2.446	0.580	0.000	0.001
ONECUT2	22.911	-0.096	0.525	0.855	0.906	22.911	-0.443	0.529	0.402	0.645	22.911	-0.347	0.614	0.572	0.760
SLC25A27	40.341	1.240	0.393	0.002	0.008	40.341	0.629	0.398	0.114	0.405	40.341	-0.612	0.451	0.175	0.392
STX8	240.776	0.641	0.255	0.012	0.039	240.776	0.467	0.255	0.067	0.354	240.776	-0.174	0.294	0.554	0.747
PIGL	134.258	0.204	0.209	0.329	0.469	134.258	0.379	0.206	0.066	0.354	134.258	0.175	0.240	0.466	0.681
PIGB	249.326	-0.118	0.137	0.391	0.530	249.326	0.096	0.134	0.476	0.697	249.326	0.213	0.158	0.177	0.394
PGS1	384.388	0.335	0.097	0.001	0.004	384.388	0.059	0.097	0.542	0.738	384.388	-0.276	0.112	0.014	0.082
SCARB1	5864.508	0.669	0.106	0.000	0.000	5864.508	0.073	0.106	0.493	0.708	5864.508	-0.596	0.122	0.000	0.000
PSMF1	2265.871	0.000	0.116	0.998	0.999	2265.871	0.065	0.116	0.573	0.758	2265.871	0.066	0.134	0.624	0.794
KIF23	1018.009	-1.090	0.198	0.000	0.000	1018.009	-0.403	0.196	0.040	0.327	1018.009	0.687	0.228	0.003	0.026
SLC4A7	678.096	-1.383	0.358	0.000	0.001	678.096	-0.754	0.357	0.034	0.322	678.096	0.629	0.413	0.128	0.323
MYOT	9.522	0.182	0.624	0.771	0.848	9.522	0.456	0.606	0.452	0.680	9.522	0.274	0.710	0.699	0.843
ACY1	191.962	-0.911	0.217	0.000	0.000	191.962	-0.027	0.210	0.899	0.949	191.962	0.884	0.249	0.000	0.007

SCARB2	2588.572	0.087	0.179	0.625	0.735	2588.572	0.130	0.179	0.465	0.689	2588.572	0.043	0.206	0.835	0.920
MAGED1	421.471	0.633	0.162	0.000	0.001	421.471	0.016	0.164	0.921	0.960	421.471	-0.617	0.188	0.001	0.014
RPH3AL	71.420	0.509	0.371	0.170	0.290	71.420	0.500	0.369	0.176	0.465	71.420	-0.009	0.426	0.982	0.990
XAGE2	182.003	0.564	0.431	0.191	0.316	182.003	0.872	0.430	0.043	0.330	182.003	0.308	0.497	0.535	0.733
CD37	10.131	0.906	0.478	0.058	0.129	10.131	-0.152	0.520	0.770	0.878	10.131	-1.058	0.576	0.066	0.217
ADAMTS1	122.581	-0.214	0.343	0.533	0.658	122.581	-0.309	0.342	0.367	0.618	122.581	-0.095	0.397	0.811	0.906
PMPCB	1430.366	-0.406	0.111	0.000	0.002	1430.366	0.070	0.110	0.526	0.729	1430.366	0.476	0.128	0.000	0.004
FXR2	811.611	-0.666	0.220	0.002	0.011	811.611	-0.260	0.219	0.235	0.512	811.611	0.406	0.254	0.110	0.296
LITAF	3031.206	0.086	0.118	0.470	0.604	3031.206	0.057	0.118	0.633	0.800	3031.206	-0.029	0.137	0.832	0.918
SPTLC2	559.091	0.203	0.161	0.207	0.336	559.091	-0.266	0.162	0.100	0.394	559.091	-0.470	0.187	0.012	0.074
GDF15	1083.042	-0.807	0.709	0.255	0.389	1083.042	-1.508	0.710	0.034	0.322	1083.042	-0.700	0.819	0.393	0.621
TBPL1	232.217	0.222	0.159	0.162	0.281	232.217	0.018	0.159	0.908	0.954	232.217	-0.204	0.184	0.268	0.503
NPEPPS	3018.199	-0.284	0.317	0.370	0.511	3018.199	0.456	0.316	0.150	0.442	3018.199	0.739	0.365	0.043	0.166
EEF1E1	106.145	-0.032	0.366	0.929	0.956	106.145	-0.442	0.367	0.229	0.507	106.145	-0.409	0.425	0.336	0.570
SCAMP1	1541.676	-0.170	0.178	0.339	0.479	1541.676	-0.029	0.178	0.868	0.933	1541.676	0.141	0.206	0.493	0.702
TECR	2491.176	0.767	0.238	0.001	0.007	2491.176	0.246	0.238	0.302	0.568	2491.176	-0.521	0.275	0.058	0.201
VPS4B	825.919	-0.670	0.189	0.000	0.003	825.919	-0.171	0.187	0.361	0.614	825.919	0.499	0.218	0.022	0.107
MPDU1	380.878	0.051	0.235	0.828	0.887	380.878	-0.285	0.236	0.227	0.505	380.878	-0.336	0.272	0.218	0.444
GOSR1	776.894	-0.492	0.161	0.002	0.011	776.894	-0.242	0.160	0.131	0.423	776.894	0.250	0.187	0.180	0.398
TMEM59	2877.095	0.026	0.242	0.915	0.946	2877.095	0.388	0.242	0.108	0.400	2877.095	0.363	0.279	0.194	0.416
BAG5	723.187	-0.551	0.246	0.025	0.069	723.187	-0.575	0.246	0.019	0.306	723.187	-0.024	0.285	0.933	0.970
ENTPD1	241.107	-0.790	0.317	0.013	0.041	241.107	-0.833	0.316	0.008	0.297	241.107	-0.043	0.368	0.908	0.958
BAG4	505.771	-0.242	0.106	0.022	0.062	505.771	0.006	0.104	0.955	0.978	505.771	0.247	0.122	0.042	0.165
BAG3	2353.838	0.117	0.129	0.363	0.504	2353.838	0.054	0.129	0.674	0.824	2353.838	-0.063	0.149	0.672	0.826
BAG2	1228.572	-0.475	0.246	0.053	0.122	1228.572	-0.506	0.246	0.040	0.327	1228.572	-0.031	0.284	0.914	0.960
POLR1C	1249.273	-0.385	0.171	0.024	0.066	1249.273	-0.171	0.170	0.314	0.577	1249.273	0.214	0.197	0.278	0.513
ZNF254	72.730	0.076	0.218	0.727	0.815	72.730	-0.058	0.217	0.790	0.890	72.730	-0.134	0.253	0.597	0.776
PTGES	267.894	2.103	0.526	0.000	0.001	267.894	0.879	0.528	0.096	0.391	267.894	-1.224	0.607	0.044	0.168
TP53I11	17.663	1.266	0.393	0.001	0.007	17.663	-0.149	0.433	0.731	0.856	17.663	-1.415	0.474	0.003	0.028
EI24	1565.886	-0.106	0.182	0.563	0.683	1565.886	-0.438	0.182	0.016	0.306	1565.886	-0.332	0.211	0.115	0.304
TP53I3	196.637	0.452	0.209	0.031	0.080	196.637	0.311	0.209	0.137	0.428	196.637	-0.141	0.241	0.558	0.750
CIR1	410.603	0.899	0.283	0.002	0.008	410.603	0.171	0.285	0.549	0.743	410.603	-0.729	0.328	0.026	0.121
NRG2	30.968	1.374	0.451	0.002	0.011	30.968	0.841	0.456	0.065	0.353	30.968	-0.533	0.516	0.301	0.537
RAB3D	1283.290	0.406	0.325	0.211	0.340	1283.290	0.682	0.324	0.036	0.324	1283.290	0.276	0.375	0.462	0.679
APBA3	416.971	0.690	0.218	0.002	0.008	416.971	0.021	0.220	0.925	0.962	416.971	-0.669	0.253	0.008	0.057
ENTPD6	918.765	0.105	0.189	0.580	0.698	918.765	-0.223	0.189	0.239	0.516	918.765	-0.327	0.219	0.134	0.334
ATP6V1G1	1573.777	0.635	0.177	0.000	0.002	1573.777	0.046	0.177	0.796	0.894	1573.777	-0.589	0.204	0.004	0.035
ATP5MF	1203.388	0.827	0.267	0.002	0.010	1203.388	-0.197	0.268	0.461	0.687	1203.388	-1.024	0.309	0.001	0.012
SPAG7	414.001	0.620	0.238	0.009	0.032	414.001	0.237	0.238	0.320	0.581	414.001	-0.383	0.275	0.163	0.377
MRPL33	410.645	-0.009	0.204	0.966	0.979	410.645	-0.165	0.204	0.417	0.655	410.645	-0.157	0.236	0.506	0.712
SEC22B	1442.812	0.271	0.140	0.052	0.120	1442.812	-0.310	0.140	0.027	0.320	1442.812	-0.581	0.162	0.000	0.006
MACROH2A1	4058.105	0.128	0.166	0.440	0.576	4058.105	-0.302	0.166	0.069	0.357	4058.105	-0.431	0.192	0.025	0.117
ATP5MPL	623.395	0.411	0.194	0.034	0.086	623.395	0.053	0.194	0.786	0.888	623.395	-0.359	0.224	0.110	0.295
CHD1L	997.845	-0.376	0.133	0.005	0.018	997.845	0.086	0.132	0.512	0.719	997.845	0.463	0.153	0.003	0.026
VPS26A	1275.358	-0.538	0.184	0.003	0.015	1275.358	-0.340	0.183	0.063	0.350	1275.358	0.198	0.212	0.350	0.583
MINPP1	348.557	0.028	0.115	0.805	0.871	348.557	0.061	0.114	0.590	0.771	348.557	0.033	0.133	0.804	0.902
H6PD	835.851	0.264	0.381	0.489	0.622	835.851	0.703	0.381	0.065	0.354	835.851	0.439	0.440	0.318	0.554
BCAR1	3533.459	0.854	0.165	0.000	0.000	3533.459	-0.103	0.165	0.533	0.733	3533.459	-0.957	0.191	0.000	0.000
GTPBP1	1622.288	-0.240	0.113	0.033	0.084	1622.288	0.005	0.112	0.965	0.981	1622.288	0.245	0.130	0.059	0.203
CEP41	184.009	-0.581	0.190	0.002	0.011	184.009	-0.409	0.188	0.029	0.322	184.009	0.172	0.221	0.437	0.658
GTF2IRD1	1257.337	-0.202	0.190	0.288	0.426	1257.337	0.319	0.190	0.092	0.387	1257.337	0.522	0.220	0.018	0.094
ENTPD5	351.462	-0.458	0.165	0.006	0.022	351.462	-0.419	0.164	0.011	0.300	351.462	0.038	0.192	0.841	0.923
GOSR2	581.857	-0.490	0.308	0.112	0.213	581.857	-0.687	0.309	0.026	0.316	581.857	-0.197	0.357	0.582	0.765
NR1D1	1710.811	0.346	0.383	0.365	0.506	1710.811	0.839	0.383	0.028	0.321	1710.811	0.492	0.442	0.265	0.499
CLOCK	570.729	-0.459	0.191	0.016	0.049	570.729	-0.302	0.191	0.113	0.405	570.729	0.157	0.221	0.477	0.690
BABAM2	440.491	0.366	0.139	0.008	0.030	440.491	0.282	0.139	0.042	0.330	440.491	-0.084	0.160	0.599	0.777
CDC42BPB	2812.564	-0.498	0.157	0.001	0.008	2812.564	-0.025	0.156	0.875	0.937	2812.564	0.473	0.181	0.009	0.060
CD40	57.581	0.831	0.330	0.012	0.038	57.581	0.353	0.333	0.289	0.557	57.581	-0.478	0.381	0.209	0.434
SOX13	276.218	0.381	0.274	0.164	0.284	276.218	0.515	0.273	0.059	0.349	276.218	0.134	0.315	0.670	0.824
PREPL	983.096	-0.901	0.204	0.000	0.000	983.096	-0.041	0.202	0.841	0.917	983.096	0.860	0.235	0.000	0.005
APOBEC3B	511.199	0.239	0.228	0.294	0.432	511.199	-0.147	0.228	0.519	0.724	511.199	-0.386	0.263	0.143	0.348
ENTPD4	539.348	0.422	0.198	0.033	0.084	539.348	-0.138	0.198	0.488	0.705	539.348	-0.559	0.229	0.015	0.083
RBM39	2407.960	-0.379	0.200	0.058	0.128	2407.960	-0.190	0.200	0.341	0.599	2407.960	0.189	0.231	0.412	0.637
KIF20B	658.057	-0.163	0.213	0.446	0.581	658.057	-0.265	0.213	0.215	0.494	658.057	-0.102	0.247	0.679	0.830
CREB5	302.068	-1.660	0.500	0.001	0.005	302.068	-1.366	0.499	0.006	0.297	302.068	0.294	0.579	0.612	0.786
MAD2L1BP	298.954	-0.230	0.170	0.177	0.299	298.954	-0.062	0.168	0.713	0.846	298.954	0.168	0.197	0.393	0.621
PRDX6	4676.945	0.431	0.159	0.007	0.025	4676.945	0.031	0.159	0.843	0.918	4676.945	-0.399	0.184	0.030	0.132

WTAP	4067.639	0.324	0.239	0.176	0.298	4067.639	-0.409	0.240	0.088	0.380	4067.639	-0.733	0.277	0.008	0.057
AKAP12	162.975	-0.608	0.368	0.098	0.193	162.975	0.040	0.360	0.913	0.957	162.975	0.648	0.423	0.126	0.320
IER2	1338.857	-0.184	0.381	0.630	0.739	1338.857	-0.740	0.381	0.052	0.341	1338.857	-0.556	0.440	0.206	0.431
CYTIP	1157.345	0.436	0.269	0.105	0.202	1157.345	0.487	0.268	0.070	0.358	1157.345	0.051	0.310	0.870	0.938
CD44	5616.993	0.042	0.161	0.795	0.865	5616.993	0.217	0.161	0.178	0.467	5616.993	0.175	0.186	0.347	0.581
PITPNM1	898.658	0.010	0.197	0.960	0.976	898.658	-0.051	0.197	0.797	0.895	898.658	-0.061	0.228	0.790	0.896
PDIA4	3090.015	-0.134	0.301	0.655	0.759	3090.015	-0.488	0.301	0.105	0.397	3090.015	-0.354	0.347	0.308	0.544
NFE2L3	109.870	0.757	0.251	0.003	0.012	109.870	-0.032	0.256	0.902	0.951	109.870	-0.788	0.293	0.007	0.052
RNF14	735.740	-0.286	0.164	0.082	0.169	735.740	-0.251	0.164	0.126	0.418	735.740	0.035	0.190	0.856	0.931
VPS9D1	352.663	-0.506	0.225	0.024	0.067	352.663	-0.317	0.224	0.157	0.449	352.663	0.190	0.260	0.466	0.681
CD47	955.946	0.205	0.131	0.119	0.222	955.946	0.092	0.131	0.481	0.700	955.946	-0.112	0.151	0.459	0.676
RIN1	752.280	-0.013	0.193	0.947	0.967	752.280	0.402	0.192	0.036	0.324	752.280	0.415	0.222	0.061	0.208
NCOR1	1095.436	-0.342	0.150	0.022	0.062	1095.436	-0.183	0.149	0.220	0.499	1095.436	0.159	0.173	0.358	0.591
NCOR2	6187.294	-0.097	0.196	0.620	0.731	6187.294	0.322	0.196	0.101	0.394	6187.294	0.419	0.226	0.064	0.213
GDA	179.612	-0.121	0.298	0.686	0.783	179.612	-0.359	0.298	0.228	0.506	179.612	-0.239	0.345	0.490	0.700
RNF7	1150.826	0.485	0.218	0.027	0.072	1150.826	0.010	0.219	0.965	0.981	1150.826	-0.475	0.253	0.060	0.205
MTRF1	90.962	0.469	0.211	0.026	0.070	90.962	0.053	0.214	0.803	0.897	90.962	-0.416	0.245	0.090	0.262
TRAF4	1315.500	-0.386	0.288	0.180	0.303	1315.500	-0.414	0.288	0.150	0.443	1315.500	-0.028	0.333	0.933	0.970
ABCG1	926.948	-2.972	0.168	0.000	0.000	926.948	0.315	0.154	0.041	0.328	926.948	3.287	0.190	0.000	0.000
CELSR1	670.768	0.169	0.167	0.313	0.452	670.768	0.138	0.167	0.409	0.649	670.768	-0.031	0.193	0.872	0.939
AATK	221.543	0.939	0.457	0.040	0.097	221.543	0.577	0.457	0.207	0.489	221.543	-0.362	0.527	0.493	0.702
SNCAIP	33.534	0.639	0.608	0.293	0.431	33.534	0.439	0.609	0.470	0.692	33.534	-0.200	0.700	0.775	0.886
RGS6	14.750	0.445	0.846	0.599	0.713	14.750	0.382	0.845	0.651	0.810	14.750	-0.062	0.975	0.949	0.976
NUP155	1164.051	-1.181	0.324	0.000	0.002	1164.051	-0.792	0.323	0.014	0.306	1164.051	0.388	0.374	0.299	0.534
SEC24C	1516.493	-0.096	0.119	0.419	0.556	1516.493	-0.019	0.118	0.870	0.934	1516.493	0.076	0.137	0.577	0.762
TESMIN	192.900	-0.188	0.556	0.735	0.821	192.900	0.743	0.554	0.180	0.468	192.900	0.931	0.641	0.146	0.353
CLCA2	37.685	-0.418	0.826	0.613	0.724	37.685	0.760	0.817	0.352	0.606	37.685	1.178	0.949	0.215	0.440
ISG15	1027.426	3.512	0.260	0.000	0.000	1027.426	0.341	0.266	0.199	0.482	1027.426	-3.171	0.301	0.000	0.000
FEZ2	738.296	-0.660	0.180	0.000	0.002	738.296	-0.191	0.179	0.284	0.554	738.296	0.469	0.208	0.024	0.115
FEZ1	7870.612	0.246	0.155	0.113	0.215	7870.612	0.211	0.155	0.174	0.463	7870.612	-0.035	0.179	0.846	0.926
ARHGEF10	1660.296	-0.097	0.207	0.638	0.745	1660.296	-0.128	0.206	0.534	0.733	1660.296	-0.031	0.239	0.896	0.951
ZNF592	730.754	-0.695	0.166	0.000	0.000	730.754	-0.075	0.164	0.649	0.808	730.754	0.621	0.191	0.001	0.015
IKBKE	192.527	0.367	0.188	0.051	0.117	192.527	-0.131	0.190	0.491	0.707	192.527	-0.499	0.219	0.023	0.111
MORF4L2	5278.671	0.188	0.171	0.272	0.409	5278.671	-0.353	0.172	0.039	0.327	5278.671	-0.541	0.198	0.006	0.048
SH3PXD2A	155.605	0.124	0.225	0.583	0.700	155.605	0.234	0.224	0.296	0.564	155.605	0.110	0.260	0.671	0.825
MICAL2	3339.891	-0.938	0.267	0.000	0.003	3339.891	-0.556	0.267	0.037	0.326	3339.891	0.381	0.309	0.217	0.443
FNIP1	547.029	-0.130	0.268	0.628	0.738	547.029	0.385	0.267	0.149	0.442	547.029	0.515	0.309	0.096	0.273
CTR9	974.947	-0.437	0.211	0.039	0.095	974.947	-0.465	0.211	0.028	0.321	974.947	-0.028	0.244	0.909	0.959
PPM1F	635.756	-0.238	0.208	0.252	0.386	635.756	-0.293	0.208	0.158	0.450	635.756	-0.055	0.241	0.819	0.910
GCC2	597.845	-0.160	0.183	0.380	0.520	597.845	-0.043	0.182	0.812	0.901	597.845	0.117	0.211	0.579	0.764
RALGPS1	15.779	0.664	0.435	0.127	0.233	15.779	0.278	0.441	0.528	0.729	15.779	-0.385	0.502	0.443	0.663
CD58	198.853	0.393	0.186	0.035	0.087	198.853	-0.191	0.189	0.311	0.575	198.853	-0.584	0.217	0.007	0.052
MTFR1	820.877	-0.659	0.237	0.005	0.021	820.877	-0.469	0.237	0.047	0.336	820.877	0.190	0.274	0.489	0.699
PLCH2	410.956	0.636	0.725	0.380	0.520	410.956	0.627	0.724	0.387	0.633	410.956	-0.008	0.836	0.992	0.996
TTC37	2592.446	-0.813	0.245	0.001	0.005	2592.446	0.152	0.244	0.533	0.733	2592.446	0.965	0.282	0.001	0.009
HS2ST1	765.130	-0.384	0.220	0.081	0.168	765.130	0.154	0.219	0.482	0.701	765.130	0.538	0.254	0.034	0.144
TTLL4	207.502	-0.423	0.249	0.089	0.180	207.502	-0.219	0.247	0.374	0.622	207.502	0.203	0.288	0.480	0.693
SOCS5	487.223	-0.974	0.220	0.000	0.000	487.223	0.055	0.217	0.798	0.895	487.223	1.030	0.254	0.000	0.001
MDC1	3793.706	-0.339	0.163	0.037	0.092	3793.706	-0.119	0.163	0.464	0.689	3793.706	0.220	0.188	0.241	0.472
IQCB1	464.977	-0.715	0.174	0.000	0.000	464.977	-0.182	0.171	0.289	0.557	464.977	0.534	0.200	0.008	0.055
ZNF516	671.951	-0.577	0.237	0.015	0.047	671.951	0.138	0.236	0.559	0.748	671.951	0.715	0.274	0.009	0.061
PDE4DIP	1762.668	0.059	0.379	0.877	0.920	1762.668	0.269	0.379	0.477	0.698	1762.668	0.211	0.438	0.630	0.799
CD59	5434.427	0.387	0.260	0.137	0.247	5434.427	-0.537	0.260	0.039	0.327	5434.427	-0.925	0.301	0.002	0.022
BMS1P20	192.221	-0.675	0.327	0.039	0.095	192.221	-0.355	0.324	0.273	0.544	192.221	0.319	0.378	0.398	0.626
CEP135	156.150	-0.754	0.269	0.005	0.020	156.150	-0.765	0.268	0.004	0.297	156.150	-0.011	0.313	0.972	0.986
LPIN2	841.796	-0.181	0.210	0.391	0.530	841.796	-0.030	0.210	0.885	0.943	841.796	0.150	0.243	0.537	0.735
MARF1	1651.712	-0.467	0.219	0.033	0.085	1651.712	0.081	0.219	0.711	0.845	1651.712	0.548	0.253	0.031	0.133
DZIP3	209.427	-0.602	0.256	0.019	0.055	209.427	0.296	0.252	0.239	0.516	209.427	0.898	0.294	0.002	0.024
SAFB2	1838.616	0.157	0.170	0.355	0.495	1838.616	-0.082	0.170	0.628	0.796	1838.616	-0.240	0.196	0.223	0.450
ZNF432	87.165	0.461	0.266	0.083	0.170	87.165	0.061	0.268	0.820	0.906	87.165	-0.400	0.309	0.195	0.417
EIF5B	3021.652	0.660	0.306	0.031	0.081	3021.652	-0.503	0.306	0.101	0.394	3021.652	-1.163	0.354	0.001	0.013
CD63	10008.133	1.325	0.166	0.000	0.000	10008.133	0.109	0.166	0.509	0.718	10008.133	-1.215	0.191	0.000	0.000
IPO13	619.648	0.249	0.222	0.262	0.398	619.648	-0.401	0.223	0.072	0.361	619.648	-0.650	0.257	0.012	0.072
SDC3	2484.872	1.209	0.108	0.000	0.000	2484.872	-0.255	0.109	0.019	0.306	2484.872	-1.464	0.125	0.000	0.000
SLC25A44	279.915	-0.195	0.288	0.498	0.629	279.915	-0.657	0.289	0.023	0.315	279.915	-0.462	0.334	0.166	0.381
KIAA0040	306.603	0.158	0.174	0.363	0.504	306.603	-0.159	0.175	0.362	0.614	306.603	-0.317	0.202	0.116	0.305
TTI1	386.453	-0.644	0.191	0.001	0.005	386.453	-0.385	0.189	0.042	0.330	386.453	0.260	0.221	0.240	0.471

TGS1	545.314	-0.778	0.253	0.002	0.010	545.314	-0.703	0.252	0.005	0.297	545.314	0.074	0.293	0.799	0.900
PPIP5K1	277.299	-0.310	0.232	0.182	0.305	277.299	0.224	0.230	0.329	0.589	277.299	0.534	0.267	0.046	0.173
PHF14	1243.976	0.064	0.120	0.590	0.706	1243.976	0.035	0.119	0.769	0.877	1243.976	-0.029	0.138	0.832	0.918
FAM53B	1169.505	-0.590	0.088	0.000	0.000	1169.505	0.077	0.086	0.371	0.620	1169.505	0.667	0.101	0.000	0.000
CD68	19.712	0.790	0.685	0.249	0.383	19.712	0.963	0.678	0.155	0.448	19.712	0.173	0.779	0.824	0.913
DEPDC5	148.874	-0.445	0.176	0.011	0.037	148.874	-0.015	0.170	0.929	0.964	148.874	0.430	0.202	0.033	0.141
KDM4A	1104.889	-0.782	0.162	0.000	0.000	1104.889	-0.220	0.161	0.173	0.463	1104.889	0.563	0.188	0.003	0.027
N4BP1	511.319	-0.174	0.141	0.218	0.348	511.319	-0.039	0.140	0.779	0.883	511.319	0.135	0.163	0.409	0.635
LRRC14	398.035	-0.103	0.194	0.595	0.709	398.035	-0.328	0.194	0.090	0.384	398.035	-0.225	0.225	0.316	0.552
CLINT1	2127.885	-0.870	0.205	0.000	0.000	2127.885	-0.322	0.205	0.116	0.408	2127.885	0.548	0.237	0.021	0.104
VGLL4	723.469	0.371	0.150	0.014	0.043	723.469	0.162	0.150	0.281	0.551	723.469	-0.209	0.174	0.230	0.459
GREB1	14.621	1.944	0.689	0.005	0.019	14.621	0.334	0.720	0.642	0.805	14.621	-1.610	0.802	0.045	0.170
NUP93	1199.160	-0.597	0.363	0.100	0.196	1199.160	-0.929	0.363	0.011	0.299	1199.160	-0.332	0.420	0.429	0.652
BZW1	5035.495	-0.455	0.172	0.008	0.029	5035.495	-0.294	0.172	0.088	0.380	5035.495	0.161	0.199	0.417	0.642
UBE3C	1906.785	-0.645	0.222	0.004	0.015	1906.785	-0.304	0.222	0.170	0.460	1906.785	0.341	0.256	0.183	0.402
PRORP	549.884	-0.231	0.155	0.135	0.245	549.884	-0.163	0.154	0.291	0.559	549.884	0.069	0.179	0.702	0.845
RAPGEF2	640.099	-1.097	0.221	0.000	0.000	640.099	-0.360	0.219	0.100	0.394	640.099	0.737	0.255	0.004	0.034
EMC2	628.212	0.178	0.129	0.167	0.286	628.212	0.058	0.128	0.650	0.809	628.212	-0.120	0.149	0.421	0.645
[truncated: 1,527,386 more chars]
